# Supplementary material for: Association between arsenic exposure and intrauterine growth restriction: A systematic review and meta-analysis
Source: PLoS One. 2025 Jun 2;20(6):e0320603. doi: 10.1371/journal.pone.0320603 (PMC12129153; doi:10.1371/journal.pone.0320603)
Supplement: S1 — (DOCX) [file pone.0320603.s001.docx]

****S1 Statements****

**Funding**：This work was supported by Nature Science Foundation of Guizhou Province (QKH-J[2022]YB612),the City School Joint Foundation Project (QKH-PTRC[2020]-018 & ZYKH-HZ-Z[2021]292), City School Joint Foundation Project (QKH-PTRC[2019]-003 & ZYKH-HZ-Z[2020]64), Start-up Foundation for Doctors of Zunyi Medical University (QKH-PTRC[2019]-032), Guizhou Provincial Education Reform Project (SJJG2022-02-166), and the High-level innovative talents in Guizhou Province (GCC[2022]039-1), Postgraduate Research Fund project of Zunyi Medical University (ZYK216), Postgraduate Research Fund project of Zunyi Medical University (ZYK214),Scientific Research Program of Guizhou Provincial Department of Education (QJJ [2023] 019).

**Conflict of Interest：** The authors have no relevant financial or non-financial interests to disclose.

**Author contributions ：**

**Yan Xie:** Conceptualization、Funding acquisition、Methodology and Writing–review & editing

**jing Jiang:** Conceptualization、Data curation、Formal analysis、Funding acquisition、Validation and Writing–original draft

**Xuan Zuo:** Conceptualization、Data curation、Formal analysis and Writing–original draft

**Song lin An:** Formal analysis、Validation and Writing–original draft

**jing Yang:** Formal analysis、Funding acquisition、Validation

**Linfei Wu:** Investigation、Visualization

**Rong Zeng:** Investigation、Software

**Qiongdan Hu :** Formal analysis、Investigation

**Lu Fan:** Formal analysis、Investigation

**Haiyu Wang:** Investigation、Visualization

**Chuanwu Yang:** Investigation

**Yi han Liang:** Investigation

**yuanzhong Zhou:** Funding acquisition、Methodology、Writing – review & editing

**Hong Pan:** Conceptualization、Methodology、Writing – review & editing

**D****ata Availability Statement**：Provided as needed.
