# Supplementary material for: Association between arsenic exposure and intrauterine growth restriction: A systematic review and meta-analysis
Source: PLoS One. 2025 Jun 2;20(6):e0320603. doi: 10.1371/journal.pone.0320603 (PMC12129153; doi:10.1371/journal.pone.0320603)
Supplement: S2 Table — (DOCX) [file pone.0320603.s003.docx]

**S2 Table: Search strategy**

| **Database** | **Search Strategies** |
| --- | --- |
| China National Knowledge Infrastructure | (Pregnant OR pregnant rats OR pregnant mice OR pregnant women OR pregnancy) AND (Arsenic OR arsenic compounds OR arsenic trioxide OR arsenic pentoxide) |
| Wan Fang Database | (Pregnant OR pregnant rats OR pregnant mice OR pregnant women OR pregnancy) AND (Arsenic OR arsenic compounds OR arsenic trioxide OR arsenic pentoxide) |
| Vip Database | (((((Title OR key word = gestational period OR title OR key word = pregnant rat) OR title OR key word = pregnant rat) OR title or key word = pregnant woman) OR title or key word = pregnancy) AND ((title or key word = arsenic or title or key word = arsenic compounds) OR Title OR keyword = arsenic trioxide) OR title or keyword = arsenic pentoxide)) |
| Pubmed | (“pregnancy” OR “Pregnancies” OR “Gestation” OR “pregnant woman” OR “woman pregnant” OR “women pregnant”)AND(“Arsenic” OR “As_2_O_3_” OR “arsenous anhydride” OR “diarsenic trioxide” OR “arsenic oxide” OR “tetraarsenic oxide” OR “Arsenolite” OR “As_4_O_6_” OR “tetra arsenic oxide” OR “Trisenox” OR “arsenic compounds” OR “compounds arsenic”) |
| Web of Sccience | (((((((((((((((((((TS=(pregnancy)) OR TS=(Pregnancies)) OR TS=(Gestation)) OR TS=(Pregnant Woman)) OR TS=(Woman, Pregnant)) OR TS=(Women, Pregnant))) AND TS=(Arsenic)) OR TS=(As_2_O_3_)) OR TS=(Arsenous Anhydride)) OR TS=(Diarsenic Trioxide)) OR TS=(Arsenic Oxide)) OR TS=(Tetraarsenic Oxide)) OR TS=(Arsenolite)) OR TS=( As_4_O_6_)) OR TS=(Tetra Arsenic Oxide)) OR TS=(Trisenox)) OR TS=(Arsenic Compounds)) OR TS=( Compounds, Arsenic)) |
| Elsevier ScienceDirect | (“pregnancy” OR “Pregnancies” OR “Gestation” OR “pregnant woman” OR “woman pregnant” OR “women pregnant”)AND(“Arsenic” OR “As_2_O_3_” OR “arsenous anhydride” OR “diarsenic trioxide” OR “arsenic oxide” OR “tetraarsenic oxide” OR “Arsenolite” OR “As_4_O_6_” OR “tetra arsenic oxide” |
