# Supplementary material for: Association between arsenic exposure and intrauterine growth restriction: A systematic review and meta-analysis
Source: PLoS One. 2025 Jun 2;20(6):e0320603. doi: 10.1371/journal.pone.0320603 (PMC12129153; doi:10.1371/journal.pone.0320603)
Supplement: S3 Table — (DOCX) [file pone.0320603.s004.docx]

**S3 Table: NTP/OHAT risk of bias rating tool**

Potential sources of bias are assessed with a set of 6 questions or “domains” and “bias associated with conflict of interest” was added as an additional category to consider. Detailed criteria are provided under each question.

This tool evaluates each study based on a set of 7 risk-of-bias questions:

**- Confounding bias [Key item]:** Did the study design or analysis (stratification, regression, matching, inverse probability weighing, etc.) account for important confounding and modifying variables?

**- Selection bias:** Did selection of study participants result in appropriate comparison groups?

**- Attrition/exclusion bias:** Were outcome data incomplete due to attrition or exclusion from analysis?

**- Detection bias [Key item]:** Can we be confident in the **exposure characterization**?

**- Detection bias [Key item]:** Can we be confident in the **outcome assessment**?

**- Selective reporting bias:** Were all measured outcomes reported?

**- Conflict of interest:** Was the study free of support from a company, study author, or other entity having a financial interest in any of the treatments studied?

| **Selection bias** | Did selection of study participants result in appropriate comparison groups? |
| --- | --- |
| Low risk of bias | There is direct evidence that subjects (both exposed and non-exposed) were similar (e.g., recruited from the same eligible population, recruited with the same method of ascertainment using the same inclusion and exclusion criteria, and were of similar age and health status), recruited within the same time frame, and had similar participation/response rates.  There is direct evidence that cases and controls were similar (e.g., recruited from the same eligible population including being of similar age, gender, ethnicity, and eligibility criteria other than outcome of interest as appropriate), recruited within the same time frame, and controls are described as having no history of the outcome.  A study will be considered low risk of bias if baseline characteristics of groups differed but these differences were considered as potential confounding or stratification variables |
| Probably low risk of bias | There is indirect evidence that subjects (both exposed and non-exposed) were similar (e.g., recruited from the same eligible population, recruited with the same method of ascertainment using the same inclusion and exclusion criteria, and were of similar age and health status), recruited within the same time frame, and had similar participation/response rates, OR differences between groups would not appreciably bias results |
| Probably high risk of bias | There is indirect evidence that subjects (both exposed and non-exposed) were not similar, recruited within very different time frames, or had very different participation/response rates, OR there is insufficient information provided about the comparison group including a different rate of non-response without an explanation (record “NR” as basis for answer). |
| Definitely high risk of bias | There is direct evidence that subjects (both exposed and non-exposed) were not similar, recruited within very different time frames, or had very different participation/response rates |
| **Attrition/exclusion bias** | Were outcome data complete without attrition or exclusion from analysis? |
| Low risk of bias | There is direct evidence that loss of subjects (i.e., incomplete outcome data) was adequately addressed and reasons were documented when human subjects were removed from a study. Acceptable handling of subject attrition includes: very little missing outcome data; reasons for missing subjects unlikely to be related to outcome (for survival data, censoring unlikely to be introducing bias); missing outcome data balanced in numbers across study groups, with similar reasons for missing data across groups, OR missing data have been imputed using appropriate methods and characteristics of subjects lost to follow up or with unavailable records are described in identical way and are not significantly different from those of the study participants. |
| Probably low risk of bias | There is indirect evidence that loss of subjects (i.e., incomplete outcome data) was adequately addressed and reasons were documented when human subjects were removed from a study, OR it is deemed that the proportion lost to follow-up would not appreciably bias results. This would include reports of no statistical differences in characteristics of subjects lost to follow up or with unavailable records from those of the study participants. Generally, the higher the ratio of participants with missing data to participants with events, the greater potential there is for bias. For studies with a long duration of follow-up, some withdrawals for such reasons are inevitable. |
| Probably high risk of bias | There is indirect evidence that loss of subjects (i.e., incomplete outcome data) was unacceptably large and not adequately addressed, OR there is insufficient information provided about numbers of subjects lost to follow-up(record “NR” as basis for answer). |
| Definitely high risk of bias | There is direct evidence that loss of subjects (i.e., incomplete outcome data) was unacceptably large and not adequately addressed. Unacceptable handling of subject attrition includes: reason for missing outcome data likely to be related to true outcome, with either imbalance in numbers or reasons for missing data across study groups; or potentially inappropriate application of imputation. |
| **Detection bias** | Can we be confident in the exposure characterization? |
| Low risk of bias | There is direct evidence that exposure was consistently assessed (i.e., under the same method and time-frame) using well-established methods that directly measure exposure (e.g., measurement of the chemical in air or measurement of the chemical in blood, plasma, urine, etc.),OR exposure was assessed using less-established methods that directly measure exposure and are validated against well-established methods. |
| Probably low risk of bias | There is indirect evidence that the exposure was consistently assessed using well-established methods that directly measure exposure, OR exposure was assessed using indirect measures (e.g., questionnaire or occupational exposure assessment by a certified industrial hygienist) that have been validated or empirically shown to be consistent with methods that directly measure exposure (i.e., inter-methods validation: one method vs. another). |
| Probably high risk of bias | There is indirect evidence that the exposure was assessed using poorly validated methods that directly measure exposure, OR there is direct evidence that the exposure was assessed using indirect measures that have not been validated or empirically shown to be consistent with methods that directly measure exposure (e.g., a job-exposure matrix or self-report without validation) (record “NR” as basis for answer),OR there is insufficient information provided about the exposure assessment, including validity and reliability, but no evidence for concern about the method used (record “NR” as basis for answer) |
| Definitely high risk of bias | There is direct evidence that the exposure was assessed using methods with poor validity, OR evidence of exposure misclassification (e.g., differential recall of self-reported exposure) |
| **Outcome assessment** | Can we be confident in the outcome assessment? |
| Low risk of bias | There is direct evidence that the outcome was assessed using well-established methods (e.g., the “gold standard” with validity and reliability >0.70 Genaidy et al. 2007), AND subjects had been followed for the same length of time in all study groups. Acceptable assessment methods will depend on the outcome, but examples of such methods may include: objectively measured with diagnostic methods, measured by trained interviewers, obtained from registries (Shamliyan et al. 2010), AND there is direct evidence that the outcome assessors (including study subjects, if outcomes were self-reported) were adequately blinded to the study group, and it is unlikely that they could have broken the blinding prior to reporting outcomes. |
| Probably low risk of bias | There is indirect evidence that the outcome was assessed using acceptable methods (i.e., deemed valid and reliable but not the gold standard) (e.g., validity and reliability ≥0.40 Genaidyet al. 2007), AND subjects had been followed for the same length of time in all study groups [Acceptable, but not ideal assessment methods will depend on the outcome, but examples of such methods may include proxy reporting of outcomes and mining of data collected for other purposes],OR it is deemed that the outcome assessment methods used would not appreciably bias results, AND there is indirect evidence that the outcome assessors (including study subjects, if outcomes were self-reported) were adequately blinded to the study group, and it is unlikely that they could have broken the blinding prior to reporting outcomes, OR it is deemed that lack of adequate blinding of outcome assessors would not appreciably bias results, which is more likely to apply to objective outcome measures. |
| Probably high risk of bias | There is indirect evidence that the outcome assessment method is an insensitive instrument (e.g., a questionnaire used to assess outcomes with no information on validation), OR the length of follow up differed by study group, OR there is indirect evidence that it was possible for outcome assessors (including study subjects if outcomes were self-reported) to infer the study group prior to reporting outcomes, OR there is insufficient information provided about blinding of outcome assessors (record “NR” as basis for answer) |
| Definitely high risk of bias | There is direct evidence that the outcome assessment method is an insensitive instrument, OR the length of follow up differed by study group, OR there is direct evidence for lack of adequate blinding of outcome assessors (including study subjects if outcomes were self-reported), including no blinding or incomplete blinding. |
| **Selective reporting of results** | Were all measured outcomes reported? |
| Low risk of bias | There is direct evidence that all of the study’s measured outcomes (primary and secondary) outlined in the protocol, methods, abstract, and/or introduction (that are relevant for the evaluation) have been reported. This would include outcomes reported with sufficient detail to be included in meta-analysis or fully tabulated during data extraction and analyses had been planned in advance. |
| Probably low risk of bias | There is indirect evidence that all of the study’s measured outcomes (primary and secondary) outlined in the protocol, methods, abstract, and/or introduction (that are relevant for the evaluation) have been reported, OR analyses that had not been planned in advance (i.e., retrospective unplanned subgroup analyses) are clearly indicated as such and it is deemed that the unplanned analyses were appropriate and selective reporting would not appreciably bias results (e.g., appropriate analyses of an unexpected effect). This would include outcomes reported with insufficient detail such as only reporting that results were statistically significant (or not). |
| Probably high risk of bias | There is indirect evidence that all of the study’s measured outcomes (primary and secondary) outlined in the protocol, methods, abstract, and/or introduction (that are relevant for the evaluation) have been reported, OR and there is indirect evidence that unplanned analyses were included that may appreciably bias results, OR there is insufficient information provided about selective outcome reporting (record “NR” as basis for answer) |
| Definitely high risk of bias | There is direct evidence that all of the study’s measured outcomes (primary and secondary) outlined in the protocol, methods, abstract, and/or introduction (that are relevant for the evaluation) have not been reported. In addition to not reporting outcomes, this would include reporting outcomes based on composite score without individual outcome components or outcomes reported using measurements, analysis methods or subsets of the data (e.g., subscales) that were not pre-specified or reporting outcomes not pre-specified, or that unplanned analyses were included that would appreciably bias results. |

**Table. Source of funding and conflict of interests have been checked in each publications.**

| **Conflict of interest** | Could source of funding affect reporting of outcomes or analyses? |
| --- | --- |
| Low risk of bias | There is direct evidence that there is no conflict of interest from authors |
| Probably low risk of bias | There is indirect evidence that there is no conflict of interest from authors |
| Probably high risk of bias | There is indirect evidence that funding/sponsor may have influenced the protocol or a selective reporting and/or there is a conflict of interest from authors |
| Definitely high risk of bias | There is direct evidence that funding/sponsor may have influenced the protocol or a selective reporting and/or there is a conflict of interest from authors |
