# Supplementary material for: Association between arsenic exposure and intrauterine growth restriction: A systematic review and meta-analysis
Source: PLoS One. 2025 Jun 2;20(6):e0320603. doi: 10.1371/journal.pone.0320603 (PMC12129153; doi:10.1371/journal.pone.0320603)
Supplement: S4 Table — (DOCX) [file pone.0320603.s005.docx]

**S4 Table. Quality assessment results of included studies**

**Table 1**

**The results of assessment of included studies quality based on AHRQ evaluation criteria (cross sectional studies).**

AHRQ: Agency for Health-care Research and Quality.

(1) Is the source of the data clear (survey, literature review)?

(2) Do you list the inclusion and exclusion criteria for exposed and non-exposed groups (cases and controls) or refer to previous publications?

(3) Is there a time period for identifying patients?

(4) If it is not the source of the crowd, are the research subjects continuous?

(5) Does the evaluator's subjective factors mask other aspects of the research participants?

(6) Describes any evaluation to ensure quality (eg testing / retesting of the main outcome indicators).

(7) explains the reasons for excluding any patients from the analysis.

(8) describes how to evaluate and / or to control measures of confounding factors.

(9) If possible, explain how the missing data was handled in the analysis.

(10) Summarize the patient’s response rate and the completeness of the data collection.

(11) If there is follow-up, identify the expected Percentage of patients with incomplete data or follow-up results.

**Table 2**

**Results of study quality evaluation included in cohort studies based on NOS (point).**

NOS: The Newcastle-Ottawa Scale;

(1)Exposure cohort representation;

(2)Non-exposed selection;

(3)Determination of exposure;

(4)No subjects had outcome events before the study began;

(5)Comparability;

(6)Assessment of the outcome event;

(7)Adequacy of follow-up;

(8)Follow-up completeness.
