# Supplementary material for: Association between arsenic exposure and intrauterine growth restriction: A systematic review and meta-analysis
Source: PLoS One. 2025 Jun 2;20(6):e0320603. doi: 10.1371/journal.pone.0320603 (PMC12129153; doi:10.1371/journal.pone.0320603)
Supplement: S5 Table — (DOCX) [file pone.0320603.s006.docx]

**S5 Table. Study screening and exclusion details**

**Table 1: Summary of Screening and Exclusion Process**

| **Screening Stage** | **Records Count** | **Reason for Exclusion** |
| --- | --- | --- |
| Initial Records | 12066 | - |
| Records after deduplication | 10833 | Duplicate records |
| Records excluded in title/abstract screening | 10766 | Not relevant for inclusion in the study |
|  |  |  |

**Table 2: Details of Full-text Screening and Excluded Studies**

| **No.** | **Study ID** | **Screening Stage** | **Title** | **Author** | **Journal** | **Year** | **Included Yes/No** | **Reason for Exclusion** |
| --- | --- | --- | --- | --- | --- | --- | --- | --- |
| 1 | 17 | Full-text Review | Arsenic and birth outcomes in a predominately lower income Hispanic pregnancy cohort in Los Angeles | Howe, C. G. | Environ Res | 2020 | NO | No relevant data |
| 2 | 27 | Full-text Review | Association between prenatal arsenic exposure, birth outcomes, and pregnancy complications: An observational study within the National Children's Study cohort | Shih, Y. H. | Environ Res | 2020 | NO | No relevant data |
| 3 | 93 | Full-text Review | Pregnancy outcomes, infant mortality, and arsenic in drinking water in West Bengal, India | von Ehrenstein, O. S. | Am J Epidemiol | 2006 | NO | No relevant outcome |
| 4 | 95 | Full-text Review | Estimating Effects of Arsenic Exposure During Pregnancy on Perinatal Outcomes in a Bangladeshi Cohort | Kile, M. L. | Epidemiology | 2016 | NO | No relevant data |
| 5 | 124 | Full-text Review | Investigating causal relation between prenatal arsenic exposure and birthweight: Are smaller infants more susceptible? | Rahman, M. L. | Environ Int | 2017 | NO | No relevant data |
| 6 | 174 | Full-text Review | Maternal arsenic exposure associated with low birth weight in Bangladesh | Huyck, K. L. | J Occup Environ Med | 2007 | NO | No relevant data |
| 7 | 180 | Full-text Review | Associations between urinary total arsenic levels, fetal development, and neonatal birth outcomes: A cohort study in Taiwan | Liao, K. W. | Sci Total Environ | 2018 | NO | No relevant data |
| 8 | 188 | Full-text Review | Environmental exposure to arsenic and cadmium during pregnancy and fetal size: a longitudinal study in rural Bangladesh | Kippler, M. | Reprod Toxicol | 2012 | NO | No relevant data |
| 9 | 226 | Full-text Review | Arsenic in drinking water and pregnancy outcomes | Ahmad, S. A. | Environ Health Perspect | 2001 | NO | No relevant outcome |
| 10 | 257 | Full-text Review | Arsenic exposure through drinking water and its effect on pregnancy outcome in Bengali women | Sen, J. | Arh Hig Rada Toksikol | 2008 | NO | No relevant outcome |
| 11 | 365 | Full-text Review | Does the metal content in soil around a pregnant woman's home increase the risk of low birth weight for her infant? | McDermott, S. | Environ Geochem Health | 2014 | NO | No relevant data |
| 12 | 378 | Full-text Review | Arsenic exposure in pregnancy: a population-based study in Matlab, Bangladesh | Vahter, M. E. | J Health Popul Nutr | 2006 | NO | No relevant data |
| 13 | 545 | Full-text Review | Chronic arsenic exposure and adverse pregnancy outcomes in bangladesh | Milton, A. H. | Epidemiology | 2005 | NO | No relevant outcome |
| 14 | 618 | Full-text Review | Arsenic in drinking water and adverse pregnancy outcome in an arseniasis-endemic area in northeastern Taiwan | Yang, C. Y. | Environ Res | 2003 | NO | No relevant outcome |
| 15 | 715 | Full-text Review | Prenatal metal mixtures and fetal size in mid-pregnancy in the MADRES study | Howe, C. G. | Environ Res | 2021 | NO | No relevant risk factors |
| 16 | 726 | Full-text Review | Arsenic exposure from drinking water and birth weight | Hopenhayn, C. | Epidemiology | 2003 | NO | No relevant objectives |
| 17 | 1154 | Full-text Review | Arsenic exposure during pregnancy and size at birth: a prospective cohort study in Bangladesh | Rahman, A. | Am J Epidemiol | 2009 | NO | No relevant data |
| 18 | 1170 | Full-text Review | Examining the Relationship Between Low Birth Weight Occurrence and Passive Measures of Environmental Arsenic by Census Tract in Escambia and Santa Rosa Counties, Florida | Scott-Richardson, M. | Environ Health Insights | 2020 | NO | No relevant data |
| 19 | 1243 | Full-text Review | A pilot study: the importance of inter-individual differences in inorganic arsenic metabolism for birth weight outcome | Gelmann, E. R. | Environ Toxicol Pharmacol | 2013 | NO | No relevant data |
| 20 | 1343 | Full-text Review | Maternal arsenic exposure, arsenic methylation efficiency, and birth outcomes in the Biomarkers of Exposure to ARsenic (BEAR) pregnancy cohort in Mexico | Laine, J. E. | Environ Health Perspect | 2015 | NO | No relevant data |
| 21 | 1487 | Full-text Review | Maternal/fetal metabolomes appear to mediate the impact of arsenic exposure on birth weight: A pilot study | Wei, Y. | J Expo Sci Environ Epidemiol | 2017 | NO | No relevant objectives |
| 22 | 1626 | Full-text Review | Early pregnancy exposure to metal mixture and birth outcomes - A prospective study in Project Viva | Rahman, M. L. | Environ Int | 2021 | NO | No relevant data |
| 23 | 1645 | Full-text Review | Combined Effects of Prenatal Exposures to Environmental Chemicals on Birth Weight | Govarts, E. | Int J Environ Res Public Health | 2016 | NO | No relevant data |
| 24 | 1868 | Full-text Review | Mediating role of arsenic in the relationship between diet and pregnancy outcomes: prospective birth cohort in Bangladesh | Lin, P. D. | Environ Health | 2019 | NO | No relevant objectives |
| 25 | 1944 | Full-text Review | Levels of heavy metals and trace elements in umbilical cord blood and the risk of adverse pregnancy outcomes: a population-based study | Zheng, G. | Biol Trace Elem Res | 2014 | NO | No relevant risk factors |
| 26 | 2017 | Full-text Review | Effects of low-dose drinking water arsenic on mouse fetal and postnatal growth and development | Kozul-Horvath, C. D. | PLoS One | 2012 | NO | No relevant outcome |
| 27 | 2088 | Full-text Review | Association between gestational arsenic exposure and intrauterine growth restriction: the role of folate content | Xu, F. X. | Environ Sci Pollut Res Int | 2022 | NO | No relevant data |
| 28 | 2325 | Full-text Review | Reproductive consequences of oral arsenate exposure during pregnancy in a mouse model | Hill, D. S. | Birth Defects Res B Dev Reprod Toxicol | 2008 | NO | No relevant objectives |
| 29 | 2329 | Full-text Review | Birth Size Outcomes in Relation to Maternal Blood Levels of Some Essential and Toxic Elements | Daniali, S. S. | Biol Trace Elem Res | 2022 | NO | No relevant objectives |
| 30 | 2337 | Full-text Review | Umbilical Cord Blood Metal Mixtures and Birth Size in Bangladeshi Children | Lee, M. S. | Environ Health Perspect | 2021 | NO | No relevant data |
| 31 | 2534 | Full-text Review | Prenatal Metal Mixtures and Birth Weight for Gestational Age in a Predominately Lower-Income Hispanic Pregnancy Cohort in Los Angeles | Howe, C. G. | Environ Health Perspect | 2020 | NO | No relevant data |
| 32 | 3068 | Full-text Review | Assessment of ten trace elements in umbilical cord blood and maternal blood: association with birth weight | Bermúdez, L. | J Transl Med | 2015 | NO | No relevant data |
| 33 | 3122 | Full-text Review | Cord serum elementomics profiling of 56 elements depicts risk of preterm birth: Evidence from a prospective birth cohort in rural Bangladesh | Huang, Hui | Environment International | 2021 | NO | No relevant outcome |
| 34 | 3194 | Full-text Review | The concentration of selected elements in the placenta according to selected sociodemographic factors and their effect on birth mass and birth length of newborns | Mazurek, Dominika | Journal of Trace Elements in Medicine and Biology | 2020 | NO | No relevant outcome |
| 35 | 3209 | Full-text Review | Effects of prenatal exposure to arsenic on neonatal birth size in Wujiang, China | Wang, Yaqian | Chemosphere | 2022 | NO | No relevant outcome |
| 36 | 3220 | Full-text Review | Environmental factors and apoptotic indices in patients with intrauterine growth retardation: A nested case-control study | El-Baz, Mona A. H. | Environmental Toxicology and Pharmacology | 2015 | NO | No relevant data |
| 37 | 3234 | Full-text Review | Evaluation of in utero exposure to arsenic in South Africa | Röllin, Halina B. | Science of The Total Environment | 2017 | NO | No relevant data |
| 38 | 3292 | Full-text Review | Arsenic in private well water and birth outcomes in the United States | Bulka, Catherine M. | Environment International | 2022 | NO | No relevant data |
| 39 | 3311 | Full-text Review | Cord blood DNA methylation of DNMT3A mediates the association between in utero arsenic exposure and birth outcomes: Results from a prospective birth cohort in Bangladesh | Bozack, Anne K. | Environmental Research | 2020 | NO | No relevant data |
| 40 | 3376 | Full-text Review | Intrauterine multi-metal exposure is associated with reduced fetal growth through modulation of the placental gene network | Deyssenroth, Maya A. | Environment International | 2018 | NO | No relevant data |
| 41 | 3405 | Full-text Review | Placental metal concentrations in relation to placental growth, efficiency and birth weight | Punshon, Tracy | Environment International | 2019 | NO | No relevant objectives |
| 42 | 4109 | Full-text Review | Reproductive outcomes in pregnant women and its association with arsenic contamination in drinking water, in a region characterized by high birth weight rates in Peru | Fano, D. | Journal of Maternal-Fetal & Neonatal Medicine | 2021 | NO | No relevant data |
| 43 | 4310 | Full-text Review | Maternal Blood Levels of Toxic and Essential Elements and Birth Outcomes in Argentina: The EMASAR Study | Xu, S. S. | International Journal of Environmental Research and Public Health | 2022 | NO | No relevant data |
| 44 | 4471 | Full-text Review | Distributions of Heavy Metals in Maternal and Cord Blood and the Association with Infant Birth Weight in China | Hu, X. B. | Journal of Reproductive Medicine | 2015 | NO | No relevant data |
| 45 | 4932 | Full-text Review | Geospatial association between adverse birth outcomes and arsenic in groundwater in New Hampshire, USA | Shi, X. | Environmental Geochemistry and Health | 2015 | NO | No relevant data |
| 46 | 5646 | Full-text Review | Heavy metals exposure levels and their correlation with different clinical forms of fetal growth restriction | Sabra, S. | Plos One | 2017 | NO | No relevant data |
| 47 | 11391 | Full-text Review | Association between Maternal Mid-Pregnancy Blood Nickel and Arsenic Levels and Fetal Growth and Development | Xiuli Ding | Journal of Environmental Hygiene | 2021 | NO | No relevant data |
| 48 | 11448 | Full-text Review | Analysis of the Effects and Related Factors of Heavy Metal Exposure During Pregnancy on Mothers and Infants | Hong Zhu | Chinese Journal of Physicians | 2010 | NO | No relevant data |
| 49 | 11613 | Full-text Review | Canonical Correlation Analysis of Umbilical Cord Blood Heavy Metal Levels and Fetal Growth and Development | Xiuli Ding | Journal of Environmental and Occupational Medicine | 2020 | NO | No relevant data |
| 50 | 11749 | Full-text Review | Effects of Maternal Heavy Metal Exposure on Early Pregnancy Outcomes and Its Mechanisms | Jie Ou | Chinese Academy of Medical Sciences & Peking Union Medical College | 2020 | NO | No relevant outcome |
| 51 | 11752 | Full-text Review | Effects of Heavy Metal Exposure on Fetal Development During Different Pregnancy Stages | Ruiran He | Occupational and Health | 2019 | NO | No relevant outcome |
| 52 | 11809 | Full-text Review | Study on the Effects of Prenatal Heavy Metal Exposure on Fetal and Neonatal Growth and Development and the Role of Placental Barrier | Qin Bian | Lanzhou University | 2018 | NO | No relevant outcome |
| 53 | 11877 | Full-text Review | Effects of Prenatal Low-Dose Heavy Metal Exposure and Their Interactions on Term Neonates with Low Birth Weight | Wenjuan Zeng | Nanchang University (School of Medicine) | 2020 | NO | No relevant data |
| 54 | 11882 | Full-text Review | Heavy Metal Levels in Newborns in Shaoguan, Guangdong, and Their Relationship with Low Birth Weight | Jinhua Zhou | Chinese Journal of Public Health | 2015 | NO | No relevant data |
| 55 | 12030 | Full-text Review | Relationship Between Intrauterine Arsenic, Cadmium, and Lead Exposure and Newborn Growth and Development | Lihua Huang | Chinese Center for Disease Control and Prevention | 2014 | NO | No relevant data |
| 56 | 12066 | Full-text Review | Maternal Exposure to Low-Level Heavy Metals During Pregnancy and Birth Size | Shirai, S. | J Environ Sci Health A Tox Hazard Subst Environ Eng | 2010 | NO | No relevant data |
