# Supplementary material for: Association between arsenic exposure and intrauterine growth restriction: A systematic review and meta-analysis
Source: PLoS One. 2025 Jun 2;20(6):e0320603. doi: 10.1371/journal.pone.0320603 (PMC12129153; doi:10.1371/journal.pone.0320603)
Supplement: S6 Table — (DOCX) [file pone.0320603.s007.docx]

**S6 Table. Included references for meta-analysis**

| **Number** | **Study ID** | **Screening Stage** | **Title** | **Author** | **Journal** | **year** | **Included Yes/No** |
| --- | --- | --- | --- | --- | --- | --- | --- |
| 1 | 80 | Full-text Review | Maternal serum arsenic level during pregnancy is positively associated with adverse pregnant outcomes in a Chinese population | Hua Wang | Toxicol Appl Pharmacol | 2018 | Yes |
| 2 | 910 | Full-text Review | Total Urinary Arsenic and Inorganic Arsenic Concentrations and Birth Outcomes in Pregnant Women of Tacna, Peru: A Cross-Sectional Study | Fano-Sizgorich | Expo Health | 2021 | Yes |
| 3 | 933 | Full-text Review | Maternal blood arsenic levels and associations with birth weight-for-gestational age | Anne M. Mullin | Environ Res | 2019 | Yes |
| 4 | 942 | Full-text Review | Metals exposure and risk of small-for-gestational age birth in a Canadian birth cohort: The MIREC study | Shari Thomas | Environ Res | 2015 | Yes |
| 5 | 1187 | Full-text Review | Maternal arsenic exposure and birth outcomes: A birth cohort study in Wuhan, China | Hongxiu Liu | Environ Pollut | 2018 | Yes |
| 6 | 2036 | Full-text Review | Maternal exposure to arsenic and mercury and associated risk of adverse birth outcomes in small-scale gold mining communities in Northern Tanzania | Elias C. Nyanza | Environ Int | 2020 | Yes |
| 7 | 3230 | Full-text Review | Arsenic in drinking water and adverse birth outcomes in Ohio | Kirsten S. Almberg | Environmental Research | 2017 | Yes |
| 8 | 3240 | Full-text Review | Exposure to atmospheric metals using moss bioindicators and neonatal health outcomes in Portland, Oregon | Saskia Comess | Environmental Pollution | 2021 | Yes |
| 9 | 3256 | Full-text Review | Placental metal concentrations and birth outcomes: The Environment and Childhood (INMA) project | Carmen Freire | International Journal of Hygiene and Environmental Health | 2019 | Yes |
| 10 | 3309 | Full-text Review | Low level arsenic contaminated water consumption and birth outcomes in Romania—An exploratory study | Michael S. Bloom | Reproductive Toxicology | 2016 | Yes |
| 11 | 4366 | Full-text Review | Prenatal Heavy Metal Exposure and Adverse Birth Outcomes in Myanmar: A Birth-Cohort Study | Kyi Mar Wai | International Journal of Environmental Research and Public Health | 2017 | Yes |
