# Supplementary material for: Association between arsenic exposure and intrauterine growth restriction: A systematic review and meta-analysis
Source: PLoS One. 2025 Jun 2;20(6):e0320603. doi: 10.1371/journal.pone.0320603 (PMC12129153; doi:10.1371/journal.pone.0320603)
Supplement: S7 Table — Note: As, arsenic; SGA, small for gestational age; LBW, low birth weight; VLBW, very low birth weight; PTB, preterm birth; VPTB, very preterm birth; USA, United States of America; CO, cohort study; CS, cross-sectional study. (DOCX) [file pone.0320603.s008.docx]

**S7 Table. Data extraction table**

| **Study ID** | **Author** | **year** | **Study design** | **Country** | **Outcome** | **Effect Size (OR/RR)** | **Sample** | **Exposure Time** | **Quality score** | **Data Extractor** | **Extraction Date** |
| --- | --- | --- | --- | --- | --- | --- | --- | --- | --- | --- | --- |
| 80 | Hua Wang | 2018 | CO | China (Asia) | LBW, SGA | 1.30 (0.79,2.15)/1.35 (1.02，1.78) | Maternal serum | Early and mid-pregnancy | 7 | JJ and XZ | 13-Jun-24 |
| 910 | Fano-Sizgorich | 2021 | CS | Peru (South America) | SGA,PTB | 0.93 (0.28, 3.08)/1.59 (0.38, 6.61) | Urine | Mid-pregnancy | 7 | JJ and XZ | 13-Jun-24 |
| 933 | Anne M. Mullin | 2019 | CO | Mexico (North America) | SGA | 1.44(1.08–1.93) | Blood | Mid to late pregnancy | 8 | JJ and XZ | 13-Jun-24 |
| 942 | Shari Thomas | 2015 | CO | Canada (North America) | SGA | 1.65 (1.10–2.47) | Urine | Throughout the entire pregnancy | 8 | JJ and XZ | 13-Jun-24 |
| 1187 | Hongxiu Liu | 2018 | CO | China (Asia) | SGA | 1.25 (1.03, 1.49) | Urine | Throughout the entire pregnancy | 8 | JJ and XZ | 14-Jun-24 |
| 2036 | Elias C. Nyanza | 2020 | CO | Tanzania (Africa) | PTB, LBW | 1.20(1.05–1.37)/1.09(0.91–1.31) | Urine | Throughout the entire pregnancy | 9 | JJ and XZ | 14-Jun-24 |
| 3230 | Kirsten S. Almberg | 2017 | CO | USA (North America) | SGA, LBW, VLBW, PTB, VPTB | 1.01 (0.99, 1.03)/1.07 (1.01, 1.13)/1.01 (0.79, 1.29)/1.09 (0.99, 1.19)/1.09 (0.89, 1.34) | Drinking water | Throughout the entire pregnancy | 7 | JJ and XZ | 14-Jun-24 |
| 3240 | Saskia Comess | 2021 | CO | USA (North America) | PTB, VPTB, SGA | 1.01 (0.97, 1.04)/0.95 (0.87, 1.04)/1.00 (0.97, 1.03) | Moss | Throughout the entire pregnancy | 7 | JJ and XZ | 14-Jun-24 |
| 3256 | Carmen Freire | 2019 | CO | Spain (Europe) | LBW, SGA, PTB | 0.52 (0.11,3.25)/1.26 (0.38, 2.21) /0.69 ( 0.08,6.84) | Placentas | Throughout the entire pregnancy | 8 | JJ and XZ | 15-Jun-24 |
| 3309 | Michael S. Bloom | 2016 | CO | Romania (Europe) | SGA,PTB | 0.91 (0.44, 1.91)/0.79 (0.22, 2.78) | Drinking water | Throughout the entire pregnancy | 8 | JJ and XZ | 15-Jun-24 |
| 4366 | Kyi Mar Wai | 2017 | CO | Myanmar (Southeast Asia) | LBW,PTB | 0.99 (0.99–1.00)/1.00 (0.99–1.00) | Urine | Throughout the entire pregnancy | 9 | JJ and XZ | 15-Jun-24 |

Note: As, arsenic; SGA, small for gestational age; LBW, low birth weight; VLBW, very low birth weight; PTB, preterm birth; VPTB, very preterm birth; USA, United States of America; CO, cohort study; CS, cross-sectional study.
