# Supplementary material for: Association between arsenic exposure and intrauterine growth restriction: A systematic review and meta-analysis
Source: PLoS One. 2025 Jun 2;20(6):e0320603. doi: 10.1371/journal.pone.0320603 (PMC12129153; doi:10.1371/journal.pone.0320603)
Supplement: S8 Table — (PDF) [file pone.0320603.s009.pdf]

S8 Table. Summary of studies identified in literature search			
**Number**	**Author**	**Article title**	**Year**
1	S. S. Xu	Maternal Blood Levels of Toxic and Essential Elements and Birth Outcomes in Argentina: The EMASAR Study	2022
2	I. Khairul	Metabolism, toxicity and anticancer activities of arsenic compounds	2017
3	Z. V. Varga	Drug-induced mitochondrial dysfunction and cardiotoxicity	2015
4	T. S. Skogheim	Metal and essential element concentrations during pregnancy and associations with autism spectrum disorder and attention-deficit/hyperactivity disorder in children	2021
5	N. Medda	Different mechanisms of arsenic related signaling in cellular proliferation, apoptosis and neo-plastic transformation	2021
6	Not found	Arsenic Trioxide	2006
7	Y. W. Kim	Retraction: comparison of As2O3 and As4O6 in the detection of SiHa cervical cancer cell growth inhibition pathway	2007
8	J. Suhl	Pre-pregnancy dietary arsenic consumption among women in the United States	2020
9	J. M. Byun	Tetraarsenic oxide and cisplatin induce apoptotic synergism in cervical cancer	2013
10	S. E. Attreed	Arsenic and Immune Response to Infection During Pregnancy and Early Life	2017
11	C. Cochet	Arsenic Trioxide Treatment during Pregnancy for Acute Promyelocytic Leukemia in a 22-Year-Old Woman	2020
12	J. Kim	Retraction: tetraarsenic oxide-mediated apoptosis in a cervical cancer cell line, SiHa	2007
13	J. M. Byun	Arsenic trioxide and tetraarsenic oxide induce cytotoxicity and have a synergistic effect with cisplatin in paclitaxel-resistant ovarian cancer cells	2019
14	S. Gao	Gene-environment interaction and maternal arsenic methylation efficiency during pregnancy	2019
15	S. Gao	Determinants of arsenic methylation efficiency and urinary arsenic level in pregnant women in Bangladesh	2019
16	J. Jeong	Tetraarsenic oxide affects non-coding RNA transcriptome through deregulating polycomb complexes in MCF7 cells	2021

17	Z. Slejkovec	Arsenic trioxide versus tetraarsenic oxide in biomedical research: misunderstandings and misinterpretations	2012
18	C. G. Howe	Arsenic and birth outcomes in a predominately lower income Hispanic pregnancy cohort in Los Angeles	2020
19	L. Zhang	Arsenic Trioxide Suppressed Migration and Angiogenesis by Targeting FOXO3a in Gastric Cancer Cells	2018
20	H. S. Chang	Comparison of diarsenic oxide and tetraarsenic oxide on anticancer effects: relation to the apoptosis molecular pathway	2007
21	X. Wang	Arsenic exposure and metabolism in relation to blood pressure changes in pregnant women	2021
22	Z. Qiu	Sulfide and arsenic compounds removal from liquid digestate by ferric coagulation and toxicity evaluation	2019
23	X. Zhao	Arsenic Trioxide and Artemisinin Act Synergistically to Kill Tumor Cells In Vitro	2018
24	P. Valero	Exposome and foetoplacental vascular dysfunction in gestational diabetes mellitus	2021
25	S. H. Woo	Diarsenic and tetraarsenic oxide inhibit cell cycle progression and bFGF- and VEGF-induced proliferation of human endothelial cells	2005
26	K. Rehman	Double-edged effects of arsenic compounds: anticancer and carcinogenic effects	2013
27	Y. H. Shih	Associations between prenatal arsenic exposure with adverse pregnancy outcome and child mortality	2017
28	Y. H. Shih	Association between prenatal arsenic exposure, birth outcomes, and pregnancy complications: An observational study within the National Children's Study cohort	2020
29	H. Bhattacharjee	Drug uptake and pharmacological modulation of drug sensitivity in leukemia by AQP9	2004
30	R. G. Ahmed	Gestational Arsenic Trioxide Exposure Acts as a Developing Neuroendocrine-Disruptor by Downregulating Nrf2/PPARγ and Upregulating Caspase-3/NF-κB/Cox2/BAX/iNOS/ROS	2019
31	M. L. Rahman	Prenatal arsenic exposure, child marriage, and pregnancy weight gain: Associations with preterm birth in Bangladesh	2018
32	D. Douer	Arsenic trioxide (trisenox) therapy for acute promyelocytic leukemia in the setting of hematopoietic stem cell transplantation	2003
33	R. Quansah	Association of arsenic with adverse pregnancy outcomes/infant mortality: a systematic review and meta-analysis	2015
34	K. Z. Zhang	Arsenic trioxide induces differentiation of CD133+ hepatocellular carcinoma cells and prolongs posthepatectomy survival by targeting GLI1 expression in a mouse model	2014

35	M. S. Bloom	Spontaneous pregnancy loss in humans and exposure to arsenic in drinking water	2010
36	A. Nagappan	Tetraarsenic hexoxide induces G2/M arrest, apoptosis, and autophagy via PI3K/Akt suppression and p38 MAPK activation in SW620 human colon cancer cells	2017
37	P. D. Lin	Associations between Diet and Toenail Arsenic Concentration among Pregnant Women in Bangladesh: A Prospective Study	2017
38	M. A. Davis	Assessment of human dietary exposure to arsenic through rice	2017
39	E. P. Swindell	Anticancer activity of small-molecule and nanoparticulate arsenic(III) complexes	2013
40	W. Y. Au	Arsenic trioxide: safety issues and their management	2008
41	T. Y. Lai	Arsenic trioxide (As_2O_3) inhibits murine WEHI-3 leukemia in BALB/c mice in vivo	2012
42	Y. Fan	Arsenic trioxide and resveratrol show synergistic anti-leukemia activity and neutralized cardiotoxicity	2014
43	P. Baláž	Arsenic in cancer treatment: challenges for application of realgar nanoparticles (a minireview)	2010
44	C. Gao	Arsenic Trioxide Induces T Cell Apoptosis and Prolongs Islet Allograft Survival in Mice	2015
45	M. Izdebska	Effect of arsenic trioxide (Trisenox) on actin organization in K-562 erythroleukemia cells	2009
46	C. Liang	Low levels of arsenic exposure during pregnancy and maternal and neonatal thyroid hormone parameters: The determinants for these associations	2020
47	R. Eguchi	Arsenic trioxide induces apoptosis through JNK and ERK in human mesothelioma cells	2011
48	C. Osorio-Yáñez	Metal exposure and bone remodeling during pregnancy: Results from the PROGRESS cohort study	2021
49	M. S. Golub	Developmental and reproductive toxicity of inorganic arsenic: animal studies and human concerns	1998
50	C. D. Heaney	Arsenic exposure and hepatitis E virus infection during pregnancy	2015
51	A. V. Skalny	Hair Trace Element and Electrolyte Content in Women with Natural and In Vitro Fertilization-Induced Pregnancy	2018
52	Y. H. Liang	Arsenic trioxide regulates the production and activities of matrix metalloproteinases-1, -2, and -9 in fibroblasts and THP-1	2012

53	A. S. Ettinger	Arsenic levels among pregnant women and newborns in Canada: Results from the Maternal-Infant Research on Environmental Chemicals (MIREC) cohort	2017
54	P. La Rosée	In vitro studies of the combination of imatinib mesylate (Gleevec) and arsenic trioxide (Trisenox) in chronic myelogenous leukemia	2002
55	W. H. Chung	Synergistic interaction between tetra-arsenic oxide and paclitaxel in human cancer cells in vitro	2009
56	S. F. Farzan	Maternal arsenic exposure and gestational diabetes and glucose intolerance in the New Hampshire birth cohort study	2016
57	R. Rivera Carvajal	Mining leachate contamination and subfecundity among women living near the USA-Mexico border	2019
58	S. G. Park	Tetra-arsenic oxide (Tetras) enhances radiation sensitivity of solid tumors by anti-vascular effect	2009
59	A. Jarošíková	Characterization and pH-dependent environmental stability of arsenic trioxide-containing copper smelter flue dust	2018
60	B. Fu	Carbohydrate-conjugated 4-(1,3,2-dithiarsolan-2-yl)aniline as a cytotoxic agent against colorectal cancer	2018
61	D. M. Liu	Chloroquine aggravates the arsenic trioxide (As2O3)-induced apoptosis of acute promyelocytic leukemia NB4 cells via inhibiting lysosomal degradation in vitro	2018
62	A. Emadi	Arsenic trioxide - An old drug rediscovered	2010
63	X. Y. Zhao	Resveratrol and arsenic trioxide act synergistically to kill tumor cells in vitro and in vivo	2014
64	L. C. Platanias	Biological responses to arsenic compounds	2009
65	S. Y. Huan	Arsenic trioxide therapy for relapsed acute promyelocytic leukemia: an useful salvage therapy	2000
66	K. Laka	Survivin Splice Variants in Arsenic Trioxide (As_2O_3)-Induced Deactivation of PI3K and MAPK Cell Signalling Pathways in MCF-7 Cells	2019
67	S. Nafisi	Interaction of arsenic trioxide As2O3 with DNA and RNA	2005
68	M. J. Kim	Arsenic hexoxide enhances TNF-α-induced anticancer effects by inhibiting NF-κB activity at a safe dose in MCF-7 human breast cancer cells	2014
69	M. Vahter	Effects of arsenic on maternal and fetal health	2009
70	S. Miyashita	Biological effects and metabolism of arsenic compounds present in seafood products	2010

71	A. J. Signes-Pastor	Prenatal exposure to arsenic and lung function in children from the New Hampshire Birth Cohort Study	2021
72	G. D. A. Lima	Fertility in male rats: Disentangling adverse effects of arsenic compounds	2018
73	S. Xi	Distribution and speciation of arsenic by transplacental and early life exposure to inorganic arsenic in offspring rats	2010
74	A. Rahman	Arsenic exposure and risk of spontaneous abortion, stillbirth, and infant mortality	2010
75	W. S. Lee	Tetraarsenic hexoxide demonstrates anticancer activity at least in part through suppression of NF-κB activity in SW620 human colon cancer cells	2015
76	S. M. Ahmed	A prospective cohort study of in utero and early childhood arsenic exposure and infectious disease in 4- to 5-year-old Bangladeshi children	2020
77	O. Aksakal	Evaluation of arsenic trioxide genotoxicity in wheat seedlings using oxidative system and RAPD assays	2015
78	A. H. Milton	A Review of the Effects of Chronic Arsenic Exposure on Adverse Pregnancy Outcomes	2017
79	J. P. Galvin	Regulation of the kinase RSK1 by arsenic trioxide and generation of antileukemic responses	2013
80	H. Wang	Maternal serum arsenic level during pregnancy is positively associated with adverse pregnant outcomes in a Chinese population	2018
81	B. M. Welch	Arsenic exposure and serum antibody concentrations to diphtheria and tetanus toxoid in children at age 5: A prospective birth cohort in Bangladesh	2019
82	H. S. Gwak	Tetraarsenic oxide-induced inhibition of malignant glioma cell invasion in vitro via a decrease in matrix metalloproteinase secretion and protein kinase B phosphorylation	2014
83	M. Alemany	The effects of arsenic trioxide (As2O3) on human megakaryocytic leukemia cell lines. With a comparison of its effects on other cell lineages	2000
84	M. Vigeh	The relation of maternal blood arsenic to anemia during pregnancy	2015
85	K. H. Chu	Arsenic trioxide alleviates airway hyperresponsiveness and eosinophilia in a murine model of asthma	2010
86	Z. Y. Wang	Arsenic compounds as anticancer agents	2001
87	I. C. Park	Tetraarsenic oxide induces apoptosis in U937 leukemic cells through a reactive oxygen species-dependent pathway	2003
88	M. H. Yoo	Reverse effects of tetraarsenic oxide on the angiogenesis induced by nerve growth factor in the rat cornea	2004

89	M. T. Rojewski	Arsenic trioxide therapy in acute promyelocytic leukemia and beyond: from bench to bedside	2004
90	E. Hikita	Effects of inorganic and organic arsenic compounds on growth and apoptosis of human T-lymphoblastoid leukemia cells	2011
91	J. Majzlan	Arsenic-rich acid mine water with extreme arsenic concentration: mineralogy, geochemistry, microbiology, and environmental implications	2014
92	Z. Chen	Treatment of acute promyelocytic leukemia with arsenic compounds: in vitro and in vivo studies	2001
93	O. S. von Ehrenstein	Pregnancy outcomes, infant mortality, and arsenic in drinking water in West Bengal, India	2006
94	M. Valdés	Low-level arsenic exposure during pregnancy and its association with postpartum depression: A cohort study of women from Arica, Chile	2017
95	M. L. Kile	Estimating Effects of Arsenic Exposure During Pregnancy on Perinatal Outcomes in a Bangladeshi Cohort	2016
96	M. Hoonjan	Investigation of HSA as a biocompatible coating material for arsenic trioxide nanoparticles	2018
97	K. J. Chang	Arsenic trioxide inhibits cancer stem-like cells via down-regulation of Gli1 in lung cancer	2016
98	T. A. Samuel	Modulatory role of genistein on placenta and maternal bone minerals composition: further insight into its influence on pregnancy and foetal development	2020
99	R. L. Spehar	Comparative toxicity of arsenic compounds and their accumulation in invertebrates and fish	1980
100	L. Li	Protective effects of oxymatrine against arsenic trioxide-induced liver injury	2017
101	P. G. Richardson	New treatments for multiple myeloma	2005
102	S. Verstovsek	Arsenic derivatives in hematologic malignancies: a role beyond acute promyelocytic leukemia?	2006
103	D. E. Carter	The metabolism of inorganic arsenic oxides, gallium arsenide, and arsine: a toxicochemical review	2003
104	A. S. Ettinger	Maternal arsenic exposure and impaired glucose tolerance during pregnancy	2009
105	L. A. Held	A Phase I study of arsenic trioxide (Trisenox), ascorbic acid, and bortezomib (Velcade) combination therapy in patients with relapsed/refractory multiple myeloma	2013
106	L. Cui	Arsenic trioxide and promyelocytic leukemia protein-adenovirus synergistically inhibit in vitro and in vivo growth of a hepatoma cell line	2010

107	R. Uslu	Arsenic trioxide-mediated cytotoxicity and apoptosis in prostate and ovarian carcinoma cell lines	2000
108	C. Chen	Synergic effect of 3'-azido-3'-deoxythymidine and arsenic trioxide in suppressing hepatoma cells	2011
109	G. Baj	Arsenic trioxide and breast cancer: analysis of the apoptotic, differentiative and immunomodulatory effects	2002
110	A. Olfati	Riboflavin recovery of spermatogenic dysfunction via a dual inhibition of oxidative changes and regulation of the PINK1-mediated pathway in arsenic-injured rat model	2021
111	H. Lee	Inhibition of STAT3/VEGF/CDK2 axis signaling is critically involved in the antiangiogenic and apoptotic effects of arsenic herbal mixture PROS in non-small lung cancer cells	2017
112	M. Guo	Speciation analysis of arsenic in urine samples from APL patients treated with single agent As(2)O(3) by HPLC-HG-AFS	2019
113	R. Huang	[Effect of arsenic trioxide combined with adriamycin on the proliferation and apoptosis of human lymphoma cells]	2009
114	C. Sakai	Effects of arsenic compounds on growth, cell-cycle distribution and apoptosis of tretinoin-resistant human promyelocytic leukemia cells	2014
115	S. Kumar	Trisenox disrupts MDM2-DAXX-HAUSP complex and activates p53, cell cycle regulation and apoptosis in acute leukemia cells	2018
116	J. Tang	Arsenic trioxide induces expression of BCL-2 expression via NF-κB and p38 MAPK signaling pathways in BEAS-2B cells during apoptosis	2021
117	E. Y. Kim	Anticancer effect of arsenic trioxide on cholangiocarcinoma: in vitro experiments and in vivo xenograft mouse model	2014
118	T. Rudnai	Arsenic in drinking water and congenital heart anomalies in Hungary	2014
119	J. D. Hamadani	Pre- and postnatal arsenic exposure and child development at 18 months of age: a cohort study in rural Bangladesh	2010
120	M. L. Susko	Low-level arsenic exposure via drinking water consumption and female fecundity - A preliminary investigation	2017
121	A. M. Evens	The potential of arsenic trioxide in the treatment of malignant disease: past, present, and future	2004
122	W. S. Ahn	Comparison of effects of As2O3 and As4O6 on cell growth inhibition and gene expression profiles by cDNA microarray analysis in SiHa cells	2004
123	T. Bachleitner-Hofmann	Arsenic trioxide: acute promyelocytic leukemia and beyond	2002
124	M. L. Rahman	Investigating causal relation between prenatal arsenic exposure and birthweight: Are smaller infants more susceptible?	2017

125	Z. Y. Wang	Differentiation and apoptosis induction therapy in acute promyelocytic leukaemia	2000
126	A. Rahman	Association of arsenic exposure during pregnancy with fetal loss and infant death: a cohort study in Bangladesh	2007
127	J. Kim	Tetraarsenic oxide-mediated apoptosis in a cervical cancer cell line, SiHa	2005
128	Y. Fan	Theoretical Study of As_2O_3 Adsorption Mechanisms on CaO surface	2019
129	Y. Cheng	Neuroprotective effect of resveratrol on arsenic trioxide-induced oxidative stress in feline brain	2014
130	M. Baumgartner	Enhancement of arsenic trioxide-mediated apoptosis using docosahexaenoic acid in arsenic trioxide-resistant solid tumor cells	2004
131	A. Rahman	Arsenic exposure in pregnancy increases the risk of lower respiratory tract infection and diarrhea during infancy in Bangladesh	2011
132	Y. H. Du	Arsenic compounds induce cytotoxicity and apoptosis in cisplatin-sensitive and -resistant gynecological cancer cell lines	2001
133	R. Meng	Arsenic trioxide promotes mitochondrial DNA mutation and cell apoptosis in primary APL cells and NB4 cell line	2010
134	Y. Moon	Arsenic trioxide (As2O3) sensitivity of carcinoma cell lines and cancer cells from patients with carcinomatosis peritonei	2004
135	K. C. Nadeau	In utero arsenic exposure and fetal immune repertoire in a US pregnancy cohort	2014
136	J. Yu	Therapeutic effect of arsenic trioxide (As2O3) on cervical cancer in vitro and in vivo through apoptosis induction	2007
137	J. Mayorga	Arsenic trioxide as effective therapy for relapsed acute promyelocytic leukemia	2002
138	L. Kong	Chemical solidification/stabilization of arsenic sulfide and oxide mixed wastes using elemental sulfur: Efficiencies, mechanisms and long-term stabilization enhancement by dicyclopentadiene	2021
139	W. Zhang	Attenuation of arsenic retention by resveratrol in lung of arsenic trioxide-exposed rats	2013
140	F. V. Andrews	A prospective study of arsenic and manganese exposures and maternal blood pressure during gestation	2022
141	M. J. Park	Arsenic trioxide (As2O3) inhibits invasion of HT1080 human fibrosarcoma cells: role of nuclear factor-kappaB and reactive oxygen species	2005
142	Y. H. Zaw	Blood heavy metals and brain-derived neurotrophic factor in the first trimester of pregnancy among migrant workers	2019

143	S. H. Liu	Low-Concentration Arsenic Trioxide Inhibits Skeletal Myoblast Cell Proliferation via a Reactive Oxygen Species-Independent Pathway	2015
144	P. Zhang	The use of arsenic trioxide (As2O3) in the treatment of acute promyelocytic leukemia	1999
145	A. H. Milton	Association between chronic arsenic exposure and nutritional status among the women of child bearing age: a case-control study in Bangladesh	2010
146	H. B. Zhao	Efficacy of intratumoral chemotherapy using arsenic trioxide (As2O3) sustained release tablets for the treatment of neurogliocytoma in nude mice	2014
147	M. J. Park	Tetraarsenic oxide, a novel orally administrable angiogenesis inhibitor	2003
148	K. Ishitsuka	Therapeutic potential of arsenic trioxide with or without interferon-alpha for relapsed/refractory adult T-cell leukemia/lymphoma	2007
149	J. Bai	Taurine protects against As2O3-induced autophagy in livers of rat offsprings through PPARγ pathway	2016
150	I. Falnoga	Arsenic trioxide (ATO) influences the gene expression of metallothioneins in human glioblastoma cells	2012
151	C. Hopenhayn	Association between arsenic exposure from drinking water and anemia during pregnancy	2006
152	W. Zhou	Arsenic trioxide disrupts glioma stem cells via promoting PML degradation to inhibit tumor growth	2015
153	D. M. Loeb	What is the optimal therapy for childhood AML?	2002
154	H. Y. Karasulu	Preparation of arsenic trioxide-loaded microemulsion and its enhanced cytotoxicity on MCF-7 breast carcinoma cell line	2004
155	P. A. Guńka	How and Why Does Helium Permeate Nonporous Arsenolite Under High Pressure?	2018
156	X. Wu	Arsenic trioxide inhibits proliferation in K562 cells by changing cell cycle and survivin expression	2004
157	G. Q. Chen	Use of arsenic trioxide (As2O3) in the treatment of acute promyelocytic leukemia (APL): I. As2O3 exerts dose-dependent dual effects on APL cells	1997
158	X. Zhao	Up-regulation of miR-21 and miR-23a Contributes to As2 O3 -induced hERG Channel Deficiency	2015
159	M. Lu	Effect of arsenic trioxide on viability, proliferation, and apoptosis in human megakaryocytic leukemia cell lines	1999
160	X. He	Impact of receipt of private well arsenic test results on maternal use of contaminated drinking water in a U.S. population	2018

161	X. X. Chen	Overexpression of the long noncoding RNA NEAT1 protects against As2O3-induced injury of cardiomyocyte by inhibiting the miR-124/NF-κB signaling pathway	2020
162	J. Liu	Mineral arsenicals in traditional medicines: orpiment, realgar, and arsenolite	2008
163	J. Suhl	Pre-pregnancy exposure to arsenic in diet and non-cardiac birth defects	2022
164	A. Khaleghian	Metabolism of arsenic trioxide in acute promyelocytic leukemia cells	2014
165	M. S. de Assis Araujo	Maternal-child exposure to metals during pregnancy in Rio de Janeiro city, Brazil: The Rio Birth Cohort Study of Environmental Exposure and Childhood Development (PIPA project)	2020
166	L. Wei	[Toxicity of arsenic trioxide to human lung adenocarcinoma cell line SPCA1 and its mechanism]	2004
167	A. F. Fleisch	Arsenic exposure during pregnancy and postpartum maternal glucose tolerance: evidence from Bangladesh	2022
168	S. Selim	Soil enrichment with actinomycete mitigates the toxicity of arsenic oxide nanoparticles on wheat and maize growth and metabolism	2021
169	I. Falnoga	Effect of arsenic trioxide on metallothionein and its conversion to different arsenic metabolites in hen liver	2000
170	W. J. Zhang	[Arsenic trioxide restores ERα expression in ERα-negative human breast cancer cells and its treatment efficacy in combination with tamoxifen in xenografts in nude mice]	2012
171	Y. J. Kim	Arsenic Toxicity in Male Reproduction and Development	2015
172	J. A. Sans	Arsenolite: a quasi-hydrostatic solid pressure-transmitting medium	2016
173	Z. X. Shen	Use of arsenic trioxide (As2O3) in the treatment of acute promyelocytic leukemia (APL): II. Clinical efficacy and pharmacokinetics in relapsed patients	1997
174	K. L. Huyck	Maternal arsenic exposure associated with low birth weight in Bangladesh	2007
175	K. Broberg	Arsenic exposure in early pregnancy alters genome-wide DNA methylation in cord blood, particularly in boys	2014
176	A. Jarošíková	Transformation of arsenic-rich copper smelter flue dust in contrasting soils: A 2-year field experiment	2018
177	X. X. Geng	[Effects of As2O3 and all-trans retinoic acid on the growth of HeLa cell line and their relation with gene NDRG1]	2011
178	J. M. DeSesso	An assessment of the developmental toxicity of inorganic arsenic	1998

179	W. Shao	Arsenic trioxide as an inducer of apoptosis and loss of PML/RAR alpha protein in acute promyelocytic leukemia cells	1998
180	K. W. Liao	Associations between urinary total arsenic levels, fetal development, and neonatal birth outcomes: A cohort study in Taiwan	2018
181	H. Maeda	Tumor growth inhibition by arsenic trioxide (As2O3) in the orthotopic metastasis model of androgen-independent prostate cancer	2001
182	A. M. Florea	Arsenic trioxide in environmentally and clinically relevant concentrations interacts with calcium homeostasis and induces cell type specific cell death in tumor and non-tumor cells	2008
183	G. Q. Chen	In vitro studies on cellular and molecular mechanisms of arsenic trioxide (As2O3) in the treatment of acute promyelocytic leukemia: As2O3 induces NB4 cell apoptosis with downregulation of Bcl-2 expression and modulation of PML-RAR alpha/PML proteins	1996
184	G. R. Sahu	Significance of intracellular arsenic trioxide for therapeutic response in acute promyelocytic leukemia	2005
185	P. A. Bommarito	Fetal-sex dependent genomic responses in the circulating lymphocytes of arsenic-exposed pregnant women in New Hampshire	2017
186	D. Chau	Azacytidine sensitizes acute myeloid leukemia cells to arsenic trioxide by up-regulating the arsenic transporter aquaglyceroporin 9	2015
187	V. Charoensuk	Differential cytotoxic effects of arsenic compounds in human acute promyelocytic leukemia cells	2009
188	M. Kippler	Environmental exposure to arsenic and cadmium during pregnancy and fetal size: a longitudinal study in rural Bangladesh	2012
189	X. Cai	Arsenic trioxide-induced apoptosis and differentiation are associated respectively with mitochondrial transmembrane potential collapse and retinoic acid signaling pathways in acute promyelocytic leukemia	2000
190	P. Jia	Arsenic trioxide induces multiple myeloma cell apoptosis via disruption of mitochondrial transmembrane potentials and activation of caspase-3	2001
191	Y. Chen	Tetrandrine enhances the anticancer effects of arsenic trioxide in vitro	2014
192	X. Duan	The antitumor effect of arsenic trioxide on hepatocellular carcinoma is enhanced by andrographolide	2017
193	Y. Wu	Tetramethylpyrazine potentiates arsenic trioxide activity against HL-60 cell lines	2012
194	S. Chakraborty	Adsorption of arsenite and arsenate onto muscovite and biotite mica	2007
195	C. Chen	Arsenic trioxide co-exposure potentiates benzo(a)pyrene genotoxicity by enhancing the oxidative stress in human lung adenocarcinoma cell	2013
196	P. Wang	As2O3 synergistically reactivate latent HIV-1 by induction of NF-κB	2013

197	W. Zhang	The Protective Role of Resveratrol against Arsenic Trioxide-Induced Cardiotoxicity	2013
198	J. Hu	Localized Chemotherapy Prevents Lung Metastasis After Incomplete Microwave Ablation of Hepatic VX2 Tumor	2019
199	I. Falnoga	Arsenic metabolism in multiple myeloma and astrocytoma cells	2007
200	C. Zhou	Induction of apoptosis and inhibition of telomerase activity by arsenic trioxide (As2O3) in endometrial carcinoma cells	2007
201	S. Chen	Arsenic trioxide targets miR-125b in glioma cells	2014
202	M. Kumazaki	α-Lipoic acid protects against arsenic trioxide-induced acute QT prolongation in anesthetized guinea pigs	2013
203	A. Zebboudj	Sodium arsenite induces apoptosis and Epstein-Barr virus reactivation in lymphoblastoid cells	2014
204	Y. Zhang	Melatonin protects against arsenic trioxide-induced liver injury by the upregulation of Nrf2 expression through the activation of PI3K/AKT pathway	2017
205	C. Soriano	Gene-mutation induction by arsenic compounds in the mouse lymphoma assay	2007
206	R. Gardner	Persistent exposure to arsenic via drinking water in rural Bangladesh despite major mitigation efforts	2011
207	M. P. Waalkes	Mechanisms underlying arsenic carcinogenesis: hypersensitivity of mice exposed to inorganic arsenic during gestation	2004
208	M. Tuhý	Metal(loid)s remobilization and mineralogical transformations in smelter-polluted savanna soils under simulated wildfire conditions	2021
209	S. Chattopadhyay	Apoptosis and necrosis in developing brain cells due to arsenic toxicity and protection with antioxidants	2002
210	W. Xu	Downregulation of hTERT: an important As2O3 induced mechanism of apoptosis in myelodysplastic syndrome	2014
211	S. McDermott	When are fetuses and young children most susceptible to soil metal concentrations of arsenic, lead and mercury?	2012
212	G. Tabellini	Phosphoinositide 3-kinase/Akt inhibition increases arsenic trioxide-induced apoptosis of acute promyelocytic and T-cell leukaemias	2005
213	Z. Guo	The optimal dose of arsenic trioxide induced opposite efficacy in autophagy between K562 cells and their initiating cells to eradicate human myelogenous leukemia	2017
214	E. J. Tokar	Arsenic exposure in utero and nonepidermal proliferative response in adulthood in Tg.AC mice	2010

215	H. Liang	MicroRNAs contribute to promyelocyte apoptosis in As2O3-treated APL cells	2013
216	D. L. Pereira	Autophagy interplays with apoptosis and cell cycle regulation in the growth inhibiting effect of Trisenox in HEP-2, a laryngeal squamous cancer	2015
217	H. Konig	Enhanced Bcr-Abl-specific antileukemic activity of arsenic trioxide (Trisenox) through glutathione-depletion in imatinib-resistant cells	2007
218	W. Yang	Arsenic trioxide eluting stent reduces neointima formation in a rabbit iliac artery injury model	2006
219	G. J. Ahlborn	Impact of life stage and duration of exposure on arsenic-induced proliferative lesions and neoplasia in C3H mice	2009
220	G. L. Lu	Arsenic trioxide modulates the central snail neuron action potential	2009
221	D. Chakraborti	Arsenic contamination of groundwater and its induced health effects in Shahpur block, Bhojpur district, Bihar state, India: risk evaluation	2016
222	L. Shen	JWA enhances As_2O_3-induced tubulin polymerization and apoptosis via p38 in HeLa and MCF-7 cells	2011
223	B. Liu	Opposing effects of arsenic trioxide on hepatocellular carcinomas in mice	2006
224	A. Tarrade	Retinoic acid and arsenic trioxide cooperate for apoptosis through phosphorylated RXR alpha	2005
225	J. Pineda	Comparison between hepatic and renal effects in rats treated with arsenic and/or antioxidants during gestation and lactation	2013
226	S. A. Ahmad	Arsenic in drinking water and pregnancy outcomes	2001
227	X. Ding	Arsenic affects on cerebellar development of mice	2013
228	W. Zhang	The induction of apoptosis and cell cycle arrest by arsenic trioxide in lymphoid neoplasms	1998
229	M. T. Antonio Garcia	Hematological effects of arsenic in rats after subchronical exposure during pregnancy and lactation: the protective role of antioxidants	2013
230	Y. Cheng	Resveratrol ameliorates the oxidative damage induced by arsenic trioxide in the feline lung	2013
231	F. Y. Chen	Inorganic phosphate-triggered release of anti-cancer arsenic trioxide from a self-delivery system: an in vitro and in vivo study	2016
232	Y. Li	Construction of a BALB/c-Nu Mouse Model of Invasive Bladder Carcinoma and Preliminary Studies on the Treatment of Bladder Tumors through Internal Iliac Arterial Infusion of Albumin-Bound Arsenic Trioxide (As2O3)	2015

233	M. Fei	Arsenic trioxide-induced growth arrest of human hepatocellular carcinoma cells involving FOXO3a expression and localization	2009
234	C. C. Dang	Two successful deliveries of healthy children by a young woman diagnosed and treated during induction and relapsed therapy for acute promyelocytic leukemia	2020
235	F. F. Yuan	[Effects of As2O3 in combination with TPA on K562 cells]	2014
236	N. Sohel	Spatial patterns of fetal loss and infant death in an arsenic-affected area in Bangladesh	2010
237	M. Jeanne	PML/RARA oxidation and arsenic binding initiate the antileukemia response of As2O3	2010
238	L. L. Song	Targeting catalase but not peroxiredoxins enhances arsenic trioxide-induced apoptosis in K562 cells	2014
239	M. Yan	Mechanism of As2O3-Induced Action Potential Prolongation and Using hiPS-CMs to Evaluate the Rescue Efficacy of Drugs With Different Rescue Mechanism	2017
240	Y. Fan	Genistein ameliorates adverse cardiac effects induced by arsenic trioxide through preventing cardiomyocytes apoptosis	2013
241	W. Qian	Arsenic trioxide induces not only apoptosis but also autophagic cell death in leukemia cell lines via up-regulation of Beclin-1	2007
242	W. J. Liu	ATRA and As_2O_3 regulate differentiation of human hematopoietic stem cells into granulocyte progenitor via alteration of HoxB8 expression	2015
243	S. Lal	Brimstone chemistry under laser light assists mass spectrometric detection and imaging the distribution of arsenic in minerals	2018
244	R. M. Gardner	Arsenic methylation efficiency increases during the first trimester of pregnancy independent of folate status	2011
245	R. Raqib	Effects of in utero arsenic exposure on child immunity and morbidity in rural Bangladesh	2009
246	M. T. Rojewski	Arsenic trioxide-induced apoptosis is independent of CD95 in lymphatic cell lines	2004
247	F. Tofail	Effect of arsenic exposure during pregnancy on infant development at 7 months in rural Matlab, Bangladesh	2009
248	J. H. Baek	Arsenic trioxide induces depolymerization of microtubules in an acute promyelocytic leukemia cell line	2012
249	E. J. Tokar	Carcinogenic effects of "whole-life" exposure to inorganic arsenic in CD1 mice	2011
250	K. K. Mann	Arsenic trioxide decreases AKT protein in a caspase-dependent manner	2008

251	Y. Kobayashi	Distribution and excretion of arsenic in cynomolgus monkey following repeated administration of diphenylarsinic acid	2008
252	R. F. da Silva	The Coadministration of N-Acetylcysteine Ameliorates the Effects of Arsenic Trioxide on the Male Mouse Genital System	2016
253	K. K. Mann	Arsenic trioxide inhibits nuclear receptor function via SEK1/JNK-mediated RXRalpha phosphorylation	2005
254	S. Lehmann	Effects of arsenic trioxide (As2O3) on leukemic cells from patients with non-M3 acute myelogenous leukemia: studies of cytotoxicity, apoptosis and the pattern of resistance	2001
255	R. Polimanti	GSTO1 uncommon genetic variants are associated with recurrent miscarriage risk	2014
256	H. R. Lee	Sulindac enhances arsenic trioxide-mediated apoptosis by inhibition of NF-kappaB in HCT116 colon cancer cells	2008
257	J. Sen	Arsenic exposure through drinking water and its effect on pregnancy outcome in Bengali women	2008
258	M. T. Rojewski	Dual effects of arsenic trioxide (As2O3) on non-acute promyelocytic leukaemia myeloid cell lines: induction of apoptosis and inhibition of proliferation	2002
259	M. H. Han	Tetraarsenic Hexoxide Induces Beclin-1-Induced Autophagic Cell Death as well as Caspase-Dependent Apoptosis in U937 Human Leukemic Cells	2012
260	C. Scholz	Arsenic trioxide triggers a regulated form of caspase-independent necrotic cell death via the mitochondrial death pathway	2005
261	C. Soriano	Arsenic trioxide mutational spectrum analysis in the mouse lymphoma assay	2008
262	J. Y. Yeh	Differential influences of various arsenic compounds on glutathione redox status and antioxidative enzymes in porcine endothelial cells	2002
263	X. H. Zhu	Apoptosis and growth inhibition in malignant lymphocytes after treatment with arsenic trioxide at clinically achievable concentrations	1999
264	H. Shan	Upregulation of microRNA-1 and microRNA-133 contributes to arsenic-induced cardiac electrical remodeling	2013
265	X. S. Wang	[The effect of As2O3 on induction of apoptosis and inhibition of telomerase activity in colon cancer LS-174T cells]	2007
266	E. Goto	Missense mutations in PML-RARA are critical for the lack of responsiveness to arsenic trioxide treatment	2011
267	X. Li	Regeneration of Commercial SCR Catalysts: Probing the Existing Forms of Arsenic Oxide	2015
268	G. V. Gibbs	Role of long-range intermolecular forces in the formation of inorganic nanoparticle clusters	2011

269	S. F. Farzan	Blood Pressure Changes in Relation to Arsenic Exposure in a U.S. Pregnancy Cohort	2015
270	A. Lindgren	Embryotoxicity of arsenite and arsenate: distribution in pregnant mice and monkeys and effects on embryonic cells in vitro	1984
271	J. Leung	Relationship of expression of aquaglyceroporin 9 with arsenic uptake and sensitivity in leukemia cells	2007
272	Z. Ai	Arsenic oxide targets stem cell marker CD133/prominin-1 in gallbladder carcinoma	2011
273	Z. Šlejkovec	Exceptions in patterns of arsenic compounds in urine of acute promyelocytic leukaemia patients treated with As2O3	2016
274	T. Zhang	Pathologic, cytogenetic and molecular assessment of acute promyelocytic leukemia patients treated with arsenic trioxide (As2O3)	2000
275	X. Yang	Arsenic trioxide induced endoplasmic reticulum stress in laryngeal squamous cell line Hep-2 cells	2014
276	J. X. Hao	[As2O3 Up-regulates the Autophagy of RPMI 8226 Cells Induced by Beclin-1]	2016
277	L. Irvine	Monomethylarsonic acid and dimethylarsinic acid: developmental toxicity studies with risk assessment	2006
278	X. Wang	Essential role of cell cycle regulatory genes p21 and p27 expression in inhibition of breast cancer cells by arsenic trioxide	2011
279	S. H. Woo	Arsenic trioxide induces apoptosis through a reactive oxygen species-dependent pathway and loss of mitochondrial membrane potential in HeLa cells	2002
280	M. L. Kile	A prospective cohort study of the association between drinking water arsenic exposure and self-reported maternal health symptoms during pregnancy in Bangladesh	2014
281	S. Chattopadhyay	Arsenic induced changes in growth development and apoptosis in neonatal and adult brain cells in vivo and in tissue culture	2002
282	H. M. Pettersson	Arsenic trioxide is highly cytotoxic to small cell lung carcinoma cells	2009
283	C. Wang	The roles of mitoferrin-2 in the process of arsenic trioxide-induced cell damage in human gliomas	2014
284	V. Selvaraj	Arsenic trioxide (As(2)O(3)) induces apoptosis and necrosis mediated cell death through mitochondrial membrane potential damage and elevated production of reactive oxygen species in PLHC-1 fish cell line	2013
285	E. Saulle	In vitro dual effect of arsenic trioxide on hemopoiesis: inhibition of erythropoiesis and stimulation of megakaryocytic maturation	2006
286	P. Huang	Phenylarsine oxide (PAO) induces apoptosis in HepG2 cells via ROS-mediated mitochondria and ER-stress dependent signaling pathways	2017

287	V. F. Taylor	Exposure to arsenolipids and inorganic arsenic from marine-sourced dietary supplements	2022
288	Q. Li	Authentication of the 31 species of toxic and potent Chinese materia medica by microscopic technique assisted by ICP-MS analysis, part 4: four kinds of toxic and potent mineral arsenical CMMs	2011
289	R. Tingting	Arsenic trioxide inhibits osteosarcoma cell invasiveness via MAPK signaling pathway	2010
290	Z. M. Liu	As2O3-induced c-Src/EGFR/ERK signaling is via Sp1 binding sites to stimulate p21WAF1/CIP1 expression in human epidermoid carcinoma A431 cells	2006
291	G. Z. Pan	RUNX3 plays an important role in As2O3-induced apoptosis and allows cells to overcome MSC-mediated drug resistance	2016
292	J. Zheng	Arsenic trioxide induces apoptosis of HPV16 DNA-immortalized human cervical epithelial cells and selectively inhibits viral gene expression	1999
293	Y. L. Zhang	[Effects of As2O3 on the Proliferation, Differentiation and Apoptosis of HL-60 Cells and Its Related Mechanisms]	2015
294	S. Zhao	Effect of As2O3 on cell cycle progression and cyclins D1 and B1 expression in two glioblastoma cell lines differing in p53 status	2002
295	H. S. Huang	Opposite effect of ERK1/2 and JNK on p53-independent p21WAF1/CIP1 activation involved in the arsenic trioxide-induced human epidermoid carcinoma A431 cellular cytotoxicity	2006
296	E. Coe	Catalase activity and arsenic sensitivity in acute leukemia	2008
297	M. Omura	Testicular toxicity of gallium arsenide, indium arsenide, and arsenic oxide in rats by repetitive intratracheal instillation	1996
298	C. W. Du	[Arsenic trioxide induces differentiation of human nasopharyngeal carcinoma in BALB/C nude mice xenograft model]	2003
299	Y. H. Gao	Inactivation of Akt by arsenic trioxide induces cell death via mitochondrial-mediated apoptotic signaling in SGC-7901 human gastric cancer cells	2014
300	C. W. Siu	Effects of oral arsenic trioxide therapy on QT intervals in patients with acute promyelocytic leukemia: implications for long-term cardiac safety	2006
301	Y. Li	Simultaneous removal of SO2 and trace As2O3 from flue gas: mechanism, kinetics study, and effect of main gases on arsenic capture	2007
302	M. P. Waalkes	Induction of tumors of the liver, lung, ovary and adrenal in adult mice after brief maternal gestational exposure to inorganic arsenic: promotional effects of postnatal phorbol ester exposure on hepatic and pulmonary, but not dermal cancers	2004
303	J. Fang	Treatment of acute promyelocytic leukemia with ATRA and As2O3: a model of molecular target-based cancer therapy	2002
304	M. Ahmad	Effectiveness of zinc in modulating perinatal effects of arsenic on the teratological effects in mice offspring	2013

305	Y. J. Chun	Enhancement of radiation response in human cervical cancer cells in vitro and in vivo by arsenic trioxide (As2O3)	2002
306	Y. Su	Arsenic trioxide increases the sensitivity of 786-0 renal carcinoma cells to radiotherapy	2012
307	J. G. Seol	Potential role of caspase-3 and -9 in arsenic trioxide-mediated apoptosis in PCI-1 head and neck cancer cells	2001
308	K. Iwama	Apoptosis induced by arsenic trioxide in leukemia U937 cells is dependent on activation of p38, inactivation of ERK and the Ca2+-dependent production of superoxide	2001
309	P. S. Ong	Differential augmentative effects of buthionine sulfoximine and ascorbic acid in As2O3-induced ovarian cancer cell death: oxidative stress-independent and -dependent cytotoxic potentiation	2011
310	M. H. Yang	Arsenic trioxide restrains lung cancer growth and metastasis by blocking the calcineurin-NFAT pathway by upregulating DSCR1	2022
311	Z. Diaz	Trolox enhances the anti-lymphoma effects of arsenic trioxide, while protecting against liver toxicity	2007
312	X. H. Zhang	Arsenic trioxide induces apoptosis in B-cell chronic lymphocytic leukemic cells through down-regulation of survivin via the p53-dependent signaling pathway	2013
313	M. V. Varghese	Oxidative stress induced by the chemotherapeutic agent arsenic trioxide	2014
314	Y. S. Pu	Cytotoxicity of arsenic trioxide to transitional carcinoma cells	2002
315	O. Merkel	Arsenic trioxide induces apoptosis preferentially in B-CLL cells of patients with unfavourable prognostic factors including del17p13	2008
316	K. L. Munro	Microprobe XRF mapping and XAS investigations of the intracellular metabolism of arsenic for understanding arsenic-induced toxicity	2008
317	T. C. Zhang	Induction of apoptosis and inhibition of human gastric cancer MGC-803 cell growth by arsenic trioxide	1999
318	J. Zhou	Preparation of arsenic trioxide-loaded albuminutes immuno-nanospheres and its specific killing effect on bladder cancer cell in vitro	2005
319	M. Breccia	Pregnancy in acute promyelocytic leukaemia after front-line therapy with arsenic trioxide and all-trans retinoic acid	2014
320	S. Kumar	Trisenox induces cytotoxicity through phosphorylation of mitogen-activated protein kinase molecules in acute leukemia cells	2018
321	Y. H. Cheng	Glutathione regulation in arsenic-induced porcine aortic endothelial cells	2008
322	Z. Diaz	A novel arsenical has antitumor activity toward As2O3-resistant and MRP1/ABCC1-overexpressing cell lines	2008

323	J. Zhou	Various tolerances to arsenic trioxide between human cortical neurons and leukemic cells	2006
324	J. Dai	Malignant cells can be sensitized to undergo growth inhibition and apoptosis by arsenic trioxide through modulation of the glutathione redox system	1999
325	M. P. Waalkes	Animal models for arsenic carcinogenesis: inorganic arsenic is a transplacental carcinogen in mice	2004
326	S. Gu	Resveratrol synergistically triggers apoptotic cell death with arsenic trioxide via oxidative stress in human lung adenocarcinoma A549 cells	2015
327	X. Wang	Stathmin is involved in arsenic trioxide-induced apoptosis in human cervical cancer cell lines via PI3K linked signal pathway	2010
328	S. Y. Ho	Combination treatment with arsenic trioxide and irradiation enhances apoptotic effects in U937 cells through increased mitotic arrest and ROS generation	2009
329	A. Chatterjee	Determination of arsenic compounds by high-performance liquid chromatography-ultrasonic nebulizer-high power nitrogen-microwave-induced plasma mass spectrometry: an accepted coupling	2000
330	E. J. Tokar	Renal, hepatic, pulmonary and adrenal tumors induced by prenatal inorganic arsenic followed by dimethylarsinic acid in adulthood in CD1 mice	2012
331	Y. P. Yang	Dynamic effects of autophagy on arsenic trioxide-induced death of human leukemia cell line HL60 cells	2008
332	W. Tang	[Double effects of arsenic trioxide (As2O3) on acute promyelocytic leukemic cell line]	1997
333	F. Cuypers	Thermal behaviour of arsenic trioxide adsorbed on activated carbon	2009
334	F. Gao	Investigation of the mechanism involved in the As2O3-regulated decrease in MDR1 expression in leukemia cells	2014
335	I. Tanaka	[Arsenic metabolism. (17) Studies of placental transfer of arsenic and the effects of antidotes and diet]	1976
336	M. Guo	Arsenic Trioxide Therapy During Pregnancy: ATO and Its Metabolites in Maternal Blood and Amniotic Fluid of Acute Promyelocytic Leukemia Patients	2022
337	G. Concha	Exposure to inorganic arsenic metabolites during early human development	1998
338	K. Shinjo	Delayed recovery of normal hematopoiesis in arsenic trioxide treatment of acute promyelocytic leukemia: a comparison to all-trans retinoic acid treatment	2005
339	M. Huang	Quantification of arsenic compounds using derivatization, solvent extraction and liquid chromatography electrospray ionization tandem mass spectrometry	2008
340	J. R. Gurr	Dithiothreitol enhances arsenic trioxide-induced apoptosis in NB4 cells	1999

341	Y. W. Kim	Synergistic anti-tumor effects of combination of photodynamic therapy and arsenic compound in cervical cancer cells: in vivo and in vitro studies	2012
342	C. Hopenhayn	Profile of urinary arsenic metabolites during pregnancy	2003
343	Z. Zhang	Resveratrol, a natural antioxidant, has a protective effect on liver injury induced by inorganic arsenic exposure	2014
344	R. Srivastava	Differential in vivo genotoxicity of arsenic trioxide in glutathione depleted mouse bone marrow cells: expressions of Nrf2/Keap1/P62	2015
345	J. Zheng	Arsenic speciation in human urine reference materials using high-performance liquid chromatography with inductively coupled plasma mass spectrometric detection	1999
346	C. Brunet	Topographic distribution of arsenous anhydride in pregnant mice using macroscopic autoradiography	1983
347	V. L. Bae-Jump	Arsenic trioxide (As2O3) inhibits expression of estrogen receptor-alpha through regulation of the mitogen-activated protein kinase (MAPK) pathway in endometrial cancer cells	2008
348	M. Jang	Carbonyl reductase 1 offers a novel therapeutic target to enhance leukemia treatment by arsenic trioxide	2012
349	A. Petit	Importance of ERK activation in As2O3-induced differentiation and promyelocytic leukemia nuclear bodies formation in neuroblastoma cells	2013
350	M. S. Bloom	Consumption of low-moderate level arsenic contaminated water does not increase spontaneous pregnancy loss: a case control study	2014
351	X. R. Huang	Effect of Rhizoma Curcumae and arsenite trioxide on proliferation and signal transduction molecule of lens epithelial cell	2007
352	A. Herrera	Toxic effects of perinatal arsenic exposure on the brain of developing rats and the beneficial role of natural antioxidants	2013
353	S. Gibaud	Organoarsenicals derived from 2-phenyl-[1,3,2]dithiarsolan-4-yl)-methanol (AsIII) with antileukaemic properties: from trypanosomicides to anticancer drugs	2007
354	X. Dong	Effects of As(2)O(3) nanoparticles on cell growth and apoptosis of NB4 cells	2015
355	M. Benbijja	Sensitivity of leukemic T-cell lines to arsenic trioxide cytotoxicity is dependent on the induction of phosphatase B220/CD45R expression at the cell surface	2014
356	S. K. Chow	Inhibition of cell proliferation and the action mechanisms of arsenic trioxide (As2O3) on human breast cancer cells	2004
357	V. Lecureur	Potassium antimonyl tartrate induces reactive oxygen species-related apoptosis in human myeloid leukemic HL60 cells	2002
358	X. Tian	mCICR is required for As2O3-induced permeability transition pore opening and cytochrome c release from mitochondria	2005

359	V. Lallemand -	Role of promyelocytic leukemia (PML) sumolation in nuclear body formation, 11S proteasome recruitment, and As2O3-induced PML or PML/retinoic acid receptor alpha degradation	2001
360	K. Kitamura	Involvement of CD95-independent caspase 8 activation in arsenic trioxide-induced apoptosis	2000
361	H. Zhang	Mechanism of arsenic trioxide induced apoptosis in cultured human lens epithelium cells	2008
362	M. A. Davis	Preliminary analysis of in utero low-level arsenic exposure and fetal growth using biometric measurements extracted from fetal ultrasound reports	2015
363	M. J. Yu	Inhibited proliferation of B-lymphoma Raji cells and down-regulated expression of VEGF by arsenic trioxide	2006
364	R. Di Noto	In vitro exposure of acute promyelocytic leukemia cells to arsenic trioxide (As2O3) induces the solitary expression of CD66c (NCA-50/90), a member of the CEA family	1999
365	S. McDermott	Does the metal content in soil around a pregnant woman's home increase the risk of low birth weight for her infant?	2014
366	X. Huang	Potentiation of arsenic trioxide-induced apoptosis by retinoic acid in retinoic acid sensitive and resistant HL-60 myeloid leukemia cells	2000
367	S. Datta	Efficacy of a potentized homoeopathic drug (Arsenicum Album-30) in reducing genotoxic effects produced by arsenic trioxide in mice: II. Comparative efficacy of an antibiotic, actinomycin D alone and in combination with either of two microdoses	1999
368	A. L. Williams	Comment on "Effects of in Utero Exposure to Arsenic during the Second Half of Gestation on Reproductive End Points and Metabolic Parameters in Female CD-1 Mice"	2016
369	A. Yamamoto	Tumorigenicity of inorganic arsenic compounds following intratracheal instillations to the lungs of hamsters	1987
370	J. M. DeSesso	Teratogen update: inorganic arsenic	2001
371	W. Glienke	Down-regulation of wt1 expression in leukemia cell lines as part of apoptotic effect in arsenic treatment using two compounds	2006
372	L. Jiang	As2O3 induces apoptosis in human hepatocellular carcinoma HepG2 cells through a ROS-mediated mitochondrial pathway and activation of caspases	2015
373	J. B. Che	Influence of As2O3 combined with ginsenosides Rg3 on inhibition of lung cancer NCI-H1299 cells and on subsistence of nude mice bearing hepatoma	2014
374	S. Xu	Multidrug resistance protein 1 (ABCC1) confers resistance to arsenic compounds in human myeloid leukemic HL-60 cells	2013
375	J. Zhou	Effects of administration styles of arsenic trioxide on intracellular arsenic concentration, cell differentiation and apoptosis	2005
376	H. Duzkale	In vitro activity of dimethylarsinic acid against human leukemia and multiple myeloma cell lines	2003

377	T. Seo	Arsenic trioxide circumvents multidrug resistance based on different mechanisms in human leukemia cell lines	2005
378	M. E. Vahter	Arsenic exposure in pregnancy: a population-based study in Matlab, Bangladesh	2006
379	Y. Wei	Down-regulation of beta1,4GalT V at protein level contributes to arsenic trioxide-induced glioma cell apoptosis	2008
380	R. Walter	Establishment and characterization of an arsenic-sensitive monoblastic leukaemia cell line (SigM5)	2000
381	X. Xu	Effects of As2O3 on tissue factor, plasminogen activator inhibitor-1 and -2 expression in NB4, HL-60 and THP-1 cells	2003
382	M. Kojima	Refractory acute promyelocytic leukemia successfully treated with combination therapy of arsenic trioxide and tamibarotene: A case report	2016
383	M. T. Rojewski	Depolarization of mitochondria and activation of caspases are common features of arsenic(III)-induced apoptosis in myelogenic and lymphatic cell lines	2004
384	E. Puccetti	BCR-ABL mediates arsenic trioxide-induced apoptosis independently of its aberrant kinase activity	2000
385	J. D. Hamadani	Critical windows of exposure for arsenic-associated impairment of cognitive function in pre-school girls and boys: a population-based cohort study	2011
386	J. T. Kloprogge	Nondestructive identification of arsenic and cobalt minerals from Cobalt city, Ontario, Canada: arsenolite, erythrite, and spherocobaltite on pararammelsbergite	2006
387	L. S. Xu	Preparation of As2O3 nanoparticles and its drug release characteristics in vitro	2010
388	M. P. Waalkes	Transplacental carcinogenicity of inorganic arsenic in the drinking water: induction of hepatic, ovarian, pulmonary, and adrenal tumors in mice	2003
389	M. Lu	Dual effects of glutathione-S-transferase pi on As2O3 action in prostate cancer cells: enhancement of growth inhibition and inhibition of apoptosis	2004
390	X. X. Wu	Enhancement of arsenic trioxide-induced apoptosis in renal cell carcinoma cells by L-buthionine sulfoximine	2004
391	K. Minakata	Electrospray ionization tandem mass spectrometric determination of monomethylarsonic acid and dimethylarsinic acid after adduct formation with citric acid	2009
392	M. Q. Fan	Effects of arsenic on nerve growth factor and nerve growth related mRNA expression in F1 hippocampal	2013
393	M. Ohsawa	Arsenic trioxide (As2O3) gradually downregulates tissue factor expression without affecting thrombomodulin expression in acute promyelocytic leukemia cells	2000
394	G. Marinova	Problems of interrupted pregnancy among working women	1978

395	J. Liu	Toxicogenomic analysis of aberrant gene expression in liver tumors and nontumorous livers of adult mice exposed in utero to inorganic arsenic	2004
396	C. S. Dubey	Anthropogenic arsenic menace in contaminated water near thermal power plants and coal mining areas of India	2022
397	H. Joshi	Arsenic contamination in parts of Yamuna sub-basin, West Bengal	2003
398	L. Nagymajtényi	Chromosomal aberrations and fetotoxic effects of atmospheric arsenic exposure in mice	1985
399	L. Berthoux	As2O3 enhances retroviral reverse transcription and counteracts Ref1 antiviral activity	2003
400	P. A. Guńka	Raman studies of hydrogen trapped in As(4)O(6)·2H(2) at high pressure and low temperature	2020
401	G. J. Wang	Therapeutic effects of combination of arsenic trioxide with low-dose all-trans retinoic acid on induction of remission acute promyeloeytic leukemia	2005
402	Y. Akao	Arsenic trioxide induces apoptosis in neuroblastoma cell lines through the activation of caspase 3 in vitro	1999
403	J. Tan	Effect of arsenic trioxide on bone marrow stromal cells of patients with multiple myeloma	2006
404	M. Tajima	Pharmacological and clinical properties of arsenic trioxide (Trisenox) for relapse or refractory acute promyelocytic leukemia therapy	2005
405	H. L. Yang	Plasma protein C activity is enhanced by arsenic but inhibited by fluorescent humic acid associated with blackfoot disease	1994
406	Y. B. Chen	Mechanism of arsenic trioxide-induced cytotoxicity on multiple myeloma cells	2003
407	P. Naumov	Direct atomic scale observation of linkage isomerization of As4S4 clusters during the photoinduced transition of realgar to pararealgar	2007
408	Y. Liu	The relationship between mental retardation and developmental delays in children and the levels of arsenic, mercury and lead in soil samples taken near their mother's residence during pregnancy	2010
409	J. Zhang	High expression of bcl-x(L) in K562 cells and its role in the low sensitivity of K562 to realgar-induced apoptosis	2005
410	J. L. Hu	[Induced RAS association domain family gene 1A gene expression by arsenic trioxide in nasopharyngeal carcinoma cell]	2009
411	Z. Zhu	Effect of Ad-p16 combined with CDDP or As2O3 on human bladder cancer cells	2003
412	R. A. Jadhav	Capture of gas-phase arsenic oxide by lime: kinetic and mechanistic studies	2001

413	G. Chen	Preliminary study on the arsenic trioxide-induced NB4 cell apoptosis and its molecular mechanisms	1997
414	G. H. Fu	As2O3 enhances the anion transport activity of band 3 and the action is related with the C-terminal 16 residues of the protein	2005
415	D. Xiao	Effects of As2O3 on the BCR/ABL protein tyrosine phosphorylation in K562 cells	1999
416	M. Filippi	Oxidation of the arsenic-rich concentrate at the Prebuz abandoned mine (Erzgebirge Mts., CZ): mineralogical evolution	2004
417	S. Bernardini	Role of GSTP1-1 in mediating the effect of As2O3 in the Acute Promyelocytic Leukemia cell line NB4	2006
418	L. Li	New insights into the deactivation mechanism of V(2)O(5)-WO(3)/TiO(2) catalyst during selective catalytic reduction of NO with NH(3): synergies between arsenic and potassium species	2019
419	M. M. Wang	Correlation of cell cycle alteration to SOCS-1 gene demethylation induced by arsenic trioxide in myeloma cell lines	2008
420	G. V. Gibbs	Role of directed van der Waals bonded interactions in the determination of the structures of molecular arsenate solids	2009
421	T. Bachleitner-Hofmann	Arsenic trioxide and ascorbic acid: synergy with potential implications for the treatment of acute myeloid leukaemia?	2001
422	Y. W. Kim	Comparison of As(2)O(3) and As(4)O(6) in the detection of SiHa cervical cancer cell growth inhibition pathway	2004
423	J. F. Holson	Absence of prenatal developmental toxicity from inhaled arsenic trioxide in rats	1999
424	X. Huang	Inhibitory effect of arsenic trioxide combined with cisplatin on human nasopharyngeal carcinoma xenograft and DAPK in nude mice	2013
425	C. Zhang	Toxic effect of fluoride-arsenic on the reproduction and development of rats	2000
426	Z. H. Wang	Proteomic analysis of nuclear matrix proteins during arsenic trioxide induced apoptosis in leukemia K562 cells	2005
427	T. M. Allen	Speciation of arsenic oxides using laser desorption/ionization time-of-flight mass spectrometry	1996
428	B. H. Jung	Arsenic Trioxide Induces Apoptosis of HL-60 Cells via Activation of Intrinsic Caspase Protease with Mitochondrial Dysfunction	2002
429	Z. Ai	Arsenic trioxide induces gallbladder carcinoma cell apoptosis via downregulation of Bcl-2	2006
430	M. Fukuda	Oral administration of arsenic trioxide induced molecular remission in relapsed acute promyelocytic leukemia	2005

431	Y. Su	A study on apoptosis induced by As_2O_3 for small cell lung cancer	2001
432	M. L. Fascineli	Fetotoxicity caused by the interaction between zinc and arsenic in mice	2002
433	W. Y. Au	Combined arsenic trioxide and all-trans retinoic acid treatment for acute promyelocytic leukaemia recurring from previous relapses successfully treated using arsenic trioxide	2002
434	Y. Tanaka	[Successful treatment of relapsed and refractory acute promyelocytic leukemia with arsenic trioxide (As2O3)]	2000
435	K. Agarwal	A Complicated Case of Acute Promyelocytic Leukemia in the Second Trimester of Pregnancy Successfully Treated with All-trans-Retinoic Acid	2015
436	S. Tabacova	Complications of pregnancy in relation to maternal lipid peroxides, glutathione, and exposure to metals	1994
437	Y. Long	Apoptosis and cell cycle arrest in lymphoma Raji cells induced by arsenic trioxide	2008
438	D. M. Li	Role of arsenolite on 8-isoprostane of asthmatic mice plasm	2005
439	R. W. Hedges	Resistance to arsenic compounds conferred by a plasmid transmissible between strains of Escherichia coli	1973
440	J. Y. Yeh	Modulation of the arsenic effects on cytotoxicity, viability, and cell cycle in porcine endothelial cells by selenium	2003
441	B. Zhang	Role of Survivin gene on the apoptosis of adenoid cystic carcinoma-2 cells induced by arsenic trioxide	2010
442	J. L. Durant	Elevated levels of arsenic in the sediments of an urban pond: sources, distribution and water quality impacts	2004
443	K. R. Lowry	Amelioration of selenium toxicity by arsenicals and cysteine	1989
444	T. Yoshida	Immunological effects of arsenic compounds on mouse spleen cells in vitro	1986
445	T. Yamano	Acute pancreatitis during the treatment of relapsed acute promyelocytic leukemia with As2O3	2006
446	M. H. Wu	Direct cardiac effects of As2O3 in rabbits: evidence of reversible chronic toxicity and tissue accumulation of arsenicals after parenteral administration	2003
447	R. J. Griffin	Use of a fluorescently labeled poly-caspase inhibitor for in vivo detection of apoptosis related to vascular-targeting agent arsenic trioxide for cancer therapy	2007
448	S. Tsutsumi	Metabolism of arsenic (15). Influence of arsenic antidotes on intestinal absorption of arsenic trioxide	1975

449	G. C. Chen	Functional repression of estrogen receptor a by arsenic trioxide in human breast cancer cells	2002
450	G. Hunder	Influence of inorganic and organic arsenicals on intestinal transfer of nutrients	1993
451	S. F. Shen	Mechanism of As(2)O(3) on hdpr1 gene demethylation in Jurkat cell line	2010
452	X. Huang	Regulation of arsenic trioxide-inducing apoptosis	1999
453	S. Körper	The role of mitochondrial targeting in arsenic trioxide-induced apoptosis in myeloid cell lines	2004
454	L. Shen	As2O3 induces apoptosis of the human B lymphoma cell line MBC-1	2000
455	A. Kamil'dzhanov	Combined effects of arsenous anhydride and germanium dioxide after their hygienic regulation in atmospheric air	1986
456	D. Xie	Arsenic trioxide (As(2)O(3)) induced apoptosis and its mechanisms in a human esophageal squamous carcinoma cell line	2002
457	G. Marras	Proposal for the utilization of sodium monosulfide as an antidote in arsenous anhydride poisoning in the rabbit by gastric and subcutaneous administration	1951
458	X. H. Zhang	Relationship between drug resistance and the expression of NF-kappaB induced in leukemic cells	2004
459	A. Kamil'dzhanov	Experimental research to study the combined action of arsenous anhydride and lead acetate in their hygienic regulation in the atmosphere	1986
460	D. Xiao	Protein tyrosine kinase (PTK) activities during the induction of apoptosis by arsenic trioxide (As2O3)	1998
461	J. T. van Elteren	Ion-exchange separation of eight arsenic compounds by high-performance liquid chromatography-UV decomposition-hydride generation-atomic fluorescence spectrometry and stability tests for food treatment procedures	1997
462	A. Marmasse	Supplement to a 1st plea on behalf of arsenous anhydride. Is the condemnation of arsenic unjust?	1965
463	P. Marcovigi	Acute poisoning from arsenous anhydride ingestion. A clinical case	1993
464	Bergouignan	Arsenic Encephalitis and Pregnancy	1945
465	Y. Zhou	Mechanisms of arsenic trioxide-induced apoptosis in myeloma cells	2001
466	D. Li	Inhibition of growth of human nasopharyngeal cancer xenografts in SCID mice by arsenic trioxide	2002

467	W. Tang	In vitro study on arsenic trioxide-inducing apoptosis in primary acute promyelocytic leukemie cells	1997
468	C. Pupp	Equilibrium vapour concentrations of some polycyclic aromatic hydrocarbons, As4O6 and SeO2 and the collection effiencies of these air pollutants	1974
469	S. Tsutsumi	Effects of arsenic trioxide (As2O3) on the uptake of 3H-thymidine in lymphocytes of mice	1980
470	S. Tamura	Effect of arsenic on the developing brain of rat (author's transl)	1978
471	K. Basmadzhieva	Carcinogenic action of diarsenic trioxide	1977
472	G. M. Carminati	Synergism between arsenic oxide and streptomycin in experimental infection with Borrelia duttoni	1956
473	Y. Shen	Glutathione synthesis inhibitor enhances arsenic trioxide-induced apoptosis	1999
474	K. C. Li	The collection of arsenic oxide vapor in water-filled impingers	1979
475	T. P. Elias	An in vitro study of the effect of arsenic (As2O3) on blood clotting	1969
476	I. Kawaguchi	Studies on As2O3-induced hyperglycemia (author's transl)	1981
477	H. Kojima	Developmental pharmacology of arsenic. 2. Effect of arsenic on pregnancy, nutritional state and hard tissue	1974
478	J. Zheng	The retention behavior of arsenic compounds on PRP-X100 column under alkaline condition	1997
479	F. Pasquinelli	Influence of some inhibitors (As2O3 and HCN) on the amino acid oxidase of the Staphylococcus albus	1951
480	J. Pagin	To complete the action of arsenous anhydride. Apropos of a recent work	1967
481	A. Lambusta	Effect of arsenic poisoning on the course of pregnancy; experimental research	1952
482	F. Cavaliere	Acute poisoning with arsenous anhydride	1985
483	D. Bradley	Therapeutic needs revive arsenic compound	2000
484	I. S. Rozenshteĭn	Medico-toxicologic assessment of low concentrations of arsenous anhydride in the air	1970

485	A. Kamil'dzhanov	Hygienic establishment of the maximum permissible concentration of arsenous anhydride in the atmosphere	1982
486	G. Moretto	Experimental studies of the possibility of preventing the toxic effect of arsenous anhydride of inner ear structures by use of 2,3-dimercapropopanol (B.A.L.)	1958
487	W. A. Watson	Acute arsenic exposure treated with oral D-penicillamine	1981
488	W. L. Sheng	The Antisense Oligodeoxynucleotides of bcl-2 Oncogene Enhances the Sensitivity of K562 Leukemic Cells to As(2)O(3)	2001
489	C. Portugal	Hazards of arsenous anhydride in dental chemical devitalization. Apropos of a clinical case	1982
490	I. Di Sacco	A case of fetal arsenous anhydride poisoning	1980
491	X. W. Qian	Studies of cytogenetic toxic effect of As2O3 on Vicia Faba root tip cells in vivo	2002
492	T. Nagira	Two cases of lung cancer associated with chronic arsenic poisoning in an arsenic oxide (As2O3) refinery	1985
493	J. Brock	Use of an arsenic oxide in blood transfusion in pediatric clinics to prevent transfusion syphilis	1950
494	I. Francesco	Comparative observations on the changes in the vaginal bacterial flora in pregnant women during topical treatment with various drugs	1961
495	A. Sadowsky	Post-arsphenamin jaundice in pregnancy	1945
496	G. Giraud	Spasmodic paraplegia by arsenotherapy, in a 7 month pregnant woman	1948
497	M. Fallani	Behavior of adenosinetriphosphatasic activity in acute experimental arsenious anhydride poisoning (As2O3)	1955
498	R. Lees	Arsenical encephalopathy occurring in the treatment of lues during pregnancy	1950
499	H. I. Kantor	Arsenical encephalopathy in pregnancy with recovery	1948
500	J. S. Sambhi	THE VALUE AND MANAGEMENT OF PERSISTENT KAHN POSITIVE IN TREATED SYPHILIS	1963
501	G. Tabellini	Phosphoinositide 3-kinase/Akt involvement in arsenic trioxide resistance of human leukemia cells	2005
502	H. Maeda	Effective treatment of advanced solid tumors by the combination of arsenic trioxide and L-buthionine-sulfoximine	2004

503	A. Wang	Reproductive and developmental toxicity of arsenic in rodents: a review	2006
504	A. M. Florea	Arsenic trioxide (As2O3) induced calcium signals and cytotoxicity in two human cell lines: SY-5Y neuroblastoma and 293 embryonic kidney (HEK)	2007
505	X. Huang	Effects of arsenic trioxide combined with cisplatin on the growth of human nasopharyngeal carcinoma cells and reversion of RASSF1A hympermethylation	2014
506	X. Jiang	Sodium arsenite and arsenic trioxide differently affect the oxidative stress, genotoxicity and apoptosis in A549 cells: an implication for the paradoxical mechanism	2013
507	A. Baysan	Arsenic trioxide induces apoptosis via the mitochondrial pathway by upregulating the expression of Bax and Bim in human B cells	2007
508	K. F. Rodriguez	Response to "Comment on 'Effects of in Utero Exposure to Arsenic during the Second Half of Gestation on Reproductive End Points and Metabolic Parameters in Female CD-1 Mice'"	2016
509	K. M. Kodigepalli	SnoN/SkiL expression is modulated via arsenic trioxide-induced activation of the PI3K/AKT pathway in ovarian cancer cells	2013
510	L. Liu	Low dose of arsenic trioxide inhibits multidrug resistant-related P-glycoprotein expression in human neuroblastoma cell line	2016
511	Y. Shi	Inhibitory mechanism on growth of MA-891 cells by arsenic trioxide	2012
512	M. Wirtitsch	Omega-3 and omega-6 polyunsaturated fatty acids enhance arsenic trioxide efficacy in arsenic trioxide-resistant leukemic and solid tumor cells	2009
513	A. Sampayo-Reyes	Tocopherol and selenite modulate the transplacental effects induced by sodium arsenite in hamsters	2017
514	K. Ning	Effect of As(2)O(3) on colorectal CSCs stained with ALDH1 in primary cell culture in vitro	2018
515	A. Tomita	Mechanisms of action and resistance to all-trans retinoic acid (ATRA) and arsenic trioxide (As2O 3) in acute promyelocytic leukemia	2013
516	T. C. Hour	Characterization of molecular events in a series of bladder urothelial carcinoma cell lines with progressive resistance to arsenic trioxide	2004
517	J. Zhu	Arsenic trioxide induces apoptosis in leukemia/lymphoma cell lines via the CD95/CD95L system	2003
518	D. P. Hanlon	Concentration and chemical status of arsenic in the blood of pregnant hamsters during critical embryogenesis. 2. Acute exposure	1986
519	F. Yuan	The inhibitory effect of As_2O_3 combined with phorbol ester on the proliferation of Kasumi-1 cells and its mechanism	2014
520	T. Kanzawa	Induction of autophagic cell death in malignant glioma cells by arsenic trioxide	2003

521	K. Kinjo	Arsenic trioxide (As2O3)-induced apoptosis and differentiation in retinoic acid-resistant acute promyelocytic leukemia model in hGM-CSF-producing transgenic SCID mice	2000
522	R. K. Kwok	A review and rationale for studying the cardiovascular effects of drinking water arsenic in women of reproductive age	2007
523	E. Puccetti	Leukemia-associated translocation products able to activate RAS modify PML and render cells sensitive to arsenic-induced apoptosis	2003
524	Y. Deng	Overexpression of Bcl-2 partly inhibits apoptosis of human cervical cancer SiHa cells induced by arsenic trioxide	2000
525	R. F. Lin	Effects of As2O3, dexamethasone and thalidomide on apoptosis and cytoplasmic [Ca2+] of myeloma cell line U266	2007
526	M. Giannì	Combined arsenic and retinoic acid treatment enhances differentiation and apoptosis in arsenic-resistant NB4 cells	1998
527	H. Zhu	Role of cofilin-1 in arsenic trioxide-induced apoptosis of NB4-R1 cells	2020
528	H. Zhang	Prevention of posterior capsule opacification by combination of arsenic trioxide and perfect capsule	2010
529	X. Wang	Inhibition on LS-174T cell growth and activity of telomerase in vitro and in vivo by arsenic trioxide	2008
530	S. K. Bergstrom	Arsenic trioxide in the treatment of a patient with multiply recurrent, ATRA-resistant promyelocytic leukemia: a case report	1998
531	S. Surdu	Consumption of arsenic-contaminated drinking water and anemia among pregnant and non-pregnant women in northwestern Romania	2015
532	X. Zhang	The impact of arsenic trioxide or all-trans retinoic acid treatment on coagulopathy in acute promyelocytic leukemia	2001
533	H. Li	Arsenic trioxide exerts synergistic effects with cisplatin on non-small cell lung cancer cells via apoptosis induction	2009
534	Y. Li	The toxicity of combination of selenium, fluoride and arsenic on rat embryos	1999
535	M. Mazumdar	Arsenic is associated with reduced effect of folic acid in myelomeningocele prevention: a case control study in Bangladesh	2015
536	Q. S. Shao	Cell cycle arrest and apoptotic cell death in cultured human gastric carcinoma cells mediated by arsenic trioxide	2005
537	X. Q. Liang	P53-induced gene 11 (PIG11) involved in arsenic trioxide-induced apoptosis in human gastric cancer MGC-803 cells	2003
538	D. Luo	Low dosage of arsenic trioxide (As(2)O(3)) inhibits angiogenesis in epithelial ovarian cancer without cell apoptosis	2018

539	W. Guo	Effect of all-trans retinoic acid and arsenic trioxide on tissue factor expression in acute promyelocytic leukemia cells	2001
540	T. Punshon	Placental arsenic concentrations in relation to both maternal and infant biomarkers of exposure in a US cohort	2015
541	P. Sengupta	Metals and female reproductive toxicity	2015
542	H. Liu	Radiosensitizing effects of arsenic trioxide on MCF-7 human breast cancer cells exposed to 89 strontium chloride	2012
543	C. N. Monty	Non-biological inhibition-based sensing (NIBS) demonstrated for the detection of toxic arsenic compounds	2011
544	Y. Xu	The effects of ultrasound and arsenic trioxide on neurogliocytoma cells and secondary activation of macrophages	2009
545	A. H. Milton	Chronic arsenic exposure and adverse pregnancy outcomes in bangladesh	2005
546	M. Goldman	Inorganic arsenic compounds: are they carcinogenic, mutagenic, teratogenic?	1991
547	B. R. Liu	A study on the apoptosis of gastric carcinoma cells induced by arsenic trioxide combined with Ad-IkappaBalphaM	2007
548	M. H. Salmani	Arsenic exposure to breast-fed infants: contaminated breastfeeding in the first month of birth	2018
549	D. Nasiry Zarrin Ghabaee	Administration of zinc against arsenic-induced nephrotoxicity during gestation and lactation in rat model	2017
550	X. Zhao	The rescuable function and mechanism of resveratrol on As_2O_3-induced hERG K^+ channel deficiency	2014
551	M. Kawaguchi	Studies on As2O3-induced rabbit hypothermia and brain monoamines (author's transl)	1980
552	S. G. Huang	Primary study of arsenic trioxide inhibits abdomino-metastatic tumor formation of human ovarian carcinoma in nude mice and its mechanisms	2002
553	P. Chen	The synergistic killing of AML cells co-cultured with HS-5 bone marrow stromal cells by As2O3 and the PI3K/Akt signaling pathway inhibitor LY294002	2015
554	L. Zhang	Retrospective analysis of 65 Chinese children with acute promyelocytic leukemia: a single center experience	2008
555	W. R. Cullen	Methylated and thiolated arsenic species for environmental and health research - A review on synthesis and characterization	2016
556	P. A. Maccarini	PHARMACOLOGY IN PREGNANCY. I. FACTORS CONDITIONING THE ACTION OF DRUGS IN PREGNANCY	1964

557	M. Kippler	Elevated childhood exposure to arsenic despite reduced drinking water concentrations--A longitudinal cohort study in rural Bangladesh	2016
558	J. X. Yu	Levels of P27Kip1 expression and apoptosis in HL-60 cells after treatment with TGF-ß1 and/or arsenic trioxide	2010
559	V. Jadhav	Biocompatible arsenic trioxide nanoparticles induce cell cycle arrest by p21(WAF1/CIP1) expression via epigenetic remodeling in LNCaP and PC3 cell lines	2016
560	L. Wang	Role of P27(Kip1) and TGF-beta1 in APL cell apoptosis induced by As(2)O(3)	2009
561	Y. Jing	Arsenic trioxide selectively induces acute promyelocytic leukemia cell apoptosis via a hydrogen peroxide-dependent pathway	1999
562	P. Kannan-Thulasiraman	Activation of the mitogen- and stress-activated kinase 1 by arsenic trioxide	2006
563	V. Devesa	Arsenicals in maternal and fetal mouse tissues after gestational exposure to arsenite	2006
564	Y. Zheng	Early pregnancy essential and non-essential metal mixtures and gestational glucose concentrations in the 2nd trimester: Results from project viva	2021
565	F. Chen	Regulation of telomerase activity in HL-60 and NB4 cells by arsenic trioxide	2000
566	J. Mathieu	Arsenic trioxide represses NF-kappaB activation and increases apoptosis in ATRA-treated APL cells	2006
567	J. Zhou	Preparation of arsenic trioxide albumin microspheres and its release characteristics in vitro	2005
568	Y. Guo	The Role of Oxidative Stress in Gastrointestinal Tract Tissues Induced by Arsenic Toxicity in Cocks	2015
569	H. Wang	Arsenic trioxide inhibits cell proliferation and human papillomavirus oncogene expression in cervical cancer cells	2014
570	G. Chen	Synergistic antitumor activity of oridonin and arsenic trioxide on hepatocellular carcinoma cells	2012
571	Y. Molin	Arsenic trioxide influences viral replication in target organs of coxsackievirus B3-infected mice	2010
572	X. J. Wang	Gene expression alteration during redox-dependent enhancement of arsenic cytotoxicity by emodin in HeLa cells	2005
573	T. D. Ninh	Water-soluble and lipid-soluble arsenic compounds in japanese flying squid Todarodes pacificus	2007
574	G. D. Shapiro	Exposure to phthalates, bisphenol A and metals in pregnancy and the association with impaired glucose tolerance and gestational diabetes mellitus: The MIREC study	2015

575	B. Zhao	Immunosuppressive effect of arsenic trioxide on islet xenotransplantation prolongs xenograft survival in mice	2018
576	F. Nolte	Depolarisation of the plasma membrane in the arsenic trioxide (As2O3)-and anti-CD95-induced apoptosis in myeloid cells	2004
577	K. Kanaki	HPLC-ICP-MS and HPLC-ES-MS/MS characterization of synthetic seleno-arsenic compounds	2007
578	M. Danan	Arsenous anhydride poisoning. Peripheral neuropathy and changes in cognitive functions	1985
579	B. Cheng	Arsenic trioxide-induced apoptosis of Hep-2 cell line through modulating intracellular glutathione (GSH) level	2010
580	M. J. Shim	Arsenic trioxide induces apoptosis in chronic myelogenous leukemia K562 cells: possible involvement of p38 MAP kinase	2002
581	F. P. Wang	Effects of Arsenic Trioxide on K562 Cell Proliferation and Its Mechanisms	2016
582	Z. Y. Shen	Nitric oxide and calcium ions in apoptotic esophageal carcinoma cells induced by arsenite	2002
583	Z. Slejkovec	Determination of arsenic compounds in reference materials by HPLC-(UV)-HG-AFS	1999
584	E. O. Uthus	Evidence for arsenic essentiality	1992
585	M. A. Davis	Infant toenails as a biomarker of in utero arsenic exposure	2014
586	D. L. Palazzolo	Arsenic trioxide and reduced glutathione act synergistically to augment inhibition of thyroid peroxidase activity in vitro	2015
587	X. N. Lu	The association of HeLa cell apoptosis induced by arsenic trioxide with the down-regulation of HPV18 E6 oncogene and inhibition of telomerase activity	2005
588	B. Drolet	Unusual effects of a QT-prolonging drug, arsenic trioxide, on cardiac potassium currents	2004
589	Y. Jiao	Telomere attrition and chromosome instability via downregulation of TRF2 contributes to arsenic trioxide-induced apoptosis of human T-Cell leukemia cell line molt-4 cells	2007
590	L. Michel	Arsenic trioxide induces apoptosis of cutaneous T cell lymphoma cells: evidence for a partially caspase-independent pathway and potentiation by ascorbic acid (vitamin C)	2003
591	D. Yu	Nuclear matrix associated protein PML: an arsenic trioxide apoptosis therapeutic target protein in HepG2 cells	2003
592	J. Liu	Liver is a target of arsenic carcinogenesis	2008

593	H. Y. Wu	Effects of arsenic trioxide combined with bortezomib on proliferation and apoptosis of K562 cells and their mechanism	2012
594	X. Qu	Arsenic trioxide exerts antimyeloma effects by inhibiting activity in the cytoplasmic substrates of histone deacetylase 6	2012
595	T. Fukuyama	Sensitizing potential of chromated copper arsenate in local lymph node assays differs with the solvent used	2008
596	T. H. Lin	Berberine enhances inhibition of glioma tumor cell migration and invasiveness mediated by arsenic trioxide	2008
597	J. Y. Chan	Effect of arsenic trioxide on multidrug resistant hepatocellular carcinoma cells	2006
598	J. Ye	Inhibition of mitogen-activated protein kinase kinase enhances apoptosis induced by arsenic trioxide in human breast cancer MCF-7 cells	2005
599	G. Jiang	Role of Myc in differentiation and apoptosis in HL60 cells after exposure to arsenic trioxide or all-trans retinoic acid	2008
600	M. Omura	Testicular toxicity evaluation of arsenic-containing binary compound semiconductors, gallium arsenide and indium arsenide, in hamsters	1996
601	C. Y. Yang	Arsenic in drinking water and adverse pregnancy outcome in an arseniasis-endemic area in northeastern Taiwan	2003
602	Y. H. Kang	The role of p38 MAPK and JNK in Arsenic trioxide-induced mitochondrial cell death in human cervical cancer cells	2008
603	K. Q. He	Synergistic effects of Fe-Mn binary oxide for gaseous arsenic removal in flue gas	2021
604	Y. Deng	Arsenic trioxide induced human esophageal cancer Ec109 cell apoptosis with downregulation of c-myc gene expression	2000
605	X. W. Chen	NB4 cell apoptosis induced by bortezomib combined with As(2)O(3) and its mechanism	2011
606	T. Ogata	Subjective symptoms and miscarriage after drinking well water exposed to diphenylarsinic acid	2014
607	Y. Sun	Arsenic trioxide induces apoptosis and the formation of reactive oxygen species in rat glioma cells	2018
608	C. C. Kuo	Combination of arsenic trioxide and BCNU synergistically triggers redox-mediated autophagic cell death in human solid tumors	2011
609	A. Aschengrau	Quality of community drinking water and the occurrence of spontaneous abortion	1989
610	W. Yang	Arsenic trioxide eluting stents to prevent restenosis of injured iliac arteries in rabbits	2006

611	E. Ammatuna	Successful pregnancy after arsenic trioxide therapy for relapsed acute promyelocytic leukaemia	2009
612	D. N. Gandhi	Developmental and neurobehavioural toxicity study of arsenic on rats following gestational exposure	2012
613	Y. Liang	P27(Kip1), cyclin E and endogenous TGF-beta1 changes in apoptosis of NB4 cells induced by As(2)O(3) and/or TGF-beta1 and their significance	2009
614	K. Zhang	Arsenic Trioxide Exposure Induces Heat Shock Protein Responses in Cock Livers	2016
615	W. Zhang	Mediating effect of ROS on mtDNA damage and low ATP content induced by arsenic trioxide in mouse oocytes	2011
616	Y. M. Li	Arsenic targets tubulins to induce apoptosis in myeloid leukemia cells	1999
617	L. Lin	Analysis of Arsenic Compounds in Blood and Urine by HPLC-ICP-MS	2018
618	J. Zhao	The association of arsenic exposure with hypertension and blood pressure: A systematic review and dose-response meta-analysis	2021
619	P. Bobé	New therapeutic perspectives for arsenic: from acute promyelocytic leukemia to autoimmune diseases	2008
620	K. Mehta	Curcumin supplementation shows modulatory influence on functional and morphological features of hippocampus in mice subjected to arsenic trioxide exposure	2020
621	J. H. Lei	Effects of HBV X gene and arsenic trioxide on the expression of p53 in cultured HepG2 cells	2007
622	S. H. Kang	Arsenic trioxide-induced apoptosis is independent of stress-responsive signaling pathways but sensitive to inhibition of inducible nitric oxide synthase in HepG2 cells	2003
623	Y. T. Ren	Prevalence of thyroid function in pregnant and lactating women in areas with different iodine levels of Shanxi province	2018
624	Q. H. Yu	Mechanisms underlying the effect of arsenic trioxide on proliferation inhibition and apoptosis induction in myeloma cell line u266	2007
625	Q. Zhu	The relationship between sensitivity to arsenic trioxide and antioxidative capacity of malignant hematopoietic cells	2000
626	T. J. Smith	Airborne arsenic exposure and excretion of methylated arsenic compounds	1977
627	S. Zhang	Arsenic trioxide inhibits Ewing's sarcoma cell invasiveness by targeting p38(MAPK) and c-Jun N-terminal kinase	2012
628	S. Zhao	Arsenic trioxide induces different gene expression profiles of genes related to growth and apoptosis in glioma cells dependent on the p53 status	2008

629	J. Xing	Adsorption mechanism and competitive adsorption of As(2)O(3) and NH(3) molecules on CuO (111) surface: a DFT study	2021
630	W. Xu	Arsenic trioxide and bortezomib interact synergistically to induce apoptosis in chronic myelogenous leukemia cells resistant to imatinib mesylate through Bcr/Abl-dependent mechanisms	2014
631	Y. Xiangbao	Humanized anti-VEGFR-2 ScFv-As2O3-stealth nanoparticles, an antibody conjugate with potent and selective anti-hepatocellular carcinoma activity	2014
632	T. Seo	Establishment of an arsenic trioxide-resistant human leukemia cell line that shows multidrug resistance	2007
633	J. Dong	Cell cycle arrest and apoptosis induced by arsenic trioxide in human lung cancer cell line	2000
634	Y. F. Li	Arsenic trioxide induced cell apoptosis by mitochondria dependent pathway in KB and KBv200 cells	2004
635	Z. Y. Shen	Intratumoral injection of arsenic to enhance antitumor efficacy in human esophageal carcinoma cell xenografts	2004
636	S. Kumar	Trisenox Disrupts MDM2-DAXX-HAUSP Complex and Induces Apoptosis in a Mouse Model of Acute Leukemia	2020
637	P. Kallinteri	Arsenic trioxide liposomes: encapsulation efficiency and in vitro stability	2004
638	K. F. W. Foley	Early Low-Level Arsenic Exposure Impacts Post-Synaptic Hippocampal Function in Juvenile Mice	2021
639	P. Binu	Acute promyelocytic leukemia drug - arsenic trioxide in the presence of eugenol shows differential action on leukemia cells (HL-60) and cardiomyocytes (H9c2) - inference from NMR study	2021
640	Y. M. Li	Study on the sensitivity of leukemic cells to arsenic trioxide enhanced by targeted suppression of mIRNA-21	2010
641	Y. F. Xiao	Effect of As(2)O(3) on expressions of COX-2 and matrix metalloproteinases in SGC7901 and K562 cells	2009
642	A. Lemarie	Human macrophages constitute targets for immunotoxic inorganic arsenic	2006
643	S. H. Woo	Arsenic trioxide sensitizes CD95/Fas-induced apoptosis through ROS-mediated upregulation of CD95/Fas by NF-kappaB activation	2004
644	L. F. Zhou	Effect of arsenic trioxide on apoptosis of pulmonary eosinophile in asthmatic guinea-pigs	2002
645	T. J. Chiou	Arsenic trioxide impairs spermatogenesis via reducing gene expression levels in testosterone synthesis pathway	2008
646	S. Matsuto	Arsenobetaine in the red crab, Chionoecetes opilio	1986

647	E. Hoffman	Arsenic trioxide: impact on the growth and differentiation of cancer cells and possible use in cancer therapy	2013
648	X. S. Ye	Methylation of P15INK4B gene in patients with myelodysplastic syndromes and demethylating effects of drugs	2007
649	J. Pinchoff	Is living in a region with high groundwater arsenic contamination associated with adverse reproductive health outcomes? An analysis using nationally representative data from India	2022
650	J. Száková	Response of pepper plants (Capsicum annum L.) on soil amendment by inorganic and organic compounds of arsenic	2007
651	W. H. Park	Arsenic trioxide-mediated growth inhibition in MC/CAR myeloma cells via cell cycle arrest in association with induction of cyclin-dependent kinase inhibitor, p21, and apoptosis	2000
652	M. P. Waalkes	Urogenital carcinogenesis in female CD1 mice induced by in utero arsenic exposure is exacerbated by postnatal diethylstilbestrol treatment	2006
653	C. A. Lammon	Effects of protein deficient diets on the developmental toxicity of inorganic arsenic in mice	2004
654	A. Gossai	Association between maternal urinary arsenic species and infant cord blood leptin levels in a New Hampshire Pregnancy Cohort	2015
655	B. T. Huang	A prospective, observational study of added medium-dose cytosine arabinoside versus As2O3 for elderly patients with acute promyelocytic leukemia	2014
656	J. C. Zheng	Arsenic Trioxide Inhibits the Metastasis of Small Cell Lung Cancer by Blocking Calcineurin-Nuclear Factor of Activated T Cells (NFAT) Signaling	2019
657	Z. B. Ma	Arsenic trioxide induces apoptosis of human gastrointestinal cancer cells	2014
658	A. Tun-Kyi	Arsenic trioxide down-regulates antiapoptotic genes and induces cell death in mycosis fungoides tumors in a mouse model	2008
659	S. M. Wang	Effects of hyperbaric oxygenation combined with As(2)O(3) on proliferation of K562 cells and associated mechanism	2012
660	X. E. He	Study of effects of HBV X gene and As2O3 on expression and activity of p53 in HepG2 cells with shRNA	2006
661	M. Nassar	The Hormetic Effect of Arsenic Trioxide on Rat Pulpal Cells: An In Vitro Preliminary Study	2021
662	Y. Wang	Arsenic trioxide inhibits breast cancer cell growth via microRNA-328/hERG pathway in MCF-7 cells	2015
663	S. Sebastian	Arsenic counteracts human immunodeficiency virus type 1 restriction by various TRIM5 orthologues in a cell type-dependent manner	2006
664	X. B. Yin	Inhibitory effect of humanized anti-VEGFR-2 ScFv-As2O3-stealth nanoparticles conjugate on growth of human hepatocellular carcinoma: in vitro and in vivo studies	2014

665	Y. Li	Inhibition of the cancer stem cells-like properties by arsenic trioxide, involved in the attenuation of endogenous transforming growth factor beta signal	2015
666	H. Zhang	Arsenic trioxide initiates ER stress responses, perturbs calcium signalling and promotes apoptosis in human lens epithelial cells	2007
667	M. K. Chelbi-alix	Arsenic enhances the activation of Stat1 by interferon gamma leading to synergistic expression of IRF-1	2003
668	H. Zhou	Inhibitory effect of arsenic trioxide on neuronal migration in vitro and its potential molecular mechanism	2015
669	C. Du	Differentiation of human nasopharyngeal carcinoma xenografts and repression of telomerase activity induced by arsenic trioxide	2004
670	J. Yu	Arsenic trioxide (As2O3) reduces the invasive and metastatic properties of cervical cancer cells in vitro and in vivo	2007
671	L. M. Lin	Synergistic effect of all-trans-retinoic acid and arsenic trioxide on growth inhibition and apoptosis in human hepatoma, breast cancer, and lung cancer cells in vitro	2005
672	J. Zhou	Effects of protein tyrosine kinase, protein tyrosine phosphatase and protein kinase C on the apoptosis of arsenic trioxide treated NB4 cells and human cortex neurons	2004
673	J. B. Lin	Precipitation of organic arsenic compounds and their degradation products during struvite formation	2016
674	Y. Cheng	Arsenic trioxide inhibits glioma cell growth through induction of telomerase displacement and telomere dysfunction	2016
675	J. A. Park	Acute intestinal pseudo-obstruction after induction treatment of relapsed acute promyelocytic leukemia with arsenic trioxide	2008
676	N. N. Ngalame	Delayed temporal increase of hepatic Hsp70 in ApoE knockout mice after prenatal arsenic exposure	2013
677	D. P. Hanlon	Concentration and chemical status of arsenic in the blood of pregnant hamsters during critical embryogenesis. 1. Subchronic exposure to arsenate utilizing constant rate administration	1986
678	T. Feng	Stathmin is key in reversion of doxorubicin resistance by arsenic trioxide in osteosarcoma cells	2014
679	X. Wang	Arsenic trioxide attenuates the invasion potential of human liver cancer cells through the demethylation-activated microRNA-491	2014
680	J. Mathieu	Clinically tolerable concentrations of arsenic trioxide induce p53-independent cell death and repress NF-kappa B activation in Ewing sarcoma cells	2006
681	T. Okui	Inhibition of human excision DNA repair by inorganic arsenic and the co-mutagenic effect in V79 Chinese hamster cells	1986
682	Y. Yu	Anti-apoptotic and apoptotic pathway analysis of arsenic trioxide-induced apoptosis in human gastric cancer SGC-7901 cells	2014

683	L. Si	Induction of the mesenchymal to epithelial transition by demethylation- activated microRNA-200c is involved in the anti-migration/invasion effects of arsenic trioxide on human breast cancer cells	2015
684	T. Samikkannu	Reactive oxygen species are involved in arsenic trioxide inhibition of pyruvate dehydrogenase activity	2003
685	M. Carré	Involvement of microtubules and mitochondria in the antagonism of arsenic trioxide on paclitaxel-induced apoptosis	2002
686	Y. Y. Zhao	Downregulation of P-gp, Ras and p-ERK1/2 contributes to the arsenic trioxide-induced reduction in drug resistance towards doxorubicin in gastric cancer cell lines	2015
687	B. T. Huang	The early addition of arsenic trioxide versus high-dose arabinoside is more effective and safe as consolidation chemotherapy for risk-tailored patients with acute promyelocytic leukemia: multicenter experience	2012
688	H. R. Zhou	Arsenic trioxide reverses hypermethylation of p16 and activates its transcription in malignant lymphoma cell line CA46	2010
689	G. Cunha De Santis	Adhesion molecules and Differentiation Syndrome: phenotypic and functional analysis of the effect of ATRA, As2O3, phenylbutyrate, and G-CSF in acute promyelocytic leukemia	2007
690	X. Cai	The mechanisms of arsenic trioxide-induced apoptosis in hematopoietic malignant cells	1999
691	J. G. Seol	Effect of arsenic trioxide on cell cycle arrest in head and neck cancer cell line PCI-1	1999
692	Y. J. Chen	Blocking effect of arsenic trioxide on the proliferation and cell cycle of human Burkitt lymphoma cells and its related mechanism	2013
693	N. Askar	Arsenic trioxide exposure to ovarian carcinoma cells leads to decreased level of topoisomerase II and cytotoxicity	2006
694	G. Q. Wei	Cytotoxic effects of arsenic trioxide in combination with chemotherapeutic drugs on acute non-promyelocytic leukemia cells	2004
695	J. Li	Arsenic trioxide promotes histone H3 phosphoacetylation at the chromatin of CASPASE-10 in acute promyelocytic leukemia cells	2002
696	A. Muto	A novel differentiation-inducing therapy for acute promyelocytic leukemia with a combination of arsenic trioxide and GM-CSF	2001
697	A. J. Mürer	Effect of seafood consumption on the urinary level of total hydride-generating arsenic compounds. Instability of arsenobetaine and arsenocholine	1992
698	C. G. Howe	Prenatal metal mixtures and fetal size in mid-pregnancy in the MADRES study	2021
699	X. Jin	Maternal exposure to arsenic and cadmium and the risk of congenital heart defects in offspring	2016
700	T. Kiguchi	Speciation of arsenic trioxide penetrates into cerebrospinal fluid in patients with acute promyelocytic leukemia	2010

701	Y. Molin	Arsenic trioxide affects the trace element balance in tissues in infected and healthy mice differently	2009
702	H. Tong	Arsenic trioxide induced p15INK4B gene expression in myelodysplastic syndrome cell line MUTZ-1	2002
703	D. J. Goussetis	Autophagy is a critical mechanism for the induction of the antileukemic effects of arsenic trioxide	2010
704	X. Zhao	Arsenic trioxide-induced apoptosis in H9c2 cardiomyocytes: implications in cardiotoxicity	2008
705	D. Lu	Hydrogen peroxide inhibits arsenic trioxide-induced apoptosis of Burkitt lymphoma cells	2004
706	W. Guo	Effects of all-trans retinoic acid, arsenic trioxide and daunorubicin on tissue factor expression in NB4 cells	1999
707	J. Dittmann	Characterization of differing effects caused by homeopathically prepared and conventional dilutions using cytochrome P450 2E1 and other enzymes as detection systems	1996
708	J. S. Yoon	Anti-tumoral effect of arsenic compound, sodium metaarsenite (KML001), in non-Hodgkin's lymphoma: an in vitro and in vivo study	2016
709	C. Hopenhayn	Arsenic exposure from drinking water and birth weight	2003
710	S. Gu	Study on the resveratrol and arsenic trioxide combination induced apoptosis and its mechanism on lung adenocarcinoma cells	2016
711	L. Zheng	Arsenic trioxide inhibits viability and induces apoptosis through reactivating the Wnt inhibitor secreted frizzled related protein-1 in prostate cancer cells	2016
712	L. Wang	Preparation of a new nanosized As2O3/Mn0.5Zn0.5Fe2O4 thermosensitive magnetoliposome and its antitumor effect on MDA_MB_231 cells	2011
713	H. Li	Urinary metabolomics revealed arsenic exposure related to metabolic alterations in general Chinese pregnant women	2017
714	X. Cai	In vitro study on arsenic trioxide-induced apoptosis of retinoic acid resistant acute promyelocytic leukemia cell line(MR-2)	1998
715	Y. F. Xiao	Effect of arsenic trioxide on vascular endothelial cell proliferation and expression of vascular endothelial growth factor receptors Flt-1 and KDR in gastric cancer in nude mice	2007
716	X. P. Cui	Inhibitory effect of arsenic trioxide on proliferation of human hepatocellular carcinoma cell line SMMC-7721 and the mechanism	2007
717	J. Zhang	Arsenic trioxide (As(2)O(3)) inhibits peritoneal invasion of ovarian carcinoma cells in vitro and in vivo	2006
718	M. H. Yang	Arsenic Trioxide Suppresses Tumor Growth through Antiangiogenesis via Notch Signaling Blockade in Small-Cell Lung Cancer	2019

719	M. A. Maroui	Requirement of PML SUMO interacting motif for RNF4- or arsenic trioxide-induced degradation of nuclear PML isoforms	2012
720	P. M. Jia	Experimental study of low dose arsenic trioxide in treatment of patients with acute promyelocytic leukemia	2002
721	T. D. Zhang	Arsenic trioxide, a therapeutic agent for APL	2001
722	C. Diepart	Arsenic trioxide treatment decreases the oxygen consumption rate of tumor cells and radiosensitizes solid tumors	2012
723	N. Wu	Adverse effects of intravenous arsenic trioxide and their prevention	2006
724	Z. Y. Shen	Reactive oxygen species and antioxidants in apoptosis of esophageal cancer cells induced by As2O3	2003
725	Y. P. Wang	lnhibition of arsenic trioxide on human tongue cancer cell line Tca8113 cells in Scid mice in vivo	1999
726	J. R. Berenson	Arsenic compounds in the treatment of multiple myeloma: a new role for a historical remedy	2006
727	M. H. Yang	Arsenic trioxide exerts anti-lung cancer activity by inhibiting angiogenesis	2014
728	S. Potin	Involvement of a Rho-ROCK-JNK pathway in arsenic trioxide-induced apoptosis in chronic myelogenous leukemia cells	2007
729	W. Lu	Effects of arsenic trioxide on the expression of ezrin in hepatocellular carcinoma	2017
730	M. Oketani	Inhibition by arsenic trioxide of human hepatoma cell growth	2002
731	L. Chen	Affect of As(2)O(3) on the sister chromatid exchanges in human blood lymphocytes	2001
732	E. J. Tokar	Tumors and proliferative lesions in adult offspring after maternal exposure to methylarsonous acid during gestation in CD1 mice	2012
733	J. El Bougrini	Arsenic enhances the apoptosis induced by interferon gamma: key role of IRF-1	2006
734	J. M. Kuiper	A method for site-specific labeling of multiple protein thiols	2009
735	P. D. Lin	Diet and erythrocyte metal concentrations in early pregnancy-cross-sectional analysis in Project Viva	2021
736	C. W. Du	Latent membrane protein-1 of Epstein - Barr virus increases sensitivity to arsenic trioxide-induced apoptosis in nasopharyngeal carcinoma cell	2005

737	Z. Diaz	Trolox selectively enhances arsenic-mediated oxidative stress and apoptosis in APL and other malignant cell lines	2005
738	Y. L. Kwong	Arsenic trioxide- and idarubicin-induced remissions in relapsed acute promyelocytic leukaemia: clinicopathological and molecular features of a pilot study	2001
739	R. Hao	Involvement of PML proteins in treatment of acute promyelocytic leukemia with arsenic trioxide	2018
740	H. Nabeshi	Arsenic trioxide inhibits human t cell-lymphotropic virus-1-induced syncytiums by down-regulating gp46	2009
741	C. Y. Li	STI571 enhances the effect of arsenic trioxide and Velcade on bcr/abl+-CD34+ cell proliferation and apoptosis	2007
742	N. C. Twaddle	Metabolism and disposition of arsenic species after repeated oral dosing with sodium arsenite in drinking water. II. Measurements in pregnant and fetal CD-1 mice	2018
743	Not found	Arsenic compounds, inorganic	2004
744	J. M. Wood	Letter: The methylation of arsenic compounds	1975
745	Q. Duan	Arsenic trioxide suppresses paclitaxel-induced mitotic arrest	2009
746	J. H. Byun	Effect of Arsenic Trioxide in TRAIL (Tumor Necrosis Factor-related Apoptosis Inducing Ligand)-Mediated Apoptosis in Multiple Myeloma Cell Lines	2003
747	J. Yi	Apoptosis susceptibility of tumor cells to arsenic trioxide and the inherent cellular level of reactive oxygen species	2002
748	C. H. Tseng	A review on environmental factors regulating arsenic methylation in humans	2009
749	J. Dittmann	Temperature dependent influence of As2O3, HgHPO4 and KCl on lysosomal acid phosphatase isolated from rat liver	1994
750	Not found	Arsenic and inorganic arsenic compounds	2011
751	V. P. Markowski	Effects of prenatal exposure to sodium arsenite on motor and food-motivated behaviors from birth to adulthood in C57BL6/J mice	2012
752	H. M. Jing	Effect of arsenic trioxide on different cell lines derived from chronic myeloid leukemia	2002
753	A. Abudoureyimu	Arsenic trioxide regulates gastric cancer cell apoptosis by mediating cAMP	2017
754	C. Li	Arsenic trioxide inhibits accelerated allograft rejection mediated by alloreactive CD8(+) memory T cells and prolongs allograft survival time	2015

755	H. Yao	Effects of arsenic trioxideon the cell apoptosis and hTERT mRNA of human tongue cancer cells	2005
756	M. Kawaguchi	Relationship between arsenic-induced hypothermia and brain monoamines. (2). The roles of noradrenaline and dopamine	1982
757	X. Wang	Nephroprotective effect of astaxanthin against trivalent inorganic arsenic-induced renal injury in wistar rats	2014
758	R. Stalder	Arsenic modulates APOBEC3G-mediated restriction to HIV-1 infection in myeloid dendritic cells	2010
759	M. Brizzi	Statistical analysis of the effect of high dilutions of arsenic in a large dataset from a wheat germination model	2000
760	G. Z. Yang	Study on the mechanism of arsenic trioxide inhibiting NB4 cells proliferation	2009
761	B. G. Ye	Synergistic effects of VPA and As2O3 on Molt-4 cells in vitro and its possible mechanisms	2008
762	D. Lu	Arsenic trioxide-induced apoptosis of human malignant lymphoma cell lines and its mechanisms	2003
763	T. Kajiguchi	Sustained activation of c-jun-terminal kinase (JNK) is closely related to arsenic trioxide-induced apoptosis in an acute myeloid leukemia (M2)-derived cell line, NKM-1	2003
764	S. Huang	Apoptosis of drug-resistant human ovarian carcinoma cell line 3AO/cDDP induced by arsenic trioxide and its mechanism	2002
765	H. Y. Eom	Determination of residual arsenic compounds in chicken muscle by ultra-performance liquid chromatography coupled with ultraviolet detection after pre-column derivatization with toluene-3,4-dithiol	2015
766	L. B. de Abreu	Desorption electrospray ionization mass spectrometry (DESI-MS) applied to the speciation of arsenic compounds from fern leaves	2013
767	R. W. Ahn	A novel nanoparticulate formulation of arsenic trioxide with enhanced therapeutic efficacy in a murine model of breast cancer	2010
768	L. Zhou	Effects of arsenic trioxide on K562 cells stably expressing two promyelocytic leukemia-specific fusion proteins	2000
769	P. Zhao	Heat Shock Protein Alteration in the Gastrointestinal Tract Tissues of Chickens Exposed to Arsenic Trioxide	2016
770	S. Sturlan	Docosahexaenoic acid enhances arsenic trioxide-mediated apoptosis in arsenic trioxide-resistant HL-60 cells	2003
771	T. Maitani	Chemical form-dependent induction of hepatic zinc-thionein by arsenic administration and effect of co-administered selenium in mice	1987
772	Y. Y. Wei	Antagonistic effect of early stage zinc on arsenic toxicity induced preterm birth during pregnancy: evidence from a rural Bangladesh birth cohort	2021

773	H. Zhang	Knockdown of SOX9 enhances apoptosis of laryngeal squamous cell carcinoma cells induced by As(2)O(3)	2018
774	C. Yedjou	Ascorbic Acid Potentiation of Arsenic Trioxide Anticancer Activity Against Acute Promyelocytic Leukemia	2009
775	V. Ettler	Oral bioaccessibility of metal(loid)s in dust materials from mining areas of northern Namibia	2019
776	J. Li	Toxic effects of arsenic trioxide on Echinococcus granulosus protoscoleces through ROS production, and Ca2+-ER stress-dependent apoptosis	2018
777	Y. Percheranc ier	Role of SUMO in RNF4-mediated promyelocytic leukemia protein (PML) degradation: sumoylation of PML and phospho-switch control of its SUMO binding domain dissected in living cells	2009
778	D. Chen	Buthionine sulfoximine enhancement of arsenic trioxide-induced apoptosis in leukemia and lymphoma cells is mediated via activation of c-Jun NH2-terminal kinase and up-regulation of death receptors	2006
779	M. Mazumdar	Does arsenic increase the risk of neural tube defects among a highly exposed population? A new case-control study in Bangladesh	2017
780	S. Talbot	Arsenic trioxide and auranofin inhibit selenoprotein synthesis: implications for chemotherapy for acute promyelocytic leukaemia	2008
781	H. Y. Fu	Hypermethylation of CpG island of p16 gene and arsenic trioxide induced p16 gene demethylation in multiple myeloma	2005
782	F. L. Huang	Comparison of antitumor efficacy between arsacetyl and arsenic trioxide in vitro	2002
783	L. Szinicz	Effect of As2O3 on gluconeogenesis	1988
784	T. L. Tsai	Association Between Prenatal Exposure to Metals and Atopic Dermatitis Among Children Aged 4 Years in Taiwan	2021
785	J. Suhl	Maternal arsenic exposure and nonsyndromic orofacial clefts	2018
786	I. Karakis	Maternal metal concentration during gestation and pediatric morbidity in children: an exploratory analysis	2021
787	C. Zhao	Effects of the combination of As(2)O(3) and AZT on proliferation inhibition and apoptosis induction of hepatoma HepG2 cells following silencing of Egr-1	2018
788	H. Li	Arsenic trioxide inhibits DNA methyltransferase and restores TMS1 gene expression in K562 cells	2015
789	M. Aggarwal	Effects of low-level arsenic exposure on the developmental toxicity of anilofos in rats	2007
790	V. M. Rodríguez	Effects of sodium arsenite exposure on development and behavior in the rat	2002

791	Y. Chou	Endothelial gap junctions are down-regulated by arsenic trioxide	2007
792	B. Elkin	Intensive arsenical treatment in women and especially pregnant women	1946
793	J. F. Holson	Evaluation of the prenatal developmental toxicity of orally administered arsenic trioxide in rats	2000
794	Y. J. Li	Effects of arsenic trioxide on the proliferation of human breast cancer SKBR-3 cell and the expression of Notch1	2012
795	K. H. Ryu	Morphological and biochemical changes induced by arsenic trioxide in neuroblastoma cell lines	2005
796	Y. M. Wei	Down-regulation of four arsenic antagonists on apoptosis and telomerase activity induced by arsenic trioxide in three myelocytic leukemia cell lines	2001
797	Y. Xie	Aberrant DNA methylation and gene expression in livers of newborn mice transplacentally exposed to a hepatocarcinogenic dose of inorganic arsenic	2007
798	B. Gollackner	An exploratory investigation of the effect of arsenic trioxide on anti-Gal antibody production in baboons	2003
799	S. Y. Huang	Acute and chronic arsenic poisoning associated with treatment of acute promyelocytic leukaemia	1998
800	G. Schwalfenberg	Heavy metal contamination of prenatal vitamins	2018
801	X. Xiong	Arsenic trioxide induces cell cycle arrest and affects Trk receptor expression in human neuroblastoma SK-N-SH cells	2018
802	X. Hao	Intravenous As(2)O(3) as a promising treatment for psoriasis - an experimental study in psoriasis-like mouse model	2022
803	M. H. Yang	Notch pathway inhibition mediated by arsenic trioxide depletes tumor initiating cells in small cell lung cancer	2022
804	S. Li	Arsenic-induced cardiotoxicity correlates with mitochondrial damage and trace elements imbalance in broiler chickens	2019
805	W. Zhang	Arsenic trioxide re-sensitizes ERα-negative breast cancer cells to endocrine therapy by restoring ERα expression in vitro and in vivo	2011
806	P. Kumar	Arsenic trioxide enhances the therapeutic efficacy of radiation treatment of oral squamous carcinoma while protecting bone	2008
807	C. Du	Downregulation of Epstein-Barr virus-encoded latent membrane protein-1 by arsenic trioxide in nasopharyngeal carcinoma cells	2006
808	Z. G. Wang	Arsenic trioxide and melarsoprol induce programmed cell death in myeloid leukemia cell lines and function in a PML and PML-RARalpha independent manner	1998

809	V. A. Ado	Experimentally increased sensitivity to arsenic compounds	1973
810	S. Anastasov	Poisoning with arsenic compounds in fruits	1957
811	Not found	Arsenic and arsenic compounds	1980
812	P. C. Minh	Carcinogenic effects of arsenic compounds	1966
813	M. Alrashed	Assessment of DNA damage in relation to heavy metal induced oxidative stress in females with recurrent pregnancy loss (RPL)	2021
814	R. E. Morrissey	Arsine: absence of developmental toxicity in rats and mice	1990
815	Y. Lin	Study on congenital cardiac anomalies induced by arsenic exposure before and during maternal pregnancy in fetal rats	2016
816	G. B. Wang	Mechanism of As2O3 induces apoptosis of glioma U87 cells	2017
817	J. X. Xu	Effect of TAK1 gene silencing on the apoptosis of Kasumi-1 cells induced by arsenic trioxide	2013
818	D. B. Qin	Mechanism of apoptosis of NB4 cells induced by arsenic trioxide and cyclooxygenase-2 expression	2011
819	Y. Liu	Effect of Arsenic Trioxide on Myelomonocytic Progenitor Cells in Patients with Myelodysplastic Syndrome in Vitro	2000
820	Q. Q. Wang	Methylated arsenic metabolites bind to PML protein but do not induce cellular differentiation and PML-RARα protein degradation	2015
821	W. K. Raja	Dose Response of MTLn3 Cells to Serial Dilutions of Arsenic Trioxide and Ionizing Radiation	2013
822	Y. Shi	Inhibitory effect of arsenic trioxide on the pulmonary metastasis of melanoma B16 cells	2011
823	X. D. Ma	Mechanism of opening of mitochondrial permeability transition pore induced by arsenic trioxide	2006
824	Y. B. Chen	Effects of arsenic trioxide on cell cycle and expression of cyclin dependent kinase inhibitors of multiple myeloma cells	2003
825	J. Tang	The expression of AQP9 in HepG2 cells affects cell biological behaviors and sensitivity to As2O3	2015
826	Y. Deng	Mechanisms of arsenic trioxide induced apoptosis of human cervical cancer HeLa cells and protection by Bcl-2	1999

827	L. F. Zhou	Arsenic trioxide, a potent inhibitor of NF-kappaB, abrogates allergen-induced airway hyperresponsiveness and inflammation	2006
828	H. Xiong	Investigation of the effect of 2-methoxyestradiol and arsenic trioxide on the apoptosis-associated gene expression profile of myeloma cells	2005
829	K. Ishitsuka	Arsenic trioxide inhibits growth of human T-cell leukaemia virus type I infected T-cell lines more effectively than retinoic acids	1998
830	G. Pershagen	Lung retention and toxicity of some inorganic arsenic compounds	1982
831	Not found	Arsenic compounds may cause genetic damage	2001
832	J. Ni	Pharmacokinetics of intravenous arsenic trioxide in the treatment of acute promyelocytic leukemia	1997
833	F. X. Reichl	Effect of glucose in mice after acute experimental poisoning with arsenic trioxide (As2O3)	1990
834	R. Soler-Blasco	Urinary arsenic species and methylation efficiency during pregnancy: Concentrations and associated factors in Spanish pregnant women	2021
835	N. Karasavvas	Vitamin C protects HL60 and U266 cells from arsenic toxicity	2005
836	H. C. Li	Effect and mechanism of arsenic trioxide on chemosensitivity of human lung adenocarcinoma cells	2003
837	Z. Y. Shen	Ezrin, actin and cytoskeleton in apoptosis of esophageal epithelial cells induced by arsenic trioxide	2003
838	W. L. Zhao	Treatment of acute promyelocytic leukemia with arsenic trioxide: clinical and basic studies	2001
839	S. G. Huang	Impact of arsenic trioxide on proliferation and metastasis of drug-resistant human ovarian carcinoma cell line	2002
840	J. M. Grad	Ascorbic acid enhances arsenic trioxide-induced cytotoxicity in multiple myeloma cells	2001
841	I. Ora	Arsenic trioxide inhibits neuroblastoma growth in vivo and promotes apoptotic cell death in vitro	2000
842	M. Brizzi	A biostatistical insight into the As(2)O(3) high dilution effects on the rate and variability of wheat seedling growth	2005
843	E. Ficker	Mechanisms of arsenic-induced prolongation of cardiac repolarization	2004
844	Z. Y. Shen	Morphological and functional changes of mitochondria in apoptotic esophageal carcinoma cells induced by arsenic trioxide	2002

845	Not found	MEDICINAL AND DIETETIC PREPARATIONS: Organic Arsenic Compounds	1909
846	S. A. Ahmad	Arsenic contamination in groundwater in Bangladesh: implications and challenges for healthcare policy	2018
847	Y. R. Wang	Apoptosis of the adriamycin-resistant leukemia cell line induced by the recombinant mutant human TNF-related apoptosis-inducing ligand combined with arsenic trioxide	2008
848	G. M. Buck Louis	Low-level environmental metals and metalloids and incident pregnancy loss	2017
849	S. V. Flanagan	Leveraging Health Care Communication Channels for Environmental Health Outreach in New Jersey	2020
850	L. Li	Apoptosis of MR2 cells induced by Tanshinone II A combined with arsenic trioxide	2009
851	X. H. Zhang	Arsenic trioxide downregulates the expression of annexin II in bone marrow cells from patients with acute myelogenous leukemia	2009
852	Y. Y. Li	Association of arsenic with unexplained recurrent spontaneous abortion: a case-control study	2019
853	W. Zhang	Protective effect of resveratrol on arsenic trioxide-induced nephrotoxicity in rats	2014
854	L. Hao	Hepatotoxicity from arsenic trioxide for pediatric acute promyelocytic leukemia	2013
855	Y. Zhen	Arsenic trioxide-mediated Notch pathway inhibition depletes the cancer stem-like cell population in gliomas	2010
856	O. Axelson	Arsenic compounds and cancer	1980
857	E. W. Da Costa	Suppression of the inhibitory effects of arsenic compounds by phosphate	1971
858	S. X. Le	Speciation of arsenic compounds in some marine organisms	1994
859	P. Bobé	Arsenic trioxide: A promising novel therapeutic agent for lymphoproliferative and autoimmune syndromes in MRL/lpr mice	2006
860	K. A. Ramsey	In utero exposure to arsenic alters lung development and genes related to immune and mucociliary function in mice	2013
861	S. Abhilash	Different administration patterns of docosahexaenoic acid in combating cytotoxic manifestations due to arsenic trioxide (acute promyelocytic leukemia drug) induced redox imbalance in hepatocytes	2018
862	C. Niu	Studies on treatment of acute promyelocytic leukemia with arsenic trioxide: remission induction, follow-up, and molecular monitoring in 11 newly diagnosed and 47 relapsed acute promyelocytic leukemia patients	1999

863	X. Zhang	Arsenic trioxide induces G2/M arrest in hepatocellular carcinoma cells by increasing the tumor suppressor PTEN expression	2012
864	H. S. Jung	Arsenic trioxide concentration determines the fate of Ewing's sarcoma family tumors and neuroblastoma cells in vitro	2006
865	P. A. Guńka	The structure and energetics of arsenic(III) oxide intercalated by ionic azides	2014
866	S. Deaglio	Evidence of an immunologic mechanism behind the therapeutical effects of arsenic trioxide (As(2)O(3)) on myeloma cells	2001
867	A. W. Turner	Bacterial oxidation of arsenite	1949
868	S. Zarazúa	Decreased nitric oxide production in the rat brain after chronic arsenic exposure	2006
869	Z. Sun	Arsenic trioxide inhibits angiogenesis in vitro and in vivo by upregulating FoxO3a	2019
870	Y. Jing	The design of selective and non-selective combination therapy for acute promyelocytic leukemia	2007
871	X. W. Xu	Effects of dexamethasone on arsenic trioxide induced apoptosis, NF-kappaB activation and gene expression in lymphoma cell line	2005
872	C. P. Lin	Successful treatment of all-trans retinoic acid resistant and chemotherapy naïve acute promyelocytic patients with arsenic trioxide--two case reports	2000
873	S. Datta	Efficacy of a potentized homoeopathic drug (Arsenicum Album-30) in reducing genotoxic effects produced by arsenic trioxide in mice: comparative studies of pre-, post- and combined pre- and post-oral administration and comparative efficacy of two microdoses	1999
874	S. Gupta	Parenthood in patients with acute promyelocytic leukemia after treatment with arsenic trioxide: a case series	2012
875	J. Liu	Global gene expression associated with hepatocarcinogenesis in adult male mice induced by in utero arsenic exposure	2006
876	H. Moulahoum	Arsenic trioxide ameliorates murine colon inflammation through inflammatory cell enzymatic modulation	2019
877	M. H. Yang	Anti-angiogenic effect of arsenic trioxide in lung cancer via inhibition of endothelial cell migration, proliferation and tube formation	2017
878	D. D. Wu	Extracellular signal-regulated kinase 8-mediated NF-κB activation increases sensitivity of human lung cancer cells to arsenic trioxide	2017
879	M. Primon	Cathepsin L silencing increases As(2)O(3) toxicity in malignantly transformed pilocytic astrocytoma MPA58 cells by activating caspases 3/7	2017
880	Y. Z. Chen	Effect of endogenous TGF-beta1 and TNF-alpha on the As(2)O(3) inducing apoptosis of HL-60 cells	2003

881	A. C. F. Souza	Effects of Arsenic Compounds on Microminerals Content and Antioxidant Enzyme Activities in Rat Liver	2018
882	D. Gradecka	Selected mechanisms of genotoxic effects of inorganic arsenic compounds	2001
883	Z. P. Xu	Effects of arsenic trioxide (As(2)O(3)) on airway remodeling in a murine model of bronchial asthma	2012
884	D. F. Qiao	As2O3-induced permeability transition pore opening in mitochondria depends on Ca2+	2006
885	L. Wei	Primary research on arsenic trioxide inhibiting human breast cancer cells growth and its mechanisms	2005
886	J. Zhou	Study on cardiac toxicity in acute promyelocyte leukemia treatment of arsenic trioxide intravenous infusion in general dose	2003
887	Z. Y. Shen	Mitochondria, calcium and nitric oxide in the apoptotic pathway of esophageal carcinoma cells induced by As2O3	2002
888	H. J. Liu	PDCD4 enhances the inhibitory effect of As(2)O(3) on the growth and NF-κB signaling pathway in neuroblastoma cells	2019
889	S. F. Farzan	Demographic predictors of urinary arsenic in a low-income predominantly Hispanic pregnancy cohort in Los Angeles	2021
890	H. A. Monat	CLINICAL CONSIDERATIONS OF AMEBIASIS	1964
891	Y. Li	Role of programmed cell death in mediating arsenic-induced rat embryo anomalies	1998
892	D. Fano-Sizgorich	Total Urinary Arsenic and Inorganic Arsenic Concentrations and Birth Outcomes in Pregnant Women of Tacna, Peru: A Cross-Sectional Study	2021
893	C. V. Watson	Characterization of trace elements exposure in pregnant women in the United States, NHANES 1999-2016	2020
894	M. Fort	Food sources of arsenic in pregnant Mediterranean women with high urine concentrations of this metalloid	2014
895	R. C. Fry	Activation of inflammation/NF-kappaB signaling in infants born to arsenic-exposed mothers	2007
896	E. Calviño	Increased apoptotic efficacy of lonidamine plus arsenic trioxide combination in human leukemia cells. Reactive oxygen species generation and defensive protein kinase (MEK/ERK, Akt/mTOR) modulation	2011
897	W. M. Cheung	Effects of arsenic trioxide on the cellular proliferation, apoptosis and differentiation of human neuroblastoma cells	2007
898	Q. Tong	Preliminary study of the in vitro growth inhibition of human bladder cancer cell line BIU-87 by arsenic trioxide	2000

899	A. Hines-Peralta	Improved tumor destruction with arsenic trioxide and radiofrequency ablation in three animal models	2006
900	M. Dawood	Multifactorial Modes of Action of Arsenic Trioxide in Cancer Cells as Analyzed by Classical and Network Pharmacology	2018
901	Y. Cha	Arsenic trioxide induces apoptosis in human colorectal adenocarcinoma HT-29 cells through ROS	2006
902	X. M. Hu	Arsenic trioxide induces apoptosis equally in T lymphoblastoid leukemia MOLT-4 cells and P-gp-expressing daunorubicin-resistant MOLT-4 cells	2003
903	C. Yan	Effects of As2O3 and Resveratrol on the Proliferation and Apoptosis of Colon Cancer Cells and the hERG-mediated Potential Mechanisms	2019
904	D. Zhao	Arsenic trioxide reduces drug resistance to adriamycin in leukemic K562/A02 cells via multiple mechanisms	2011
905	L. Luo	Arsenic trioxide synergizes with B7H3-mediated immunotherapy to eradicate hepatocellular carcinomas	2006
906	G. J. Roboz	Arsenic trioxide induces dose- and time-dependent apoptosis of endothelium and may exert an antileukemic effect via inhibition of angiogenesis	2000
907	D. Wang	Impaired lipid and glucose homeostasis in male mice offspring after combined exposure to low-dose bisphenol A and arsenic during the second half of gestation	2018
908	J. W. Sunderman, Jr.	A review of the carcinogenicities of nickel, chromium and arsenic compounds in man and animals	1976
909	N. Ishinishi	Symptoms and diagnosis of poisoning: arsenic and arsenic compounds (metalloid)	1973
910	T. Jiang	Experimental research on the effect of arsenic trioxide on the growth of prostate cancer PC-3 cell lines	2004
911	S. Remy	Expression of the sFLT1 gene in cord blood cells is associated to maternal arsenic exposure and decreased birth weight	2014
912	N. Eguchi	Metabolites of arsenic induced tetraploids and mitotic arrest in cultured cells	1997
913	E. R. Solomon	Developmental arsenic exposure is associated with sex differences in the epigenetic regulation of stress genes in the adult mouse frontal cortex	2020
914	S. J. Carpenter	Developmental analysis of cephalic axial dysraphic disorders in arsenic-treated hamster embryos	1987
915	A. M. Mullin	Maternal blood arsenic levels and associations with birth weight-for-gestational age	2019
916	C. L. Li	Arsenic trioxide induces autophagy and antitumor effects in Burkitt's lymphoma Raji cells	2014

917	Y. Zheng	Arsenic trioxide (As(2)O(3)) induces apoptosis through activation of Bax in hematopoietic cells	2005
918	D. R. Hwang	Inhibition of hepatitis C virus replication by arsenic trioxide	2004
919	X. Jiang	Regulation of ABCG2 by nuclear factor kappa B affects the sensitivity of human lung adenocarcinoma A549 cells to arsenic trioxide	2018
920	J. Zhou	Clinical and experimental study of cardiac effects of conventional dosage arsenic trioxide in APL patients	2004
921	G. Corsi	An episode of subacute poisoning by inorganic arsenic compounds (author's transl)	1979
922	G. Yan	Inhibition of accelerated rejection mediated by alloreactive $CD4^{+}$ memory T cells and prolonged allograft survival by arsenic trioxide	2013
923	Y. H. Ling	Arsenic trioxide produces polymerization of microtubules and mitotic arrest before apoptosis in human tumor cell lines	2002
924	S. Thomas	Metals exposure and risk of small-for-gestational age birth in a Canadian birth cohort: The MIREC study	2015
925	S. Nisole	Differential Roles of PML Isoforms	2013
926	H. Y. Fu	n-MSP detection of p16 gene demethylation and transcription in human multiple myeloma U266 cell line induced by arsenic trioxide	2007
927	L. Yang	Effect of As2O3 on demethylation of SHP-1 gene in human lymphoma cell line T2	2009
928	G. Pershagen	The carcinogenicity of arsenic	1981
929	F. I. Ataullakhanov	Human erythrocyte membrane permeability for arsenate and formation of trivalent arsenic compounds	1978
930	I. E. Okonishnikova	Experimental therapy and prevention of acute poisoning with arsenic compounds	1965
931	C. W. Ballard	The determination of arsenic in organic arsenic compounds	1946
932	M. Zuccaro	Mass medico-functional evaluations	1965
933	M. O. Correa	On the treatment of trichuriasis with glycobiarsol	1962
934	R. Fischer	For Gutzeit's arsenic sample	1949

935	J. Williamson	Melarsen and melarsen oxide	1948
936	J. Qian	Arsenic trioxide in the treatment of advanced primary liver and gallbladder cancer	2001
937	J. Ni	Effects of arsenic trioxide on the subcellular localization of PML/PML-RARalpha protein in leukemic cells	1997
938	L. Song	Corrigendum to "Exposure to arsenic during pregnancy and newborn mitochondrial DNA copy number: A birth cohort study in Wuhan, China" [Chemosphere 243 (2020) 125335]	2021
939	G. Lazo	Use of arsenic trioxide (As2O3) in the treatment of patients with acute promyelocytic leukemia: the M. D. Anderson experience	2003
940	C. A. Lammon	Pretreatment with periodate-oxidized adenosine enhances developmental toxicity of inorganic arsenic in mice	2003
941	J. Cheng	Antitumor effect of arsenic trioxide in human K562 and K562/ADM cells by autophagy	2012
942	X. Cui	Arsenic trioxide inhibits DNA methyltransferase and restores methylation-silenced genes in human liver cancer cells	2006
943	X. H. Lü	Influence of As(2)O(3) on proteasome beta(1)-subunit in NB4 cells	2009
944	T. Hayashi	Arsenic trioxide inhibits growth of human multiple myeloma cells in the bone marrow microenvironment	2002
945	F. Foldvari	Experiences with the mapharsen	1949
946	H. Nabeshi	Arsenic trioxide induces down-regulation of gp46 via protein oxidation: proteomics analysis of oxidative modified proteins in As2O3-treated HTLV-1-infected cells	2010
947	Z. B. Xia	Study on inhibitory effect of arsenic trioxide on growth of rat C6 glioma cells	2008
948	H. Kreppel	Efficacy of various dithiol compounds in acute As2O3 poisoning in mice	1990
949	R. D. Hood	Uptake, distribution, and metabolism of trivalent arsenic in the pregnant mouse	1988
950	R. N. Bieter	Chemotherapy of cotton rat filariasis with certain antimony and arsenic compounds	1947
951	Z. Macek	Arsenic neuromyelitis	1949
952	A. Munoz	Synthesis of arsenotherpics	1948

953	A. Chan-Hon-Tong	Exposure to food contaminants during pregnancy	2013
954	R. Lauwerys	Arsenic speciation in urine from humans intoxicated by inorganic arsenic compounds	1985
955	L. Haffert	Processes of attenuation of dissolved arsenic downstream from historic gold mine sites, New Zealand	2008
956	R. C. Vineetha	L-Ascorbic Acid and α-Tocopherol Synergistically Triggers Apoptosis Inducing Antileukemic Effects of Arsenic Trioxide via Oxidative Stress in Human Acute Promyelocytic Leukemia Cells	2020
957	O. Bairey	Arsenic-trioxide-induced apoptosis of chronic lymphocytic leukemia cells	2010
958	D. P. Cox	Production of trimethylarsine gas from various arsenic compounds by three sewage fungi	1973
959	G. Zarković	Health changes in workers exposed to increased concentration of arsenic compounds	1971
960	D. Sumi	Signal transduction pathways and transcription factors triggered by arsenic trioxide in leukemia cells	2010
961	K. P. Siu	Effect of arsenic trioxide on human hepatocellular carcinoma HepG2 cells: inhibition of proliferation and induction of apoptosis	2002
962	D. A. Berberian	The trichomonacidal activity of milibis (glycobiarsol)	1954
963	T. H. Maren	The fate of two arsenoxides in the dog	1949
964	Hofbauer	Salvarsandermatitis treated with BAL	1948
965	J. Delort	Arseno-distance	1947
966	S. M. Ahmed	A Prospective Cohort Study Examining the Associations of Maternal Arsenic Exposure With Fetal Loss and Neonatal Mortality	2019
967	A. Lemarie	Arsenic trioxide induces apoptosis of human monocytes during macrophagic differentiation through nuclear factor-kappaB-related survival pathway down-regulation	2006
968	F. Zhang	Low dosage of arsenic trioxide inhibits vasculogenic mimicry in hepatoblastoma without cell apoptosis	2018
969	J. Zhou	Arsenic trioxide induces procoagulant activity through phosphatidylserine exposure and microparticle generation in endothelial cells	2011
970	M. Brizzi	The efficacy of ultramolecular aqueous dilutions on a wheat germination model as a function of heat and aging-time	2011

971	A. M. Ramos	Pharmacologic inhibitors of PI3K/Akt potentiate the apoptotic action of the antileukemic drug arsenic trioxide via glutathione depletion and increased peroxide accumulation in myeloid leukemia cells	2005
972	S. S. Han	Arsenic trioxide represses constitutive activation of NF-kappaB and COX-2 expression in human acute myeloid leukemia, HL-60	2005
973	A. Tanaka	Comparative study of the toxic effects of gallium arsenide, indium arsenide and arsenic trioxide following intratracheal instillations to the lung of Syrian golden hamsters	2000
974	F. Zhong	Arsenic trioxide inhibits cholangiocarcinoma cell growth and induces apoptosis	2010
975	R. Verma	Ameliorative effect of three medicinal plants (P. fraternus, Terminelia a., and Moringa oleifera) on arsenic trioxide induced alteration of lipid peroxidation and protein contents in chicken liver homogenate: an in vitro study	2007
976	Y. W. Xue	Reversal effect and mechanism of arsenic trioxide on multidrug resistance of gastric carcinoma cells SGC7901	2007
977	G. P. Qu	Arsenic trioxide inhibits the growth of human lung cancer cell lines via cell cycle arrest and induction of apoptosis at both normoxia and hypoxia	2009
978	E. Bourdonnay	Redox-sensitive regulation of gene expression in human primary macrophages exposed to inorganic arsenic	2009
979	L. Zhou	Glutathione-S-transferase pi inhibits As2O3-induced apoptosis in lymphoma cells: involvement of hydrogen peroxide catabolism	2005
980	O. S. von Ehrenstein	Children's intellectual function in relation to arsenic exposure	2007
981	C. Li	Data on arsenic trioxide modulates Treg/Th17/Th1/Th2 cells in treatment-naïve rheumatoid arthritis patients and collagen-induced arthritis model mice	2019
982	Z. Meng	Demethylation effect of inhibitor As2O3 on expression of SHP-1 and C-kit genes in leukemia HL-60 cells	2013
983	T. H. Lin	Enhancement of esculetin on arsenic trioxide-provoked apoptosis in human leukemia U937 cells	2009
984	H. Yan	Hypoxia-simulating agents and selective stimulation of arsenic trioxide-induced growth arrest and cell differentiation in acute promyelocytic leukemic cells	2005
985	W. Guo	Effects of all-trans retinoic acid and arsenic trioxide on tissue factor expression of acute promyelocytic leukemia cells	2000
986	L. A. Birari	Aloin protects against arsenic trioxide-induced myocardial membrane damage and release of inflammatory cytokines	2020
987	H. C. Ting	Humic acid enhances the cytotoxic effects of arsenic trioxide on human cervical cancer cells	2010
988	J. Shen	Effects of arsenic trioxide on apoptosis and proliferation of human lung cancer cells under hypoxia	2008

989	Z. H. Wang	Proteome analysis of nuclear matrix proteins during arsenic trioxide induced apoptosis in K562 cells	2005
990	Y. F. Wang	Inhibiting effect of arsenic trioxide on telomerase activity of NB4 and Jurkat cell lines	2003
991	V. Lecureur	Potassium antimonyl tartrate induces caspase- and reactive oxygen species-dependent apoptosis in lymphoid tumoral cells	2002
992	W. J. Chen	Urinary total arsenic and arsenic methylation capacity in pregnancy and gestational diabetes mellitus: A case-control study	2021
993	B. Fängström	Impaired arsenic metabolism in children during weaning	2009
994	A. Chatterjee	Determination of total cationic and total anionic arsenic species in oyster tissue using microwave-assisted extraction followed by HPLC-ICP-MS	2000
995	W. P. Tse	Arsenic trioxide, arsenic pentoxide, and arsenic iodide inhibit human keratinocyte proliferation through the induction of apoptosis	2008
996	M. C. Gomes	Treatment of trichocephaliasis with glycobiarsol	1962
997	C. Carducci-Artenisio	Arsenoxide chrysiodontalgic	1949
998	P. Hu	Research on the removal of As(2)O(3) by γ-Al(2)O(3) adsorption based on density functional theory	2020
999	A. Thomas-Schoeman	Arsenic trioxide exerts antitumor activity through regulatory T cell depletion mediated by oxidative stress in a murine model of colon cancer	2012
1000	S. J. Um	Down-regulation of human papillomavirus E6/E7 oncogene by arsenic trioxide in cervical carcinoma cells	2002
1001	N. K. Mak	Involvement of tumor necrosis factor (TNF-alpha) in arsenic trioxide induced apoptotic cell death of murine myeloid leukemia cells	2002
1002	D. A. Berberian	Treatment of Trichuris vulpis infection of dogs with glycobiarsol	1963
1003	D. Kim	Arsenic hexoxide has differential effects on cell proliferation and genome-wide gene expression in human primary mammary epithelial and MCF7 cells	2021
1004	M. Weatherall	The antidotal activity of some dithiols and acetyldithiols in mice poisoned with oxophenarsine	1949
1005	J. G. Basnuevo	Whipworm and pentavalent organic arsenic	1948
1006	J. E. Laine	Correction to: Maternal one carbon metabolism and arsenic methylation in a pregnancy cohort in Mexico	2019

1007	Y. Pan	Expression of survivin in adenoid cystic carcinoma of the lacrimal gland and the effect of intervention with arsenic trioxide in vitro	2015
1008	S. Park	NDRG2 Sensitizes Myeloid Leukemia to Arsenic Trioxide via GSK3β-NDRG2-PP2A Complex Formation	2019
1009	F. Gao	Expression of P-gp in acute myeloid leukemia and the reversal function of As(2)O(3) on drug resistance	2015
1010	N. Kavian	Arsenic trioxide prevents murine sclerodermatous graft-versus-host disease	2012
1011	X. Li	Biologic changes in MDS-L cell line induced by As2O3 and/or TRAIL	2004
1012	Y. Zhu	Study of As(2)O(3) regulating proliferation and apoptosis of Tca8113 cells by inhibiting the expression of Id-1	2019
1013	L. Vernhet	Inorganic arsenic induces necrosis of human CD34-positive haematopoietic stem cells	2008
1014	F. J. Lu	Arsenic as a promoter in the effect of humic substances on plasma prothrombin time in vitro	1990
1015	X. Li	Effects of arsenic poisoning on neuronal cell apoptosis and mRNA and protein expression of calpain 1, calpain 2, and cdk5/p25	2014
1016	S. S. Wang	Effect of arsenic trioxide on rat hepatocellular carcinoma and its renal cytotoxity	2003
1017	M. Oppenhei m	Pentavalent arsenic compounds in treatment of pemphigus	1950
1018	H. Kuske	Arsenic keratoses	1949
1019	H. Zhang	Comparative Studies of Effects of Vapor- and Liquid-Phase As(2)O(3) on Catalytic Behaviors of V(2)O(5)-WO(3)/TiO(2) Catalysts for NH(3)-SCR	2020
1020	Y. Wu	Preparation of PLGA microspheres loaded with 10-hydroxycamptothecin and arsenic trioxide and their treatment for rabbit hepatocellular carcinoma	2021
1021	Y. F. Xiao	Effect of arsenic trioxide on vascular endothelial growth factor-C and its receptor (VEGFR-3) in nude mice with gastric cancer	2008
1022	Y. H. Wu	Alteration of expression of survivin in HL-60 cells treated with chemotherapeutic drugs	2006
1023	J. Meulenhof f	The determination of arsenic in arsenic compounds according to the 5th edition of the netherland pharmacopeia	1965
1024	P. C. Beaver	Experimental chemoprophylaxis of amebiasis	1956

1025	M. M. Guseinov	Treatment of cutaneous leishmaniasis with organic arsenic compounds in glycerin	1953
1026	E. W. Prosser-Thomas	Treatment of arsenical reaction with BAL	1948
1027	J. A. Szymańska	Health effects of exposure of humans to inorganic arsenic compounds	1991
1028	M. Vahter	Methylation of inorganic arsenic in different mammalian species and population groups	1999
1029	T. Edwards	Rapid, sensitive method for the separation and detection of arsenic compounds in biological systems	1975
1030	M. P. Waalkes	Enhanced urinary bladder and liver carcinogenesis in male CD1 mice exposed to transplacental inorganic arsenic and postnatal diethylstilbestrol or tamoxifen	2006
1031	C. J. Meakin	Inorganic arsenic and its methylated metabolites as endocrine disruptors in the placenta: Mechanisms underpinning glucocorticoid receptor (GR) pathway perturbations	2020
1032	T. D. Ninh	Quantification of seven arsenic compounds in seafood products by liquid chromatography/electrospray ionization-single quadrupole mass spectrometry (LC/ESI-MS)	2006
1033	C. W. Liu	Tissue accumulation of arsenic compounds in aquacultural and wild mullet (Mugil cephalus)	2006
1034	D. Małysz	Assays of phosphorus compounds in the presence of arsenic compounds in medicinal preparations	1976
1035	G. Di Bacco	The colorimetric determination of compounds of phosphorus and arsenic. II. Arsenic compounds	1954
1036	D. Fano	Arsenic Concentrations in Household Drinking Water: A Cross-Sectional Survey of Pregnant Women in Tacna, Peru, 2019	2020
1037	Y. Liu	Arsenite-induced downregulation of occludin in mouse lungs and BEAS-2B cells via the ROS/ERK/ELK1/MLCK and ROS/p38 MAPK signaling pathways	2020
1038	A. Safaroghli-Azar	Stimulatory Effect of Indolic Hormone on As(2)O(3) Cytotoxicity in Breast Cancer Cells: NF-κB-dependent Mechanism of Action of Melatonin	2018
1039	R. F. da Silva	Arsenic trioxide exposure impairs testicular morphology in adult male mice and consequent fetus viability	2017
1040	Y. Mei	Arsenic trioxide induces apoptosis of fibroblast-like synoviocytes and represents antiarthritis effect in experimental model of rheumatoid arthritis	2011
1041	Y. Gao	Effects of arsenic trioxide under different administration ways on T-cell lymphoma xenografts in nude mice: in vivo and in vitro experiments	2009
1042	C. W. Du	Arsenic trioxide reduces the invasive and metastatic properties of nasopharyngeal carcinoma cells in vitro	2006

1043	J. Karlsson	Arsenic trioxide-induced death of neuroblastoma cells involves activation of Bax and does not require p53	2004
1044	Y. Jing	Combined effect of all-trans retinoic acid and arsenic trioxide in acute promyelocytic leukemia cells in vitro and in vivo	2001
1045	S. A. Niño	Demyelination associated with chronic arsenic exposure in Wistar rats	2020
1046	R. I. Neamtu	Heavy metal ion concentration in the amniotic fluid of preterm and term pregnancies from two cities with different industrial output	2022
1047	M. Pereiro Miguens	A case of syphilis with intolerance to arsenical therapy	1949
1048	F. Zhang	Hyperoside enhances the suppressive effects of arsenic trioxide on acute myeloid leukemia cells	2015
1049	F. Gao	Ascorbic acid enhances the apoptosis of U937 cells induced by arsenic trioxide in combination with DMNQ and its mechanism	2002
1050	I. Delnon	On the therapy of leukorrhea with Wynal	1961
1051	M. Vahter	Prenatal and childhood arsenic exposure through drinking water and food and cognitive abilities at 10 years of age: A prospective cohort study	2020
1052	A. Marçais	Arsenic trioxide (As(2)O(3)) as a maintenance therapy for adult T cell leukemia/lymphoma	2020
1053	S. W. Li	Assessment of arsenic trioxide toxicity on cock muscular tissue: alterations of oxidative damage parameters, inflammatory cytokines and heat shock proteins	2017
1054	K. Ishitsuka	Arsenic trioxide induces apoptosis in HTLV-I infected T-cell lines and fresh adult T-cell leukemia cells through CD95 or tumor necrosis factor alpha receptor independent caspase activation	2002
1055	Z. Shen	Morphological changes of mitochondria in apoptosis of esophageal carcinoma cells induced by As(2)O(3)	2000
1056	N. P. Foo	Arsenic compounds activate the MAPK and caspase pathways to induce apoptosis in OEC-M1 gingival epidermal carcinoma	2020
1057	B. Mondal	Modulation of Cellular Response to Arsenic Trioxide Toxicity by Resveratrol	2018
1058	I. Marotti	Transcriptome Profiling of Wheat Seedlings following Treatment with Ultrahigh Diluted Arsenic Trioxide	2014
1059	F. Raffoul	SnoN/SkiL, a TGFβ signaling mediator: a participant in autophagy induced by arsenic trioxide	2010
1060	W. Zhao	Tissue factor expression during all-trans retinoic acid or arsenic trioxide treatment in acute promyelocytic leukemia	1998

1061	H. Grajeta	Human health hazard caused by pollution of the natural environment by arsenic compounds	1987
1062	P. H. Van Thiel	Therapy of experimental toxoplasmosis with some sulfanomides, arsenic compounds and antimalarials	1949
1063	S. Shimotsuura	Antineoplastic action of As2O3	1986
1064	Y. Ye	Arsenic trioxide induces regulatory functions of plasmacytoid dendritic cells through interferon- α inhibition	2020
1065	M. V. Varghese	Attenuation of arsenic trioxide induced cardiotoxicity through flaxseed oil in experimental rats	2017
1066	S. Rahman	E2F1-mediated FOS induction in arsenic trioxide-induced cellular transformation: effects of global H3K9 hypoacetylation and promoter-specific hyperacetylation in vitro	2015
1067	R. Z. Lu	Arsenic trioxide inhibits cell growth in imatinib-resistant bcr-abl mutant cell lines in vitro	2009
1068	Z. H. Li	Effect of arsenic trioxide on apoptosis in lymphoblastoid Raji cell line with relation to expression of mcl-1 gene	2009
1069	Y. X. Cai	In vitro effect of bortezomib alone or in combination with harringtonine or arsenic trioxide on proliferation and apoptosis of multidrug resistant leukemia cells	2008
1070	S. Müller	Trivalent antimonials induce degradation of the PML-RAR oncoprotein and reorganization of the promyelocytic leukemia nuclear bodies in acute promyelocytic leukemia NB4 cells	1998
1071	A. Rahman	Infant and mother related outcomes from exposure to metals with endocrine disrupting properties during pregnancy	2016
1072	K. K. Mann	Death by arsenic: implications of PML sumoylation	2004
1073	M. C. Estañ	Apoptotic efficacy of etomoxir in human acute myeloid leukemia cells. Cooperation with arsenic trioxide and glycolytic inhibitors, and regulation by oxidative stress and protein kinase activities	2014
1074	N. P. Govorov	Arsenic compounds in the prevention of lung diseases in young animals	1968
1075	M. E. Auerbach	A spectrophotometric method for the assay of glycobiarsol	1960
1076	C. S. Leonard	Influence of oxophenarsine on hypoglycemic action of insulin	1948
1077	N. M. Mónaco	Low arsenic concentrations impair memory in rat offpring exposed during pregnancy and lactation: Role of α7 nicotinic receptor, glutamate and oxidative stress	2018
1078	Y. Hubert	Arsenic and respiratory neoplasms, risks, prevention	1988

1079	A. Arnold	The determination of arsenic in organic and inorganic arsenic compounds: a radioisotope-dilution substoicheiometric application	1969
1080	H. E. Henderson	The human isospora	1963
1081	P. Oudot	Chophytol in the prophylaxis of arsenic medication accidents	1947
1082	P. Colombo	On the arseno-sensitivity of the megaloblast	1947
1083	B. Dattner	BAL in arsenical optic neuritis	1949
1084	H. Garner	Arsenical polyneuritis	1948
1085	Y. Guo	Effects of Arsenic Trioxide Exposure on Heat Shock Protein Response in the Immune Organs of Chickens	2016
1086	A. Karimi	Exposure of hepatocellular carcinoma cells to low-level As_2O_3 causes an extra toxicity pathway via L1 retrotransposition induction	2014
1087	J. H. Park	Combination treatment with arsenic trioxide and sulindac enhances apoptotic cell death in lung cancer cells via activation of oxidative stress and mitogen-activated protein kinases	2008
1088	B. Liang	Maintenance therapy with all-trans retinoic acid and arsenic trioxide improves relapse-free survival in adults with low- to intermediate-risk acute promyelocytic leukemia who have achieved complete remission after consolidation therapy	2017
1089	P. S. Ong	Microarray analysis revealed dysregulation of multiple genes associated with chemoresistance to As(2)O(3) and increased tumor aggressiveness in a newly established arsenic-resistant ovarian cancer cell line, OVCAR-3/AsR	2012
1090	K. Zhang	Middle-low-temperature oxidation and adsorption of arsenic from flue gas by Fe-Ce-based composite catalyst	2022
1091	C. Yedjou	GENOTOXIC MECHANISMS OF ARSENIC TRIOXIDE IN HUMAN JURKAT T-LYMPHOMA CELLS	2008
1092	T. Kumagai	19-Nor-1,25(OH)2D2 (a novel, noncalcemic vitamin D analogue), combined with arsenic trioxide, has potent antitumor activity against myeloid leukemia	2005
1093	Z. Y. Shen	The inhibition of growth and angiogenesis in heterotransplanted esophageal carcinoma via intratumoral injection of arsenic trioxide	2003
1094	H. L. Wei	Arsenic trioxide inhibits P-glycoprotein expression in multidrug-resistant human leukemia K562/ADM cell line that overexpresses mdr-1 gene and enhances their chemotherapeutic sensitivity	2003
1095	E. M. Rego	Retinoic acid (RA) and As2O3 treatment in transgenic models of acute promyelocytic leukemia (APL) unravel the distinct nature of the leukemogenic process induced by the PML-RARalpha and PLZF-RARalpha oncoproteins	2000
1096	P. Rousselot	Arsenic trioxide and melarsoprol induce apoptosis in plasma cell lines and in plasma cells from myeloma patients	1999

1097	I. G. White	Arsenic poisoning in sheep	1948
1098	R. C. Webster	Arsenical encephalopathy treated with BAL	1948
1099	S. Xu	Arsenic trioxide combined with co-stimulatory molecule blockade prolongs survival of cardiac allografts in alloantigen-primed mice	2010
1100	E. Dopp	Subcellular distribution of inorganic and methylated arsenic compounds in human urothelial cells and human hepatocytes	2008
1101	S. Katano	Arsenic compounds accumulated in pearl oyster Pinctada fucata	2003
1102	H. S. Ducoff	Biological studies with arsenic; excretion and tissue localization	1948
1103	J. M. Gernay	Action of arsenic derivatives on the striated frog muscle	1948
1104	E. Oguz	Effect of lycopene on As(2)O(3) induced oxidative stress in SH-SY5Y cells	2021
1105	X. C. Huang	Synergistic effects of arsenic trioxide combined with ascorbic acid in human osteosarcoma MG-63 cells: a systems biology analysis	2014
1106	B. L. Powell	Arsenic trioxide improves event-free and overall survival for adults with acute promyelocytic leukemia: North American Leukemia Intergroup Study C9710	2010
1107	B. Cheng	Arsenic trioxide induced the apoptosis of laryngeal cancer via down-regulation of survivin mRNA	2008
1108	D. L. Dong	Arsenic trioxide-induced hela cell death is partially prevented by K+ channel blockers	2005
1109	N. Cherry	Stillbirth in rural Bangladesh: arsenic exposure and other etiological factors: a report from Gonoshasthaya Kendra	2008
1110	A. J. Henegar	Native Oxide Transport and Removal During Atomic Layer Deposition of TiO2 Films on GaAs(100) Surfaces	2016
1111	J. F. Shanaphy	Leukorrhea therapy of 510 cases	1955
1112	S. Grzycki	Arsenic and arsenic compounds in the digestive mucosa	1954
1113	J. Pellerat	Accidental dermatitis in workers working in the manufacture of arsenical products	1949
1114	E. M. Beaucham p	Direct binding of arsenic trioxide to AMPK and generation of inhibitory effects on acute myeloid leukemia precursors	2015

1115	R. A. Call	Arsenical encephalopathy; report of a case	1949
1116	L. W. Spolyar	Generation of deadly amount of arsine from dross	1949
1117	M. Rahman	Prenatal arsenic exposure and drowning among children in Bangladesh	2015
1118	D. C. Koestler	Differential DNA methylation in umbilical cord blood of infants exposed to low levels of arsenic in utero	2013
1119	P. Navasumrit	Exposure to arsenic in utero is associated with various types of DNA damage and micronuclei in newborns: a birth cohort study	2019
1120	T. Ueki	Delayed hematological recovery following autologous transplantation utilizing peripheral blood stem cells harvested after treatment with arsenic trioxide	2008
1121	L. Betti	Extremely low doses of arsenic affect in vitro pollen germination	2013
1122	X. W. Zhang	Arsenic trioxide controls the fate of the PML-RARalpha oncoprotein by directly binding PML	2010
1123	S. Ichikawa	Ingestion and excretion of arsenic compounds present in edible brown algae, Hijikia fusiforme, by mice	2010
1124	P. Kouri	Arsenical toxic reactions; idiosyncrasy or hyper-susceptibility to these drugs	1948
1125	F. Coste	Recurrence of arsenical arythroderma by the use of a tonic wine	1947
1126	D. H. Clark	Drugs for vaginal trichomoniasis: results of a comparative study	1962
1127	F. Starey	Experiences with viasept in the treatment of trichuriasis	1959
1128	J. Shen	Fetal onset of aberrant gene expression relevant to pulmonary carcinogenesis in lung adenocarcinoma development induced by in utero arsenic exposure	2007
1129	L. Wang	Arsenic trioxide is an immune adjuvant in liver cancer treatment	2017
1130	L. L. Wang	Efficacy Analysis of Arsenic Trioxide Combined with All Trans Retinoic Acid for Acute Promyelocytic Leukemia	2015
1131	M. M. Wang	Arsenic trioxide induces socs-1 gene demethylation in myeloma cell lines	2008
1132	C. Bermudes	Considerations on the modern treatment of leukorrhea	1962

1133	A. Strickler	Arsenical treatment for pemphigus	1948
1134	R. Kubota	Chemical speciation of arsenic in the livers of higher trophic marine animals	2002
1135	J. Liu	Transplacental arsenic plus postnatal 12-O-teradecanoyl phorbol-13-acetate exposures associated with hepatocarcinogenesis induce similar aberrant gene expression patterns in male and female mouse liver	2006
1136	A. Rahman	Arsenic exposure during pregnancy and size at birth: a prospective cohort study in Bangladesh	2009
1137	N. Abdollahzade	Attenuation of chronic arsenic neurotoxicity via melatonin in male offspring of maternal rats exposed to arsenic during conception: Involvement of oxidative DNA damage and inflammatory signaling cascades	2021
1138	D. Gilbert-Diamond	Rice consumption contributes to arsenic exposure in US women	2011
1139	W. Zhang	Resveratrol attenuates hepatotoxicity of rats exposed to arsenic trioxide	2013
1140	X. P. Qian	Effect of arsenic trioxide on drug transporting molecules in acute promyelocytic leukemia cell line	2004
1141	L. Chen	Synergistic inhibitory effect of STI571 in combination with arsenic trioxide on a multidrug-resistant leukemia cell line expressing bcr-abl	2004
1142	J. Yi	The inherent cellular level of reactive oxygen species: one of the mechanisms determining apoptotic susceptibility of leukemic cells to arsenic trioxide	2002
1143	C. H. Yang	Arsenic trioxide sensitivity is associated with low level of glutathione in cancer cells	1999
1144	K. Davison	Glutathione depletion overcomes resistance to arsenic trioxide in arsenic-resistant cell lines	2003
1145	Q. Tong	Apoptosis inducing effects of arsenic trioxide on human bladder cancer cell line BIU-87	2001
1146	V. Vondracek	Concentration of 3,4-benzpyrene and arsenic compounds in the prague atmosphere	1963
1147	J. Száková	Comparison of mild extraction procedures for determination of plant-available arsenic compounds in soil	2005
1148	M. A. Rebollo	General paralysis in children	1964
1149	C. K. Banks	3-Amino-4-hydroxybenzenearsonic acid; oxophenarsine hydrochloride; decomposition rate	1949
1150	F. P. Merklen	Distribution of arsenic in the blood between plasma and red blood cells	1949

1151	M. Scott-Richardson	Examining the Relationship Between Low Birth Weight Occurrence and Passive Measures of Environmental Arsenic by Census Tract in Escambia and Santa Rosa Counties, Florida	2020
1152	C. Q. Feng	Analysis with DNA chips of the changes of gene expressions in K562 cells in response to As2O3 treatment	2002
1153	T. C. Zhang	Opposite biological effects of arsenic trioxide and arsacetin involve a different regulation of signaling in human gastric cancer MGC-803 cells	2002
1154	C. A. Nau	The accidental generation of arsine gas in an industry	1948
1155	Z. M. Bacq	Thioloprive bodies; New treatment for arsenical poisoning	1947
1156	Y. Wang	lncRNA OTUD6B-AS1 Exacerbates As(2)O(3)-Induced Oxidative Damage in Bladder Cancer via miR-6734-5p-Mediated Functional Inhibition of IDH2	2020
1157	R. C. Vineetha	L-Ascorbic Acid and α-Tocopherol Reduces Hepatotoxicity Associated with Arsenic Trioxide Chemotherapy by Modulating Nrf2 and Bcl2 Transcription Factors in Chang liver Cells	2018
1158	R. B. Gartenhaus	Arsenic trioxide cytotoxicity in steroid and chemotherapy-resistant myeloma cell lines: enhancement of apoptosis by manipulation of cellular redox state	2002
1159	E. B. Cosendey	STUDIES ON TRICHOCEPHALIASIS IN CHILDHOOD	1963
1160	X. H. Jiang	Arsenic trioxide induces apoptosis in human gastric cancer cells through up-regulation of p53 and activation of caspase-3	2001
1161	F. Lo-Coco	Trisenox: a paradigm shift in APL therapy, an interview with Francesco Lo-Coco	2016
1162	Fruhwald	Treatment of neurosyphilis	1955
1163	H. Griffon	Double poisoning by arsenic hydrogen, including one fatal, during a descaling operation	1949
1164	S. M. Harris	Identification of environmental chemicals targeting miscarriage genes and pathways using the comparative toxicogenomics database	2020
1165	A. Sandoval-Carrillo	Arsenic exposure and risk of preeclampsia in a Mexican mestizo population	2016
1166	S. Trojan	Effect of arsenic compounds on resistance of the central nervous system of the rat to hypoxia and anoxia during ontogenesis	1967
1167	O. Arias	Historical aspects of pinta	1964
1168	H. Liu	Maternal arsenic exposure and birth outcomes: A birth cohort study in Wuhan, China	2018

1169	H. H. Fox	Arsenicals; sulfonyloxy, sulfonamido, and benzyloxy derivatives of phenylarsonic acids and their reduction products	1947
1170	L. He	A Meta-Analysis of Arsenic Trioxide Combined with Transcatheter Arterial Chemoembolization for Treatment of Primary Hepatic Carcinoma	2016
1171	Y. Molin	Sequential effects of daily arsenic trioxide treatment on essential and nonessential trace elements in tissues in mice	2008
1172	J. Wu	Inhibitory effect of sorafenib combined with arsenic trioxide on hepatocellular carcinoma cells	2008
1173	L. Vernhet	Resistance of human multidrug resistance-associated protein 1-overexpressing lung tumor cells to the anticancer drug arsenic trioxide	2001
1174	C. V. Rudge	The placenta as a barrier for toxic and essential elements in paired maternal and cord blood samples of South African delivering women	2009
1175	B. J. Lagerkvist	Biological monitoring of arsenic, lead and cadmium in occupationally and environmentally exposed pregnant women	1993
1176	A. Furuya	Central nervous system involvement of acute promyelocytic leukemia, three case reports	2017
1177	Y. P. Zhu	Fluoride and arsenic exposure affects spatial memory and activates the ERK/CREB signaling pathway in offspring rats	2017
1178	E. Gyorgy	The jaundices of syphilitics treated by arseno-benzene	1947
1179	X. Jiang	Critical role of cellular glutathione homeostasis for trivalent inorganic arsenite-induced oxidative damage in human bronchial epithelial cells	2014
1180	A. Ray	As2O3 toxicity in rat hepatocytes: manifestation of caspase-mediated apoptosis	2008
1181	F. Chen	The influence of arsenic trioxide combined with cisplatin on the growth and expression of X-linked inhibitor of apoptosis protein, XIAP of human non-small cell lung cancer cells	2007
1182	R. Piga	Cytotoxic effects of various stressors on PC12 cells: involvement of oxidative stress and effect of antioxidants	2007
1183	B. Singh	Arsenical encephalopathy during treatment of tropical eosinophilia	1948
1184	D. T. Marsh	The comparative pharmacology of the N-alkyl-arterenols	1948
1185	R. L. Frost	Raman spectroscopy of selected arsenates--implications for soil remediation	2003
1186	B. A. Katsnel'son	Substantiation of a unified hygienic standard for inorganic arsenic compounds in the air of the work area	1988

1187	C. K. Banks	The determination of arsenic in organic compounds	1948
1188	J. Grubmuller	Arsenic polyneuritis after therapeutic arsenic doses	1947
1189	J. Zhou	Effects of arsenic trioxide administration styles on leukocytosis	2006
1190	M. U. Khan	A case of arsenical dermatitis	1947
1191	J. D. Brender	Agricultural Compounds in Water and Birth Defects	2016
1192	L. Zhang	Near infrared imaging of EGFR of oral squamous cell carcinoma in mice administered arsenic trioxide	2012
1193	H. J. Kuehnelt	New possibilities of leukorrhea therapy in practice	1962
1194	M. P. Waalkes	Transplacental arsenic carcinogenesis in mice	2007
1195	A. A. Hemmati	Ellagic acid protects against arsenic trioxide-induced cardiotoxicity in rat	2018
1196	Y. P. Yen	Arsenic induces apoptosis in myoblasts through a reactive oxygen species-induced endoplasmic reticulum stress and mitochondrial dysfunction pathway	2012
1197	X. Bi	Different pathways are involved in arsenic-trioxide-induced cell proliferation and growth inhibition in human keratinocytes	2010
1198	T. Wang	Arsenic trioxide combined with buthionine sulfoximine enhances apoptosis in multidrug-resistant human leukemia K562/ADM cells in vitro	2008
1199	J. Zhu	The molecular mechanism of arsenic trioxide-induced apoptosis and oncosis in leukemia/lymphoma cell lines	2003
1200	C. R. Kumana	Systemic availability of arsenic from oral arsenic-trioxide used to treat patients with hematological malignancies	2002
1201	C. Osorio-Yáñez	Dietary intake and urinary metals among pregnant women in the Pacific Northwest	2018
1202	L. De Leener	Serious accident following an intramuscular injection of trivalent arsenic	1948
1203	R. L. Straube	Biological studies with arsenic; preparation of arsenic by pile irradiation of cacodylic acid	1948
1204	M. L. Kile	Prenatal arsenic exposure and DNA methylation in maternal and umbilical cord blood leukocytes	2012

1205	F. Cifuentes	Synchronization in the Heart Rate and the Vasomotion in Rat Aorta: Effect of Arsenic Trioxide	2016
1206	F. X. Reichl	Effect of glucose treatment on carbohydrate content in various organs in mice after acute As2O3 poisoning	1991
1207	S. Zarazúa	Decreased arginine methylation and myelin alterations in arsenic exposed rats	2010
1208	J. Liu	Fetal arsenic exposure appears to facilitate endocrine disruption by postnatal diethylstilbestrol in neonatal mouse adrenal	2009
1209	W. Weistenhöfer	Tracing for arsenic exposure--a differentiation of arsenic compounds is essential for the health assessment	2016
1210	Y. Yoon	Phytotoxicity of arsenic compounds on crop plant seedlings	2015
1211	E. Argese	Distribution of arsenic compounds in Mytilus galloprovincialis of the Venice lagoon (Italy)	2005
1212	S. Hukovic	Protective effect of histamine liberator 48/80 against fatal doses of arsenic compounds	1960
1213	H. H. Anderson	Thioarsenites in amebiasis; a clinical appraisal of new amebacides	1949
1214	F. Serri	Arsenobenzols, arsenoxides and prothrombinemia; clinical and experimental research	1949
1215	J. P. Albou	In the form of a "necrologie": As2O3	1981
1216	G. Bisht	Uv - induced synthesis of amino acids from aqueous sterilized solution of ammonium formate and ammonia under hetrogeneous conditions	1990
1217	Y. S. Pu	OGG1 and MYH are involved in the incision of trivalent arsenical-induced DNA adducts	2006
1218	Y. W. Jing	Dicoumarol alters cellular redox state and inhibits nuclear factor kappa B to enhance arsenic trioxide-induced apoptosis	2004
1219	K. Ishitsuka	Bone marrow necrosis in a patient with acute promyelocytic leukemia during re-induction therapy with arsenic trioxide	2004
1220	V. D. Warner	Infections caused by resistant organisms: Could organic arsenic compounds be an effective treatment?	2017
1221	B. Cristau	Routes and kinetics of arsenic elimination in rats after administration of organic arsenic compounds. II. Study of melarsoprol and potassic melarsonyl	1972
1222	L. V. Beck	Action of BAL on the tumor-damaging potency of certain trivalent arsenic compounds	1952

1223	C. K. Banks	3-Amino-4-hydroxybenzenearsonous acid; oxophenarsine hydrochloride; decomposition products	1949
1224	E. R. Gelmann	A pilot study: the importance of inter-individual differences in inorganic arsenic metabolism for birth weight outcome	2013
1225	S. A. Niño	Cortical Synaptic Reorganization Under Chronic Arsenic Exposure	2021
1226	Y. Suzuki	Rapid and effective speciation analysis of arsenic compounds in human urine using anion-exchange columns in HPLC-ICP-MS	2009
1227	R. A. Peters	Biochemical injury in arsenical intoxication and the British Anti-Lewisite	1948
1228	H. Huellstrung	About the ability of organic arsenic compounds to penetrate the nerve fluid	1946
1229	O. A. Pereira	Trichuriasis, General considerations. (Trichurotic?) hemorrhagic typhlitis. Therapy with glycobiarsol	1962
1230	O. Delzant	Impetiginiform lesions in arsenic resistance in a child treated for two years with sulfarsenol	1947
1231	Q. F. Ge	Effects of arsenic trioxide combined with bortezomib on apoptosis of multiple myeloma cell line KM3 and its mechanisms	2012
1232	J. Q. Li	Re-expression of p16 gene in myeloma cell line U266 by arsenic trioxide	2004
1233	V. D. Martinez	Health Effects Associated With Pre- and Perinatal Exposure to Arsenic	2021
1234	P. Hu	Effects of O(2), SO(2), H(2)O and CO(2) on As(2)O(3) adsorption by γ-Al(2)O(3) based on DFT analysis	2021
1235	J. L. McNeer	Arsenic trioxide-dependent activation of thousand-and-one amino acid kinase 2 and transforming growth factor-beta-activated kinase 1	2010
1236	D. L. Fei	Association between In Utero arsenic exposure, placental gene expression, and infant birth weight: a US birth cohort study	2013
1237	L. Li	Nutritional status has marginal influence on the metabolism of inorganic arsenic in pregnant Bangladeshi women	2008
1238	G. Lammler	Chemotherapy of trichuriasis; studies on experimental infection of dogs with Trichuris vulpis	1958
1239	A. Milzer	Treatment of intestinal amebiasis with bismuth glycolyl arsanilate	1956
1240	E. De Gregorio	Contribution to the study of arsenical lichen planus	1948

1241	M. P. Muñoz	Urinary Inorganic Arsenic Concentration and Gestational Diabetes Mellitus in Pregnant Women from Arica, Chile	2018
1242	F. Moghaddaskho	Demethylation and alterations in the expression level of the cell cycle-related genes as possible mechanisms in arsenic trioxide-induced cell cycle arrest in human breast cancer cells	2017
1243	M. C. Terek	Arsenic trioxide-loaded, microemulsion-enhanced cytotoxicity on MDAH 2774 ovarian carcinoma cell line	2006
1244	M. V. Milich	How to evaluate a positive serological reaction after the completion of antisyphilitic treatment?	1964
1245	H. F. Huang	Regulation of all-trans retinoic acid and arsenic trioxide on CD44v6 expression in NB4 cells	2012
1246	Z. Chen	Acute promyelocytic leukemia: cellular and molecular basis of differentiation and apoptosis	1997
1247	X. Huang	PRMT5-mediated RNF4 methylation promotes therapeutic resistance of APL cells to As(2)O(3) by stabilizing oncoprotein PML-RARα	2022
1248	M. Riser	Treatment of neurosyphilis; classical chemotherapy or antibiotics?	1955
1249	G. L. Rocha	Syphilis: modern methods of treatment	1953
1250	A. Lemarie	Inorganic arsenic activates reduced NADPH oxidase in human primary macrophages through a Rho kinase/p38 kinase pathway	2008
1251	U. Norum	Arsenic compounds in the haemolymph of the Dungeness crab, Cancer magister, as determined by using HPLC on-line with inductively coupled plasma mass spectrometry	2005
1252	E. Bourdonnay	Global effects of inorganic arsenic on gene expression profile in human macrophages	2009
1253	Y. L. Lai	Combined effect of topical arsenic trioxide and radiation therapy on skin-infiltrating lesions of breast cancer-a pilot study	2003
1254	S. Müller	Conjugation with the ubiquitin-related modifier SUMO-1 regulates the partitioning of PML within the nucleus	1998
1255	S. Liu	Effect of RNA Interference-silenced TAK1 on Kasumi-1 cell Proliferation Inhibition Induced by As(2)O(3) and Its Mechanism	2017
1256	W. J. Reich	Trichomonas vaginitis; diagnosis and treatment with milibis	1955
1257	P. Armando	More comments on amebiasis in Honduras	1955
1258	J. L. Switzer	Marked eosinophilia secondary to chronic uncomplicated amebiasis; recession with carbarsone	1949

1259	E. Doerr Zavala	Bismuth glycolylarsanilate and fumagillin in therapy of intestinal amebiasis	1954
1260	A. D. Infante	New antiamebic drugs: bismuth glycolylarsanilate and fumagillin	1953
1261	F. Ravenna	On a new antidote against arsenic and heavy metal poisoning, it BAL	1947
1262	D. Chakraborti	Arsenic groundwater contamination and its health effects in Patna district (capital of Bihar) in the middle Ganga plain, India	2016
1263	H. R. Bird	Effect of arsonic acid derivatives in stimulating growth of chicks fed certain diets	1948
1264	J. Lee	Arsenicals, the Integrated Stress Response, and Epstein-Barr Virus Lytic Gene Expression	2021
1265	Y. Ye	Low-dose arsenic trioxide combined with aclacinomycin A synergistically enhances the cytotoxic effect on human acute myelogenous leukemia cell lines by induction of apoptosis	2015
1266	K. M. Kodigepalli	Phospholipid Scramblase 1, an interferon-regulated gene located at 3q23, is regulated by SnoN/SkiL in ovarian cancer cells	2013
1267	A. Mirzaei	Human prostate cancer cell epithelial-to-mesenchymal transition as a novel target of arsenic trioxide and curcumin therapeutic approach	2022
1268	X. Wang	Arsenic trioxide in the mechanism of drug resistance reversal in MCF-7/ADM cell line of human breast cancer	2002
1269	K. Imaeda	Studies on oxygen determination by carrier gas methods. IV. Determination of oxygen in arsenic and arsenic compounds (author's transl)	1973
1270	E. Arias Vallejo	Homologous serum jaundice and jaundice of syphilitics treated with arsenicals	1949
1271	G. Garnier	Review Of the Treatment Of Recent Syphilis By Arsenins	1948
1272	C. Li	Arsenic trioxide improves Treg and Th17 balance by modulating STAT3 in treatment-naïve rheumatoid arthritis patients	2019
1273	C. M. Yen	Novel Combination of Arsenic Trioxide (As(2)O(3)) Plus Resveratrol in Inducing Programmed Cell Death of Human Neuroblastoma SK-N-SH Cells	2018
1274	P. Song	Arsenic trioxide combined with transarterial chemoembolization for unresectable primary hepatic carcinoma: A systematic review and meta-analysis	2018
1275	L. Yang	Taurine protects against arsenic trioxide-induced insulin resistance via ROS-Autophagy pathway in skeletal muscle	2019
1276	C. L. Wang	Influence of Fe_3O_4Magnetic Nanoparticles Combined with As2O3 and Adriamycin on Raji Cell Apoptosis and Autophagy	2015

1277	N. Haga	Involvement of mitochondrial aggregation in arsenic trioxide (As2O3)-induced apoptosis in human glioblastoma cells	2005
1278	E. D. Karlovsky	Milibis-tampax in vaginal infection	1955
1279	P. Liu	Co-exposure to fluoride and arsenic disrupts intestinal flora balance and induces testicular autophagy in offspring rats	2021
1280	P. G. Smith	An investigation of arsenic compounds in fur and feathers using X-ray absorption spectroscopy speciation and imaging	2008
1281	R. Borno	New perspectives in the treatment of vaginitis	1958
1282	C. Huriez	Interest in the measurement of arsenemia during the treatment of recent syphilis	1949
1283	H. Peng	Suppression of NRF2-ARE activity sensitizes chemotherapeutic agent-induced cytotoxicity in human acute monocytic leukemia cells	2016
1284	J. M. Smeller	Potassium Bromate Assay by Redox Titrimetry Using Arsenic Trioxide	2003
1285	J. Tempski	Agranulocytosis during therapy of syphilis	1955
1286	F. Rovello	Effect of trivalent arsenic compounds on prothrombin	1953
1287	L. M. Farner	The hazards associated with the use of lead arsenate in apple orchards	1949
1288	M. A. Valdés Salgado	Is prenatal arsenic exposure associated with salivary cortisol in infants in Arica, Chile? An exploratory cohort study	2019
1289	M. F. Naujokas	The broad scope of health effects from chronic arsenic exposure: update on a worldwide public health problem	2013
1290	B. M. Welch	Evaluating the effects between metal mixtures and serum vaccine antibody concentrations in children: a prospective birth cohort study	2020
1291	R. Ríos	Decreased nitric oxide markers and morphological changes in the brain of arsenic-exposed rats	2009
1292	Y. P. Wang	Concentration of arsenic trioxide in animal tongue tissue by transartery catheterization	2000
1293	M. A. Hussein	Arsenic trioxide: a new immunomodulatory agent in the management of multiple myeloma	2001
1294	A. Marmasse	Is the condemnation of arsenic (As-2-O-3) justified?	1965

1295	T. G. Rossman	Genetic and epigenetic effects of environmental arsenicals	2011
1296	C. S. Blom-Ides	On the frequency of the Jarisch-Herxheimer reaction	1955
1297	W. S. Liu	Polymorphisms in arsenic (+ 3 oxidation state) methyltransferase (AS3MT) predict the occurrence of hyperleukocytosis and arsenic metabolism in APL patients treated with As(2)O(3)	2020
1298	B. Gesundheit	Neuroblastoma cell death is induced by inorganic arsenic trioxide (As(2)O(3)) and inhibited by a normal human bone marrow cell-derived factor	2008
1299	D. Amrán	Arsenic trioxide sensitizes promonocytic leukemia cells to TNFalpha-induced apoptosis via p38-MAPK-regulated activation of both receptor-mediated and mitochondrial pathways	2007
1300	D. R. Webb	Comparative pulmonary toxicity of gallium arsenide, gallium(III) oxide, or arsenic(III) oxide intratracheally instilled into rats	1986
1301	U. Giri	Elimination of the differential chemoresistance between the murine B-cell lymphoma LY-ar and LY-as cell lines after arsenic (As2O3) exposure via the overexpression of gsto1 (p28)	2005
1302	Not found	Regulation of trade and use of soluble mineral arsenic compounds for the destruction of insects and harmful animals in agriculture	1950
1303	S. Hawkesworth	Early exposure to toxic metals has a limited effect on blood pressure or kidney function in later childhood, rural Bangladesh	2013
1304	J. Liu	Arsenic-induced aberrant gene expression in fetal mouse primary liver-cell cultures	2008
1305	T. Narukawa	A New Candidate Reference Material for Inorganic Arsenic and Arsenosugars in Hijiki Seaweed: First Results from an Inter-laboratory Study	2020
1306	Z. Slejkovec	Arsenosugars and other arsenic compounds in littoral zone algae from the Adriatic Sea	2006
1307	L. Wang	A proteomics research of apoptosis of leukemia cells induced by arsenic trioxide	2017
1308	L. Wang	Arsenic trioxide inhibits lung metastasis of mouse colon cancer via reducing the infiltration of regulatory T cells	2016
1309	S. Fukuda	Phosphatidylarsenocholine, one of the major arsenolipids in marine organisms: synthesis and metabolism in mice	2011
1310	A. E. Geiszinger	The marine polychaete Arenicola marina: its unusual arsenic compound pattern and its uptake of arsenate from seawater	2002
1311	M. Vahter	Concentrations of arsenic in urine of the general population in Sweden	1986
1312	X. J. Ma	Efficacy analysis of sequential treatment with chemotherapy, ATRA and As(2)O(3) for acute promyelocytic leukemia	2010

1313	L. Rausch	Studies in blood chemistry regarding the toxicity of the arsenobenzol-bismuth treatment	1955
1314	P. T. Pisciotto	Induction of mucosal glutathione synthesis by arsenic	1980
1315	B. George	Treatment of children with newly diagnosed acute promyelocytic leukemia with arsenic trioxide: a single center experience	2004
1316	Y. S. Pu	Arsenic trioxide as a novel anticancer agent against human transitional carcinoma--characterizing its apoptotic pathway	2002
1317	F. Wasik	Remote results of the treatment of early syphilis with penicillin and arsenic-bismuth compounds	1961
1318	S. C. Kasdon	Postarsenical encephalopathy in the treatment of syphilis in women	1948
1319	Z. Somolnokyova	The effect of arsenic compounds on the quality of drinking and superficial waters in the area of a heat-electric works eno	1963
1320	J. L. Carrera	Relative innoxiousness of bismuth-arsenic treatments during the last years of use of said treatment	1955
1321	W. H. Tucker	Treatment and prognosis of amebiasis	1953
1322	L. Tropeano	Potassium arsenite, Fowler's arsenical liqueur, in the treatment of chronic leukemic myelosis	1949
1323	J. E. Laine	Maternal arsenic exposure, arsenic methylation efficiency, and birth outcomes in the Biomarkers of Exposure to ARsenic (BEAR) pregnancy cohort in Mexico	2015
1324	H. Prochacki	Remote results of the treatment of early symptomatic syphilis with penicillin and 1 or 2 arsenobismuth cures in 1947-1957	1962
1325	L. V. Beck	Effect of growth of mouse sarcoma 37 on resistance of mice to semilethal doses of certain trivalent arsenic compounds	1954
1326	H. Thaler	A case of intrahepatic occlusive icterus related to the ninth day response to arsenobenzene treatment	1949
1327	J. L. Sawyers	Condensation of 2,3-dimercaptopropanol with oxophenarsine hydrochloride; toxicity and chemotherapeutic effect	1949
1328	D. Guha Mazumder	Chronic arsenic toxicity: studies in West Bengal, India	2011
1329	M. V. Varghese	Omega-3 Fatty Acid Protects Against Arsenic Trioxide-Induced Cardiotoxicity In Vitro and In Vivo	2017
1330	H. J. Kim	Efficacy of transarterial embolization with arsenic trioxide oil emulsion in a rabbit VX2 liver tumor model	2009

1331	S. Yan	Arsenic trioxide attenuated the rejection of major histocompatibility complex fully-mismatched cardiac allografts in mice	2009
1332	J. L. Zhao	Effect of early and non-early controlled-release of arsenic-trioxide eluting stents on restenosis inhibition in a canine model	2007
1333	M. Xu	Experimental study on combination of Ad-p53 with CDDP or As(2)O(3) in human lung adenocarcinoma cell line GLC-82	2000
1334	J. Liu	Transplacental exposure to inorganic arsenic at a hepatocarcinogenic dose induces fetal gene expression changes in mice indicative of aberrant estrogen signaling and disrupted steroid metabolism	2007
1335	E. F. Winterbottom	Transcriptome-wide analysis of changes in the fetal placenta associated with prenatal arsenic exposure in the New Hampshire Birth Cohort Study	2019
1336	C. Wang	Grape Seed Procyanidin Extract Reduces Arsenic-Induced Renal Inflammatory Injury in Male Mice	2017
1337	L. Liu	miR-153 sensitized the K562 cells to As2O3-induced apoptosis	2012
1338	M. Kadiiska	Effect of acute intoxication with some heavy metals on drug metabolism	1980
1339	A. Pecker	Deadly arsenical encephalopathy, late trial of B.A.L treatment	1947
1340	S. W. Li	Assessment of arsenic trioxide in the heart of Gallus gallus: alterations of oxidative damage parameters, inflammatory cytokines, and cardiac enzymes	2017
1341	G. Yu	Arsenic trioxide reduces chemo-resistance to 5-fluorouracil and cisplatin in HBx-HepG2 cells via complex mechanisms	2015
1342	N. Sadaf	Arsenic trioxide induces apoptosis and inhibits the growth of human liver cancer cells	2018
1343	H. Y. Hua	Arsenic trioxide and triptolide synergistically induce apoptosis in the SKM-1 human myelodysplastic syndrome cell line	2016
1344	X. Wen	Arsenic trioxide induces cervical cancer apoptosis, but specifically targets human papillomavirus-infected cell populations	2012
1345	X. Han	Protective Effects of 6-Gingerol on Cardiotoxicity Induced by Arsenic Trioxide Through AMPK/SIRT1/PGC-1α Signaling Pathway	2022
1346	L. M. Jalali	Amniotic fluid minerals, trace elements, and prenatal supplement use in humans emerge as determinants of fetal growth	2018
1347	Y. Ishii	The relation of As2O3-induced analgesia and monoamine levels in the brain of mice	1986
1348	B. Cristau	Routes and kinetics of arsenic elimination in rats after administration of organic-arsenic compounds. I. Method of determination of arsenic in biological media	1972

1349	E. S. Orechkin	Further observations on simultaneous therapy of syphilis with penicillin, arsenic, and bismuth	1957
1350	P. Popchristo v	The correlations between penicillin, arsenobenzols and bismuth in vitro and in vivo	1955
1351	P. Gay	Remote results of arseno-bismuth therapy of syphilis; data of a survey	1953
1352	J. Katz	Arsenolysis and phosphorolysis of the amylose and amylopectin fractions of starch	1948
1353	S. C. Srivastava	OX-217 (B.A.L.) as an antidote in complication of arsenic therapy	1947
1354	Coumel	Paraplegia in a specific (arsenical myelitis)	1947
1355	T. Fojo	Arsenic trioxide (As(2)O(3)): still a mystery	2002
1356	J. Z. Szpakows ki	Evaluation of the effectiveness of catheterization of the thoracic duct and peritoneal dialysis in elimination of arsenic compounds absorbed by intestines in experimental studies	1984
1357	J. Sanchez	Evaluation of the newer amebacides	1953
1358	J. Krischek	A contribution to the question of Salvarsan damage to the nervous system	1948
1359	J. J. Gordon	Effects of organic arsenic compounds on tissue enzymes and proteins and on tissue metabolism	1947
1360	C. Karachalio u	Arsenic exposure promotes the emergence of cardiovascular diseases	2021
1361	J. W. Robertson	Squamous cell carcinoma following prolonged use of arsenic; case report	1948
1362	K. Sung	Perinatal Exposure to Arsenic in Drinking Water Alters Glutamatergic Neurotransmission in the Striatum of C57BL/6 Mice	2019
1363	Z. Estrov	Phenylarsine oxide blocks interleukin-1beta-induced activation of the nuclear transcription factor NF-kappaB, inhibits proliferation, and induces apoptosis of acute myelogenous leukemia cells	1999
1364	A. J. Signes-Pastor	Urinary Arsenic Speciation in Children and Pregnant Women from Spain	2017
1365	D. Yu	The effect of arsenic trioxide on the expression of Hsc and HNF4 in nuclear matrix proteins in HepG2 cells	2001
1366	A. M. Mitchell	Arsenic trioxide stabilizes accumulations of adeno-associated virus virions at the perinuclear region, increasing transduction in vitro and in vivo	2013

1367	G. He	YY1 is a novel potential therapeutic target for the treatment of HPV infection-induced cervical cancer by arsenic trioxide	2011
1368	D. W. Kim	Enhancement of Arsenic Trioxide (As(2)O(3))- Mediated Apoptosis Using Berberine in Human Neuroblastoma SH-SY5Y Cells	2007
1369	J. K. Altman	Regulatory effects of mammalian target of rapamycin-mediated signals in the generation of arsenic trioxide responses	2008
1370	H. Y. Tong	Study on the mechanisms of telomerase regulations during apoptosis of the human MDS-RAEB cell line MUTZ-1 cells induced by arsenic trioxide	2005
1371	H. Li	Effects of arsenic trioxide on the methylation of TMS1 gene in K562 cells	2014
1372	K. Davison	JNK activation is a mediator of arsenic trioxide-induced apoptosis in acute promyelocytic leukemia cells	2004
1373	J. E. Rager	Prenatal arsenic exposure and the epigenome: altered microRNAs associated with innate and adaptive immune signaling in newborn cord blood	2014
1374	J. Zhou	A case of M2a with complex chromosome aberrations obtained complete remission through modified As2O3 medication	2004
1375	L. Martínez	Impact of early developmental arsenic exposure on promotor CpG-island methylation of genes involved in neuronal plasticity	2011
1376	S. Tabacova	Placental arsenic and cadmium in relation to lipid peroxides and glutathione levels in maternal-infant pairs from a copper smelter area	1994
1377	H. Tamura	The mechanisms of antagonistic action of prostaglandin E1 against As2O3-induced hypothermia	1984
1378	H. Skröder Löveborn	Arsenic Metabolism in Children Differs From That in Adults	2016
1379	J. J. Lijopavia	Atrophy of the optic nerve: prognosis and treatment	1964
1380	F. Jung	The pathology of the red blood cells; About the hemolytic effects of hydrogen arsenic	1947
1381	E. J. Martinez-Finley	Reduced expression of MAPK/ERK genes in perinatal arsenic-exposed offspring induced by glucocorticoid receptor deficits	2011
1382	C. McDonald	Prevalence of arsenic-related skin lesions in 53 widely-scattered villages of Bangladesh: an ecological survey	2006
1383	A. M. Emran	Synthesis and identification of [76As]arsenic trichloride	1986
1384	D. Sinha	Modulation of arsenic induced cytotoxicity by tea	2003

1385	C. Cervantes	Bacterial resistance to arsenic compounds	1995
1386	P. F. Minaev	Modified radio-sensitivity of the nerve tissue under the influence of arsenic compounds	1964
1387	K. Hueni	On the effect of arsenic compounds as a ration supplement on the arsenic content of meat and liver	1963
1388	N. A. Cellerino	Several considerations on the specific & paraspecific treatment of intestinal amebiasis	1959
1389	K. J. Chang	Arsenic trioxide inhibits the growth of cancer stem cells derived from small cell lung cancer by downregulating stem cell-maintenance factors and inducing apoptosis via the Hedgehog signaling blockade	2020
1390	J. Y. Bae	Low-dose 1,25-dihydroxyvitamin D(3) combined with arsenic trioxide synergistically inhibits proliferation of acute myeloid leukemia cells by promoting apoptosis	2013
1391	Y. Z. Shao	The apoptosis in arsenic-induced oxidative stress is associated with autophagy in the testis tissues of chicken	2018
1392	A. Habib	Arsenic trioxide inhibits ATRA-induced prostaglandin E2 and cyclooxygenase-1 in NB4 cells, a model of acute promyelocytic leukemia	2008
1393	F. X. Reichl	Effects on mitochondrial metabolism in livers of guinea pigs after a single or repeated injection of As2O3	1989
1394	Y. Z. Zhao	Preliminary observation of the combination of arsenic trioxide and all-trans retinoic acid for the treatment of acute promyelocytic leukemia	2003
1395	J. A. Offergelt	Relation between airborne arsenic trioxide and urinary excretion of inorganic arsenic and its methylated metabolites	1992
1396	P. Leffler	Lung retention of antimony and arsenic in hamsters after the intratracheal instillation of industrial dust	1984
1397	I. K. Takeuchi	Embryotoxicity of arsenic acid: light and electron microscopy of its effect on neurulation-stage rat embryo	1979
1398	A. Rahman	A cohort study of the association between prenatal arsenic exposure and age at menarche in a rural area, Bangladesh	2021
1399	L. Betti	A review of three simple plant models and corresponding statistical tools for basic research in homeopathy	2010
1400	M. H. Mao	Additive antitumor effect of arsenic trioxide combined with intravesical bacillus Calmette-Guerin immunotherapy against bladder cancer through blockade of the IER3/Nrf2 pathway	2018
1401	J. Lu	AS3MT Polymorphisms, Arsenic Metabolism, and the Hematological and Biochemical Values in APL Patients Treated with Arsenic Trioxide	2018
1402	M. Y. Feng	Metastasis-induction and apoptosis-protection by TWIST in gastric cancer cells	2009

1403	Assouly	Arsenic intoxication during acid descaling	1949
1404	E. Hadida	Erythroderma during a novarsno-bismuthic treatment quickly cured by penicillin	1948
1405	C. Funk	THE EFFECT OF ARSENIC COMPOUNDS ON THE ROUS CHICKEN SARCOMA	1915
1406	Z. Wang	Arsenic and benzo[a]pyrene co-exposure acts synergistically in inducing cancer stem cell-like property and tumorigenesis by epigenetically down-regulating SOCS3 expression	2020
1407	A. Datta	ac conduction in sol-gel-derived glasses in the SiO2-As2O3 system	1992
1408	G. Gay	Observations concerning cases of poisoning by arsenicals; hypothesis of a causal relationship between accidents of intoxication and cases of lymphocytic leukosis observed later	1948
1409	C. Flandin	Late arsenical erythroderma in a patient treated with novar, penicillin, bismuth	1947
1410	J. P. Buchet	Comparison of several methods for the determination of arsenic compounds in water and in urine. Their application for the study of arsenic metabolism and for the monitoring of workers exposed to arsenic	1980
1411	E. B. Kearney	The inactivation of L-amino acid oxidase by inorganic phosphate and arsenate	1949
1412	Z. Liu	AID expression is correlated with Bcr-Abl expression in CML-LBC and can be down-regulated by As2O3 and/or imatinib	2011
1413	A. Arita	The effect of exposure to carcinogenic metals on histone tail modifications and gene expression in human subjects	2012
1414	E. Guillamet	In vitro DNA damage by arsenic compounds in a human lymphoblastoid cell line (TK6) assessed by the alkaline Comet assay	2004
1415	J. Gate	A particular form of professional arsenicism, the arsenicism of winegrowers	1949
1416	S. S. Chen	Effect of Decitabine in Combination with Arsenic Trioxide on Prolife-ration and Apoptosis of Human Acute Myeloid Leukemia MV4-11 Cells	2016
1417	W. Liu	Arsenic trioxide-induced growth arrest of breast cancer MCF-7 cells involving FOXO3a and I κB kinase β expression and localization	2012
1418	W. W. Guo	Expression of homeobox A9 in myeloid leukemia cell line HL-60 and effect of drugs on its expression	2012
1419	K. F. Rodriguez	Effects of in Utero Exposure to Arsenic during the Second Half of Gestation on Reproductive End Points and Metabolic Parameters in Female CD-1 Mice	2016
1420	X. Zhou	Arotinoid trometamol inhibits arsenic trioxide-stimulated keratinocyte proliferation via the Wnt, Shh, and bone morphogenetic protein signaling pathways	2019

1421	J. Qin	JAB1 expression is associated with inverse expression of p27(kip1) in hepatocellular carcinoma	2010
1422	D. D. Jhala	Mitigating effects of some antidotes on fluoride and arsenic induced free radical toxicity in mice ovary	2008
1423	X. P. Xu	Arsenic trioxide induced leukemic cell apoptosis relative to NF-kappaB activation	2005
1424	Z. Lai	Effects of As(2)O(3) on BCR-ABL protein level and signal transduction in CML cells	2002
1425	Y. F. Cheng	Can As2O3 improve the prognosis of childhood acute promyelocytic leukemia?--A single center experience	2008
1426	L. el Bahri	Arsenic poisoning in livestock	1991
1427	X. W. Chen	Mechanism of apoptosis induced by bortezomib and As2O3 in APL cell line NB4	2011
1428	S. P. Tu	Induction of apoptosis by arsenic trioxide and hydroxy camptothecin in gastriccancer cells in vitro	2000
1429	J. A. Weatherall	The effect of dithiols on survival time in rats and mice poisoned with organic arsenicals	1949
1430	C. Murai	Experimental studies on porphyrin metabolism in various heavy metal poisoning. (II). Effect of lead, mercury and arsenic compounds on delta-aminolevulinic dehydratase activity (author's transl)	1975
1431	K. H. Butzengeiger	About chronic Aasenic poisoning; ECG changes and other manifestations of the heart and vasculature; mucosal symptoms and pathogenesis	1949
1432	W. Y. Au	Frequent varicella zoster reactivation associated with therapeutic use of arsenic trioxide: portents of an old scourge	2005
1433	S. F. Farzan	Maternal and infant inflammatory markers in relation to prenatal arsenic exposure in a U.S. pregnancy cohort	2017
1434	J. Karlsson	Multidrug-resistant neuroblastoma cells are responsive to arsenic trioxide at both normoxia and hypoxia	2005
1435	Y. Shen	Studies on the clinical efficacy and pharmacokinetics of low-dose arsenic trioxide in the treatment of relapsed acute promyelocytic leukemia: a comparison with conventional dosage	2001
1436	A. Lewin	Univariate predictors of maternal concentrations of environmental chemicals: The MIREC study	2017
1437	Z. H. Wang	Alteration of nuclear matrix protein composition of neuroblastoma cells after arsenic trioxide treatment	2001
1438	J. E. Laine	Maternal one carbon metabolism and arsenic methylation in a pregnancy cohort in Mexico	2018

1439	M. P. Waalkes	Lung tumors in mice induced by "whole-life" inorganic arsenic exposure at human-relevant doses	2014
1440	P. Liu	Anticancer activity in human multiple myeloma U266 cells: synergy between cryptotanshinone and arsenic trioxide	2013
1441	V. V. Mathews	Mitigation of hepatotoxic effects of arsenic trioxide through omega-3 fatty acid in rats	2014
1442	D. B. Xue	Regulating effects of arsenic trioxide on cell death pathways and inflammatory reactions of pancreatic acinar cells in rats	2007
1443	S. Yuksel	Arsenic trioxide and methylprednisolone use different signal transduction pathways in leukemic differentiation	2002
1444	B. Wlodarczyk	Arsenic-induced congenital malformations in genetically susceptible folate binding protein-2 knockout mice	2001
1445	J. Brocato	10th NTES Conference: Nickel and Arsenic Compounds Alter the Epigenome of Peripheral Blood Mononuclear Cells	2015
1446	F. I. Abdullaev	Cytotoxic effect of three arsenic compounds in HeLa human tumor and bacterial cells	2001
1447	X. Li	Changes of cdk5, p35 and p53 gene expression levels in arsenic-induced neural cell apoptosis	2012
1448	J. L. Zhao	Effects of control-releasing arsenic trioxide-eluting stent on intimal smooth muscle cells and type III collagen in canine coronary artery post-stent model	2010
1449	W. Chen	Effects of As2O3 on apoptosis and Bcl-2/Bax expression of rat spermatogenic cells	2008
1450	Y. F. Liu	Clinical observation of the short-term efficacy of the treatment with combination of all-trans retinoic acid (ATRA) and arsenic trioxide (As2O3) in newly diagnosed acute promyelocytic leukemia (APL)	2003
1451	Y. Wang	Effects of arsenic exposure on D-serine metabolism in the hippocampus of offspring mice at different developmental stages	2020
1452	K. Engström	Polymorphisms in arsenic(+III oxidation state) methyltransferase (AS3MT) predict gene expression of AS3MT as well as arsenic metabolism	2011
1453	Not found	Disease & its treatment; a new medication against all intestinal protozoas: aceto-xifenil	1956
1454	G. Marras	Determination of 2,3-dimercaptopropanol by the colorimetric method in mixture with arsenic trioxide or other arsenic compounds in various conditions of temperature and of pH and possible interpretations of antidotism	1953
1455	F. Meidinger	Sodium thiomalate, a sulfhydrile compound as a protective substance in mercurial, arsenical, lead poisoning and by halogenated hydrocarbons	1949
1456	A. Pell	LC-ICP-MS analysis of arsenic compounds in dominant seaweeds from the Thermaikos Gulf (Northern Aegean Sea, Greece)	2013

1457	Y. M. Huang	Capillary electrophoresis of arsenic compounds with indirect fluorescence detection	1998
1458	J. M. Fuenzalida	Treatment of intestinal amebiasis with chloroquine with bismuth glycolylarsanilate and with fumagillin	1955
1459	F. Hanse	Arsenic contents of human organs after fatal poisoning with arsenic trioxide and other arsenical compounds, with some remarks on the manifestations of arsenic poisoning	1949
1460	G. Gilmartin	A comparison of the determination and speciation of inorganic arsenic using general HPLC methodology with UV, MS and MS/MS detection	2018
1461	I. Holmqvist	Occupational arsenical dermatitis; a study among employees at a copper ore smelting work including investigations of skin reactions to contact with arsenic compounds	1951
1462	A. V. Ferrari	BAL, a new remedy for the treatment of poisoning from arsenic and other heavy metals	1947
1463	G. Izar	Acute hepatosis from arsenobenzols and bismuth in excessive doses; treatment, healing	1947
1464	B. L. T. Ramaekers	Arsenic Trioxide for Treating Acute Promyelocytic Leukaemia: An Evidence Review Group Perspective of a NICE Single Technology Appraisal	2019
1465	Y. Sánchez	Curcumin stimulates reactive oxygen species production and potentiates apoptosis induction by the antitumor drugs arsenic trioxide and lonidamine in human myeloid leukemia cell lines	2010
1466	Z. Niu	Mitophagy inhibits proliferation by decreasing cyclooxygenase-2 (COX-2) in arsenic trioxide-treated HepG2 cells	2016
1467	Y. Wei	Maternal/fetal metabolomes appear to mediate the impact of arsenic exposure on birth weight: A pilot study	2017
1468	M. Cosenza	Biological effects of Atra and Arsenic Trioxide on short term cultures of non-M3 leukemic blasts	2005
1469	C. M. Chen	Inorganic Arsenic Exposure Decreases Muscle Mass and Enhances Denervation-Induced Muscle Atrophy in Mice	2020
1470	J. Chayapong	Arsenic trioxide induces ROS activity and DNA damage, leading to G0/G1 extension in skin fibroblasts through the ATM-ATR-associated Chk pathway	2017
1471	J. Y. Wang	Arsenic trioxide enhances TRAIL inducing human lung cancer cell line A549 cells apoptosis by down-regulate the expression of NF-kappaB	2012
1472	H. Wei	Arsenic trioxide inhibits p-glycoprotein expression in multidrug-resistant human leukemia cells that overexpress the MDR1 gene	2003
1473	Y. Zheng	Essential role of the voltage-dependent anion channel (VDAC) in mitochondrial permeability transition pore opening and cytochrome c release induced by arsenic trioxide	2004
1474	X. Zhang	Arsenic trioxide, retinoic acid and Ara-c regulated the expression of annexin II on the surface of APL cells, a novel co-receptor for plasminogen/tissue plasminogen activator	2002

1475	N. M. Coelho	Determination of arsenic compounds in beverages by high-performance liquid chromatography-inductively coupled plasma mass spectrometry	2005
1476	L. Wang	Chemotherapeutics-induced osteonecrosis of the jaw in a patient with acute promyelocytic leukemia: A rare case report	2022
1477	L. Wang	Arsenic trioxide and sorafenib combination therapy for human hepatocellular carcinoma functions via up-regulation of TNF-related apoptosis-inducing ligand	2018
1478	H. Eyvani	Arsenic trioxide induces cell cycle arrest and alters DNA methylation patterns of cell cycle regulatory genes in colorectal cancer cells	2016
1479	T. Kanzawa	Arsenic trioxide induces autophagic cell death in malignant glioma cells by upregulation of mitochondrial cell death protein BNIP3	2005
1480	K. Norpoth	Influence of vinyl chloride monomer (VCM) and As2O3 on rat liver cell proliferation after partial hepatectomy	1980
1481	Z. Y. He	A prospective trial to evaluate the clinical efficacy and safety of neoadjuvant chemotherapy with arsenic trioxide and carboplatin in locally advanced cervical cancer: a study protocol for randomized controlled clinical	2022
1482	K. L. Chen	Oral treatment of mule ducks with arsenicals for inducing fatty liver	2001
1483	E. Tavernier	Early relapse after non myeloablative allogeneic stem cell transplantation in a patient with acute promyelocytic leukemia in complete molecular remission	2003
1484	J. Gay Prieto	Results of the treatment of early syphilis by maximal arsenic-bismuth therapy; inquiry conducted in the official antivenereal services of Spain	1953
1485	J. L. Carrera	Results obtained in the treatment of recent syphilis employing so-called long combined methods. II. Arsenosan-bismuth	1955
1486	B. J. Wlodarczyk	Arsenic-induced neural tube defects in mice: alterations in cell cycle gene expression	1996
1487	Y. Wang	Arsenic trioxide increases expression of secreted frizzled-related protein 1 gene and inhibits the WNT/β-catenin signaling pathway in Jurkat cells	2017
1488	X. M. Hu	Arsenic trioxide induces apoptosis in cells of MOLT-4 and its daunorubicin-resistant cell line via depletion of intracellular glutathione, disruption of mitochondrial membrane potential and activation of caspase-3	2003
1489	R. Wang	Elevated non-essential metals and the disordered metabolism of essential metals are associated to abnormal pregnancy with spontaneous abortion	2020
1490	J. Liu	Transplacental arsenic exposure produced 5-methylcytosine methylation changes and aberrant microRNA expressions in livers of male fetal mice	2020
1491	G. B. Gerber	Transfer of antimony and arsenic to the developing organism	1982
1492	C. Y. Chiu	Exposure of low-concentration arsenic induces myotube atrophy by inhibiting an Akt signaling pathway	2020

1493	Y. Yu	Diamide and cyclosporin A enhanced arsenic trioxide-induced apoptosis in NB4 cells	2002
1494	X. Song	Effects of surface modification of As(2)O(3)-loaded PLGA nanoparticles on its anti-liver cancer ability: An in vitro and in vivo study	2018
1495	J. Karlsson	Arsenic trioxide-induced neuroblastoma cell death is accompanied by proteolytic activation of nuclear Bax	2007
1496	J. Zhu	Tissue factors on acute promyelocytic leukemia and endothelial cells are differently regulated by retinoic acid, arsenic trioxide and chemotherapeutic agents	1999
1497	W. Y. Au	Arsenic trioxide in comparison with chemotherapy and bone marrow transplantation for the treatment of relapsed acute promyelocytic leukaemia	2003
1498	H. Liu	The Relationship Between Preeclampsia and Arsenic Concentration in the Peripheral Blood	2022
1499	V. P. Bermudez Abreu	EI BAL in the treatment of poisoning by arsenical and auric preparations	1949
1500	C. M. De Godoy	Differential diagnosis between the eclamptic seizures and convulsions produced by arsenical encephalopathy in pregnant women	1949
1501	V. Lallemand -	Curing APL through PML/RARA degradation by As2O3	2012
1502	W. Ma	MARVELD1 attenuates arsenic trioxide-induced apoptosis in liver cancer cells by inhibiting reactive oxygen species production	2019
1503	L. Yang	As2O3 induces demethylation and up-regulates transcription of SHP-1 gene in human lymphoma cell line T2 cells	2009
1504	J. Hu	Long-term survey of outcome in acute promyelocytic leukemia	2000
1505	H. Li	The action of different kinds of ions on free radical reaction induced by Vc in rat liver homogenate	2003
1506	H. Lorofi	Trial of intravenous novocaine treatment for visual disturbances caused by trypanocidal arsenicals	1947
1507	M. Block	Biologic studies with arsenic; the effect of arsenic 76 upon the clinical course of patients with tumors of the hematopoietic tissues	1949
1508	C. Lin	Arsenic trioxide induces human tumor cell apoptosis and G2 + M arrest whereas causes HPV16 DNA immortalized cervical epithelial cells G1 block	2000
1509	B. T. Beaulieu	Arsenate adsorption structures on aluminum oxide and phyllosilicate mineral surfaces in smelter-impacted soils	2005
1510	M. Filippova	Inorganic and dimethylated arsenic species induce cellular p53	2003

1511	D. Guo	Effect of colloidal gold nanoparticles on cell interface and their enhanced intracellular uptake of arsenic trioxide in leukemia cancer cells	2009
1512	Y. Jing	The PML-RARalpha fusion protein and targeted therapy for acute promyelocytic leukemia	2004
1513	F. X. Reichl	Effect of arsenic on carbohydrate metabolism after single or repeated injection in guinea pigs	1988
1514	Y. L. Li	A comprehensive analysis of Wnt/β-catenin signaling pathway-related genes and crosstalk pathways in the treatment of As(2)O(3) in renal cancer	2018
1515	L. Betti	Number of succussion strokes affects effectiveness of ultra-high-diluted arsenic on in vitro wheat germination and polycrystalline structures obtained by droplet evaporation method	2017
1516	M. Xing	Inflammatory Factor Alterations in the Gastrointestinal Tract of Cocks Overexposed to Arsenic Trioxide	2015
1517	H. Chen	Folate-mediated intracellular drug delivery increases the anticancer efficacy of nanoparticulate formulation of arsenic trioxide	2009
1518	K. Ohnishi	Arsenic trioxide therapy for relapsed or refractory Japanese patients with acute promyelocytic leukemia: need for careful electrocardiogram monitoring	2002
1519	P. Zhang	Seven years' summary report on the treatment of acute promyelocytic leukemia with arsenic trioxide--an analysis of 242 cases	2000
1520	J. D. Brender	Maternal exposure to arsenic, cadmium, lead, and mercury and neural tube defects in offspring	2006
1521	L. Ghiringhelli	[Dermatosis caused by hypersensitivity to arsenic compounds: case report]	1956
1522	K. Nakamura	Detoxification system for inorganic arsenic: transformation of As2O3 into TMAO by vitamin B12 derivatives and conversion of TMAO into arsenobetaine	2008
1523	K. Poma	Lack of synergistic effect between arsenic, mercury and ethyl methane sulfonate on the frequency of chromosomal aberrations in mice	1984
1524	Y. F. Xiao	Inhibitory effect of arsenic trioxide on angiogenesis and expression of vascular endothelial growth factor in gastric cancer	2006
1525	C. Perkins	Arsenic induces apoptosis of multidrug-resistant human myeloid leukemia cells that express Bcr-Abl or overexpress MDR, MRP, Bcl-2, or Bcl-x(L)	2000
1526	D. J. Goussetis	Autophagic degradation of the BCR-ABL oncoprotein and generation of antileukemic responses by arsenic trioxide	2012
1527	L. Che-Pin	Retinoic acid syndrome induced by arsenic trioxide in treating recurrent all-trans retinoic acid resistant acute promyelocytic leukemia	2000
1528	N. Ishinishi	Preliminary experimental study on carcinogenicity of arsenic trioxide in rat lung	1977

1529	Q. Zhong	Association of maternal arsenic exposure with birth size: A systematic review and meta-analysis	2019
1530	W. Zhao	Effect of size and processing method on the cytotoxicity of realgar nanoparticles in cancer cell lines	2011
1531	J. Zhou	The effect of arsenic trioxide on QT interval prolongation during APL therapy	2003
1532	I. Mancini	Bioactive poly(arsenic) compounds	2013
1533	R. Pedroza	Plasmid-encoded resistance to arsenic compounds in Gram-negative bacteria isolated from a hospital environment in Venezuela	1997
1534	G. Lunde	Occurrence and transformation of arsenic in the marine environment	1977
1535	C. H. Huang	Complete atrioventricular block after arsenic trioxide treatment in an acute promyelocytic leukemic patient	1999
1536	A. Kuruvilla	Arsenic poisoning in childhood. An unusual case report with special notes on therapy with penicillamine	1975
1537	T. Tsuchiya	Inorganic arsenic compounds and methylated metabolites induce morphological transformation in two-stage BALB/c 3T3 cell assay and inhibit metabolic cooperation in V79 cell assay	2005
1538	W. Li	Evaluation of three antidotes on arsenic toxicity in the common earthworm (Lumbricus terrestris)	1994
1539	N. Varma	Structural perturbation by arsenic triggers the aggregation of hen egg white lysozyme by promoting oligomers formation	2018
1540	H. M. Wortelboer	Glutathione-dependent interaction of heavy metal compounds with multidrug resistance proteins MRP1 and MRP2	2008
1541	S. Y. Gao	Arsenic trioxide prevents rat pulmonary fibrosis via miR-98 overexpression	2014
1542	X. Chen	L-type calcium current (ICa,L) and inward rectifier potassium current (IK1) are involved in QT prolongation induced by arsenic trioxide in rat	2010
1543	P. Zhang	On arsenic trioxide in the clinical treatment of acute promyelocytic leukemia	2017
1544	J. L. Carrera	Long-term results of intensive treatment of recent syphilis with penicillin combined with arsenoxide and bismuth, administered simultaneously	1955
1545	Y. Jin	Arsenic speciation transported through the placenta from mother mice to their newborn pups	2006
1546	T. Tsuda	A case of lung cancer associated with chronic arsenic poisoning caused by neighborhood exposure of As2O3 from Toroku mine	1987

1547	U. C. Nygaard	Cord blood T cell subpopulations and associations with maternal cadmium and arsenic exposures	2017
1548	P. Chen	The effect to IL-3Ralpha, downstream PI3k/Akt signaling of all-trans retinoic acid and arsenic trioxide in NB4 cells	2014
1549	Y. Wang	Arsenic trioxide induces the apoptosis of human breast cancer MCF-7 cells through activation of caspase-3 and inhibition of HERG channels	2011
1550	W. Guo-Bao	Arsenic Trioxide overcomes cell adhesion-mediated drug resistance through down-regulating the expression of beta(1)-integrin in K562 chronic myelogenous leukemia cell line	2010
1551	H. Chen	Lipid encapsulation of arsenic trioxide attenuates cytotoxicity and allows for controlled anticancer drug release	2006
1552	F. A. Erturk	Effects of As2O3 on DNA methylation, genomic instability, and LTR retrotransposon polymorphism in Zea mays	2015
1553	P. M. Jia	Effects of PML-RARalpha on cAMP-induced AML cell differentiation	2008
1554	X. H. Zhang	Effect of arsenic trioxide on the expression of apoptosis-related genes in NB4 cells	2007
1555	S. Roy	Arsenic-induced changes in optic tectal histoarchitecture and acetylcholinesterase-acetylcholine profile in Channa punctatus: amelioration by selenium	2006
1556	H. R. Kim	Combination treatment with arsenic trioxide and sulindac augments their apoptotic potential in lung cancer cells through activation of caspase cascade and mitochondrial dysfunction	2006
1557	H. Y. Wang	Gene expression profile changes in NB4 cells induced by arsenic trioxide	2003
1558	Q. Q. Wang	Effect of arsenic compounds on the in vitro differentiation of mouse embryonic stem cells into cardiomyocytes	2015
1559	T. Ochi	Glutathione plays different roles in the induction of the cytotoxic effects of inorganic and organic arsenic compounds in cultured BALB/c 3T3 cells	1994
1560	F. Hayakawa	Phosphorylation of PML by mitogen-activated protein kinases plays a key role in arsenic trioxide-mediated apoptosis	2004
1561	Y. Zou	Influence of As2O3-lipiodol emulsion via transarterial embolization on a VX2 liver tumor model in rabbits	2015
1562	P. Yoon	Activation of mammalian target of rapamycin and the p70 S6 kinase by arsenic trioxide in BCR-ABL-expressing cells	2006
1563	X. Cai	Arsenic trioxide-induced mitotic arrest and apoptosis in acute promyelocytic leukemia cells	2003
1564	S. Li	Relationship between maternal heavy metal exposure and congenital heart defects: a systematic review and meta-analysis	2022

1565	T. D. Ninh	Unusual arsenic speciation in sea anemones	2008
1566	S. Chang	Nutritional composition and heavy metal content of the human placenta	2017
1567	M. E. Muse	Relation between in utero arsenic exposure and growth during the first year of life in a New Hampshire pregnancy cohort	2020
1568	M. L. Rahman	Regulation of birthweight by placenta-derived miRNAs: evidence from an arsenic-exposed birth cohort in Bangladesh	2018
1569	B. Claus Henn	Prenatal Arsenic Exposure and Birth Outcomes among a Population Residing near a Mining-Related Superfund Site	2016
1570	P. Chen	Combination of Homoharringtonine with Arsenic Trioxide Induces Apoptosis of Human Acute Myeloid Leukemia Cell Line U937	2016
1571	Y. Lin	Arsenic trioxide is a novel agent for combination therapy to prolong heart allograft survival in allo-primed T cells transferred mice	2011
1572	H. Y. Fu	Arsenic trioxide inhibits DNA methyltransferase and restores expression of methylation-silenced CDKN2B/CDKN2A genes in human hematologic malignant cells	2010
1573	L. Zhang	[Retrospective analysis of 76 children with acute promyelocytic leukemia]	2009
1574	W. Zhao	Hemostatic abnormalities associated with acute promyelocytic leukemia and corrective effects of all-trans-retinoic acid or arsenic trioxide treatment	2000
1575	S. Z. Wu	Arsenic compounds induce apoptosis by activating the MAPK and caspase pathways in FaDu oral squamous carcinoma cells	2022
1576	Y. Zhang	Endothelial to mesenchymal transition contributes to arsenic-trioxide-induced cardiac fibrosis	2016
1577	R. Venè	The cystine/cysteine cycle and GSH are independent and crucial antioxidant systems in malignant melanoma cells and represent druggable targets	2011
1578	P. Banerjee	Comparative efficacy of two microdoses of a potentized homeopathic drug, arsenicum album, to ameliorate toxicity induced by repeated sublethal injections of arsenic trioxide in mice	2008
1579	B. Kong	Arsenic trioxide induces apoptosis in cisplatin-sensitive and -resistant ovarian cancer cell lines	2005
1580	K. Mehta	Resveratrol attenuates arsenic-induced cognitive deficits via modulation of Estrogen-NMDAR-BDNF signalling pathway in female mouse hippocampus	2021
1581	M. M. Ommati	Paternal exposure to arsenic resulted in oxidative stress, autophagy, and mitochondrial impairments in the HPG axis of pubertal male offspring	2019
1582	H. Zhao	Subchronic arsenism-induced oxidative stress and inflammation contribute to apoptosis through mitochondrial and death receptor dependent pathways in chicken immune organs	2017

1583	K. Zhang	Arsenic Trioxide Attenuates NF-κB and Cytokine mRNA Levels in the Livers of Cocks	2016
1584	H. L. Sun	Choline-modulated arsenic trioxide-induced prolongation of cardiac repolarization in Guinea pig	2006
1585	T. Zhang	Arsenic trioxide induces apoptosis of rat hepatocellular carcinoma cells in vivo	2003
1586	Z. Wang	Experimental study on apoptosis of HL-60 cell induced by arsenic trioxide	2000
1587	K. Yamazaki	Arrhythmogenic effects of arsenic trioxide in patients with acute promyelocytic leukemia and an electrophysiological study in isolated guinea pig papillary muscles	2006
1588	S. Gupta	Arsenic trioxide induces apoptosis in peripheral blood T lymphocyte subsets by inducing oxidative stress: a role of Bcl-2	2003
1589	V. Mathews	Arsenic trioxide in the treatment of newly diagnosed acute promyelocytic leukemia: a single center experience	2002
1590	H. Hua	Pharmacokinetics of arsenic trioxide (As_2O_3) in Chinese primary hepatocarcinoma patients	2011
1591	E. H. Kim	Arsenic trioxide sensitizes human glioma cells, but not normal astrocytes, to TRAIL-induced apoptosis via CCAAT/enhancer-binding protein homologous protein-dependent DR5 up-regulation	2008
1592	C. M. Aelion	Associations of estimated residential soil arsenic and lead concentrations and community-level environmental measures with mother-child health conditions in South Carolina	2012
1593	D. Liu	Arsenic Trioxide Reduces Global Histone H4 Acetylation at Lysine 16 through Direct Binding to Histone Acetyltransferase hMOF in Human Cells	2015
1594	J. G. Parsons	Arsenic speciation in biological samples using XAS and mixed oxidation state calibration standards of inorganic arsenic	2009
1595	H. Y. Xu	Effect of arsenic trioxide on human hepatocarcinoma in nude mice	2004
1596	S. Bao	Multiple metal exposure and platelet counts during pregnancy: A repeated measure study	2020
1597	T. D. Wang	Arsenic Trioxide Combining Leflunomide Activates Nrf2-ARE-HO-1 Signaling Pathway and Protects Heart Xenografts	2021
1598	D. D. Wu	Antitumor effect and mechanisms of arsenic trioxide on subcutaneously implanted human gastric cancer in nude mice	2010
1599	E. Dopp	Forced uptake of trivalent and pentavalent methylated and inorganic arsenic and its cyto-/genotoxicity in fibroblasts and hepatoma cells	2005
1600	S. Mazan	Porous graphitic carbon as stationary phase for LC-ICPMS separation of arsenic compounds in water	2002

1601	K. Nakamuro	Comparative studies of chromosomal aberration induced by trivalent and pentavalent arsenic	1981
1602	W. Y. Au	Oral arsenic trioxide-based maintenance regimens for first complete remission of acute promyelocytic leukemia: a 10-year follow-up study	2011
1603	J. E. Chang	Phase II study of arsenic trioxide and ascorbic acid for relapsed or refractory lymphoid malignancies: a Wisconsin Oncology Network study	2009
1604	X. F. Wu	Protein kinase C inhibitor Gö6976 sensitizes arsenic trioxide-induced cell apoptosis in chronic myeloid leukemic cells	2005
1605	Q. Lou	Low-dose arsenic trioxide enhances membrane-GLUT1 expression and glucose uptake via AKT activation to support L-02 cell aberrant proliferation	2022
1606	M. L. Rahman	Early pregnancy exposure to metal mixture and birth outcomes - A prospective study in Project Viva	2021
1607	W. Yan	Mutant p53 protein is targeted by arsenic for degradation and plays a role in arsenic-mediated growth suppression	2011
1608	A. J. Mürer	Estimation of the method evaluation function for the determination of hydride-generating arsenic compounds in urine by flow-injection atomic-absorption spectrometry	1992
1609	P. A. Guńka	Spatial dispersion of lone electron pairs?--experimental charge density of cubic arsenic(III) oxide	2015
1610	R. Donckaster	Treatment of chronic intestinal amebiasis with tetracycline and chloroquine with bismuth glycolylarsanilate and parasitological control by the combined Telemann and polyvinyl alcohol methods	1957
1611	C. Li	Single Cell RNA-Seq Analysis Identifies Differentially Expressed Genes of Treg Cell in Early Treatment-Naive Rheumatoid Arthritis By Arsenic Trioxide	2021
1612	B. I. Kaplan	Results of therapy of latent and asymptomatic syphilis in a prison population. I. Clinical outcome with reference to cardiovascular and central nervous system syphilis and related to a nonsyphilitic control population	1958
1613	C. Lu	Comparison of speciated arsenic levels in the liver and brain of mice between arsenate and arsenite exposure at the early life	2014
1614	S. Mukhopadhyay	Effect of arsenic on cell growth of the cellular slime mould, Dictyostelium discoideum	2003
1615	S. C. Chaurasia	Room-temperature isopiestic distillation of in situ generated arsenious chloride and its application for the determination of trace level impurities in arsenious oxide	2002
1616	Z. Wang	Taurine protected As(2)O(3)-induced the activation of hepatic stellate cells through inhibiting PPARα-autophagy pathway	2019
1617	X. Zhou	Effects of cysteine on the cytotoxicity of arsenic compounds	2003
1618	M. Grotti	Arsenic species in certified reference material MURST-ISS-A2 (Antarctic krill)	2010

1619	J. Wang	The impact of Tegillarca granosa extract haishengsu on HL-60 cell	2015
1620	X. Wang	Exploring the Mechanisms of Arsenic Trioxide (Pishuang) in Hepatocellular Carcinoma Based on Network Pharmacology	2021
1621	R. D. Hood	Distribution, metabolism, and fetal uptake of pentavalent arsenic in pregnant mice following oral or intraperitoneal administration	1987
1622	C. Gu	Molecular targeting therapy against promyelocytic leukemia protein using arsenic acids in experimental intracranial medulloblastoma	2012
1623	P. R. Subbarayan	Arsenic trioxide/ascorbic acid therapy in patients with refractory metastatic colorectal carcinoma: a clinical experience	2007
1624	M. A. Valdés Salgado	Structural Equation Modelling in the exploration and analysis of intrauterine environmental exposures with infant health effects	2019
1625	E. Govarts	Combined Effects of Prenatal Exposures to Environmental Chemicals on Birth Weight	2016
1626	S. McDermott	Are different soil metals near the homes of pregnant women associated with mild and severe intellectual disability in children?	2014
1627	Y. Jia	Expression of AFP and STAT3 is involved in arsenic trioxide-induced apoptosis and inhibition of proliferation in AFP-producing gastric cancer cells	2013
1628	S. Tao	The significance of combined therapy of arsenic trioxide and all-trans retinoic acid in treating acute promyelocytic leukemia	2009
1629	K. A. Bailey	Prenatal arsenic exposure and shifts in the newborn proteome: interindividual differences in tumor necrosis factor (TNF)-responsive signaling	2014
1630	P. Y. Zhang	Antiapoptotic Effect of the Leukemia Associated Gene MLAA-34 in HeLa Cells	2016
1631	Z. M. Liu	Inhibitory role of TGIF in the As2O3-regulated p21 WAF1/CIP1 expression	2008
1632	L. Betti	Effects of homeopathic arsenic on tobacco plant resistance to tobacco mosaic virus. Theoretical suggestions about system variability, based on a large experimental data set	2003
1633	R. F. de Farias	Effects of Adsorbed Polyaniline on Redox Processes on As(2)O(3) Surfaces	2000
1634	W. Y. Au	Solid tumors subsequent to arsenic trioxide treatment for acute promyelocytic leukemia	2007
1635	J. J. Noh	Anti-Cancer Activity of As(4)O(6) and its Efficacy in a Series of Patient-Derived Xenografts for Human Cervical Cancer	2020
1636	A. H. Smith	Chronic respiratory symptoms in children following in utero and early life exposure to arsenic in drinking water in Bangladesh	2013

1637	L. Zong	FTIR microspectroscopic study of biomacromolecular changes in As(2)O(3) induced MGC803 cells apoptosis	2021
1638	C. R. Kumana	Resurrection of Oral Arsenic Trioxide for Treating Acute Promyelocytic Leukaemia: A Historical Account From Bedside to Bench to Bedside	2020
1639	J. Bai	Taurine protects against As2O3-induced autophagy in pancreas of rat offsprings through Nrf2/Trx pathway	2016
1640	W. Y. Au	Determinants of cerebrospinal fluid arsenic concentration in patients with acute promyelocytic leukemia on oral arsenic trioxide therapy	2008
1641	I. Tarkanyi	Retinoid/arsenic combination therapy of promyelocytic leukemia: induction of telomerase-dependent cell death	2005
1642	H. D. Halicka	Arsenic trioxide arrests cells early in mitosis leading to apoptosis	2002
1643	Y. Lou	Inhibitory effects of recombinant human neurotrophin-4/5 protein on neurotoxicity caused by arsenic trioxide	1999
1644	T. Zhang	A laser scanning confocal microscopy method. Simultaneous detection of intracellular Ca2+ and apoptosis using Fluo-3 and Hoechst 33342	2000
1645	C. Andre	The PML and PML/RARalpha domains: from autoimmunity to molecular oncology and from retinoic acid to arsenic	1996
1646	Z. Slejkovec	Unexpected arsenic compounds in low-rank coals	2005
1647	R. Kubota	Occurrence of several arsenic compounds in the liver of birds, cetaceans, pinnipeds, and sea turtles	2003
1648	Y. Oya-Ohta	Induction of chromosomal aberrations in cultured human fibroblasts by inorganic and organic arsenic compounds and the different roles of glutathione in such induction	1996
1649	E. Zhang	Separation and recovery of arsenic from As, Cu, and Zn rich leaching liquor using a reduction-crystallization approach	2021
1650	S. Xu	Speciation characterization of arsenic-bearing phase in arsenic sulfide sludge and the sequential leaching mechanisms	2022
1651	C. L. Tsai	Protective Effects of Baicalin on Arsenic Trioxide-induced Oxidative Damage and Apoptosis in Human Umbilical Vein Endothelial Cells	2021
1652	A. Gaion	Bioaccumulation and biotransformation of arsenic compounds in Hediste diversicolor (Muller 1776) after exposure to spiked sediments	2014
1653	S. N. Pedersen	Liquid chromatography electrospray mass spectrometry with variable fragmentor voltages gives simultaneous elemental and molecular detection of arsenic compounds	2000
1654	S. Tamaki	Environmental biochemistry of arsenic	1992

1655	Y. Tsutsumi	Lung infiltration of adult T-cell leukemia cells following the administration of arsenic trioxide. Lung infiltration of ATL by AS(2)O(3)	2006
1656	Y. Xia	Study on reproductive and immune toxicity of male rats exposed to As2O3	2009
1657	H. Skröder	Associations between Methylated Metabolites of Arsenic and Selenium in Urine of Pregnant Bangladeshi Women and Interactions between the Main Genes Involved	2018
1658	S. A. Ahmad	Arsenicosis: sex differentials	1999
1659	Y. Hiwatashi	Antiproliferative and anti-invasive effects of inorganic and organic arsenic compounds on human and murine melanoma cells in vitro	2011
1660	M. Beerbom	Synchrotron-induced photoemission of GaAs electrodes after electrochemical treatment in aqueous electrolytes	2002
1661	X. Li	The clinical activity of arsenic trioxide, ascorbic acid, ifosfamide and prednisone combination therapy in patients with relapsed and refractory multiple myeloma	2015
1662	N. J. Seong	Effects of arsenic trioxide on radiofrequency ablation of VX2 liver tumor: intraarterial versus intravenous administration	2012
1663	Y. K. Sun	Effect of arsenic pentaoxide on proliferation and apoptosis of human umbilical vein endothelial cell	2009
1664	Z. Zhao	Effect of arsenic trioxide on inhibition of restenosis after rabbit vascular injury and its mechanism	2002
1665	S. W. Li	Assessment of 28 trace elements and 17 amino acid levels in muscular tissues of broiler chicken (Gallus gallus) suffering from arsenic trioxide	2017
1666	M. M. Bonaventura	Arsenite in drinking water produces glucose intolerance in pregnant rats and their female offspring	2017
1667	N. Y. Chen	Effects of STI571 combined with As_2O_3 on proliferation, apoptosis and caspase 3, Bcl-xL expression of K562 cells	2010
1668	J. Hu	Long-term survival and prognostic study in acute promyelocytic leukemia treated with all-trans-retinoic acid, chemotherapy, and As2O3: an experience of 120 patients at a single institution	1999
1669	Y. L. Zhang	Arsenic trioxide-induced cell apoptosis and cell cycle arrest are potentiated by 1,25-dihydroxyvitamin D3 in human leukemia K562 cells	2021
1670	M. M. Ommati	The mechanisms of arsenic-induced ovotoxicity, ultrastructural alterations, and autophagic related paths: An enduring developmental study in folliculogenesis of mice	2020
1671	H. Zhao	Arsenic trioxide inhibits the growth of adriamycin resistant osteosarcoma cells through inducing apoptosis	2010
1672	J. J. Li	Role of oxidative stress in the apoptosis of hepatocellular carcinoma induced by combination of arsenic trioxide and ascorbic acid	2006

1673	S. Ahmed	Arsenic exposure and cell-mediated immunity in pre-school children in rural Bangladesh	2014
1674	N. Salmeri	Maternal Arsenic Exposure and Gestational Diabetes: A Systematic Review and Meta-Analysis	2020
1675	M. Geron	Racial/ethnic and neighborhood disparities in metals exposure during pregnancy in the Northeastern United States	2022
1676	Suhendrayatna	Studies on the accumulation and transformation of arsenic in freshwater organisms I. Accumulation, transformation and toxicity of arsenic compounds on the Japanese medaka, Oryzias latipes	2002
1677	L. Liu	Anti-angiogenesis effect of arsenic trioxide plus cinobufacin on human hepatocarcinoma transplantation model nude mice	2011
1678	V. Voiculescu	From Normal Skin to Squamous Cell Carcinoma: A Quest for Novel Biomarkers	2016
1679	X. C. Le	Speciation of arsenic compounds by HPLC with hydride generation atomic absorption spectrometry and inductively coupled plasma mass spectrometry detection	1994
1680	S. Lösler	Antimony-trioxide- and arsenic-trioxide-induced apoptosis in myelogenic and lymphatic cell lines, recruitment of caspases, and loss of mitochondrial membrane potential are enhanced by modulators of the cellular glutathione redox system	2009
1681	P. J. Landrigan	Occupational exposure to arsine. An epidemiologic reappraisal of current standards	1982
1682	V. Olsen	Arsenic poisoning	2004
1683	R. J. Griffin	Preferential action of arsenic trioxide in solid-tumor microenvironment enhances radiation therapy	2005
1684	H. Fu	Hypermethylation of CpG island of DLC-1 gene and arsenic trioxide-induced DLC-1 gene demethylation in multiple myeloma	2014
1685	Z. X. Shen	All-trans retinoic acid/As2O3 combination yields a high quality remission and survival in newly diagnosed acute promyelocytic leukemia	2004
1686	M. S. Bloom	Maternal arsenic exposure and birth outcomes: a comprehensive review of the epidemiologic literature focused on drinking water	2014
1687	H. Bhattacharjee	Aquaglyceroporins and metalloid transport: implications in human diseases	2009
1688	C. Soeroes	Thio arsenosugars in freshwater mussels from the Danube in Hungary	2005
1689	M. D. Nemec	Developmental toxicity assessment of arsenic acid in mice and rabbits	1998
1690	D. Yang	Activation of the Nrf2 Signaling Pathway Involving KLF9 Plays a Critical Role in Allicin Resisting Against Arsenic Trioxide-Induced Hepatotoxicity in Rats	2017

1691	D. P. Lu	Current study of APL treatment in China	2002
1692	J. Ashley-Martin	Association between maternal urinary speciated arsenic concentrations and gestational diabetes in a cohort of Canadian women	2018
1693	S. A. Niño	Arsenic Exposure Contributes to the Bioenergetic Damage in an Alzheimer's Disease Model	2019
1694	C. L. Tsai	Protective Effects of Crocetin on Arsenic Trioxide-induced Oxidative Stress in Human Umbilical Vein Endothelial Cells	2021
1695	T. Feng	Decrease in stathmin expression by arsenic trioxide inhibits the proliferation and invasion of osteosarcoma cells via the MAPK signal pathway	2017
1696	E. Fahrenkrug	Electrodeposition of crystalline GaAs on liquid gallium electrodes in aqueous electrolytes	2013
1697	F. L. Qu	Multicenter phase II clinical trial of arsenic trioxide injection in the treatment of primary hepatocarcinoma	2011
1698	W. Guo	Mechanism of tissue factor expression on NB4 cells down-regulated by all-trans retinoic acid and arsenic trioxide	2000
1699	F. X. Reichl	Pyruvate and lactate metabolism in livers of guinea pigs perfused with chelating agents after repeated treatment with As2O3	1991
1700	X. Cui	Metabolism and the paradoxical effects of arsenic: carcinogenesis and anticancer	2008
1701	G. Q. Chen	Hypoxia inducible factor-1alpha and leukemic cell differentiation	2006
1702	S. Tamura	Metabolism of arsenic (Report 22). Effect of arsenic on the spontaneous motor activity, avoidance conditioning, extinction and swimming record in rats (author's transl)	1978
1703	W. Fei	Construction of arsenic-metal complexes loaded nanodrugs for solid tumor therapy: A mini review	2020
1704	H. Chen	JWA as a functional molecule to regulate cancer cells migration via MAPK cascades and F-actin cytoskeleton	2007
1705	I. Sfaxi	Grape seed and skin extract protects against arsenic trioxide induced oxidative stress in rat heart	2016
1706	Y. Jing	Targeted removal of PML-RARalpha protein is required prior to inhibition of histone deacetylase for overcoming all-trans retinoic acid differentiation resistance in acute promyelocytic leukemia	2002
1707	R. Marasca	Missense mutations in the PML/RARalpha ligand binding domain in ATRA-resistant As(2)O(3) sensitive relapsed acute promyelocytic leukemia	1999
1708	X. Zhang	Arsenic exposure via drinking water during pregnancy and lactation induces autism-like behaviors in male offspring mice	2022

1709	M. A. Essers	Targeting leukemic stem cells by breaking their dormancy	2010
1710	B. Vantroyen	Survival after a lethal dose of arsenic trioxide	2004
1711	R. Raqib	Humoral Immunity in Arsenic-Exposed Children in Rural Bangladesh: Total Immunoglobulins and Vaccine-Specific Antibodies	2017
1712	E. G. Rodrigues	Neurodevelopmental outcomes among 2- to 3-year-old children in Bangladesh with elevated blood lead and exposure to arsenic and manganese in drinking water	2016
1713	D. Bironaite	A variety of mild stresses upregulate stanniocalcin-1 (STC-1) and induce mitohormesis in neural crest-derived cells	2013
1714	M. V. Rao	Arsenic induced free radical toxicity in brain of mice	2004
1715	S. Galimberti	Arsenic and all-trans retinoic acid as induction therapy before autograft in a case of relapsed resistant secondary acute promyelocytic leukemia	1999
1716	G. Raber	Identification of arsenolipids with GC/MS	2009
1717	J. L. Domingo	Amelioration by BAL (2,3-dimercapto-1-propanol) and DMPS (sodium 2,3-dimercapto-1-propanesulfonic acid) of arsenite developmental toxicity in mice	1992
1718	A. Ganguli	Heavy Metals in Indigenous Preparations Used for Sex Selection During Pregnancy in India	2019
1719	P. Montazeri	Socioeconomic position and exposure to multiple environmental chemical contaminants in six European mother-child cohorts	2019
1720	Y. Maimaitiyiming	Role of arsenic (+3 oxidation state) methyltransferase in arsenic mediated APL treatment: an in vitro investigation	2018
1721	Y. Jiang	Protective effect of edible marine algae, Laminaria japonica and Porphyra haitanensis, on subchronic toxicity in rats induced by inorganic arsenic	2013
1722	F. Gumilar	Locomotor activity and sensory-motor developmental alterations in rat offspring exposed to arsenic prenatally and via lactation	2015
1723	J. D. Cross	A suicide by ingestion of a mixture of copper, chromium and arsenic compounds	1979
1724	K. A. Francesconi	Uptake of arsenic-betaines by the mussel Mytilus edulis	1999
1725	T. Ochi	Dimethylarsinic acid causes apoptosis in HL-60 cells via interaction with glutathione	1996
1726	Y. Han	Chronic arsenic exposure lowered sperm motility via impairing ultra-microstructure and key proteins expressions of sperm acrosome and flagellum formation during spermiogenesis in male mice	2020

1727	M. M. Ommati	The Footprints of Oxidative Stress and Mitochondrial Impairment in Arsenic Trioxide-Induced Testosterone Release Suppression in Pubertal and Mature F1-Male Balb/c Mice via the Downregulation of 3β-HSD, 17β-HSD, and CYP11a Expression	2020
1728	H. A. Vu	Speciation Analysis of Arsenic Compounds by High-Performance Liquid Chromatography in Combination with Inductively Coupled Plasma Dynamic Reaction Cell Quadrupole Mass Spectrometry: Application for Vietnamese Rice Samples	2019
1729	X. H. Zhang	Effects of arsenic trioxide or retinoic acid on mRNA and protein expression of tissue factor and thrombomodulin and procoagulant activity in NB4 cells	2007
1730	L. Nejdl	Interaction study of arsenic (III and V) ions with metallothionein gene (MT2A) fragment	2015
1731	R. Sierra-Alvarez	Methanogenic inhibition by arsenic compounds	2004
1732	M. Vahter	Gender differences in the disposition and toxicity of metals	2007
1733	B. J. Wlodarczyk	Mthfr gene ablation enhances susceptibility to arsenic prenatal toxicity	2014
1734	M. M. Shi	Gender-dependent expression of ERalpha in arsenic exposed mice offspring's lung tissue	2015
1735	D. Rojas	Prenatal arsenic exposure and the epigenome: identifying sites of 5-methylcytosine alterations that predict functional changes in gene expression in newborn cord blood and subsequent birth outcomes	2015
1736	M. M. Ommati	Arsenic-induced autophagic alterations and mitochondrial impairments in HPG-S axis of mature male mice offspring (F1-generation): A persistent toxicity study	2020
1737	J. Yi	Emodin enhances arsenic trioxide-induced apoptosis via generation of reactive oxygen species and inhibition of survival signaling	2004
1738	A. Santolaria	Acute Promyelocytic Leukemia during Pregnancy: A Systematic Review of the Literature	2020
1739	J. Zhang	Inhibition of angiogenesis by arsenic trioxide via TSP-1-TGF-β1-CTGF-VEGF functional module in rheumatoid arthritis	2017
1740	M. Yu	Resveratrol protects against arsenic trioxide-induced nephrotoxicity by facilitating arsenic metabolism and decreasing oxidative stress	2013
1741	J. M. Heraud	The efficacy of combined therapy of arsenic trioxide and alpha interferon in human T-cell leukemia virus type-1-infected squirrel monkeys (Saimiri sciureus)	2006
1742	Q. Zhu	In vitro study of the effects of arsenic trioxide combined with 8-CPT-cAMP on differentiation induction in retinoic acid resistant acute promyelocytic leukemia cells	2003
1743	A. König	Comparative activity of melarsoprol and arsenic trioxide in chronic B-cell leukemia lines	1997
1744	T. Tisler	Aquatic toxicity of selected chemicals as a basic criterion for environmental classification	2003

1745	J. D. Miller	Geophagic earths consumed by women in western Kenya contain dangerous levels of lead, arsenic, and iron	2018
1746	J. E. Laine	Neonatal Metabolomic Profiles Related to Prenatal Arsenic Exposure	2017
1747	E. C. Nyanza	Maternal exposure to arsenic and mercury in small-scale gold mining areas of Northern Tanzania	2019
1748	S. Selim	Elevated CO(2) differently suppresses the arsenic oxide nanoparticles-induced stress in C3 (Hordeum vulgare) and C4 (Zea maize) plants via altered homeostasis in metabolites specifically proline and anthocyanin metabolism	2021
1749	M. Herlin	Exploring telomere length in mother-newborn pairs in relation to exposure to multiple toxic metals and potential modifying effects by nutritional factors	2019
1750	R. Pei	Long term curative effects of sequential therapy with all-trans retinoic acid, arsenious oxide and chemotherapy on patients with acute promyelocytic leukemia	2012
1751	Y. Yoshino	Speciation of arsenic trioxide metabolites in blood cells and plasma of a patient with acute promyelocytic leukemia	2009
1752	L. I. Privalova	The umbilical blood levels of lead and some other toxic metals as a biomarker of environment-induced exposure	2007
1753	Ş. Özel	Maternal second trimester blood levels of selected heavy metals in pregnancies complicated with neural tube defects	2019
1754	Y. Zhang	Effects of arsenic trioxide combined with platinum drugs in treatment of cervical cancer: A protocol for systematic review and meta-analysis of randomized controlled trials	2020
1755	P. Jain	Long-term control of refractory follicular lymphoma after treatment of secondary acute promyelocytic leukemia with arsenic trioxide (As(2)O(3)) and all-trans retinoic acid (ATRA)	2018
1756	P. L. Toogood	Mitochondrial drugs	2008
1757	A. Ovayolu	Amniotic fluid levels of selected trace elements and heavy metals in pregnancies complicated with neural tube defects	2020
1758	J. Zhou	Phosphatidylserine exposure and procoagulant activity in acute promyelocytic leukemia	2010
1759	Y. Liu	Arsenic trioxide inhibits invasion/migration in SGC-7901 cells by activating the reactive oxygen species-dependent cyclooxygenase-2/matrix metalloproteinase-2 pathway	2011
1760	F. Huaux	Lung toxicity of hard metal particles and production of interleukin-1, tumor necrosis factor-alpha, fibronectin, and cystatin-c by lung phagocytes	1995
1761	S. Gunia	Molecular interaction between arsenic hydrate microcrystals and the cell-surface endopeptidase CD10 (neprilysin) - a possible link to the development of renal and cutaneous malignancies upon occupational exposure to arsenic compounds?	2010
1762	Z. Braszczyńska	Degree of exposure to arsenic compounds of workers in zinc smelteries	1983

1763	S. Becherirat	The antitumor effects of an arsthinol-cyclodextrin complex in a heterotopic mouse model of glioma	2013
1764	S. Lischka	The high diversity of arsenolipids in herring fillet (Clupea harengus)	2013
1765	B. Daus	Analytical investigations of phenyl arsenicals in groundwater	2008
1766	P. P. Binu	Protective Effects of Eugenol against Hepatotoxicity Induced by Arsenic Trioxide: An Antileukemic Drug	2018
1767	Z. Hu	Accumulation and suppressive function of regulatory T cells in malignant ascites: Reducing their suppressive function using arsenic trioxide in vitro	2018
1768	Y. He	Simultaneous analysis 26 mineral element contents from highly consumed cultured chicken overexposed to arsenic trioxide by inductively coupled plasma mass spectrometry	2016
1769	J. Peremartí	Arsenic exposure disrupts the normal function of the FA/BRCA repair pathway	2014
1770	V. Stibilj	The effect of dietary arsenic additions on the distribution of selenium and iodine in eggs and tissues of laying hens	2004
1771	H. Pelicano	Inhibition of mitochondrial respiration: a novel strategy to enhance drug-induced apoptosis in human leukemia cells by a reactive oxygen species-mediated mechanism	2003
1772	J. Jaafar	Online preconcentration of arsenic compounds by dynamic pH junction-capillary electrophoresis	2007
1773	B. J. Lafferty	Methyl arsenic adsorption and desorption behavior on iron oxides	2005
1774	C. B'Hymer	Arsenic and its speciation analysis using high-performance liquid chromatography and inductively coupled plasma mass spectrometry	2004
1775	Z. Mester	Speciation of dimethylarsinic acid and monomethylarsonic acid by solid-phase microextraction-gas chromatography-ion trap mass spectrometry	2000
1776	D. H. Wang	Arsenic trioxide overcomes apoptosis inhibition in K562/ADM cells by regulating vital components in apoptotic pathway	2005
1777	O. Vall	Assessment of prenatal exposure to arsenic in Tenerife Island	2012
1778	M. Bassil	Lead, cadmium and arsenic in human milk and their socio-demographic and lifestyle determinants in Lebanon	2018
1779	S. Li	NF-κB-mediated inflammation correlates with calcium overload under arsenic trioxide-induced myocardial damage in Gallus gallus	2017
1780	S. Sarkar	Differential modulation of cellular antioxidant status in zebrafish liver and kidney exposed to low dose arsenic trioxide	2017

1781	B. K. Selvan	Synthesis and characterization of nano zerovalent iron-kaolin clay (nZVI-Kaol) composite polyethersulfone (PES) membrane for the efficacious As(2)O(3) removal from potable water samples	2022
1782	C. Yang	Integrity of zinc finger motifs in PML protein is necessary for inducing its degradation by antimony	2019
1783	E. Kamynina	Arsenic trioxide targets MTHFD1 and SUMO-dependent nuclear de novo thymidylate biosynthesis	2017
1784	Y. H. Jiang	Phenylarsine Oxide Can Induce the Arsenite-Resistance Mutant PML Protein Solubility Changes	2017
1785	Z. F. Miao	Increased aquaglyceroporin 9 expression disrupts arsenic resistance in human lung cancer cells	2009
1786	M. A. Khan	Inhibition of apoptosis in acute promyelocytic leukemia cells leads to increases in levels of oxidized protein and LMP2 immunoproteasome	2004
1787	P. La Rosée	In vitro efficacy of combined treatment depends on the underlying mechanism of resistance in imatinib-resistant Bcr-Abl-positive cell lines	2004
1788	Y. Shi	Arsenic trioxide induced apoptosis and expression of p53 and bcl-2 genes in human small cell lung cancer cells	2002
1789	Z. Y. Wang	Mechanism of action of all-trans retinoic acid and arsenic trioxide in the treatment of acute promyelocytic leukemia	2002
1790	H. Kreppel	Lack of effectiveness of D-penicillamine in experimental arsenic poisoning	1989
1791	A. M. Ramos	Pharmacologic inhibitors of extracellular signal-regulated kinase (ERKs) and c-Jun NH(2)-terminal kinase (JNK) decrease glutathione content and sensitize human promonocytic leukemia cells to arsenic trioxide-induced apoptosis	2006
1792	K. K. Saha	Pre- and postnatal arsenic exposure and body size to 2 years of age: a cohort study in rural Bangladesh	2012
1793	J. Dong	Effects of As2O3, MNNG and B(a)P on epithelia of human fetal tracheae and rat tracheae in organ culture	1990
1794	H. Yi	Genotoxicity of arsenic evaluated by Allium-root micronucleus assay	2007
1795	M. Hall	Determinants of arsenic metabolism: blood arsenic metabolites, plasma folate, cobalamin, and homocysteine concentrations in maternal-newborn pairs	2007
1796	L. Zhang	Effect of arsenic trioxide on the treatment of children with newly diagnosed acute promyelocytic leukemia in China	2011
1797	M. Singer	Arsenic trioxide reduces 2,4,6-trinitrobenzene sulfonic acid-induced murine colitis via nuclear factor-κB down-regulation and caspase-3 activation	2011
1798	J. Zhou	Clinical observation and following up of two administration methods of arsenic trioxide in treatment of acute promyelocytic leukemia	2004

1799	Q. Zhu	Synergic effects of arsenic trioxide and cAMP during acute promyelocytic leukemia cell maturation subtends a novel signaling cross-talk	2002
1800	S. J. Chen	Basic and clinical studies of the gene product-targeting therapy based on leukemogenesis--editorial	2005
1801	C. W. Hu	Oxidatively damaged DNA induced by humic acid and arsenic in maternal and neonatal mice	2010
1802	E. C. Chang	Arsenic oxide-induced thermotolerance in Saccharomyces cerevisiae	1989
1803	N. S. Bourguignon	Evaluation of sodium arsenite exposure on reproductive competence in pregnant and postlactational dams and their offspring	2017
1804	J. Clark	Maternal serum concentrations of one-carbon metabolism factors modify the association between biomarkers of arsenic methylation efficiency and birth weight	2022
1805	E. de Water	Prenatal metal mixture concentrations and reward motivation in children	2022
1806	Z. J. Su	Establishment of arsenic speciation analysis method and application in rice	2018
1807	M. Lozano	Exposure to metals and metalloids among pregnant women from Spain: Levels and associated factors	2022
1808	S. F. Farzan	Infant Infections and Respiratory Symptoms in Relation to in Utero Arsenic Exposure in a U.S. Cohort	2016
1809	Y. Bao	Nondestructive analysis of alterations of Chinese jade artifacts from Jinsha, Sichuan Province, China	2020
1810	R. S. Braman	Applications of arsine evolution methods to environmental analyses	1977
1811	J. H. Li	Comutagenesis of sodium arsenite with ultraviolet radiation in Chinese hamster V79 cells	1991
1812	E. Marafante	Dissolution of two arsenic compounds by rabbit alveolar macrophages in vitro	1987
1813	N. Yu	Th(As(III)4As(V)4O18): a mixed-valent oxoarsenic(III)/arsenic(V) actinide compound obtained under extreme conditions	2014
1814	P. Mahieu	The metabolism of arsenic in humans acutely intoxicated by As2O3. Its significance for the duration of BAL therapy	1981
1815	S. Klejna	Understanding 'clean-up' of III-V native oxides during atomic layer deposition using bulk first principles models	2011
1816	V. V. Mathews	Myocardial toxicity of acute promyelocytic leukaemia drug-arsenic trioxide	2013

1817	Y. H. Kang	Role of p38 MAPK and JNK in enhanced cervical cancer cell killing by the combination of arsenic trioxide and ionizing radiation	2008
1818	L. Liu	Speciation analysis of arsenic compounds by capillary electrophoresis on-line coupled with inductively coupled plasma mass spectrometry using a novel interface	2013
1819	U. Arroyo-Abad	Detection of arsenic-containing hydrocarbons in canned cod liver tissue	2010
1820	K. Yoshida	Urinary excretion of arsenic metabolites after long-term oral administration of various arsenic compounds to rats	1998
1821	T. Umezu	Simultaneous blood and brain microdialysis in a free-moving mouse to test blood-brain barrier permeability of chemicals	2020
1822	K. Loźna	The occurrence of arsenc in the environment and food	2008
1823	W. Goessler	Accurate quantification and transformation of arsenic compounds during wet ashing with nitric acid and microwave assisted heating	2003
1824	Y. A. Bioud	Chemical Composition of Nanoporous Layer Formed by Electrochemical Etching of p-Type GaAs	2016
1825	F. F. Wang	Deficiency of SUMO-specific protease 1 induces arsenic trioxide-mediated apoptosis by regulating XBP1 activity in human acute promyelocytic leukemia	2016
1826	C. M. Nellessen	Successful treatment of acute promyelocytic leukemia in pregnancy with single-agent all-trans retinoic acid	2018
1827	P. Hu	Arsenic adsorption enhancement performances of Mn-modified γ-Al(2)O(3) with flue gas constituents involved	2022
1828	R. Haque	Immunomodulatory Role of Arsenic in Regulatory T Cells	2017
1829	P. Chen	Prognostic significance of CD44v6/v7 in acute promyelocytic leukemia	2012
1830	E. C. Ive	Therapeutic effect of Arsenicum album on leukocytes	2012
1831	D. R. Yoo	Proteome profiling of arsenic trioxide-treated human hepatic cancer cells	2009
1832	V. Mathews	Single-agent arsenic trioxide in the treatment of newly diagnosed acute promyelocytic leukemia: durable remissions with minimal toxicity	2006
1833	S. S. Han	L-ascorbic acid represses constitutive activation of NF-kappaB and COX-2 expression in human acute myeloid leukemia, HL-60	2004
1834	W. A. Maher	The presence of arsenobetaine in marine animals	1985

1835	B. Wan	Studies of cytogenetic effects of sodium arsenicals on mammalian cells in vitro	1982
1836	L. Da Sacco	Chitin and chitosan as multipurpose natural polymers for groundwater arsenic removal and AS2O3 delivery in tumor therapy	2010
1837	J. Hu	Local delivery of arsenic trioxide nanoparticles for hepatocellular carcinoma treatment	2019
1838	Y. S. Pu	8-Oxoguanine DNA glycosylase and MutY homolog are involved in the incision of arsenite-induced DNA adducts	2007
1839	J. Qin	Simultaneous and ultrarapid determination of reactive oxygen species and reduced glutathione in apoptotic leukemia cells by microchip electrophoresis	2005
1840	S. F. Farzan	In utero arsenic exposure and infant infection in a United States cohort: a prospective study	2013
1841	G. Formenton	PM(10)-bound arsenic emissions from the artistic glass industry in Murano (Venice, Italy) before and after the enforcement of REACH authorisation	2021
1842	G. Liu	Arsenic compounds: The wide application and mechanisms applied in acute promyelocytic leukemia and carcinogenic toxicology	2021
1843	G. Caumette	Arsenobetaine formation in plankton: a review of studies at the base of the aquatic food chain	2012
1844	L. Liu	Influence of HMGB1/MAPK/m-TOR signaling pathway on cell autophagy and chemotherapy resistance in K562 cells	2016
1845	D. Muramatsu	Comparison of sensitivity to arsenic compounds between a Bhas 42 cell transformation assay and a BALB/c 3T3 cell transformation assay	2009
1846	S. Xi	Prenatal and early life arsenic exposure induced oxidative damage and altered activities and mRNA expressions of neurotransmitter metabolic enzymes in offspring rat brain	2010
1847	B. Tan	Anti-hepatoma effect of arsenic trioxide on experimental liver cancer induced by 2-acetamidofluorene in rats	2005
1848	P. D. Lin	Mediating role of arsenic in the relationship between diet and pregnancy outcomes: prospective birth cohort in Bangladesh	2019
1849	A. Mehta	Monoisoamyl dimercaptosuccinic acid induced changes in pregnant female rats during late gestation and lactation	2006
1850	F. Kayama	Exposure assessment of lead from food and airborne dusts and biomonitoring in pregnant mothers, their fetus and siblings in Karachi, Pakistan and Shimotsuke, Japan	2016
1851	F. Qureshi	Protective role of vitamin C and E against sodium arsenate induced changes in developing kidney of albino mice	2009
1852	H. Y. Tong	Inhibition effect of arsenic trioxide on the growth of human MDS cell line MUTZ-1 cells	2004

1853	A. Spanu	The role of irrigation techniques in arsenic bioaccumulation in rice (Oryza sativa L.)	2012
1854	N. De Keyser	Reviving degraded colors of yellow flowers in 17th century still life paintings with macro- and microscale chemical imaging	2022
1855	S. Ahmed	Arsenic exposure alters lung function and airway inflammation in children: A cohort study in rural Bangladesh	2017
1856	T. Nakazato	Green tea component, catechin, induces apoptosis of human malignant B cells via production of reactive oxygen species	2005
1857	P. Liu	Treatment of acute promyelocytic leukemia and other hematologic malignancies with arsenic trioxide: review of clinical and basic studies	2003
1858	T. Espevik	Effects of cadmium on survival and morphology of cultured rat Sertoli cells	1982
1859	A. Takeshita	P-glycoprotein (P-gp) and multidrug resistance-associated protein 1 (MRP1) are induced by arsenic trioxide (As(2)O(3)), but are not the main mechanism of As(2)O(3)-resistance in acute promyelocytic leukemia cells	2003
1860	O. Jie	Biomarkers of metal toxicity in embryos in the general population	2019
1861	S. Hornhardt	Comparative investigations of sodium arsenite, arsenic trioxide and cadmium sulphate in combination with gamma-radiation on apoptosis, micronuclei induction and DNA damage in a human lymphoblastoid cell line	2006
1862	G. Raber	Determination of 'arsenosugars' in algae with anion-exchange chromatography and an inductively coupled plasma mass spectrometer as element-specific detector	2000
1863	R. Z. Pei	Clinical investigation of homoharringtonine in combination with all-transretinoic acid and arsenic trioxide for acute promyelocytic leukemia	2013
1864	T. Mrak	Extraction of arsenic compounds from lichens	2006
1865	L. Hu	Differential mechanistic investigation of protective effects from imperatorin and sec-O-glucosylhamaudol against arsenic trioxide-induced cytotoxicity in vitro	2016
1866	G. F. Yang	Preparation, characterization, in vivo and in vitro studies of arsenic trioxide Mg-Fe ferrite magnetic nanoparticles	2009
1867	L. L. Zhou	Effect of arsenic trioxide combined with bortezomib on proliferation, apoptosis and beta-catenin level in myeloma cell lines	2008
1868	T. Wang	The effect of arsenic trioxide (As2O3) combined with BSO on K562/ADM cell and its mechanisms	2007
1869	F. X. Reichl	Effect of DMPS and various adsorbents on the arsenic excretion in guinea-pigs after injection with As2O3	1995
1870	L. Yu	Microfluidic chip-based cell electrophoresis with multipoint laser-induced fluorescence detection system	2007

1871	K. A. Francesconi	The identification of arsenobetaine as the sole water-soluble arsenic constituent of the tail muscle of the western king prawn Penaeus latisulcatus	1987
1872	S. M. Htway	Effects of maternal exposure to arsenic on social behavior and related gene expression in F2 male mice	2021
1873	T. Qiu	Taurine attenuates arsenic-induced pyroptosis and nonalcoholic steatohepatitis by inhibiting the autophagic-inflammasomal pathway	2018
1874	J. Zhou	Effects of Tat peptide on intracellular delivery of arsenic trioxide albumin microspheres	2012
1875	H. Sun	Arsenic trioxide regulates the apoptosis of glioma cell and glioma stem cell via down-regulation of stem cell marker Sox2	2011
1876	S. Y. Ho	Mechanisms of apoptosis induction and cell cycle regulation in irradiated leukemia U937 cells and enhancement by arsenic trioxide	2006
1877	V. F. Taylor	Distinct arsenic metabolites following seaweed consumption in humans	2017
1878	T. Llorente-Mirandes	Measurement of arsenic compounds in littoral zone algae from the Western Mediterranean Sea. Occurrence of arsenobetaine	2010
1879	A. Hartwig	Interaction of arsenic(III) with nucleotide excision repair in UV-irradiated human fibroblasts	1997
1880	M. McCabe	The effects of arsenic compounds on human and bovine lymphocyte mitogenesis in vitro	1983
1881	Y. Wu	microRNAs expression profile in acute promyelocytic leukemia cell differentiation induced by all-trans retinoic acid and arsenic trioxide	2012
1882	K. J. Lee	Assessment of zero-valent iron as a permeable reactive barrier for long-term removal of arsenic compounds from synthetic water	2009
1883	S. Y. Chen	Effects of arsenic trioxide and ATRA on PLZF-RARalpha-positive U937 leukemic cells	2007
1884	Z. Y. Yu	Melissoidesin G, a diterpenoid purified from Isodon melissoides, induces leukemic-cell apoptosis through induction of redox imbalance and exhibits synergy with other anticancer agents	2007
1885	P. Apostoli	Metal ions affecting reproduction and development	2011
1886	Y. Zhang	Effect of bcl-2 antisense oligodeoxynucleotides on drug sensitivity of leukemic cells	2003
1887	C. M. Bulka	Predictors of toxic metal exposures among US women of reproductive age	2019
1888	Z. Xing	A novel method for dearsenization from arsenic-bearing waste slag by selective chlorination and low-temperature volatilization	2022

1889	R. Venè	Glycogen synthase kinase 3 regulates cell death and survival signaling in tumor cells under redox stress	2014
1890	R. Wang	Synergism between carnosic acid and arsenic trioxide on induction of acute myeloid leukemia cell apoptosis is associated with modulation of PTEN/Akt signaling pathway	2012
1891	L. Li	Down-regulation of expression of vascular endothelial growth factor induced by arsenic trioxide in bone marrow cells of chronic myeloid leukemia	2003
1892	D. C. Bellinger	Prenatal Exposures to Environmental Chemicals and Children's Neurodevelopment: An Update	2013
1893	X. Tian	Deregulation of autophagy is involved in nephrotoxicity of arsenite and fluoride exposure during gestation to puberty in rat offspring	2020
1894	K. Schofield	An Important Need to Monitor from an Early Age the Neurotoxins in the Blood or by an Equivalent Biomarker	2019
1895	R. E. Morrissey	Arsenic-induced exencephaly in the mouse and associated lesions occurring during neurulation	1983
1896	J. B. Mason	Iodine fortification is related to increased weight-for-age and birthweight in children in Asia	2002
1897	S. Zierler	Chemical quality of maternal drinking water and congenital heart disease	1988
1898	J. Bornstein	Arsenic Trioxide inhibits the growth of human ovarian carcinoma cell line	2005
1899	J. Vuky	Phase II trial of arsenic trioxide in patients with metastatic renal cell carcinoma	2002
1900	W. H. Miller, Jr.	Molecular targets of arsenic trioxide in malignant cells	2002
1901	M. S. Bloom	Associations between blood metals and fecundity among women residing in New York State	2011
1902	J. E. Heck	Risk of leukemia in relation to exposure to ambient air toxics in pregnancy and early childhood	2014
1903	K. Nouri	The incidence of recurrent herpes simplex and herpes zoster infection during treatment with arsenic trioxide	2006
1904	J. E. Biaglow	G6PD deficient cells and the bioreduction of disulfides: effects of DHEA, GSH depletion and phenylarsine oxide	2000
1905	J. D. Tariman	Understanding novel therapeutic agents for multiple myeloma	2003
1906	Y. Zhang	Determination of arsenic in As2O3-treated esophagoscope-transplant mouse tissue by graphite furnace atomic absorption spectrometry	2004

1907	G. La Paglia	Possible etiologic role of occupational exposure to arsenic anhydride in a case of bladder carcinoma	1996
1908	A. F. Machado	Teratogenic response to arsenite during neurulation: relative sensitivities of C57BL/6J and SWV/Fnn mice and impact of the splotch allele	1999
1909	O. Landgren	Environmental pollution and delivery outcome in southern Sweden: a study with central registries	1996
1910	L. Makgoo	Downregulation of RBBP6 variant 1 during arsenic trioxide-mediated cell cycle arrest and curcumin-induced apoptosis in MCF-7 breast cancer cells	2019
1911	H. Zhao	Adenovirus-delivered PDCD5 counteracts adriamycin resistance of osteosarcoma cells through enhancing apoptosis and inhibiting Pgp	2014
1912	S. Y. Woo	Arsenic trioxide inhibits cell growth in SH-SY5Y and SK-N-AS neuroblastoma cell lines by a different mechanism	2006
1913	Y. Zhang	Up-regulation of telomere-binding TRF1, TRF2 related to reactive oxygen species induced by As(2)O(3) in MGC-803 cells	2005
1914	B. Salazard	Low-level arsenite activates the transcription of genes involved in adipose differentiation	2004
1915	M. H. Koken	Retinoic acid, but not arsenic trioxide, degrades the PLZF/RARalpha fusion protein, without inducing terminal differentiation or apoptosis, in a RA-therapy resistant t(11;17)(q23;q21) APL patient	1999
1916	C. Marie	Exposure to arsenic in tap water and gestational diabetes: A French semi-ecological study	2018
1917	G. Onicescu	Bayesian importance parameter modeling of misaligned predictors: soil metal measures related to residential history and intellectual disability in children	2014
1918	X. Song	Surface-modified PLGA nanoparticles with PEG/LA-chitosan for targeted delivery of arsenic trioxide for liver cancer treatment: Inhibition effects enhanced and side effects reduced	2019
1919	Y. Zhang	Arsenic trioxide-induced hERG K(+) channel deficiency can be rescued by matrine and oxymatrine through up-regulating transcription factor Sp1 expression	2013
1920	D. M. Smith	Arsenic trioxide induces a beclin-1-independent autophagic pathway via modulation of SnoN/SkiL expression in ovarian carcinoma cells	2010
1921	T. Sakurai	Inorganic and methylated arsenic compounds induce cell death in murine macrophages via different mechanisms	1998
1922	J. Z. Wu	Speciation of inorganic and methylated arsenic compounds by capillary zone electrophoresis with indirect UV detection. Application to the analysis of alkali extracts of As2S2 (realgar) and As2S3 (orpiment)	2004
1923	E. Marafante	Solubility, retention, and metabolism of intratracheally and orally administered inorganic arsenic compounds in the hamster	1987
1924	G. Zheng	Levels of heavy metals and trace elements in umbilical cord blood and the risk of adverse pregnancy outcomes: a population-based study	2014

1925	K. Karimov	Morphological peculiarities of rat liver reaction to chronic xenobiotic exposure	2002
1926	J. C. Lui	Cordycepin induced eryptosis in mouse erythrocytes through a Ca2+-dependent pathway without caspase-3 activation	2007
1927	C. Hogstedt	A cohort study on mortality among long-time employed Swedish chimney sweeps	1982
1928	A. Stajnko	Arsenic metabolites; selenium; and AS3MT, MTHFR, AQP4, AQP9, SELENOP, INMT, and MT2A polymorphisms in Croatian-Slovenian population from PHIME-CROME study	2019
1929	C. C. Lin	In utero exposure to environmental lead and manganese and neurodevelopment at 2 years of age	2013
1930	H. X. Yan	Pediatric acute myeloid leukemia patients with i(17)(q10) mimicking acute promyelocytic leukemia: Two case reports	2022
1931	G. Xie	Eriodictyol attenuates arsenic trioxide-induced liver injury by activation of Nrf2	2017
1932	K. Rehman	Trivalent methylated arsenic metabolites induce apoptosis in human myeloid leukemic HL-60 cells through generation of reactive oxygen species	2014
1933	J. W. Liu	Synergistic effect of cell differential agent-II and arsenic trioxide on induction of cell cycle arrest and apoptosis in hepatoma cells	2003
1934	F. X. Reichl	Effect of various antidotes on biliary excretion of arsenic in isolated perfused livers of guinea pigs after acute experimental poisoning with As2O3	1992
1935	F. X. Reichl	Effect of chelating agents on biliary excretion of arsenic in perfused livers of guinea pigs pretreated with As2O3	1990
1936	H. Roels	The possible role of direct ingestion on the overall absorption of cadmium or arsenic in workers exposed to CdO or As2O3 dust	1982
1937	P. Auberger	BCR-ABL/p62/SQSTM1: a cannibal embrace	2012
1938	A. M. Padula	Drinking water contaminants in California and hypertensive disorders in pregnancy	2021
1939	C. Zhang	Arsenic downregulates gene expression at the postsynaptic density in mouse cerebellum, including genes responsible for long-term potentiation and depression	2014
1940	C. Fernández	12-O-tetradecanoylphorbol-13-acetate may both potentiate and decrease the generation of apoptosis by the antileukemic agent arsenic trioxide in human promonocytic cells. Regulation by extracellular signal-regulated protein kinases and glutathione	2004
1941	H. T. Yang	Lifelong inorganic arsenic compounds consumption affected blood pressure in rats	2007
1942	C. G. Yedjou	Modulation of p53, c-fos, RARE, cyclin A, and cyclin D1 expression in human leukemia (HL-60) cells exposed to arsenic trioxide	2009

1943	Z. Li	Portal Vein Stenting Combined with (125)I Particle Chain Implantation Followed by As(2)O(3) in the Treatment of Hepatocellular Carcinoma with Portal Vein Tumour Thrombus	2020
1944	J. Liu	Arsenic (III) or/and copper (II) exposure induce immunotoxicity through trigger oxidative stress, inflammation and immune imbalance in the bursa of chicken	2020
1945	P. Binu	Studies on curative efficacy of monoterpene eugenol on anti- leukemic drug arsenic trioxide induced cardiotoxicity	2017
1946	Z. C. Fu	As2O3 may be a treatment option for adenoid cystic carcinoma of salivary gland	2010
1947	C. W. Dai	Use of all-trans retinoic acid in combination with arsenic trioxide for remission induction in patients with newly diagnosed acute promyelocytic leukemia and for consolidation/maintenance in CR patients	2009
1948	M. Marty	Arsenic trioxide-induced osteo-necrosis treatment in a child: mini-review and case report	2016
1949	D. E. Wilcox	Arsenic. Can this toxic metalloid sustain life?	2013
1950	Suhendrayatna	Studies on the accumulation and transformation of arsenic in freshwater organisms II. Accumulation and transformation of arsenic compounds by Tilapia mossambica	2002
1951	J. W. Lv	Gestational arsenic exposure induces anxiety-like behaviors in adult offspring by reducing DNA hydroxymethylation in the developing brain	2021
1952	J. M. Rogers	Elevated blood pressure in offspring of rats exposed to diverse chemicals during pregnancy	2014
1953	H. Q. Yuan	Nearly isotropic superconductivity in (Ba,K)Fe(2)As(2)	2009
1954	H. Neumann	Toxicity testing of heavy metals with the Rhizobium-legume symbiosis: High sensitivity to cadmium and arsenic compounds	1998
1955	T. Rossman	Effects of sodium arsenite on the survival of UV-irradiated Escherichia coli: inhibition of a recA-dependent function	1975
1956	B. George	Molecular remission with arsenic trioxide in patients with newly diagnosed acute promyelocytic leukemia	2004
1957	B. Li	Defect creation in metal-organic frameworks for rapid and controllable decontamination of roxarsone from aqueous solution	2016
1958	A. M. Sakuma	Arsenic exposure assessment of children living in a lead mining area in Southeastern Brazil	2010
1959	Y. P. Song	DNA hydroxymethylation reprogramming of β-oxidation genes mediates early-life arsenic-evoked hepatic lipid accumulation in adult mice	2022
1960	M. Gasparetto	Targeted therapy for a subset of acute myeloid leukemias that lack expression of aldehyde dehydrogenase 1A1	2017

1961	K. Dix	Arsenic speciation by capillary gas-liquid chromatography	1987
1962	G. Cimino-Reale	Combined in utero and juvenile exposure of mice to arsenate and atrazine in drinking water modulates gene expression and clonogenicity of myeloid progenitors	2008
1963	J. L. Domingo	Oral meso-2,3-dimercaptosuccinic acid in pregnant Sprague-Dawley rats: teratogenicity and alterations in mineral metabolism. I. Teratological evaluation	1990
1964	M. E. Dávila-Esqueda	Effects of arsenic exposure during the pre- and postnatal development on the puberty of female offspring	2012
1965	K. F. Tonissen	Thioredoxin system inhibitors as mediators of apoptosis for cancer therapy	2009
1966	W. C. Chou	Maternal arsenic exposure and DNA damage biomarkers, and the associations with birth outcomes in a general population from Taiwan	2014
1967	M. M. Howlader	Formation of gallium arsenide nanostructures in Pyrex glass	2013
1968	L. Zhan	Regulatory role of KEAP1 and NRF2 in PPARγ expression and chemoresistance in human non-small-cell lung carcinoma cells	2012
1969	V. H. Ferm	Arsenic as a teratogenic agent	1977
1970	S. Li	Taurine Supplementation Ameliorates Arsenic-Induced Hepatotoxicity and Oxidative Stress in Mouse	2019
1971	Y. Zheng	Protection of Taurine Against Arsenic-Induced DNA Damage of Mice Kidneys	2017
1972	L. Hu	Arsenic-induced sumoylation of Mus81 is involved in regulating genomic stability	2017
1973	Y. Tan	Thioredoxin-1 inhibitor PX-12 induces human acute myeloid leukemia cell apoptosis and enhances the sensitivity of cells to arsenic trioxide	2014
1974	Z. Yu	N-(beta-Elemene-13-yl)tryptophan methyl ester induces apoptosis in human leukemia cells and synergizes with arsenic trioxide through a hydrogen peroxide dependent pathway	2008
1975	Q. Mei	Analysis of high-energy x-ray diffraction data at high pressure: the case of vitreous As(2)O(3) at 32 GPa	2007
1976	H. Q. Ye	Membrane toxicity accounts for apoptosis induced by realgar nanoparticles in promyelocytic leukemia HL-60 cells	2005
1977	H. Yu	Arterial embolization hyperthermia using As2O3 nanoparticles in VX2 carcinoma-induced liver tumors	2011
1978	C. Dittrich	Acute self-poisoning with arsenic and treatment with BAL (author's transl)	1978

1979	J. M. Skeaff	A new method for the characterisation and quantitative speciation of base metal smelter stack particulates	2011
1980	X. Li	[The effects of different post-remission treatment on long-term survival of acute promyelocytic leukemia]	2006
1981	F. X. Reichl	Effect of various antidotes on the biliary and intestinal excretion of arsenic in situ and into the feces in vivo in guinea-pigs after injection of As2O3	1994
1982	J. E. Wahlberg	Contact sensitivity to arsenical compounds. Clinical and experimental studies	1986
1983	G. K. Tam	Determination of arsenic in urine and feces by dry ashing, atomic absorption spectrometry	1980
1984	K. S. Ljung	Maternal and early life exposure to manganese in rural Bangladesh	2009
1985	A. J. Houben	Factors Affecting Elevated Arsenic and Methyl Mercury Concentrations in Small Shield Lakes Surrounding Gold Mines near the Yellowknife, NT, (Canada) Region	2016
1986	B. Kumari	Acute exposure of arsenic tri-oxide produces hyperglycemia in both sexes of an Indian teleost, Clarias batrachus (Linn.)	2011
1987	A. Wedrychowski	DNA-protein crosslinking by heavy metals in Novikoff hepatoma	1986
1988	Y. Jing	Alteration of subcellular redox equilibrium and the consequent oxidative modification of nuclear factor kappaB are critical for anticancer cytotoxicity by emodin, a reactive oxygen species-producing agent	2006
1989	R. Soler-Blasco	Prenatal arsenic exposure, arsenic methylation efficiency, and neuropsychological development among preschool children in a Spanish birth cohort	2022
1990	J. Bai	JWA regulates melanoma metastasis by integrin alphaVbeta3 signaling	2010
1991	F. Gao	The cell cycle related apoptotic susceptibility to arsenic trioxide is associated with the level of reactive oxygen species	2004
1992	T. Kawai	ZIP kinase triggers apoptosis from nuclear PML oncogenic domains	2003
1993	U. Kohlmeyer	Simultaneous separation of 17 inorganic and organic arsenic compounds in marine biota by means of high-performance liquid chromatography/inductively coupled plasma mass spectrometry	2002
1994	J. E. Rager	Benchmark Dose Modeling Estimates of the Concentrations of Inorganic Arsenic That Induce Changes to the Neonatal Transcriptome, Proteome, and Epigenome in a Pregnancy Cohort	2017
1995	M. Bronkowska	Influence of arsenic on selected biochemical blood parameters in rats fed diet with different fat and protein content	2015
1996	S. Lehmann	Arsenic efficient in acute promyelocytic leukemia	1999

1997	C. D. Kozul-Horvath	Effects of low-dose drinking water arsenic on mouse fetal and postnatal growth and development	2012
1998	K. V. F. Weyde	Gestational blood levels of toxic metal and essential element mixtures and associations with global DNA methylation in pregnant women and their infants	2021
1999	S. Ahmed	Arsenic-associated oxidative stress, inflammation, and immune disruption in human placenta and cord blood	2011
2000	Z. Rivera-Núñez	Association of biomarkers of exposure to metals and metalloids with maternal hormones in pregnant women from Puerto Rico	2021
2001	G. I. Kaletin	Unidirectionality of elemental state disorders in workers contacting arsenic compounds and in oncologic patients	2009
2002	X. Xia	Association between serum arsenic levels and gestational diabetes mellitus: A population-based birth cohort study	2018
2003	C. K. Colapinto	Is there a relationship between tea intake and maternal whole blood heavy metal concentrations?	2016
2004	K. L. Kuo	2-methoxyestradiol induces mitotic arrest, apoptosis, and synergistic cytotoxicity with arsenic trioxide in human urothelial carcinoma cells	2013
2005	M. T. Park	Combination treatment with arsenic trioxide and phytosphingosine enhances apoptotic cell death in arsenic trioxide-resistant cancer cells	2007
2006	T. Tanvetyanon	Herpes zoster during treatment with arsenic trioxide	2004
2007	Z. Slejkovec	Preliminary studies on arsenic species in some environmental samples	1996
2008	Y. F. Mu	Arsenic compounds induce apoptosis through caspase pathway activation in MA-10 Leydig tumor cells	2019
2009	J. Morton	Speciation of arsenic compounds in urine from occupationally unexposed and exposed persons in the U.K. using a routine LC-ICP-MS method	2006
2010	N. K. Erraguntla	An updated inhalation unit risk factor for arsenic and inorganic arsenic compounds based on a combined analysis of epidemiology studies	2012
2011	W. Mitchell	In situ ATR-FTIR and surface complexation modeling studies on the adsorption of dimethylarsinic acid and p-arsanilic acid on iron-(oxyhydr)oxides	2011
2012	R. Kubota	Placental transfer of arsenic to fetus of Dall's porpoises (Phocoenoides dalli)	2005
2013	S. S. Kim	Urinary trace metals individually and in mixtures in association with preterm birth	2018
2014	X. Wang	Evaluation of arsenic species in leukocytes and granulocytes of acute promyelocytic leukemia patients treated with arsenic trioxide	2021

2015	M. Song	Detection and clinical significance of PML protein expression of acute promyelocytic leukemia cells	2017
2016	E. C. Nyanza	Maternal exposure to arsenic and mercury and associated risk of adverse birth outcomes in small-scale gold mining communities in Northern Tanzania	2020
2017	M. Hartung	Malignant diseases of the inner nose--epidemiology and occupational medicine aspects	1989
2018	X. Wan	Oxidative inactivation of the lipid phosphatase phosphatase and tensin homolog on chromosome ten (PTEN) as a novel mechanism of acquired long QT syndrome	2011
2019	J. R. Gallego	Comprehensive waste characterization and organic pollution co-occurrence in a Hg and As mining and metallurgy brownfield	2015
2020	A. Lau	Arsenic inhibits autophagic flux, activating the Nrf2-Keap1 pathway in a p62-dependent manner	2013
2021	J. Luo	Maternal and early life arsenite exposure impairs neurodevelopment and increases the expression of PSA-NCAM in hippocampus of rat offspring	2013
2022	K. Inoue	Air Pollution and Adverse Pregnancy and Birth Outcomes: Mediation Analysis Using Metabolomic Profiles	2020
2023	S. M. Prabu	Ameliorative effect of diallyl trisulphide on arsenic-induced oxidative stress in rat erythrocytes and DNA damage in lymphocytes	2014
2024	C. Iyare	Consumption of calcium carbide-ripened banana by pregnant rats may programme for infertility in female offspring	2020
2025	M. T. Colomina	Influence of maternal stress on the effects of prenatal exposure to methylmercury and arsenic on postnatal development and behavior in mice: a preliminary evaluation	1997
2026	M. Izdebska	Arsenic trioxide preferentially induces nonapoptotic cell deaths as well as actin cytoskeleton rearrangement in the CHO AA8 cell line	2014
2027	S. Ahmed	Arsenic exposure affects plasma insulin-like growth factor 1 (IGF-1) in children in rural Bangladesh	2013
2028	S. A. Pergantis	Investigation of arsine-generating reactions using deuterium-labeled reagents and mass spectrometry	1997
2029	J. S. Lewis	Venus: halide cloud condensation and volatile element inventories	1982
2030	M. Taylor	Effects of oral exposure to arsenobetaine during pregnancy and lactation in Sprague-Dawley rats	2013
2031	K. Jensen	Arsenic as an environmental problem	2000
2032	D. Schmähl	Etiology of bronchial cancer: smoking, passive smoking, environment and occupation	1991

2033	A. Christakopoulos	Cellular metabolism of arsenocholine	1988
2034	G. Chen	Penetration of Arsenic and Deactivation of a Honeycomb V(2)O(5)-WO(3)/TiO(2) Catalyst in a Glass Furnace	2021
2035	Z. Gao	Effects of oxygen functional complexes on arsenic adsorption over carbonaceous surface	2018
2036	Y. E. Huang	Biological toxicity of heavy metals to Caenorhabditis elegans	2015
2037	S. Mishra	Inhibition of ribonuclease and protease activities in arsenic exposed rice seedlings: role of proline as enzyme protectant	2006
2038	P. G. Richardson	Novel biological therapies for the treatment of multiple myeloma	2005
2039	I. D. Clark	Sources and circulation of water and arsenic in the Giant Mine, Yellowknife, NWT, Canada	2004
2040	N. E. Keon	Validation of an arsenic sequential extraction method for evaluating mobility in sediments	2001
2041	R. A. Silva	Effects of dietary fat on benz-a-pyrene-induced forestomach tumorigenesis in mice chronically exposed to arsenic	2000
2042	M. Bellés	Interactions in developmental toxicology: effects of concurrent exposure to lead, organic mercury, and arsenic in pregnant mice	2002
2043	Y. Zhou	Cytotoxicity of arsenic trioxide in single leukemia cells by time-resolved ICP-MS together with lanthanide tags	2017
2044	S. Ganapathy	Chronic low dose arsenic exposure preferentially perturbs mitotic phase of the cell cycle	2019
2045	S. A. Pergantis	Speciation of arsenic animal feed additives by microbore high-performance liquid chromatography with inductively coupled plasma mass spectrometry	1997
2046	Đ. Miodragović	Iodide Analogs of Arsenoplatins-Potential Drug Candidates for Triple Negative Breast Cancers	2021
2047	X. Y. Li	The antitumor effects of arsenic trioxide in mantle cell lymphoma via targeting Wnt/β-catenin pathway and DNA methyltransferase-1	2017
2048	Z. J. Long	ATO/ATRA/anthracycline-chemotherapy sequential consolidation achieves long-term efficacy in primary acute promyelocytic leukemia	2014
2049	F. A. Alexander, Jr.	Design and validation of a multi-electrode bioimpedance system for enhancing spatial resolution of cellular impedance studies	2013
2050	B. Dolniak	Regulation of arsenic trioxide-induced cellular responses by Mnk1 and Mnk2	2008

2051	D. M. Sayah	Selection for loss of Ref1 activity in human cells releases human immunodeficiency virus type 1 from cyclophilin A dependence during infection	2004
2052	J. S. Jia	Effect of arsenic trioxide on the expression of cyclins gene in HL60 cells	2003
2053	Y. Y. Chang	The down-regulation of galectin-1 expression is a specific biomarker of arsenic toxicity	2011
2054	P. Gokhalé	The effect of intracellular ascorbate on the susceptibility of HL60 and Jurkat cells to chemotherapy agents	2006
2055	G. Wang	An efficient therapeutic approach to patients with acute promyelocytic leukemia using a combination of arsenic trioxide with low-dose all-trans retinoic acid	2004
2056	K. Kitamura	Histone deacetylase inhibitor but not arsenic trioxide differentiates acute promyelocytic leukaemia cells with t(11;17) in combination with all-trans retinoic acid	2000
2057	R. Loch-Caruso	Inhibition of metabolic coupling by metals	1991
2058	A. List	Opportunities for Trisenox (arsenic trioxide) in the treatment of myelodysplastic syndromes	2003
2059	J. E. Rager	Prenatal exposure to arsenic and cadmium impacts infectious disease-related genes within the glucocorticoid receptor signal transduction pathway	2014
2060	P. Grandjean	Relation of a seafood diet to mercury, selenium, arsenic, and polychlorinated biphenyl and other organochlorine concentrations in human milk	1995
2061	S. Dominguez	Neurobehavioral and neurochemical effects in rats offspring co-exposed to arsenic and fluoride during development	2021
2062	J. H. Zhang	Pharmacokinetics of bacteria bioleaching solution of realgar in rat	2010
2063	K. Minakata	Simple and selective determination of arsenite and arsenate by electrospray ionization mass spectrometry	2009
2064	E. Schmeisser	Volatile analytes formed from arsenosugars: determination by HPLC-HG-ICPMS and implications for arsenic speciation analyses	2004
2065	S. Li	A novel fusion protein TBLR1-RARα acts as an oncogene to induce murine promyelocytic leukemia: identification and treatment strategies	2021
2066	H. Zhu	Synergistic Induction of Apoptosis by Chemotherapeutic Drugs and Cytokines in Mouse T-Lymphoma Cell Line	2000
2067	C. F. Jelinek	Levels of arsenic in the United States food supply	1977
2068	F. X. Xu	Association between gestational arsenic exposure and intrauterine growth restriction: the role of folate content	2022

2069	J. F. Obrycki	A case-control analysis of maternal diet and risk of neural tube defects in Bangladesh	2019
2070	K. S. Engström	Chronic exposure to cadmium and arsenic strongly influences concentrations of 8-oxo-7,8-dihydro-2'-deoxyguanosine in urine	2010
2071	O. Imataki	Chromosomal abnormality of acute promyelocytic leukemia other than PML-RARA: a case report of acute promyelocytic leukemia with del(5q)	2016
2072	Y. Komiya	Study and application of molluscicides in Japan	1961
2073	F. Rydbeck	Maternal urinary iodine concentration up to 1.0 mg/L is positively associated with birth weight, length, and head circumference of male offspring	2014
2074	P. Y. Shuai	Determination of arsenic species in Solanum Lyratum Thunb using capillary electrophoresis with inductively coupled plasma mass spectrometry	2016
2075	S. García Salgado	Determination of soluble toxic arsenic species in alga samples by microwave-assisted extraction and high performance liquid chromatography-hydride generation-inductively coupled plasma-atomic emission spectrometry	2006
2076	F. Kitagawa	Analysis of arsenic compounds by capillary electrophoresis using indirect UV and mass spectrometric detections	2006
2077	C. F. Yeh	Speciation of arsenic compounds in fish and oyster tissues by capillary electrophoresis-inductively coupled plasma-mass spectrometry	2005
2078	D. Cholujova	Realgar nanoparticles versus ATO arsenic compounds induce in vitro and in vivo activity against multiple myeloma	2017
2079	Y. Fang	Determination of total arsenic in sinters by hydride generation atomic fluorescence spectrometry	2002
2080	A. O. Summers	Plasmid-determined resistance to tellurium compounds	1977
2081	H. Newesely	Lead and arsenic resorption in the hard tissues (crystallochemical studies)	1975
2082	M. S. Hossain	Smokeless tobacco consumption and stillbirth: Population-based case-control study in rural Bangladesh	2018
2083	J. Lu	Treatment outcomes in relapsed acute promyelocytic leukemia patients initially treated with all-trans retinoic acid and arsenic compound-based combined therapies	2014
2084	Y. L. Li	Form and valence of arsenic in dry and fresh Cordyceps breeding products based on HPLC-ICP-MS and its risk assessment	2022
2085	F. Firdaus	Evaluation of phyto-medicinal efficacy of thymoquinone against Arsenic induced mitochondrial dysfunction and cytotoxicity in SH-SY5Y cells	2019
2086	N. Giafis	Role of the p38 mitogen-activated protein kinase pathway in the generation of arsenic trioxide-dependent cellular responses	2006

2087	Y. H. Kang	Caspase-independent cell death by arsenic trioxide in human cervical cancer cells: reactive oxygen species-mediated poly(ADP-ribose) polymerase-1 activation signals apoptosis-inducing factor release from mitochondria	2004
2088	D. Lu	Hydrogen peroxide in the Burkitt's lymphoma cell line Raji provides protection against arsenic trioxide-induced apoptosis via the phosphoinositide-3 kinase signalling pathway	2004
2089	J. W. Dube	Homoleptic pnictogen-chalcogen coordination complexes	2012
2090	L. Jedynak	Speciation analysis of arsenic in terrestrial plants from arsenic contaminated area	2009
2091	W. Zhang	Efficient removal and recovery of arsenic from copper smelting flue dust by a roasting method: Process optimization, phase transformation and mechanism investigation	2021
2092	K. Zhang	An extracellular pH-driven targeted multifunctional manganese arsenite delivery system for tumor imaging and therapy	2019
2093	L. Chen	Meta-analysis of all-trans retinoic acid-linked arsenic trioxide treatment for acute promyelocytic leukemia	2014
2094	S. Liu	Arsenic-induced inhibition of hippocampal neurogenesis and its reversibility	2012
2095	R. C. Vineetha	L-ascorbic acid and α-tocopherol attenuate arsenic trioxide-induced toxicity in H9c2 cardiomyocytes by the activation of Nrf2 and Bcl2 transcription factors	2018
2096	H. Skröder	Kidney function and blood pressure in preschool-aged children exposed to cadmium and arsenic--potential alleviation by selenium	2015
2097	X. Y. Zhao	Resveratrol protects against arsenic trioxide-induced cardiotoxicity in vitro and in vivo	2008
2098	E. Matsumoto-	Determination of Inorganic Arsenic in Seaweed and Seafood by LC-ICP-MS: Method Validation	2019
2099	P. Ramos-Morales	Genotoxicity of two arsenic compounds in germ cells and somatic cells of Drosophila melanogaster	1995
2100	Z. J. Li	Oxidation of Humic Acid Complexing As(III) by As(III)-Oxidizing Bacteria	2018
2101	S. M. Ding	Effects of Arsenic Trioxide on Cdc20 and Mad2 in Acute Myeloid Leukemia HL-60 Cell Line	2018
2102	D. L. Palazzolo	The minimal arsenic concentration required to inhibit the activity of thyroid peroxidase activity in vitro	2008
2103	P. Hantson	Acute arsenic poisoning treated by intravenous dimercaptosuccinic acid (DMSA) and combined extrarenal epuration techniques	2003
2104	J. L. Domingo	meso-2,3-Dimercaptosuccinic acid and prevention of arsenite embryotoxicity and teratogenicity in the mouse	1991

2105	N. Kannan	Structural and elemental characterization of traditional Indian Siddha formulation: Thalagak karuppu	2017
2106	H. Zhou	Postnatal low-concentration arsenic exposure induces autism-like behavior and affects frontal cortex neurogenesis in rats	2018
2107	S. Sarkar	Low dose of arsenic trioxide triggers oxidative stress in zebrafish brain: expression of antioxidant genes	2014
2108	Y. H. Wu	Reversal of drug resistance by silencing Survivin gene expression in acute myeloid leukemia cells	2008
2109	W. Zhao	Effects of all-trans-retinoic acid and arsenic trioxide on the hemostatic disturbance associated with acute promyelocytic leukemia	2001
2110	A. Kodre	A study of transferability of atomic background on EXAFS spectra of simple gaseous compounds of As	2001
2111	Y. Wang	Protective effect of taurine on down-regulated expression of thyroid hormone receptor genes in brains of mice exposed to arsenic	2013
2112	M. Brown	Emodin and DHA potently increase arsenic trioxide interferon-alpha-induced cell death of HTLV-I-transformed cells by generation of reactive oxygen species and inhibition of Akt and AP-1	2007
2113	F. Brugnoli	PLC-beta2 monitors the drug-induced release of differentiation blockade in tumoral myeloid precursors	2006
2114	D. Dermatas	An evaluation of arsenic release from monolithic solids using a modified semi-dynamic leaching test	2004
2115	E. Dopp	Cellular uptake, subcellular distribution and toxicity of arsenic compounds in methylating and non-methylating cells	2010
2116	S. G. Salgado	Optimisation of sample treatment for arsenic speciation in alga samples by focussed sonication and ultrafiltration	2006
2117	I. Baró-Camarasa	Essential and non-essential trace element concentrations in muscle and liver of a pregnant Munk's pygmy devil ray (Mobula munkiana) and its embryo	2021
2118	W. Zhang	Co-treatment of copper smelting flue dust and arsenic sulfide residue by a pyrometallurgical approach for simultaneous removal and recovery of arsenic	2021
2119	C. K. Chen	Adsorption Configurations of Iron Complexes on As(III) Adsorption Over Sludge Biochar Surface	2021
2120	C. E. Schuh	Controls governing the spatial distribution of sediment arsenic concentrations and solid-phase speciation in a lake impacted by legacy mining pollution	2019
2121	L. Zhang	One-Pot Synthesis of GeAs Ultrafine Particles from Coal Fly Ash by Vacuum Dynamic Flash Reduction and Inert Gas Condensation	2017
2122	M. Niu	Preventive effects of lutein on liver toxicity in mice induced by arsenic	2015

2123	H. Mückter	Isolated rat kidney tubules as a screening system for arsenic antidotes	1993
2124	M. S. Tsai	Children's environmental health based on birth cohort studies of Asia (2) - air pollution, pesticides, and heavy metals	2019
2125	C. Q. Feng	Detection of cell apoptosis by MTT assay	2002
2126	J. Ludvigsson	Toxic metals in cord blood and later development of Type 1 diabetes	2019
2127	M. Wulff	Cancer incidence for children born in a smelting community	1996
2128	R. D. Hood	Effects in the mouse and rat of prenatal exposure to arsenic	1977
2129	A. N. Tchernitchin	Influence of environmental pollutants on human gestation: Cause of adult pathologies	2018
2130	M. W. Taubeneck	Altered maternal zinc metabolism following exposure to diverse developmental toxicants	1994
2131	S. Shamim	Antibacterial, antifungal and enzymatic activities of azithromycin-heavy metal complexes: Newly synthesized and characterized	2021
2132	G. R. Hasegawa	Proposals for chemical weapons during the American Civil War	2008
2133	K. A. Francesconi	A novel arsenical in clam kidney identified by liquid chromatography/electrospray ionisation mass spectrometry	2001
2134	V. Lope	Cytogenetic status in newborns and their parents in Madrid: the BioMadrid study	2010
2135	J. Kluza	Exploiting mitochondrial dysfunction for effective elimination of imatinib-resistant leukemic cells	2011
2136	X. Li	Identification of the arsenic resistance on MoO3 doped CeO2/TiO2 catalyst for selective catalytic reduction of NOx with ammonia	2016
2137	Z. Wang	Differentiation therapy for acute promyelocytic leukemia with all-trans retinoic acid: 10-year experience of its clinical application	1999
2138	S. Davies	Pre-conception serum ferritin concentrations are associated with metal concentrations in blood during pregnancy: A cohort study in Benin	2021
2139	L. Polak-Juszczak	Arsenic speciation in fish from Baltic Sea close to chemical munitions dumpsites	2021
2140	D. Lerche	Ranking of chemical substances based on the Japanese Pollutant Release and Transfer Register using partial order theory and random linear extensions	2004

2141	T. Ochi	Arsenic compound-induced increases in glutathione levels in cultured Chinese hamster V79 cells and mechanisms associated with changes in gamma-glutamylcysteine synthetase activity, cystine uptake and utilization of cysteine	1997
2142	G. F. Nordberg	Metal interactions in carcinogenesis: enhancement, inhibition	1981
2143	L. Maitre	Urine Metabolic Signatures of Multiple Environmental Pollutants in Pregnant Women: An Exposome Approach	2018
2144	M. H. Nguyen	Speciation Analysis of Arsenic Compounds by HPLC-ICP-MS: Application for Human Serum and Urine	2018
2145	R. A. Menezes	Contribution of Yap1 towards Saccharomyces cerevisiae adaptation to arsenic-mediated oxidative stress	2008
2146	P. R. Gentry	Comparison of tissue dosimetry in the mouse following chronic exposure to arsenic compounds	2005
2147	J. A. Lehmann-Horn	As NQR studies on FeAs2	2015
2148	Z. Meng	Effects of arsenic on DNA synthesis in human lymphocytes stimulated by phytohemagglutinin	1993
2149	C. I. Nagy	Coregulated genes link sulfide:quinone oxidoreductase and arsenic metabolism in Synechocystis sp. strain PCC6803	2014
2150	M. D. Seftel	A Canadian consensus on the management of newly diagnosed and relapsed acute promyelocytic leukemia in adults	2014
2151	M. Fort	Assessment of exposure to trace metals in a cohort of pregnant women from an urban center by urine analysis in the first and third trimesters of pregnancy	2014
2152	J. Wu	[Current researches in microbial remediation of arsenic pollution]	2011
2153	R. A. Glabonjat	A 2-O-Methylriboside Unknown Outside the RNA World Contains Arsenic	2017
2154	A. Polatajko	Speciation of arsenic in chicken meat by anion-exchange liquid chromatography with inductively coupled plasma-mass spectrometry	2004
2155	Z. Meng	Effects of arsenic on DNA synthesis in human lymphocytes	1993
2156	L. Song	Exposure to arsenic during pregnancy and newborn mitochondrial DNA copy number: A birth cohort study in Wuhan, China	2020
2157	H. Ahsan	Health Effects of Arsenic Longitudinal Study (HEALS): description of a multidisciplinary epidemiologic investigation	2006
2158	S. Saritha	Effects of combined arsenic and lead exposure on the brain monoaminergic system and behavioral functions in rats: Reversal effect of MiADMSA	2019

2159	C. Freire	Concentrations and determinants of lead, mercury, cadmium, and arsenic in pooled donor breast milk in Spain	2022
2160	Y. Maimaitiyiming	Irreversibility of arsenic trioxide induced PML/RARα fusion protein solubility changes	2019
2161	H. Guan	Protection of Taurine Against Impairment in Learning and Memory in Mice Exposed to Arsenic	2017
2162	I. Ajana	Arsthinol nanosuspensions: pharmacokinetics and anti-leukaemic activity on NB4 promyelocytic leukaemia cells	2009
2163	V. Avilov	Resistive Switching of GaAs Oxide Nanostructures	2020
2164	W. Zhao	The apoptotic mechanism of hepatocellular carcinoma cell line (HepG2) induced by arsenic trioxide	2014
2165	F. A. Guardiola	Immunotoxicological effects of inorganic arsenic on gilthead seabream (Sparus aurata L.)	2013
2166	L. Wang	A study on the thermochemotherapy effect of nanosized As2O3/MZF thermosensitive magnetoliposomes on experimental hepatoma in vitro and in vivo	2011
2167	P. G. Smith	Arsenic speciation analysis of cultivated white button mushrooms (Agaricus bisporus) using high-performance liquid chromatography-inductively coupled plasma mass spectrometry, and X-ray absorption spectroscopy	2007
2168	N. A. Nasser	Lacustrine Arcellinina (Testate Amoebae) as Bioindicators of Arsenic Contamination	2016
2169	C. Li	Arsenic trioxide induces cardiac fibroblast apoptosis in vitro and in vivo by up-regulating TGF-β1 expression	2013
2170	T. J. Cheng	Protection against arsenic trioxide-induced autophagic cell death in U118 human glioma cells by use of lipoic acid	2007
2171	K. Hagenfeldt	Trace elements in the human endometrium and decidua. A multielement analysis	1977
2172	J. T. Liu	Association of the levels of heavy metals and trace elements during pregnancy with congenital heart defects in offspring: a prospective cohort study	2022
2173	B. M. Piraccini	Drug-induced nail disorders: incidence, management and prognosis	1999
2174	Y. Wang	Arsenic down-regulates the expression of Camk4, an important gene related to cerebellar LTD in mice	2009
2175	W. C. Chou	Acute promyelocytic leukemia: recent advances in therapy and molecular basis of response to arsenic therapies	2005
2176	H. Li	In vitro effects of metal ions on lipid peroxidation induced by alcohol in mice liver homogenate	2003

2177	W. Meng	Study on the relationship between NB4 cell apoptosis induced by tanshinone IIA and the cell mitochondrial transmembrane potential	2002
2178	Q. An	Joint effects of arsenic and lead on lipid peroxidation of human and rat erythrocytes	1998
2179	K. Y. Ding	Effect of recombinant VEGF-C secreted from eukaryotic cells on proliferation and chemotherapy-induced apoptosis of leukemic cells	2005
2180	W. R. Gao	Effect of 2-methoxyestradiol on proliferation and apoptosis of myeloma cell lines	2005
2181	K. T. Lim	Physical, chemical, and biological methods for the removal of arsenic compounds	2014
2182	D. Obregón	Physicians, prostitution, and venereal disease in Colombia (1886-1951)	2002
2183	H. Norin	Identification and quantification of arsenocholine and acetylarsenocholine in trace amounts in biological material by use of pyrolysis gas chromatography/mass spectrometry	1987
2184	Y. Xu	Intrauterine exposure of mice to arsenite induces abnormal and transgenerational glycometabolism	2022
2185	H. Qi	Concomitant induction of heme oxygenase-1 attenuates the cytotoxicity of arsenic species from lumbricus extract in human liver HepG2 cells	2012
2186	J. Száková	Mobility of arsenic and its compounds in soil and soil solution: the effect of soil pretreatment and extraction methods	2009
2187	T. C. Stummann	Embryotoxicity hazard assessment of cadmium and arsenic compounds using embryonic stem cells	2008
2188	T. Mohri	Arsenic intake and excretion by Japanese adults: a 7-day duplicate diet study	1990
2189	R. Recio-Vega	In utero and early childhood exposure to arsenic decreases lung function in children	2015
2190	F. Harari	Exposure to Lithium and Cesium Through Drinking Water and Thyroid Function During Pregnancy: A Prospective Cohort Study	2015
2191	L. Liu	Efficient interface for online coupling of capillary electrophoresis with inductively coupled plasma-mass spectrometry and its application in simultaneous speciation analysis of arsenic and selenium	2014
2192	G. Caumette	Arsenic speciation in plankton organisms from contaminated lakes: transformations at the base of the freshwater food chain	2011
2193	B. J. Majer	Genotoxic effects of dietary and lifestyle related carcinogens in human derived hepatoma (HepG2, Hep3B) cells	2004
2194	M. Stýblo	Identification of methylated metabolites of inorganic arsenic by thin-layer chromatography	1995

2195	K. L. Rice	The acute promyelocytic leukaemia success story: curing leukaemia through targeted therapies	2014
2196	J. Stone	Exposure to toxic metals and per- and polyfluoroalkyl substances and the risk of preeclampsia and preterm birth in the United States: a review	2021
2197	F. Au	Blood metal levels and third trimester maternal plasma matrix metalloproteinases (MMPs)	2016
2198	A. S. M. Fazle Bari	Geochemical fractionation and mineralogy of metal(loid)s in abandoned mine soils: Insights into arsenic behaviour and implications to remediation	2020
2199	T. Tandarić	Design of Exceptionally Strong Organic Superbases Based on Aromatic Pnictogen Oxides: Computational DFT Analysis of the Oxygen Basicity in the Gas Phase and Acetonitrile Solution	2018
2200	L. Gong	Assessment of 12 Essential and Toxic Elements in Whole Blood of Pregnant and Non-pregnant Women Living in Wuhan of China	2021
2201	X. Wang	Urinary concentrations of environmental metals and associating factors in pregnant women	2019
2202	Y. Wang	Exposure to multiple metals and prevalence for preeclampsia in Taiyuan, China	2020
2203	M. Suwalsky	Arsenite interactions with phospholipid bilayers as molecular models for the human erythrocyte membrane	2007
2204	V. M. Rodríguez	The effects of arsenic exposure on the nervous system	2003
2205	P. E. Crossen	Arsenic and SCE in human lymphocytes	1983
2206	E. Dopp	Uptake of inorganic and organic derivatives of arsenic associated with induced cytotoxic and genotoxic effects in Chinese hamster ovary (CHO) cells	2004
2207	D. Kuehnelt	Nitrogen purity influences the occurrence of As+ ions in high-performance liquid chromatography/electrospray ionization mass spectrometric analysis of four common arsenosugars	2003
2208	H. Imoto	The Dawn of Functional Organoarsenic Chemistry	2019
2209	C. Q. Feng	Research advances on effect of arsenic trioxide on tumor	2002
2210	H. Hahn	Genetically determined susceptibility markers in skin cancer and their application to chemoprevention	2001
2211	S. Shooshtary	Arsenic trioxide binding to serum proteins	2015
2212	L. E. Garner	Covalent Surface Modification of Gallium Arsenide Photocathodes for Water Splitting in Highly Acidic Electrolyte	2017

2213	K. H. Tsui	Metallothionein 3 Is a Hypoxia-Upregulated Oncogene Enhancing Cell Invasion and Tumorigenesis in Human Bladder Carcinoma Cells	2019
2214	A. Huang	SurvivinT34A increases the therapeutic efficacy of arsenic trioxide in mouse hepatocellular carcinoma models	2016
2215	K. K. Mann	Antimony trioxide-induced apoptosis is dependent on SEK1/JNK signaling	2006
2216	L. Singh	Environmental toxic metals in placenta and their effects on preterm delivery-current opinion	2020
2217	K. Kita	Structure-effect relationship in the down-regulation of glutaminase in cultured human cells by phenylarsenic compounds	2009
2218	A. P. Davis	The Comparative Toxicogenomics Database facilitates identification and understanding of chemical-gene-disease associations: arsenic as a case study	2008
2219	K. Kuroda	Microbial metabolite of dimethylarsinic acid is highly toxic and genotoxic	2004
2220	J. D. Cha	Analysis of the association between bladder carcinoma and arsenic concentration in soil and water in southeast Brazil	2018
2221	M. Pepi	Membrane fatty acids adaptive profile in the simultaneous presence of arsenic and toluene in Bacillus sp. ORAs2 and Pseudomonas sp. ORAs5 strains	2008
2222	M. P. Longnecker	Environmental contaminants as etiologic factors for diabetes	2001
2223	P. M. Newberne	Food additives and contaminants. An update	1986
2224	P. Wyrsch	Cytosolic Ca2+ shifts as early markers of cytotoxicity	2013
2225	T. Liu	Effect of NF-κB inhibitors on the chemotherapy-induced apoptosis of the colon cancer cell line HT-29	2012
2226	J. J. Stevens	Cytotoxic Effect of Arsenic Trioxide in Adenocarcinoma Colorectal Cancer (HT-29) Cells	2008
2227	M. H. Cohen	Drug approval summaries: arsenic trioxide, tamoxifen citrate, anastrazole, paclitaxel, bexarotene	2001
2228	L. Smeester	Toxic metals in amniotic fluid and altered gene expression in cell-free fetal RNA	2017
2229	F. A. Guardiola	Evaluation of waterborne exposure to heavy metals in innate immune defences present on skin mucus of gilthead seabream (Sparus aurata)	2015
2230	Y. Hong	Subchronic exposure to arsenic decreased Sdha expression in the brain of mice	2009

2231	T. Tachiwada	Isolation and characterization of arsenite-resistant human epidermoid carcinoma KB cells	2007
2232	M. Lindskog	Neuroblastoma cell death in response to docosahexaenoic acid: sensitization to chemotherapy and arsenic-induced oxidative stress	2006
2233	G. S. Chen	A possible pathogenesis for Blackfoot disease--effects of trivalent arsenic (As2O3) on cultured human umbilical vein endothelial cells	1990
2234	G. Y. Park	Reduction of total, organic, and inorganic arsenic content in Hizikia fusiforme (Hijiki)	2019
2235	T. Rezanka	Biologically active compounds of semi-metals	2008
2236	I. Mancini	On the first polyarsenic organic compound from nature: arsenicin A from the New Caledonian marine sponge Echinochalina bargibanti	2006
2237	T. Gebel	Comparative and environmental genotoxicity of antimony and arsenic	1997
2238	E. Byeon	Toxicity mechanisms of arsenic compounds in aquatic organisms	2021
2239	R. T. Xu	Construction and identification of human p-selectin promotor luciferase reporter gene vector	2016
2240	B. R. You	Arsenic trioxide induces human pulmonary fibroblast cell death via increasing ROS levels and GSH depletion	2012
2241	A. F. Gerdelidani	Arsenic geochemistry and mineralogy as a function of particle-size in naturally arsenic-enriched soils	2021
2242	M. Zidane	Non-Essential Trace Elements Dietary Exposure in French Polynesia: Intake Assessment, Nail Bio Monitoring and Thyroid Cancer Risk	2019
2243	A. A. Saucedo-Velez	Speciation analysis of organoarsenic compounds in livestock feed by microwave-assisted extraction and high performance liquid chromatography coupled to atomic fluorescence spectrometry	2017
2244	A. C. Schmidt	Analysis of accumulation, extractability, and metabolization of five different phenylarsenic compounds in plants by ion chromatography with mass spectrometric detection and by atomic emission spectroscopy	2008
2245	P. R. Gentry	Analysis of genomic dose-response information on arsenic to inform key events in a mode of action for carcinogenicity	2010
2246	K. Kitamura	New retinoids and arsenic compounds for the treatment of refractory acute promyelocytic leukemia: clinical and basic studies for the next generation	1997
2247	M. A. Lala	The reaction of allyl and benzylarsonic acids with thiols: mechanistic aspects and implications for dioxygen activation by trivalent arsenic compounds	2003
2248	T. R. Radabaugh	Enzymatic reduction of arsenic compounds in mammalian systems: reduction of arsenate to arsenite by human liver arsenate reductase	2000

2249	X. W. He	Effect of As_2O_3on Hedgehog Pathway in Chronic Myeloid Leukemia Cells	2015
2250	K. Schofield	The Metal Neurotoxins: An Important Role in Current Human Neural Epidemics?	2017
2251	X. Sun	Maternal Heavy Metal Exposure, Thyroid Hormones, and Birth Outcomes: A Prospective Cohort Study	2019
2252	Q. D. Lin	Regulatory effect of As_2O_3on imbalance between adipogenic and osteogenic differentiation of BM-MSC from patients with aplastic anemia	2014
2253	M. Moradnia	Monitoring of urinary arsenic (As) and lead (Pb) among a sample of pregnant Iranian women	2021
2254	A. R. Smith	Prospective Associations of Early Pregnancy Metal Mixtures with Mitochondria DNA Copy Number and Telomere Length in Maternal and Cord Blood	2021
2255	T. Kumagai	RWJ-241947 (MCC-555), a unique peroxisome proliferator-activated receptor-gamma ligand with antitumor activity against human prostate cancer in vitro and in beige/nude/ X-linked immunodeficient mice and enhancement of apoptosis in myeloma cells induced by arsenic	2004
2256	B. Daus	Concentrations and speciation of arsenic in groundwater polluted by warfare agents	2010
2257	Z. Y. Wang	Ham-Wasserman lecture: treatment of acute leukemia by inducing differentiation and apoptosis	2003
2258	R. B. Pearce	Fungal volatilization of arsenic and antimony and the sudden infant death syndrome	1998
2259	H. V. Aposhian	Enzymatic methylation of arsenic species and other new approaches to arsenic toxicity	1997
2260	J. N. Pirl	Death by arsenic: a comparative evaluation of exhumed body tissues in the presence of external contamination	1983
2261	M. A. Argudín	Heavy metal and disinfectant resistance genes among livestock-associated methicillin-resistant Staphylococcus aureus isolates	2016
2262	H. Hu	Effects of Arsenic Trioxide on INF-gamma Gene Expression in MRL/lpr Mice and Human Lupus	2018
2263	M. Wei	Alleviation of Arsenic-Induced Pulmonary Oxidative Damage by GSPE as Shown during In vivo and In vitro Experiments	2018
2264	F. Zhao	Effects of developmental arsenite exposure on hippocampal synapses in mouse offspring	2017
2265	Y. Yang	A preliminary study on the use of meconium for the assessment of prenatal exposure to heavy metals in Japan	2013
2266	F. Zhao	Alterations of NMDA and AMPA receptors and their signaling apparatus in the hippocampus of mouse offspring induced by developmental arsenite exposure	2019

2267	X. Xu	Accuracy of Effective Core Potentials and Basis Sets for Density Functional Calculations, Including Relativistic Effects, As Illustrated by Calculations on Arsenic Compounds	2011
2268	A. B. Islam	Arsenic mineral dissolution and possible mobilization in mineral-microbe-groundwater environment	2013
2269	Y. Long	V(2)O(5)-WO(3)/TiO(2) Catalyst for Efficient Synergistic Control of NO(x) and Chlorinated Organics: Insights into the Arsenic Effect	2021
2270	X. Jiang	Arsenic (III) and/or Antimony (III) induced disruption of calcium homeostasis and endoplasmic reticulum stress resulting in apoptosis in mice heart	2021
2271	Y. Wang	Interplay between elemental imbalance-related PI3K/Akt/mTOR-regulated apoptosis and autophagy in arsenic (III)-induced jejunum toxicity of chicken	2018
2272	K. Yang	Determination of human health risk incorporating experimentally derived site-specific bioaccessibility of arsenic at an old abandoned smelter site	2015
2273	M. Pastorek	Realgar (As4S4) nanoparticles and arsenic trioxide (As2O3) induced autophagy and apoptosis in human melanoma cells in vitro	2014
2274	X. Liu	Protective effect of taurine on the decreased biogenic amine neurotransmitter levels in the brain of mice exposed to arsenic	2013
2275	J. L. Xu	Stratification therapy in patients with acute promyelocytic leukemia after a complete remission by all-trans retinoic acid	2011
2276	S. L. Soignet	Clinical study of an organic arsenical, melarsoprol, in patients with advanced leukemia	1999
2277	W. H. Park	MAPK inhibitors and siRNAs differentially affect cell death and ROS levels in arsenic trioxide-treated human pulmonary fibroblast cells	2012
2278	K. Ebisuda	Lipid-soluble and water-soluble arsenic compounds in blubber of ringed seal (Pusa hispida)	2003
2279	T. Agusa	Specific accumulation of arsenic compounds in green turtles (Chelonia mydas) and hawksbill turtles (Eretmochelys imbricata) from Ishigaki Island, Japan	2008
2280	Y. Li	Conformational fluctuations coupled to the thiol-disulfide transfer between thioredoxin and arsenate reductase in Bacillus subtilis	2007
2281	J. K. Nag	Inorganic arsenic species in groundwater: a case study from Purbasthali (Burdwan), India	1996
2282	D. H. Cox	Arsine evolution-electrothermal atomic absorption method for the determination of nanogram levels of total arsenic in urine and water	1980
2283	E. A. Crecelius	Changes in the chemical speciation of arsenic following ingestion by man	1977
2284	W. H. Park	Upregulation of thioredoxin and its reductase attenuates arsenic trioxide-induced growth suppression in human pulmonary artery smooth muscle cells by reducing oxidative stress	2020

2285	J. Lu	Influence of AS3MT polymorphisms on arsenic metabolism and liver injury in APL patients treated with arsenic trioxide	2019
2286	S. F. Farzan	Prenatal lead exposure and elevated blood pressure in children	2018
2287	S. S. A. Alkurdi	Inorganic arsenic species removal from water using bone char: A detailed study on adsorption kinetic and isotherm models using error functions analysis	2021
2288	A. M. Walker	Arsenic trioxide modulates DNA synthesis and apoptosis in lung carcinoma cells	2010
2289	T. T. Zuo	HPLC-ICP-MS speciation analysis and risk assessment of arsenic in Cordyceps sinensis	2018
2290	G. K. Dalapati	Surface passivation and interface properties of bulk GaAs and epitaxial-GaAs/Ge using atomic layer deposited TiAlO alloy dielectric	2013
2291	H. Naranmandura	Comparative toxicity of arsenic metabolites in human bladder cancer EJ-1 cells	2011
2292	P. Wang	High-performance liquid chromatography-inductively coupled plasma mass spectrometry based method for the determination of organic arsenic feed additives and speciation of anionic arsenics in animal feed	2010
2293	Y. Shibata	Selenium and arsenic in biology: their chemical forms and biological functions	1992
2294	M. M. Nearing	Uptake and transformation of arsenic during the vegetative life stage of terrestrial fungi	2015
2295	M. L. Magnuson	Speciation of selenium and arsenic compounds by capillary electrophoresis with hydrodynamically modified electroosmotic flow and on-line reduction of selenium(VI) to selenium(IV) with hydride generation inductively coupled plasma mass spectrometric	1997
2296	H. Norin	A rapid method for the selective analysis of total urinary metabolites of inorganic arsenic	1981
2297	K. J. Irgolic	Characterization of arsenic compounds formed by Daphnia magna and Tetraselmis chuii from inorganic arsenate	1977
2298	R. D. Hood	Evaluation of the effect of BAL (2,3-dimercaptopropanol) on arsenite-induced teratogenesis in mice	1984
2299	C. L. McCarthy	Solution-Phase Conversion of Bulk Metal Oxides to Metal Chalcogenides Using a Simple Thiol-Amine Solvent Mixture	2015
2300	C. C. Hofmeister	Phase II clinical trial of arsenic trioxide with liposomal doxorubicin, vincristine, and dexamethasone in newly diagnosed multiple myeloma	2008
2301	B. Pierson	Dissolution of crystalline gallium arsenide in aqueous solutions containing complexing agents	1989
2302	H. Liu	Toxicant Deposition and Transport in Alveolus: A Classical Density Functional Prediction	2018

2303	D. S. Gutiérrez-Torres	Prenatal Exposure to Sodium Arsenite Alters Placental Glucose 1, 3, and 4 Transporters in Balb/c Mice	2015
2304	O. A. Adebambo	Cadmium disrupts signaling of the hypoxia-inducible (HIF) and transforming growth factor (TGF-β) pathways in placental JEG-3 trophoblast cells via reactive oxygen species	2018
2305	D. S. Hill	Reproductive consequences of oral arsenate exposure during pregnancy in a mouse model	2008
2306	C. Rodrigues-Pousada	Yeast AP-1 like transcription factors (Yap) and stress response: a current overview	2019
2307	A. J. DeGraffenreid	Dithiol Aryl Arsenic Compounds as Potential Diagnostic and Therapeutic Radiopharmaceuticals	2016
2308	T. Narukawa	Preparation and certification of arsenobetaine reference material NMIJ CRM 7901-a	2007
2309	S. S. Daniali	Birth Size Outcomes in Relation to Maternal Blood Levels of Some Essential and Toxic Elements	2022
2310	G. Mao	Iodine deficiency in pregnant women after the adoption of the new provincial standard for salt iodization in Zhejiang Province, China	2018
2311	C. Luvonga	Analytical Methodologies for the Determination of Organoarsenicals in Edible Marine Species: A Review	2020
2312	G. Cassone	Interaction between As(III) and Simple Thioacids in Water: An Experimental and ab Initio Molecular Dynamics Investigation	2019
2313	S. Tanda	Arsenic speciation in aerosols of a respiratory therapeutic cave: A first approach to study arsenicals in ultrafine particles	2019
2314	S. Simon	Simultaneous determination of twelve inorganic and organic arsenic compounds by liquid chromatography-ultraviolet irradiation-hydride generation atomic fluorescence spectrometry	2004
2315	J. N. Cottrell	Rural and urban differences in prenatal exposure to essential and toxic elements	2018
2316	P. García-Fortea	Toxic elements in hair and in vitro fertilization outcomes: A prospective cohort study	2018
2317	M. S. Lee	Umbilical Cord Blood Metal Mixtures and Birth Size in Bangladeshi Children	2021
2318	Y. Wang	Subchronic exposure to arsenic induces apoptosis in the hippocampus of the mouse brains through the Bcl-2/Bax pathway	2015
2319	G. Q. Chen	Methylated metabolites of arsenic trioxide are more potent than arsenic trioxide as apoptotic but not differentiation inducers in leukemia and lymphoma cells	2003
2320	Y. Li	Myocardial toxicity of arsenic trioxide in a mouse model	2002

2321	A. Mithander	Assessment of museum staff exposure to arsenic while handling contaminated exhibits by urinalysis of arsenic species	2017
2322	T. Agusa	Relationship between Arsenic (+3 Oxidation State) Methyltransferase Genetic Polymorphisms and Methylation Capacity of Inorganic Arsenic	2015
2323	C. Qian	Suppression of pancreatic tumor growth by targeted arsenic delivery with anti-CD44v6 single chain antibody conjugated nanoparticles	2013
2324	B. Özpolat	Acute promyelocytic leukemia and differentiation therapy: molecular mechanisms of differentiation, retinoic acid resistance and novel treatments	2009
2325	B. Ramanathan	Resistance to paclitaxel is proportional to cellular total antioxidant capacity	2005
2326	J. M. Galloway	Organic matter control on the distribution of arsenic in lake sediments impacted by ~65years of gold ore processing in subarctic Canada	2018
2327	M. Perri	BCL-xL/MCL-1 inhibition and RARγ antagonism work cooperatively in human HL60 leukemia cells	2014
2328	K. G. Raghu	Evaluation of adverse cardiac effects induced by arsenic trioxide, a potent anti-APL drug	2009
2329	G. Cepriá	Electrochemical screening procedure for arsenic contaminated soils	2005
2330	O. Kovalchuk	A sensitive transgenic plant system to detect toxic inorganic compounds in the environment	2001
2331	Z. Šlejkovec	Arsenic speciation and elemental composition of rice samples from the Slovenian market	2021
2332	H. Han	Effects of reaction conditions on the emission behaviors of arsenic, cadmium and lead during sewage sludge pyrolysis	2017
2333	A. L. Femia	Synthesis of a fluorescently labeled compound for the detection of arsenic-induced apoptotic HL60 cells	2012
2334	Y. Morita	Study on simultaneous speciation of arsenic and antimony by HPLC-ICP-MS	2007
2335	R. D. Tripathi	Arsenic hazards: strategies for tolerance and remediation by plants	2007
2336	P. Boffetta	Carcinogenicity of trace elements with reference to evaluations made by the International Agency for Research on Cancer	1993
2337	A. Malin Igra	Environmental metal exposure and growth to 10 years of age in a longitudinal mother-child cohort in rural Bangladesh	2021
2338	A. P. Sanders	Prenatal and early childhood critical windows for the association of nephrotoxic metal and metalloid mixtures with kidney function	2022

2339	Z. Li	Growth and differentiation effects of Homer3 on a leukemia cell line	2013
2340	D. Pakulska	Hazardous effects of arsine: a short review	2006
2341	V. W. Lai	Arsenic speciation in human urine: are we all the same?	2004
2342	C. Cervantes	Resistance to arsenic compounds in microorganisms	1994
2343	H. Yamauchi	Metabolism and excretion of orally and intraperitoneally administered gallium arsenide in the hamster	1986
2344	W. M. Jongen	Genotoxicity testing of arsenobetaine, the predominant form of arsenic in marine fishery products	1985
2345	D. Lukasz	Dissolution of arsenic minerals mediated by dissimilatory arsenate reducing bacteria: estimation of the physiological potential for arsenic mobilization	2014
2346	M. S. Taleshi	Synthesis and Characterization of Arsenolipids: Naturally Occurring Arsenic Compounds in Fish and Algae	2014
2347	S. Man	Anticancer drugs from traditional toxic Chinese medicines	2012
2348	Y. Huang	Induction of cytoplasmic accumulation of p53: a mechanism for low levels of arsenic exposure to predispose cells for malignant transformation	2008
2349	T. Phenrat	A SEM and X-ray study for investigation of solidified/stabilized arsenic-iron hydroxide sludge	2005
2350	O. M. Faroon	A review of the carcinogenicity of chemicals most frequently found at National Priorities List sites	1994
2351	X. C. Le	Human urinary arsenic excretion after one-time ingestion of seaweed, crab, and shrimp	1994
2352	J. Xue	Studies on the Inhibitory Effects of Nano-Hydroxyapatite-Loaded As_2O_3 on Hepatoma Cells	2020
2353	S. Hirano	Biotransformation of arsenic and toxicological implication of arsenic metabolites	2020
2354	E. G. Rodrigues	Maternal-infant biomarkers of prenatal exposure to arsenic and manganese	2015
2355	K. Agay-Shay	Exposure to Endocrine-Disrupting Chemicals during Pregnancy and Weight at 7 Years of Age: A Multi-pollutant Approach	2015
2356	S. Keller	Traditional beliefs part of people's lives	1996

2357	R. Y. Wang	Speciation analysis of arsenic and selenium compounds in environmental and biological samples by ion chromatography-inductively coupled plasma dynamic reaction cell mass spectrometer	2007
2358	M. Moradzadeh	Cuscuta campestris induces apoptosis by increasing reactive oxygen species generation in human leukemic cells	2018
2359	H. Ma	Insights into the All-trans-Retinoic Acid and Arsenic Trioxide Combination Treatment for Acute Promyelocytic Leukemia: A Meta-Analysis	2015
2360	N. Ma	Protection effect of taurine on nitrosative stress in the mice brain with chronic exposure to arsenic	2010
2361	X. Li	Long-term survival analysis in 170 cases of acute promyelocytic leukemia	2006
2362	V. Foà	The speciation of the chemical forms of arsenic in the biological monitoring of exposure to inorganic arsenic	1984
2363	J. Y. Cabon	Effects of various salts on the determination of arsenic by graphite furnace atomic absorption spectrometry. Direct determination in seawater	2000
2364	A. M. Aquino	Arsenic exposure during prepuberty alters prostate maturation in pubescent rats	2019
2365	T. Hoffmann	Arsenobetaine: an ecophysiologically important organoarsenical confers cytoprotection against osmotic stress and growth temperature extremes	2018
2366	S. C. A. Mana	The fate and transport of arsenic species in the aquatic ecosystem: a case study on Bestari Jaya, Peninsular Malaysia	2017
2367	S. E. Baltazar	Surface rearrangement of nanoscale zerovalent iron: the role of pH and its implications in the kinetics of arsenate sorption	2014
2368	S. Retsas	Medicinal use of earths and minerals from Hippocrates to Sir Hans Sloane and beyond	2012
2369	J. L. Pittman	Experimental studies of electroosmotic flow dynamics during sample stacking for capillary electrophoresis	2003
2370	R. Gornati	Arsenic toxicity and HSP70 expression in Xenopus laevis embryos	2002
2371	R. Mukhopadhyay	Microbial arsenic: from geocycles to genes and enzymes	2002
2372	P. Varela	An immunological strategy To monitor In situ the phosphate starvation state in thiobacillus ferrooxidans	1998
2373	G. Zhu	Novel treatment of acute promyelocytic leukemia: As_2O_3, retinoic acid and retinoid pharmacology	2013
2374	S. Huang	JWA, a novel signaling molecule, involved in all-trans retinoic acid induced differentiation of HL-60 cells	2006

2375	B. W. Gu	Feasibility and clinical significance of real-time quantitative RT-PCR assay of PML-RARalpha fusion transcript in patients with acute promyelocytic leukemia	2001
2376	T. Hideshima	NF-kappa B as a therapeutic target in multiple myeloma	2002
2377	H. L. Zhu	The expression of Fas, FasL and Bcl-2 on RMA cells during the process of apoptosis induced by chemotherapeutic drugs	2002
2378	U. K. Chowdhury	Pattern of excretion of arsenic compounds [arsenite, arsenate, MMA(V), DMA(V)] in urine of children compared to adults from an arsenic exposed area in Bangladesh	2003
2379	S. J. Giovannoni	A Parasitic Arsenic Cycle That Shuttles Energy from Phytoplankton to Heterotrophic Bacterioplankton	2019
2380	P. Singh	Benefits of Alcohol on Arsenic Toxicity in Rats	2017
2381	R. T. Ferreira	E4-Ubiquitin ligase Ufd2 stabilizes Yap8 and modulates arsenic stress responses independent of the U-box motif	2015
2382	I. López-García	Rapid screening of water soluble arsenic species in edible oils using dispersive liquid-liquid microextraction	2015
2383	M. C. Kruger	Bacterial metabolism of environmental arsenic--mechanisms and biotechnological applications	2013
2384	C. Huang	Transactivation of RARE and GRE in the cellular response to arsenic	2001
2385	J. Dich	Pesticides and cancer	1997
2386	L. Romeo	Acute arsine intoxication as a consequence of metal burnishing operations	1997
2387	S. Mirjalili	Effects of co-administration of arsenic trioxide and Schiff base oxovanadium complex on the induction of apoptosis in acute promyelocytic leukemia cells	2021
2388	W. Sun	Metabolism of Reactive Oxygen Species in Osteosarcoma and Potential Treatment Applications	2019
2389	X. Xiao	pH-triggered sustained release of arsenic trioxide by polyacrylic acid capped mesoporous silica nanoparticles for solid tumor treatment in vitro and in vivo	2016
2390	H. Gill	Oral arsenic trioxide-based regimen as salvage treatment for relapsed or refractory mantle cell lymphoma	2014
2391	M. Primon	Cathepsin L silencing enhances arsenic trioxide mediated in vitro cytotoxicity and apoptosis in glioblastoma U87MG spheroids	2013
2392	T. Zhou	Evidence for Vpr-dependent HIV-1 replication in human CD4+ CEM.NKR T-cells	2012

2393	J. Z. Wu	Evaluation of the in vitro activity and in vivo bioavailability of realgar nanoparticles prepared by cryo-grinding	2006
2394	Z. L. Liu	Improved RT-PCR for detection of PML/RARalpha fusion gene in rapid diagnosis of acute promyelocytic leukemia	2003
2395	X. Thomas	Arsenic: a beneficial therapeutic poison - a historical overview	2009
2396	Y. L. Kwong	Arsenic trioxide in the treatment of haematological malignancies	2004
2397	C. D. Kamat	Role of HIF signaling on tumorigenesis in response to chronic low-dose arsenic administration	2005
2398	P. Hantson	Sister chromatid exchanges in human peripheral blood lymphocytes after ingestion of high doses of arsenicals	1996
2399	L. Freije-Carrelo	Instrumental Setup for Simultaneous Total and Speciation Analysis of Volatile Arsenic Compounds in Gas and Liquefied Gas Samples	2017
2400	Y. L. Liu	Absorption and metabolism mechanisms of inorganic arsenic in plants: a review	2012
2401	A. Pyszel	Effect of metals, benzene, pesticides and ethylene oxide on the haematopoietic system	2005
2402	D. Bornemann	Deoxygenative Fluorination of Phosphine Oxides: A General Route to Fluorinated Organophosphorus(V) Compounds and Beyond	2020
2403	G. Colotti	Metal- and metalloid-containing drugs for the treatment of trypanosomatid diseases	2018
2404	Y. Sun	Thiolated arsenicals in arsenic metabolism: Occurrence, formation, and biological implications	2016
2405	H. Zhang	Application of high performance liquid chromatography and hyphenated techniques in analysis of arsenic species	2007
2406	L. S. Milstein	Selection of a suitable mobile phase for the speciation of four arsenic compounds in drinking water samples using ion-exchange chromatography coupled to inductively coupled plasma mass spectrometry	2002
2407	L. Polissar	Pathways of human exposure to arsenic in a community surrounding a copper smelter	1990
2408	C. B. Jiang	Fetal exposure to environmental neurotoxins in Taiwan	2014
2409	D. B. Galloway	An outbreak of gonadal hypoplasia in a sheep flock: clinical, pathological and endocrinological features, and aetiological studies	1992
2410	Z. Lengyel	Changes in the central nervous activity of rats treated with dimethoate in combination with other neurotoxicants in different phases of ontogenesis	2005

2411	I. Han	Characterization of urinary concentrations of heavy metals among socioeconomically disadvantaged black pregnant women	2020
2412	S. E. Moore	Early-life nutritional and environmental determinants of thymic size in infants born in rural Bangladesh	2009
2413	J. L. Corlett	Mineral content of culinary and medicinal plants cultivated by Hmong refugees living in Sacramento, California	2002
2414	N. S. Alamolhodaei	Arsenic cardiotoxicity: An overview	2015
2415	H. Guan	Maternal and fetal exposure to four carcinogenic environmental metals	2010
2416	Y. Moroishi	Infant infections, respiratory symptoms, and allergy in relation to timing of rice cereal introduction in a United States cohort	2022
2417	C. Kim	Maternal Metals/Metalloid Blood Levels Are Associated With Lipidomic Profiles Among Pregnant Women in Puerto Rico	2021
2418	A. Stojsavljević	Levels of non-essential trace metals and their impact on placental health: a review	2022
2419	J. Forns	Exposure to metals during pregnancy and neuropsychological development at the age of 4 years	2014
2420	S. McDermott	Probability of intellectual disability is associated with soil concentrations of arsenic and lead	2011
2421	Y. Sun	The thioredoxin system mediates redox-induced cell death in human colon cancer cells: implications for the mechanism of action of anticancer agents	2008
2422	O. Cantoni	Mitochondrial ROS, ER Stress, and Nrf2 Crosstalk in the Regulation of Mitochondrial Apoptosis Induced by Arsenite	2022
2423	B. C. Menezes MĂ	Arsenic in Sediments, Soil and Plants in a Remediated Area of the Iron Quadrangle, Brazil, and its Accumulation and Biotransformation in Eleocharis geniculata	2020
2424	A. D. Páez-Espino	ArsH protects Pseudomonas putida from oxidative damage caused by exposure to arsenic	2020
2425	M. Kumar	Medication related osteonecrosis of jaw in a leukemia patient undergoing systemic arsenic trioxide therapy: A rare case report	2019
2426	A. Sarkar	The global menace of arsenic and its conventional remediation - A critical review	2016
2427	S. Chandra	Arsenic Uptake and Accumulation in Okra (Abelmoschus esculentus) as Affected by Different Arsenical Speciation	2016
2428	B. Hernández-Castro	Effect of arsenic on regulatory T cells	2009

2429	Z. Slejkovec	Arsenic speciation patterns in freshwater fish	2004
2430	T. Mrak	Uptake and biotransformation of arsenate in the lichen Hypogymnia physodes (L.) Nyl	2008
2431	E. Wildfang	Enzymatic methylation of arsenic compounds. IX. Liver arsenite methyltransferase and arsenate reductase activities in primates	2001
2432	K. Saeki	Arsenic accumulation in three species of sea turtles	2000
2433	K. Kitamura	Toxic effects of arsenic (As3+) and other metal ions on acute promyelocytic leukemia cells	1997
2434	R. A. Zakharyan	Enzymatic Methylation of Arsenic Compounds	1996
2435	M. Buratti	Significance of arsenic metabolic forms in urine. Part I: Chemical speciation	1984
2436	M. Börzsönyi	Agriculturally-related carcinogenic risk	1984
2437	A. Léonard	Carcinogenicity, teratogenicity and mutagenicity of arsenic	1980
2438	Z. Zhao	Arsenic removal from copper slag matrix by high temperature sulfide-reduction-volatilization	2021
2439	M. Sharma	Chronic toxicity study of Sameera Pannaga Rasa in Charle's foster albino rats	2020
2440	J. Kretzschmar	Phenylarsonic acid-DMPS redox reaction and conjugation investigated by NMR spectroscopy and X-ray diffraction	2022
2441	L. L. Song	Research progress in mineral Chinese medicine realgar	2019
2442	N. Höher	Toxic effects of chemical warfare agent mixtures on the mussel Mytilus trossulus in the Baltic Sea: A laboratory exposure study	2019
2443	V. Chatain	Effect of indigenous bacterial activity on arsenic mobilization under anaerobic conditions	2005
2444	A. Hartwig	Modulation of DNA repair processes by arsenic and selenium compounds	2003
2445	A. J. Bednar	Photodegradation of roxarsone in poultry litter leachates	2003
2446	H. Sakamoto	Determination of trace amounts of total arsenic in environmental samples by hydride generation flow injection-AAS using a mixed acid as a pretreatment agent	2001

2447	T. Gebel	Arsenic and antimony: comparative approach on mechanistic toxicology	1997
2448	R. O. Sterling	Reaction of arsenic vapor species with fly ash compounds: kinetics and speciation of the reaction with calcium silicates	2003
2449	C. Yedjou	Basic mechanisms of arsenic trioxide (ATO)-induced apoptosis in human leukemia (HL-60) cells	2010
2450	F. Trabelsi	Cytotoxicity and genotoxicity effects of arsenic trioxide on SQ20B human laryngeal carcinoma cells	2017
2451	K. H. Antman	Introduction: the history of arsenic trioxide in cancer therapy	2001
2452	C. J. Sandroff	GaAs Clusters in the Quantum Size Regime: Growth on High Surface Area Silica by Molecular Beam Epitaxy	1989
2453	S. N. Ugwu	Optimization of iron-enhanced anaerobic digestion of agro-wastes for biomethane production and phosphate release	2022
2454	D. Yong	Detecting total toxicity in water using a mediated biosensor system with flow injection	2015
2455	Y. Wang	Simultaneously multi-parameter determination of hematonosis cell apoptosis by two-photon and confocal laser scanning microscopy	2004
2456	C. E. Chiang	Prolongation of cardiac repolarization by arsenic trioxide	2002
2457	N. Rascovan	Metagenomic study of red biofilms from Diamante Lake reveals ancient arsenic bioenergetics in haloarchaea	2016
2458	J. Hu	Arsenic in the treatment of newly diagnosed acute promyelocytic leukemia: current status and future research direction	2011
2459	A. Bollini	Arsenic intoxication, a hemorheologic view	2010
2460	R. Sierra-Alvarez	Methanogenic inhibition by roxarsone (4-hydroxy-3-nitrophenylarsonic acid) and related aromatic arsenic compounds	2010
2461	A. Hernández	Genetic variations associated with interindividual sensitivity in the response to arsenic exposure	2008
2462	T. Itakura	Arsenic recovery from water containing arsenite and arsenate ions by hydrothermal mineralization	2007
2463	R. A. Zakharyan	Enzymatic methylation of arsenic compounds. III. The marmoset and tamarin, but not the rhesus, monkeys are deficient in methyltransferases that methylate inorganic arsenic	1996
2464	D. Gandhi	Non-malignant respiratory illness associated with exposure to arsenic compounds in the environment	2022

2465	N. Bansal	Arsenic and cadmium resistance in environmental isolates of Yersinia enterocolitica and Yersinia intermedia	2000
2466	B. Do	On-line reversed-phase liquid chromatography hydride generation emission spectrometry: speciation of arsenic in urine of patients intravenously treated with As2O3	2000
2467	B. Deng	Interface of on line coupling capillary electrophoresis with hydride generation electrothermal atomic absorption spectrometry and its application to arsenic speciation in sediment	2013
2468	X. Li	Arsenic impairs embryo development via down-regulating Dvr1 expression in zebrafish	2012
2469	S. Prikler	Improving detection power in trace analysis using wavelet transform	2012
2470	L. Di Giampaolo	In vitro effects of different arsenic compounds on PBMC (preliminary study)	2004
2471	K. Ramanathan	Ascorbic acid and alpha-tocopherol as potent modulators on arsenic induced toxicity in mitochondria	2003
2472	J. Aono	Activation of Nrf2 and accumulation of ubiquitinated A170 by arsenic in osteoblasts	2003
2473	T. S. Wang	Endonuclease III, formamidopyrimidine-DNA glycosylase, and proteinase K additively enhance arsenic-induced DNA strand breaks in human cells	2002
2474	S. Nagendra	Leukemias resembling acute promyelocytic leukemia, microgranular variant	2002
2475	R. A. Zakharyan	Enzymatic methylation of arsenic compounds. VII. Monomethylarsonous acid (MMAIII) is the substrate for MMA methyltransferase of rabbit liver and human hepatocytes	1999
2476	E. Kashiwada	Aneuploidy induced by dimethylarsinic acid in mouse bone marrow cells	1998
2477	T. Hirohata	Incidence and etiology of lung cancer in the Pacific Basin	1979
2478	Y. Sun	Surfactantless synthesis of silver nanoplates and their application in SERS	2007
2479	J. Zhao	Valency distributions and geochemical fractions of arsenic and antimony in non-ferrous smelting soils with varying particle sizes	2022
2480	V. Mbabajende	[Historical survey of modern reversible contraceptive methods]	1986
2481	D. Kumar	In vitro elicitation, isolation, and characterization of conessine biomolecule from Holarrhena antidysenterica (L.) Wall. callus and its larvicidal activity against malaria vector, Anopheles stephensi Liston	2018
2482	Y. X. Wáng	Top five medical innovations in China mainland since Xinhai revolution [1911]: results of AME survey-002	2015

2483	M. A. García-Sevillano	Analysis of the biological response of mouse liver (Mus musculus) exposed to As2O3 based on integrated -omics approaches	2013
2484	N. K. Kortei	Elemental minerals and microbial compositions as well as knowledge and perceptions regarding kaolin (clay) consumption by pregnant women in the Ho municipality of Ghana	2019
2485	Y. Zhang	Contribution of trace element exposure to gestational diabetes mellitus through disturbing the gut microbiome	2021
2486	M. Zhang	Individual and mixtures of metal exposures in associations with biomarkers of oxidative stress and global DNA methylation among pregnant women	2022
2487	H. Huang	Child marriage, maternal serum metal exposure, and risk of preterm birth in rural Bangladesh: evidence from mediation analysis	2021
2488	P. Ashrap	Predictors of urinary and blood Metal(loid) concentrations among pregnant women in Northern Puerto Rico	2020
2489	I. Y. Hwang	Toxoplasma gondii infection inhibits the mitochondrial apoptosis through induction of Bcl-2 and HSP70	2010
2490	S. Hirano	The accumulation and toxicity of methylated arsenicals in endothelial cells: important roles of thiol compounds	2004
2491	M. A. Jakupec	Gallium and other main group metal compounds as antitumor agents	2004
2492	L. Cui	Downregulation of B7-H4 in the MHCC97-H hepatocellular carcinoma cell line by arsenic trioxide	2016
2493	J. Ventura-Lima	Toxicological responses in Laeonereis acuta (annelida, polychaeta) after arsenic exposure	2007
2494	W. L. Xu	Experimental study on the role of VEGF autocrine loop in K562 leukemia cells	2007
2495	L. Xin	A survival study and prognostic factors analysis on acute promyelocytic leukemia at a single center	2007
2496	W. G. Mao	Expressions of JWA protein and heat stress protein 70 induced by cell differentiation inducers combined with heat stress in K562 cells	2004
2497	S. S. Kim	Urinary trace metals in association with fetal ultrasound measures during pregnancy	2020
2498	S. Ling	Metformin inhibits proliferation and enhances chemosensitivity of intrahepatic cholangiocarcinoma cell lines	2014
2499	Z. X. Jiao	As2 O3 combined with leflunomide prolongs heart xenograft survival via suppressing the response of Th1, Th2, and B cells in a rat model	2016
2500	J. L. Sun	Arsenite promotes intestinal tumor cell proliferation and invasion by stimulating epithelial-to-mesenchymal transition	2014

2501	L. A. Hansen	Retinoids in chemoprevention and differentiation therapy	2000
2502	H. Han	Inhibitory effects of CaO/Fe2O3 on arsenic emission during sewage sludge pyrolysis	2016
2503	S. Roy	Arsenic-induced histopathology and synthesis of stress proteins in liver and kidney of Channa punctatus	2006
2504	E. M. Rego	Analysis of the molecular genetics of acute promyelocytic leukemia in mouse models	2001
2505	G. Cassone	Stability of hydrolytic arsenic species in aqueous solutions: As(3+)vs. As(5)	2018
2506	D. F. Romagnolo	Epigenetics of breast cancer: Modifying role of environmental and bioactive food compounds	2016
2507	C. M. GuŢu	Comparative evaluation of short-term toxicity of inorganic arsenic compounds on Artemia salina	2015
2508	M. Czaplicka	Application of advanced oxidation processes for cleaning of industrial water generated in wet dedusting of shaft furnace gases	2013
2509	A. Pell	Occurrence of arsenic species in algae and freshwater plants of an extreme arid region in northern Chile, the Loa River Basin	2013
2510	C. M. Liang	Trace element profiles in pregnant women's sera and umbilical cord sera and influencing factors: Repeated measurements	2019
2511	R. J. Leke	Regional and geographical variations in infertility: effects of environmental, cultural, and socioeconomic factors	1993
2512	C. G. Howe	Prenatal Metal Mixtures and Birth Weight for Gestational Age in a Predominately Lower-Income Hispanic Pregnancy Cohort in Los Angeles	2020
2513	A. Nawrocka	Simple and reliable determination of total arsenic and its species in seafood by ICP-MS and HPLC-ICP-MS	2022
2514	A. Situm	ATR-FTIR and Flow Microcalorimetry Studies on the Initial Binding Kinetics of Arsenicals at the Organic-Hematite Interface	2017
2515	Y. Zhang	Arsenic Primes Human Bone Marrow CD34+ Cells for Erythroid Differentiation	2015
2516	F. Kucuksezgin	Total and inorganic arsenic levels in some marine organisms from Izmir Bay (Eastern Aegean Sea): a risk assessment	2014
2517	J. X. Liu	Arsenic compounds: revived ancient remedies in the fight against human malignancies	2012
2518	T. Llorente-Mirandes	Determination of water-soluble arsenic compounds in commercial edible seaweed by LC-ICPMS	2011

2519	P. Pinel-Raffaitin	Distribution and fate of inorganic and organic arsenic species in landfill leachates and biogases	2007
2520	L. H. Yih	Changes in gene expression profiles of human fibroblasts in response to sodium arsenite treatment	2002
2521	G. R. Cornelis	The virulence plasmid of Yersinia, an antihost genome	1998
2522	E. Chaineau	Embryotoxic effects of sodium arsenite and sodium arsenate on mouse embryos in culture	1990
2523	A. Trdin	Trace elements and APOE polymorphisms in pregnant women and their new-borns	2020
2524	L. Jin	Concentrations of selected heavy metals in maternal blood and associated factors in rural areas in Shanxi Province, China	2014
2525	H. Yang	Sol-gel synthesis and photoluminescence of III-V semiconductor InAs nanocrystals embedded in silica glasses	2005
2526	M. Kito	Antitumor effect of arsenic trioxide in murine xenograft model	2003
2527	J. L. Griffin	High-resolution magic angle spinning 1H-NMR spectroscopy studies on the renal biochemistry in the bank vole (Clethrionomys glareolus) and the effects of arsenic (As3+) toxicity	2001
2528	R. J. Griffin	Use of arsenic trioxide as an antivascular and thermosensitizing agent in solid tumors	2000
2529	X. Huang	Study on the relationship between calcium-induced calcium release from mitochondria and PTP opening	2000
2530	L. Tulić	Toxic Metal and Trace Element Concentrations in Blood and Outcome of In Vitro Fertilization in Women	2019
2531	J. L. Yang	Target induced aggregation of Ce(III)-based coordination polymer nanoparticles for fluorimetric detection of As(III)	2018
2532	P. Ning	Removal of mercury (II), elemental mercury and arsenic from simulated flue gas by ammonium sulphide	2015
2533	T. Marino	Arsenic removal by liquid membranes	2015
2534	J. Fujihara	Association of XRCC1 polymorphisms with arsenic methylation	2016
2535	L. G. Konshina	Assessment of the quality of drinking water in the industrial city and risk for public health	2014
2536	R. A. Glabonjat	Quantification of arsenolipids in the certified reference material NMIJ 7405-a (Hijiki) using HPLC/mass spectrometry after chemical derivatization	2014

2537	S. Meyer	In vitro toxicological characterisation of three arsenic-containing hydrocarbons	2014
2538	N. Rivera-Reyna	Photocatalytical removal of inorganic and organic arsenic species from aqueous solution using zinc oxide semiconductor	2013
2539	J. Scott	Dual action of phenylarsine oxide on the glucose transport activity of GLUT1	2009
2540	T. Jiang	Nrf2 protects against As(III)-induced damage in mouse liver and bladder	2009
2541	A. L. Välimaa	Real-time Monitoring of Non-specific Toxicity Using a Saccharomyces cerevisiae Reporter System	2008
2542	P. J. Dilda	Arsenical-based cancer drugs	2007
2543	A. Vahidnia	Arsenic-induced toxicity: effect on protein composition in sciatic nerve	2006
2544	M. Rizki	Metabolism of arsenic in Drosophila melanogaster and the genotoxicity of dimethylarsinic acid in the Drosophila wing spot test	2006
2545	P. Bobrowicz	Isolation of three contiguous genes, ACR1, ACR2 and ACR3, involved in resistance to arsenic compounds in the yeast Saccharomyces cerevisiae	1997
2546	O. Jimenez de Blas	Determination and speciation of arsenic in human urine by ion-exchange chromatography/flow injection analysis with hydride generation/atomic absorption spectroscopy	1994
2547	J. Fu	A Novel Mobile Element ICERspD18B in Rheinheimera sp. D18 Contributes to Antibiotic and Arsenic Resistance	2020
2548	S. C. Chen	The Great Oxidation Event expanded the genetic repertoire of arsenic metabolism and cycling	2020
2549	A. Hata	Metabolism of 3-[5'-deoxy-5'-(dimethylarsinoyl)-β-ribofuranosyloxy]-2-hydroxypropylene glycol in an artificial digestive system	2019
2550	I. Ben Fekih	Distribution of Arsenic Resistance Genes in Prokaryotes	2018
2551	M. Liu	The Migration and Transformation of Heavy Metals in Sewage Sludge during Hydrothermal Carbonization Combined with Combustion	2018
2552	K. Lewchalermvong	Arsenic Speciation and Accumulation in Selected Organs after Oral Administration of Rice Extracts in Wistar Rats	2018
2553	C. Lou	Distribution patterns and possible influencing factors of As speciation in ornithogenic sediments from the Ross Sea region, East Antarctica	2016
2554	E. J. Tokar	Metal ions in human cancer development	2011

2555	A. Adamescu	Insights into the surface complexation of dimethylarsinic acid on iron (oxyhydr)oxides from ATR-FTIR studies and quantum chemical calculations	2010
2556	M. Suwalsky	Monomethylarsonate (MMAv) exerts stronger effects than arsenate on the structure and thermotropic properties of phospholipids bilayers	2008
2557	R. Raml	Improved chromatographic separation of thio-arsenic compounds by reversed-phase high performance liquid chromatography-inductively coupled plasma mass spectrometry	2006
2558	K. Kanaki	Precursor ion scanning for the non-targeted detection of individual arsenosugars in extracts of marine organisms	2006
2559	H. Sun	Treatment of groundwater polluted by arsenic compounds by zero valent iron	2006
2560	A. M. Florea	Intracellular calcium disturbances induced by arsenic and its methylated derivatives in relation to genomic damage and apoptosis induction	2005
2561	J. Fujihara	Arsenic accumulation in livers of pinnipeds, seabirds and sea turtles: subcellular distribution and interaction between arsenobetaine and glycine betaine	2003
2562	M. Vilanó	Determination of arsenic in seafood by focused microwave digestion and hydride generation-atomic fluorescence detection	2001
2563	M. Wilhelm	Duplicate study on the dietary intake of some metals/metalloids by children in Germany. I. Arsenic and mercury	1995
2564	M. H. Draper	Analysis of nickel refinery dusts	1994
2565	H. Yamauchi	Metabolism and excretion of orally administrated arsenic trioxide in the hamster	1985
2566	X. Zhang	HIV-1 Vpr increases Env expression by preventing Env from endoplasmic reticulum-associated protein degradation (ERAD)	2016
2567	S. Sadiq	Metal toxicity at the synapse: presynaptic, postsynaptic, and long-term effects	2012
2568	E. K. Noh	Gefitinib enhances arsenic trioxide (AS2O3)-induced differentiation of acute promyelocytic leukemia cell line	2010
2569	J. H. Son	Crystal engineering through face interactions between tetrahedral and octahedral building blocks: crystal structure of [epsilon-Al13O4(OH)24(H2O)12]2[V2W4O19]3(OH)2).27H2O	2004
2570	P. P. Pandolfi	Histone deacetylases and transcriptional therapy with their inhibitors	2001
2571	S. Torii	Human Daxx regulates Fas-induced apoptosis from nuclear PML oncogenic domains (PODs)	1999
2572	G. Hu	Protective effects of sodium selenite and selenomethionine on genotoxicity to human peripheral lymphocytes induced by arsenic	1996

2573	H. Mahandra	Leaching characteristics and stability assessment of sequestered arsenic in flue dust based glass	2021
2574	J. Zhang	Double-Sided Personality: Effects of Arsenic Trioxide on Inflammation	2018
2575	D. Yang	Ligand-directed conformation of inorganic-organic molecular capsule and cage	2014
2576	H. El Hajj	Therapy-induced selective loss of leukemia-initiating activity in murine adult T cell leukemia	2010
2577	C. Gabbiani	Outstanding plasmodicidal properties within a small panel of metallic compounds: Hints for the development of new metal-based antimalarials	2009
2578	X. Qi	Expression of Dlk1 gene in myelodysplastic syndrome determined by microarray, and its effects on leukemia cells	2008
2579	A. Holcman	Arsenic residues in eggs from laying hens fed with a diet containing arsenic (III) oxide	1997
2580	Đ. Miodragović	Beyond Cisplatin: Combination Therapy with Arsenic Trioxide	2019
2581	X. Chen	A novel NPM1-RARG-NPM1 chimeric fusion in acute myeloid leukaemia resembling acute promyelocytic leukaemia but resistant to all-trans retinoic acid and arsenic trioxide	2019
2582	K. Li	TRIB3 Promotes APL Progression through Stabilization of the Oncoprotein PML-RARα and Inhibition of p53-Mediated Senescence	2017
2583	M. Malbec	Murine double minute 2 as a modulator of retroviral restrictions mediated by TRIM5alpha	2010
2584	C. Vandecasteele	Solidification/stabilisation of arsenic bearing fly ash from the metallurgical industry. Immobilisation mechanism of arsenic	2002
2585	I. Park	A novel arsenic immobilization strategy via a two-step process: Arsenic concentration from dilute solution using schwertmannite and immobilization in Ca-Fe-AsO(4) compounds	2021
2586	A. Abdelrady	Characterisation of the impact of dissolved organic matter on iron, manganese, and arsenic mobilisation during bank filtration	2020
2587	X. Wang	Exposure to multiple metals in early pregnancy and gestational diabetes mellitus: A prospective cohort study	2020
2588	E. C. Nyanza	Effects of prenatal exposure and co-exposure to metallic or metalloid elements on early infant neurodevelopmental outcomes in areas with small-scale gold mining activities in Northern Tanzania	2021
2589	X. K. Zhang	Effect of arsenic trioxide and 5-aza-2'-deoxycytidine on SHP-1, JAK3, TYK2 gene expression in K562 cells	2014
2590	H. Y. Cheng	Application of ATP based bioluminescence tumor chemosensitivity assay in the chemotherapy of pediatric solid tumor	2009

2591	J. Zhang	Preparation of a nanosized as(2)o(3)/mn(0.5)zn(0.5)fe(2)o(4) complex and its anti-tumor effect on hepatocellular carcinoma cells	2009
2592	F. Wu	A study of tissue factor expression and hemostatic molecular markers in patients with acute promyelocytic leukemia	2001
2593	C. Infante-Rivard	Drinking water contaminants and childhood leukemia	2001
2594	A. Zelenik Pevec	As(2)O (3) oxidation by vitamin C: cell culture studies	2012
2595	V. Chat	Association between number of children and carotid intima-media thickness in Bangladesh	2018
2596	A. Kupsco	Prenatal Metal Concentrations and Childhood Cardiometabolic Risk Using Bayesian Kernel Machine Regression to Assess Mixture and Interaction Effects	2019
2597	J. R. Camurati	On-line speciation analysis of arsenic compounds in commercial edible seaweed by HPLC-UV-thermo-oxidation-HG-AFS	2021
2598	L. Yao	Soil attribute regulates assimilation of roxarsone metabolites by rice (Oryza sativa L.)	2019
2599	M. M. Nearing	Arsenic speciation in edible mushrooms	2014
2600	P. R. Gentry	The impact of recent advances in research on arsenic cancer risk assessment	2014
2601	R. Raml	Thio-dimethylarsinate is a common metabolite in urine samples from arsenic-exposed women in Bangladesh	2007
2602	A. Fukuda	Development of analysis of drugs and toxic substances that can play an immediate role in emergency medical service: what is to be analyzed?	2005
2603	A. Geiszinger	Arsenic biotransformation by the brown macroalga, Fucus serratus	2001
2604	J. Cai	Use of a luminescent bacterial biosensor for biomonitoring and characterization of arsenic toxicity of chromated copper arsenate (CCA)	1997
2605	J. G. Farmer	Assessment of occupational exposure to inorganic arsenic based on urinary concentrations and speciation of arsenic	1990
2606	J. Guo	Associations of blood metal exposure with thyroid hormones in Chinese pregnant women: A cross-sectional study	2018
2607	E. M. Ostrea	Prevalence of fetal exposure to environmental toxins as determined by meconium analysis	2002
2608	E. Byeon	Interspecific biotransformation and detoxification of arsenic compounds in marine rotifer and copepod	2020

2609	P. Picco	Arsenic species uptake and translocation in Elodea canadensis	2019
2610	M. A. Sabur	Temperature-dependent infrared and calorimetric studies on arsenicals adsorption from solution to hematite nanoparticles	2015
2611	A. Raab	Comprehensive analysis of lipophilic arsenic species in a brown alga (Saccharina latissima)	2013
2612	J. Tofan-Lazar	Kinetic ATR-FTIR studies on phosphate adsorption on iron (oxyhydr)oxides in the absence and presence of surface arsenic: molecular-level insights into the ligand exchange mechanism	2012
2613	K. Banerjee	Kinetic and thermodynamic aspects of adsorption of arsenic onto granular ferric hydroxide (GFH)	2008
2614	N. Suzuki	Theoretical calculations and reaction analysis on the interaction of pentavalent thioarsenicals with biorelevant thiol compounds	2008
2615	P. G. Smith	Uptake, transport and transformation of arsenate in radishes (Raphanus sativus)	2008
2616	A. M. Hays	Arsenic and cigarette smoke synergistically increase DNA oxidation in the lung	2006
2617	D. Fattorini	Arsenic speciation in tissues of the Mediterranean polychaete Sabella spallanzanii	2004
2618	U. Kohlmeyer	Benefits of high resolution IC-ICP-MS for the routine analysis of inorganic and organic arsenic species in food products of marine and terrestrial origin	2003
2619	K. Ramanathan	Protective role of ascorbic acid and alpha-tocopherol on arsenic-induced microsomal dysfunctions	2003
2620	K. Francesconi	Arsenic species in an arsenic hyperaccumulating fern, Pityrogramma calomelanos: a potential phytoremediator of arsenic-contaminated soils	2002
2621	P. Kavanagh	Urinary arsenic species in Devon and Cornwall residents, UK. A pilot study	1998
2622	K. Yamanaka	Metabolic methylation is a possible genotoxicity-enhancing process of inorganic arsenics	1997
2623	O. Ademuyiwa	Arsenic-copper interaction in the kidney of the rat: influence of arsenic metabolites	1996
2624	J. Lisiewicz	Immunotoxic and hematotoxic effects of occupational exposures	1993
2625	S. Muzaffar	Mechanistic understanding of the toxic effects of arsenic and warfare arsenicals on human health and environment	2022
2626	C. D. Butts	Toxic elements in follicular fluid adversely influence the likelihood of pregnancy and live birth in women undergoing IVF	2021

2627	C. Hjelm	Pre- and postnatal environmental boron exposure and infant growth: Results from a mother-child cohort in northern Argentina	2019
2628	H. Cordero	Inorganic arsenic causes apoptosis cell death and immunotoxicity on European sea bass (Dicentrarchus labrax)	2018
2629	Y. Song	Synthesis and Cytotoxicity of N-Substituted Dibenzo[a,j]xanthene-3,11-dicarboxamide Derivatives	2017
2630	C. Pérez-Sirvent	Influence of waterborne arsenic on nutritive and potentially harmful elements in gilthead seabream (Sparus aurata)	2016
2631	F. Binet	Interaction between arsenic trioxide and human primary cells: emphasis on human cells of myeloid origin	2009
2632	E. Kim	Acute toxic effect of sodium dichromate on metabolism	1990
2633	C. E. Weir	Compressibility of Eleven Inorganic Materials	1965
2634	M. R. Karagas	Association of Rice and Rice-Product Consumption With Arsenic Exposure Early in Life	2016
2635	X. Xu	Ultrasensitive detection of four organic arsenic compounds at the same time using a five-link cardboard-based assay	2022
2636	K. Khorsandi	Characteristics of circRNA and its approach as diagnostic tool in melanoma	2021
2637	Z. Huang	Absorption and speciation of arsenic by microalgae under arsenic-copper Co-exposure	2021
2638	A. Abi	Circular RNAs: epigenetic regulators in cancerous and noncancerous skin diseases	2020
2639	E. Osdaghi	Phenotypic and Molecular-Phylogenetic Analysis Provide Novel Insights into the Diversity of Curtobacterium flaccumfaciens	2018
2640	S. Mindlin	Resistance of Permafrost and Modern Acinetobacter lwoffii Strains to Heavy Metals and Arsenic Revealed by Genome Analysis	2016
2641	E. Ricevuto	Arsenic speciation and susceptibility to oxidative stress in the fanworm Sabella spallanzanii (Gmelin) (Annelida, Sabellidae) under naturally acidified conditions: An in situ transplant experiment in a Mediterranean CO2 vent system	2016
2642	D. Kotyzová	Differential influences of various arsenic compounds on antioxidant defense system in liver and kidney of rats	2013
2643	D. Feller	Thermodynamic properties of arsenic compounds and the heat of formation of the As atom from high level electronic structure calculations	2011
2644	B. Daus	Uptake and toxicity of hexafluoroarsenate in aquatic organisms	2010

2645	L. Wang	Identification of an arsenic resistance and arsenic-sensing system in Campylobacter jejuni	2009
2646	M. K. Sengupta	Arsenic burden of cooked rice: Traditional and modern methods	2006
2647	M. M. Adel	The background state leading to arsenic contamination of Bengal basin groundwater	2005
2648	N. Larochette	Arsenite induces apoptosis via a direct effect on the mitochondrial permeability transition pore	1999
2649	W. Zheng	Choroid plexus protects cerebrospinal fluid against toxic metals	1991
2650	A. C. F. Souza	Effects of Sodium Arsenite and Arsenate in Testicular Histomorphometry and Antioxidants Enzymes Activities in Rats	2016
2651	M. Molin	Major and minor arsenic compounds accounting for the total urinary excretion of arsenic following intake of blue mussels (Mytilus edulis): a controlled human study	2012
2652	H. Zhang	Second-order modeling of arsenite transport in soils	2011
2653	S. Zheng	TiO(2) Photocatalytic Degradation of Phenylarsonic Acid	2010
2654	C. Sullivan	Disposal of water treatment wastes containing arsenic - a review	2010
2655	P. J. Peshut	Arsenic speciation in marine fish and shellfish from American Samoa	2008
2656	L. S. Milstein	Development and application of a robust speciation method for determination of six arsenic compounds present in human urine	2003
2657	W. T. Klimecki	Effects of acute and chronic arsenic exposure of human-derived keratinocytes in an In Vitro human skin equivalent system: a novel model of human arsenicism	1997
2658	G. L. Czarnecki	Arsenic-sulfur amino acid interactions in the chick	1984
2659	J. H. Graziano	The pharmacology of 2,3-dimercaptosuccinic acid and its potential use in arsenic poisoning	1978
2660	D. R. Woods	Acriflavine uptake and resistance in Serratia marcescens cells and spheroplasts	1973
2661	J. Park	Effect of slag composition on the distribution and separation behavior of arsenic between CaO-based slag and liquid copper	2022
2662	M. Kulik	Dielectric functions, chemical and atomic compositions of the near surface layers of implanted GaAs by In(+) ions	2018

2663	Z. Wang	Arsenic Trioxide and Icariin Show Synergistic Anti-leukemic Activity	2015
2664	S. Y. Oh	Chemical extraction of arsenic from contaminated soil under subcritical conditions	2011
2665	X. Song	Abrogating HSP response augments cell death induced by As2O3 in glioma cell lines	2010
2666	K. K. Yadav	Sublethal exposure of heavy metals induces micronuclei in fish, Channa punctata	2009
2667	Z. Yang	Evaluation of arsenic trioxide-loaded albumin nanoparticles as carriers: preparation and antitumor efficacy	2008
2668	F. Gao	The Susceptibility of Leukemia Cells to Arsenic Trioxide-induced Apoptosis is Determined by Cellular Reactive Oxygen Species Level	2001
2669	D. N. Veeramachaneni	Long-term effects on male reproduction of early exposure to common chemical contaminants in drinking water	2001
2670	E. Baser	Environmental Exposures in the Etiology of Abortion: Placental Toxic and Trace Element Levels	2020
2671	J. Ashley-Martin	Maternal blood metal levels and fetal markers of metabolic function	2015
2672	H. B. Röllin	Selenium Status, Its Interaction with Selected Essential and Toxic Elements, and a Possible Sex-Dependent Response In Utero, in a South African Birth Cohort	2021
2673	E. N. Parovichnikova	Russian multicenter clinical trials in acute leukemias	2019
2674	I. Alvarado-Cruz	Environmental Epigenetic Changes, as Risk Factors for the Development of Diseases in Children: A Systematic Review	2018
2675	Y. Zhou	Controlled high-quality interface of a Ti(2.5)O(3)(0 1 0)/GaAs(0 0 1) heterostructure enabled by minimized lattice mismatch and suppressed ion diffusion	2020
2676	J. Zou	Evaluation of the change in sphingolipids in the human multiple myeloma cell line U266 and gastric cancer cell line MGC-803 treated with arsenic trioxide	2015
2677	G. C. Zhang	Effects of combination therapy with all-trans retinoic acid and arsenic trioxide on acute promyelocytic leukemia	2004
2678	A. Ashok	Exposure to As-, Cd-, and Pb-mixture induces Aβ, amyloidogenic APP processing and cognitive impairments via oxidative stress-dependent neuroinflammation in young rats	2015
2679	T. J. Zimmerman	Hepatic minerals of white-tailed and mule deer in the southern Black Hills, South Dakota	2008
2680	Y. Jia	Species-specific bioaccumulation and correlated health risk of arsenic compounds in freshwater fish from a typical mine-impacted river	2018

2681	K. P. Luzhetskiy	Risk factors of lipid metabolism disorders in residents of multi-environmental exposure to cadmium and arsenic	2016
2682	M. Díaz-Somoano	The stability of arsenic and selenium compounds that were retained in limestone in a coal gasification atmosphere	2010
2683	X. B. Yin	On-line preconcentration for capillary electrophoresis-atomic fluorescence spectrometric determination of arsenic compounds	2004
2684	C. J. Brown	Assessment of effects of chromated copper arsenate (CCA)-treated timber on nontarget epibiota by investigation of fouling community development at seven European sites	2003
2685	K. Zhan	Phosphorylation of eukaryotic initiation factor 2 by heme-regulated inhibitor kinase-related protein kinases in Schizosaccharomyces pombe is important for fesistance to environmental stresses	2002
2686	A. E. Geiszinger	Biotransformation of arsenate to the tetramethylarsonium ion in the marine polychaetes Nereis diversicolor and Nereis virens	2002
2687	T. G. Rossman	Arsenite is a cocarcinogen with solar ultraviolet radiation for mouse skin: an animal model for arsenic carcinogenesis	2001
2688	W. C. Chang	Cytoprotective effect of reduced glutathione in arsenical-induced endothelial cell injury	1991
2689	J. H. Li	Mechanism of comutagenesis of sodium arsenite with n-methyl-n-nitrosourea	1989
2690	E. W. Da Costa	Variation in the toxicity of arsenic compounds to microorganisms and the suppression of the inhibitory effects by phosphate	1972
2691	C. B. Shukalek	Arsenic Triglutathione [As(GS)3] Transport by Multidrug Resistance Protein 1 (MRP1/ABCC1) Is Selectively Modified by Phosphorylation of Tyr920/Ser921 and Glycosylation of Asn19/Asn23	2016
2692	J. Fei	Effect of simulated acid rain on stability of arsenic calcium residue in residue field	2020
2693	M. Yang	Raman spectra of thiolated arsenicals with biological importance	2018
2694	T. Bikashvili	EFFECT OF ARSENIC EXPOSURE ON BEHAVIOR OF RATS OF VARIOUS AGE GROUPS	2017
2695	K. P. Kowalski	Implementation of zero-valent iron (ZVI) into drinking water supply - role of the ZVI and biological processes	2014
2696	X. Zhou	Differential binding of monomethylarsonous acid compared to arsenite and arsenic trioxide with zinc finger peptides and proteins	2014
2697	E. J. Tokar	Cancer in experimental animals exposed to arsenic and arsenic compounds	2010
2698	J. H. Huang	Emissions of inorganic and organic arsenic compounds via the leachate pathway from pretreated municipal waste materials: a landfill reactor study	2009

2699	J. L. Gómez-Ariza	Comparative study of atomic fluorescence spectroscopy and inductively coupled plasma mass spectrometry for mercury and arsenic multispeciation	2005
2700	E. Lengfelder	Treatment of relapsed acute promyelocytic leukemia	2003
2701	A. Hartwig	Interference by toxic metal ions with DNA repair processes and cell cycle control: molecular mechanisms	2002
2702	C. J. Brown	Effects of CCA (copper-chrome-arsenic) preservative treatment of wood on the settlement and recruitment of barnacles and tube building polychaete worms	2000
2703	Z. Y. Shen	Arsenic trioxide induces apoptosis of oesophageal carcinoma in vitro	1999
2704	A. A. Appleton	Prenatal exposure to neurotoxic metals is associated with increased placental glucocorticoid receptor DNA methylation	2017
2705	M. A. El-Ghiaty	Arsenic: Various species with different effects on cytochrome P450 regulation in humans	2021
2706	C. Hackethal	Total arsenic and water-soluble arsenic species in foods of the first German total diet study (BfR MEAL Study)	2021
2707	T. Ochi	Induction of Aneuploidy, Centrosome Abnormality, Multipolar Spindle, and Multipolar Division in Cultured Mammalian Cells Exposed to an Arsenic Metabolite, Dimethylarsinate	2016
2708	L. Zhang	Arsenic sulfide combined with JQ1, chemotherapy agents, or celecoxib inhibit gastric and colon cancer cell growth	2015
2709	M. H. Abnosi	Induction of Apoptosis in the Rat Bone Marrow Mesenchymal Stem Cells Following Sodium Arsenite Treatment with the Dose Lesser than that Used for Treatment of Malignant Patient	2012
2710	Y. L. Chu	Speciation analysis of arsenic compounds in edible oil by ion chromatography-inductively coupled plasma mass spectrometry	2011
2711	D. Chen	Development of a high-performance liquid chromatography method for the simultaneous quantification of four organoarsenic compounds in the feeds of swine and chicken	2011
2712	S. N. Kales	Elevated urine arsenic: un-speciated results lead to unnecessary concern and further evaluations	2006
2713	H. V. Aposhian	Oxidation and detoxification of trivalent arsenic species	2003
2714	M. Sordo	Cytotoxic and genotoxic effects of As, MMA, and DMA on leukocytes and stimulated human lymphocytes	2001
2715	X. C. Le	Determination of monomethylarsonous acid, a key arsenic methylation intermediate, in human urine	2000
2716	R. Zakharyan	Enzymatic methylation of arsenic compounds: assay, partial purification, and properties of arsenite methyltransferase and monomethylarsonic acid methyltransferase of rabbit liver	1995

2717	L. Guo	Mechanochemical activation on selective leaching of arsenic from copper smelting flue dusts	2021
2718	J. Hu	Effect of combination of all-trans retinoic acid and arsenic trioxide on apoptosis of acute promyelocytic leukemia cells	2019
2719	S. A. Amin	Determining the equation of state of amorphous solids at high pressure using optical microscopy	2012
2720	V. Ettler	Mineralogy of air-pollution-control residues from a secondary lead smelter: environmental implications	2005
2721	A. B. Jha	Carbohydrate metabolism in growing rice seedlings under arsenic toxicity	2004
2722	X. Yu	Effects of humic acid on lipid peroxidation in arsenosis prevalent areas	1999
2723	A. Szymańska-	The relationship between PNP, GSTO-1, AS3MT and ADRB3 gene polymorphisms and urinary arsenic concentration among copper smelter and refinery employers	2020
2724	S. Braeuer	A unique arsenic speciation profile in Elaphomyces spp. ("deer truffles")-trimethylarsine oxide and methylarsonous acid as significant arsenic compounds	2018
2725	E. Leese	The Investigation of Unexpected Arsenic Compounds Observed in Routine Biological Monitoring Urinary Speciation Analysis	2017
2726	K. Hagiwara	On-site quantitation of arsenic in drinking water by disk solid-phase extraction/mobile X-ray fluorescence spectrometry	2015
2727	N. S. Keller	Determination of arsenic speciation in sulfidic waters by Ion Chromatography Hydride-Generation Atomic Fluorescence Spectrometry (IC-HG-AFS)	2014
2728	Z. Shen	The contribution of ArsB to arsenic resistance in Campylobacter jejuni	2013
2729	C. W. Liu	Bioaccumulation of arsenic compounds in aquacultural clams (Meretrix lusoria) and assessment of potential carcinogenic risks to human health by ingestion	2007
2730	W. Zhang	Arsenic speciation and distribution in an arsenic hyperaccumulating plant	2002
2731	S. M. Healy	Enzymatic methylation of arsenic compounds. V. Arsenite methyltransferase activity in tissues of mice	1998
2732	S. M. Healy	Enzymatic methylation of arsenic compounds: IV. In vitro and in vivo deficiency of the methylation of arsenite and monomethylarsonic acid in the guinea pig	1997
2733	X. Wang	Identifying a critical window of maternal metal exposure for maternal and neonatal thyroid function in China: A cohort study	2020
2734	P. Drbohlav	Detection of cadmium and zinc in the blood and follicular fluid in women in the IVF and ET program	1998

2735	S. Wu	Associations of toxic and essential trace elements in serum, follicular fluid, and seminal plasma with In vitro fertilization outcomes	2020
2736	J. Ashley-Martin	Maternal and cord blood manganese (Mn) levels and birth weight: The MIREC birth cohort study	2018
2737	D. Luce	Investigation of occupational and environmental causes of respiratory cancers (ICARE): a multicenter, population-based case-control study in France	2011
2738	K. S. Chen	Tannic acid-induced apoptosis and -enhanced sensitivity to arsenic trioxide in human leukemia HL-60 cells	2009
2739	H. S. Yu	Alterations of mitogenic responses of mononuclear cells by arsenic in arsenical skin cancers	1992
2740	Y. Y. Zhu	Anti-apoptosis effect of VEGF on the human chronic myelocytic leukemia cell line K562	2005
2741	E. Amaya	Placental concentrations of heavy metals in a mother-child cohort	2013
2742	K. Nohara	Augmenting effects of gestational arsenite exposure of C3H mice on the hepatic tumors of the F_2 male offspring via the F_1 male offspring	2016
2743	M. Yuan	Arsenic-induced autophagy regulates apoptosis in AML-12 cells	2021
2744	Y. Zhao	A novel mechanism of inhibiting in-stent restenosis with arsenic trioxide drug-eluting stent: Enhancing contractile phenotype of vascular smooth muscle cells via YAP pathway	2021
2745	Q. Zhang	Taurine rescues the arsenic-induced injury in the pancreas of rat offsprings and in the INS-1 cells	2019
2746	C. Li	Antileukemic activity of an arsenomolybdate in the human HL-60 and U937 leukemia cells	2017
2747	J. Du	MicroRNA-451 regulates stemness of side population cells via PI3K/Akt/mTOR signaling pathway in multiple myeloma	2015
2748	O. Sharaf el dein	Increased expression of VDAC1 sensitizes carcinoma cells to apoptosis induced by DNA cross-linking agents	2012
2749	J. C. Martins	Oxygen consumption by Daphnia magna Straus as a marker of chemical stress in the aquatic environment	2007
2750	O. A. Adebambo	Toxicological responses of environmental mixtures: Environmental metal mixtures display synergistic induction of metal-responsive and oxidative stress genes in placental cells	2015
2751	B. Holmdahl	Glimpses from the history of abortion	1992
2752	P. Kulawik	Heavy metal contamination, microbiological spoilage and biogenic amine content in sushi available on the Polish market	2018

2753	J. G. Heller	New views on the hypothesis of respiratory cancer risk from soluble nickel exposure; and reconsideration of this risk's historical sources in nickel refineries	2009
2754	B. Yuan	Multidrug resistance-associated protein 4 is a determinant of arsenite resistance	2016
2755	E. Lengfelder	Treatment concepts of acute promyelocytic leukemia	2005
2756	P. G. Smith	X-ray absorption near-edge structure analysis of arsenic species for application to biological environmental samples	2005
2757	F. Zhao	Effects of sodium arsenite and dimethyl arsenic acid on Liaoning cashmere goat skin fibroblasts	2021
2758	A. Medrano-Soto	Expansion of the Transporter-Opsin-G protein-coupled receptor superfamily with five new protein families	2020
2759	R. T. Mertens	Exploring six-coordinate germanium(IV)-diketonate complexes as anticancer agents	2020
2760	R. J. Erickson	The effects of arsenic speciation on accumulation and toxicity of dietborne arsenic exposures to rainbow trout	2019
2761	T. Masuda	Hepatic histopathological changes and dysfunction in primates following exposure to organic arsenic diphenylarsinic acid	2018
2762	O. F. Ordoñez	Haloarchaea from the Andean Puna: Biological Role in the Energy Metabolism of Arsenic	2018
2763	C. Pace	Monomethylarsonous acid, but not inorganic arsenic, is a mitochondria-specific toxicant in vascular smooth muscle cells	2016
2764	D. H. Baek	Embryotoxicity assessment of developmental neurotoxicants using a neuronal endpoint in the embryonic stem cell test	2012
2765	C. Shen-Tu	Arsenic species analysis by ion chromatography-bianode electrochemical hydride generator-atomic fluorescence spectrometry	2008
2766	K. Piatek	Monomethylarsonous acid destroys a tetrathiolate zinc finger much more efficiently than inorganic arsenite: mechanistic considerations and consequences for DNA repair inhibition	2008
2767	C. M. Tseng	Field cryofocussing hydride generation applied to the simultaneous multi-elemental determination of alkyl-metal(loid) species in natural waters using ICP-MS detection	2000
2768	R. A. Zakharyan	Enzymatic reduction of arsenic compounds in mammalian systems: the rate-limiting enzyme of rabbit liver arsenic biotransformation is MMA(V) reductase	1999
2769	M. I. Fonseca	Inhibition of muscarinic cholinergic receptors by disulfide reducing agents and arsenicals. Differential effect on locust and rat	1991
2770	I. Herath	Thiolated arsenic in natural systems: What is current, what is new and what needs to be known	2018

2771	M. Molin	Humans seem to produce arsenobetaine and dimethylarsinate after a bolus dose of seafood	2012
2772	R. Colognato	Genotoxicity induced by arsenic compounds in peripheral human lymphocytes analysed by cytokinesis-block micronucleus assay	2007
2773	R. D. Mitchell	Systemic indicators of inorganic arsenic toxicity in four animal species	2000
2774	E. Wildfang	Enzymatic methylation of arsenic compounds. VI. Characterization of hamster liver arsenite and methylarsonic acid methyltransferase activities in vitro	1998
2775	J. S. Woods	Effects of chronic arsenic exposure on hematopoietic function in adult mammalian liver	1977
2776	M. Zheng	Differing effects of inorganic and organic arsenic on uptake and distribution of multi-elements in Rice grain	2021
2777	M. Aminul Haque	Sustainability assessment of arsenic-iron bearing groundwater treatment soil mixed mortar in developing countries, Bangladesh	2020
2778	A. J. DeGraffenreid	Trithiols and their arsenic compounds for potential use in diagnostic and therapeutic radiopharmaceuticals	2016
2779	N. Nahar	In silico and in vivo studies of an Arabidopsis thaliana gene, ACR2, putatively involved in arsenic accumulation in plants	2012
2780	T. Yin	Combined effects of As4S4 and imatinib on chronic myeloid leukemia cells and BCR-ABL oncoprotein	2004
2781	C. J. Brown	Effects of chromated copper arsenate (CCA) wood preservative on early fouling community formation	2001
2782	M. J. Mass	Methylated trivalent arsenic species are genotoxic	2001
2783	T. Ochi	Different effects of inorganic and dimethylated arsenic compounds on cell morphology, cytoskeletal organization, and DNA synthesis in cultured Chinese hamster V79 cells	1998
2784	B. K. Puri	Determination of arsenic in crude petroleum and liquid hydrocarbons	1989
2785	M. J. Palmer	Mineralogical, geospatial, and statistical methods combined to estimate geochemical background of arsenic in soils for an area impacted by legacy mining pollution	2021
2786	Đ. Miodragović	Arsenoplatin-1 Is a Dual Pharmacophore Anticancer Agent	2019
2787	P. Zhao	Neurotoxicity induced by arsenic in Gallus Gallus: Regulation of oxidative stress and heat shock protein response	2017
2788	Z. Yu	ETME, a novel β-elemene derivative, synergizes with arsenic trioxide in inducing apoptosis and cell cycle arrest in hepatocarcinoma cells via a p53-dependent pathway	2014

2789	C. Liu	Direct toxicity assessment of toxic chemicals with electrochemical method	2009
2790	C. Y. Hsieh	Combined exposure to heavy metals in PM(2.5) and pediatric asthma	2021
2791	C. Soobramoney	The Screening of Nails for Selected Essential and Toxic Elements in Normotensive and Pre-Eclamptic Women	2019
2792	H. Zhou	Strategies for arsenic pollution control from copper pyrometallurgy based on the study of arsenic sources, emission pathways and speciation characterization in copper flash smelting systems	2021
2793	X. Cai	Preparation and anti-Raji lymphoma efficacy of a novel pH sensitive and magnetic targeting nanoparticles drug delivery system	2020
2794	M. H. Hoang	Fucosterol is a selective liver X receptor modulator that regulates the expression of key genes in cholesterol homeostasis in macrophages, hepatocytes, and intestinal cells	2012
2795	J. Zhao	Novel polyselenidoarsenate and selenidoarsenate: solvothermal synthesis and characterization of [Co(phen)3][As2Se2(μ-Se3)(μ-Se5)] and [Co(phen)3]2[As8Se14]	2011
2796	S. B. Gibson	A matter of balance between life and death: targeting reactive oxygen species (ROS)-induced autophagy for cancer therapy	2010
2797	T. Narukawa	Preparation and certification of arsenate [As(V)] reference material, NMIJ CRM 7912-a	2010
2798	H. R. Seo	Radiation-induced cathepsin S is involved in radioresistance	2009
2799	M. V. Aldrich	Examination of arsenic(III) and (V) uptake by the desert plant species mesquite (Prosopis spp.) using X-ray absorption spectroscopy	2007
2800	G. De la Rosa	Spectroscopic study of the impact of arsenic speciation on arsenic/phosphorus uptake and plant growth in tumbleweed (Salsola kali)	2006
2801	Y. Wu	The influence of curcumin on the cell cycle of HL-60 cells and contrast study	2000
2802	D. Y. Lin	Promyelocytic leukemia protein (PML) functions as a glucocorticoid receptor co-activator by sequestering Daxx to the PML oncogenic domains (PODs) to enhance its transactivation potential	2003
2803	E. Kim	Effect of sodium dichromate on carbohydrate metabolism	1991
2804	C. Y. Shen	Prenatal Exposure to Endocrine-Disrupting Chemicals and Subsequent Brain Structure Changes Revealed by Voxel-Based Morphometry and Generalized Q-Sampling MRI	2021
2805	Y. Zhang	Simultaneous catalytic oxidation of elemental mercury and arsine over CeO(2)(111) surface: a density functional theory study	2022
2806	K. Zhang	Arsenite-loaded albumin nanoparticles for targeted synergistic chemo-photothermal therapy of HCC	2021

2807	Z. Rakvács	The human ABCB6 protein is the functional homologue of HMT-1 proteins mediating cadmium detoxification	2019
2808	M. Ahadi	The HER-2 as a Target Gene of Curcumin to Protect Hepatocytes Against the Arsenic-induced Carcinoma in Mice	2017
2809	M. Lin	Recent advances in nanosized Mn-Zn ferrite magnetic fluid hyperthermia for cancer treatment	2014
2810	F. Binet	Arsenic trioxide induces endoplasmic reticulum stress-related events in neutrophils	2010
2811	Y. Han	Arsenic influences spermatogenesis by disorganizing the elongation of spermatids in adult male mice	2020
2812	M. Ubaid Ali	Evaluation of floor-wise pollution status and deposition behavior of potentially toxic elements and nanoparticles in air conditioner dust during urbanistic development	2019
2813	Z. Dong	Epicatechin rescues the As(2) O(3) -induced HERG K(+) channel deficiency possibly through upregulating transcription factor SP1 expression	2017
2814	R. Luo	Determination of arsenic and lead in single hair strands by laser ablation inductively coupled plasma mass spectrometry	2017
2815	P. Heitland	Comparison of different medical cases in urinary arsenic speciation by fast HPLC-ICP-MS	2009
2816	G. L. Czarnecki	Reduction of liver copper concentration by the organic arsenical, 3-nitro-4-hydroxyphenylarsonic acid	1985
2817	S. Zhu	Correlation between level of metallic elements in urine and childhood acute leukemia	2011
2818	S. M. Rahman	Manganese in Drinking Water and Cognitive Abilities and Behavior at 10 Years of Age: A Prospective Cohort Study	2017
2819	S. Peng	A nested case-control study indicating heavy metal residues in meconium associate with maternal gestational diabetes mellitus risk	2015
2820	J. Zhang	Transcriptomic Responses During Early Development Following Arsenic Exposure in Western Clawed Frogs, Silurana tropicalis	2015
2821	F. Azizian-Farsani	Impact of sodium arsenite on chromosomal aberrations with respect to polymorphisms of detoxification and DNA repair genes	2014
2822	A. Noormohamed	Arsenic resistance and prevalence of arsenic resistance genes in Campylobacter jejuni and Campylobacter coli isolated from retail meats	2013
2823	J. R. Saunders	Use of biomarkers to show sub-cellular effects in meadow voles (Microtus pennsylvanicus) living on an abandoned gold mine site	2009
2824	V. Kokilavani	Combined efficacies of DL-alpha-lipoic acid and meso 2,3 dimercaptosuccinic acid against arsenic induced toxicity in antioxidant systems of rats	2005

2825	C. J. Brown	Toxicity of chromated copper arsenate (CCA)-treated wood to non-target marine fouling communities in Langstone Harbour, Portsmouth, UK	2001
2826	Y. Zhao	Influence of operating parameters on arsenic transformation during municipal sewage sludge incineration with cotton stalk	2018
2827	K. J. Whaley-Martin	Arsenic speciation in blue mussels (Mytilus edulis) along a highly contaminated arsenic gradient	2012
2828	M. K. Uroic	Chemotrapping-atomic fluorescence spectrometric method as a field method for volatile arsenic in natural gas	2009
2829	L. Zhang	A study on arsenic speciation analysis in animal origin seafood	2008
2830	P. B. Tchounwou	Important considerations in the development of public health advisories for arsenic and arsenic-containing compounds in drinking water	1999
2831	A. Das	Depletion of S-adenosylmethionine pool and promoter hypermethylation of Arsenite methyltransferase in arsenic-induced skin lesion individuals: A case-control study from West Bengal, India	2021
2832	R. Guillod-Magnin	Arsenic species in rice and rice-based products consumed by toddlers in Switzerland	2018
2833	M. Di Carlo	Trace elements and arsenic speciation in tissues of tube dwelling polychaetes from hydrothermal vent ecosystems (East Pacific Rise): An ecological role as antipredatory strategy?	2017
2834	Y. Cui	MicroRNA-181b and microRNA-9 mediate arsenic-induced angiogenesis via NRP1	2012
2835	M. A. Peraza	Toxicity and metabolism of subcytotoxic inorganic arsenic in human renal proximal tubule epithelial cells (HK-2)	2003
2836	C. J. Langdon	Arsenic speciation in the earthworms Lumbricus rubellus and Dendrodrilus rubidus	2003
2837	M. Hours	[Occupational exposure and malignant hemopathies: a case-control study in Lyon (France)]	1995
2838	Q. Lou	Arsenic exposure elevated ROS promotes energy metabolic reprogramming with enhanced AKT-dependent HK2 expression	2022
2839	R. Zhao	The response of Pyropia haitanensis to inorganic arsenic under laboratory culture	2020
2840	K. H. Aung	Inhibition of neurite outgrowth and alteration of cytoskeletal gene expression by sodium arsenite	2013
2841	Y. Yoshimura	Acute arsine poisoning confirmed by speciation analysis of arsenic compounds in the plasma and urine by HPLC-ICP-MS	2011
2842	S. Yadav	Arsenite induces apoptosis in human mesenchymal stem cells by altering Bcl-2 family proteins and by activating intrinsic pathway	2010

2843	S. H. Liou	Hypersensitivity to mitomycin C-induced sister chromatid exchange as a biomarker of past exposure to arsenic	1996
2844	A. Mathee	A cross-sectional analytical study of geophagia practices and blood metal concentrations in pregnant women in Johannesburg, South Africa	2014
2845	K. A. Winship	Toxicity of antimony and its compounds	1987
2846	Y. P. Yu	BMP8A promotes survival and drug resistance via Nrf2/TRIM24 signaling pathway in clear cell renal cell carcinoma	2020
2847	A. Shibayama	Treatment of smelting residue for arsenic removal and recovery of copper using pyro-hydrometallurgical process	2010
2848	M. L. Astolfi	A prophylactic multi-strain probiotic treatment to reduce the absorption of toxic elements: In-vitro study and biomonitoring of breast milk and infant stools	2019
2849	Y. Yong	An all-in-one strategy for resource recovery and immobilization of arsenic from arsenic-bearing gypsum sludge	2022
2850	K. Nagai	Combination of ATO with FLT3 TKIs eliminates FLT3/ITD+ leukemia cells through reduced expression of FLT3	2018
2851	J. M. Laparra	As2O3-induced oxidative stress and cycle progression in a human intestinal epithelial cell line (Caco-2)	2008
2852	L. Xia	Upregulation of Bfl-1/A1 in leukemia cells undergoing differentiation by all-trans retinoic acid treatment attenuates chemotherapeutic agent-induced apoptosis	2006
2853	K. Mitra	Efficacy of a potentized homoeopathic drug (Arsenicum Album-30) in reducing toxic effects produced by arsenic trioxide in mice: II. On alterations in body weight, tissue weight and total protein	1999
2854	A. Singh	Arsenic: a Culpable Element and a Possible Menace for HIV/AIDS Patients	2022
2855	J. Bajpai	Acute promyelocytic leukemia: an experience from a tertiary care centre in north India	2011
2856	T. Charrier	A multi-channel bioluminescent bacterial biosensor for the on-line detection of metals and toxicity. Part II: technical development and proof of concept of the biosensor	2011
2857	S. Datta	Chronic exposure to low concentration of arsenic is immunotoxic to fish: role of head kidney macrophages as biomarkers of arsenic toxicity to Clarias batrachus	2009
2858	Z. C. Yan	Effects of aqueous extracts of Aconitum carmichaeli, Rhizoma bolbostemmatis, Phytolacca acinosa, Panax notoginseng and Gekko swinhonis Gūenther on Bel-7402 cells	2007
2859	M. Kadiyska	Influence of the acute intoxication with salts of some heavy metals on hexobarbital sleep and hexobarbital metabolism	1979
2860	A. Ücker	Critical Evaluation of Specific Efficacy of Preparations Produced According to European Pharmacopeia Monograph 2371	2022

2861	B. Song	In-Furnace Control of Arsenic Vapor Emissions Using Fe(2)O(3) Microspheres with Good Sintering Resistance	2021
2862	Y. Wang	Arsenite renal apoptotic effects in chickens co-aggravated by oxidative stress and inflammatory response	2018
2863	J. Yang	Inactivation of miR-100 combined with arsenic treatment enhances the malignant transformation of BEAS-2B cells via stimulating epithelial -mesenchymal transition	2017
2864	H. Sun	Inhibition of IRE1α-driven pro-survival pathways is a promising therapeutic application in acute myeloid leukemia	2016
2865	Y. Li	Subchronic exposure to arsenic inhibits spermatogenesis and downregulates the expression of ddx3y in testis and epididymis of mice	2012
2866	W. H. Park	Arsenic trioxide induces human pulmonary fibroblast cell death via the regulation of Bcl-2 family and caspase-8	2012
2867	S. M. Gao	Synergistic apoptosis induction in leukemic cells by miR-15a/16-1 and arsenic trioxide	2010
2868	V. Stibilj	A study of low level selenium determination by hydride generation atomic fluorescence spectrometry in water soluble protein and peptide fractions	2003
2869	C. J. Shih	Arsenic contaminated site at an abandoned copper smelter plant: waste characterization and solidification/stabilization treatment	2003
2870	X. Hu	Ailing No. I in treating 62 cases of acute promyelocytic leukemia	1999
2871	Z. Liu	Involvement of autophagy in realgar quantum dots (RQDs) inhibition of human endometrial cancer JEC cells	2020
2872	P. Khanmohammadi	Purification of the leaching solution of recycling zinc from the hazardous electric arc furnace dust through an as-bearing jarosite	2020
2873	B. A. Boswell	Upregulation and maintenance of gap junctional communication in lens cells	2009
2874	E. F. Zama	The removal of arsenic from solution through biochar-enhanced precipitation of calcium-arsenic derivatives	2022
2875	Y. L. Li	The molecular mechanism of a novel derivative of BTO-956 induced apoptosis in human myelomonocytic lymphoma cells	2021
2876	Y. Shimoda	Differences in apoptotic signaling and toxicity between dimethylmonothioarsinic acid (DMMTA(V)) and its active metabolite, dimethylarsinous acid (DMA(III)), in HepaRG cells: Possibility of apoptosis cascade based on diversity of active metabolites of DMMTA(V)	2018
2877	X. Fuku	Cytochrome c biosensor for determination of trace levels of cyanide and arsenic compounds	2012
2878	S. C. Shen	Reactive oxygen species-dependent HSP90 protein cleavage participates in arsenical As(+3)- and MMA(+3)-induced apoptosis through inhibition of telomerase activity via JNK activation	2008

2879	B. P. Jackson	Fate of arsenic compounds in poultry litter upon land application	2006
2880	B. Li	Distribution of elements binding to molecules with different molecular weights in aqueous extract of Antarctic krill by size-exclusion chromatography coupled with inductively coupled plasma mass spectrometry	2005
2881	J. W. Severinghaus	Fire-air and dephlogistication. Revisionisms of oxygen's discovery	2003
2882	B. K. Mandal	Identification of dimethylarsinous and monomethylarsonous acids in human urine of the arsenic-affected areas in West Bengal, India	2001
2883	P. Ashrap	In utero and peripubertal metals exposure in relation to reproductive hormones and sexual maturation and progression among girls in Mexico City	2019
2884	A. Terol	Fast Determination of Toxic Arsenic Species in Food Samples Using Narrow-bore High-Performance Liquid-Chromatography Inductively Coupled Plasma Mass Spectrometry	2016
2885	J. Ventura-Lima	Effects of different inorganic arsenic species in Cyprinus carpio (Cyprinidae) tissues after short-time exposure: bioaccumulation, biotransformation and biological responses	2009
2886	W. Ding	Inorganic arsenic compounds cause oxidative damage to DNA and protein by inducing ROS and RNS generation in human keratinocytes	2005
2887	L. H. Yih	Arsenite induces prominent mitotic arrest via inhibition of G2 checkpoint activation in CGL-2 cells	2005
2888	T. G. Rossman	Arsenite cocarcinogenesis: an animal model derived from genetic toxicology studies	2002
2889	M. Goldman	Lewisite: its chemistry, toxicology, and biological effects	1989
2890	S. Bal	Shielding effect of anethole against arsenic induced genotoxicity in cultured human peripheral blood lymphocytes and effect of GSTO1 polymorphism	2018
2891	S. K. Stamatelos	A semi-mechanistic integrated toxicokinetic-toxicodynamic (TK/TD) model for arsenic(III) in hepatocytes	2013
2892	S. C. Shen	Quercetin enhancement of arsenic-induced apoptosis via stimulating ROS-dependent p53 protein ubiquitination in human HaCaT keratinocytes	2012
2893	L. Pari	Protective role of sinapic acid against arsenic: induced toxicity in rats	2011
2894	Y. C. Huang	Effects of MEK and DNMT inhibitors on arsenic-treated human uroepithelial cells in relation to Cyclin-D1 and p16	2011
2895	Y. C. Huang	Sodium arsenite-induced DAPK promoter hypermethylation and autophagy via ERK1/2 phosphorylation in human uroepithelial cells	2009
2896	J. R. Landolph	Molecular mechanisms of transformation of C3H/10T1/2 C1 8 mouse embryo cells and diploid human fibroblasts by carcinogenic metal compounds	1994

2897	C. G. Howe	Prenatal metal mixtures and child blood pressure in the Rhea mother-child cohort in Greece	2021
2898	E. Kalaeva	Incidence risk of bronchopneumonia in newborn calves associated with intrauterine diselementosis	2020
2899	K. Maduray	Elemental analysis of serum and hair from pre-eclamptic South African women	2017
2900	P. Biswas	High-Protein Diet Ameliorates Arsenic-Induced Oxidative Stress and Antagonizes Uterine Apoptosis in Rats	2019
2901	Y. Shao	Arsenic and/or copper caused inflammatory response via activation of inducible nitric oxide synthase pathway and triggered heat shock protein responses in testis tissues of chicken	2018
2902	M. Moradzadeh	Ferula gummosa gum induces apoptosis via ROS mechanism in human leukemic cells	2017
2903	Y. Song	Design, synthesis and anticancer activity of N(3),N(11)-bis(2-hydroxyethyl)-14-aryl-14H-dibenzo[a,j]xanthenes-3,11-dicarboxamide	2013
2904	C. C. Yen	Inorganic arsenic causes cell apoptosis in mouse cerebrum through an oxidative stress-regulated signaling pathway	2011
2905	G. F. Ouyang	[Arsenic trioxide enhances the effects of bortezomib, dexamethasone on multiple myeloma cell line KM3 in vitro.]	2010
2906	D. Jia	Direct solvothermal growth, crystal structures, and optical properties of one-dimensional lanthanide selenidoarsenate(v) polymers [Ln(dien)2(micro3-AsSe4)] (Ln = Nd, Sm): the first example of an AsSe4(3-) anion acting as a ligand to a lanthanide complex	2007
2907	Y. Jing	The cleavage product deltaPML-RARalpha contributes to all-trans retinoic acid-mediated differentiation in acute promyelocytic leukemia cells	2003
2908	C. Liang	Arsenic induces dysfunctional autophagy via dual regulation of mTOR pathway and Beclin1-Vps34/PI3K complex in MLTC-1 cells	2020
2909	C. P. Cheng	Atomic Nature of the Growth Mechanism of Atomic Layer Deposited High-κ Y(2)O(3) on GaAs(001)-4 × 6 Based on in Situ Synchrotron Radiation Photoelectron Spectroscopy	2018
2910	M. C. Guillemin	In vivo activation of cAMP signaling induces growth arrest and differentiation in acute promyelocytic leukemia	2002
2911	Y. T. Liu	A retrospective lung cancer mortality study of people exposed to insoluble arsenic and radon	1996
2912	Q. C. Bi	Sevelamer arsenite nanoparticle as a Pi-responsive drug carrier and embolic agent for chemoembolization	2022
2913	J. Zhang	Subchronic exposure to arsenic disturbed the biogenic amine neurotransmitter level and the mRNA expression of synthetase in mice brains	2013
2914	Y. Liu	Targeting X box-binding protein-1 (XBP1) enhances sensitivity of glioma cells to oxidative stress	2011

2915	L. Aram	VDAC1 cysteine residues: topology and function in channel activity and apoptosis	2010
2916	H. L. Shen	[Experimental study on apoptosis of leukemia cell line NB4 transfected with WT1 gene]	2005
2917	H. Zhao	The cardiotoxicity of the common carp (Cyprinus carpio) exposed to environmentally relevant concentrations of arsenic and subsequently relieved by zinc supplementation	2019
2918	J. Liu	Arsenic trioxide and/or copper sulfate induced apoptosis and autophagy associated with oxidative stress and perturbation of mitochondrial dynamics in the thymus of Gallus gallus	2019
2919	J. Shao	Toxicogenomics-based identification of mechanisms for direct immunotoxicity	2013
2920	M. T. Malloy	Trafficking of the transcription factor Nrf2 to promyelocytic leukemia-nuclear bodies: implications for degradation of NRF2 in the nucleus	2013
2921	H. G. Kim	Polycomb (PcG) proteins, BMI1 and SUZ12, regulate arsenic-induced cell transformation	2012
2922	F. Piao	Abnormal expression of 8-nitroguanine in the brain of mice exposed to arsenic subchronically	2011
2923	N. Keinan	Oligomerization of the mitochondrial protein voltage-dependent anion channel is coupled to the induction of apoptosis	2010
2924	T. Bacquart	Direct speciation analysis of arsenic in sub-cellular compartments using micro-X-ray absorption spectroscopy	2010
2925	M. Giannì	In acute promyelocytic leukemia NB4 cells, the synthetic retinoid CD437 induces contemporaneously apoptosis, a caspase-3-mediated degradation of PML/RARalpha protein and the PML retargeting on PML-nuclear bodies	1999
2926	A. C. Schmidt	Qualitative and quantitative characterization of the arsenic-binding behaviour of sulfur-containing peptides and proteins by the coupling of reversed phase liquid chromatography to electrospray ionization mass spectrometry	2012
2927	K. A. Mir	Extraction and speciation of arsenic in plants grown on arsenic contaminated soils	2007
2928	S. B. Jonnalagadda	Toxicity, bioavailability and metal speciation	1993
2929	D. J. Carlin	Arsenic and Environmental Health: State of the Science and Future Research Opportunities	2016
2930	X. Zhang	Oxidation of structural cysteine residues in thioredoxin 1 by aromatic arsenicals enhances cancer cell cytotoxicity caused by the inhibition of thioredoxin reductase 1	2015
2931	A. Y. Efremenko	Evaluation of gene expression changes in human primary lung epithelial cells following 24-hr exposures to inorganic arsenic and its methylated metabolites and to arsenic trioxide	2015
2932	H. Naranmandura	Release of apoptotic cytochrome C from mitochondria by dimethylarsinous acid occurs through interaction with voltage-dependent anion channel in vitro	2012

2933	N. Demir	The relationship between mother and infant plasma trace element and heavy metal levels and the risk of neural tube defect in infants	2019
2934	W. Wen	[Changes in mRNA expression of p53 and related downstream genes in peripheral blood lymphocytes in workers occupationally exposed to arsenic]	2015
2935	S. García-Salgado	Levels of toxic arsenic species in native terrestrial plants from soils polluted by former mining activities	2014
2936	R. C. Reedy	Unsaturated zone arsenic distribution and implications for groundwater contamination	2007
2937	A. R. Mistry	The molecular pathogenesis of acute promyelocytic leukaemia: implications for the clinical management of the disease	2003
2938	X. Baur	[Bronchopulmonary precancerous conditions and tumors--risk groups from the occupational medicine viewpoint]	1994
2939	K. Łoźna	Arsenic contents in rats' fur as an indicator of exposure to arsenic. Preliminary studies	2014
2940	K. Rehman	Mechanisms underlying the inhibitory effects of arsenic compounds on protein tyrosine phosphatase (PTP)	2012
2941	C. P. Verdon	Determination of seven arsenic compounds in urine by HPLC-ICP-DRC-MS: a CDC population biomonitoring method	2009
2942	M. R. Gigante	[Evaluation of the role of occupational and environmental exposure to inorganic arsenic in the urinary excretion of the metal: preliminary data]	2006
2943	M. S. Rahman	In vitro percutaneous absorption of monosodium methanearsonate and disodium methanearsonate in female B6C3F1 mice	1994
2944	Q. X. Ren	Effects of Proanthocyanidins on Arsenic Methylation Metabolism and Efflux in Human Hepatocytes L-02	2019
2945	M. Yaghmaie	Characterization of arsenic-induced cytogenetic alterations in acute promyelocytic leukemia cell line, NB4	2012
2946	Q. Wu	Chemical form of metals in traditional medicines underlines potential toxicity in cell cultures	2011
2947	S. W. Burchiel	Immunotoxicity and biodistribution analysis of arsenic trioxide in C57Bl/6 mice following a 2-week inhalation exposure	2009
2948	S. Jimi	Mechanisms of cell death induced by cadmium and arsenic	2004
2949	Y. Zhang	MiR-451 Promotes Cell Apoptosis and Inhibits Autophagy in Pediatric Acute Myeloid Leukemia by Targeting HMGB1	2021
2950	M. M. Bradley	Colorimetric-solid phase extraction method for trace level determination of arsenite in water	2011

2951	T. Ito	Establishment and characterization of a novel imatinib-sensitive chronic myeloid leukemia cell line MYL, and an imatinib-resistant subline MYL-R showing overexpression of Lyn	2007
2952	E. M. Rego	Leukemia with distinct phenotypes in transgenic mice expressing PML/RAR alpha, PLZF/RAR alpha or NPM/RAR alpha	2006
2953	Y. K. Ham	Effects of Toxic Heavy Metal Salts on Oxidative Quality Deterioration in Ground Pork Model during Aerobic Display Storage	2022
2954	K. Mehta	Resveratrol protects against inorganic arsenic-induced oxidative damage and cytoarchitectural alterations in female mouse hippocampus	2021
2955	M. Guo	Elemental imbalance elicited by arsenic and copper exposures leads to oxidative stress and immunotoxicity in chicken gizzard, activating the protective effects of heat shock proteins	2019
2956	L. Berthoux	Lv1 inhibition of human immunodeficiency virus type 1 is counteracted by factors that stimulate synthesis or nuclear translocation of viral cDNA	2004
2957	S. Chupradit	Hepatoprotective and therapeutic effects of resveratrol: A focus on anti-inflammatory and antioxidative activities	2022
2958	H. Zhou	The role of Hipk2-p53 pathways in arsenic-induced autistic behaviors: A translational study from rats to humans	2020
2959	F. F. Lyu	[Effect of arsenic trioxide on differentiation induction of chronic myeloid leukemia k562 cells and its potential mechanism]	2015
2960	B. Liu	Three banana-shaped arsenomolybdates encapsulating a hexanuclear transition-metal central magnetic cluster: [As(III)2Fe(III)5MMo22O85(H2O)]n- (M = Fe3+, n = 14; M = Ni2+ and Mn2+, n = 15)	2011
2961	K. Lundholm	Fate of Cu, Cr, and As during combustion of impregnated wood with and without peat additive	2007
2962	L. Helsen	Development of a sampling train for arsenic in pyrolysis vapours resulting from pyrolysis of arsenic containing wood waste	2003
2963	J. Kolařík	Impact of inorganic ions and natural organic matter on arsenates removal by ferrate(VI): Understanding a complex effect of phosphates ions	2018
2964	S. Ciardullo	Arsenic speciation in freshwater fish: focus on extraction and mass balance	2010
2965	N. A. Rey	Equilibrium characterization of the As(III)-cysteine and the As(III)-glutathione systems in aqueous solution	2004
2966	H. R. Hansen	Metabolism of arsenic by sheep chronically exposed to arsenosugars as a normal part of their diet. 1. Quantitative intake, uptake, and excretion	2003
2967	M. Takahashi	Transformation by inorganic arsenic compounds of normal Syrian hamster embryo cells into a neoplastic state in which they become anchorage-independent and cause tumors in newborn hamsters	2002
2968	H. H. Rahman	Association of albumin to creatinine ratio with urinary arsenic and metal exposure: evidence from NHANES 2015-2016	2022

2969	K. K. Jinadasa	Ionic imprinted polymer solid-phase extraction for inorganic arsenic selective pre-concentration in fishery products before high-performance liquid chromatography - inductively coupled plasma-mass spectrometry speciation	2020
2970	S. Zarazúa	Arsenic affects expression and processing of amyloid precursor protein (APP) in primary neuronal cells overexpressing the Swedish mutation of human APP	2011
2971	D. B. Richardson	Occupational risk factors for non-Hodgkin's lymphoma: a population-based case-control study in Northern Germany	2008
2972	Y. H. Hwang	Monitoring of arsenic exposure with speciated urinary inorganic arsenic metabolites for ion implanter maintenance engineers	2002
2973	L. H. Yih	Arsenite induces p53 accumulation through an ATM-dependent pathway in human fibroblasts	2000
2974	Y. H. Liang	[Acetyl-11-keto-beta-boswellic acid and arsenic trioxide regulate the productions and activities of matrix metalloproteinases in human skin fibroblasts and human leukemia cell line THP-1]	2010
2975	K. M. Wai	Protective role of selenium in the shortening of telomere length in newborns induced by in utero heavy metal exposure	2020
2976	R. C. Lewis	Urinary metal concentrations among mothers and children in a Mexico City birth cohort study	2018
2977	A. Zanobetti	Ambient Particle Components and Newborn Blood Pressure in Project Viva	2021
2978	A. S. Veyhe	The Northern Norway Mother-and-Child Contaminant Cohort (MISA) Study: PCA analyses of environmental contaminants in maternal sera and dietary intake in early pregnancy	2015
2979	K. Zhu	MOF-on-MOF Membrane with Cascading Functionality for Capturing Dichromate Ions and p-Arsanilic Acid Turn-On Sensing	2020
2980	A. Takeuchi	Development of an analytical method for the determination of arsenic in urine by gas chromatography-mass spectrometry for biological monitoring of exposure to inorganic arsenic	2012
2981	A. S. Lewis	Speciated arsenic in air: measurement methodology and risk assessment considerations	2012
2982	R. Jakob	Atmospheric stability of arsines and the determination of their oxidative products in atmospheric aerosols (PM10): evidence of the widespread phenomena of biovolatilization of arsenic	2010
2983	M. E. Parent	Does exposure to agricultural chemicals increase the risk of prostate cancer among farmers?	2009
2984	S. de Rosemond	Arsenic concentration and speciation in five freshwater fish species from Back Bay near Yellowknife, NT, CANADA	2008
2985	Y. C. Wang	Comparison of the cytotoxicity induced by different exposure to sodium arsenite in two fish cell lines	2004
2986	J. Zheng	Speciation of arsenic in water, sediment, and plants of the Moira watershed, Canada, using HPLC coupled to high resolution ICP-MS	2003

2987	F. A. Pitten	Risk assessment of a former military base contaminated with organoarsenic-based warfare agents: uptake of arsenic by terrestrial plants	1999
2988	C. C. Kuo	The association of arsenic exposure and arsenic metabolism with all-cause, cardiovascular and cancer mortality in the Strong Heart Study	2022
2989	V. Bencko	The history of arsenical pesticides and health risks related to the use of Agent Blue	2017
2990	M. Boulanger	Agricultural exposure and risk of bladder cancer in the AGRIculture and CANcer cohort	2017
2991	D. Fattorini	Levels and chemical speciation of arsenic in representative biota and sediments of a tropical mangrove wetland, India	2013
2992	A. t Mannetje	Occupational exposure to metal compounds and lung cancer. Results from a multi-center case-control study in Central/Eastern Europe and UK	2011
2993	K. O. Amayo	Identification and quantification of arsenolipids using reversed-phase HPLC coupled simultaneously to high-resolution ICPMS and high-resolution electrospray MS without species-specific standards	2011
2994	J. W. de Mello	Arsenic speciation in arsenic-rich Brazilian soils from gold mining sites under anaerobic incubation	2007
2995	T. Lech	Significance of copper determination in late onset of Wilson's disease	2007
2996	B. Sun	Separation of organic and inorganic arsenic species by capillary electrophoresis using direct spectrophotometric detection	2002
2997	N. Melitas	Electrochemical study of arsenate and water reduction on iron media used for arsenic removal from potable water	2002
2998	N. Melitas	Understanding soluble arsenate removal kinetics by zerovalent iron media	2002
2999	D. Liu	Differential effects of arsenic species on Nrf2 and Bach1 nuclear localization in cultured hepatocytes	2021
3000	M. Chakraborty	Prenatal arsenic exposure interferes in postnatal immunocompetence despite an absence of ongoing arsenic exposure	2020
3001	F. Gong	Heparin-immobilized polymers as non-inflammatory and non-thrombogenic coating materials for arsenic trioxide eluting stents	2010
3002	S. Irshad	Geochemical fractionation and spectroscopic fingerprinting for evaluation of the environmental transformation of potentially toxic metal(oid)s in surface-subsurface soils	2021
3003	P. R. Subbarayan	Arsenic trioxide suppresses thymidylate synthase in 5-FU-resistant colorectal cancer cell line HT29 In Vitro re-sensitizing cells to 5-FU	2010
3004	W. G. Mao	[Effect of differentiation inducer and heat stress on the expression of JWA protein and Hsp70 of K562 cells]	2003

3005	M. Lyczko	1,2-Benzenedithiol and Toluene-3,4-dithiol Arsenic(III) Complexes-Synthesis, Structure, Spectroscopic Characterization and Toxicological Studies	2019
3006	C. Y. Weng	Arsenic trioxide induces unfolded protein response in vascular endothelial cells	2014
3007	J. Z. Wu	Comparing the relative oxidative DNA damage caused by various arsenic species by quantifying urinary levels of 8-hydroxy-2'-deoxyguanosine with isotope-dilution liquid chromatography/mass spectrometry	2009
3008	J. Q. Liu	[Inhibition effect of topotecan on human myelodysplastic syndrome cells in vitro and in vivo]	2006
3009	J. Zhang	Angiogenesis is Inhibited by Arsenic Trioxide Through Downregulation of the CircHIPK3/miR-149-5p/FOXO1/VEGF Functional Module in Rheumatoid Arthritis	2021
3010	B. Fu	The key roles of Fe-bearing minerals on arsenic capture and speciation transformation during high-As bituminous coal combustion: Experimental and theoretical investigations	2021
3011	W. C. Hu	Combinatorial Low Dose Arsenic Trioxide and Cisplatin Exacerbates Autophagy via AMPK/STAT3 Signaling on Targeting Head and Neck Cancer Initiating Cells	2020
3012	O. S. Fagbenro	Experimental modeling of the acute toxicity and cytogenotoxic fate of composite mixtures of chromate, copper and arsenate oxides associated with CCA preservative using Clarias gariepinus (Burchell 1822)	2019
3013	J. S. Yoon	Anti-leukemic effect of sodium metaarsenite (KML001) in acute myeloid leukemia with breaking-down the resistance of cytosine arabinoside	2015
3014	K. K. Yadav	Chromosomal aberrations in a fish, Channa punctata after in vivo exposure to three heavy metals	2009
3015	V. Dutré	Oxidation of arsenic bearing fly ash as pretreatment before solidification	1999
3016	H. K. Park	Combination of Arsenic Trioxide and Valproic Acid Efficiently Inhibits Growth of Lung Cancer Cells via G2/M-Phase Arrest and Apoptotic Cell Death	2020
3017	M. Guo	Oxidative damage under As(3+) and/or Cu(2+) stress leads to apoptosis and autophagy and may be cross-talking with mitochondrial disorders in bursa of Fabricius	2020
3018	J. Liu	Alterations of antioxidant indexes and inflammatory cytokine expression aggravated hepatocellular apoptosis through mitochondrial and death receptor-dependent pathways in Gallus gallus exposed to arsenic and copper	2018
3019	C. Tang	Clusters [Co(As3S3)2]2-, [Ni(As3S3)2]2-, and [{Co(en)}6(μ3-S)4(AsS3)4]2- with Co-As or Ni-As bonds: solvothermal syntheses and characterizations of thioarsenates containing transition-metal complexes	2013
3020	D. Jia	Solvothermal synthesis and characterization of polyselenidoarsenate salts of transition metal complex cations	2011
3021	M. A. Park	A serotype 5/3 adenovirus expressing MDA-7/IL-24 infects renal carcinoma cells and promotes toxicity of agents that increase ROS and ceramide levels	2011
3022	S. Mishra	Arsenite treatment induces oxidative stress, upregulates antioxidant system, and causes phytochelatin synthesis in rice seedlings	2011

3023	D. E. Stephanopoulos	Treatment and toxicokinetics of acute pediatric arsenic ingestion: danger of arsenic insecticides in children	2002
3024	K. Koshiuka	Novel therapeutic approach: organic arsenical melarsoprol) alone or with all-trans-retinoic acid markedly inhibit growth of human breast and prostate cancer cells in vitro and in vivo	2000
3025	J. Xi	Acid Water-ground Nano-realgar Is Superior to Crude Realgar in Promoting Apoptosis of MCF-7 Breast Cancer Cells	2022
3026	Y. Zhu	Arsenic immobilization from aqueous solution by the precipitation of the pseudo-octahedral arsenate-substituted natroalunite solid solutions	2019
3027	B. Sun	Evaluated the Twenty-Six Elements in the Pectoral Muscle of As-Treated Chicken by Inductively Coupled Plasma Mass Spectrometry	2016
3028	T. Wang	Nicotinamide-mediated inhibition of SIRT1 deacetylase is associated with the viability of cancer cells exposed to antitumor agents and apoptosis	2013
3029	Y. A. Kuryshev	Antimony-based antileishmanial compounds prolong the cardiac action potential by an increase in cardiac calcium currents	2006
3030	Z. Wang	Arsenic speciation in urine from acute promyelocytic leukemia patients undergoing arsenic trioxide treatment	2004
3031	X. Zhang	Single-cell transcriptomics profiling the compatibility mechanism of As(2)O(3)-indigo naturalis formula based on bone marrow stroma cells	2022
3032	N. Gerasimchuk	New Solids in As-O-Mo, As(P)-O-Mo(W) and As(P)-O-Nb(W) Systems That Exhibit Nonlinear Optical Properties	2021
3033	Y. P. Chung	Arsenic induces human chondrocyte senescence and accelerates rat articular cartilage aging	2020
3034	M. Tuominen	Oxidation-Induced Changes in the ALD-Al(2)O(3)/InAs(100) Interface and Control of the Changes for Device Processing	2018
3035	X. Sun	Arsenic affects inflammatory cytokine expression in Gallus gallus brain tissues	2017
3036	Y. Ma	Arsenic and fluoride induce apoptosis, inflammation and oxidative stress in cultured human umbilical vein endothelial cells	2017
3037	N. Wang	'Prodrug-Like' Acetylmannosamine Modified Liposomes Loaded With Arsenic Trioxide for the Treatment of Orthotopic Glioma in Mice	2020
3038	P. Pei	Inorganic arsenic induces pyroptosis and pancreatic β cells dysfunction through stimulating the IRE1α/TNF-α pathway and protective effect of taurine	2019
3039	X. Sun	Synergistic effect of copper and arsenic upon oxidative stress, inflammation and autophagy alterations in brain tissues of Gallus gallus	2018
3040	T. Ezedom	Effect of a controlled food-chain mediated exposure to cadmium and arsenic on oxidative enzymes in the tissues of rats	2016

3041	J. Rault	A universal modified van der Waals equation of state. Part I: Polymer and mineral glass formers	2014
3042	S. Weisthal	Ca(2+)-mediated regulation of VDAC1 expression levels is associated with cell death induction	2014
3043	N. J. Bahlis	Feasibility and correlates of arsenic trioxide combined with ascorbic acid-mediated depletion of intracellular glutathione for the treatment of relapsed/refractory multiple myeloma	2002
3044	Y. C. Jou	Gene expression and DNA methylation regulation of arsenic in mouse bladder tissues and in human urothelial cells	2019
3045	Y. Chervona	Associations between arsenic exposure and global posttranslational histone modifications among adults in Bangladesh	2012
3046	L. Bermúdez	Assessment of ten trace elements in umbilical cord blood and maternal blood: association with birth weight	2015
3047	H. Tanaka	[Excess mortality among 5,064 victims of arsenic poisoning from ingestion of arsenic-contaminated "Morinaga dry-milk" in 1955: a prospective study from 1982 to 2004]	2007
3048	J. L. Zhao	[Effects of controlled-release arsenic-trioxide-eluting stents on in-stent neointimal hyperplasia in coronary artery: experiment with dog model]	2007
3049	B. Claus Henn	Maternal and Cord Blood Manganese Concentrations and Early Childhood Neurodevelopment among Residents near a Mining-Impacted Superfund Site	2017
3050	X. Yang	Characterization of Two Highly Arsenic-Resistant Caulobacteraceae Strains of Brevundimonas nasdae: Discovery of a New Arsenic Resistance Determinant	2022
3051	J. Li	A bioactive probe for glutathione-dependent antioxidant capacity in breast cancer patients: implications in measuring biological effects of arsenic compounds	2014
3052	S. Dastgiri	Arsenic exposure, dermatological lesions, hypertension, and chromosomal abnormalities among people in a rural community of northwest Iran	2010
3053	K. Schmid	[Internal hazardous substance burden of persons from various regions of origin--studies of lead, mercury, arsenic and cadmium exposure]	1996
3054	K. Krüger	Effects of monomethylarsonic and monomethylarsonous acid on evoked synaptic potentials in hippocampal slices of adult and young rats	2009
3055	M. Costa	DNA-protein cross-links produced by various chemicals in cultured human lymphoma cells	1997
3056	T. Kang	Arsenic sulfide induces RAG1-dependent DNA damage for cell killing by inhibiting NFATc3 in gastric cancer cells	2019
3057	M. Pizzato	Lv4 Is a Capsid-Specific Antiviral Activity in Human Blood Cells That Restricts Viruses of the SIVMAC/SIVSM/HIV-2 Lineage Prior to Integration	2015
3058	Â. Saito	Human Regulatory Protein Ki-1/57 Is a Target of SUMOylation and Affects PML Nuclear Body Formation	2017

3059	X. Zhang	Dual effects of arsenic trioxide on tumor cells and the potential underlying mechanisms	2018
3060	X. Wan	New experimental evidence for mechanism of arrhythmogenic membrane potential alternans based on balance of electrogenic I(NCX)/I(Ca) currents	2012
3061	W. L. Xu	[Effect of YB-1 gene knockdown on human leukemia cell line K562/A02]	2009
3062	L. X. Liu	[The effect and mechanism of arsenic trioxide on hepatocellular carcinoma]	2005
3063	X. Mao	Titania immobilized polypropylene hollow fiber as a disposable coating for stir bar sorptive extraction-high performance liquid chromatography-inductively coupled plasma mass spectrometry speciation of arsenic in chicken tissues	2011
3064	S. Akatsu	THE RESISTANCE OF SPIROCHETES TO THE ACTION OF HEXAMETHYLENETETRAMINE DERIVATIVES AND MERCURIAL AND ARSENIC COMPOUNDS	1917
3065	C. Della Torre	Interaction of ABC transport proteins with toxic metals at the level of gene and transport activity in the PLHC-1 fish cell line	2012
3066	K. E. King	Geographic clustering of elevated blood heavy metal levels in pregnant women	2015
3067	C. Xu	Serum nickel is associated with craniosynostosis risk: Evidence from humans and mice	2021
3068	C. Gao	Clinical pharmacokinetics and safety profile of single agent arsenic trioxide by continuous slow-rate infusion in patients with newly diagnosed acute promyelocytic leukemia	2018
3069	X. M. Liu	Possible roles of a tumor suppressor gene PIG11 in hepatocarcinogenesis and As2O3-induced apoptosis in liver cancer cells	2009
3070	G. Zhong	The protective role of autophagy against arsenic trioxide-induced cytotoxicity and ROS-dependent pyroptosis in NCTC-1469 cells	2021
3071	H. L. Shen	[Influence of YB-1 protein on the biological behaviour in K562/A02 cells]	2011
3072	J. Yuan	Enhanced GRP78 protein expression via the IRE1α/ASK1/p38 MAPK pathway during As(2)O(3)-induced endoplasmic reticulum stress in BEAS-2B cells	2021
3073	R. K. Manthari	Arsenic induces autophagy in developmental mouse cerebral cortex and hippocampus by inhibiting PI3K/Akt/mTOR signaling pathway: involvement of blood-brain barrier's tight junction proteins	2018
3074	Y. Cheng	Long-term prognosis of childhood acute promyelocytic leukaemia with arsenic trioxide administration in induction and consolidation chemotherapy phases: a single-centre experience	2013
3075	F. Minichilli	[State of health of populations residing in geothermal areas of Tuscany]	2012
3076		CADTH Rapid Response Reports	2019

3077	X. Liu	Reference Values of 14 Serum Trace Elements for Pregnant Chinese Women: A Cross-Sectional Study in the China Nutrition and Health Survey 2010-2012	2017
3078	J. Domínguez-Álvarez	Capillary electrophoresis coupled to electrospray mass spectrometry for the determination of organic and inorganic arsenic compounds in water samples	2020
3079	J. J. Zhao	[As(2)O(3) Up-Regulates the Proportion of CD4(+)CD25(+)CD127(low) Tregs in Peripheral Blood of Patients with Severe Aplastic Anemia]	2018
3080	Y. Lu	A novel RGDyC/PEG co-modified PAMAM dendrimer-loaded arsenic trioxide of glioma targeting delivery system	2018
3081	Z. Y. Wang	Nanosized As2O3/Fe2O3 complexes combined with magnetic fluid hyperthermia selectively target liver cancer cells	2009
3082	H. L. Shen	[Inhibitory effect of WT1 gene isoform transfection on proliferation of leukemia cell line NB4]	2006
3083	Z. Y. Wang	A study on the preparation and characterization of plasmid DNA and drug-containing magnetic nanoliposomes for the treatment of tumors	2011
3084	Y. X. Zhang	[Construction of subtracted cDNA library in human Jurkat T cell line induced by arsenic trioxide in vitro]	2003
3085	M. Mania	Total and inorganic arsenic in fish, seafood and seaweeds--exposure assessment	2015
3086	A. Ahmed-Ouameur	The effects of drug complexation on the stability and conformation of human serum albumin: protein unfolding	2006
3087	W. Y. Mo	Fermented food waste for culturing jade perch and Nile tilapia: Growth performance and health risk assessment based on metal/loids	2019
3088	M. Mania	Exposure assessment of the population in Poland to the toxic effects of arsenic compounds present in rice and rice based products	2017
3089	Y. Hu	[The regulation mechanism of protein kinase Cδ on arsenic liver injury caused by coal-burning]	2013
3090	M. L. Yao	[The establishment of the arsenic poisoning rats model caused by corn flour baked by high-arsenic coal]	2013
3091		Final report on the safety assessment of capsicum annuum extract, capsicum annuum fruit extract, capsicum annuum resin, capsicum annuum fruit powder, capsicum frutescens fruit, capsicum frutescens fruit extract, capsicum frutescens resin, and capsaicin	2007
3092	E. Amaya	Placental concentrations of heavy metals in a mother–child cohort	2013
3093	H. Huang	Cord serum elementomics profiling of 56 elements depicts risk of preterm birth: Evidence from a prospective birth cohort in rural Bangladesh	2021
3094	A. Ramu Ganesan	A comparison of nutritional value of underexploited edible seaweeds with recommended dietary allowances	2020

3095	K. Li	Associations between blood heavy metal(loid)s and serum heme oxygenase-1 in pregnant women: Do their distribution patterns matter?	2021
3096	W. Jay Christian	Distribution of urinary selenium and arsenic among pregnant women exposed to arsenic in drinking water	2006
3097	X. Jia	Associations between endocrine-disrupting heavy metals in maternal hair and gestational diabetes mellitus: A nested case-control study in China	2021
3098	M. L. Rahman	Early pregnancy exposure to metal mixture and birth outcomes – A prospective study in Project Viva	2021
3099	Q. Zhang	Joint effect of urinary arsenic species and serum one-carbon metabolism nutrients on gestational diabetes mellitus: A cross-sectional study of Chinese pregnant women	2021
3100	I. Neamtiu	Pregnant women in Timis County, Romania are exposed primarily to low-level (<10μg/l) arsenic through residential drinking water consumption	2015
3101	M. A. Castallo	Quinine iodobismuthate in the treatment of syphilis complicating pregnancy	1938
3102	A. Li	Heavy metals in maternal and cord blood in Beijing and their efficiency of placental transfer	2019
3103	Y. Wang	Multiple metal concentrations and gestational diabetes mellitus in Taiyuan, China	2019
3104	Z. Drobná	Analysis of maternal polymorphisms in arsenic (+3 oxidation state)-methyltransferase AS3MT and fetal sex in relation to arsenic metabolism and infant birth outcomes: Implications for risk analysis	2016
3105	K. Okamura	Gestational arsenite exposure augments hepatic tumors of C3H mice by promoting senescence in F1 and F2 offspring via different pathways	2020
3106	D. Mazurek	The concentration of selected elements in the placenta according to selected sociodemographic factors and their effect on birth mass and birth length of newborns	2020
3107	C. V. Watson	Characterization of trace elements exposure in pregnant women in the United States, NHANES 1999–2016	2020
3108	M. Ren	Associations between hair levels of trace elements and the risk of preterm birth among pregnant women: A prospective nested case-control study in Beijing Birth Cohort (BBC), China	2022
3109	Y. Wang	Effects of prenatal exposure to arsenic on neonatal birth size in Wujiang, China	2022
3110	H. T. Davis	Potential sources and racial disparities in the residential distribution of soil arsenic and lead among pregnant women	2016
3111	C. L. Moore	Developmental neurotoxicity of inorganic arsenic exposure in Sprague-Dawley rats	2019
3112	H. I. Kantor	Arsenical enoephalopathy in pregnancy with recovery	1948

3113	M. A. H. El-Baz	Environmental factors and apoptotic indices in patients with intrauterine growth retardation: A nested case-control study	2015
3114	W. T. Daily	Syphilis in pregnancy	1937
3115	A. C. Callan	Maternal exposure to metals—Concentrations and predictors of exposure	2013
3116	Y. Dai	Early-life exposure to widespread environmental toxicants and maternal-fetal health risk: A focus on metabolomic biomarkers	2020
3117	K. S. Almberg	Arsenic in drinking water and adverse birth outcomes in Ohio	2017
3118	F. R. Minnich	Treatment of Syphilis in Pregnancy: A Comparison of Arsenicals	1941
3119	E. Akhtar	A longitudinal study of rural Bangladeshi children with long-term arsenic and cadmium exposures and biomarkers of cardiometabolic diseases	2021
3120	A. Sulimanec Grgec	Potential risks and health benefits of fish in the diet during the childbearing period: Focus on trace elements and n-3 fatty acid content in commonly consumed fish species from the Adriatic Sea	2022
3121	H. B. Röllin	Evaluation of in utero exposure to arsenic in South Africa	2017
3122	C. M. Aelion	Associations of estimated residential soil arsenic and lead concentrations and community-level environmental measures with mother–child health conditions in South Carolina	2012
3123	C. Kuehn	Arsenical encephalitis during pregnancy	1938
3124	E. D. Plass	Hemorrhagic encephalitis (neoarsphenamine) in obstetric patients	1935
3125	K. Nohara	Gestational arsenic exposure and paternal intergenerational epigenetic inheritance	2020
3126	S. Comess	Exposure to atmospheric metals using moss bioindicators and neonatal health outcomes in Portland, Oregon	2021
3127	C. Wang	Maternal exposure to heavy metals and risk for severe congenital heart defects in offspring	2022
3128	M. Sobolewski	Endocrine active metals, prenatal stress and enhanced neurobehavioral disruption	2018
3129	H. M. Angelucci	Myelogenous leucemia complicating pregnancy	1944
3130	K. S. Engström	Chronic exposure to cadmium and arsenic strongly influences concentrations of 8-oxo-7,8-dihydro-2′-deoxyguanosine in urine	2010

3131	C.-S. Li	Sodium arsenite inhibits migration of extravillous trophoblast cells in vitro	2007
3132	C. Liang	Domain- and sex-specific effects of prenatal exposure to low levels of arsenic on children's development at 6 months of age: Findings from the Ma'anshan birth cohort study in China	2020
3133	C. Freire	Placental metal concentrations and birth outcomes: The Environment and Childhood (INMA) project	2019
3134	M. Kippler	Elevated childhood exposure to arsenic despite reduced drinking water concentrations — A longitudinal cohort study in rural Bangladesh	2016
3135	T.-L. Tsai	Maternal and childhood exposure to inorganic arsenic and airway allergy – A 15-Year birth cohort follow-up study	2021
3136	L. Song	Association of prenatal exposure to arsenic with newborn telomere length: Results from a birth cohort study	2019
3137	H. Rattner	The Treatment of Syphilis in Pregnancy by the Five-Day Massive Dose Method	1943
3138	N. Sakai	Source profiling of arsenic and heavy metals in the Selangor River basin and their maternal and cord blood levels in Selangor State, Malaysia	2017
3139	E. J. Dashner-Titus	Metal exposure and oxidative stress markers in pregnant Navajo Birth Cohort Study participants	2018
3140	K. Miyazaki	The effects of gestational arsenic exposure and dietary selenium deficiency on selenium and selenoenzymes in maternal and fetal tissues in mice	2005
3141	E. D. Plass	Syphilis in obstetrics	1942
3142	A. Malin Igra	Environmental metal exposure and growth to 10 years of age in a longitudinal mother–child cohort in rural Bangladesh	2021
3143	Q. Guo	Effect of arsenic and/or fluoride gestational exposure on renal autophagy in offspring mice	2020
3144	F. Ruan	Association between prenatal exposure to metal mixtures and early childhood allergic diseases	2022
3145	I. Karakis	Exposure to metals and congenital anomalies: A biomonitoring study of pregnant Bedouin-Arab women	2015
3146	N. H. Orak	A Hybrid Bayesian Network Framework for Risk Assessment of Arsenic Exposure and Adverse Reproductive Outcomes	2020
3147	R. P. Parajuli	Association of cord blood levels of lead, arsenic, and zinc with neurodevelopmental indicators in newborns: A birth cohort study in Chitwan Valley, Nepal	2013
3148	Y. Yu	Recommended acceptable levels of maternal serum typical toxic metals from the perspective of spontaneous preterm birth in Shanxi Province, China	2019

3149	C. M. Bulka	Arsenic in private well water and birth outcomes in the United States	2022
3150	X. Li	Effect of gestational exposure to arsenic on puberty in offspring female mice	2018
3151	H. E. Laue	Nutrient-toxic element mixtures and the early postnatal gut microbiome in a United States longitudinal birth cohort	2020
3152	W. T. Daily	Syphilis complicating pregnancy	1938
3153	M.-S. Tsai	Children's environmental health based on birth cohort studies of Asia (2) – air pollution, pesticides, and heavy metals	2019
3154	V. Tsang	The epigenetic effects of a high prenatal folate intake in male mouse fetuses exposed in utero to arsenic	2012
3155	X. Pi	Concentrations of selected heavy metals in placental tissues and risk for neonatal orofacial clefts	2018
3156	K. M. Wai	Impact of prenatal heavy metal exposure on newborn leucocyte telomere length: A birth-cohort study	2018
3157	K. M. Khan	Health effects of arsenic exposure in Latin America: An overview of the past eight years of research	2020
3158	J. Ashley-Martin	Blood metal levels and early childhood anthropometric measures in a cohort of Canadian children	2019
3159	M. S. Bloom	Low level arsenic contaminated water consumption and birth outcomes in Romania—An exploratory study	2016
3160	A. K. Bozack	Cord blood DNA methylation of DNMT3A mediates the association between in utero arsenic exposure and birth outcomes: Results from a prospective birth cohort in Bangladesh	2020
3161	A. Abuawad	Nutrition, one-carbon metabolism and arsenic methylation	2021
3162	C. Marie	In utero exposure to arsenic in tap water and congenital anomalies: A French semi-ecological study	2018
3163	I. Al-Saleh	Heavy metals (lead, cadmium, methylmercury, arsenic) in commonly imported rice grains (Oryza sativa) sold in Saudi Arabia and their potential health risk	2017
3164	Y.-M. Hsueh	The combined effects of nucleotide-binding domain-like receptor protein 3 polymorphisms and levels of blood lead on developmental delays in preschool children	2022
3165	J. Snoj Tratnik	Results of the first national human biomonitoring in Slovenia: Trace elements in men and lactating women, predictors of exposure and reference values	2019
3166	S. F. Farzan	Arsenic exposure from drinking water and endothelial dysfunction in Bangladeshi adolescents	2022

3167	A. Vriens	Neonatal exposure to environmental pollutants and placental mitochondrial DNA content: A multi-pollutant approach	2017
3168	M. A. Sabino	The role of tropical small-scale fisheries in trace element delivery for a Small Island Developing State community, the Seychelles	2022
3169	G. Tindula	Parental metal exposures as potential risk factors for spina bifida in Bangladesh	2021
3170	P. Kaushal	Ameliorative role of antioxidant supplementation on sodium-arsenite induced adverse effects on the developing rat cerebellum	2020
3171	M. García-Villarino	Exposure to metal mixture and growth indicators at 4–5 years. A study in the INMA-Asturias cohort	2022
3172	M. Sakamoto	Changes in body burden of mercury, lead, arsenic, cadmium and selenium in infants during early lactation in comparison with placental transfer	2012
3173	S. Peng	Short-term exposure to fine particulate matter and its constituents may affect renal function via oxidative stress: A longitudinal panel study	2022
3174	D. S. Hill	Arsenate-induced maternal glucose intolerance and neural tube defects in a mouse model	2009
3175	A. Toolabi	Spatial distribution, occurrence, and health risk assessment of nitrate, fluoride, and arsenic in Bam groundwater resource, Iran	2021
3176	S. L. Steinhausen	Heavy metals in fish nearby electronic waste may threaten consumer's health. Examples from Accra, Ghana	2022
3177	O. Laurent	Sources and contents of air pollution affecting term low birth weight in Los Angeles County, California, 2001–2008	2014
3178	S. Casteel	Refining the risk assessment of metal-contaminated soils	2001
3179	K. Mar Wai	Arsenic exposure through drinking Water and oxidative stress Status: A cross-sectional study in the Ayeyarwady region, Myanmar	2019
3180	E. Maeda	Associations of environmental exposures to methylmercury and selenium with female infertility: A case–control study	2019
3181	A. Miklavčič	Mercury, arsenic and selenium exposure levels in relation to fish consumption in the Mediterranean area	2013
3182	E. F. Winterbottom	GLI3 Links Environmental Arsenic Exposure and Human Fetal Growth	2015
3183	H. Perveen	Dietary CCPS from bitter gourd attenuates sodium arsenite induced female reproductive ailments cum infertility in wistar rats: anti-inflammatory and anti-apoptotic role	2019
3184	J. E. Laine	A Systems Toxicology-based Approach Reveals Biological Pathways Dysregulated by Prenatal Arsenic Exposure	2016

3185	H. Skröder	Kidney function and blood pressure in preschool-aged children exposed to cadmium and arsenic - potential alleviation by selenium	2015
3186	M. R. R. F. Abdelmale	Scanning electron microscopic study of the effect of chlorpyrifos on the developing neural tube in comparison with Arsenic in mouse embryo	2016
3187	C. Marjorie Aelion	Use of a general toxicity test to predict heavy metal concentrations in residential soils	2007
3188	A. Stajnko	Trace elements and ALAD gene polymorphisms in general population from three uranium legacy sites – A case study in Kyrgyzstan	2020
3189	C.-W. Chang	Significant association between blood lead (Pb) level and haemoglobin A1c in non-diabetic population	2021
3190	S. Tabacova	Developmental Toxicity of Inorganic Arsenic in Whole Embryo Culture: Oxidation State, Dose, Time, and Gestational Age Dependence	1996
3191	P. Chen	Arsenic exposure during juvenile and puberty significantly affected reproductive system development of female SD rats	2022
3192	W. Xia	Spatial and interspecies differences in concentrations of eight trace elements in wild freshwater fishes at different trophic levels from middle and eastern China	2019
3193	C.-C. Kuo	Early-life arsenic exposure promotes atherogenic lipid metabolism in adolescence: A 15-year birth cohort follow-up study in central Taiwan	2018
3194	R. K. Kwok	Drinking water arsenic exposure and blood pressure in healthy women of reproductive age in Inner Mongolia, China	2007
3195	F. Parvez	Arsenic exposures alter clinical indicators of anemia in a male population of smokers and non-smokers in Bangladesh	2017
3196	L. Jin	Placental concentrations of mercury, lead, cadmium, and arsenic and the risk of neural tube defects in a Chinese population	2013
3197	B. Wang	Prenatal exposure to arsenic and neurobehavioral development of newborns in China	2018
3198	F. Richter	Maternal exposure to arsenic in drinking water and risk of congenital heart disease in the offspring	2022
3199	R.-L. Hsieh	Relation of polymorphism of arsenic metabolism genes to arsenic methylation capacity and developmental delay in preschool children in Taiwan	2017
3200	M. N. Llanos	Fetal growth restriction is related to placental levels of cadmium, lead and arsenic but not with antioxidant activities	2009
3201	J. F. Rasco	Effects of maternal restraint stress and sodium arsenate in mice	1994
3202	F. Ferraris	Essential and toxic elements in sustainable and underutilized seafood species and derived semi-industrial ready-to-eat products	2021

3203	M. A. Deyssenroth	Intrauterine multi-metal exposure is associated with reduced fetal growth through modulation of the placental gene network	2018
3204	G. V. Iyengar	Global outlook on nutrition and the environment: meeting the challenges of the next millennium	2000
3205	Z. Ceja-Galicia	Leptin and adiponectin synthesis and secretion in mature 3T3-L1 adipocytes are differentially down-regulated by arsenic and palmitic acid exposure throughout different stages of adipogenesis	2022
3206	H. Wang	TNF-α derived from arsenite-induced microglia activation mediated neuronal necroptosis	2022
3207	M. M. Wolle	Speciation analysis of arsenic in prenatal and children's dietary supplements using microwave-enhanced extraction and ion chromatography–inductively coupled plasma mass spectrometry	2014
3208	Y. Amitai	High risk for neural tube defects; the role of arsenic in drinking water and rice in Asia	2018
3209	F. Samiee	Exposure to arsenic through breast milk from mothers exposed to high levels of arsenic in drinking water: Infant risk assessment	2019
3210	T. Kabir	Arsenic hampered embryonic development: An in vivo study using local Bangladeshi Danio rerio model	2020
3211	T. J. Flanigan	Neurobehavioral and neurochemical effects of perinatal arsenite exposure in Sprague-Dawley rats	2022
3212	R. L. Calderon	The epidemiology of chemical contaminants of drinking water	2000
3213	J. W. Lee	An Integrated Gaussian Graphical Model to evaluate the impact of exposures on metabolic networks	2019
3214	F. Gumilar	Locomotor activity and sensory–motor developmental alterations in rat offspring exposed to arsenic prenatally and via lactation	2015
3215	K. E. Caldwell	Prenatal arsenic exposure alters the programming of the glucocorticoid signaling system during embryonic development	2015
3216	B. Wang	Association of maternal chronic arsenic exposure with the risk of neural tube defects in Northern China	2019
3217	K. K. Caldwell	Arsenic exposure during embryonic development alters the expression of the long noncoding RNA growth arrest specific-5 (Gas5) in a sex-dependent manner	2018
3218	T. Jiang	Exposure to multiple toxic metals and the risk of early embryonic arrest among women undergoing assisted reproductive techniques	2022
3219	K. F. Rodriguez	In utero exposure to arsenite contributes to metabolic and reproductive dysfunction in male offspring of CD-1 mice	2020
3220	M. Kippler	Early life low-level cadmium exposure is positively associated with increased oxidative stress	2012

3221	L. Fábelová	Hair concentration of trace elements and growth in homeless children aged <6years: Results from the ENFAMS study	2018
3222	H. Huang	Investigation of association between environmental and socioeconomic factors and preterm birth in California	2018
3223	T. Punshon	Placental metal concentrations in relation to placental growth, efficiency and birth weight	2019
3224	F. M. Branch	Metal(loid)s and human semen quality: The LIFE Study	2021
3225	J. J. Lee	Anthropometric measures at birth and early childhood are associated with neurodevelopmental outcomes among Bangladeshi children aged 2–3years	2017
3226	A. C. F. Souza	Impact of prenatal arsenic exposure on the testes and epididymides of prepubertal rats	2021
3227	M. Rodríguez-Barranco	Postnatal arsenic exposure and attention impairment in school children	2016
3228	H.-y. Yu	Links between environmental geochemistry and rate of birth defects: Shanxi Province, China	2011
3229	G. H. O. Rocha	Trace metal levels in serum and urine of a population in southern Brazil	2016
3230	J. A. Ansari	Perinatal arsenic exposure-induced sustained microglial activation leads to impaired cognitive response in BALB/c mice	2022
3231	Y.-X. Wang	Concentrations of vanadium in urine and seminal plasma in relation to semen quality parameters, spermatozoa DNA damage and serum hormone levels	2018
3232	C. C. Willhite	Arsenic-induced axial skeletal (dysraphic) disorders	1981
3233	V. H. Ferm	Synergistic teratogenic effects of arsenic and hyperthermia in hamsters	1977
3234	J. Burger	Heavy metals in fish from the Aleutians: Interspecific and locational differences	2014
3235	P. Phookphan	Hypomethylation of inflammatory genes (COX2, EGR1, and SOCS3) and increased urinary 8-nitroguanine in arsenic-exposed newborns and children	2017
3236	N. E. Tinkelman	Associations of maternal arsenic exposure with adult fasting glucose and insulin resistance in the Strong Heart Study and Strong Heart Family Study	2020
3237	L. Friberg	The GESAMP evaluation of potentially harmful substances in fish and other seafood with special reference to carcinogenic substances	1988
3238	R. P. Parajuli	Home environment and cord blood levels of lead, arsenic, and zinc on neurodevelopment of 24 months children living in Chitwan Valley, Nepal	2015

3239	G. M. Nelson	Transcriptional changes associated with reduced spontaneous liver tumor incidence in mice chronically exposed to high dose arsenic	2009
3240	T. Zhang	Relationships between urinary antimony concentrations and depressive symptoms in adults	2022
3241	L. López-Carrillo	Inorganic arsenic methylation capacity and breast cancer by immunohistochemical subtypes in northern Mexican women	2020
3242	M. Sakamoto	Mercury and heavy metal profiles of maternal and umbilical cord RBCs in Japanese population	2010
3243	M. A. Baki	Concentration of heavy metals in seafood (fishes, shrimp, lobster and crabs) and human health assessment in Saint Martin Island, Bangladesh	2018
3244	P. Hinhumpatch	Oxidative DNA damage and repair in children exposed to low levels of arsenic in utero and during early childhood: Application of salivary and urinary biomarkers	2013
3245	G. Krishan	Occurrences of potentially toxic trace metals in groundwater of the state of Punjab in northern India	2021
3246	C. D. Butts	Seafood consumption is associated with higher follicular fluid arsenic (As) and mercury (Hg) concentrations in women undergoing in vitro fertilization (IVF)	2020
3247	D. A. Parodi	Alteration of mammary gland development and gene expression by in utero exposure to arsenic	2015
3248	B. van Wendel de Joode	Manganese concentrations in drinking water from villages near banana plantations with aerial mancozeb spraying in Costa Rica: Results from the Infants' Environmental Health Study (ISA)	2016
3249	M. Tian	Environmental doses of arsenic exposure are associated with increased reproductive-age male urinary hormone excretion and in vitro Leydig cell steroidogenesis	2021
3250	G. Koppen	Pooled analysis of genotoxicity markers in relation to exposure in the Flemish Environment and Health Studies (FLEHS) between 1999 and 2018	2020
3251	T. R. Sanchez	An atlas of metallome and metabolome interactions and associations with incident diabetes in the Strong Heart Family Study	2021
3252	S. Huang	Associations of multiple plasma metals with the risk of metabolic syndrome: A cross-sectional study in the mid-aged and older population of China	2022
3253	R. N. Alves	Oral bioaccessibility of toxic and essential elements in raw and cooked commercial seafood species available in European markets	2018
3254	P. Brooke Bland	The investigation of a new pentavalent arsenical, aldarsone, in the treatment of trichomonas vaginitis	1936
3255	T. Wang	Associations of plasma multiple metals with risk of hyperuricemia: A cross-sectional study in a mid-aged and older population of China	2022
3256	P. Mukhopadhyay	Impact of prenatal arsenate exposure on gene expression in a pure population of migratory cranial neural crest cells	2019

3257	X. Lai	Individual and joint associations of co-exposure to multiple plasma metals with telomere length among middle-aged and older Chinese in the Dongfeng-Tongji cohort	2022
3258	L. Smeester	Chronic early childhood exposure to arsenic is associated with a TNF-mediated proteomic signaling response	2017
3259	D. Unis	Arsenite exposure compromises early embryonic development in the Golden hamster	2009
3260	C. Salazar-Camacho	A human health risk assessment of methylmercury, arsenic and metals in a tropical river basin impacted by gold mining in the Colombian Pacific region	2022
3261	P. Kaushal	Dendritic processes as targets for arsenic induced neurotoxicity: Protective role of curcumin	2018
3262	K. Cai	Cycling and total risks of multiple As fractions in the Beijing–Tianjin–Hebei area on the agricultural plain, China	2020
3263	M. F. Hughes	Research approaches to address uncertainties in the risk assessment of arsenic in drinking water	2007
3264	M.-J. Hu	Thyroid hormones in relation to polybrominated diphenyl ether and metals exposure among rural adult residents along the Yangtze River, China	2021
3265	N. González	Dietary intake of arsenic, cadmium, mercury and lead by the population of Catalonia, Spain: Analysis of the temporal trend	2019
3266	S. M. Young	Human placenta processed for encapsulation contains modest concentrations of 14 trace minerals and elements	2016
3267	F. Samiee	Exposure to heavy metals released to the environment through breastfeeding: A probabilistic risk estimation	2019
3268	J. Wang	Arsenic in outdoor air particulate matter in China: Tiered study and implications for human exposure potential	2020
3269	K. Granby	Growth performance, bioavailability of toxic and essential elements and nutrients, and biofortification of iodine of rainbow trout (Onchorynchus mykiss) fed blends with sugar kelp (Saccharina latissima)	2020
3270	J. De Loma	Arsenite methyltransferase (AS3MT) polymorphisms and arsenic methylation in children in rural Bangladesh	2018
3271	D. Kaya-Akyüzlü	Does maternal MDR1 C1236T polymorphism have an effect on placental arsenic levels?	2016
3272	E. Dartey	Essential and non-essential trace elements among working populations in Ghana	2017
3273	V. K. Nguyen	A comprehensive analysis of racial disparities in chemical biomarker concentrations in United States women, 1999–2014	2020
3274	J. F. Robinson	Arsenic- and cadmium-induced toxicogenomic response in mouse embryos undergoing neurulation	2011

3275	R. P. Parajuli	Home environment and prenatal exposure to lead, arsenic and zinc on the neurodevelopment of six-month-old infants living in Chitwan Valley, Nepal	2014
3276	C. Liang	Exposure to multiple toxic metals and polycystic ovary syndrome risk: Endocrine disrupting effect from As, Pb and Ba	2022
3277	G. Izydorczyk	Hair mineral analysis in the population of students living in the Lower Silesia region (Poland) in 2019: Comparison with biomonitoring study in 2009 and literature data	2021
3278	P. L. C. M. van Riel	Metals	1991
3279	K. Hobbie	Use of study-specific MOE-like estimates to prioritize health effects from chemical exposure for analysis in human health assessments	2020
3280	C. M. Aelion	Soil metal concentrations and toxicity: Associations with distances to industrial facilities and implications for human health	2009
3281	A. M. Bolt	Arsenite exposure in human lymphoblastoid cell lines induces autophagy and coordinated induction of lysosomal genes	2010
3282	J. A. Ansari	Minocycline reverses developmental arsenic exposure-induced microglia activation and functional alteration in BALB/c mice	2022
3283	M. M. Oliveira	Essential and toxic elements in human milk concentrate with human milk lyophilizate: A preclinical study	2020
3284	Z.-Z. Wan	Metal/metalloid levels in urine and seminal plasma in relation to computer-aided sperm analysis motion parameters	2019
3285	H. Wu	Developmental arsenic exposure induces dysbiosis of gut microbiota and disruption of plasma metabolites in mice	2022
3286	G. Ponce	Determination of a guidance value for the communication of individual-level biomonitoring data for urinary arsenic	2022
3287	A. Banerjee	High arsenic tolerance in Brevundimonas aurantiaca PFAB1 from an arsenic-rich Indian hot spring	2021
3288	H.-H. Chao	Arsenic, Cadmium, Lead, and Aluminium Concentrations in Human Milk at Early Stages of Lactation	2014
3289	N. González	Dietary exposure to total and inorganic arsenic via rice and rice-based products consumption	2020
3290	L. Garcia Barcia	Mercury and arsenic in processed fins from nine of the most traded shark species in the Hong Kong and China dried seafood markets: The potential health risks of shark fin soup	2020
3291	C. K. Singh	Arsenic contamination in Rapti River Basin, Terai region of India	2018
3292	M. Vahter	Metals and Women's Health	2002

3293	S. A. Niño	Life-long arsenic exposure damages the microstructure of the rat hippocampus	2022
3294	L. Joca	Systematic review of differential inorganic arsenic exposure in minority, low-income, and indigenous populations in the United States	2016
3295	X. T. Li	Association between Plasma Metal Levels and Diabetes Risk: a Case-control Study in China	2017
3296	J. P. Goullé	Un nouveau moyen d'investigation biologique: l'analyse des cheveux. Intérêt en pratique médicale	1996
3297	Y. Chen	Early life and adolescent arsenic exposure from drinking water and blood pressure in adolescence	2019
3298	F. Ligate	Geogenic contaminants and groundwater quality around Lake Victoria goldfields in northwestern Tanzania	2022
3299	S. V. Flanagan	Arsenic in private well water part 1 of 3: Impact of the New Jersey Private Well Testing Act on household testing and mitigation behavior	2016
3300	Q. Wang	Occurrence and health risk assessment of residual heavy metals in the Chinese mitten crab (Eriocheir sinensis)	2021
3301	H. Skröder	Predictors of selenium biomarker kinetics in 4–9-year-old Bangladeshi children	2018
3302	D. Beene	A mass-balance approach to evaluate arsenic intake and excretion in different populations	2022
3303	S. S. Ameer	The effects of arsenic exposure on blood pressure and early risk markers of cardiovascular disease: Evidence for population differences	2015
3304	R.-L. Hsieh	Arsenic methylation capacity and developmental delay in preschool children in Taiwan	2014
3305	M. Gamberg	Contaminants in two West Greenland caribou populations	2016
3306	Z. Akram	Adverse effects of arsenic exposure on uterine function and structure in female rat	2010
3307	N. J. Eastman	The arsenic content of the human placenta following arsphenamine therapy	1931
3308	Y. He	Heavy metal exposure, oxidative stress and semen quality: Exploring associations and mediation effects in reproductive-aged men	2020
3309	X. Liu	In situ analysis of variations of arsenicals, microbiome and transcriptome profiles along murine intestinal tract	2022
3310	S. I. Alekseenko	Mucociliary transport as a link between chronic rhinosinusitis and trace element dysbalance	2019

3311	L. Dai	Elevated whole blood arsenic level is associated with type 2 diabetes in coal-burning areas in Guizhou	2020
3312	C. R. Tyler	Fluoxetine treatment ameliorates depression induced by perinatal arsenic exposure via a neurogenic mechanism	2014
3313	T. K. A. Tengku Nur Alia	Comparative study of raw and cooked farmed sea bass (Lates calcarifer) in relation to metal content and its estimated human health risk	2020
3314	M. Cornejo	Arsenic trioxide-increased MDCK cells proliferation requires activator protein 1-mediated increase of the sodium/proton exchanger 1 activity	2021
3315	E. Solgi	Zoning and human health risk assessment of arsenic and nitrate contamination in groundwater of agricultural areas of the twenty two village with geostatistics (Case study: Chahardoli Plain of Qorveh, Kurdistan Province, Iran)	2021
3316	P. Dhar	Preliminary morphological and morphometric study of rat cerebellum following sodium arsenite exposure during rapid brain growth (RBG) period	2007
3317	E. Haque	Validation of blood arsenic and manganese assessment from archived clotted erythrocyte fraction in an urban cohort of mother-child dyads	2022
3318	F. Chen	Sub-chronic low-dose arsenic in rice exposure induces gut microbiome perturbations in mice	2021
3319	B. Nemery	Assessing exposure to metals using biomonitoring: Achievements and challenges experienced through surveys in low- and middle-income countries	2018
3320	K. Ljung	Metal and arsenic distribution in soil particle sizes relevant to soil ingestion by children	2006
3321	S. W. Al-Rmalli	Betel quid chewing elevates human exposure to arsenic, cadmium and lead	2011
3322	K. Kordas	Multiple-metal exposure, diet, and oxidative stress in Uruguayan school children	2018
3323	M. M. Rahman	The magnitude of arsenic contamination in groundwater and its health effects to the inhabitants of the Jalangi—one of the 85 arsenic affected blocks in West Bengal, India	2005
3324	Kiran	Effect of heavy metals: An overview	2022
3325	A. D. Monnot	Risks associated with arsenic exposure resulting from the consumption of California wines sold in the United States	2016
3326	R. A. Rocha	Arsenic and fluoride induce neural progenitor cell apoptosis	2011
3327	E. Doménech	Formulation and application of the probability of exceedance metric for risk characterization of non-threshold chemical hazards in food	2021
3328	M. Topal	Investigation of potential health risks in terms of arsenic in grapevine exposed to gallery waters of an abandoned mining area in Turkey	2020

3329	P. Kumarathilaka	Rice genotype's responses to arsenic stress and cancer risk: The effects of integrated birnessite-modified rice hull biochar-water management applications	2021
3330	A. J. Signes-Pastor	Inorganic arsenic exposure and neuropsychological development of children of 4–5 years of age living in Spain	2019
3331	S. Sannadi	Reversal effect of monoisoamyl dimercaptosuccinic acid (MiADMSA) for arsenic and lead induced perturbations in apoptosis and antioxidant enzymes in developing rat brain	2013
3332	M. R. Garry	In utero arsenic exposure in mice and early life susceptibility to cancer	2015
3333	L. Fishbein	Natural non-nutrient substances in the food chain	1972
3334	S. C. Santra	Arsenic in Foodchain and Community Health Risk: A Study in Gangetic West Bengal	2013
3335	R. Hu	A scientometric and visualization analysis of studies on arsenic for acute promyelocytic leukemia	2022
3336	Y.-X. Wang	Relationships between seminal plasma metals/metalloids and semen quality, sperm apoptosis and DNA integrity	2017
3337	E. Begu	Simultaneous separation of arsenic and cadmium from interfering salt matrix of multivitamin/mineral supplements by sequential coprecipitation and determination by inductively coupled plasma mass spectrometry	2019
3338	H. Correia	Seaweeds rehydration and boiling: Impact on iodine, sodium, potassium, selenium, and total arsenic contents and health benefits for consumption	2021
3339	L. López-Carrillo	Arsenic methylation capacity is associated with breast cancer in northern Mexico	2014
3340	R. Akter	Effect of Allium sativum methanol extract in amelioration of arsenic-induced toxicity in Swiss albino mice	2022
3341	M. López-Alonso	Use of dogs as indicators of metal exposure in rural and urban habitats in NW Spain	2007
3342	M. Wilhelm	Dietary intake of arsenic, mercury and selenium by children from a German North Sea island using duplicate portion sampling	2003
3343	R. Hayder	Challenges for sustainable water use in the northern part of Pakistan focusing on hydrology assessment of non-industrial zone	2022
3344	P. Olmedo	Determination of toxic elements (mercury, cadmium, lead, tin and arsenic) in fish and shellfish samples. Risk assessment for the consumers	2013
3345	K. Yang	Low- and moderate- levels of arsenic exposure in young adulthood and incidence of chronic kidney disease: Findings from the CARDIA Trace Element Study	2021
3346	J. Burger	Heavy metals in commercial fish in New Jersey	2005

3347	J. Yang	Arsenic burden in e-waste recycling workers – A cross-sectional study at the Agbogbloshie e-waste recycling site, Ghana	2020
3348	K. M. Khan	Thyroid hormones and neurobehavioral functions among adolescents chronically exposed to groundwater with geogenic arsenic in Bangladesh	2019
3349	D. Zuzolo	Arsenic: Geochemical distribution and age-related health risk in Italy	2020
3350	G. Schoeters	Three cycles of human biomonitoring in Flanders − Time trends observed in the Flemish Environment and Health Study	2017
3351	C. Bao	Health risk assessment of arsenic and some heavy metals in the edible crab (Portunus trituberculatus) collected from Hangzhou Bay, China	2021
3352	K. Kim	Associations of exposure to metal and metal mixtures with thyroid hormones: Results from the NHANES 2007–2012	2022
3353	M. H. Rahbar	The role of drinking water sources, consumption of vegetables and seafood in relation to blood arsenic concentrations of Jamaican children with and without Autism Spectrum Disorders	2012
3354	E. J. Martinez	Moderate perinatal arsenic exposure alters neuroendocrine markers associated with depression and increases depressive-like behaviors in adult mouse offspring	2008
3355	M. Kalia	Brain development: anatomy, connectivity, adaptive plasticity, and toxicity	2008
3356	K. A. Graeme	Heavy Metal Toxicity, Part I: Arsenic and Mercury	1998
3357	S. Shahriar	Concentrations of toxic elements and health risk assessment in arum grown in arsenic-contaminated areas of Bangladesh	2021
3358	C. G. Sotomayor	Plasma Lead Concentration and Risk of Late Kidney Allograft Failure: Findings From the TransplantLines Biobank and Cohort Studies	2022
3359	C. M. Aelion	Metal concentrations in rural topsoil in South Carolina: Potential for human health impact	2008
3360	Y. Suzuki	Dietary exposure to arsenic species in Japan in 2019 using a total diet study based on composite sample with market basket approach at the national level	2022
3361	B. Gamboa-Loira	Genetic susceptibility to breast cancer risk associated with inorganic arsenic exposure	2017
3362	I. Molina-Villalba	Biomonitoring of arsenic, cadmium, lead, manganese and mercury in urine and hair of children living near mining and industrial areas	2015
3363	J. F. Robinson	Methylmercury induced toxicogenomic response in C57 and SWV mouse embryos undergoing neural tube closure	2010
3364	A. M. Harvey	Clinical investigation of chronic diseases: Its successful pursuit in an outpatient setting	1980

3365	A. J. Signes-Pastor	Concentrations of urinary arsenic species in relation to rice and seafood consumption among children living in Spain	2017
3366	P. Trumbo	Dietary Reference Intakes: Vitamin A, Vitamin K, Arsenic, Boron, Chromium, Copper, Iodine, Iron, Manganese, Molybdenum, Nickel, Silicon, Vanadium, and Zinc	2001
3367	J. Wu	Association between risk of birth defects occurring level and arsenic concentrations in soils of Lvliang, Shanxi province of China	2014
3368	P. Allison	Disruption of canonical TGFβ-signaling in murine coronary progenitor cells by low level arsenic	2013
3369	J. A. R. Herrera	The burden of disease of three food-associated heavy metals in clusters in the Danish population – Towards targeted public health strategies	2021
3370	A. Rodríguez-Carrillo	Exploring the relationship between metal exposure, BDNF, and behavior in adolescent males	2022
3371	C.-C. Chang	Deleterious Effects of Arsenic, Benomyl and Carbendazim on Human Endometrial Cell Proliferation In Vitro	2010
3372	G. Souza Valasques	Extraction induced by emulsion breaking for As, Se and Hg determination in crude palm oil by vapor generation-AFS	2020
3373	B. Li	Astrocytes in heavy metal neurotoxicity and neurodegeneration	2021
3374	Z. Bao	Arsenic trioxide blocked proliferation and cardiomyocyte differentiation of human induced pluripotent stem cells: Implication in cardiac developmental toxicity	2019
3375	J. Rowan	Contribution of household drinking water intake to arsenic and lead exposure among Uruguayan schoolchildren	2022
3376	E. Olivas-Calderón	Lung inflammation biomarkers and lung function in children chronically exposed to arsenic	2015
3377	N. González	Dietary exposure to potentially toxic elements through sushi consumption in Catalonia, Spain	2021
3378	A. Ghavamzadeh	Treatment of acute promyelocytic leukemia with arsenic trioxide without ATRA and/or chemotherapy	2006
3379	S. Llop	Gender differences in the neurotoxicity of metals in children	2013
3380	O. Spiegelstein	Effects of dietary folate intake and folate binding protein-2 (Folbp2) on urinary speciation of sodium arsenate in mice	2005
3381	D. O. Carpenter	Environmental causes of violence	2010
3382	E. Junqué	Environmental and dietary determinants of metal exposure in four-year-old children from a cohort located in an industrial area (Asturias, Northern Spain)	2022

3383	A. M. Weber	Arsenic speciation in rice bran: Agronomic practices, postharvest fermentation, and human health risk assessment across the lifespan	2021
3384	M. E. Huq	Arsenic in a groundwater environment in Bangladesh: Occurrence and mobilization	2020
3385	S. L. Huang	Particle size and metals concentrations of dust from a paint manufacturing plant	2010
3386	L. Z. Crandall	Differential effects of arsenic on folate binding protein 2 (Folbp2) null and wild type fibroblasts	2002
3387	A. M. Allan	Sex-dependent effects of developmental arsenic exposure on methylation capacity and methylation regulation of the glucocorticoid receptor system in the embryonic mouse brain	2015
3388	K. Fatema	Effects of arsenic and heavy metals on metabolic pathways in cells of human origin: Similarities and differences	2021
3389	Q. Zhong	Multiple metal exposure and obesity: A prospective cohort study of adults living along the Yangtze River, China	2021
3390	R. Mohamed	Method validation and determination of heavy metals in cocoa beans and cocoa products by microwave assisted digestion technique with inductively coupled plasma mass spectrometry	2020
3391	T. Gonzalez-Cortes	DNA methylation of extracellular matrix remodeling genes in children exposed to arsenic	2017
3392	B. Owhorji	Chronic consumption of calabash chalk diet impairs locomotor activities and social behaviour in Swiss white Cd-1 mice	2019
3393	K. Yang	Low to moderate toenail arsenic levels in young adulthood and incidence of diabetes later in life: findings from the CARDIA Trace Element study	2019
3394	B. Wei	The relationships between arsenic methylation and both skin lesions and hypertension caused by chronic exposure to arsenic in drinking water	2017
3395	M. Rhonda Folio	A comparison of five toxic metals among rural and urban children	1982
3396	X. Lin	Infant exposure to trace elements in breast milk, infant formulas and complementary foods from southern China	2022
3397	P. Gomez-Rubio	Association between body mass index and arsenic methylation efficiency in adult women from southwest U.S. and northwest Mexico	2011
3398	A. Rahman	T helper 2-driven immune dysfunction in chronic arsenic-exposed individuals and its link to the features of allergic asthma	2021
3399	J. S. Tsuji	Health effect levels for risk assessment of childhood exposure to arsenic	2004
3400	C.-H. Tseng	The potential biological mechanisms of arsenic-induced diabetes mellitus	2004

3401	H. Eslami	Potentially toxic metal concentration, spatial distribution, and health risk assessment in drinking groundwater resources of southeast Iran	2022
3402	C. Zhang	Relationship between long-term exposure to low-level arsenic in drinking water and the prevalence of abnormal blood pressure	2013
3403	J. De Loma	Arsenic exposure and biomarkers for oxidative stress and telomere length in indigenous populations in Bolivia	2022
3404	Z. Kılıç	Determination of lead and copper in chewing gum samples by electrothermal-flame atomic absorption spectrometry using various chemical modifiers and arsenic by hydride generation	2002
3405	M. Calatayud	Arsenic exposure of child populations in Northern Argentina	2019
3406	Á. Mérida-Ortega	Breast cancer and urinary metal mixtures in Mexican women	2022
3407	S. M. Hays	Biomonitoring Equivalents for inorganic arsenic	2010
3408	G. Schoeters	Internal exposure of Flemish teenagers to environmental pollutants: Results of the Flemish Environment and Health Study 2016–2020 (FLEHS IV)	2022
3409	U. Mandal	Arsenic retention in cooked rice: Effects of rice type, cooking water, and indigenous cooking methods in West Bengal, India	2019
3410	E. Villalba	Geogenic arsenic contamination of wet-meadows associated with a geothermal system in an arid region and its relevance for drinking water	2020
3411	J. Shen	A comparative study of the sub-chronic toxic effects of three organic arsenical compounds on the urothelium in F344 rats; gender-based differences in response	2006
3412	E. Tolpeznikaite	Influence of fermentation on the characteristics of Baltic Sea macroalgae, including microbial profile and trace element content	2021
3413	J. E. Lancet	A phase 2 study of ATRA, arsenic trioxide, and gemtuzumab ozogamicin in patients with high-risk APL (SWOG 0535)	2020
3414	J. Jing	Changes in the synaptic structure of hippocampal neurons and impairment of spatial memory in a rat model caused by chronic arsenite exposure	2012
3415	X.-Y. Liao	Soil As contamination and its risk assessment in areas near the industrial districts of Chenzhou City, Southern China	2005
3416	S. Medina	Low level arsenite exposures suppress the development of bone marrow erythroid progenitors and result in anemia in adult male mice	2017
3417	M. d. P. T. Alves	LOW-LEVEL LASER THERAPY AFTER CARPAL TUNNEL RELEASE	2011
3418	A. Z. Pollack	Trace elements and endometriosis: The ENDO Study	2013

3419	L. M. Patel	Cutaneous signs of systemic disease	2011
3420	C. R. Tyler	Prenatal arsenic exposure alters REST/NRSF and microRNA regulators of embryonic neural stem cell fate in a sex-dependent manner	2017
3421	T. Pollock	Trends in environmental chemical concentrations in the Canadian population: Biomonitoring data from the Canadian Health Measures Survey 2007–2017	2021
3422	J. M. Goldman	Chronic Myeloid Leukemia: A Historical Perspective	2010
3423	L. Carrizales	Exposure to arsenic and lead of children living near a copper-smelter in San Luis Potosi, Mexico: Importance of soil contamination for exposure of children	2006
3424	X. Wang	Urinary metals and adipokines in midlife women: The Study of Women's Health Across the nation (SWAN)	2021
3425	D. Ferrario	Toxicity of inorganic arsenic and its metabolites on haematopoietic progenitors “in vitro”: Comparison between species and sexes	2008
3426	L. McLeod	Ecological analysis of associations between groundwater quality and hypertension and cardiovascular disease in rural Saskatchewan, Canada using Bayesian hierarchical models and administrative health data	2018
3427	M. Liu	A case-control study on the association of mineral elements exposure and thyroid tumor and goiter	2021
3428	N. Sathiakumar	Post-deepwater horizon blowout seafood consumption patterns and community-specific levels of concern for selected chemicals among children in Mobile County, Alabama	2017
3429	D. Rongvaux-Gaïda	High Response Rate and Corticosteroid Sparing with Arsenic Trioxide-Based First-Line Therapy in Chronic Graft-versus-Host Disease after Allogeneic Hematopoietic Stem Cell Transplantation	2022
3430	L. Spiezia	Short-term exposure to high levels of air pollution as a risk factor for acute isolated pulmonary embolism	2014
3431	G. A. Wasserman	Arsenic and manganese exposure and children's intellectual function	2011
3432	G. A. Wasserman	A cross-sectional study of water arsenic exposure and intellectual function in adolescence in Araihazar, Bangladesh	2018
3433	P. K. Kadeyala	Alterations in apoptotic caspases and antioxidant enzymes in arsenic exposed rat brain regions: Reversal effect of essential metals and a chelating agent	2013
3434	R. Mercorio	Effects of metal-rich particulate matter exposure on exogenous and endogenous viral sequence methylation in healthy steel-workers	2017
3435	A. C. de Groot	Dermatological drugs, topical drugs and cosmetics	1994
3436	B. Chau	Lung developmental is altered after inhalation exposure to various concentrations of calcium arsenate	2021

3437	J. M. Levengood	Elements of concern in fillets of bighead and silver carp from the Illinois River, Illinois	2014
3438	M. Vahter	Mechanisms of arsenic biotransformation	2002
3439	A.-L. Lindberg	The risk of arsenic induced skin lesions in Bangladeshi men and women is affected by arsenic metabolism and the age at first exposure	2008
3440	T. Agusa	Human exposure to arsenic from drinking water in Vietnam	2014
3441	S. V. Flanagan	Comparative case study of legislative attempts to require private well testing in New Jersey and Maine	2018
3442	S. S. Ameer	Arsenic exposure from drinking water is associated with decreased gene expression and increased DNA methylation in peripheral blood	2017
3443	N. F. Fitz	Genome-wide alteration of histone methylation profiles associated with cognitive changes in response to developmental arsenic exposure in mice	2022
3444	T. R. Sanchez	Provision of well-water treatment units to 600 households in Bangladesh: A longitudinal analysis of urinary arsenic indicates fading utility	2016
3445	R. Köhler	Protein, amino acid and mineral composition of some edible insects from Thailand	2019
3446	X. Li	Identification of an exposure risk to heavy metals from pharmaceutical-grade rubber stoppers	2017
3447	I. Baer	Performance of laboratories in speciation analysis in seafood – Case of methylmercury and inorganic arsenic	2011
3448	G. G. Kesici	Arsenic related hearing loss in miners	2016
3449	C.-T. Su	Plasma selenium influences arsenic methylation capacity and developmental delays in preschool children in Taiwan	2019
3450	M. Ackah	Soil elemental concentrations, geoaccumulation index, non-carcinogenic and carcinogenic risks in functional areas of an informal e-waste recycling area in Accra, Ghana	2019
3451	D. S. Paul	Examination of the effects of arsenic on glucose homeostasis in cell culture and animal studies: Development of a mouse model for arsenic-induced diabetes	2007
3452	Y. Li	A predictive risk model of groundwater arsenic contamination in China applied to the Huai River Basin, with a focus on the region’s cluster of elevated cancer mortalities	2017
3453	M.-A. Jones	A risk assessment approach to contaminants in Port Curtis, Queensland, Australia	2005
3454	Á. Rodríguez-Hernández	Assessment of human health hazards associated with the dietary exposure to organic and inorganic contaminants through the consumption of fishery products in Spain	2016

3455	R. S. Gibson	Changes in hair arsenic levels in breast and bottle fed infants during the first year of infancy	1982
3456	R. Goswami	Arsenic in the groundwater of the Upper Brahmaputra floodplain: Variability, health risks and potential impacts	2022
3457	S. K. Singh	An analysis of the cost-effectiveness of arsenic mitigation technologies: Implications for public policy	2017
3458	S. Miyauchi	Functional Characteristics of NaS2, a Placenta-specific Na+-coupled Transporter for Sulfate and Oxyanions of the Micronutrients Selenium and Chromium	2006
3459	H.-L. Zeng	Urinary trace elements in association with disease severity and outcome in patients with COVID-19	2021
3460	Y. Yang	Evaluating the potential health risk of toxic trace elements in vegetables: Accounting for variations in soil factors	2017
3461	L. Xu	Assessment of hypertension association with arsenic exposure from food and drinking water in Bihar, India	2021
3462	L. M. Yañez	Absorption of arsenic from soil and water by two chard (Beta vulgaris L.) varieties: A potential risk to human health	2018
3463	Z.-J. Han	Oxidative stress is implicated in arsenic-induced neural tube defects in chick embryos	2011
3464	L. M. Chiesa	Mussels and clams from the italian fish market. is there a human exposition risk to metals and arsenic?	2018
3465	R. D. Singh	Arsenic exposure causes epigenetic dysregulation of IL-8 expression leading to proneoplastic changes in kidney cells	2015
3466	M. Nicola	Egyptian blue in the Castelseprio mural painting cycle. Imaging and evidence of a non-traditional manufacture	2018
3467	T. Pedron	Mitigation of arsenic in rice grains by polishing and washing: Evidencing the benefit and the cost	2019
3468	C. Gu	Arsenite-induced transgenerational glycometabolism is associated with up-regulation of H3K4me2 via inhibiting spr-5 in caenorhabditis elegans	2020
3469	R. A. Silva	Biodegradation mechanism of arsenopyrite mine tailing with Acidithiobacillus ferrooxidans and influence of ferric supplements	2020
3470	Y. Qiu	Gut microbiota perturbations and neurodevelopmental impacts in offspring rats concurrently exposure to inorganic arsenic and fluoride	2020
3471	K. E. Nachman	Mitigating dietary arsenic exposure: Current status in the United States and recommendations for an improved path forward	2017
3472	M. N. Asadullah	Poisoning the mind: Arsenic contamination of drinking water wells and children's educational achievement in rural Bangladesh	2011

3473	J. Burger	Metal levels in flathead sole (Hippoglossoides elassodon) and great sculpin (Myoxocephalus polyacanthocephalus) from Adak Island, Alaska: Potential risk to predators and fishermen	2007
3474	S. Dixit	Effect of α-lipoic acid on spatial memory and structural integrity of developing hippocampal neurons in rats subjected to sodium arsenite exposure	2020
3475	B. Gamboa-Loira	Physical activity, body mass index and arsenic metabolism among Mexican women	2021
3476	S. F. Farzan	Urinary arsenic and relative telomere length in 5–7 year old children in Bangladesh	2021
3477	Y. Arfala	Assessment of heavy metals released into the air from the cement kilns co-burning waste: Case of Oujda cement manufacturing (Northeast Morocco)	2018
3478	N. Arnich	Dietary exposure to trace elements and health risk assessment in the 2nd French Total Diet Study	2012
3479	J. De Loma	Human adaptation to arsenic in Bolivians living in the Andes	2022
3480	C. M. Bulka	Changes in blood pressure associated with lead, manganese, and selenium in a Bangladeshi cohort	2019
3481	J. Souza-Araujo	The consumption of shark meat in the Amazon region and its implications for human health and the marine ecosystem	2021
3482	H. R. Pohl	Six interaction profiles for simple mixtures	2003
3483	S. Parmar	Phase II trial of arsenic trioxide in relapsed and refractory acute myeloid leukemia, secondary leukemia and/or newly diagnosed patients at least 65 years old	2004
3484	K. Saldaña-Villanueva	A preliminary study on health impacts of Mexican mercury mining workers in a context of precarious employment	2022
3485	B. Mukherjee	Platelet hyperactivity, neurobehavioral symptoms and depression among Indian women chronically exposed to low level of arsenic	2014
3486	J. Costa Moreira	Threats by heavy metals: human and environmental contamination in Brazil	1996
3487	M. Rahman	Arsenic exposure and young adult's mortality risk: A 13-year follow-up study in Matlab, Bangladesh	2019
3488	T. Fillman	Association of cadmium and arsenic exposure with salivary telomere length in adolescents in Terai, Nepal	2016
3489	A. M. Weber	Assessment of potentially toxic trace element contamination in urban allotment soils and their uptake by onions: A preliminary case study from Sheffield, England	2019
3490	Y.-X. Wang	Associations of urinary metal levels with serum hormones, spermatozoa apoptosis and sperm DNA damage in a Chinese population	2016

3491	J. M. Torres-Arellano	Natriuretic peptides and echocardiographic parameters in Mexican children environmentally exposed to arsenic	2020
3492	Y. Takayama	Associations between blood arsenic and urinary arsenic species concentrations as an exposure characterization tool	2021
3493	S.-S. Liu	Zoledronic acid exerts antitumor effects in NB4 acute promyelocytic leukemia cells by inducing apoptosis and S phase arrest	2014
3494	X. Wang	Associations of cumulative exposure to heavy metal mixtures with obesity and its comorbidities among U.S. adults in NHANES 2003–2014	2018
3495	I. Giannenas	Effect of a Polyherbal or an Arsenic-Containing Feed Additive on Growth Performance of Broiler Chickens, Intestinal Microbiota, Intestinal Morphology, and Lipid Oxidation of Breast and Thigh Meat	2019
3496	R. A. F. Jack	Anorexia, allergy or arsenic?	1986
3497	C. Fabrice Elegbede	TDS exposure project: How and when to consider seasonalityin a total diet study?	2017
3498	R. Hernández-Martínez	Survey of total mercury and arsenic content in infant cereals marketed in Spain and estimated dietary intake	2013
3499	L.-S. An	Progesterone production requires activation of caspase-3 in preovulatory granulosa cells in a serum starvation model	2012
3500	M. Brummer-Holder	Interrelationships Between Age and Trace Element Concentration in Horse Mane Hair and Whole Blood	2020
3501	Y. Jin	Distribution of speciated arsenicals in mice exposed to arsenite at the early life	2010
3502	M. J. Spratlen	Targeted metabolomics to understand the association between arsenic metabolism and diabetes-related outcomes: Preliminary evidence from the Strong Heart Family Study	2019
3503	L.-H. C. Hsieh	Removal of arsenic from groundwater by electro-ultrafiltration	2008
3504	Y. Chen	Arsenic exposure at low-to-moderate levels and skin lesions, arsenic metabolism, neurological functions, and biomarkers for respiratory and cardiovascular diseases: Review of recent findings from the Health Effects of Arsenic Longitudinal Study (HEALS) in	2009
3505	A. Navas-Acien	Seafood intake and urine concentrations of total arsenic, dimethylarsinate and arsenobetaine in the US population	2011
3506	G. Desai	A cross-sectional study of general cognitive abilities among Uruguayan school children with low-level arsenic exposure, potential effect modification by methylation capacity and dietary folate	2018
3507	Y.-H. Shih	Urinary arsenic concentration, airway inflammation, and lung function in the U.S. adult population	2019
3508	A. B. M. Moore	Concentrations of trace elements in a rare and threatened coastal shark from the Arabian Gulf (smoothtooth blacktip Carcharhinus leiodon)	2015

3509	M. H. Qazilbash	Arsenic Trioxide with Ascorbic Acid and High-Dose Melphalan: Results of a Phase II Randomized Trial	2008
3510	J. E. Smits	Food as medicine: Selenium enriched lentils offer relief against chronic arsenic poisoning in Bangladesh	2019
3511	M. Costa	Review of arsenic toxicity, speciation and polyadenylation of canonical histones	2019
3512	X. Wang	Urinary metals and metal mixtures in midlife women: The Study of Women's Health Across the Nation (SWAN)	2019
3513	J. M. Gilbert	Metal and metalloid concentrations in the tissues of dusky Carcharhinus obscurus, sandbar C. plumbeus and white Carcharodon carcharias sharks from south-eastern Australian waters, and the implications for human consumption	2015
3514	S. F. Farzan	Gene–arsenic interaction in longitudinal changes of blood pressure: Findings from the Health Effects of Arsenic Longitudinal Study (HEALS) in Bangladesh	2015
3515	H. Yamauchi	Arsenic metabolism differs between child and adult patients during acute arsenic poisoning	2021
3516	X. Wang	Low-level environmental arsenic exposure correlates with unexplained male infertility risk	2016
3517	Z. Wang	Mechanisms of the synergistic lung tumorigenic effect of arsenic and benzo(a)pyrene combined- exposure	2021
3518	S. E. Rothenberg	Co-exposure to methylmercury and inorganic arsenic in baby rice cereals and rice-containing teething biscuits	2017
3519	A. E. Siddique	Association between chronic arsenic exposure and the characteristic features of asthma	2020
3520	F. González-Martínez	Arsenic exposure, profiles of urinary arsenic species, and polymorphism effects of glutathione-s-transferase and metallothioneins	2018
3521	J.-l. He	Associations of exposure to multiple trace elements with the risk of goiter: A case-control study	2021
3522	S. V. Flanagan	Arsenic in private well water part 3 of 3: Socioeconomic vulnerability to exposure in Maine and New Jersey	2016
3523	L. J. Bain	Arsenic inhibits stem cell differentiation by altering the interplay between the Wnt3a and Notch signaling pathways	2016
3524	C. S. Ha	p53-Based strategy to reduce hematological toxicity of chemotherapy: A proof of principle study	2016
3525	L. C. Platanias	Biological Responses to Arsenic Compounds*	2009
3526	B. K. Thakur	Valuing health damages due to groundwater arsenic contamination in Bihar, India	2019

3527	V. Mondal	Arsenic exposure-related hyperglycemia is linked to insulin resistance with concomitant reduction of skeletal muscle mass	2020
3528	G. Song	Effects of choline on sodium arsenite-induced neural tube defects in chick embryos	2012
3529	N. C. Twaddle	Metabolism and disposition of arsenic species from oral dosing with sodium arsenite in neonatal CD-1 mice. IV. Toxicokinetics following gavage administration and lactational transfer	2019
3530	M. L. Erickson	Drinking water quality in the glacial aquifer system, northern USA	2019
3531	L. Kozłowska	A urinary metabolomics study of a Polish subpopulation environmentally exposed to arsenic	2019
3532	A. K. M. A. Ullah	Dietary intake of heavy metals from eight highly consumed species of cultured fish and possible human health risk implications in Bangladesh	2017
3533	S. Suntararuks	Sodium arsenite exposure impairs B cell proliferation and enhances vascular inflammation in Plasmodium berghei mouse model	2019
3534	N. Thakur	Arsenic sequestration by iron oxide coated geopolymer microspheres	2021
3535	R. R. Reddy	Evaluation of arsenic field test kits for drinking water: Recommendations for improvement and implications for arsenic affected regions such as Bangladesh	2020
3536	W. A. Wattigney	Biomonitoring of toxic metals, organochlorine pesticides, and polybrominated biphenyl 153 in Michigan urban anglers	2022
3537	R. B. Jain	Association of arsenic exposure with smoking, alcohol, and caffeine consumption: Data from NHANES 2005–2010	2015
3538	M. Allred	Bilateral environmental and occupational health program with India	2003
3539	B. Škrbić	Concentrations of arsenic, cadmium and lead in selected foodstuffs from Serbian market basket: Estimated intake by the population from the Serbia	2013
3540	S. Chattopadhyay	Role of dietary GSH in the amelioration of sodium arsenite-induced ovarian and uterine disorders	2010
3541	H.-H. Zhu	Oral arsenic plus retinoic acid versus intravenous arsenic plus retinoic acid for non-high-risk acute promyelocytic leukaemia: a non-inferiority, randomised phase 3 trial	2018
3542	S. R. Aldhaheri	Dimercapto-1-propanesulfonic acid (DMPS) induces metaphase II mouse oocyte deterioration	2017
3543	W. Wang	Human arsenic exposure and lung function impairment in coal-burning areas in Guizhou, China	2020
3544	M. Dash	Mitigation of arsenic driven utero-ovarian malfunction and changes of apoptotic gene expression by dietary NAC	2020

3545	M. Calatayud	Trivalent arsenic species induce changes in expression and levels of proinflammatory cytokines in intestinal epithelial cells	2014
3546	A. Domingo-Relloso	Arsenic exposure and human blood DNA methylation and hydroxymethylation profiles in two diverse populations from Bangladesh and Spain	2022
3547	Y. Zhang	A portable and field optical emission spectrometry coupled with microplasma trap for high sensitivity analysis of arsenic and antimony simultaneously	2020
3548	L. López-Carrillo	Dietary micronutrient intake and its relationship with arsenic metabolism in Mexican women	2016
3549	E. Avigliano	Heavy metals and trace elements in muscle of silverside (Odontesthes bonariensis) and water from different environments (Argentina): aquatic pollution and consumption effect approach	2015
3550	Y. Karim	Dose-dependent relationships between chronic arsenic exposure and cognitive impairment and serum brain-derived neurotrophic factor	2019
3551	A. Kumar	Arsenic enrichment in groundwater and associated health risk in Bari doab region of Indus basin, Punjab, India	2020
3552	M. Taseidifar	Removal of heavy metal ions from water using ion flotation	2017
3553	Q. Zeng	Silencing GSK3β instead of DKK1 can inhibit osteogenic differentiation caused by co-exposure to fluoride and arsenic	2019
3554	M. Dey	Assessment of contamination level, pollution risk and source apportionment of heavy metals in the Halda River water, Bangladesh	2021
3555	Y. L. Fang	Health risk assessment of trace elements in Chinese raisins produced in Xinjiang province	2010
3556	M. P. Taylor	Atmospherically deposited trace metals from bulk mineral concentrate port operations	2015
3557	H. Shi	Biomonitoring human urinary levels of 26 metal elements in multi-race coexistence region of Xinjiang, China	2020
3558	P. Prasad	Effect of low- and high-level groundwater arsenic on peripheral blood and lung function of exposed rural women	2020
3559	I. Aguilera	Biomonitoring of urinary metals in a population living in the vicinity of industrial sources: A comparison with the general population of Andalusia, Spain	2008
3560	G. Wang	Roles of biomarkers in evaluating interactions among mixtures of lead, cadmium and arsenic	2008
3561	V. Sirot	French infant total diet study: Exposure to selected trace elements and associated health risks	2018
3562	L. A. Hoo Fung	Evaluation of dietary exposure to minerals, trace elements and heavy metals from the muscle tissue of the lionfish Pterois volitans (Linnaeus 1758)	2013

3563	L. Xu	Association of low-level inorganic arsenic exposure from rice with age-standardized mortality risk of cardiovascular disease (CVD) in England and Wales	2020
3564	S. J. S. Flora	Monoisoamyl dimercaptosuccinic acid abrogates arsenic-induced developmental toxicity in human embryonic stem cell-derived embryoid bodies: Comparison with in vivo studies	2009
3565	Y. Pang	Environmental complex exposure and the risk of influenza-like illness among housewives: A case study in Shanxi Province, China	2020
3566	A. P. Sanders	Arsenic in North Carolina: Public Health Implications	2012
3567	B. C. Kelly	Human exposure to trace elements in central Cambodia: Influence of seasonal hydrology and food-chain bioaccumulation behaviour	2018
3568	O. D. Uluozlu	Assessment of trace element contents of chicken products from turkey	2009
3569	S. N. do Nasciment o	Cognitive deficits and ALA-D-inhibition in children exposed to multiple metals	2015
3570	Y. Zhou	Evaluation of urinary metal concentrations and sperm DNA damage in infertile men from an infertility clinic	2016
3571	C.-p. Liu	Arsenic contamination and potential health risk implications at an abandoned tungsten mine, southern China	2010
3572	S. Masjosthu smann	Arsenite interrupts neurodevelopmental processes of human and rat neural progenitor cells: The role of reactive oxygen species and species-specific antioxidative defense	2019
3573	S. Mehrzadi	Ellagic acid: A promising protective remedy against testicular toxicity induced by arsenic	2018
3574	M. Yokohira	Severe systemic toxicity and urinary bladder cytotoxicity and regenerative hyperplasia induced by arsenite in arsenic (+3 oxidation state) methyltransferase knockout mice. A preliminary report	2010
3575	Y. Liu	Heavy metals (As, Hg and V) and stable isotope ratios (δ13C and δ15N) in fish from Yellow River Estuary, China	2018
3576	M. Valbonesi	Clinical application of therapeutic erythrocytapheresis (TEA)	2000
3577	R. Pérez	Influence of diet in urinary levels of metals in a biomonitoring study of a child population of the Valencian region (Spain)	2018
3578	Q.-b. Zeng	Arsenic may be involved in fluoride-induced bone toxicity through PTH/PKA/AP1 signaling pathway	2014
3579	P. Liu	The association between metal exposure and semen quality in Chinese males: The mediating effect of androgens	2020
3580	M. S. M. Yusof	Arsenic adsorption mechanism on palm oil fuel ash (POFA) powder suspension	2020

3581	M. Argos	Arsenic exposure from drinking water, and all-cause and chronic-disease mortalities in Bangladesh (HEALS): a prospective cohort study	2010
3582	T. B. Haddy	Medical progress: Factors of importance in breast milk	1952
3583	J. P. Goullé	Analyse des cheveux: intérêt en pratique médicale hospitalière	1996
3584	Y. Zheng	Effect of arsenic-containing hydrocarbon on the long-term potentiation at Schaffer Collateral-CA1 synapses from infantile male rat	2021
3585	A. Heshmati	Dietary exposure to toxic and essential trace elements by consumption of wild and farmed carp (Cyprinus carpio) and Caspian kutum (Rutilus frisii kutum) in Iran	2017
3586	P. K. Gusso-Choueri	Metals and arsenic in fish from a Ramsar site under past and present human pressures: Consumption risk factors to the local population	2018
3587	D. N. R. Veeramachaneni	Impact of environmental pollutants on the male: Effects on germ cell differentiation	2008
3588	L. L. Aylward	Evaluation of urinary speciated arsenic in NHANES: Issues in interpretation in the context of potential inorganic arsenic exposure	2014
3589	S. Dixit	Alpha lipoic acid (ALA) modulates expression of apoptosis associated proteins in hippocampus of rats exposed during postnatal period to sodium arsenite (NaAsO2)	2015
3590	É. Lampron-Goulet	Association between consumption of private well water contaminated by low levels of arsenic and dysglycemia in a rural region of Quebec, Canada	2017
3591	S. V. Flanagan	Arsenic in private well water part 2 of 3: Who benefits the most from traditional testing promotion?	2016
3592	R. Yang	Thiol-functionalized chitin nanofibers for As (III) adsorption	2015
3593	P. Dhar	Antioxidant supplementation upregulates calbindin expression in cerebellar Purkinje cells of rat pups subjected to post natal exposure to sodium arsenite	2018
3594	J. Buschmann	Contamination of drinking water resources in the Mekong delta floodplains: Arsenic and other trace metals pose serious health risks to population	2008
3595	S. H. K. Takeda	Trace element levels in blood and associated factors in adults living in the metropolitan area of São Paulo, Brazil	2017
3596	S. Biswas	In vivo evaluation of arsenic-associated behavioral and biochemical alterations in F0 and F1 mice	2020
3597	T. O. Randhir	Decision-making under surprise and uncertainty: Arsenic contamination of water supplies	2018
3598	R. A. Alyea	Is the current product safety assessment paradigm protective for epigenetic mechanisms?	2012

3599	A. Cardenas	Cross sectional association of arsenic and seroprevalence of hepatitis B infection in the United States (NHANES 2003–2014)	2018
3600	J. Burger	Species differences in contaminants in fish on and adjacent to the Oak Ridge Reservation, Tennessee	2004
3601	F. Castiello	Association of urinary metal concentrations with blood pressure and serum hormones in Spanish male adolescents	2020
3602	C.-Y. Huang	Comparison of arsenic methylation capacity and polymorphisms of arsenic methylation genes between bladder cancer and upper tract urothelial carcinoma	2018
3603	D. F. Bajorin	Arsenic Trioxide in Recurrent Urothelial Cancer: A Cancer and Leukemia Group B Phase II Trial (CALGB 99903)	2009
3604	V. Sharma	Prenatal exposure to arsenic promotes sterile inflammation through the Polycomb repressive element EZH2 and accelerates skin tumorigenesis in mouse	2022
3605	C. W. McCollum	Embryonic exposure to sodium arsenite perturbs vascular development in zebrafish	2014
3606	C. R. Tyler	Developmental exposure to 50 parts-per-billion arsenic influences histone modifications and associated epigenetic machinery in a region- and sex-specific manner in the adult mouse brain	2015
3607	O. Spiegelstein	Developmental consequences of in utero sodium arsenate exposure in mice with folate transport deficiencies	2005
3608	B. Pavilonis	Characterization and risk of exposure to elements from artisanal gold mining operations in the Bolivian Andes	2017
3609	S. L. Yu	Increased PIT1 and PIT2 Expression in Streptozotocin (STZ)-induced Diabetic Mice Contributes to Uptake of iAs(V)	2017
3610	M. M. Rahman	Consumption of arsenic and other elements from vegetables and drinking water from an arsenic-contaminated area of Bangladesh	2013
3611	C.-W. Liu	Geochemical, mineralogical and statistical characteristics of arsenic in groundwater of the Lanyang Plain, Taiwan	2019
3612	D. P. Hanlon	The concentration and chemical status of arsenic in the early placentas of arsenate-dosed hamsters	1987
3613	J.-W. Chen	Arsenic methylation, GSTO1 polymorphisms, and metabolic syndrome in an arseniasis endemic area of southwestern Taiwan	2012
3614	J.-J. Lee	Evaluation of potential health risk of arsenic-affected groundwater using indicator kriging and dose response model	2007
3615	Y. Abaza	Long-term outcome of acute promyelocytic leukemia treated with all-trans-retinoic acid, arsenic trioxide, and gemtuzumab	2017
3616	P. Roy	Evaluation of genetic damage in tobacco and arsenic exposed population of Southern Assam, India using buccal cytome assay and comet assay	2016

3617	H. T. Davis	Identifying natural and anthropogenic sources of metals in urban and rural soils using GIS-based data, PCA, and spatial interpolation	2009
3618	V. Yusà	Exposure and risk assessment to arsenic species in Spanish children using biomonitoring	2018
3619	S. Orecchio	Determination of trace elements in gluten-free food for celiac people by ICP-MS	2014
3620	B. J. Wlodarczyk	Arsenic-induced gene expression changes in the neural tube of folate transport defective mouse embryos	2006
3621	R. X. Armijos	Elevated blood lead and metal/metalloid levels and environmental exposure sources in urban Ecuadorian school-age children and mothers	2021
3622	B. Sharma	Arsenic toxicity induced endothelial dysfunction and dementia: Pharmacological interdiction by histone deacetylase and inducible nitric oxide synthase inhibitors	2013
3623	S. Naqvi	Comparative efficacy of Nano and Bulk Monoisoamyl DMSA against arsenic-induced neurotoxicity in rats	2020
3624	Á. A. Carbonell-Barrachina	Inorganic arsenic contents in rice-based infant foods from Spain, UK, China and USA	2012
3625	K. T. Kitchin	The role of protein binding of trivalent arsenicals in arsenic carcinogenesis and toxicity	2008
3626	J.-J. Lee	Assessing carcinogenic risks associated with ingesting arsenic in farmed smeltfish (Ayu, Plecoglossus altirelis) in aseniasis-endemic area of Taiwan	2008
3627	A. Das	Pollution index and health risk assessment of arsenic through different groundwater sources and its load on soil-paddy-rice system in a part of Murshidabad district of West Bengal, India	2021
3628	S. F. Ahmed	Heavy metal toxicity, sources, and remediation techniques for contaminated water and soil	2022
3629	P. Koedrith	Toxicogenomic approaches for understanding molecular mechanisms of heavy metal mutagenicity and carcinogenicity	2013
3630	K. Lo	Associations between blood and urinary manganese with metabolic syndrome and its components: Cross-sectional analysis of National Health and Nutrition Examination Survey 2011–2016	2021
3631	D. Chakraborti	Groundwater arsenic contamination in Bangladesh—21 Years of research	2015
3632	A. Jurkiewicz	Metal content in femoral head spongious bone of people living in regions of different degrees of environmental pollution in Southern and Middle Poland	2004
3633	S. K. Paul	Higher risk of hyperglycemia with greater susceptibility in females in chronic arsenic-exposed individuals in Bangladesh	2019
3634	O. Saphir	Isolated myocarditis	1942

3635	J. Nriagu	High levels of uranium in groundwater of Ulaanbaatar, Mongolia	2012
3636	H. Zhang	Estimating and comparing the cancer risks from THMs and low-level arsenic in drinking water based on disability-adjusted life years	2018
3637	N. Yoshida	Non-monotonic relationships between arsenic and selenium excretion and its implication on arsenic methylation pattern in a Bangladeshi population	2015
3638	J. Shen	Induction of glutathione S-transferase placental form positive foci in liver and epithelial hyperplasia in urinary bladder, but no tumor development in male Fischer 344 rats treated with monomethylarsonic acid for 104 weeks	2003
3639	M. Dash	The consequence of NAC on sodium arsenite-induced uterine oxidative stress	2018
3640	A. Abuawad	Association between body mass index and arsenic methylation in three studies of Bangladeshi adults and adolescents	2021
3641	T. Bacquart	Multiple inorganic toxic substances contaminating the groundwater of Myingyan Township, Myanmar: Arsenic, manganese, fluoride, iron, and uranium	2015
3642	O. Spiegelstein	Effects of dietary folate intake and folate binding protein-1 (Folbp1) on urinary speciation of sodium arsenate in mice	2003
3643	J. Li	MicroRNA-15b in extracellular vesicles from arsenite-treated macrophages promotes the progression of hepatocellular carcinomas by blocking the LATS1-mediated Hippo pathway	2021
3644	A. Simić	Trace element status in patients with type 2 diabetes in Norway: The HUNT3 Survey	2017
3645	V. F. de Oliveira	Assessing mineral and toxic elements content in rice grains grown in southern Brazil	2021
3646	T. Joseph	Human health risk assessment from arsenic exposures in Bangladesh	2015
3647	F. Conte	First data on trace elements in Haliotis tuberculata (Linnaeus, 1758) from southern Italy: Safety issues	2015
3648	L. P. Chandravanshi	Reversibility of changes in brain cholinergic receptors and acetylcholinesterase activity in rats following early life arsenic exposure	2014
3649	M. Saeed	Arsenic uptake and toxicity in wheat (Triticum aestivum L.): A review of multi-omics approaches to identify tolerance mechanisms	2021
3650	N. Saint-Jacques	Estimating the risk of bladder and kidney cancer from exposure to low-levels of arsenic in drinking water, Nova Scotia, Canada	2018
3651	M. U. Rehman	Fate of arsenic in living systems: Implications for sustainable and safe food chains	2021
3652	A. K. Upadhyay	Augmentation of arsenic enhances lipid yield and defense responses in alga Nannochloropsis sp	2016

3653	E. J. Martinez-Finley	Learning deficits in C57BL/6J mice following perinatal arsenic exposure: Consequence of lower corticosterone receptor levels?	2009
3654	A.-L. Lindberg	Gender and age differences in the metabolism of inorganic arsenic in a highly exposed population in Bangladesh	2008
3655	K. S. Engström	Low 8-oxo-7,8-dihydro-2′-deoxyguanosine levels and influence of genetic background in an Andean population exposed to high levels of arsenic	2010
3656	R. S. Yadav	Neuroprotective efficacy of curcumin in arsenic induced cholinergic dysfunctions in rats	2011
3657	N. C. Twaddle	Metabolism and disposition of arsenic species from controlled dosing with sodium arsenite in adult female CD-1 mice. III. Toxicokinetic studies following oral and intravenous administration	2018
3658	S. Srivastava	In utero arsenic exposure induces early onset of atherosclerosis in ApoE−/− mice	2007
3659	J. R. Berenson	A Prospective, Open-Label Safety and Efficacy Study of Combination Treatment with Melphalan, Arsenic Trioxide, and Ascorbic Acid in Patients with Relapsed or Refractory Multiple Myeloma	2004
3660	M. K. Hailer	Assessing human metal accumulations in an urban superfund site	2017
3661	F. Maekawa	Effects of sodium arsenite on neurite outgrowth and glutamate AMPA receptor expression in mouse cortical neurons	2013
3662	K. Kordas	Prevalence and predictors of exposure to multiple metals in preschool children from Montevideo, Uruguay	2010
3663	P. Kaushal	Curcumin induced up-regulation of Myelin basic protein (MBP) ameliorates sodium arsenite induced neurotoxicity in developing rat cerebellum	2014
3664	E. Estey	Use of all-trans retinoic acid plus arsenic trioxide as an alternative to chemotherapy in untreated acute promyelocytic leukemia	2006
3665	P. M. Bradley	Mixed organic and inorganic tapwater exposures and potential effects in greater Chicago area, USA	2020
3666	S. Valles	Exposure to low doses of inorganic arsenic induces transgenerational changes on behavioral and epigenetic markers in zebrafish (Danio rerio)	2020
3667	M. Ferreira	Biological variables and health status affecting inorganic element concentrations in harbour porpoises (Phocoena phocoena) from Portugal (western Iberian Peninsula)	2016
3668	E. M. Kenyon	A concise review of the toxicity and carcinogenicity of dimethylarsinic acid	2001
3669	S. Butaciu	Chemical modeling of groundwater in the Banat Plain, southwestern Romania, with elevated As content and co-occurring species by combining diagrams and unsupervised multivariate statistical approaches	2017
3670	J. P. Nater	Drugs used on the skin	1982

3671	J.-F. Coppin	Interplay between Cellular Methyl Metabolism and Adaptive Efflux during Oncogenic Transformation from Chronic Arsenic Exposure in Human Cells*	2008
3672	Z. Usydus	Fish products available in Polish market – Assessment of the nutritive value and human exposure to dioxins and other contaminants	2009
3673	A. S. Dickerson	Autism spectrum disorder prevalence and proximity to industrial facilities releasing arsenic, lead or mercury	2015
3674	V. Bencko	Health aspects of burning coal with a high arsenic content: II. Hearing changes in exposed children	1977
3675	A. Raab	Arsenic accumulation and speciation analysis in wool from sheep exposed to arsenosugars	2002
3676	A. Szymańska-	The relationship between selected VDR, HFE and ALAD gene polymorphisms and several basic toxicological parameters among persons occupationally exposed to lead	2015
3677	B. E. Birgisdottir	Essential and toxic element concentrations in blood and urine and their associations with diet: Results from a Norwegian population study including high-consumers of seafood and game	2013
3678	X. Yan	Co-exposure to inorganic arsenic and fluoride prominently disrupts gut microbiota equilibrium and induces adverse cardiovascular effects in offspring rats	2021
3679	R. Ríos	Methyl group balance in brain and liver: Role of choline on increased S-adenosyl methionine (SAM) demand by chronic arsenic exposure	2012
3680	J. A. Kaufman	Arsenic, blood pressure, and hypertension in the Strong Heart Family Study	2021
3681	X. Guo	Multi-generational impacts of arsenic exposure on genome-wide DNA methylation and the implications for arsenic-induced skin lesions	2018
3682	C. Vieira	Mercury, cadmium, lead and arsenic levels in three pelagic fish species from the Atlantic Ocean: Intra- and inter-specific variability and human health risks for consumption	2011
3683	B. G. Wei	Blood Pressure Associated with Arsenic Methylation and Arsenic Metabolism Caused by Chronic Exposure to Arsenic in Tube Well Water	2017
3684	P. Ravenscroft	Stable groundwater quality in deep aquifers of Southern Bangladesh: The case against sustainable abstraction	2013
3685	K. T. Kitchin	Arsenite binding to synthetic peptides based on the Zn finger region and the estrogen binding region of the human estrogen receptor-α	2005
3686	E. Allen	Trichomonas vaginalis vaginitis	1936
3687	G. Zhong	Arsenic or/and antimony induced mitophagy and apoptosis associated with metabolic abnormalities and oxidative stress in the liver of mice	2021
3688	L. Erdinger	The Aral Sea disaster – human biomonitoring of Hg, As, HCB, DDE, and PCBs in children living in Aralsk and Akchi, Kazakhstan	2004

3689	S. Kim	Harmonization of transcriptomic and methylomic analysis in environmental epidemiology studies for potential application in chemical risk assessment	2022
3690	L. A. Gibson	Life cycle exposure of the frog Silurana tropicalis to arsenate: Steroid- and thyroid hormone-related genes are differently altered throughout development	2016
3691	Z. He	SOX2 modulated astrocytic process plasticity is involved in arsenic-induced metabolic disorders	2022
3692	M. P. Reilly	Prepubertal exposure to arsenic(III) suppresses circulating insulin-like growth factor-1 (IGF-1) delaying sexual maturation in female rats	2014
3693	U. Bardullas	Atrazine is primarily responsible for the toxicity of long-term exposure to a combination of atrazine and inorganic arsenic in the nigrostriatal system of the albino rat	2013
3694	L. A. Henríquez-Hernández	Biomonitoring of 45 inorganic elements measured in plasma from Spanish subjects: A cross-sectional study in Andalusian population	2020
3695	J. H. M. Van Tongeren	FOLIC-ACID DEFICIENCY IN CHRONIC ARSENIC POISONING	1965
3696	S. Barton	TWO CASES OF ARSENICAL PERIPHERAL NEURITIS	1890
3697	V. G. Standen	Prehistoric polydactylism: Biological evidence and rock art representation from the Atacama Desert in northern Chile	2018
3698	R. O. Wright	Neuropsychological correlates of hair arsenic, manganese, and cadmium levels in school-age children residing near a hazardous waste site	2006
3699	H. V. Warren	Geology, trace elements and health	1989
3700	S. L. Goggin	Perinatal exposure to 50ppb sodium arsenate induces hypothalamic-pituitary-adrenal axis dysregulation in male C57BL/6 mice	2012
3701	M. E. Alao	Urinary arsenic is associated with wasting and underweight status in young children in rural Bangladesh	2021
3702	L. Maurice	Drinking water quality in areas impacted by oil activities in Ecuador: Associated health risks and social perception of human exposure	2019
3703	J.-W. Chen	The association between total urinary arsenic concentration and renal dysfunction in a community-based population from central Taiwan	2011
3704	K. L. Brown	Metals in the stomach contents and brain, gonad, kidney, and liver tissues of subsistence-harvested northern sea otters (Enhydra lutris kenyoni) from Icy Strait, Alaska	2021
3705	Y.-T. Liao	Elevated lactate dehydrogenase activity and increased cardiovascular mortality in the arsenic-endemic areas of southwestern Taiwan	2012
3706	P. C. Blair	Comparative toxicity of arsine gas in B6C3F1 mice, Fischer 344 rats, and Syrian Golden hamsters: System organ studies and comparison of clinical indices of exposure	1990

3707	X. Duan	Acute arsenic exposure induces inflammatory responses and CD4+ T cell subpopulations differentiation in spleen and thymus with the involvement of MAPK, NF-kB, and Nrf2	2017
3708	M. J. Spratlen	Arsenic, one carbon metabolism and diabetes-related outcomes in the Strong Heart Family Study	2018
3709	Z. Drobna	Activation of Lrrk2 and α-Synuclein in substantia nigra, striatum, and cerebellum after chronic exposure to arsenite	2020
3710	C. A. Sánchez	Land use, season, and parasitism predict metal concentrations in Australian flying fox fur	2022
3711	C. Formigaro	Trace element concentrations in the Mediterranean monk seal (Monachus monachus) in the eastern Mediterranean Sea	2017
3712	R. Peruru	Devil's claw (Harpagophytum procumbens) ameliorates the neurobehavioral changes and neurotoxicity in female rats exposed to arsenic	2020
3713	H. J. Iland	All-trans-retinoic acid, idarubicin, and IV arsenic trioxide as initial therapy in acute promyelocytic leukemia (APML4)	2012
3714	X.-Y. Zhang	Reactive oxygen species-evoked genotoxic stress mediates arsenic-induced suppression of male germ cell proliferation and decline in sperm quality	2021
3715	Q. Cui	Deficiency of long isoforms of Nfe2l1 sensitizes MIN6 pancreatic β cells to arsenite-induced cytotoxicity	2017
3716	R. C. Lantz	In utero and postnatal exposure to arsenic alters pulmonary structure and function	2009
3717	E. Nassireslami	How sodium arsenite improve amyloid β-induced memory deficit?	2016
3718	F. Cubadda	Human exposure to dietary inorganic arsenic and other arsenic species: State of knowledge, gaps and uncertainties	2017
3719	B. Yang	Deficiency in the nuclear factor E2-related factor 2 renders pancreatic β-cells vulnerable to arsenic-induced cell damage	2012
3720	C. Watanabe	Water intake in an Asian population living in arsenic-contaminated area	2004
3721	L. Xu	Urinary element profiles and associations with cardiometabolic diseases: A cross-sectional study across ten areas in China	2022
3722	D. B. Szymkowicz	Embryonic-only arsenic exposure in killifish (Fundulus heteroclitus) reduces growth and alters muscle IGF levels one year later	2017
3723	H. J. Clewell	Research toward the development of a biologically based dose response assessment for inorganic arsenic carcinogenicity: A progress report	2007
3724	D. J. MacLachlan	Arsenic, cadmium, cobalt, copper, lead, mercury, molybdenum, selenium and zinc concentrations in liver, kidney and muscle in Australian sheep	2016

3725	M. Molin	Urinary excretion of arsenicals following daily intake of various seafoods during a two weeks intervention	2014
3726	L. A. Baldissarelli	Arsenic alters behavioral parameters and brain ectonucleotidases activities in zebrafish (Danio rerio)	2012
3727	A. Karimi	Exposure of hepatocellular carcinoma cells to low-level As2O3 causes an extra toxicity pathway via L1 retrotransposition induction	2014
3728	A. M. Higham	Determination of trace quantities of selenium and arsenic in canned tuna fish by using electroanalytical techniques	1993
3729	H. Y. Kim	Differential epigenetic effects of chlorpyrifos and arsenic in proliferating and differentiating human neural progenitor cells	2016
3730	S.-G. Sia	Enrichment of arsenic, lead, and antimony in Balingian coal from Sarawak, Malaysia: Modes of occurrence, origin, and partitioning behaviour during coal combustion	2012
3731	M. Collotta	Epigenetics and pesticides	2013
3732	V. Delgado Quezada	Arsenic in geoenvironments of Nicaragua: Exposure, health effects, mitigation and future needs	2020
3733	J.-S. Chang	Effect of arsenic on p53 mutation and occurrence of teratogenic salamanders: Their potential as ecological indicators for arsenic contamination	2009
3734	P. Bhattacharjee	Systems biology approaches to evaluate arsenic toxicity and carcinogenicity: An overview	2013
3735	Z.-Y. Du	Risk–benefit evaluation of fish from Chinese markets: Nutrients and contaminants in 24 fish species from five big cities and related assessment for human health	2012
3736	L. Yin	Arsenic-induced apoptosis in the p53-proficient and p53-deficient cells through differential modulation of NFkB pathway	2018
3737	P. Kapahi	Inhibition of NF-κB Activation by Arsenite through Reaction with a Critical Cysteine in the Activation Loop of IκB Kinase*	2000
3738	M. Berglund	Gender and age differences in mixed metal exposure and urinary excretion	2011
3739	Y.-H. Kao	Effect of sulfidogenesis cycling on the biogeochemical process in arsenic-enriched aquifers in the Lanyang Plain of Taiwan: Evidence from a sulfur isotope study	2015
3740	P. Sykora	Modulation of DNA polymerase beta-dependent base excision repair in cultured human cells after low dose exposure to arsenite	2008
3741	T. Ochi	Cytotoxic, genotoxic and cell-cycle disruptive effects of thio-dimethylarsinate in cultured human cells and the role of glutathione	2008
3742	D. Mondal	A comparison of two techniques for calculating groundwater arsenic-related lung, bladder and liver cancer disease burden using data from Chakdha block, West Bengal	2008

3743	M. A. Rahman	Arsenic, iron and chloride in drinking water at primary school, Satkhira, Bangladesh	2019
3744	H. M. Lidiard	Iodine in the reclaimed upland soils of a farm in the Exmoor National Park, Devon, U.K. and its impact on livestock health	1995
3745	M. Fujioka	Examination of in vivo mutagenicity of sodium arsenite and dimethylarsinic acid in gpt delta rats	2016
3746	J. Li	Tissue-specific distributions of inorganic arsenic and its methylated metabolites, especially in cerebral cortex, cerebellum and hippocampus of mice after a single oral administration of arsenite	2017
3747	S. Frankel	Arsenic exposure disrupts neurite growth and complexity in vitro	2009
3748	P. Andrén	Environmental exposure to lead and arsenic among children living near a glassworks	1988
3749	P. Tirkey	Assessment of groundwater quality and associated health risks: A case study of Ranchi city, Jharkhand, India	2017
3750	S. Takumi	In vivo mutagenicity of arsenite in the livers of gpt delta transgenic mice	2014
3751	J. U. Skaare	Levels of polychlorinated biphenyls, organochlorine pesticides, mercury, cadmium, copper, selenium, arsenic, and zinc in the harbour seal, Phoca vitulina, in Norwegian waters	1990
3752	Z. Li	Association between exposure to arsenic, nickel, cadmium, selenium, and zinc and fasting blood glucose levels	2019
3753	D. B. Szymkowicz	Embryonic-only arsenic exposure alters skeletal muscle satellite cell function in killifish (Fundulus heteroclitus)	2018
3754	A. Thomson	ON THE PREPARATION AND NATURE OF THE IODIDE OF ARSENIC;: WITH OBSERVATIONS AND EXPERIMENTS ON ITS MEDICINAL AND POISONOUS PROPERTIES	1838
3755	X. Zhou	Arsenic co-carcinogenesis: Inhibition of DNA repair and interaction with zinc finger proteins	2021
3756	M. López-Guzmán	Simultaneous removal of fluoride and arsenic from well water by electrocoagulation	2019
3757	F. Couto-Santos	Prepubertal arsenic exposure alters phosphoproteins profile, quality, and fertility of epididymal spermatozoa in sexually mature rats	2021
3758	X. Yu	Gene expression profiling analysis reveals arsenic-induced cell cycle arrest and apoptosis in p53-proficient and p53-deficient cells through differential gene pathways	2008
3759	J. Dron	Contaminant signatures and stable isotope values qualify European conger (Conger conger) as a pertinent bioindicator to identify marine contaminant sources and pathways	2019
3760	H. Dermott	CASE OF POISONING BY ARSENIC, AND RECOVERY	1851

3761	F. Zhao	Effects of arsenite on glutamate metabolism in primary cultured astrocytes	2012
3762	D. Chakraborti	Environmental arsenic contamination and its health effects in a historic gold mining area of the Mangalur greenstone belt of Northeastern Karnataka, India	2013
3763	R. Sleno	Conformational biosensors reveal allosteric interactions between heterodimeric AT1 angiotensin and prostaglandin F2α receptors	2017
3764	J. M. R. Antoine	Dietary intake of minerals and trace elements in rice on the Jamaican market	2012
3765	M. Wéry	Drug used in the treatment of sleeping sickness (human African trypanosomiasis: HAT)	1994
3766	A. K. Mensah	Phytoavailability and uptake of arsenic in ryegrass affected by various amendments in soil of an abandoned gold mining site	2022
3767	M. Boisset	Les « Métaux Lourds » dans l'alimentation : quels risques pour les consommateurs ?	2017
3768	F. Inesta-Vaquera	Application of the in vivo oxidative stress reporter Hmox1 as mechanistic biomarker of arsenic toxicity	2021
3769	B. G. Campbell	Broadsheet number 48: mercury, cadmium and arsenic: toxicology and laboratory investigation	1999
3770	A. P. Sanders	Combined exposure to lead, cadmium, mercury, and arsenic and kidney health in adolescents age 12–19 in NHANES 2009–2014	2019
3771	W. Wei	Arsenic exposure and its joint effects with cigarette smoking and physical exercise on lung function impairment: Evidence from an occupational cohort study	2021
3772	D. Cortés-Arriagada	Aluminum and iron doped graphene for adsorption of methylated arsenic pollutants	2016
3773	P. Mondal	Laboratory based approaches for arsenic remediation from contaminated water: Recent developments	2006
3774	R. S. Yadav	Neuroprotective effect of curcumin in arsenic-induced neurotoxicity in rats	2010
3775	L. Wu	The ubiquitination and acetylation of histones are associated with male reproductive disorders induced by chronic exposure to arsenite	2020
3776	E. De Felip	Priority persistent contaminants in people dwelling in critical areas of Campania Region, Italy (SEBIOREC biomonitoring study)	2014
3777	E. S. Hansen	Shared risk factors for cancer and atherosclerosis—a review of the epidemiological evidence	1990
3778	L. P. Chandravanshi	Early life arsenic exposure and brain dopaminergic alterations in rats	2014

3779	R. A. Zingaro	Arsenic—A classic example of chemophobia	1993
3780	J. P. Brown	Arsenic: risk assessment for california drinking water standards	1994
3781	A. Chatterjee	All-trans retinoic acid protects against arsenic-induced uterine toxicity in female Sprague–Dawley rats	2011
3782	D. Finlay	ON A CASE OF PROGRESSIVE ANÆMIA.1: CURED BY IRON AFTER FAILURE OF ARSENIC TO AFFORD RELIEF	1885
3783	M. Dutta	High fat diet aggravates arsenic induced oxidative stress in rat heart and liver	2014
3784	U. Bardullas	Chronic low-level arsenic exposure causes gender-specific alterations in locomotor activity, dopaminergic systems, and thioredoxin expression in mice	2009
3785	M. Madajewicz	Can information alone change behavior? Response to arsenic contamination of groundwater in Bangladesh	2007
3786	D. L. Ely	Aerometric and hair trace metal content in learning-disabled children	1981
3787	W. Harwood Nutt	ARSENIC CANCER:: A Case under the Care of	1913
3788	L. M. Klevay	Pharmacology and toxicology of heavy metals: Arsenic	1976
3789	R. Garnier	Exposition de la population française à l'arsenic inorganique. Identification de valeurs toxicologiques de référence	2020
3790	A. A. Jennings	Analysis of worldwide regulatory guidance values for the most commonly regulated elemental surface soil contamination	2013
3791	N. K. Rai	Exposure to As, Cd and Pb-mixture impairs myelin and axon development in rat brain, optic nerve and retina	2013
3792	H. M. Kantarjian	CLINICAL COURSE AND THERAPY OF CHRONIC MYELOGENOUS LEUKEMIA WITH INTERFERON-ALPHA AND CHEMOTHERAPY	1998
3793	U. Varanasi	Chemical contaminants in gray whales (Eschrichtius robustus) stranded along the west coast of North America	1994
3794	M. Liu	Contamination features, geo-accumulation, enrichments and human health risks of toxic heavy metal(loids) from fish consumption collected along Swat river, Pakistan	2020
3795	X. He	Induction of Metallothionein I by Arsenic via Metal-activated Transcription Factor 1: CRITICAL ROLE OF C-TERMINAL CYSTEINE RESIDUES IN ARSENIC SENSING	2009
3796	N. D. Das	Sodium arsenite dependent protein expression analysis on human embryonic carcinoma (NCCIT) cell line	2011

3797	G. J. Ahlborn	Dose response evaluation of gene expression profiles in the skin of K6/ODC mice exposed to sodium arsenite	2008
3798	B. Bocca	Toxic metals contained in cosmetics: A status report	2014
3799	R. Cornelis	Sample Collection Guidelines for Trace Elements in Blood and Urine	1996
3800	C. M. Wenyon	The Second Royal Society of Tropical Medicine and Hygiene Chadwick Lecture. Observations on trypanosomissis in the Belgian Congo	1947
3801	J. Sibilia	Quoi de neuf en médecine en 2007 ?	2007
3802	E. H. Hudson	Bejel: The endemic syphilis of the euphrates arab	1937
3803	P. J. Landrigan	Exposure of children to heavy metals from smelters: Epidemiology and toxic consequences	1981
3804	P. Torka	Swallowing a bitter pill-oral arsenic trioxide for acute promyelocytic leukemia	2016
3805	P. A. Gunka	Toward Heterolytic Bond Dissociation of Dihydrogen: The Study of Hydrogen in Arsenolite under High Pressure	2019
3806	J. A. Sans	Ordered helium trapping and bonding in compressed arsenolite: Synthesis of As4O6 center dot 2He	2016
3807	J. Tremlova	Arsenic compounds occurring in ruderal plant communities growing in arsenic contaminated soils	2016
3808	F. Rydbeck	Urinary iodine concentrations of pregnant women in rural Bangladesh: A longitudinal study	2014
3809	E. C. Nyanza	Geophagy practices and the content of chemical elements in the soil eaten by pregnant women in artisanal and small scale gold mining communities in Tanzania	2014
3810	N. Mise	Hijiki seaweed consumption elevates levels of inorganic arsenic intake in Japanese children and pregnant women	2019
3811	L. Li	Arsenic pre-removal from antimony oxide powder by roasting with pyrite (FeS2) for decreasing arsenic transfer and pollution in the followed antimony smelting process	2022
3812	H. L. Lei	Relationship between risk factors for infertility in women and lead, cadmium, and arsenic blood levels: a cross-sectional study from Taiwan	2015
3813	K. Okabe	INVESTIGATION INTO FETAL TOXICITY BY ARSENIC EXPOSURE TO PREGNANT WOMEN	2017
3814	N. Mise	Concentration of folic acid (FA) in serum of Japanese pregnant women	2020

3815	A. J. White	Urine and toenail cadmium levels in pregnant women: A reliability study	2018
3816	I. Mancini	Synthesis and in-vitro anticancer evaluation of polyarsenicals related to the marine sponge derived Arsenicin A	2017
3817	P. A. Gunka	Compressed Arsenolite As4O6 and Its Helium Clathrate As4O6 center dot 2He	2015
3818	M. A. Domanski	Arsenic(III)-oxide Intercalates with Potassium Chloride: Water-Induced Varieties and New Synthesis Methods	2021
3819	P. A. Gunka	Water induced variety of arsenic(III) oxide intercalates with alkali chlorides	2021
3820	A. M. Gil	Assessing Exposome Effects on Pregnancy through Urine Metabolomics of a Portuguese (Estarreja) Cohort	2018
3821	J. H. Hoover	Exposure to uranium and co-occurring metals among pregnant Navajo women	2020
3822	V. A. Fedorov	Processes for the Preparation of High-Purity Arsenic and Its Compounds	2021
3823	J. E. Rager	Review of the environmental prenatal exposome and its relationship to maternal and fetal health	2020
3824	L. C. Yang	Evaluation of Iodine Nutritional Status Among Pregnant Women in China	2020
3825	W. Qiao	Molecular Evidence of Arsenic Mobility Linked to Biodegradable Organic Matter	2020
3826	H. Khosla	First report of successful management of acute promyelocytic leukemia in a pregnant female with All-Trans-Retinoic Acid and Arsenic Trioxide-based induction regimen	2020
3827	Z. L. Liu	Three-layer core-shell magnetic Fe3O4@C@Fe2O3 microparticles as a high-performance sorbent for the capture of gaseous arsenic from SO2-containing flue gas	2019
3828	D. Lu	Sulfur Derivatives of the Natural Polyarsenical Arsenicin A: Biologically Active, Organometallic Arsenic Sulfur Cages Related to the Minerals Realgar and Uzonite	2015
3829	D. Fano	Reproductive outcomes in pregnant women and its association with arsenic contamination in drinking water, in a region characterized by high birth weight rates in Peru	2021
3830	Z. Q. Lin	Individual heavy metal exposure and birth outcomes in Shenqiu county along the Huai River Basin in China	2018
3831	V. A. Fedorov	Ultrapure Arsenic and Its Compounds for Optical and Semiconductor Materials	2016
3832	W. Y. Liu	Cadmium Body Burden and Gestational Diabetes Mellitus: A Prospective Study	2018

3833	X. S. Du	Molecular Transformations of Arsenic Species in the Flue Gas of Typical Power Plants: A Density Functional Theory Study	2016
3834	C. C. Zhou	Effect of ash composition on the partitioning of arsenic during fluidized bed combustion	2017
3835	Y. H. Yang	Insight of arsenic transformation behavior during high-arsenic coal combustion	2019
3836	G. M. Mao	Survey of iodine nutritional status in 2011, Zhejiang, China	2015
3837	Y. H. Xi	Performance and mechanism of arsenic removal in waste acid by combination of CuSO4 and zero-valent iron	2019
3838	W. Qiao	Unraveling roles of dissolved organic matter in high arsenic groundwater based on molecular and optical signatures	2021
3839	H. H. Zhou	Formation mechanism of arsenic-containing dust in the flue gas cleaning process of flash copper pyrometallurgy: A quantitative identification of arsenic speciation	2021
3840	N. Guttenberger	Synthesis of two arsenic-containing cyclic ethers: model compounds for a novel group of naturally-occurring arsenolipids	2016
3841	S. A. Rabb	A novel approach to converting alkylated arsenic to arsenic acid for accurate ICP-OES determination of total arsenic in candidate speciation standards	2018
3842	M. Motas	Heavy Metals and Trace Elements in Human Breast Milk from Industrial/Mining and Agricultural Zones of Southeastern Spain	2021
3843	Y. He	Solidification of sulfur and arsenic in gold concentrate and leaching of the gold by thiosulfate	2020
3844	S. N. Kambunga	Review of the nature of some geophagic materials and their potential health effects on pregnant women: some examples from Africa	2019
3845	S. N. Kambunga	The geochemistry of geophagic material consumed in Onangama Village, Northern Namibia: a potential health hazard for pregnant women in the area	2019
3846	H. Y. Hu	Speciation transformation of arsenic during municipal solid waste incineration	2015
3847	P. Michalak	Crystal Structure and Energetics of Arsenic(III)-Oxide Intercalates with Rubidium Chloride and Their Comparison with Isostructural Intercalates of Potassium Halides	2022
3848	X. X. Dong	Arsenic exposure and intestinal microbiota in children from Sirajdikhan, Bangladesh	2017
3849	A. Kana	Controlled preparation of arsenic nanoparticles	2021
3850	B. Bocca	Human biomonitoring to evaluate exposure to toxic and essential trace elements during pregnancy. Part B: Predictors of exposure	2020

3851	M. S. Kim	Anti-tumor effects of tetraarsenic oxide (TAO, As4O6) in human cervical cancer	2019
3852	A. Chowdhury	Synthesis of arsenical adduct: Synthesis and transformation of dimercapto compound to arsenical adduct	2002
3853	H. I. Mohamed	Influence of Nitric Oxide Application on Some Biochemical Aspects, Endogenous Hormones, Minerals and Phenolic Compounds of Vicia faba Plant Grown under Arsenic Stress	2016
3854	H. Long	Comparison of arsenic(V) removal with different lead-containing substances and process optimization in aqueous chloride solution	2019
3855	S. Tripathi	Therapeutic effects of CoenzymeQ10, Biochanin A and Phloretin against arsenic and chromium induced oxidative stress in mouse (Mus musculus) brain	2022
3856	S. K. Pal	Determination of arsenic in water using fluorescent ZnO quantum dots	2016
3857	T. Onat	The Relationship Between Heavy Metal Exposure, Trace Element Level, and Monocyte to HDL Cholesterol Ratio with Gestational Diabetes Mellitus	2021
3858	L. M. Zhang	Genetic interpretation and health risk assessment of arsenic in Hetao Plain of inner Mongolia, China	2022
3859	Q. H. Guo	Enhanced Removal of Arsenic from Water by Synthetic Nanocrystalline Iowaite	2017
3860	W. Qiao	Identification of processes mobilizing organic molecules and arsenic in geothermal confined groundwater from Pliocene aquifers	2021
3861	H. B. Hu	Advances in Electrochemical Detection Electrodes for As(III)	2022
3862	J. A. Emond	Better Diet Quality during Pregnancy Is Associated with a Reduced Likelihood of an Infant Born Small for Gestational Age: An Analysis of the Prospective New Hampshire Birth Cohort Study	2018
3863	P. H. Ser	Arsenic exposure increases maternal but not cord serum IgG in Bangladesh	2015
3864	N. Yu	Th((As4As4O18)-As-III-O-V): a Mixed-Valent Oxoarsenic(III)/arsenic(V) Actinide Compound Obtained under Extreme Conditions	2014
3865	Q. S. Hu	Adsorption behavior and mechanism of different arsenic species on mesoporous MnFe2O4 magnetic nanoparticles	2017
3866	R. Wang	CHANGES OF ARSENIC SPECIATION DURING SWINE MANURE WINDROW COMPOSTING AT FULL SCALE	2018
3867	Y. W. Kim	Vascular shutdown anti-tumor effect of tetra-arsenic oxide on cervical cancer cells	2016
3868	S. Ishihara	Mechanochemical Treatment to Remove Arsenic from Copper Ore	2019

3869	Y. Yu	Mechanism of CaO and Fe2O3 capture gaseous arsenic species in the flue gas: DFT combined thermodynamic study	2022
3870	I. Neamtiu	Pregnant women in Timis County, Romania are exposed primarily to low-level (< 10 mu g/l) arsenic through residential drinking water consumption	2015
3871	L. Castro	Arsenate and Arsenite Sorption Using Biogenic Iron Compounds: Treatment of Real Polluted Waters in Batch and Continuous Systems	2021
3872	C. V. Amburose	mu-oxobridged heterotrinuclear compounds containing antimony(V) and arsenic(III). Crystal structures of Ar3Sb(mu-OAsPh2)(2) (Ar = Ph or p-tol)	1999
3873	M. S. Rahaman	Environmental arsenic exposure and its contribution to human diseases, toxicity mechanism and management	2021
3874	J. M. Yang	Adsorption Characteristics of Modified Eucalyptus Sawdust for Cadmium and Arsenic and Its Potential for Soil Remediation	2022
3875	P. V. Ioannou	The reaction of dithioerythritol and dithiothreitol with As(III), Sb(III), and Bi(III) compounds	2015
3876	E. C. Fru	The rise of oxygen-driven arsenic cycling at ca. 2.48 Ga	2019
3877	M. Sanchez-Cantu	Evaluation of the mixed oxides produced from hydrotalcite-like compound's thermal treatment in arsenic uptake	2016
3878	B. Seynnaeve	Oxygen-rich poly-bisvanillonitrile embedded amorphous zirconium oxide nanoparticles as reusable and porous adsorbent for removal of arsenic species from water	2021
3879	K. Q. He	Highly efficient sorption and immobilization of gaseous arsenic from flue gas on MnO2/attapulgite composite with low secondary leaching risks	2021
3880	V. Gilhotra	Electrocoagulation technology for high strength arsenic wastewater: Process optimization and mechanistic study	2018
3881	J. E. Bisanz	Randomized Open-Label Pilot Study of the Influence of Probiotics and the Gut Microbiome on Toxic Metal Levels in Tanzanian Pregnant Women and School Children	2014
3882	K. H. Zhang	Arsenic removal from water using a novel amorphous adsorbent developed from coal fly ash	2016
3883	A. Z. Terlikbayeva	Producing Metallic Antimony with Low Arsenic Content from Antimony Concentrate	2018
3884	S. M. T. Hasan	Magnitude and determinants of inadequate third-trimester weight gain in rural Bangladesh	2018
3885	E. Papadopoulou	Diet as a Source of Exposure to Environmental Contaminants for Pregnant Women and Children from Six European Countries	2019
3886	B. M. Liu	Effective and simultaneous removal of organic/inorganic arsenic using polymer-based hydrated iron oxide adsorbent: Capacity evaluation and mechanism	2020

3887	G. P. Gallios	Adsorption of Arsenate by Nano Scaled Activated Carbon Modified by Iron and Manganese Oxides	2017
3888	M. Trinder	Probiotic lactobacilli: a potential prophylactic treatment for reducing pesticide absorption in humans and wildlife	2015
3889	N. K. Kortei	Potential health risk assessment of toxic metals contamination in clay eaten as pica (geophagia) among pregnant women of Ho in the Volta Region of Ghana	2020
3890	A. S. S. Wang	Antagonistic effect of N-ethylmaleimide on arsenic-mediated oxidative stress-induced poly(ADP-ribosyl)ation and cytotoxicity	2017
3891	M. Molin	Arsenic in the human food chain, biotransformation and toxicology - Review focusing on seafood arsenic	2015
3892	Y. H. Zhang	Separation of arsenic and extraction of zinc and copper from high-arsenic copper smelting dusts by alkali leaching followed by sulfuric acid leaching	2021
3893	R. M. Bruce	Uncooked rice consumption: Causes, implications, regulation, and interventions	
3894	W. Zhou	Arsenic nano complex induced degradation of YAP sensitized ESCC cancer cells to radiation and chemotherapy	2020
3895	S. Tanda	Occurrence, Seasonal Variation, and Size Resolved Distribution of Arsenic Species in Atmospheric Particulate Matter in an Urban Area in Southeastern Austria	2020
3896	I. E. Karaagacli oglu	Investigation of the interactions of arsenic with gangue minerals in colemanite calcination	2022
3897	Z. C. Li	Adsorption behavior of arsenicals on MIL-101(Fe): The role of arsenic chemical structures	2019
3898	D. R. Sunuwar	Factors affecting anaemia among women of reproductive age in Nepal: a multilevel and spatial analysis	2021
3899	S. V. Mamyache nkov	Extraction of Nonferrous Metals and Arsenic from Thin Dusts of Copper Fuel Production by Combined Technology	2021
3900	S. E. Baltazar	Adsorption of As(III) and As(V) compounds on Fe3O4(001) surfaces: A first principle study	2017
3901	N. F. Khedr	New insights into arsenic, lead, and iron neurotoxicity: Activation of MAPK signaling pathway and oxidative stress	2022
3902	M. Tanaka	An EXAFS study on the adsorption structure of phenyl-substituted organoarsenic compounds on ferrihydrite	2014
3903	B. Yao	p-Arsanilic acid decontamination over a wide pH range using biochar-supported manganese ferrite material as an effective persulfate catalyst: Performances and mechanisms	2022
3904	B. Bocca	Human biomonitoring to evaluate exposure to toxic and essential trace elements during pregnancy. Part A. concentrations in maternal blood, urine and cord blood	2019

3905	P. K. Singh	Nitric oxide-mediated alleviation of arsenic stress involving metalloid detoxification and physiological responses in rice (Oryza sativa L.)	2022
3906	M. S. Lee	Household use of crop residues and fuelwood for cooking and newborn birth size in rural Bangladesh	2022
3907	M. Vermeulen	Visualization of As(III) and As(V) distributions in degraded paint micro-samples from Baroque- and Rococo-era paintings	2016
3908	P. Adamse	Cadmium, lead, mercury and arsenic in animal feed and feed materials - trend analysis of monitoring results	2017
3909	P. Kumarathi laka	Arsenic speciation dynamics in paddy rice soil-water environment: sources, physico-chemical, and biological factors - A review	2018
3910	M. Kofronova	Strong antioxidant capacity of horseradish hairy root cultures under arsenic stress indicates the possible use of Armoracia rusticana plants for phytoremediation	2019
3911	K. Keune	Tracking the transformation and transport of arsenic sulfide pigments in paints: synchrotron-based X-ray micro-analyses	2015
3912	C. D. Butts	A pilot study of low-moderate drinking water arsenic contamination and chronic diseases among reproductive age women in Timis County, Romania	2015
3913	L. J. Parker	Glutathione transferase P1-1 as an arsenic drug-sequestering enzyme	2017
3914	H. Y. Cheng	Graphene oxide as a stationary phase for speciation of inorganic and organic species of mercury, arsenic and selenium using HPLC with ICP-MS detection	2018
3915	D. W. Zhang	Trimester-specific reference ranges for thyroid hormones in pregnant women	2019
3916	P. Hu	High-Efficiency Simultaneous Oxidation of Organoarsenic and Immobilization of Arsenic in Fenton Enhanced Plasma System	2015
3917	S. Beniwal	Arsenic(III) mixed derivatives having oximes and morpholinedithiocarbamate along with their cytotoxic, antimicrobial, and antioxidant studies	2020
3918	J. C. Lizardo-Huerta	Kinetic modeling of the thermal destruction of lewisite	2020
3919	L. Rodriguez-Flores	Adsorption of arsenic in dacitic tuff pretreated with magnesium oxide	2015
3920	L. Falchi	The evolution of arsenic in the treatment of acute promyelocytic leukemia and other myeloid neoplasms: Moving toward an effective oral, outpatient therapy	2016
3921	C. H. Nguyen	Cytotoxicity Assessment of Gallium- and Indium-Based Nanoparticles Toward Human Bronchial Epithelial Cells Using an Impedance-Based Real-Time Cell Analyzer	2020
3922	T. Yamauchi	Possible production of arsenic hemoglobin adducts via exposure to arsine	2015

3923	G. M. Tsivgoulis	The oxidation of trialkyl trithioarsenites, (RS)(3)As, by octasulfur/triethylamine and dioxygen	2006
3924	D. Ghosh	Sub-surface Biogeochemical Characteristics and Its Effect on Arsenic Cycling in the Holocene Gray Sand Aquifers of the Lower Bengal Basin	2017
3925	G. F. Qu	Preparation of ferric nitrate-graphene nanocomposite and its adsorption of arsenic(V) from simulated arsenic-containing wastewater	2019
3926	Y. Z. Zhao	Speciation transformation of arsenic during municipal sewage sludge incineration with cotton stalk as additive	2017
3927	C. Chen	Efficient degradation of roxarsone and simultaneous in-situ adsorption of secondary inorganic arsenic by a combination of Co3O4-Y2O3 and peroxymonosulfate	2021
3928	J. A. Bhat	Main nitric oxide (NO) hallmarks to relieve arsenic stress in higher plants	2021
3929	L. Hussain	Phenylarsine Oxide Can Induce Degradation of PLZF-RAR alpha Variant Fusion Protein of Acute Promyelocytic Leukemia	2019
3930	T. Kanduc	Multielemental composition and arsenic speciation in low rank coal from the Velenje Basin, Slovenia	2019
3931	M. Botsivali	Transplacental exposure to carcinogens and risks to children: evidence from biomarker studies and the utility of omic profiling	2019
3932	H. M. Liu	Vaporization model for arsenic during single-particle coal combustion: Model development	2019
3933	V. P. Cuenca-Gotor	Vibrational and elastic properties of As4O6 and As4O6 center dot 2He at high pressures: Study of dynamical and mechanical stability	2016
3934	F. L. Barbieri	Toxic trace elements in maternal and cord blood and social determinants in a Bolivian mining city	2016
3935	A. F. Seliverstov	Sorption of arsenic onto inorganic sorbents modified with iron compounds	2017
3936	H. Y. Yu	Arsenic mobility and bioavailability in paddy soil under iron compound amendments at different growth stages of rice	2017
3937	L. G. Kahn	Environmental Toxicant Exposure and Hypertensive Disorders of Pregnancy: Recent Findings	2018
3938	H. L. Rahman	Iron-Incorporated Activated Carbon Synthesis from Biomass Mixture for Enhanced Arsenic Adsorption	2020
3939	A. Kumar	Polyvinylidene fluoride/boehmite nanocomposite membrane for effective removal of arsenate ion from water	2022
3940	S. S. Xu	Maternal Blood Levels of Toxic and Essential Elements and Birth Outcomes in Argentina: The EMASAR Study	2022

3941	O. H. Elshenawy	Down-regulation of cytochrome P450 1A1 by monomethylarsonous acid in human HepG2 cells	2017
3942	D. Ghosh	Elemental and biomarker characteristics in a Pleistocene aquifer vulnerable to arsenic contamination in the Bengal Delta Plain, India	2015
3943	J. Susperreg ui	The in vitro trypanocidal activity of organotin compounds	1997
3944	T. Bartsch	New quaternary arsenide oxides with square planar coordination of gold(I) - structure, Au-197 Mossbauer spectroscopic, XANES and XPS characterization of Nd10Au3As8O10 and Sm(10)Au(3)As(8)O10	2015
3945	J. Tang	Arsenic trioxide induces expression of BCL-2 expression via NF-kappa B and p38 MAPK signaling pathways in BEAS-2B cells during apoptosis	2021
3946	A. F. Seliversto v	Inorganic Sorbents Modified with Iron Compounds for Absorption of As(V) from Water	2019
3947	N. P. Foo	Arsenic compounds activate the MAPK and caspase pathways to induce apoptosis in OEC-M1 gingival epidermal carcinoma	2020
3948	T. K. Menshchik ova	Physicochemical Properties of Arsenic-Containing Lewisite Detoxification Products	2020
3949	Z. P. Wen	Porous biochar-supported MnFe2O4 magnetic nanocomposite as an excellent adsorbent for simultaneous and effective removal of organic/inorganic arsenic from water	2021
3950	W. Zhao	Structure-Reactivity Relationships in the Adsorption and Degradation of Substituted Phenylarsonic Acids on Birnessite (delta-MnO2)	2020
3951	Z. L. Song	Structural Modification of Aminophenylarsenoxides Generates Candidates for Leukemia Treatment via Thioredoxin Reductase Inhibition	2021
3952	S. Dale	Modeling packed bed sorbent systems with the Pore Surface Diffusion Model: Evidence of facilitated surface diffusion of arsenate in nano-metal (hydr)oxide hybrid ion exchange media	2016
3953	K. Leelarungr oj	Leaching mechanisms of heavy metals from fly ash stabilised soils	2018
3954	C. Ye	Multifunctional capacity of CoMnFe-LDH/LDO activated peroxymonosulfate for p-arsanilic acid removal and inorganic arsenic immobilization: Performance and surface-bound radical mechanism	2022
3955	D. Pineda	Surface properties of enargite in MAA depressant solutions	2015
3956	B. Y. Xu	Determination of Total Arsenic, Soluble Arsenic, Total Mercury and Sol-uble Mercury for a Realgar and Cinnabar-containing Traditional Chinese Medicine Compound Niuhuang Xiaoyan Capsule by Semi-bionic Extrac-tion-ICP-MS	2022
3957	V. Diacoman olis	Relationship of arsenic speciation and bioavailability in mine wastes for human health risk assessment	2016
3958	M. Vermeulen	The darkening of copper- or lead-based pigments explained by a structural modification of natural orpiment: a spectroscopic and electrochemical study	2017

3959	J. P. M. Vink	When soils become sediments: Large-scale storage of soils in sandpits and lakes and the impact of reduction kinetics on heavy metals and arsenic release to groundwater	2017
3960	H. Tsuyama	Arsenite suppresses NO production evoked by lipopolysaccharide and poly(I:C) via the suppression of interferon-beta expression in RAW264.7 cells	2019
3961	G. W. Dong	Role of MnO2 in controlling iron and arsenic mobilization from illuminated flooded arsenic-enriched soils	2021
3962	L. L. Wang	Birnessite (delta-MnO2) Mediated Degradation of Organoarsenic Feed Additive p-Arsanilic Acid	2015
3963	E. Giubilato	Comparative occupational risk assessment to support the substitution of Substances of Very High Concern: Alternatives assessment for diarsenic trioxide in Murano artistic glass production	2016
3964	J. W. V. de Mello	Effectiveness of Arsenic Co-Precipitation with Fe-Al Hydroxides for Treatment of Contaminated Water	2018
3965	A. A. Gasanov	World and Russian markets of arsenic	2016
3966	R. C. Vineetha	L-ascorbic acid and alpha-tocopherol attenuate arsenic trioxide-induced toxicity in H9c2 cardiomyocytes by the activation of Nrf2 and Bcl2 transcription factors	2018
3967	G. Caumette	Arsenic cycling in freshwater phytoplankton and zooplankton cultures	2014
3968	E. Zanzo	Aging and arsenite loading control arsenic mobility from ferrihydrite-arsenite coprecipitates	2017
3969	P. V. Ioannou	Thiolates of arsenic(III), antimony(III), and bismuth(III) with DL-alpha-dihydrolipoic acid	2014
3970	M. M. Wolle	Matrix-induced transformation of arsenic species in seafoods	2019
3971	J. W. Choi	Surface Modified Mesostructured Iron Oxyhydroxide: Synthesis, Ecotoxicity, and Application	2014
3972	X. Wang	Silver nanoparticles protect against arsenic induced genotoxicity via attenuating arsenic bioaccumulation and elevating antioxidation in mammalian cells	2021
3973	E. Silina	Synthesis and crystal structure of a solvate of arsenic 4,6-dimethyl-8-quinolinethiolate	2012
3974	L. M. Beaver	Combinatorial effects of zinc deficiency and arsenic exposure on zebrafish (Danio rerio) development	2017
3975	Y. V. Babak	Removal of arsenic compounds from natural waters using a hybrid system (photocatalysis-reverse osmosis)	2014
3976	A. M. Adachi-Mejia	If Providers Had Recommended It, We Would Have Had It Tested: Rural Mothers' Perspectives on Barriers and Facilitators to Testing for Arsenic in Their Well Water	2019

3977	N. Svarovskaya	Synthesis of novel hierarchical micro/nanostructures AlOOH/AlFe and their application for As(V) removal	2022
3978	K. M. Wai	Prenatal Heavy Metal Exposure and Adverse Birth Outcomes in Myanmar: A Birth-Cohort Study	2017
3979	M. Gonzalez-Moscoso	Impact of Silicon Nanoparticles on the Antioxidant Compounds of Tomato Fruits Stressed by Arsenic	2019
3980	E. V. Avtomonov	Syntheses and structures of cyclopentadienyl arsenic compounds .1. Pentamethylcylopentadienyl arsenic dihalides (Cp(*)AsX(2), X=F, Cl, Br, I)	1996
3981	K. Megges	Syntheses and structures of cyclopentadienyl arsenic (III) compounds Part III: Tetraisopropylcyclopentadienyl arsenic (III) dibromide and tetraisopropylcyclopentadienyl arsenic (III) diiodide (TipCpAsBr(2), TipCpAsI(2))	1998
3982	J. G. Dorea	Exposure to environmental neurotoxic substances and neurodevelopment in children from Latin America and the Caribbean	2021
3983	G. Retamal-Morales	Detection of arsenic-binding siderophores in arsenic-tolerating Actinobacteria by a modified CAS assay	2018
3984	M. S. Safarzadeh	Thermodynamic Analysis of the Cu-As-S-(O) System Relevant to Sulfuric Acid Baking of Enargite at 473 K (200 degrees C)	2014
3985	R. B. Viana	Tailoring the electronic properties among oxoarsine, arsinoyl and arsine oxide isomers: the simplest molecular systems with an arsenic-oxygen bond	2016
3986	I. S. Nizamov	Reactions of Lawesson's reagent with arsenic (III) alkoxides	1997
3987	N. Yu	Topologically identical, but geometrically isomeric layers in hydrous alpha-, beta-Rb UO2(AsO3OH)(AsO2(OH)(2)) center dot H2O and anhydrous Rb UO2(AsO3OH)(AsO2(OH)(2))	2014
3988	Y. Azhniuk	Raman evidence for surface oxidation of amorphous As2S3 thin films under ultraviolet irradiation	2019
3989	A. A. Mashentseva	Cu/CuO Composite Track-Etched Membranes for Catalytic Decomposition of Nitrophenols and Removal of As(III)	2020
3990	H. Z. Alkhathlan	APPLICATION OF PHOSPHORUS, ARSENIC AND ANTIMONY REAGENTS IN THE SYNTHESIS OF HETEROCYCLIC-COMPOUNDS	1991
3991	N. D. de Figueiredo	Metal mixtures in pregnant women and umbilical cord blood at urban populations-Rio de Janeiro, Brazil	2020
3992	F. Zulfiqar	Antioxidants as modulators of arsenic-induced oxidative stress tolerance in plants: An overview	2022
3993	O. O. Onireti	Mobilization of soil-borne arsenic by three common organic acids: Dosage and time effects	2016
3994	S. I. Miyashita	Arsenic metabolism in cyanobacteria	2016

3995	X. M. Luo	Remediation of arsenic-contaminated groundwater using media-injected permeable reactive barriers with a modified montmorillonite: sand tank studies	2016
3996	S. Demeshko	An Arsenic-Nitrogen Biradicaloid: Synthesis, Properties, and Reactivity	2013
3997	C. Plackowski	An XPS investigation of surface species formed by electrochemically induced surface oxidation of enargite in the oxidative potential range	2014
3998	G. D'Orazio	Arsenical C-Glucoside Derivatives with Promising Antitumor Activity	2015
3999	M. M. Nearing	Complementary arsenic speciation methods: A review	2014
4000	H. Y. Gong	Insight of particulate arsenic removal from coal-fired power plants	2019
4001	G. Kerr	Metal redistribution during cementation of historic processing residues, Macraes gold mine, New Zealand	2021
4002	M. Contreras-Acuna	Arsenic metabolites in human serum and urine after seafood (Anemonia sulcata) consumption and bioaccessibility assessment using liquid chromatography coupled to inorganic and organic mass spectrometry	2014
4003	X. Y. Li	A pilot study of mothers and infants reveals fetal sex differences in the placental transfer efficiency of heavy metals	2019
4004	M. A. Phillips	Deducing signaling pathways from parallel actions of arsenite and antimonite in human epidermal keratinocytes	2020
4005	M. B. Shakoor	Human health implications, risk assessment and remediation of As-contaminated water: A critical review	2017
4006	D. Kuehnelt	Selenium metabolism to the trimethylselenonium ion (TMSe) varies markedly because of polymorphisms in the indolethylamine N-methyltransferase gene	2015
4007	V. Chandrakar	Nitric Oxide and Dimethylthiourea Up-regulates Pyrroline-5-Carboxylate Synthetase Expression to Improve Arsenic Tolerance in Glycine max L	2019
4008	D. Al Aboud	Protective efficacy of thymoquinone or ebselen separately against arsenic-induced hepatotoxicity in rat	2021
4009	M. Durandurdu	Local structure of As2O3 glass from first principles simulations	2016
4010	A. Sassolini	Evaluation of Molecular Markers and Analytical Methods Documenting the Occurrence of Mustard Gas and Arsenical Warfare Agents in Soil	2016
4011	M. N. Haikou	The autoxidation of triaryl trithioarsenites, (ArS)(3)As: Evidence for binding and activation of triplet dioxygen by arsenic(III)	2006
4012	P. Navasumrit	Arsenic projects in SE Asia	2016

4013	S. Klos	The Gallate Pnictides Ba-9 GaO4 (3)Pn (Pn=Sb, Bi) and a Partially Oxidized Zintl Anion in Ba-2 GaO2As	2022
4014	A. Aguilar-Garrido	Arsenic Fixation in Polluted Soils by Peat Applications	2020
4015	V. A. Fedorov	Physicochemical Aspects of the Preparation of High-Purity Arsenic-Containing Substances from Various Raw Materials	2017
4016	J. H. Lee	Microbially Facilitated Incorporation of As(III) into Bioreduced Fe-(hydr)oxide Minerals	2014
4017	M. M. Hussain	Arsenic speciation and biotransformation pathways in the aquatic ecosystem: The significance of algae	2021
4018	P. A. Gunka	High-pressure and low-temperature structural study of claudetite I, a monoclinic layered As2O3 polymorph	2021
4019	M. Gonzalez-Moscoso	Silicon nanoparticles decrease arsenic translocation and mitigate phytotoxicity in tomato plants	2022
4020	S. Z. Chen	Analysis of Arsenic Metabolites of Realgar in Rat Viscera by HPLC-ICP-MS	2014
4021	N. Yan	Developmental arsenic exposure impairs cognition, directly targets DNMT3A, and reduces DNA methylation	2022
4022	M. A. Lala	Preparation of 2,3,4-trihydroxybutylarsonic acid: A starting compound for novel arsonolipids	2007
4023	R. Y. Mao	Characteristics and compound-specific carbon isotope compositions of sedimentary lipids in high arsenic aquifers in the Hetao basin, Inner Mongolia	2018
4024	G. Z. Kyzas	Adsorption of As(III) and As(V) onto colloidal microparticles of commercial cross-linked polyallylamine (Sevelamer) from single and binary ion solutions	2016
4025	S. J. Zou	Nanocomposites of graphene and zirconia for adsorption of organic-arsenic drugs: Performances comparison and analysis of adsorption behavior	2021
4026	S. Li	Re-using of coal-fired fly ash for arsenic vapors in-situ retention before SCR catalyst: Experiments and mechanisms	2020
4027	E. R. Naranov	Development of Ni-Mo Sorption-Catalytic Materials for Removing Arsenic Compounds from Middle Distillates	2018
4028	X. W. Yu	Mono-acyl arsenosugar phospholipids in the edible brown alga Kombu (Saccharina japonica)	2018
4029	S. Braeuer	Homoarsenocholine - A novel arsenic compound detected for the first time in nature	2018
4030	I. Mirkov	Plant Extracts and Isolated Compounds Reduce Parameters of Oxidative Stress Induced by Heavy Metals: An up-to-Date Review on Animal Studies	2020

4031	N. Jing	Ultra-Performance Liquid Chromatography-Tandem Mass Spectrometry for the Analysis of Complex Compounds in Serum and Its Application in Accurate Detection of Early Arsenic Exposure	2021
4032	D. Piacentini	Nitric oxide alleviates cadmium- but not arsenic-induced damages in rice roots	2020
4033	M. S. Taleshi	Arsenic-containing hydrocarbons: natural compounds in oil from the fish capelin, Mallotus villosus	2008
4034	R. K. Mohanta	Blood Biochemistry, Thyroid Hormones, and Oxidant/Antioxidant Status of Guinea Pigs Challenged with Sodium Arsenite or Arsenic Trioxide	2014
4035	A. O. Sydykov	Fire Refining of Rough Antimony from Impurities for Obtaining High-Grade Antimony	2022
4036	M. J. Amenabar	Mechanisms of Mineral Substrate Acquisition in a Thermoacidophile	2018
4037	F. Makavipour	Low-Level Arsenic Removal from Drinking Water	2019
4038	M. Kuramata	Arsinothricin, a novel organoarsenic species produced by a rice rhizosphere bacterium	2016
4039	M. Parvez	Suffering water of Pakistan: arsenic - A major threat	2016
4040	Y. F. Li	Chronic Arsenic Poisoning Probably Caused by Arsenic-Based Pesticides: Findings from an Investigation Study of a Household	2016
4041	M. R. Ramudzuli	Arsenic residues in soil at cattle dip tanks in the Vhembe district, Limpopo Province, South Africa	2014
4042	H. Y. Guo	Structural influences of arsenic-vanadium clusters and transition metal complexes on final structures of arsenic-vanadium-based hybrids	2016
4043	S. Sineva	Development of Experimental Approach for the Phase Equilibria Study of Arsenic-Containing Systems	2019
4044	C. Zhou	Trace elements profiles of maternal blood, umbilical cord blood, and placenta in Beijing, China	2019
4045	H. R. Hansen	2-dimethylarsinothioyl acetic acid identified in a biological sample: The first occurrence of a mammalian arsinothioyl metabolite	2004
4046	A. Pardo	Monodisperse superparamagnetic nanoparticles separation adsorbents for high-yield removal of arsenic and/or mercury metals in aqueous media	2021
4047	H. Viltres	Degradation study of arsenic oxides under XPS measurements	2020
4048	C. Song	The effect of particle size and metal contents on arsenic distribution in coal-fired fly ash	2014

4049	B. M. Huang	Anticancer effect of arsenic compounds on apoptosis in oral cavity cancer cells	2018
4050	X. B. Hu	Distributions of Heavy Metals in Maternal and Cord Blood and the Association with Infant Birth Weight in China	2015
4051	T. Tziaras	Investigating the Occurrence and Environmental Significance of Methylated Arsenic Species in Atmospheric Particles by Overcoming Analytical Method Limitations	2015
4052	A. Hauser	Reactivity of mono- and divalent aluminium compounds towards group 15 nanoparticles	2021
4053	X. S. Du	Adsorption of As4O6 from flue gas by zeolites: Influence of pore structure and Al substitution	2017
4054	C. C. Ni	Self-powered peroxi-coagulation for the efficient removal of p-arsanilic acid: pH-dependent shift in the contributions of peroxidation and electrocoagulation	2020
4055	N. Chen	Fast transformation of roxarsone into toxic arsenic species with ferrous iron and tetrapolyphosphate	2019
4056	S. Goldsberry	Development of instrumental analysis lab experiment for the determination of organo-arsenic compounds	2017
4057	E. Navarro-Tapia	Toxic Elements in Traditional Kohl-Based Eye Cosmetics in Spanish and German Markets	2021
4058	M. Stehlik	I-divergence based statistical inference for heteroscedasticity and compounds of arsenic contamination in Chile	2022
4059	H. D. B. Jenkins	Thermodynamic data for crystalline arsenic and phosphorus compounds M2O5 center dot nH(2)O re-examined using the Thermodynamic Difference Rules	2016
4060	B. M. Kim	Fabrication and characterisation of field-effect transistor-type pressure sensor with metal-oxide-semiconductor/microelectromechanical systems processes	2015
4061	M. Driess	Lithium arsanylalanates as arsenide sources: A simple route to the Zintl anion As-7(3-) and the synthesis of a tetraarsatetrasilacubane	1996
4062	B. Islam	Toxic neuropathy: An unaddressed menace to Bangladesh	2020
4063	Y. Wu	The effects of iron(II) on the kinetics of arsenic oxidation and sorption on manganese oxides	2015
4064	R. Albrecht	Chalcogenides by Reduction of their Dioxides in Ultra-Alkaline Media	2021
4065	V. V. Turygin	Physicochemical Principles behind the Preparation of High-Purity Arsenic Compounds from Lewisite Detoxification Products	2017
4066	I. Rujido-Santos	Metal Content in Textile and (Nano)Textile Products	2022

4067	M. Driess	DIPHOSPHANYL-SUBSTITUTED AND DIARSANYL-SUBSTITUTED CARBENE HOMOLOGS - GERMANEDIYLS, STANNANEDIYLS, AND PLUMBANEDIYLS WITH REMARKABLE ELECTRONIC-STRUCTURES	1995
4068	Y. L. Lin	Preparation of CuZnAl hydrotalcite-like catalysts for AsH3 abatement at low temperatures	2019
4069	J. Tremlova	A profile of arsenic species in different vegetables growing in arsenic-contaminated soils	2017
4070	P. Binu	Eugenol, a plant-derived phenolic nutraceutical, protects thiol (SH) group in myocardium from ROS-mediated oxidation under chemotherapeutic stress induced by arsenic trioxide - a in vivo model study	2018
4071	M. Seidl	The Chemistry of Yellow Arsenic	2019
4072	O. Linhart	Determination of As by UV-photochemical generation of its volatile species with AAS detection	2016
4073	J. W. Zabinski	Advancing Dose-Response Assessment Methods for Environmental Regulatory Impact Analysis: A Bayesian Belief Network Approach Applied to Inorganic Arsenic	2016
4074	C. Schoo	Samarium Polyarsenides Derived from Nanoscale Arsenic	2019
4075	S. A. Viczek	Arsenic-Containing Phosphatidylcholines: A New Group of Arsenolipids Discovered in Herring Caviar	2016
4076	R. Chauhan	Selenite modulates the level of phenolics and nutrient element to alleviate the toxicity of arsenite in rice (Oryza sativa L.)	2017
4077	J. M. Goodrich	First trimester maternal exposures to endocrine disrupting chemicals and metals and fetal size in the Michigan Mother-Infant Pairs study	2019
4078	N. C. Sumedha	Cardiac mitochondrial oxidative stress and dysfunction induced by arsenic and its amelioration by diallyl trisulphide (Publication with Expression of Concern. See vol. 9, pg. 855, 2020) (Publication with Expression of Concern. See vol. 10, pg. 664, 2021) (Retracted	2015
4079	V. Urriade	Arsenic inorganic compounds cause oxidative stress mediated by the transcription factor PHO4 in Candida albicans	2017
4080	J. M. Byun	EFFECTS OF ARSENIC COMPOUNDS ON CELL-CYCLE DISTRIBUTION AND APOPTOSIS OF CHEMORESISTANT OVARIAN CANCER CELL LINES	2018
4081	C. Wang	Mechanochemical synthesis of bismuth-based anion exchange materials to immobilize arsenic pollution - Prospects for advanced treatment of anion-containing wastewater	2022
4082	Y. J. Wang	Removal of roxarsone from aqueous solution by Fe/La-modified montmorillonite	2016
4083	Y. W. Wu	Theoretical insight into the interaction mechanism between V2O5/TiO2 (001) surface and arsenic oxides in flue gas	2021
4084	J. Majzlan	Thermodynamic properties of mansfieldite (AlAsO4 center dot 2H(2)O), angelellite (Fe-4(AsO4)(2)O-3) and kamarizaite (Fe-3(AsO4)(2)(OH)(3)center dot 3H(2)O)	2018

4085	N. C. Sumedha	Diallyl trisulfide ameliorates arsenic-induced hepatotoxicity by abrogation of oxidative stress, inflammation, and apoptosis in rats	2015
4086	B. E. Tew	Growth and Thermal Characterization of TbAs Nanoparticles Grown by Inert Gas Condensation	2020
4087	S. Braeuer	Arsenic species in mushrooms, with a focus on analytical methods for their determination - A critical review	2019
4088	J. Hellal	Experimental Column Setup for Studying Anaerobic Biogeochemical Interactions Between Iron (Oxy) Hydroxides, Trace Elements, and Bacteria	2017
4089	J. Falandysz	Arsenic and its compounds in mushrooms: A review	2016
4090	J. Y. Zheng	Thermodynamic research on the effect of CaO on the transformation of arsenic and sulfur in coal in different pyrolysis atmospheres	
4091	Y. H. Wang	Effects of different dissolved organic matter on microbial communities and arsenic mobilization in aquifers	2021
4092	M. C. Ruiz	Digestion kinetics of arsenic removal from enargite-tennantite concentrates	2015
4093	C. Durand	Assessment of exposure to soils contaminated with lead, cadmium, and arsenic near a zinc smelter, Cassiopee Study, France, 2008	2015
4094	T. T. Shi	Arsenic removal from arsenic-containing copper dust by vacuum carbothermal reduction-vulcanization roasting	2021
4095	E. V. Avtomonov	Syntheses and structures of cyclopentadienyl arsenic compounds .2. Pentamethyl- and tetraisopropylcyclopentadienyl arsenic amido derivatives	1997
4096	M. Button	Arsenic speciation in the bracket fungus Fomitopsis betulina from contaminated and pristine sites	2020
4097	U. Wirringa	THE 1ST HETEROALLYL METAL-COMPLEXES OF ARSENIC WITH THE COORDINATION NUMBER-2	1993
4098	S. Chandra	Assessment of Arsenic Toxicity and Tolerance Characteristics of Bean Plants (Phaseolus Vulgaris) Exposed to Different Species of Arsenic	2018
4099	S. Braeuer	Unusual arsenic metabolism in Giant Pandas	2017
4100	M. Stiboller	Arsenolipids Detected in the Milk of Nursing Mothers	2017
4101	A. Nakahashi	1,4-dihydro-1,4-diarsinine: Facile synthesis via nonvolatile arsenic intermediates by radical reactions	2007
4102	O. Hegen	The Lewis-Base-Stabilized Diphenyl-Substituted Arsanylborane: A Versatile Building Block for Arsanylborane Oligomers	2018

4103	S. Hesse	Chemical vapor generation by coupling high-pressure liquid flow injection to high-resolution continuum source hydride generation atomic absorption spectrometry for determination of arsenic	2015
4104	L. Dheilly	SYNTHESIS OF C-GLYCOSIDES - USE OF AN ARSENIC DERIVATIVE	1993
4105	T. V. Bezyazychnaya	INFLUENCE OF VACANCIES ON INDIUM ATOM DISTRIBUTION IN InGaAs AND InGaN COMPOUNDS	2015
4106	P. Saharan	Removal of Water Contaminants by Iron Oxide Nanomaterials	2014
4107	Y. Kumagai	EVALUATION OF CARDIOVASCULAR SAFETY OF DARINAPARSIN (ORGANIC ARSENIC COMPOUND) IN JAPANESE AND KOREAN PATIENTS WITH PERIPHERAL T-CELL LYMPHOMA	2015
4108	J. H. Jo	KML001, AN ARSENIC COMPOUND, AS A PALLIATIVE CHEMOTHERAPY IN ADVANCED BILIARY TRACT CANCERS RESISTANT TO GEMCITABINE : A PROSPECTIVE STUDY	2018
4109	V. Vishwanath	Evolution of arsenic in high fluence plasma immersion ion implanted silicon: Behavior of the as-implanted Surface	2015
4110	B. Radke	Arsenic-Based Warfare Agents: Production, Use, and Destruction	2014
4111	M. J. Clemente	Dietary Compounds To Reduce In Vivo Inorganic Arsenic Bioavailability	2019
4112	W. Zhang	Simultaneous adsorption and oxidation of para arsanilic acid by a highly efficient nanostructured Fe-Ti-Mn composite oxide	2021
4113	Y. H. Li	Solvothermal syntheses, structures, and characterizations of four thioarsenates A(7)Cu(4)As(3)S(13) (A = Rb, Cs), Rb2Cu5As3S8, and CsCu2AsS3	2022
4114	C. Tian	Enhanced removal of roxarsone by Fe3O4@3D graphene nanocomposites: synergistic adsorption and mechanism	2017
4115	R. D. Foust	Arsenic transfer and biotransformation in a fully characterized freshwater food web	2016
4116	J. Ming	Oral Arsenic-Containing Qinghuang Powder (QHP): A Potential Drug for Myelodysplastic Syndromes	
4117	A. V. Marchenko	Structural chemical states of dopant atoms of platinum and gold in glass-like arsenic selenides	2016
4118	B. Radke	Many faces of arsenic	2019
4119	R. L. Carpenter	Safety and Tolerability of Sonic Hedgehog Pathway Inhibitors in Cancer	2019
4120	J. E. Rager	Benchmark Dose Modeling Estimates of the Concentrations-of Inorganic Arsenic That Induce Changes to the Neonatal Transcriptome, Proteome, and Epigenome in a Pregnancy Cohort	2017

4121	O. Benavente	Stabilizing Arsenic in Copper Heap Leaching Residues	2020
4122	J. Xu	A novel transformation pathway of p-arsanilic acid in water by colloid ferric hydroxide under UVA light	2022
4123	M. Y. Ren	Associations between hair levels of trace elements and the risk of preterm birth among pregnant Wwomen: A prospective nested case-control study in Beijing Birth Cohort (BBC), China	2022
4124	K. Y. Koh	Critical review on lanthanum-based materials used for water purification through adsorption of inorganic contaminants	2022
4125	F. Ardini	Arsenic speciation analysis of environmental samples	2020
4126	F. L. Fu	Synthesis and use of bimetals and bimetal oxides in contaminants removal from water: a review	2015
4127	Y. W. Wu	Effect of WO3 and MoO3 doping on the interaction mechanism between arsenic oxide and V2O5-based SCR catalyst: A theoretical account	2021
4128	L. Weerasundara	Selective removal of arsenic in water: A critical review	2021
4129	H. Imoto	Recent progress on arsenic-containing functional polymers	2022
4130	S. H. Yu	Insight into As2O3 adsorption characteristics by mineral oxide sorbents: Experimental and DFT study	2021
4131	I. Biancarosa	Replacing fish meal with insect meal in the diet of Atlantic salmon (Salmo salar) does not impact the amount of contaminants in the feed and it lowers accumulation of arsenic in the fillet	2019
4132	Y. H. Duan	Photochemical Degradation of Arsenic and Selenium with Advanced Reduction Processes-Effects of Reagents	2017
4133	G. Somer	A new and sensitive method for the determination of trace arsenic using differential pulse polarography	2014
4134	J. L. Wu	Broad-spectrum rescue compounds for structural p53 mutations: perspective on 'Arsenic trioxide rescues structural p53 mutations through a cryptic allosteric site'	2021
4135	Y. Z. Zhao	Effect of the Addition of Biomass on the Fate and Speciation of Arsenic during Sewage Sludge Combustion	2018
4136	J. P. Maity	Advanced application of nano-technological and biological processes as well as mitigation options for arsenic removal	2021
4137	B. O. Anyanwu	Heavy Metal Mixture Exposure and Effects in Developing Nations: An Update	2018
4138	W. S. Lee	Tetraarsenic hexoxide demonstrates anticancer activity at least in part through suppression of NF-kappa B activity in SW620 human colon cancer cells	2015

4139	K. M. Wai	Global Atmospheric Transport and Source-Receptor Relationships for Arsenic	2016
4140	E. Conrad	Coordination of Arsine Ligands as a General Synthetic Approach to Rare Examples of Arsenic-Antimony and Arsenic-Bismuth Bonds	2009
4141	W. Lorenc	Arsenic species and their transformation pathways in marine plants. Usefulness of advanced hyphenated techniques HPLC/ICP-MS and UPLC/ESI-MS/MS in arsenic species analysis	2020
4142	B. D. Ellis	Stabilised phosphorus(I) and arsenic(I) iodide: readily-synthesised reagents for low oxidation state main group chemistry	2003
4143	J. Zhang	Investigating the Neurotoxic Impacts of Arsenic and the Neuroprotective Effects of Dictyophora Polysaccharide Using SWATH-MS-Based Proteomics	2022
4144	P. Grandjean	Trace elements as paradigms of developmental neurotoxicants: Lead, methylmercury and arsenic	2015
4145	Y. Guan	Two arsenic capped Dawson-type supramolecular hybrid assemblies induced by benzimidazole for photo-/electro-catalytic performance	2020
4146	J. F. Xu	Arsenic compound sensitizes homologous recombination proficient ovarian cancer to PARP inhibitors	2021
4147	Z. Y. Zhao	Selective sequestration of p-arsanilic acid from water by using nano-hydrated zirconium oxide encapsulated inside hyper-cross-linked anion exchanger	2020
4148	G. Pfeifer	Clicking the Arsenic-Carbon Triple Bond: An Entry into a New Class of Arsenic Heterocycles	2016
4149	Z. Zhang	Theoretical Investigation of Arsenic and Selenium Species Adsorption Behavior on Different Mineral Adsorbents	2019
4150	T. T. Zhang	Enhanced arsenic removal from water by mechanochemical synthesis of Ca-Al-Fe ternary composites	2021
4151	W. S. Tay	C-As Bond Formation Reactions for the Preparation of Organoarsenic(III) Compounds	2020
4152	Y. C. Lv	Remediation of organic arsenic contaminants with heterogeneous Fenton process mediated by SiO2-coated nano zero-valent iron	2020
4153	Z. Olah	Optimization of the reduction of As-74(V) to As-74(III) and of the labelling of dithiol dihydrolipoic acid	2019
4154	C. Afonso	Bioaccessibility in risk-benefit analysis of raw and cooked seabream consumption	2018
4155	Y. Wu	Iron and Arsenic Speciation During As(III) Oxidation by Manganese Oxides in the Presence of Fe(II): Molecular-Level Characterization Using XAFS, Mossbauer, and TEM Analysis	2018
4156	M. Al-Sid-Cheikh	Interactions between natural organic matter, sulfur, arsenic and iron oxides in re-oxidation compounds within riparian wetlands: NanoSIMS and X-ray adsorption spectroscopy evidences	2015

4157	A. K. Serikbaeva	Development of a Method for Reprocessing Technogenic Lead Production Raw Materials to Extract Rhenium and Arsenic	2021
4158	I. Shiue	Urinary arsenic, pesticides, heavy metals, phthalates, polyaromatic hydrocarbons, and polyfluoroalkyl compounds are associated with sleep troubles in adults: USA NHANES, 2005-2006	2017
4159	M. Khan	Preliminary studies on the stability of arsenolipids: Implications for sample handling and analysis	2016
4160	G. Bjorklund	Effects of arsenic toxicity beyond epigenetic modifications	2018
4161	A. C. Niehoff	Imaging by Elemental and Molecular Mass Spectrometry Reveals the Uptake of an Arsenolipid in the Brain of Drosophila melanogaster	2016
4162	M. C. Shui	Impact of roxarsone on the UASB reactor performance and its degradation	2016
4163	E. E. Ramsay	Glutathione S-conjugates as prodrugs to target drug-resistant tumors	2014
4164	D. Cortes-Arriagada	Removal of arsenic from water using iron-doped phosphorene nanoadsorbents: A theoretical DFT study with solvent effects	2020
4165	D. Lu	Arsenicin A, A Natural Polyarsenical: Synthesis and Crystal Structure	2010
4166	M. S. Rahaman	Investigating the protective actions of D-pinitol against arsenic-induced toxicity in PC12 cells and the underlying mechanism	2020
4167	X. Z. Gong	Chinese Medicine Might Be A Promising Way for A Solution to Arsenic Nephrotoxicity	2020
4168	S. Das	Bioaccumulation and cytological alteration of immune organs of chicken following inorganic arsenic exposure	2020
4169	I. C. F. Vasques	Arsenite removal from contaminated water by precipitation of aluminum, ferrous and ferric (hydr)oxides	2018
4170	S. U. Dani	Chronic arsenic intoxication diagnostic score (CAsIDS)	2018
4171	M. A. Dovick	Extreme Arsenic and Antimony Uptake and Tolerance in Toad Tadpoles during Development in Highly Contaminated Wetlands	2020
4172	Y. N. Vodyanitskii	Biogeochemistry of heavy metals in contaminated excessively moistened soils (Analytical review)	2014
4173	S. Pinol	Arsenic levels in immigrant children from countries at risk of consuming arsenic polluted water compared to children from Barcelona	2015
4174	J. W. Wang	Effect of Coordinated Air Pollution Control Devices in Coal-Fired Power Plants on Arsenic Emissions	2017

4175	S. Sabbagh	Arsenic contamination in rice, radiation and chemical methods of measurement, and implications for food safety	
4176	N. K. Mondal	Chicken litter: a potential source of arsenic in agricultural soil and its contamination in Cajanus cajan	2022
4177	C. X. Deng	Passivating effect of dehydrated sludge and sepiolite on arsenic contaminated soil	2018
4178	X. D. Xie	Rapid degradation of p-arsanilic acid with simultaneous arsenic removal from aqueous solution using Fenton process	2016
4179	A. B. Sravani	Human papillomavirus infection, cervical cancer and the less explored role of trace elements	
4180	H. H. Zhou	Mineralogical and morphological factors affecting the separation of copper and arsenic in flash copper smelting slag flotation beneficiation process	2021
4181	C. Zaccone	Methylated arsenic species throughout a 4-m deep core from a free-floating peat island	2018
4182	J. Wang	Biotransformation and biomethylation of arsenic by Shewanella oneidensis MR-1	2016
4183	J. Bresien	As-N and As-N-P Cage Compounds Generated by 2+2 Addition of Diazenes and Diphosphenes to Diarsadiazanediyls	2018
4184	C. Grassl	Synthesis of arsenic-rich As-n ligand complexes from yellow arsenic	2015
4185	N. Guttenberger	Facile access to arsenic-containing triacylglycerides	2017
4186	W. S. Kim	Two Phase I and Pharmacokinetic Studies of Darinaparsin (Organic Arsenic Compound) in Japanese and Korean Patients with Relapsed or Refractory (R/R) Peripheral T-Cell Lymphoma (PTCL)	2015
4187	A. Doddi	Isolation of Carbene-Stabilized Arsenic Monophosphide AsP and its Radical Cation AsP (+.) and Dication AsP (2+)	2019
4188	A. M. Koval	Density functional theory (DFT) investigation of the oxidative degradation of NaAsO2 via hydroxyl radical	2022
4189	R. G. Ahmed	Gestational Arsenic Trioxide Exposure Acts as a Developing Neuroendocrine-Disruptor by Downregulating Nrf2/PPAR gamma and Upregulating Caspase-3/NF-B/Cox2/BAX/iNOS/ROS	2019
4190	H. J. Sanchez	Arsenic Speciation by X-Ray Spectroscopy using Resonant Raman Scattering	2014
4191	D. S. Babu	Detoxification of water and wastewater by advanced oxidation processes	2019
4192	N. Guttenberger	Synthetic access to arsenic-containing phosphatidylcholines	2017

4193	R. Bai	ACCUMULATION AND REDUCTION OF ARSENIC BY Tetrahymena thermophila strain SB-4220	2015
4194	P. V. Ioannou	Trithioarsenites (RS)(3)As , dithioarsonites R-As(SR ')(2) and thioarsinites R2As-SR ' : Preparations, chemical, biochemical and biological properties	2022
4195	A. E. Seitz	Different Reactivity of As-4 towards Disilenes and Silylenes	2017
4196	C. A. Gaulke	Marginal Zinc Deficiency and Environmentally Relevant Concentrations of Arsenic Elicit Combined Effects on the Gut Microbiome	2018
4197	F. P. Freitas	Lipids that contain arsenic in the Mediterranean mussel, Mytilus galloprovincialis	2020
4198	W. Q. Chen	Anthropogenic arsenic cycles: A research framework and features	2016
4199	C. Zou	The effect of CO on the transformation of arsenic species: A quantum chemistry study	2019
4200	E. M. Gross	Sensitive response of sediment-grown Myriophyllum spicatum L. to arsenic pollution under different CO2 availability	2018
4201	C. Ritter	Investigation on Arsenic-Antimony- and Arsenic-Bismuth Bond Containing Interpnictogen Chain Molecules	2021
4202	S. Ince	Boron ameliorates arsenic-induced DNA damage, proinflammatory cytokine gene expressions, oxidant/antioxidant status, and biochemical parameters in rats	2019
4203	M. Mirazimi	Kinetics and mechanisms of arsenic and sulfur release from crystalline orpiment	2021
4204	G. Jenness	Nature of contaminants in arid soil: A computational study on the adsorption of munition compounds and arsenic species on the (0001) surface of alpha-Fe2O3 and alpha-Al2O3	2018
4205	T. Llorente-Mirandes	Occurrence of inorganic arsenic in edible Shiitake (Lentinula edodes) products	2014
4206	J. Shim	Arsenic inhibits mast cell degranulation via suppression of early tyrosine phosphorylation events	2016
4207	S. L. Chadha	ARSENIC(III) ALKOXIDES CONTAINING 2,2,2-TRIFLUOROETHOXY AND 1,1,1,3,3,3-HEXAFLUOROISOPROPOXY LIGANDS	1995
4208	M. Vermeulen	Identification by Raman spectroscopy of pararealgar as a starting material in the synthesis of amorphous arsenic sulfide pigments	2018
4209	M. N. Zhang	Facile electrochemical synthesis of nano iron porous coordination polymer using scrap iron for simultaneous and cost-effective removal of organic and inorganic arsenic	2018
4210	D. Bockfeld	Isolation of N-Heterocyclic Carbene-Stabilized Phosphorus and Arsenic Mononitride	2020

4211	A. Murciego	Characterization of secondary products in arsenopyrite-bearing mine wastes: influence of cementation on arsenic attenuation	2019
4212	J. Lei	Effects of anions on calcium arsenate crystalline structure and arsenic stability	2018
4213	M. Rolle	Mixing and Reactive Fronts in the Subsurface	2019
4214	S. Mucha	Mechanisms of arsenic toxicity and transport in microorganisms	2017
4215	K. Karimov	Deposition of Arsenic from Nitric Acid Leaching Solutions of Gold-Arsenic Sulphide Concentrates	2021
4216	C. Liang	Arsenic trioxide and all-trans retinoic acid suppress the expression of FLT3-ITD	2020
4217	R. Juncos	Interspecific differences in the bioaccumulation of arsenic of three Patagonian top predator fish: Organ distribution and arsenic speciation	2019
4218	S. F. Gamper	SYNTHESIS AND MOLECULAR-STRUCTURE OF HETEROCYCLES CONTAINING 2 PHOSPHORUS(V) CENTERS BRIDGED BY 2-COORDINATE PHOSPHORUS AND ARSENIC	1993
4219	L. Chi	Chronic Arsenic Exposure Induces Oxidative Stress and Perturbs Serum Lysolipids and Fecal Unsaturated Fatty Acid Metabolism	2019
4220	R. Keren	Global genomic analysis of microbial biotransformation of arsenic highlights the importance of arsenic methylation in environmental and human microbiomes	2022
4221	A. Hernandez	Micronucleus frequency in copper-mine workers exposed to arsenic is modulated by the AS3MT Met287Thr polymorphism	2014
4222	H. Y. Guo	New self-assembly hybrid compounds based on arsenic-vanadium clusters and transition metal mixed-organic-ligand complexes	2016
4223	P. Millard	Isotopic Studies of Metabolic Systems by Mass Spectrometry: Using Pascal's Triangle To Produce Biological Standards with Fully Controlled Labeling Patterns	2014
4224	J. Lv	Selective detection of two representative organic arsenic compounds in aqueous medium with metal-organic frameworks	2019
4225	S. Hwang	Characteristics of Arsenic Leached from Sediments: Agricultural Implications of Abandoned Mines	2019
4226	T. L. Tsai	Maternal and childhood exposure to inorganic arsenic and airway allergy - A 15-Year birth cohort follow-up study	2021
4227	C. Xiong	Transport of arsenolipids to the milk of a nursing mother after consuming salmon fish	2020
4228	M. Goudjil	Synthesis and crystal structure of a series of stoichiometric (n)-ITB molybdenum-bronze oxides containing trivalent arsenic	2019

4229	F. Di Caprio	Two-phase synthesis of Fe-loaded hydrochar for As removal: The distinct effects of initial pH, reaction time and Fe/hydrochar ratio	2022
4230	R. H. Lara	Arsenopyrite weathering under conditions of simulated calcareous soil	2016
4231	A. Roy	Electronic structure and properties of Cd4As2Br3 and Cd4Sb2I3, analogues of CdSe and CdTe	2017
4232	Z. Q. Shen	Identification of a Novel Membrane Transporter Mediating Resistance to Organic Arsenic in Campylobacter jejuni	2014
4233	Y. K. Li	Removal and immobilization of arsenic from copper smelting wastewater using copper slag by in situ encapsulation with silica gel	2020
4234	D. Xu	Acclimation and adaptation to elevated pCO(2) increase arsenic resilience in marine diatoms	2021
4235	G. A. Belogolova	Speciation of arsenic and its accumulation by plants from rhizosphere soils under the influence of Azotobacter and Bacillus bacteria	2015
4236	E. Modestin	Arsenic in Caribbean bivalves in the context of Sargassum beachings: A new risk for seafood consumers	2022
4237	H. X. Xing	In-Furnace Control of Arsenic Vapor Emissions Using Kaolinite during Low-Rank Coal Combustion: Influence of Gaseous Sodium Compounds	2019
4238	M. A. Petrie	SYNTHESES, STRUCTURES, AND SPECTROSCOPIC STUDIES OF SEVERAL NEW CLASSES OF COMPOUNDS HAVING BORON ARSENIC BONDS	1993
4239	X. L. Liu	Occurrence, speciation analysis and health risk assessment of arsenic in Chinese mitten crabs (Eriocheir sinensis) collected from China	2020
4240	D. P. Wang	Total arsenic and speciation analysis of saliva and urine samples from individuals living in a chronic arsenicosis area in China	2017
4241	M. Nowicka	CHARACTERIZATION OF EUKARYOTIC ARSENATE REDUCTASES OF RHODANESE SUPERFAMILY	2020
4242	X. M. Xue	Biosynthesis of arsenolipids by the cyanobacterium Synechocystis sp. PCC 6803	2014
4243	M. Hadizadeh	Arsenic Removal from Lead Concentrate-Containing Mimetite Mineral to Solve the Environmental Problem for Smelting Process	2021
4244	X. D. Xie	A simple treatment method for phenylarsenic compounds: Oxidation by ferrate (VI) and simultaneous removal of the arsenate released with in situ formed Fe(III) oxide-hydroxide	2019
4245	D. Mandal	Isolation and identification of arsenic resistant bacteria: a tool for bioremediation of arsenic toxicity	
4246	K. Renu	An appraisal on molecular and biochemical signalling cascades during arsenic-induced hepatotoxicity	2020

4247	H. Y. Hu	Adsorption and reaction mechanism, of arsenic vapors over gamma-Al2O3 in the simulated flue gas containing acid gases	2017
4248	S. Liu	An efficient and clean method for the selective separation of arsenic from scrap copper anode slime containing high arsenic and tin	2022
4249	I. Palma-Lara	Arsenic exposure: A public health problem leading to several cancers	2020
4250	K. Hassler	Tridecamethyl-1-phospha- and tridecamethyl-1-arsa-2,3,4,5,6,7,8-heptasilabicyclo 2.2.2 octane	1997
4251	S. Tanaka	A practical method for the generation of organoarsenic nucleophiles towards the construction of a versatile arsenic library	2016
4252	Y. Fransisca	Assessment of arsenic in Australian grown and imported rice varieties on sale in Australia and potential links with irrigation practises and soil geochemistry	2015
4253	P. P. Wang	A review on completing arsenic biogeochemical cycle: Microbial volatilization of arsines in environment	2014
4254	E. M. Bomhard	The toxicology of gallium oxide in comparison with gallium arsenide and indium oxide	2020
4255	L. Wang	Ethanol enhances arsenic-induced cyclooxygenase-2 expression via both NFAT and NF-kappa B signalings in colorectal cancer cells	2015
4256	A. Oliveira	Fractionation of inorganic arsenic by adjusting hydrogen ion concentration	2016
4257	W. C. Song	Immobilization of As(V) in Rhizopus oryzae Investigated by Batch and XAFS Techniques	2016
4258	S. L. Chadha	Phosphorus(III), phosphoryl(V), and arsenic(III) alkoxides containing 2,2,2,-trichloroethoxy groups and their pyridine adducts	1997
4259	J. C. Mao	Study on interaction mechanism of different atomic ratio of neodymium, arsenic and iron	2021
4260	Y. Y. Xu	Assessing the Role of Nrf2/GPX4-Mediated Oxidative Stress in Arsenic-Induced Liver Damage and the Potential Application Value of Rosa roxburghii Tratt Rosaceae	2022
4261	A. Arrieta	The birthweight toll of mining pollution: evidence from the most contaminated mine site in the Andean region	2018
4262	Q. L. Yang	Novel Ionic Grafts That Enhance Arsenic Removal via Forward Osmosis	2019
4263	S. Q. Asadullayeva	Cross-relaxation energy transfer between the Er3+ ions in vitreous arsenic chalcogenide	2019
4264	X. Zhou	Rational design an amorphous multifunctional delta-MnO2@Fe/Mg-MIL-88B nanocomposites with tailored components for efficient and rapid removal of arsenic in water	2020

4265	M. S. Safarzadeh	The behavior of arsenic trioxide in non-ferrous extractive metallurgical processing	2014
4266	T. Shepherd	Post-depositional behaviour of mercury and arsenic in submarine mine tailings deposited in Buyat Bay, North Sulawesi, Indonesia	2018
4267	D. E. Pratama	Unconventional separation of arsenic trioxide from unused aqueous chemotherapeutic agents by direct evaporative crystallization	2021
4268	H. M. Marwani	Cellulose acetate-iron oxide nanocomposites for trace detection of fluorene from water samples by solid-phase extraction technique	2018
4269	S. S. Garje	Synthesis and characterisation of organoarsenic(III) xanthates and dithiocarbamates. X-ray crystal structures of RAs(S2CNEt2)(2), R=Me and Ph	1997
4270	S. H. Lee	Feasibility of quantitative inorganic arsenic speciation at the parts-per-trillion level using solid phase extraction and femtosecond laser ablation inductively coupled plasma mass spectrometry	2021
4271	D. Tong	Arsenic Inhibits DNA Mismatch Repair by Promoting EGFR Expression and PCNA Phosphorylation	2015
4272	W. B. Lee	Accurate Measurement of Total Arsenic in Rice and Oyster by Considering Arsenic Species	2019
4273	H. Zhang	Recovery of Arsenic Trioxide from a Sludge-Like Waste by Alkaline Leaching and Acid Precipitation	2014
4274	Z. Q. Qiu	Species distribution characteristics of arsenic in shellfish seafood collected from Fujian Province of China	2018
4275	M. Gulden	Chrysin and silibinin sensitize human glioblastoma cells for arsenic trioxide	2017
4276	M. Silahli	The relationship between placental transfusion, and thymic size and neonatal morbidities in premature infants - A Randomized Control Tiral	2018
4277	M. Shpotyuk	Surface oxidation in glassy arsenic trisulphide induced by high-energy gamma-irradiation	2014
4278	V. Jadhav	Synthesis and Characterization of Arsenic Trioxide Nanoparticles and Their In Vitro Cytotoxicity Studies on Mouse Fibroblast and Prostate Cancer Cell Lines	2016
4279	P. Perez-Portilla	Potential of arsenic bioremediation by a cyanobacterium isolated from the Salado River in the Atacama Desert	2021
4280	S. Das	GCMS analysis of sadagura (smokeless tobacco), its enhanced genomic instability causing potential due to arsenic co-exposure, and vitamin-C supplementation as a possible remedial measure: a study involving multiple model test systems	2022
4281	B. Wang	Pure total flavonoids from Citrus paradisi Macfadyen act synergistically with arsenic trioxide in inducing apoptosis of Kasumi-1 leukemia cells in vitro	2015
4282	Y. X. Zheng	Separation of arsenic and tin from Cu-As alloy based on phase transformation in a vacuum to form Cu-Fe-S compounds	2021

4283	J. Zhang	Arsenic and arsenic speciation in mushrooms from China: A review	2020
4284	V. Pilicita	Filter design for arsenic species in aqueous environments: An ab initio optimization of the absorbing capacity of magnetite-based arsenic filters	2021
4285	G. Melinte	Electrochemical Fingerprint of Arsenic (III) by Using Hybrid Nanocomposite-Based Platforms	2019
4286	R. Garbe	Phosphane iminato complexes of arsenic. Crystal structures of AsCl(NPMe(3)) (2)Cl-2, AsCl(NPMe(3))(2)SbCl4 SbCl6, and AsCl(NPMe(3))(2)SnCl4 center dot CH3CN	1996
4287	E. Matsumoto	Determination of Inorganic Arsenic in Fish Oil and Fish Oil Capsules by LC-ICP-MS	2021
4288	H. M. Andrade	Arsenic toxicity: cell signalling and the attenuating effect of nitric oxide in Eichhornia crassipes	2016
4289	V. M. Mikoushkin	Elemental arsenic in the natural oxide on the MBE GaAs surface	2020
4290	S. Heinl	(Cp2As4)-As-PEt-An Organic-Substituted As-4 Butterfly Compound	2016
4291	K. Strey	Gesche Gottfried's Mouse Butter	
4292	Q. H. Guo	Effective treatment of arsenic-bearing water by a layered double metal hydroxide: Iowaite	2017
4293	C. M. Donahue	Synthesis, characterization and structural comparisons of phosphonium and arsenic dithiocarbamates with alkyl and phenyl substituents	2014
4294	S. Nishida	Enhanced arsenic sensitivity with excess phytochelatin accumulation in shoots of a SULTR1;2 knockout mutant of Arabidopsis thaliana (L.) Heynh	2016
4295	G. Bia	Arsenic-bearing phases in South Andean volcanic ashes: Implications for As mobility in aquatic environments	2015
4296	L. Peng	2-nitrobenzylarsonium compounds that photorelease heavy-atom cholinergic ligands for time-resolved crystallographic studies on cholinesterases	1998
4297	B. P. Mohanty	Curcumin Has Protective Effect on the Eye Lens Against Arsenic Toxicity	2021
4298	F. Heidari	Fumaric acids as a novel antagonist of TLR-4 pathway mitigates arsenic-exposed inflammation in human monocyte-derived dendritic cells	2019
4299	W. Sheng	LEACHING OF HIGH ARSENIC CONTENT DUST AND A NEW PROCESS FOR THE PREPARATION OF COPPER ARSENATE	2018
4300	B. L. Bearson	The Role of Salmonella Genomic Island 4 in Metal Tolerance of Salmonella enterica Serovar I 4, 5 ,12:i:- Pork Outbreak Isolate USDA15WA-1	2020

4301	X. D. Xie	Permanganate oxidation and ferric ion precipitation (KMnO4-Fe(III)) process for treating phenylarsenic compounds	2019
4302	H. P. S. Chauhan	Mixed arsenic(III) bis(dimethyldithiocarbamato) derivatives with some oxygen and sulfur donor ligands	2019
4303	A. Wang	Reductive removal of arsenic from waste acid containing high-acidity and arsenic levels through iodide and copper powder synergy	2019
4304	P. R. Guvvala	Protective role of epigallocatechin-3-gallate on arsenic induced testicular toxicity in Swiss albino mice	2017
4305	T. P. Joshi	Transformation of para arsanilic acid by manganese oxide: Adsorption, oxidation, and influencing factors	2017
4306	D. Z. Yang	Reclamation of a waste arsenic-bearing gypsum as a soil conditioner via acid treatment and subsequent Fe(II)-As stabilization	2019
4307	E. Bustaffa	Genotoxic and epigenetic mechanisms in arsenic carcinogenicity	2014
4308	L. H. Fan	Dual oligopeptides modification mediates arsenic trioxide containing nanoparticles to eliminate primitive chronic myeloid leukemia cells inside bone marrow niches	2020
4309	Z. R. Jiang	Decorating S-doped Cu-La bimetallic oxides with UIO-66 to increase the As (III) adsorption capacity via synchronous oxidation and adsorption	2021
4310	M. S. Rahaman	Effects of curcumin, D-pinitol alone or in combination in cytotoxicity induced by arsenic in PC12 cells	2020
4311	J. Zhou	Lipidomic profiling of subchronic As4S4 exposure identifies inflammatory mediators as sensitive biomarkers in rats	2019
4312	A. Yanitch	Transcriptomic Response of Purple Willow (Salix purpurea) to Arsenic Stress	2017
4313	S. K. Shukla	Synthesis and characterization of some new triorganophosphorus, -arsenic and -antimony(V) amido derivatives	2003
4314	T. A. Shaikh	Synthesis and characterization of a rare arsenic trithiolate with an organic disulfide linkage and 2-chloro-benzo-1,3,2-dithiastibole	2006
4315	T. C. Mohan	Cytokinin Determines Thiol-Mediated Arsenic Tolerance and Accumulation	2016
4316	S. J. Kim	Complete genome of Halomonas aestuarii Hb3, isolated from tidal flat	2018
4317	A. Popowich	Arsenobetaine: the ongoing mystery	2016
4318	Q. Z. Zhu	Study on the Clinical Safe and Effective Methods of Arsenic-Containing Compound-Qinghuang Powder in the Treatment of Myelodysplastic Syndrome	2017

4319	M. C. Ruiz	Selective arsenic removal from enargite by alkaline digestion and water leaching	2014
4320	P. R. Agrawal	The removal of pentavalent arsenic by graphite intercalation compound functionalized carbon foam from contaminated water	2019
4321	M. Sonksen	Current status and future prospects of nanomedicine for arsenic trioxide delivery to solid tumors	2022
4322	M. U. Ijaz	Evaluation of possible protective role of Chrysin against arsenic-induced nephrotoxicity in rats	
4323	G. L. Yu	Extraction of arsenic from arsenic-containing cobalt and nickel slag and preparation of arsenic-bearing compounds	2014
4324	Z. Zhu	Structural Transition in Layered As1-xPx Compounds: A Computational Study	2015
4325	Z. Magdziak	Profile and concentration of the low molecular weight organic acids and phenolic compounds created by two-year-old Acer platanoides seedlings growing under different As forms	2020
4326	M. Scheer	Complexes containing phosphorus and arsenic as terminal ligands	1996
4327	R. A. Glabonjat	Arsenolipids in Cultured Picocystis Strain ML and Their Occurrence in Biota and Sediment from Mono Lake, California	2020
4328	D. Halter	Arsenic hypertolerance in the protist Euglena mutabilis is mediated by specific transporters and functional integrity maintenance mechanisms	2015
4329	K. Nan	Arsenic speciation in tree moss by mass spectrometry based hyphenated techniques	2018
4330	D. Piacentini	Nitric Oxide Cooperates With Auxin to Mitigate the Alterations in the Root System Caused by Cadmium and Arsenic	2020
4331	W. Cai	Potential, risks, and benefits of the extract recycled from Pteris vittata arsenic-rich biomass as a broiler growth promoter	2022
4332	G. Bjorklund	Developmental toxicity of arsenic: a drift from the classical dose-response relationship	2020
4333	Y. Zhang	Removal of Gas-Phase As2O3 by Metal Oxide Adsorbents: Effects of Experimental Conditions and Evaluation of Adsorption Mechanism	2015
4334	A. Aguilar-Garrido	Carbonated waste valorisation from a peat bog exploitation in the treatment of arsenic-polluted waters	2022
4335	P. Dabrowski	A case of syphilis with high bone arsenic concentration from early modern cemetery (Wroclaw, Poland)	2019
4336	C. Chmielowska	Benzalkonium chloride and heavy metal resistance profiles of Listeria monocytogenes strains isolated from fish, fish products and food-producing factories in Poland	2021

4337	E. McGrory	Occurrence, Geochemistry and Speciation of Elevated Arsenic Concentrations in a Fractured Bedrock Aquifer System	2021
4338	G. A. Leao	Phytoremediation of arsenic-contaminated water: the role of antioxidant metabolism of Azolla caroliniana Willd. (Salviniales)	2017
4339	V. A. Plyuta	Effects of Volatile Organic Compounds Synthesized by Bacteria on the Expression from Promoters of the zntA, copA, and arsR Genes Induced in Response to Copper, Zinc, and Arsenic	2020
4340	Y. B. Luo	Synthetic arsenic sulfides in Japanese prints of the Meiji period	2016
4341	N. N. Phan	The novel regulations of MEF2A, CAMKK2, CALM3, and TNNI3 in ventricular hypertrophy induced by arsenic exposure in rats	2014
4342	H. Y. Peng	Liquid chromatography combined with atomic and molecular mass spectrometry for speciation of arsenic in chicken liver	2014
4343	P. Khownpurk	Removal of As(III) from aqueous solution by the oyster shell powder-treated rice husk ash composite (OS-TRHA) pellet	2019
4344	C. M. Carmean	Braving the Element: Pancreatic beta-Cell Dysfunction and Adaptation in Response to Arsenic Exposure	2019
4345	S. Wongrod	Assessing arsenic redox state evolution in solution and solid phase during As (III) sorption onto chemically-treated sewage sludge digestate biochars	2019
4346	I. A. Mekkawy	Significance Assessment of Amphora coffeaeformis in Arsenic-Induced Hemato-Biochemical Alterations of African Catfish (Clarias gariepinus)	2020
4347	J. Borovicka	Resurrection of Cortinarius coalescens: taxonomy, chemistry, and ecology	2017
4348	G. J. Ahammed	Anthocyanin-mediated arsenic tolerance in plants	2022
4349	R. Recio-Vega	MRP1 expression in bronchoalveolar lavage cells in subjects with lung cancer who were chronically exposed to arsenic	2015
4350	S. Palei	Oxidation behavior with quantum dots formation from amorphous GaAs thin films	2018
4351	K. L. Liu	Prenatal heavy metal exposure, total immunoglobulin E, trajectory, and atopic diseases: A 15-year follow-up study of a Taiwanese birth cohort	2021
4352	M. Herberhold	BICYCLIC ARSINO SULFUR DIIMIDES	1992
4353	V. A. Bybin	Influence of Heavy Metals and Arsenic on Survival and Biofilm Formation of Some Saprotrophic Soil Microorganisms	2021
4354	C. H. Jeong	Arsenic downregulates tight junction claudin proteins through p38 and NF-kappa B in intestinal epithelial cell line, HT-29	2017

4355	G. J. Farias	Chemical properties and protective effect of Rosmarinus officinalis: mitigation of lipid peroxidation and DNA-damage from arsenic exposure	2018
4356	Y. T. Kao	Arsenic treatment increase Aurora-A overexpression through E2F1 activation in bladder cells	2017
4357	B. Ringler	Building Chains - Three Different Pnictogen Atoms in an Entirely tert-Butyl Substituted Molecule	2018
4358	N. Nurnaeimah	The Effects of Hydrogen Peroxide on Plant Growth, Mineral Accumulation, as Well as Biological and Chemical Properties of Ficus deltoidea	2020
4359	T. P. Robinson	On the Ambiphilic Reactivity of Geometrically Constrained Phosphorus(III) and Arsenic(III) Compounds: Insights into Their Interaction with Ionic Substrates	2016
4360	K. O. Amayo	Arsenolipids show different profiles in muscle tissues of four commercial fish species	2014
4361	F. L. F. da Silva	Non-chromatographic arsenic speciation analyses in wild shrimp (Farfantepenaeus brasiliensis) using functionalized magnetic iron-nanoparticles	2021
4362	L. V. Brumatti	Impact of Methylmercury and Other Heavy Metals Exposure on Neurocognitive Function in Children Aged 7 Years: Study Protocol of the Follow-up	2021
4363	P. B. Hitchcock	beta-Diiminato complexes of arsenic including the formally As-I compound As3L3 L = {N(C6H3Pr2i-2,6)C(H)}(2)CPh	2009
4364	F. Ehlers	Keto-stabilized Arsenic Ylides and their Coordination to Gold(I)	2020
4365	D. J. Thomas	Role of complex organic arsenicals in food in aggregate exposure to arsenic	2016
4366	M. A. Sanz	Management of acute promyelocytic leukemia: updated recommendations from an expert panel of the European LeukemiaNet	2019
4367	M. S. Rahaman	Curcumin alleviates arsenic-induced toxicity in PC12 cells via modulating autophagy/apoptosis	2020
4368	M. S. Taleshi	Arsenolipids in oil from blue whiting Micromesistius poutassou - evidence for arsenic-containing esters	2014
4369	F. Ebert	Cellular toxicological characterization of a thioxolated arsenic-containing hydrocarbon	2020
4370	D. M. Babanly	Thermodynamic functions of arsenic selenides	2017
4371	J. Kretzschmar	Kinetics and activation parameters of the reaction of organoarsenic(V) compounds with glutathione	2014
4372	H. P. Wang	Discovery and identification of arsenic removal products from molten steel by adding rare earth	2022

4373	T. Bartsch	Palladium pnictide oxides Nd10Pd3As8O10 and Sm10Pd3As8O10 - low temperature structural phase transition and physical properties	2016
4374	A. Lashgari	Morphological Investigation and Fractal Properties of Realgar Nanoparticles	2015
4375	T. Saal	Lewis adduct formation of hydrogen cyanide and nitriles with arsenic and antimony pentafluoride	2019
4376	M. M. Nearing	Uptake and transformation of arsenic during the reproductive life stage of Agaricus bisporus and Agaricus campestris	2016
4377	G. Baba	Functionalised 1-Alkynylarsines: Synthesis, Characterisation, and Attempts of Rearrangement into Functionalised Arsaalkynes	2014
4378	M. A. Jebelli	Isolation and identification of indigenous prokaryotic bacteria from arsenic-contaminated water resources and their impact on arsenic transformation	2017
4379	P. Marzenell	Aminoferrocene-Based Prodrugs and Their Effects on Human Normal and Cancer Cells as Well as Bacterial Cells	2013
4380	M. Khan	A method for determining arsenolipids in seawater by HPLC-high resolution mass spectrometry	2016
4381	S. Sharma	Nondestructive and Rapid Probing of Biochemical Response of Arsenic Stress on the Leaves of Wheat Seedlings Using Attenuated Total Reflectance Fourier Transform Infrared Spectroscopy	2019
4382	N. K. Mondal	Prevalence of Arsenic in chicken feed and its contamination pattern in different parts of chicken flesh: a market basket study	2020
4383	P. Bhowmick	Amorphous carbon nanotubes as potent sorbents for removal of a phenolic derivative compound and arsenic: theoretical support of experimental findings	2016
4384	A. Hartwig	Metals and their compounds as contaminants in food. Arsenic, cadmium, lead and aluminum	2017
4385	K. Deering	Monitoring of arsenic, mercury and organic pesticides in particulate matter, ambient air and settled dust in natural history collections taking the example of the Museum fur Naturkunde, Berlin	2019
4386	F. Ledderboge	High-pressure investigations of yttrium(III) oxoarsenate(V): Crystal structure and luminescence properties of Eu3+-doped scheelite-type Y AsO4 from xenotime-type precursors	2018
4387	A. H. Petursdottir	Environmental effects on arsenosugars and arsenolipids in Ectocarpus (Phaeophyta)	2016
4388	T. Huynh	Measurement of labile arsenic speciation in water and soil using diffusive gradients in thin films (DGT) and X-ray absorption near edge spectroscopy (XANES)	2015
4389	K. A. Karimov	Oxidation Sulfuric Acid Autoclave Leaching of Copper Smelting Production Fine Dust	2019
4390	Y. Madaule	ARSENIC COMPOUNDS IN ORGANIC-SYNTHESIS - PENTAMETHINIUM SALTS FROM AMINOARSANES AND PYRYLIUM-SALTS	1991

4391	J. K. Frediani	Arsenic exposure and risk of nonalcoholic fatty liver disease (NAFLD) among US adolescents and adults: an association modified by race/ethnicity, NHANES 2005-2014	2018
4392	H. Yamamichi	Synthesis and Characterization of Monohaloalkoxyarsoranes Bearing a Novel Tridentate Ligand Occupying One Apical and Two Equatorial Sites	2010
4393	Y. Zhou	Efficient removal of roxarsone and emerging organic contaminants by a solar light-driven in-situ Fenton system	2022
4394	T. A. Shaikh	Structural characteristics of 2-halo-1,3,2-dithiarsenic compounds and tris-(pentafluorophenylthio)-arsen	2006
4395	Y. Y. Hou	2D black arsenic phosphorus and its application for anodes of lithium ion batteries	2020
4396	D. V. Ladonin	Heavy Metals and Arsenic in Soils and Street Dust of the Southeastern Administrative District of Moscow: Long-Term Data	2020
4397	F. L. Li	Impact of natural organic matter on arsenic removal by modified granular natural siderite: Evidence of ternary complex formation by HPSEC-UV-ICP-MS	2017
4398	J. Alchouron	Household arsenic contaminated water treatment employing iron oxide/ bamboo biochar composite: An approach to technology transfer	2021
4399	J. C. Fei	Aromatic organoarsenic compounds (ADCs) occurrence and remediation methods	2018
4400	C. Marquardt	Anionic Chains of Parent Pnictogenylboranes	2016
4401	S. N. Chen	Simultaneous removal of para-arsanilic acid and the released inorganic arsenic species by CuFe2O4 activated peroxymonosulfate process	2020
4402	Y. S. Yu	Identification of a MarR Subfamily That Regulates Arsenic Resistance Genes	2021
4403	G. Hierlmeier	Synthesis and Reactivity of Nickel-Stabilised mu(2):eta(2),eta(2)-P-2, As-2 and PAs Units	2018
4404	S. K. Shukla	Some reactions and spectroscopic studies of tris(pentafluorophenyl)arsenic and tris(pentafluorophenyl)-antimony(III and V) derivatives	2003
4405	T. P. Joshi	Adsorption of aromatic organoarsenic compounds by ferric and manganese binary oxide and description of the associated mechanism	2017
4406	H. Y. Xie	Fate and Risk Assessment of Arsenic Compounds in Soil Amended with Poultry Litter Under Aerobic and Anaerobic Circumstances	2015
4407	K. Kulik-Kupka	Arsenic - poison or medicine?	2016
4408	P. De Francisco	Interactions with Arsenic: Mechanisms of Toxicity and Cellular Resistance in Eukaryotic Microorganisms	2021

4409	E. R. Pereira	Arsenic containing medium and long chain fatty acids in marine fish oil identified as degradation products using reversed-phase HPLC-ICP-MS/ESI-MS	2016
4410	R. Kuschel	Structures of some organoarsenic fluorides	1993
4411	X. Shi	Geospatial association between adverse birth outcomes and arsenic in groundwater in New Hampshire, USA	2015
4412	Y. Zheng	At the crossroads: Hazard assessment and reduction of health risks from arsenic in private well waters of the northeastern United States and Atlantic Canada	2015
4413	S. M. Shaheen	Redox effects on release kinetics of arsenic, cadmium, cobalt, and vanadium in Wax Lake Deltaic freshwater marsh soils	2016
4414	T. F. Liang	Degradation of roxarsone in a silt loam soil and its toxicity assessment	2014
4415	R. C. J. Campbell	Controls on the Valence Species of Arsenic in Tobacco Smoke: XANES Investigation with Implications for Health and Regulation	2014
4416	A. M. Jalaludeen	Biochanin A Ameliorates Arsenic-Induced Hepatoand Hematotoxicity in Rats	2016
4417	P. K. Krishnakumar	Arsenic and arsenic species in shellfish and finfish from the western Arabian Gulf and consumer health risk assessment	2016
4418	C. E. Barragan	RNA transcript response by an Acidithiobacillus spp. mixed culture reveals adaptations to growth on arsenopyrite	2021
4419	A. Poznyak	Peculiar Porous Aluminum Oxide Films Produced via Electrochemical Anodizing in Malonic Acid Solution with Arsenazo-I Additive	2021
4420	D. M. C. Ould	Diazaphospholene and Diazaarsolene Derived Homogeneous Catalysis	2020
4421	K. L. Cooper	Contribution of NADPH oxidase to the retention of UVR-induced DNA damage by arsenic	2022
4422	Y. A. Sarinana-Ruiz	Assessment of arsenic and fluorine in surface soil to determine environmental and health risk factors in the Comarca Lagunera, Mexico	2017
4423	K. Kunkel	A mass spectrometric and quantum chemical study of the vaporisation of lead monoxide in a flow of gaseous arsenic and antimony trioxides	2014
4424	Q. Y. Huang	Seminal plasma metabolites mediate the associations of multiple environmental pollutants with semen quality in Chinese men	2019
4425	Z. W. Zhao	Microbial mobilization of arsenic from iron-bearing clay mineral through iron, arsenate, and simultaneous iron-arsenate reduction pathways	2021
4426	Y. C. Lv	Removal of p-arsanilic acid by an amino-functionalized indium-based metal-organic framework: Adsorption behavior and synergetic mechanism	2018

4427	Z. Bujnakova	Arsenic sulfide nanoparticles prepared by milling: properties, free-volume characterization, and anti-cancer effects	2015
4428	S. Chen	Arsenic Trioxide Rescues Structural p53 Mutations through a Cryptic Allosteric Site	2021
4429	H. P. S. Chauhan	Mixed dithiolato alkylene dithiophosphate derivatives of arsenic(III) and antimony(III)	2000
4430	R. Balint	Organic phosphorus affects the retention of arsenite and arsenate by goethite	2020
4431	M. Ranjan	A review of bismuth-based sorptive materials for the removal of major contaminants from drinking water	2020
4432	R. Singh	Interplay of Calcium and Nitric Oxide in improvement of Growth and Arsenic-induced Toxicity in Mustard Seedlings	2020
4433	L. Weber	2-Phospha- and 2-Arsaethynolates - Versatile Building Blocks in Modern Synthetic Chemistry	2018
4434	E. V. Polyakova	Capillary zone electrophoresis as a simple approach for the study of p-arsanilic acid transformation in the process of photolytic degradation	2021
4435	H. Ramirez-Aldaba	Chemical and surface analysis during evolution of arsenopyrite oxidation by Acidithiobacillus thiooxidans in the presence and absence of supplementary arsenic	2016
4436	P. A. Gunka	Raman studies of hydrogen trapped in As4O6 center dot 2H(2) at high pressure and low temperature	2020
4437	J. Y. Xing	A comprehensive exploration about the effects of O-2, SO2 and NO on As2O3 adsorption over Cu/gamma-Al2O3 SCR catalyst: A DFT study	2022
4438	Y. O. Santiago-Saenz	Effect of a Supplementation with Two Quelites on Urinary Excretion of Arsenic in Adolescents Exposed to Water Contaminated with the Metalloid in a Community in the State of Guanajuato, Mexico	2020
4439	R. T. Bray	Application of PIX 112 to Arsenic Removal from Groundwater by Surface Coagulation in the Quartz Sand-Pyrolusite Filter Media	2016
4440	R. M. Potdukhe	Root transcripts associated with arsenic accumulation in hyperaccumulator Pteris vittata	2018
4441	S. M. Nunes	Biochemical responses induced by co-exposition to arsenic and titanium dioxide nanoparticles in the estuarine polychaete Laeonereis acuta	2017
4442	A. Weitze	Polysulfonylamines: part LXXIX. Synthesis of arsenic(III) and antimony(III) dimesylamides and crystal structures of As N(SO2Me)(2) Cl-2 and MeAs N(SO2Me)(2) Cl	1997
4443	C. X. Liu	Quantitative Determination of Arsenic Species in Feed Using Liquid Chromatography-Hydride Generation Atomic Fluorescence Spectrometry	2018
4444	C. W. Tsai	Dithiothreitol Enhanced Arsenic-Trioxide-Induced Cell Apoptosis in Cultured Oral Cancer Cells via Mitochondrial Dysfunction and Endoplasmic Reticulum Stress	2017

4445	Y. S. Yu	As(III) Exposure Induces a Zinc Scarcity Response and Restricts Iron Uptake in High-Level Arsenic-Resistant Paenibacillus taichungensis Strain NC1	2022
4446	R. Deka	Carbene chemistry of arsenic, antimony, and bismuth: origin, evolution and future prospects	2022
4447	R. Garla	Quantum mechanical treatment of As3+-thiol model compounds: implication for the core structure of As(III)-metallothionein	2017
4448	M. Choi	Quantitative depth profile analysis of InP/InGaAs hetero-interfaces by as carry-over	2020
4449	L. Hibbard	Case Studies for General Chemistry: Teaching with a Newsworthy Story	2019
4450	H. Chen	Amidoxime-functionalized covalent organic framework as simultaneous luminescent sensor and adsorbent for organic arsenic from water	2022
4451	X. Shi	Metabolomic Analysis of the Effects of Chronic Arsenic Exposure in a Mouse Model of Diet-Induced Fatty Liver Disease	2014
4452	J. Castro-Severyn	Genomic Variation and Arsenic Tolerance Emerged as Niche Specific Adaptations by DifferentExiguobacteriumStrains Isolated From the Extreme Salar de Huasco Environment in Chilean - Altiplano	2020
4453	E. Kociolek-Balawejde	Hybrid polymers containing brochantite/tenorite obtained using gel type anion exchanger	2018
4454	T. T. Han	Speciation analysis of arsenic compounds in seafood by ion chromatography-atomic fluorescence spectrometry	2017
4455	F. Lehnfeld	Synthesis and Reactivity of a Lewis-Base-Stabilized tert-Butyl Arsanylborane: A Versatile Building Block for Arsenic-Boron Oligomers	2022
4456	P. Zhao	Association of Gene Mutations with Response to Arsenic-Containing Compound Qinghuang Powder in Patients with Myelodysplastic Syndromes	2019
4457	X. D. Jiang	Formation of epoxides from pentacoordinated organoarsenic compounds with a beta-hydroxyethyl group	2010
4458	G. N. George	Observation of the seleno bis-(S-glutathionyl) arsinium anion in rat bile	2016
4459	S. M. Reichman	Probing the plant growth-promoting and heavy metal tolerance characteristics of Bradyrhizobium japonicum CB1809	2014
4460	S. Vinoth	Surface engineering of roselike lanthanum molybdate electrocatalyst modified screen-printed carbon electrode for robust and highly sensitive sensing of antibiotic drug	2021
4461	D. M. C. Ould	Arsenic Catalysis: Hydroboration of Aldehydes Using aBenzo-Fused Diaza-benzyloxy-arsole	2018
4462	W. Cai	Effective strategy to recycle arsenic-accumulated biomass of Pteris vittata with high benefits	2021

4463	S. Maheshwari	Synthesis and spectroscopic characterization of tris(O,O '-ditolyl dithiophosphato) arsenic/antimony/bismuth(III) compounds: Crystal structures of As{S2P(OC6H4Me-m)(2)}(3) center dot 0.5C(6)H(14), Sb{S2P(OC6H4Me-m)(2)}(3) and Bi{S2P(OC6H4Me-	2009
4464	C. Kruppa	A new route to iminoarsanes	2000
4465	H. Imoto	Development of macromolecules and supramolecules based on silicon and arsenic chemistries	2018
4466	J. Werner	Recent trends in microextraction techniques used in determination of arsenic species	2018
4467	A. E. Galvan	Identification of the Biosynthetic Gene Cluster for the Organoarsenical Antibiotic Arsinothricin	2021
4468	Y. H. Jiao	Rat Liver Mitochondrial Dysfunction Induced by an Organic Arsenical Compound 4-(2-Nitrobenzaliminyl) Phenyl Arsenoxide	2015
4469	Z. Yang	Structural and electronic properties of the V-V compounds isoelectronic to GaN and isostructural to gray arsenic	2018
4470	A. Sharma	ESTIMATION OF ARSENIC(III) IN ORGANIC ARSINES AND ITS COMPLEXES USING POTASSIUM BROMATE AND POTASSIUM IODATE AS OXIDANTS	2016
4471	S. Gonzilez-Montiel	Synthesis, characterisation and properties of As-monohalogenated dibenzoarsocines S(C6H4S)(2)AsHal (Hal = Cl, Br, I) - A study of the transannular interaction S -> As	2006
4472	N. Das	Expression of rice MATE family transporter OsMATE2 modulates arsenic accumulation in tobacco and rice	2018
4473	G. Soultani	Elements of toxicological concern and the arsenolipids' profile in the giant-red Mediterranean shrimp, Aristaeomorpha foliacea	2021
4474	G. Cassone	Stability of hydrolytic arsenic species in aqueous solutions: As3+ vs. As5+	2018
4475	V. Taylor	Human exposure to organic arsenic species from seafood	2017
4476	C. K. Chen	Specifically designed magnetic biochar from waste wood for arsenic removal	2021
4477	M. Stiboller	Quantifying Inorganic Arsenic and Other Water-Soluble Arsenic Species in Human Milk by HPLC/ICPMS	2017
4478	X. Wu	Comparative health risk assessment of realgar and NiuHuangJieDu tablets based on tissue arsenic levels after multiple oral administration to rats	2020
4479	C. Clobes	Stepwise Synthesis of Siloxane-Substituted Oligoarsanes and Structural Investigation of Alkaline Earth Metal Derivatives	2015
4480	J. S. Edmonds	Diastereoisomerism of thiol complexes of arsenic acids and pseudoasymmetry of arsenic: a H-1 and C-13 NMR study	2006

4481	D. Pang	Superior removal of inorganic and organic arsenic pollutants from water with MIL-88A(Fe) decorated on cotton fibers	2020
4482	S. Stice	Determination of multiple human arsenic metabolites employing high performance liquid chromatography inductively coupled plasma mass spectrometry	2016
4483	S. Mondal	Morin encapsulated chitosan nanoparticles (MCNPs) ameliorate arsenic induced liver damage through improvement of the antioxidant system and prevention of apoptosis and inflammation in mice	2022
4484	E. P. Jahrman	Assessing arsenic species in foods using regularized linear regression of the arsenic K-edge X-ray absorption near edge structure	2022
4485	R. J. Cheng	Mechanism research on arsenic removal from arsenopyrite ore during a sintering process	2017
4486	L. J. Taylor	Hydride Abstraction and Deprotonation - an Efficient Route to Low Coordinate Phosphorus and Arsenic Species	2016
4487	F. L. Fu	The use of zero-valent iron for groundwater remediation and wastewater treatment: A review	2014
4488	L. Ruzik	Speciation of Arsenic(III) and Arsenic(V) in Plant-Based Drinks	2022
4489	E. Allevato	Mechanisms of arsenic assimilation by plants and countermeasures to attenuate its accumulation in crops other than rice	2019
4490	A. Tariq	Comparative analysis of the Magnesium Ferrite (MgFe2O4) nanoparticles synthesised by three different routes	2019
4491	J. K. Saunders	Complete arsenic-based respiratory cycle in the marine microbial communities of pelagic oxygen-deficient zones	2019
4492	C. Liang	Tetrandrine and arsenic trioxide synergistically inhibit proliferation of HCC1937 triple negative breast cancer cells	2017
4493	J. Chen	Organoarsenical compounds: Occurrence, toxicology and biotransformation	2020
4494	S. X. Qiu	Simultaneous removal of arsenic and antimony from mining wastewater using granular TiO2: Batch and field column studies	2019
4495	H. Y. Guo	First Organic-Inorganic Hybrid Compounds Formed by Ge-V-O Clusters and Transition Metal Complexes of Aromatic Organic Ligands	2022
4496	N. K. Maier	Arsenic Trioxide and Other Arsenical Compounds Inhibit the NLRP1, NLRP3, and NAIP5/NLRC4 Inflammasomes	2014
4497	M. Sijko	Influence of Dietary Compounds on Arsenic Metabolism and Toxicity. Part II-Human Studies	2021
4498	J. Hara	Characteristics of arsenic in humic substances extracted from natural organic sediments	2018

4499	A. Hinz	A Monoanionic Arsenide Source: Decarbonylation of the 2-Arsaethynolate Anion upon Reaction with Bulky Stannylenes	2016
4500	J. Tremlova	Distribution of arsenic compounds in Plantaginaceae and Cyperaceae plants growing in contaminated soil	2016
4501	Z. Y. Yu	ETME, a novel beta-elemene derivative, synergizes with arsenic trioxide in inducing apoptosis and cell cycle arrest in hepatocarcinoma cells via a p53-dependent pathway	2014
4502	T. Hu	Two-dimensional As1-XPX binary compounds: Highly tunable electronic structure and optical properties	2017
4503	S. K. Gupta	Transfer of arsenic from poultry feed to poultry litter: A mass balance study	2018
4504	J. Kwasny	Use of manometric zirconia for removing arsenic compounds from water	2016
4505	Y. Yang	Low-density solvent-based dispersive liquid-liquid microextraction followed by HPLC-ICP-MS for speciation analysis of phenylarsenics in lake water	2019
4506	H. Niemikoski	Studying the metabolism of toxic chemical warfare agent-related phenylarsenic chemicals in vitro in cod liver	2020
4507	M. Kato	Multidisciplinary approach to assess the toxicities of arsenic and barium in drinking water	2020
4508	R. Inaba	Systematic Study on the Catalytic Arsa-Wittig Reaction	2020
4509	J. Mourao	Tolerance to arsenic contaminant among multidrug-resistant and copper-tolerant Salmonella successful clones is associated with diverse ars operons and genetic contexts	2020
4510	M. A. Sabur	Surface interactions of monomethylarsonic acid with hematite nanoparticles studied using ATR-FTIR: adsorption and desorption kinetics	2015
4511	X. K. Tian	Design and synthesis of a molecule with aggregation-induced emission effects and its application in the detection of arsenite in groundwater	2017
4512	X. M. Xue	The enigma of environmental organoarsenicals	
4513	D. Dobrzynski	Hydrogeochemical and biomedical insights into germanium potential of curative waters: a case study of health resorts in the Sudetes Mountains (Poland)	2018
4514	M. Pumera	2D Monoelemental Arsenene, Antimonene, and Bismuthene: Beyond Black Phosphorus	2017
4515	B. M. Gardner	Triamidoamine uranium(IV)-arsenic complexes containing one-, two- and threefold U-As bonding interactions	2015
4516	M. R. Perry	Arsenic Exposure and Outcomes of Antimonial Treatment in Visceral Leishmaniasis Patients in Bihar, India: A Retrospective Cohort Study	2015

4517	S. H. Nam	Feasibility of Separation and Quantification of Inorganic Arsenic Species Using Ion-Exchange Membranes and Laser-Induced Breakdown Spectroscopy	2018
4518	B. L. Rivas	Water-Soluble and Insoluble Polymers, Nanoparticles, Nanocomposites and Hybrids With Ability to Remove Hazardous Inorganic Pollutants in Water	2018
4519	J. H. Redmon	Is Food Irrigated with Oilfield-Produced Water in the California Central Valley Safe to Eat? A Probabilistic Human Health Risk Assessment Evaluating Trace Metals Exposure	2021
4520	D. D. Han	Curcumin Synergistically Enhances the Cytotoxicity of Arsenic Trioxide in U266 Cells by Increasing Arsenic Uptake	2021
4521	R. M. Matos	New synthetic route and characterisation of phosphorus and arsenic heterocyclic compounds	2002
4522	K. O. Amayo	Identification of arsenolipids and their degradation products in cod-liver oil	2014
4523	G. Kerr	Arsenic residues from historic gold extraction, Snowy River, Westland, New Zealand	2021
4524	W. H. Zhang	INAA with anticoincidence counting significantly reduces interferences from the 554.3-keV photopeak of Br-82 to allow reliable measurements of nanogram levels of arsenic in solid biological materials via the 559.1-keV photopeak of As-76	2018
4525	V. J. Thomas	Application of Graphene and Graphene Compounds for Environmental Remediation	2016
4526	E. P. Wildman	Triamidoamine thorium-arsenic complexes with parent arsenide, arsinidiide and arsenido structural motifs	2017
4527	T. Foyzun	Polyphenolics with Strong Antioxidant Activity from Acacia nilotica Ameliorate Some Biochemical Signs of Arsenic-Induced Neurotoxicity and Oxidative Stress in Mice	2022
4528	S. S. D. Elanchezhiyan	Lanthanum-substituted bimetallic magnetic materials assembled carboxylate-rich graphene oxide nanohybrids as highly efficient adsorbent for perfluorooctanoic acid adsorption from aqueous solutions	2020
4529	F. R. Chen	Determination of Arsenic Speciation in Scomberomorus Niphonius by Capillary Electrophoresis-Inductively Coupled Plasma Mass Spectrometry	2014
4530	Y. L. Yu	Arsenosugar standards extracted from algae: Isolation, characterization and use for identification and quantification purposes	2020
4531	R. Jalilian	Ultrasonic-assisted micro solid phase extraction of arsenic on a new ion-imprinted polymer synthesized from chitosan-stabilized pickering emulsion in water, rice and vegetable samples	2020
4532	R. A. Glabonjat	Arsenolipid biosynthesis by the unicellular alga Dunaliella tertiolecta is influenced by As/P ratio in culture experiments	2018
4533	M. J. Kim	Arsenic hexoxide enhances TNF-alpha-induced anticancer effects by inhibiting NF-kappa B activity at a safe dose in MCF-7 human breast cancer cells	2014
4534	A. Hille-Rehfeld	BIOANORGANIC CHEMISTRY Variety of organic Arsenic Compounds	2016

4535	S. Gushgari-Doyle	Effects of Arsenic on Trichloroethene-Dechlorination Activities of Dehalococcoides mccartyi 195	2020
4536	J. H. Qin	Fenton reagent reduces the level of arsenic in paddy rice grain	2017
4537	A. E. Seitz	Facile storage and release of white phosphorus and yellow arsenic	2018
4538	J. H. T. Luong	Recent advances in electrochemical detection of arsenic in drinking and ground waters	2014
4539	P. Yox	Unprecedented superstructure in the type I family of clathrates	2021
4540	M. B. Steffensen	Single-molecule analysis of chirality in a multicomponent reaction network	2014
4541	S. Y. Lee	Enhanced Arsenic (III and V) Removal in Anoxic Environments by Hierarchically Structured Citrate/FeCO3 Nanocomposites	2020
4542	L. Gatti	Improved Apoptotic Cell Death in Drug-Resistant Non-Small-Cell Lung Cancer Cells by Tumor Necrosis Factor-Related Apoptosis-Inducing Ligand-Based Treatments	2014
4543	J. D. Hong	Fabrication of Al2O3/CaO with anti-sintering for efficient removal of As2O3 in simulated flue gas: Experimental and DFT study	2022
4544	Y. Kazui	Rapid and robust speciation/quantitative analysis of arsenous acid and related metabolites in serum by liquid chromatography-inductively coupled plasma-tandem mass spectrometry	2019
4545	V. Mesa	Use of Endophytic and Rhizosphere Bacteria To Improve Phytoremediation of Arsenic-Contaminated Industrial Soils by Autochthonous Betula celtiberica	2017
4546	P. Stevant	Biomass soaking treatments to reduce potentially undesirable compounds in the edible seaweeds sugar kelp (Saccharina latissima) and winged kelp (Alaria esculenta) and health risk estimation for human consumption	2018
4547	N. Burford	Identification of new N-Sb topologies: understanding the sequential dehydrochloride coupling of primary amines and trichloropnictines	2005
4548	Z. F. Wang	Supercritical fluid extraction and gas chromatography analysis of arsenic species from solid matrices	2016
4549	M. Fernandez	Paralogous Regulators ArsR1 and ArsR2 of Pseudomonas putida KT2440 as a Basis for Arsenic Biosensor Development	2016
4550	G. L. Diamond	Evaluating the mouse model for estimation of arsenic bioavailability: Comparison of estimates of absolute bioavailability of inorganic arsenic in mouse, humans, and other species	2022
4551	J. A. Lopez-Carvallo	Highly diluted bioactive compounds in marine aquaculture: A potential alternative for sustainable production	2022
4552	J. Y. Xing	Mechanism of the arsenic adsorption over Cu/?-Al2O3 SCR catalyst: An experimental combined theoretical analysis	2022

4553	J. L. Zhou	The Arene-Stabilized eta(5)-Pentamethylcyclopentadienyl Arsenic Dication (eta(5)-Cp*)As(toluene) (2+)	2019
4554	J. Chen	The Pseudomonas putida NfnB nitroreductase confers resistance to roxarsone	2020
4555	C. Wei	Acute promyelocytic leukemia in a twin pregnancy	2016
4556	S. Braeuer	Arsenocholine-O-sulfate: A novel compound as major arsenic species in the parasitic mushroom Tolypocladium ophioglossoides	2021
4557	M. Fritz	Small Chains of Main Group Elements by BH3 Adduct Formation of tBu(2)E-N(H)-EtBu2 (E = P, As)	2020
4558	K. C. K. Swamy	Synthesis, reactivity and structures of cyclic arsenites with an N -> AS bond	1999
4559	C. Tan	Separation of arsenic and antimony from dust with high content of arsenic by a selective sulfidation roasting process using sulfur	2018
4560	M. Rodriguez-Ruiz	Arsenate disrupts ion balance, sulfur and nitric oxide metabolisms in roots and leaves of pea (Pisum sativum L.) plants	2019
4561	J. Camargo	Toenail concentrations of trace elements and occupational history in pancreatic cancer	2019
4562	J. W. Jun	Effect of Central Metal Ions of Analogous Metal-Organic Frameworks on Adsorption of Organoarsenic Compounds from Water: Plausible Mechanism of Adsorption and Water Purification	2015
4563	T. Hong	Selective recovery of Rhenium from industrial leach solutions by synergistic solvent extraction	2020
4564	S. Mohammadi	Contamination of breast milk with lead, mercury, arsenic, and cadmium in Iran: a systematic review and meta-analysis	2022
4565	L. Y. Du	Influence of Arsenic Stress on Physiological, Biochemical, and Morphological Characteristics in Seedlings of Two Cultivars of Maize (Zea mays L.)	2017
4566	K. Huang	Biotransformation of arsenic-containing roxarsone by an aerobic soil bacterium Enterobacter sp. CZ-1	2019
4567	C. Gurnari	When Poisons Cure: The Case of Arsenic in Acute Promyelocytic Leukemia	2020
4568	A. Sujak	Mallards Anas platyrhynchos shot in Eastern Poland: ecological risk evaluated by analysis of trace elements in liver	2019
4569	D. P. Zhong	Separation of Arsenic from the Antimony-Bearing Dust through Selective Oxidation Using CuO	2017
4570	M. Kapitein	NHC-stabilized silylphosphino- and silylarsinogallanes	2016

4571	Z. W. Zhao	Anaerobic oxidation of arsenite by bioreduced nontronite	2021
4572	M. Westerhausen	Homoleptic triisopropylsilylarsandiides of magnesium and divalent tin	2000
4573	S. Radic	Toxicological and chemical assessment of arsenic-contaminated groundwater after electrochemical and advanced oxidation treatments	2016
4574	K. P. Mangalgiri	Organoarsenicals in poultry litter: Detection, fate, and toxicity	2015
4575	A. Harchani	The new heteropolyoxometalate compound (C6H8N)(5) HAs2Mo6O26 (H2O) center dot 3H(2)O: crystal structure and Hirshfeld surface analysis	2018
4576	J. Almeida	Electrodialytic removal of tungsten and arsenic from secondary mine resources - Deep eutectic solvents enhancement	2020
4577	L. Belter	Polycyclic amides with As and Sb	2016
4578	X. J. Li	Heavy metal exposure causes changes in the metabolic health-associated gut microbiome and metabolites	2019
4579	Y. V. Bakhtiyarova	Crystal structure of new carboxylate phosphabetaines and phosphonium salts conjugated with them	2016
4580	Y. Wang	Hierarchically structured two-dimensional magnetic microporous biochar derived from hazelnut shell toward effective removal of p-arsanilic acid	2021
4581	P. Kubacek	Laser ablation synthesis of arsenic-phosphide AsmPn clusters from As-P mixtures. Laser desorption ionisation with quadrupole ion trap time-of-flight mass spectrometry: The mass spectrometer as a synthesizer	2018
4582	V. Gandin	Metal- and Semimetal-Containing Inhibitors of Thioredoxin Reductase as Anticancer Agents	2015
4583	D. Ben-Hail	Novel Compounds Targeting the Mitochondrial Protein VDAC1 Inhibit Apoptosis and Protect against Mitochondrial Dysfunction	2016
4584	H. Viitala	Hot Corrosion Mechanism of Steels Exposed to Heavy Metal Chlorides and Sulphates in SO2 Environment	2016
4585	N. Bourguignon	Exposure to Inorganic Arsenic during Pregnancy: Effects on the Reproductive Axis and Fertility	2014
4586	F. Gucmann	Optimization of UV-assisted wet oxidation of GaAs	2017
4587	M. Mukwaturi	Mobilization of heavy metals from urban contaminated soils under water inundation conditions	2015
4588	P. A. Gunka	Spatial dispersion of lone electron pairs? - Experimental charge density of cubic arsenic(III) oxide	2015

4589	S. Farouk	Exogenous melatonin-mediated modulation of arsenic tolerance with improved accretion of secondary metabolite production, activating antioxidant capacity and improved chloroplast ultrastructure in rosemary herb	2019
4590	R. A. Glabonjat	Origin of arsenolipids in sediments from Great Salt Lake	2019
4591	P. D. C. Dietzel	Synthesis and crystal structure of tris- 3,5-bis(trifluoromethyl)phenyl arsine	2004
4592	Y. Lu	Arsanilic acid causes apoptosis and oxidative stress in rat kidney epithelial cells (NRK-52e cells) by the activation of the caspase-9 and-3 signaling pathway	2014
4593	R. P. Tan	THE REACTION OF TETRAMESITYLDISILENE WITH AS-4 - SYNTHESIS AND STRUCTURE OF A NOVEL ARSENIC-SILICON TRICYCLIC RING-SYSTEM	1992
4594	I. Komorowicz	Arsenic speciation in mushrooms using dimensional chromatography coupled to ICP-MS detector	2019
4595	M. Taseidifar	Ion flotation removal of a range of contaminant ions from drinking water	2019
4596	X. D. Xie	Phenylarsonics in concentrated animal feeding operations: Fate, associated risk, and treatment approaches	2022
4597	T. Rezanka	Arsenolipids in the green alga Coccomyxa (Trebouxiophyceae, Chlorophyta)	2019
4598	J. C. Guillemin	Reactions of allenyltri-n-butylstannane with halides of phosphorus, arsenic, antimony, germanium, tin, and boron. Preparation of propargylic and/or allenic derivatives	1999
4599	M. Aulitto	Genomic Insight of Alicyclobacillus mali FL18 Isolated From an Arsenic-Rich Hot Spring	2021
4600	A. Jordan	Nickel and cadmium-induced SLBP depletion: A potential pathway to metal mediated cellular transformation	2017
4601	W. A. Schenk	REACTIVE ARSENIC HETEROCYCLIC-COMPOUNDS .3. 3-ARSOLENES - SYNTHESIS AND REACTIONS WITH ARSENIC	1994
4602	F. Plewniak	A Genomic Outlook on Bioremediation: The Case of Arsenic Removal	2018
4603	Y. Y. Li	Assessing the Impact of Atrazine on the Availability of Arsenic in Soils Using DGT Technique	
4604	M. Mori	Association of beer consumption with arsenic concentration in urine: a result from a cross-sectional study of the general Japanese population	2016
4605	A. B. Peltekov	Behavior of arsenic in hydrometallurgical zinc production and environmental impact	2014
4606	S. Y. Wu	Metabolomic assessment of arsenite toxicity and novel biomarker discovery in early development of zebrafish embryos	2018

4607	C. Riesinger	Synthesis and Redox Chemistry of a Homoleptic Iron Arsenic Prismane Cluster	
4608	H. Uesugi	Facile synthesis of colloidal InAs nanocrystals using triphenylarsine as an arsenic source	2014
4609	E. L. Bakota	Heavy metals screening of rice bran oils and its relation to composition	2015
4610	L. A. Beyer	A Comparative Cancer Risk Evaluation of MTBE and Other Compounds (Including Naturally Occurring Compounds) in Drinking Water in New Hampshire	2020
4611	F. M. Francisca	Arsenic removal by coagulation-flocculation processes	2014
4612	H. Sinczuk-Walczak	Neurological and neurophysiological examinations of workers exposed to arsenic levels exceeding hygiene standards	2014
4613	G. Retamal-Morales	Isolation and characterization of arsenic-binding siderophores from Rhodococcus erythropolis S43: role of heterobactin B and other heterobactin variants	2021
4614	G. Kaur	Human red blood cell uptake and sequestration of arsenite and selenite: Evidence of seleno-bis(S-glutathionyl) arsinium ion formation in human cells	2020
4615	J. A. Arcibar-Orozco	Simultaneous desulfuration and denitrogenation of model diesel fuel by Fe-Mn microwave modified activated carbon: Iron crystalline habit influence on adsorption capacity	2019
4616	J. W. Lee	Efficacy and functional study of tetraarsenic oxide as an anticancer drug in cervical cancer cell lines and cervical cancer patient-derived xenograft mouse	2018
4617	A. H. Petursdottir	Quantification of labile and stable non-polar arsenolipids in commercial fish meals and edible seaweed samples	2018
4618	C. N. Peng	Research progress on speciation analysis of arsenic in traditional Chinese medicine	2022
4619	S. L. Zhang	Semiconductor-topological insulator transition of two-dimensional SbAs induced by biaxial tensile strain	2016
4620	Z. B. Zhang	Arsenic Speciation by Sequential Extraction from As-Fe Precipitates Formed Under Different Coagulation Conditions	2016
4621	H. Gonzalez	Effect of pH and citric acid on the growth, arsenic accumulation, and phytochelatin synthesis in Eupatorium cannabinum L., a promising plant for phytostabilization	2019
4622	B. Constantinescu	PIXE and PGAA - Complementary methods for studies on ancient glass artefacts (from Byzantine, late medieval to modern Murano glass)	2018
4623	Y. Tanaka	Utilization of layered double hydroxide to remove arsenic and suppress pH decrement during ultrasound oxidation of arsenious acid	2018
4624	H. M. Shan	Sorption of Monothioarsenate to the Natural Sediments and Its Competition with Arsenite and Arsenate	2021

4625	K. Bambino	Zebrafish in Toxicology and Environmental Health	2017
4626	M. Czub	Acute aquatic toxicity of arsenic-based chemical warfare agents to Daphnia magna	2021
4627	P. Bonazzi	Molecular versus layered structure in arsenic sulphide minerals: the case of duranusite, As4S	2016
4628	T. Marino	The role of arsenic in the hydrolysis and DNA metalation processes in an arsenous acid-platinum(II) anticancer complex	2017
4629	B. A. Marinho	As(III) and Cr(VI) oxyanion removal from water by advanced oxidation/reduction processesa review	2019
4630	M. Weil	Arsenates of Divalent Metals Comprising Arsenic Acid-An Update	2019
4631	J. Assouik	Chemical, structural and electrical resistivity of two first stage arsenic-potassium-graphite intercalation compounds	2015
4632	J. Y. Xing	The effect of W and Mo modification on arsenic adsorption over Cu/gamma-Al2O3 catalyst: Experimental and theoretical analysis	2022
4633	J. F. Sun	First-principles study of tantalum-arsenic binary compounds	2017
4634	A. Pawlowska	Effect of Rhamnolipids and Lipopolysaccharides on the Bioleaching of Arsenic-Bearing Waste	2021
4635	A. Cammerata	Use of Air-Classification Technology to Manage Mycotoxin and Arsenic Contaminations in Durum Wheat-Derived Products	2022
4636	K. Deering	Exposure assessment of toxic metals and organochlorine pesticides among employees of a natural history museum	2020
4637	M. J. Clemente	In Vitro Reduction of Arsenic Bioavailability Using Dietary Strategies	2017
4638	V. G. Guzman-Fierro	Isolation and characterization of an aerobic bacterial consortium able to degrade roxarsone	2015
4639	C. F. Edwardson	Transformation of monothioarsenate by haloalkaliphilic, anoxygenic photosynthetic purple sulfur bacteria	2014
4640	S. Chouchene	Structural, thermal and vibrational study of superprotonic behavior in a cesium-potassium dihydrogen arsenate: CS(0.5)K(0.5)H(2)ASO(4)	2017
4641	J. Schnabel	Zn-MOF-74 as pH-Responsive Drug-Delivery System of Arsenic Trioxide	2020
4642	J. A. Lehmann-Horn	As-75 NQR studies on FeAs2	2015

4643	J. E. Laine	Maternal one carbon metabolism and arsenic methylation in a pregnancy cohort in Mexico (vol 28, pg 505, 2018)	2019
4644	M. Yilmaz	Current and emerging treatments for acute promyelocytic leukemia	2019
4645	L. Ansone-Bertina	Sorption of V and VI group metalloids (As, Sb, Te) on modified peat sorbents	2016
4646	C. Pandey	Arsenic affects the production of glucosinolate, thiol and phytochemical compounds: A comparison of two Brassica cultivars	2017
4647	M. J. Bromstad	The characterization, mobility, and persistence of roaster-derived arsenic in soils at Giant Mine, NWT	2017
4648	M. D. A. Gonzalez-Chavez	Localization and speciation of arsenic in Glomus intraradices by synchrotron radiation spectroscopic analysis	2014
4649	L. Gao	High-Pressure Synthesis and Thermal Transport Properties of Polycrystalline BAsx*	2020
4650	X. J. Liao	Application of macromolecular organic polymer S-7261A in arsenic removal by flotation of refractory mixed copper ore	2022
4651	M. Kampouri	Gestational and childhood urinary iodine concentrations and children's cognitive function in a longitudinal mother-child cohort in rural Bangladesh	
4652	T. Dunaj	Binary interpnictogen compounds bearing diaryl bismuth fragments bound to all tighter pnictogens	2022
4653	A. I. Mireles-Arriaga	PHYSIOLOGICAL MODIFICATIONS AND ANTIOXIDANTS OF WHEAT (Triticuin durum) SEEDLINGS DUE TO ARSENIATE TOXICITY	2019
4654	B. Witt	Toxicity of two classes of arsenolipids and their water-soluble metabolites in human differentiated neurons	2017
4655	M. Cascais	Effects of Heat Treatment Processes: Health Benefits and Risks to the Consumer	2021
4656	A. Singh	Density Functional Theory Study on Sensing and Dielectric Properties of Arsenic Trisulfide Nanosheets for Detecting Volatile Organic Compounds	2021
4657	M. D. Yu	p-Arsanilic acid degradation and arsenic immobilization by a disilicate-assisted iron/aluminum electrolysis process	2019
4658	C. Navarro	Arsenite provides a selective signal that coordinates arsenate uptake and detoxification through the regulation of PHR1 stability in Arabidopsis	2021
4659	V. M. Nurchi	Arsenic Toxicity: Molecular Targets and Therapeutic Agents	2020
4660	V. L. Bilic	First Extensive Polyphenolic Profile ofErodium cicutariumwith Novel Insights to Elemental Composition and Antioxidant Activity	2020

4661	J. C. Han	Rapid Release of Arsenite from Roxarsone Bioreduction by Exoelectrogenic Bacteria	2017
4662	J. Sharma	Phenylarsenic(III) derivatives of heterocyclic dithiocarbamates; Synthesis and characterization	1995
4663	R. Ozola	FeOOH-modified clay sorbents for arsenic removal from aqueous solutions	2019
4664	R. Albrecht	Formation of Tetraselenodiarsenate(II) Anions in Ultra-alkaline Medium	
4665	J. A. L. Cooke	FIRST EXAMPLE OF AN ALUMINUM-PHOSPHORUS-ARSENIC MIXED-PNICOGEN RING COMPOUND - X-RAY CRYSTAL-STRUCTURE OF ET(2)ALP(SIME(3))(2)AL(ET)(2)AS(SIME(3))(2)	1995
4666	D. Y. Zhang	Speciation of inorganic and organic species of mercury and arsenic in lotus root using high performance liquid chromatography with inductively coupled plasma mass spectrometric detection in one run	2019
4667	D. Rottschafer	Isolation of 1,4-Diarsinine-1,4-diide and 1,4-Diarsinine Derivatives	2021
4668	K. Mei	The migrated behavior and bioavailability of arsenic in mangrove sediments affected by pH and organic acids	2020
4669	A. A. Osipov	Thermodynamics of Processes in the Liquid-Metal Pyrolysis of Waste Car Tires	2019
4670	A. J. Ashe	THE 1ST STIBEPINE - SYNTHESIS AND STRUCTURE OF SB-CHLOROBENZO D STIBEPINE	1992
4671	N. Gao	Taurine improves low-level inorganic arsenic-induced insulin resistance by activating PPAR-mTORC2 signalling and inhibiting hepatic autophagy	2019
4672	E. F. Zama	The removal of arsenic from solution through biochar-enhanced precipitation of calcium-arsenic derivatives*	2022
4673	M. J. Clemente	Arsenic speciation in cooked food and its bioaccessible fraction using X-ray absorption spectroscopy	2021
4674	M. Sarker	Adsorption of organic arsenic acids from water over functionalized metal-organic frameworks	2017
4675	A. Nam	Melarsomine suppresses canine osteosarcoma cell survival via inhibition of Hedgehog-GLI signaling	2019
4676	N. Lambrechts	Low level arsenic exposure during pregnancy in the 3xG cohort in Flanders: Less efficient detoxification is associated with higher oxidative stress	2016
4677	W. Zhang	Comparative contribution of trophic transfer and biotransformation on arsenobetaine bioaccumulation in two marine fish	2016
4678	S. Y. Choi	Electrodialysis of groundwater with heavy metal and nitrate ions under low conductivity and effects of superficial velocities	2016

4679	H. Y. Peng	Methylated Phenylarsenical Metabolites Discovered in Chicken Liver	2017
4680	G. H. Zhu	Potential of arsenate-reducing bacterial inoculants to enhance field-scale remediation of arsenic contaminated soils by Pteris vittata L	2021
4681	S. Li	Preparation of CeO2/CaO with Anti-sintering for Efficient Capture of As2O3 from Flue Gas at a High Temperature	2021
4682	W. Ali	Insights into the mechanisms of arsenic-selenium interactions and the associated toxicity in plants, animals, and humans: A critical review	2021
4683	H. Savli	Bortezomib and Arsenic Trioxide Activity on a Myelodysplastic Cell Line (P39): A Gene Expression Study	2015
4684	N. Karimi	Antioxidant enzymes and compounds complement each other during arsenic detoxification in shoots of Isatis cappadocica Desv	2016
4685	D. A. Devault	Micropollutant content of Sargassum drifted ashore: arsenic and chlordecone threat assessment and management recommendations for the Caribbean	
4686	S. Daoud	Structural, elastic, piezoelectric and electronic properties of (B3) AlP compound under pressure	2014
4687	M. J. Clemente	Dietary Strategies To Reduce the Bioaccessibility of Arsenic from Food Matrices	2016
4688	C. Soares	Multi-Step Subcritical Water Extracts of Fucus vesiculosus L. and Codium tomentosum Stackhouse: Composition, Health-Benefits and Safety	2021
4689	S. Zheng	Adsorption and photocatalytic degradation of aromatic organoarsenic compounds in TiO2 suspension	2014
4690	D. Y. Zhang	Simultaneous multi-elemental speciation of As, Hg and Pb by inductively coupled plasma mass spectrometry interfaced with high-performance liquid chromatography	2020
4691	L. L. Song	Exposure to arsenic during pregnancy and newborn mitochondrial DNA copy number: A birth cohort study in Wuhan, China (vol 243, 125335, 2020)	2021
4692	K. R. Malloch	Arsenic mineralogy and distribution at the historic Alexander gold mine, Reefton goldfield, New Zealand	2017
4693	O. M. Kekia	Mixed arsenic-sulfur metallacyclic complexes. Synthesis and structures of (eta(5)-Cp)(2)M(-SCH3AsSCH3AsS-) (M = Ti, Zr, Hf) and (eta(5)-Cp*)(2)Zr(-SCH3AsS-)	1997
4694	S. Hinrichsen	Effect of sulfide on the cytotoxicity of arsenite and arsenate in human hepatocytes (HepG2) and human urothelial cells (UROtsa)	2014
4695	Y. van Dyke	On the formation of hornesite in a Fatimid manuscript folio	2018
4696	D. Chille	Complexation of As(III) by phosphonate ligands in aqueous fluids: Thermodynamic behavior, chemical binding forms and sequestering abilities	2020

4697	X. Y. Tian	The solvothermal synthesis and characterization of quaternary arsenic chalcogenides CsTMAsQ(3)(TM = Hg, Cd; Q = S, Se) using Cs(+)as a structure directing agent: from 1D anionic chains to 2D anionic layers	2020
4698	F. C. Goerigk	The monoclinic rare earth metal(III) chloride oxidoarsenates(III) with the composition RE5Cl3 AsO3 (4) (RE = La-Nd, Sm)	2019
4699	I. Varsanyi	Distribution of dissolved arsenic in a sedimentary environment from the near-surface to a depth of 2500 m, and factors controlling distribution	2017
4700	D. A. Ilutiu-Varvara	Researching the Hazardous Potential of Metallurgical Solid Wastes	2016
4701	J. P. Green	Effect of Arsenic Coordination State on the Structure, Aromaticity, and Optical Properties of Dithieno 3,2-b:2 ',3 '-d arsoles	2019
4702	M. Kato	Uranium in well drinking water of Kabul, Afghanistan and its effective, low-cost depuration using Mg-Fe based hydrotalcite-like compounds	2016
4703	I. Tolbatov	Reactivity of arsenoplatin complex versus water and thiocyanate: a DFT benchmark study	2020
4704	J. Tanaka	Organic Arsenicals as Functional Motifs in Polymer and Biomaterials Science	2018
4705	J. A. Diaz	Bioleaching of Arsenic-Bearing Copper Ores	2018
4706	A. Faucher	Feasibility of arsenic and antimony NMR spectroscopy in solids: An investigation of some group 15 compounds	2014
4707	S. Cernansky	Arsenic ashy soils in Central Slovakia and their chemical and microbiological properties	2017
4708	K. Barrera	Treatment of acidic mine drainage in an adsorption process using calcium silicate modified with Fe(III)	2017
4709	J. Chen	The antibiotic action of methylarsenite is an emergent property of microbial communities	2019
4710	D. Giordano	Arsenic, lead and cadmium distribution in the pearled fractions of different winter wheat cultivars (Triticum aestivum L.)	2018
4711	R. A. Glabonjat	Arsenolipids in Plankton from High- and Low-Nutrient Oceanic Waters Along a eTransect in the North Atlantic	2021
4712	A. Nikiforova	Application of lime in two-stage purification of leaching solution of spent vanadium catalysts for sulfuric acid production	2017
4713	L. Arabuli	Arsenobetaines - potential bidentate ligands and optimised crystal structure of new metal(ii)-arsenic hybrid compounds	2014
4714	J. A. Strauss	Arsenic behavior during the treatment of refractory gold ores via POX: Characterization of Fe-AsO4-SO4 precipitates	2021

4715	H. Zhao	Impedance Monitoring of SH-SY5Y Cell Adhesion and Responses to As2O3 Treatment by Indium Tin Oxide Microelectrode Arrays	2017
4716	F. Arcega-Cabrera	Environmental Exposure of Children to Toxic Trace Elements (Hg, Cr, As) in an Urban Area of Yucatan, Mexico: Water, Blood, and Urine Levels	2018
4717	E. D. Gezer	Effects of wood species and retention levels on removal of copper, chromium, and arsenic from CCA-treated wood using sodium hypochlorite	2016
4718	L. P. de Matos	Simultaneous removal of sulfate and arsenic using immobilized non-traditional SRB mixed culture and alternative low-cost carbon sources	2018
4719	V. Nesterov	Advances in Phosphasilene Chemistry	2017
4720	Y. D. Xue	Electrochemical detoxification and recovery of spent SCR catalyst by in-situ generated reactive oxygen species in alkaline media	2017
4721	Y. H. Sun	Arsenic-Centered Molybdenum-Vanadium Polyoxometalate Supporting Three Copper Complexes	2014
4722	R. L. Wells	Synthesis and characterization of tert-butylgallium-antimony compounds: X-ray crystal structures of t-Bu3Ga center dot Sb(SiMe3)(3), t-Bu2GaSb(SiMe3)(2) (2), and t-Bu2GaSb(SiMe3)(2)Ga(t-Bu)(2)Cl, the first example of a gallium-antimony mixed-bridge	1997
4723	P. Colomban	Non-invasive on-site Raman study of blue-decorated early soft-paste porcelain: The use of arsenic-rich (European) cobalt ores - Comparison with huafalang Chinese porcelains	2018
4724	J. Lan	Curcumin alleviates arsenic-induced injury in duck skeletal muscle via regulating the PINK1/Parkin pathway and protecting mitochondrial function	2022
4725	Z. Liu	Sulfur reduces arsenic accumulation in rice shoot by enhancing root retention and altering arsenic metabolism	2022
4726	Y. Zhang	Experiment and mechanism research on gas-phase As2O3 adsorption of Fe2O3/gamma-Al2O3	2016
4727	A. J. Henegar	Native oxide transport and removal during the atomic layer deposition of Ta2O5 on InAs(100) surfaces	2016
4728	E. Bralatei	Detection of Inorganic Arsenic in Rice Using a Field Test Kit: A Screening Method	2015
4729	Y. N. Zhu	Dissolution and Solubility of the Synthetic Natroalunite and the Arsenic-Incorporated Natroalunite at pH of 2.00-5.60 and 25-45 degrees C	2019
4730	W. X. Qin	Oxidation mechanism of As(III) in the presence of polyphenols: New insights into the reactive oxygen species	2016
4731	M. Ayiania	Characterization of solid and vapor products from thermochemical conversion of municipal solid waste woody fractions	2019
4732	V. Diacomanolis	Bioavailability and pharmacokinetics of arsenic are influenced by the presence of cadmium	2014

4733	A. Weitze	Polysulfonylamines .55. synthesis and solid-state structure of organoarsenic(iii) dimesylamides - molecules with unusually long as-n bonds and intramolecullar as...o interactions	1993
4734	S. K. Hadjikakou	Recent advances on antimony(III/V) compounds with potential activity against tumor cells	2015
4735	A. T. Lucero	Formation of a ZnO/ZnS interface passivation layer on (NH4)(2)S treated In0.53Ga0.47As: Electrical and in-situ X-ray photoelectron spectroscopy characterization	2016
4736	Z. Y. Guo	Cr and As decrease lindane sorption on river solids	2015
4737	Y. Mitsumoto	Synthesis, structure, and reactivity of (triphenylarsoranylidene)-methylcyclohepta-2,4,6-trienone derivatives: reactions with heterocumulenes and an activated acetylene	2002
4738	S. Kochmann	Hyphenation of capillary high-performance ion-exchange chromatography with mass spectrometry using sheath-flow electrospray ionization	2014
4739	S. Kagami	Solid-source vapor growth and optoelectronic properties of arsenic-based layered group-IV monopnictides	2022
4740	P. Chooto	Determination of inorganic arsenic species by hydride generation atomic absorption spectrophotometry and cathodic stripping voltammetry	2015
4741	B. C. Monseur	High groundwater arsenic contamination is associated with stillbirth, recurrent pregnancy loss, and infertility: a population-based study in india of 643,944 reproductive aged women	2021
4742	S. Sarkar	Recent advances in semimetallic pnictogen (As, Sb, Bi) based anodes for sodium-ion batteries: Structural design, charge storage mechanisms, key challenges and perspectives	2021
4743	S. Mafla	Biodegradation of roxarsone by a bacterial community of underground water and its toxic impact	2015
4744	P. Y. Lai	Arsenic and Rice: Translating Research to Address Health Care Providers' Needs	2015
4745	M. Sijko	Influence of Dietary Compounds on Arsenic Metabolism and Toxicity. Part I-Animal Model Studies	2021
4746	J. Assouik	Heavy alkali metal-arsenic alloy-based graphite intercalation compounds: Investigation of their synthesis and of their physical properties	2017
4747	U. Winkler	Through-bond interactions in silicon-phosphorus and silicon-arsenic compounds: A facile synthesis of dodecamethyl-2,3,5,6,7,8-hexasila-1 lambda(3),4 lambda(3)-diphosphabicylo 2.2.2 octane, its arsenic analogue, and related compounds	1997
4748	F. Yang	Do homegrown cage-free chickens from an old arsenic mine pose health risks to consumers?	2022
4749	X. L. Cai	Impact of Organic Matter on Microbially-Mediated Reduction and Mobilization of Arsenic and Iron in Arsenic(V)-Bearing Ferrihydrite	2021
4750	R. D. Lobato	The effect of diet enriched with lipoic acid in the accumulation and metabolization of metals in different organs of Litopenaeus vannamei	2018

4751	J. J. Milledge	Sargassum Inundations in Turks and Caicos: Methane Potential and Proximate, Ultimate, Lipid, Amino Acid, Metal and Metalloid Analyses	2020
4752	A. Panyushkina	Understanding Stress Response to High-Arsenic Gold-Bearing Sulfide Concentrate in Extremely Metal-Resistant AcidophileSulfobacillus thermotolerans	2020
4753	D. Kreutz	Response Profiling Using Shotgun Proteomics Enables Global Metallodrug Mechanisms of Action To Be Established	2017
4754	C. Marquardt	Cationic Chains of Phosphanyl-and Arsanylboranes	2014
4755	L. M. Frensemeier	Investigation of the oxidative transformation of roxarsone by electrochemistry coupled to hydrophilic interaction liquid chromatography/mass spectrometry	2017
4756	D. You	KML001 Induces Apoptosis and Autophagic Cell Death in Prostate Cancer Cells via Oxidative Stress Pathway	2015
4757	J. P. Ogalde	Multi-instrumental identification of orpiment in archaeological mortuary contexts	2014
4758	J. S. Xiu	Quantitative toxicological study of dose-dependent arsenic-induced cells via synchrotron-based STXM and FTIR measurement	2020
4759	X. Pi	Arsenic Exposure, Periconceptional Folic Acid Supplementation, and the Risk for Neural Tube Defects: A Case-Control Study	
4760	S. Bisone	Geochemical characterization and modeling of arsenic behavior in a highly contaminated mining soil	2016
4761	M. H. Rahbar	Correlation between concentrations of four heavy metals in cord blood and childhood blood of Jamaican children	2021
4762	T. Demonchaux	Chemical nature of the anion antisite in dilute phosphide GaAs1-xPx alloy grown at low temperature	2018
4763	S. Paul	Impact of soil addendum on arsenic uptake by rice plant in the alluvial soil of gangetic West Bengal, India	2020
4764	H. J. Sun	Arsenic and selenium toxicity and their interactive effects in humans	2014
4765	P. Wu	The Synergistic Effects of Decitabine Combined with Arsenic Trioxide (ATO) in the Human Myelodysplastic Syndrome Cell Line SKM-1	2016
4766	J. Delafiori	Clinical applications of HPLC-ICP-MS element speciation: A review	2016
4767	I. Dhouib	Structural, vibrational and thermal studies of a new nonlinear optical crystal tetrapropylammonium dihydrogenmonoarsenate bis arsenic acid	2014
4768	D. Podar	The role of roots and rhizosphere in providing tolerance to toxic metals and metalloids	2022

4769	N. Matsuo	Extraction Behavior of Arsenic, Selenium, and Antimony Using Cyclopentyl Methyl Ether from Acidic Chloride Media	2019
4770	A. Ruttens	Arsenic speciation in food in Belgium. Part 2: Cereals and cereal products	2018
4771	B. Basaran	An assessment of heavy metal level in infant formula on the market in Turkey and the hazard index	2022
4772	R. S. Dickson	The assessment of some sb-te single-source compounds for mocvd applications	1995
4773	H. W. Jiang	Recovery of arsenic and practical utilization of aqueous phase in hydrothermal liquefaction of hyperaccumulator	2022
4774	E. I. Musina	A Series of Cu2I2 Complexes of 10-(Aryl)phenoxarsines: Synthesis and Structural Diversity	2017
4775	T. A. Afolabi	A selective and efficient chemosensor for the rapid detection of arsenic ions in aqueous medium	2022
4776	M. Ceppatelli	Single-Bonded Cubic AsN from High-Pressure and High-Temperature Chemical Reactivity of Arsenic and Nitrogen	2022
4777	T. Pflaum	Carcinogenic compounds in alcoholic beverages: an update	2016
4778	H. Kihara	Synthesis of main-chain-type triphenylarsine polymers	
4779	M. Seidl	Stepwise Formation of a 1,3-Butadiene Analogue of Mixed Heavier Group 15 Elements	2016
4780	M. Cruells	Jarosites: Formation, Structure, Reactivity and Environmental	2022
4781	J. Kim	Quantum chemical study of molecular properties of AsXn (X = F and Cl, n=1-5) and AsXn- (X = F and Cl, n=1-6)	2020
4782	M. Schmidt	Arsenic-Rich Polyarsenides Stabilized by Cp*Fe Fragments	2017
4783	W. Maringgele	Reaction of lithium 2-hydroxybiphenyl and 2'-hydroxy-m-terphenyl with halogen compounds of silicon and germanium and phosphorus and arsenic	1994
4784	F. Spitzer	The Influence of beta-diiminato Ligands on As-4 Activation by Cobalt Complexes	2018
4785	J. Assouik	Synthesis, structure and electrical behavior of the heavy alkali metal-arsenic alloys based graphite intercalation compounds	2016
4786	A. Hata	Metabolism of 3- 5 '-deoxy-5 '-(dimethylarsinoyl)-beta-ribofuranosyloxy -2-hydroxypropylene glycol in an artificial digestive system	2019

4787	X. Wang	LG-362B targets PML-RAR alpha and blocks ATRA resistance of acute promyelocytic leukemia	2016
4788	M. Haimerl	Coordination of cage compounds by Cu(I) nacnac compounds	2022
4789	Y. Y. Chang	Highly Selective Fluorescent Sensing of Phosphite through Recovery of Poisoned Nickel Oxide Nanozyme	2020
4790	J. Castro-Severyn	Arsenic Response of Three Altiplanic Exiguobacterium Strains With Different Tolerance Levels Against the Metalloid Species: A Proteomics Study	2019
4791	U. Muller	Ionothermal Synthesis, Crystal Structure, and Chemical Bonding of the Niobium(IV) Complex Nb-2(Se-2)(2)(AlCl4)(4)	2014
4792	H. Steffenfauseweh	Isolation of an Arsenic Diradicaloid with a Cyclic C2As2-Core	
4793	P. C. Zhao	Microscopic Spherical alpha-Fe2O3 for Highly Efficient Gaseous Arsenic Capture in Simulated Flue Gas Under a Wide Temperature Range	2021
4794	K. Megges	Synthesis and crystal structure of 1,2-Di-t-butyl-1,2-diiododiarsane (t-C4H9AsI)(2) and synthesis of 1,2,3,4-tetrakis(t-butyl)tetraarsetane (t-C4H9As)(4) via selective reduction of t-butylarsenic diiodide t-C4H9AsI2	1997
4795	M. A. Brown	Synthesis and molecular-structure of methyl 4,6-benzylidene-3-deoxy-3-diphenylarsino-alpha-d-altropyranoside	1995
4796	P. Margiastuti	An unusual iodinated 5 '-deoxyxylofuranosyl nucleoside from an Okinawan ascidian, Diplosoma sp	2008
4797	M. Tuchowska	Immobilization of arsenic compounds by bog iron ores	2019
4798	H. H. Rahman	Association between environmental toxic metals, arsenic and polycyclic aromatic hydrocarbons and chronic obstructive pulmonary disease in the US adult population	
4799	M. Lopez-Guzman	Electrocoagulation Process: An Approach to Continuous Processes, Reactors Design, Pharmaceuticals Removal, and Hybrid Systems-A Review	2021
4800	J. Y. Y. Wong	The Relationship Between Occupational Metal Exposure and Arterial Compliance	2015
4801	A. R. Kampf	Mauriziodiniite, NH4(As2O3)(2)I, the ammonium and iodine analogue of lucabindiite from the Torrecillas mine, Iquique Province, Chile	2020
4802	C. H. Syu	Effect of applying calcium peroxide on the accumulation of arsenic in rice plants grown in arsenic-elevated paddy soils	2020
4803	R. W. Burli	Novel inhibitors of As(III) S-adenosylmethionine methyltransferase (AS3MT) identified by virtual screening	2018
4804	I. Shiue	Urinary arsenic, heavy metals, phthalates, pesticides, polyaromatic hydrocarbons but not parabens, polyfluorinated compounds are associated with self-rated health: USA NHANES, 2011-2012	2015

4805	E. Kociolek-Balawejde	CuO and Cu-2(OH)(3)Cl loaded gel-type anion exchange hybrid polymers obtained via tetrachlorocuprate ionic form	2017
4806	X. Y. Tian	Strong Photocurrent Response of Selenoarsenates With Different Transition Metal Complexes as Structure-Directing Agents	2022
4807	F. Yang	Effects of Methanol Addition on Arsenic Speciation Analysis with HPLC-ICP-MS	2018
4808	D. Nikolova	Novel binary compounds of group 15 elements: Synthesis and characterisation of Sb-4(PSiMe(2)Thex)4 , Sb-4(AsSiiPr(3))(4) and Sb-2(PSiPh(2)tBU)4	2005
4809	M. E. Brier	Serum trace metal association with response to erythropoiesis stimulating agents in incident and prevalent hemodialysis patients	2020
4810	A. V. Skalny	Toxicological and nutritional status of trace elements in hair of women with in vitro fertilization (IVF) pregnancy and their 9-month-old children	2018
4811	Z. Bujnakova	Preparation, properties and anticancer effects of mixed As4S4/ZnS nanoparticles capped by Poloxamer 407	2017
4812	E. Fidrus	Inhibitors of Nucleotide Excision Repair Decrease UVB-Induced Mutagenesis-An In Vitro Study	2021
4813	M. H. Jin	Arsenic chemistry in municipal sewage sludge dewatering, thermal drying, and steam gasification: Effects of Fenton-CaO conditioning	2022
4814	L. X. Guo	Determination of Arsenic Species in Ophiocordyceps sinensis from Major Habitats in China by HPLC-ICP-MS and the Edible Hazard Assessment	2018
4815	I. M. Barsoe	Drinking Water Arsenic and Adverse Reproductive Outcomes in Men and Women: A Systematic PRISMA Review	2021
4816	A. Brosius	Synthesis and properties of s-(trifluoromethyl)polysulfanemonosulfonates cf(3)s(x)so(2)m x=1, m=oh, nh4o, 1/2 hgo2, (ch3)(3)sio, (ch3)(2)n, nh2 x=2, m=nh4o	1994
4817	H. F. Bakhat	Arsenic uptake, accumulation and toxicity in rice plants: Possible remedies for its detoxification: A review	2017
4818	W. B. Wang	Application of weak magnetic field coupling with zero-valent iron for remediation of groundwater and wastewater: A review	2020
4819	L. Prokes	Laser ablation synthesis of new gold arsenides using nano-gold and arsenic as precursors. Laser desorption ionisation time-of-flight mass spectrometry and spectrophotometry	2014
4820	O. V. Perlova	Hydrated titanium dioxide modified with potassium cobalt hexacyanoferrate(II) for sorption of cationic and anionic complexes of uranium(VI)	2022
4821	V. Q. Nguyen	Effect of aluminum and tellurium tetrachloride addition on the loss of arsenic selenide optical fiber	2017
4822	C. Mangia	Arsenic contamination assessment 40years after an industrial disaster: measurements and deposition modeling	2018

4823	H. G. Li	Hydrothermal liquefaction accelerates the toxicity and solubility of arsenic in biowaste	2021
4824	C. Jadan-Piedra	Dietary compounds as modulators of metals and metalloids toxicity	2018
4825	N. Sawant	Chemically Functionalized Polysaccharide-Based Chelating Agent for Heavy Metals and Nitrogen Compound Remediation from Contaminated Water	2022
4826	B. Demircioglu	Effects of Curcumin on Sodium Arsenite Induced Neoplastic Cell Transformation in Balb/c 3T3 Cells	2021
4827	H. Niemikoski	Detection of chemical warfare agent related phenylarsenic compounds and multibiomarker responses in cod (Gadus morhua) from munition dumpsites	2020
4828	M. H. Al Amin	Variation in arsenolipid concentrations in seafood consumed in Japan	2020
4829	A. Polo	Identifying a panel of genes/proteins/miRNAs modulated by arsenicals in bladder, prostate, kidney cancers	2018
4830	H. T. Nguyen	C<bold>haracteristics of Amorphous As2S3 Semiconductor Films Obtained via Spin Coating</bold>	2018
4831	Y. Liu	Hg5AsS2I3 - A Narrow-Band-Gap 2D Layered Compound with Different Trapped I- Anions	2015
4832	M. N. Islam	Remediation approach for organic compounds and arsenic co-contaminated soil using the pressurized hot water extraction process	2019
4833	A. Firrincieli	Transcriptomic Analysis of the Dual Response of Rhodococcus aetherivorans BCP1 to Inorganic Arsenic Oxyanions	2022
4834	J. T. Ahlemann	The role of the 2,4,6-tris(trifluoromethyl)phenylamino group in stabilizing new phosphorus-, arsenic-, and germanium-containing main-group compounds and transition-metal derivatives	1997
4835	X. M. Xue	Identification of Steps in the Pathway of Arsenosugar Biosynthesis	2019
4836	U. Lewandowska	Synergistic Interactions Between Anticancer Chemotherapeutics and Phenolic Compounds and Anticancer Synergy Between Polyphenols	2014
4837	S. Le Serre	Allylation of phosphorus, arsenic, and antimony trihalides by allylic stannanes. Synthesis, spectroscopic characterization, and quantum chemical investigations of allylic phosphines, arsines, and stibines	1998
4838	D. C. Gordon	Synthesis and characterization of volatile trifluoromethyl alkyl tellurides	1992
4839	J. C. Guillemin	Primary and secondary vinylarsines - synthesis, stability, and characterization	1994
4840	M. Nentwig	Single crystal structure elucidation and thermoelectric properties of a long-periodically ordered germanium arsenic telluride	2017

4841	T. Masuda	Long-term accumulation of diphenylarsinic acid in the central nervous system of cynomolgus monkeys	2017
4842	S. Koechler	Arsenite response in Coccomyxa sp Carn explored by transcriptomic and non-targeted metabolomic approaches	2016
4843	R. Agrawal	Syntheses, reactions, characterization, and antifungal activities of chloro-bis-(2,2-dithio-1,3,2-dioxaphospholane/dioxaphosphorinane)bismuth (III)	2011
4844	X. Y. Guo	Selective removal of As from arsenic-bearing dust rich in Pb and Sb	2019
4845	J. Z. Liu	A Cross-Validated Ensemble Approach to Robust Hypothesis Testing of Continuous Nonlinear Interactions: Application to Nutrition-Environment Studies	2022
4846	V. Dan'ko	The nanostructuring of surfaces and films using interference lithography and chalcogenide photoresist	2015
4847	R. Sinitkul	Children's Environmental Health in Thailand: Past, Present, and Future	2018
4848	J. Oyen	Lean-seafood intake increases urinary iodine concentrations and plasma selenium levels: a randomized controlled trial with crossover design	2021
4849	V. Y. Lee	A "Push-Pull" Phosphasilene and Phosphagermene and Their Anion-Radicals	2009
4850	J. X. Qian	A novel and facile synthesis of 2,3-dihydrofuran derivatives containing trifluoromethyl group	2007
4851	P. J. Lv	An Unusual Bi-arsenic Capped Well-Dawson Arsenomolybdate Hybrid Supramolecular Material with Photocatalytic Property and Anticancer Activity	2018
4852	S. Chouchene	Structural, thermal behavior, vibrational study and superprotonic behavior of a new rubidium dihydrogen phosphate-arsenate: RbH2(PO4)0.65(ASO(4))0.35	2017
4853	V. M. Ngole-Jeme	An analysis of human exposure to trace elements from deliberate soil ingestion and associated health risks	2018
4854	G. Kerr	Experimental Metalloid Mobilisation from a New Zealand Orogenic Gold Deposit	2015
4855	J. H. Cheng	Antioxidant xanthone derivatives induce cell cycle arrest and apoptosis and enhance cell death induced by cisplatin in NTUB1 cells associated with ROS	2011
4856	K. E. Levine	Characterization of Zinc Carbonate Basic as a Source of Zinc in a Rodent Study Investigating the Effects of Dietary Deficiency or Excess	2017
4857	P. Wlodarczyk	Impact of silicon doping on the magnetocaloric effect of MnFeP0.35As0.65 powder	2016
4858	K. Lewinska	Arsenic Forms in Soils of Various Settings in the Historical Ore Mining and Processing Site of Radzimowice, Western Sudetes	2021

4859	Z. F. Wang	Evaluation of single and joint toxicity of perfluorooctanoic acid and arsenite to earthworm (Eisenia fetida): A multi-biomarker approach	2022
4860	L. Coll-SanMartin	Gene Amplification-Associated Overexpression of the Selenoprotein tRNA Enzyme TRIT1 Confers Sensitivity to Arsenic Trioxide in Small-Cell Lung Cancer	2021
4861	Y. Berset	Mechanistic Modeling of Genetic Circuits for ArsR Arsenic Regulation	2017
4862	P. Lake	The Influence of Underlying Stresses from Environmental Hazards on Resilience in Bangladesh: A System View	2019
4863	M. Vermeulen	Evidence of early amorphous arsenic sulfide production and use in Edo period Japanese woodblock prints by Hokusai and Kunisada	2019
4864	Y. Kapadia	A comprehensive study on amalgamation of sustainable solar powered distillation for arsenic and fluoride removal from groundwater	2021
4865	M. Marmiroli	Abiotic Stress Response to As and As plus Si, Composite Reprogramming of Fruit Metabolites in Tomato Cultivars	2017
4866	K. Ma	A computational study on the adsorption of arsenic pollutants on graphene-based single-atom iron adsorbents	2022
4867	W. T. Lin	Determining the Residual Characteristics of Alkylphenols, Arsenic, and Lead as well as Assessing the Exposures of 1,4-Dioxane from Household Food Detergents	2017
4868	C. L. Li	Ebb-and-Flow of Macroautophagy and Chaperone-Mediated Autophagy in Raji Cells Induced by Starvation and Arsenic Trioxide	2014
4869	C. Chen	Sulfate addition and rising temperature promote arsenic methylation and the formation of methylated thioarsenates in paddy soils	2021
4870	X. M. Ma	Mossbauer study of magnetic fluctuation in the iron-arsenic layer of Sr2VO3FeAs	2014
4871	A. K. R. Kumar	Remediation of bio-refinery wastewater containing organic and inorganic toxic pollutants by adsorption onto chitosan-based magnetic nanosorbent	2020
4872	Y. F. Liu	Tetra-arsenic tetra-sulfide (As4S4) promotes apoptosis in retinoid acid -resistant human acute promyelocytic leukemic NB4-R1 cells through downregulation of SET protein	2014
4873	A. Ghaffar	Cumulative Effects of Sodium Arsenate and Diammonium Phosphate on Growth Performance, Hemato-Biochemistry and Protoplasm in Commercial Layer	2017
4874	M. H. Yang	Multicenter randomized trial of arsenic trioxide and Realgar-Indigo naturalis formula in pediatric patients with acute promyelocytic leukemia: Interim results of the SCCLG-APL clinical study	2018
4875	A. von Dollen	Coordination chemistry of lipoic acid and related compounds, 2 - Models for the inhibition of dithiol-containing enzymes by organoarsenic compounds: Synthetic routes and the structure of PhAs(HlipS(2)) (HlipS(2)(2-)=reduced-lipoic-acid)	1998
4876	J. Serrano	Removal of Arsenic Using Acid/Metal-Tolerant Sulfate Reducing Bacteria: A New Approach for Bioremediation of High-Arsenic Acid Mine Waters	2017

4877	Y. H. Bai	Antimony oxidation and adsorption by in-situ formed biogenic Mn oxide and Fe-Mn oxides	2017
4878	G. L. Zhong	Methionine Sulfoxide Reductases Are Related to Arsenic Trioxide-Induced Oxidative Stress in Mouse Liver	2020
4879	W. Zhang	Arsenic biokinetics and bioavailability in deposit-feeding clams and polychaetes	2018
4880	M. Haimerl	Conversion of E-4 (E-4=P-4, As-4, AsP3) by Ni(0) and Ni(I) Synthons - A Comparative Study	2021
4881	K. Guemiza	Treatment technologies used for the removal of As, Cr, Cu, PCP and/or PCDD/F from contaminated soil: A review	2017
4882	P. Weis	Silver Coordination Chemistry of the Weakly Basic Cage As4S4	2018
4883	K. Manquian-Cerda	Preparation of nanoscale iron (oxide, oxyhydroxides and zero-valent) particles derived from blueberries: Reactivity, characterization and removal mechanism of arsenate	2017
4884	Y. Zhao	Removal of arsenic from flue gas by using NaClO solution	2017
4885	Q. L. Fu	Extraction and speciation analysis of roxarsone and its metabolites from soils with different physicochemical properties	2016
4886	J. A. Sorrentino	p16(INK4a) reporter mice reveal age-promoting effects of environmental toxicants	2014
4887	J. G. Zhao	Structural Phase Transitions and Metallized Phenomena in Arsenic Telluride under High Pressure	2016
4888	F. N. Khosroshahi	A New, Second Generation Trithiol Bifunctional Chelate for (72,77)AS: Trithiol(b)-(Ser)(2)-RM2	2021
4889	K. Radinovic	Electroanalytical sensing of trace amounts of As(III) in water resources by Gold-Rare Earth alloys	2020
4890	J. C. Philippe	Elasto-Raman scattering: Arsenic optical phonon as a probe of nematicity in BaFe2As2	2022
4891	S. Heinl	Unexpected differences in the reactivity between the phosphorus and arsenic derivatives ((CpFe)-Fe-BIG)(2)(,(4:4)-E-4) (E = P and As)	2018
4892	S. Musil	Speciation without Chromatography Using Selective Hydride Generation: Inorganic Arsenic in Rice and Samples of Marine Origin	2014
4893	J. H. Shin	Investigation of Spectral Variation of Pine Needles as an Indicator of Arsenic Content in Soils	2019
4894	W. Liao	Change of Arsenic Speciation in Shellfish after Cooking and Gastrointestinal Digestion	2018

4895	M. J. Pennino	Wildfires can increase regulated nitrate, arsenic, and disinfection byproduct violations and concentrations in public drinking water supplies	2022
4896	M. Stern	Human Ntera2 cells as a predictive in vitro test system for developmental neurotoxicity	2014
4897	C. J. Ollson	Influence of sample matrix on the bioavailability of arsenic, cadmium and lead during co-contaminant exposure	2017
4898	H. G. Du	Synthesis and characterization of homoannularly disubstituted and heteroannularly trisubstituted ferrocene derivatives by arsenic and silicon or by arsenic and tin	2007
4899	E. J. Ditzel	Altered Hepatic Transport by Fetal Arsenite Exposure in Diet-Induced Fatty Liver Disease	2016
4900	J. Magura	Toxic metals (As and Pb) in Sargassum elegans Suhr (1840) and its bioactive compounds	2019
4901	L. Yan	Recent progress of arsenic adsorption on TiO2 in the presence of coexisting ions: A review	2016
4902	G. Bia	Arsenic in natural carbonates: The role of the biogeochemical conditions in its solid speciation	2021
4903	M. Rafiq	Comparative effect of calcium and EDTA on arsenic uptake and physiological attributes of Pisum sativum	2017
4904	J. Ramos-Trevino	Toxic Effect of Cadmium, Lead, and Arsenic on the Sertoli Cell: Mechanisms of Damage Involved	2018
4905	V. Verma	Acute promyelocytic leukemia during pregnancy: a systematic analysis of outcome	2016
4906	I. de la Calle	Nanoparticle-assisted stabilization of metal species as an alternative to conventional approaches for avoiding volatilization errors in total reflection X-ray fluorescence: A review	2020
4907	S. T. Li	Sodium arsenite-mediated upregulation of circDHX34 promotes apoptosis in hormone-independent breast cancer cells by regulating apoptotic genes	2022
4908	M. Kippler	Elevated childhood exposure to arsenic despite reduced drinking water concentrations - A longitudinal cohort study in rural Bangladesh	2016
4909	G. C. Song	Coupling effects of mineral components on arsenic transformation during coal combustion	2022
4910	C. Nunez	Development of an amperometric sensor based on the synergistic action between alginic acid and nPEDOT on a gold nanoparticle-modified screen-printed carbon electrode for As(III) determination in natural water samples	2021
4911	P. Mondal	Environmental exposure of arsenic and fluoride and their combined toxicity: A recent update	2020
4912	M. Zhu	Diphenylarsinic acid sorption mechanisms in soils using batch experiments and EXAFS spectroscopy	2020

4913	F. L. Forray	Synthesis, characterization and thermochemistry of synthetic Pb-As, Pb-Cu and Pb-Zn jarosites	2014
4914	S. K. Singh	Mapping composite vulnerability to groundwater arsenic contamination: an analytical framework and a case study in India	2015
4915	X. Li	Green and Simple Extraction of Arsenic Species from Rice Flour Using a Novel Ultrasound-Assisted Enzymatic Hydrolysis Method	2022
4916	H. Uesugi	Synthesis of size-controlled colloidal InAs quantum dots using triphenylarsine as a stable arsenic source	2015
4917	H. Schumann	Synthesis, structure determination and catalytic activity of (+)-cis- dicarbonyl-mu-chloro-mu-5-beta-methyl-2-alpha-(1-methylethyl)cyclohexanethiolato-bis tris(1,1-dimethylethyl)arsine dirhodium	1991
4918	V. P. Cuenca-Gotor	Structural, Vibrational, and Electronic Study of alpha-As2Te3 under Compression	2016
4919	R. V. Okunev	Free Amino Acid Accumulation in Soil and Tomato Plants (Solanum lycopersicum L.) Associated with Arsenic Stress	2019
4920	E. Nies	New reference assessment values of the Committee for Risk Assessment (RAC) for chemicals subject to authorisation	2014
4921	A. Chlebicki	Fungi are not involved in biofilm formation on rock wall in subterranean arsenic mine in Poland	2014
4922	M. Czaplicka	Determination of selected organoarsenic compounds by SPME/GC-MS in aquatic samples	2019
4923	L. L. Yu	SI traceable determination of arsenic species in kelp (Thallus laminariae)	2017
4924	J. Contreras-Garcia	Borates or phosphates? That is the question	2020
4925	S. Cavalera	Stoichiometric molecular imprinting using polymerisable urea and squaramide receptors for the solid phase extraction of organo-arsenic compound roxarsone	2020
4926	A. Garbagnati	Halogenation and Nucleophilic Quenching: Two Routes to E-X Bond Formation in Cobalt Triple-Decker Complexes (E = As, P; X = F, Cl, Br, I)	2022
4927	C. Q. Zhu	Two-temperature synthesis of non-linear optical compound CdGeAs2	2016
4928	N. El Omari	Anticancer mechanisms of phytochemical compounds: focusing on epigenetic targets	2021
4929	D. Delgado-Diaz	N-S co-doped TiO2 synthesized by microwave precipitation method: Effective photocatalytic performance for the removal of organoarsenic compounds	2021
4930	M. Schoneich	Element allotropes and polyanion compounds of pnicogenes and chalcogenes: stability, mechanisms of formation, controlled synthesis and characterization	2017

4931	J. Noda	Investigation of chromated copper arsenate-treated waste wood used for bedding material in the Hokkaido area	2018
4932	J. C. Beaulieu	Green Processing, Germinating and Wet Milling Brown Rice (Oryza sativa) for Beverages: Physicochemical Effects	2020
4933	J. F. Binder	Synthesis of Heavy Dicyanamide Homologues from Air-Stable Precursors	2018
4934	P. Le Pape	Local environment of arsenic in sulfide minerals: insights from high-resolution X-ray spectroscopies, and first-principles calculations at the As K-edge	2018
4935	D. K. Sharma	Coordination chemistry of trivalent and pentavalent organoarsenic heterocyclic dithiocarbamate derivatives: synthesis and characterization	2014
4936	N. Coltella	Synergistic Leukemia Eradication by Combined Treatment with Retinoic Acid and HIF Inhibition by EZN-2208 (PEG-SN38) in Preclinical Models of PML-RAR alpha and PLZF-RAR alpha-Driven Leukemia	2015
4937	R. L. Wells	Dehalosilylation reactions involving e(sime3)3 and ph2incl - synthesis and x-ray structures of ph2ine(sime3)2 2 (e = p or as)	1993
4938	K. Schwendtner	M+M23+As(HAsO4)(6) and alpha- and beta-M+M3+(HAsO4)(2)(M+M3+ = RbAl or CsFe): six new compounds crystallizing in three closely related structure types	2018
4939	M. A. K. Weinhart	NHC-stabilized Parent Arsanylalanes and -gallanes	2021
4940	M. E. Romano	Maternal urinary cadmium, glucose intolerance and gestational diabetes in the New Hampshire Birth Cohort Study	2019
4941	K. Drwal	Photoactive Materials for Decomposition of Organic Matter Prior to Water Analysis-A Review Containing Original Research	2022
4942	S. Sauve	A review of what is an emerging contaminant	2014
4943	I. Shiue	Urinary heavy metals, phthalates, perchlorate, nitrate, thiocyanate, hydrocarbons, and polyfluorinated compounds are associated with adult hearing disturbance: USA NHANES, 2011-2012	2015
4944	X. Y. Huang	Iron(III)-induced photooxidation of arsenite in the presence of carboxylic acids and phenols as model compounds of natural organic matter	2021
4945	L. F. Mashadieva	Phase Equilibria in the Cu2Se-Cu3AsSe4-Se System and Thermodynamic Properties of Cu3AsSe4	2018
4946	K. Merz	In situ Crystallization of N(SiMe3)(3) and As(SiMe3)(3): Trigonal planar or pyramidal coordination of the central atoms?	2014
4947	Q. Li	Exploring the associations between microRNA expression profiles and environmental pollutants in human placenta from the National Children's Study (NCS)	2015
4948	M. C. Jalova	Assessment of heavy metals found in commonly consumed fishes from Lake Lanao, Philippines	2021

4949	M. Stulovic	Leaching of toxic elements from secondary alkaline lead slag and stabilized/solidified products	2019
4950	I. Shiue	Arsenic, heavy metals, phthalates, pesticides, hydrocarbons and polyfluorinated compounds but not parabens or phenols are associated with adult remembering condition: US NHANES, 2011-2012	2015
4951	E. Alvarez-Ayuso	Antimony distribution and mobility in different types of waste derived from the exploitation of stibnite ore deposits	2022
4952	S. L. Man	Treatment for liver cancer: From sorafenib to natural products	2021
4953	S. R. M. Pinson	Relationships Among Arsenic-Related Traits, Including Rice Grain Arsenic Concentration and Straighthead Resistance, as Revealed by Genome-Wide Association	2022
4954	Y. F. Ouyang	Modulation of thiol-dependent redox system by metal ions via thioredoxin and glutaredoxin systems	2018
4955	W. J. Liu	Indices of the dual roles of OM as electron donor and complexing compound involved in As and Fe mobilization in aquifer systems of the Datong Basin	2020
4956	M. Wang	Narrow ridge GaSb-based cascade diode lasers fabricated by methane-hydrogen reactive ion etching	2017
4957	J. O. Olowoyo	Health Risk Assessments of Selected Trace Elements and Factors Associated with Their Levels in Human Breast Milk from Pretoria, South Africa	2021
4958	A. L. Popovic	Hybrid amino-terminated lignin microspheres loaded with magnetite and manganese oxide nanoparticles: An effective hazardous oxyanions adsorbent	2022
4959	R. Karra	Synthesis and structural elucidation of some new phenylarsenic(III) derivatives of N(-substituted) S-benzyl dithio carbazates	2000
4960	T. Charette	Assessment of In Vitro Bioaccessibility and In Vivo Oral Bioavailability as Complementary Tools to Better Understand the Effect of Cooking on Methylmercury, Arsenic, and Selenium in Tuna	2021
4961	J. Y. Zhang	Effect of Moisture Control and Air Venting on H2S Production and Leachate Quality in Mature C&D Debris Landfills	2014
4962	H. Long	Separation and recovery of arsenic and alkali products during the treatment of antimony smelting residues	2020
4963	L. Xu	RXR alpha ligand Z-10 induces PML-RARa cleavage and APL cell apoptosis through disrupting PML-RARa/RXRa complex in a cAMP-independent manner	2017
4964	C. W. Liu	Bioaccumulation and Translocation of Arsenic in the Ecosystem of the Guandu Wetland, Taiwan	2014
4965	J. M. McArthur	Groundwater Quality beneath an Asian Megacity on a Delta: Kolkata's (Calcutta's) Disappearing Arsenic and Present Manganese	2018
4966	V. M. O. Carioni	Use of neutron activation analysis and LC-ICP-MS in the development of candidate reference materials for As species determination	2014

4967	T. Masuda	High-sensitivity quantitative analysis reveals the non-linear relationship between the dose and deposition of diphenylarsinic acid in the rat central nervous system following its subchronic exposure	2018
4968	J. Liu	An Important Function of Petrosiol E in Inducing the Differentiation of Neuronal Progenitors and in Protecting Them against Oxidative Stress	2017
4969	Y. H. Wu	A pH-responsive supramolecular draw solute that achieves high-performance in arsenic removal via forward osmosis	2019
4970	S. Thongnok	Mitigation of arsenic toxicity and accumulation in hydroponically grown rice seedlings by co-inoculation with arsenite-oxidizing and cadmium-tolerant bacteria	2018
4971	Z. Es'haghi	Arsenic removal from water/wastewater using nanoparticle-assisted hollow fiber solid-phase microextraction combined with hydride generation-atomic fluorescence spectroscopy	2014
4972	A. M. Canalis	Experimental acute arsenotoxicity in Balb/c mice: organic markers and splenic involvement	2021
4973	S. Yamamura	Effect of extracellular electron shuttles on arsenic-mobilizing activities in soil microbial communities	2018
4974	M. P. Martelli	Arsenic trioxide and all-trans retinoic acid target NPM1 mutant oncoprotein levels and induce apoptosis in NPM1-mutated AML cells	2015
4975	R. Khare	Differential sulphur assimilation mechanism regulates response of Arabidopsis thaliana natural variation towards arsenic stress under limiting sulphur condition	2017
4976	X. Yang	Main Anti-tumor Angiogenesis Agents Isolated From Chinese Herbal Medicines	2015
4977	J. Farasin	Comparison of biofilm formation and motility processes in arsenic-resistant Thiomonas spp. strains revealed divergent response to arsenite	2017
4978	M. Jablonska-Czapla	Arsenic, antimony and chromium speciation using HPLC-ICP-MS in selected river ecosystems of Upper Silesia, Poland - a preliminary study and validation of methodology	2016
4979	A. Giangrande	Heavy metals in five Sabellidae species (Annelida, Polychaeta): ecological implications	2017
4980	T. C. Thounaojam	Transporters: the molecular drivers of arsenic stress tolerance in plants	2021
4981	H. Y. Chen	Dihydroartemisinin Sensitizes Human Lung Adenocarcinoma A549 Cells to Arsenic Trioxide via Apoptosis	2017
4982	L. Wang	Mechanistic insights into red mud, blast furnace slag, or metakaolin-assisted stabilization/solidification of arsenic-contaminated sediment	2019
4983	M. Tuchowska	Organo-Modified Vermiculite: Preparation, Characterization, and Sorption of Arsenic Compounds	2019
4984	C. Zeng	Ecotoxicity assessment of ionic As(III), As(V), In(III) and Ga(III) species potentially released from novel III-V semiconductor materials	2017

4985	W. M. Dai	Asymmetric Wittig reactions of chiral arsonium ylides. Part 2: Atroposelective olefination of axially chiral N,N-dialkyl 2-formyl-1-naphthamides	2001
4986	J. Moseley	Impact of dopant-induced optoelectronic tails on open-circuit voltage in arsenic-doped Cd(Se)Te solar cells	2020
4987	L. Cordeiro	Co-exposure to nTiO(2) impairs arsenic metabolism and affects antioxidant capacity in the marine shrimp Litopenaeus vannamei	2021
4988	E. A. Plis	Dark current reduction in InAs/InAsSb superlattice mid-wave infrared detectors through restoration etch	2015
4989	M. R. Qian	Occurrence of trace elements and antibiotics in manure- based fertilizers from the Zhejiang Province of China	2016
4990	W. R. Williams	Tumour initiation, store-operated calcium entry (SOCE) and apoptosis: cyclic nucleotide dependence	2020
4991	A. Sattar	Metabolism and toxicity of arsenicals in mammals	2016
4992	K. Schafer	Synthesis and structure determinantion of the first lead arsenide phosphide Pb2AsxP14-x (x similar to 3.7)	2016
4993	K. Huang	Glutathione Is Involved in the Reduction of Methylarsenate to Generate Antibiotic Methylarsenite in Enterobacter sp. Strain CZ-1	2022
4994	P. Karimi	Accelerated two-step arsenic photoredox sedimentation in the sequential UV/SO32- reduction and UV/MnO(2)( )oxidation processes	2022
4995	A. Panyushkina	Unraveling the Central Role of Sulfur-Oxidizing Acidiphilium multivorum LMS in Industrial Bioprocessing of Gold-Bearing Sulfide Concentrates	2021
4996	D. Stoiber	Perovskite Distortion Inverted: Crystal Structures of (A(3)N)As (A = Mg, Ca, Sr, Ba)	2019
4997	X. N. Wang	Chinese Medicines in the Treatment of Prostate Cancer: From Formulas to Extracts and Compounds	2018
4998	S. Duangthong	Flow injection-differential pulse anodic stripping voltammetry to measure As(III) and As(V) in natural water samples	2016
4999	W. Zhang	Comparison of Bioavailability and Biotransformation of Inorganic and Organic Arsenic to Two Marine Fish	2016
5000	P. A. Vasilevskii	Organization of high quality lead-antimony shot production	2015
5001	S. Thongnok	AsIII-oxidizing and Cd-tolerant plant growth-promoting bacteria synergistically reduce arsenic translocation, toxicity and accumulation in KDML105 rice	2021
5002	P. S. Ghosal	Adsorptive removal of arsenic by novel iron/olivine composite: Insights into preparation and adsorption process by response surface methodology and artificial neural network	2018

5003	S. Vimalraj	MicroRNAs: Impaired vasculogenesis in metal induced teratogenicity	2017
5004	B. Avula	Arsenic Speciation and Fucoxanthin Analysis from Seaweed Dietary Supplements Using LC-MS	2015
5005	M. Grodzicki	Properties of Bare and Thin-Film-Covered GaN(0001) Surfaces	2021
5006	S. Mishra	Arsenic modified (Ge11.5Te12.5Se67.5)(100-x) compound for IR application	2020
5007	Y. Yabe	Palladium on charcoal-catalyzed ligand-free Stille coupling	2010
5008	S. Sabra	Heavy metals exposure levels and their correlation with different clinical forms of fetal growth restriction	2017
5009	V. H. Flores	Alkaline Decomposition of Solid Solution of Ammonium-Sodium Jarosite with Arsenic	2022
5010	N. M. Monaco	Low arsenic concentrations impair memory in rat offpring exposed during pregnancy and lactation: Role of alpha 7 nicotinic receptor, glutamate and oxidative stress	2018
5011	M. Wang	Exposure to Inorganic Arsenic and Lead and Autism Spectrum Disorder in Children: A Systematic Review and Meta-Analysis	2019
5012	P. M. Loiseau	Synthesis and in vitro anthelmintic properties of some new dithiaarsanes	1999
5013	X. Q. Wang	Enhanced immobilization of arsenic and cadmium in a paddy soil by combined applications of woody peat and Fe(NO3)(3): Possible mechanisms and environmental implications	2019
5014	X. Y. Fan	Oxidative stress-mediated intrinsic apoptosis in human promyelocytic leukemia HL-60 cells induced by organic arsenicals	2016
5015	P. V. Bharatam	Design, Synthesis, and Structural Analysis of Divalent NI Compounds and Identification of a New Electron-Donating Ligand	2016
5016	M. Villalobos	The influence of particle size and structure on the sorption and oxidation behavior of birnessite: I. Adsorption of As(V) and oxidation of As(III)	2014
5017	K. Iuchi	Upregulation of nuclear factor (erythroid-derived 2)-like 2 protein level in the human colorectal adenocarcinoma cell line DLD-1 by a heterocyclic organobismuth(III) compound Effect of organobismuth(III) compound on NRF2 signaling	2020
5018	K. R. Malloch	Comparison of contrasting gold mine processing residues in a temperate rain forest, New Zealand	2017
5019	Q. Peng	Removal of p-arsanilic acid and phenylarsonic acid from water by Fenton coagulation process: influence of substituted amino group	2021
5020	J. Bundschu h	Seven potential sources of arsenic pollution in Latin America and their environmental and health impacts	2021

5021	M. L. Huang	An in situ gold-decorated 3D branched ZnO nanocomposite and its enhanced absorption and photo-oxidation performance for removing arsenic from water	2016
5022	P. A. Gunka	Cascade of High-Pressure Transitions of Claudetite II and the First Polar Phase of Arsenic(III) Oxide	2015
5023	S. G. Ardo	Oxidative Degradation of Nalidixic Acid by Nano-magnetite via Fe2+/O-2-Mediated Reactions	2015
5024	B. Sivarajah	Multiple environmental variables influence diatom assemblages across an arsenic gradient in 33 subarctic lakes near abandoned gold mines	2019
5025	O. Richard	Mechanisms of GaAs surface passivation by a one-step dry process using low-frequency plasma enhanced chemical deposition of silicon nitride	2020
5026	D. A. Rubinos	Acute toxicity of arsenic to Aliivibrio fischeri (Microtox (R) bioassay) as influenced by potential competitive-protective agents	2014
5027	A. S. Pronin	Tetrahedral Rhenium Cluster Complexes with Mixed-Ligand Cores {Re(4)As(3)Q}(5+) (Q = S, Se) and {Re4As2S2}(6+)	2019
5028	M. Zolfaghari	Unwanted metals and hydrophobic contaminants in bioreactor effluents are associated with the presence of humic substances	2017
5029	X. Fan	Simultaneous 3-/4-Hydroxybenzoates Biodegradation and Arsenite Oxidation by Hydrogenophaga sp. H7	2019
5030	X. Y. Li	Performance of Mg-Ti modified iron-based catalyst in NH3-SCR of NO at the presence of arsenic: Influence of oxygen and temperature	2021
5031	W. Tyrra	Silver compounds in synthetic chemistry Part 4. 4-tetrafluoropyridyl silver(I), AgC5F4N in redox transmetallations - possibilities and limitations in reactions with group 15 elements	2006
5032	A. Saxena	Source Mineral for the Release of Arsenic in the Groundwater of Karanda Block, Ghazipur District, Uttar Pradesh	2014
5033	K. Nocon	Soluble Inorganic Arsenic Species in Atmospheric Submicron Particles in Two Polish Urban Background Sites	2020
5034	D. Arslan-Acaroz	In vivo assessment of polydatin, a natural polyphenol compound, on arsenic-induced free radical overproduction, gene expression, and genotoxicity	2018
5035	M. W. Franco	Arsenic biotransformation by cyanobacteria from mining areas: evidences from culture experiments	2015
5036	N. Gonzalez	Concentrations of arsenic and vanadium in environmental and biological samples collected in the neighborhood of petrochemical industries: A review of the scientific literature	2021
5037	B. A. Chalmers	Rhodium(III) and iridium(III) half-sandwich complexes with tertiary arsine and stibine ligands	2015
5038	J. C. Guillemin	Synthesis and characterization of ethylidynearsine	1994

5039	R. Ceaolivares	Synthesis and structural characterization of phenoxarsin-10-yl dithiocarbamates - o(c6h4)(2)ass2cn(ch2ch2)2, a compound containing an asymmetric mononuclear biconnective 1,1-dithiolato ligand	1995
5040	X. Z. Min	Enhancing As(V) and As(III) adsorption performance of low alumina fly ash with ferric citrate modification: Role of FeSiO3 and monosodium citrate	2021
5041	M. Matsumura	Synthesis, Structural Characterization, and Optical Properties of Benzene-Fused Tetracyclic and Pentacyclic Stiboles	2021
5042	W. L. Lu	Simultaneous Biosorption of Arsenic and Cadmium onto Chemically Modified Chlorella vulgaris and Spirulina platensis	2021
5043	T. R. Steel	Metalloproteomics for molecular target identification of protein-binding anticancer metallodrugs	2020
5044	H. Y. Cheng	Coupling electrophoretic separation with inductively coupled plasma spectroscopic detection: interfaces and applications from elemental speciation, metal-ligand interaction to indirect determination	2016
5045	E. Schultze	Synergistic and additive effects of ATRA in combination with different anti-tumor compounds	2018
5046	W. Y. Jin	Tannic acid ameliorates arsenic trioxide-induced nephrotoxicity, contribution of NF-kappa B and Nrf2 pathways	2020
5047	C. Escudero-Lourdes	Toxicity mechanisms of arsenic that are shared with neurodegenerative diseases and cognitive impairment: Role of oxidative stress and inflammatory responses	2016
5048	Y. W. Tian	Arsenic Sulfide Promotes Apoptosis in Retinoid Acid Resistant Human Acute Promyelocytic Leukemic NB4-R1 Cells through Downregulation of SET Protein	2014
5049	N. Maheshwari	3,4-Dihydroxybenzaldehyde lowers ROS generation and protects human red blood cells from arsenic(III) induced oxidative damage	2018
5050	H. P. Hao	Insights into drug discovery from natural medicines using reverse pharmacokinetics	2014
5051	M. Baumgartner	Inorganic SnIP-Type Double Helices in Main-Group Chemistry	2017
5052	V. Heinl	E-4 Transfer (E=P, As) to Ni Complexes	2021
5053	Y. Y. Xu	Efficient removal of low-concentration organoarsenic by Zr-based metal-organic frameworks: cooperation of defects and hydrogen bonds	2019
5054	A. E. Seitz	Pnictogen-Silicon Analogues of Benzene	2016
5055	N. Maheshwari	Sodium meta-arsenite induced reactive oxygen species in human red blood cells: impaired antioxidant and membrane redox systems, haemoglobin oxidation, and morphological changes	2017
5056	G. M. Darone	On the New Oxyarsenides Eu5Zn2As5O and Eu5Cd2As5O	2020

5057	P. Colomban	Non-invasive on-site Raman study of polychrome and white enamelled glass artefacts in imitation of porcelain assigned to Bernard Perrot and his followers	2020
5058	A. L. Huang	Honokiol attenuate the arsenic trioxide-induced cardiotoxicity by reducing the myocardial apoptosis	2022
5059	D. Thilakan	Plant-Derived Iron Nanoparticles for Removal of Heavy Metals	2022
5060	P. Manduca	Long Term Risks to Neonatal Health from Exposure to War-9 Years Long Survey of Reproductive Health and Contamination by Weapon-Delivered Heavy Metals in Gaza, Palestine	2020
5061	T. P. Joshi	The removal efficiency and insight into the mechanism of para arsanilic acid adsorption on Fe-Mn framework	2017
5062	L. Castro	Heavy metal adsorption using biogenic iron compounds	2018
5063	G. Sanchez-Sanz	Aromatic behaviour of benzene and naphthalene upon pnictogen substitution	2015
5064	H. Y. Guo	The synthesis and characterization of three organic-inorganic hybrids based on different transition metal complexes and {As8V14O42(H2O)} clusters	2014
5065	A. Escalante-Mane	Enhanced removal of persistent contaminants and toxicity reduction through the application of a triple-stage fenton process to sanitary landfill leachates from yucatan, mexico	
5066	C. E. Enyoh	An overview of emerging pollutants in air: Method of analysis and potential public health concern from human environmental exposure	2020
5067	H. Pfau	Low work function in the 122-family of iron-based superconductors	2020
5068	T. Lee	Fabrication of carbon-slag composite via a pyrolytic platform and its environmental application for arsenic removal as a case study	2019
5069	V. Grabez	Seaweed Inclusion in Finishing Lamb Diet Promotes Changes in Micronutrient Content and Flavour-Related Compounds of Raw Meat and Dry-Cured Leg (Fenalar)	2022
5070	M. V. Russo	Halogenated Volatile Organic Compounds in Water Samples and Inorganic Elements Levels in Ores for Characterizing a High Anthropogenic Polluted Area in the Northern Latium Region (Italy)	2021
5071	A. Wikedzi	Recovery of antimony compounds from alkaline sulphide leachates	2016
5072	A. Hinz	The 2-Arsaethynolate Anion: Synthesis and Reactivity Towards Heteroallenes	2016
5073	R. Eujen	Donor-free bis(trifluoromethyl)cadmium, (cf3)(2)cd - a readily available low-temperature difluorocarbene source	1995
5074	E. Fasano	Evaluation of the impact of anthropogenic activities on arsenic, cadmium, chromium, mercury, lead, and polycyclic aromatic hydrocarbon levels in seafood from the Gulf of Naples, Italy	2018

5075	S. Pal	Time evolution studies of laser induced chemical changes in InAs nanowire using Raman spectroscopy	2014
5076	A. Garcia-Fuente	Tunable gap in stable arsenene nanoribbons opens the door to electronic applications	2019
5077	R. G. Hamza	Ameliorating Effect of Gamma-irradiated Moringa Leaves Against Arsenic-Induced Oxidative Damage in Rat Liver	2016
5078	M. L. Coghlan	Combined DNA, toxicological and heavy metal analyses provides an auditing toolkit to improve pharmacovigilance of traditional Chinese medicine (TCM)	2015
5079	H. T. Teunissen	Reactivity of organozinc derivatives of phosphinines	1996
5080	J. B. Bell	Differential Response of Glioma Stem Cells to Arsenic Trioxide Therapy Is Regulated by MNK1 and mRNA Translation	2018
5081	G. Kerr	Authigenic realgar and gold in dynamic redox gradients developed on historic mine wastes, New Zealand	2018
5082	S. G. Henao	Heavy Metals in Soils and the Remediation Potential of Bacteria Associated With the Plant Microbiome	2021
5083	D. Valli	Has Drug Repurposing Fulfilled Its Promise in Acute Myeloid Leukaemia?	2020
5084	M. Uchida	Characterization of fermented seaweed sauce prepared from nori (Pyropia yezoensis)	2017
5085	P. T. Wang	Arsenate induced chlorosis 1/translocon at the outer envelope membrane of chloroplasts 132 protects chloroplasts from arsenic toxicity	2018
5086	P. Song	Realgar transforming solution displays anticancer potential against human hepatocellular carcinoma HepG2 cells by inducing ROS	2017
5087	H. Figueiredo	Tailored zeolites for the removal of metal oxyanions: Overcoming intrinsic limitations of zeolites	2014
5088	S. Kim	Mitigating translocation of arsenic from rice field to soil pore solution by manipulating the redox conditions	2021
5089	J. Borovicka	Speciation analysis of elements accumulated in Cystoderma carcharias from clean and smelter-polluted sites	2019
5090	K. Ezoe	Automated determinations of selenium in thermal power plant wastewater by sequential hydride generation and chemiluminescence detection	2016
5091	E. Pagliano	Application of direct analysis in real time to a multiphase chemical system: Identification of polymeric arsanes generated by reduction of monomethylarsenate with sodium tetrahydroborate	2014
5092	T. Dordevic	Mineralogy and weathering of realgar-rich tailings at a former as-sb-cr mine at lojane, north macedonia	2019

5093	D. Broka	Methylation of inorganic arsenic by murine fetal tissue explants	2016
5094	H. P. Xiao	Study on the Volatilization Behavior of Heavy Metals (As, Cd) During Co-Processing in Furnaces and Boilers	2017
5095	Z. Gajdosechova	CRM rapid response approach for the certification of arsenic species and toxic trace elements in baby cereal coarse rice flour certified reference material BARI-1	2020
5096	U. Arroyo-Abad	Synthesis of two new arsenolipids and their identification in fish	2016
5097	A. Spinazze	Occupational Exposure to Arsenic and Cadmium in Thin-Film Solar Cell Production	2015
5098	J. Ermert	Labelling with positron emitters of pnicogens and chalcogens	2018
5099	J. H. Chen	A structural survey of the binary transition metal phosphides and arsenides of the d-block elements	2018
5100	E. Chupakhin	Thioredoxin reductase inhibitors: updated patent review (2017-present)	2021
5101	M. S. Rahman	Deciphering the origin of Cu, Pb and Zn contamination in school dust and soil of Dhaka, a megacity in Bangladesh	2021
5102	S. Trivedi	Epidemiology of Peripheral Neuropathy: An Indian Perspective	2017
5103	A. Monroy-Licht	Effect of phosphate on arsenic species uptake in plants under hydroponic conditions	
5104	I. Amigo-Jimenez	Gene expression profile induced by arsenic trioxide in chronic lymphocytic leukemia cells reveals a central role for heme oxygenase-1 in apoptosis and regulation of matrix metalloproteinase-9	2016
5105	M. Hosseinzadehdehkor	Dual effect of curcumin targets reactive oxygen species, adenosine triphosphate contents and intermediate steps of mitochondria-mediated apoptosis in lung cancer cell lines	2015
5106	M. E. Vezza	Glutathione, a key compound for As accumulation and tolerance in soybean plants treated with AsV and AsIII	2019
5107	A. Hosseinzadeh	Ameliorative effect of gallic acid on sodium arsenite-induced spleno-, cardio- and hemato-toxicity in rats	2019
5108	S. Islama	Arsenic accumulation in rice: Consequences of rice genotypes and management practices to reduce human health risk	2016
5109	S. Q. Liu	Removal of dimethylarsinic acid (DMA) in the Fe/C system: roles of Fe(II) release, DMA/Fe(II) and DMA/Fe(III) complexation	2022
5110	Y. Chen	The role of nodes in arsenic storage and distribution in rice	2015

5111	N. Marwa	Effect of rhizospheric inoculation of isolated arsenic (As) tolerant strains on growth, As-uptake and bacterial communities in association with Adiantum capillus-veneris	2020
5112	M. Z. Alam	Effect of Arbuscular Mycorrhizal Fungi, Selenium and Biochar on Photosynthetic Pigments and Antioxidant Enzyme Activity Under Arsenic Stress in Mung Bean (Vigna radiata)	2019
5113	J. X. Gao	Investigating the Roles of Dissolved Organic Matter on Arsenic Mobilization and Speciation in Environmental Water	2016
5114	M. M. Liu	Exploration of the hepatoprotective effect and mechanism of magnesium isoglycyrrhizinate in mice with arsenic trioxide-induced acute liver injury	2021
5115	F. C. Chang	A combined analytical method for biological monitoring of arsenic, benzene and polycyclic aromatic hydrocarbons in human urine by liquid chromatography tandem mass spectrometry	2019
5116	X. Q. Sun	Investigation of the ameliorative effects of baicalin against arsenic trioxide-induced cardiac toxicity in mice	2021
5117	R. Saeed ur	The ameliorative effects of exogenous inoculation of Piriformospora indica on molecular, biochemical and physiological parameters of Artemisia annua L. under arsenic stress condition	2020
5118	M. Raghav	Understanding abiotic ferrihydrite re-mineralization by ferrous ions	2015
5119	Y. K. Li	Eco-utilization of steel slag: Preparation of Fe-based calcium silicate hydrate and its application in As(V) removal	2022
5120	H. Z. Lin	Variation in composition and relative content of accumulated photopigments in a newly isolated Rhodobacter capsulatus strain XJ-1 in response to arsenic	2014
5121	A. M. Orlov	Kinetic features of the oxide formation on {111} polar planes upon anode treatment of n-GaAs	2016
5122	A. Haghi	Effects of Sorafenib and Arsenic Trioxide on u937 and KG-1 Cell Lines: Apoptosis or Autophagy?	2020
5123	F. Bruck	Overestimation of geogenic arsenic in landfill leachate: interference of humic and phosphinic acid in a commercial test kit	2014
5124	X. Luan	Compound-based Chinese medicine formula: From discovery to compatibility mechanism	2020
5125	I. Amigo-Jimenez	Bone marrow stroma-induced resistance of chronic lymphocytic leukemia cells to arsenic trioxide involves Mcl-1 upregulation and is overcome by inhibiting the PI3K delta or PKC beta signaling pathways	2015
5126	W. Li	Icariin Synergizes with Arsenic Trioxide to Suppress Human Hepatocellular Carcinoma	2014
5127	A. V. Oppenheimer	Prenatal Exposure to Chemical Mixtures and Cognitive Flexibility among Adolescents	2021
5128	M. Zhu	Sorption mechanisms of diphenylarsinic acid on ferrihydrite, goethite and hematite using sequential extraction, FTIR measurement and XAFS spectroscopy	2019

5129	P. V. Muhasina	Gallium Ligand Coordinated Group 15 Compounds (LGa-ECp', L=(CHNMe)(2)CH, E=N - Bi, Cp'=eta(1)-C5H5): Changeover from Electron-Sharing to Donor-Acceptor sigma-Interaction	2021
5130	Z. H. Lin	CTAB-functionalized delta-FeOOH for the simultaneous removal of arsenate and phenylarsonic acid in phenylarsenic chemical warfare	2022
5131	A. El-Naggar	Mechanistic insights into the (im)mobilization of arsenic, cadmium, lead, and zinc in a multi-contaminated soil treated with different biochars	2021
5132	M. H. Saleem	Silicon Enhances Morpho-Physio-Biochemical Responses in Arsenic Stressed Spinach (Spinacia oleracea L.) by Minimizing Its Uptake	
5133	C. Schwarzmaier	Selective Formation and Unusual Reactivity of Tetraarsabicyclo- 1.1.0 butane Complexes	2014
5134	U. Qumar	Photocatalysis vs adsorption by metal oxide nanoparticles	2022
5135	H. Palma-Gudiel	The impact of prenatal insults on the human placental epigenome: A systematic review	2018
5136	S. Sainas	Targeting Acute Myelogenous Leukemia Using Potent Human Dihydroorotate Dehydrogenase Inhibitors Based on the 2-Hydroxypyrazolo 1,5-a pyridine Scaffold: SAR of the Biphenyl Moiety	2021
5137	C. Bao	Spatial and temporal distribution of toxic compounds in sediments and potential ecological effects on macrobenthic faunal species in Hangzhou Bay from 2003 to 2015	2021
5138	A. El-bouazzaoui	Alternative flotation collectors for the environmental desulfurization of gersdorffite (NiAsS) bearing mine tailings: Surface chemistry	2022
5139	M. A. Shenashen	Bushy sphere dendrites with husk-shaped branches axially spreading out from the core for photo-catalytic oxidation/remediation of toxins	2017
5140	B. T. Doherty	Chemical exposures assessed via silicone wristbands and endogenous plasma metabolomics during pregnancy	2022
5141	M. Z. Bani-Fwaz	Synthesis, crystal structures, and quantum chemical calculations of trialkyl-substituted 1 lambda(3),3 lambda(3),5 lambda(3)-triphospha dewarbenzenes	2017
5142	W. C. Tang	Halogenated arsenenes as Dirac materials	2016
5143	N. A. Kolpakova	Problems of choosing metal-modifiers for graphite electrodes used in inversion voltammetry	2017
5144	T. Bito	Bioactive Compounds of Edible Purple Laver Porphyra sp (Nori)	2017
5145	W. R. Meier	Optimization of the crystal growth of the superconductor CaKFe4As4 from solution in the FeAs-CaFe2As2-KFe2As2 system	2017
5146	L. J. Jones	Isolation and characterization of an indium mixed-pnicogen 4-membered-ring compound - crystal-structure of (me3sich2)2inas(sime3)2in(ch2sime3)2p(sime3)2	1994

5147	O. Hegen	Bidentate Phosphanyl- and Arsanylboranes	2019
5148	D. E. Schipper	Iron carbonyl clusters with ECl2 units (E = P, As)	2017
5149	U. Krieger-Ballhausen	Trisenox (R) (Arsenic Trioxide) receives Approval for First-Line Therapy in acute Promyelocytic Leukemia	2017
5150	R. L. Liu	Residential Exposure to Estrogen Disrupting Hazardous Air Pollutants and Breast Cancer Risk The California Teachers Study	2015
5151	C. Y. Liang	Overview of all-trans-retinoic acid (ATRA) and its analogues: Structures, activities, and mechanisms in acute promyelocytic leukaemia	2021
5152	A. Gyepes	Synthesis and chromatographic study of methyl-2,3-O-isopropylidene-5-dimethyl-arsinoyl-beta-D-ribofuranoside and methyl-2,3-O-isopropylidene-5-deoxy-5-dimethyl-thioarsinoyl-beta-D-ribof uranoside	2008
5153	C. Y. Wang	A new one-dimensional coordination polymer synthesized from zinc and guanazole: Superior capture of organic arsenics	2020
5154	A. S. Pronin	Cation effect on the structure of tetrahedral rhenium arsenide cyano clusters	2020
5155	V. Vromman	Risk ranking priority of carcinogenic and/or genotoxic environmental contaminants in food in Belgium	2014
5156	C. Klink	Investigation of Acidithiobacillus ferrooxidans in pure and mixed-species culture for bioleaching of Theisen sludge from former copper smelting	2016
5157	M. S. McCann	Environmental toxicants in the brain: A review of astrocytic metabolic dysfunction	2021
5158	H. Brunner	Optically active transition-metal complexes Part 124. Chiral 1-phospha 1 ferrocenophanes and 1,12-diphospha 1.1 ferrocenophanes - synthesis, characterization and ring-opening polymerization	2000
5159	P. Jutzi	Synthesis and characterization of new cyclopentadienylarsenic compounds	1998
5160	O. V. Nipruk	Chemical stability of rare-earth elements' uranyl arsenates with general formula M-III(AsUO6)(3)center dot 16H(2)O (M-III-La-Lu) in aqueous solution	2021
5161	E. Fahrenkrug	Electrochemically Gated Alloy Formation of Crystalline InAs Thin Films at Room Temperature in Aqueous Electrolytes	2014
5162	N. Burford	Synthesis and characterization of an homologous series of bis(amido)diazadipnictetidines (Pnict = P, As, Sb, Bi)	2001
5163	B. Arikan	Exogenous hesperidin and chlorogenic acid alleviate oxidative damage induced by arsenic toxicity in Zea mays through regulating the water status, antioxidant capacity, redox balance and fatty acid composition	2022
5164	S. Mirfakhraie	Protective effect of folic acid against arsenic induced weight loss during gestation, lactation and postnatal growth in mice	2018

5165	V. M. Mikoushkin	Composition and Band Structure of the Native Oxide Nanolayer on the Ion Beam Treated Surface of the GaAs Wafer	2018
5166	L. Mielczarek	Combinations of isothiocyanates with drugs - a chance or threat to chemoprevention and cancer treatment?	2018
5167	V. M. Cangelosi	A Surprising "Folded-In" Conformation of a Self-Assembled Arsenic-Thiolate Macrocycle	2010
5168	A. Kasiuliene	Hydrothermal carbonisation of peat-based spent sorbents loaded with metal(loid)s	2019
5169	A. Kato	Pharmacodynamics of S-dimethylarsino-glutathione, a putative metabolic intermediate of inorganic arsenic, in mice	2017
5170	V. Crescente	Identification of novel members of the bacterial azoreductase family in Pseudomonas aeruginosa	2016
5171	P. R. Hunt	C-elegans Development and Activity Test detects mammalian developmental neurotoxins	2018
5172	M. J. Huang	Atmospheric Arsenic Deposition in the Pearl River Delta Region, South China: Influencing Factors and Speciation	2018
5173	S. R. Woo	KML001, a Telomere-Targeting Drug, Sensitizes Glioblastoma Cells to Temozolomide Chemotherapy and Radiotherapy through DNA Damage and Apoptosis	2014
5174	J. M. Galloway	Organic matter control on the distribution of arsenic in lake sediments impacted by similar to 65 years of gold ore processing in subarctic Canada	2018
5175	Y. Zhou	Heavy metal-induced lipogenic gene aberration, lipid dysregulation and obesogenic effect: a review	2022
5176	V. Heinl	Coordination Behavior of Cp " Zr-2(mu(1:1)-As-4) towards Lewis Acids	2021
5177	M. Poor	Effects of phosphate binders on the gastrointestinal absorption of arsenate and of an SGLT2 inhibitor drug on the urinary excretion of arsenite in mice	2017
5178	P. A. Alekseev	Observing visible-range photoluminescence in GaAs nanowires modified by laser irradiation	2017
5179	H. S. Choi	Different degradation mechanism by conduction region in AsTeGeSiN threshold switching device	2020
5180	M. K. Bamgbose	Electronic structure and thermoelectric properties of HfRhZ(Z = As, Sb and Bi) half-Heusler compounds	2020
5181	E. J. Itumoh	Influence of mining and agricultural activities on the quality of groundwater from some rural areas of south-eastern nigeria	2015
5182	J. Fanfrlik	The Interplay between Various sigma- and pi-Hole Interactions of Trigonal Boron and Trigonal Pyramidal Arsenic Triiodides	2017

5183	A. Kumari	Synthesis, characterization, antimicrobial and DNA cleavage study of organoantimony(III) and organoarsenic(III) complexes with monofunctional bidentate Schiff base	2021
5184	M. T. Rahman	Origin, Function, and Fate of Metallothionein in Human Blood	2017
5185	V. Bermanec	Novel arsenic hyper-resistant bacteria from an extreme environment, Crven Dol mine, Allchar, North Macedonia	2021
5186	F. H. Reboredo	Elemental Composition of Algae-Based Supplements by Energy Dispersive X-ray Fluorescence	2021
5187	C. Nunez	Determination of arsenic in the presence of copper by adsorptive stripping voltammetry using pyrrolidine dithiocarbamate or diethyl dithiophosphate as chelating-adsorbent agents. Effect of CPS on the sensitivity of the method	2016
5188	M. Stiboller	Simultaneous determination of glycine betaine and arsenobetaine in biological samples by HPLC/ICPMS/ESMS and the application to some marine and freshwater fish samples	2015
5189	R. R. Samelo	Low concentrations of sodium arsenite induce hepatotoxicity in prepubertal male rats	2020
5190	V. Kopackov a	Assessing forest health via linking the geochemical properties of a soil profile with the biochemical parameters of vegetation	2015
5191	E. Revesz	Reductive dissolution of scorodite in the presence of Shewanella sp CN32 and Shewanella sp ANA-3	2015
5192	P. Hajeb	Toxic Elements in Food: Occurrence, Binding, and Reduction Approaches	2014
5193	S. Chouchen e	Structural and dielectric properties of a new cesium-rubidium dihydrogen arsenate: Cs0.2Rb0.8H2AsO4	2017
5194	R. L. Wells	Synthesis, isolation, and characterization of dimeric compounds containing 2 different exocyclic ligands on the group-13 center - x-ray crystal-structures of r(me3sich2)ine(sime3)2 2 (r = ph, e = as r = me, e = as, p r = cl, e = p)	1993
5195	J. Clark	Pre-pregnancy BMI-associated miRNA and mRNA expression signatures in the placenta highlight a sexually-dimorphic response to maternal underweight status	2021
5196	A. Ektarawon g	First-principles prediction of stabilities and instabilities of compounds and alloys in the ternary B-As-P system	2017
5197	M. Mac Monagail	Quantification and feed to food transfer of total and inorganic arsenic from a commercial seaweed feed	2018
5198	I. C. Yadav	Reductive dissolution of iron-oxyhydroxides directs groundwater arsenic mobilization in the upstream of Ganges River basin, Nepal	2015
5199	L. Zhan	Novel Recycle Technology for Recovering Gallium Arsenide from Scraped Integrated Circuits	2020
5200	E. J. M. Joy	Elemental composition of Malawian rice	2017

5201	M. X. Zhang	The role of interaction between low molecular weight neutral organic compounds and a polyamide RO membrane in the rejection mechanism	2020
5202	H. G. Ang	Preparation and properties of CF3As ON(H)CF3 (2) and (CF3)(2)AsON(H)CF3	1996
5203	C. S. Sharma	Dialkyl(aryl) phosphoryl derivatives of alkylene dithiophosphates	2004
5204	C. Autieri	Low energy bands and transport properties of chromium arsenide	2017
5205	O. V. Nipruk	State of uranyl arsenates (MAsUO6)-As-I center dot nH(2)O (M-I-H+, Li+, Na+, K+, Rb+, Cs+, NH4+) in aqueous solution	2020
5206	W. T. K. Chan	Syntheses and structures of Me2Si{As((PBu)-Bu-t)(3)}(2) and (CyP)(3)SiMe2 (Cy = cyclohexyl, C6H11)	2010
5207	M. B. Liu	Experimental study on treatment of heavy metal-contaminated soil by manganese-oxidizing bacteria	2022
5208	A. J. A. Ranjitsingh	Antiviral and cytotoxic effects of a traditional drug KanthaRasaVillai with a cocktail of metallic nanoparticles	2022
5209	W. A. Al-Megrin	Nephroprotective effects of chlorogenic acid against sodium arsenite-induced oxidative stress, inflammation, and apoptosis	2020
5210	D. Geiss	Si=P Double Bonds: Experimental and Theoretical Study of an NHCStabilized Phosphasilenylidene	2015
5211	I. Tissot	Unveiling the art of Rene Lalique with XRF and Raman spectroscopy - Technological innovation in jewellery production	2018
5212	M. Vitkova	Metal(loid)s behaviour in soils amended with nano zero-valent iron as a function of pH and time	2017
5213	A. Miri	Study of photodegradation performance and ability of lead removal of green synthesised maghemite nanoparticles	
5214	S. H. Lee	Enhanced adsorption of arsenate and antimonate by calcined Mg/Al layered double hydroxide: Investigation of comparative adsorption Check for mechanism by surface characterization	2018
5215	M. Czaplicka	Photo-oxidation of p-arsanilic acid in acidic solutions: Kinetics and the identification of by-products and reaction pathways	2014
5216	A. L. Bandini	Farther study on the reactivity of cationic dinuclear platinum trihydrido compounds with unsaturated hydrocarbons: Synthesis of mu-hydrido mu-alkylidene complexes, NMR characterization and mechanism insights	2015
5217	L. R. Terry	Microbiological Oxidation of Antimony(III) with Oxygen or Nitrate by Bacteria Isolated from Contaminated Mine Sediments	2015
5218	D. E. Schipper	Transformations in Transition-Metal Carbonyls Containing Arsenic: Exploring the Chemistry of Et4N (2) HAs{Fe(CO)(4)}(3) in the Search for Single-Source Precursors for Advanced Metal Pnictide Materials	2016

5219	P. Nookongbut	Arsenic contamination in areas surrounding mines and selection of potential As-resistant purple nonsulfur bacteria for use in bioremediation based on their detoxification mechanisms	2016
5220	M. Liu	Release of volatile organic compounds (VOCs) from colorectal cancer cell line LS174T	2019
5221	S. C. Fu	Arsenic induces autophagy-dependent apoptosis via Akt inactivation and AMPK activation signaling pathways leading to neuronal cell death	2021
5222	J. L. Hobman	Bacterial antimicrobial metal ion resistance	2015
5223	Z. Chen	The Role of Low-Molecular-Weight Organic Carbons in Facilitating the Mobilization and Biotransformation of As(V)/Fe(III) from a Realgar Tailing Mine Soil	2018
5224	R. Khare	Genome-wide expression and variation in nucleotide sequences lead to differential response of Arabidopsis thaliana ecotypes towards arsenic stress under sulfur limiting condition	2022
5225	S. Z. Chen	Use of fibrous TiO2@graphitic carbon nitride nanocomposites in dispersive micro-solid phase extraction for arsenic species before inductively coupled plasma mass spectrometry determination	2020
5226	F. R. Li	A High-Symmetrical 3D Pure Inorganic Photocatalyst Based on the Highest Connectivity of {AsW12O40} Heteropoly Blue and Potassium Ions	2018
5227	V. Stepanova	Laser ablation generation of clusters from As-Te mixtures, As-Te glass nano-layers and from Au-As-Te nano-composites. Quadrupole ion trap time-of-flight mass spectrometry	2015
5228	H. Bose	Impact of arsenic on microbial community structure and their metabolic potential from rice soils of West Bengal, India	2022
5229	Y. T. Feng	A trithiol bifunctional chelate for (72,77)AS : A matched pair theranostic complex with high in vivo stability	2018
5230	S. Feng	Limited roles of anthropogenic activities on arsenic mobilization in groundwater from the Yinchuan Basin, China	2022
5231	S. M. Nunes	Impact of different crystalline forms of nTiO 2 on metabolism and arsenic toxicity in Limnoperna fortunei	2020
5232	N. S. Randhawa	Iron oxide waste to clean arsenic-contaminated water	2014
5233	P. A. Lamela	Analysis of occurrence, bioaccumulation and molecular targets of arsenic and other selected volcanic elements in Argentinean Patagonia and Antarctic ecosystems	2019
5234	D. Ghereg	Versatile 1,2- and 1,3-Dipole Behavior of the 1-Arsa-3-Germaallene Tip(t-Bu)Ge=C=AsMes*: Formation of a Heterocyclic Arsa(germa)carbene (AsGeHC)	2012
5235	J. Kowalska	The role of phytochelatins in Sinapis alba L. response to stress caused by two toxic elements As and Tl	2015
5236	F. Parada	Leaching kinetics of enargite in alkaline sodium sulphide solutions	2014

5237	C. Schwarzmaier	E-4 Butterfly Complexes (E=P, As) as Chelating Ligands	2015
5238	L. H. He	Study on the interaction mechanism in the Pr-Fe-As system	2020
5239	W. Keller	Syntheses and Crystal Structures of Brominated Polyhedral Arsa- and Phosphaboranes	2017
5240	B. Vriens	Quantification of Methylated Selenium, Sulfur, and Arsenic in the Environment	2014
5241	A. S. Makarova	Induced Phytoextraction of Mercury	2022
5242	J. Song	Enhanced adsorption of roxarsone onto humic acid modified goethite from aqueous solution	2019
5243	N. Reinfandt	From a nanoparticular solid-state material to molecular organo-f-element-polyarsenides	2022
5244	S. Hu	Groundwater Arsenic Adsorption on Granular TiO2: Integrating Atomic Structure, Filtration, and Health Impact	2015
5245	C. Heindl	Progress in Polyarsolyl Chemistry	2016
5246	C. H. Zhou	Numerical simulation of pollutant transport in soils surrounding subway infrastructure	2018
5247	G. M. Tsivgoulis	The Reaction of Diethylthiophosphinyl Iodide, Et2P(S)I, with Nucleophiles	2017
5248	L. Chen	A computational method for the identification of new candidate carcinogenic and non-carcinogenic chemicals	2015
5249	M. Niinipuu	Influence of water matrix and hydrochar properties on removal of organic and inorganic contaminants	2020
5250	X. J. Feng	Zintl-Phase Sr3LiAs2H: Crystal Structure and Chemical Bonding Analysis by the Electron Localizability Approach	2015
5251	M. A. Limmer	The role of small molecules in restricting rice accumulation of dimethylarsinic acid	2020
5252	M. Khandaker	Trichosanthes dioica Roxb.: A vegetable with diverse pharmacological properties	2018
5253	U. Ribic	Transporters and Efflux Pumps Are the Main Mechanisms Involved in Staphylococcus epidermidis Adaptation and Tolerance to Didecyldimethylammonium Chloride	2020
5254	M. Hyla	Network-Forming Nanoclusters in Binary As-S/Se Glasses: From Ab Initio Quantum Chemical Modeling to Experimental Evidences	2017

5255	L. Ansone-Bertina	Immobilised Humic Substances as Low-Cost Sorbents for Emerging Contaminants	2021
5256	S. Khullar	Cadmium and arsenic responses in the ectomycorrhizal fungus Laccaria bicolor: glutathione metabolism and its role in metal(loid) homeostasis	2019
5257	J. Muse	An Overview of Capillary Electrophoresis In Element Speciation Analysis of the Environment	2014
5258	F. Wieronska -	Assessment of the Applicability of Ca-Based Sorbents for Arsenic Removal from Flue Gases	
5259	I. C. A. Ribeiro	Fast and effective arsenic removal from aqueous solutions by a novel low-cost eggshell byproduct	2021
5260	Y. Saitoh	Combination of Hedgehog inhibitors and standard anticancer agents synergistically prevent osteosarcoma growth	2016
5261	A. C. Mandigo	Chemical contamination of soils in the New York City area following Hurricane Sandy	2016
5262	G. W. Chen	Shewanella oneidensis MR-1-Induced Fe(III) Reduction Facilitates Roxarsone Transformation	2016
5263	P. Zhang	Discovery of Novel Benzothiazepinones as Irreversible Covalent Glycogen Synthase Kinase 3 beta Inhibitors for the Treatment of Acute Promyelocytic Leukemia	2021
5264	T. Dordevic	Three new Sr-bearing arsenates, hydrothermally synthesized in the system SrO-MO-As2O5-H2O (M2+ = Mg, Cu, Zn)	2018
5265	C. Bulutay	Nuclear magnetic resonance inverse spectra of InGaAs quantum dots: Atomistic level structural information	2014
5266	V. Llabjani	Bimodal responses of cells to trace elements: Insights into their mechanism of action using a biospectroscopy approach	2014
5267	A. Hildebrand	Aurophilic interaction leads to distortion of the ten-membered centrosymmetric Au2As2S2C4 ring in Au-2{mu-(SC6H4-2-AsPh2)-kappa S,kappa As}(2)	2022
5268	B. B. Rajs	Characterization of Croatian Rape (Brassica sp.) Honey by Pollen Spectrum, Physicochemical Characteristics, and Multielement analysis by ICP-OES	2017
5269	R. L. Wells	Synthesis and characterization of novel organogallium phosphorus-compounds - x-ray crystal-structures of ph2gap(sime3)2ga(ph)2cl, ph2(cl)ga.p(sime3)3, and ph3ga.p(sime3)3	1992
5270	S. Kumar	TRISENOX disrupts MDM2-DAXX-HAUSP interaction and increased promyelocytes formation in murine model of APL	2016
5271	L. Mochalov	Structural and optical properties of As-Se-Te chalcogenide films prepared by plasma-enhanced chemical vapor deposition	2019
5272	L. E. Pracht	Molecular characterization of organic matter mobilized from Bangladeshi aquifer sediment: tracking carbon compositional change during microbial utilization	2018

5273	E. Varghese	Auranofin, an Anti-Rheumatic Gold Compound, Modulates Apoptosis by Elevating the Intracellular Calcium Concentration (Ca2+ i) in MCF-7 Breast Cancer Cells	2014
5274	K. F. Quader	Pressure-Driven Enthalpic and Lifshitz Transition in 122-Pnictides	2015
5275	V. A. Fedorov	Physicochemical and Methodological Approaches to the Development of Integrated Processes for the Preparation of High-Purity Substances	2019
5276	J. H. Muessig	Oxidative addition of arsenic halides to platinum(0)	2019
5277	J. Vrana	N -> As intramolecularly coordinated organoarsenic(III) chalcogenides: Isolation of terminal As-S and As-Se bonds	2013
5278	M. Mohammadzadeh	Determination of potentially toxic metals in depilatory products in the Iranian markets: human health risk assessment	2022
5279	S. C. Chmely	Influence of Ring Methylation in Group 15 Tetramethylcyclopentadienyl Complexes, M(C5Me4H)(n)I3-n (M = As, Sb)	2010
5280	H. Zeng	Exposure to barium and blood pressure in children and adolescents: results from the 2003-2018 National Health and Nutrition Examination Survey	
5281	I. Lyakhova	Alkaloids of fascaplysin are promising chemotherapeutic agents for the treatment of glioblastoma: Review	2020
5282	J. L. Bentz	Realgar and hornesite precipitation in an iron-poor, sulfate-rich mudflat, laguna chiar khota, bolivia	2017
5283	C. M. Deus	Targeting mitochondrial function for the treatment of breast cancer	2014
5284	E. M. Dionisi	Triphosphenium salts: air-stable precursors for phosphorus(I) chemistry	2020
5285	N. Bilici	Blood and Stool Arsenic Levels Are Decisive for Diagnosing Children's Functional Gastrointestinal Disease (FGD)	2022
5286	C. U. Msoffe	The sources and chemical content of edible soil sticks sold in markets in Tanzania: a cross-sectional analytical study	2019
5287	M. Piesch	Redox Chemistry of Heterobimetallic Polypnictogen Triple-Decker Complexes - Rearrangement, Fragmentation and Transfer	2021
5288	S. Andleeb	Transplacental hepato-curative potential of garlic against sodium arsenate induced oxidative stress in mice	2021
5289	J. C. Lee	Synergistic effects of the combination of oxalate and ascorbate on arsenic extraction from contaminated soils	2017
5290	B. Yuan	Effects of active bufadienolide compounds on human cancer cells and CD4(+)CD25(+)Foxp3(+) regulatory T cells in mitogen-activated human peripheral blood mononuclear cells	2016

5291	J. A. Sans	Arsenolite: a quasi-hydrostatic solid pressure-transmitting medium (vol 28, 475403, 2016)	2016
5292	S. M. Rahman	Manganese exposure through drinking water during pregnancy and size at birth: A prospective cohort study	2015
5293	M. Naeem	Carrageenan oligomers and salicylic acid act in tandem to escalate artemisinin production by suppressing arsenic uptake and oxidative stress in Artemisia annua (sweet wormwood) cultivated in high arsenic soil	2021
5294	I. E. Akyildi	Identification of the rice syrup adulterated honey by introducing a candidate marker compound for Brown rice syrups	2022
5295	S. V. Serves	Reaction of arsenic(III) oxide, arsenous and arsenic acids with thiols	1995
5296	F. M. Uckun	Phenylarsonic acid compounds with broad-spectrum and potent cytotoxic activity against human cancer cells	2003
5297	F. X. Pan	Tetrahedral Sb(AuMe)(4) (3-) Occurring in Multimetallic Cluster Syntheses: About the Structure-Directing Role of Methyl Groups	2021
5298	T. G. Do	Aurophilicity and Photoluminescence of (6-Diphenylpnicogenoacenaphth-5-yl)gold Compounds	2019
5299	V. Nastopoulos	Tris(2-sulfidopyridine N-oxide-kappa O-2,S)arsenic(III): An arsenic(III) complex having three 5-membered rings	2014
5300	E. J. Kim	Abiotic reductive extraction of arsenic from contaminated soils enhanced by complexation: Arsenic extraction by reducing agents and combination of reducing and chelating agents	2015
5301	L. G. Romero-Esquivel	Iron oxide for arsenic removal in water: synthesis and characterization	2017
5302	K. M. Wai	In-utero arsenic exposure and growth of infants from birth to 6 months of age: a prospective cohort study in rural Bangladesh	2020
5303	S. R. Hu	Determination of trace arsenic by solid substrate-room temperature phosphorescence quenching method based on the catalyzed reaction of H2O2 oxidizing 9-hydroxy-2,3,4,9-tetrahydro-1, 10-anthraquinone	2007
5304	A. Menevseoglu	Evaluating the chemical and metal contamination of commercial Raki, a grape-based alcoholic beverage from Turkey	2021
5305	P. Nookongbut	Arsenic resistance genes of As-resistant purple nonsulfur bacteria isolated from As-contaminated sites for bioremediation application	2017
5306	M. Z. Bani-Fwaz	Crystal structures and quantum chemical calculations of dichloro 4-(dimethylamino)phenyl arsine and tris 4-(dimethylamino)phenyl arsine	2017
5307	R. I. Alekberov	Local structures and optical properties of As-Se-Te(S) chalcogenide glasses	2018
5308	I. Bae	As4O6 suppresses triple-negative breast cancer via inhibiting EGFR/mTOR mediated EMT in vitro and in vivo	2019

5309	O. Hegen	A Convenient Route to Mixed Pnictogenylboranes	2017
5310	F. Thomas	Synthesis and X-ray crystal structures of pnictogen-bridged, bimetallic complexes containing main group metal and tricarbonylnickel fragments	2001
5311	D. Baragano	Zero valent iron nanoparticles and organic fertilizer assisted phytoremediation in a mining soil: Arsenic and mercury accumulation and effects on the antioxidative system of Medicago sativa L	2022
5312	Q. Q. Liu	Accumulation and Transport of Roxarsone, Arsenobetaine, and Inorganic Arsenic Using the Human Immortalized Caco-2 Cell Line	2016
5313	H. Hasegawa	Freshwater phytoplankton: biotransformation of inorganic arsenic to methylarsenic and organoarsenic	2019
5314	F. M. Yunus	Relationship between arsenic skin lesions and the age of natural menopause	2014
5315	Y. Fu	Enabling simultaneous redox transformation of toxic chromium(VI) and arsenic(III) in aqueous media-A review	2021
5316	S. Bagheri	The Effect of Arsenic Trioxide on All-trans Retinoic Acid Binding to Human Serum Albumin	2019
5317	A. Embiale	Health risk assessment of trace elements through exposure of particulate matter-10 during the cooking of Ethiopian traditional dish sauces	2020
5318	X. Chen	Roxarsone Promotes Glycolysis and Angiogenesis by Inducing Hypoxia-Inducible Factor-1 alpha In Vitro and In Vivo	2021
5319	N. Khalil	Endocrine disruptive compounds and cardio-metabolic risk factors in children	2014
5320	O. Supplie	In situ controlled heteroepitaxy of single-domain GaP on As-modified Si(100)	2015
5321	L. Han	Estimating Soil Arsenic Content with Visible and Near-Infrared Hyperspectral Reflectance	2020
5322	X. R. Yang	Foliar application of the sulfhydryl compound 2,3-dimercaptosuccinic acid inhibits cadmium, lead, and arsenic accumulation in rice grains by promoting heavy metal immobilization in flag leaves	2021
5323	X. Y. Wu	Artocarmitin B enhances intracellular antioxidant capacity via activation of Nrf2 signaling pathway in human lung epithelial cells	2019
5324	N. V. Campos	Differences in phosphorus translocation contributes to differential arsenic tolerance between plants of Borreria verticillata (Rubiaceae) from mine and non-mine sites	2014
5325	V. Fristak	Preparation and Characterization of Novel Magnesium Composite/Walnut Shells-Derived Biochar for As and P Sorption from Aqueous Solutions	2021
5326	A. Johnson	Emerging Metallopharmaceuticals for the Treatment of Cancer	2021

5327	H. Guo	Nanofiltration for drinking water treatment: a review	2022
5328	B. U. Mueller	Myelodysplastic syndromes and acute myeloid leukemias in the elderly	2018
5329	A. Daneshyar	Electrochemical synthesis of a new phosphonium betaine. Kinetic evaluation and antibacterial susceptibility	2019
5330	J. Deitersen	Anthraquinones and autophagy - Three rings to rule them all?	2019
5331	A. Vega	Increasing Heavy Metal Tolerance by the Exogenous Application of Organic Acids	2022
5332	J. Kubacki	Temperature-Driven Changes of Electronic Structure Through the Phase Transition in Magnetocaloric Compound Mn1.1Fe0.9P0.55As0.45	2017
5333	Y. Gonzalez-Castanedo	Arsenic species in atmospheric particulate matter as tracer of the air quality of Donana Natural Park (SW Spain)	2015
5334	W. Y. Pan	A case-control study of arsenic exposure with the risk of primary ovarian insufficiency in women	2020
5335	T. Kato	Facile synthesis and properties of dithieno 3,2-b:2 ',3 '-d arsoles	2016
5336	H. L. Chen	Terpenoids Induce Cell Cycle Arrest and Apoptosis from the Stems of Celastrus kusanoi Associated with Reactive Oxygen Species	2010
5337	M. Sellin	Eightfold Electrophilic Methylation of Octacyanotungstate W(CN)(8) (4-/3-): Preparation of Homoleptic, Eight-Coordinate Methyl Isocyanide Complexes W(CNMe)(8) (4+/5+)	2021
5338	G. Thaler	Bis bis(trimethylsilyl)cyclopentadienyl selane as a ligand: selena-2,2 ',4,4 '-tetrakis(trimethylsilyl)- 1 ferrocenophane and 1,2,3-triselena-2,2 ',4,4 '-tetrakis(trimethylsilyl)-3 ferrocenophane	2001
5339	A. A. Alqadami	Determination of heavy metals in skin-whitening cosmetics using microwave digestion and inductively coupled plasma atomic emission spectrometry	2017
5340	S. M. Hocaoglu	Separation of arsenic(V) by composite adsorbents of metal oxide nanoparticles immobilized on silica flakes and use of adsorbent coated alumina tubes as an alternative method	2019
5341	L. L. Pruteanu	Targeting Cell Death Mechanism Specifically in Triple Negative Breast Cancer Cell Lines	2022
5342	N. Okibe	Microbial formation of crystalline scorodite for treatment of As(III)-bearing copper refinery process solution using Acidianus brierleyi	2014
5343	U. Monkowius	Ligand properties of tri(2-thienyl)- and tri(2-furyl)phosphine and -arsine (2-C4H3E)(3)P/As (E = O, S) in gold(I) complexes	2003
5344	N. J. Collier	The safety and efficacy of sonidegib for the treatment of locally advanced basal cell carcinoma	2016

5345	V. Paliwal	Pseudomonas putida CSV86: A Candidate Genome for Genetic Bioaugmentation	2014
5346	S. B. Bortnikova	Arsenic and metal quantities in abandoned arsenide tailings in dissolved, soluble, and volatile forms during 20 years of storage	2021
5347	P. Gunka	Dispersed Lone Electron Pairs in Cubic Polymorph of Arsenic(III) Oxide	2014
5348	S. Y. M. Chooi	Stereoelectronic effects on the chelating properties of 2-(methylsulfinyl)ethyl diphenylarsine and its phosphorus analog	1994
5349	S. Wang	Arsenopyrite weathering in acidic water: Humic acid affection and arsenic transformation	2021
5350	X. Ma	A novel method for preparing an As(V) solution for scorodite synthesis from an arsenic sulphide residue in a Pb refinery	2019
5351	T. Marzo	Strike a Balance: Between Metals and Non-Metals, Metalloids as a Source of Anti-Infective Agents	2021
5352	M. X. Chen	The Anticancer Properties of Herba Epimedii and Its Main Bioactive Componentsicariin and Icariside II	2016
5353	K. P. Pfeuffer	Afterglow of a microwave microstrip plasma as an ion source for mass spectrometry	2015
5354	M. Weil	The Mixed-valent Mercury(I/II) Compounds Hg-3(HAsO4)(2) and Hg6As2O10	2014
5355	N. Kanagathara	Experimental and theoretical (DFT) investigation of crystallographic, spectroscopic and Hirshfeld surface analysis of anilinium arsenate	2021
5356	J. G. Yang	Risks for Using FeCl3 Under a Submerged Condition, and Different Water Management to Reduce Uptake of Antimony and Cadmium in a Rice Plant	2021
5357	E. J. Kim	Enhanced reductive extraction of arsenic from contaminated soils by a combination of dithionite and oxalate	2015
5358	T. N. Van	Arsenic Speciation and Extraction and the Significance of Biodegradable Acid on Arsenic Removal-An Approach for Remediation of Arsenic-Contaminated Soil	2017
5359	E. Weightman	Stratigraphy and mineralogy of tailings at Macraes gold mine, southern New Zealand	
5360	M. K. Sharma	Crystalline Divinyldiarsene Radical Cations and Dications	2019
5361	A. Mohajerani	Chromated copper arsenate timber: A review of products, leachate studies and recycling	2018
5362	S. Z. Chen	Simultaneous Species Analysis of Arsenic, Selenium, Bromine, and Iodine in Bottled Drinking Water and Fruit Juice by High-Performance Liquid Chromatography-Inductively Coupled Plasma Mass Spectrometry	2021

5363	Q. Zhang	Polymeric micelles for GSH-triggered delivery of arsenic species to cancer cells	2014
5364	Y. N. Hu	Public health risk of trace metals in fresh chicken meat products on the food markets of a major production region in southern China	2018
5365	E. Revesz	Reductive dissolution of arsenical ferrihydrite by bacteria	2016
5366	C. Marquardt	Cationic Chains of Parent Arsanylboranes and Substituted Phosphanylboranes	2017
5367	J. J. Wang	Kinetic release of arsenic after exogenous inputs into two different types of soil	2018
5368	A. Gautam	Sucrose plays key role in amelioration of arsenic induced phytotoxicity through modulating phosphate and silicon transporters, physiological and biochemical responses in C-3 (Oryza sativa L.) and C-4 (Zea mays L.)	2020
5369	Y. M. Huang	A transcriptomic (RNA-seq) analysis of genes responsive to both cadmium and arsenic stress in rice root	2019
5370	A. J. Signes-Pastor	Toenail manganese as biomarker of drinking water exposure: a reliability study from a US pregnancy cohort	2019
5371	M. N. Islam	Toxic compounds in honey	2014
5372	J. A. Laske	Reactions of trimethylaluminum with secondary arsines: Synthesis and characterization of phenyl (trimethylsilyl)methyl arsine and the X-ray crystal structures of the trimers Me(2)AlAsPh(2) 3 center dot(C7H8)(2) and Me(2)AlAs(CH(2)SiMe(3))Ph (3)	1996
5373	P. A. Gunka	Lone electron pair dispersion experimental charge density study of cubic arsenic(III) oxide	2015
5374	J. Du	Pharmacological restoration and therapeutic targeting of the B-cell phenotype in classical Hodgkin lymphoma	2017
5375	S. B. Zhou	Risk assessment of pollutants in flowback and produced waters and sludge in impoundments	2022
5376	X. D. Xie	Mechanism, kinetics, and pathways of self-sensitized sunlight photodegradation of phenylarsonic compounds	2016
5377	V. Kumar	ANN-Based Integrated Risk Ranking Approach: A Case Study of Contaminants of Emerging Concern of Fish and Seafood in Europe	2021
5378	J. Lu	New insights on nanostructure of ordered mesoporous Fe-Mn bimetal oxides (OMFMs) by a novel inverse micelle method and their superior arsenic sequestration performance: Effect of calcination temperature and role of Fe/Mn oxides	2021
5379	A. D. Gupta	Adsorptive and photocatalytic properties of metal oxides towards arsenic remediation from water: A review	2021
5380	A. Valente	Metal- and metalloid-based compounds to target and reverse cancer multidrug resistance	2021

5381	G. Larrabure	A review on the negative impact of different elements during cyanidation of gold and silver from refractory ores and strategies to optimize the leaching process	2021
5382	E. J. Kim	Arsenic speciation and bioaccessibility in arsenic-contaminated soils: Sequential extraction and mineralogical investigation	2014
5383	L. Oruganti	Plant Polyphenolic Compounds Potentiates Therapeutic Efficiency of Anticancer Chemotherapeutic Drugs: A Review	2021
5384	A. Nandan	Assessment of environmental and ergonomic hazard associated to printing and photocopying: a review	2019
5385	P. Tahtinen	New Sulfur-Containing Polyarsenicals from the New Caledonian Sponge Echinochalina bargibanti	2018
5386	A. R. Pfaff	Medicinal Thiols: Current Status and New Perspectives	2020
5387	X. H. Wu	Dissolved Organic Matter Affects Arsenic Mobility and Iron(III) (hydr)oxide Formation: Implications for Managed Aquifer Recharge	2019
5388	N. L. Luo	Passivating Effect of Dewatered Sludge and Biochar on As-Contaminated Soil	2020
5389	M. Zhu	Contrasting effects of iron reduction on thionation of diphenylarsinic acid in a biostimulated Acrisol	2020
5390	A. Botero	In vitro drug susceptibility of two strains of the wildlife trypanosome, Trypanosoma copemani: A comparison with Trypanosoma cruzi	2017
5391	K. Ramirez-Muniz	Evaluation of natural iron mineral as possible collectors of arsenic and fluoride	2017
5392	A. Kasiuliene	Leaching of metal(loid)s from ashes of spent sorbent and stabilisation effect of calcium-rich additives	2020
5393	T. Ogata	Adsorption of Arsenic from Alkaline Solutions	2017
5394	J. G. Kim	Photocatalytic co-oxidation of As(III) and Orange G using urea-derived g-C3N4 and persulfate	2018
5395	K. J. Azcorra-May	Sargassum biorefineries: potential opportunities towards shifting from wastes to products	
5396	I. Galkina	Crystal structure of phosphonium carboxylate complexes. The role of the metal coordination geometry, ligand conformation and hydrogen bonding	2014
5397	E. M. Balboa	Valorization of Sargassum muticum Biomass According to the Biorefinery Concept	2015
5398	J. Liu	Facile synthesis of flower-like CoFe2O4 particles for efficient sorption of aromatic organoarsenicals from aqueous solution	2020

5399	Y. T. Feng	Evaluation of Se-72/As-72 generator and production of Se-72 for supplying As-73 as a potential PET imaging radionuclide	2019
5400	J. Y. Li	Antimony contamination, consequences and removal techniques: A review	2018
5401	J. B. Wu	Emerging low-dimensional materials for mid-infrared detection	2021
5402	E. T. Ciornea	Heavy Metal Pollution Affects the Antioxidant Potential of Rosa canina L. Species	2018
5403	V. M. Boddu	Environmentally Responsive Poly(N-isopropylacrylamide)-co-poly(acrylic acid) Hydrogels for Separation of Toxic Metals and Organic Explosive Compounds from Water	2019
5404	W. Bennarndt	Domains of molecular beam epitaxial growth of Ga(In)AsBi on GaAs and InP substrates	2016
5405	K. M. Wollin	Critical evaluation of human health risks due to hydraulic fracturing in natural gas and petroleum production	2020
5406	A. Hershcovitch	Molecular ion sources for low energy semiconductor ion implantation (invited)	2016
5407	M. Popielarski	Thioredoxin and thioredoxin reductase in the pathogenesis of selected human diseases, part II	2015
5408	D. A. Bashirov	Synthesis and Structure of Fe-3(mu(3)-Q)(mu(3)-AsN(i-Bu)(2))(CO)(9) (Q= Se, Te) Clusters and Products of Their Hydrolysis	2020
5409	H. Tian	Low-symmetry two-dimensional materials for electronic and photonic applications	2016
5410	W. A. Schenk	Reactive arsenic heterocyclic-compounds .4. transition-metal complexes of 2,3,4,5-tetramethylarsolene	1994
5411	Y. L. Li	High-temperature interaction of Ce-Fe-As ternary system	2019
5412	A. Ullah	Effective Amendments on Cadmium, Arsenic, Chromium and Lead Contaminated Paddy Soil for Rice Safety	2020
5413	M. Murko	Dose and Diet - Sources of Arsenic Intake in Mouse in Utero Exposure Scenarios	2018
5414	J. M. Purenovic	Physicochemically modified peat by thermal and oxidation processes as an active material for purification of wastewaters from certain hazardous pollutants	2017
5415	D. V. Khasnis	Tetraaza macrocyclic derivatives of arsenic - hcyclenas, hcyclenas.game3, and hcyclamas	1992
5416	K. D. Moiseev	Features of an InAsSbP epilayer formation on an InAs support by metalorganic vapor phase epitaxy	2016

5417	C. J. Thomas	Synthesis and x-ray crystal-structure of (p-ch3c6h4)(3)as=n-s3n3	1995
5418	Z. L. Hildenbrand	Rapid Analysis of Eukaryotic Bioluminescence to Assess Potential Groundwater Contamination Events	2015
5419	L. Wang	Red mud-enhanced magnesium phosphate cement for remediation of Pb and As contaminated soil	2020
5420	S. Khan	Concentration of Aflatoxin M-1 and selected heavy metals in mother milk samples from Pakistan	2018
5421	K. F. Pi	Arsenic Oxidation by Flavin-Derived Reactive Species under Oxic and Anoxic Conditions: Oxidant Formation and pH Dependence	2019
5422	R. J. Smith	Environmentally relevant arsenic exposure affects morphological and molecular endpoints associated with reproduction in the Western mosquitofish, Gambusia affinis	2022
5423	M. Gasecka	Arsenic uptake, speciation and physiological response of tree species (Acer pseudoplatanus, Betula pendula and Quercus robur) treated with dimethylarsinic acid	2021
5424	M. Durandurdu	Liquid and amorphous states of boron subarsenide	2020
5425	U. Koenigs	Isolation of high purity Se-73 using solid phase extraction after selective 4,5- Se-73 benzopiazselenol formation with aminonaphthalene	2018
5426	A. Menahem	Transport of gadolinium- and arsenic-based pharmaceuticals in saturated soil under various redox conditions	2016
5427	X. L. Song	Enhanced Anticancer Cells Effects of Optimized Suspension Stable As2O3-Loaded Poly(lactic-co-glycolic acid) Nanocapsules	2015
5428	L. D. Mafu	Adsorption studies for the simultaneous removal of arsenic and selenium using naturally prepared adsorbent materials	2014
5429	R. J. Baker	9-Triptycenyl complexes of group 13 and 15 halides and hydrides	2004
5430	S. Steinlechner	Characterization and process development for the selective removal of sn, sb, and as from anode slime obtained from electrolytic copper refining	2018
5431	N. Saca	Leaching behavior of some demolition wastes	2017
5432	E. R. Melendez-Sanchez	Review: Biotechnological Potential of As- and Zn-Resistant Autochthonous Microorganisms from Mining Process	2021
5433	R. J. Wehmschulte	Reactions of (H(2)AlMes*)(2) (Mes*=2,4,6-(t-Bu)(3)C6H2) with H(2)EAr (E=N,P, or As; Ar=aryl): Characterization of the ring compounds (Mes*AlNPh)(2) and (Mes*AlEPh)(3) (E=P or As)	1996
5434	J. Stuckey	Arsenic release metabolically limited to permanently water-saturated soil in Mekong Delta	2016

5435	J. Han	A prediction of arsenic and selenium emission during the process of bituminous and lignite coal co-combustion	2020
5436	C. Monchanin	Honey bees cannot sense harmful concentrations of metal pollutants in food	2022
5437	N. Balasundaram	Metabolic adaptation drives arsenic trioxide resistance in acute promyelocytic leukemia	2022
5438	B. K. Chaudhary	Understanding Regeneration of Arsenate-Loaded Ferric Hydroxide-Based Adsorbents	2015
5439	A. I. Baranov	Defect properties of solar cells with layers of GaP based dilute nitrides grown by molecular beam epitaxy	2020
5440	D. J. Heuschele	Metabolic Responses to Arsenite in Rice Seedlings that Differed in Grain Arsenic Concentration	2017
5441	J. Y. Wu	Challenges for Safe and Healthy Drinking Water in China	2020
5442	F. A. C. Lopes	Microbial Community Profile and Water Quality in a Protected Area of the Caatinga Biome	2016
5443	W. Z. Zhang	Ferrisilicalite-1 Zeolite Monolith-Based Affinitive Recognition of Intracellular Phosphorylated Protein Alteration in Tetrahymena thermophila Exposed to Photodegradates of Roxarsone and p-Arsanilic Acid	2014
5444	A. Krauklis	FeOOH and Mn8O10Cl3 modified zeolites for As(V) removal in aqueous medium	2017
5445	R. Sulaiman	Exposure to Aluminum, Cadmium, and Mercury and Autism Spectrum Disorder in Children: A Systematic Review and Meta-Analysis	2020
5446	X. Y. Li	Determination on the activity of formed CaSO4 for arsenic adsorption during arsenic capture by CaO with the presence of SO2: Experimental and density functional theory study	2022
5447	A. K. Bozack	DNA methylation in cord blood as mediator of the association between prenatal arsenic exposure and gestational age	2018
5448	S. I. Siddiqui	Iron oxide and its modified forms as an adsorbent for arsenic removal: A comprehensive recent advancement	2017
5449	M. Girdhar	Comparative assessment for hyperaccumulatory and phytoremediation capability of three wild weeds	2014
5450	A. E. Nigra	Poultry Consumption and Arsenic Exposure in the US Population	2017
5451	H. Ramirez-Aldaba	Changes in biooxidation mechanism and transient biofilm characteristics by As(V) during arsenopyrite colonization with Acidithiobacillus thiooxidans	2018
5452	K. Sharma	Thermodynamic and Kinetic Studies of Methylene Blue Degradation Using Reactive Adsorption and Its Comparison with Adsorption	2017

5453	J. Gomez-Pastora	Analysis of separators for magnetic beads recovery: From large systems to multifunctional microdevices	2017
5454	L. Chen	Application of Metal Oxide Heterostructures in Arsenic Removal from Contaminated Water	2014
5455	R. R. Somasagara	Targeted therapy of human leukemia xenografts in immunodeficient zebrafish	2021
5456	S. I. Siddiqui	A review on graphene oxide and its composites preparation and their use for the removal of As3+ and As5+ from water under the effect of various parameters: Application of isotherm, kinetic and thermodynamics	2018
5457	E. N. Selivanov	Structure of Arsenic Sulfide Cake and Solubility of Its Alloys with Sulfur	2021
5458	A. Aguiar	Acid mine drainage treatment by nanofiltration: A study of membrane fouling, chemical cleaning, and membrane ageing	2018
5459	J. Vunduk	Addition of Zeolites to Improve the Functional Characteristics of the Hen of the Wood or Maitake Medicinal Mushroom, Grifola frondosa (Agaricomycetes) `	2016
5460	T. H. Bui	Effective adsorbent for arsenic removal: core/shell structural nano zero-valent iron/manganese oxide	2017
5461	H. Lee	Preparation of DMMTA(V) and DMDTA(V) Using DMA(V) for Environmental Applications: Synthesis, Purification, and Confirmation	2018
5462	J. Moskwa	Polish and New Zealand Propolis as Sources of Antioxidant Compounds Inhibit Glioblastoma (T98G, LN-18) Cell Lines and Astrocytoma Cells Derived from Patient	2022
5463	N. A. Valisheva	Optical properties of native (anodic) layer on the InAlAs surface of different morphology	2021
5464	B. B. Green	Epigenome-Wide Assessment of DNA Methylation in the Placenta and Arsenic Exposure in the New Hampshire Birth Cohort Study (USA)	2016
5465	L. F. O. Silva	Nanometric particles of high economic value in coal fire region: Opportunities for social improvement	2020
5466	M. E. Islam	Distribution of arsenic in core sediments and groundwater in the Chapai Nawabganj district, Bangladesh	2019
5467	H. M. Guzman	Release of arsenic from metal oxide sorbents under simulated mature landfill conditions	2016
5468	Y. J. Liang	Stabilization of arsenic sludge with mechanochemically modified zero valent iron	2017
5469	N. Calace	Antarctic snow: metals bound to high molecular weight dissolved organic matter	2017
5470	R. Sharifi	Assessment of Health Risks of Arsenic Exposure via Consumption of Crops	2018

5471	D. P. Zhong	Recovery of antimony from antimony-bearing dusts through reduction roasting process under CO-CO2 mixture gas atmosphere after firstly oxidation roasted	2018
5472	S. M. Meier-Menches	A Proteomic Platform Enables to Test for AML Normalization In Vitro	2022
5473	A. Duffy	Autophagy modulation: a target for cancer treatment development	2015
5474	X. Han	Protective Effects of 6-Gingerol on Cardiotoxicity Induced by Arsenic Trioxide Through AMPK/SIRT1/PGC-1 alpha Signaling Pathway	2022
5475	C. Abbehausen	Zinc finger domains as therapeutic targets for metal-based compounds - an update	2019
5476	L. T. M. Thy	Fabrication and adsorption properties of magnetic graphene oxide nanocomposites for removal of arsenic (V) from water	2020
5477	C. Fuchs	Electrical injection type-II (GaIn)As/Ga(AsSb)/(GaIn)As single "W'-quantum well laser at 1.2 mu m	2016
5478	J. Zhu	Preparation of Iron and Manganese Oxides/Carbon Composite Materials for Arsenic Removal from Aqueous Solution	2014
5479	A. R. D'Amico	Embryonic arsenic exposure reduces the number of muscle fibers in killifish (Fundulus heteroclitus)	2014
5480	A. Godelitsas	Amorphous As-sulfide precipitates from the shallow-water hydrothermal vents off Milos Island (Greece)	2015
5481	C. Zou	The effect of H2O on formation mechanism of arsenic oxide during arsenopyrite oxidation: Experimental and theoretical analysis	2020
5482	S. Balarastaghi	Mechanisms of Arsenic Exposure-Induced Hypertension and Atherosclerosis: an Updated Overview	
5483	J. T. Hou	The remarkable effect of the coexisting arsenite and arsenate species ratios on arsenic removal by manganese oxide	2017
5484	M. L. Tarlton	Systematic Investigation of the Molecular and Electronic Structure of Thorium and Uranium Phosphorus and Arsenic Complexes	2021
5485	S. Strekopytov	Arsenic and mercury in bird feathers: Identification and quantification of inorganic pesticide residues in natural history collections using multiple analytical and imaging techniques	2017
5486	N. Ajith	Evidences on As(III) and As(V) interaction with iron(III) oxides: Hematite and goethite	2021
5487	J. D. Palcic	Lewisite exposure biomarkers in urine by liquid chromatography - inductively coupled plasma tandem mass spectrometry: with an accelerated matrix-matched stability study	2015
5488	D. W. Wu	The role of SO2 in arsenic removal by carbon-based sorbents: A DFT study	2021

5489	R. H. Li	Ionic Potential: A General Material Criterion for the Selection of Highly Efficient Arsenic Adsorbents	2014
5490	P. Chen	Bioavailable arsenic and amorphous iron oxides provide reliable predictions for arsenic transfer in soil-wheat system	2020
5491	D. Postma	A model for the evolution in water chemistry of an arsenic contaminated aquifer over the last 6000 years, Red River floodplain, Vietnam	2016
5492	M. El Youssfi	Trace elements in Foodstuffs from the Mediterranean Basin-Occurrence, Risk Assessment, Regulations, and Prevention strategies: A review	
5493	P. C. Ke	Synthesis, characterization, and property test of crystalline polyferric sulfate adsorbent used in treatment of contaminated water with a high As(III) content	2018
5494	Z. T. Li	Zeolite-supported nanoscale zero-valent iron: New findings on simultaneous adsorption of Cd(II), Pb(II), and As(III) in aqueous solution and soil	2018
5495	C. Zou	Effect of CO(2)on the As2O3 adsorption over carbonaceous surface: Experiment and quantum chemistry study	2022
5496	P. L. G. Ventzek	Formation, nature, and stability of the arsenic-silicon-oxygen alloy for plasma doping of non-planar silicon structures	2014
5497	S. Padungthon	Polymeric anion exchanger supported hydrated Zr(IV) oxide nanoparticles: A reusable hybrid sorbent for selective trace arsenic removal	2015
5498	C. Falagan	Acidibacter ferrireducens gen. nov., sp. nov.: an acidophilic ferric iron-reducing gammaproteobacterium	2014
5499	D. Ravi	The Novel Organic Arsenical Darinaparsin Induces MAPK-Mediated and SHP1-Dependent Cell Death in T-cell Lymphoma and Hodgkin Lymphoma Cells and Human Xenograft Models	2014
5500	J. S. Yang	Adsorption of Arsenic from Aqueous Solutions by Iron Oxide Coated Sand Fabricated with Acid Mine Drainage	2015
5501	L. Coudert	Treatment of As-rich mine effluents and produced residues stability: Current knowledge and research priorities for gold mining	2020
5502	G. A. Leao	Sulfur metabolism: Different tolerances of two aquatic macrophytes exposed to arsenic	2014
5503	A. Rahman	Early life arsenic exposure, infant and child growth, and morbidity: a systematic review	2017
5504	H. Liu	Geochemical fates and unusual distribution of arsenic in natural ferromanganese duricrust	2017
5505	P. Shukla	Nitric oxide mediated amelioration of arsenic toxicity which alters the alternative oxidase (Aox1) gene expression in Hordeum vulgare L	2015
5506	X. X. Wu	Theoretical sight into hydrogen bond interactions between arsenious acid and thiols in aqueous and HEPES solutions	2021

5507	A. Bauer	Skin Cancer as Occupational Disease	2016
5508	J. Cai	Exposure to particulate air pollution during early pregnancy is associated with placental DNA methylation	2017
5509	A. P. J. Scandelai	Intensification of supercritical water oxidation (ScWO) by ion exchange with zeolite for the reuse of landfill leachates	2021
5510	M. D. Moghadam	Ameliorative Effects of Caffeic Acid Against Arsenic-Induced Testicular Injury in Mice	2021
5511	J. E. Lubov	Medicinal Plant Extracts and Natural Compounds for the Treatment of Cutaneous Lupus Erythematosus: A Systematic Review	2022
5512	S. Esmaielzadeh	Methyl jasmonate increases aluminum tolerance in rice by augmenting the antioxidant defense system, maintaining ion homeostasis, and increasing nonprotein thiol compounds	2022
5513	S. H. Yu	Deep insight into the effect of NaCl/HCl/SO2/CO2 in simulated flue gas on gas-phase arsenic adsorption over mineral oxide sorbents	2021
5514	I. Kopljar	Chronic drug-induced effects on contractile motion properties and cardiac biomarkers in human induced pluripotent stem cell-derived cardiomyocytes	2017
5515	C. F. Harrington	Analytical approaches to investigating metal-containing drugs	2015
5516	T. O. M. Lopes	Metals, arsenic, pesticides, and microcystins in tilapia (Oreochromis niloticus) from aquaculture parks in Brazil	2020
5517	F. J. Zhao	Toxic metals and metalloids: Uptake, transport, detoxification, phytoremediation, and crop improvement for safer food	2022
5518	T. Negishi	Diphenylarsinic Acid Induced Activation of Cultured Rat Cerebellar Astrocytes: Phosphorylation of Mitogen-Activated Protein Kinases, Upregulation of Transcription Factors, and Release of Brain-Active Cytokines	2016
5519	Y. M. Dong	A novel mechanism study of microplastic and As co-contamination on indica rice (Oryza sativa L.)	2022
5520	Y. Q. Fu	Arsenic speciation and bioaccessibility in raw and cooked seafood: Influence of seafood species and gut microbiota	2021
5521	S. S. Zhao	Effects of Arsenic Trioxide-Loaded PLGA Nanoparticles on Proliferation and Migration of Human Vascular Smooth Muscle Cells	2021
5522	W. Satoh	Synthesis of arsenic(V) octaethylporphyrins and X-ray crystal structure of (OEP)As(Me)(OH) ClO4	1997
5523	P. Rout	Exploring Nanostructured Zr/Cu Composite Oxide (NZCO) as an Efficient Adsorbent for Removal of As(III) and As(V) from Aqueous Solution	2019
5524	J. Ahmad	Differential impact of some metal(loid)s on oxidative stress, antioxidant system, sulfur compounds, and protein profile of Indian mustard (Brassica junceaL.)	2020

5525	E. S. J. Gontijo	Effects of Fe(III) and quality of humic substances on As(V) distribution in freshwater: Use of ultrafiltration and Kohonen neural network	2017
5526	F. Meloni	Total and Leached Arsenic, Mercury and Antimony in the Mining Waste Dumping Area of Abbadia San Salvatore (Mt. Amiata, Central Italy)	2021
5527	E. Heiderscheidt	Design, construction and monitoring of pilot systems to evaluate the effect of freeze-thaw cycles on pollutant retention in wetlands	2020
5528	S. E. Scanlon	Nickel induces transcriptional down-regulation of DNA repair pathways in tumorigenic and non-tumorigenic lung cells	2017
5529	A. L. Maulvault	Assessing the effects of seawater temperature and pH on the bioaccumulation of emerging chemical contaminants in marine bivalves	2018
5530	M. Wiech	Undesirables in Mesopelagic Species and Implications for Food and Feed Safety-Insights from Norwegian Fjords	2020
5531	E. F. Winterbottom	The aquaglyceroporin AQP9 contributes to the sex-specific effects of in utero arsenic exposure on placental gene expression	2017
5532	S. N. Chen	Degradation of p-arsanilic acid by pre-magnetized Fe-0/persulfate system: Kinetics, mechanism, degradation pathways and DBPs formation during subsequent chlorination	2021
5533	B. C. Jurgens	A Ternary Age-Mixing Model to Explain Contaminant Occurrence in a Deep Supply Well	2014
5534	Y. Tian	Enhanced antitumor activity of realgar mediated by milling it to nanosize	2014
5535	S. A. Hosseini	Removal of arsenic from aqueous solutions using MgFe2O4 nano spinel and GO/MgFe2O4 nanocomposite: an application of response surface methodology	2017
5536	A. Gonzalez-Vergara	Assessment of chalcone-vanillin as a selective chemosensor of As(III) in aqueous solution	2022
5537	I. W. K. Ouedraogo	Removal of arsenic (V) from aqueous medium using manganese oxide coated lignocellulose/silica adsorbents	2016
5538	I. H. Nam	Arsenic (As) Removal Using Talaromyces sp. KM-31 Isolated from As-Contaminated Mine Soil	2019
5539	S. Sacmaci	Determination of Arsenic(III) and Total Arsenic at Trace Levels in Baby Food Samples via a New Functionalized Magnetic Graphane Oxide Nanocomposite	2021
5540	J. Bai	Taurine protects against As2O3-induced autophagy in livers of rat offsprings through PPAR gamma pathway	2016
5541	Z. P. Wen	Facile inverse micelle fabrication of magnetic ordered mesoporous iron cerium bimetal oxides with excellent performance for arsenic removal from water	2020
5542	T. H. Bui	Removal of Arsenic from Water Using a Composite of Iron-Manganese Oxide Incorporated Active Rice Husk Silica	2021

5543	C. Xiao	Arsenic releasing characteristics during the compaction of muddy sediments	2016
5544	V. G. Dubrovskii	Model of selective growth of III-V nanowires	2015
5545	Z. H. Yu	Effects of a manganese oxide-modified biochar composite on adsorption of arsenic in red soil	2015
5546	Q. Huang	Reduction of arsenic toxicity in two rice cultivar seedlings by different nanoparticles	2018
5547	S. Aliwaini	The palladacycle, AJ-5, exhibits anti-tumour and anti-cancer stem cell activity in breast cancer cells	2015
5548	A. S. Pronin	Synthesis and structure of a tetrahedral rhenium complex with a mixed-ligand cluster core {re4as0.75(aso)(2.)te-25}(5+)	2020
5549	S. N. Gorbov	Genotoxicity and contamination of natural and anthropogenically transformed soils of the city of Rostov-on-Don with heavy metals	2015
5550	M. Miguel	Determination of arsenic in sediment samples from a well in the comarca lagunera, mexico	2018
5551	A. Chen	Removal of aqueous arsenic using abundant boehmite coated zeolite	2017
5552	J. An	Formation of dimethyldithioarsinic acid in a simulated landfill leachate in relation to hydrosulfide concentration	2016
5553	L. Natterman n	An experimental approach for real time mass spectrometric CVD gas phase investigations	2018
5554	J. B. Vaney	Thermoelectric Properties of the alpha-As2Te3 Crystalline Phase	2016
5555	P. C. Srivastava	Synthesis and characterization of bis(ferrocenylcarboxylato)telluranes	2007
5556	E. M. Muehe	Arsenic(V) Incorporation in Vivianite during Microbial Reduction of Arsenic(V)-Bearing Biogenic Fe(III) (Oxyhydr)oxides	2016
5557	A. S. D. Stahlich	Permethylated Disila 2 metallocenophanes of Group 14 and 15 Elements	2019
5558	H. Su	High-performance iron oxide-graphene oxide nanocomposite adsorbents for arsenic removal	2017
5559	B. A. Rather	Appraisal of functional significance of sulfur assimilatory products in plants under elevated metal accumulation	2022
5560	M. Piesch	Element-Element Bond Formation upon Oxidation and Reduction	2020

5561	M. Seidl	Reaction of Pentelidene Complexes with Diazoalkanes: Stabilization of Parent 2,3-Dipnictabutadienes	2016
5562	B. Jacob	Surface Passivation of III-V GaAs Nanopillars by Low-Frequency Plasma Deposition of Silicon Nitride for Active Nanophotonic Devices	
5563	J. Braese	Gold(I) Complexes Containing Phosphanyl- and Arsanylborane Ligands	2018
5564	A. Coccato	On the stability of mediaeval inorganic pigments: a literature review of the effect of climate, material selection, biological activity, analysis and conservation treatments	2017
5565	M. C. Bayer	Structure and Properties of Fumaryl Fluoride	2021
5566	W. Szeto	Recent developments of titanium dioxide materials for aquatic antifouling application	2021
5567	Z. Karancsi	Clinical pharmacology of anthelminthics in the small animal medicine Literature review	2019
5568	C. C. Kuo	Arsenic Exposure, Arsenic Metabolism, and Incident Diabetes in the Strong Heart Study	2015
5569	H. Sigel	The bio-relevant metals of the periodic table of the elements	2019
5570	D. Chakraborti	Groundwater arsenic contamination and its health effects in India	2017
5571	G. Eperon	Treatment options for second-stage gambiense human African trypanosomiasis	2014
5572	S. Maheshwari	Synthesis and spectroscopic characterization of toluene-3,4-dithiolatoarsenic(III)-O,O'-ditolyl/alkylene dithiophosphate compounds: crystal structure of CH3C6H3S2As{S2P(OC6H4Me-m)(2)}	2014
5573	J. Conde-Vancells	Combining mouse embryonic stem cells and zebrafish embryos to evaluate developmental toxicity of chemical exposure	2018
5574	X. L. Xiong	Approaches towards understanding the mechanism-of-action of metallodrugs	2022
5575	X. C. Liu	Structural Variability versus Structural Flexibility. A Case Study of Eu9Cd4+xSb9 and Ca9Mn4+xSb9 (x approximate to 1/2)	2015
5576	E. Martinez-Galero	Preclinical antitoxic properties of Spirulina (Arthrospira)	2016
5577	H. P. S. Chauhan	Synthesis, spectroscopic, structural characterization, and antimicrobial studies of 1,3-dithia-2-arsacyclopentane derivatives with oxygen and sulfur donor ligands	2011
5578	G. E. Seralini	Toxic compounds in herbicides without glyphosate	2020

5579	T. Nicolas-Mendez	The role of resveratrol on heavy metal-induced oxidative stress	2020
5580	G. Y. Wang	Determination of volatile organic compounds in SW620 colorectal cancer cells and tumor-bearing mice	2019
5581	Z. Mazej	Crystal Growth and Characterization of the Mixed-Cation Rb+/ XeF5 (+) and Cs+/ XeF5 (+) Salts	2017
5582	M. S. Safarzadeh	Thermal removal of arsenic from copper concentrates: Three-dimensional isothermal predominance diagrams for the Cu-As-S-O system	2018
5583	E. C. Spencer	Gallium Arsenate Dihydrate under Pressure: Elastic Properties, Compression Mechanism, and Hydrogen Bonding	2015
5584	C. Yassine	Structural, elastic and dynamical properties of YP1-xAsx alloys from first principles calculations	2019
5585	A. Kumar	Recovery of Trace and Heavy Metals from Coal Combustion Residues for Reuse and Safe Disposal: A Review	2016
5586	M. E. Moussa	Versatile Coordination of Ag-I and Cu-I Ions towards cyclo-As-5 Ligands	2021
5587	J. M. Liu	Synthesis, crystal structure and properties of sandwich type compounds based on {AsW9} and a hexa-nuclear unit with three supporting TM-triazole complexes	2015
5588	Y. F. Liu	Long-term outcome of 31 cases of refractory acute promyelocytic leukemia treated with compound realgar natural indigo tablets administered alternately with chemotherapy	2015
5589	Z. Y. Gao	Identifying the active sites of carbonaceous surface for the adsorption of gaseous arsenic trioxide: A theoretical study	2020
5590	J. Pazusiene	Cytogenetic damage in native Baltic Sea fish species: environmental risks associated with chemical munition dumping in the Gotland Basin of the Baltic Sea	2021
5591	J. Feldmann	Advancing Trace-Element Speciation in the Environment, Food, and Biota	2015
5592	N. T. Suen	Synthesis, crystal structures, and physical properties of the new Zintl phases A(21)Zn(4)Pn(18) (A = Ca, Eu; Pn=As, Sb)-Versatile arrangements of ZnPn(4) tetrahedra	2015
5593	T. G. Bean	An in vitro method for determining the bioaccessibility of pharmaceuticals in wildlife	2016
5594	W. Z. Zeng	Studies of Anode Slime Sintering/Coalescence and Its Effects on Anode Slime Adhesion and Cathode Purity in Copper Electrorefining	2016
5595	S. Gorecki	Human health risks related to the consumption of foodstuffs of plant and animal origin produced on a site polluted by chemical munitions of the First World War	2017
5596	R. Agrawal	Mixed Chloro Bis Alkylenedithiophosphato Antimony(III) and Their Heterobinuclear Derivatives with Boron Tetraisopropoxide: Synthesis and Characterization	2010

5597	A. E. Coman	A study for a class of flame retardant systems based on thermal, optical and mechanical analysis	2019
5598	L. D. Garbinski	Organoarsenicals inhibit bacterial peptidoglycan biosynthesis by targeting the essential enzyme MurA	2020
5599	E. R. C. Cardoso	Organic matter quality by pyrolysis-gas chromatography/mass spectrometry and lead and arsenic adsorption	2022
5600	F. Wu	Effects of zinc oxide nanoparticles on arsenic stress in rice (Oryza sativa L.): germination, early growth, and arsenic uptake (vol 27, pg 26974, 2020)	2021
5601	J. Y. Xing	A deep insight into the role of O-2 on As2O3 capture over gamma-Al2O3 sorbent: Experimental and DFT study	2021
5602	V. Noel	FeS colloids - formation and mobilization pathways in natural waters	2020
5603	A. Seyfferth	Fate of arsenic at the soil-plant interface: Impacts of soil-incorporation of plant-available silicon on arsenic desorption, iron oxide plaque, and plant uptake	2015
5604	M. K. Sharma	Crystalline Divinyldiarsenes and Cleavage of the As=As Bond	2019
5605	C. W. Neil	Fe3+ Addition Promotes Arsenopyrite Dissolution and Iron(III) (Hydr)oxide Formation and Phase Transformation	2016
5606	Q. T. Shi	Interactions between arsenic and ferric iron during ferric coprecipitation treatment: Effect of arsenic on ferric oxides formation, and possible formation of ferric arsenate complexes	2019
5607	W. B. Xin	Effect of Arsenic and Copper plus Arsenic on High Temperature Oxidation and Hot Shortness Behavior of C-Mn Steel	2016
5608	D. Parrone	A multi-method approach for the assessment of natural background levels in groundwater	2019
5609	P. B. Hu	Research on operation parameters and properties of flue gas on adsorption of As(2)O3 by gamma-Al2O3: An experiment and simulation study	2021
5610	N. Ajith	Study on the performance and interaction of different synthetic iron oxides for arsenic uptake using As-76 radiotracer	2019
5611	X. L. Song	Preparation and Investigation of Arsenic Trioxide-loaded Polylactic Acid/Magnetic Hybrid Nanoparticles	2014
5612	Z. C. He	As2O3 inhibits the expression of Th17 cell-related transcription factors in MRL_1pr mice	2019
5613	T. Fujii	Drastic Enhancement of Photosensitized Energy Transfer Efficiency of a Eu(III) Complex Driven by Arsenic	2021
5614	K. Laka	Cytotoxic effect of arsenic trioxide-beta-cyclodextrin fly ash-derived carbon nanospheres (As2O3-beta-cyclodextrin CNSs)	2019

5615	W. Zhang	A novel nanostructured Fe-Ti-Mn composite oxide for highly efficient arsenic removal: Preparation and performance evaluation	2019
5616	S. Kumar	Fabrication and characterization of highly sensitive and selective arsenic sensor based on ultra-thin graphene oxide nanosheets	2016
5617	V. Reshetnikov	Chemical Tools for Targeted Amplification of Reactive Oxygen Species in Neutrophils	2018
5618	R. Zhu	Alkaline refining of crude lead: a method of arsenic removal and the behavior of arsenic in the process	2022
5619	A. T. Sonne	Linking ecological health to co-occurring organic and inorganic chemical stressors in a groundwater-fed stream system	2018
5620	X. X. Chen	Overexpression of the long noncoding RNA NEAT1 protects against As2O3-induced injury of cardiomyocyte by inhibiting the miR-124/NF-kappa B signaling pathway	2020
5621	F. Teoldi	Air quality in the Olona Valley and in vitro human health effects	2017
5622	J. Dedic	An Experimental Investigation of the Environmental Risk of a Metallurgical Waste Deposit	2022
5623	T. T. Chen	Influences of the petroleum-recovering activity on the arsenic level in groundwater of Kuitun, Xinjiang, China	2018
5624	T. Yorifuji	Height and blood chemistry in adults with a history of developmental arsenic poisoning from contaminated milk powder	2017
5625	K. Laka	Survivin Splice Variants in Arsenic Trioxide (As2O3)-Induced Deactivation of PI3K and MAPK Cell Signalling Pathways in MCF-7 Cells	2019
5626	H. Long	Purification of crude As2O3 recovered from antimony smelting arsenic-alkali residue	2020
5627	C. Z. Chen	Resveratrol Protects Against Arsenic Trioxide-Induced Oxidative Damage Through Maintenance of Glutathione Homeostasis and Inhibition of Apoptotic Progression	2015
5628	P. Pei	Inorganic arsenic induces pyroptosis and pancreatic beta cells dysfunction through stimulating the IRE1 alpha/TNF-alpha pathway and protective effect of taurine	2019
5629	L. Bindi	From ancient pigments to modern optoelectronic applications of arsenic sulfides: bonazziite, the natural analogue of beta-As4S4 from Khaidarkan deposit, Kyrgyzstan	2015
5630	S. Ren	Poisoning effects of KCl and As2O3 on selective catalytic reduction of NO with NH3 over Mn-Ce/AC catalysts at low temperature	2018
5631	Z. Guo	Effect of soil layer thickness on organic carbon mineralization in improved sandy land	2022
5632	G. Z. Pan	RUNX3 plays an important role in As2O3-induced apoptosis and allows cells to overcome MSC-mediated drug resistance	2016

5633	H. Su	Carbon nanosphere-iron oxide nanocomposites as high-capacity adsorbents for arsenic removal	2017
5634	W. J. Liu	ATRA and As2O3 regulate differentiation of human hematopoietic stem cells into granulocyte progenitor via alteration of HoxB8 expression	2015
5635	L. Y. Chai	Behavior, distribution and environmental influence of arsenic in a typical lead smelter	2015
5636	S. Rakhimbekova	Occurrence of Arsenic in Nearshore Aquifers Adjacent to Large Inland Lakes	2021
5637	M. A. Mejia-Gonzalez	Mechanisms that release arsenic to the groundwater of the Laguna Region, states of Coahuila and Durango, Mexico	2014
5638	N. Priyadarshni	Sustainable removal of arsenate, arsenite and bacterial contamination from water using biochar stabilized iron and copper oxide nanoparticles and associated mechanism of the remediation process	2020
5639	K. Javed	Rice husk ash adsorbent modified by iron oxide with excellent adsorption capacity for arsenic removal from water	
5640	H. Long	Study on arsenic removal in aqueous chloride solution with lead oxide	2019
5641	H. Y. Sun	High concentration of arsenic removal from acid leaching solution of zinc oxide dust by water-quenched slag	2018
5642	J. J. Wouters	Performance of SiO2, ZrO2, TiO2, Al2O3 or Fe2O3 Coatings on Ti Electrodes for Arsenic (V) Detection Utilizing Electrochemical Impedance Spectroscopy	2018
5643	B. Huang	Arsenic Modulates Posttranslational S-Nitrosylation and Translational Proteome in Keratinocytes	2014
5644	D. D. La	Fabrication of a GNP/Fe-Mg Binary Oxide Composite for Effective Removal of Arsenic from Aqueous Solution	2017
5645	Y. N. Jiang	Enhanced removal of arsenic from a highly laden industrial effluent using a combined coprecipitation/nano-adsorption process	2014
5646	A. Osono	Characteristics of the immobilization process of arsenic depending on the size fraction released from excavated rock/sediment after the addition of immobilization materials	2021
5647	R. Soni	Synthesis of fly ash based zeolite-reduced graphene oxide composite and its evaluation as an adsorbent for arsenic removal	2019
5648	J. L. Hu	Dynamic desorption of arsenic from polymer-supported hydrated iron(III) oxide in a wastewater treatment plant	2017
5649	M. S. Bono	Iron oxide xerogels for improved water quality monitoring of arsenic(iii) in resource-limited environments via solid-phase extraction, preservation, storage, transportation, and analysis of trace contaminants (SEPSTAT)	2021
5650	M. Wang	AZT sensitizes hepatocellular carcinoma cells to As2O3 by up-regulating the arsenic transporter aquaglyceroporin 9	2018

5651	D. Dutta	Iron oxide coated hollow poly(methylmethacrylate) as an efficient adsorption media for removal of arsenic from water	2021
5652	W. Cheng	Etching synthesis of iron oxide nanoparticles for adsorption of arsenic from water	2016
5653	A. Salanitro	Electrochemical behavior of archaeological arsenical bronzes according to the concentration of arsenic in the alloy	2021
5654	Z. C. Zhou	Genome- and Community-Level Interaction Insights in Carbon Utilization and Element Cycling Functions of Hydrothermarchaeota in Hydrothermal Sediment	2020
5655	E. Sazakli	Arsenic and antimony removal from drinking water by adsorption on granular ferric oxide	2015
5656	S. W. Liu	Micro/nanostructured porous Fe-Ni binary oxide and its enhanced arsenic adsorption performances	2015
5657	M. Vazquez-Jaime	Effective removal of arsenic from an aqueous solution by ferrihydrite/goethite graphene oxide composites using the modified Hummers method	2020
5658	F. B. Hussein	Extended performance analysis of polyurethane-iron oxide nanocomposite for efficient removal of arsenic species from water	2017
5659	R. P. Liu	Review on heterogeneous oxidation and adsorption for arsenic removal from drinking water	2021
5660	S. Maji	Efficiency evaluation of arsenic(III) adsorption of novel graphene oxide@ iron-aluminium oxide composite for the contaminated water purification	2018
5661	D. D. La	Graphene-Supported Spinel CuFe2O4 Composites: Novel Adsorbents for Arsenic Removal in Aqueous Media	2017
5662	P. B. Hu	Research on the removal of As2O3 by gamma-Al2O3 adsorption based on density functional theory	2020
5663	E. Vences-Alvarez	Enhanced arsenic removal from water by a bimetallic material ZrOx-FeOx with high OH density	2020
5664	J. P. Stanoeva	LC/DAD/MS" and ICP-AES Assay and Correlations between Phenolic Compounds and Toxic Metals in Endemic Thymus alsarensis from the Thallium Enriched Allchar Locality	2017
5665	R. L. Wells	Aluminum-phosphorus chemistry - preparation and structural characterization of et2alp(sime3)2 2, et(cl)2al.p(sime3)3, and i-bu2(cl)al.p(sime3)3	1993
5666	H. B. Zhu	Refining mechanisms of arsenic in the hydrogen reduction process of tungsten oxide	2015
5667	M. S. Gao	Arsenic speciation transformation in soils with high geological background: New insights from the governing role of Fe	2022
5668	V. M. Mikoushkin	Arsenic Diffusion in the Natural Oxidation of the Heavily Defected GaAs Surface	2019

5669	C. M. Bulka	Arsenic in private well water and birth outcomes in the United States & nbsp;	2022
5670	M. Zhang	Comparison of Arsenic Adsorption on Goethite and Amorphous Ferric Oxyhydroxide in Water	2017
5671	C. C. H. Cheng	Polymer-collapsed iron oxide nanoparticles for arsenic uptake	2014
5672	D. Setyono	Metal oxide incorporated microcapsules for arsenic removal	2014
5673	S. Klejna	Decomposition of Metal Alkylamides, Alkyls, and Halides at Reducible Oxide Surfaces: Mechanism of 'Clean-up' During Atomic Layer Deposition of Dielectrics onto III-V Substrates	2014
5674	X. T. Liang	Efficient removal of arsenite through oxidation and adsorption on MWCNTs-decorated Ce-Mn binary oxide nanoparticles	2022
5675	H. Kim	Shifting the Specificity of E. coli Biosensor from Inorganic Arsenic to Phenylarsine Oxide through Genetic Engineering	2020
5676	H. C. Zhu	Role of cofilin-1 in arsenic trioxide-induced apoptosis of NB4-R1 cells	2020
5677	M. Naddaf	Structural and optical properties of electrochemically etched p(+)-type GaAs surfaces: influence of HF presence in the etching electrolyte	2017
5678	Y. M. Fan	Theoretical Study of As2O3 Adsorption Mechanisms on CaO surface	2019
5679	J. Y. Qi	Efficient removal of arsenic from water using a granular adsorbent: Fe-Mn binary oxide impregnated chitosan bead	2015
5680	S. Ashraf	Titanium-based nanocomposite materials for arsenic removal from water: A review	2019
5681	A. Maldonado-Reyes	Electro Coagulation Removal of As from Water: the Role of Phases Formation	2015
5682	R. Clough	Atomic spectrometry update: review of advances in elemental speciation	2020
5683	A. Musolff	Unexpected release of phosphate and organic carbon to streams linked to declining nitrogen depositions	2017
5684	M. T. Tye	An Adult Zebrafish Diet Contaminated with Chromium Reduces the Viability of Progeny	2018
5685	H. P. S. Chauhan	Synthesis, characterization, structural elucidation and biological screening of some bis(diisobutyldithiophosphato)antimony(III) complexes	2019
5686	I. Dhouib	Vibrational spectroscopy, electrical characterization, nonlinear optical properties and DFT calculation of (NEt4) (H2AsO4)(H3AsO4)(2)	2017

5687	H. A. Alhazmi	Determination of Phytocomponents of Twenty-one Varieties of Smokeless Tobacco using Gas Chromatography-Mass Spectroscopy (GC-MS)	2019
5688	K. N. Palansooriya	Soil amendments for immobilization of potentially toxic elements in contaminated soils: A critical review	2020
5689	L. Costa	Unveiling the Ambrotype: Characterization of Two 19th Century Photographs	2019
5690	M. S. Safarzadeh	Analysis and visualization of enargite and tennantite roasting using Cu-As-S-O system predominance volume diagrams	2018
5691	Y. Yoon	Synthesis of magnetite-nonoxidized graphene composite and application for arsenic removal: Comparison with magnetite-graphene oxide and magnetite-reduced graphene oxide	2014
5692	L. Monico	Development of a multi-method analytical approach based on the combination of synchrotron radiation X-ray micro-analytical techniques and vibrational micro-spectroscopy methods to unveil the causes and mechanism of darkening of "fake-gilded" decorations in a Cimabue	2022
5693	N. Y. Zhu	Arsenic immobilization through regulated ferrolysis in paddy field amendment with bismuth impregnated biochar	2019
5694	B. M. Cossairt	Molecular Gallium Arsenide Phosphide Clusters Prepared from AsP3, P-4, and {GaC(SiMe3)(3)}(4)	2010
5695	V. Weippert	High Thermoelectric Properties in the Sodalite Compounds BaGe8As14 and AGe(7)As(15) (A = Rb, Cs)	2021
5696	D. Afzali	Nano-iron oxide coated on sand as a new sorbent for removal of arsenic from drinking water	2016
5697	T. Taskin	Phenolic compounds, biological activities and trace elements of Capparis ovata var. canescens	2020
5698	F. N. Xu	Arsenic adsorption and removal by a new starch stabilized ferromanganese binary oxide in water	2019
5699	K. Liu	Electron shuttle-induced oxidative transformation of arsenite on the surface of goethite and underlying mechanisms	2022
5700	C. Liu	Pnictogens in medicinal chemistry: evolution from erstwhile drugs to emerging layered photonic nanomedicine	2021
5701	M. Lucia	Trace Element Concentrations in Relation to the Trophic Behaviour of Endangered Ivory Gulls (Pagophila eburnea) During Their Stay at a Breeding Site in Svalbard	2016
5702	N. Jiang	Responses of antioxidant enzymes and key resistant substances in perennial ryegrass (Lolium perenne L.) to cadmium and arsenic stresses	2022
5703	Y. Z. Zhao	The Characteristics of Zinc and Arsenic from Co-firing of Municipal Sewage Sludge with Biomass in a Fluidized Bed	2017
5704	S. R. Alley	Synthesis and characterisation of ferrocenyl-phosphonic and -arsonic acids	2001

5705	J. M. Yuan	Enhanced GRP78 protein expression via the IRE1 alpha/ASK1/p38 MAPK pathway during As2O3-induced endoplasmic reticulum stress in BEAS-2B cells	2021
5706	A. P. Panda	Synthesis of nanostructured copper oxide loaded boehmite (CuO_Boehmite) for adsorptive removal of As(III/V) from aqueous solution	2020
5707	Z. D. Wang	Taurine protected As2O3-induced the activation of hepatic stellate cells through inhibiting PPAR alpha-autophagy pathway	2019
5708	A. A. Fouad	Protective effect of telmisartan treatment against arsenic-induced testicular toxicity in rats	2015
5709	S. Q. Kong	Magnetic nanoscale Fe-Mn binary oxides loaded zeolite for arsenic removal from synthetic groundwater	2014
5710	J. Q. Jiang	Removal of Arsenic (III) from groundwater applying a reusable Mg-Fe-Cl layered double hydroxide	2015
5711	Y. X. Zhang	Arsenic pollution characteristics and variation trends in yangzonghai lake, china	2019
5712	Y. Glocheux	Adsorption study using optimised 3D organised mesoporous silica coated with Fe and Al oxides for specific As(III) and As(V) removal from contaminated synthetic groundwater	2014
5713	A. I. A. Sherlala	Adsorption of arsenic using chitosan magnetic graphene oxide nanocomposite	2019
5714	R. Clough	Atomic spectrometry updates. Review of advances in elemental speciation	2014
5715	K. Nakamoto	Arsenate and arsenite adsorbents composed of nano-sized cerium oxide deposited on activated alumina	2019
5716	X. D. Zhou	Large magneto-optical effects in hole-doped blue phosphorene and gray arsenene	2017
5717	D. Marescotti	High Content Screening Analysis to Evaluate the Toxicological Effects of Harmful and Potentially Harmful Constituents (HPHC)	2016
5718	E. Wachtler	Compounds of the types Pn(pyS)(3) (Pn = P, As, Bi; pyS: pyridine-2-thiolate) and Sb(pyS)xPh(3-x) (x=3-1); molecular structures and electronic situations of the Pn atoms	2021
5719	C. M. van Genuchten	Achieving arsenic concentrations of <1 mu g/L by Fe(0) electrolysis: The exceptional performance of magnetite	2020
5720	F. D. Klute	Characterization of dielectric barrier discharges for analytical chemistry	2018
5721	N. Li	A Vanadogermanate Dimer-Based Chain with Magnetic and Luminescent Properties	2016
5722	B. S. Rathi	A review on sources, identification and treatment strategies for the removal of toxic Arsenic from water system	2021

5723	K. Babaeivelni	Adsorption and removal of arsenic (V) using crystalline manganese (II,III) oxide: Kinetics, equilibrium, effect of pH and ionic strength	2014
5724	N. L. Nguyen	Ionomics and metabolomics analysis reveal the molecular mechanism of metal tolerance of Pteris vittata L. dominating in a mining site in Thai Nguyen province, Vietnam	
5725	A. Mallah	Separation and Determination of Chromium (III) Chromium (VI), Gold (III) and Arsenic (V) by Capillary Zone Electrophoresis Using 2-Acetylpyridine-4-phenylthiosemicarbazone as Complexing Reagent	2014
5726	N. Nocelli	Roles of Extracellular Polysaccharides and Biofilm Formation in Heavy Metal Resistance of Rhizobia	2016
5727	Q. Zheng	As(III) adsorption on Fe-Mn binary oxides: Are Fe and Mn oxides synergistic or antagonistic for arsenic removal?	2020
5728	R. K. Mishra	Discovery of novel Mnk inhibitors using mutation-based induced-fit virtual high-throughput screening	2019
5729	J. H. Jo	KML001, an arsenic compound, as salvage chemotherapy in refractory biliary tract cancers: A prospective study	2019
5730	R. Lopez	Speciation of organoarsenicals in aqueous solutions by Raman spectrometry and quantum chemical calculations	2022
5731	S. Kurwadkar	Groundwater pollution: Occurrence, detection, and remediation of organic and inorganic pollutants	2020
5732	D. J. V. Lopes	The Use of Chemical and Biological Agents in the Recovery of Heavy Metals from Treated Woods A Brief Review	2019
5733	M. Latorre	The bioleaching potential of a bacterial consortium	2016
5734	E. Shoham-Frider	Trace elements in striped dolphins (Stenella coeruleoalba) from the Eastern Mediterranean: A 10-years perspective	2016
5735	V. S. T. Ciminelli	Low arsenic bioaccessibility by fixation in nanostructured iron (Hydr)oxides: Quantitative identification of As-bearing phases	2018
5736	D. Prochazkova	The impact of increased soil risk elements on carotenoid contents	2014
5737	P. Maheswari	Impact of nano-ZnO consolidated poly (ether ether sulfone) nano filtration membrane for evacuation of hazardous metal particles	2022
5738	H. Y. Cheng	3D cross-point phase-change memory for storage-class memory	2019
5739	J. B. Vaney	Low-Temperature Transport Properties of Bi-Substituted beta-As2Te3 Compounds	2016
5740	X. Y. Li	Insight into the homogenous and heterogeneous transformation behavior of arsenic on commercial V2O5-WO3-TiO2 and novel gamma-Fe2O3 catalysts during selective catalytic reduction of NOx	2021

5741	M. Piesch	Synthesis of a Heterobimetallic Arsenic Triple-Decker Complex and Its Fragmentation Chemistry	2020
5742	T. A. Shestimerova	Metal-inorganic frameworks with pnictogen linkers	2018
5743	Y. L. Wang	Tuning the Structures of AsMo12 and AsW12 into Chiral Crystals by Introducing CH3CN and H2O	2015
5744	M. P. Garrido	Current Treatments and New Possible Complementary Therapies for Epithelial Ovarian Cancer	2022
5745	M. Czaplicka	Study of photodegradation and photooxidation of p-arsanilic acid in water solutions at pH=7: kinetics and by-products	2015
5746	C. Rajak	Metal toxicity and natural antidotes: prevention is better than cure	2020
5747	L. Li	Protective effects of oxymatrine against arsenic trioxideinduced liver injury	2017
5748	Y. Wang	Nickel-Refining Fumes Induced DNA Damage and Apoptosis of NIH/3T3 Cells via Oxidative Stress	2016
5749	R. C. Vineetha	L-Ascorbic Acid and alpha-Tocopherol Synergistically Triggers Apoptosis Inducing Antileukemic Effects of Arsenic Trioxide via Oxidative Stress in Human Acute Promyelocytic Leukemia Cells	2020
5750	H. Gill	Long-Term Outcome of Relapsed Acute Promyelocytic Leukemia Treated With Oral Arsenic Trioxide-Based Reinduction and Maintenance Regimens: A 15-Year Prospective Study	2018
5751	M. E. Moussa	The Potential of the Diarsene Complex (C5H5)(2)Mo-2(CO)(4)(mu,eta(2)-As-2) as a Connector Between Silver Ions	2020
5752	A. Cheepsattayakorn	Lung Cancer Chemotherapy, New Treatment and Related Patents	2014
5753	C. R. Majhi	Acetaminophen Increases the Risk of Arsenic-Mediated Development of Hepatic Damage in Rats by Enhancing Redox-Signaling Mechanism	2014
5754	L. Perillo	Quantification of Some Heavy Metals in Hair of Dairy Cows Housed in Different Areas from Sicily as a Bioindicator of Environmental Exposure-A Preliminary Study	2021
5755	V. Guzman-Fierro	Transformation of Roxarsone by bacterial consortium Isolated from soil in aerobic condition	2014
5756	H. G. Yao	Solvothermal syntheses and characterization of three new silver(I)/copper(I)-thioarsenates based on As2+/As3+ ions	2017
5757	E. Rukundo	Low-temperature precursors for zinc oxide nanomaterials for arsenic remediation	2014
5758	M. Orlando-Bonaca	Spatial and temporal distribution of trace elements in Padina pavonica from the northern Adriatic Sea	2021

5759	M. Kuprat	Synthesis and characterization of a stable non-cyclic bis(amino)arsenium cation	2018
5760	S. Krug	Portable X-ray fluorescence analysis of pesticides in the textile collection at the. German Historical Museum, Berlin	2014
5761	M. Sanchez-Alvarez	Sestrins as a Therapeutic Bridge between ROS and Autophagy in Cancer	2019
5762	L. D. Nghiem	Water reclamation and nitrogen extraction from municipal solid waste landfill leachate	2016
5763	O. Shpotyuk	Microstructure Hierarchical Model of Competitive e(+)-Ps Trapping in Nanostructurized Substances: from Nanoparticle-Uniform to Nanoparticle-Biased Systems	2017
5764	A. I. Isayev	Role of charged defects in the photoconductivity of Se95As5 chalcogenide glassy semiconductor with the EuF3 impurity	2014
5765	J. M. Wang	Synergistic toxic effect of arsenic and environmentally friendly metal oxide nanoparticles	2014
5766	D. D. Fu	Fabrication of alpha-FeOOH decorated graphene oxide-carbon nanotubes aerogel and its application in adsorption of arsenic species	2017
5767	Y. S. Zhu	Metals and molecular carcinogenesis	2020
5768	T. Grassl	Reactions of Polyarsenides with Acetylene: Synthesis and Characterization of Cs(18 crown-6) (2)As7C14H11 center dot 6NH(3) and As2C6H6	2015
5769	J. M. Hua	Adsorption of low-concentration arsenic from water by co-modified bentonite with manganese oxides and poly(dimethyldiallylammonium chloride)	2018
5770	A. Gomez-Tomas	Concentrations of trace elements and KRAS mutations in pancreatic ductal adenocarcinoma	2019
5771	D. Karthik	Chemometric identification of a few heavy metals, pesticides and plasticides in edible sunflower oil for health risk assessment	2018
5772	B. Lajin	Elution with 1,2-Hexanediol Enables Coupling of ICPMS with Reversed-Pase Liquid Chromatography under Standard Conditions	2022
5773	Y. J. Sun	Lattice thermal conductivity of monolayer AsP from first-principles molecular dynamics	2018
5774	D. N. Shan	Simultaneous and continuous stabilization of As and Cd in contaminated soil by a half wrapping-structured amendment	2021
5775	J. Q. Zheng	The syntheses and structures of a series of polyoxometalate-based metal-organic arsonates constructed from a dual-ligand strategy with organic arsenic acids and N-donor ligands	2019
5776	A. V. Zmozinski	Direct solid sample analysis with graphite furnace atomic absorption spectrometry-A fast and reliable screening procedure for the determination of inorganic arsenic in fish and seafood	2015

5777	B. C. Maki	The influence of hydrous ferric oxide, earthworms, and a hypertolerant plant on arsenic and iron bioavailability, fate, and transport in soils	2017
5778	D. Atzei	Determination of the limit of detection by X-ray photoelectron spectroscopy for As, Zn and Pb oxides in SiO2 matrix as model systems for environmental investigations	2016
5779	X. Zhao	The rescuable function and mechanism of resveratrol on As2O3-induced hERG K+ channel deficiency	2014
5780	Y. Wu	Meet-in-metabolite analysis: A novel strategy to identify connections between arsenic exposure and male infertility	2021
5781	B. Farkas	Aspergillus niger Decreases Bioavailability of Arsenic(V) via Biotransformation of Manganese Oxide into Biogenic Oxalate Minerals	2020
5782	C. M. Su	Arsenic removal from water by nanoscaled zerovalent iron and iron oxides	2016
5783	C. Y. Xu	Assessment of arsenic in colostrum and cord serum and risk exposure to neonates from an island population in China	2016
5784	I. de la Calle	Liquid-phase microextraction combined with graphite furnace atomic absorption spectrometry: A review	2016
5785	S. A. Krayuhin	Thermodynamic prediction of melting of copper-electrolyte slime	2016
5786	S. Mir	A Review on Recycling of End-of-Life Light-Emitting Diodes for Metal Recovery	2022
5787	L. Savage	Elevated Trimethylarsine Oxide and Inorganic Arsenic in Northern Hemisphere Summer Monsoonal Wet Deposition	2017
5788	N. Burford	Synthesis and characterization of bis(2,4,8-tris(trifluoromethyl)phenyl) derivatives of arsenic and antimony: X-ray crystal structures of As(R-F)(2)Cl, Sb(R-F)(2)Cl, and Sb(R-F)(2)OSO2CF3	2000
5789	P. B. Hu	Effects of O-2, SO2, H2O and CO2 on As2O3 adsorption by gamma-Al2O3 based on DFT analysis	2021
5790	X. Gu	Occurrence, migration, and allocation of arsenic in multiple media of a typical semi-enclosed bay	2020
5791	Y. S. Jun	Arsenopyrite-water interface chemistry: Roles of iron (hydr)oxides in arsenic mobility	2017
5792	X. H. Wu	Effects of Phosphate, Silicate, and Bicarbonate on Arsenopyrite Dissolution and Secondary Mineral Precipitation	2020
5793	W. R. Diephuis	The Effect of Agglomeration on Arsenic Adsorption Using Iron Oxide Nanoparticles	2022
5794	U. S. Rashid	Modeling arsenic removal by nanoscale zero-valent iron	2020

5795	M. Polizzotto	Quantifying the controls of manganese oxides on geogenic arsenic release to groundwater	2016
5796	H. Ji	Inhibition of transforming growth factor beta/SMAD signal by MiR-155 is involved in arsenic trioxide-induced anti-angiogenesis in prostate cancer	2014
5797	T. Sowers	Sorption of arsenic to biogenic iron (oxyhydr)oxides produced in circumneutral environments	2017
5798	X. X. Zhang	Separation behavior of arsenic and lead from antimony during vacuum distillation and zone refining	2020
5799	X. X. Zhao	A newly isolated indigenous metal-reducing bacterium induced Fe and Mn oxides synergy for enhanced in situ As(III/V) immobilization in groundwater	2022
5800	Y. Yu	Immunotoxic effect of arsenic trioxide on Caenorhabditis elegans	2016
5801	Y. K. Penke	Aluminum Substituted Cobalt Ferrite (Co-Al-Fe) Nano Adsorbent for Arsenic Adsorption in Aqueous Systems and Detailed Redox Behavior Study with XPS	2017
5802	H. M. Lu	Arsenic (III) induces oxidative stress and inflammation in the gills of common carp, which is ameliorated by zinc (II)	2021
5803	S. Miltonprabu	Diallyl trisulfide, a garlic polysulfide protects against As-induced renal oxidative nephrotoxicity, apoptosis and inflammation in rats by activating the Nrf2/ARE signaling pathway (Retracted article. See vol. 85, 2020)	2017
5804	T. S. Sakthivel	One-pot synthesis of a ceria-graphene oxide composite for the efficient removal of arsenic species	2017
5805	K. Baek	Oxalate based remediation of arsenic bound to amorphous Fe oxide in soil	2014
5806	I. Andjelkovic	Microwave-hydrothermal method for the synthesis of composite materials for removal of arsenic from water	2016
5807	Y. C. Lin	Association of plasma folate, vitamin B12 levels, and arsenic methylation capacity with developmental delay in preschool children in Taiwan	2019
5808	C. Navarathna	Removal of Arsenic from water using iron oxide precipitated Douglas fir biochar	2019
5809	P. Pourrezaei	Removal of organic compounds and trace metals from oil sands process-affected water using zero valent iron enhanced by petroleum coke	2014
5810	K. J. McDonald	Intrinsic properties of cupric oxide nanoparticles enable effective filtration of arsenic from water	2015
5811	C. Kim	Engineering superparamagnetic metal oxide nanocrystals for chromium and arsenic sorption, and separation	2015
5812	A. P. Panda	Enhanced performance of a core-shell structured Fe(0)@Fe oxide and Mn(0)@Mn oxide (ZVIM) nanocomposite towards remediation of arsenic contaminated drinking water	2020

5813	S. C. Chaudhry	A new method of synthesis of arsenic trithiophenoxide	1992
5814	A. Callegari	Assessment of arsenic removal efficiency by an iron oxide-coated sand filter process	2018
5815	A. M. Tondreau	Sodium phosphaethynolate, Na(OCP), as a "P" transfer reagent for the synthesis of N-heterocyclic carbene supported P-3 and PAsP radicals	2014
5816	M. S. Safarzadeh	Predominance Area Diagrams Bounding the Cu-As-S-O System's 3D Predominance Diagram at 900 K (627 degrees C)	2019
5817	S. Amirnia	Manganese-mediated immobilization of arsenic by calcifying macro-algae, Chara braunii	2019
5818	M. J. Kim	Concentrations, health risks, and sources of hazardous air pollutants in Seoul-Incheon, a megacity area in Korea	2021
5819	S. M. Habibi-Khorassan	Ionic strength effect on the kinetics and mechanism of N-vinyl compound formation in the presence of heterocyclic biological base: empirical and theoretical approaches	2021
5820	Y. Yoon	Comparative evaluation of magnetite-graphene oxide and magnetite-reduced graphene oxide composite for As(III) and As(V) removal	2016
5821	I. Langasco	Assessment and validation of ICP-MS and IC-ICP-MS methods for the determination of total, extracted and speciated arsenic. Application to samples from a soil-rice system at varying the irrigation method	2022
5822	E. Przezdzieczka	Arsenic chemical state in MBE grown epitaxial ZnO layers - doped with As, N and Sb	2016
5823	A. Seid-mohammadi	Kinetic study of real landfill leachate treated by non-thermal plasma (NTP) and granular sequential batch reactors (GSBR)	2021
5824	T. K. Das	Ultra-high arsenic adsorption by graphene oxide iron nanohybrid: Removal mechanisms and potential applications	2020
5825	J. J. Xie	Simultaneous removal of arsenic and antimony from mining wastewater	2020
5826	C. P. Zhang	Geochemical behavior of arsenic in reducing sulfidic sediments of reservoir contaminated by acid mine drainage	2014
5827	Z. N. Zhang	The fate of arsenic adsorbed on iron oxides in the presence of arsenite-oxidizing bacteria	2016
5828	R. Khanam	Prenatal Environmental Metal Exposure and Preterm Birth: A Scoping Review	2021
5829	Y. Yu	Anti-apoptotic and apoptotic pathway analysis of arsenic trioxide-induced apoptosis in human gastric cancer SGC-7901 cells	2014
5830	R. Balint	Defective Bismuth Oxide as Effective Adsorbent for Arsenic Removal from Water and Wastewater	2021

5831	P. P. Sun	Isolation and characterisation of Fe(II)-oxidising bacteria and their application in the removal of arsenic in an aqueous solution	
5832	I. Mihajlov	Arsenic contamination of Bangladesh aquifers exacerbated by clay layers	2020
5833	Y. Z. Liao	Experimental Study of Hepatic Artery Infusion of Vascular Endothelial Growth Factor Receptor (VEGFR)-2As2O3 Nanospheres for Targeted Therapy of Implanted Hep G2 Liver Cancer in Rats	2022
5834	S. Goldberg	Modeling inorganic arsenic adsorption by oxides, clay minerals, and soils using surface complexation models	2016
5835	Y. P. Dai	Effects of Chronic Exposure to Sodium Arsenite on Expressions of VEGF and VEGFR2 Proteins in the Epididymis of Rats	2017
5836	S. Akiya	Nanostructural characterization of large-scale porous alumina fabricated via anodizing in arsenic acid solution	2017
5837	R. Zowada	Porous Hydrogels Embedded with Hydrated Ferric Oxide Nanoparticles for Arsenate Removal	2019
5838	S. Alka	Arsenic removal technologies and future trends: A mini review	2021
5839	B. Moraga	Copolymer-hydrous zirconium oxide hybrid microspheres for arsenic sorption	2019
5840	M. L. Erickson	Months-long spike in aqueous arsenic following domestic well installation and disinfection: Short- and long-term drinking water quality implications	2021
5841	G. J. Qu	UiO-66(Zr)-derived t-zirconia with abundant lattice defect for remarkably enhanced arsenic removal	2022
5842	Y. Kang	Removing arsenic from groundwater in Cambodia using high performance iron adsorbent	2014
5843	E. M. Ahmed	Thermal Kinetics and Theoretical Third Order Nonlinear Optical Susceptibility of some As2O3 center dot V2O5 center dot FeO Glasses	2018
5844	N. G. Pech	Feasibility study of iron oxide nanoparticles prepared by different synthetic methods for arsenic removal	2015
5845	A. Yurum	Arsenic (V) removal from water by iron oxide/activated carbon system manufactured by microwave heating	2014
5846	S. Chowdhury	Alginate-based biotechnology: a review on the arsenic removal technologies and future possibilities	2019
5847	M. N. Pervez	alpha-FeOOH quantum dots impregnated graphene oxide hybrids enhanced arsenic adsorption: The mediation role of environmental organic ligands	2021
5848	N. Chaali	New insights into arsenic and cadmium distribution and origin in paddy soils using electrical resistivity tomography	2022

5849	Z. D. Zhang	Lactobacillus enshiensis sp. nov., a novel arsenic-resistant bacterium	2020
5850	M. H. Cao	Remediation of arsenic contaminated soil by coupling oxalate washing with subsequent ZVI/Air treatment	2016
5851	B. B. Zhang	Quantitative wPhenotyping-Based In Vivo Chemical Screening in a Zebrafish Model of Leukemia Stem Cell Xenotransplantation	2014
5852	S. K. Sanyal	Cycling of biogenic elements drives biogeochemical gold cycling	2019
5853	G. G. Briand	The relative stabilities of PhE(NH-t-Bu)(2) and PhE(mu-N-t-Bu)(2)EPh (E = As, Sb, and Bi): X-ray structures of {Li-2 PhAs(N-t-Bu)(2) }(2) and PhE(mu-N-t-Bu)(2)EPh (E = Sb, Bi)	2003
5854	A. Cuccaro	Sperm quality assessment in Ficopomatus enigmaticus (Fauvel, 1923): Effects of selected organic and inorganic chemicals across salinity levels	2021
5855	E. Rukundo	Influence of acid-base properties of zinc oxide nanomaterials on their arsenic uptake capacity	2015
5856	Y. T. Bao	Efficient removal of arsenic by electrodeposited CuFeOx foam	2021
5857	S. Ghosh	A comparative study on As(v) removal by graphene oxide (GO) and functionalized reduced graphene oxide (fRGO)	2019
5858	X. Ge	beta-FeOOH Nanorods/Carbon Foam-Based Hierarchically Porous Monolith for Highly Effective Arsenic Removal	2017
5859	S. Samanta	Recent trend in nanoparticle research in regulating arsenic bioaccumulation and mitigating arsenic toxicity in plant species	2021
5860	M. B. Black	Using gene expression profiling to evaluate cellular responses in mouse lungs exposed to V2O5 and a group of other mouse lung tumorigens and non-tumorigens	2015
5861	Q. L. Fu	Sorption of roxarsone onto soils with different physicochemical properties	2016
5862	T. K. Das	Comparative study of arsenic removal by iron-based nanomaterials: Potential candidates for field applications	2021
5863	P. X. Wang	Highly efficient removal of p-arsanilic acid with Fe(II)/peroxydisulfate under near-neutral conditions	2020
5864	P. Sharma	Efficient removal of arsenic from water using regenerated microfibrillated cellulose supported zinc oxide/hydroxide nanoparticles	2018
5865	H. L. Zhang	Adsorption performance and mechanism of the commonly used collectors with Oxygen-containing functional group on the ilmenite surface: A DFT study	2022
5866	S. Pirsa	Synthesis of Magnetic Gluten/Pectin/Fe3O4 Nano-hydrogel and Its Use to Reduce Environmental Pollutants from Lake Urmia Sediments	2020

5867	Y. S. Jun	Natural organic matter and arsenic create different reactive interfaces of iron(III) (hydr)oxide nanoparticles	2016
5868	P. Liu	Arsenic trioxide inhibits the activity of SphK1 by decreasing the level of phosphatidylserine and phosphatidic acid in the human gastric cancer cell line MGC-803	2022
5869	X. H. Lv	Arsenic trioxide combined with transarterial chemoembolization for primary liver cancer: A meta-analysis	2017
5870	P. K. Mishra	Ultrafast removal of arsenic using solid solution of aero-gel based Ce1-XTixO2-Y oxide nanoparticles	2019
5871	S. Mohammadian	In Situ Remediation of Arsenic-Contaminated Groundwater by Injecting an Iron Oxide Nanoparticle-Based Adsorption Barrier	2022
5872	H. Liu	The Application of Eco-Friendly Fe-Al Bimetallic Oxide/Biochar Adsorbent Composites with Waste Rice Husk for Removal of Arsenic at Low Concentration	2022
5873	J. L. Duan	Interactions between gold, thiol and As(iii) for colorimetric sensing	2020
5874	J. J. LeMonte	Sea Level Rise Induced Arsenic Release from Historically Contaminated Coastal Soils	2017
5875	W. Li	Extremely high arsenic removal capacity for mesoporous aluminium magnesium oxide composites	2016
5876	X. J. Liao	Occurrence of arsenic in fruit of mango plant (Mangifera indica L.) and its relationship to soil properties	2014
5877	Z. P. Wen	Redox transformation of arsenic by magnetic thin-film MnO2 nanosheet-coated flowerlike Fe3O4 nanocomposites	2017
5878	L. Ling	Visualizing Arsenate Reactions and Encapsulation in a Single Zero-Valent Iron Nanoparticle	2017
5879	J. S. Fischel	Kinetics of arsenic oxidation by manganese oxide minerals: The influence of origin and structure on reactivity	2014
5880	S. A. Hosseini	Adsorptive removal of arsenic from real sample of polluted water using magnetic GO/ZnFe2O4 nanocomposite and ZnFe2O4 nanospinel	2019
5881	J. Q. Sun	Preparation of Fe-Co based MOF-74 and its effective adsorption of arsenic from aqueous solution	2019
5882	S. S. Pradhan	Multifunctional Iron oxide embedded reduced graphene oxide as a versatile adsorbent candidate for effectual arsenic and dye removal	2020
5883	H. X. Son	Removal of arsenic from water using crumpled graphite oxide	2018
5884	S. Hassanpoor	Ultra-trace determination of arsenic species in environmental waters, food and biological samples using a modified aluminum oxide nanoparticle sorbent and AAS detection after multivariate optimization	2015

5885	M. Asadullah	Preparation of microporous activated carbon and its modification for arsenic removal from water	2014
5886	J. Rawson	Numerical Modeling of Arsenic Mobility during Reductive Iron-Mineral Transformations	2016
5887	S. I. Siddiqui	Promising prospects of nanomaterials for arsenic water remediation: A comprehensive review	2019
5888	J. Liu	Effects of copper oxide nanoparticles and arsenic on the whole-life growth of rice (Oryza sativa japonica)	2018
5889	C. M. Babu	Dihalogen Cross linked Fe3O4-Reduced Graphene Oxide Nanocomposites for Arsenic and Mercury Adsorption	2015
5890	S. H. Lee	Efficient Removal of Arsenic Using Magnetic Multi-Granule Nanoclusters	2014
5891	J. Perez	Organic-inorganic interpenetrated hybrids based on cationic polymer and hydrous, zirconium oxide for arsenate and arsenite removal	2016
5892	J. F. Li	Removal of trace arsenic to below drinking water standards using a Mn-Fe binary oxide	2017
5893	Y. K. Penke	Aluminum substituted nickel ferrite (Ni-Al-Fe): a ternary metal oxide adsorbent for arsenic adsorption in aqueous medium	2016
5894	R. C. Vineetha	L-Ascorbic Acid and -Tocopherol Reduces Hepatotoxicity Associated with Arsenic Trioxide Chemotherapy by Modulating Nrf2 and Bcl2 Transcription Factors in Chang liver Cells	2018
5895	H. Amal	Low Doses of Arsenic in a Mouse Model of Human Exposure and in Neuronal Culture Lead to S-Nitrosylation of Synaptic Proteins and Apoptosis via Nitric Oxide	2020
5896	L. L. Hao	A critical review on arsenic removal from water using iron-based adsorbents	2018
5897	V. M. Mikoushkin	Composition Depth Profiling of the GaAs Native Oxide Irradiated by an Ar+ Ion Beam	2018
5898	Y. Du	Nanoconfined hydrous titanium oxides with excellent acid stability for selective and efficient removal of As(V) from acidic wastewater	2020
5899	J. Yu	Single crystal growth and characterization of the 112-type iron-pnictide EuFeAs2	2018
5900	F. Jiang	Inhibition of TGF-beta/SMAD3/NF-kappa B signaling by microRNA-491 is involved in arsenic trioxide-induced anti-angiogenesis in hepatocellular carcinoma cells	2014
5901	S. H. Yoon	Re-examination of effects of sulfur treatment on Al2O3/InGaAs metal-oxide-semiconductor interface properties	2019
5902	A. K. Chandrashekhar	Contamination and mobilization of arsenic in the soil and groundwater and its influence on the irrigated crops, Manipur Valley, India	2016

5903	M. M. Rashid	Application of natural organic matter coated magnetic iron oxide nanoparticles for the remediation of arsenic and selenium	2017
5904	E. Gillispie	Investigating the role of sediment manganese oxides as a potential predictor for future arsenic contamination of groundwater	2017
5905	Y. Peng	Comparison of MoO3 and WO3 on arsenic poisoning V2O5/TiO2 catalyst: DRIFTS and DFT study	2016
5906	H. Gill	Oral arsenic trioxide incorporation into frontline treatment with all-trans retinoic acid and chemotherapy in newly diagnosed acute promyelocytic leukemia: A 5-year prospective study	2019
5907	D. Papadopoulos	A quantitative histopathological study of the effects of arsenic oxides on inflammation, demyelination and neurodegeneration in multiple sclerosis	2014
5908	R. Castaneda	Product Prediction: Intermediates Formed During Rare Earth Reactions	2014
5909	R. H. Li	Hydrous cerium oxides coated glass fiber for efficient and long-lasting arsenic removal from drinking water	2021
5910	B. J. Ouyang	Microbial reductive transformation of iron-rich tailings in a column reactor and its environmental implications to arsenic reactive transport in mining tailings	2019
5911	N. I. Gonzalez-Pech	Feasibility of iron-based sorbents for arsenic removal from groundwater	
5912	E. Easter	Risk assessment of soils identified on firefighter turnout gear	2016
5913	B. Kopp	Synergic toxic effects of food contaminant mixtures in human cells	2020
5914	Z. G. Liu	Role of Fe(III) in preventing humic interference during As(III) detection on gold electrode: Spectroscopic and voltammetric evidence	2014
5915	D. Stolpman	Decontamination of metals from firefighter turnout gear	2022
5916	W. Lorenc	Lc/icp-ms and complementary techniques in bespoke and nontargeted speciation analysis of elements in food samples	2022
5917	M. Kunaseth	DFT study of arsine adsorption on palladium doped graphene: Effects of palladium cluster size	2016
5918	H. Masuda	Arsenic cycling in the Earth's crust and hydrosphere: interaction between naturally occurring arsenic and human activities	2018
5919	T. Teshome	Phase Coexistence and Strain-Induced Topological Insulator in Two-Dimensional BiAs	2018
5920	Y. L. Chen	Stereoselective synthesis of trans-beta-methoxycarbonyl-gamma-aryl-gamma-butyrolactones	2002

5921	Y. C. Li	Utilization of red mud and Pb/Zn smelter waste for the synthesis of a red mud-based cementitious material	2018
5922	C. Zhang	Effect of biogas slurry and sucrose addition on electrokinetic removal of arsenic from paddy soil	
5923	A. Sigdel	Arsenic removal from aqueous solutions by adsorption onto hydrous iron oxide-impregnated alginate beads	2016
5924	W. B. Xin	Effect of rare earth Ce on the isothermal oxidation behavior in air of arsenic bearing steels	2019
5925	E. J. Kim	Selective recovery of ferrous oxalate and removal of arsenic and other metals from soil-washing wastewater using a reduction reaction	2019
5926	D. Raiser	Phosphine-Stabilized Pnictinidenes	2021
5927	P. R. Nair	Filomicelles Deliver a Chemo-Differentiation Combination of Paclitaxel and Retinoic Acid That Durably Represses Carcinomas in Liver to Prolong Survival	2018
5928	R. Sprocati	Charge interactions, reaction kinetics and dimensionality effects on electrokinetic remediation: A model-based analysis	2020
5929	S. Michlewska	Ruthenium metallodendrimers with anticancer potential in an acute promyelocytic leukemia cell line (HL60)	2017
5930	L. Shi	Arsenic trioxide inhibits cell growth and elevates T cell subgroup of bladder cancer in vitro and in vivo	2016
5931	R. C. Matos	Comparative Cr, As and CCA induced Cytostaticity in mice kidney: A contribution to assess CCA toxicity	2020
5932	Aliev, II	Synthesis and X-ray Powder Diffraction Study of Alloys in the As2Se3-InSe System	2019
5933	M. Grotti	Speciation analysis by small-bore HPLC coupled to ICP-MS	2014
5934	A. Gupta	Triorganoarsenic(V) compounds with internally functionalized oximes: synthetic and spectroscopic aspects of R3As(Cl)L , R3As(OH)L and R3AsL2 : crystal and molecular structure of (Pr3AsOH)-As-i Cl-+(-)	2003
5935	Y. Teng	Biodegradation of pentachloronitrobenzene by Cupriavidus sp YNS-85 and its potential for remediation of contaminated soils	2017
5936	A. M. Reyes-Salas	Petrographic, geochemical and mineralogical study of the San Jose de Lourdes fulgurite, Zacatecas, Mexico	2017
5937	O. A. Adebambo	Cadmium disrupts signaling of the hypoxia-inducible (HIF) and transforming growth factor (TGF-beta) pathways in placental JEG-3 trophoblast cells via reactive oxygen species	2018
5938	J. Markovski	Improving arsenic sorption capacity by doping metal (hydr)oxide nano-enabled hybrid media with more electronegative transition metal	2017

5939	S. F. Taghizadeh	Polycyclic aromatic hydrocarbons, pesticides, and metals in olive: analysis and probabilistic risk assessment	2021
5940	H. M. Alayan	Hybridizing carbon nanomaterial with powder activated carbon for an efficient removal of Bisphenol A from water: the optimum growth and adsorption conditions	2017
5941	Y. Liu	KML001 and doxercalciferol induce synergistic antileukemic effect in acute lymphoid leukemia cells	2017
5942	Z. Diana	Plastic pellets trigger feeding responses in sea anemones	2020
5943	X. X. Zhao	Synergy of Fe and biogenic Mn oxide components mediated by a newly isolated indigenous bacterium to enhance As(III/V) immobilization in groundwater	2022
5944	R. Muthaiah	Thermal conductivity of magnesium selenide (MgSe)-A first principles study	2021
5945	S. R. Kanel	The use of carbon nanotube yarn as a filter medium to treat nitroaromatic-contaminated water	2016
5946	M. Honda	Study on Chromium Accumulation Mechanism of Chinese Break Fern (Pteris Vittata L.) by Synchrotron Radiation X-ray Fluorescence Analysis	2015
5947	N. Antonatos	Black arsenic: a new synthetic method by catalytic crystallization of arsenic glass	2020
5948	F. Aslam	Essential Gene Clusters Identified in Stenotrophomonas MB339 for Multiple Metal/Antibiotic Resistance and Xenobiotic Degradation	2018
5949	P. M. Nuccio	Pollution of waters and soils by contaminants of magmatic origin	2016
5950	J. H. Fang	Bacterially mediated release and mobilization of As/Fe coupled to nitrate reduction in a sediment environment	2021
5951	S. Khan	Biosynthesized Iron Oxide Nanoparticles (Fe3O4 NPs) Mitigate Arsenic Toxicity in Rice Seedlings	2021
5952	I. Jurgelane	Shungite application for treatment of drinking water - is it the right choice?	2021
5953	H. A. Al-Sanad	Properties and behavior of the dust fallout in the State of Kuwait	2018
5954	U. Kristan	Assessment of pollution level using Mytilus galloprovincialis as a bioindicator species: The case of the Gulf of Trieste	2014
5955	F. Winter	Structure and solid-state NMR spectroscopy of the ternary pnictides Li3LaX2 (X = P, As, Sb, Bi)	2014
5956	P. G. Y. Garcia	Synthesis and multinuclear (H-1 C-13, P-31) NMR studies of arsocanes substituted with a cyclic dithiophosphate ligand. Molecular structure of O(CH2CH2S)(2)AsS2P(OCH2)(2)CEt2	2002

5957	J. A. Rodriguez-Jimenez	Computational study of GanAsm (m plus n=2-9) clusters using DFT calculations	2019
5958	S. J. Kim	Antitumoral effect of arsenic compound, sodium metaarsenite (KML001), on multiple myeloma cells	2017
5959	M. Flores-Galvan	Genotoxic Assessment of Some Inorganic Compounds in Desert Pupfish (Cyprinodon macularius) in the Evaporation Pond from a Geothermal Plant	2017
5960	S. Bibi	Occurrence and methods to remove arsenic and fluoride contamination in water	2017
5961	T. Y. Nayir	Container washing wastewater treatment by combined electrocoagulation-electrooxidation	2018
5962	X. Y. Wang	The influence of redox conditions on aqueous-solid partitioning of arsenic and selenium in a closed coal ash impoundment	2022
5963	M. H. H. Fischel	The influence of environmental conditions on kinetics of arsenite oxidation by manganese-oxides	2015
5964	M. Vyssotski	Phospholipids of New Zealand Edible Brown Algae	2017
5965	S. Aacharya	Adsorption Behavior of Arsenic to an Isolated Ferric Ion Combined on Chelate Resin	2017
5966	X. X. Peng	Roles of humic substances redox activity on environmental remediation	2022
5967	A. G. Karydas	Handheld XRF analysis of the old Mexican feather headdress in the Weltmuseum Vienna	2014
5968	R. C. Longo	Mechanism of Arsenic Monolayer Doping of Oxide-Free Si(111)	2016
5969	T. Mansouri	The effects of hematite nanoparticles on phytoavailability of arsenic and corn growth in contaminated soils	2017
5970	S. B. Huang	Multivariate analysis of the heterogeneous geochemical processes controlling arsenic enrichment in a shallow groundwater system	2014
5971	A. Kumar	Remediation of Arsenic by Metal/ Metal Oxide Based Nanocomposites/ Nanohybrids: Contamination Scenario in Groundwater, Practical Challenges, and Future Perspectives	2021
5972	A. Haider	Detection of trace amount of arsenic in groundwater by laser-induced breakdown spectroscopy and adsorption	2014
5973	D. Li	Circular RNAs as biomarkers and therapeutic targets in environmental chemical exposure-related diseases	2020
5974	J. Burt	Coordination chemistry of the main group elements with phosphine, arsine and stibine ligands	2014

5975	M. D. Dutton	Towards an exposure narrative for metals and arsenic in historically contaminated Ni refinery soils: Relationships between speciation, bioavailability, and bioaccessibility	2019
5976	S. H. Al-Rekabi	Hydrous ferric oxide-magnetite-reduced graphene oxide nanocomposite for optical detection of arsenic using surface plasmon resonance	2019
5977	R. Garla	Effect of Zinc on Hepatic and Renal Tissues of Chronically Arsenic Exposed Rats: A Biochemical and Histopathological Study	2021
5978	J. L. Crane	Ambient sediment quality conditions in Minnesota lakes, USA: Effects of watershed parameters and aquatic health implications	2017
5979	M. E. Ortiz-Santaliestr	Pollutant accumulation patterns in nestlings of an avian top predator: biochemical and metabolic effects	2015
5980	H. P. Wang	Effect of Steel-refractory Reactions on Removal of Arsenic from Molten Steel with Lanthanum Additions	2020
5981	S. Sarpong-Kumanko	Organ damage by toxic metals is critically determined by the bloodstream	2018
5982	C. W. Neil	Water Chemistry Impacts on Arsenic Mobilization from Arsenopyrite Dissolution and Secondary Mineral Precipitation: Implications for Managed Aquifer Recharge	2014
5983	B. D. Kocar	Deciphering and predicting spatial and temporal concentrations of arsenic within the Mekong Delta aquifer	2014
5984	T. Wan	Arsenic accumulation and chemical fraction distribution changes in sludge in a low-sludge wastewater treatment system	2014
5985	S. Y. Yang	Status assessment and probabilistic health risk modeling of metals accumulation in agriculture soils across China: A synthesis	2019
5986	B. Knopf	Seasonal variability in metal and metalloid burdens of mussels: using data from the German Environmental Specimen Bank to evaluate implications for long-term mussel monitoring programs	2020
5987	A. B. Alkhasov	Comprehensive Utilization of Low-Potential Geothermal Waters of Southern Russia for Heat and Water Supply and Solution of Environmental Problems	2019
5988	A. M. S. de la Campa	2009-2017 trends of PM10 in the legendary Riotinto mining district of SW Spain	2020
5989	M. M. El-Moselhy	Synthesis and characterization of hybrid iron oxide silicates for selective removal of arsenic oxyanions from contaminated water	2017
5990	J. M. Hua	Synthesis and characterization of bentonite based inorgano-organo-composites and their performances for removing arsenic from water	2015
5991	J. Zhang	Potential molecular mechanisms underlying the effect of arsenic on angiogenesis	2019
5992	V. K. Rathore	Competitive Adsorption of Arsenic and Fluoride onto Economically Prepared Aluminum Oxide/Hydroxide Nanoparticles: Multicomponent Isotherms and Spent Adsorbent Management	2017

5993	C. Qin	Mesoporous Magnetic Ferrum-Yttrium Binary Oxide: a Novel Adsorbent for Efficient Arsenic Removal from Aqueous Solution	2016
5994	S. Randar	Adsorption of arsenic (V) from aqueous solution using modified saxaul ash: isotherm and thermodynamic study	2019
5995	G. L. Di	Simultaneous removal of several pharmaceuticals and arsenic on Zn-Fe mixed metal oxides: Combination of photocatalysis and adsorption	2017
5996	A. Ghorbani	Nitrate Reductase is Needed for Methyl Jasmonate-Mediated Arsenic Toxicity Tolerance of Rice by Modulating the Antioxidant Defense System, Glyoxalase System and Arsenic Sequestration Mechanism	
5997	E. Araya	Physical and chemical characterization of iron (III) oxide produced by SORAS technique for the removal of arsenic in drinking water	2017
5998	L. M. Yang	A competitive coordination-based immobilization-free electrochemical biosensor for highly sensitive detection of arsenic(v) using a CeO2-DNA nanoprobe	2020
5999	Y. H. Zhang	Two-stage leaching of zinc and copper from arsenic-rich copper smelting hazardous dusts after alkali leaching of arsenic	2019
6000	J. S. Choi	Fabrication of chitosan/graphene oxide-gadolinium nanorods as a novel nanocomposite for arsenic removal from aqueous solutions	2020
6001	T. Garcia-Rodriguez	Arsenic and lead bioaccessibilities measured with a novel reactor system using the mexican standard and pbet methods: comparison with in vivo and in vitro reported data	2021
6002	M. Zhu	Solid-solution partitioning and thionation of diphenylarsinic acid in a flooded soil under the impact of sulfate and iron reduction	2016
6003	A. Pillai	Towards developing a low-cost gravity-driven arsenic filtration system using iron oxide nanoparticle-loaded PU foam	2020
6004	W. C. Yang	Distribution and Behavior of Arsenic During the Reducing-Matting Smelting Process	2017
6005	G. K. Das	Ultra-long magnetic nanochains for highly efficient arsenic removal from water	2014
6006	X. Y. Li	Comprehensive insight into the role of HCl on arsenic capture by CaO during coal combustion: A combined experimental and theoretical study	2022
6007	F. J. Perez-Vazquez	Concentrations of persistent organic pollutants (POPs) and heavy metals in soil from San Luis Potosi, Mexico	2015
6008	D. Y. Xu	Coresistance to Benzalkonium Chloride Disinfectant and Heavy Metal Ions in Listeria monocytogenes and Listeria innocua Swine Isolates from China	2019
6009	P. V. Ioannou	The Reaction of Bunsen's Cacodyl Disulfide, Me2As(S)-S-AsMe2, with Iodine: Preparation and Properties of Dimethylarsinosulfenyl Iodide, Me2As-S-I	2015
6010	H. Zhi	Arsenic(V) Removal from Drinking Water by Polyaluminum Chloride in a Sand Filter Medium	2017

6011	P. T. L. Huong	Functional manganese ferrite/graphene oxide nanocomposites: effects of graphene oxide on the adsorption mechanisms of organic MB dye and inorganic As(V) ions from aqueous solution	2018
6012	K. Babaeivelni	Removal of arsenic from water using manganese (III) oxide: Adsorption of As(III) and As(V)	2016
6013	B. J. Sengupta	A simplified method for determination of contents of arsenic in ground water from major oxide element of iron by regression analysis	2017
6014	S. Ushakov	Meeting future emission regulation at sea by combining low-pressure EGR and seawater scrubbing	2020
6015	L. P. Lingamdinne	Potential of the magnetic hollow sphere nanocomposite (graphene oxide-gadolinium oxide) for arsenic removal from real field water and antimicrobial applications	2021
6016	P. Montoro-Leal	Semiautomatic method for the ultra-trace arsenic speciation in environmental and biological samples via magnetic solid phase extraction prior to HPLC-ICP-MS determination	2021
6017	D. Morillo	Efficient arsenic(V) and arsenic(III) removal from acidic solutions with Novel Forager Sponge-loaded superparamagnetic iron oxide nanoparticles	2015
6018	M. A. Mansour	Combination of arsenic trioxide and cisplatin synergistically inhibits both hexokinase activity and viability of Ehrlich ascites carcinoma cells	2019
6019	Z. P. Gao	Unraveling influences of nitrogen cycling on arsenic enrichment in groundwater from the Hetao Basin using geochemical and multi-isotopic approaches	2021
6020	M. F. Ahmed	Hybrid Beads of Zero Valent Iron Oxide Nanoparticles and Chitosan for Removal of Arsenic in Contaminated Water	2021
6021	R. Siddiqui	Crocodiles and alligators: Antiamoebic and antitumor compounds of crocodiles	2017
6022	D. Predoi	Removal and Oxidation of As(III) from Water Using Iron Oxide Coated CTAB as Adsorbent	2020
6023	V. Urrialde	The Pho4 transcription factor mediates the response to arsenate and arsenite in Candida albicans	2015
6024	R. C. Smith	Nexus between polymer support and metal oxide nanoparticles in hybrid nanosorbent materials (HNMs) for sorption/desorption of target ligands	2015
6025	S. Dudek	Enhanced Arsenic(V) Removal on an Iron-Based Sorbent Modified by Lanthanum(III)	2020
6026	R. Singh	Interplay of Calcium and Nitric Oxide in improvement of Growth and Arsenic-induced Toxicity in Mustard Seedlings (vol 10, 6900, 2020)	2020
6027	I. Andjelkovic	Graphene Aerogels Decorated with alpha-FeOOH Nanoparticles for Efficient Adsorption of Arsenic from Contaminated Waters	2015
6028	B. Lajin	Fluoroalkylamines: Novel, Highly Volatile, Fast-Equilibrating, and Electrospray Ionization-Mass Spectrometry Signal-Enhancing Cationic Ion-Interaction Reagents	2020

6029	F. S. Guo	Main Group Chemistry at the Interface with Molecular Magnetism	2019
6030	F. Ju	Bioreactor microbial ecosystems with differentiated methanogenic phenol biodegradation and competitive metabolic pathways unraveled with genome-resolved metagenomics	2018
6031	M. Fleischmann	A comparative study of the coordination behavior of cyclo-P-5 and cyclo-As-5 ligand complexes towards the trinuclear Lewis acid complex (perfluoro-ortho-phenylene)mercury	2015
6032	A. Dogan	Evaluation of the in vitro and in vivo effects of the ethanolic lyophilized extract obtained from pholiota aurivella mushroom on cci4-induced toxicity in rats	2019
6033	B. J. Yates	Engineering aspects of ferrate in water and wastewater treatment - a review	2014
6034	M. Khatamian	Efficient removal of arsenic using graphene-zeolite based composites	2017
6035	C. W. Neil	Fractal aggregation and disaggregation of newly formed iron(III) (hydr)oxide nanoparticles in the presence of natural organic matter and arsenic	2016
6036	P. H. Rao	Preparation and application of amorphous Fe-Ti bimetal oxides for arsenic removal	2015
6037	V. Ryzhi	Far-infrared photodetectors based on graphene/black-AsP heterostructures	2020
6038	M. Tramsek	Crystal structure determination of Pb2F2(HF)(SbF6)(2), PbFSbF6 and Ba(HF)(AF(6))(2): (A = As, Sb)	2015
6039	C. S. Cao	Understanding Periodic and Non-periodic Chemistry in Periodic Tables	2021
6040	T. Dordevic	Hydrothermal and ionothermal synthesis of mineral-related arsenates in the system CdO-MO-As2O5 (M2+ = Mg, Co, Ni, Cu, Zn) and their crystal structures	2017
6041	E. Bunin	Study of Arsenic-Contaminated Soil Bacterial Community Using Biochip Technology	2020
6042	S. Deshmukh	Red Mud-Reduced Graphene Oxide Nanocomposites for the Electrochemical Sensing of Arsenic	2020
6043	H. Afroz	Inhibition of Microbial Methylation via arsM in the Rhizosphere: Arsenic Speciation in the Soil to Plant Continuum	2019
6044	P. Singh	Fabrication of Economical Thiol-Tethered Bifunctional Iron Composite as Potential Commercial Applicant for Arsenic Sorption Application	2018
6045	M. Thakkar	Synthesis of diatom-FeOx composite for removing trace arsenic to meet drinking water standards	2015
6046	M. E. Moussa	Mixed Organometallic-Organic Hybrid Assemblies Based on the Diarsene Complex Cp2Mo2(CO)(4)(mu,eta(2)-As-2) , Ag-I Salts and N-Donor Organic Molecules	2021

6047	S. Byrne	Persistent Organochlorine Pesticide Exposure Related to a Formerly Used Defense Site on St. Lawrence Island, Alaska: Data from Sentinel Fish and Human Sera	2015
6048	Y. Wang	Release and transformation of arsenic from As-bearing iron minerals by Fe-reducing bacteria	2016
6049	Y. Ji	Fate and chemical speciation of antimony (Sb) during uptake, translocation and storage by rye grass using XANES spectroscopy	2017
6050	L. El Alaoui	Lead, zinc and arsenic contamination of pit lake waters in the Zeida abandoned mine (High Moulouya, Morocco)	2021
6051	J. Gorny	Redox behaviour of arsenic in the surface sediments of the Marque River (Northern France)	2018
6052	M. Mohsennia	Thermodynamic and kinetic studies of As2O3 toxicological effects on human insulin in generation diabetes mellitus	2017
6053	H. Rehman	SYNTHESIS AND CHARACTERIZATION OF ZnO NANOPARTICLES AND THEIR USE AS AN ADSORBENT FOR THE ARSENIC REMOVAL FROM DRINKING WATER	2019
6054	T. S. Peixe	Occupational exposure profile of Pb, Mn, and Cd in nonferrous Brazilian sanitary alloy foundries	2014
6055	W. R. Cullen	Chemical Mechanism of Arsenic Biomethylation	2014
6056	Y. Wang	Design of Scorodite@Fe3O4 Core-Shell Materials and the Fe3O4 Shell Prevents Leaching of Arsenic from Scorodite in Neutral and Alkaline Environments	2019
6057	M. Glodowska a	Arsenic mobilization by anaerobic iron-dependent methane oxidation	2020
6058	Y. H. Wang	Arsenic(III) and Arsenic(V) Speciation during Transformation of Lepidocrocite to Magnetite	2014
6059	M. M. Rashid	Study of the photochemical and adsorptive properties of NOM grafted iron oxide nanoparticles for the potential remediation of toxic arsenic from water	2018
6060	T. Davydiuk	Removal of inorganic arsenic from water using metal organic frameworks	2020
6061	C. Peng	Micronucleus formation by single and mixed heavy metals/loids and PAH compounds in HepG2 cells	2015
6062	Y. L. Kong	Influence of humic acid on the removal of arsenate and arsenic by ferric chloride: effects of pH, As/Fe ratio, initial As concentration, and co-existing solutes	2017
6063	J. Park	Effect of neutralizing agents on bioaccessibility in the process of in situ stabilization for arsenic contaminated soil by co-precipitation with iron oxides	2019
6064	E. B. Simsek	Equilibrium arsenic adsorption onto metallic oxides : Isotherm models, error analysis and removal mechanism	2014

6065	W. A. H. Altowayti	Removal of arsenic from wastewater by using different technologies and adsorbents: a review	
6066	B. Chen	One-pot, solid-phase synthesis of magnetic multiwalled carbon nanotube/iron oxide composites and their application in arsenic removal	2014
6067	K. Keune	Analytical imaging studies of the migration of degraded orpiment, realgar, and emerald green pigments in historic paintings and related conservation issues	2016
6068	A. T. Sonne	Assessing the chemical contamination dynamics in a mixed land use stream system	2017
6069	E. M. Farrow	Reducing arsenic accumulation in rice grain through iron oxide amendment	2015
6070	N. Zhu	Investigating photo-driven arsenics' behavior and their glucose metabolite toxicity by the typical metallic oxides in ambient PM2.5	2020
6071	M. S. Ko	Reductive dissolution and sequestration of arsenic by microbial iron and thiosulfate reduction	2019
6072	E. Richardson	Assessing the Impact of LED Lighting on the Stability of Selected Yellow Paint Formulations	2020
6073	Y. X. Huang	Removal of Arsenic and Phosphate from Aqueous Solution by Metal (Hydr-)oxide Coated Sand	2014
6074	J. Torres	Solution Chemistry of Arsenic Anions in the Presence of Metal Cations	2017
6075	H. D. Watts	Arsenic Adsorption onto Minerals: Connecting Experimental Observations with Density Functional Theory Calculations	2014
6076	A. Y. Zhang	Heterogeneous Fenton decontamination of organoarsenicals and simultaneous adsorption of released arsenic with reduced secondary pollution	2018
6077	S. Rezania	Nitrile-calixarene grafted magnetic graphene oxide for removal of arsenic from aqueous media: Isotherm, kinetic and thermodynamic studies	2021
6078	C. M. McCann	In situ arsenic oxidation and sorption by a Fe-Mn binary oxide waste in soil	2018
6079	K. A. Rychlik	Environmental exposures during pregnancy: Mechanistic effects on immunity	2019
6080	B. K. Kang	Efficient removal of arsenic by strategically designed and layer-by-layer assembled PS@+rGO@GO@Fe3O4 composites	2017
6081	M. Kumar	Effect of binary zinc-magnesium oxides on polyphenylsulfone/cellulose acetate derivatives hollow fiber membranes for the decontamination of arsenic from drinking water	2021
6082	C. Z. Hu	Coagulation of methylated arsenic from drinking water: Influence of methyl substitution	2015

6083	F. Tan	Development of cerium oxide-based diffusive gradients in thin films technique for in-situ measurement of dissolved inorganic arsenic in waters	2019
6084	A. D. Gupta	Silica derived from rice husk ash and loaded with iron oxide for As(III) adsorption from water: experimental and modelling studies	
6085	M. Martinez-Cabanas	Green synthesis of iron oxide nanoparticles. Development of magnetic hybrid materials for efficient As(V) removal	2016
6086	L. E. Rios-Saldana	Synthesis of a granular composite based on polyvinyl alcohol-Fe:Ce bimetallic oxide particles for the selective adsorption of As(V) from water	2022
6087	S. K. Wu	Simultaneous degradation of p-arsanilic acid and inorganic arsenic removal using M-rGO/PS Fenton-like system under neutral conditions	2020
6088	R. Z. Hu	Engineering 2D Arsenic-Phosphorus Theranostic Nanosheets	2021
6089	H. Y. Lu	Characterization of Extractable Metals from the Aquifers with Arsenic Contamination in the Tsengwen Creek, Taiwan	2014
6090	D. D. Wu	Extracellular signal-regulated kinase 8-mediated NF-kappa B activation increases sensitivity of human lung cancer cells to arsenic trioxide	2017
6091	C. P. Liu	Arsenic availability in rice from a mining area: Is amorphous iron oxide-bound arsenic a source or sink?	2015
6092	Z. Q. Huang	Comparison of the effects of competitive adsorption and reductive dissolution on migration of arsenic in lake sediment	2021
6093	M. A. Deyssenroth	Placental Gene Transcript Proportions are Altered in the Presence of In Utero Arsenic and Cadmium Exposures, Genetic Variants, and Birth Weight Differences	2022
6094	N. Chen	Simulated solar light driven roxarsone degradation and arsenic immobilization with hematite and oxalate	2020
6095	P. Shukla	Nitric oxide mitigates arsenic-induced oxidative stress and genotoxicity in Vicia faba L	2015
6096	I. Jacukowicz-Sobala	Evaluation of hybrid anion exchanger containing cupric oxide for As(III) removal from water	2019
6097	E. B. Simsek	Factorial design analysis of As(V) adsorption onto iron-aluminum binary oxide-doped clinoptilolite	2014
6098	Y. S. Han	Arsenic removal characteristics of natural Mn-Fe binary coating on waste filter sand from a water treatment facility	2022
6099	A. M. Channa	Equilibrium, kinetics and thermodynamic studies for the removal of arsenic from water using newly synthesized amino resin supported hydrous ferric oxide nano composite	2022
6100	S. Saha	Sublethal Effect of Arsenic on Oxidative Stress and Antioxidant Status in Scylla serrata	2014

6101	M. Turpin	Lead, chromium, and arsenic accumulation associated with naturally accumulating iron-manganese oxide coatings on in-situ stream substrates comparing geothermal and non-geothermal waters	2017
6102	S. G. Wu	Stripping Analysis of Trace Arsenic Based on the MnOx/AuNPs Composite Film Modified Electrode in Alkaline Media	2014
6103	J. C. Bullen	Improved accuracy in multicomponent surface complexation models using surface-sensitive analytical techniques: Adsorption of arsenic onto a TiO2/Fe2O3 multifunctional sorbent	2020
6104	X. Y. Wei	Reusable electrospun carbon nanofiber composite for selective removal of inorganic arsenic species in water	2021
6105	D. Ghosh	Role of iron slime in modifying soil factors to mitigate arsenic in contaminated soils	2020
6106	S. X. Ye	Da-KGM based GO-reinforced FMBO-loaded aerogels for efficient arsenic removal in aqueous solution	2017
6107	F. Firdaus	Ellagic acid mitigates arsenic-trioxide-induced mitochondrial dysfunction and cytotoxicity in SH-SY5Y cells	2018
6108	S. Owings	Development of a rate law for arsenite oxidation by manganese oxides to assess the impact of the recycling of arsenic on microbial respiration processes	2015
6109	L. Valeri	The Joint Effect of Prenatal Exposure to Metal Mixtures on Neurodevelopmental Outcomes at 20-40 Months of Age: Evidence from Rural Bangladesh	2017
6110	X. W. Xu	Control of arsenic mobilization in paddy soils by manganese and iron oxides	2017
6111	T. Huang	Electrokinetic removals of arsenate and arsenite from the aqueous environment by a fluidized bed of superparamagnetic iron oxide nanoparticle-coated pyrite microelectrodes	2021
6112	A. Y. Cetinkaya	Performance and mechanism of direct As(III) removal from aqueous solution using low-pressure graphene oxide-coated membrane	2018
6113	T. S. Owen	Active Site Targeting of Hedgehog Precursor Protein with Phenylarsine Oxide	2015
6114	S. Khuntia	Adsorption of As(V) on zirconium-based adsorbents	2016
6115	Y. Wang	Arsenite and arsenate leaching and retention on iron (hydr)oxide-coated sand column	2016
6116	M. Watson	Repurposing spent filter sand from iron and manganese removal systems as an adsorbent for treating arsenic contaminated drinking water	2022
6117	D. Ocinski	Optimization of hybrid polymer preparation by ex situ embedding of waste Fe/Mn oxides into chitosan matrix as an effective As(III) and As(V) sorbent	2019
6118	M. Q. Shi	Raman and FTIR spectra of modified iron phosphate glasses containing arsenic	2015

6119	Y. Mustapha Kamil	Arsenic Detection Using Surface Plasmon Resonance Sensor With Hydrous Ferric Oxide Layer	2022
6120	D. H. K. Reddy	Characterization of binary oxide photoactive material and its application for inorganic arsenic removal	2014
6121	E. C. Fru	Arsenic-induced phosphate limitation under experimental Early Proterozoic oceanic conditions	2016
6122	Q. Wang	Bifunctional magnesium oxide crystal successively as adsorbent and matrix modifier for preconcentration and determination of arsenic by graphite furnace atomic absorption spectrometry	2017
6123	J. Yang	Comparison among soil additives for enhancing Pteris vittata L.: Phytoremediation of As-contaminated soil	2018
6124	M. S. Reid	Arsenic speciation analysis: A review with an emphasis on chromatographic separations	2020
6125	A. S. Horvath	Manganese-reducing Pseudomonas fluorescens-group bacteria control arsenic mobility in gold mining-contaminated groundwater	2014
6126	L. N. Lin	Removal and Oxidation of Arsenic from Aqueous Solution by Biochar Impregnated with Fe-Mn Oxides	2019
6127	S. E. H. Mehdi	Sources, chemistry, bioremediation and social aspects of arsenic-contaminated waters: a review	2021
6128	S. A. E. Bashandy	Antioxidant Potential of Spirulina platensis Mitigates Oxidative Stress and Reprotoxicity Induced by Sodium Arsenite in Male Rats	2016
6129	S. Y. Gu	Novel Dielectric Barrier Discharge Trap for Arsenic Introduced by Electrothermal Vaporization: Possible Mechanism and Its Application	2021
6130	S. Shahrin	Adsorptive Removal of As(V) Ions from Water using Graphene Oxide-Manganese Ferrite and Titania Nanotube-Manganese Ferrite Hybrid Nanomaterials	2018
6131	A. G. Nastovjak	Reasons of Crystallite Formation during the Self-Catalyzed GaAs Nanowire Growth	2020
6132	K. Yang	Water leaching of arsenic trioxide from metallurgical dust with emphasis on its kinetics	2019
6133	X. P. Huang	PRMT5-mediated RNF4 methylation promotes therapeutic resistance of APL cells to As2O3 by stabilizing oncoprotein PML-RAR alpha	2022
6134	S. A. Baig	Arsenic Removal from Natural Water Using Low Cost Granulated Adsorbents: A Review	2015
6135	X. T. Sun	Simultaneous suppression of magnetic nanoscale powder and fermented bark amendment for arsenic and cadmium uptake by radish sprouts grown in agar medium	2019
6136	B. Zhao	Immunosuppressive effect of arsenic trioxide (A2O3) on xeno-islet transplantation	2016

6137	D. Snigurenko	XPS study of arsenic doped ZnO grown by Atomic Layer Deposition	2014
6138	G. X. Huang	Effect of sample pretreatment on the fractionation of arsenic in anoxic soils	2015
6139	Y. K. Penke	Anti-bacterial and arsenic remediation insights in aqueous systems onto heterogeneous metal oxide (Cu0.52Al0.1Fe0.47O4)/rGO hybrid: an approach towards airborne microbial degradation	
6140	Z. M. Cinan	Gamma irradiation, thermal conductivity, and phase change tests of the cement-hyperbranched poly amino-ester-block-poly cabrolactone-polyurathane plaster-lead oxide and arsenic oxide composite for development of radiation shielding material	2021
6141	K. Gedik	Adsorption and desorption of arsenate in Louisiana rice soils	2016
6142	J. Antelo	Stability of naturally occurring AMD-schwertmannite in the presence of arsenic and reducing agents	2021
6143	M. T. Keley	Effects of Supercritical CO2 on Quantity and Quality of Extracted Oil from Myctophidae Fish and Comparison It with the Wet Pressing as a Commercial Method	2022
6144	L. Silaghi-Dumitrescu	Supramolecular architecture of AsPh2Br2 (2) (Br-3) center dot center dot center dot (Br-2) center dot center dot center dot (Br-3) obtained by bromination of (AsPh2)(2)S	2018
6145	P. Koedrith	Integrative toxicogenomics-based approach to risk assessment of heavy metal mixtures/complexes: strategies and challenges	2015
6146	C. J. Newell	Monitored Natural Attenuation to Manage PFAS Impacts to Groundwater: Scientific Basis	2021
6147	T. V. Blankenship	LiCa3As2H and Ca14As6X7 (X = C, H, N): Two New Arsenide Hydride Phases Grown from Ca/Li Metal Flux	2014
6148	C. Monchanin	Current permissible levels of metal pollutants harm terrestrial invertebrates	2021
6149	A. K. Singh	Removal of trivalent and pentavalent arsenic from water using chemically modified chitosan beads	2020
6150	A. Ghosh	Redox-Assisted Arsenic(III) Adsorption for Removal from Aqueous Solution by Cerium(IV)-Incorporated Zirconium Oxide Nanocomposites	2020
6151	S. Park	NDRG2 Sensitizes Myeloid Leukemia to Arsenic Trioxide via GSK3-NDRG2-PP2A Complex Formation	2019
6152	V. Bottero	Key Disease Mechanisms Linked to Amyotrophic Lateral Sclerosis in Spinal Cord Motor Neurons	2022
6153	E. R. T. Tiekink	A Survey of Supramolecular Aggregation Based on Main Group ElementMIDLINE HORIZONTAL ELLIPSISSelenium Secondary Bonding Interactions-A Survey of the Crystallographic Literature	2020
6154	C. Y. Ying	Highly enhanced oxidation of arsenite at the surface of birnessite in the presence of pyrophosphate and the underlying reaction mechanisms	2020

6155	C. M. Bulka	Epigenetics at the Intersection of COVID-19 Risk and Environmental Chemical Exposures	
6156	F. Dell'Anno	Degradation of Hydrocarbons and Heavy Metal Reduction by Marine Bacteria in Highly Contaminated Sediments	2020
6157	B. Michalke	The importance of speciation analysis in neurodegeneration research	2018
6158	Y. F. Wu	Anaerobic As(III) Oxidation Coupled with Nitrate Reduction and Attenuation of Dissolved Arsenic by Noviherbaspirillum Species	2021
6159	M. Dai	Combined Electrosorption and Chemisorption of As(V) in Water by Using Fe-rGO@AC Electrode	2017
6160	C. Lopes	Chitin production from crustacean biomass: Sustainability assessment of chemical and enzymatic processes	2018
6161	L. Mochalov	Structural and optical properties of arsenic sulfide films synthesized by a novel PECVD-based approach	2017
6162	L. X. Yao	Phosphate enhances uptake of As species in garland chrysanthemum (C-coronarium) applied with chicken manure bearing roxarsone and its metabolites	2015
6163	M. Rasmussen	Mn2+ substitution within the {V14As8} polyoxovanadate archetype results in {Mn2V12As8} shells with trans-positioned heterometal positions	2021
6164	C. di Nicola	Synthesis and structural characterisation of some mononuclear 1:1:1 complexes of coinage metal(I) compounds with tertiary phosphines (arsines) and 1,2-diamines, MX(EPh3)(N,N '-1,2-diamine)	2021
6165	D. Jacimovic	ECOLOGICAL ENTREPRENEURSHIP-OLIVE PRODUCTION POTENTIAL IN MONTENEGRO	2017
6166	D. Postma	Fate of Arsenic during Red River Water Infiltration into Aquifers beneath Hanoi, Vietnam	2017
6167	A. Elgehama	Blockade of the interaction between Bcr-Abl and PTB1B by small molecule SBF-1 to overcome imatinib-resistance of chronic myeloid leukemia cells	2016
6168	Y. Babaee	Removal of arsenic (III) and arsenic (V) from aqueous solutions through adsorption by Fe/Cu nanoparticles	2018
6169	W. N. Zhang	Simultaneous Determination of Mercury and Antimony in Lead-Base Alloys by Hydride Generation-Double Channel Atomic Fluorescence Spectrometry with Solid-phase Extraction Using Amberlite XAD-16 Resin Micro-column	2014
6170	M. Rashid	Kinetic and Mechanistic Evaluation of Inorganic Arsenic Species Adsorption onto Humic Acid Grafted Magnetite Nanoparticles	2018
6171	M. Wang	AZT sensitizes hepatocellular carcinoma cells to As2O3 by upregulating the arsenic transporter aquaglyceroporin9	2019
6172	G. N. Lu	How different are the arsenic fractions inhibit alkaline phosphatases on aggregates scale?	2021

6173	S. Morais	Environmental and Health Hazards of Chromated Copper Arsenate-Treated Wood: A Review	2021
6174	T. Mukherjee	Plant Growth-Promoting Traits of a Thermophilic Strain of the Klebsiella Group with its Effect on Rice Plant Growth	2020
6175	J. Bodin	Can Exposure to Environmental Chemicals Increase the Risk of Diabetes Type 1 Development?	2015
6176	W. Lee	Occurrence and removal of hazardous chemicals and toxic metals in 27 industrial wastewater treatment plants in Korea	2015
6177	N. Yaghi	Effect of oxide coatings, pH and competing anion on the sorption of arsenic species onto Light Expanded Clay Aggregates (LECA's)	2018
6178	T. M. Clancy	Evaluating the cement stabilization of arsenic-bearing iron wastes from drinking water treatment	2015
6179	L. M. Gajic-Krstajic	Fe-Mo alloy coatings as cathodes in chlorate production process	2016
6180	N. Quinete	Drinking water pollutants may affect the immune system: concerns regarding COVID-19 health effects	2021
6181	F. J. Sun	Genetic characterization of two fully sequenced multi-drug resistant plasmids pP10164-2 and pP10164-3 from Leclercia adecarboxylata	2016
6182	V. G. Krivovichev	Number of minerals of various chemical elements: Statistics 2012 (a new approach to an old problem)	2014
6183	H. Q. Shen	Analytical aspects of meet-in-metabolite analysis for molecular pathway reconstitution from exposure to adverse outcome	2022
6184	H. S. Choi	Downscaling AsTeGeSiN threshold switching devices for high-density 3D memories	2018
6185	W. H. Chang	Armillaridin induces autophagy-associated cell death in human chronic myelogenous leukemia K562 cells	2016
6186	J. N. Ding	Investigation and assessment of environmental pollution in Gonghu Bay, Taihu Lake, China: A year-long study	2020
6187	W. J. Huang	Causal relationships among biological toxicity, geochemical conditions and derived DBPs in groundwater	2015
6188	E. C. Pastrana	Fabrication and characterization of copper (II) oxide/iron (III) oxide thin film heterostructures for trace arsenic (III) removal in water	2021
6189	A. O. Fayiga	Arsenic hyperaccumulating fern: Implications for remediation of arsenic contaminated soils	2016
6190	Z. M. Xie	Interactions between arsenic adsorption/desorption and indigenous bacterial activity in shallow high arsenic aquifer sediments from the Jianghan Plain, Central China	2018

6191	G. Schoeters	Three cycles of human biomonitoring in Flanders - Time trends observed in the Flemish Environment and Health Study	2017
6192	B. C. Pan	Acid and organic resistant nano-hydrated zirconium oxide (HZO)/polystyrene hybrid adsorbent for arsenic removal from water	2014
6193	Y. N. Dhoble	Treatment of wastewater generated from coke oven by adsorption on steelmaking slag and its effect on cementitious properties	2019
6194	S. V. S. Rana	Perspectives in Endocrine Toxicity of Heavy Metals-A Review	2014
6195	J. Wu	DDX5-targeting fully human monoclonal autoantibody inhibits proliferation and promotes differentiation of acute promyelocytic leukemia cells by increasing ROS production	2020
6196	T. Nishinaga	Progress in art and science of crystal growth and its impacts on modern society	2015
6197	A. C. F. Souza	Effects of sodium arsenate and arsenite on male reproductive functions in Wistar rats	2016
6198	K. Taleb	Efficient arsenic removal by cross-linked macroporous polymer impregnated with hydrous iron oxide: Material performance	2015
6199	W. Q. Zhang	Resveratrol Reduces Oxidative Stress and Improves Arsenic Efflux in Rats Exposed To Arsenic Trioxide	2014
6200	Y. Yu	Yttrium-doped iron oxide magnetic adsorbent for enhancement in arsenic removal and ease in separation after applications	2018
6201	B. Gholipour	Phase-change-driven dielectric-plasmonic transitions in chalcogenide metasurfaces	2018
6202	C. Z. Li	Biological and environmental hazards associated with exposure to chemical warfare agents: arsenicals	2016
6203	E. R. Hebbard	Regrowth of arsenate-sulfate efflorescences on processing plant walls at the Ottery arsenic-tin mine, New South Wales, Australia: Implications for arsenic mobility and remediation of mineral processing sites	2017
6204	M. Lei	Using Fe-Mn binary oxide three-dimensional nanostructure to remove arsenic from aqueous systems	2016
6205	C. O. Cope	Arsenate adsorption onto iron oxide amended rice husk char	2014
6206	C. Tanase	Assessment of Heavy Metal Content in Tree Barks: Picea abies, Pinus sylvestris, and Pinus nigra	2021
6207	H. W. Jiang	Study on the bio-oil characterization and heavy metals distribution during the aqueous phase recycling in the hydrothermal liquefaction of As-enriched Pteris vittata L	2020
6208	R. T. Bray	Sizes of iron hydroxide particles formed during ferric coagulation processes	2017

6209	H. Ahmad	Preconcentration and speciation of arsenic by using a graphene oxide nanoconstruct functionalized with a hyperbranched polyethyleneimine	2018
6210	S. Kayser	Updates on targeted therapies for acute myeloid leukaemia	2022
6211	Y. X. Meng	Research progress of puckered honeycomb monolayers	2021
6212	G. Bonanno	Chemical elements in Mediterranean macroalgae. A review	2018
6213	J. Liu	Distribution and Speciation of Copper and Arsenic in Rice Plants (Oryza sativa japonica 'Koshihikari') Treated with Copper Oxide Nanoparticles and Arsenic during a Life Cycle	2019
6214	R. C. Hott	Adsorption of arsenic from water and its recovery as a highly active photocatalyst	2016
6215	M. Massoudinejad	Investigation of photo-catalytic removal of arsenic from aqueous solutions using UV/H2O2 in the presence of ZnO nanoparticles	2020
6216	M. Z. Huang	Effects and mechanisms of bortezomib combined with arsenic trioxide on multiple myeloma	2019
6217	H. Li	Ameliorative effect of graphene nanosheets against arsenic-induced toxicity in mice by oral exposure	2021
6218	X. S. Cao	New insight into the mechanism of graphene oxide-enhanced phytotoxicity of arsenic species	2021
6219	G. Z. Nie	Surface chemistry of polymer-supported nano-hydrated ferric oxide for arsenic removal: effect of host pore structure	2015
6220	L. Yu	Rapid adsorption removal of arsenate by hydrous cerium oxide-graphene composite	2015
6221	E. D. Bergerova	Investigation of arsenic removal from aqueous solution through selective sorption and nanofiber-based filters	2021
6222	W. B. Xu	Arsenic trioxide and bortezomib interact synergistically to induce apoptosis in chronic myelogenous leukemia cells resistant to imatinib mesylate through Bcr/Abl-dependent mechanisms	2014
6223	I. A. Adedara	Neuroprotective mechanisms of selenium against arsenic-induced behavioral impairments in rats	2020
6224	E. T. F. Freitas	Natural attenuation of arsenic in the environment by immobilization in nanostructured hematite	2015
6225	T. G. Asere	Adsorption of As(III) versus As(V) from aqueous solutions by cerium-loaded volcanic rocks	2017
6226	C. L. Yang	Effect of reductive dissolution of iron (hydr)oxides on arsenic behavior in a water-sediment system: First release, then adsorption	2015

6227	D. Ocinski	Highly efficient arsenic sorbent based on residual from water deironing - Sorption mechanisms and column studies	2020
6228	S. Sharma	Betaine attenuates sodium arsenite-induced renal dysfunction in rats	
6229	Y. M. Tian	Insight into regeneration mechanism with sulfuric acid for arsenic poisoned commercial SCR catalyst	2020
6230	S. W. Yan	Zinc oxide nanoparticles alleviate the arsenic toxicity and decrease the accumulation of arsenic in rice (Oryza sativa L.)	2021
6231	P. H. Shao	Lattice-Defect-Enhanced Adsorption of Arsenic on Zirconia Nanospheres: A Combined Experimental and Theoretical Study	2019
6232	Y. S. Han	Beam-induced redox transformation of arsenic during As K-edge XAS measurements: availability of reducing or oxidizing agents and As speciation	2018
6233	C. Sudhakar	Species-Specific Uptake of Arsenic on Confined Metastable 2-Line Ferrihydrite: A Combined Raman-X-Ray Photoelectron Spectroscopy Investigation of the Adsorption Mechanism	2018
6234	A. Gomez-Hernandez	Alluvial and gypsum karst geological transition favors spreading arsenic contamination in Matehuala, Mexico	2020
6235	J. H. Walrod	Arsenic mobility in Karnak soils after multi-year application of poultry litter containing roxarsone	2016
6236	Y. S. Ye	Arsenic trioxide induces regulatory functions of plasmacytoid dendritic cells through interferon-alpha inhibition	2020
6237	H. D. Pan	Traditional Chinese Medicine as a Treatment for Rheumatoid Arthritis: From Empirical Practice to Evidence-Based Therapy	2019
6238	S. Avram	Prevention of Deficit in Neuropsychiatric Disorders through Monitoring of Arsenic and Its Derivatives as Well as Through Bioinformatics and Cheminformatics	2019
6239	I. Kumar	Cost-effective synthesis and characterization of CuO NPs as a nanosize adsorbent for As (III) remediation in synthetic arsenic-contaminated water	2020
6240	N. Ullah	Study of the effect of inorganic and organic complexes of arsenic metal on the status of GSH in T. cells and B. cells of blood	2015
6241	S. W. Li	NF-kappa B-mediated inflammation correlates with calcium overload under arsenic trioxide-induced myocardial damage in Gallus gallus	2017
6242	A. M. Mawia	Inorganic arsenic toxicity and alleviation strategies in rice	2021
6243	A. A. Nghiem	Aquifer-Scale Observations of Iron Redox Transformations in Arsenic-Impacted Environments to Predict Future Contamination	2020
6244	M. N. Pervez	A bifunctional alpha-FeOOH@GCA nanocomposite for enhanced adsorption of arsenic and photo Fenton-like catalytic conversion of As(III)	2021

6245	K. J. McDonald	Removal of arsenic from groundwater in West Bengal, India using CuO nanoparticle adsorbent	2015
6246	A. Heredia	Compared arsenic removal from aqueous solutions by synthetic mixed oxides and modified natural zeolites	2019
6247	N. Torasso	Enhancing arsenic adsorption via excellent dispersion of iron oxide nanoparticles inside poly(vinyl alcohol) nanofibers	2021
6248	A. Pranudta	Synthesis optimization and X-ray absorption spectroscopy investigation of polymeric anion exchanger supported binary Fe/Mn oxides nanoparticles for enhanced As(III) removal	2020
6249	H. M. Shan	Development of LC-HGAFS method for direct measurement of monothioarsenate and application for its adsorption characteristics	2020
6250	Y. Hu	2D Arsenene and Arsenic Materials: Fundamental Properties, Preparation, and Applications	2022
6251	X. T. Yang	ARSENIC ADSORPTION FROM WATER USING GRAPHENE-BASED MATERIALS AS ADSORBENTS: A CRITICAL REVIEW	2017
6252	L. P. Lingamdinne	Porous graphene oxide based inverse spinel nickel ferrite nanocomposites for the enhanced adsorption removal of arsenic	2016
6253	Z. Zhang	Temporal influence of reaction atmosphere and chlorine on arsenic release in combustion, gasification and pyrolysis of sawdust	2020
6254	P. B. Hu	Research on As2O3 adsorption enhancement characteristics of Mn-modified gamma-Al2O3	2021
6255	I. Polowczyk	Synthetic Iron Oxides for Adsorptive Removal of Arsenic	2018
6256	X. B. Min	Fe-FeS2 adsorbent prepared with iron powder and pyrite by facile ball milling and its application for arsenic removal	2017
6257	F. H. Dong	Factors influencing the morphology and adsorption performance of cellulose nanocrystal/iron oxide nanorod composites for the removal of arsenic during water treatment	2020
6258	Y. H. Li	Alkaline oxidative pressure leaching of arsenic and antimony bearing dusts	2016
6259	G. Gurdal	The properties of Can Basin coals (Canakkale-Turkey): Spontaneous combustion and combustion by-products	2015
6260	Z. D. Yuan	Effect of hydroquinone-induced iron reduction on the stability of scorodite and arsenic mobilization	2016
6261	I. Newman	Composition of Unrecorded Distilled Alcohol (bai jiu) Produced in Small Rural Factories in Central China	2017
6262	D. W. Wu	As2O3 capture from incineration flue gas by Fe2O3-modified porous carbon: Experimental and DFT insights	2022

6263	J. Nikic	Synthesis, characterization and application of magnetic nanoparticles modified with Fe-Mn binary oxide for enhanced removal of As(III) and As(V)	2021
6264	J. A. Faust	Real-Time Detection of Arsenic Cations from Ambient Air in Boreal Forest and Lake Environments	2016
6265	M. Mazari-Hiriart	Challenges and Opportunities on Urban Water Quality in Mexico City	2019
6266	C. Franken	Environmental exposure to human carcinogens in teenagers and the association with DNA damage	2017
6267	Y. Wang	Multivariate statistical analyses on the enrichment of arsenic with different oxidation states in the Quaternary sediments of the Pearl River Delta, China	2014
6268	H. M. Skroder	Selenium status in pregnancy influences children's cognitive function at 1.5 years of age	2015
6269	Z. H. Yu	Effects of manganese oxide-modified biochar composites on arsenic speciation and accumulation in an indica rice (Oryza sativa L.) cultivar	2017
6270	C. S. Yue	Stabilization of Soil Arsenic with Iron and Nano-Iron Materials: A Review	2021
6271	L. D. Garbinski	Pathways of arsenic uptake and efflux	2019
6272	J. B. Awuah	A density functional theory study of arsenic immobilization by the Al(III)-modified zeolite clinoptilolite	2016
6273	V. Dimitrov	Optical basicity and single bond strength of Sb2O3 containing glasses	2014
6274	M. A. Alsina	Arsenic speciation in sinter mineralization from a hydrothermal channel of El Tatio geothermal field, Chile	2014
6275	L. Stolze	Model-Based Interpretation of Groundwater Arsenic Mobility during in Situ Reductive Transformation of Ferrihydrite	2019
6276	J. X. Yu	Simultaneous removal of arsenate and arsenite in water using a novel functional halloysite nanotube composite	
6277	T. Kato	Removal of arsenic from a denitration catalyst by reduction using methanol as a carbon source	2019
6278	E. Przezdziecka	Evidence of magnesium impact on arsenic acceptor state: Study of ZnMgO:As molecular beam epitaxy layers	2018
6279	F. M. Rebelo	Arsenic, lead, mercury and cadmium: Toxicity, levels in breast milk and the risks for breastfed infants	2016
6280	D. B. Pal	Arsenic removal from synthetic waste water by CuO nano-flakes synthesized by aqueous precipitation method	2017

6281	S. Chauhan	A sensitive conductivity sensor for arsenic detection in environmental samples	2022
6282	D. Colleoni	Arsenic related defect states resonant with the semiconductor conduction band at the In0.53Ga0.47As/oxide interface: A density functional study	2015
6283	Z. Souri	Elucidating the physiological mechanisms underlying enhanced arsenic hyperaccumulation by glutathione modified superparamagnetic iron oxide nanoparticles in Isatis cappadocica	2020
6284	S. Lu	Geochemical characteristics of arsenic in groundwater during riverbank filtration: a case study of Liao River, Northeast China	2020
6285	F. S. Farnese	The Involvement of Nitric Oxide in Integration of Plant Physiological and Ultrastructural Adjustments in Response to Arsenic (vol 8, 516, 2017)	2017
6286	P. Dhanasekaran	Arsenic removal from groundwater by Anjili tree sawdust impregnated with ferric hydroxide and activated alumina	2016
6287	D. Korsak	Characterization of nonpathogenic Listeria species isolated from food and food processing environment	2016
6288	V. Chandrakar	Arsenic-induced metabolic disturbances and their mitigation mechanisms in crop plants: A review	2016
6289	E. Meez	Activated Carbons for Arsenic Removal from Natural Waters and Wastewaters: A Review	2021
6290	X. Y. Li	Performance of Mo modified gamma-Fe2O3 catalyst for selective catalytic reduction of NOx with ammonia: Presence of arsenic in flue gas	2021
6291	L. N. Lin	Capacity and mechanism of arsenic adsorption on red soil supplemented with ferromanganese oxide-biochar composites	2018
6292	D. J. Fisher	Environmental Concerns of Roxarsone in Broiler Poultry Feed and Litter in Maryland, USA	2015
6293	M. Habuda-Stanic	Arsenic removal by nanoparticles: a review	2015
6294	K. E. A. Bantol	Perspectives from the Society for Pediatric Research: contaminants of water and children's health: Can we do better?	2020
6295	X. Ge	A 3D porous carbon foam loaded with Fe3O4/graphene oxide for highly effective As(iii) removal	2020
6296	H. Fakour	Equilibrium Modeling of Arsenic Adsorption in a Ternary Arsenic-Iron Oxide-Natural Organic Matter System	2016
6297	S. K. Ponnaiah	Clinically Pertinent Manganese Oxide/Polyoxytyramine/Reduced Graphene Oxide Nanocomposite for Voltammetric Detection of Salivary and Urinary Arsenic	2020
6298	S. Mukhopadhyay	Arsenic removal from soil with high iron content using a natural surfactant and phosphate	2015

6299	O. Moradlou	Magnetite nanoplates decorated on anodized aluminum oxide nanofibers as a novel adsorbent for efficient removal of As(III)	2016
6300	C. H. Wang	Superior removal of arsenic from water with zirconium metal-organic framework UiO-66	2015
6301	S. Moghaddam	High Removal Capacity of Arsenic from Drinking Water Using Modified Magnetic Polyurethane Foam Nanocomposites	2019
6302	X. Y. Zhu	Secondary minerals of weathered orpiment-realgar-bearing tailings in Shimen carbonate-type realgar mine, Changde, Central China	2015
6303	D. Meza-Figueroa	Metal bioaccessibility, particle size distribution and polydispersity of playground dust in synthetic lysosomal fluids	2020
6304	R. Alarcon	Liberation of Adsorbed and Co-Precipitated Arsenic from Jarosite, Schwertmannite, Ferrihydrite, and Goethite in Seawater	2014
6305	X. W. Yan	p-Azidophenylarsenoxide: An Arsenical "Bait" for the In Situ Capture and Identification of Cellular Arsenic-Binding Proteins	2016
6306	Z. P. Wen	Simultaneous oxidation and immobilization of arsenite from water by nanosized magnetic mesoporous iron manganese bimetal oxides (Nanosized-MMIM): Synergistic effect and interface catalysis	2020
6307	Y. Wang	Removal of As(V) from wastewaters using magnetic iron oxides formed by zero-valent iron electrocoagulation	2022
6308	Z. P. Wen	Synthesis of ordered mesoporous iron manganese bimetal oxides for arsenic removal from aqueous solutions	2014
6309	M. A. Gabris	Magnetic graphene oxide nanocomposite functionalized with glucamine for the trace extraction of arsenic (III) from aqueous media	2021
6310	M. Z. Cao	As-Hg Compound Pollution: Rice Growth, Yield, and Environmental Safety Limits	2020
6311	K. Debiec	The influence of thermal treatment on bioweathering and arsenic sorption capacity of a natural iron (oxyhydr)oxide-based adsorbent	2017
6312	J. T. Hou	Enhanced oxidation of arsenite to arsenate using tunable K+ concentration in the OMS-2 tunnel	2018
6313	M. Rakibuddin	Sol-gel derived Fe3O4 quantum dot decorated silica composites for effective removal of arsenic (III) from water	2020
6314	J. A. Saunders	Bioremediation of arsenic-contaminated groundwater by sequestration of arsenic in biogenic pyrite	2018
6315	V. M. Mikoushkin	Modification of the GaAs native oxide surface layer into the layer of the Ga2O3 dielectric by an Ar+ ion beam	2018
6316	X. Y. Meng	Arsenic solubilization and redistribution under anoxic conditions in three aquifer sediments from a basin-fill aquifer in Northern Utah: The role of natural organic carbon and carbonate minerals	2016

6317	H. Kolya	Recent Advances in Colorimetric Detection of Arsenic Using Metal-Based Nanoparticles	2021
6318	L. B. Huhmann	A mass-balance model to assess arsenic exposure from multiple wells in Bangladesh	2022
6319	B. Peng	Physicochemical properties of arsenic-bearing lime-ferrate sludge and its leaching behaviors	2017
6320	H. Basu	Graphene oxide-MnO2-goethite microsphere impregnated alginate: A novel hybrid nanosorbent for As (III) and As (V) removal from groundwater	2021
6321	D. Setyono	Multi-metal oxide incorporated microcapsules for efficient As(III) and As(V) removal from water	2014
6322	S. E. Mische	Novel Treatment of Acute Promyelocytic Leukemia: As2O3, Retinoic Acid and Retinoid Pharmacology (vol 14, pg 849, 2013)	2014
6323	X. Y. Meng	Mineralogy and geochemistry affecting arsenic solubility in sediment profiles from the shallow basin-fill aquifer of Cache Valley Basin, Utah	2017
6324	A. Chiavola	Comparison of different iron oxide adsorbents for combined arsenic, vanadium and fluoride removal from drinking water	2019
6325	D. D. He	Arsenic (III) Removal from a High-Concentration Arsenic (III) Solution by Forming Ferric Arsenite on Red Mud Surface	2020
6326	I. Saha	Synthesis, characterization and As(III) adsorption behavior of beta-cyclodextrin modified hydrous ferric oxide	2014
6327	M. Deng	Well-dispersed TiO2 nanoparticles anchored on Fe3O4 magnetic nanosheets for efficient arsenic removal	2019
6328	J. Nikic	Arsenic adsorption on Fe-Mn modified granular activated carbon (GAC-FeMn): batch and fixed-bed column studies	2019
6329	Y. P. Song	DNA hydroxymethylation reprogramming of beta-oxidation genes mediates early-life arsenic-evoked hepatic lipid accumulation in adult mice	2022
6330	M. S. Angotzi	As-(III,As- V) Uptake from Nanostructured Iron Oxides and Oxyhydroxides: The Complex Interplay between Sorbent Surface Chemistry and Arsenic Equilibria	2022
6331	A. Kana	Analysis of Cationic Species of Arsenic in Seafood	2018
6332	Z. Souri	Arsenic Hyperaccumulation Strategies: An Overview	2017
6333	V. K. Rathore	Stabilization of arsenic and fluoride bearing spent adsorbent in clay bricks: Preparation, characterization and leaching studies	2017
6334	C. H. Tsai	Identification of Id1 as a downstream effector for arsenic-promoted angiogenesis via PI3K/Akt, NF-kappa B and NOS signaling	2016

6335	W. S. Ng	The Fate of the Arsenic Species in the Pressure Oxidation of Refractory Gold Ores: Practical and Modelling Aspects	
6336	V. Minzatu	Biopolymers - Carbon Sources for Composite Materials Used as Adsorbents for As (V)	2019
6337	Y. N. Wang	Effective stabilization of arsenic in contaminated soils with biogenic manganese oxide (BMO) materials	2020
6338	A. Maghsodi	Optimization of effective parameters in the synthesis of nanopore anodic aluminum oxide membrane and arsenic removal by prepared magnetic iron oxide nanoparicles in anodic aluminum oxide membrane via ultrasonic-hydrothermal method	2018
6339	M. Zeeshan	Amelioration of AsV toxicity by concurrent application of ZnO-NPs and Se-NPs is associated with differential regulation of photosynthetic indexes, antioxidant pool and osmolytes content in soybean seedling	2021
6340	Q. Xue	Arsenite and arsenate binding to ferrihydrite organo-mineral coprecipitate: Implications for arsenic mobility and fate in natural environments	2019
6341	K. Ahmad	Removal of decidedly lethal metal arsenic from water using metal organic frameworks: a critical review	2022
6342	F. Liu	Distribution of arsenic in shallow aquifers of Guangzhou region, China: natural and anthropogenic impacts	2014
6343	M. E. Mejia-Santillan	Physical and arsenic adsorption properties of maghemite and magnetite sub-microparticles	2018
6344	T. Dordevic	An update on the mineral-like Sr-containing transition metal arsenates	2021
6345	D. G. Ahoule	Arsenic in African Waters: A Review	2015
6346	M. D. Ma	Arsenic removal from water by nanometer iron oxide coated single-wall carbon nanotubes	2018
6347	P. T. L. Huong	Application of Graphene Oxide-MnFe2O4 Magnetic Nanohybrids as Magnetically Separable Adsorbent for Highly Efficient Removal of Arsenic from Water	2016
6348	Y. P. Wang	Impact of Arsenic Related Defects on Electronic Performance of ZrO2/GaAs: Density Functional Theory Calculations	2015
6349	A. Malakar	Occurrence of arsenite in surface and groundwater associated with a perennial stream located in Western Nebraska, USA	2021
6350	C. B. Wang	Review of arsenic behavior during coal combustion: Volatilization, transformation, emission and removal technologies	2018
6351	M. Cozannet	New Insights into the Ecology and Physiology of Methanomassiliicoccales from Terrestrial and Aquatic Environments	2021
6352	V. Minzatu	Synthesis, Characterization and Adsorptive Performances of a Composite Material Based on Carbon and Iron Oxide Particles	2019

6353	B. Bouhafs	Electronic structure theory of unusually matched BAs1-xPx alloys using high-throughput ab-initio computation	2020
6354	G. Pallocca	Identification of transcriptome signatures and biomarkers specific for potential developmental toxicants inhibiting human neural crest cell migration	2016
6355	N. Liu	Biomonitorization of concentrations of 28 elements in serum and urine among workers exposed to indium compounds	2021
6356	L. K. Wu	Graphene oxide/CuFe2O4 foam as an efficient absorbent for arsenic removal from water	2018
6357	A. A. Yakout	High performance Zr-MnO2@reduced graphene oxide nanocomposite for efficient and simultaneous remediation of arsenates As(V) from environmental water samples	2021
6358	H. B. Ahmad	SYNTHESIS AND CHARACTERIZATION OF KAOLIN SUPPORTED METALLIC NANOCOMPOSITES FOR THE REMOVAL OF ARSENIC	2015
6359	L. Lei	Co-adsorption of arsenite and arsenate on mixed-valence Fe(II,III) (hydr) oxides under reducing conditions	2018
6360	S. W. Curtis	Exposure to polybrominated biphenyl and stochastic epigenetic mutations: application of a novel epigenetic approach to environmental exposure in the Michigan polybrominated biphenyl registry	2019
6361	S. Medici	Noble metals in medicine: Latest advances	2015
6362	H. D. Pappalardo	Cynara cardunculus L. as a Multipurpose Crop for Plant Secondary Metabolites Production in Marginal Stressed Lands	2020
6363	Z. D. Saeid	Investigation of synergistic action between coronatine and nitric oxide in alleviating arsenic-induced toxicity in sweet basil seedlings	2014
6364	A. A. Nghiem	Quantifying Riverine Recharge Impacts on Redox Conditions and Arsenic Release in Groundwater Aquifers Along the Red River, Vietnam	2019
6365	L. Ling	Sequestration of Arsenate in Zero-Valent Iron Nanoparticles: Visualization of Intraparticle Reactions at Angstrom Resolution	2014
6366	C. A. Coles	Effect of aeration, iron and arsenic concentrations, and groundwater matrix on arsenic removal using laboratory sand filtration	2020
6367	Y. L. Li	A comprehensive analysis of Wnt/-catenin signaling pathway-related genes and crosstalk pathways in the treatment of As2O3 in renal cancer	2018
6368	L. P. Lingamdinne	Facile Synthesis, Characterization, and Adsorption Insights of Lanthanum Oxide Nanorods	2020
6369	M. A. Bari	Concentrations, sources and human health risk of inhalation exposure to air toxics in Edmonton, Canada	2017
6370	A. C. Reynosa-Martinez	Effect of the degree of oxidation of graphene oxide on As(III) adsorption	2020

6371	C. Nieto-Delgado	Modified activated carbon with interconnected fibrils of iron-oxyhydroxides using Mn2+ as morphology regulator, for a superior arsenic removal from water	2019
6372	S. Paikaray	Arsenic Geochemistry of Acid Mine Drainage	2015
6373	V. Sridhar	Metal Organic Framework Derived MnO2-Carbon Nanotubes for Efficient Oxygen Reduction Reaction and Arsenic Removal from Contaminated Water	2020
6374	Q. Q. Wang	Methylated arsenic metabolites bind to PML protein but do not induce cellular differentiation and PML-RAR' protein degradation	2015
6375	T. Q. Liao	Removal of high-concentration of arsenic in acidic wastewater through zero-valent aluminium powder and characterisation of products	2021
6376	E. J. Kim	Effects of natural organic matter on the coprecipitation of arsenic with iron	2015
6377	Y. Wu	Multiple elements related to metabolic markers in the context of gestational diabetes mellitus in meconium	2018
6378	H. J. Cui	Fabrication of magnetic porous Fe-Mn binary oxide nanowires with superior capability for removal of As(III) from water	2014
6379	G. Sargazi	Fabrication of PVA/ZnO fibrous composite polymer as a novel sorbent for arsenic removal: design and a systematic study	2019
6380	D. Setyono	Chemically Modified Sawdust as Renewable Adsorbent for Arsenic Removal from Water	2014
6381	M. P. Mejia	Soil Microbial Community Composition and Tolerance to Contaminants in an Urban Brownfield Site	
6382	R. C. Leal	G3(MP2)//B3-SBK: A revision of a composite theory for calculations of thermochemical properties including some non-transition elements beyond the fourth period	2019
6383	O. Shpotyuk	Nanostructurization effects in PVP-stabilized tetra-arsenic tetra-sulfide As4S4 nanocomposites	2017
6384	M. Ranjan	Concurrent removal of nitrate, fluoride and arsenic by mixed hydrous bismuth oxide from water	2020
6385	S. Leaper	POSS-Functionalized Graphene Oxide/PVDF Electrospun Membranes for Complete Arsenic Removal Using Membrane Distillation	2021
6386	Y. Q. Yang	Comparative effects on arsenic uptake between iron (hydro)oxides on root surface and rhizosphere of rice in an alkaline paddy soil	2020
6387	J. Kumpiene	Leaching of arsenic, copper and chromium from thermally treated soil	2016
6388	R. B. Neumann	Biodegradable Organic Carbon in Sediments of an Arsenic-Contaminated Aquifer in Bangladesh	2014

6389	F. O. Ochedi	A review on coal fl y ash -based adsorbents for mercury and arsenic removal	2020
6390	R. S. Salunke	Electrodeposition of gold nanoparticles decorated single polypyrrole nanowire for arsenic detection in potable water: a chemiresistive sensor device	2017
6391	M. S. Park	Surface passivation and aging of InGaAs/InP heterojunction phototransistors	2017
6392	H. B. Yin	Removal of arsenic from water by porous charred granulated attapulgite-supported hydrated iron oxide in bath and column modes	2017
6393	S. F. Wu	Coupling of fully symmetric As phonon to magnetism in Ba(Fe1-xAux)(2)As-2	2020
6394	A. P. Panda	Core-shell structured zero-valent manganese (ZVM): a novel nanoadsorbent for efficient removal of As(iii) and As(v) from drinking water	2019
6395	M. M. Ommati	The Footprints of Oxidative Stress and Mitochondrial Impairment in Arsenic Trioxide-Induced Testosterone Release Suppression in Pubertal and Mature F1-Male Balb/c Mice via the Downregulation of 3 beta-HSD, 17 beta-HSD, and CYP11a Expression	2020
6396	B. Fu	Determination of Chemical Speciation of Arsenic and Selenium in High-As Coal Combustion Ash by X-ray Photoelectron Spectroscopy: Examples from a Kentucky Stoker Ash	2018
6397	A. I. A. Sherlala	Synthesis and characterization of magnetic graphene oxide for arsenic removal from aqueous solution	2019
6398	H. Sun	Study on the migration and transformation of arsenic and antimony in the rhizosphere of plants grown in zinc smelting slag	2021
6399	J. Y. Lin	Coupled Kinetics Model for Microbially Mediated Arsenic Reduction and Adsorption/Desorption on Iron Oxides: Role of Arsenic Desorption Induced by Microbes	2019
6400	M. Damaraju	Marigold wastewater treatment in a lab-scale and a field-scale continuous bipolar-mode electrocoagulation system	2020
6401	E. Preziosi	Disentangling natural and anthropogenic impacts on groundwater by hydrogeochemical, isotopic and microbiological data: Hints from a municipal solid waste landfill	2019
6402	T. J. Sorg	Regenerating an Arsenic Removal Iron-Based Adsorptive Media System, Part 2: Performance and Cost	2017
6403	S. W. Jeen	Reactive Transport Modeling for Mobilization of Arsenic in a Sediment Downgradient from an Iron Permeable Reactive Barrier	2017
6404	N. O. Kitterod	Hydrogeology and groundwater quality in the Nordic and Baltic countries	
6405	J. H. Lin	G(2)/M cell cycle arrest and apoptosis induced by COH-203 in human promyelocytic leukemia HL-60 cells	2021
6406	P. V. Ioannou	Dimethylphosphinato and dimethylarsinato complexes of Sb(III) and Bi(III) and their chemistry	2012

6407	S. A. Khan	Batch experiments on arsenic removal efficiencies through adsorption using synthetic and natural sand samples	2021
6408	F. Kabir	Arsenic removal methods for drinking water in the developing countries: technological developments and research needs	2017
6409	J. Liu	Eugenol attenuates concanavalin A-induced hepatitis through modulation of cytokine levels and inhibition of mitochondrial oxidative stress	2019
6410	Y. J. Yoon	Synthesis of magnetite/non-oxidative graphene composites and their application for arsenic removal	2017
6411	M. Kesavan	Atorvastatin restores arsenic-induced vascular dysfunction in rats: Modulation of nitric oxide signaling and inflammatory mediators	2014
6412	L. S. Altoe	Could vitamin C and zinc chloride protect the germ cells against sodium arsenite?	2017
6413	M. M. Liu	Vitamin C increases viral mimicry induced by 5-aza-2 '-deoxycytidine	2016
6414	A. Toth	A Novel Mathematical Model Describing Adaptive Cellular Drug Metabolism and Toxicity in the Chemoimmune System	2015
6415	M. K. Mondal	A comprehensive review on removal of arsenic using activated carbon prepared from easily available waste materials	2017
6416	A. V. Zmozinski	Establishment of a method for determination of arsenic species in seafood by LC-ICP-MS	2015
6417	B. Q. Yang	Preparation of a spindle delta-MnO2@Fe/Co-MOF-74 for effective adsorption of arsenic from water	2021
6418	R. Scala	Impact of arsenic and phosphorus concentration on oxygen content in heavily doped silicon single crystal	2020
6419	S. Kempahanumakkagar	Nanomaterial-based electrochemical sensors for arsenic - A review	2017
6420	I. Dutto	Biology of the cell cycle inhibitor p21(CDKN1A): molecular mechanisms and relevance in chemical toxicology	2015
6421	A. J. Ashe	Aromatic heterocycles containing arsenic and sulfur: The synthesis of 1,2- and 1,3-thiaarsole and the gas-phase molecular structure of 1,2-thiaarsole	2000
6422	P. Mani	Complete arsenite removal from groundwater by UV activated potassium persulfate and iron oxide impregnated granular activated carbon	2021
6423	C. Ma	Molecular Mechanisms Involving the Sonic Hedgehog Pathway in Lung Cancer Therapy: Recent Advances	2022
6424	K. Arora	Valorization of Wastewater Resources Into Biofuel and Value-Added Products Using Microalgal System	2021

6425	R. Masciale	Assessing Natural Background Levels in the Groundwater Bodies of the Apulia Region (Southern Italy)	2021
6426	T. J. H. Banch	Factorial Design and Optimization of Landfill Leachate Treatment Using Tannin-Based Natural Coagulant	2019
6427	L. X. H. Lambert	Evaluating water quality using social media and federal agency data	2021
6428	S. Rukh	Arsenate and arsenite adsorption in relation with chemical properties of alluvial and loess soils	2017
6429	W. W. Wong	Recent advances in exploitation of nanomaterial for arsenic removal from water: a review	2017
6430	Y. Y. Dang	Curcumin prevents As3+-induced carcinogenesis through regulation of GSK3 beta/Nrf2	2021
6431	P. K. Mishra	Aero-Gel Based Cerium Doped Iron Oxide Solid Solution for Ultrafast Removal of Arsenic	2018
6432	K. B. Juybari	Evaluation of serum arsenic and its effects on antioxidant alterations in relapsing-remitting multiple sclerosis patients	2018
6433	S. Y. Chen	Effect of Ultrasonic Treatment on Transformations of Arsenic Species in Edible Mushrooms	2020
6434	J. E. Borger	Transient Dipnictyl Analogues of Acrylamides, R-E=E '-CONR2, and a Related Diphosphadigalletane from Na OCP and (R2N)(2)ECl (E, E '=P, As, Ga)	2019
6435	J. Szakova	The risk elementuptake by chamomile (Matricaria recutita (L.) Rauschert) growing in four differentsoils	2018
6436	C. Q. Wu	Novel Continuous Column Process for As(III) Oxidation from Concentrated Acidic Solutions with Activated Carbon Catalysis	2020
6437	Y. Zhao	Arsenic oxidation and removal from flue gas using H2O2/Na2S2O8 solution	2017
6438	J. Liu	Copper oxide nanoparticles and arsenic interact to alter seedling growth of rice (Oryza sativa japonica)	2018
6439	F. B. Hussein	Synthesis, characterization and performance of polyurethane foam nanocomposite for arsenic removal from drinking water	2016
6440	R. A. Motiyenko	The rotational spectrum of Methylarsine	2020
6441	M. Tagliabue	Metal-rich sludge from mine water treatment: from waste to effective arsenate adsorbent	2017
6442	E. R. Melendez-Sanchez	Phylogenetic analysis of strains isolated from mine tailings and evaluation of their resistance to As and Zn	2022

6443	S. L. Jia	Elucidation of the Mechanism of Action for Metal Based Anticancer Drugs by Mass Spectrometry-Based Quantitative Proteomics	2019
6444	N. Zhou	SORPTION OF ROXARSONE FROM WATER ONTO TYPICAL NATURAL CLAY MINERALS	2015
6445	Z. H. Yang	Effects of Silicon and Iron Application on Arsenic Absorption and Physiological Characteristics of Rice (Oryza sativa L.)	2022
6446	M. V. Schaefer	Arsenic leaching from ceramic water filters	2018
6447	J. J. Wang	Adsorption enhanced the oxidase-mimicking catalytic activity of octahedral-shape Mn3O4 nanoparticles as a novel colorimetric chemosensor for ultrasensitive and selective detection of arsenic	2021
6448	K. V. Zhai	Flavonoids Synergistically Enhance the Anti-Glioblastoma Effects of Chemotherapeutic Drugs	2021
6449	J. Wu	In Situ Preparation of Stabilized Iron Sulfide Nanoparticle-Impregnated Alginate Composite for Selenite Remediation	2018
6450	P. P. Song	Electrocoagulation treatment of arsenic in wastewaters: A comprehensive review	2017
6451	O. Shpotyuk	Milling-driven nanonization of AsxS100-x alloys from second glass-forming region: The case of lower-crystalline arsenicals (56 < x < 66)	2020
6452	I. H. Nam	Effects of Heavy Metals on Biodegradation of Fluorene by a Sphingobacterium sp Strain (KM-02) Isolated from Polycyclic Aromatic Hydrocarbon-Contaminated Mine Soil	2015
6453	M. N. Gil	Nutritive and Xenobiotic Compounds in the Alien Algae Undaria pinnatifida From Argentine Patagonia	2015
6454	K. Vaxevanidou	Role of Indigenous Arsenate and Iron(III) Respiring Microorganisms in Controlling the Mobilization of Arsenic in a Contaminated Soil Sample	2015
6455	S. Guo	Superior As(III) removal performance of hydrous MnOOH nanorods from water	2015
6456	M. Pal	Purifying arsenic and fluoride-contaminated water by a novel graphene-based nanocomposite membrane of enhanced selectivity and sustained flux	2018
6457	S. I. Miyashita	A Simple and Effective Method for Speciation Analysis of 13 Arsenic Species Using HPLC on a Fluorocarbon Stationary Phase Coupled to ICP-MS	2021
6458	I. Michalak	The application of seaweeds in environmental biotechnology	2020
6459	R. B. Finkelman	The health impacts of coal use in China	2018
6460	C. Sturzer	Site Preference of Rare Earth Doping in Palladium-Iron-Arsenide Superconductors	2014

6461	P. Meszaros	Plant chitinase responses to different metal-type stresses reveal specificity	2014
6462	S. Mura	Ferrates for water remediation	2017
6463	S. A. Ali	In-situ monitoring of xenobiotics using genetically engineered whole-cell-based microbial biosensors: recent advances and outlook	2021
6464	J. Haney	Development of an inhalation unit risk factor for cadmium	2016
6465	L. Z. Li	Evolved Bacterial Biosensor for Arsenite Detection in Environmental Water	2015
6466	H. B. Hu	A High-Response Electrochemical As(III) Sensor Using Fe3O4-rGO Nanocomposite Materials	2021
6467	A. A. Dalvi	Sorption of arsenic on manganese dioxide synthesized by solid state reaction	2015
6468	C. Zou	Effect of CO2 in Flue Gas on Arsenic Adsorption over a Carbonaceous Surface	2019
6469	J. Garcia-Bellido	Recent advances in GC-ICP-MS: Focus on the current and future impact of MS/MS technology	2020
6470	N. Stilinovic	The level of elements and antioxidant activity of commercial dietary supplement formulations based on edible mushrooms	2014
6471	R. Lagoa	Molecular mechanisms linking environmental toxicants to cancer development: Significance for protective interventions with polyphenols	2022
6472	P. K. Singh	Decoding the role of hypothetical protein All3255 of Anabaena PCC7120 in heavy metal stress management in Escherichia coli	2018
6473	T. Ukena	Speciation and Determination of Inorganic Arsenic in Rice Using Liquid Chromatography-Inductively Coupled Plasma/Mass Spectrometry: Collaborative Study	2014
6474	J. Zhu	Adsorption behavior and removal mechanism of arsenic on graphene modified by iron-manganese binary oxide (FeMnOx/RGO) from aqueous solutions	2015
6475	O. Shpotyuk	Milling-driven nanonization of AsxS100-x alloys from second glass-forming region: The case of higher-crystalline arsenicals (51 < x < 56)	2020
6476	M. A. Fox	Meeting the public health challenge of protecting private wells: Proceedings and recommendations from an expert panel workshop	2016
6477	Y. F. Yang	Adsorption of p-Arsanilic Acid on Iron (Hydr)oxides and Its Implications for Contamination in Soils	2021
6478	S. Shahrin	Adsorptive mixed matrix membrane incorporating graphene oxide-manganese ferrite (GMF) hybrid nanomaterial for efficient As(V) ions removal	2019

6479	J. Nikic	Arsenic removal from water using a one-pot synthesized low-cost mesoporous Fe-Mn-modified biosorbent	2019
6480	Y. Cao	Capture of arsenic in coal combustion flue gas at high temperature in the presence of CaSiO3 with good anti-sintering	2020
6481	A. Piotrowsk a-	Auxins and Cytokinins Regulate Phytohormone Homeostasis and Thiol-Mediated Detoxification in the Green Alga Acutodesmus obliquus Exposed to Lead Stress	2020
6482	S. K. Stylianou	Novel Water Treatment Processes Based on Hybrid Membrane-Ozonation Systems: A Novel Ceramic Membrane Contactor for Bubbleless Ozonation of Emerging Micropollutants	2015
6483	J. He	Novel chitosan goethite bionanocomposite beads for arsenic remediation	2016
6484	W. S. Liu	Polymorphisms in arsenic (+3 oxidation state) methyltransferase (AS3MT) predict the occurrence of hyperleukocytosis and arsenic metabolism in APL patients treated with As2O3	2020
6485	P. M. Barrett	Increased exposure of plankton to arsenic in contaminated weakly-stratified lakes	2018
6486	Y. Wang	The design of scorodite@FeOOH core-shell materials and its stability treatment for arsenide	2019
6487	Y. Salameh	Arsenic(III,V) adsorption onto charred dolomite: Charring optimization and batch studies	2015
6488	K. Renu	Role of arsenic exposure in adipose tissue dysfunction and its possible implication in diabetes pathophysiology	2018
6489	E. Marcinko wska	Vitamin D Derivatives in Acute Myeloid Leukemia: The Matter of Selecting the Right Targets	2022
6490	W. Gao	Novel two-dimensional monoelemental and ternary materials: growth, physics and application	2020
6491	J. Gorny	Arsenic behavior in river sediments under redox gradient: A review	2015
6492	X. Zhang	Developing new adsorptive membrane by modification of support layer with iron oxide microspheres for arsenic removal	2018
6493	Y. Wang	Arsenic trioxide increases expression of secreted frizzled-related protein 1 gene and inhibits the WNT/beta-catenin signaling pathway in Jurkat cells	2017
6494	W. K. Biftu	Iron-alginate beads doped with green synthesised 'nano-CeO2-ZrO2' as an effective adsorbent for removal of highly toxic Arsenic-ions from polluted water	
6495	F. Wu	Effects of zinc oxide nanoparticles on arsenic stress in rice (Oryza sativa L.): germination, early growth, and arsenic uptake	2020
6496	A. A. Shoppert	Increased As Adsorption on Maghemite-Containing Red Mud Prepared by the Alkali Fusion-Leaching Method	2019

6497	J. D. Griffin	Blood's 70th anniversary: arsenic-from poison pill to magic bullet	2016
6498	T. Zhu	Synthesis of novel hydrated ferric oxide biochar nanohybrids for efficient arsenic removal from wastewater	2022
6499	A. J. Yao	Effects of an iron-silicon material, a synthetic zeolite and an alkaline clay on vegetable uptake of As and Cd from a polluted agricultural soil and proposed remediation mechanisms	2017
6500	Z. P. Wen	Effective As(III) and As(V) immobilization from aqueous solution by nascent ferrous hydroxide colloids (FHC)	2017
6501	Y. L. Zhang	Hybrid Flow System for Automatic Dynamic Fractionation and Speciation of Inorganic Arsenic in Environmental Solids	2015
6502	J. E. Seo	Theoretical Analysis of Ballistic Current Transport in Monolayer Black Arsenic MOSFETs	2020
6503	J. Z. Wu	A novel calcium-based magnetic biochar is effective in stabilization of arsenic and cadmium co-contamination in aerobic soils	2020
6504	O. E. Nworie	Differential Effects of Low-Molecular-Weight Organic Acids on the Mobilization of Soil-Borne Arsenic and Trace Metals	2017
6505	D. M. Xu	Mechanistic insight into the release behavior of arsenic (As) based on its geochemical fractions in the contaminated soils around lead/zinc (Pb/ Zn) smelters	2022
6506	S. Ghosh	Technology alternatives for decontamination of arsenic-rich groundwater-A critical review	2019
6507	J. J. Xie	Bioavailability/speciation of arsenic in atmospheric PM2.5 and their seasonal variation: A case study in Baoding city, China	2019
6508	A. A. Shah	Potassium silicate and zinc oxide nanoparticles modulate antioxidant system, membranous H+-ATPase and nitric oxide content in faba bean (Vicia faba) seedlings exposed to arsenic toxicity	
6509	G. Bhanjana	Novel electrochemical sensing of arsenic ions using a simple graphite pencil electrode modified with tin oxide nanoneedles	2018
6510	R. C. Hott	Purification of arsenic-contaminated water using iron molybdate filters and monitoring of their genotoxic, mutagenic, and cytotoxic effects through bioassays	2021
6511	X. X. Wang	Zinc Fertilizers Modified the Formation and Properties of Iron Plaque and Arsenic Accumulation in Rice (Oryza sativa L.) in a Life Cycle Study	2022
6512	S. Arikan	ARSENIC REMOVAL FROM AQUEOUS SOLUTIONS USING IRON OXIDE COATED SEPIOLITE	2017
6513	Y. Li	Arsenic release from shallow aquifers of the Hetao basin, Inner Mongolia: evidence from bacterial community in aquifer sediments and groundwater	2014
6514	G. Y. Cai	Self-enhanced and efficient removal of As(III) from water using Fe-Cu-Mn composite oxide under visible-light irradiation: Synergistic oxidation and mechanisms	2022

6515	D. W. Cho	Effect of Mn substitution on the oxidation/adsorption abilities of iron(III) oxyhydroxides	2018
6516	G. S. Zhang	Efficient Sorption of Arsenic on Nanostructured Fe-Cu Binary Oxides: Influence of Structure and Crystallinity	2022
6517	R. Zakhar	Adsorptive Removal of Pentavalent Arsenic from Aqueous Solutions by Granular Ferric Oxide	2022
6518	K. Yang	The effect of arsenic chemical form and mixing regime on arsenic mass transfer from soil to magnetite	2017
6519	Z. M. Liu	Synthesis of uniform-sized and microporous MIL-125(Ti) to boost arsenic removal by chemical adsorption	2021
6520	M. Zubair	Review on arsenic-induced toxicity in male reproductive system and its amelioration	2017
6521	J. G. Kim	Simultaneous oxidation and adsorption of arsenic by one-step fabrication of alum sludge and graphitic carbon nitride (g-C3N4)	2020
6522	M. Lopez-Garcia	New polymeric/inorganic hybrid sorbents based on red mud and nanosized magnetite for large scale applications in As(V) removal	2017
6523	Z. Y. Xiao	Effects of irrigation-induced water table fluctuation on arsenic mobilization in the unsaturated zone of the Datong Basin, northern China	2018
6524	L. Savage	Biovolatilization of Arsenic as Arsines from Seawater	2018
6525	L. W. Ye	In situ infrared spectroscopy study of the interface self-cleaning during the atomic layer deposition of HfO2 on GaAs(100) surfaces	2014
6526	Z. P. Gao	Experiment-based geochemical modeling of Arsenic(V) and Arsenic(III) adsorption onto aquifer sediments from an inland basin	2020
6527	N. Y. Zhu	Synthesis of mesoporous bismuth-impregnated aluminum oxide for arsenic removal: Adsorption mechanism study and application to a lab-scale column	2018
6528	W. B. Hu	Proton Self-Enhanced Hydroxyl-Enriched Cerium Oxide for Effective Arsenic Extraction from Strongly Acidic Wastewater	2022
6529	L. Savage	Maritime Deposition of Organic and Inorganic Arsenic	2019
6530	M. M. Rahman	Highly Efficient Iron Oxide Nanoparticles Immobilized on Cellulose Nanofibril Aerogels for Arsenic Removal from Water	2021
6531	A. Hartland	Association of Arsenic and Phosphorus with Iron Nanoparticles between Streams and Aquifers: Implications for Arsenic Mobility	2015
6532	K. M. Ko	Development of an Arsenobetaine Standard Solution with Metrological Traceability to the SI by an Arsenic-specific Mass Balance Method	2017

6533	M. H. Rahbar	Concentration of Lead, Mercury, Cadmium, Aluminum, Arsenic and Manganese in Umbilical Cord Blood of Jamaican Newborns	2015
6534	D. Abulizi	ENHANCED REMOVAL OF AQUEOUS As(III) USING A GOETHITE BIOCHAR COMPOSITE	2020
6535	M. Zubair	Rising level of arsenic in water and fodder: a growing threat to livestock and human populations in Pakistan	2018
6536	C. L. Jiao	Preparation and Arsenic Removal Mechanism of Iron Oxyhydroxide/expanded Graphite Composite Material	2017
6537	M. Ciopec	As(III) Removal by Dynamic Adsorption onto Amberlite XAD7 Functionalized with Crown Ether and Doped with Fe(III) Ions	2019
6538	S. Olivera	Cerium dioxide and composites for the removal of toxic metal ions	2018
6539	K. M. Hunt	The mechanistic basis of arsenicosis: Pathogenesis of skin cancer	2014
6540	Y. Zhang	Response of soil microbial communities to additions of straw biochar, iron oxide, and iron oxide-modified straw biochar in an arsenic-contaminated soil	2020
6541	H. M. Hu	Mechanochemically synthesized Fe-Mn binary oxides for efficient As(III) removal: Insight into the origin of synergy action from mutual Fe and Mn doping	2022
6542	I. A. Adedara	Selenium abates reproductive dysfunction via attenuation of biometal accumulation, oxido-inflammatory stress and caspase-3 activation in male rats exposed to arsenic	2019
6543	Y. Liu	Effects of salinity and (an)ions on arsenic behavior in sediment of Bosten Lake, Northwest China	2015
6544	J. R. Huling	Enhanced adsorption of arsenic through the oxidative treatment of reduced aquifer solids	2017
6545	L. Zhan	Recycle Gallium and Arsenic from GaAs-Based E-Wastes via Pyrolysis Vacuum Metallurgy Separation: Theory and Feasibility	2018
6546	K. Yang	Extraction of elemental arsenic and regeneration of calcium oxide from waste calcium arsenate produced from wastewater treatment	2019
6547	A. Plak	Land-use impact on selected forms of arsenic and phosphorus in soils of different functions	2017
6548	B. Y. Li	Mitigating arsenic accumulation in rice (Oryza sativa L.) from typical arsenic contaminated paddy soil of southern China using nanostructured alpha-MnO2: Pot experiment and field application	2019
6549	D. Zhang	In-situ mobilization and transformation of iron oxides-adsorbed arsenate in natural groundwater	2017
6550	N. Tara	Synthesis of antibacterial, antioxidant and magnetic Nigella sativa-graphene oxide based nanocomposite BC-GO@Fe3O4 for water treatment	2020

6551	H. Y. Hua	Arsenic trioxide and triptolide synergistically induce apoptosis in the SKM-1 human myelodysplastic syndrome cell line	2016
6552	L. A. Eaves	A role for microRNAs in the epigenetic control of sexually dimorphic gene expression in the human placenta	2020
6553	Y. H. Wang	Arsenic Speciation in Mekong Delta Sediments Depends on Their Depositional Environment	2018
6554	S. Paul	Co-cultivation as a Strategy to Reduce Food Chain-Mediated Arsenic Contamination in Human Beings	
6555	L. Otero-Gonzalez	Novel nanostructured iron oxide cryogels for arsenic (As(III)) removal	2020
6556	A. Ahmad	Mobility and redox transformation of arsenic during treatment of artificially recharged groundwater for drinking water production	2020
6557	Z. M. Lou	Enhanced removal of As(III)/(V) from water by simultaneously supported and stabilized Fe-Mn binary oxide nanohybrids	2017
6558	X. W. Liu	Synthesis and Characterization of Novel Fe-Mn-Ce Ternary Oxide-Biochar Composites as Highly Efficient Adsorbents for As(III) Removal from Aqueous Solutions	2018
6559	T. C. Prathna	Development of iron oxide nanoparticle adsorbents for arsenic and fluoride removal	2017
6560	V. Kamath	Comparative study of using five different leaf extracts in the green synthesis of iron oxide nanoparticles for removal of arsenic from water	2020
6561	J. Wang	Preparation and evaluation of magnetic nanoparticles impregnated chitosan beads for arsenic removal from water	2014
6562	H. X. Zhang	Distribution and genetic mechanism of high arsenic geothermal water in the Batang area, Western Sichuan	2021
6563	Z. X. Zhao	Adsorption and Oxidation of As(III) on Iron (Hydro)Oxides	2018
6564	A. Ortega-Guerrero	Evaporative concentration of arsenic in groundwater: health and environmental implications, La Laguna Region, Mexico	2017
6565	Z. X. Zhao	Effect of sulfide on As(III) and As(V) sequestration by ferrihydrite	2017
6566	H. S. Wang	Removal of arsenic from aqueous solution using microflower-like delta-Bi2O3 as adsorbent: adsorption characteristics and mechanisms	2020
6567	B. H. Li	Bioremediation of Nitrate- and Arsenic-Contaminated Groundwater Using Nitrate- Dependent Fe(II) Oxidizing Clostridium sp Strain pxl2	2016
6568	H. B. Ling	Selective removal of arsenic from crude antimony trioxide by leaching with nitric acid	2022

6569	M. Vermeulen	Study of dry- and wet-process amorphous arsenic sulfides: Synthesis, Raman reference spectra, and identification in historical art materials	2019
6570	D. Dickson	Adsorption kinetics and isotherms of arsenite and arsenate on hematite nanoparticles and aggregates	2017
6571	S. U. Khan	Evaluation of Fe-Mg Binary Oxide for As (III) Adsorption-Synthesis, Characterization and Kinetic Modelling	2021
6572	M. O. Stahl	River bank geomorphology controls groundwater arsenic concentrations in aquifers adjacent to the Red River, Hanoi Vietnam	2016
6573	J. M. Blake	Zinc, copper, nickel, and arsenic monitoring in natural streams using in-situ iron-manganese oxide coated stream pebbles	2015
6574	M. A. Carneiro	Current Trends of Arsenic Adsorption in Continuous Mode: Literature Review and Future Perspectives	2021
6575	D. C. Antonio	Effects of aluminum and soil mineralogy on arsenic bioaccessibility	2021
6576	H. Skroder	Predictors of selenium biomarker kinetics in 4-9-year-old Bangladeshi children	2018
6577	S. Tresintsi	A novel approach for arsenic adsorbents regeneration using MgO	2014
6578	D. Humelnicu	Adsorptive Performance of Soy Bran and Mustard Husk Towards Arsenic (V) Ions from Synthetic Aqueous Solutions	2019
6579	D. V. Dmitriev	Transformation of the InP(001) surface upon annealing in an arsenic flux	2021
6580	Y. Guo	The Brd4/c-myc/hTert signal pathway was involved in the differentiation induction of HL-60 cells induced by low concentration As2O3 plus VPA	2019
6581	Y. Y. Chang	Promotion and inhibition of oxidase-like nanoceria and peroxidase-like iron oxide by arsenate and arsenite	2021
6582	B. Kollander	Inorganic arsenic in food products on the Swedish market and a risk-based intake assessment	2019
6583	M. Deng	A facile route of mesoporous TiO2 shell for enhanced arsenic removal	2021
6584	M. K. Yadav	Status and management of arsenic pollution in groundwater: A comprehensive appraisal of recent global scenario, human health impacts, sustainable field-scale treatment technologies	2021
6585	L. F. Hu	COMPARISON OF THE STABILIZING CAPABILITIES OF IRON AND ALUMINUM IN TREATING WASTE HIGHLY CONTAMINATED WITH ARSENIC	2016
6586	E. C. Robbins	Removal of As(III) and As(V) in surface modified ceramic filters	2014

6587	S. Q. Kong	Adsorption/Oxidation of Arsenic in Groundwater by Nanoscale Fe-Mn Binary Oxides Loaded on Zeolite	2014
6588	S. Garcia	Study of As(III) and As(V) oxoanion adsorption onto single and mixed ferrite and hausmannite nanomaterials	2014
6589	T. J. Sorg	Regenerating an Arsenic Removal Iron-Based Adsorptive Media System, Part 1: The Regeneration Process	2017
6590	J. Sanchez	LIQUID-PHASE POLYMER-BASED RETENTION TO REMOVE ARSENIC FROM WATER	2019
6591	M. B. Gumpu	Electrochemical sensing platform for the determination of arsenite and arsenate using electroactive nanocomposite electrode	2018
6592	P. B. Hu	Arsenic adsorption enhancement performances of Mn-modified gamma-Al2O3 with flue gas constituents involved	2022
6593	R. Haas-Nuesch	Mineralogical characterization of scalings formed in geothermal sites in the Upper Rhine Graben before and after the application of sulfate inhibitors	2018
6594	K. Rufanov	Polyelement substituted cyclopentadienes and indenes - Novel ligand precursors for organotransition metal chemistry	1997
6595	D. C. Ellinsworth	Arsenic, Reactive Oxygen, and Endothelial Dysfunction	2015
6596	R. W. Veloso	Seasonal impacts on arsenic mobility and geochemistry in streams surrounding a gold mineralization area, Paracatu, Brazil	2019
6597	M. Barman	Nutritional impact on Immunological maturation during Childhood in relation to the Environment (NICE): a prospective birth cohort in northern Sweden	2018
6598	E. Toral-Sanchez	Characterization of iron-modified carbon paste electrodes and their application in As(V) detection	2016
6599	Y. J. Shih	Adsorptive removal of arsenic using a novel akhtenskite coated waste goethite	2015
6600	W. D. Chen	Distribution and ecological risk assessment of arsenic and some trace elements in soil of different land use types, Tianba Town, China	2021
6601	O. Lazareva	Understanding arsenic behavior in carbonate aquifers: Implications for aquifer storage and recovery (ASR)	2015
6602	K. Y. Kum	Danshen (Salvia miltiorrhiza) on the Global Market: What Are the Implications for Products' Quality?	2021
6603	D. P. Zhong	Separation of arsenic from arsenic-antimony-bearing dust through selective oxidation-sulfidation roasting with CuS	2020
6604	I. A. Katsoyiannis	Arsenic occurrence in Europe: emphasis in Greece and description of the applied full-scale treatment plants	2015

6605	W. Wu	Pancreatic islet-autonomous effect of arsenic on insulin secretion through endoplasmic reticulum stress-autophagy pathway	2018
6606	Y. Peng	Insight into Deactivation of Commercial SCR Catalyst by Arsenic: An Experiment and DFT Study	2014
6607	C. L. Yuan	Community dynamics of As(V)-reducing and As(III)-oxidizing genes during a wet-dry cycle in paddy soil amended with organic matter, gypsum, or iron oxide	2020
6608	A. A. H. Faisal	Humic acid coated sand as a novel sorbent in permeable reactive barrier for environmental remediation of groundwater polluted with copper and cadmium ions	2020
6609	A. Negrea	Phosphonium grafted styrene-divinylbenzene resins impregnated with iron(III) and crown ethers for arsenic removal	2014
6610	W. Piao	Arsenic trioxide degrades NPM-ALK fusion protein and inhibits growth of ALK-positive anaplastic large cell lymphoma	2017
6611	A. P. Sanders	Association between arsenic, cadmium, manganese, and lead levels in private wells and birth defects prevalence in North Carolina: a semi-ecologic study	2014
6612	P. K. Raul	Iron oxide hydroxide nanoflower assisted removal of arsenic from water	2014
6613	E. Islam	Biochemical mechanisms of signaling: Perspectives in plants under arsenic stress	2015
6614	Q. Lu	Regeneration of commercial SCR catalyst deactivated by arsenic poisoning in coal-fired power plants	2019
6615	C. Nunez	Development of a fast and sensitive method for the determination of As(III) at trace levels in urine by differential pulse anodic voltammetry using a simple graphene screen-printed electrode	2020
6616	M. Yu	Adsorption kinetic properties of As(III) on synthetic nano Fe-Mn binary oxides	2016
6617	K. Wu	Removal of arsenic(III, V) by a granular Mn-oxide-doped Al oxide adsorbent: surface characterization and performance	2017
6618	P. J. Harvey	Chemical, biological, and DNA markers for tracing slaughterhouse effluent	2017
6619	S. U. Khan	Energy Efficient Rapid Removal of Arsenic in an Electrocoagulation Reactor with Hybrid Fe/Al Electrodes: Process Optimization Using CCD and Kinetic Modeling	2020
6620	A. A. MacKay	Seasonal Arsenic Accumulation in Stream Sediments at a Groundwater Discharge Zone	2014
6621	C. Yin	Structure-tunable trivalent Fe-Al-based bimetallic organic frameworks for arsenic removal from contaminated water	2022
6622	A. Dey	Cobalt ferrite nanoparticles aggregated schwertmannite: A novel adsorbent for the efficient removal of arsenic	2014

6623	Z. Jamal	Arsenic-induced immunomodulatory effects disorient the survival-death interface by stabilizing the Hsp90/Beclin1 interaction	2020
6624	I. Andjelkovic	Bacterial iron-oxide nanowires from biofilm waste as a new adsorbent for the removal of arsenic from water	2017
6625	J. Chen	Volatilization of Arsenic from Polluted Soil by Pseudomonas putida Engineered for Expression of the arsM Arsenic(III) S-Adenosine Methyltransferase Gene	2014
6626	S. Joshi	Arsenic Removal from Water by Adsorption onto Iron Oxide/Nano-Porous Carbon Magnetic Composite	2019
6627	H. Sugita	Effects of Silicic Acid on Leaching Behavior of Arsenic from Spent Magnesium-Based Adsorbents Containing Arsenite	2022
6628	B. M. Gunasekaran	Electrochemical Sensing of Arsenic Ions Using a Covalently Functionalized Benzotriazole-Reduced Graphene Oxide-Modified Screen-Printed Carbon Electrode	2022
6629	M. Jablonska-Czapla	The mobility of arsenic and its species in selected herbs	2019
6630	W. G. Cao	Increases in groundwater arsenic concentrations and risk under decadal groundwater withdrawal in the lower reaches of the Yellow River basin, Henan Province, China	2022
6631	L. H. Liu	Arsenic detoxification by iron-manganese nodules under electrochemically controlled redox: Mechanism and application	2021
6632	J. Bae	Adsorptive Removal of Arsenic by Mesoporous Iron Oxide in Aquatic Systems	2020
6633	N. Chomchoey	Effect of Calcination Temperature on the Magnetic Characteristics of Synthetic Iron Oxide Magnetic Nanoparticles for Arsenic Adsorption	2018
6634	D. Ocinski	Evaluation of hybrid polymer containing iron oxides as As(III) and As(V) sorbent for drinking water purification	2014
6635	N. Ajith	Interaction of arsenic(III) and arsenic(V) on manganese dioxide: XPS and electrochemical investigations	2019
6636	E. A. Karam	Study of interaction effect between triacontanol and nitric oxide on alleviating of oxidative stress arsenic toxicity in coriander seedlings	2017
6637	J. Liu	Environmental Behavior, Potential Phytotoxicity, and Accumulation of Copper Oxide Nanoparticles and Arsenic in Rice Plants	2018
6638	C. B. Wang	Experimental and Mechanism Study of Gas-Phase Arsenic Adsorption Over Fe2O3/gamma-Al2O3 Sorbent in Oxy-Fuel Combustion Flue Gas	2016
6639	M. Khatamian	Synthesis and characterization of RGO/zeolite composites for the removal of arsenic from contaminated water	2015
6640	B. Yadu	Dimethylthiourea antagonizes oxidative responses by up-regulating expressions of pyrroline-5-carboxylate synthetase and antioxidant genes under arsenic stress	2019

6641	T. G. Asere	Removal of Arsenic (V) from Aqueous Solutions Using Chitosan-Red Scoria and Chitosan-Pumice Blends	2017
6642	M. L. Zhang	An alternative approach for nitrate and arsenic removal from wastewater via a nitrate-dependent ferrous oxidation process	2018
6643	K. M. Smiech	Comparing Mixed-Media and Conventional Slow-Sand Filters for Arsenic Removal from Groundwater	2018
6644	H. Ahmad	Magnetite Loaded Cross-linked Polystyrene Composite Particles Prepared by Modified Suspension Polymerization and Their Potential Use as Adsorbent for Arsenic(III)	2017
6645	C. C. Li	Occurrence and behavior of arsenic in groundwater-aquifer system of irrigated areas	2022
6646	R. R. Pawar	Efficient removal of hazardous lead, cadmium, and arsenic from aqueous environment by iron oxide modified clay-activated carbon composite beads	2018
6647	H. T. Wang	Bioavailability and risk assessment of arsenic in surface sediments of the Yangtze River estuary	2016
6648	E. D. Stewart	Correlating Bedrock Folds to Higher Rates of Arsenic Detection in Groundwater, Southeast Wisconsin, USA	2021
6649	S. Pathan	Arsenic Removal Using "Green" Renewable Feedstock-Based Hydrogels: Current and Future Perspectives	2018
6650	S. Purwajanti	Synthesis of Magnesium Oxide Hierarchical Microspheres: A Dual-Functional Material for Water Remediation	2015
6651	A. K. Patel	Arsenic mobility and potential co-leaching of fluoride from the sediments of three tributaries of the Upper Brahmaputra floodplain, Lakhimpur, Assam, India	2019
6652	Y. Yu	Rare-earth metal based adsorbents for effective removal of arsenic from water: A critical review	2018
6653	H. Hernandez-Flores	Concrete/maghemite nanocomposites as novel adsorbents for arsenic removal	2018
6654	Y. Yu	Cerium oxide modified activated carbon as an efficient and effective adsorbent for rapid uptake of arsenate and arsenite: Material development and study of performance and mechanisms	2017
6655	E. Gullberg	Selection of a Multidrug Resistance Plasmid by Sublethal Levels of Antibiotics and Heavy Metals	2014
6656	H. T. Hu	Arsenic trioxide intravenous infusion combined with transcatheter arterial chemoembolization for the treatment of hepatocellular carcinoma with pulmonary metastasis: Long-term outcome analysis	2017
6657	E. Gliozzo	Pigments-Arsenic-based yellows and reds	2022
6658	R. Nistico	Sustainable magnet-responsive nanomaterials for the removal of arsenic from contaminated water	2018

6659	C. Grover	The nail as an investigative tool in medicine: What a dermatologist ought to know	2017
6660	L. N. Lin	Reduced arsenic accumulation in indica rice (Oryza sativa L.) cultivar with ferromanganese oxide impregnated biochar composites amendments	2017
6661	F. Zhang	Effect of different DOM components on arsenate complexation in natural water	2021
6662	T. Umezu	Toxicokinetic characteristics and effects of diphenylarsinic acid on dopamine in the striatum of free-moving mice	2021
6663	H. Fakour	Effect of Humic Acid on Arsenic Adsorption and Pore Blockage on Iron-Based Adsorbent	2015
6664	G. L. Li	Unusual Structures of the Parent Molecules Diarsene, Distibene, and Dibismuthene: Toward Their Observation	2020
6665	S. Misra	Selenite promotes all-trans retinoic acid-induced maturation of acute promyelocytic leukemia cells	2016
6666	H. Kohlmann	Crystal structure and hydrogenation properties of Pd5As	2016
6667	F. Hamidi	Ionic gelation synthesis, characterization and adsorption studies of cross-linked chitosan-tripolyphosphate (CS-TPP) nanoparticles for removal of As (V) ions from aqueous solution: kinetic and isotherm studies	
6668	I. A. Reyes	A study on the dissolution rates of K-Cr(VI)-jarosites: kinetic analysis and implications	2016
6669	Y. E. Tyutereva	Synergetic effect of potassium persulfate on photodegradation of para-arsanilic acid in Fe(III) oxalate system	2021
6670	M. Havelcova	Chemical characterization of mountain forest soils: impact of long-term atmospheric deposition loadings (Czech-Polish-German border region)	2020
6671	R. N. Moussawi	Modification of nanostructured ZnO surfaces with curcumin: fluorescence-based sensing for arsenic and improving arsenic removal by ZnO	2016
6672	U. Feistel	Field tests of a small pilot plant for the removal of arsenic in groundwater using coagulation and filtering	2016
6673	F. Luziatelli	Genome Sequencing of Pantoea agglomerans C1 Provides Insights into Molecular and Genetic Mechanisms of Plant Growth-Promotion and Tolerance to Heavy Metals	2020
6674	Z. J. Yong	A sequential treatment of intermediate tropical landfill leachate using a sequencing batch reactor (SBR) and coagulation	2018
6675	L. F. Li	A Cereblon Modulator CC-885 Induces CRBN- and p97-Dependent PLK1 Degradation and Synergizes with Volasertib to Suppress Lung Cancer	2020
6676	M. A. Gomez-Moron	A new insight into the vaults of the kings in the Alhambra (Granada, Spain) by combination of portable XRD and XRF	2016

6677	D. Cecconet	In situ groundwater remediation with bioelectrochemical systems: A critical review and future perspectives	2020
6678	R. K. Alfarawati	Solar photocatalytic removal of arsenic from polluted water using carbon-modified titanium oxide nanoparticles supported on activated carbon	2020
6679	W. C. Stafford	Irreversible inhibition of cytosolic thioredoxin reductase 1 as a mechanistic basis for anticancer therapy	2018
6680	C. C. Yang	Influence of tunneling barrier width on the forward current characteristics of In As-based microwave p-n diode	2015
6681	Q. Z. Tian	Effect of Si/Al molar ratio on the immobilization of selenium and arsenic oxyanions in geopolymer	2021
6682	M. V. Martinez	Simple Electrochemical Detection Method Employing a Hydrogel Soft Matrix: Application in Tap Water	2018
6683	I. Lopez-Garcia	Magnetic ferrite particles combined with electrothermal atomic absorption spectrometry for the speciation of low concentrations of arsenic	2018
6684	H. Niemikoski	Metabolism and cytotoxicity of diphenylarsinic acid, a degradation product of sea-dumped chemical warfare agents, in a rainbow trout liver cell line	2021
6685	M. Mercurio	May a comprehensive mineralogical study of a jackstone calculus and some other human bladder stones unveil health and environmental implications?	
6686	M. Berger	Understanding Our Energy Footprint: Undergraduate Chemistry Laboratory Investigation of Environmental Impacts of Solid Fossil Fuel Wastes	2017
6687	S. Yoshida	Removal of As(III) and As(V) with Fe-Al type Composite Oxides	2017
6688	E. Demers	Macrophyte Potential to Treat Leachate Contaminated with Wood Preservatives: Plant Tolerance and Bioaccumulation Capacity	2020
6689	G. Orasen	Continuous Flooding or Alternate Wetting and Drying Differently Affect the Accumulation of Health-Promoting Phytochemicals and Minerals in Rice Brown Grain	2019
6690	J. Hrabeta	Metal Containing Cytostatics and Their Interaction with Cellular Thiol Compounds Causing Chemoresistance	2016
6691	S. Ghosh	Graveoline Isolated from Ethanolic Extract of Ruta graveolens Triggers Apoptosis and Autophagy in Skin Melanoma Cells: A Novel Apoptosis-Independent Autophagic Signaling Pathway	2014
6692	P. Bellavite	Cell sensitivity, non-linearity and inverse effects	2015
6693	R. Xu	Novel slow release ammonium persulfate capsules for in situ remediation of high arsenic groundwater	2021
6694	X. H. Yang	Removal of arsenic from water through ceramic filter modified by nano-CeO2: A cost-effective approach for remote areas	2021

6695	P. Zhang	Identification of a new high-molecular-weight Fe-citrate species at low citrate-to-Fe molar ratios: Impact on arsenic removal with ferric hydroxide	2018
6696	H. Zhang	Parity-breaking in single-element phases: ferroelectric-like elemental polar metals	2018
6697	B. C. Lipchick	Oxidative stress and proteasome inhibitors in multiple myeloma	2016
6698	F. Renard	Siderite dissolution coupled to iron oxyhydroxide precipitation in the presence of arsenic revealed by nanoscale imaging	2017
6699	E. Bigsby	Information Seeking and Risk Reduction Intentions in Response to Environmental Threat Messages: The Role of Message Processing	
6700	Z. Q. Zhou	Perpendicular Optical Reversal of the Linear Dichroism and Polarized Photodetection in 2D GeAs	2018
6701	A. A. Meharg	The Pedosphere as a Sink, Source, and Record of Anthropogenic and Natural Arsenic Atmospheric Deposition	2021
6702	T. Yasutaka	Development of a green remediation tool in Japan	2016
6703	J. M. Fan	Enhanced As (V) Removal from Aqueous Solution by Biochar Prepared from Iron-Impregnated Corn Straw	2018
6704	W. Y. Lee	Development of Isotope Dilution LC-MS/MS Method for Accurate Determination of Arsenobetaine in Oyster Certified Reference Material	2014
6705	H. X. Linh	Electrochemical mass production of graphene nanosheets for arsenic removal from aqueous solutions	2019
6706	S. R. Chowdhury	Recycling of nickel smelter slag for arsenic remediation-an experimental study	2014
6707	X. J. Zha	Hydrochemical characteristics of surface waters and their relationships to the Kashin-Beck Disease in Longzi County, Tibet	2022
6708	A. Arjoon	Kinetics of heavy metal inhibition of 1,2-dichloroethane biodegradation in co-contaminated water	2015
6709	M. Kersten	Surface complexation modeling of arsenate adsorption by akageneite (beta-FeOOH)-dominant granular ferric hydroxide	2014
6710	R. Shirmehenji	A Green Approach to the Bio-based Synthesis of Selenium Nanoparticles from Mining Waste	2021
6711	N. Zhang	Source profile and excess cancer risk evaluation of environmental tobacco smoking under real conditions, China	2019
6712	M. Eskola	Towards a dietary-exposome assessment of chemicals in food: An update on the chronic health risks for the European consumer	2020

6713	S. Chen	Biogeochemical transformation of sulfur and its effects on arsenic mobility in paddy fields polluted by acid mine drainage	2022
6714	A. P. Singh	A protective role for nitric oxide and salicylic acid for arsenite phytotoxicity in rice (Oryza sativa L.)	2017
6715	D. P. Wu	Evolution of In-Plane Electrical Transport Properties of Ru Doped BaFe2As2 System Ba(Fe1-xRux)(2)As-2	2020
6716	A. Fromant	Wide range of metallic and organic contaminants in various tissues of the Antarctic prion, a planktonophagous seabird from the Southern Ocean	2016
6717	G. Borisova	Thiols as biomarkers of heavy metal tolerance in the aquatic macrophytes of Middle Urals, Russia	2016
6718	Z. Yang	Effects of brining on the corrosion of ZVI and its subsequent As(III/V) and Se(IV/VI) removal from water	2017
6719	B. Paul	Graphene in the Fe3O4 nano-composite switching the negative influence of humic acid coating into an enhancing effect in the removal of arsenic from water	2015
6720	S. Saha	Attenuative role of mangiferin in oxidative stress-mediated liver dysfunction in arsenic-intoxicated murines	2016
6721	X. J. Xie	In situ treatment of arsenic contaminated groundwater by aquifer iron coating: Experimental study	2015
6722	B. Mueller	Climatic variations and de-coupling between arsenic and iron in arsenic contaminated ground water in the lowlands of Nepal	2018
6723	G. S. Zhang	Polyvinyl alcohol-stabilized granular Fe-Mn binary oxide as an effective adsorbent for simultaneous removal of arsenate and arsenite	2020
6724	M. P. Jian	Adsorptive removal of arsenic from aqueous solution by zeolitic imidazolate framework-8 (ZIF-8) nanoparticles	2015
6725	K. Khanna	Metal resistant PGPR lowered Cd uptake and expression of metal transporter genes with improved growth and photosynthetic pigments in Lycopersicon esculentum under metal toxicity	2019
6726	P. Rodriguez	In situ cleaning of InGaAs surfaces prior to low contact resistance metallization	2016
6727	P. Devi	Progress in the materials for optical detection of arsenic in water	2019
6728	J. B. Wang	Molecular mechanisms and therapeutic relevance of gasdermin E in human diseases	2022
6729	X. N. Guo	Pigment epithelium-derived factor (PEDF) ameliorates arsenic-induced vascular endothelial dysfunction in rats and toxicity in endothelial EA.hy926 cells	2020
6730	M. J. Uddin	Review: Efficiently performing periodic elements with modern adsorption technologies for arsenic removal	2020

6731	Y. F. Huang	Efficient oxidation and adsorption of As(III) and As(V) in water using a Fenton-like reagent, (ferrihydrite)-loaded biochar	2020
6732	A. K. Mensah	Biochar, compost, iron oxide, manure, and inorganic fertilizer affect bioavailability of arsenic and improve soil quality of an abandoned arsenic-contaminated gold mine spoil	2022
6733	Y. F. Lin	One-pot synthesis of paramagnetic iron(III) hydroxide nanoplates and ferrimagnetic magnetite nanoparticles for the removal of arsenic ions	2014
6734	N. I. Ilic	The sorption of inorganic arsenic on modified sepiolite: the effect of hydrated iron(III) oxide	2014
6735	J. C. M. Gamboa	Vibrating screen printed electrode of gold nanoparticle-modified carbon nanotubes for the determination of arsenic(III)	2014
6736	Q. Yu	Reactive transport model for predicting arsenic transport in groundwater system in Datong Basin	2018
6737	K. S. Sista	Iron Powders as a Potential Material for Arsenic Removal in Aqueous Systems	2021
6738	M. Kumar	Removal of toxic arsenic from aqueous media using polyphenylsulfone/cellulose acetate hollow fiber membranes containing zirconium oxide	2020
6739	G. H. Qiu	Mechanisms of interaction between arsenian pyrite and aqueous arsenite under anoxic and oxic conditions	2018
6740	J. W. Reid	Crystal structure and X-ray absorption spectroscopy of trimethylarsine oxide dihydrate, (CH3)(3)AsO.2H(2)O	2020
6741	M. Kofronova	Two facets of world arsenic problem solution: crop poisoning restriction and enforcement of phytoremediation	2018
6742	S. I. Siddiqui	Nanohybrid composite Fe2O3-ZrO2/BC for inhibiting the growth of bacteria and adsorptive removal of arsenic and dyes from water	2019
6743	H. U. So	Arsenic in Holocene aquifers of the Red River floodplain, Vietnam: Effects of sediment-water interactions, sediment burial age and groundwater residence time	2018
6744	X. Sun	Arsenic-induced testicular toxicity in Gallus gallus: Expressions of inflammatory cytokines and heat shock proteins	2017
6745	Y. Choi	Adsorptive removal of arsenate using inorganic magnetite particles	2016
6746	T. Siddique	Electrospun Composite Nanofiltration Membranes for Arsenic Removal	2022
6747	M. Wei	Remediation of arsenic-cationic metals from smelter contaminated soil by washings of Na(2)EDTA and phosphoric acid: removal efficiencies and mineral transformation	2021
6748	S. Bae	Provision of folic acid for reducing arsenic toxicity in arsenic-exposed children and adults	2021

6749	H. Khan	Significance of Inflammation and Apoptosis in Hepatocellular Death in Rat, Co-treated with Arsenic and Fluoride	2022
6750	A. J. Henegar	Comparison of the reactivity of alkyl and alkyl amine precursors with native oxide GaAs(100) and InAs(100) surfaces	2016
6751	M. D. Gott	Chromatographic separation of germanium and arsenic for the production of high purity As-77	2016
6752	S. R. Safi	Development and regeneration of composite of cationic gel and iron hydroxide for adsorbing arsenic from ground water	2019
6753	B. C. Thompson	Nicotinamide Enhances Repair of Arsenic and Ultraviolet Radiation-Induced DNA Damage in HaCaT Keratinocytes and Ex Vivo Human Skin	2015
6754	D. V. Dmitriev	Substitution of Phosphorus at the InP(001) Surface Upon Annealing in an Arsenic Flux	2021
6755	C. N. Lange	Mineral profile exploratory analysis for rice grains traceability	2019
6756	B. W. Chen	Identification of Methylated Dithioarsenicals in the Urine of Rats Fed with Sodium Arsenite	2016
6757	H. Fakour	Experimental determination and modeling of arsenic complexation with humic and fulvic acids	2014
6758	S. S. Ramim	Removal of arsenic from groundwater using iron-coated jute-mesh structure	2017
6759	C. Xiao	Arsenic releasing mechanisms during clayey sediments compaction: An experiment study	2021
6760	Z. Chen	Addition of graphene sheets enhances reductive dissolution of arsenic and iron from arsenic contaminated soil	2018
6761	Z. Olah	Novel ion exchange chromatography method for nca arsenic separation	2017
6762	D. Magnone	Biomarker-indicated extent of oxidation of plant-derived organic carbon (OC) in relation to geomorphology in an arsenic contaminated Holocene aquifer, Cambodia	2017
6763	N. Dutta	Electrocoagulation for Arsenic Removal: Field Trials in Rural West Bengal	2021
6764	F. Barraque	Arsenate removal from aqueous solution by montmorillonite and organo-montmorillonite magnetic materials	2021
6765	T. Rana	Effect of Pleurotus florida lectin (PFL) on arsenic-induced activities of splenoctyes in rat	2014
6766	X. Peng	One-step and acid free synthesis of gamma-Fe2O3/SBA-15 for enhanced arsenic removal	2018

6767	M. Martinez-Cabanas	Achieving sub-10 ppb arsenic levels with iron based biomass-silica gel composites	2015
6768	B. Rathi	Processes governing arsenic retardation on Pleistocene sediments: Adsorption experiments and model-based analysis	2017
6769	Y. Peng	Deactivation and regeneration of a commercial SCR catalyst: Comparison with alkali metals and arsenic	2015
6770	N. Shabnam	Iron (III) oxide nanoparticles alleviate arsenic induced stunting in Vigna radiata	2019
6771	T. T. Q. Nguyen	Removing arsenic from water with an original and modified natural manganese oxide ore: batch kinetic and equilibrium adsorption studies	2020
6772	E. Shumilin	Increasing arsenic mobility in the fine fraction of the dry stream sediments of the semi-arid San Antonio gold mining district (Baja California peninsula, Mexico)	2015
6773	N. Rajindran	Physicochemical Properties of a New Green Honey from Banggi Island, Sabah	2022
6774	B. America	Geochemical trends of arsenic and iron in a small reservoir that receives mine drainage	2018
6775	J. M. Mao	Arsenic trioxide mediates HAPI microglia inflammatory response and subsequent neuron apoptosis through p38/JNK MAPK/STAT3 pathway	2016
6776	S. Zakari	Influence of sulfur amendments on heavy metals phytoextraction from agricultural contaminated soils: A meta-analysis*	2021
6777	J. S. Zhou	Adsorption behavior and mechanism of arsenic on mesoporous silica modified by iron-manganese binary oxide (FeMnOx/SBA-15) from aqueous systems	2020
6778	J. F. Sui	Iron-naphthalenedicarboxylic acid gels and their high efficiency in removing arsenic(v)	2016
6779	C. Kaya	Salicylic acid-induced nitric oxide enhances arsenic toxicity tolerance in maize plants by upregulating the ascorbate-glutathione cycle and glyoxalase system	2020
6780	F. Xiao	One-step synthesis of aluminum magnesium oxide nanocomposites for simultaneous removal of arsenic and lead ions in water	2015
6781	Q. T. Shi	Oxidation of Arsenite by Epoxy Group on Reduced Graphene Oxide/Metal Oxide Composite Materials	2020
6782	P. Drahota	Mobility and attenuation of arsenic in sulfide-rich mining wastes from the Czech Republic	2016
6783	H. Liu	Separate As(V) from solution by mesoporous Y-Al binary oxide: batch experiments	2018
6784	A. Ghorbani	Nitric oxide could allay arsenic phytotoxicity in tomato (Solanum lycopersicum L.) by modulating photosynthetic pigments, phytochelatin metabolism, molecular redox status and arsenic sequestration	2021

6785	L. P. Lingamdinne	Process modeling and optimization of an iron oxide immobilized graphene oxide gadolinium nanocomposite for arsenic adsorption	2020
6786	K. Kim	Three-dimensional, printed water-filtration system for economical, on-site arsenic removal	2020
6787	C. J. Meakin	Inorganic Arsenic as an Endocrine Disruptor: Modulation of the Glucocorticoid Receptor Pathway in Placental Cells via CpG Methylation	2019
6788	Y. Shi	Adsorption performance and its mechanism of aqueous As(III) on polyporous calcined oyster shell-supported Fe-Mn binary oxide	2022
6789	C. M. Hammond	Arsenic and iron speciation and mobilization during phytostabilization of pyritic mine tailings	2020
6790	P. K. Singh	Nitric oxide mediated transcriptional modulation enhances plant adaptive responses to arsenic stress	2017
6791	K. Z. Elwakeel	Arsenic(V) sorption using chitosan/Cu(OH)(2) and chitosan/CuO composite sorbents	2015
6792	A. D. Robles	Geochemical mobility of arsenic in the surficial waters from Argentina	2016
6793	Y. W. Yin	Adsorption of arsenic by activated charcoal coated zirconium-manganese nanocomposite: Performance and mechanism	2019
6794	K. V. Saravi	Contribution of Funneliformis mosseae symbiosis to the regulation of sulfur assimilation, glyoxalase system and ionic homeostasis in Aloysia citriodora Palau under cadmium toxicity	
6795	K. Z. Benis	A binary oxide-biochar composite for adsorption of arsenic from aqueous solutions: Combined microwave pyrolysis and electrochemical modification	2022
6796	D. D. Sprague	Legacy Arsenic Pollution of Lakes Near Cobalt, Ontario, Canada: Arsenic in Lake Water and Sediment Remains Elevated Nearly a Century After Mining Activity Has Ceased	2018
6797	R. Nagar	Drinking Water Treatment Residual Amendment Lowers Inorganic Arsenic Bioaccessibility in Contaminated soils: a Long-Term Study	2015
6798	A. Ahmad	Synergistic effects of nitric oxide and silicon on promoting plant growth, oxidative stress tolerance and reduction of arsenic uptake in Brassica juncea	2021
6799	O. H. Elshenawy	Modulation of aryl hydrocarbon receptor-regulated enzymes by trimethylarsine oxide in C57BL/6 mice: In vivo and in vitro studies	2015
6800	S. Bortnikova	Mechanisms of low-temperature vapor-gas streams formation from sulfide mine waste	2019
6801	M. I. R. Khan	Phosphorus supplementation modulates nitric oxide biosynthesis and stabilizes the defence system to improve arsenic stress tolerance in mustard	2021
6802	R. Singh	Arsenic contamination, consequences and remediation techniques: A review	2015

6803	T. K. Sahu	Efficient and Rapid Removal of Environmental Malignant Arsenic(III) and Industrial Dyes Using Reusable, Recoverable Ternary Iron Oxide - ORMOSIL - Reduced Graphene Oxide Composite	2017
6804	U. K. Sahu	Application of Box-Behnken Design in response surface methodology for adsorptive removal of arsenic from aqueous solution using CeO2/Fe2O3/graphene nanocomposite	2018
6805	T. S. Sarath	Atorvastatin ameliorates arsenic-induced hypertension and enhancement of vascular redox signaling in rats	2014
6806	B. O'Shea	Heterogeneous arsenic enrichment in meta-sedimentary rocks in central Maine, United States	2015
6807	E. Khan	Detecting inorganic arsenic below WHO threshold limit; A comparative study of various sensors	
6808	A. Majumder	Green synthesis of iron oxide nanoparticles for arsenic remediation in water and sludge utilization	2019
6809	M. L. Chen	Akaganeite decorated graphene oxide composite for arsenic adsorption/removal and its proconcentration at ultra-trace level	2015
6810	B. Liu	A review of functional sorbents for adsorptive removal of arsenic ions in aqueous systems	2020
6811	B. A. Bessinger	Effectiveness of Monitored Natural Attenuation (MNA) as a Groundwater Remedy for Arsenic in Phosphatic Wastes	2019
6812	A. Roy	Concrete stabilization of arsenic-bearing iron sludge generated from an electrochemical arsenic remediation plant	2019
6813	C. Osorio-Lopez	As(V) adsorption on forest and vineyard soils and pyritic material with or without mussel shell: Kinetics and fractionation	2014
6814	B. Gankhurel	Arsenic and uranium contamination of Orog Lake in the Valley of Gobi Lakes, Mongolia: Field evidence of conservative accumulation of U in an alkaline, closed-basin lake during evaporation	2022
6815	A. K. O. Huq	Equilibrium, kinetics, and thermodynamics studies of polypyrrole adsorbent for arsenic ions	2018
6816	S. R. Panthi	Kinetic study of adsorption of arsenic onto New Zealand Ironsand (NZIS)	2014
6817	S. R. S. Bandaru	Rapid and Efficient Arsenic Removal by Iron Electrocoagulation Enabled with in Situ Generation of Hydrogen Peroxide	2020
6818	Z. Jiang	Arsenic mobilization in a high arsenic groundwater revealed by metagenomic and Geochip analyses	2019
6819	B. H. Li	Effect of temperature on clostridium sp. strain pxl2 and removal of arsenic and nitrate from groundwater	2019
6820	A. K. Tolkou	Removal of Arsenic, Chromium and Uranium from Water Sources by Novel Nanostructured Materials Including Graphene-Based Modified Adsorbents: A Mini Review of Recent Developments	2020

6821	H. B. Ahmad	Purification of drinking water by hydroponic technique	2017
6822	M. A. Rehman	Arsenic Adsorption Using Palm Oil Waste Clinker Sand Biotechnology: an Experimental and Optimization Approach	2015
6823	H. Jelenova	Geochemical and mineralogical characterization of the arsenic-, iron-, and sulfur-rich mining waste dumps near Kank, Czech Republic	2018
6824	T. Narukawa	Reversed Phase Column HPLC-ICP-MS Conditions for Arsenic Speciation Analysis of Rice Flour	2015
6825	Q. H. Wu	Effects and Mechanisms of Copper Oxide Nanoparticles with Regard to Arsenic Availability in Soil-Rice Systems: Adsorption Behavior and Microbial Response	2022
6826	H. B. Kim	Photo-induced redox coupling of dissolved organic matter and iron in biochars and soil system: Enhanced mobility of arsenic	2019
6827	T. Liang	Adsorption of As(V) by the Novel and Efficient Adsorbent Cerium-Manganese Modified Biochar	2020
6828	X. L. Yan	Enhanced delivery of engineered Fe-Mn binary oxides in heterogeneous porous media for efficient arsenic stabilization	2022
6829	S. Y. Wang	Fabrication of chitosan-based MCS/ZnO@Alg gel microspheres for efficient adsorption of As(V)	2019
6830	Z. X. Li	Neuroprotective effects of protocatechuic acid on sodium arsenate induced toxicity in mice: Role of oxidative stress, inflammation, and apoptosis	2021
6831	X. S. Wei	Application of mesoporous zirconia coating on coal cinder for inorganic arsenic removal from aqueous solution	2015
6832	Y. S. Liu	Band Structure, Band Offsets, and Intrinsic Defect Properties of Few-Layer Arsenic and Antimony	2020
6833	L. A. Zemskova	Chitosan based composite sorbents for arsenic removal	2019
6834	I. Carabante	Reutilization of Porous Sintered Hematite Bodies as Effective Adsorbents for Arsenic(V) Removal from Water	2014
6835	S. Ploychompoo	Fast and efficient aqueous arsenic removal by functionalized MIL-100(Fe)/rGO/delta-MnO2 ternary composites: Adsorption performance and mechanism	2020
6836	A. L. Singh	Arsenic sequestration by manganese-oxidizing Acinetobacter sp	2016
6837	G. Lee	Passive treatment of arsenic and heavy metals contaminated circumneutral mine drainage using granular polyurethane impregnated by coal mine drainage sludge	2018
6838	A. Otero-Farina	Surface Complexation Modelling of Arsenic and Copper Immobilization by Iron Oxide Precipitates Derived from Acid Mine Drainage	2015

6839	R. Fl'akova	OCCURRENCE OF ANTIMONY AND ARSENIC AT MINING SITES IN SLOVAKIA: IMPLICATIONS FOR THEIR MOBILITY	2017
6840	S. Deeprasert	Dimpled SiO2@gamma-Fe2O3 nanocomposites - fabrication and use for arsenic adsorption in aqueous medium	2021
6841	Y. Yin	Removal of inorganic arsenic from aqueous solution by Fe-modified ceramsite: batch studies and remediation trials	2021
6842	M. E. Lee	Simultaneous application of oxalic acid and dithionite for enhanced extraction of arsenic bound to amorphous and crystalline iron oxides	2018
6843	J. S. Liang	Microbial Interspecies Interactions Affect Arsenic Fate in the Presence of Mn-II	2017
6844	D. R. Zhang	Red mud regulates arsenic fate at acidic pH via regulating arsenopyrite bio-oxidation and S, Fe, Al, Si speciation transformation	2021
6845	H. B. Qin	Enrichment mechanisms of antimony and arsenic in marine ferromanganese oxides: Insights from the structural similarity	2019
6846	S. Lal	Exploring carbonaceous nanomaterials for arsenic and chromium removal from wastewater	2020
6847	X. P. Chen	The fate of arsenic in contaminated paddy soil with gypsum and ferrihydrite amendments	2015
6848	R. D. C. Soltani	Decontamination of arsenic(V)-contained liquid phase utilizing Fe3O4/bone char nanocomposite encapsulated in chitosan biopolymer	2017
6849	T. A. Siddique	Nanofiltration for Arsenic Removal: Challenges, Recent Developments, and Perspectives	2020
6850	Y. H. Li	Fixed-bed column adsorption of arsenic(v) by porous composite of magnetite/hematite/carbon with eucalyptus wood microstructure	2018
6851	B. W. Liu	DNA adsorption by magnetic iron oxide nanoparticles and its application for arsenate detection	2014
6852	E. E. Rios-Valenciana	Dissolution and final fate of arsenic associated with gypsum, calcite, and ferrihydrite: Influence of microbial reduction of As(V), sulfate, and Fe(III)	2020
6853	P. A. Bommarito	Effects of prenatal exposure to endocrine disruptors and toxic metals on the fetal epigenome	2017
6854	C. L. Fausey	Removal of arsenic with reduced graphene oxide-TiO2-enabled nanofibrous mats	2019
6855	E. Przezdziecka	The chemical states of As 3d in highly doped ZnO grown by Molecular Beam Epitaxy and annealed in different atmospheres	2016
6856	J. W. Farrell	Arsenic Removal by Nanoscale Magnetite in Guanajuato, Mexico	2014

6857	M. Ferreira	Pre-birth world and the development of the immune system: Mum's diet affects our adult health New insight on how the diet during pregnancy permanently influences offspring health and immune fitness	2014
6858	S. Lata	Removal of arsenic from water using nano adsorbents and challenges: A review	2016
6859	C. Sprenger	Hydrogeochemistry of Urban Floodplain Aquifer Under the Influence of Contaminated River Seepage in Delhi (India)	2014
6860	X. J. Peng	Immobilization of phosphorus, copper, zinc and arsenic in swine manure by activated red mud	2014
6861	P. Waghe	Arsenic causes aortic dysfunction and systemic hypertension in rats: Augmentation of angiotensin II signaling	2015
6862	J. H. Park	Comparison of arsenic co-precipitation and adsorption by iron minerals and the mechanism of arsenic natural attenuation in a mine stream	2016
6863	S. E. Reese	Epigenome-wide meta-analysis of DNA methylation and childhood asthma	2019
6864	L. L. Yu	An approach for identification and determination of arsenic species in the extract of kelp	2015
6865	A. Karthika	A novel highly efficient and accurate electrochemical detection of poisonous inorganic Arsenic (III) ions in water and human blood serum samples based on SrTiO3/beta-cyclodextrin composite	2019
6866	R. Foroutan	Efficient arsenic(V) removal from contaminated water using natural clay and clay composite adsorbents	2019
6867	Z. B. Zhang	The Influence of Dosing Modes of Coagulate on Arsenic Removal	2014
6868	W. Zhang	Enhanced arsenate removal by novel Fe-La composite (hydr)oxides synthesized via coprecipitation	2014
6869	H. Movassagh	Maternal gestational mercury exposure in relation to cord blood T cell alterations and placental gene expression signatures	2021
6870	V. K. Tchieda	Removal of Arsenic by Alumina: Effects of Material Size, Additives, and Water Contaminants	2016
6871	A. Olaniran	Treatment additives reduced arsenic and cadmium bioavailability and increased 1,2-dichloroethane biodegradation and microbial enzyme activities in co-contaminated soil	2017
6872	S. A. Salmataj	Amelioration of arsenic-induced oxidative stress in CHO cells by Ixora coccinea flower extract	2018
6873	J. B. Wang	A kinetic study of concurrent arsenic adsorption and phosphorus release during sediment resuspension	2018
6874	J. A. R. Guivar	Adsorption of arsenite and arsenate on binary and ternary magnetic nanocomposites with high iron oxide content	2018

6875	A. Aftabtalab	Review on the interactions of arsenic, iron (oxy)(hydr)oxides, and dissolved organic matter in soils, sediments, and groundwater in a ternary system	2022
6876	A. Praveen	Mixed plantation of wheat and accumulators in arsenic contaminated plots: A novel way to reduce the uptake of arsenic in wheat and load on antioxidative defence of plant	2019
6877	H. B. Eriksen	Factors Associated with Thymic Size at Birth among Low and Normal Birth-Weight Infants	2014
6878	Lalhmunsiama	Iron-oxide modified sericite alginate beads: A sustainable adsorbent for the removal of As(V) and Pb(II) from aqueous solutions	2017
6879	G. H. Li	Novel iron-supported ZSM-5 molecular sieve remove arsenic from wastewater by heterogeneous nucleation with pH limit breaking	2022
6880	V. Singh	Hijacking microglial glutathione by inorganic arsenic impels bystander death of immature neurons through extracellular cystine/glutamate imbalance	2016
6881	A. Praveen	Iron Oxide Nanoparticles as Nano-adsorbents: A Possible Way to Reduce Arsenic Phytotoxicity in Indian Mustard Plant (Brassica juncea L.)	2018
6882	T. Ghazi	Prenatal Air Pollution Exposure and Placental DNA Methylation Changes: Implications on Fetal Development and Future Disease Susceptibility	2021
6883	M. A. Gomez-Gonzalez	Spread and partitioning of arsenic in soils from a mine waste site in Madrid province (Spain)	2014
6884	J. Y. Zhu	Calcined layered double hydroxides/reduced graphene oxide composites with improved photocatalytic degradation of paracetamol and efficient oxidation-adsorption of As(III)	2018
6885	P. Ahmad	Zinc Oxide Nanoparticles Application Alleviates Arsenic (As) Toxicity in Soybean Plants by Restricting the Uptake of as and Modulating Key Biochemical Attributes, Antioxidant Enzymes, Ascorbate-Glutathione Cycle and Glyoxalase System	2020
6886	M. Filippi	Arsenic mineralogy and mobility in the arsenic-rich historical mine waste dump	2015
6887	M. Mahdavi	A comparison study of granular activated carbon modification by FeCl3 under the acidic and basic condition for arsenic removal from water	2019
6888	Z. Y. Fang	Enhanced Arsenite Removal from Silicate-containing Water by Using Redox Polymer-based Fe(III) Oxides Nanocomposite	2021
6889	B. Zhi	Ordered mesoporous MnO2 as a synergetic adsorbent for effective arsenic(III) removal	2014
6890	T. Gupte	Highly Sensitive As3+ Detection Using Electrodeposited Nanostructured MnOx and Phase Evolution of the Active Material during Sensing	2019
6891	Y. K. Penke	Arsenic surface complexation behavior in aqueous systems onto Al substituted Ni, Co, Mn, and Cu based ferrite nano adsorbents	2019
6892	L. J. Huo	Arsenic availability and uptake by edible rape (Brassica campestris L.) grown in contaminated soils spiked with carboxymethyl cellulose-stabilized ferrihydrite nanoparticles	2018

6893	A. R. Ganesan	Evaluation of in vivo sub-chronic and heavy metal toxicity of under-exploited seaweeds for food application	2020
6894	B. M. Lee	Perspectives on trace chemical safety and chemophobia: risk communication and risk management	2019
6895	C. Tian	Enhanced Adsorption of p-Arsanilic Acid from Water by Amine-Modified UiO-67 as Examined Using Extended X-ray Absorption Fine Structure, X-ray Photoelectron Spectroscopy, and Density Functional Theory Calculations	2018
6896	C. Wu	Effect of silicate on arsenic fractionation in soils and its accumulation in rice plants	2016
6897	N. A. Morales	Total and bioaccessible arsenic and lead in soils impacted by mining exploitation of Fe-oxide-rich ore deposit at Cerro de Mercado, Durango, Mexico	2015
6898	F. V. de Campos	Phytoremediation of arsenite-contaminated environments: is Pistia stratiotes L. a useful tool?	2019
6899	N. Kannan	Physiochemical characterization and toxicity assessment of colloidal mercuric formulation-'Sivanar amirtham'	2021
6900	H. N. Pham	Impact of metal stress on the production of secondary metabolites in Pteris vittata L. and associated rhizosphere bacterial communities	2017
6901	S. Arlt	Cyanido Antimonate(III) and Bismuthate(III) Anions	2016
6902	L. K. de Oliveira	Interaction of arsenic species with tropical river aquatic humic substances enriched with aluminum and iron	2016
6903	X. M. Wan	Simultaneous removal of arsenic, cadmium, and lead from soil by iron-modified magnetic biochar	2020
6904	M. H. Dalsaniya	Electron density modulation of a metallic GeSb monolayer by pnictogen doping for excellent hydrogen evolution	2020
6905	H. C. Serrano	Mapping Exposure to Multi-Pollutants Using Environmental Biomonitors-A Multi-Exposure Index	2017
6906	L. R. Zein	Organelle-specific mechanisms of drug-induced autophagy-dependent cell death	2021
6907	W. Ding	A novel removal strategy for copper and arsenic by photooxidation coupled with coprecipitation: Performance and mechanism	2020
6908	J. Alvarez-Quintana	Arsenic in Soils Affected by Mining: Microscopic Studies vs. Sequential Chemical Extraction	2020
6909	H. W. Jeon	Effective Removal of Arsenic in Drinking Water Using Facile Synthesized Fe2O3 Coated N-Doped TiO2 Nanoparticles	2020
6910	M. C. Hughey	Short-Term Exposure to Coal Combustion Waste Has Little Impact on the Skin Microbiome of Adult Spring Peepers (Pseudacris crucifer)	2016

6911	T. J. Sorg	Removing co-occurring contaminants of arsenic and vanadium with full-scale arsenic adsorptive media systems	2021
6912	S. S. Wang	The Herbal Constituents in An-Gong-Niu-Huang Wan (AGNH) Protect against Cinnabar- and Realgar-Induced Hepatorenal Toxicity and Accumulations of Mercury and Arsenic in Mice	2021
6913	S. Y. Fung	Nutrient and Chemical Analysis of Fruiting Bodies of a Cultivar of the Chinese Caterpillar Mushroom, Ophiocordyceps sinensis (Ascomycetes)	2018
6914	N. Seco-Reigosa	Mixtures including wastes from the mussel shell processing industry: retention of arsenic, chromium and mercury	2014
6915	S. A. Chaudhry	Fe(III)-Sn(IV) mixed binary oxide-coated sand preparation and its use for the removal of As(III) and As(V) from water: Application of isotherm, kinetic and thermodynamics	2016
6916	S. Liu	Influence of the digestive process on intestinal toxicity of polystyrene microplastics as determined by in vitro Caco-2 models	2020
6917	N. Sharma	Molecular dissemination of emerging antibiotic, biocide, and metal co-resistomes in the Himalayan hot springs	2022
6918	K. Y. Liu	Fabrication of amino-modified electrospun nanofibrous cellulose membrane and adsorption for typical organoarsenic contaminants: Behavior and mechanism	2020
6919	M. Yoshinaga	A comprehensive study including monitoring, assessment of health effects and development of a remediation method for chromium pollution	2018
6920	A. A. Ahribesh	Influence of the synthesis parameters on the properties of the sepiolite-based magnetic adsorbents	2017
6921	D. Ocinski	Freeze-drying as the post-processing technique improving adsorptive properties of waste Fe/Mn oxides entrapped in polymer beads towards As(III) and As(V)	2020
6922	X. R. Wang	Effect mechanism of arsenic on the growth of ultrafine tungsten carbide powder	2018
6923	J. Chen	Facile fabrication of nanostructured cerium-manganese binary oxide for enhanced arsenite removal from water	2018
6924	S. Pirgalioglu	Crosslinked polyDADMAC gels as highly selective and reusable arsenate binding materials	2015
6925	A. Azaroff	Priority and emerging micropollutants distribution from coastal to continental slope sediments: A case study of Capbreton Submarine Canyon (North Atlantic Ocean)	2020
6926	G. Kalyvas	A critical assessment on arsenic partitioning in mine-affected soils by using two sequential extraction protocols	2018
6927	D. W. Tam	Stabilization of s-wave superconductivity through arsenic p-orbital hybridization in electron-doped BaFe2As2	2018
6928	J. R. Gallego	Insights into a 20-ha multi-contaminated brownfield megasite: An environmental forensics approach	2016

6929	M. Javahershenas	The effects of Lahijan landfill leachate on the quality of surface and groundwater resources	2022
6930	Y. Y. Hou	Electrochemical Performance of Graphene Oxide/Black Arsenic Phosphorus/Carbon Nanotubes as Anode Material for LIBs	2022
6931	L. Fouquat	Insights into the Arsenic Shell Decapping Mechanisms in As/GaAs Nanowires by X-ray and Electron Microscopy	2021
6932	J. Nikic	Adsorption mechanism of As(V) and As(III) on Fe-Mn binary oxides in synthetic and real water matrices	2016
6933	M. I. R. Khan	Crosstalk of plant growth regulators protects photosynthetic performance from arsenic damage by modulating defense systems in rice	2021
6934	N. T. H. Mai	Adsorption and desorption of arsenic to aquifer sediment on the Red River floodplain at Nam Du, Vietnam	2014
6935	A. Yurum	Fast deposition of porous iron oxide on activated carbon by microwave heating and arsenic (V) removal from water	2014
6936	Y. Yan	Simultaneous oxidation of As(III) and reduction of Cr(VI) by NiS-CdS@biochar through efficient oxalate activation: The key role of enhanced generation of reactive oxygen species	2022
6937	P. L. Cao	Mercapto propyltrimethoxysilane- and ferrous sulfate-modified nano-silica for immobilization of lead and cadmium as well as arsenic in heavy metal-contaminated soil	2020
6938	N. Mehta	Geochemical conditions conducive for retention of trace elements and radionuclides during shale-fluid interactions	2019
6939	P. Verlicchi	Surface Water and Groundwater Quality in South Africa and Mozambique-Analysis of the Most Critical Pollutants for Drinking Purposes and Challenges in Water Treatment Selection	2020
6940	Y. S. Zhu	RUNX2/miR-31/SATB2 pathway in nickel-induced BEAS-2B cell transformation	2021
6941	R. Daenzer	Oxidation of Ferrous Sulfate Hydrolyzed Slurry-Kinetic Aspects and Impact on As(V) Removal	2015
6942	B. Moe	Comparative cytotoxicity of fourteen trivalent and pentavalent arsenic species determined using real-time cell sensing	2016
6943	C. Tiberg	Immobilization of Cu and As in two contaminated soils with zero-valent iron - Long-term performance and mechanisms	2016
6944	S. Deng	Rapid and effective preparation of a HPEI modified biosorbent based on cellulose fiber with a microwave irradiation method for enhanced arsenic removal in water	2016
6945	M. Gonzalez-Moscoso	Nitric oxide modified growth, nutrient uptake and the antioxidant defense system in tomato seedlings stressed with arsenic	2021
6946	Y. Wang	Zinc exerts its renal protection effect on arsenic-exposed common carp: A signaling network comprising Nrf2, NF-kappa B and MAPK pathways	2020

6947	N. S. Dong	Co-exposure to Arsenic-Fluoride Results in Endoplasmic Reticulum Stress-Induced Apoptosis Through the PERK Signaling Pathway in the Liver of Offspring Rats	2020
6948	C. Biancacci	Annual compositional variation in wild Osmundea pinnatifida (Hudson) Stackhouse from the west coast of Scotland	2022
6949	M. Ortuzar	Micromonospora metallophores: A plant growth promotion trait useful for bacterial-assisted phytoremediation?	2020
6950	H. Wasito	Immobilized bacterial biosensor for rapid and effective monitoring of acute toxicity in water	2019
6951	I. Andelkovic	Investigation of mechanism and critical parameters for removal of arsenic from water using Zr-TiO2 composite	2017
6952	M. Szlachta	Adsorptive Removal of Arsenic Species from Aqueous Solutions Using Granular Ferric Hydroxide	2016
6953	X. J. Gong	Arsenic adsorption by innovative iron/calcium in-situ-impregnated mesoporous activated carbons from low-temperature water and effects of the presence of humic acids	2020
6954	J. Gerding	Metal exposure of workers during recycling of electronic waste: a cross-sectional study in sheltered workshops in Germany	2021
6955	Z. Y. Fang	Enhanced water decontamination from methylated arsenic by utilizing ultra-small hydrated zirconium oxides encapsulated inside gel-type anion exchanger	2022
6956	H. Bidi	Iron oxide nanoparticles alleviate arsenic phytotoxicity in rice by improving iron uptake, oxidative stress tolerance and diminishing arsenic accumulation	2021
6957	K. L. Muedi	Effective removal of arsenate from wastewater using aluminium enriched ferric oxide-hydroxide recovered from authentic acid mine drainage	2021
6958	A. M. Khair	Fluoride and Arsenic Hydrogeochemistry of Groundwater at Yuncheng Basin, Northern China	2014
6959	P. V. Sierra-Trejo	Arsenic Sorption on Chitosan-Based Sorbents: Comparison of the Effect of Molybdate and Tungstate Loading on As(V) Sorption Properties	2020
6960	N. Z. Akha	Removal of arsenic by metal organic framework/chitosan/carbon nanocomposites: Modeling, optimization, and adsorption studies	2022
6961	B. Chen	Facile Hydrothermal Synthesis of Nanostructured Hollow Iron-Cerium Alkoxides and Their Superior Arsenic Adsorption Performance	2014
6962	Y. Cheng	Arsenite removal from groundwater by iron-manganese oxides filter media: Behavior and mechanism	2019
6963	A. Kana	Simultaneous determination of oxo- and thio-arsenic species using HPLC-ICP-MS	2020
6964	A. K. Maurya	Development of artificial neural networks software for arsenic adsorption from an aqueous environment	2022

6965	X. L. Lin	The toxicity of exogenous arsenic to soil-dwelling springtail Folsomia candida in relation to soil properties and aging time	2019
6966	M. Kremplova	Influence of Oxidation Stage and Exfoliation Extent of Carbon-Based Materials on Electrochemical Detection of As(III)	2016
6967	P. J. Tsai	Source and health risk apportionment for PM2.5 collected in Sha-Lu area, Taiwan	2020
6968	C. Longhi	Extraintestinal Pathogenic Escherichia coli: Beta-Lactam Antibiotic and Heavy Metal Resistance	2022
6969	M. H. G. Berntssen	Processing Mixed Mesopelagic Biomass from the North-East Atlantic into Aquafeed Resources; Implication for Food Safety	2021
6970	R. M. Dhoble	Development of bark-based magnetic iron oxide particle (BMIOP), a bio-adsorbent for removal of arsenic (III) from water	2018
6971	W. B. Xin	Effect of Cerium on Characteristic of Inclusions and Grain Boundary Segregation of Arsenic in Iron Melts	2015
6972	G. Mininni	Production and characteristics of sewage sludge in Italy	2019
6973	T. Agustiany	Stable iridium-modified boron-doped diamond electrode for the application in electrochemical detection of arsenic (III)	2020
6974	T. Chen	In-situ forming Sub-2 nm hydrous iron oxide particles in MOFs for deep-treatment and high anti-interference in arsenic removal	2022
6975	Q. H. Wu	Regulatory Mechanism of Copper Oxide Nanoparticles on Uptake of Different Species of Arsenic in Rice	2021
6976	P. Cao	Characterization and Gold Extraction of Gold-bearing Dust from Carbon-bearing Gold Concentrates	2022
6977	Y. Zhang	Density Functional Theory Study of Arsenic Adsorption on the Fe2O3 (001) Surface	2019
6978	V. I. Gavrilov	Reaction of 10-phenyl-5,10-dihydrophenarsazine with p-toluenesulfonyl Chloride	1999
6979	C. Waterlot	Determining the influence of the physicochemical parameters of urban soils on As availability using chemometric methods: A preliminary study	2016
6980	F. Arab	Efficiency of sophorolipids for arsenic removal from mine tailings	2020
6981	J. G. Kim	Bifunctional iron-modified graphitic carbon nitride (g-C3N4) for simultaneous oxidation and adsorption of arsenic	2020
6982	W. J. Zhu	Optimization of As(V) Removal from Contaminated Water with Mesoporous Alumina: Effects of pH, Contact Time, Concentration and Temperature	2017

6983	A. P. Singh	Nitric Oxide Alleviated Arsenic Toxicity by Modulation of Antioxidants and Thiol Metabolism in Rice (Oryza sativa L.)	2016
6984	H. Rahman	Modifying the Parboiling of Rice to Remove Inorganic Arsenic, While Fortifying with Calcium	2019
6985	J. Tapia	Origin, distribution, and geochemistry of arsenic in the Altiplano-Puna plateau of Argentina, Bolivia, Chile, and Peru	2019
6986	Y. W. Cao	Application of calcined iowaite in arsenic removal from aqueous solution	2016
6987	J. C. Lee	Oxalate-based remediation of arsenic bound to amorphous Fe and Al hydrous oxides in soil	2016
6988	C. Feng	Removal of arsenic from gold processing circuits by use of novel magnetic nanoparticles	2018
6989	E. Vences-Alvarez	New bimetallic adsorbent material based on cerium-iron nanoparticles highly selective and affine for arsenic(V)	2022
6990	C. Feng	Removal of arsenic from gold cyanidation process waters by use of cerium-based magnetic adsorbents	2018
6991	K. D. Brahman	Simultaneously removal of inorganic arsenic species from stored rainwater in arsenic endemic area by leaves of Tecomella undulata: a multivariate study	2016
6992	W. S. Hu	Deactivation mechanism of arsenic and resistance effect of SO42- on commercial catalysts for selective catalytic reduction of NOx with NH3	2016
6993	C. H. Zong	Critical review of bio/nano sensors for arsenic detection	2021
6994	C. Mangwandi	Design, production and characterisation of granular adsorbent material for arsenic removal from contaminated wastewater	2016
6995	C. B. Tabelin	Acid mine drainage formation and arsenic mobility under strongly acidic conditions: Importance of soluble phases, iron oxyhydroxides/oxides and nature of oxidation layer on pyrite	2020
6996	B. K. Mahata	Arsenic adsorption on rusting iron nails	2018
6997	A. Robledo-Peralta	Arsenic and Fluoride in Groundwater, Prevalence and Alternative Removal Approach	2021
6998	J. Tucek	Zero-Valent Iron Nanoparticles Reduce Arsenites and Arsenates to As(0) Firmly Embedded in Core-Shell Superstructure: Challenging Strategy of Arsenic Treatment under Anoxic Conditions	2017
6999	D. D. Zhao	Zirconium/PVA modified flat-sheet PVDF membrane as a cost-effective adsorptive and filtration material: A case study on decontamination of organic arsenic in aqueous solutions	2016
7000	Y. D. Huang	A deep insight into arsenic adsorption over gamma-Al2O3 in the presence of SO2/NO	2019

7001	N. Rahman	Development of polyacrylamide chromium oxide as a new sorbent for solid phase extraction of As(III) from food and environmental water samples	2015
7002	A. C. Canali	Solubility of platinum-arsenide melt and sperrylite in synthetic basalt at 0.1 MPa and 1200 degrees C with implications for arsenic speciation and platinum sequestration in mafic igneous systems	2017
7003	A. Ghosh	Fixed-bed column performance of Mn-incorporated iron(III) oxide nanoparticle agglomerates on As(III) removal from the spiked groundwater in lab bench scale	2014
7004	V. P. Isupov	Synthesis of Arsenic Sorbent by the Reaction of Magnesium Hydroxide with Aqueous Iron(III) Chloride Solution	2017
7005	Y. H. Bai	The role of biogenic Fe-Mn oxides formed in situ for arsenic oxidation and adsorption in aquatic ecosystems	2016
7006	X. Y. Zhang	Stability study of the As(V)-Fe(III) oxyhydroxide coprecipitate over a broad pH range: Characteristics and mechanism	2022
7007	D. E. Ortega	Tailoring the arsenic(III) removal ability from water using metal-organic frameworks via metal exchange-A computational study	2022
7008	A. K. Sharma	Ameliorative role of bosentan, an endothelin receptor antagonist, against sodium arsenite-induced renal dysfunction in rats	2021
7009	S. R. Safi	Removal of Arsenic Using a Cationic Polymer Gel Impregnated with Iron Hydroxide	2019
7010	B. Wen	Coupled S and Sr isotope evidences for elevated arsenic concentrations in groundwater from the world's largest antimony mine, Central China	2018
7011	N. Khalid	Adsorption of arsenic from aqueous media using lateritic minerals: Equilibrium, kinetic and thermodynamic studies	2014
7012	S. Saha	Arsenic mitigation by chitosan-based porous magnesia-impregnated alumina: performance evaluation in continuous packed bed column	2016
7013	K. Pillai	Modeling Transport and Adsorption of Arsenic Ions in Iron-Oxide Laden Porous Media. Part I: Theoretical Developments	2021
7014	L. T. Gonul	Chemical speciation and ecological risk assessment of arsenic in marine sediments from Izmir Bay (Eastern Aegean Sea)	2015
7015	B. Yan	Superior removal of As(III) and As(V) from water with Mn-doped beta-FeOOH nanospindles on carbon foam	2021
7016	R. A. Pepper	A novel akaganeite sorbent synthesised from waste red mud: Application for treatment of arsenate in aqueous solutions	2018
7017	J. Y. Liu	Arsenic Partitioning Behavior During Sludge Co-combustion: Thermodynamic Equilibrium Simulation	2019
7018	N. Y. Yin	In vitro study of soil arsenic release by human gut microbiota and its intestinal absorption by Caco-2 cells	2017

7019	X. L. Song	A novel cactus-like Fe3O4/Halloysite nanocomposite for arsenite and arsenate removal from water	2019
7020	A. S. Gugushe	Application of Response Surface Methodology and Desirability Function in the Optimization of Adsorptive Remediation of Arsenic from Acid Mine Drainage Using Magnetic Nanocomposite: Equilibrium Studies and Application to Real Samples	2019
7021	N. A. Valisheva	Passivation Mechanism of the Native Oxide/InAs Interface by Fluorine	2017
7022	K. Gupta	Recent advances in adsorptive removal of heavy metal and metalloid ions by metal oxide-based nanomaterials	2021
7023	X. L. Zhang	Iron oxide nanoparticles confined in mesoporous silicates for arsenic sequestration: effect of the host pore structure	2017
7024	N. Jain	Arsenic adsorbent derived from the ferromanganese slag	2021
7025	J. Baruah	Modelling and optimization of factors influencing adsorptive performance of agrowaste-derived Nanocellulose Iron Oxide Nanobiocomposites during remediation of Arsenic contaminated groundwater	2020
7026	H. Y. Zhan	Determination of adsorption characteristics of metal oxide nanomaterials: application as adsorbents	2014
7027	L. A. Richards	High resolution profile of inorganic aqueous geochemistry and key redox zones in an arsenic bearing aquifer in Cambodia	2017
7028	A. Pranudta	Immobilization of arsenic in wastewater from regeneration of fixed-bed adsorbent by co-precipitation with zirconium nano-sludge for disposal in landfills	2022
7029	T. A. Ngantcha-Kwimi	As(V) and PO4 Removal by an Iron-Impregnated Activated Carbon in a Single and Binary Adsorbate System: Experimental and Surface Complexation Modeling Results	2016
7030	X. Li	Mechanism of arsenic poisoning on SCR catalyst of CeW/Ti and its novel efficient regeneration method with hydrogen	2016
7031	E. C. Gillispie	Chemical variability of sediment and groundwater in a Pleistocene aquifer of Cambodia: Implications for arsenic pollution potential	2019
7032	S. Sahoo	Gold Nano Particle and Reduced Graphene Oxide Composite Modified Carbon Paste Electrode for the Ultra Trace Detection of Arsenic (III)	2017
7033	A. K. SenGupta	Transforming a Global Water Crisis into an Economic Opportunity: Unmet Needs and Lessons Learned during the Last Two Decades	2021
7034	R. Prabhakar	Use of adsorption-influencing parameters for designing the batch adsorber and neural network-based prediction modelling for the aqueous arsenate removal using combustion synthesised nano-alumina	2020
7035	N. J. Liu	Arsenic, Iron and Organic Matter in Quaternary Aquifer Sediments from Western Hetao Basin, Inner Mongolia	2017
7036	J. Park	Effect of neutralizing agents on the type of As co-precipitates formed by in situ Fe oxides synthesis and its impact on the bioaccessibility of As in soil	2020

7037	S. Suwannatrai	Oxidation-adsorption of arsenite contaminated water over ceria nanorods	2020
7038	I. E. Tyschenko	Diffusion of In Atoms in SiO2 Films Implanted with As+ Ions	2021
7039	L. Q. Guo	Three-dimensional Fe3O4-graphene macroscopic composites for arsenic and arsenate removal	2015
7040	U. K. Sahu	Preparation and characterization of mesoporous cerium oxide for toxic as(v) removal: performance and mechanistic studies	2022
7041	S. Chakraborty	Rapid assessment of regional soil arsenic pollution risk via diffuse reflectance spectroscopy	2017
7042	Y. K. Penke	Arsenic remediation onto redox and photo-catalytic/electrocatalytic Mn-Al-Fe impregnated rGO: Sustainable aspects of sludge as supercapacitor	2020
7043	Y. J. Lee	Characteristics of In0.7Ga0.3As MOS Capacitors with Sulfur and Hydrazine Pretreatments	2021
7044	V. Kumari	Mesoporous ZnAl2O4: an efficient adsorbent for the removal of arsenic from contaminated water	2015
7045	A. M. Nasir	Highly adsorptive polysulfone/hydrous iron-nickel-manganese (PSF/HINM) nanocomposite hollow fiber membrane for synergistic arsenic removal	2019
7046	T. Ahmed	Synthesis and analysis of cellulose based layered double hydroxide nanocomposite adsorbent for arsenic removal from water	2022
7047	N. Y. Yin	The role of soil arsenic fractionation in the bioaccessibility, transformation, and fate of arsenic in the presence of human gut microbiota	2021
7048	M. Massoudinejad	A comprehensive study (kinetic, thermodynamic and equilibrium) of arsenic (V) adsorption using KMnO4 modified clinoptilolite	2015
7049	A. Suda	Functional effects of manganese and iron oxides on the dynamics of trace elements in soils with a special focus on arsenic and cadmium: A review	2016
7050	M. Z. Xue	Nitric oxide signaling is involved in arsenic-induced guard cell death in Vicia faba L. (Fabaceae)	2017
7051	S. Sawan	The Use of Voltammetry for Sorption Studies of Arsenic (III) Ions by Magnetic Beads Functionalized with Nucleobase Hydrazide Derivatives	2021
7052	P. R. Agrawal	Rapid adsorption of arsenate from water on a novel hybrid of zirconia oxide anchored rGO functionalised carbon foam	2021
7053	S. Lilhare	Calcium Alginate Beads with Entrapped Iron Oxide Magnetic Nanoparticles Functionalized with Methionine-A Versatile Adsorbent for Arsenic Removal	2021
7054	D. Malwal	Rapid and efficient removal of arsenic from water using electrospun CuO-ZnO composite nanofibers	2016

7055	M. A. Acheampong	Treatment of gold mining effluent in pilot fixed bed sorption system	2014
7056	M. K. Lee	Field-scale bioremediation of arsenic-contaminated groundwater using sulfate-reducing bacteria and biogenic pyrite	2019
7057	P. Intamo	Metals and arsenic concentrations of Ultisols adjacent to mine sites on limestone in Western Thailand	2016
7058	N. K. Asmel	High concentration arsenic removal from aqueous solution using nano-iron ion enrich material (NIIEM) super adsorbent	2017
7059	J. Im	Role of phosphate and Fe-oxides on the acid-aided extraction efficiency and readsorption of As in field-aged soil	2015
7060	J. Rowley	Synthesis and characterization of polyethersulfone membranes impregnated with (3-aminopropyltriethoxysilane) APTES-Fe3O4 nanoparticles for As(V) removal from water	2019
7061	J. W. Stuckey	Peat formation concentrates arsenic within sediment deposits of the Mekong Delta	2015
7062	A. Wojciechowska	Synthesis and Characterization of Magnetic Nanomaterials with Adsorptive Properties of Arsenic Ions	2020
7063	X. L. Song	Halloysite nanotubes stabilized polyurethane foam carbon coupled with iron oxide for high-efficient and fast treatment of arsenic(III/V) wastewater	2021
7064	M. K. Chini	Carbon-Based Adsorbents from Naturally Available Bermuda Grasses: Removal of TDS and Arsenic Ions	2020
7065	J. Ma	Fractions and colloidal distribution of arsenic associated with iron oxide minerals in lead-zinc mine-contaminated soils: Comparison of tailings and smelter pollution	2019
7066	Z. Q. Lao	Catalytic Wittig and aza-Wittig reactions	2016
7067	Z. J. Zhang	A facile route to core-shell nanoparticulate formation of arsenic trioxide for effective solid tumor treatment	2016
7068	M. L. Pantoja	The removal of arsenate from water using iron-modified diatomite (D-Fe): isotherm and column experiments	2014
7069	D. P. Webster	An arsenic-specific biosensor with genetically engineered Shewanella oneidensis in a bioelectrochemical system	2014
7070	W. Xiu	Arsenic Removal and Transformation by Pseudomonas sp Strain GE-1-Induced Ferrihydrite: Co-precipitation Versus Adsorption	2015
7071	S. Saleh	Photooxidation/adsorption of arsenic (III) in aqueous solution over bentonite/chitosan/TiO2 heterostructured catalyst	2021
7072	W. Ding	Photooxidation of arsenic(III) in the presence of fulvic acid	2016

7073	Z. Zhou	Sorption performance and mechanisms of arsenic(V) removal by magnetic gelatin-modified biochar	2017
7074	J. J. Wang	Multi-functionalization of magnetic graphene by surface-initiated ICAR ATRP mediated by polydopamine chemistry for adsorption and speciation of arsenic	2019
7075	B. L. Huhmann	Changes in arsenic exposure in Araihazar, Bangladesh from 2001 through 2015 following a blanket well testing and education campaign	2019
7076	G. Pratesi	An insight into the inverse transformation of realgar altered by light	2015
7077	K. Yu	Anthropogenic influences on dissolved organic matter transport in high arsenic groundwater: Insights from stable carbon isotope analysis and electrospray ionization Fourier transform ion cyclotron resonance mass spectrometry	2020
7078	H. X. Chen	Arsenic and cadmium removal from water by a calcium-modified and starch-stabilized ferromanganese binary oxide	2020
7079	L. Y. Lin	Migration and arsenic adsorption study of starch-modified Fe-Ce oxide on a silicon-based micromodel observation platform	2017
7080	J. A. Ramos-Guivar	Differentiating Nanomaghemite and Nanomagnetite and Discussing Their Importance in Arsenic and Lead Removal from Contaminated Effluents: A Critical Review	2021
7081	A. Barron	In situ arsenic immobilisation for coastal aquifers using stimulated iron cycling: Lab-based viability assessment	2022
7082	J. M. Hua	Synthesis and characterization of gold nanoparticles (AuNPs) and ZnO decorated zirconia as a potential adsorbent for enhanced arsenic removal from aqueous solution	2021
7083	X. X. Zhou	Peroxynitrite contributes to arsenic-induced PARP-1 inhibition through ROS/RNS generation	2019
7084	X. M. Ma	Simultaneous reduction of arsenic (As) and cadmium (Cd) accumulation in rice by zinc oxide nanoparticles	2020
7085	S. R. Safi	The effect of gamma-FeOOH on enhancing arsenic adsorption from groundwater with DMAPAAQ plus FeOOH gel composite	2019
7086	G. Neeraj	Adsorptive potential of dispersible chitosan coated iron-oxide nanocomposites toward the elimination of arsenic from aqueous solution	2016
7087	Y. Kobayashi	Distribution and Excretion of Arsenic Metabolites after Oral Administration of Seafood-Related Organoarsenicals in Rats	2016
7088	S. Gazzari	Interaction of trivalent arsenic on different topologies of Fe-doped graphene nanosheets at water environments: A computational study	2019
7089	H. B. Zhang	Direct separation of arsenic and antimony oxides by high-temperature filtration with porous FeAl intermetallic	2017
7090	X. Li	Comparison of the Structures and Mechanism of Arsenic Deactivation of CeO2-MoO3 and CeO2-WO3 SCR Catalysts	2016

7091	T. M. S. Attia	Synthesised magnetic nanoparticles coated zeolite (MNCZ) for the removal of arsenic (As) from aqueous solution	2014
7092	A. Sundman	Reactivity of Fe from a natural stream water towards As(V)	2015
7093	M. Ogun	Oleuropein ameliorates arsenic induced oxidative stress in mice	2016
7094	T. C. Prathna	Synthesis, characterization and performance of iron oxide/alumina-based nanoadsorbents for simultaneous arsenic and fluoride removal	2018
7095	Z. P. Wen	Nanocasted synthesis of magnetic mesoporous iron cerium bimetal oxides (MMIC) as an efficient heterogeneous Fenton-like catalyst for oxidation of arsenite	2015
7096	S. Ploychompoo	Fabrication of Zn-MOF-74/polyacrylamide coated with reduced graphene oxide (Zn-MOF-74/rGO/PAM) for As(III) removal	2021
7097	Z. Souri	Enhanced Phytoextraction by As Hyperaccumulator Isatis cappadocica Spiked with Sodium Nitroprusside	2017
7098	R. Gubler	Ferrous iron enhances arsenic sorption and oxidation by non-stoichiometric magnetite and maghemite	2021
7099	Y. Bai	Application of iron-based materials for removal of antimony and arsenic from water: Sorption properties and mechanism insights	2022
7100	Y. J. Yang	The amino - functionalized magnetic graphene oxide combined with graphite furnace atomic absorption spectrometry for determination of trace inorganic arsenic species in water samples	2021
7101	A. R. Liu	Nanoencapsulation of arsenate with nanoscale zero-valent iron (nZVI): A 3D perspective	2018
7102	J. L. Cui	Partitioning and (im)mobilization of arsenic associated with iron in arsenic-bearing deep subsoil profiles from Hong Kong*	2022
7103	D. Baragano	Arsenic release from pyrite ash waste over an active hydrogeological system and its effects on water quality	2020
7104	A. E. Fryar	Variability in groundwater flow and chemistry in the Mekong River alluvial aquifer (Thailand): implications for arsenic and manganese occurrence	2021
7105	Y. X. Wang	Removal of As(III) and As(V) by ferric salts coagulation - Implications of particle size and zeta potential of precipitates	2014
7106	J. L. Hu	Adsorption characteristic of As(III) on goethite waste generated from hydrometallurgy of zinc	2017
7107	X. X. Wang	Impact of Three Copper Amendments on Arsenic Accumulation and Speciation in Rice (Oryza sativa L.) in a Life Cycle Study	2022
7108	S. Dudek	Arsenic(V) removal on the lanthanum-modified ion exchanger with quaternary ammonium groups based on iron oxide	2022

7109	N. Guenifi	Boron phosphorus and arsenic diffusion in MOS transistors: Simulation and analysis in 2D and 3D	2018
7110	G. Garcia-Rosales	Carbon biogenic with iron nanoparticles for removal of As(V) from water	2019
7111	W. Zhang	Arsenic Bioaccumulation and Biotransformation in Clams (Asaphis violascens) Exposed to Inorganic Arsenic: Effects of Species and Concentrations	2019
7112	X. Yang	The Activation of Heat-Shock Protein After Copper(II) and/or Arsenic(III)-Induced Imbalance of Homeostasis, Inflammatory Response in Chicken Rectum	2020
7113	D. E. Pratama	Quick-fix agarose beads impregnated with hydrous ferric oxide for As(III) species removal from pharmaceutical wastewater	2022
7114	P. I. Premovic	Cretaceous-Paleogene Boundary Clays from Spain and New Zealand: Arsenic Anomalies	2015
7115	M. Aide	Soil Profile Arsenic Concentration Distributions in Missouri Soils Having Cambic and Argillic Soil Horizons	2014
7116	Y. Zhang	Arsenic in soils contaminated by arsenic-containing chemical weapons in a site of Jilin, China: fraction and bioaccessibility	2022
7117	S. Matsumoto	Inhibition of arsenic accumulation in Japanese rice by the application of iron and silicate materials	2015
7118	I. Corrales-Mendoza	Growth of NdFeAsO Films by a Combination of Metal-Organic Chemical Vapor Deposition and Arsenic Diffusion Processes	2014
7119	J. Zhu	The Chemical Oxidation and Immobilization of Arsenic and Antimony in Simulated AMD in Karst Areas	2022
7120	Z. L. Qi	Adsorption combined with superconducting high gradient magnetic separation technique used for removal of arsenic and antimony	2018
7121	M. Kumar	Hydrophilic nano-aluminum oxide containing polyphenylsulfone hollow fiber membranes for the extraction of arsenic (As-V) from drinking water	2021
7122	H. Sharifan	Foliar Application of Zn Agrichemicals Affects the Bioavailability of Arsenic, Cadmium and Micronutrients to Rice (Oryza sativa L.) in Flooded Paddy Soil	2021
7123	R. Razavi	Eco-friendly synthesis by Rosemary extract and characterization of Fe3O4@SiO2 magnetic nanocomposite as a potential adsorbent for enhanced arsenic removal from aqueous solution: isotherm and kinetic studies	
7124	H. Neidhardt	Phosphate immobilisation dynamics and interaction with arsenic sorption at redox transition zones in floodplain aquifers: Insights from the Red River Delta, Vietnam	2021
7125	I. Andjelkovic	Investigation of sorbents synthesised by mechanical-chemical reaction for sorption of As(III) and As(V) from aqueous medium	2014
7126	M. K. Ke	Interface-Promoted Direct Oxidation of p-Arsanilic Acid and Removal of Total Arsenic by the Coupling of Peroxymonosulfate and Mn-Fe-Mixed Oxide	2021

7127	A. Pramanik	Cytotoxicity Assessment of Heavy Metal Arsenic (Arsenic Trioxide) Using Nigella sativa L. (Black Cumin) as Test System	2019
7128	M. D. Li	delta S-34 and delta O-18 of dissolved sulfate as biotic tracer of biogeochemical influences on arsenic mobilization in groundwater in the Hetao Plain, Inner Mongolia, China	2014
7129	S. G. Liu	Kinetic and mechanistic aspects of selenite oxidation by chlorine, bromine, monochloramine, ozone, permanganate, and hydrogen peroxide	2019
7130	X. W. Liu	Fe-Mn-Ce oxide-modified biochar composites as efficient adsorbents for removing As(III) from water: adsorption performance and mechanisms	2019
7131	M. E. Talukder	Chitosan-functionalized sodium alginate-based electrospun nanofiber membrane for As (III) removal from aqueous solution	2021
7132	S. Barakan	FeAl12-polyoxocations intercalated nano-bentonite fabrication in concentrated suspension using one-step ultrasonic-microwave irradiation for arsenic removal from alkaline wastewater	2020
7133	P. Gogoi	Nature of sorption of trivalent arsenic on novel iron oxyhydroxide stabilized starch/OMMT composite: A mechanistic approach	2021
7134	J. Mertens	Arsenate uptake by Al nanoclusters and other Al-based sorbents during water treatment	2016
7135	P. Cao	Separation and recovery of iron and arsenic from acid leaching wastewater by valence state transformation	2021
7136	K. K. Jinadasa	New adsorbents based on imprinted polymers and composite nanomaterials for arsenic and mercury screening/speciation: A review	2020
7137	A. U. Khan	Scaled-up development of recyclable Pd@ZnO/CuO nanostructure for efficient removal of arsenic from wastewater	2022
7138	N. Pous	Anaerobic arsenite oxidation with an electrode serving as the sole electron acceptor: A novel approach to the bioremediation of arsenic-polluted groundwater	2015
7139	F. H. Li	Adsorption of As(V) on aluminum-, iron-, and manganese-(oxyhydr)oxides: equilibrium and kinetics	2015
7140	X. X. Zhou	S-nitrosation on zinc finger motif of PARP-1 as a mechanism of DNA repair inhibition by arsenite	2016
7141	Y. Yoshino	Enhanced cytotoxic effects of arsenite in combination with anthocyanidin compound, delphinidin, against a human leukemia cell line, HL-60	2018
7142	S. Zecchin	Exposure to different arsenic species drives the establishment of iron- and sulfur-oxidizing bacteria on rice root iron plaques	2019
7143	K. Kordas	Iron and Zinc Supplementation Does Not Impact Urinary Arsenic Excretion in Mexican School Children	2017
7144	Y. Matano	Diverse structures and remarkable oxidizing ability of triarylbismuthane oxides. Comparative study on the structure and reactivity of a series of triarylpnictogen oxides	2004

7145	P. Sharma	Phytoremediation potential of heavy metal accumulator plants for waste management in the pulp and paper industry	2020
7146	N. H. Yu	(-)-Epigallocatechin-3-Gallate Inhibits Arsenic-Induced Inflammation and Apoptosis through Suppression of Oxidative Stress in Mice	2017
7147	A. S. C. Chen	Regeneration of iron-based adsorptive media used for removing arsenic from groundwater	2015
7148	N. Wang	Biochar increases arsenic release from an anaerobic paddy soil due to enhanced microbial reduction of iron and arsenic	2017
7149	G. Sappa	Geochemical modeling and multivariate statistical evaluation of trace elements in arsenic contaminated groundwater systems of Viterbo Area, (Central Italy)	2014
7150	J. Nikic	Arsenic Removal from Water by Green Synthesized Magnetic Nanoparticles	2019
7151	I. Jacukowicz-Sobala	Hybrid polymer containing ferric oxides obtained using a redox polymer Part I. Synthesis and characterization	2014
7152	J. A. Luque-Espinar	Multiscale analysis of the spatial variability of heavy metals and organic matter in soils and groundwater across Spain	2018
7153	M. L. Li	Algae-based sorbents for removal of gallium from semiconductor manufacturing wastewater	2018
7154	J. H. Wu	Arsenic Removal from Cu-As-Containing Filter Cakes by Na2CO3 Leaching	2019
7155	A. Maghsodi	In-situ chemical deposition as a new method for the preparation of Fe3O4 nanopaeticles embedded on anodic aluminum oxide membrane (Fe3O4@AAO): Characterization and application for arsenic removal using response surface methodology	2019
7156	A. D'Errico	Exposure to occupational hazards and risk of sinonasal epithelial cancer: results from an extended Italian case-control study	2021
7157	E. Hiller	Geochemistry of Mine Tailings from Processing of Siderite-Cu Ores and Mobility of Selected Metals and Metalloids Evaluated by a Pot Leaching Experiment at the Slovinky Impoundment, Eastern Slovakia	2016
7158	Q. Y. Luo	Comparison and characterization of polyacrylonitrile, polyvinylidene fluoride, and polyvinyl chloride composites functionalized with ferric hydroxide for removing arsenic from water	2021
7159	A. M. Garcia-Serna	Prenatal and Perinatal Environmental Influences Shaping the Neonatal Immune System: A Focus on Asthma and Allergy Origins	2021
7160	P. L. Shao	Remediation of Cu and As contaminated water and soil utilizing biochar supported layered double hydroxide: Mechanisms and soil environment altering	2023
7161	Y. Lee	Rare earth real wastewater treatment by pilot scale using new concept continuous treatment process	2021
7162	S. Nizet	Clinoptilolite in Dextran Sulphate Sodium-Induced Murine Colitis: Efficacy and Safety of a Microparticulate Preparation	2018

7163	J. Nikic	Application of Nanomaterials in Water Treatment: Arsenic and Natural Organic Matter Removal	2021
7164	V. B. Centurion	Unveiling resistome profiles in the sediments of an Antarctic volcanic island	2019
7165	N. Seco-Reigosa	Adsorption, desorption and fractionation of As(V) on untreated and mussel shell-treated granitic material	2015
7166	R. Shahzad	Bacillus amyloliquefaciens RWL-1 as a New Potential Strain for Augmenting Biochemical and Nutritional Composition of Fermented Soybean	2020
7167	J. D. Martin-Ramos	Non-destructive pigment characterization in the painting Little Madonna of Foligno by X-ray Powder Diffraction	2017
7168	Y. Lee	Arsenic adsorption study in acid mine drainage using fixed bed column by novel beaded adsorbent	2022
7169	H. T. Lu	Fenton-Like Catalysis and Oxidation/Adsorption Performances of Acetaminophen and Arsenic Pollutants in Water on a Multimetal Cu-Zn Fe-LDH	2016
7170	A. Darma	Significance of Shewanella Species for the Phytoavailability and Toxicity of Arsenic-A Review	2022
7171	M. B. Gumpu	Electrocatalytic nanocauliflower structured fluorine doped CdO thin film as a potential arsenic sensor	2016
7172	B. Liu	Unraveling ecological risk of As/Sb and other metal(loid)s and fungal community responses in As/Sb smelting-intensive zone: A typical case study of Southwest China	2022
7173	S. Cortes	A Positive Relationship between Exposure to Heavy Metals and Development of Chronic Diseases: A Case Study from Chile	2021
7174	S. Martinez-Vargas	As(III) and As(V) adsorption on manganese ferrite nanoparticles	2018
7175	S. Gunatilake	Glyphosate's Synergistic Toxicity in Combination with Other Factors as a Cause of Chronic Kidney Disease of Unknown Origin	2019
7176	B. Gholamine	Gallic acid ameliorates sodium arsenite-induced renal and hepatic toxicity in rats	2021
7177	A. Pratush	Adverse effect of heavy metals (As, Pb, Hg, and Cr) on health and their bioremediation strategies: a review	2018
7178	D. T. D. Qadah	Determination of Aromatic Arsines in Environmental Solids by Direct Thermal Desorption Gas Chromatography	2018
7179	L. Bindi	A crystallographic excursion in the extraordinary world of minerals: the case of Cu- and Ag-rich sulfosalts	2018
7180	P. He	Influence of arsanilic acid, Cu2+, PO43- and their interaction on anaerobic digestion of pig manure	2018

7181	B. Casentini	Arsenate and arsenite removal from contaminated water by iron oxides nanoparticles formed inside a bacterial exopolysaccharide	2019
7182	Y. H. Li	Kinetics and thermodynamics of adsorption for arsenate ions on the hierarchical porous adsorbent of alpha-Fe2O3/Fe3O4/C with bamboo bio-template	2017
7183	Z. H. Wang	A small molecular compound CC1007 induces cross-lineage differentiation by inhibiting HDAC7 expression and HDAC7/MEF2C interaction in BCR-ABL1(-) pre-B-ALL	2020
7184	W. L. Ji	Hydrophobic Ce-doped beta-PbO2-SDS anode achieving synergistic effects for enhanced electrocatalytic oxidation of As(III)	2022
7185	C. Chen	Facile transmetallation of Sb-III(DOTA) (-) renders it unsuitable for medical applications	2022
7186	C. Mares	Bisphenol-A and other Plastics: Review of Endocrine Disrupting Effects on Prostate Cancer	2020
7187	J. Jung	Biochemical and Biodiversity Insights into Heavy Metal Ion-Responsive Transcription Regulators for Synthetic Biological Heavy Metal Sensors	2019
7188	J. F. Obrycki	Evaluating Public and Regulatory Acceptance for Urban Soil Management Approaches	2017
7189	R. D'Adamo	The Effect of Floods on Sediment Contamination in a Microtidal Coastal Lagoon: The Lagoon of Lesina, Italy	2014
7190	K. Ahmad	Synthesis and characterization of water stable polymeric metallo organic composite (PMOC) for the removal of arsenic and lead from brackish water	2022
7191	M. Zhao	OGP46 Induces Differentiation of Acute Myeloid Leukemia Cells via Different Optimal Signaling Pathways	2021
7192	Y. C. Cheng	Biomarkers of Exposure among Adult Smokeless Tobacco Users in the Population Assessment of Tobacco and Health Study (Wave 1, 2013-2014)	2020
7193	N. Khardori	Antibiotics: From the Beginning to the Future: Part 1	2020
7194	F. Breitsameter	Acyclic, ring, and cage As,C compounds from trimethylsilyl ylides and AsCl3	2000
7195	N. A. Hodyl	Cord Blood DNA Methylation Biomarkers for Predicting Neurodevelopmental Outcomes	2016
7196	R. P. Liu	Simultaneous removal of arsenic and fluoride by freshly-prepared aluminum hydroxide	2015
7197	W. Zhang	Enhanced removal of arsenite and arsenate by a multifunctional Fe-Ti-Mn composite oxide: Photooxidation, oxidation and adsorption	2018
7198	J. D. Pleil	Estimating Common Parameters of Lognormally Distributed Environmental and Biomonitoring Data: Harmonizing Disparate Statistics From Publications	2014

7199	J. Zhang	Inhibition of angiogenesis by arsenic trioxide via TSP-1-TGF-beta 1-CTGF-VEGF functional module in rheumatoid arthritis	2017
7200	A. C. Remigio	Geochemical cycles of arsenic in historic tin tailings from multiple ore sources: an example from Australia	2021
7201	Y. J. Lin	Deciphering the effect of citric acid on arsenic adsorption with phosphorene in aqueous solution	2019
7202	J. A. Frantz	Arsenic selenide thin film degradation and its mitigation	2018
7203	T. Wang	Cu doped Fe3O4 magnetic adsorbent for arsenic: synthesis, property, and sorption application	2015
7204	A. Y. Al-Brakati	Role of thymoquinone and ebselen in the prevention of sodium arsenite-induced nephrotoxicity in female rats	2019
7205	O. Andersen	A review of pitfalls and progress in chelation treatment of metal poisonings	2016
7206	J. H. Kamstra	Zebrafish as a model to study the role of DNA methylation in environmental toxicology	2015
7207	H. Wiltsche	Matrix effects of carbon and bromine in inductively coupled plasma optical emission spectrometry	2015
7208	C. W. Wu	Arsenite-induced apoptosis can be attenuated via depletion of mTOR activity to restore autophagy	2019
7209	A. A. Okunola	Mutagenicity of automobile workshop soil leachate and tobacco industry wastewater using the Ames Salmonella fluctuation and the SOS chromotests	2016
7210	C. Protano	Polycyclic Aromatic Hydrocarbons and Metals in Transplanted Lichen (Pseudovernia furfuracea) at Sites Adjacent to a Solid-waste Landfill in Central Italy	2014
7211	H. J. Xu	The process of biotransformation can produce insect protein and promote the effective inactivation of heavy metals	2021
7212	Z. Y. Zhao	FeS2/H2O2 mediated water decontamination from p-arsanilic acid via coupling oxidation, adsorption and coagulation: Performance and mechanism	2020
7213	Z. Gersey	Therapeutic Targeting of the Notch Pathway in Glioblastoma Multiforme	2019
7214	A. Ruiz-Hernandez	Environmental chemicals and DNA methylation in adults: a systematic review of the epidemiologic evidence	2015
7215	S. Lee	Biopolymer mixture-entrapped modified graphene oxide for sustainable treatment of heavy metal contaminated real surface water	2022
7216	E. C. Fru	Arsenic and high affinity phosphate uptake gene distribution in shallow submarine hydrothermal sediments	2018

7217	K. Kinska	Speciation of metals in indigenous plants growing in post-mining areas: Dihydroxynicotianamine identified as the most abundant Cu and Zn ligand in Hypericum laricifolium	2022
7218	R. Sprocati	Integrating Process-Based Reactive Transport Modeling and Machine Learning for Electrokinetic Remediation of Contaminated Groundwater	2021
7219	X. X. Ouyang	Immobilization and release risk of arsenic associated with partitioning and reactivity of iron oxide minerals in paddy soils	2020
7220	S. Negi	Tattoo inks are toxicological risks to human health: A systematic review of its ingredients, fate inside skin, toxicity due to polycyclic aromatic hydrocarbons, primary aromatic amines, metals, and overview of regulatory frameworks	2022
7221	Y. G. Chen	Application of Modern Research Methods for the Physicochemical Characterization of Ion Exchangers	2021
7222	L. X. Cheng	Adsorption and interface reaction in direct active bonding of GaAs to GaAs using Sn-Ag-Ti solder filler	2021
7223	A. Ramalho	Characterization of a Coproduct from the Sea Cucumber Cucumaria frondosa and Its Effects on Visceral Adipocyte Size in Male Wistar Rats	2020
7224	A. Turner	Hazardous metal additives in plastics and their environmental impacts	2021
7225	M. Askeland	Comparative characterization of biochars produced at three selected pyrolysis temperatures from common woody and herbaceous waste streams	2019
7226	C. Ardau	Chemical stabilization of metals in mine wastes by transformed red mud and other iron compounds: laboratory tests	2014
7227	M. A. Ferraz	SEDIMENT TOXICITY IDENTIFICATION EVALUATION (TIE PHASES I AND II) BASED ON MICROSCALE BIOASSAYS FOR DIAGNOSING CAUSES OF TOXICITY IN COASTAL AREAS AFFECTED BY DOMESTIC SEWAGE	2017
7228	L. Khezami	KINETIC AND THERMODYNAMIC STUDIES OF TRIVALENT ARSENIC REMOVAL BY INDIUM-DOPED ZINC OXIDE NANOPOWDER	2016
7229	J. P. Liebling	An Analysis of Over-the-Counter Cannabidiol Products in the United Kingdom	2022
7230	R. Yepsen	Flotation behavior of enargite in the process of flotation using seawater	2019
7231	C. Rocco	Impact of waste-derived organic and inorganic amendments on the mobility and bioavailability of arsenic and cadmium in alkaline and acid soils	2018
7232	E. M. Stewart	A Paleoenvironmental Study Tracking Eutrophication, Mining Pollution, and Climate Change in Niven Lake, the First Sewage Lagoon of Yellowknife (Northwest Territories)	2018
7233	M. Fleischmann	Complexes of Monocationic Group 13 Elements with Pentaphospha- and Pentaarsaferrocene	2014
7234	S. A. Viczek	Origins and carriers of Sb, As, Cd, Cl, Cr, Co, Pb, Hg, and Ni in mixed solid waste - A literature-based evaluation	2020

7235	V. H. Mendez-Garcia	Si-doped AlGaAs/GaAs(631)A heterostructures grown by MBE as a function of the As-pressure	2015
7236	B. Van Aken	Environmental Contaminants in Coal Slurry Intended for Underground Injection in the State of West Virginia	2015
7237	M. O. Munoz	Arsenic and other trace elements in thermal springs and in cold waters from drinking water wells on the Bolivian Altiplano	2015
7238	T. H. Nguyen	Laterite as a low-cost adsorbent in a sustainable decentralized filtration system to remove arsenic from groundwater in Vietnam	2020
7239	Z. Peng	Arsenic-induced deterioration of the CeWAlOx catalyst for selective catalytic reduction of NO by NH3	2017
7240	R. Shang	Tight ceramic UF membrane as RO pre-treatment: The role of electrostatic interactions on phosphate rejection	2014
7241	N. Faris	The Direct Leaching of Nickel Sulfide Flotation Concentrates - A Historic and State-of-the-Art Review Part I: Piloted Processes and Commercial Operations	
7242	G. Rodriguez-Moro	Targeted and untargeted metabolomic analysis of Procambarus clarkii exposed to a "chemical cocktail" of heavy metals and diclofenac	2022
7243	M. Krasowska	Balancing Donor-Acceptor and Dispersion Effects in Heavy Main Group Element pi Interactions: Effect of Substituents on the Pnictogen...pi Arene Interaction	2019
7244	D. H. Gan	Biphasic Effect of Pirfenidone on Angiogenesis	2022
7245	S. Suleiman	Anticancer effects of an extract from a local planarian species on human acute myeloid leukemia HL-60 cells in vitro	2020
7246	S. M. F. Bessada	Pulses and food security: Dietary protein, digestibility, bioactive and functional properties	2019
7247	C. Alptekin	Observation of Excess Heavy Metal Concentrations in Water Resources to Infer Surface Water Influences on Shallow Groundwater: a Typical Example of the Porsuk River (Eskisehir-Turkey)	2016
7248	J. K. Cleal	The placental exposome, placental epigenetic adaptations and lifelong cardio-metabolic health	2022
7249	S. Mantovani	Scalable synthesis and purification of functionalized graphene nanosheets for water remediation	2021
7250	P. Iodice	Air pollution monitoring using emission inventories combined with the moss bag approach	2016
7251	W. Salama	Significance of ferruginous pisoliths and interface sampling for gold exploration in the covered terrains	2022
7252	D. Parajuli	Adsorption of ng L-1-level arsenic by ZIF-8 nanoparticles: application to the monitoring of environmental water	2018

7253	Y. N. Wang	Effect of aging on the bioavailability and fractionation of arsenic in soils derived from five parent materials in a red soil region of Southern China	2015
7254	Q. Huang	Effect of nanomaterials on arsenic volatilization and extraction from flooded soils	2018
7255	S. G. Dong	Hydro-geochemical control of high arsenic and fluoride groundwater in arid and semi-arid areas: A case study of Tumochuan Plain, China	2022
7256	M. Jiang	Highly sensitive and stable analysis of trace arsenic(III) and mercury(II) in water by Low-pulse-energy (15 mJ) laser-induced breakdown spectroscopy assisted by active controllable spark discharge and electrochemical enrichment	2020
7257	M. Buyukseke rci	Dynamic thiol/disulphide homeostasis as indicator of oxidative stress in automotive workers	2020
7258	I. Spinello	The small-molecule compound AC-73 targeting CD147 inhibits leukemic cell proliferation, induces autophagy and increases the chemotherapeutic sensitivity of acute myeloid leukemia cells	2019
7259	T. Yamaguch i	Post-depositional changes in elemental leaching from recovered soils separated from disaster waste and tsunami deposits generated by the Great East Japan Earthquake and tsunami	2019
7260	V. N. Matthaios	Sources of indoor PM2.5 gross alpha and beta activities measured in 340 homes	2021
7261	F. S. Kot	The Effect of Natural Geochemical Background on Neurological and Mental Health	2020
7262	B. L. Li	In vivo study of erysolin metabolic profile by ultra high performance liquid chromatography coupleded to Fourier transform ion cyclotron resonance mass spectrometry	2018
7263	R. F. Huang	Characterization and distribution of metal and nonmetal elements in the Alberta oil sands region of Canada	2016
7264	B. V. Kelly	Synthesis and structural characterization of tris(phenolate)amine complexes of antimony derived from kappa(4)-N(CH2ArBu2 ' O)(3) Sb	2007
7265	N. Manap	Data analysis for environmental impact of dredging	2016
7266	Y. Y. Chen	Emodin enhances ATRA-induced differentiation and induces apoptosis in acute myeloid leukemia cells	2014
7267	A. Lopez	Tuning DNA adsorption affinity and density on metal oxide and phosphate for improved arsenate detection	2017
7268	A. S. C. Chen	Removing arsenic and co-occurring contaminants from drinking water by full-scale ion exchange and point-of-use/point-of-entry reverse osmosis systems	2020
7269	M. Khaska	Tracking natural and anthropogenic origins of dissolved arsenic during surface and groundwater interaction in a post-closure mining context: Isotopic constraints	2015
7270	H. S. M. Abd-Rabboh	Cacodylate Sensors and their Application in the Determination of Amino Acid Levels in Biological Samples	2021

7271	J. L. Yin	Photocatalytic oxidation activity enhanced by iron-oxalate chelates for Fenton-like oxidation of As(III) in oxalate systems	2022
7272	X. Han	As(III) removal and speciation of Fe (Oxyhydr)oxides during simultaneous oxidation of As(III) and Fe(II)	2016
7273	G. S. Zhang	Efficient arsenic(III) removal from aqueous solution by a novel nanostructured iron-copper-manganese trimetal oxide	2020
7274	G. Zhang	Effects of four cost-effective amendments on the synchronous stabilization of As, Zn, Cu and Cd in contaminated mixture of residue and soil from an arsenic smelting site	2022
7275	C. J. Adams	Syntheses of Group 4 ansa-Trovacene Complexes and Conversion of 1 Silatrovacenophanes into Paramagnetic Metallopolymers by Ring-Opening Polymerization	2011
7276	A. A. Shah	Spermine-mediated polyamine metabolism enhances arsenic-stress tolerance in Phaseolus vulgaris by expression of zinc-finger proteins related genes and modulation of mineral nutrient homeostasis and antioxidative system	2022
7277	L. N. Lin	Arsenic removal in aqueous solution by a novel Fe-Mn modified biochar composite: Characterization and mechanism	2017
7278	T. Mushtaq	Synergistic ameliorative effect of iron oxide nanoparticles andBacillus subtilisS4 against arsenic toxicity inCucurbita moschata: polyamines, antioxidants, and physiochemical studies	2020
7279	Y. Xiong	Arsenic transformation and adsorption by iron hydroxide/manganese dioxide doped straw activated carbon	2017
7280	S. Lunge	Magnetic iron oxide (Fe3O4) nanoparticles from tea waste for arsenic removal	2014
7281	P. Tangviroon	Change in Arsenic Leaching from Silty Soil by Adding Slag Cement	2020
7282	R. DhanaRamalakshmi	Arsenic removal usingProsopis spicigera L.wood (PsLw) carbon-iron oxide composite	2020
7283	H. Dai	Graphene oxide antagonizes the toxic response to arsenic via activation of protective autophagy and suppression of the arsenic-binding protein LEC-1 in Caenorhabditis elegans	2018
7284	G. G. Zhang	Effect of Fe-Mn-Ce modified biochar composite on microbial diversity and properties of arsenic-contaminated paddy soils	2020
7285	J. Wurl	Arsenic content in groundwater from the southern part of the San Antonio-El Triunfo mining district, Baja California Sur, Mexico	2014
7286	Y. Wu	Effect of Iron(II) on Arsenic Sequestration by delta-MnO2: Desorption Studies Using Stirred-Flow Experiments and X-Ray Absorption FineStructure Spectroscopy	2015
7287	C. Liu	Highly efficient removal of As(III) by Fe-Mn-Ca composites with the synergistic effect of oxidation and adsorption	2021
7288	Y. J. Zhang	Transcriptomic Analysis Reveals Adaptive Responses of an Enterobacteriaceae Strain LSJC7 to Arsenic Exposure	2016

7289	M. Ciopec	Studies Regarding As(V) Adsorption from Underground Water by Fe-XAD8-DEHPA Impregnated Resin. Equilibrium Sorption and Fixed-Bed Column Tests	2014
7290	A. K. Sharma	Ameliorative role of inducible nitric oxide synthase inhibitors against sodium arsenite-induced renal and hepatic dysfunction in rats	
7291	E. Zahran	Modulatory role of dietary Chlorella vulgaris powder against arsenic-induced immunotoxicity and oxidative stress in Nile tilapia (Oreochromis niloticus)	2014
7292	M. M. Rahman	Reactive transport modeling of subsurface arsenic removal systems in rural Bangladesh	2015
7293	M. A. Rahman	Ecotoxicological Effects of an Arsenic Remediation Method on Three Freshwater Organisms-Lemna disperma, Chlorella sp CE-35 and Ceriodaphnia cf. dubia	2015
7294	D. M. Wang	Biochar-templated surface precipitation and inner-sphere complexation effectively removes arsenic from acid mine drainage	2021
7295	D. R. Zhang	Fe(II) bio-oxidation mediates red mud transformations to form Fe(III)/Al (hydr)oxide adsorbent for efficient As(V) removal under acidic conditions	2022
7296	M. Sadeghi	Removal of Arsenic (III) from natural contaminated water using magnetic nanocomposite: kinetics and isotherm studies	2016
7297	S. Boonkaew wan	Mechanisms of arsenic contamination associated with hydrochemical characteristics in coastal alluvial aquifers using multivariate statistical technique and hydrogeochemical modeling: a case study in Rayong province, eastern Thailand	2021
7298	C. S. Zhi	High-Arsenic Groundwater in Paleochannels of the Lower Yellow River, China: Distribution and Genesis Mechanisms	2021
7299	F. Rosario	In Vitro Hepatotoxic and Neurotoxic Effects of Titanium and Cerium Dioxide Nanoparticles, Arsenic and Mercury Co-Exposure	2022
7300	M. M. Nazir	Calcium Oxide Nanoparticles Have the Role of Alleviating Arsenic Toxicity of Barley	2022
7301	J. Zhang	Methodology for assessing thioarsenic formation potential in sulfidic landfill environments	2014
7302	M. E. Romano	Maternal body burden of cadmium and offspring size at birth	2016
7303	D. I. Fox	Combining Ferric Salt and Cactus Mucilage for Arsenic Removal from Water	2016
7304	J. H. Zhou	BiOCl0.875Br0.125/polydopamine functionalized PVDF membrane for highly efficient visible-light-driven photocatalytic degradation of roxarsone and simultaneous arsenic immobilization	2020
7305	Z. Razmara	Synthesis, Characterization and Magnetic Properties of Bi-metallic Copper Complex, as a Precursor for the Preparation of CuO Nanoparticles and Its Application for Removal of Arsenic from Water	2018
7306	V. Minzatu	Eco-materials for Arsenium and Selenium Removal from Aqueous Solutions	2019

7307	B. Jovanovic	Thioredoxin 1 is required for stress granule assembly upon arsenite-induced oxidative stress	2021
7308	T. Kaartinen	Arsenic Removal from Mine Waters with Sorption Techniques	2017
7309	E. Roy	A single solution for arsenite and arsenate removal from drinking water using cysteine@ZnS:TiO2 nanoparticle modified molecularly imprinted biofouling-resistant filtration membrane	2016
7310	N. Singh	Regulation of arsenic stress response by ethylene biosynthesis and signaling in Arabidopsis thaliana	2021
7311	V. B. Fatima	CHEMICAL AND MINERALOGICAL CHARACTERIZATION OF SEDIMENTS FORMED BY MINE WASTES LEACHATES	2018
7312	T. Mannan	Maternal Micronutrient Supplementation and Long Term Health Impact in Children in Rural Bangladesh	2016
7313	S. Chatterjee	Adsorptive removal of arsenic from groundwater using a novel high flux polyacrylonitrile (PAN)-laterite mixed matrix ultrafiltration membrane	2015
7314	A. Das	Co-relation of Arsenic contamination with water table fluctuations and groundwater flow dynamics: A case study in a part of Bengal basin	
7315	Z. M. Liu	Synthesis of magnetic orderly mesoporous alpha-Fe2O3 nanocluster derived from MIL-100(Fe) for rapid and efficient arsenic(III,V) removal	2018
7316	B. K. Nath	Iron oxide Permeated Mesoporous rice-husk nanobiochar (IPMN) mediated removal of dissolved arsenic (As): Chemometric modelling and adsorption dynamics	2019
7317	A. Chakraborty	Efficient Removal of Arsenic (V) from Water Using Steel-Making Slag	2014
7318	D. Li	Dearsenization of caustic solution by synthetic hydrocalumite	2016
7319	A. M. Igra	Early-Life Cadmium Exposure and Bone-Related Biomarkers: A Longitudinal Study in Children	2019
7320	J. S. Wei	Selenium and arsenic removal from water using amine sorbent, competitive adsorption and regeneration	2021
7321	P. C. Ke	Synthesis, in-situ coating and characterization of scorodite with high leaching stability	2019
7322	F. Q. Meng	Novel Dendrimerlike Magnetic Biosorbent Based on Modified Orange Peel Waste: Adsorption-Reduction Behavior of Arsenic	2017
7323	Z. P. Gao	Quantifying Geochemical Processes of Arsenic Mobility in Groundwater From an Inland Basin Using a Reactive Transport Model	2020
7324	S. K. Merid	Epigenome-wide meta-analysis of blood DNA methylation in newborns and children identifies numerous loci related to gestational age	2020

7325	M. Y. Zhang	Enhanced removal of As(III) and As(V) from aqueous solution using ionic liquid-modified magnetic graphene oxide	2019
7326	V. T. Nguyen	Chemical speciation and bioavailability concentration of arsenic and heavy metals in sediment and soil cores in estuarine ecosystem, Vietnam	2018
7327	T. Iwai	Arsenic Speciation and Cadmium Determination in Tobacco Leaves, Ash and Smoke	2016
7328	L. K. Wu	Highly porous copper ferrite foam: A promising adsorbent for efficient removal of As(III) and As(V) from water	2018
7329	P. N. D. Duoc	A novel electrochemical sensor based on double-walled carbon nanotubes and graphene hybrid thin film for arsenic(V) detection	2020
7330	B. J. Han	Practical evaluation of inorganic contaminant presence in a drinking water distribution system after hydraulic disturbance	2018
7331	C. Chung	Synthesis of P-Type ZnO Thin Films with Arsenic Doping and Post Annealing	2016
7332	I. R. Boboev	Development of a Production Scheme for Processing Rebellious Gold-Containing Flotation Concentrates by Autoclave Oxidation	2018
7333	S. Matsumoto	Evaluation of the effects of application of iron materials on the accumulation and speciation of arsenic in rice grain grown on uncontaminated soil with relatively high levels of arsenic	2016
7334	B. Chen	Iron Oxide Supported Sulfhydryl-Functionalized Multiwalled Carbon Nanotubes for Removal of Arsenite from Aqueous Solution	2015
7335	Y. Ye	Synthesis of Three-Dimensional Fe3O4/Graphene Aerogels for the Removal of Arsenic Ions from Water	2015
7336	Q. Yang	Arsenic removal from water by adsorption on iron-contaminated cryptocrystalline graphite	2017
7337	T. G. Asere	Uptake of arsenate by aluminum (hydr)oxide coated red scoria and pumice	2017
7338	D. B. Wu	Online Sequential Fractionation Analysis of Arsenic Adsorbed onto Ferrihydrite by ICP-MS	2020
7339	Y. Q. Gan	Hydrogeochemistry and arsenic contamination of groundwater in the Jianghan Plain, central China	2014
7340	B. Rathi	Process-based modeling of arsenic(III) oxidation by manganese oxides under circumneutral pH conditions	2020
7341	S. Molinari	Smart synthetic maghemite nanoparticles with unique surface properties encode binding specificity toward As-III	2020
7342	S. Deng	Insight into the Influence of Mineralogical Properties of Pristine Pyrite on Its Bioleaching with Thermophiles	2022

7343	B. H. Li	Anaerobic nitrate reduction with oxidation of Fe(II) by Citrobacter Freundii strain PXL1-a potential candidate for simultaneous removal of As and nitrate from groundwater	2015
7344	L. N. Lin	Influence of the application of Fe-Mn-La ternary oxide-biochar composites on the properties of arsenic-polluted paddy soil	2020
7345	A. V. Gamble	Arsenic Speciation and Availability in Orchard Soils Historically Contaminated with Lead Arsenate	2018
7346	H. Neidhardt	Arsenic accumulation in the roots of Helianthus annuus and Zea mays by irrigation with arsenic-rich groundwater: Insights from synchrotron X-ray fluorescence imaging	2015
7347	M. A. Khan	Co application of biofertilizer and zinc oxide nanoparticles upregulate protective mechanism culminating improved arsenic resistance in maize	2022
7348	J. Chen	As(III) Adsorption and Oxidation by Metal (Hydro) Oxides Enriched on Alligator Weed Root	2015
7349	L. Sailo	Arsenic mobilization in the Brahmaputra plains of Assam: groundwater and sedimentary controls	2014
7350	O. A. Akintomide	Pleistocene sands of the Mississippi River Alluvial Aquifer produce the highest groundwater arsenic concentrations in southern Louisiana, USA	2021
7351	I. Allegretta	Characterization of As-polluted soils by laboratory X-ray-based techniques coupled with sequential extractions and electron microscopy: the case of Crocette gold mine in the Monte Rosa mining district (Italy)	2018
7352	Y. O. Kolomiyets	Adsorption of Arsenic by Hybrid Anion-Exchanger Based on Titanium Oxyhydrate	2017
7353	S. Jeong	Importance of chemical binding type between As and iron-oxide on bioaccessibility in soil: Test with synthesized two line ferrihydrite	2017
7354	A. D. Martins	Arsenic, cadmium, and mercury-induced hypertension: mechanisms and epidemiological findings	2018
7355	D. Ocinski	Oxidation and Adsorption of Arsenic Species by means of Hybrid Polymer Containing Manganese Oxides	2014
7356	E. E. C. Kurz	Iron-based subsurface arsenic removal (SAR): Results of a long-term pilot-scale test in Vietnam	2020
7357	V. N. Ermolaeva	Forms of Arsenic Concentration in Sulfide-Free Endogenic Pb-Zn-Sb Ores of the Pelagonian Massif, Republic of North Macedonia	2019
7358	C. Tiberg	Phosphate competition with arsenate on poorly crystalline iron and aluminum (hydr)oxide mixtures	2020
7359	E. T. F. Freitas	Arsenic entrapment by nanocrystals of Al-magnetite: The role of Al in crystal growth and As retention	2016
7360	Z. L. Zhao	Reduced graphene oxide nanosheets modified with plasmonic gold-based hybrid nanostructures and with magnetite (Fe3O4) nanoparticles for cyclic voltammetric determination of arsenic(III)	2019

7361	Z. Yin	Adsorption of arsenic(V) onto single sheet iron oxide: X-ray absorption fine structure and surface complexation	2019
7362	K. F. Pi	Vertical variability of arsenic concentrations under the control of iron-sulfur-arsenic interactions in reducing aquifer systems	2018
7363	M. Laatikainen	Comparison of ion exchange process configurations for arsenic removal from natural waters	2016
7364	X. W. Yu	Ultrafast and deep removal of arsenic in high-concentration wastewater: A superior bulk adsorbent of porous Fe2O3 nanocubes-impregnated graphene aerogel	2019
7365	X. Y. Guo	Magnetic nano capture agent with enhanced anion internal layer diffusion performance for removal of arsenic from human blood	2019
7366	J. Y. Fan	Degradation of p-arsanilic acid and simultaneous in-situ removal of arsenic species with ferrate(VI): Kinetics, intermediate and degradation pathway	2018
7367	K. Schneider	Occurrence of arsenic in ultramafic rocks' alterites from nickel mines in New Caledonia: implications for the contamination of surface waters	2020
7368	C. Gutierrez	Electrochemical peroxidation using iron nanoparticles to remove arsenic from copper smelter wastewater	2015
7369	R. Suter	Tris(2-pyridyl) phosphine as a versatile ligand for pnictogen acceptors	2017
7370	Y. Glocheux	Production of porous aluminium and iron sulphated oxyhydroxides using industrial grade coagulants for optimised arsenic removal from groundwater	2015
7371	F. Nemati	Highly efficient removal of toxic ions by the activated carbon derived fromCitrus limon tree leaves	2021
7372	Z. Y. Qian	Arsenic Transformation in Soil-Rice System Affected by Iron-Oxidizing Strain (Ochrobactrum sp.) and Related Soil Metabolomics Analysis	2022
7373	B. Kim	Sulfuric Acid Baking-Water Leaching for Gold Enrichment and Arsenic Removal from Gold Concentrate	2021
7374	M. F. Siddiqui	Gelatin-polyvinyl alcohol/lanthanum oxide composite: A novel adsorbent for sequestration of arsenic species from aqueous environment	2020
7375	S. Mondal	Evaluation of arsenic adsorption capacity of indigenous materials for their suitability as filter media	2017
7376	K. Ishijima	2,3-Diarylbenzo b arsole: Structural Modification and Polymerization for Tuning of Photophysical Properties	2021
7377	X. Q. Zhang	Predicting the modifying effect of soils on arsenic phytotoxicity and phytoaccumulation using soil properties or soil extraction methods	2020
7378	J. A. Cox	Critical Review-Electrocatalytic Sensors for Arsenic Oxo Species	2020

7379	K. Shehzad	Synthesis of ultra-large ZrO2 nanosheets as novel adsorbents for fast and efficient removal of As(III) from aqueous solutions	2019
7380	S. S. Nielsen	Full scale amendment of a contaminated wood impregnation site with iron water treatment residues	2016
7381	X. X. Li	Lithological controls on arsenic accumulation in cultivated soils: observations from typical karst areas in Central Guizhou, Southwest China	
7382	H. Xu	Functional suppression of macrophages derived from THP-1 cells by environmentally-relevant concentrations of arsenite	2018
7383	S. Ghosh	Delivering Arsenic-free Drinking Water-Made Practically Possible: Continuous Scale Electrochemical Arsenic Remediation Process Furnished, based on Experimental Studies and ANN Simulation	2021
7384	X. Guan	GaAs nanowires with oxidation-proof arsenic capping for the growth of an epitaxial shell	2016
7385	X. J. Liu	Simultaneous removal of cationic heavy metals and arsenic from drinking water by an activated carbon supported nanoscale zero-valent iron and nanosilver composite	2022
7386	K. X. Tian	Tailoring electronic configurations in adsorptive sites for the enhancement of As(V) removal from groundwater	2020
7387	Z. Zhou	Competitive adsorption of arsenic and fluoride on {201} TiO2	2019
7388	T. S. Munonde	Preparation of magnetic Fe3O4 nanocomposites modified with MnO2, Al2O3, Au and their application for preconcentration of arsenic in river water samples	2018
7389	L. N. Pincus	Multifunctional photoactive and selective adsorbent for arsenite and arsenate: Evaluation of nano titanium dioxide-enabled chitosan cross-linked with copper	2018
7390	J. L. Cui	A review of arsenic interfacial geochemistry in groundwater and the role of organic matter	2019
7391	Z. G. Liu	Facile Electrodeposition of MoOx onto Gold Microwire Electrode: Application to Voltammetric Determination of As(III) under Mild Conditions	2015
7392	W. H. Zhou	Anisotropic In-Plane Ballistic Transport in Monolayer Black Arsenic-Phosphorus FETs	2020
7393	Q. Sun	In-situ characterization and assessment of arsenic mobility in lake sediments	2016
7394	E. Di Iorio	Characterization of magnetite nanoparticles synthetized from Fe(II)/nitrate solutions for arsenic removal from water	2019
7395	J. F. Cardenas-Gonzalez	Bioremoval of arsenic (V) from aqueous solutions by chemically modified fungal biomass	2017
7396	M. Ciopec	Testing of Chemically Activated Cellulose Fibers as Adsorbents for Treatment of Arsenic Contaminated Water	2021

7397	M. D. Oztel	Arsenite removal by adsorption onto iron oxide-coated pumice and sepiolite	2015
7398	Y. Y. Zhang	Arsenic Phosphorus Monolayer: A Promising Candidate for H2S Sensor and NO Degradation With High Sensitivity and Selectivity	2017
7399	H. Y. Zhang	Mechanism of Fluoride and Arsenic Removal by Ce/gamma-AlO(0)3 Based on XRD and FTIR	2020
7400	L. C. Maia	A review on the use of lignocellulosic materials for arsenic adsorption	2021
7401	Z. Kolbert	Involvement of nitric oxide (NO) in plant responses to metalloids	2021
7402	I. Jacukowicz-Sobala	Photocatalytically-assisted oxidative adsorption of As(III) using sustainable multifunctional composite material - Cu2O doped anion exchanger	2022
7403	M. Stiboller	Lipid-soluble arsenic species identified in the brain of the marine fish skipjack tuna (Katsuwonus pelamis) using a sequential extraction and HPLC/mass spectrometry	2019
7404	Z. J. Bajic	Adsorption Study of Arsenic Removal by Novel Hybrid Copper Impregnated Tufa Adsorbents in a Batch System	2016
7405	G. H. Moon	Platinum-like Behavior of Reduced Graphene Oxide as a Cocatalyst on TiO2 for the Efficient Photocatalytic Oxidation of Arsenite	2014
7406	X. L. Yan	Arsenic stabilization performance of a novel starch-modified Fe-Mn binary oxide colloid	2020
7407	R. K. Chellappan	High-temperature thermal stability study of 1 nm Al2O3 deposited on InAs surfaces investigated by synchrotron radiation based photoemission spectroscopy	2014
7408	R. K. Patel	Elimination of Fluoride, Arsenic, and Nitrate from Water Through Adsorpation onto Nano-Adsorbent: A Review	2019
7409	J. R. Schilz	Removal of Trace Elements by Cupric Oxide Nanoparticles from Uranium In Situ Recovery Bleed Water and Its Effect on Cell Viability	2015
7410	J. Yang	High- Content, Well- Dispersed. gamma-Fe-2 O-3 Nanoparticles Encapsulated in Macroporous Silica with Superior Arsenic Removal Performance	2014
7411	F. Q. Guo	Kinetics of iron(III)-catalyzed oxidation of arsenic(III) in acidic solutions with SO2/O-2 gas mixture using different iron sources	2019
7412	A. A. Alswat	Zeolite modified with copper oxide and iron oxide for lead and arsenic adsorption from aqueous solutions	2016
7413	Z. Ulhassan	Attenuation mechanisms of arsenic induced toxicity and its accumulation in plants by engineered nanoparticles: A review	2022
7414	Y. Q. Song	Uptake of arsenic(V) using iron and magnesium functionalized highly ordered mesoporous MCM-41 (Fe/Mg-MCM-41) as an effective adsorbent	2022

7415	S. R. Ryu	Adsorption of As(III) and As(V) in groundwater by Fe-Mn binary oxide-impregnated granular activated carbon (IMIGAC)	2017
7416	L. Peng	Enhancing immobilization of arsenic in groundwater: A model-based evaluation	2017
7417	G. Verma	Exogenous application of methyl jasmonate alleviates arsenic toxicity by modulating its uptake and translocation in rice (Oryza sativa L.)	2020
7418	Z. Souri	Nitric oxide improves tolerance to arsenic stress in Isatis cappadocica desv. Shoots by enhancing antioxidant defenses	2020
7419	J. Liu	Exposure to Copper Oxide Nanoparticles and Arsenic Causes Intergenerational Effects on Rice (Oryza sativa japonica Koshihikari) Seed Germination and Seedling Growth	2019
7420	K. Ohe	Development of Yttrium(III)-Incorporated Magnetite for Adsorptive Removal of Arsenic and a Study of the Adsorption Mechanism	2019
7421	B. J. Maciag	Speciation of arsenic and antimony in basaltic magmas	2020
7422	Z. F. He	Multiple-pathway arsenic oxidation and removal from wastewater by a novel manganese-oxidizing aerobic granular sludge	2019
7423	D. Ocinski	Alginate beads containing water treatment residuals for arsenic removal from water-formation and adsorption studies	2016
7424	D. Piacentini	Cadmium and arsenic-induced-stress differentially modulates Arabidopsis root architecture, peroxisome distribution, enzymatic activities and their nitric oxide content	2020
7425	O. H. Kwon	Adsorption of As(III), As(V) and Cu(II) on zirconium oxide immobilized alginate beads in aqueous phase	2016
7426	P. Yuan	Tumor targeted self-synergistic nanoplatforms for arsenic-sensitized photodynamic therapy	2020
7427	S. Ullah	Application of visible light activated thiolated cobalt doped ZnO nanoparticles towards arsenic removal from aqueous systems	2022
7428	M. E. Josende	Graphene oxide and GST-omega enzyme: An interaction that affects arsenic metabolism in the shrimp Litopenaeus vannamei	2020
7429	H. Pan	High catalytic oxidation of As(III) by Molecular oxygen over Fe-loaded Silicon carbide with MW activation	2018
7430	N. Tian	Well-dispersed magnetic iron oxide nanocrystals on sepiolite nanofibers for arsenic removal	2015
7431	S. S. Wu	Enhanced removal of organoarsenic by chlorination: Kinetics, effect of humic acid, and adsorbable chlorinated organoarsenic	2022
7432	T. Mukherjee	Bioremediation potential of arsenic by non-enzymatically biofabricated silver nanoparticles adhered to the mesoporous carbonized fungal cell surface of Aspergillus foetidus MTCC8876	2017

7433	M. Sridharan	Chemometric tool to study the mechanism of arsenic contamination in groundwater of Puducherry region, South East coast of India	2018
7434	M. Cable	A study of refining. Part 1: Measurements of the refining of a soda-lime-silica glass with and without refining agents	2016
7435	W. Cheng	Dispersion-precipitation synthesis of nanosized magnetic iron oxide for efficient removal of arsenite in water	2015
7436	X. Li	Extraordinary Deactivation Offset Effect of Arsenic and Calcium on CeO2-WO3 SCR Catalysts	2018
7437	N. Das	Provenance, prevalence and health perspective of co-occurrences of arsenic, fluoride and uranium in the aquifers of the Brahmaputra River floodplain	2018
7438	Y. K. Penke	Redox synergistic Mn-Al-Fe and Cu-Al-Fe ternary metal oxide nano adsorbents for arsenic remediation with environmentally stable As(0) formation	2019
7439	C. Y. Chang	Bioavailability of antimony and arsenic in a flowering cabbage-soil system: Controlling factors and interactive effect	2022
7440	M. J. Al Marri	Mechanism of GaAs Surface Sulfidation	2014
7441	L. Y. Wang	The effect of extracellular electron transfer on arsenic speciation transformation in a soil bioelectrochemical system	2020
7442	S. M. Howard	Three-Dimensional Predominance Volume Diagrams: The Ni-As-S-O System	2018
7443	T. Ahmed	Green magnesium oxide nanoparticles-based modulation of cellular oxidative repair mechanisms to reduce arsenic uptake and translocation in rice (Oryza sativa L.) plants	2021
7444	J. C. Bullen	On the application of photocatalyst-sorbent composite materials for arsenic (III) remediation: Insights from kinetic adsorption modelling	2020
7445	X. L. Yan	Stabilization of soil arsenic by natural limonite after mechanical activation and the associated mechanisms	2020
7446	M. Kong	Performance impact and poisoning mechanism of arsenic over commercial V2O5-WO3/TiO2 SCR catalyst	2015
7447	X. X. Wang	Differential impacts of copper oxide nanoparticles and Copper(II) ions on the uptake and accumulation of arsenic in rice (Oryza sativa)	2019
7448	B. X. Liu	Sulfur-doped Fe-Cu-La trimetallic oxides as a novel magnetic adsorbent for efficient removal of As(iii) and As(v) from aqueous solution	2021
7449	Q. Zhang	Impact of Redox Condition on Fractionation and Bioaccessibility of Arsenic in Arsenic-Contaminated Soils Remediated by Iron Amendments: A Long-Term Experiment	2018
7450	H. H. Du	Tungsten distribution and vertical migration in soils near a typical abandoned tungsten smelter	2022

7451	S. M. Prabhu	Self-tuning tetragonal zirconia-based bimetallic nano(hydr)oxides as superior and recyclable adsorbents in arsenic-tolerant environment: Template-free in and ex situ synthetic methods, stability, and mechanisms	2020
7452	A. C. Heredia	Cathodic stripping square-wave voltammetry for assessing As(III) removal with synthetic mixed oxides	2017
7453	X. X. Wang	Simultaneous mitigation of arsenic and cadmium accumulation in rice (Oryza sativa L.) seedlings by silicon oxide nanoparticles under different water management schemes	2020
7454	J. B. Long	Mycorrhiza and Iron Tailings Synergistically Enhance Maize Resistance to Arsenic on Medium Arsenic-Polluted Soils Through Increasing Phosphorus and Iron Uptake	2021
7455	S. Sert	Removal of arsenic(III) ions from aqueous solutions by modified hazelnut shell	2017
7456	C. G. Lee	Removal of arsenic and selenium from aqueous solutions using magnetic iron oxide nanoparticle/multi-walled carbon nanotube adsorbents	2016
7457	H. M. Liu	Vaporization model of arsenic during single-particle coal combustion: Numerical simulation	2021
7458	Y. H. Hu	Grape seed proanthocyanidin extract alleviates arsenic-induced lung damage through NF-kappa B signaling	2019
7459	K. X. Zhang	Arsenic Trioxide Attenuates NF-kappa B and Cytokine mRNA Levels in the Livers of Cocks	2016
7460	J. C. J. Gude	Fate of low arsenic concentrations during full-scale aeration and rapid filtration	2016
7461	E. Weidner	Removal of Hazardous Oxyanions from the Environment Using Metal-Oxide-Based Materials	2019
7462	L. A. Zemskova	Composite sorbents based on synthetic manganese oxide and carbon fiber	2016
7463	A. Romero-Freire	Influence of soil properties on the bioaccumulation and effects of arsenic in the earthworm Eisenia andrei	2015
7464	H. Yoshida	Arsenic Removal from Contaminated Water Using the CaO-SiO2-FeO Glassy Phase in Steelmaking Slag	2017
7465	J. L. Cui	Speciation, mobilization, and bioaccessibility of arsenic in geogenic soil profile from Hong Kong	2018
7466	J. O. Aremu	Kinetic and isotherm studies on adsorption of arsenic using silica based catalytic media	2019
7467	B. Tunno	Separating spatial patterns in pollution attributable to woodsmoke and other sources, during daytime and nighttime hours, in Christchurch, New Zealand	2019
7468	X. J. Xie	Soil geochemistry and groundwater contamination in an arsenic-affected area of the Datong Basin, China	2014

7469	S. C. Lee	Hierarchically three-dimensional (3D) nanotubular sea urchin -shaped iron oxide and its application in heavy metal removal and solar-induced photocatalytic degradation	2018
7470	C. M. Babu	Characterization of reduced graphene oxide supported mesoporous Fe2O3/TiO2 nanoparticles and adsorption of As(III) and As(V) from potable water	2016
7471	A. K. Mensah	Arsenic contamination in abandoned and active gold mine spoils in Ghana: Geochemical fractionation, speciation, and assessment of the potential human health risk	2020
7472	S. U. Din	Detailed kinetics study of arsenate adsorption by a sequentially precipitated binary oxide of iron and silicon	2019
7473	S. Ismail	Electrochemical Detection of Arsenite Using a Silica Nanoparticles-Modified Screen-Printed Carbon Electrode	2020
7474	A. Lock	Rates and processes affecting As speciation and mobility in lake sediments during aging	2018
7475	A. M. Yousif	Fast and selective adsorption of As(V) on prepared modified cellulose containing Cu(II) moieties	2016
7476	O. S. Thirunavukkarasu	Performance of reverse osmosis and manganese greensand plants in removing naturally occurring substances in drinking water	2014
7477	W. R. Gallegos-Perez	Effect of UV radiation on the structure of graphene oxide in water and its impact on cytotoxicity and As(III) adsorption	2020
7478	M. Orellana-Saez	In-Depth Genomic and Phenotypic Characterization of the Antarctic Psychrotolerant Strain Pseudomonas sp. MPC6 Reveals Unique Metabolic Features, Plasticity, and Biotechnological Potential	2019
7479	N. Wang	Fe2O3 enhanced high-temperature arsenic resistance of CeO2-La2O3/TiO2 catalyst for selective catalytic reduction of NOx with NH3	2021
7480	Z. Huang	Selective removal of high concentration arsenate from aqueous solution by magnetic Fe-Y binary oxide	2020
7481	J. Liu	Mobility of arsenic in the growth media of rice plants (Oryza sativa subsp. japonica. 'Koshihikari') with exposure to copper oxide nanoparticles in a life-cycle greenhouse study	2021
7482	L. Onnby	Improved arsenic(III) adsorption by Al2O3 nanoparticles and H2O2: Evidence of oxidation to arsenic(V) from X-ray absorption spectroscopy	2014
7483	L. Newsome	NanoSIMS imaging of extracellular electron transport processes during microbial iron(III) reduction	2018
7484	M. Pigna	Arsenic in the Soil Environment: Mobility and Phytoavailability	2015
7485	H. Eslami	Enhanced coagulation process by Fe-Mn bimetal nano-oxides in combination with inorganic polymer coagulants for improving As(V) removal from contaminated water	2019
7486	P. Drahota	Comparison of Pb, Zn, Cd, As, Cr, Mo and Sb Adsorption onto Natural Surface Coatings in a Stream Draining Natural As Geochemical Anomaly	2014

7487	Q. Sun	Improved Diffusive Gradients in Thin Films (DGT) Measurement of Total Dissolved Inorganic Arsenic in Waters and Soils Using a Hydrous Zirconium Oxide Binding Layer	2014
7488	V. V. Gavrilenko	Highly efficient synthesis of trimethylarsine	1996
7489	L. K. McDonough	Changes in groundwater dissolved organic matter character in a coastal sand aquifer due to rainfall recharge	2020
7490	L. Peng	Removal of Trace As(V) from Water with the Titanium Dioxide/ACF Composite Electrode	2015
7491	H. D. Zhang	Synergistic effect of arsenic and different potassium species on V2O5-WO3/TiO2 catalyst poisoning: Comparison of Cl-, SO42- and NO3- anions	2020
7492	X. S. Liu	Hg-0 oxidation and SO3, Pb-0, PbO, PbCl2 and As2O3 adsorption by graphene-based bimetallic catalyst ((Fe,Co)@N-GN): A DFT study	2019
7493	Y. Liu	Peroxymonosulfate Improves the Activity and Stability of Manganese Oxide for Oxidation of Arsenite to Arsenate	2020
7494	X. J. Xie	In-situ arsenic remediation by aquifer iron coating: Field trial in the Datong basin, China	2016
7495	S. H. Yao	Arsenic removal from aqueous solutions by adsorption onto iron oxide/activated carbon magnetic composite	2014
7496	J. Zhu	Fe3O4 and MnO2 assembled on honeycomb briquette cinders (HBC) for arsenic removal from aqueous solutions	2015
7497	H. Alijani	Effective aqueous arsenic removal using zero valent iron doped MWCNT synthesized by in situ CVD method using natural alpha-Fe2O3 as a precursor	2017
7498	E. K. Jeon	Enhanced adsorption of arsenic onto alum sludge modified by calcination	2018
7499	S. P. Dubey	Synthesis and characterization of metal-doped reduced graphene oxide composites, and their application in removal of Escherichia coli, arsenic and 4-nitrophenol	2015
7500	M. S. Safarzadeh	The pyrometallurgy of enargite: A literature update	2016
7501	C. Zou	Theoretical study of the reactions between arsenic and nitrogen oxides during coal combustion	2019
7502	H. T. Lu	In Situ Oxidation and Efficient Simultaneous Adsorption of Arsenite and Arsenate by Mg-Fe-LDH with Persulfate Intercalation	2016
7503	C. H. Nguyen	Microbial toxicity of gallium- and indium-based oxide and arsenide nanoparticles	2020
7504	M. Purnima	AC conductivity and dielectric properties of MgO-Li2O-B2O3-As2O3 glasses	2016

7505	G. N. Lu	The distribution of arsenic fractions and alkaline phosphatase activities in different soil aggregates following four months As(V) ageing	2019
7506	D. D. Zhao	Fabrication and testing of zirconium-based nanoparticle-doped activated carbon fiber for enhanced arsenic removal in water	2016
7507	A. Arencibia	Efficient aqueous As(III) removal by adsorption on thiol-functionalized mesoporous silica	2020
7508	Z. J. Li	Hydrogeochemical controls on arsenic contamination potential and health threat in an intensive agricultural area, northern China	2020
7509	O. Font	Origin and speciation of major and trace PM elements in the barcelona subway system	2019
7510	S. A. Schmidt	Pilot study on arsenic removal from groundwater using a small-scale reverse osmosis system - Towards sustainable drinking water production	2016
7511	P. Drahota	Arsenic fractionation and mobility in sulfidic wetland soils during experimental drying	2021
7512	H. P. Zeng	Arsenic(V) removal by granular adsorbents made from water treatment residuals materials and chitosan	2020
7513	J. Curko	Adsorption Characteristics of Different Adsorbents and Iron(III) Salt for Removing As(V) from Water	2016
7514	Y. D. Yang	The catalytic aerial oxidation of As(III) in alkaline solution by Mn-loaded diatomite	2022
7515	M. Jablonska-Czapla	Antimony, Arsenic and Chromium Speciation Studies in Biala Przemsza River (Upper Silesia, Poland) Water by HPLC-ICP-MS	2015
7516	P. B. Hu	Strengthened arsenic adsorption over Ni-modified gamma-Al2O3 under different operation conditions: An experimental and simulation study	2022
7517	M. A. Carneiro	Efficient removal of arsenic from aqueous solution by continuous adsorption onto iron-coated cork granulates	2022
7518	Z. P. Wen	Facile template-free fabrication of iron manganese bimetal oxides nanospheres with excellent capability for heavy metals removal	2017
7519	S. Purwajanti	Mesoporous Magnesium Oxide Hollow Spheres as Superior Arsenite Adsorbent: Synthesis and Adsorption Behavior	2016
7520	S. Sharma	Adsorption of arsenic (V) ions onto cellulosic-ferric oxide system: kinetics and isotherm studies	2016
7521	C. K. Chen	Specifically designed amine functional group doped sludge biochar for inorganic and organic arsenic removal	2021
7522	J. R. Xi	Preparation of carbon/Al2O3/nZVI magnetic nanophase materials produced from drinking water sludge for the removal of As(V) from aqueous solutions	2021

7523	D. Zhao	Rapid Preparation of TiO2-x and Its Photocatalytic Oxidation for Arsenic Adsorption under Visible Light	2020
7524	N. Alozie	Biochar immobilizes soil-borne arsenic but not cationic metals in the presence of low-molecular-weight organic acids	2018
7525	U. R. Raonic	Influence of processing conditions on the optical band gap in thermo-mechanically doped amorphous as_2s_3	2017
7526	L. J. Lan	Rapid and effective removal of As(III) and As(V) using spore@Ti4+ microspheres	2018
7527	N. V. Solenkova	Metal pollutants and cardiovascular disease: Mechanisms and consequences of exposure	2014
7528	N. M. Silveira	Nitric Oxide Attenuates Oxidative Stress Induced by Arsenic in Lettuce (Lactuca sativa) Leaves	2015
7529	L. Yang	Distribution and genetic diversity of the microorganisms in the biofilter for the simultaneous removal of arsenic, iron and manganese from simulated groundwater	2014
7530	N. Arancibia-Miranda	Mechanistic insights into simultaneous removal of copper, cadmium and arsenic from water by iron oxide-functionalized magnetic imogolite nanocomposites	2020
7531	Z. F. Chi	Selective removal of As(III) using magnetic graphene oxide ion-imprinted polymer in porous media: Potential effect of external magnetic field	2021
7532	E. B. Simsek	Novel composite sorbents based on carbon fibers decorated with ferric hydroxides-Arsenic removal	2018
7533	J. X. Zhao	Rapid and efficient catalytic oxidation of As(III) with oxygen over a Pt catalyst at increased temperature	2017
7534	Y. L. Zhang	Microbial reduction of As(V)-loaded Schwertmannite by Desulfosporosinus meridiei	2021
7535	H. H. Su	Arsenic removal from water by photocatalytic functional Fe2O3-TiO2 porous ceramic	2017
7536	Z. W. Zhao	The effects of antimony oxide on the structure of iron phosphate glass for the immobilisation of arsenic	2015
7537	A. Udupa	Selective area formation of arsenic oxide-rich octahedral microcrystals during photochemical etching of n-type GaAs	2018
7538	J. J. Xie	Comparison of arsenic fractions and health risks in PM2.5 before and after coal-gas replacement	2020
7539	M. Kumar	Use of cellulose acetate/polyphenylsulfone derivatives to fabricate ultrafiltration hollow fiber membranes for the removal of arsenic from drinking water	2019
7540	T. V. Mal'tseva	Hybrid adsorbents based on hydrated oxides of Zr(IV), Ti(IV), Sn(IV), and Fe(III) for arsenic removal	2017

7541	H. Parschova	The Effect of Accompanying Anions on Arsenate Sorption onto Selective Sorbents	2015
7542	S. Wu	Fate of As(III) and As(V) during Microbial Reduction of Arsenic-Bearing Ferrihydrite Facilitated by Activated Carbon	2018
7543	Z. Y. Fang	Enhanced removal of arsenic from water by using sub-10 nm hydrated zirconium oxides confined inside gel-type anion exchanger	2021
7544	G. N. Liang	The generation of biogenic manganese oxides and its application in the removal of As(III) in groundwater	2017
7545	M. Tripathy	Hematite decorated functional porous graphitic carbon nitride binary nanohybrid: Mechanistic insight into the formation and arsenic adsorption study	2022
7546	K. Matsunaga	Development of the simple analytical method for determination of arsenate(V) ion using fluorescence-labeled DNA and cerium oxide nanoparticles	2022
7547	A. Sigdel	Immobilization of hydrous iron oxides in porous alginate beads for arsenic removal from water	2018
7548	C. Yokoyama	Pretreatment Effects on High-k/InxGa1-xAs MOS Interface Properties and Their Physical Model	2018
7549	J. Byun	Magnetic BaFe12O19 nanofiber filter for effective separation of Fe3O4 nanoparticles and removal of arsenic	2014
7550	A. O. Barimah	Sensitive label-free Cu2O/Ag fused chemometrics SERS sensor for rapid detection of total arsenic in tea	2021
7551	S. Agrawal	Removal of arsenic from aqueous solution by an adsorbent nickel ferrite-polyaniline nanocomposite	2016
7552	H. Fakour	Effect of Humic Acid on As Redox Transformation and Kinetic Adsorption onto Iron Oxide Based Adsorbent (IBA)	2014
7553	D. E. Ortega	Exploring the Nature of Interaction and Stability between Water-Soluble Arsenic Pollutants and Metal-Phosphorene Hybrids: A Density Functional Theory Study	2020
7554	K. F. Pi	Hydrogeochemistry of co-occurring geogenic arsenic, fluoride and iodine in groundwater at Datong Basin, northern China	2015
7555	L. Li	Revisiting the biogeochemistry of arsenic in the Baltic Sea: Impact of anthropogenic activity	2018
7556	X. L. Duan	Gaseous Arsenic Capture in Flue Gas by CuCl2-Modified Halloysite Nanotube Composites with High-Temperature NOx and SOx Resistance	2022
7557	E. M. Ahmed	Electrical conduction and switching properties of As2O3 center dot V2O5 center dot FeO glasses	2018
7558	Z. W. Qiu	Efficient oxidation and absorption of As(III) from aqueous solutions for environmental remediation via CuO@MNW membranes	2020

7559	M. S. Podder	Fixed-bed column study for As(III) and As(V) removal and recovery by bacterial cells immobilized on Sawdust/MnFe2O4 composite	2016
7560	T. Yang	Highly effective oxidation of roxarsone by ferrate and simultaneous arsenic removal with in situ formed ferric nanoparticles	2018
7561	A. A. T. Bafrooee	Ethylenediamine functionalized magnetic graphene oxide (Fe3O4@GO-EDA) as an efficient adsorbent in Arsenic(III) decontamination from aqueous solution	2021
7562	M. Zabihi	Competitive adsorption of arsenic and mercury on nano-magnetic activated carbons derived from hazelnut shell	2022
7563	N. M. Shafik	Protective Effects of Combined Selenium and Punica granatum Treatment on Some Inflammatory and Oxidative Stress Markers in Arsenic-Induced Hepatotoxicity in Rats	2016
7564	A. H. Baghaie	Effect of Nano Fe-oxide and Endophytic Fungus (P. indica) on Petroleum Hydrocarbons Degradation in an Arsenic Contaminated Soil under Barley Cultivation	2019
7565	H. P. Zeng	Characterization and Arsenic Adsorption Behaviors of Water Treatment Residuals from Waterworks for Iron and Manganese Removal	2019
7566	D. Sathiyaseelan	Wavelet based spectral approach for solving surface coverage model in an electrochemical arsenic sensor - An operational matrix approach	2018
7567	K. Khanna	Arsenic as hazardous pollutant: Perspectives on engineering remediation tools	2022
7568	C. F. Ding	Modeling the transfer of arsenic from soil to carrot (Daucus carota L.)-a greenhouse and field-based study	2015
7569	I. Shiue	Urinary heavy metals, phthalates and polyaromatic hydrocarbons independent of health events are associated with adult depression: USA NHANES, 2011-2012	2015
7570	C. Sudhakar	Interference of Phosphate in Adsorption of Arsenate and Arsenite over Confined Metastable Two-Line Ferrihydrite and Magnetite	2021
7571	X. J. Xie	Arsenic removal by manganese-doped mesoporous iron oxides from groundwater: Performance and mechanism	2022
7572	S. Pathan	Acid functionalized-nanoporous carbon/MnO2 composite for removal of arsenic from aqueous medium	2019
7573	B. Dousova	Leaching effect on arsenic mobility in agricultural soils	2016
7574	S. Y. Li	Effects of elevated sulfate concentration on the mobility of arsenic in the sediment-water interface	2018
7575	S. W. Liu	A nanoparticulate liquid binding phase based DGT device for aquatic arsenic measurement	2016
7576	M. B. Shakoor	Remediation of arsenic-contaminated water using agricultural wastes as biosorbents	2016

7577	D. Tussipkan	Alfalfa (Medicago Sativa L.): Genotypic Diversity and Transgenic Alfalfa for Phytoremediation	2022
7578	L. Fan	Hypoxia enhances the cytotoxic effect of As4S4 on rat ventricular H9c2 cells through activation of ubiquitin-proteasome system	2021
7579	D. Tetreault	Water Grabbing via Institutionalised Corruption in Zacatecas, Mexico	2018
7580	Y. S. Lee	Sodium meta-arsenite prevents the development of autoimmune diabetes in NOD mice	2015
7581	S. K. Ahmmad	FT-IR and Raman spectroscopic studies of ZnF2-ZnO-As2O3-TeO2	2016
7582	Y. Wakui	Visual Detection of Selenium(IV) Using a Gallium(III) Complex Retained in a Support Filter	2022
7583	Y. Du	Enrichment of Geogenic Ammonium in Quaternary Alluvial- Lacustrine Aquifer Systems: Evidence from Carbon Isotopes and DOM Characteristics	2020
7584	S. Mohammadi	A Chelation-enhanced Fluorescence Assay using Thiourea Capped Carbonaceous Fluorescent Nanoparticles for As (III) Detection in Water Samples	2022
7585	S. Sachdeva	MiADMSA abrogates sodium tungstate-induced oxidative stress in rats	
7586	L. Bindi	Twinning, Superstructure and Chemical Ordering in Spryite, Ag-8(As0.503+As0.505+)S-6, at Ultra-Low Temperature: An X-Ray Single-Crystal Study	2021
7587	M. R. Gherase	Probing Trace Elements in Human Tissues with Synchrotron Radiation	2020
7588	K. Jordaan	An integrated insight into the response of bacterial communities to anthropogenic contaminants in a river: A case study of the Wonderfonteinspruit catchment area, South Africa	2019
7589	G. Mehetre	Untapped bacterial diversity and metabolic potential within Unkeshwar hot springs, India	2018
7590	C. Wu	Remediation of arsenic-contaminated paddy soil by iron-modified biochar	2018
7591	N. H. Matthews	Exposure to Trace Elements and Risk of Skin Cancer: A Systematic Review of Epidemiologic Studies	2019
7592	D. Davis	Biomass composition of the golden tide pelagic seaweeds Sargassum fluitans and S. natans (morphotypes I and VIII) to inform valorisation pathways	2021
7593	I. Shiue	Urinary heavy metals, phthalates, phenols, thiocyanate, parabens, pesticides, polyaromatic hydrocarbons but not arsenic or polyfluorinated compounds are associated with adult oral health: USA NHANES, 2011-2012	2015
7594	T. Negishi	Dysregulation of MAP Kinase Signaling Pathways Including p38MAPK, SAPK/JNK, and ERK1/2 in Cultured Rat Cerebellar Astrocytes Exposed to Diphenylarsinic Acid	2017

7595	W. C. Yu	The application of multivariate statistical analysis to the analysis of characteristics and assessment methods of heavy Metal soil contamination in Taiwan	2017
7596	N. Hattab	Effect of fresh and mature organic amendments on the phytoremediation of technosols contaminated with high concentrations of trace elements	2015
7597	R. Sharifi	Mobility and chemical fate of arsenic and antimony in water and sediments of Sarouq River catchment, Takab geothermal field, northwest Iran	2016
7598	I. Andjelkovic	Fe Doped TiO2 Prepared by Microwave-Assisted Hydrothermal Process for Removal of As(III) and As(V) from Water	2014
7599	B. Nfor	Effects of Electronic and Electrical Waste-Contaminated Soils on Growth and Reproduction of Earthworm (Alma nilotica)	2022
7600	I. Y. Ilyin	Reactions of K-2 Fe(3)Q(CO)(9) (Q = Se, Te) with AsI3: Synthesis and Structures of the First {Fe3TeAs} Clusters with Capping A mu(3)-AsI and Bridging A mu(6),kappa(3):kappa(3)-As-2 Ligands	2015
7601	J. Sanchez	Bio-Based Hydrogels With Ion Exchange Properties Applied to Remove Cu(II), Cr(VI), and As(V) Ions From Water	2021
7602	S. L. Nicholas	Solid-phase arsenic speciation in aquifer sediments: A micro-X-ray absorption spectroscopy approach for quantifying trace-level speciation	2017
7603	B. Pierri	Exposure study on susceptible people-SPES: An integrative biomonitoring approach	2022
7604	T. T. Zhu	Quantitative X-ray photoelectron spectroscopy-based depth profiling of bioleached arsenopyrite surface by Acidithiobacillus ferrooxidans	2014
7605	O. E. R. Ramos	Geochemical processes controlling mobilization of arsenic and trace elements in shallow aquifers and surface waters in the Antequera and Poopo mining regions, Bolivian Altiplano	2014
7606	S. Poorsadeghi	Removal of Arsenic from Water Using Aluminum Nanoparticles Synthesized through Arc Discharge Method	2017
7607	Y. Fang	Advances in design of metal-organic frameworks activating persulfate for water decontamination	2021
7608	K. Frischkorn	De novo assembly of Aureococcus anophagefferens transcriptomes reveals diverse responses to the low nutrient and low light conditions present during blooms	2014
7609	M. Battistel	Model-based interpretation of hydrogeochemistry and arsenic mobility in a low-enthalpy hydrothermal system	2020
7610	M. Islam	Solvothermal synthesis of greigite (Fe3S4)- Conducting polypyrrole nanocomposite and its application towards arsenic removal	2017
7611	P. Rai	Iron oxide nanoparticles impart cross tolerance to arsenate stress in rice roots through involvement of nitric oxide*	2022
7612	R. T. Emeny	Prenatal exposure to mercury in relation to infant infections and respiratory symptoms in the New Hampshire Birth Cohort Study	2019

7613	R. Nishi	Simultaneous Arsenic and Iron Oxidation for One-Step Scorodite Crystallization Using Mn Oxide	2021
7614	X. Li	PM2.5-bound elements in Hebei Province, China: Pollution levels, source apportionment and health risks	2022
7615	M. Z. Zhang	UV-induced highly efficient removal of As(III) through synergistic photo-oxidation in the presence of Fe(II)	
7616	P. Yadav	HMO-incorporated electrospun nanofiber recyclable membranes: Characterization and adsorptive performance for Pb(II) and As(V)	2021
7617	S. Dutta	Hollow Polyaniline Microsphere/Fe3O4 Nanocomposite as an Effective Adsorbent for Removal of Arsenic from Water	2020
7618	K. F. Pi	Arsenic immobilization by in-situ iron coating for managed aquifer rehabilitation	2020
7619	L. Yu	Development and characterization of yttrium-ferric binary composite for treatment of highly concentrated arsenate wastewater	2019
7620	T. Bechshoft	Monitoring spatially resolved trace elements in polar bear hair using single spot laser ablation ICP-MS	2020
7621	R. Gajek	Determination of ultra-trace elements in human plasma or serum by ICP-MS using sodium in the presence of carbon as a single calibration matrix-match component	2015
7622	M. S. German	Evidence of Economically Sustainable Village-Scale Microenterprises for Arsenic Remediation in Developing Countries	2019
7623	B. Lalinska-Volekova	Hydrous ferric oxides (HFO's) precipitated from contaminated waters at several abandoned Sb deposits - Interdisciplinary assessment	2022
7624	J. Hahn	Joint recording of contamination status, multi-element dynamics, and source identification on a sub-catchment scale: The example Lahn River (Germany)	2021
7625	Z. Chen	Impacts of enhanced microbial-photoreductive and suppressed dark microbial reductive dissolution on the mobility of As and Fe in flooded tailing soils with zinc sulfide	2019
7626	M. T. Naseri	Determination of lewisite metabolite 2-chlorovinylarsonous acid in urine by use of dispersive derivatization liquid-liquid microextraction followed by gas chromatography-mass spectrometry	2014
7627	Y. L. Kong	Carbothermal synthesis of nano-iron-carbon composites for arsenate removal from high-arsenic acid wastewater	2022
7628	S. C. Maguffin	Influence of manganese abundances on iron and arsenic solubility in rice paddy soils	2020
7629	J. Ma	Arsenic Adsorption and its Fractions on Aquifer Sediment: Effect of pH, Arsenic Species, and Iron/Manganese Minerals	2015
7630	S. J. Zhou	Stabilization of arsenic and antimony Co-contaminated soil with an iron-based stabilizer: Assessment of strength, leaching and hydraulic properties and immobilization mechanisms	2022

7631	S. Whitacre	Modification of an existing in vitro method to predict relative bioavailable arsenic in soils	2017
7632	M. Yazdani	Synthesis, characterization and exploitation of nano-TiO2/feldspar-embedded chitosan beads towards UV-assisted adsorptive abatement of aqueous arsenic (As)	2017
7633	I. Fuentes	Long-term trace element assessment after a mine spill: Pollution persistence and bioaccumulation in the trophic web	2020
7634	D. Coniglio	Positional Assignment of C-C Double Bonds in Fatty Acyl Chains of Intact Arsenosugar Phospholipids Occurring in Seaweed Extracts by Epoxidation Reactions	2022
7635	Z. J. Lin	Effective and selective adsorption of organoarsenic acids from water over a Zr-based metal-organic framework	2019
7636	K. Wu	Magnetic Fe3O4@CuO nanocomposite assembled on graphene oxide sheets for the enhanced removal of arsenic(III/V) from water	2019
7637	M. M. Shen	Partitioning and reactivity of iron oxide minerals in aquifer sediments hosting high arsenic groundwater from the Hetao basin, P. R. China	2018
7638	Y. Z. Zhou	Distribution of groundwater arsenic in Xinjiang, PR China	2017
7639	Z. Hassan	Iron Cycling Potentials of Arsenic Contaminated Groundwater in Bangladesh as Revealed by Enrichment Cultivation	2016
7640	A. Bari	Soil washing of arsenic from mixed contaminated abandoned mine soils and fate of arsenic after washing	2022
7641	K. Simeonidis	Regeneration of arsenic spent adsorbents by Fe/MgO nanoparticles	2017
7642	C. T. Clark	Walrus teeth as biomonitors of trace elements in Arctic marine ecosystems	2021
7643	S. Gallardo	Chemical, Leaching, and Toxicity Characteristics of Coal Ashes from Circulating Fluidized Bed of a Philippine Coal-Fired Power Plant	2015
7644	W. Shin	Evaluation of multiple PRPs' contributions to soil contamination in reclaimed sites around an abandoned smelter	2018
7645	W. D. Xu	Reno-Protective Effect of Realgar Nanoparticles on Lupus Nephritis of MRL/Lpr Mice through STAT1	2019
7646	M. H. Chien	Tricetin Induces Apoptosis of Human Leukemic HL-60 Cells through a Reactive Oxygen Species-Mediated c-Jun N-Terminal Kinase Activation Pathway	2017
7647	I. Shiue	Urinary polyaromatic hydrocarbons are associated with adult emphysema, chronic bronchitis, asthma, and infections: US NHANES, 2011-2012	2016
7648	S. I. Siddiqui	Arsenic removal from water by starch functionalized maghemite nano-adsorbents: Thermodynamics and kinetics investigations	2020

7649	R. Ventura-Houle	Groundwater arsenic contamination and their variations on episode of drought: Ter River delta in Catalonia, Spain	2018
7650	G. H. Li	Copper slag gel encapsulates sludge through encapsulation and precipitation in weakly acidic to strongly basic environments	2021
7651	N. Kolarova	Trace elements in aquatic environment. Origin, distribution, assessment and toxicity effect for the aquatic biota	2021
7652	L. Bartonova	Effect of CaO on retention of S, Cl, Br, As, Mn, V, Cr, Ni, Cu, Zn, W and Pb in bottom ashes from fluidized-bed coal combustion power station	2014
7653	S. Barakan	Thermodynamic, kinetic and equilibrium isotherm studies of As(V) adsorption by Fe(III)-impregnated bentonite	2020
7654	E. I. Jassim	CFD Modeling of Toxic Element Evolved During Coal Combustion	2015
7655	R. Lopez-Adams	Dissimilatory Fe(III) Reduction Controls on Arsenic Mobilization: A Combined Biogeochemical and NanoSIMS Imaging Approach	2021
7656	P. Tripathi	Arsenite stress variably stimulates pro-oxidant enzymes, anatomical deformities, photosynthetic pigment reduction, and antioxidants in arsenic-tolerant and sensitive rice seedlings	2015
7657	J. Zhang	Arsenic methylation by a genetically engineered Rhizobium-legume symbiont	2017
7658	X. J. Chen	Exploration of As(III)/As(V) Uptake from Aqueous Solution by Synthesized Calcium Sulfate Whisker	2014
7659	T. Bartsch	The quaternary arsenide oxides Ce9Au5-xAs8O6 and Pr9Au5-xAs8O6	2016
7660	P. Maziarz	Highly effective magnet-responsive LDH-Fe oxide composite adsorbents for As(V) removal	2019
7661	A. Karczewska	Transformation of beech forest litter as a factor that triggers arsenic solubility in soils developed on historical mine dumps	2018
7662	N. Inchaurrondo	Synthesis and adsorption behavior of mesoporous alumina and Fe-doped alumina for the removal of dominant arsenic species in contaminated waters	2019
7663	Y. F. Wei	Efficient removal of arsenic from groundwater using iron oxide nanoneedle array-decorated biochar fibers with high Fe utilization and fast adsorption kinetics	2019
7664	S. Garrido-Hoyos	Synthesis of Minerals with Iron Oxide and Hydroxide Contents as a Sorption Medium to Remove Arsenic from Water for Human Consumption	2016
7665	Y. Bentahar	Adsorptive properties of Moroccan clays for the removal of arsenic(V) from aqueous solution	2016
7666	D. Ghosh	Iron Slime: An Amendment in Mitigating Arsenic Accumulation in Rice	2022

7667	S. Hossain	Redox processes and occurrence of arsenic in a volcanic aquifer system of Kumamoto Area, Japan	2016
7668	S. A. Radosavljevic	Rujevac Sb-Pb-Zn-As polymetallic deposit, Boranja orefield, Western Serbia: native arsenic and arsenic mineralization	2014
7669	M. S. Nejad	Super-efficient removal of arsenic and mercury ions from wastewater by nanoporous biochar-supported poly 2-aminothiophenol	2022
7670	S. Fakhreddine	The effect of porewater ionic composition on arsenate adsorption to clay minerals	2021
7671	G. C. Velazquez-Pena	As(V) sorption by different natural zeolite frameworks modified with Fe, Zr and FeZr	2019
7672	S. X. Huang	Effects of exogenous substance treatment on arsenic accumulation in rice based on environmental protection concept	2020
7673	A. Saldana-Robles	Effects of the presence of organic matter on the removal of arsenic from groundwater	2018
7674	X. H. Feng	A Quantitative Model for the Coupled Kinetics of Arsenic Adsorption/Desorption and Oxidation on Manganese Oxides	2018
7675	C. Xiao	Impact process of the aquitard to regional arsenic accumulation of the underlying aquifer in Central Yangtze River Basin	2021
7676	L. C. Dalboni	Biological Actions, Electrical Conductance and Silicon-Containing Microparticles of Arsenicum Album Prepared in Plastic and Glass Vials	2019
7677	J. T. Tong	Arsenic contamination of the soil-wheat system irrigated with high arsenic groundwater in the Hetao Basin, Inner Mongolia, China	2014
7678	K. Kikuchi	Phosphorescent Metallacrown Ethers Enchained Through Coordination of Arsafluorene to Platinum(II) Dihalide	2021
7679	X. Ge	Hierarchical iron containing gamma-MnO2 hollow microspheres: A facile one-step synthesis and effective removal of As(III) via oxidation and adsorption	2016
7680	M. S. Podder	Sequestering of As(III) and As(V) from wastewater using a novel neem leaves/MnFe2O4 composite biosorbent	2016
7681	T. Kim	Mechanisms at Different pH for Stabilization of Arsenic in Mine Tailings Using Steelmaking Slag	2020
7682	C. Wang	Removal of As(III) and As(V) from aqueous solutions using nanoscale zero valent iron-reduced graphite oxide modified composites	2014
7683	R. Sitko	Green Approach for Ultratrace Determination of Divalent Metal Ions and Arsenic Species Using Total-Reflection X-ray Fluorescence Spectrometry and Mercapto-Modified Graphene Oxide Nanosheets as a Novel Adsorbent	2015
7684	G. Zhang	Porous Nanobimetallic Fe-Mn Cubes with High Valent Mn and Highly Efficient Removal of Arsenic(III)	2017

7685	M. Pal	Removing toxic contaminants from groundwater by graphene oxide nanocomposite in a membrane module under response surface optimization	2019
7686	K. Keser	Simple, Rapid and Sensitive Detection of Phenylarsine Oxide in Drinking Water Using Quartz Crystal Microbalance: A Novel Surface Functionalization Technique	2020
7687	J. Ding	Fabrication of Fe3O4@reduced graphene oxide composite via novel colloid electrostatic self-assembly process for removal of contaminants from water	2015
7688	R. Choudhury	The impact of Aquifer Flushing on Groundwater Arsenic Across a 35-Km Transect Perpendicular to the Upper Brahmaputra River in Assam, India	2018
7689	H. Y. Wang	Vertical redox zones of Fe-S-As coupled mineralogy in the sediments of Hetao Basin - Constraints for groundwater As contamination	2021
7690	X. H. Li	Electrocatalytical oxidation of arsenite by reduced graphene oxide via in-situ electrocatalytic generation of H2O2	2019
7691	S. Das	Modeling and Analysis of Electro-Thermal Impact of Crosstalk Induced Gate Oxide Reliability in Pristine and Intercalation Doped MLGNR Interconnects	2019
7692	E. N. Bakatula	The Removal of Arsenic and Uranium from Aqueous Solutions by Sorption onto Iron Oxide-Coated Zeolite (IOCZ)	2017
7693	P. Mondal	Behavior of As(V) with ZVI-H2O System and the Reduction to As(0)	2014
7694	M. H. Sadeghi	One-dimensional graphene for efficient aqueous heavy metal adsorption: Rapid removal of arsenic and mercury ions by graphene oxide nanoribbons (GONRs)	2020
7695	G. A. Kloster	Adsorption of arsenic onto films based on chitosan and chitosan/nano-iron oxide	2020
7696	S. Faiz-ul Islam	Reducing greenhouse gas emissions and grain arsenic and lead levels without compromising yield in organically produced rice	2020
7697	T. T. Li	Application of Exogenous Iron Alters the Microbial Community Structure and Reduces the Accumulation of Cadmium and Arsenic in Rice (Oryza sativa L.)	2022
7698	M. Lee	Sorption of Bioavailable Arsenic on Clay and Iron Oxides Elevates the Soil Microbial Activity	2020
7699	X. Guan	GaAs Core/SrTiO3 Shell Nanowires Grown by Molecular Beam Epitaxy	2016
7700	D. H. Kim	Heterogeneous Catalytic Oxidation of As(III) on Nonferrous Metal Oxides in the Presence of H2O2	2015
7701	L. H. Liu	Enhanced adsorption removal of arsenic from mining wastewater using birnessite under electrochemical redox reactions	2019
7702	R. Xu	Paper Simultaneous removal of antimony(III/V) and arsenic(III/V) from aqueous solution by bacteria-mediated kaolin@Fe-Mn binary (hydr) oxides composites	2022

7703	P. Ahmad	Sodium nitroprusside (SNP) improves tolerance to arsenic (As) toxicity in Vicia faba through the modifications of biochemical attributes, antioxidants, ascorbate-glutathione cycle and glyoxalase cycle	2020
7704	N. T. V. Hoan	Fe3O4/Reduced Graphene Oxide Nanocomposite: Synthesis and Its Application for Toxic Metal Ion Removal	2016
7705	P. T. Yang	Evolution of As speciation with depth in a soil profile with a geothermal As origin	2020
7706	G. Z. Kyzas	Graphene oxide and its application as an adsorbent for wastewater treatment	2014
7707	S. L. Yang	Hierarchical flowerlike magnesium oxide hollow spheres with extremely high surface area for adsorption and catalysis	2016
7708	S. L. Zhang	Sulfidization of As(V)-containing schwertmannite and its impact on arsenic mobilization	2016
7709	F. H. Li	Evaporation-induced self-assembly (EISA) synthesized mesoporous bimetallic oxides (MBOs) enabling enhanced co-uptake of arsenate and fluoride from water	2019
7710	L. Q. Zhang	Study on the Mineralogical and Geochemical Characteristics of Arsenic in Permian Coals: Focusing on the Coalfields of Shanxi Formation in Northern China	2022
7711	Q. T. Shi	Arsenic Adsorption on Lanthanum-Impregnated Activated Alumina: Spectroscopic and DFT Study	2015
7712	X. Yang	Remediation of As-contaminated soils using citrate extraction coupled with electrochemical removal	2022
7713	B. V. Yesudhason	Exploiting the unique phenotypes of the earthworm Eudrilus eugeniae to evaluate the toxicity of chemical substances	2018
7714	C. Pandey	Impact of silicon on Indian mustard (Brassica juncea L.) root traits by regulating growth parameters, cellular antioxidants and stress modulators under arsenic stress	2016
7715	C. Osorio-Yanez	The ADMA/DDAH/NO pathway in human vein endothelial cells exposed to arsenite	2017
7716	K. Wrighton-Araneda	Removal of water-soluble inorganic arsenicals with phosphorene oxide nanoadsorbents: A first-principles study	2021
7717	W. Kanoua	Hydrochemical evolution and arsenic release in shallow aquifer in the Titas Upazila, Eastern Bangladesh	2017
7718	S. G. Sia	Geochemistry of Trace Elements as One of the Important Coal Quality Parameter: An Example from Balingian Coal, Malaysia	2017
7719	M. Simon	Are soil amendments able to restore arsenic-contaminated alkaline soils?	2015
7720	W. Zhang	Aptamer-functionalized screen-printed electrode coupled with graphene oxide and methylene blue nanocomposite as enhanced signal label for total arsenic determination in shellfish	2021

7721	H. Ahmad	Bioinspired 2D carbon sheets decorated with MnFe2O4 nanoparticles for preconcentration of inorganic arsenic, and its determination by ICP-OES	2019
7722	F. Melak	Evaluation of natural quartz and zeolitic tuffs for As(V) removal from aqueous solutions: a mechanistic approach	2018
7723	T. T. Jia	Impacts of crystal orientation of GaAs on the interfacial structures and electrical properties of Hf0.6La0.4Ox films	2014
7724	N. Sobolev	Iodine and bromine in fish consumed by indigenous peoples of the Russian Arctic	2020
7725	B. V. Sladkopevtsev	Investigation of the Current-Voltage Characteristics of New MnO2/GaAs(100) and V2O5/GaAs(100) Heterostructures Subjected to Heat Treatment	2019
7726	Y. Luo	Graphite felt incorporated with MoS2/rGO for electrochemical detoxification of high-arsenic fly ash	2020
7727	M. A. Gomez-Gonzalez	Iron oxide - clay composite vectors on long-distance transport of arsenic and toxic metals in mining-affected areas	2018
7728	J. Sun	Arsenic mobilization from iron oxides in the presence of oxalic acid under hydrodynamic conditions	2018
7729	M. Vigeh	Hair Metal Levels and Childhood Weight Gain	2020
7730	M. Hori	Detection of arsenic donor electrons using gate-pulse-induced spin-dependent recombination in silicon transistors	2021
7731	Y. C. Du	FeOOH-MnO2/Sepiolite and Fe2O3-MnO2/Diatomite: Highly efficient adsorbents for the removal of As(V)	2022
7732	A. Adamescu	Density functional theory calculations on the adsorption of monomethylarsonic acid onto hydrated iron (oxyhydr)oxide clusters	2017
7733	A. Lock	Validation of an updated fractionation and indirect speciation procedure for inorganic arsenic in oxic and suboxic soils and sediments	2016
7734	S. S. Wang	Manganese oxide-modified biochars: Preparation, characterization, and sorption of arsenate and lead	2015
7735	M. Kimura	Synthesis, structures, and some reactions of (thioacyl)thio - and (acylseleno)antimony and -bismuth derivatives ((RCSS)(x)MR3-x1 (RCOSe)(x)MR3-x1 with M=Sb, Bi and x=1-3)	2006
7736	T. Kato	In-situ Iodination of Organoarsenic Homocycles: Facile Synthesis of 9-Arsafluorene	2015
7737	K. Z. Benis	Treatment of aqueous arsenic - A review of biosorbent preparation methods	2020
7738	M. Modheji	Efficient pre-concentration of As(III) in food samples using guanidine-modified magnetic mesoporous silica	2020

7739	G. Bhanjana	Robust and direct electrochemical sensing of arsenic using zirconia nanocubes	2016
7740	B. Casentini	Mining Rock Wastes for Water Treatment: Potential Reuse of Fe- and Mn-Rich Materials for Arsenic Removal	2019
7741	Y. K. Sun	Effect of Weak Magnetic Field on Arsenate and Arsenite Removal from Water by Zerovalent Iron: An XAFS Investigation	2014
7742	R. N. Gow	Spectroelectrochemistry of enargite III: Alkaline sulfide leaching	2015
7743	Z. H. Zhao	Real-Time Monitoring of Arsenic Trioxide Release and Delivery by Activatable T-1 Imaging	2015
7744	Y. Y. Yin	Iron and manganese oxides modified maize straw to remove tylosin from aqueous solutions	2018
7745	S. S. Salih	Competitive adsorption of As(III) and As(V) onto chitosan/diatomaceous earth adsorbent	2019
7746	M. M. Rahman	Exploratory experiments to determine the effect of alternative operations on the efficiency of subsurface arsenic removal in rural Bangladesh	2015
7747	L. N. Lin	Mitigating arsenic accumulation in rice (Oryza sativaL.) using Fe-Mn-La-impregnated biochar composites in arsenic-contaminated paddy soil	2020
7748	H. T. Ren	Oxalic Acid-Induced Photodissolution of Ferrihydrite and the Fate of Loaded As(V): Kinetics and Mechanism	2019
7749	P. K. Mishra	Surfactant-Free One-Pot Synthesis of Low-Density Cerium Oxide Nanoparticles for Adsorptive Removal of Arsenic Species	2018
7750	R. P. Mock	Influence of Fe(II) on Arsenic(III) Oxidation by Birnessite in Diffusion-Limited Systems	2019
7751	B. F. Urbano	Cationic polymer-TiO2 nanocomposite sorbent for arsenate removal	2015
7752	C. Garcia-Gomez	Electrocoagulated Metal Hydroxide Sludge for Fluoride and Arsenic Removal in Aqueous Solution: Characterization, Kinetic, and Equilibrium Studies	2016
7753	J. A. Sigrist	ATR-IR spectroelectrochemical studies of arsenic speciation at the ferrihydrite-solution interface	2019
7754	X. H. Sun	Flotation Depression of Arsenopyrite Using Sodium Nitrobenzoate under Alkaline Conditions	2021
7755	R. Schouwenaars	Removal of arsenic III and V from laboratory solutions and contaminated groundwater by metallurgical slag through anion-induced precipitation	2017
7756	J. Bai	Release of arsenic and iron in aquifer to groundwater under the variation of REDOX environment during bank infiltration: a case study in Huangjia groundwater source area, Northeastern China	2019

7757	J. H. Lee	Thermodynamic Modeling of the Na2O-SiO2-As(2)O(5)System and Its Application to Arsenic Immobilization Using Glass Formation	2020
7758	R. Liu	Low temperature synthesized ultrathin gamma-Fe2O3 nanosheets show similar adsorption behaviour for As(III) and As(V)	2016
7759	Y. H. Li	Promotion effect of KMnO4 on the oxidation of As(III) by air in alkaline solution	2014
7760	T. H. Fang	Arsenic speciation and diffusion flux in Danshuei Estuary sediments, Northern Taiwan	2015
7761	F. Aprile	A Large Tn7-like Transposon Confers Hyperresistance to Copper in Pseudomonas syringae pv. syringae	2021
7762	C. Feng	Removal of arsenic from alkaline process waters of gold cyanidation by use of gamma-Fe2O3@ZrO2 nanosorbents	2017
7763	A. S. Bailey	Geochemical characterization of dust from arsenic-bearing tailings, Giant Mine, Canada	2021
7764	M. J. Lopez-Munoz	Removal of As(III) from aqueous solutions through simultaneous photocatalytic oxidation and adsorption by TiO2 and zero-valent iron	2017
7765	Y. J. Han	Preparation of Cu-Y binary oxysulfide and its application in the removal of arsenic from aqueous solutions	2019
7766	R. Hallaj	Magnetically induced catalytic electrooxidation of As(III) on GC modified Fe@Cu-BTC MOF nanoparticles: Application for determination of As(III)	2022
7767	R. Ettlinger	In Vitro Studies of Fe3O4-ZIF-8 Core-Shell Nanoparticles Designed as Potential Theragnostics	2020
7768	G. B. Chen	Synthesis of Pure Micro- and Nanopyrite and Their Application for As (III) Removal from Aqueous Solution	2016
7769	I. Jacukowicz-Sobala	Evaluation of ferromagnetic hybrid polymers obtained using cation exchangers	2015
7770	H. J. Han	Metal arsenic mediated enhancement of type-2 immunity in brains with altered locomotive activities in mice with autism-like behavioral characteristics	2022
7771	R. M. Dhoble	Removal of arsenic(III) from water by magnetic binary oxide particles (MBOP): Experimental studies on fixed bed column	2017
7772	C. Tikka	Immune disruption occurs through altered gut microbiome and NOD2 in arsenic induced mice: Correlation with colon cancer markers	2020
7773	P. Tangviroon	Modeling and Evaluating the Performance of River Sediment on Immobilizing Arsenic from Hydrothermally Altered Rock in Laboratory Column Experiments with Hydrus-1D	2017
7774	J. Kim	Chemical and toxicological assessment of arsenic sorption onto Fe-sericite composite powder and beads	2018

7775	H. P. S. Makkar	Seaweeds for livestock diets: A review	2016
7776	H. Vojoudi	Post-modification of nanoporous silica type SBA-15 by bis(3-triethoxysilylpropyl)tetrasulfide as an efficient adsorbent for arsenic removal	2017
7777	P. E. Raynal	GaAs WET and Siconi Cleaning Sequences for an Efficient Oxide Removal	2019
7778	W. C. Su	Manganese-doped ferrihydrite/cellulose/polyvinyl alcohol composite membrane: Easily recyclable adsorbent for simultaneous removal of arsenic and cadmium from soil	2022
7779	A. Mohamed	Surface functionalized composite nanofibers for efficient removal of arsenic from aqueous solutions	2017
7780	D. Cortes-Arriagada	A theoretical investigation of the removal of methylated arsenic pollutants with silicon doped graphene	2016
7781	Q. T. Shi	Adsorption of arsenate on lanthanum-impregnated activated alumina: In situ ATR-FTIR and two-dimensional correlation analysis study	2015
7782	T. T. Luo	Arsenite (III) removal via manganese-decoration on cellulose nanocrystal -grafted polyethyleneimine nanocomposite	2022
7783	B. Sun	Preparation and evaluation of granular Fe-impregnated attapulgite adsorbents (Fe-ATP) for arsenic removal from contaminated groundwater	2019
7784	S. G. Johnston	Fire Promotes Arsenic Mobilization and Rapid Arsenic(III) Formation in Soil via Thermal Alteration of Arsenic-Bearing Iron Oxides	2019
7785	F. M. Kusin	Geo-ecological evaluation of mineral, major and trace elemental composition in waste rocks, soils and sediments of a gold mining area and potential associated risks	2019
7786	G. X. Huang	Changes of arsenic fractionation and bioaccessibility in wastewater-irrigated soils as a function of aging: Influence of redox condition and arsenic load	2016
7787	M. V. Miljkovic	Remediation of arsenic contaminated water by a novel carboxymethyl cellulose bentonite adsorbent	2019
7788	K. Huang	N-Hydroxyarylamine O-Acetyltransferases Catalyze Acetylation of 3-Amino-4-Hydroxyphenylarsonic Acid in the 4-Hydroxy-3-Nitrobenzenearsonic Acid Transformation Pathway of Enterobacter sp. Strain CZ-1	2020
7789	V. Smolikova	Development and evaluation of the iron oxide-hydroxide based resin gel for the diffusive gradient in thin films technique	2020
7790	P. C. S. Carvalho	Human health risks in an old gold mining area with circum-neutral drainage, central Portugal	2017
7791	F. F. Tafti	A simple and straightforward combination of surfactant-assisted magnetic dispersive micro-solid-phase extraction and hydride generation procedure to determine arsenic (III) species in environmental, biological, and fruit juice samples	2022
7792	J. Saiz	Functionalized magnetic nanoparticles as new adsorption materials for arsenic removal from polluted waters	2014

7793	A. R. Gliga	Prenatal arsenic exposure is associated with increased plasma IGFBP3 concentrations in 9-year-old children partly via changes in DNA methylation	2018
7794	K. M. Nguyen	Adsorption of Arsenic and Heavy Metals from Solutions by Unmodified Iron-Ore Sludge	2019
7795	W. J. Shen	Effect of divalent manganese ions on the roxarsone removal efficiency from aqueous solution with iron oxide	2022
7796	E. Stopelli	Spatial and temporal evolution of groundwater arsenic contamination in the Red River delta, Vietnam: Interplay of mobilisation and retardation processes	2020
7797	S. Lata	As(V) removal using biochar produced from an agricultural waste and prediction of removal efficiency using multiple regression analysis	2019
7798	M. Usman	Performance Evaluation of Small Sized Powdered Ferric Hydroxide as Arsenic Adsorbent	2018
7799	L. Z. Chen	Health risks associated with multiple metal(loid)s in groundwater: A case study at Hetao Plain, northern China	2020
7800	Y. H. Wu	Optimizing adsorption of arsenic(III) by NH2-MCM-41 using response surface methodology	2014
7801	J. Perez	Arsenic sorption onto an aluminum oxyhydroxide-poly (4-vinylbenzyl)trimethylammonium chloride hybrid sorbent	2016
7802	A. Praveen	Nitric oxide confronts arsenic stimulated oxidative stress and root architecture through distinct gene expression of auxin transporters, nutrient related genes and modulates biochemical responses in Oryza sativa L	2018
7803	M. S. Aksenov	Effect of fluorine on the electrical properties of anodic oxide/InAs(111)A interface	2014
7804	S. J. Doherty	Evaluation of amendments to reduce arsenic and antimony leaching from co-contaminated soils	2017
7805	M. Senila	Preliminary investigation on arsenic fractionation in soil from ogosta river floodplain using a seven-step extraction procedure	2016
7806	J. Z. Wu	A novel calcium-based magnetic biochar reduces the accumulation of As in grains of rice (Oryza sativa L.) in As-contaminated paddy soils	2020
7807	Y. Li	Efficient removal of As(III) via simultaneous oxidation and adsorption by magnetic sulfur-doped Fe-Cu-Y trimetal oxide nanoparticles	2020
7808	A. E. Tufo	Arsenic adsorption onto aluminium-substituted goethite	2016
7809	O. Rady	Adsorption and catalytic oxidation of arsenite on Fe-Mn nodules in the presence of oxygen	2020
7810	M. Wada	Two-step synthesis of triarylmetals (As, Sb, Bi) starting from the metal oxides and 2,6-dimethoxybenzenethiol	1997

7811	S. F. Wang	The effect of microbial sulfidogenesis on the stability of As-Fe coprecipitate with low Fe/As molar ratio under anaerobic conditions	2016
7812	W. Cabrera	Diffusion of In0.53Ga0.47As elements through hafnium oxide during post deposition annealing	2014
7813	K. Li	TRIB3 Promotes APL Progression through Stabilization of the Oncoprotein PML-RAR alpha and Inhibition of p53-Mediated Senescence	2017
7814	B. Song	Retention of arsenic in coal combustion flue gas at high temperature in the presence of CaO	2020
7815	L. F. Jin	Preparation of magnetic Fe3O4@Cu/Ce microspheres for efficient catalytic oxidation co-adsorption of arsenic(III)	2020
7816	S. Sklari	Ceramic membrane materials and process for the removal of as(iii)/as(v) ions from water	2015
7817	Z. F. He	Simultaneous remediation of As(III) and dibutyl phthalate (DBP) in soil by a manganese-oxidizing bacterium and its mechanisms	2019
7818	Y. A. Wang	Dynamic arsenic aging processes and their mechanisms in nine types of Chinese soils	2017
7819	I. Jacukowicz-Sobala	Sorption of arsenates(III) and (V) on a hybrid iron and manganese oxides polymer-containing made by modification of a strong-base anion exchanger	2014
7820	M. K. Tripathi	Arsenic alters nitric oxide signaling similar to autism spectrum disorder and Alzheimer's disease-associated mutations	2022
7821	L. Costa	Effects of Bioirrigation and Salinity on Arsenic Distributions in Ferruginous Concretions from Salt Marsh Sediment Cores (Southern Brazil)	2021
7822	D. K. Tripathi	Silicon induces adventitious root formation in rice under arsenate stress with involvement of nitric oxide and indole-3-acetic acid	2021
7823	J. Baruah	Enhancing arsenic sequestration on ameliorated waste molasses nanoadsorbents using response surface methodology and machine-learning frameworks	2021
7824	Z. Wang	High arsenic groundwater in the Guide basin, northwestern China: Distribution and genesis mechanisms	2018
7825	C. Yu	Variations and driving mechanism of dissolved arsenic in sediment porewater near wetland	2022
7826	S. Zhang	Mechanisms of efficient As(III) and As(V) removal by Ni-coprecipitated hausmannite nanocomposites	2022
7827	G. Zhao	Electrochemical Deposition of Gold Nanoparticles on Reduced Graphene Oxide by Fast Scan Cyclic Voltammetry for the Sensitive Determination of As(III)	2019
7828	L. Xu	A simple and novel method to enhance As (V) removal by zero valent iron and activated iron media through air injection at intervals	2019

7829	J. H. Guo	Synthesis of a novel ternary HA/Fe-Mn oxides-loaded biochar composite and its application in cadmium(II) and arsenic(V) adsorption	2019
7830	T. T. Chen	Influences of Simulated Organic Residues in Petroleum-Exploiting Areas on the Dissolution and Speciation of Arsenic in Soil-Mineral Solid	2020
7831	J. X. Fan	Effect of iron oxide reductive dissolution on the transformation and immobilization of arsenic in soils: New insights from X-ray photoelectron and X-ray absorption spectroscopy	2014
7832	S. Thanawatpoontawee	Iron-loaded zein beads as a biocompatible adsorbent for arsenic(V) removal	2016
7833	S. H. Chi	Magnetically Separated meso-g-C3N4/Fe3O4: Bifuctional Composites for Removal of Arsenite by Simultaneous Visible-Light Catalysis and Adsorption	2016
7834	M. J. Ying	The structure, optical and magnetic properties of arsenic implanted ZnO films prepared by molecular beam epitaxy	2016
7835	H. W. Wang	Simultaneous removal of tetracycline hydrochloride and As(III) using poorly-crystalline manganese dioxide	2015
7836	Z. H. Ren	Effect of Selenium on Brain Injury in Chickens with Subacute Arsenic Poisoning	2022
7837	L. Jurkovic	Natural attenuation of antimony and arsenic in soils at the abandoned Sb-deposit Poproc, Slovakia	2019
7838	M. B. Shakoor	The evaluation of arsenic contamination potential, speciation and hydrogeochemical behaviour in aquifers of Punjab, Pakistan	2018
7839	J. S. Mankar	Molecularly imprinted nanoparticles (nanoMIPs): an efficient new adsorbent for removal of arsenic from water	2020
7840	P. Sherugar	Fabrication of zinc doped aluminium oxide/polysulfone mixed matrix membranes for enhanced antifouling property and heavy metal removal	2021
7841	S. L. Shumlas	Oxidation of arsenite to arsenate on birnessite in the presence of light	2016
7842	K. F. Pi	Multilevel hydrogeochemical monitoring of spatial distribution of arsenic: A case study at Datong Basin, northern China	2016
7843	J. W. Stuckey	Delineating the Convergence of Biogeochemical Factors Responsible for Arsenic Release to Groundwater in South and Southeast Asia	2016
7844	W. B. Postek	Electrocatalytic effects during redox reactions of arsenic at platinum nanoparticles in acid medium: Possibility of preconcentration, electroactive film formation, and detection of As(III) and As(V)	2019
7845	Q. Zheng	Insights into the underlying mechanisms of stability working for As(III) removal by Fe-Mn binary oxide as a highly efficient adsorbent	2021
7846	E. Solorzano	Reactive Oxygen Species (ROS) Metabolism and Nitric Oxide (NO) Content in Roots and Shoots of Rice (Oryza sativa L.) Plants under Arsenic-Induced Stress	2020

7847	A. Ghosh	Calcium ion incorporated hydrous iron(III) oxide: synthesis, characterization, and property exploitation towards water remediation from arsenite and fluoride	2019
7848	K. Ehlert	Impact of Birnessite on Arsenic and Iron Speciation during Microbial Reduction of Arsenic-Bearing Ferrihydrite	2014
7849	A. M. A. Pintor	Establishing the state-of-the-art on the adsorption of coexisting pnictogens in water: A literature review	2022
7850	J. P. H. Perez	The interfacial reactivity of arsenic species with green rust sulfate (GR(SO4))	2019
7851	Y. Yu	An innovative yttrium nanoparticles/PVA modified PSF membrane aiming at decontamination of arsenate	2018
7852	I. R. McFarlane	Field effect transistors based on semiconductive microbially synthesized chalcogenide nanofibers	2015
7853	M. Banerjee	Dynamic alteration in miRNA and mRNA expression profiles at different stages of chronic arsenic exposure-induced carcinogenesis in a human cell culture model of skin cancer	2021
7854	E. K. Chiwandik a	Volatilization Behavior of Arsenic from a Hematite Ore During Non-isothermal Heating in Argon Atmosphere	2022
7855	L. Cacciabue	Processes that control the dissolution of loess sediments and contribution of arsenic release in the Chaco-Pampean plain, Argentina	2022
7856	A. R. Gliga	Maternal exposure to cadmium during pregnancy is associated with changes in DNA methylation that are persistent at 9 years of age	2022
7857	C. Foti	Recent Advances of Graphene-Based Strategies for Arsenic Remediation	2020
7858	A. Zialame	Stabilized magnetite nanoparticles for the remediation of arsenic contaminated soil	2021
7859	E. C. Gillispie	Chemical controls on abiotic and biotic release of geogenic arsenic from Pleistocene aquifer sediments to groundwater	2016
7860	X. S. Su	Redox zonation for different groundwater flow paths during bank filtration: a case study at Liao River, Shenyang, northeastern China	2018
7861	Z. J. Zhao	Effects of dam construction on arsenic mobility and transport in two large rivers in Tibet, China	2020
7862	M. Naeem	Salicylic acid restrains arsenic induced oxidative burst in two varieties of Artemisia annua L. by modulating antioxidant defence system and artemisinin production	2020
7863	J. Z. Wu	Remediation of As(III) and Cd(II) co-contamination and its mechanism in aqueous systems by a novel calcium-based magnetic biochar	2018
7864	Y. Tang	Reducing arsenic toxicity using the interfacial oxygen nanobubble technology for sediment remediation	2021

7865	M. A. Hashem	Hair burning and liming in tanneries is a source of pollution by arsenic, lead, zinc, manganese and iron	2017
7866	C. B. Wenk	Factors affecting arsenic and uranium removal with zerovalent iron: laboratory tests with Kanchan-type iron nail filter columns with different groundwaters	2014
7867	L. Trakal	AMOchar: Amorphous manganese oxide coating of biochar improves its efficiency at removing metal(loid)s from aqueous solutions	2018
7868	N. J. Raju	Arsenic in the geo-environment: A review of sources, geochemical processes, toxicity and removal technologies	2022
7869	L. Q. Duan	Dynamics of arsenic and its interaction with Fe and S at the sediment-water interface of the seasonal hypoxic Changjiang Estuary	2021
7870	K. Turunen	Factors Controlling the Migration of Tailings-Derived Arsenic: A Case Study at the Yara Siilinjarvi Site	2016
7871	N. V. Thinh	Biogeochemical properties and potential risk of shallow arsenic-rich sediment layers to groundwater quality in Western Bangladesh	
7872	J. Ai	Catalytic pyrolysis coupling to enhanced dewatering of waste activated sludge using KMnO4-Fe(II) conditioning for preparing multi-functional material to treat groundwater containing combined pollutants	2019
7873	P. C. Lin	Nanoplastics aggravate the toxicity of arsenic to AGS cells by disrupting ABC transporter and cytoskeleton	2021
7874	S. T. Li	Dual-functional Sites for Selective Adsorption of Mercury and Arsenic ions in SnS4 (4-)/MgFe-LDH from Wastewater	2021
7875	M. Asgher	Hydrogen peroxide modulates activity and expression of antioxidant enzymes and protects photosynthetic activity from arsenic damage in rice (Oryza sativa L.)	2021
7876	T. Gougousi	Low-Temperature Dopant-Assisted Crystallization of HfO2 Thin Films	2021
7877	M. E. Lee	Adsorption characteristics of arsenic and phosphate onto iron impregnated biochar derived from anaerobic granular sludge	2018
7878	T. T. Zhang	Efficient As(III) removal directly as basic iron arsenite by in-situ generated Fe(III) hydroxide from ferrous sulfate on the surface of CaCO3	2019
7879	C. E. Schuh	Solid-phase speciation and post-depositional mobility of arsenic in lake sediments impacted by ore roasting at legacy gold mines in the Yellowknife area, Northwest Territories, Canada	2018
7880	M. A. Sandoval	Arsenic and fluoride removal by electrocoagulation process: A general review	2021
7881	L. B. Liao	Using Ionic Liquid Modified Zeolite as a Permeable Reactive Wall to Limit Arsenic Contamination of a Freshwater Lake-Pilot Tests	2018
7882	K. Nakakubo	Dithiocarbamate-modified cellulose resins: A novel adsorbent for selective removal of arsenite from aqueous media	2019

7883	H. Y. Luan	Adsorptive filtration of As(III) from drinking water by CuFe2O4 particles embedded in carbon nanotube membranes	2019
7884	X. P. Huang	Facet-dependent contaminant removal properties of hematite nanocrystals and their environmental implications	2018
7885	L. G. Zhang	Arsenic Removal and Recovery of Germanium and Tungsten in Toxic Coal Fly Ash from Lignite by Vacuum Distillation with a Sulfurizing Reagent	2021
7886	V. Chandrakar	Carbon dot induces tolerance to arsenic by regulating arsenic uptake, reactive oxygen species detoxification and defense-related gene expression in Cicer arietinum L	2020
7887	D. D. La	Effective removal of Pb(II) using a graphene@ternary oxides composite as an adsorbent in aqueous media	2017
7888	Y. J. Zhao	Enhanced adsorption of As(V) from aqueous solution by mesoporous goethite: kinetics, isotherms, thermodynamics, and mechanism	2020
7889	Y. L. Wang	Lanthanum hydroxide: a highly efficient and selective adsorbent for arsenate removal from aqueous solution	2020
7890	W. Gustave	Arsenic mitigation in paddy soils by using microbial fuel cells	2018
7891	L. Guo	Insight into the role and mechanism of polysaccharide in polymorphous magnesium oxide nanoparticle synthesis for arsenate removal	2022
7892	X. G. Hu	Graphene oxide amplifies the phytotoxicity of arsenic in wheat	2014
7893	V. Manirethan	Adsorptive removal of trivalent and pentavalent arsenic from aqueous solutions using iron and copper impregnated melanin extracted from the marine bacterium Pseudomonas stutzeri	2020
7894	K. Acuna	Photocatalytic degradation of roxarsone by using synthesized ZnO nanoplates	2017
7895	R. Attinti	Adsorption of arsenic(V) from aqueous solutions by goethite/silica nanocomposite	2015
7896	M. Cable	A study of refining. Part 3: The function of arsenious oxide during melting and refining	2016
7897	I. Kurganskaya	A modeling approach for unveiling adsorption of toxic ions on iron oxide nanocrystals	2021
7898	S. Ramanathan	Electrochemical Detection of Trace Amounts of Arsenic (III) in Poultry Using a Graphene Oxide-Bis(2-(4,5-diphenyl-1H-imidazol-2-yl)phenoxy)Cobalt Composite Modified Electrode	2019
7899	A. R. Kampf	Cuatrocapaite-(NH4) and cuatrocapaite-(K), two new minerals from the Torrecillas mine, Iquique Province, Chile, related to lucabindiite and gajardoite	2019
7900	R. Al-Gaashani	Preparation and properties of novel activated carbon doped with aluminum oxide and silver for water treatment	2021

7901	J. Tapia	The solid-state partitioning, distribution, and mineralogical associations of arsenic and antimony: Integrated findings from the Altiplano Puna, South America and international comparisons	2022
7902	T. Li	Detoxification and selective separation of Cr(VI) and As(III) in wastewater based on interfacial coupling in BiOBr with {110} facet under visible-light irradiation	2022
7903	D. J. Zhang	Schwertmannite modified with ethanol: A simple and feasible method for improving As(III) adsorption capacity	2022
7904	A. Ahmad	Mechanisms of arsenate removal and membrane fouling in ferric based coprecipitation-low pressure membrane filtration systems	2020
7905	S. N. Lundgren	Maternal diet during pregnancy is related with the infant stool microbiome in a delivery mode-dependent manner	2018
7906	E. Martin	Epigenetics and Preeclampsia: Defining Functional Epimutations in the Preeclamptic Placenta Related to the TGF-beta Pathway	2015
7907	T. V. Maltseva	Composite anion-exchangers modified with nanoparticles of hydrated oxides of multivalent metals	2019
7908	A. Jimana	Voltammetric Analysis of As(III) at a Cobalt Nanoparticles/Reduced Graphene Oxide Modified Exfoliated Graphite Electrode	2018
7909	J. Yondonjamts	Geochemical source and dispersion of copper, arsenic, lead, and zinc in the topsoil from the vicinity of Erdenet mining area, Mongolia	2019
7910	J. Liu	Physiological Effects of Copper Oxide Nanoparticles and Arsenic on the Growth and Life Cycle of Rice (Oryza sativa japonica 'Koshihikari')	2018
7911	M. Kuzu	Inhibitory properties of some heavy metals on carbonic anhydrase I and II isozymes activities purified from Van Lake fish (Chalcalburnus Tarichi) gill	2018
7912	C. Kaya	Sodium hydrosulfite together with silicon detoxifies arsenic toxicity in tomato plants by modulating the AsA-GSH cycle	2022
7913	A. E. Evans	Indicator of redox in soil (IRIS) films as a water management tool for rice farmers	2021
7914	G. S. Zhang	Respective Role of Fe and Mn Oxide Contents for Arsenic Sorption in Iron and Manganese Binary Oxide: An X-ray Absorption Spectroscopy Investigation	2014
7915	G. S. Du	A label-free colorimetric progesterone aptasensor based on the aggregation of gold nanoparticles	2016
7916	A. R. Kampf	Gajardoite, KCa0.5As43+O6Cl2 center dot 5H(2)O, a new mineral related to lucabindiite and torrecillasite from the Torrecillas mine, Iquique Province, Chile	2016
7917	Y. B. Peng	Smart Human-Serum-Albumin-As2O3 Nanodrug with Self-Amplified Folate Receptor-Targeting Ability for Chronic Myeloid Leukemia Treatment	2017
7918	Y. Okamoto	Global DNA Methylation in Cord Blood as a Biomarker for Prenatal Lead and Antimony Exposures	2022

7919	J. H. Tan	Chemical characteristics and source apportionment of PM2.5 in Lanzhou, China	2017
7920	M. A. Gabris	Chitosan magnetic graphene grafted polyaniline doped with cobalt oxide for removal of Arsenic(V) from water	2022
7921	R. F. Al-Thani	Perspectives of future water sources in Qatar by phytoremediation: biodiversity at ponds and modern approach	2021
7922	N. Iszatt	Environmental toxicants in breast milk of Norwegian mothers and gut bacteria composition and metabolites in their infants at 1 month	2019
7923	M. Benchehima	First-principles calculations of the structural and optoelectronic properties of BSb1-x As-x ternary alloys in zinc blende structure	2017
7924	S. K. Sahoo	In-situ functionalization of GO sheets with AlOOH-FeOOH composite nanorods: An eco-friendly nanoadsorbent for removal of toxic arsenate ions from water	2019
7925	S. H. Do	As3+ removal by Ca-Mn-Fe3O4 with and without H2O2: Effects of calcium oxide in Ca-Mn-Fe3O4	2014
7926	Z. J. Zeng	Early-life exposure to widespread environmental toxicants and children's health risks: A focus on the post-vaccination antibody potency or immunoglobulin levels	2021
7927	R. B. Brennan	Management of landfill leachate: The legacy of European Union Directives	2016
7928	R. Flores-Ramirez	Assessment of exposure to mixture pollutants in Mexican indigenous children	2016
7929	C. Okio	Systematics of boron halide complexes with dichalcogenoether ligands - Synthesis, structures and reaction chemistry	2018
7930	G. Schoeters	Internal exposure of Flemish teenagers to environmental pollutants: Results of the Flemish Environment and Health Study 2016-2020 (FLEHS IV)	2022
7931	R. T. Kapoor	Nitric oxide and spermidine alleviate arsenic-incited oxidative damage in Cicer arietinum by modulating glyoxalase and antioxidant defense system	
7932	A. Ali	Different cropping systems regulate the metabolic capabilities and potential ecological functions altered by soil microbiome structure in the plastic shed mono-cropped cucumber rhizosphere	2021
7933	S. Nakaya	Forms of trace arsenic, cesium, cadmium, and lead transported into river water for the irrigation of Japanese paddy rice fields	2018
7934	J. Pumarega	Timing of Toenail Collection and Concentrations of Metals in Pancreatic Cancer. Evidence Against Disease Progression Bias	
7935	M. Peters	Geochemical and multiple stable isotope (N, O, S) investigation on tap and bottled water from Beijing, China	2015
7936	Madhu	Exploration of glutathione reductase for abiotic stress response in bread wheat (Triticum aestivum L.)	2022

7937	T. Pollock	Trends in environmental chemical concentrations in the Canadian population: Biomonitoring data from the Canadian Health Measures Survey 2007-2017	2021
7938	U. G. C. Bandara	Arsenic-rich shallow groundwater in sandy aquifer systems buffered by rising carbonate waters: A geochemical case study from Mannar Island, Sri Lanka	2018
7939	L. Stolze	Surface complexation reactions in sandy porous media: Effects of incomplete mixing and mass-transfer limitations in flow-through systems	2022
7940	M. B. Fox	Measurement and modeling of proton-induced reactions on arsenic from 35 to 200 MeV	2021
7941	I. Shiue	Urinary polyaromatic hydrocarbons are associated with adult celiac disease and kidney stones: USA NHANES, 2011-2012	2016
7942	J. F. Teng	Polyphyllin VI, a saponin from Trillium tschonoskii Maxim: induces apoptotic and autophagic cell death via the ROS triggered mTOR signaling pathway in non-small cell lung cancer	2019
7943	M. Hendrikse	Treatment of oil sands process-affected waters using a pilot-scale hybrid constructed wetland	2018
7944	A. Sommella	Nature and reactivity of layered double hydroxides formed by coprecipitating Mg, Al and As(V): Effect of arsenic concentration, pH, and aging	2015
7945	N. S. Alsaiari	Polyethylenimine-Modified Magnetic Chitosan for the Uptake of Arsenic from Water	2021
7946	N. U. A. Babar	Highly Sensitive and Selective Detection of Arsenic Using Electrogenerated Nanotextured Gold Assemblage	2019
7947	H. Goyal	Life cycle assessment (LCA) of the arsenic and fluoride removal from groundwater through adsorption and electrocoagulation: A comparative study	2022
7948	K. C. Freel	Effect of arsenite and growth in biofilm conditions on the evolution of Thiomonas sp. CB2	2020
7949	S. Ghosh	Performance of -aluminium oxide nanoparticles for arsenic removal from groundwater	2019
7950	H. W. Chen	Adsorptive properties of alluvial soil for arsenic(V) and its potential for protection of the shallow groundwater among Changsha, Zhuzhou, and Xiangtan cities, China	2017
7951	S. F. Wu	Evaluation of toxic effects induced by arsenic trioxide or/and antimony on autophagy and apoptosis in testis of adult mice	2021
7952	Y. Yao	The Fe3O4-modified biochar reduces arsenic availability in soil and arsenic accumulation in indica rice (Oryza sativa L.)	2021
7953	M. Kumar	Concentrations of inorganic arsenic in groundwater, agricultural soils and subsurface sediments from the middle Gangetic plain of Bihar, India	2016
7954	R. Kumar	Novel insights into adsorption of heavy metal ions using magnetic graphene composites	2021

7955	Z. H. Li	Distribution and Risk Assessment of Toxic Pollutants in Surface Water of the Lower Yellow River, China	2021
7956	H. M. Mahmoud	Pigments and plasters from the Roman temple of Deir El-Hagar, Dakhla Oasis, Egypt: vibrational spectroscopic characterization	2019
7957	X. S. Cao	Interaction of graphene oxide with co-existing arsenite and arsenate: Adsorption, transformation and combined toxicity	2019
7958	T. Nur	Removing arsenic from water by coprecipitation with iron: Effect of arsenic and iron concentrations and adsorbent incorporation	2019
7959	T. Makino	Simultaneous decrease of arsenic and cadmium in rice (Oryza sativa L.) plants cultivated under submerged field conditions by the application of iron-bearing materials	2016
7960	T. Kiany	Effects of silicon and titanium dioxide nanoparticles on arsenic accumulation, phytochelatin metabolism, and antioxidant system by rice under arsenic toxicity	2022
7961	V. H. Phan	As release under the microbial sulfate reduction during redox oscillations in the upper Mekong delta aquifers, Vietnam: A mechanistic study	2019
7962	B. Yuan	Cytotoxic Effects of Arsenite in Combination With Gamabufotalin Against Human Glioblastoma Cell Lines	2021
7963	O. S. Vereshchagin	Gasparite-(La), La(AsO4), a new mineral from Mn ores of the Ushkatyn-III deposit, Central Kazakhstan, and metamorphic rocks of the Wanni glacier, Switzerland	2019
7964	F. S. Farnese	Effects of adding nitroprusside on arsenic stressed response of pistia stratiotes l. under hydroponic conditions	2014
7965	K. L. Yang	Removal of Sb(V) from aqueous solutions using Fe-Mn binary oxides: The influence of iron oxides forms and the role of manganese oxides	2018
7966	S. W. Li	Arsenic bioaccessibility in contaminated soils: Coupling in vitro assays with sequential and HNO3 extraction	2015
7967	A. S. Abboud	Alginate caged graphene oxide -modified metformin beads for the removal of Arsenic (III) and (V) from aqueous media; kinetic and equilibrium, thermodynamic studies	
7968	S. W. Zou	Adsorption of organic and inorganic arsenic from aqueous solution: Optimization, characterization and performance of Fe-Mn-Zr ternary magnetic sorbent	2022
7969	P. Wan	Arsenate removal by reactive mixed matrix PVDF hollow fiber membranes with UIO-66 metal organic frameworks	2020
7970	I. L. A. Ouma	Thermodynamic, kinetic and spectroscopic investigation of arsenite adsorption mechanism on pine cone-magnetite composite	2018
7971	Y. W. Chen	Arsenic speciation in surface waters and lake sediments in an abandoned mine site and field observations of arsenic eco-toxicity	2019
7972	Y. L. Sun	Stabilization of available arsenic in arsenic-calcium residue (ACR) using a developed cost-effective composite Fe-based stabilizer	2022

7973	S. Smith	Arsenic Release and Attenuation Processes in a Groundwater Aquifer During Anaerobic Remediation of TCE with Biostimulation	2019
7974	M. L. Kang	Redox reaction of aqueous selenite with As-rich pyrite from Jiguanshan ore mine (China): Reaction products and pathway	2014
7975	I. Koch	Arsenic(+3) and DNA methyltransferases, and arsenic speciation in tadpole and frog life stages of western clawed frogs (Silurana tropicalis) exposed to arsenate	2015
7976	Y. Y. Sun	Reduction of arsenic bioavailability by amending seven inorganic materials in arsenic contaminated soil	2015
7977	K. T. Yan	Effects of thiolation and methylation on arsenic sorption to geothermal sediments	2022
7978	S. O. Azubuike-Osu	Virgin Coconut Oil Resists Arsenic-Induced Cerebral Neurotoxicity and Cholesterol Imbalance via Suppression of Oxidative Stress, Adenosine Deaminase and Acetylcholinesterase Activities in Rats	2021
7979	S. Paikaray	Arsenic fractionation and mobilization in agricultural soils of NE Punjab, India	2022
7980	B. R. C. Vieira	Arsenic removal from water using iron-coated seaweeds	2017
7981	E. T. S. Costa	Comparing the sorptive affinity of an aluminum-mining by-product for cationic and anionic pollutants	2021
7982	D. Cakmak	Identification of different sources and forms of arsenic in the vicinity of ore mining in serbia	2015
7983	Z. Y. Zhang	Phosphate enhanced abiotic and biotic arsenic mobilization in the wetland rhizosphere	2017
7984	P. B. Hu	Adsorption of NOx with NH3 on gamma-Al2O3 and Its Effects on the Adsorption of As2O3 Based on DFT	2021
7985	S. Soleimani	Application of surfactant-modified montmorillonitefor As(III) removal from aqueous solutions: kinetics and isotherm study	2018
7986	M. L. Chou	Inhibition of ethylenediaminetetraacetic acid ferric sodium salt (EDTA-Fe) and calciumperoxide (CaO2) on arsenic uptake by vegetables in arsenic-rich agricultural soil	2016
7987	E. G. Duncan	The formation and fate of organoarsenic species in marine ecosystems: do existing experimental approaches appropriately simulate ecosystem complexity?	2015
7988	Z. D. Nejad	Reclamation of arsenic contaminated soils around mining site using solidification/stabilization combined with revegetation	2017
7989	B. Liu	Comparing adsorption of arsenic and antimony from single-solute and bi-solute aqueous systems onto ZIF-8	2018
7990	R. L. Smith	Anoxic nitrate reduction coupled with iron oxidation and attenuation of dissolved arsenic and phosphate in a sand and gravel aquifer	2017

7991	A. S. Saleemi	Synthesis and sensing efficiency of CN-wrapped ZnFe(2)O(4)microsphere-ionic liquid composites towards ultra-high sensitive arsenic(iii) monitoring of ground drinking water	2020
7992	C. Sbarbati	Redox Dependent Arsenic Occurrence and Partitioning in an Industrial Coastal Aquifer: Evidence from High Spatial Resolution Characterization of Groundwater and Sediments	2020
7993	M. Kulik	Chemical composition of native oxides on noble gases implanted GaAs	2016
7994	N. Das	Seasonal disparity in the co-occurrence of arsenic and fluoride in the aquifers of the Brahmaputra flood plains, Northeast India	2017
7995	I. Jacukowicz-Sobala	Hybrid polymer containing ferric oxides obtain using a redox polymer. Part II. Sorption properties towards chromate	2014
7996	X. M. Dou	Identification of Fe and Zr oxide phases in an iron-zirconium binary oxide and arsenate complexes adsorbed onto their surfaces	2018
7997	H. Wang	Randomized clinical control study of locoregional therapy combined with arsenic trioxide for the treatment of hepatocellular carcinoma	2015
7998	N. Boujelben	Removal of phosphorus ions from aqueous solutions using manganese-oxide-coated sand and brick	2014
7999	J. Villalobos	Structural and functional role of anions in electrochemical water oxidation probed by arsenate incorporation into cobalt-oxide materials	2019
8000	J. Chen	Biosorption of As(V) onto dried alligator weed root: role of metal (hydro) oxides	2016
8001	Y. C. Dai	Comparison of DGT with traditional extraction methods for assessing arsenic bioavailability to Brassica chinensis in different soils	2018
8002	V. Chandrakar	Modulation of antioxidant enzymes by salicylic acid in arsenic exposed Glycine max L	2016
8003	R. R. Zhao	KH2PO4-aided soil washing for removing arsenic from water-stable soil aggregates collected in southern China	2016
8004	C. Garca-Carvajal	Arsenic (V) removal from aqueous solutions using natural clay ceramic monoliths	2019
8005	Z. Q. Zhu	Preparation and Properties of Porous Composite of Hematite/Magnetite/Carbon with Eucalyptus Wood Biotemplate	2015
8006	L. Panneerselvam	Ferulic acid attenuates arsenic-induced cardiotoxicity in rats	2020
8007	K. H. Johannesson	Biogeochemical and reactive transport modeling of arsenic in groundwaters from the Mississippi River delta plain: An analog for the As-affected aquifers of South and Southeast Asia	2019
8008	I. Andjelkovic	Microwave-hydrothermal synthesis of TiO2 and zirconium doped TiO2 adsorbents for removal of As(III) and As(V)	2015

8009	H. W. Wang	Fabricating biogenic Fe(III) flocs from municipal sewage sludge using NAFO processes: Characterization and arsenic removal ability	2019
8010	Y. H. Qi	In Situ Dielectric Barrier Discharge Trap for Ultrasensitive Arsenic Determination by Atomic Fluorescence Spectrometry	2018
8011	S. Majumder	The role of agronomic factors (rice cultivation practices and soil amendments) on Arsenic fractionation: A strategy to minimise Arsenic uptake by rice, with some observations related to cadmium	2021
8012	C. Liu	Facile Synthesis of Manganese Dioxide Nanoparticles for Efficient Removal of Aqueous As(III)	2020
8013	X. H. Zhu	Facile surface modification of mesoporous silica with heterocyclic silanes for efficiently removing arsenic	2019
8014	P. J. J. Alvarez	Emerging opportunities for nanotechnology to enhance water security	2018
8015	J. Z. Zhou	Enhanced arsenite immobilization via ternary layered double hydroxides and application to paddy soil remediation	2017
8016	L. Wang	Rational design, synthesis, adsorption principles and applications of metal oxide adsorbents: a review	2020
8017	H. S. Yun	Remediation of arsenic-contaminated soils via waste-reclaimed treatment agents: Batch and field studies	2018
8018	L. Balcells	One-Step Route to Iron Oxide Hollow Nanocuboids by Cluster Condensation: Implementation in Water Remediation Technology	2016
8019	J. Suba	Enhancement of the adsorption capacity of Fe-clay by bioleaching and activation of Fe sites	2018
8020	M. A. Rahman	Is arsenic biotransformation a detoxification mechanism for microorganisms?	2014
8021	L. P. Lingamdinne	Facile synthesis of flowered mesoporous graphene oxide-lanthanum fluoride nanocomposite for adsorptive removal of arsenic	2019
8022	C. Claudio	Iron Oxide Nanoparticles in Soils: Environmental and Agronomic Importance	2017
8023	J. Druzbicka	Metalloid Attenuation from Runoff Waters at an Historic Orogenic Gold Mine, New Zealand	2015
8024	W. Cheng	Synthesis of porous superparamagnetic iron oxides from colloidal nanoparticles: Effect of calcination temperature and atmosphere	2015
8025	L. Ye	Biotransformation of adsorbed arsenic on iron minerals by coexisting arsenate-reducing and arsenite-oxidizing bacteria	2020
8026	S. A. Baig	Arsenic Removal from Aqueous Solutions Using Fe3O4-HBC Composite: Effect of Calcination on Adsorbents Performance	2014

8027	D. W. Wang	Iron Mesh-Based Metal Organic Framework Filter for Efficient Arsenic Removal	2018
8028	K. Huang	Efficient Arsenic Methylation and Volatilization Mediated by a Novel Bacterium from an Arsenic-Contaminated Paddy Soil	2016
8029	B. Peng	Facile synthesis of Fe3O4@Cu(OH)(2) composites and their arsenic adsorption application	2016
8030	F. Pinakidou	Monitoring the role of Mn and Fe in the As-removal efficiency of tetravalent manganese feroxyhyte nanoparticles from drinking water: An X-ray absorption spectroscopy study	2016
8031	A. A. Shah	Iron oxide nanoparticles and selenium supplementation improve growth and photosynthesis by modulating antioxidant system and gene expression of chlorophyll synthase (CHLG) and protochlorophyllide oxidoreductase (POR) in arsenic-stressed Cucumis melo	2022
8032	M. Baghayeri	Application of graphene/zinc-based metal-organic framework nanocomposite for electrochemical sensing of As(III) in water resources	2020
8033	N. Kandhol	Nanoparticles as a potential protective agent for arsenic toxicity alleviation in plants	2022
8034	Z. Z. Liu	Synergistic activation of sulfite for As(III) oxidation by basic copper(II) carbonate in homogeneous and heterogeneous processes at near-neutral conditions	2022
8035	J. R. Rivera-Hernandez	Strong Acid Mixture and Sequential Geochemical Arsenic Extractions in Surface Sediments from the Santa Maria La Reforma Coastal Lagoon, Mexico: A Bioavailability Assessment	2016
8036	A. Kovalskiy	Structural origin of surface transformations in arsenic sulfide thin films upon UV-irradiation	2017
8037	L. M. Tung	Superparamagnetic Iron Oxide@Carbon Core-Shell Nanoparticles as Advanced Adsorbent for Efficient Removal of As(V) Ions From Wastewater	2018
8038	J. Chen	ArsH is an organoarsenical oxidase that confers resistance to trivalent forms of the herbicide monosodium methylarsenate and the poultry growth promoter roxarsone	2015
8039	S. Fakhreddine	Controlling Arsenic Mobilization during Managed Aquifer Recharge: The Role of Sediment Heterogeneity	2020
8040	Z. M. Lv	MOFs-derived magnetic chestnut shell-like hollow sphere NiO/Ni@C composites and their removal performance for arsenic(V)	2019
8041	T. S. Zhang	Amorphous Fe/Mn bimetal-organic frameworks: outer and inner structural designs for efficient arsenic(iii) removal	2019
8042	Q. X. Hou	Effects of Particle Size and Redox Potential on Arsenic Fractionation in Soils Irrigated with Arsenate-rich Water	2017
8043	M. M. Yan	Efficient removal of arsenic (III) from aqueous solutions by new layered hydroxides (Ca-Fe-Cl LDHs)	2021
8044	E. Iakovleva	Modified and unmodified low-cost iron-containing solid wastes as adsorbents for efficient removal of As(III) and As(V) from mine water	2016

8045	M. S. Ko	Field assessment of arsenic immobilization in soil amended with iron rich acid mine drainage sludge	2015
8046	X. Y. Bi	Adsorption of Arsenic Ions Transforms Surface Reactivity of Engineered Cerium Oxide Nanoparticles	2020
8047	L. J. Huo	Enhanced removal of As (V) from aqueous solution using modified hydrous ferric oxide nanoparticles	2017
8048	M. Rotiroti	Origin of Arsenic in Groundwater from the Multilayer Aquifer in Cremona (Northern Italy)	2014
8049	J. Song	Formation of iron (hydr)oxides during the abiotic oxidation of Fe(II) in the presence of arsenate	2015
8050	Z. Q. Shi	Quantifying Microbially Mediated Kinetics of Ferrihydrite Transformation and Arsenic Reduction: Role of the Arsenate-Reducing Gene Expression Pattern	2020
8051	H. Pak	Adsorptive Removal of Arsenic and Lead by Stone Powder/Chitosan/Maghemite Composite Beads	2021
8052	D. N. Zhang	The adsorption of As(V) on poorly crystalline Fe oxyhydroxides, revisited: Effect of the reaction media and the drying treatment	2021
8053	A. M. Anielak	Removal of As (III) and Cr (III) on Modified Clinoptylolite	2015
8054	S. Saif	Adsorption Studies of Arsenic(V) by CuO Nanoparticles Synthesized by Phyllanthus emblica Leaf-Extract-Fueled Solution Combustion Synthesis	2021
8055	Z. N. Deng	From laboratory to large-scale manufacture of anion exchange resin-supported nano-hydrated zirconium oxide for As(V) removal from water solutions	2021
8056	Y. X. Li	Efficient removal of As(III) from aqueous solution by S-doped copper-lanthanum bimetallic oxides: Simultaneous oxidation and adsorption	2020
8057	Y. X. Li	Application of magnetic chitosan nanocomposites modified by graphene oxide and polyethyleneimine for removal of toxic heavy metals and dyes from water	2021
8058	C. H. Zong	Adsorption of Arsenite on Gold Nanoparticles Studied with DNA Oligonucleotide Probes	2019
8059	B. Li	Cadmium and arsenic availability in soil under submerged incubation: The influence of humic substances on iron speciation	2021
8060	P. Tangviroon	Effects of Additional Layer(s) on the Mobility of Arsenic from Hydrothermally Altered Rock in Laboratory Column Experiments	2017
8061	Y. R. He	Concurrent Removal of Selenium and Arsenic from Water Using Polyhedral Oligomeric Silsesquioxane (POSS)-Polyamide Thin-Film Nanocomposite Nanofiltration Membranes	2016
8062	M. Vithanage	Effects of soil type and fertilizer on As speciation in rice paddy contaminated with As-containing pesticide	2014

8063	Y. Tanveer	Ameliorative effects of plant growth promoting bacteria, zinc oxide nanoparticles and oxalic acid on Luffa acutangula grown on arsenic enriched soil*	2022
8064	S. J. Zhou	Simultaneous reduction in cadmium and arsenic accumulation in rice (Oryza sativa L.) by iron/iron-manganese modified sepiolite	2022
8065	J. Ayala	Industrial waste materials as adsorbents for the removal of As and other toxic elements from an abandoned mine spoil heap leachate: a case study in Asturias	2020
8066	O. O. Onireti	Combined effects of low-molecular-weight organic acids on mobilization of arsenic and lead from multi-contaminated soils	2017
8067	T. Okazaki	Development of Novel Spectrophotometric Analytical Methods for Evaluating Environmental Water Quality and Its Applications	2015
8068	X. Y. Wang	Accumulation, transformation and subcellular distribution of arsenite associated with five carbon nanomaterials in freshwater zebrafish specific-tissues	2021
8069	L. N. Lin	Effects of Fe-Mn modified biochar composite treatment on the properties of As-polluted paddy soil	2019
8070	L. W. Wang	Long-term immobilization of soil metalloids under simulated aging: Experimental and modeling approach	2022
8071	G. Zhao	A flexible and disposable electrochemical sensor for the evaluation of arsenic levels: A new and efficient method for the batch fabrication of chemically modified electrodes	2022
8072	L. Richter	Dissolved arsenic in the upper Paraguay River basin and Pantanal wetlands	2019
8073	P. L. Zamora	Radical Model of Arsenic(III) Toxicity: Theoretical and EPR Spin Trapping Studies	2014
8074	B. Dousova	Removal of arsenate and antimonate by acid-treated Fe-rich clays	2018
8075	M. Tang	Accumulation and Release of Arsenic from Cast Iron: Impact of Initial Arsenic and Orthophosphate Concentrations	2021
8076	V. Uwamariya	Effect of calcium on adsorptive removal of As(III) and As(V) by iron oxide-based adsorbents	2014
8077	J. Rawson	Quantifying Reactive Transport Processes Governing Arsenic Mobility after Injection of Reactive Organic Carbon into a Bengal Delta Aquifer	2017
8078	J. P. Cabilla	Adverse effects induced by chromium VI, cadmium and arsenic exposure on hypothalamus-pituitary physiology	2016
8079	P. Z. Ray	Inorganic nano-adsorbents for the removal of heavy metals and arsenic: a review	2015
8080	M. Jain	Copper oxide nanoparticles for the removal of divalent nickel ions from aqueous solution	2021

8081	R. Z. Sun	Mitigation of arsenic accumulation in arugula (Eruca sativaMill.) using Fe/Al/Zn impregnated biochar composites	2021
8082	K. Leus	Removal of arsenic and mercury species from water by covalent triazine framework encapsulated gamma-Fe2O3 nanoparticles	2018
8083	B. Y. Song	Effects of Ni incorporation on the reactivity and stability of hausmannite (Mn3O4): Environmental implications for Mn, Ni, and As solubility and cycling	2020
8084	F. Lian	Effects of Fe-Mn-Ce oxide-modified biochar on As accumulation, morphology, and quality of rice (Oryza sativa L.)	2020
8085	Z. M. Gusiatin	Tannic acid for remediation of historically arsenic-contaminated soils	2019
8086	Q. X. Hou	The bioaccessibility and fractionation of arsenic in anoxic soils as a function of stabilization using low-cost Fe/Al-based materials: A long-term experiment	2020
8087	S. Yamamura	Soil Microbial Communities Involved in Reductive Dissolution of Arsenic from Arsenate-Laden Minerals with Different Carbon Sources	2019
8088	A. Kasiuliene	Removal of metal(oid)s from contaminated water using iron-coated peat sorbent	2018
8089	S. L. DeSisto	Subsurface variations in arsenic mineralogy and geochemistry following long-term weathering of gold mine tailings	2016
8090	D. Ocinski	Water treatment residuals containing iron and manganese oxides for arsenic removal from water - Characterization of physicochemical properties and adsorption studies	2016
8091	A. Karimi	Single-Particle Investigation of Environmental Redox Processes of Arsenic on Cerium Oxide Nanoparticles by Collision Electrochemistry	2019
8092	T. Ariga	Study on the Formation Process of Oxide Ion and the Influence of Carbon Matrix in Inductively Coupled Plasma Mass Spectrometry Using O-18-Labeled Arsenous Acid	2021
8093	F. Arab	An eco-friendly method for heavy metal removal from mine tailings	2018
8094	J. M. Raez	Combination of immobilized TiO2 and zero valent iron for efficient arsenic removal in aqueous solutions	2021
8095	Z. J. Lin	Nitrate reduced arsenic redox transformation and transfer in flooded paddy soil-rice system	2018
8096	M. Patel	Salinity alleviates the arsenic toxicity in the facultative halophyte Salvadora persica L. by the modulations of physiological, biochemical, and ROS scavenging attributes	2021
8097	P. Katiyar	Titanium nanoparticles attenuates arsenic toxicity by up-regulating expressions of defensive genes in Vigna radiata L	2020
8098	Z. H. Khan	Mechanism of As(III) removal properties of biochar-supported molybdenum-disulfide/iron-oxide system	2021

8099	M. S. Angotzi	Meso- and macroporous silica-based arsenic adsorbents: effect of pore size, nature of the active phase, and silicon release	2021
8100	S. Feng	Relative importance of hydrogeochemical and hydrogeological processes on arsenic enrichment in groundwater of the Yinchuan Basin, China	2022
8101	X. J. Xie	Effect of irrigation on Fe(III)-SO42- redox cycling and arsenic mobilization in shallow groundwater from the Datong basin, China: Evidence from hydrochemical monitoring and modeling	2015
8102	J. Z. Beiyuan	Fate of arsenic before and after chemical-enhanced washing of an arsenic-containing soil in Hong Kong	2017
8103	M. Ibrahim	Biochars mitigate greenhouse gas emissions and bioaccumulation of potentially toxic elements and arsenic speciation in Phaseolus vulgaris L	2017
8104	H. P. Zeng	Removal of As(V) by a core-shell magnetic nanoparticles synthesized with iron-containing water treatment residuals	2021
8105	Y. Abdellaoui	Iron-zirconium microwave-assisted modification of small-pore zeolite W and its alginate composites for enhanced aqueous removal of As(V) ions: Experimental and theoretical studies	2021
8106	J. S. He	New insight into adsorption and co-adsorption of arsenic and tetracycline using a Y-immobilized graphene oxide-alginate hydrogel: Adsorption behaviours and mechanisms	2020
8107	S. Y. Ren	Simultaneous evaluation of kinetic release of labile arsenic and phosphorus in agricultural soils using cerium oxide-based DGT	2022
8108	J. Abeywickrama	Geochemical characterization of fouling on mine water driven plate heat exchangers in Saxon mining region, Germany	2021
8109	C. Catrouillet	Coupled As and Mn Redox Transformations in an Fe(0) Electrocoagulation System: Competition for Reactive Oxidants and Sorption Sites	2020
8110	Y. X. Wang	Sedimentogenesis and hydrobiogeochemistry of high arsenic Late Pleistocene-Holocene aquifer systems	2019
8111	J. S. Li	Arsenic-containing soil from geogenic source in Hong Kong: Leaching characteristics and stabilization/solidification	2017
8112	M. Baikousi	Synthesis and characterization of robust zero valent iron/mesoporous carbon composites and their applications in arsenic removal	2015
8113	W. Wisawapipat	Sulfur amendments to soil decrease inorganic arsenic accumulation in rice grain under flooded and nonflooded conditions: Insights from temporal dynamics of porewater chemistry and solid-phase arsenic solubility	2021
8114	S. Zhou	Enhanced Bioaccumulation and Toxicity of Arsenic in Marine Mussel Perna viridis in the Presence of CuO/Fe3O4 Nanoparticles	2021
8115	V. Uwamariya	Effect of Fulvic Acid on Adsorptive Removal of Cr(VI) and As(V) from Groundwater by Iron Oxide-Based Adsorbents	2015
8116	G. Abbas	Arsenic Uptake, Toxicity, Detoxification, and Speciation in Plants: Physiological, Biochemical, and Molecular Aspects	2018

8117	A. Vermeulen	Sources of variation in innate immunity in great tit nestlings living along a metal pollution gradient: An individual-based approach	2015
8118	M. Faizan	Modulation of Cellular Redox Status and Antioxidant Defense System after Synergistic Application of Zinc Oxide Nanoparticles and Salicylic Acid in Rice (Oryza sativa) Plant under Arsenic Stress	2021
8119	S. F. Araujo	Basic oxygen furnace sludge to treat industrial arsenic- and sulfate-rich acid mine drainage	2022
8120	J. Lin	First observation of labile arsenic stratification in aluminum sulfate-amended sediments using high resolution Zr-oxide DGT	2017
8121	L. Onnby	gamma-Al2O3-based nanocomposite adsorbents for arsenic(V) removal: Assessing performance, toxicity and particle leakage	2014
8122	E. Roy	Europium doped magnetic graphene oxide-MWCNT nanohybrid for estimation and removal of arsenate and arsenite from real water samples	2016
8123	M. Iwai-Shimada	Exposure profile of mercury, lead, cadmium, arsenic, antimony, copper, selenium and zinc in maternal blood, cord blood and placenta: the Tohoku Study of Child Development in Japan	2019
8124	Y. Zhu	Macroscopic and Spectroscopic Assessment of the Cosorption of Fe(II) with As(III) and As(V) on Al-Oxide	2015
8125	Y. F. Wei	Deep oxidation and removal of arsenite in groundwater by rationally positioning oxidation and adsorption sites in binary Fe-Cu oxide/TiO2	2018
8126	M. Rotiroti	Overlapping redox zones control arsenic pollution in Pleistocene multi-layer aquifers, the Po Plain (Italy)	2021
8127	A. Praveen	Nitric oxide alters nitrogen metabolism and PIN gene expressions by playing protective role in arsenic challenged Brassica juncea L	2019
8128	F. Rosario	Unravelling the Potential Cytotoxic Effects of Metal Oxide Nanoparticles and Metal(Loid) Mixtures on A549 Human Cell Line	2020
8129	Z. L. Zhao	MOF derived iron oxide-based smart plasmonic Ag/Au hollow and porous nanoshells "ultra-microelectrodes" for ultra-sensitive detection of arsenic	2018
8130	M. M. Rahman	Electrodeposition of Gold on Fluorine-Doped Tin Oxide: Characterization and Application for Catalytic Oxidation of Nitrite	2014
8131	M. Tahmaseb poor	Arsenate removal from contaminated water using Fe2O3-clinoptilolite powder and granule	2022
8132	P. Panigrahi	Efficient Adsorption Characteristics of Pristine and Silver-Doped Graphene Oxide Towards Contaminants: A Potential Membrane Material for Water Purification?	2018
8133	L. J. Wilk	Removal of sulfides from water using a hybrid ion exchanger containing manganese(IV) oxide	2020
8134	I. Safarik	Magnetically-modified natural biogenic iron oxides for organic xenobiotics removal	2015

8135	Z. N. Li	Portal Vein Stenting Combined with I-125 Particle Chain Implantation Followed by As2O3 in the Treatment of Hepatocellular Carcinoma with Portal Vein Tumour Thrombus	2020
8136	D. N. Thanh	Amorphous nanosized Al-Ti-Mn trimetal hydrous oxides: synthesis, characterization and enhanced performance in arsenic removal	2016
8137	M. Zhu	Speciation and sorption structure of diphenylarsinic acid in soil clay mineral fractions using sequential extraction and EXAFS spectroscopy	2020
8138	S. L. Wachowski	Fabrication and Structural Properties of LaNb1-xAsxO4 Ceramics	2019
8139	D. B. Pal	Cadmium removal by composite copper oxide/ceria adsorbent from synthetic wastewater	
8140	J. M. Harrington	Validation of a Metallomics Analysis of Placenta Tissue by Inductively-Coupled Plasma Mass Spectrometry	2016
8141	M. A. Armienta	Arsenic and metals mobility in soils impacted by tailings at Zimapan, M,xico	2016
8142	M. Babudurai	Mechanical activation of TiO2/Fe2O3 nanocomposite for arsenic adsorption: effect of ball-to-powder ratio and milling time	2021
8143	D. Choudhary	Nanoalumina-supported Mn2O3 as efficient adsorbent for removal of fluoride and arsenic from water: a study from lab to field	2022
8144	S. J. Zhang	Sulfidation of ferric (hydr)oxides and its implication on contaminants transformation: a review	2022
8145	E. M. Ahmed	Limited frequency domain of the ac conductivity of some As2O3.V2O5.CuO. Nd2O3 glasses	2021
8146	S. Zhang	Effects of cobalt doping on the reactivity of hausmannite for As(III) oxidation and As(V) adsorption	2022
8147	R. Siripuram	Physical, Optical, Structural and Thermal Studies of xWO(3) + (30-x)As2O3+70TeO(2) (where x=10, 20, 30 mol%) glasses by UV-vis, Raman, IR and DSC Studies	2020
8148	K. Ehlert	Effects of Manganese Oxide on Arsenic Reduction and Leaching from Contaminated Floodplain Soil	2016
8149	M. Annaduzzaman	Arsenic removal from iron-containing groundwater by delayed aeration in dual-media sand filters	2021
8150	A. Puente-Urbina	Porous Materials Modified with Fe3O4 Nanoparticles for Arsenic Removal in Drinking Water	2017
8151	B. Dousova	Thermal stability of arsenic complexes in soils	2021
8152	Q. W. Cao	Arsenic(V) removal behavior of schwertmannite synthesized by KMnO4 rapid oxidation with high adsorption capacity and Fe utilization	2021

8153	X. P. Nie	As3+ or/and Cu2+ exposure triggers oxidative stress imbalance, induces inflammatory response and apoptosis in chicken brain	2020
8154	E. M. Ahmed	Effect of Nd3+ ions on the structural, physical and emission properties of some As2O3 center dot V2O5 center dot CuO glasses	2020
8155	P. Benjwal	Enhanced photocatalytic degradation of methylene blue and adsorption of arsenic(III) by reduced graphene oxide (rGO)-metal oxide (TiO2/Fe3O4) based nanocomposites	2015
8156	Z. Cetinkaya	Production of red mud based nanofibers and their potential in arsenate removal from waste water	2022
8157	T. Y. Lin	Arsenic oxyanion binding to NOM from dung and aquaculture pond sediments in Bangladesh: Importance of site-specific binding constants	2017
8158	G. H. Mao	Neurological Toxicity of Individual and Mixtures of Low Dose Arsenic, Mono and Di (n-butyl) Phthalates on Sub-Chronic Exposure to Mice	2016
8159	Q. R. Zhang	Efficient phosphate sequestration for water purification by unique sandwich-like MXene/magnetic iron oxide nanocomposites	2016
8160	K. Shehzad	Mesoporous zirconia nanostructures (MZN) for adsorption of As(III) and As(V) from aqueous solutions	2019
8161	I. Jacukowicz-Sobala	Synthesis and characterization of hybrid materials containing iron oxide for removal of sulfides from water	2015
8162	I. Jacukowicz-Sobala	Cu(II)-Fe(III) oxide doped anion exchangers - Multifunctional composites for arsenite removal from water via As(III) adsorption and oxidation	2020
8163	Z. H. Wang	Abiotic oxidation of arsenite in natural and engineered systems: Mechanisms and related controversies over the last two decades (1999-2020)	2021
8164	R. Mudzielwana	Enhanced As(III) and As(V) Adsorption From Aqueous Solution by a Clay Based Hybrid Sorbent	2020
8165	S. C. Ma	Removal of As(III) by Electrically Conducting Ultrafiltration Membranes	2021
8166	L. Ou	Impact of soil organic carbon on monosodium methyl arsenate (MSMA) sorption and species transformation	2017
8167	A. Suda	The effects of soil amendments on arsenic concentrations in soil solutions after long-term flooded incubation	2015
8168	B. K. Chaudhary	Preparation and Characterization of Homopolymer Polyacrylonitrile-Based Fibrous Sorbents for Arsenic Removal	2014
8169	L. Liu	How different nitrogen fertilizers affect arsenic mobility in paddy soil after straw incorporation?	2022
8170	L. Yohai	Nanocomposite functionalized membranes based on silica nanoparticles cross-linked to electrospun nanofibrous support for arsenic(v) adsorption from contaminated underground water	2019

8171	L. Li	Arsenic removal in synthetic ground water using iron electrolysis	2014
8172	Y. J. You	Modeling coupled kinetics of arsenic adsorption/desorption and oxidation in ferrihydrite-Mn(II)/manganese (oxyhydr)oxides systems	2020
8173	H. I. Afridi	Distribution of Arsenic, Cadmium, Lead, and Nickel Levels in Biological Samples of Pakistani Hypertensive Patients and Control Subjects	2014
8174	S. J. Yan	Efficient removal of As(V) from diluted aqueous solutions by Fe/La oxide magnetic microspheres	2020
8175	M. A. Argudin	Heavy metal resistance in bacteria from animals	2019
8176	L. N. Lin	Enhanced As(III) removal from aqueous solution by Fe-Mn-La-impregnated biochar composites	2019
8177	H. Uppal	Facile chemical synthesis and novel application of zinc oxysulfide nanomaterial for instant and superior adsorption of arsenic from water	2019
8178	M. Bhaumik	Polyaniline/Fe-0 composite nanofibers: An excellent adsorbent for the removal of arsenic from aqueous solutions	2015
8179	J. T. Lessl	Pteris vittata continuously removed arsenic from non-labile fraction in three contaminated-soils during 3.5 years of phytoextraction	2014
8180	S. W. Wang	Evaluation of the dual-process approach for in-situ groundwater arsenic removal	
8181	L. F. Zhang	Simultaneous oxidation and adsorption of As(III) from water by cerium modified chitosan ultrafine nanobiosorbent	2016
8182	R. Mukhopadhyay	Aquaglyceroporins: Generalized metalloid channels	2014
8183	X. Yang	Ball-milled magnetite for efficient arsenic decontamination: Insights into oxidation-adsorption mechanism	2022
8184	X. Zheng	Efficient removal of As(V) from simulated arsenic-contaminated wastewater via a novel metal-organic framework material: Synthesis, structure, and response surface methodology	2020
8185	V. N. Priya	Chitosan assisted Fe-Al double layered hydroxide/reduced graphene oxide composites for As(V) removal	2020
8186	M. Taneez	Adsorption of inorganic pollutants on bauxite residues: An example of methodology to simulate adsorption in complex solids mixtures	2017
8187	J. Valentin-Reyes	Abatement of hydrated silica, arsenic, and coexisting ions from groundwater by electrocoagulation using iron electrodes	2022
8188	A. A. Alsahli	Hydrogen Sulfide (H2S) Mitigates Arsenic (As)-Induced Toxicity in Pea (Pisum sativum L.) Plants by Regulating Osmoregulation, Antioxidant Defense System, Ascorbate Glutathione Cycle and Glyoxalase System	2021

8189	C. Pandey	Selenium amelioration of arsenic toxicity in rice shows genotypic variation: A transcriptomic and biochemical analysis	2018
8190	P. Singh	Kinetics and mechanism of arsenic removal using sulfide-modified nanoscale zerovalent iron	2021
8191	H. T. H. Nguyen	Pilot-Scale Removal of Arsenic and Heavy Metals from Mining Wastewater Using Adsorption Combined with Constructed Wetland	2019
8192	N. P. Raval	Geogenic arsenic removal through core - shell based functionalized nanoparticles: Groundwater in-situ treatment perspective in the post - COVID anthropocene	2021
8193	M. Chakraborty	A Review of Groundwater Arsenic in the Bengal Basin, Bangladesh and India: from Source to Sink	2015
8194	S. G. Chung	Modified composites based on mesostructured iron oxyhydroxide and synthetic minerals: A potential material for the treatment of various toxic heavy metals and its toxicity	2014
8195	Q. Tang	AuNPs-polyaniline nanosheet array on carbon nanofiber for the determination of As(III)	2020
8196	A. K. Nighojkar	Use of marble and iron waste additives for enhancing arsenic and E. coli contaminant removal capacity and strength of porous clay ceramic materials for point of use drinking water treatment	2019
8197	F. P. Larroca	Water Treatment Plant Prototype with pH Control Modeled on Fuzzy Logic for Removing Arsenic Using Fe(VI) and Fe(III)	2020
8198	N. Y. Yin	In Vitro Method To Assess Soil Arsenic Metabolism by Human Gut Microbiota: Arsenic Speciation and Distribution	2015
8199	H. Parthasarathy	A method for preparation and cleaning of uniformly sized arsenopyrite particles	2014
8200	A. Borcinov Radkov	Mineralogical controls on antimony and arsenic mobility during tetrahedrite-tennantite weathering at historic mine sites Spania Dolina-Piesky and Lubietova-Svatodusna, Slovakia	2017
8201	H. P. Zeng	Preparation of manganese sludge strengthened chitosan-alginate hybrid adsorbent and its potential for As(III) removal	2020
8202	G. Bianchini	Origin of Fluoride and Arsenic in the Main Ethiopian Rift Waters	2020
8203	B. Te	Investigation of Arsenic Removal from Water by Iron-Mixed Mesoporous Pellet in a Continuous Fixed-Bed Column	2018
8204	A. Rahman	Calcium Mitigates Arsenic Toxicity in Rice Seedlings by Reducing Arsenic Uptake and Modulating the Antioxidant Defense and Glyoxalase Systems and Stress Markers	2015
8205	W. Xiu	Genome-Resolved Metagenomic Analysis of Groundwater: Insights into Arsenic Mobilization in Biogeochemical Interaction Networks	
8206	M. Annaduzzaman	Anoxic storage to promote arsenic removal with groundwater-native iron	2021

8207	T. H. Bui	Enhanced selective removal of arsenic(V) using a hybrid nanoscale zirconium molybdate embedded anion exchange resin	2019
8208	J. Bai	Multi-isotope constraints on biogeochemical processes during bank filtration: A case study of the Liao River, Northeast China	2020
8209	X. J. Long	Simultaneous removal of antimony and arsenic by nano-TiO2-crosslinked chitosan (TA-chitosan) beads	
8210	R. C. T. Porto	Zinc oxide nanoparticles synthesized by the polyol method: characterization and evaluation of the antibacterial activity	2017
8211	Y. X. Deng	Redox-dependent effects of phosphate on arsenic speciation in paddy soils	2020
8212	T. F. Wang	Two-dimension black arsenic-phosphorus as a promising NO sensor: A DFT study	2022
8213	L. C. Kong	One-step construction of hierarchical porous channels on electrospun MOF/polymer/graphene oxide composite nanofibers for effective arsenate removal from water	2022
8214	J. Sun	Effect of oxalic acid treatment on sediment arsenic concentrations and lability under reducing conditions	2016
8215	A. Molinari	Geochemical modeling of arsenic release from a deep natural solid matrix under alternated redox conditions	2014
8216	X. L. Cai	Biogeochemical processes of arsenic transformation and redistribution in contaminated soils: Combined effects of iron, sulfur, and organic matter	2022
8217	Y. L. Wang	Simultaneous removal and oxidation of arsenic from water by delta-MnO2 modified activated carbon	2020
8218	J. Dobrzynska	A hybrid Zr/amine-modified mesoporous silica for adsorption and preconcentration of as before its FI HG AAS determination in water	2021
8219	B. Mueller	Results of the First Improvement Step Regarding Removal Efficiency of Kanchan Arsenic Filters in the Lowlands of Nepal-A Case Study	2021
8220	R. Mudzielwana	Uptake of As(V) from Groundwater Using Fe-Mn Oxides Modified Kaolin Clay: Physicochemical Characterization and Adsorption Data Modeling	2019
8221	X. Q. Wang	Effects of Simultaneous Application of Ferrous Iron and Nitrate on Arsenic Accumulation in Rice Grown in Contaminated Paddy Soil	2018
8222	B. Dousova	Effect of organic matter on arsenic(V) and antimony(V) adsorption in soils	2015
8223	H. Claes	Arsenic anomalies in soils and rocks occurring in the southwestern part of the Grand Duchy of Luxembourg	2021
8224	H. Viitala	Influence of nickel alloying on hot corrosion of stainless steels in SO2-environment	2017

8225	M. D. Sharma	Graphene/fluorescein dye-based sensor for detecting As(III) in drinking water	2021
8226	J. Ge	Challenges of arsenic removal from municipal wastewater by coagulation with ferric chloride and alum	2020
8227	J. P. H. Perez	Arsenic removal from natural groundwater using 'green rust': Solid phase stability and contaminant fate	2021
8228	C. D. Quarles	Rapid automated total arsenic and arsenic speciation by inductively coupled plasma mass spectrometry	2022
8229	P. I. Omwene	Arsenic removal from naturally arsenic contaminated ground water by packed-bed electrocoagulator using Al and Fe scrap anodes	2019
8230	M. Magro	Colloidal maghemite nanoparticles with oxyhydroxide-like interface and chiroptical properties	2020
8231	Z. L. Gao	Metal-organic gels of simple chemicals and their high efficacy in removing arsenic(V) in water	2018
8232	C. Betti	Jasmonates, Ethylene and Brassinosteroids Control Adventitious and Lateral Rooting as Stress Avoidance Responses to Heavy Metals and Metalloids	2021
8233	L. Y. Lin	A Combined Remediation Strategy of Arsenic and Cadmium in the Paddy Soil of Polymetallic Mining Areas	2021
8234	Y. B. Cai	Sources, speciation and transformation of arsenic in the gold mining impacted Jiehe River, China	2017
8235	M. A. Shenashen	Effective, Low-Cost Recovery of Toxic Arsenate Anions from Water by Using Hollow-Sphere Geode Traps	2017
8236	S. M. Su	Is soil dressing a way once and for all in remediation of arsenic contaminated soils? A case study of arsenic re-accumulation in soils remediated by soil dressing in Hunan Province, China	2015
8237	W. C. Lee	Sequestration of arsenate from aqueous solution using 2-line ferrihydrite: equilibria, kinetics, and X-ray absorption spectroscopic analysis	2014
8238	F. F. Che	Arsenic distribution and speciation in multiphase media of a lake basin, Tibet: The influences of environmental factors on arsenic biogeochemical behavior in the cold arid plateau lake	2020
8239	Y. Sun	Impacts of External Organic Carbon on Arsenic Release in Aquifer of Jianghan Plain, Central China	2021
8240	C. D. Powell	Magnetic nanoparticle recovery device (MagNERD) enables application of iron oxide nanoparticles for water treatment	2020
8241	S. K. Panda	Hepatorenal Toxicity of Inorganic Arsenic in White Pekin Ducks and Its Amelioration by Using Ginger	
8242	B. Majumder	Evaluation of arsenic induced toxicity based on arsenic accumulation, translocation and its implications on physio-chemical changes and genomic instability in indica rice (Oryza sativa L.) cultivars	2020

8243	S. Majumder	Size-fractionation of groundwater arsenic in alluvial aquifers of West Bengal, India: The role of organic and inorganic colloids	2014
8244	K. Huang	Genetically Engineering Bacillus subtilis with a Heat-Resistant Arsenite Methyltransferase for Bioremediation of Arsenic-Contaminated Organic Waste	2015
8245	C. M. Genuchten	Emerging investigator series: interdependency of green rust transformation and the partitioning and binding mode of arsenic	2019
8246	A. H. Almuqrin	Influence of Li2O Incrementation on Mechanical and Gamma-Ray Shielding Characteristics of a TeO2-As2O3-B2O3 Glass System	2021
8247	Y. Li	Comparison of Four Amendments for Arsenic and Cadmium Combined Contaminated Soil	2020
8248	I. Rozahun	GaAs monolayer: Excellent SHG responses and semi metallic to metallic transition modulated by vacancy effect	2018
8249	A. Deonarine	Arsenic Speciation in Bituminous Coal Fly Ash and Transformations in Response to Redox Conditions	2016
8250	L. S. Qu	Role of PTEN-Akt-CREB Signaling Pathway in Nervous System impairment of Rats with Chronic Arsenite Exposure	2016
8251	L. Burbery	Evaluation of pollution swapping phenomena from a woodchip denitrification wall targetting removal of nitrate in a shallow gravel aquifer	2022
8252	J. J. Liang	Facile construction of 3D magnetic graphene oxide hydrogel via incorporating assembly and chemical bubble and its application in arsenic remediation	2019
8253	X. H. Tang	Selective Separation and Preconcentration of Arsenite from Arsenic Enriched Natural Waters with Three Different Adsorbents	2015
8254	C. H. Syu	Arsenic phytotoxicity and accumulation in rice seedlings grown in arsenic-contaminated soils as influenced by the characteristics of organic matter amendments and soils	2019
8255	V. Llaneza	Polyvinylpyrrolidone and arsenic-induced changes in biological responses of model aquatic organisms exposed to iron-based nanoparticles	2016
8256	E. Uchida	Experimental investigation of the simultaneous partitioning of divalent cations between lollingite or safflorite and 2 moUL aqueous chloride solutions under supercritical conditions	2020
8257	C. J. Da-Silva	Oxidative stress triggered by arsenic in a tropical macrophyte is alleviated by endogenous and exogenous nitric oxide	2018
8258	N. Colombani	Fate of arsenic, phosphate and ammonium plumes in a coastal aquifer affected by saltwater intrusion	2015
8259	G. V. Zakharov	Obtaining of Ligatures from Manganese Production Wastes by Self-Propagating High-Temperature Synthesis	2021
8260	S. Obiri	Levels of arsenic, mercury, cadmium, copper, lead, zinc and manganese in serum and whole blood of resident adults from mining and non-mining communities in Ghana	2016

8261	T. Rahmatinia	The effect of PM2.5-related hazards on biomarkers of bronchial epithelial cells (A549) inflammation in Karaj and Fardis cities	2022
8262	A. H. Hong	Examining molluscs as bioindicators of shrimp aquaculture effluent contamination in a southeast Asian mangrove	2020
8263	J. L. M. Viana	In situ arsenic speciation at the soil/water interface of saline-alkaline lakes of the Pantanal, Brazil: A DGT-based approach	2022
8264	N. F. Yang	Comparison of arsenic and molybdenum geochemistry in meromictic lakes of the McMurdo Dry Valleys, Antarctica: Implications for oxyanion-forming trace element behavior in permanently stratified lakes	2015
8265	N. Meisser	Richardsollyite, TlPbAsS3, a new sulfosalt from the Lengenbach quarry, Binn Valley, Switzerland	2017
8266	M. Mullard	Sonic Hedgehog Signature in Pediatric Primary Bone Tumors: Effects of the GLI Antagonist GANT61 on Ewing's Sarcoma Tumor Growth	2020
8267	H. T. Yu	Distribution, bioavailability, and human health risk assessment of arsenic in groundwater-soil-rice system in the Jianghan Plain, Central China	2022
8268	B. Tsoi	Neuroprotective Effects and Hepatorenal Toxicity of Angong Niuhuang Wan Against Ischemia-Reperfusion Brain Injury in Rats	2019
8269	Z. Mazej	Synthesis and crystal structures of HgFAsF6, Hg(HF)(2)(AsF6)(2), Hg(HF)(AsF6)(2) and Hg(AsF6)(SO3F)	2015
8270	R. J. Cheng	Arsenic Removal from Arsenopyrite-Bearing Iron Ore and Arsenic Recovery from Dust Ash by Roasting Method	2019
8271	S. Janvier	Impurity profiling of the most frequently encountered falsified polypeptide drugs on the Belgian market	2018
8272	V. V. Brazhkin	High-precision measurements of the compressibility and the electrical resistivity of bulk g-As2Te3 glasses at a hydrostatic pressure up to 8.5 GPa	2017
8273	J. C. Xia	Mass balance of nine trace elements in two karst catchments in southwest China	2021
8274	F. Pasquali	Listeria monocytogenes Sequence Types 121 and 14 Repeatedly Isolated Within One Year of Sampling in a Rabbit Meat Processing Plant: Persistence and Ecophysiology	2018
8275	X. J. Xie	Pathways of arsenic from sediments to groundwater in the hyporheic zone: Evidence from an iron isotope study	2014
8276	N. R. Pradhan	Raman and electrical transport properties of few-layered arsenic-doped black phosphorus	2019
8277	X. L. An	Oxidative dissolution of amorphous FeS and speciation of secondary Fe minerals: Effects of pH and As(III) concentration	2017
8278	J. Kim	Application of iron-modified biochar for arsenite removal and toxicity reduction	2019

8279	E. B. Simsek	Novel composite sorbents based on carbon fibers decorated with ferric hydroxides - simultaneous removal of antimonate and arsenate from aqueous solutions	2019
8280	H. S. Gill	Oral arsenic trioxide, all-trans retinoic acid, and ascorbic acid maintenance after first complete remission in acute promyelocytic leukemia: Long-term results and unique prognostic indicators	2020
8281	Y. H. Yuan	Electrochemical assay for As (III) by combination of highly thiol-rich trithiocyanuric acid and conductive reduced graphene oxide nanocomposites	2018
8282	C. Li	Removal of arsenic from dusts produced during the pyrometallurgical refining of copper by vacuum carbothermal reduction	2021
8283	Y. Lu	Alkali dilution of blood samples for high throughput ICP-MS analysis-comparison with acid digestion	2015
8284	B. L. Wu	Surface Functional Group Engineering of CeO2 Particles for Enhanced Phosphate Adsorption	2020
8285	J. Hu	Adsorption of roxarsone by iron (hydr)oxide-modified multiwalled carbon nanotubes from aqueous solution and its mechanisms	2014
8286	K. T. Peter	Functionalized polymer-iron oxide hybrid nanofibers: Electrospun filtration devices for metal oxyanion removal	2017
8287	H. U. So	Arsenite adsorption controlled by the iron oxide content of Holocene Red River aquifer sediment	2018
8288	A. Karthika	Electrochemical behaviour and voltammetric determination of mercury (II) ion in cupric oxide/poly vinyl alcohol nanocomposite modified glassy carbon electrode	2019
8289	C. Xu	In situ low temperature As-doping of Ge films using As(SiH3)(3) and As(GeH3)(3): fundamental properties and device prototypes	2015
8290	Y. Liu	Effect of peracetic acid as a depressant on the flotation separation of chalcopyrite from arsenopyrite	2022
8291	O. Oberemok	Stimulated oxygen impurity gettering under ultra-shallow junction formation in silicon	2014
8292	M. M. Du	Effect of pyrite on enhancement of zero-valent iron corrosion for arsenic removal in water: A mechanistic study	2019
8293	M. Berube	The fate of arsenic in groundwater discharged to the Meghna River, Bangladesh	2018
8294	I. Y. Mittova	Preparation of Dielectric Films via Thermal Oxidation of MnO2/GaAs	2018
8295	G. Lofrano	Polymer functionalized nanocomposites for metals removal from water and wastewater: An overview	2016
8296	S. Banerjee	Synthesis and application of Zn/Ce bimetallic oxides for the decontamination of arsenite (As-III) ions from aqueous solutions	2019

8297	C. Q. He	Synthesis of segregative, reusable, and high-efficiency spherical chitosan/zirconium macro-biocomposite for removal of aqueous As(V)	2020
8298	M. T. H. Siddiqui	Synthesis of magnetic carbon nanocomposites by hydrothermal carbonization and pyrolysis	2018
8299	J. G. Yang	Separation of Silver from a Zinc Sulfide Concentrate by a Co-smelting Process	2015
8300	C. Zhang	Phase transformation of crystalline iron oxides and their adsorption abilities for Pb and Cd	2016
8301	T. Guo	Mechanism of Cd(II) and Cu(II) Adsorption onto Few-Layered Magnetic Graphene Oxide as an Efficient Adsorbent	2021
8302	M. T. Ma	Metal Effects on the Asymmetric Cycloaddition Reaction between 3,4-Dimethyl-1-phenylarsole and Diphenylvinylphosphine Oxide	2009
8303	S. H. Pak	Adsorption behavior of poly(methacrylic acid)/iron-oxide-coated zeolite for the removal of Mn(II), Fe(II), and As(III) from aqueous solution	2018
8304	K. W. Juang	Assessing human health risk of arsenic for rice consumption by an iron plaque based partition ratio model	2021
8305	L. Khanikar	Groundwater appraisal of Dhekiajuli, Assam, India: an insight of agricultural suitability and arsenic enrichment	2017
8306	B. G. Singidas	Graphene transfer passivates GaAs	2020
8307	M. Kumar	Coupling fractionation and batch desorption to understand arsenic and fluoride co-contamination in the aquifer system	2016
8308	J. N. Li	Arsenic immobilization and removal in contaminated soil using zero-valent iron or magnetic biochar amendment followed by dry magnetic separation	2021
8309	X. X. Wang	Elucidating the Effects of Cerium Oxide Nanoparticles and Zinc Oxide Nanoparticles on Arsenic Uptake and Speciation in Rice (Oryza sativa) in a Hydroponic System	2018
8310	S. G. Johnston	Arsenic solid-phase speciation in an alluvial aquifer system adjacent to the Himalayan forehills, Nepal	2015
8311	Z. Y. Xie	Electrochemical Detection of As(III) by a rGO/Fe3O4-modified Screen-Printed Carbon Electrode	2016
8312	S. Y. Ding	Distribution and speciation of arsenic in seasonally stratified reservoirs: Implications for biotransformation mechanisms governing interannual variability	2022
8313	X. Y. He	Exceptional adsorption of arsenic by zirconium metal-organic frameworks: Engineering exploration and mechanism insight	2019
8314	S. Kundu	Eugenol and carvone as relaxants of arsenic and mercury hypercontracted rat trachea	2016

8315	W. Xiu	Linking microbial community composition to hydrogeochemistry in the western Hetao Basin: Potential importance of ammonium as an electron donor during arsenic mobilization	2020
8316	L. Yan	Organic-matter composition and microbial communities as key indicators for arsenic mobility in groundwater aquifers: Evidence from PLFA and 3D fluorescence	2020
8317	C. L. DeVore	Mobilization of As, Fe, and Mn from Contaminated Sediment in Aerobic and Anaerobic Conditions: Chemical or Microbiological Triggers?	
8318	A. Nabi	A comprehensive review of adaptations in plants under arsenic toxicity: Physiological, metabolic and molecular interventions	2021
8319	Y. P. Zhao	Roxarsone transformation and its impacts on soil enzyme activity in paddy soils: A new insight into water flooding effects	2021
8320	M. Panthri	Facets of iron in arsenic exposed Oryza sativa varieties: A manifestation of plant's adjustment at morpho-biochemical and enzymatic levels	2019
8321	X. N. Wang	Amplification of arsenic genotoxicity by TiO2 nanoparticles in mammalian cells: new insights from physicochemical interactions and mitochondria	2017
8322	S. Das	Soil microbial response to silicate fertilization reduces bioavailable arsenic in contaminated paddies	2021
8323	H. B. Kim	Effect of dissolved organic carbon from sludge, Rice straw and spent coffee ground biochar on the mobility of arsenic in soil	2018
8324	K. Sanyal	Arsenic quantification and speciation at trace levels in natural water samples by total reflection X-ray fluorescence after pre-concentration with N-methyl-D-glucamine functionalized quartz supports	2020
8325	M. Kofronova	Multi-Component Antioxidative System and Robust Carbohydrate Status, the Essence of Plant Arsenic Tolerance	2020
8326	Y. R. He	Novel thin-film composite nanofiltration membranes consisting of a zwitterionic co-polymer for selenium and arsenic removal	2018
8327	I. Jerzykowska	Mineralogy and speciation of Zn and As in Fe-oxide-clay aggregates in the mining waste at the MVT Zn-Pb deposits near Olkusz, Poland	2014
8328	C. Gogoi	Removal of Fluoride from Water by Locally Available Sand Modified with a Coating of Iron Oxides	2018
8329	U. M. Sutopo	A Role of Mineral Oxides on Trace Elements Behavior during Pulverized Coal Combustion	2021
8330	B. J. Abu Tarboush	Metal-Organic Framework-74 for Ultratrace Arsenic Removal from Water: Experimental and Density Functional Theory Studies	2018
8331	E. A. Soria	Immunotoxicological effects of arsenic bioaccumulation on spatial metallomics and cellular enzyme response in the spleen of male Wistar rats after oral intake	2017
8332	M. R. Gao	Investigation of the combined use of capping and oxidizing agents in the immobilization of arsenic in sediments	2021

8333	Q. T. Shi	Competing Interactions of As Adsorption and Fe(III) Polymerization during Ferric Coprecipitation Treatment	2018
8334	L. R. Brunson	Methods for Optimizing Activated Materials for Removing Fluoride from Drinking Water Sources	2016
8335	M. Xiao	Enhanced photocatalytic oxidation of As(III) by TiO2 modified with Fe3O4 through Ti - O-Fe interface bonds	2022
8336	S. C. Lou	Enhanced removal of As(III) and As(V) from water by a novel zirconium-chitosan modified spherical sodium alginate composite	2021
8337	J. X. Li	Improving the Reactivity of Zerovalent Iron by Taking Advantage of Its Magnetic Memory: Implications for Arsenite Removal	2015
8338	S. Bhowmick	Montmorillonite-supported nanoscale zero-valent iron for removal of arsenic from aqueous solution: Kinetics and mechanism	2014
8339	J. H. Wang	Effective immobilization of arsenic in waters and sediments using novel zirconium-loaded lanthanum-modified bentonite capping	2022
8340	T. Y. Kim	Biogenic Realgar As4S4 Molecular Clusters Formed by a One-Pot Microbial-Driven Process as a Li-Ion Storage Material	2017
8341	Y. H. Li	Nanocomposites of immobilized nano-zirconia on low-cost activated carbon derived from hazelnut shell for enhanced removal of 3-Nitro-4-Hydroxy-Phenylarsonic acid from water	2022
8342	J. Ma	Remediation of Arsenic contaminated soil using malposed intercropping of Pteris vittata L. and maize	2018
8343	J. B. Huo	Porous walnut-like La2O2CO3 derived from metal-organic frameworks for arsenate removal: A study of kinetics, isotherms, and mechanism	2021
8344	M. S. Karmacharya	Removal of As(III) and As(V) using rubber tire derived activated carbon modified with alumina composite	2016
8345	W. B. Xie	Controlling phosphate releasing from poultry litter using stabilized Fe-Mn binary oxide nanoparticles	2016
8346	T. J. Mohajerin	Geochemistry of Tungsten and Arsenic in Aquifer Systems: A Comparative Study of Groundwaters from West Bengal, India, and Nevada, USA	2014
8347	T. Y. Sun	Efficient degradation of p-arsanilic acid with arsenic adsorption by magnetic CuO-Fe3O4 nanoparticles under visible light irradiation	2018
8348	F. Muhammad	Lethal drug combination: Arsenic loaded multiple drug mesoporous silica for theranostic applications	2014
8349	P. S. Kumar	Arsenic adsorption by iron-aluminium hydroxide coated onto macroporous supports: Insights from X-ray absorption spectroscopy and comparison with granular ferric hydroxides	2016
8350	B. Barik	Ionic liquid assisted mesoporous silica-graphene oxide nanocomposite synthesis and its application for removal of heavy metal ions from water	2020

8351	P. Y. Zhang	Contrasting effects of dry-wet and freeze-thaw aging on the immobilization of As in As-contaminated soils amended by zero-valent iron-embedded biochar	2022
8352	L. N. Lin	Arsenic volatilization in flooded paddy soil by the addition of Fe-Mn-modified biochar composites	2019
8353	F. B. Ono	Investigation of arsenic species in tailings and windblown dust from a gold mining area	2016
8354	B. Mohapatra	Geochemical, metagenomic, and physiological characterization of the multifaceted interaction between microbiome of an arsenic contaminated groundwater and aquifer sediment	2021
8355	V. Nagarajan	Adsorptive Removal of As(V) from Aqueous Solution onto Steel Slag Recovered Iron - Chitosan Composite: Response Surface Modeling and Kinetics	2021
8356	C. Lobo	Functional iron chitosan microspheres synthesized by ionotropic gelation for the removal of arsenic (V) from water	2020
8357	T. Y. Sun	Efficient degradation of p-arsanilic acid with released arsenic removal by magnetic CeO2-Fe3O4 nanoparticles through photo-oxidation and adsorption	2019
8358	M. Yang	Reliable electrochemical sensing arsenic(III) in nearly groundwater pH based on efficient adsorption and excellent electrocatalytic ability of AuNPs/CeO2-ZrO2 nanocomposite	2018
8359	G. Ungureanu	Arsenic and antimony in water and wastewater: Overview of removal techniques with special reference to latest advances in adsorption	2015
8360	P. Nookongbut	Reduction in arsenic toxicity and uptake in rice (Oryza sativa L.) by As-resistant purple nonsulfur bacteria	2018
8361	M. Quemeneur	Bacterial Community Structure and Functional arrA Gene Diversity Associated with Arsenic Reduction and Release in an Industrially Contaminated Soil	2016
8362	C. B. Tabelin	Characterization and evaluation of arsenic and boron adsorption onto natural geologic materials, and their application in the disposal of excavated altered rock	2014
8363	P. M. Nekhungu ni	Investigation of As(V) removal from acid mine drainage by iron (hydr) oxide modified zeolite	2017
8364	Natasha	Hydrogeochemical and health risk evaluation of arsenic in shallow and deep aquifers along the different floodplains of Punjab, Pakistan	2021
8365	P. Colomban	Pigments and glassy matrix of the 17th-18th century enamelled French watches: A non-invasive on-site Raman and pXRF study	2020
8366	T. Custodio	Ranking nano-enabled hybrid media for simultaneous removal of contaminants with different chemistries: Pseudo-equilibrium sorption tests versus column tests	2017
8367	A. Emamverdian	Zinc Oxide Nanoparticles Improve Pleioblastus pygmaeus Plant Tolerance to Arsenic and Mercury by Stimulating Antioxidant Defense and Reducing the Metal Accumulation and Translocation	2022
8368	A. Baranik	Determination and speciation of ultratrace arsenic and chromium species using aluminium oxide supported on graphene oxide	2018

8369	J. W. Zhao	Arsenic (III) removal by mechanochemically sulfidated microscale zero valent iron under anoxic and oxic conditions	2021
8370	J. S. Yang	Removal of As(III) and As(V) using iron-rich sludge produced from coal mine drainage treatment plant	2014
8371	M. Suhel	GABA Requires Nitric Oxide for Alleviating Arsenate Stress in Tomato and Brinjal Seedlings	
8372	K. E. Greenstein	Performance comparison of hematite (alpha-Fe2O3)-polymer composite and core-shell nanofibers as point-of-use filtration platforms for metal sequestration	2019
8373	K. Wu	Evaluation of Al-Based Nanoparticle-Impregnated Sawdust as an Adsorbent from Byproduct for the Removal of Arsenic(V) from Aqueous Solutions	2017
8374	S. K. Back	The effect of NaOH for the recovery of elemental mercury from simulated mixture wastes and waste sludge from an industrial process using a thermal desorption process	2020
8375	B. Y. Ren	Electrochemical Detection of As (III) on a Manganese Oxide-Ceria (Mn2O3/CeO2) Nanocube Modified Au Electrode	2018
8376	S. Chakraborty	Predicting soil arsenic pools by visible near infrared diffuse reflectance spectroscopy	2017
8377	M. Wei	Remediation of sandy soil contaminated by heavy metals with Na(2)EDTA washing enhanced with organic reducing agents: element distribution and spectroscopic analysis	2018
8378	P. Leksungnoen	Biochar and ash derived from silicon-rich rice husk decrease inorganic arsenic species in rice grain	2019
8379	M. Li	Recent advances in application of iron-manganese oxide nanomaterials for removal of heavy metals in the aquatic environment	2022
8380	Y. Ling	Interaction mechanism between gaseous arsenic and the unburned carbon in coal-fired fly ash: A DFT combined thermodynamics study	2021
8381	T. T. Q. Nguyen	Removing arsenate from water using modified manganese oxide ore: Column adsorption and waste management	2020
8382	J. P. Green	An Air-Stable Semiconducting Polymer Containing Dithieno 3,2-b:2 ',3 '-d arsole	2016
8383	W. B. Xin	Effect of arsenic on the high temperature oxidation and hot shortness behaviour of copper-bearing steel	2022
8384	T. A. Ngantcha-Kwimi	Competitive Adsorption of As(III), As(V), and PO(4)by an Iron Oxide Impregnated Activated Carbon: Surface Complex Modeling	2020
8385	X. N. Feng	Arsenopyrite weathering in acid rain: Arsenic transfer and environmental implications	2021
8386	R. A. Dar	Green synthesis of a silver nanoparticle-graphene oxide composite and its application for As(III) detection	2014

8387	L. Li	Monolithic Fe2O3/graphene hybrid for highly efficient lithium storage and arsenic removal	2014
8388	X. L. Cai	Arsenate-reducing bacteria-mediated arsenic speciation changes and redistribution during mineral transformations in arsenate-associated goethite	2020
8389	H. Qiu	Column adsorption of Cu(II) by polymer-supported nano-iron oxides in the presence of sulfate: Experimental and mathematical modeling	2017
8390	M. Yang	Enhanced electrochemical sensing arsenic(III) with excellent anti-interference using amino-functionalized graphene oxide decorated gold microelectrode: XPS and XANES evidence	2017
8391	H. K. Goswami	Pteridophytes: evolutionary boon as medicinal plants	2016
8392	M. S. Kim	Assessment of Fraction and Mobility of Arsenic in Soil Near the Mine Waste Dam	2020
8393	M. Imran	Synthesis, characterization and application of novel MnO and CuO impregnated biochar composites to sequester arsenic (As) from water: Modeling, thermodynamics and reusability	2021
8394	A. Agnihotri	Exogenously applied nitrate improves the photosynthetic performance and nitrogen metabolism in tomato (Solanum lycopersicum L. cv Pusa Rohini) under arsenic (V) toxicity	2016
8395	E. V. Tomina	Effect of surface V2O5 nanolayers on the thermal oxidation kinetics of GaAs and the composition and morphology of resulting films	2015
8396	M. K. Yadav	Modeling and analysis of adsorptive removal of arsenite by Mg-Fe-(CO3) layer double hydroxide with its application in real-life groundwater	2019
8397	A. Ghosh	Morphology Controlled Fabrication of Highly Permeable Carbon Coated Rod-Shaped Magnesium Oxide as a Sustainable Arsenite Adsorbent	2019
8398	L. Alidokht	Recent advances in the application of nanomaterials for the remediation of arsenic-contaminated water and soil	2021
8399	L. W. Yin	Simultaneous removal of arsenite and arsenate from mining wastewater using ZIF-8 embedded with iron nanoparticles	2022
8400	A. Suda	Inhibitory effect of active aluminum on arsenic solubility in anaerobic soils	2021
8401	T. E. D. Lazarini	Canned sardines commercialized in Brazil: Packaging and inorganic contaminants evaluation	2019
8402	Y. D. Xue	Reinforced As(III) oxidation by the in-situ electro-generated hydrogen peroxide on MoS2 ultrathin nanosheets modified carbon felt in alkaline media	2017
8403	L. X. Yao	Soil calcium significantly promotes uptake of inorganic arsenic by garland chrysanthemum (ChrysanthemumL coronarium) fertilized with chicken manure bearing roxarsone and its metabolites	2017
8404	J. Zhang	Arsenic Methylation and Volatilization by Arsenite S-Adenosylmethionine Methyltransferase in Pseudomonas alcaligenes NBRC14159	2015

8405	T. Boschetti	CO2 Inflow and Elements Desorption Prior to a Seismic Sequence, Amatrice-Norcia 2016, Italy	2019
8406	K. Y. Kum	Trace metal contents of three tricalcium silicate materials: MTA Angelus, Micro Mega MTA and Bioaggregate	2014
8407	X. S. Nguyen	Photo-oxidation efficient As (III) under visible light using microporous Zn doped-Fe3O4 sphere	2020
8408	A. Hartland	Phosphorus and arsenic distributions in a seasonally stratified, iron- and manganese-rich lake: microbiological and geochemical controls	2015
8409	P. M. Amaibi	Mineralogy, solid-phase fractionation and chemical extraction to assess the mobility and availability of arsenic in an urban environment	2019
8410	S. Scurti	Superparamagnetic Iron Oxide Nanoparticle Nanodevices Based on Fe3O4 Coated by Megluminic Ligands for the Adsorption of Metal Anions from Water	2022
8411	M. B. Gumpu	A review on detection of heavy metal ions in water - An electrochemical approach	2015
8412	J. G. Hering	Arsenic Removal from Drinking Water: Experiences with Technologies and Constraints in Practice	2017
8413	X. L. Otero	Geographical variations in arsenic contents in rice plants from Latin America and the Iberian Peninsula in relation to soil conditions	2020
8414	P. N. Mandaliev	Arsenic Species Formed from Arsenopyrite Weathering along a Contamination Gradient in Circumneutral River Floodplain Soils	2014
8415	A. M. A. Pintor	Arsenate and arsenite adsorption onto iron-coated cork granulates	2018
8416	F. J. Pereira	Effect of temperature on the crystallinity, size and fluorescent properties of zirconia-based nanoparticles	2015
8417	K. K. Jinadasa	Smart materials for mercury and arsenic determination in food and beverages	2022
8418	A. D. Gupta	Batch and continuous reactor studies for the adsorption of As(III) from wastewater using a hybrid biochar loaded with transition metal oxides: Kinetics and mass transfer analysis	2021
8419	S. U. Khan	Application of Fe-Cu binary oxide nanoparticles for the removal of hexavalent chromium from aqueous solution	2016
8420	G. X. Huang	Groundwater is important for the geochemical cycling of phosphorus in rapidly urbanized areas: a case study in the Pearl River Delta	2020
8421	J. Jin	Arsenic release from Floridan Aquifer rock during incubations simulating aquifer storage and recovery operations	2016
8422	Y. L. Wang	Toxicity of arsenite to earthworms and subsequent effects on soil properties	2018

8423	H. Neidhardt	Insights into arsenic retention dynamics of Pleistocene aquifer sediments by in situ sorption experiments	2018
8424	Y. M. Dong	As(III) adsorption onto different-sized polystyrene microplastic particles and its mechanism	2020
8425	M. Zeeshan	Interplay of ZnONPs and/or SeNPs induces arsenic tolerance in soybean by regulation of antioxidants pool, WRKY genes, and expression of arsenic transporters	2022
8426	Z. B. Xu	Unraveling iron speciation on Fe-biochar with distinct arsenic removal mechanisms and depth distributions of As and Fe	2021
8427	D. Wichelns	Managing Water and Soils to Achieve Adaptation and Reduce Methane Emissions and Arsenic Contamination in Asian Rice Production	2016
8428	M. Burachevskaya	Accumulation, translocation, and toxicity of arsenic in barley grown in contaminated soil	2021
8429	M. Gifford	Ranking traditional and nano-enabled sorbents for simultaneous removal of arsenic and chromium from simulated groundwater	2017
8430	M. K. Yadav	pH mediated facile preparation of hydrotalcite based adsorbent for enhanced arsenite and arsenate removal: Insights on physicochemical properties and adsorption mechanism	2017
8431	X. Liu	Microbial siderophores and root exudates enhanced goethite dissolution and Fe/As uptake by As-hyperaccumulator Pteris vittata	2017
8432	L. Panda	Characterization and utilization of coal fly ash: a review	2020
8433	Y. Luo	Facile crystal-structure-controlled synthesis of iron oxides for adsorbents and anode materials of lithium batteries	2016
8434	M. Shafiquzzaman	Evaluating configuration of dual unit ceramic filter for arsenic removal from highly contaminated groundwater	2022
8435	Y. Li	Spatial variation in dissolved phosphorus and interactions with arsenic in response to changing redox conditions in floodplain aquifers of the Hetao Basin, Inner Mongolia	2022
8436	U. K. Sahu	Cigarette soot activated carbon modified with Fe(3)o(4) nanoparticles as an effective adsorbent for As(III) and As(V): Material preparation, characterization and adsorption mechanism study	2017
8437	X. Zhang	Delineating the controlling mechanisms of arsenic release into groundwater and its associated health risks in the Southern Loess Plateau, China	2022
8438	S. Bagherifam	In situ stabilization of As and Sb with naturally occurring Mn, Al and Fe oxides in a calcareous soil: Bioaccessibility, bioavailability and speciation studies	2014
8439	D. W. Wu	Experimental and theoretical study of arsenic removal by porous carbon from MSW incineration flue gas	2022
8440	A. Dradrach	Arsenic accumulation by red fescue (Festuca rubra) growing in mine affected soils - Findings from the field and greenhouse studies	2020

8441	K. Northrup	Effects of Extreme Events on Arsenic Cycling in Salt Marshes	2018
8442	V. Singh	Isolation and Characterization of Microglia from Adult Mouse Brain: Selected Applications for ex Vivo Evaluation of Immunotoxicological Alterations Following in Vivo Xenobiotic Exposure	2014
8443	J. S. He	A novel Zr-based nanoparticle-embedded PSF blend hollow fiber membrane for treatment of arsenate contaminated water: Material development, adsorption and filtration studies, and characterization	2014
8444	N. Mushtaq	Health risk assessment and source identification of groundwater arsenic contamination using agglomerative hierarchical cluster analysis in selected sites from upper Eastern parts of Punjab province, Pakistan	2021
8445	M. R. Robinson	As(V) and As(III) sequestration by starch functionalized magnetite nanoparticles: influence of the synthesis route onto the trapping efficiency	2020
8446	A. G. Meselhy	Nanoscale Sulfur Improves Plant Growth and Reduces Arsenic Toxicity and Accumulation in Rice (Oryza sativa L.)	2021
8447	M. Glodowsk a	Fermentation, methanotrophy and methanogenesis influence sedimentary Fe and As dynamics in As-affected aquifers in Vietnam	2021
8448	E. Shabani	Removal of Arsenic and Copper from Water Solution Using Magnetic Iron/Bentonite Nanoparticles (Fe3O4/Bentonite)	2019
8449	S. S. Sathe	Simultaneous influence of indigenous microorganism along with abiotic factors controlling arsenic mobilization in Brahmaputra floodplain, India	2018
8450	A. Deonarine	Mobilization of arsenic from coal fly ash in the presence of dissolved organic matter	2021
8451	T. Ponnugou nder	Natural occurrence of arsenic in the soil and rice plant system in the Bashkandi Block of Barak Valley, Assam, Northeastern India	2020
8452	S. X. He	Using rice as a remediating plant to deplete bioavailable arsenic from paddy soils	2020
8453	A. L. Seyfferth	On the Use of Silicon as an Agronomic Mitigation Strategy to Decrease Arsenic Uptake by Rice	2018
8454	X. X. Huang	Mechanisms for As(OH)(3) and H3AsO4 adsorption at anhydrous and hydrated surfaces of gibbsite and possibility for anionic As(III) and As(V) formation	2020
8455	K. Yang	Effect of biogeochemical interactions on bioaccessibility of arsenic in soils of a former smelter site in Republic of Korea	2016
8456	A. Biswas	Role of competing ions in the mobilization of arsenic in groundwater of Bengal Basin: Insight from surface complexation modeling	2014
8457	S. Balarastag hi	Melatonin improves arsenic-induced hypertension through the inactivation of the Sirt1/autophagy pathway in rat	2022
8458	R. Khosravi	Early diagenetic behavior of arsenic in the sediment of the hypersaline Maharlu Lake, southern Iran	2019

8459	N. E. Nieva	Mineralogical control on arsenic release during sediment-water interaction in abandoned mine wastes from the Argentina Puna	2016
8460	C. W. Liu	Assessing and simulating the major pathway and hydrogeochemical transport of arsenic in the Beitou-Guandu area, Taiwan	2016
8461	Y. F. Wei	Fast and efficient removal of As(III) from water by CuFe2O4 with peroxymonosulfate: Effects of oxidation and adsorption	2019
8462	T. Li	Enhanced Hydrolysis of p-Nitrophenyl Phosphate by Iron (Hydr)oxide Nanoparticles: Roles of Exposed Facets	2020
8463	Z. C. Ding	Novel mesoporous Fe-Al bimetal oxides for As(III) removal: Performance and mechanism	2017
8464	G. Yadav	Light Intensity Alters the Extent of Arsenic Toxicity in Helianthus annuus L. Seedlings	2014
8465	R. Siddique	Arsenic Trioxide-based Nanomedicines as a Therapeutic Combination Approach for Treating Gliomas: A Review	2021
8466	Z. G. Yu	Transport of the arsenic (As)-loaded nano zero-valent iron in groundwater-saturated sand columns: Roles of surface modification and As loading	2019
8467	M. Kumar	Hydrogeo-morphological influences for arsenic release and fate in the central Gangetic Basin, India	2018
8468	T. Y. Sun	Efficient As(III) removal by magnetic CuO-Fe(3)o(4) nanoparticles through photo-oxidation and adsorption under light irradiation	2017
8469	T. H. Bui	Performance analysis of hydrated Zr(IV) oxide nanoparticle-impregnated anion exchange resin for selective phosphate removal	2021
8470	P. R. Sharma	Efficient Removal of Arsenic Using Zinc Oxide Nanocrystal-Decorated Regenerated Microfibrillated Cellulose Scaffolds	2019
8471	H. A. Maitlo	Metal-air fuel cell electrocoagulation techniques for the treatment of arsenic in water	2019
8472	V. V. T. Padil	Electrospun membrane composed of poly acrylonitrile-co-(methyl acrylate)-co-(itaconic acid) terpolymer and ZVI nanoparticles and its application for the removal of arsenic from water	2016
8473	P. C. Pandey	Organotrialkoxysilane-Functionalized Prussian Blue Nanoparticles-Mediated Fluorescence Sensing of Arsenic(III)	2021
8474	K. D. Brahman	Biosorptive removal of inorganic arsenic species and fluoride from aqueous medium by the stem of Tecomella undulate	2016
8475	Y. Cha	Bayesian modeling approach for characterizing groundwater arsenic contamination in the Mekong River basin	2016
8476	A. Romero-Freire	Toxicity of arsenic in relation to soil properties: implications to regulatory purposes	2014

8477	N. Sankararamakrishna	Synthesis of functionalized carbon nanotubes by floating catalytic chemical vapor deposition method and their sorption behavior toward arsenic	2016
8478	A. Abu-Nada	Recent Advances in Applications of Hybrid Graphene Materials for Metals Removal from Wastewater	2020
8479	S. Lan	Efficient catalytic As(III) oxidation on the surface of ferrihydrite in the presence of aqueous Mn(II)	2018
8480	M. F. Hamza	As(V) sorption from aqueous solutions using quaternized algal/polyethyleneimine composite beads	2020
8481	A. Ahangari	Capabilities of nickel zinc ferrite and its nanocomposite with CNT for adsorption of arsenic (V) ions from wastewater	2019
8482	J. Wragg	The Link between Soil Geochemistry in South-West England and Human Exposure to Soil Arsenic	2018
8483	A. Chiavola	Comparison of Adsorptive Removal of Fluoride from Water by Different Adsorbents under Laboratory and Real Conditions	2022
8484	A. N. Jijana	The Ultra-sensitive Electrochemical Detection of As(III) in Ground Water Using Disposable L-cysteine/Lipoic Acid Functionalised Gold Nanoparticle Modified Screen-Printed Electrodes	2021
8485	D. Abulizi	DIFFERENT ADSORPTION PATTERN OF MERCURY AND ARSENIC ON PRISTINE AND MnOx LOADED BIOCHAR	2020
8486	M. Kumar	Hydrogeochemical controls on mobilization of arsenic and associated health risk in Nagaon district of the central Brahmaputra Plain, India	2017
8487	J. M. Blake	Sediment record of mining legacy and water quality from a drinking-water reservoir, Aztec, New Mexico, USA	2020
8488	Z. Chen	A novel AQDS-rGO composite to enhance the bioreduction of As(v)/Fe(III) from the flooded arsenic-rich soil	2017
8489	X. J. Xie	Impact of sedimentary provenance and weathering on arsenic distribution in aquifers of the Datong basin, China: Constraints from elemental geochemistry	2014
8490	Z. L. Yang	Contradictory tendency of As(V) releasing from Fe-As complexes: Influence of organic and inorganic anions	2022
8491	M. Goudarzi	The possible neuroprotective effect of ellagic acid on sodium arsenate-induced neurotoxicity in rats	2018
8492	Y. Yao	In situ, high resolution ZrO-Chelex DGT for the investigation of iron-coupled inactivation of arsenic in sediments by macrozoobenthos bioturbation and hydrodynamic interactions	2016
8493	J. Y. Zhang	Enhanced Adsorption of Trivalent Arsenic from Water by Functionalized Diatom Silica Shells	2015
8494	T. Sivasothy	Voltammetric characteristics of hydrous Fe(III) oxide embedded into Nafion and immobilised onto a screen-printed carbon electrode: binding of arsenate versus phosphate	2018

8495	Y. R. Deng	Arsenate removal from underground water by polystyrene-confined hydrated ferric oxide (HFO) nanoparticles:effect of humic acid	2020
8496	S. Kumar	Graphene Oxide-MnFe2O4 Magnetic Nanohybrids for Efficient Removal of Lead and Arsenic from Water	2014
8497	V. N. Scheverin	Novel iron-based nanocomposites for arsenic removal in groundwater: insights from their synthesis to implementation for real groundwater remediation	2022
8498	W. J. Jin	Impacts of biochar and silicate fertilizer on arsenic accumulation in rice (Oryza sativa L.)	2020
8499	S. Pathan	Aggregation-induced enhanced photoluminescence in magnetic graphene oxide quantum dots as a fluorescence probe for As(iii) sensing	2019
8500	A. R. Lopez	Periphyton and abiotic factors influencing arsenic speciation in aquatic environments	2018
8501	F. Corsetti	A first-principles study of As doping at a disordered Si-SiO2 interface	2014
8502	H. Wei	Destabilization and exchange removal of arsenic in contaminated soils by washing: A new remediation strategy with high efficiency and low mineral loss	2022
8503	C. S. Zhi	Genesis of As in the groundwater with extremely high salinity in the Yellow River Delta, China	2022
8504	M. K. Upadhyay	Arsenic in Rice Agro-Ecosystem: Solutions for Safe and Sustainable Rice Production	2020
8505	Y. M. Dong	Adsorption mechanism of As(III) on polytetrafluoroethylene particles of different size	2019
8506	D. H. Xie	Bifunctional NH2-MIL-88(Fe) metal-organic framework nanooctahedra for highly sensitive detection and efficient removal of arsenate in aqueous media	2017
8507	C. Zhang	Synthesis and properties of a high-capacity iron oxide adsorbent for fluoride removal from drinking water	2017
8508	S. Y. Li	A novel fusion protein TBLR1-RAR alpha acts as an oncogene to induce murine promyelocytic leukemia: identification and treatment strategies	2021
8509	M. M. Hussain	The role of various ameliorants on geochemical arsenic distribution and CO2-carbon efflux under paddy soil conditions	
8510	C. S. Zhi	Spatial distribution of arsenic along groundwater flow path in Chaobai River alluvial-proluvial fan, North China Plain	2019
8511	H. T. Ha	Synthesis of Iron Oxide Nanoparticle Functionalized Activated Carbon and Its Applications in Arsenic Adsorption	2021
8512	L. J. Wilk	Adsorptive-Oxidative Removal of Sulfides from Water by MnO2-Loaded Carboxylic Cation Exchangers	2020

8513	J. R. Rivera-Hernandez	Geosorption of As(III) from Aqueous Solution by Red Clays: Kinetic Studies	2014
8514	C. Ganzel	Extramedullary disease in APL: A real phenomenon to contend with or not?	2014
8515	C. Pizarro	Evaluation of zeolite, nanomagnetite, and nanomagnetite-zeolite composite materials as arsenic (V) adsorbents in hydroponic tomato cultures	2021
8516	S. M. Baba	Influence of bcr-3 PML-RAR alpha transcript on outcome in Acute Promyelocytic Leukemia patients of Kashmir treated with all-trans retinoic acid and/or arsenic tri-oxide	2019
8517	Q. Xia	Bioaccessibility of arsenic and cadmium assessed for in vitro bioaccessibility in spiked soils and their interaction during the Unified BARGE Method (UBM) extraction	2016
8518	F. Fang	Facile one-pot preparation of goethite/parabutlerite nanocomposites and their removal properties and mechanism toward As(V) ions	2015
8519	R. Nishi	Oxidation of arsenite by self-regenerative bioactive birnessite in a continuous flow column reactor	2020
8520	Y. Yu	Introduction of an Yttrium-Manganese Binary Composite That Has Extremely High Adsorption Capacity for Arsenate Uptake in Different Water Conditions	2015
8521	P. Drahota	Selectivity assessment of an arsenic sequential extraction procedure for evaluating mobility in mine wastes	2014
8522	K. Holland	Upgrading copper slag cleaning tailings for re-use	2019
8523	O. F. Zaid	Synthesis of microporous nano-composite (hollow spheres) for fast detection and removal of As(V) from contaminated water	2020
8524	I. Shiue	IgE antibodies and urinary trimethylarsine oxide accounted for 1-7 % population attributable risks for eczema in adults: USA NHANES 2005-2006	2015
8525	L. Zhao	Soil migration of antimony and arsenic facilitated by colloids in lysimeter studies	2020
8526	P. Drahota	Arsenic mineralogy of near-neutral soils and mining waste at the Smolotely-Lisnice historical gold district, Czech Republic	2018
8527	T. Mahmood	Equilibrium, kinetics, mechanism and thermodynamics studies of As(Ill) adsorption from aqueous solution using iron impregnated used tea	2018
8528	M. S. Podder	SD/MnFe(2)0(4) composite, a biosorbent for As(III) and As(V) removal from wastewater: Optimization and isotherm study	2015
8529	Q. Zhang	Terpolymer Micelles for the Delivery of Arsenic to Breast Cancer Cells: The Effect of Chain Sequence on Polymeric Micellar Characteristics and Cancer Cell Uptake	2016
8530	Y. Ogawa	The fate of arsenic in a river acidified by volcanic activity and an acid thermal water and sedimentation mechanism	2014

8531	X. Wu	Digestive solubilization of particle-associated arsenate by deposit feeders: The roles of proteinaceous and surfactant materials	2019
8532	F. S. Farnese	The Involvement of Nitric Oxide in Integration of Plant Physiological and Ultrastructural Adjustments in Response to Arsenic	2017
8533	C. Miranda	Degradation of Organoarsenicals by Heterogeneous Photocatalysis using ZnO, TiO2 and UVA	2016
8534	R. Xiong	Facile synthesis of magnetic nanocomposites of cellulose@ultrasmall iron oxide nanoparticles for water treatment	2014
8535	S. Dutta	Environmental and occupational exposure of metals and female reproductive health	
8536	X. J. Hu	Effects of inorganic electrolyte anions on enrichment of Cu(II) ions with aminated Fe3O4/graphene oxide: Cu(II) speciation prediction and surface charge measurement	2015
8537	R. A. Kemkina	First Find of Trace Metal Minerals at the Albaza Gold Deposit	2018
8538	C. C. Almeida	Adsorption and desorption of arsenic and its immobilization in soils	2021
8539	L. N. Van	Removal mechanism of arsenic (V) by stainless steel slags obtained from scrap metal recycling	2020
8540	X. Q. Zhang	Anaerobic oxidation of methane mediated by microbial extracellular respiration	2021
8541	H. Jelenova	Arsenic-rich stalactites from abandoned mines: Mineralogy and biogeochemistry	2021
8542	C. V. Breton	Small-Magnitude Effect Sizes in Epigenetic End Points are Important in Children's Environmental Health Studies: The Children's Environmental Health and Disease Prevention Research Center's Epigenetics Working Group	2017
8543	H. M. Guo	Controls of organic matter bioreactivity on arsenic mobility in shallow aquifers of the Hetao Basin, PR China	2019
8544	B. Chen	Nanocasted synthesis of ordered mesoporous cerium iron mixed oxide and its excellent performances for As(V) and Cr(VI) removal from aqueous solutions	2014
8545	A. Nashine	Iron oxide impregnated sugarcane bagasse waste material as sorbent for As(III) removal from water: kinetic, equilibrium and thermodynamic studies	2016
8546	D. M. Jia	Effective removal of glyphosate from water by resin-supported double valent nano-sized hydroxyl iron oxide	2017
8547	S. J. Gibson	Model of patterned self-assisted nanowire growth	2014
8548	E. Alam	Synthesis of Fe3O4@mZrO(2)-Re (Re = Y/La/Ce) by Using Uniform Design, Surface Response Methodology, and Orthogonal Design & Its Application for As3+ and As5+ Removal	2021

8549	A. Dia	Bacteria-mediated reduction of As(V)-doped lepidocrocite in a flooded soil sample	2015
8550	S. R. Chowdhury	Application of recycling waste products for ex situ and in situ water treatment methods	2020
8551	J. S. Li	Effects of low-alkalinity binders on stabilization/solidification of geogenic As-containing soils: Spectroscopic investigation and leaching tests	2018
8552	D. K. Singh	Modeling of adsorption behavior of the amine-rich GOPEI aerogel for the removal of As(III) and As(V) from aqueous media	2016
8553	C. Wang	In situ high-resolution evaluation of labile arsenic and mercury in sediment of a large shallow lake	2016
8554	D. Lee	A novel dielectrophoresis activated cell sorter (DACS) to evaluate the apoptotic rate of K562 cells treated with arsenic trioxide (As2O3)	2016
8555	A. Crepet	Integrating Selection and Risk Assessment of Chemical Mixtures: A Novel Approach Applied to a Breast Milk Survey	2022
8556	A. Sharma	Spatial statistical modeling of arsenic accumulation in microsites of diverse soils	2022
8557	A. M. A. Pintor	Complexation mechanisms in arsenic and phosphorus adsorption onto iron-coated cork granulates	2020
8558	S. Mehrzadi	Ellagic acid mitigates sodium arsenite-induced renal and hepatic toxicity in male Wistar rats	2018
8559	S. Tang	Magnetic core-shell iron(II,III) oxide@layered double oxide microspheres for removal of 2,5-dihydroxybenzoic acid from aqueous solutions	2015
8560	L. Y. Lin	Reducing Arsenic Concentration in Panax notoginseng via Contaminant Immobilization in Soil Using Fe-Ce Oxide	2018
8561	T. Frohne	Contamination of Floodplain Soils along theWupper River, Germany, with As, Co, Cu, Ni, Sb, and Zn and the Impact of Pre-definite Redox Variations on the Mobility of These Elements	2014
8562	M. Schabikowski	Rotary jet-spinning of hematite fibers	2015
8563	T. T. Sheng	Development, characterization and evaluation of iron-coated honeycomb briquette cinders for the removal of As(V) from aqueous solutions	2014
8564	M. M. N. Amin	Arsenic release in contaminated soil amended with unmodified and modified biochars derived from sawdust and rice husk	2020
8565	D. C. Liu	As(III) and As(V) adsorption on nanocomposite of hydrated zirconium oxide coated carbon nanotubes	2018
8566	S. A. Baig	Influence of calcination on magnetic honeycomb briquette cinders composite for the adsorptive removal of As(III) in fixed-bed column	2014

8567	D. Guaya	Simultaneous nutrients (N, P) removal by using a hybrid inorganic sorbent impregnated with hydrated manganese oxide	2017
8568	L. Yu	Magnetically-Confined Fe-Mn Bimetallic Oxide Encapsulation as an Efficient and Recoverable Adsorbent for Arsenic(III) Removal	2016
8569	S. Singh	Multifunctional nanohybrid for simultaneous detection and removal of Arsenic(III) from aqueous solutions	2022
8570	Y. Chen	Decreasing arsenic accumulation but promoting arsenate biotransformation in Microcystis aeruginosa regulated by nano-Fe2O3	
8571	C. W. Neil	Arsenite oxyanions affect CeO2 nanoparticle dissolution and colloidal stability	2021
8572	Y. F. Wei	Enhanced removal of As(III) by heterogeneous catalytic oxidation of As(III) on Fe-biochar fibers with H2O2 and hydroxylamine	2022
8573	H. C. Vu	Magnetite graphene oxide encapsulated in alginate beads for enhanced adsorption of Cr(VI) and As(V) from aqueous solutions: Role of crosslinking metal cations in pH control	2017
8574	N. Abrosimova	Assessment of the acid mine drainage potential of waste rocks at the Ak-Sug porphyry Cu-Mo deposit	2015
8575	X. Q. Wang	The translocation of antimony in soil-rice system with comparisons to arsenic: Alleviation of their accumulation in rice by simultaneous use of Fe(II) and NO3	2019
8576	D. Craw	Placer gold and associated supergene mineralogy at Macraes Flat, East Otago, New Zealand	2017
8577	M. Y. Gutekunst	Impacts of soil incorporation of pre-incubated silica-rich rice residue on soil biogeochemistry and greenhouse gas fluxes under flooding and drying	2017
8578	S. Bernardini	Raman spectra of natural manganese oxides	2019
8579	S. S. Li	Engineering multi-shell Mn-Co oxide for ultrasensitive electroanalysis of Pb(II) in mining subsidence area water with promotion of adsorption and electron mediation: Behaviors and mechanisms of Mn(II)/Mn(III) and Co(II)/Co(III) cycles	2020
8580	D. Montalvo	Efficient removal of arsenate from oxic contaminated water by colloidal humic acid-coated goethite: Batch and column experiments	2018
8581	Q. Guo	Hydrogeochemical contrasts between low and high arsenic groundwater and its implications for arsenic mobilization in shallow aquifers of the northern Yinchuan Basin, P.R. China	2014
8582	S. H. Wen	Electrochemical sensor for arsenite detection using graphene oxide assisted generation of prussian blue nanoparticles as enhanced signal label	2018
8583	G. N. Koptsik	The response of forest ecosystems to reduction in industrial atmospheric emission in the Kola Subarctic	2016
8584	J. Majzlan	Thermodynamic properties of tooeleite, Fe63+As3+O3)(4)(SO4)(OH)(4).4H(2)O	2016

8585	E. Kociolek-Balawejde	Iron(III) (hydr) oxide loaded anion exchange hybrid polymers obtained via tetrachloroferrate ionic form-Synthesis optimization and characterization	2017
8586	R. C. Miao	Synergistic effect of nutlin-3 combined with aspirin in hepatocellular carcinoma HepG2 cells through activation of Bcl-2/Bax signaling pathway	2018
8587	G. R. Helz	Activity of zero-valent sulfur in sulfidic natural waters	2014
8588	K. M. Adkison	Suitability of binary oxides for molecular-beam epitaxy source materials: A comprehensive thermodynamic analysis	2020
8589	R. Zaidi	Investigation of kinetics and adsorption isotherm for fluoride removal from aqueous solutions using mesoporous cerium-aluminum binary oxide nanomaterials	2021
8590	X. Zhang	Effects of oxygen-inserted layers on diffusion of boron, phosphorus, and arsenic in silicon for ultra-shallow junction formation	2018
8591	F. J. Pereira	Optical properties of ThO2-based nanoparticles	2017
8592	V. K. Nguyen	A comprehensive analysis of racial disparities in chemical biomarker concentrations in United States women, 1999-2014	2020
8593	A. Pholosi	Clean application of magnetic biomaterial for the removal of As (III) from water	2018
8594	J. Lee	Effect of Groundwater-Lake Interactions on Arsenic Enrichment in Freshwater Beach Aquifers	2014
8595	S. Bagchi	Comparison of the composition profiles of the low explosives in India from forensic exhibits and a brief discussion on preventive forensic techniques	2016
8596	V. Venkatachalam	Structural and electronic effects of alkyl substituents on the main group dithiocarbamato complexes: Crystal and molecular structures of tris(N,N'-iminodiethanoldithiocarbamato)metal(III) complexes (M=As, Sb and Bi) and cyclic	1997
8597	Q. Wang	Precise Targeting Therapy of Orthotopic Gastric Carcinoma by siRNA and Chemotherapeutic Drug Codelivered in pH-Sensitive Nano Platform	2021
8598	Z. H. Wang	Coprecipitated arsenate inhibits thermal transformation of 2-line ferrihydrite: Implications for long-term stability of ferrihydrite	2015
8599	F. F. Hanum	Preliminary Study on Additives for Controlling As, Se, B, and F Leaching from Coal Fly Ash	2018
8600	N. M. Khusayfan	Formation Mechanism, Structural and Optoelectronic Properties of As2Se3/CdS Heterojunctions Prepared by Physical Vapor Deposition Technique	2019
8601	M. S. Hosseini	A novel spectrofluorometric method for the determination of arsenic in human hair using Dy2O3-doped CeO2 nanoparticles	2014
8602	H. B. Wang	Arsenic concentration, speciation, and risk assessment in sediments of the Xijiang River basin, China	2019

8603	A. Sharma	Synthesis and characterization of anionic/nonionic surfactant-interceded iron-doped TiO2 to enhance sorbent/photo-catalytic properties	2015
8604	C. Lanctot	Behaviour, development and metal accumulation in striped marsh frog tadpoles (Limnodynastes peronii) exposed to coal mine wastewater	2016
8605	Y. X. Zhou	Controlled high-quality interface of a Ti2.5O3(010)/GaAs(001) heterostructure enabled by minimized lattice mismatch and suppressed ion diffusion	2020
8606	W. Zhang	Efficient oxidation and sorption of arsenite using a novel titanium(IV)-manganese(IV) binary oxide sorbent	2018
8607	Z. P. Wen	One-step facile hydrothermal synthesis of flowerlike Ce/Fe bimetallic oxides for efficient As(V) and Cr(VI) remediation: Performance and mechanism	2018
8608	M. Yamazaki-Nishioka	Human plasma metabolic profiles of benzydamine, a flavin-containing monooxygenase probe substrate, simulated with pharmacokinetic data from control and humanized-liver mice	2018
8609	S. Q. Kong	Competitive adsorption of humic acid and arsenate on nanoscale iron-manganese binary oxide-loaded zeolite in groundwater	2014
8610	I. Wallis	The river-groundwater interface as a hotspot for arsenic release	2020
8611	A. Ahmad	Impact of phosphate, silicate and natural organic matter on the size of Fe(III) precipitates and arsenate co-precipitation efficiency in calcium containing water	2020
8612	D. A. Almasri	High performance hydroxyiron modified montmorillonite nanoclay adsorbent for arsenite removal	2018
8613	Z. Michalkova	Selected Fe and Mn (nano)oxides as perspective amendments for the stabilization of As in contaminated soils	2016
8614	G. Zhang	Synthesis of tin oxide nanospheres under ambient conditions and their strong adsorption of As(III) from water	2015
8615	D. X. Kong	Synthesis and characterization of cellulose-goethite composites and their adsorption properties with roxarsone	2017
8616	J. Choi	Effects of arsenic implantation and rapid thermal annealing on ZnO nanorods for p-type doping	2020
8617	J. Kumpiene	LONG-TERM stability of arsenic in iron amended contaminated soil	2021
8618	B. N. Mahato	Rapid adsorption of As(V) from aqueous solution by ZnO embedded in mesoporous aluminosilicate nanocomposite adsorbent: Parameter optimization, kinetic, and isotherms studies	2021
8619	R. Biela	Monitoring the effectiveness of advanced sorption materials for removing selected metals from water	2017
8620	A. Ravikumar	MoS2 nanosheets as an effective fluorescent quencher for successive detection of arsenic ions in aqueous system	2018

8621	N. Ghorbanzadeh	Influence of clay minerals on sorption and bioreduction of arsenic under anoxic conditions	2015
8622	S. Muller	Sustaining efficient production of aqueous iron during repeated operation of Fe(0)-electrocoagulation	2019
8623	H. G. M. Edwards	English delftware (c. 1770) from Bristol, Lancaster and Liverpool: A composition study using Raman spectroscopy and electron microscopy	2022
8624	M. Elmes	Single-particle analysis of atmospheric particulate matter using automated mineralogy: the potential for monitoring mine-derived emissions	2020
8625	C. M. van Genuchten	Groundwater As Removal by As(III), Fe(II), and Mn(II) Co-Oxidation: Contrasting As Removal Pathways with O-2, NaOCl, and KMnO4	2020
8626	G. T. LaHue	Alternate wetting and drying in high yielding direct-seeded rice systems accomplishes multiple environmental and agronomic objectives	2016
8627	B. Y. Song	Reactivity of binary manganese oxide mixtures towards arsenite removal: Evidence of synergistic effects	2021
8628	A. Iriel	Arsenic Adsorption on Iron-Modified Montmorillonite: Kinetic Equilibrium and Surface Complexes	2020
8629	A. E. Tufo	Synthesis and characterization of pure and Al-substituted akaganeites and evaluation of their performance to adsorb As(V)	2018
8630	A. Bettoschi	Arsenic speciation in marine sediments: A comparison between two sequential extraction procedures	2018
8631	R. Freitas	The influence of Arsenic on the toxicity of carbon nanoparticles in bivalves	2018
8632	I. P. Pozdnyakov	Photochemical transformation of an iron(III)-arsenite complex in acidic aqueous solution	2016
8633	M. Kuramata	Arsenic biotransformation by Streptomycessp isolated from rice rhizosphere	2015
8634	A. S. K. Kumar	Synthesis of magnetically separable and recyclable magnetic nanoparticles decorated with beta-cyclodextrin functionalized graphene oxide an excellent adsorption of As(V)/(III)	2017
8635	A. Sundman	Complexation and precipitation reactions in the ternary As(V)-Fe(III)-OM (organic matter) system	2014
8636	H. P. Feng	Cu-Doped Fe@Fe2O3 core-shell nanoparticle shifted oxygen reduction pathway for high-efficiency arsenic removal in smelting wastewater	2018
8637	M. S. Podder	Bacteria immobilization on neem leaves/MnFe2O4 composite surface for removal of As(III) and As(V) from wastewater	2019
8638	K. Wu	The enhanced removal of arsenite from water by double-shell CuOx@ MnOy hollow spheres (DCMHS): behavior and mechanisms	

8639	N. Tara	Reduce graphene oxide-manganese oxide-black cumin based hybrid composite (rGO-MnO2/BC): A novel material for water remediation	2020
8640	M. E. Huq	Distribution and hydrogeochemical behavior of arsenic enriched groundwater in the sedimentary aquifer comparison between Datong Basin (China) and Kushtia District (Bangladesh)	2018
8641	Z. H. Xu	Nanoscale iron oxides loaded granular activated carbon (GAC-NSIO) for cadmium removal	2016
8642	S. H. Zhang	Study on the precipitation of iron and the synchronous removal mechanisms of antimony and arsenic in the AMD under the induction of carbonate rocks	
8643	A. Penezic	Spatial variability of arsenic speciation in the Gironde Estuary: Emphasis on dynamic (potentially bioavailable) inorganic arsenite and arsenate fractions	2020
8644	Y. J. Liu	Magnetite recovery from copper tailings increases arsenic distribution in solution phase and uptake in native grass	2017
8645	R. Kaur	A Schiff base modified graphene oxide film for anodic stripping voltammetric determination of arsenite	2019
8646	E. Kociolek-Balawejde	Hybrid ion exchangers containing Fe(III)-Cu(II) binary oxides obtained using macroreticular anion exchanger	2018
8647	L. J. Ding	Influences of arsenate and/or phosphate adsorption to ferrihydrite on iron-reducing and arsenic-reducing microbial communities in paddy soil revealed by rRNA-C-13-acetate probing	2022
8648	L. J. Zhong	Solubility of nano-sized metal oxides evaluated by using in vitro simulated lung and gastrointestinal fluids: implication for health risks	2017
8649	R. J. Buszek	On the Comparison of Small Nitrogen and Phosphorus Oxide Cages	2015
8650	J. C. Hower	Chemistry and petrology of paired feed coal and combustion ash from anthracite-burning stoker boilers	2017
8651	P. B. Hu	As2O3 adsorption enhancement over Ni-modified gamma-Al2O3 in complex flue gas constituents	2022
8652	R. Kabir	Inorganic arsenic induces sex-dependent pathological hypertrophy in the heart	2021
8653	Y. A. Shang	Cellulose based multifunctional hybrid material for sequestering phosphate in stratified water purification columns	2018
8654	Q. B. Zhang	CuO nanostructures: Synthesis, characterization, growth mechanisms, fundamental properties, and applications	2014
8655	D. Yao	Promotion mechanism of natural clay colloids in the adsorption of arsenite on iron oxide particles in water	2020
8656	J. B. Lu	Nano iron oxide impregnated in chitosan bead as a highly efficient sorbent for Cr(VI) removal from water	2017

8657	R. Thacher	Modeling and Performance Prediction of Chromate Reduction by Iron Oxide Coated Sand in Adsorber Reactors	2016
8658	A. M. Nasir	Adsorptive nanocomposite membranes for heavy metal remediation: Recent progresses and challenges	2019
8659	Y. N. Chen	Synthesis, application and mechanisms of Ferro-Manganese binary oxide in water remediation: A review	2020
8660	S. Lath	Mixed-Mode Remediation of Cadmium and Arsenate Ions Using Graphene-Based Materials	2018
8661	K. D. Hristovski	Engineering metal (hydr)oxide sorbents for removal of arsenate and similar weak-acid oxyanion contaminants: A critical review with emphasis on factors governing sorption processes	2017
8662	H. Singh	Chemical fractionation of heavy metals and nutrients in sludge and waste water generated by Coca-Cola soft drink industry	2015
8663	S. B. Liu	Effects of single- and multi-organic acid ligands on adsorption of copper by Fe3O4/graphene oxide-supported DCTA	2016
8664	A. Praveen	Protective role of nitric oxide on nitrogen-thiol metabolism and amino acids profiling during arsenic exposure inOryza sativaL	2020
8665	K. A. Taleb	Efficient pollutants removal by amino-modified nanocellulose impregnated with iron oxide	2016
8666	A. Chiavola	Arsenic Removal from a Highly Contaminated Groundwater by a Combined Coagulation-Filtration-Adsorption Process	2019
8667	M. Rotiroti	Considering a Threshold Energy in Reactive Transport Modeling of Microbially Mediated Redox Reactions in an Arsenic-Affected Aquifer	2018
8668	S. Pourbeyram	Time Resolved Direct Determination of Arsenate in the Presence of Arsenite on Pencil Graphite Electrode Modified by Graphene Oxide and Zirconium	2018
8669	Y. Wang	Enhanced adsorption of hexavalent chromium from aqueous solutions on facilely synthesized mesoporous iron-zirconium bimetal oxide	2015
8670	T. Inboonchuay	Amounts and associations of heavy metals in paddy soils of the Khorat Basin, Thailand	2016
8671	Y. B. Xie	Superior activity of Ce-HZSM-5 catalyst for catalytic oxidation of arsine at low oxygen	2019
8672	M. Govindasamy	Investigation of sonochemically synthesized sphere-like metal tungstate nanocrystals decorated activated carbon sheets network and its application towards highly sensitive detection of arsenic drug in biological samples	2020
8673	K. Wu	Competitive adsorption behaviors of arsenite and fluoride onto manganese-aluminum binary adsorbents	2017
8674	C. Y. Han	Adsorption performance and mechanism of As(V) uptake over mesoporous Y-Al binary oxide	2016

8675	B. Kalyani	Electron paramagnetic resonance and optical properties of Cu+2 in PbO-Li2O-As2O3-B2O3 glasses	2020
8676	L. Arfaoui	Characterization and adsorption performance of Pb(II) on CuO nanorods synthesized by the hydrothermal method	2015
8677	E. K. Skierszkan	Release of geogenic uranium and arsenic results in water-quality impacts in a subarctic permafrost region of granitic and metamorphic geology	2020
8678	W. Gustave	Soil organic matter amount determines the behavior of iron and arsenic in paddy soil with microbial fuel cells	2019
8679	M. Pedrot	Unravelling the fate of arsenic during re-oxidation of reduced wetland waters: Experimental constraints and environmental consequences	2015
8680	A. G. Pietralonga	Lanthanum immobilization by iron and aluminum colloids	2017
8681	J. Xie	Distribution and chemical speciation of arsenic in different sized atmospheric particulate matters	2021
8682	F. Franco	Low-cost aluminum and iron oxides supported on dioctahedral and trioctahedral smectites: A comparative study of the effectiveness on the heavy metal adsorption from water	2016
8683	L. Chen	Performance and mechanism of hierarchically porous Ce-Zr oxide nanospheres encapsulated calcium alginate beads for fluoride removal from water	2016
8684	X. F. Chen	Small structures with big impact: Multi-walled carbon nanotubes enhanced remediation efficiency in hyperaccumulator Solanum nigrum L. under cadmium and arsenic stress	2021
8685	Z. D. Yuan	Effect of pH on oxygen-induced abio-oxidation and removal of As from As-rich acid mine drainage in the co-existence of Zn(II)	2022
8686	R. L. Yu	Effect of mixed moderately thermophilic adaptation on leachability and mechanism of high arsenic gold concentrate in an airlift bioreactor	2015
8687	J. M. Luo	Zirconia (ZrO2) Embedded in Carbon Nanowires via Electrospinning for Efficient Arsenic Removal from Water Combined with DFT Studies	2016
8688	A. A. Artemyeva	Molecular scale studies that inform trace element sulfide evaporation and atomization behavior during coal combustion	2017
8689	I. Lee	Functionalization of activated carbon fiber through iron oxide impregnation for As(V) removal: equilibrium, kinetic, and thermodynamic analyses	2016
8690	M. H. Siddiqui	Molybdenum and hydrogen sulfide synergistically mitigate arsenic toxicity by modulating defense system, nitrogen and cysteine assimilation in faba bean (Vicia faba L.) seedlings	2021
8691	B. Murtaza	Hydrogeochemical investigation of arsenic in drinking water of schools and age dependent risk assessment in Vehari District, Punjab Pakistan: a multivariate analysis	2020
8692	N. T. H. Ha	Uptake of arsenic and heavy metals by native plants growing near Nui Phao multi-metal mine, northern Vietnam	2019

8693	J. M. Luo	Arsenic adsorption on alpha-MnO2 nanofibers and the significance of (100) facet as compared with (110)	2018
8694	Q. Sun	The oxidation and sorption mechanism of Sb on delta-MnO2	2018
8695	B. J. Little	The Relationship Between Iron Oxides/Oxyhydroxides and Toxic Metal Ions in Drinking Water Distribution Systems A Review	2017
8696	N. Sohrabi	A probabilistic-deterministic analysis of human health risk related to the exposure to potentially toxic elements in groundwater of Urmia coastal aquifer (NW of Iran) with a special focus on arsenic speciation and temporal variation	2021
8697	G. X. Huang	A multidisciplinary investigation on bio-oxidation gold mine tailings from Dandong, China as a potential arsenic pollution source	2019
8698	E. Smith	Influence of in vitro assay pH and extractant composition on As bioaccessibility in contaminated soils	2014
8699	M. Y. Liang	Arsenite oxidation and arsenic adsorption on birnessite in the absence and the presence of citrate or EDTA	2020
8700	J. H. Li	Vanadium Dioxide Nanocoating Induces Tumor Cell Death through Mitochondrial Electron Transport Chain Interruption	2019
8701	X. L. Wu	Effective removal of arsenide from aqueous solutions using mesoporous CoFe2O4/graphene oxide nanocomposites assisted by artificial intelligence	2021
8702	I. Suzuki	First Principles Calculation of Electrical and Optical Properties of Cu3AsO4: Promising Thin-Film Solar Cell Absorber from Nonferrous Metal Manufacturing By-Products	2022
8703	C. B. Tabelin	Solid-phase partitioning and release-retention mechanisms of copper, lead, zinc and arsenic in soils impacted by artisanal and small-scale gold mining (ASGM) activities	2020
8704	C. M. van Genuchten	Formation of macroscopic surface layers on Fe(0) electrocoagulation electrodes during an extended field trial of arsenic treatment	2016
8705	Y. Georgiou	Surface decoration of amine-rich carbon nitride with iron nanoparticles for arsenite (As-III) uptake: The evolution of the Fe-phases under ambient conditions	2016
8706	I. Jacukowicz-Sobala	Iron and aluminium oxides containing industrial wastes as adsorbents of heavy metals: Application possibilities and limitations	2015
8707	A. C. Meneguelli-Souza	Arsenic in the lower drainage basin of the Paraiba do Sul River (Southeast Brazil): dynamics between the water column and sediment	2021
8708	G. Z. Nie	Efficient removal of phosphate by a millimeter-sized nanocomposite of titanium oxides encapsulated in positively charged polymer	2019
8709	X. Q. Xie	Microstructure and surface control of MXene films for water purification	2019
8710	E. C. Lewis	First-Principles Density Functional Theory Characterisation of the Adsorption Complexes of H3AsO3 on Cobalt Ferrite (Fe2CoO4) Surfaces	2021

8711	M. Reyes-Becerril	Methylmercury, cadmium and arsenic(III)-induced toxicity, oxidative stress and apoptosis in Pacific red snapper leukocytes	2019
8712	S. Hafeznezami	Arsenic mobilization in an oxidizing alkaline groundwater: Experimental studies, comparison and optimization of geochemical modeling parameters	2016
8713	C. Kohfahl	Algae metabolism and organic carbon in sediments determining arsenic mobilisation in ground- and surface water. A field study in Donana National Park, Spain	2016
8714	M. B. Javed	Arsenic fractionation and mineralogical characterization of sediments in the Cold Lake area of Alberta, Canada	2014
8715	B. E. Monarrez-Cordero	Synthesis and characterization of composite Fe-Ti oxides nanoparticles with high surface area obtained via AACVD	2018
8716	M. P. Subbaiah	Synthesis and Modification Strategies of Chitosan and Its Interaction with Metal Ions	2021
8717	M. Kumar	Scenario, perspectives and mechanism of arsenic and fluoride Co-occurrence in the groundwater: A review	2020
8718	I. Herath	Natural Arsenic in Global Groundwaters: Distribution and Geochemical Triggers for Mobilization	2016
8719	M. Adil	Naringin ameliorates sodium arsenite-induced renal and hepatic toxicity in rats: decisive role of KIM-1, Caspase-3, TGF-beta, and TNF-alpha	2015
8720	S. Doherty	Antimony and arsenic particle size distribution in a mining contaminated freshwater river: Implications for sediment quality assessment and quantifying dispersion	2022
8721	H. Esmaeili	Activated carbon@MgO@Fe(3)O(4)as an efficient adsorbent for As (III) removal	2021
8722	H. C. Chang	Modified nanoporous membranes on centrifugal microfluidic platforms for detecting heavy metal ions	2014
8723	S. Hasan	Dispersion of FeOOH on Chitosan Matrix for Simultaneous Removal of As(III) and As(V) from Drinking Water	2014
8724	Z. D. Yuan	Effect of hydroquinone-induced iron reduction on the stability of Fe(III)-As(V) Co-precipitate and arsenic mobilization	2018
8725	T. L. Noble	Mobility of arsenic and environmentally significant elements in mine tailings following liming	2016
8726	E. Alvarez-Ayuso	Stabilization methods for the treatment of weathered arsenopyrite mine wastes: Arsenic immobilization under selective leaching conditions	2021
8727	S. Wagner	Arsenic redox transformations and cycling in the rhizosphere of Pteris vittata and Pteris quadriaurita	2020
8728	P. P. Liu	Synthesis of nano-scale zero-valent iron-reduced graphene oxide-silica nano-composites for the efficient removal of arsenic from aqueous solutions	2019

8729	K. Marschner	Validation and inter-laboratory study of selective hydride generation for fast screening of inorganic arsenic in seafood	2019
8730	E. B. V. Freire	Exploring the effect of substitutional doping on the electronic properties of graphene oxide	2018
8731	D. Cortes-Arriagada	Performance of doped graphene nanoadsorbents with first-row transition metals (Sc-Zn) for the adsorption of water-soluble trivalent arsenicals: A DFT study	2019
8732	J. An	Evaluation of the effectiveness of in situ stabilization in the field aged arsenic-contaminated soil: Chemical extractability and biological response	2019
8733	Q. Sun	Evaluation of the diffusive gradients in thin films technique using a mixed binding gel for measuring iron, phosphorus and arsenic in the environment	2015
8734	E. Alvarez-Ayuso	Arsenic distribution in soils and rye plants of a cropland located in an abandoned mining area	2016
8735	A. M. Cabrales-Gonzalez	Bioleaching of As from mine tailings using an autochthonous Bacillus cereus strain	2022
8736	W. J. Yang	The adsorption characteristics of As2O3, Pb-0, PbO and PbCl2 on single atom iron adsorbent with graphene-based substrates	2019
8737	W. J. Huang	Surface protection method for the magnetic core using covalent organic framework shells and its application in As(III) depth removal from acid wastewater	2022
8738	N. Y. Yin	In Vitro Assessment of Arsenic Release and Transformation from As(V)-Sorbed Goethite and Jarosite: The Influence of Human Gut Microbiota	2020
8739	S. E. Al Garni	Exploring the Optical Dynamics in the ITO/As2Se3 Interfaces	2019
8740	A. Parviainen	Raman identification of Fe precipitates and evaluation of As fate during phase transformation in Tinto and Odiel River Basins	2015
8741	S. Contreras	Arsenic transport and adsorption modeling in columns using a copper nanoparticles composite	2017
8742	S. Ghosh	Microcosm based analysis of arsenic release potential of Bacillus sp. strain IIIJ3-1 under varying redox conditions	2020
8743	K. L. Dubrawski	Production and Transformation of Mixed-Valent Nanoparticles Generated by Fe(0) Electrocoagulation	2015
8744	F. Li	Novel ball-milled biochar-vermiculite nanocomposites effectively adsorb aqueous As(V)	2020
8745	J. R. Tong	Coupled dynamics of As-containing ferrihydrite transformation and As desorption/re-adsorption in presence of sulfide	2020
8746	C. Hemmingsson	Evaluation of phosphate-uptake mechanisms by Fe(III) (oxyhydr)oxides in Early Proterozoic oceanic conditions	2018

8747	C. M. van Genuchten	Bacterial bioreporter detection of arsenic associated with iron oxides	2018
8748	H. L. Cao	The effect of groundwater velocities on sulfidation of arsenic-bearing ferrihydrite: Insight from column experiments	2020
8749	M. A. Limmer	Carryover effects of silicon-rich amendments in rice paddies	2021
8750	M. B. Javed	Thermally Released Arsenic in Porewater from Sediments in the Cold Lake Area of Alberta, Canada	2016
8751	E. A. Soria	Effect of Arsenite on Nitrosative Stress in Human Breast Cancer Cells and Its Modulation by Flavonoids	2015
8752	S. Abbasi	The influence of long-term ageing on arsenic ecotoxicity in soil	2021
8753	D. Renock	Electrochemical Investigation of Arsenic Redox Processes on Pyrite	2017
8754	Y. Zhang	Investigation of Arsenic Poisoned Selective Catalytic Reduction Catalyst Performance and Lifetime in Coal-Fired Power Plants	2020
8755	J. A. Prieto-Amparan	Multivariate and Spatial Analysis of Physicochemical Parameters in an Irrigation District, Chihuahua, Mexico	2018
8756	J. Sun	In Situ Magnetite Formation and Long-Term Arsenic Immobilization under Advective Flow Conditions	2016
8757	B. Y. Li	Mechanistic insights into the enhanced removal of roxsarsone and its metabolites by a sludge-based, biochar supported zerovalent iron nanocomposite: Adsorption and redox transformation	2020
8758	M. M. H. Chowdhury	Recent advances in bionanomaterials for liver cancer diagnosis and treatment	2021
8759	P. A. Gunka	Towards a quantitative bond valence description of coordination spheres - the concepts of valence entropy and valence diversity coordination numbers	2019
8760	V. Rimondi	Bioaccessible arsenic in soil of thermal areas of Viterbo, Central Italy: implications for human health risk	2022
8761	S. A. Lone	Geogenic groundwater arsenic in high altitude bedrock aquifers of upper Indus river basin (UIRB), Ladakh	2020
8762	C. H. Liu	Mechanism of Arsenic Adsorption on Magnetite Nanoparticles from Water: Thermodynamic and Spectroscopic Studies	2015
8763	M. Hamayun	Equilibrium and kinetics studies of arsenate adsorption by FePO4	2014
8764	M. Ahmaruzzaman	Environmentally benign fabrication of SnO2-CNT nanohybrids and their multifunctional efficiency as an adsorbent, catalyst and antimicrobial agent for water decontamination	2019

8765	Z. M. Sha	Minerals loaded with oxygen nanobubbles mitigate arsenic translocation from paddy soils to rice	2020
8766	D. A. Pass	The effect of anthropogenic arsenic contamination on the earthworm microbiome	2015
8767	Y. B. Xie	Magnetic-field-assisted catalytic oxidation of arsine over Fe/HZSM-5 catalyst: Synergistic effect of Fe species and activated surface oxygen	2022
8768	H. Guenet	Highlighting the wide variability in arsenic speciation in wetlands: A new insight into the control of the behavior of arsenic	2017
8769	Q. Yu	Effects of short-term flooding on arsenic transport in groundwater system: A case study of the Datong Basin	2015
8770	S. De Dalui	Binding of As3+ and As5+ to Fe(III) Oxyhydroxide Clusters and the Influence of Aluminum Substitution: A Molecular Perspective	2022
8771	X. Q. Zhong	Mineral medicine: from traditional drugs to multifunctional delivery systems	2022
8772	S. Gibaud	(2-phenyl-1,3,2 dithiarsolan-4-yl)-methanol derivatives show in vitro antileukemic activity	2006
8773	A. Onno	Understanding what limits the voltage of polycrystalline CdSeTe solar cells	2022
8774	D. You	Tuning the effective utilization of adsorption sites in La-MOFs via a steric hindrance effect towards enhanced As(iii) removal	2021
8775	C. F. Kerl	Iron Plaque at Rice Roots: No Barrier for Methylated Thioarsenates	2019
8776	C. H. Chen	One-pot synthesis of ternary zero-valent iron/phosphotungstic acid/g-C3N4 composite and its high performance for removal of arsenic(V) from water	2017
8777	L. Zhang	Recycling the steel pickling waste liquor to produce a low-cost material for immobilization of heavy metal(loid)s	2021
8778	D. H. Phillips	Arsenic and fluoride removal from contaminated drinking water with Haix-Fe-Zr and Haix-Zr resin beads	2018
8779	Y. Wang	Review on Removal of SO2, NOx, Mercury, and Arsenic from Flue Gas Using Green Oxidation Absorption Technology	2021
8780	P. Langner	Oxidation of Organosulfur-Coordinated Arsenic and Realgar in Peat: Implications for the Fate of Arsenic	2014
8781	C. L. Dlamini	Polymeric ion exchanger supported ferric oxide nanoparticles as adsorbents for toxic metal ions from aqueous solutions and acid mine drainage	2019
8782	T. T. Yang	Removal mechanisms of Cd from water and soil using Fe-Mn oxides modified biochar	2022

8783	K. Wu	The enhanced As(III) removal by Fe-Mn-Cu ternary oxide via synergistic oxidation: Performances and mechanisms	2021
8784	J. M. Barazesh	Trace Element Removal in Distributed Drinking Water Treatment Systems by Cathodic H2O2 Production and UV Photolysis	2018
8785	H. Atallah	A highly stable indium based metal organic framework for efficient arsenic removal from water	2018
8786	M. Alimohammady	Highly efficient simultaneous adsorption of Cd(II), Hg(II) and As(III) ions from aqueous solutions by modification of graphene oxide with 3-aminopyrazole: central composite design optimization	2017
8787	L. Sailo	Natural attenuation processes of arsenic in the groundwater of the Brahmaputra floodplain of Assam, India	2016
8788	T. V. Rakotonimaro	Compaction of peat cover over desulfurized gold mine tailings changes: Arsenic speciation and mobility	2021
8789	S. Sacmaci	Grafting of glutathione to magnetic graphene oxide and application for the determination of As(III)/(V) in food samples via a zeta potential analyzer	2018
8790	A. Ramos-Ruiz	Gallium arsenide (GaAs) leaching behavior and surface chemistry changes in response to pH and O-2	2018
8791	J. Z. J. Iv	Identifying the source of groundwater contaminants in West-Central Wisconsin, USA: Geochemical and mineralogical characterization of the Cambrian sandstone aquifer	2022
8792	M. Schindler	Deciphering the Distribution and Crystal-Chemical Environment of Arsenic, Lead, Silica, Phosphorus, Tin, and Zinc in a Porous Ferrihydrite Grain Using Transmission Electron Microscopy and Atom Probe Tomography	2022
8793	J. Z. Cui	New application of lanthanum-modified bentonite (Phoslock (R)) for immobilization of arsenic in sediments	2021
8794	J. A. Bhat	Newly-synthesized iron-oxide nanoparticles showed synergetic effect with citric acid for alleviating arsenic phytotoxicity in soybean	2022
8795	I. Iqbal	THE COMBINED EFFECT OF ZnO NANO PARTICLES AND TOXICITY OF HEAVY METALS (ARSENIC AND CHROMIUM) ON THE MORPHOLOGICAL, BIOCHEMICAL ATTRIBUTES OF WHEAT (TRITICUM AESTIVUM L.) AS WELL AS SOIL AND	
8796	W. J. Yang	High-efficiency and fast removal of As(III) from water by cerium oxide needles decorated macroporous carbon sponge	2022
8797	A. Baranik	Graphene Oxide Decorated with Cerium(IV) Oxide in Determination of Ultratrace Metal Ions and Speciation of Selenium	2018
8798	M. Yuan	Simultaneous adsorption and oxidation of Sb(III) from water by the pH-sensitive superabsorbent polymer hydrogel incorporated with Fe-Mn binary oxides composite	2022
8799	S. Dudek	Arsenate removal on the iron oxide ion exchanger modified with Neodymium(III) ions	2022
8800	S. B. Chen	HOXA9 is critical in the proliferation, differentiation, and malignancy of leukaemia cells both in vitro and in vivo	2017

8801	X. Y. Lou	Enhanced arsenite removal by superparamagnetic iron oxide nanoparticles in-situ synthesized on a commercial cube-shape sponge: adsorption-oxidation mechanism	2022
8802	J. C. J. Gude	Effect of supernatant water level on As removal in biological rapid sand filters	2018
8803	W. Sun	2-Methoxy-6-acetyl-7-methyljuglone (MAM), a natural naphthoquinone, induces NO-dependent apoptosis and necroptosis by H2O2-dependent JNK activation in cancer cells	2016
8804	A. A. Alswata	Preparation of Zeolite/Zinc Oxide Nanocomposites for toxic metals removal from water	2017
8805	S. K. Maji	Dynamic column adsorption of As on iron-oxide-coated natural rock (IOCNR) and sludge management	2015
8806	P. Zhang	Novel "loose" GO/MoS2 composites membranes with enhanced permeability for effective salts and dyes rejection at low pressure	2019
8807	R. J. Yang	Biosurfactant rhamnolipid affacts the desorption of sorbed As(III), As(V), Cr (VI), Cd(II) and Pb(II) on iron (oxyhydr)oxides and clay minerals	2020
8808	K. Kameda	Stabilization of arsenic and lead by magnesium oxide (MgO) in different seawater concentrations	2018
8809	R. Nagarajah	Nano-Structured Magnesium Oxide Coated Iron Ore: Its Application to the Remediation of Wastewater Containing Lead	2015
8810	U. Baig	Facile Synthesis of Polypyrrole-Zirconium(IV) Oxide-Ethanolamine Anion Exchange Nanocomposite and Its Utilization in Membrane Electrode Development for Sensing and Quantitative Detection of As(III) in Water	2016
8811	L. Y. Lin	Simultaneous immobilization of arsenic and cadmium in paddy soil by Fe-Mn binary oxide: A field-scale study	2020
8812	N. M. Myers	Green design of a paper test card for urinary iodine analysis	2017
8813	E. Kociolek-Balawejde	Synthesis and characterization of CuO-loaded macroreticular anion exchange hybrid polymer	2016
8814	J. J. Liang	Ultra-fast enrichment and reduction of As(V)/Se(VI) on three dimensional graphene oxide sheets-oxidized carbon nanotubes hydrogels	2019
8815	B. Zhu	PML-RARa stabilized by zinc in human acute promyelocytic leukemia NB4 cells	2017
8816	B. Hudcova	Stability and stabilizing efficiency of Mg-Fe layered double hydroxides and mixed oxides in aqueous solutions and soils with elevated As(V), Pb (II) and Zn(II) contents	2019
8817	C. Kim	Surface functionalized nanoscale metal oxides for arsenic(v), chromium(vi), and uranium(vi) sorption: considering single- and multi-sorbate dynamics	2020
8818	L. Mu	L-Cysteine: A biocompatible, breathable and beneficial coating for graphene oxide	2015

8819	G. Zelmanov	Boron removal from water and its recovery using iron (Fe+3) oxide/hydroxide-based nanoparticles (NanoFe) and NanoFe-impregnated granular activated carbon as adsorbent	2014
8820	D. K. Singh	Preparation of CuO nanoparticles using Tamarindus indica pulp extract for removal of As(III): Optimization of adsorption process by ANN-GA	2017
8821	H. M. Shan	Highly efficient removal of As(III) from aqueous solutions using goethite/graphene oxide/chitosan nanocomposite	2020
8822	D. Gounden	Electroanalytical detection of heavy metals using metallophthalocyanine and silica-coated iron oxide composites	2018
8823	L. C. Terron-Camero	Role of nitric oxide in plant responses to heavy metal stress: exogenous application versus endogenous production	2019
8824	M. S. Islam	Needle-like iron oxide@CacO(3) adsorbents for ultrafast removal of anionic and cationic heavy metal ions	2017
8825	C. Y. Li	Adsorption of Cd2+ and Ni2+ from Aqueous Single-Metal Solutions on Graphene Oxide-Chitosan-Poly(vinyl alcohol) Hydrogels	2019
8826	H. Neidhardt	Organic carbon induced mobilization of iron and manganese in a West Bengal aquifer and the muted response of groundwater arsenic concentrations	2014
8827	A. A. Ensafi	A novel aptasensor based on 3D-reduced graphene oxide modified gold nanoparticles for determination of arsenite	2018
8828	X. L. You	Phosphate recovery from treated municipal wastewater using hybrid anion exchangers containing hydrated ferric oxide nanoparticles	2018
8829	W. W. Li	Facile one-pot and rapid synthesis of surfactant-free Au-reduced graphene oxide nanocomposite for trace arsenic (III) detection	2015
8830	X. J. Su	Direct Microwave Roasting of Arsenic-Bearing Pyrite Concentrates	2014
8831	J. Phanthasri	Enhancement of arsenite removal using manganese oxide coupled with iron (III) trimesic	2018
8832	V. Gupta	Spatial distribution, pollution levels, and risk assessment of potentially toxic metals in road dust from major tourist city, Dehradun, Uttarakhand India	
8833	J. G. T. Armstrong	Mobilisation of arsenic, selenium and uranium from Carboniferous black shales in west Ireland	2019
8834	K. S. Nitzsche	Arsenic removal from drinking water by a household sand filter in Vietnam - Effect of filter usage practices on arsenic removal efficiency and microbiological water quality	2015
8835	J. Xu	Mechanism of miR-21 Antagonist Packaged with Arsenic Trioxide (As2O3) Nanoparticles in Restraining Invasion and Metastasis of Intestinal Cancer by Targeted C-C Motif Chemokine Receptor 7 (CCR7) Protein Through Induction of PI3K/Akt Signal Pathway	2021
8836	N. Alamos	High-resolution inventory of atmospheric emissions from transport, industrial, energy, mining and residential activities in Chile	2022

8837	T. Sha	Influence of the structure and composition of Fe-Mn binary oxides on rGO on As(III) removal from aquifers	2020
8838	S. Kutschke	Bioleaching of Kupferschiefer blackshale - A review including perspectives of the Ecometals project	2015
8839	A. Pasquier	How to Control HTLV-1-Associated Diseases: Preventing de Novo Cellular Infection Using Antiviral Therapy	2018
8840	M. Louzon	Telomere dynamic in humans and animals: Review and perspectives in environmental toxicology	2019
8841	M. A. Vega	Biogeochemical Controls on the Release and Accumulation of Mn and As in Shallow Aquifers, West Bengal, India	2017
8842	Z. W. Zhang	EFFECT OF 25-HYDROXYVITAMIN D3 AND DIVALENT CALCIUM ION SUPPLEMENTATION IN SOWS' DIETS ON TRACE ELEMENTS IN BLOOD AND MILK	2022
8843	P. Bataillard	Iron and arsenic speciation in marine sediments undergoing a resuspension event: the impact of biotic activity	2014
8844	A. Singh	Investigation on depth resolved compositions of e-beam deposited ZrO2 thin film	2017
8845	C. L. Vithana	Liberation of acidity and arsenic from schwertmannite: Effect of fulvic acid	2014
8846	A. Saedi	Utilization of lead-zinc mine tailings as cement substitutes in concrete construction: Effect of sulfide content	2022
8847	Y. Tanimoto	Synthesis of 2 ferrocenophanes containing trivalent diphosphine units	2012
8848	Y. H. Shih	Gravidity, parity, blood pressure and mortality among women in Bangladesh from the HEALS cohort	2020
8849	M. Driess	Synthesis and unusual reactivity of compounds containing silicon phosphorus and silicon arsenic double bonds: New silylidenephosphanes and -arsanes of the type R(2)Si=E(SiR(3)) (E=P, As)	1996
8850	M. H. Jin	Effects of Excessive Iodine on the BDNF-TrkB Signaling Pathway and Related Genes in Offspring of EAT Rats	
8851	O. Laurent	Sources and contents of air pollution affecting term low birth weight in Los Angeles County, California, 2001-2008	2014
8852	F. M. Nilsen	A Meta-Analysis of Stressors from the Total Environment Associated with Children's General Cognitive Ability	2020
8853	K. Gustin	Methylmercury exposure and cognitive abilities and behavior at 10 years of age	2017
8854	J. J. Lee	Growth parameters at birth mediate the relationship between prenatal manganese exposure and cognitive test scores among a cohort of 2-to 3-year-old Bangladeshi children	2018

8855	S. N. Lundgren	Microbial Communities in Human Milk Relate to Measures of Maternal Weight	2019
8856	S. S. Su	Efficient transformation and elimination of roxarsone and its metabolites by a new alpha-FeOOH@GCA activating persulfate system under UV irradiation with subsequent As(V) recovery	2019
8857	H. Y. Chen	Highly efficient As(III) removal through simultaneous oxidation and adsorption by N-CQDs modified MIL-53(Fe)	2022
8858	K. H. Gao	Intensified redox co-conversion of As(III) and Cr(VI) with MIL-125(Ti)-derived COOH functionalized TiO2: Performance and mechanism	2019
8859	D. Mohanta	Bio-inspired adsorption of arsenite and fluoride from aqueous solutions using activated carbon@SnO2 nanocomposites: Isotherms, kinetics, thermodynamics, cost estimation and regeneration studies	2018
8860	D. Baragano	Nanoremediation of As and metals polluted soils by means of graphene oxide nanoparticles	2020
8861	F. Valentini	Raman, X-Ray Fluorescence Spectroscopies and Graphene Oxide Modified Screen Printed Electrodes to Identify the Pigments and Earth Present in Ancient Leather Samples	2017
8862	S. T. Klosterman	Adsorption Model for Arsenate Transport in Corroded Iron Pipes with Application to a Simulated Intrusion in a Water Distribution Network	2014
8863	M. Perri	BCL-x(L)/MCL-1 inhibition and RAR gamma antagonism work cooperatively in human HL60 leukemia cells	2014
8864	A. Shahzad	Heavy metals removal by EDTA-functionalized chitosan graphene oxide nanocomposites	2017
8865	Y. S. Jun	Heterogeneous Nucleation and Growth of Nanoparticles at Environmental Interfaces	2016
8866	Z. D. Yu	Rapid degradation of p-arsanilic acid and simultaneous removal of the released arsenic species by Co-Fe@C activated peroxydisulfate process	2022
8867	Y. J. Lin	Developing sustainable graphene-doped titanium nano tube coated to superparamagnetic nanoparticles for arsenic recovery	2017
8868	A. Gianfagna	As-RICH APATITE FROM MT. CALVARIO: CHARACTERIZATION BY MICRO-RAMAN SPECTROSCOPY	2014
8869	S. R. Choi	Hydrogen Evolution Inorganic Inhibitors in Alkaline Electrolyte for Aluminum-Air Battery	2020
8870	D. Budimirovic	Efficient As(V) removal by alpha-FeOOH and alpha-FeOOH/alpha-MnO2 embedded PEG-6-arm functionalized multiwall carbon nanotubes	2017
8871	T. Pardo	Maghemite nanoparticles and ferrous sulfate for the stimulation of iron plaque formation and arsenic immobilization in Phragmites australis	2016
8872	J. Sun	Enhanced and stabilized arsenic retention in microcosms through the microbial oxidation of ferrous iron by nitrate	2016

8873	A. Chroneos	Connecting bulk properties of germanium with the behavior of self- and dopant diffusion	2015
8874	H. P. Zeng	Optimization and regeneration of chitosan-alginate hybrid adsorbent embedding iron-manganese sludge for arsenic removal	2020
8875	X. Zhang	Effects of oxygen-inserted layers and oxide capping layer on dopant activation for the formation of ultrashallow p-n junctions in silicon	2018
8876	G. Priyadarshana	Synthesis of Magnetite Nanoparticles by Top-Down Approach from a High Purity Ore	2015
8877	S. Dubey	Toxicity and detoxification of heavy metals during plant growth and metabolism	2018
8878	M. K. Shahid	Evaluation of arsenate adsorption efficiency of mill-scale derived magnetite particles with column and plug flow reactors	2019
8879	Q. Qiao	Electrochemical adsorption of cadmium and arsenic by natural Fe-Mn nodules	2020
8880	P. Tan	Efficient removal of arsenate from water by lanthanum immobilized electrospun chitosan nanofiber	2020
8881	S. F. Farzan	Prenatal and postnatal mercury exposure and blood pressure in childhood	2021
8882	G. Choppala	Tooeleite Transformation and Coupled As(III) Mobilization Are Induced by Fe(II) under Anoxic, Circumneutral Conditions	
8883	Z. Hassan	Ample Arsenite Bio-Oxidation Activity in Bangladesh Drinking Water Wells: A Bonanza for Bioremediation?	2019
8884	A. Padilla-Rodriguez	Adsorption of Arsenic(V) Oxyanion from Aqueous Solutions by Using Protonated Chitosan Flakes	2015
8885	M. Aschner	Ferroptosis as a mechanism of non-ferrous metal toxicity	2022
8886	E. Przezdziecka	Influence of As doping on the properties of nonpolar ZnO	2021
8887	E. M. Muehe	Are rice (Oryza sativa L.) phosphate transporters regulated similarly by phosphate and arsenate? A comprehensive study	2014
8888	H. Kim	Remediation of Toxic Heavy Metal Contaminated Soil by Combining a Washing Ejector Based on Hydrodynamic Cavitation and Soil Washing Process	2022
8889	X. Yang	Pig carcass-derived biochar caused contradictory effects on arsenic mobilization in a contaminated paddy soil under fluctuating controlled redox conditions	2022
8890	M. M. Rahman	Arsenic sensor development based on modification with (E)-N-(2-nitrobenzylidine)-benzenesulfonohydrazide: a real sample analysis	2019

8891	S. Lan	The Effect of Proton and Arsenic Concentration on As(III) Removal by Hematite and Kaolin Complexes	2021
8892	A. F. Abu Bakar	Heavy Metals Removal from Automotive Wastewater using Chemically Modified Sand	2016
8893	R. Mudhulkar	Preparation and characterization of carrageenan-embedded lanthanum iron oxide nanocomposite for efficient removal of arsenite ions from water	2022
8894	T. Ahmed	Green molybdenum nanoparticles-mediated bio-stimulation of Bacillus sp. strain ZH16 improved the wheat growth by managing in planta nutrients supply, ionic homeostasis and arsenic accumulation	2022
8895	J. Jansson	Freeze lining formation in continuous converting calcium ferrite slags. I	2014
8896	Y. R. Deng	Tetracycline-Induced Release and Oxidation of As(III) Coupled with Concomitant Ferrihydrite Transformation	
8897	M. Hasanzadeh	Synthesis and application of CeO2/sawdust nanocomposite for removal of As(III) ions from aqueous solutions using a fixed bed column system	2017
8898	F. Liu	Removal of Cr(VI) using polyacrylonitrile/ferrous chloride composite nanofibers	2017
8899	E. Mehran	Structural and magnetic properties of turmeric functionalized CoFe2O4 nanocomposite powder	2016
8900	X. Mao	The distribution and elevated solubility of lead, arsenic and cesium in contaminated paddy soil enhanced with the electrokinetic field	2016
8901	F. R. Wu	Photoinduced effects of monochromatic visible light with different wavelengths on realgar	
8902	Y. Wang	Zinc offers splenic protection through suppressing PERK/IRE1-driven apoptosis pathway in common carp (Cyprinus carpio) under arsenic stress	2021
8903	M. A. Halim	Geochemical Evaluation of Arsenic and Manganese in Shallow Groundwater and Core Sediment in Singair Upazila, Central Bangladesh	2014
8904	V. N. Priya	Alginate coated layered double hydroxide/reduced graphene oxide nanocomposites for removal of toxic As (V) from wastewater	2021
8905	M. M. Alam	Electrochemical oxidation of As(III) on Pd immobilized Pt surface: kinetics and sensing performance	2018
8906	A. Kontny	Iron mineral transformations and their impact on As (im)mobilization at redox interfaces in As-contaminated aquifers	2021
8907	T. H. Koo	Formation of pharmacosiderite (KFe4(AsO4)3(OH)(4)center dot 6-7H(2)O) in the acid-sulfate-chloride (ASC) geothermal spring, Norris Geyser Basin, Yellowstone National Park, USA: Implication of Fe and As redox reaction associated with microbe and clay minerals	2022
8908	A. K. Deb	Magnetite Nanoparticles Loaded into Halloysite Nanotubes for Arsenic(V) Removal from Water	

8909	E. Leiva	Arsenic removal mediated by acidic pH neutralization and iron precipitation in microbial fuel cells	2018
8910	W. T. Zhao	Fraction distribution of heavy metals and its relationship with iron in polluted farmland soils around distinct mining areas	2021
8911	S. Tardif	Factors governing the solid phase distribution of Cr, Cu and As in contaminated soil after 40 years of ageing	2019
8912	S. A. Chaudhry	Isotherm, kinetic and thermodynamics of arsenic adsorption onto Iron-Zirconium Binary Oxide-Coated Sand (IZBOCS): Modelling and process optimization	2017
8913	M. Mortazavi	Microstructural and Analytical Study of Uncommon Corrosion Features on Archaeological Cu-As Alloys from Tappeh Graziani in Southeastern Iran	2022
8914	Y. Georgiou	A Hybrid {Silk@Zirconium MOF} Material as Highly Efficient As-III-sponge	2020
8915	R. Prabhakar	Low cost and easy synthesis of aluminium oxide nanoparticles for arsenite removal from groundwater: A complete batch study	2018
8916	Y. Z. Shi	Hierarchical 13X zeolite/reduced graphene oxide porous material for trace Pb (II) capturing from drinking water	2022
8917	B. Song	Effects of structural cobalt on the stability and reactivity of hausmannite and manganite: Cobalt coordination chemistry and arsenite oxidation	2021
8918	Z. D. Yuan	The Effects of Solution Concentration, Drying, Aging Time, and Mixture of Goethite with As(V)-Fe(OH)(3) on the Chemical Extraction of Arsenate Associated with Iron (Hydr)oxides	2017
8919	M. A. Inam	Removal of Arsenic Oxyanions from Water by Ferric Chloride-Optimization of Process Conditions and Implications for Improving Coagulation Performance	2021
8920	M. A. Inam	Interaction of Arsenic Species with Organic Ligands: Competitive Removal from Water by Coagulation-Flocculation-Sedimentation (C/F/S)	2019
8921	Y. X. Wang	Coupled iron, sulfur and carbon isotope evidences for arsenic enrichment in groundwater	2014
8922	J. T. Hou	Morphology-dependent enhancement of arsenite oxidation to arsenate on birnessite-type manganese oxide	2017
8923	J. T. Hou	Tremendous effect of oxygen vacancy defects on the oxidation of arsenite to arsenate on cryptomelane-type manganese oxide	2016
8924	A. B. Covarrubias	Arsenic mobility in the sediments of a dam that receives runoffs from epithennal mines	2020
8925	Q. Rong	Immobilization of As and Sb by combined applications Fe-Mn oxides with organic amendments and alleviation their uptake by Brassica campestris L	2021
8926	X. M. Wang	Promotion of copper electrolyte self-purification with antimonic oxides	2018

8927	Q. W. Zhou	Supplementation with ferromanganese oxide-impregnated biochar composite reduces cadmium uptake by indica rice (Oryza sativa L.)	2018
8928	Y. Y. Zhang	Unexpected Favorable Role of Ca2+ in Phosphate Removal by Using Nanosized Ferric Oxides Confined in Porous Polystyrene Beads	2019
8929	G. Q. Zhang	Arsenic redistribution and transformation during Fe(II)-catalyzed recrystallization of As-adsorbed ferrihydrite under anaerobic conditions	2019
8930	X. Yang	Synergistic adsorption of Cd(II) and As(V) on birnessite under electrochemical control	2020
8931	V. L. Berkovits	Effects of local field and inherent strain in reflectance anisotropy spectra of A(III)B(V) semiconductors with naturally oxidized surfaces	2015
8932	B. Hoagland	Arsenic sequestration in gold mine wastes under changing pH and experimental rewetting cycles	2021
8933	Y. Jia	Arsenic bioavailability to rice plant in paddy soil: influence of microbial sulfate reduction	2015
8934	C. Y. Liu	The accuracy in the assessment of arsenic toxicity using soil alkaline phosphatase depends on soil water contents	2019
8935	E. Kociolek-Balawejde	Freeze dried and thermally dried anion exchanger doped with iron(III) (hydr)oxide - Thermogravimetric studies	2019
8936	J. H. Qin	Dynamics of Fe, Mn, and Al Liberated from Contaminated Soil by Low-Molecular-Weight Organic Acids and Their Effects on the Release of Soil-Borne Trace Elements	2018
8937	P. R. L. Alves	Ecotoxicological impact of arsenic on earthworms and collembolans as affected by attributes of a highly weathered tropical soil	2018
8938	H. T. Ren	Performance and mechanism involved in the cascade oxidation of Mn(II) and As(III) by Bi2.15WO6 under alkaline conditions	2021
8939	M. Y. Liang	Effects of low molecular weight organic acids with different functional groups on arsenate adsorption on birnessite	2022
8940	C. X. Ma	Graphitic Carbon Nitride (C3N4) Reduces Cadmium and Arsenic Phytotoxicity and Accumulation in Rice (Oryza sativa L.)	2021
8941	Y. P. Ji	Effect of phosphate on amorphous iron mineral generation and arsenic behavior in paddy soils	2019
8942	Y. Q. Sun	Reduction of iron (hydr)oxide-bound arsenate: Evidence from high depth resolution sampling of a reducing aquifer in Yinchuan Plain, China	2021
8943	B. Neppolian	Sonochemically synthesized mono and bimetallic Au-Ag reduced graphene oxide based nanocomposites with enhanced catalytic activity	2014
8944	X. Ma	Evaluating the mobility and labile of As and Sb using diffusive gradients in thin-films (DGT) in the sediments of Nansi Lake, China	2020

8945	T. Chowdhury	Removal of Arsenic(III) from Aqueous Solution Using Metal Organic Framework-Graphene Oxide Nanocomposite	2018
8946	S. Liu	Low levels of graphene and graphene oxide inhibit cellular xenobiotic defense system mediated by efflux transporters	2016
8947	G. R. Goncalves	Synthesis of nanostructured iron oxides dispersed in carbon materials and in situ XRD study of the changes caused by thermal treatment	2015
8948	X. Y. Wang	Application and uncertainty of a geochemical speciation model for predicting oxyanion leaching from coal fly ash under different controlling mechanisms	2022
8949	T. Kokab	Development of tribenzamide functionalized electrochemical sensor for femtomolar level sensing of multiple inorganic water pollutants	2020
8950	S. Naseem	Arsenic and other water-quality issues affecting groundwater, Indus alluvial plain, Pakistan	2018
8951	J. C. Ayers	Sources of salinity and arsenic in groundwater in southwest Bangladesh	2016
8952	J. Alchouron	Assessing South American Guadua chacoensis bamboo biochar and Fe3O4 nanoparticle dispersed analogues for aqueous arsenic(V) remediation	2020
8953	R. S. Bangari	Magnetite-Coated Boron Nitride Nanosheets for the Removal of Arsenic(V) from Water	2019
8954	A. I. A. Sherlala	A review of the applications of organo-functionalized magnetic graphene oxide nanocomposites for heavy metal adsorption	2018
8955	C. W. Liu	Reactive transport of arsenic-enriched geothermal spring water into a sedimentary aquifer	2019
8956	R. X. Yuan	Soluble components of sediments and their relation with dissolved arsenic in aquifers from the Hetao Basin, Inner Mongolia	2017
8957	A. Das	Influence of basin-wide geomorphology on arsenic distribution in Nadia district	2021
8958	S. Song	Lactobacillus plantarum L67 glycoprotein protects against cadmium chloride toxicity in RAW 264.7 cells	2016
8959	Z. Chen	Titanium Dioxide Nanoparticles Induced an Enhanced and Intimately Coupled Photoelectrochemical-Microbial Reductive Dissolution of As(V) and Fe(III) in Flooded Arsenic-Enriched Soils	2019
8960	K. F. Pi	Role of sulfur redox cycling on arsenic mobilization in aquifers of Datong Basin, northern China	2017
8961	C. Wu	Arsenic sorption by red mud-modified biochar produced from rice straw	2017
8962	S. Alamri	Molybdenum-induced endogenous nitric oxide (NO) signaling coordinately enhances resilience through chlorophyll metabolism, osmolyte accumulation and antioxidant system in arsenate stressed-wheat (Triticum aestivum L.) seedlings	2022

8963	J. W. Tang	Characterization of arsenic serious-contaminated soils from Shimen realgar mine area, the Asian largest realgar deposit in China	2016
8964	R. Ganguly	The Role of NO in the Amelioration of Heavy Metal Stress in Plants by Individual Application or in Combination with Phytohormones, Especially Auxin	2022
8965	L. A. Malik	The emerging role of quantum computations in elucidating adsorption mechanism of heavy metal ions: a review	2022
8966	J. T. Tsiepe	Fe3O4-beta-cyclodextrin-Chitosan Bionanocomposite for Arsenic Removal from Aqueous Solution	2018
8967	L. Lin	Sorption of metals and metalloids from reverse osmosis concentrate on drinking water treatment solids	2014
8968	H. W. Wang	A microscopic and spectroscopic study of rapid antimonite sequestration by a poorly crystalline phyllomanganate: differences from passivated arsenite oxidation	2017
8969	Y. Wang	Efficient removal arsenate from water by biochar-loaded Ce3+-enriched ultra-fine ceria nanoparticles through adsorption-precipitation	2021
8970	Z. H. Khan	The sorbed mechanisms of engineering magnetic biochar composites on arsenic in aqueous solution	2020
8971	Q. Yang	Flow and sorption controls of groundwater arsenic in individual boreholes from bedrock aquifers in central Maine, USA	2015
8972	V. Marjanovic	Selenate Adsorption from Water Using the Hydrous Iron Oxide-Impregnated Hybrid Polymer	2020
8973	J. J. Lin	The stabilizing mechanism of cadmium in contaminated soil using green synthesized iron oxide nanoparticles under long-term incubation	2019
8974	I. E. M. Carpio	Graphene oxide functionalized with ethylenediamine triacetic acid for heavy metal adsorption and anti-microbial applications	2014
8975	Y. F. Li	Characterization of the cell-Fe mineral aggregate from nitrogen removal employing ferrous and its adsorption features to heavy metal	2017
8976	E. Arco-Lazaro	Arsenic adsorption and plant availability in an agricultural soil irrigated with As-rich water: Effects of Fe-rich amendments and organic and inorganic fertilisers	2018
8977	K. Telfeyan	Arsenic, vanadium, iron, and manganese biogeochemistry in a deltaic wetland, southern Louisiana, USA	2017
8978	W. Han	A mesoporous titanium glycolate with exceptional adsorption capacity to remove multiple heavy metal ions in water	2017
8979	Y. Shi	In situ regeneration of commercial NH3-SCR catalysts with high-temperature water vapor	2018
8980	S. F. Zhou	Electrochemical detection of As(III) through mesoporous MnFe2O4 nanocrystal clusters by square wave stripping voltammetry	2015

8981	S. G. Johnston	Rapid arsenic(V)-reduction by fire in schwertmannite-rich soil enhances arsenic mobilisation	2018
8982	M. Sepulveda	IN SITU X-RAY FLUORESCENCE ANALYSIS OF ROCK ART PAINTINGS ALONG THE COAST AND VALLEYS OF THE ATACAMA DESERT, NORTHERN CHILE	2015
8983	R. Tanta	Morphology and composition of oxidized InAs nanowires studied by combined Raman spectroscopy and transmission electron microscopy	2016
8984	D. B. Sims	Trace Element Scavenging in Dry Wash Surficial Sediments in an Arid Region of Southern Nevada, USA	2017
8985	A. V. Maiorova	Extraction of Zinc and Arsenic from Metallurgical Furnace Dust	2021
8986	B. H. Li	Effects of alfalfa root exudates on the microbial remediation of nitrate-heavy metal complex pollution in groundwater	2021
8987	H. Guenet	Evidence of organic matter control on As oxidation by iron oxides in riparian wetlands	2016
8988	M. Weil	Two Modifications of (TeO)(HAsO4) and its Dehydration Product (Te3O3)(AsO4)(2) - Three more Examples of Tellurium(IV) with a Fivefold Oxygen Coordination	2014
8989	A. Mickiewicz	Groundwater geochemical evolution under the influence of polymetallic deposit in Czarnow (Western Sudetes, SW Poland)	2016
8990	P. E. Evans	The role of amine in the sequestration of As (III) on functionalized indium tin oxide	2021
8991	H. Rezala	Adsorption of Methylene Blue by Hydroxyl-Aluminum Pillared Montmorillonite	2020
8992	Y. M. Kamil	Di-Iron Trioxide Hydrate-Multi-Walled Carbon Nanotube Nanocomposite for Arsenite Detection Using Surface Plasmon Resonance Technique	2019
8993	J. Wang	A vacuum calcination route to high-surface-area MgO nanoplates for superior arsenate adsorption and catalytic properties	2018
8994	C. D. Moffat	Molecular Recognition and Scavenging of Arsenate from Aqueous Solution Using Dimetallic Receptors	2014
8995	L. Zhou	New trends of molecular probes based on the fluorophore 4-amino-1,8-naphthalimide	2019
8996	J. W. Stuckey	Reactivity and speciation of mineral-associated arsenic in seasonal and permanent wetlands of the Mekong Delta	2015
8997	C. Tan	Separation of As from high As-Sb dust using Fe2O3 as a fixative under O-2-N-2 atmosphere	2018
8998	A. M. R. Neiva	Uranium and arsenic contamination in the former Mondego Sul uranium mine area, Central Portugal	2016

8999	E. Hernandez -Mendiola	Arsenic and lead in the soils of San Antonio-El Triunfo mining district, BCS, Mexico: a human health risk assessment	2022
9000	W. W. Zhai	Reduced Cd, Pb, and As accumulation in rice (Oryza sativa L.) by a combined amendment of calcium sulfate and ferric oxide	2020
9001	L. K. ThomasArrigo	Iron and Arsenic Speciation and Distribution in Organic Flocs from Streambeds of an Arsenic-Enriched Peat land	2014
9002	H. M. Guo	Arsenic mobilization in aquifers of the southwest Songnen basin, PR China: Evidences from chemical and isotopic characteristics	2014
9003	P. Devi	Selective electrochemical sensing for arsenite using rGO/Fe3O4 nanocomposites	2017
9004	X. H. Wang	Functionalized Biochar/Clay Composites for Reducing the Bioavailable Fraction of Arsenic and Cadmium in River Sediment	2019
9005	W. H. An	Effects of biochar/AQDS on As(III)-adsorbed ferrihydrite reduction and arsenic (As) and iron (Fe) transformation: Abiotic and biological conditions	2022
9006	X. Y. Qin	In situ surface and interface study of crystalline (3 x 1)-O on InAs	2016
9007	H. Q. Huang	Noble-metal-free Fe3O4/Co3S4 nanosheets with oxygen vacancies as an efficient electrocatalyst for highly sensitive electrochemical detection of As(III)	2022
9008	D. Y. Tao	Determination of As species distribution and variation with time in extracted groundwater samples by on-site species separation method	2022
9009	X. Q. Chi	Targeted arsenite-loaded magnetic multifunctional nanoparticles for treatment of hepatocellular carcinoma	2019
9010	P. Maneechakr	Adsorption behavior of As(V) from aqueous solution by using Fe3+-MnO4--modified activated carbon (Leucaena leucocephala (Lam) de Wit)	2018
9011	R. C. Cagnin	Arsenic enrichment in sediment on the eastern continental shelf of Brazil	2017
9012	I. Fatemi	Mechanisms involved in the possible protective effect of chrysin against sodium arsenite-induced liver toxicity in rats	2021
9013	Y. F. Zhang	Persistent arsenate-iron(iii) oxyhydroxide-organic matter nanoaggregates observed in coal	2021
9014	Q. Li	Comparative removal of As(V) and Sb(V) from aqueous solution by sulfide-modified alpha-FeOOH	2020
9015	R. C. Rodriguez	Au nanoparticles embedded in mesoporous ZrO2 films: Multifunctional materials for electrochemical detection	2018
9016	H. Y. Yang	Lanthanum(III)-Coated Ceramics as a Promising Material in Point-of-Use Water Treatment for Arsenite and Arsenate Removal	2019

9017	F. Liu	Simultaneous Oxidation and Sequestration of Arsenic(III) from Aqueous Solution by Copper Aluminate with Peroxymonosulfate: A Fast and Efficient Heterogeneous Process	2021
9018	M. S. Podder	Studies on the removal of As(III) and As(V) through their adsorption onto granular activated carbon/MnFe2O4 composite: isotherm studies and error analysis	2016
9019	H. P. Zeng	Adsorption of As(III) from aqueous solutions using MnO2 strengthened WTRs-chitosan beads made by homogenous method with freeze-drying	2021
9020	J. C. J. Gude	Biological As(III) oxidation in rapid sand filters	2018
9021	Z. W. Qiu	Ternary metal composite membrane FCMNCM enhances the separation of As(III) in water through the multifunctional cooperation	2021
9022	L. N. Pincus	Selective adsorption of arsenic over phosphate by transition metal cross-linked chitosan	2021
9023	B. B. RasulKhan	A new CQDs/f-MWCNTs/GO nanocomposite electrode for arsenic (10(-12) M) quantification in bore-well water and industrial effluents	2020
9024	J. Jeong	Arsenic(III) detection using electrochemical-chemical-chemical redox cycling at bare indium-tin oxide electrodes	2014
9025	X. Yang	Elucidating the redox-driven dynamic interactions between arsenic and iron-impregnated biochar in a paddy soil using geochemical and spectroscopic techniques	2022
9026	A. Y. Zhang	Photochemical pollutant degradation on facet junction-engineered TiO2 promoted by organic arsenical: Governing roles of arsenic-terminated surface chemistry and bulk-free radical speciation	2020
9027	J. Cantu	Removal of arsenic from water using synthetic Fe7S8 nanoparticles	2016
9028	G. Etiope	A thermogenic hydrocarbon seep in shallow Adriatic Sea (Italy): Gas origin, sediment contamination and benthic foraminifera	2014
9029	B. Zawisza	Cellulose mini-membranes modified with TiO2 for separation, determination, and speciation of arsenates and selenites	2020
9030	J. J. Hou	In-situ preparation of novel sedimentary rock-like Fe3O4 by rice-husk mesoporous silica as templates for effective remove As(III) from aqueous solutions	2021
9031	P. Devi	Co-electrodeposited rGO/MnO2 nanohybrid for arsenite detection in water by stripping voltammetry	2016
9032	N. Jordanova	Advanced mineral magnetic and geochemical investigations of road dusts for assessment of pollution in urban areas near the largest copper smelter in SE Europe	2021
9033	A. A. Elabd	Uranyl ions adsorption by novel metal hydroxides loaded Amberlite IR120	2014
9034	S. B. Li	Adsorptive Bromate Removal from Aqueous Solution by Commercial Strongly Basic Resin Impregnated with Hydrated Ferric Oxide (HFO): Kinetics and Equilibrium Studies	2016

9035	A. Balati	Heterojunction of vertically aligned MoS2 layers to Hydrogenated Black TiO2 and Rutile Based Inorganic Hollow Microspheres for the highly enhanced visible light arsenic photooxidation	2020
9036	M. S. Aksenov	Anodic layer formation on the InAlAs surface in Townsend gas-discharge plasma	2019
9037	M. Sadani	Arsenic selective adsorption using a nanomagnetic ion imprinted polymer: Optimization, equilibrium, and regeneration studies	2020
9038	A. Li	Hepatorenal protective effects of medicinal herbs in An-Gong-Niu-Huang Wan (AGNH) against cinnabar- and realgar-induced oxidative stress and inflammatory damage in mice	2018
9039	H. B. Li	Comparison of arsenic bioaccessibility in housedust and contaminated soils based on four in vitro assays	2015
9040	A. Ebrahiminezhad	Green synthesis and characterization of zero-valent iron nanoparticles using stinging nettle (Urtica dioica) leaf extract	2017
9041	A. S. Sokhan	Effects of N-Acetyl Cysteine on the Expression of Matrix Metalloproteinases 2 and 9 in the Lung Tissue of Rats Exposed to Cadmium	2020
9042	X. Y. Jin	Relationship between Carlin-type gold deposits and paleo-petroleum reservoirs in SW Guizhou, China: Evidence from gas compositions of fluid inclusions and Raman spectroscopic characteristics of bitumen	2016
9043	V. K. Sharma	Magnetic graphene-carbon nanotube iron nanocomposites as adsorbents and antibacterial agents for water purification	2015
9044	C. I. Weng	Detection of Arsenic(III) through Pulsed Laser-Induced Desorption/Ionization of Gold Nanoparticles on Cellulose Membranes	2014
9045	J. Na	Phosphoric acid-peroxide mixture surface preparation for the improvement of InGaAs channel characteristics	2021
9046	M. Ueberschaar	Challenges for critical raw material recovery from WEEE - The case study of gallium	2017
9047	M. Kaur	Magnesium ferrite-nitrogen-doped graphene oxide nanocomposite: effective adsorptive removal of lead(II) and arsenic(III)	2022
9048	M. B. Baskan	Removal of arsenate using graphene oxide-iron modified clinoptilolite-based composites: adsorption kinetic and column study	2021
9049	J. V. Vaghasiya	Pick up and dispose of pollutants from water via temperature-responsive micellar copolymers on magnetite nanorobots	2022
9050	T. Mahmood	ADSORPTION OFAs(III) FROMAQUEOUS SOLUTION ONTO IRON IMPREGNATED USED TEA ACTIVATED CARBON: EQUILIBRIUM, KINETIC AND THERMODYNAMIC STUDY	2018
9051	K. Wrighton-Araneda	Effective removal of water-soluble methylated arsenic contaminants with phosphorene oxide nanoflakes: A DFT study	2021
9052	H. W. Chen	Comparing arsenic(V) adsorption by two types of red soil weathered from granite and sandstone in Hunan, China	2021

9053	M. Porta	Influence of KRAS mutations, persistent organic pollutants, and trace elements on survival from pancreatic ductal adenocarcinoma	2020
9054	Y. P. Wang	Straw biochar enhanced removal of heavy metal by ferrate	2021
9055	C. Shan	Enhanced Fenton-like Oxidation of As(III) over Ce-Ti Binary Oxide: A New Strategy to Tune Catalytic Activity via Balancing Bimolecular Adsorption Energies	2020
9056	T. W. Chen	Facile synthesis of mesoporous WS2 nanorods decorated N-doped RGO network modified electrode as portable electrochemical sensing platform for sensitive detection of toxic antibiotic in biological and pharmaceutical samples	2019
9057	L. Chen	Preferable removal of phosphate from water using hydrous zirconium oxide-based nanocomposite of high stability	2015
9058	Q. H. Yan	A comprehensive review on selective catalytic reduction catalysts for NOx emission abatement from municipal solid waste incinerators	2016
9059	C. M. van Genuchten	Mn(II) Oxidation in Fenton and Fenton Type Systems: Identification of Reaction Efficiency and Reaction Products	2017
9060	H. J. Zhang	Carboxyl-functionalized graphene oxide polyamide nanofiltration membrane for desalination of dye solutions containing monovalent salt	2017
9061	X. L. You	Phosphate removal from aqueous solution using a hybrid impregnated polymeric sorbent containing hydrated ferric oxide (HFO)	2016
9062	M. Gifford	Reducing environmental impacts of metal (hydr)oxide nanoparticle embedded anion exchange resins using anticipatory life cycle assessment	2016
9063	L. B. Zhong	Facile one-pot synthesis of urchin-like Fe-Mn binary oxide nanoparticles for effective adsorption of Cd(II) from water	2016
9064	H. de The	Acute Promyelocytic Leukemia: A Paradigm for Oncoprotein-Targeted Cure	2017
9065	J. Xue	Studies on the Inhibitory Effects of Nano-Hydroxyapatite-Loaded As2O3 on Hepatoma Cells	2020
9066	K. Z. Benis	Enhanced arsenate removal by Fe-impregnated canola straw: assessment of XANES solid-phase speciation, impacts of solution properties, sorption mechanisms, and evolutionary polynomial regression (EPR) models	2021
9067	U. A. Usman	Trace metals geochemistry for health assessment coupled with adsorption remediation method for the groundwater of Lorong Serai 4, Hulu Langat, west coast of Peninsular Malaysia	2020
9068	E. Di Iorio	Arsenate retention mechanisms on hematite with different morphologies evaluated using AFM, TEM measurements and vibrational spectroscopy	2018
9069	R. Veenema	Inorganic arsenic exposure induces sex-disparate effects and exacerbates ischemia-reperfusion injury in the female heart	2019
9070	N. Pawlas	Telomere length in children environmentally exposed to low-to-moderate levels of lead	2015

9071	D. A. Chernikova	The premature infant gut microbiome during the first 6 weeks of life differs based on gestational maturity at birth	2018
9072	I. Q. Lima	Geochemical mechanisms of natural arsenic mobility in the hydrogeologic system of Lower Katari Basin, Bolivian Altiplano	2021
9073	Y. X. Deng	Understanding major NOM properties controlling its interactions with phosphorus and arsenic at goethite-water interface	2019
9074	J. Dobrzynska	Raspberry stalks-derived biochar, magnetic biochar and urea modified magnetic biochar-Synthesis, characterization and application for As(V) and Cr(VI) removal from river water	2022
9075	K. S. Nitzsche	Microbial community composition of a household sand filter used for arsenic, iron, and manganese removal from groundwater in Vietnam	2015
9076	G. England-Mason	Prenatal exposure to phthalates and peripheral blood and buccal epithelial DNA methylation in infants: An epigenome-wide association study	2022
9077	A. K. Bozack	Prenatal metal exposure, cord blood DNA methylation and persistence in childhood: an epigenome-wide association study of 12 metals	2021
9078	H. V. Kulkarni	Influence of monsoonal recharge on arsenic and dissolved organic matter in the Holocene and Pleistocene aquifers of the Bengal Basin	2018
9079	Y. F. Jia	Sources of groundwater salinity and potential impact on arsenic mobility in the western Hetao Basin, Inner Mongolia	2017
9080	P. D. Sly	The interplay between environmental exposures and COVID-19 risks in the health of children	2021
9081	Z. Y. Zhang	Synchronous release of labile phosphorus, labile ferrum, and labile arsenic in water-sediment interface using ZrO-Chelex measured in the Aibi Lake of Northwest China	2018
9082	S. Khan	Arsenic interaction and bioaccumulation in food crops grown on degraded soil: Effect on plant nutritional components and other dietary qualities	2019
9083	M. Y. Stuckman	Biotic arsenic release from spent adsorbents under anaerobic landfill conditions	2017
9084	L. A. Richards	Contrasting sorption behaviours affecting groundwater arsenic concentration in Kandal Province, Cambodia	2019
9085	M. Tripathy	Maghemite and Graphene Oxide Embedded Polyacrylonitrile Electrospun Nanofiber Matrix for Remediation of Arsenate Ions	2020
9086	V. L. Tauson	Dualistic Distribution Coefficients of Trace Elements in the System Mineral-Hydrothermal Solution. IV. Platinum and Silver in Pyrite	2017
9087	J. Herrmann	Secondary Fe-As-Tl mineralization in soils near Buus in the Swiss Jura Mountains	2018
9088	M. Usman	Pre-deposited dynamic membrane adsorber formed of microscale conventional iron oxide-based adsorbents to remove arsenic from water: application study and mathematical modeling	2021

9089	Z. F. He	Simultaneous removal of As(III) and Cu(II) from real bottom ash leachates by manganese-oxidizing aerobic granular sludge: Performance and mechanisms	2020
9090	V. N. Priya	Removal of As (V) in water using beta-Cyclodextrin intercalated Fe-Al layered double hydroxide/reduced Graphene Oxide nanocomposites	2020
9091	Y. H. Bai	Treatment of groundwater containing Mn(II), Fe(II), As(III) and Sb(III) by bioaugmented quartz-sand filters	2016
9092	M. M. Hussain	Arsenic biogeochemical cycling in paddy soil-rice system: Interaction with various factors, amendments and mineral nutrients	2021
9093	S. V. S. Rana	Endoplasmic Reticulum Stress Induced by Toxic Elements-a Review of Recent Developments	2020
9094	M. C. Moncur	Source and distribution of naturally occurring arsenic in groundwater from Alberta's Southern Oil Sands Regions	2015
9095	S. H. Lee	Removal of As(V) and Sb(V) in water using magnetic nanoparticle-supported layered double hydroxide nanocomposites	2018
9096	A. K. Lightfoot	Noble gas constraints on the fate of arsenic in groundwater	2022
9097	M. A. Dkhil	Chlorogenic acid prevents hepatotoxicity in arsenic-treated mice: role of oxidative stress and apoptosis	2020
9098	S. Crognale	Biological As(III) oxidation in biofilters by using native groundwater microorganisms	2019
9099	S. Bavec	Geochemical investigation of potentially harmful elements in household dust from a mercury-contaminated site, the town of Idrija (Slovenia)	2017
9100	H. Y. Wang	Indications that weathering of evaporite minerals affects groundwater salinity and As mobilization in aquifers of the northwestern Hetao Basin, China	2019
9101	S. A. Ronchetti	In vivo xenoestrogenic actions of cadmium and arsenic in anterior pituitary and uterus	2016
9102	P. Biswas	Anomalous diffusion of Ga and As from semi-insulating GaAs substrate into MOCVD grown ZnO films as a function of annealing temperature and its effect on charge compensation	2014
9103	A. Gholami	Pentoxifylline Attenuates Arsenic Trioxide-Induced Cardiac Oxidative Damage in Mice	2021
9104	L. Li	Effectiveness and limitation of A-nZVI for restoration of a highly As-contaminated soil	2021
9105	P. Gimenez-Gomez	Automated Determination of As(III) in Waters with an Electrochemical Sensor Integrated into a Modular Microfluidic System	2019
9106	C. Wang	Occurrence state of co-existing arsenate and nickel ions at the ferrihydrite-water interface: Mechanisms of surface complexation and surface precipitation via ATR-IR spectroscopy	2018

9107	S. W. Yun	Dissolution behavior of As and Cd in submerged paddy soil after treatment with stabilizing agents	2016
9108	B. Xu	Influence of Br24 and Gr24 on the Accumulation and Uptake of Cd and As by Rice Seedlings Grown in Nutrient Solution	2019
9109	S. A. Hashemi	Effective removal of mercury, arsenic and lead from aqueous media using Polyaniline-Fe3O4-silver diethyldithiocarbamate nanostructures	2019
9110	H. Badri	Geochemical compositional controls on DNA strand breaks induced in in vitro cell-free assays by crushed rock powders from the Panasqueira mine area, Portugal	2021
9111	S. R. Ryu	A combination of reducing and chelating agents for electrolyte conditioning in electrokinetic remediation of As-contaminated soil	2017
9112	J. A. Arcibar-Orozco	Interactions between reactive oxygen groups on nanoporous carbons and iron oxyhydroxide nanoparticles: effect on arsenic(V) removal	2016
9113	B. Kalyani	Effect of Bi2O3 on AC conductivity and dielectric properties of alkali arsenate bro-bismuthate glasses	2022
9114	J. G. Wang	What has traditional Chinese medicine delivered for modern medicine?	2018
9115	X. Y. Zang	Aging of exogenous arsenic in flooded paddy soils: Characteristics and predictive models	2021
9116	Z. F. He	Complex effects of pH and organic shocks on arsenic oxidation and removal by manganese-oxidizing aerobic granular sludge in sequencing batch reactors	2020
9117	G. Arce	Enhancement of particle aggregation in the presence of organic matter during neutralization of acid drainage in a stream confluence and its effect on arsenic immobilization	2017
9118	L. H. Liu	High-efficiency As(III) oxidation and electrocoagulation removal using hematite with a charge-discharge technique	2020
9119	D. A. Da'ana	Removal of Toxic Elements and Microbial Contaminants from Groundwater Using Low-Cost Treatment Options	2021
9120	A. T. Jacobson	Evaluation of natural goethite on the removal of arsenate and selenite from water	2019
9121	A. Paltseva	Accumulation of arsenic and lead in garden-grown vegetables: Factors and mitigation strategies	2018
9122	F. Yang	Complex role of titanium dioxide nanoparticles in the trophic transfer of arsenic from Nannochloropsis maritima to Artemia sauna nauplii	2018
9123	A. Varga	ORIGIN OF NATURAL ARSENIC AND ANTIMONY CONTENTS IN THE PERMIAN TO LOWER TRIASSIC SILICICLASTIC ROCKS OF THE WESTERN MECSEK MOUNTAINS, SW HUNGARY	2017
9124	X. Q. Wang	Arsine adsorption in copper-exchanged zeolite under low temperature and micro-oxygen conditions	2017

9125	M. Wu	Arsenic(V) removal in wetland filters treating drinking water with different substrates and plants	2014
9126	S. Ramos-Vargas	Removal of Fluoride and Arsenate from Aqueous Solutions by Aluminum-Modified Guava Seeds	2018
9127	A. Biswas	Geochemistry of arsenic in low sulfide-high carbonate coal waste rock, Elk Valley, British Columbia, Canada	2017
9128	R. M. Couture	On-Off Mobilization of Contaminants in Soils during Redox Oscillations	2015
9129	A. Rodriguez-Hernandez	Geochemical and mineralogical characterization of stream sediments impacted by mine wastes containing arsenic, cadmium and lead in North-Central Mexico	2021
9130	R. Khosravi	Geochemical and hydrological controls of arsenic concentrations across the sediment-water interface at Maharlu Lake, Southern Iran	2019
9131	C. Chen	Fe(II)-Induced Mineral Transformation of Ferrihydrite-Organic Matter Adsorption and Co-precipitation Complexes in the Absence and Presence of As(III)	2018
9132	M. Yang	Electrochemical Detection of Trace Arsenic(III) by Nanocomposite of Nanorod-Like alpha-MnO2 Decorated with similar to 5 nm Au Nanoparticles: Considering the Change of Arsenic Speciation	2016
9133	C. Y. Li	Ultrahigh sensitivity electroanalysis of trace As(III) in water and human serum via gold nanoparticles uniformly anchored to Co3O4 porous microsheets	2021
9134	M. Goudarzi	Protective Effect of Ellagic Acid Against Sodium Arsenite-Induced Cardio- and Hematotoxicity in Rats	2018
9135	A. T. Craig	Heated column experiments: A proxy for investigating the effects of in situ thermal recovery operations on groundwater geochemistry	2021
9136	N. Dalla Libera	Conceptual Model of Arsenic Mobility in the Shallow Alluvial Aquifers Near Venice (Italy) Elucidated Through Machine Learning and Geochemical Modeling	2020
9137	G. A. Zanor	Sources and distribution of arsenic in agricultural soils of Central Mexico	2019
9138	Z. Y. Wang	Arsenate adsorption on iron-impregnated ordered mesoporous carbon: Fast kinetics and mass transfer evaluation	2019
9139	R. Prabhakar	Efficient loading of nano Mn particles on calcined laterite soil (Lt-nMn) for higher removal of As(III) ions from groundwater: adsorption and eco-scale analysis	2022
9140	P. Pham	Removal of As(III) from Water Using the Adsorptive and Photocatalytic Properties of Humic Acid-Coated Magnetite Nanoparticles	2020
9141	R. Martin	Size-dependent characterisation of historical gold mine wastes to examine human pathways of exposure to arsenic and other potentially toxic elements	2016
9142	T. A. Saleh	Factorial design, physical studies and rapid arsenic adsorption using newly prepared polymer modified perlite adsorbent	2022

9143	K. R. Hakeem	Biochar promotes arsenic (As) immobilization in contaminated soils and alleviates the As-toxicity in soybean (Glycine max (L.) Merr.)	2022
9144	D. Pedretti	A geologically-based approach to map arsenic risk in crystalline aquifers: Analysis of the Tampere region, Finland	2019
9145	J. T. Markwiese	Natural attenuation of coal combustion waste in river sediments	2014
9146	S. Das	Arsenate adsorption onto hematite nanoparticles under alkaline conditions: effects of aging	2014
9147	J. M. Peralta	Targeting redox metabolism of the maize-Azospirillum brasilense interaction exposed to arsenic-affected groundwater	2021
9148	S. Madan	Enhancing corrosion stability and shelf life of nanoscale zero-valent iron via encapsulation in porous Ze-TiO2 matrix: An interface for simultaneous oxidation and adsorption of As(III)	2020
9149	M. Arsic	Diffusive Gradients in Thin Films Reveals Differences in Antimony and Arsenic Mobility in a Contaminated Wetland Sediment during an Oxic-Anoxic Transition	2018
9150	R. Singh	Regulation of oxidative stress and mineral nutrient status by selenium in arsenic treated crop plant Oryza sativa	2018
9151	L. L. Min	Preparation of chitosan based electrospun nanofiber membrane and its adsorptive removal of arsenate from aqueous solution	2015
9152	L. W. Yin	Synthesis and characterization of Nanoscale Zero-Valent Iron (nZVI) as an adsorbent for the simultaneous removal of As(III) and As(V) from groundwater	2022
9153	S. Liu	The provenance of deep groundwater and its relation to arsenic distribution in the northwestern Hetao Basin, Inner Mongolia	2020
9154	C. J. Jiang	A comparative study on Fe(III)-chitosan and Fe(III)-chitosan-CTAB composites for As(V) removal from water: preparation, characterization and reaction mechanism	
9155	K. Wang	The fate of arsenic in rice plants (Oryza sativa L.): Influence of different forms of selenium	2021
9156	F. B. Quinodoz	The development of a conceptual model for arsenic mobilization in a fluvio-eolian aquifer using geochemical and statistical methods	2019
9157	H. Guenet	Characterization of iron-organic matter nano-aggregate networks through a combination of SAXS/SANS and XAS analyses: impact on As binding	2017
9158	A. Dados	In situ trapping of As, Sb and Se hydrides on nanometer-sized ceria-coated iron oxide-silica and slurry suspension introduction to ICP-OES	2014
9159	J. Gao	Microbially mediated mobilization of arsenic from aquifer sediments under bacterial sulfate reduction	2021
9160	D. Spanu	Photoelectrocatalytic oxidation of As(III) over hematite photoanodes: A sensible indicator of the presence of highly reactive surface sites	2018

9161	S. Hafeznezami	Adsorption and desorption of arsenate on sandy sediments from contaminated and uncontaminated saturated zones: Kinetic and equilibrium modeling	2016
9162	H. Liu	Titanium Dioxide-Coated Carbon Nanotube Network Filter for Rapid and Effective Arsenic Sorption	2014
9163	S. Verma	Influence of geology on groundwater-sediment interactions in arsenic enriched tectono-morphic aquifers of the Himalayan Brahmaputra river basin	2016
9164	J. Q. Zeng	Chitosan functionalized iron nanosheet for enhanced removal of As(III) and Sb(III): Synergistic effect and mechanism	2020
9165	S. Bagherifam	Evaluation of Zn-Al-SO4 layered double hydroxide for the removal of arsenite and arsenate from a simulated soil solution: Isotherms and kinetics	2014
9166	Z. Y. Chen	Detection of mercury in spiked cosmetics by surface enhanced Raman spectroscopy using silver shelled iron oxide nanoparticles	2021
9167	A. B. Radkova	Antimony mobility during the early stages of stibnite weathering in tailings at the Beaver Brook Sb deposit, Newfoundland	2020
9168	J. C. J. Gude	As(III) oxidation by MnO2 during groundwater treatment	2017
9169	G. Visani	Nanomedicine strategies for hematological malignancies: what is next?	2014
9170	L. L. Sui	Nanocomposites of Fe2O3@rGO for adsorptive removal of arsanilic acid from aqueous solution	2021
9171	Y. H. Wang	Diffusion- and pH-Dependent Reactivity of Layer-Type MnO2: Reactions at Particle Edges versus Vacancy Sites	2018
9172	S. S. Wang	Enhanced arsenic removal by biochar modified with nickel (Ni) and manganese (Mn) oxyhydroxides	2016
9173	W. S. Choi	Nanostructured Materials for Water Purification: Adsorption of Heavy Metal Ions and Organic Dyes	2022
9174	Y. Takaya	Removal mechanisms of arsenite by coprecipitation with ferrihydrite	2021
9175	M. Alimohammady	Competent Heavy Metal Adsorption by Modified MWCNTs and Optimization Process by Experimental Design	2018
9176	Y. X. Zhang	A facile solution approach for the synthesis of akaganeite (beta-FeOOH) nanorods and their ion-exchange mechanism toward As(V) ions	2014
9177	R. Wilson	Mine-to-smelter integration framework for regional development of porphyry copper deposits within the Chilean context	2022
9178	J. Hua	Anoxic oxidation of As(III) during Fe(II)-induced goethite recrystallization: Evidence and importance of Fe(IV) intermediate	2022

9179	M. Li	Layered Double Hydroxide/Chitosan Nanocomposite Beads as Sorbents for Selenium Oxoanions	2018
9180	H. Tong	Microaerophilic Oxidation of Fe(II) Coupled with Simultaneous Carbon Fixation and As(III) Oxidation and Sequestration in Karstic Paddy Soil	2021
9181	A. B. Chimezie	Fabrication of reduced graphene oxide-magnetic nanocomposite (rGO-Fe3O4) as an electrochemical sensor for trace determination of As(III) in water resources	2017
9182	S. Alamri	Priming of tomato seedlings with 2-oxoglutarate induces arsenic toxicity alleviatory responses by involving endogenous nitric oxide	2021
9183	T. Sukitprapanon	Nature of redox concentrations in a sequence of agriculturally developed acid sulfate soils in Thailand	2020
9184	P. Xing	Cleaning of lead smelting flue gas scrubber sludge and recovery of lead, selenium and mercury by the hydrometallurgical route	2018
9185	M. E. Mahmoud	Engineered nano-zirconium oxide-crosslinked-nanolayer of carboxymethyl cellulose for speciation and adsorptive removal of Cr(III) and Cr(VI)	2017
9186	L. D. R. Garcia	Solubility and Disintegration of New Calcium Aluminate Cement (EndoBinder) Containing Different Radiopacifying Agents	2014
9187	A. A. Markeb	Phosphate removal and recovery from water using nanocomposite of immobilized magnetite nanoparticles on cationic polymer	2016
9188	V. Vaiano	Enhanced photocatalytic oxidation of arsenite to arsenate in water solutions by a new catalyst based on MoOx supported on TiO2	2014
9189	T. C. Prathna	Nanoparticles in household level water treatment: An overview	2018
9190	A. Voegelin	Thallium Speciation and Extractability in a Thallium- and Arsenic-Rich Soil Developed from Mineralized Carbonate Rock	2015
9191	I. Blinova	Ecotoxicity of nanosized magnetite to crustacean Daphnia magna and duckweed Lemna minor	2017
9192	E. Kelepertzis	Geochemical associations for evaluating the availability of potentially harmful elements in urban soils: Lessons learnt from Athens, Greece	2015
9193	Y. John	A Comparative Study on Removal of Hazardous Anions from Water by Adsorption: A Review	2018
9194	Y. Wang	Alleviative effect of selenium on inflammatory damage caused by lead via inhibiting inflammatory factors and heat shock proteins in chicken testes	2017
9195	L. J. Wu	Dephosphorization using ceramsites modified by coprecipitation with FeSo(4) and KMnO4 and high-temperature combustion	2020
9196	P. C. Bennett	Source and migration of dissolved manganese in the Central Nile Delta Aquifer, Egypt	2014

9197	T. J. Jiang	Gold electrode modified with ultrathin SnO2 nanosheets with high reactive exposed surface for electrochemical sensing of As(III)	2016
9198	S. T. El-Wakeel	Efficient removal of Pb(II), Hg(II) and As(III) ions by a recyclable low cost magnetic hydroxyapatite nanocomposite from aqueous solution	2021
9199	T. A. Vu	Arsenic removal from aqueous solutions by adsorption using novel MIL-53(Fe) as a highly efficient adsorbent	2015
9200	S. T. Yang	Comparison of Arsenate and Molybdate Speciation in Hydrogenetic Ferromanganese Nodules	2019
9201	S. B. Gautam	Adsorption of As(III) from aqueous solutions by iron-impregnated quartz, lignite, and silica sand: kinetic study and equilibrium isotherm analysis	2014
9202	X. Q. Chi	Recent advances of nanomedicines for liver cancer therapy	2020
9203	W. Liu	High temperature MIC on an offshore pipeline and the strong arsenate-reduction function in the hyperthermophiles	2014
9204	J. Han	Identification of Bernalite Transformation and Tridentate Arsenate Complex at Nano-goethite under Effects of Drying, pH and Surface Loading	2018
9205	Y. Z. Zhou	Characteristics and implication of stable carbon isotope in high arsenic groundwater systems in the northwest Hetao Basin, Inner Mongolia, China	2018
9206	X. M. Wang	Binding Geometries of Silicate Species on Ferrihydrite Surfaces	2018
9207	G. Araya	Leaching of Oxide Copper Ores by Addition of Weak Acid from Copper Smelters	2020
9208	L. Bucker-Neto	Interactions between plant hormones and heavy metals responses	2017
9209	Z. M. Liu	Novel hematite nanorods and magnetite nanoparticles prepared from MIL-100(Fe) template for the removal of As(V)	2014
9210	C. Shan	Non-radical pathway dominated catalytic oxidation of As(III) with stoichiometric H2O2 over nanoceria	2019
9211	M. Sedki	Linker-Free Magnetite-Decorated Gold Nanoparticles (Fe3O4-Au): Synthesis, Characterization, and Application for Electrochemical Detection of Arsenic (III)	2021
9212	D. Habrant	Reactivity of aldehydes with semi-stabilised arsonium ylide anions: Synthesis of terminal (E)-1,3-dienes	2007
9213	M. B. Tahir	Role of Nano-Photocatalysts in Detoxification of Toxic Heavy Metals	2021
9214	L. Yu	Depression mechanism involving Fe3+ during arsenopyrite flotation	2019

9215	Z. Olah	Development of Ge-77/As-77 parent-daughter system for periodic removal of As-77 for environmental sanitation and biochemical purposes	2015
9216	J. P. H. Perez	Adsorption and Reduction of Arsenate during the Fe2+-Induced Transformation of Ferrihydrite	2019
9217	W. J. Ma	Molecular Mechanisms of Cardiomyocyte Death in Drug-Induced Cardiotoxicity	2020
9218	P. L. Tuan	Investigations of chemical and atomic composition of native oxide layers covering SI GaAs implanted with Xe ions	2020
9219	M. Lv	Fighting against hematological malignancy in China: from unique system to global impact	2015
9220	J. M. O. Souza	Distribution of arsenic and oxidative stress in mice after rice ingestion	2017
9221	C. Y. Chang	Impact of La2O3/InGaAs MOS Interfaces on the Performance of InGaAs MOSFETs	2017
9222	J. A. Sans	Unveiling the role of the lone electron pair in sesquioxides at high pressure: compressibility of beta-Sb2O3	2021
9223	Y. Y. Dong	The molecular targets of taurine confer anti-hyperlipidemic effects	2021
9224	S. D. Singh	Evaluation of interfacial structure of 111 and 001 oriented epitaxial NiO layers on GaAs substrate by non-destructive techniques	2019
9225	P. A. Gunka	Crystal Structure and Non-Hydrostatic Stress-Induced Phase Transition of Urotropine Under High Pressure	2021
9226	E. D. Flynn	Reductive transformations of layered manganese oxides by small organic acids and the fate of trace metals	2019
9227	Q. Y. Chen	Arsenic: A Global Environmental Challenge	2021
9228	R. V. Ghita	Evolution of surface oxides on GaAs	2015
9229	K. Udupa	Treatment of Acute Promyelocytic Leukemia with Single Agent Arsenic Trioxide: Experience from a Tertiary Care Center in India	2017
9230	J. S. Yamani	Adsorption of selenite and selenate by nanocrystalline aluminum oxide, neat and impregnated in chitosan beads	2014
9231	F. Y. Piao	Taurine Attenuates As2O3-Induced Autophagy in Cerebrum of Mouse Through Nrf2 Pathway	2017
9232	J. M. Long	The leaching of antimony and arsenic by simulated acid rain in three soil types from the world's largest antimony mine area	

9233	C. Waterlot	Arsenic mobility and speciation in contaminated kitchen garden and lawn soils: an evaluation of water for assessment of As phytoavailability	2015
9234	P. K. Wang	On the Electrodeposition of Arsenic in a Choline Chloride/Ethylene Glycol Deep Eutectic Solvent	2017
9235	K. N. Kumar	Investigations on electrical characteristics of (PbO)(30)(CuO)(x)(As2O3)((70-x)) glass ceramics	2017
9236	Y. M. Deng	Effect of microbially mediated iron mineral transformation on temporal variation of arsenic in the Pleistocene aquifers of the central Yangtze River basin	2018
9237	R. Q. Zhao	A new solvothermal route to crystalline proustite Ag3AsS3 with photocatalytic properties	2014
9238	Y. J. Yang	Iron isotope evidence for arsenic mobilization in shallow multi-level alluvial aquifers of Jianghan Plain, central China	2020
9239	J. L. McCall	Prenatal Cadmium Exposure Alters Proliferation in Mouse CD4(+) T Cells via LncRNA Snhg7	2022
9240	H. K. Christenson	Quantifying arsenate partitioning in aquatic systems: Narrowing the laboratory-real world gap with kinetic sediment extractions and the Diffuse Layer Model	2015
9241	T. Chen	Construction of Time-Resolved Luminescence Nanoprobe and Its Application in As(III) Detection	2020
9242	B. A. Ziegler	Rates of As and Trace-Element Mobilization Caused by Fe Reduction in Mixed BTEX-Ethanol Experimental Plumes	2015
9243	X. J. Hu	Statistical Analysis of Main and Interaction Effects on Cu(II) and Cr(VI) Decontamination by Nitrogen-Doped Magnetic Graphene Oxide	2016
9244	I. Hindersmann	Trace Element Solubility in a Multimetal-Contaminated Soil as Affected by Redox Conditions	2014
9245	A. Markowska	Hypoxia and its importance in the course of gynaecological cancers	2019
9246	C. M. Su	Environmental implications and applications of engineered nanoscale magnetite and its hybrid nanocomposites: A review of recent literature	2017
9247	L. J. Sun	Impact of sulfur (S) fertilization in paddy soils on copper (Cu) accumulation in rice (Oryza sativa L.) plants under flooding conditions	2016
9248	M. B. Gumpu	Amperometric determination of As(III) and Cd(II) using a platinum electrode modified with acetylcholinesterase, ruthenium(II)-tris(bipyridine) and graphene oxide	2018
9249	S. Ye	Fluorescent probes for in vitro and in vivo quantification of hydrogen peroxide	2020
9250	J. H. Bell	Fracture-focused fluid flow in an acid and redox-influenced system: diagenetic controls on cement mineralogy and geomorphology in the Navajo Sandstone	2014

9251	C. Y. Chang	Heavy metal accumulation in balsam pear and cowpea related to the geochemical factors of variable-charge soils in the Pearl River Delta, South China	2014
9252	K. M. Zhang	Exploration of Cs-O co-adsorption on GaAs(100) beta(2) (2x 4) surface at different sites from first-principles calculation	2021
9253	J. L. Wang	Bcl-2 hijacks the arsenic trioxide resistance in SH-SY5Y cells	2022
9254	X. Z. Yang	Adsorption of Trivalent Antimony from Aqueous Solution Using Graphene Oxide: Kinetic and Thermodynamic Studies	2015
9255	H. Dong	Electro-assisted regeneration of pH-sensitive ion exchangers for sustainable phosphate removal and recovery	2020
9256	Z. Wang	Flooding-drainage regulate the availability and mobility process of Fe, Mn, Cd, and As at paddy soil	2022
9257	M. F. Cai	Bioaccessibility of As and Pb in orchard and urban soils amended with phosphate, Fe oxide and organic matter	2017
9258	M. Nodehi	Preparation of GO/Fe3O4@PMDA/AuNPs nanocomposite for simultaneous determination of As3+ and Cu2+ by stripping voltammetry	2021
9259	Z. Wang	Synthesis of ?-MnO2@MIL-100(Fe) composite for p-arsanilic acid removal	2022
9260	S. Kundu	Al-Mg-Ca-Layered Double Oxides for Efficient Removal of As(V) from Water: The Role of Amides	2019
9261	P. Sepulveda	As(V) removal capacity of FeCu bimetallic nanoparticles in aqueous solutions: The influence of Cu content and morphologic changes in bimetallic nanoparticles	2018
9262	L. L. Min	Functionalized chitosan electrospun nanofiber for effective removal of trace arsenate from water	2016
9263	O. H. Elshenawy	Modulation of aryl hydrocarbon receptor regulated genes by acute administration of trimethylarsine oxide in the lung, kidney and heart of C57BL/6 mice	2015
9264	B. Bajnoczi	Archaeometric characterization of 17th-century tin-glazed Anabaptist (Hutterite) faience artefacts from North-East-Hungary	2014
9265	D. C. Amaral	A New Approach to Sampling Intact Fe Plaque Reveals Si-Induced Changes in Fe Mineral Composition and Shoot As in Rice	2017
9266	H. Qiu	Cancer stem cells: a potential target for cancer therapy	2015
9267	L. Rubio	Nanoceria acts as antioxidant in tumoral and transformed cells	2018
9268	S. F. AbdulSalam	Self-Cyclizing Antioxidants to Prevent DNA Damage Caused by Hydroxyl Radical	2017

9269	K. N. Kumar	Dielectric features, relaxation dynamics and a.c. conductivity studies on Ag+ mixed lead arsenate glass ceramics	2018
9270	J. C. Madan	Association of Cesarean Delivery and Formula Supplementation With the Intestinal Microbiome of 6-Week-Old Infants	2016
9271	D. T. Huang	Silica sub-microspheres induce autophagy in an endocytosis dependent manner	2017
9272	Y. Xu	Preparation, Stabilization and Applications of Nano-Zero-Valent Iron Composites in Water Treatment	2022
9273	A. Gabor	Behaviour of Silica and Florisil as Solid Supports in the Removal Process of As(V) from Aqueous Solutions	2015
9274	N. Gatcha-Bandjun	WATER TREATMENT WITH Fe-0/H2O SYSTEMS: LEARNING FROM INTERNAL ELECTROLYSIS	2014
9275	X. Chen	Molecular Interaction and Evolution of Jasmonate Signaling With Transport and Detoxification of Heavy Metals and Metalloids in Plants	2021
9276	H. Gomaa	Highly-efficient removal of As-V, Pb2+, Fe3+, and Al3+ pollutants from water using hierarchical, microscopic TiO2 and TiOF2 adsorbents through batch and fixed-bed columnar techniques	2018
9277	P. Colomban	Non-Invasive On-Site Raman Study of Pigments and Glassy Matrix of 17th-18th Century Painted Enamelled Chinese Metal Wares: Comparison with French Enamelling Technology	2020
9278	S. Hafezneza mi	Remediation of groundwater contaminated with arsenic through enhanced natural attenuation: Batch and column studies	2017
9279	Z. H. Cheng	Adsorption, oxidation, and reduction behavior of arsenic in the removal of aqueous As(III) by mesoporous Fe/Al bimetallic particles	2016
9280	A. Courtin-Nomade	Arsenic and lead mobility: From tailing materials to the aqueous compartment	2016
9281	S. Khaska	Innovative isotopic method to evaluate bioaccumulation of As and MTEs in Vitis vinifera	2019
9282	L. Verma	Performance of a novel iron infused biochar developed from Raphanus sativus and Artocarpus heterophyllus refuse for trivalent and pentavalent arsenic adsorption from an aqueous solution: mechanism, isotherm and kinetics study	2022
9283	T. Dordevic	Synergetic Tl and As retention in secondary minerals: An example of extreme arsenic and thallium pollution	2021
9284	R. Mudzielw ana	Evaluation of the adsorptive properties of locally available alumino-silicate clay in As(III) and As(V) remediation from groundwater	2019
9285	J. C. Yang	Moderate wetting and drying increases rice yield and reduces water use, grain arsenic level, and methane emission	2017
9286	X. J. Xie	In situ Fe-sulfide coating for arsenic removal under reducing conditions	2016

9287	J. A. Olimah	Does ochre have the potential to be a remedial treatment for As-contaminated soils?	2015
9288	E. Zuniga-Puelles	Electrical and thermal transport properties of natural and synthetic FeAsxS2-x (x <= 0.01)	2021
9289	M. E. Mahmoud	Rapid and efficient removal of lead from water by alpha-FeOOH/Cellulose/TiO2 nanocomposite	2020
9290	N. Perchiazzi	Crystal-chemical study of ecdemite from Harstigen, a new natural member of the layered lead oxyhalides group	2019
9291	R. I. Gonzalez	Model for Self-Rolling of an Aluminosilicate Sheet into a Single-Walled Imogolite Nanotube	2014
9292	M. Martinez-Eixarch	Multiple environmental benefits of alternate wetting and drying irrigation system with limited yield impact on European rice cultivation: The Ebre Delta case	2021
9293	I. Roque-Alvarez	Spatial distribution, mobility and bioavailability of arsenic, lead, copper and zinc in low polluted forest ecosystem in North-western Mexico	2018
9294	J. Z. Li	GEOCHEMICAL PARTITION OF HEAVY METALS IN AQUATIC SYSTEM AND ECOSYSTEMATIC RISK ASSESSMENT	2020
9295	S. B. Liu	Enhancement of As(V) adsorption from aqueous solution by a magnetic chitosan/biochar composite	2017
9296	S. J. Tae	Immobilization of Cr (VI) in stainless steel slag and Cd, As, and Pb in wastewater using blast furnace slag via a hydrothermal treatment	2017
9297	B. Verbinnen	Adsorption of Oxyanions from Industrial Wastewater using Perlite-Supported Magnetite	2016
9298	J. Gorny	Passive sampler measurements of inorganic arsenic species in environmental waters: A comparison between 3-mercapto-silica, ferrihydrite, Metsorb (R), zinc ferrite, and zirconium dioxide binding gels	2019
9299	F. S. Sosa-Rodriguez	Spatial distribution, mobility and potential health risks of arsenic and lead concentrations in semiarid fine top-soils of Durango City, Mexico	2020
9300	J. B. Huo	Recyclable high-affinity arsenate sorbents based on porous Fe2O3/La2O2CO3 composites derived from Fe-La-C frameworks	2020
9301	J. Z. Beiyuan	Chelant-enhanced washing of CCA-contaminated soil: Coupled with selective dissolution or soil stabilization	2018
9302	S. Purwajanti	Mg(OH)(2)-MgO@reduced graphene oxide nanocomposites: the roles of composition and nanostructure in arsenite sorption	2017
9303	Y. M. Dong	The influence of humic and fulvic acids on polytetrafluoroethylene-adsorbed arsenic: a mechanistic study	2021
9304	M. Ishfaq	Alternate wetting and drying: A water-saving and ecofriendly rice production system	2020

9305	M. A. Saghiri	Functional role of inorganic trace elements in angiogenesis part III: (Ti, Li, Ce, As, Hg, Va, Nb and Pb)	2016
9306	Y. Yang	Arsenic(iii) removal from aqueous solution using TiO2-loaded biochar prepared by waste Chinese traditional medicine dregs	2022
9307	J. Naskar	Recent Advances of Nanotechnology in Mitigating Emerging Pollutants in Water and Wastewater: Status, Challenges, and Opportunities	2022
9308	F. R. Carrillo Pedroza	ADSORPTION OF CHROMIUM FROM STEEL PLATING WASTEWATER USING BLAST FURNACE DUST	2017
9309	D. G. Coelho	Is arsenite more toxic than arsenate in plants?	2020
9310	D. M. Prieto	Influence of epipsammic biofilm on the biogeochemistry of arsenic in freshwater environments	2016
9311	M. A. Inam	Adsorption Capacities of Iron Hydroxide for Arsenate and Arsenite Removal from Water by Chemical Coagulation: Kinetics, Thermodynamics and Equilibrium Studies	2021
9312	A. N. Thomas	Oxidative Weathering Decreases Bioaccessibility of Toxic Metal(loid)s in PM10 Emissions From Sulfide Mine Tailings	2018
9313	P. Abad-Valle	Evaluation of ferrihydrite as amendment to restore an arsenic-polluted mine soil	2015
9314	Y. H. Jiao	Simultaneous stabilization of Sb and As co-contaminated soil by FeMg modified biochar	2022
9315	M. Glodowska	Microbial transformation of biogenic and abiogenic Fe minerals followed by in-situ incubations in an As-contaminated vs. non-contaminated aquifer	2021
9316	A. S. Maghsoudi	Recent Advances in Nanotechnology-Based Biosensors Development for Detection of Arsenic, Lead, Mercury, and Cadmium	2021
9317	S. S. Xin	A full-wave rectified alternating current wireless electrocoagulation strategy for the oxidative remediation of As(III) in simulated anoxic groundwater	2018
9318	R. Lopez-Adams	Elucidating heterogeneous iron biomineralization patterns in a denitrifying As(iii)-oxidizing bacterium: implications for arsenic immobilization	2022
9319	B. B. Shen	The optimum pH and Eh for simultaneously minimizing bioavailable cadmium and arsenic contents in soils under the organic fertilizer application	2020
9320	C. Ash	Different low-molecular-mass organic acids specifically control leaching of arsenic and lead from contaminated soil	2016
9321	D. Alderton	DISTRIBUTION AND MOBILITY OF ARSENIC AND ANTIMONY AT MINE SITES IN FYR MACEDONIA	2014
9322	S. Khazaei	Environmental and ecological factors of stomach cancer incidence and mortality: a systematic review study on ecological studies	2020

9323	B. W. Cave	Geology, Apatite Geochronology, and Geochemistry of the Ernest Henry Inter-lens: Implications for a Re-Examined Deposit Model	2018
9324	K. F. Pi	Geochemical effects of dissolved organic matter biodegradation on arsenic transport in groundwater systems	2015
9325	J. M. Peralta	Unraveling the impact of arsenic on the redox response of peanut plants inoculated with two different Bradyrhizobium sp. strains	2020
9326	I. Labastida	Kinetic approach for the appropriate selection of indigenous limestones for acid mine drainage treatment with passive systems	2019
9327	X. W. Xu	Microbial sulfate reduction decreases arsenic mobilization in flooded paddy soils with high potential for microbial Fe reduction	2019
9328	Y. J. Jung	Arsenic removal properties by electrolyzed and calcined manganese dioxide	2020
9329	T. C. Egbosiuba	Enhanced adsorption of As(V) and Mn(VII) from industrial wastewater using multi-walled carbon nanotubes and carboxylated multi-walled carbon nanotubes	2020
9330	C. A. Tamanaha-Vegas	Surface magnetic properties of a ternary nanocomposite and its ecotoxicological properties in Daphnia magna	2022
9331	Y. Y. Miao	Antimony(V) removal from water by hydrated ferric oxides supported by calcite sand and polymeric anion exchanger	2014
9332	S. E. Panchu	Enriching Trace Level Adsorption Affinity of As3+ Ion Using Hydrothermally Synthesized Iron-Doped Hydroxyapatite Nanorods	2022
9333	P. Y. Ji	Arsenic and sulfur dioxide co-exposure induce renal injury via activation of the NF-kappa B and caspase signaling pathway	2019
9334	N. H. Kamaludin	Exposure to Silica, Arsenic, and Chromium (VI) in Cement Workers: A Probability Health Risk Assessment	2020
9335	W. Li	Plasma CC16 mediates the associations between urinary metals and fractional exhaled nitric oxide: A cross-sectional study	2020
9336	Y. T. Fernandez	Phytostabilization of arsenic in soils with plants of the genus Atriplex established in situ in the Atacama Desert	2016
9337	L. Ma	Arsenite simultaneous sorption and oxidation by natural ferruginous manganese ores with various ratios of Mn/Fe	2020
9338	A. Biswas	Geochemistry of zinc and cadmium in coal waste rock, Elk Valley, British Columbia, Canada	2022
9339	W. H. Liu	Arsenic in hydrothermal apatite: Oxidation state, mechanism of uptake, and comparison between experiments and nature	2017
9340	A. Francova	Health risk assessment of metal(loid)s in soil and particulate matter from industrialized regions: A multidisciplinary approach	2020

9341	G. H. Moon	Synergistic combination of bandgap-modified carbon nitride and WO3 for visible light-induced oxidation of arsenite accelerated by in-situ Fenton reaction	2017
9342	D. Purushotham	Deciphering Heavy Metal Contamination Zones in Parts of Nalgonda District, Telangana	2017
9343	Z. Michalkova	Stability, transformations and stabilizing potential of an amorphous manganese oxide and its surface-modified form in contaminated soils	2016
9344	A. El-Naggar	Release dynamics of As, Co, and Mo in a biochar treated soil under pre-definite redox conditions	2019
9345	D. C. W. Tsang	Metal distribution and spectroscopic analysis after soil washing with chelating agents and humic substances	2014
9346	A. H. Batista	More aggressive sequential extraction procedure to access stable forms of Pb and As in clay minerals of soils	2017
9347	S. Sahay	Photosynthetic activity and RAPD profile of polyethylene glycol treated B. juncea L. under nitric oxide and abscisic acid application	2020
9348	U. A. Usman	Natural sources of iron and manganese in groundwater of the lower Kelantan River Basin, North-eastern coast of Peninsula Malaysia: water quality assessment and an adsorption-based method for remediation	2021
9349	J. Zukal	Bats as bioindicators of heavy metal pollution: history and prospect	2015
9350	M. L. Witten	Early life inhalation exposure to mine tailings dust affects lung development	2019
9351	F. Gosetti	Monitoring of water quality inflow and outflow of a farm in Italian Padana plain for rice cultivation: a case study of two years	2019
9352	L. C. Herbert	Glacial controls on redox-sensitive trace element cycling in Arctic fjord sediments (Spitsbergen, Svalbard)	2020
9353	X. J. Xie	Insights into paleoenvironment and chemical weathering as controls on arsenic accumulation in Late Pleistocene to Holocene aquifers	2022
9354	S. X. Ye	Fabrication and characterization of KGM-based FMBO-containing aerogels for removal of arsenite in aqueous solution	2015
9355	F. G. Huang	Reductive dissolution of ferrihydrite with the release of As(V) in the presence of dissolved S(-II)	2015
9356	S. G. Gu	Nano-biochar modulates the formation of iron plaque through facilitating iron-involved redox reactions on aquatic plant root surfaces	2022
9357	X. L. Zhang	Simultaneous Oxidation and Sequestration of As(III) from Water by Using Redox Polymer-Based Fe(III) Oxide Nanocomposite	2017
9358	V. V. Dev	Pushing the boundaries of heavy metal adsorption: A commentary on strategies to improve adsorption efficiency and modulate process mechanisms	2022

9359	K. X. Li	Novel in situ method based on diffusive gradients in thin-films with lanthanum oxide nanoparticles for measuring As, Sb, and V and in waters	2020
9360	G. K. Sarma	Nanomaterials as versatile adsorbents for heavy metal ions in water: a review	2019
9361	R. Z. He	Synthesis and characterization of an iron-impregnated biochar for aqueous arsenic removal	2018
9362	Q. Ramos	Evaluation of the interactions of arsenic (As), boron (B), and lead (Pb) from geothermal production wells with agricultural soils	2021
9363	C. B. Miller	Lake-specific controls on the long-term stability of mining-related, legacy arsenic contamination and geochemical baselines in a changing northern environment, Tundra Mine, Northwest Territories, Canada	2019
9364	A. A. Abdelhafez	Feasibility of biochar manufactured from organic wastes on the stabilization of heavy metals in a metal smelter contaminated soil	2014
9365	A. Kumar	Regulation of groundwater arsenic concentrations in the Ravi, Beas, and Sutlej floodplains of Punjab, India	2020
9366	Z. L. Yang	Re-activation of aged-ZVI by iron-reducing bacterium Shewanella putrefaciens for enhanced reductive dechlorination of trichloroethylene	2017
9367	M. Channegowda	Recent advances in environmentally benign hierarchical inorganic nano-adsorbents for the removal of poisonous metal ions in water: a review with mechanistic insight into toxicity and adsorption	2020
9368	C. F. Chen	A novel and sensitive electrochemical sensor based on nanoporous gold for determination of As(III)	2020
9369	M. S. Othman	The potential effect of berberine in mercury-induced hepatorenal toxicity in albino rats	2014
9370	R. Marques	Chemistry of volcanic soils used for agriculture in Brava Island (Cape Verde) envisaging a sustainable management	2018
9371	M. D. Luu	A new perovskite-type NdFeO3 adsorbent: synthesis, characterization, and As(V) adsorption	2016
9372	J. L. Cui	Speciation and leaching of trace metal contaminants from e-waste contaminated soils	2017
9373	S. Venkateswarlu	Bioinspired 2D-Carbon Flakes and Fe3O4 Nanoparticles Composite for Arsenite Removal	2016
9374	R. Shabnam	Novel Magnetically Doped Epoxide Functional Cross-linked Hydrophobic Poly(lauryl methacrylate) Composite Polymer Particles for Removal of As(III) from Aqueous Solution	2017
9375	Y. R. Peng	Zirconium hydroxide nanoparticle encapsulated magnetic biochar composite derived from rice residue: Application for As(III) and As(V) polluted water purification	2022
9376	F. Ogata	Po-4(3-) adsorption in a complex solution by nickel-cobalt hydroxide, and its cytotoxicity on bovine aortic endothelial cells	2019

9377	K. Z. Benis	Electrochemically modified adsorbents for treatment of aqueous arsenic: Pore diffusion in modified biomass vs. biochar	2021
9378	B. Xu	The development of an environmentally friendly leaching process of a high C, As and Sb bearing sulfide gold concentrate	2016
9379	W. Xu	Arsenic trioxide decreases the amount and inhibits the function of regulatory T cells, which may contribute to its efficacy in the treatment of acute promyelocytic leukemia	2018
9380	L. R. Silva	Human eosinophils are direct targets to nanoparticles: Zinc oxide nanoparticles (ZnO) delay apoptosis and increase the production of the pro-inflammatory cytokines IL-1 beta and IL-8	2016
9381	T. N. Wang	Facile co-removal of As(V) and Sb(V) from aqueous solution using Fe-Cu binary oxides: Structural modification and self-driven force field of copper oxides	2022
9382	K. Hockmann	Antimony mobility in sulfidic systems: Coupling with sulfide-induced iron oxide transformations	2020
9383	H. Y. Deng	Evolution of Raman spectra in n-InAs wafer with annealing temperature	2014
9384	B. H. Choi	PTEN is a negative regulator of mitotic checkpoint complex during the cell cycle	2017
9385	L. Y. Wang	Recent advances in the development of electrochemical aptasensors for detection of heavy metals in food	2020
9386	L. L. Geng	Aerobic oxidative coupling of alcohols and amines to imines over iron catalysts supported on mesoporous carbon	2016
9387	G. P. Nortje	Factors That Determine the Sorption of Mineral Elements in Soils and Their Impact on Soil and Water Pollution	2021
9388	P. I. Angulo-Bejarano	Metal and Metalloid Toxicity in Plants: An Overview on Molecular Aspects	2021
9389	S. R. Al Harbi	Au/As2Se3/Ag/As2Se3/Yb Schottky Barriers Designed as Multifunctional Devices	2020
9390	B. Jiang	Energy-Efficient Electrochemical Strategy for the Oxidative Sequestration of As(III) in Synthesized Anoxic Groundwater	2018
9391	J. H. Park	Mobility of multiple heavy metalloids in contaminated soil under various redox conditions: Effects of iron sulfide presence and phosphate competition	2018
9392	X. T. Chu	Possible Monitoring and Removal of As(III) by an Integrated System of Electrochemical Sensor and Nanocomposite Materials	2018
9393	G. Kwon	Beneficial use of Fe-impregnated bentonite as a catalyst for pyrolysis of grass cut into syngas, bio-oil and biochar	2022
9394	K. Marschner	Demethylation of Methylated Arsenic Species during Generation of Arsanes with Tetrahydridoborate(1-) in Acidic Media	2016

9395	S. J. Tesh	Nano-Composites for Water Remediation: A Review	2014
9396	J. Li	Characteristics of arsenate removal from water by metal-organic frameworks (MOFs)	2014
9397	N. G. Domenech	Recent progress and future prospects in development of advanced materials for nanofiltration	2020
9398	C. C. Fu	Schottky Barrier Height Tuning via the Dopant Segregation Technique through Low-Temperature Microwave Annealing	2016
9399	W. A. Suk	Benefits of basic research from the Superfund Research Program	2020
9400	I. Ozdemir	Effect of point defects on electronic and magnetic properties of single-layer SiO	2019
9401	S. Kalia	Magnetic polymer nanocomposites for environmental and biomedical applications	2014
9402	S. K. Ray	Fano Resonance in Plasmonic Crystals Enables High-Sensitive Arsenite Detection	
9403	R. Khatik	Integrin alpha(v)beta(3) Receptor Overexpressing on Tumor-Targeted Positive MRI-Guided Chemotherapy	2020
9404	W. W. Tang	Impact of humic/fulvic acid on the removal of heavy metals from aqueous solutions using nanomaterials: A review	2014
9405	M. Navarrete-Magana	Improved photocatalytic oxidation of arsenic (III) with WO3/TiO2 nanomaterials synthesized by the sol-gel method	2021
9406	P. P. Gao	Fabrication, performance and mechanism of MgO meso-/macroporous nanostructures for simultaneous removal of As(III) and F in a groundwater system	2016
9407	A. Romero	A review of metal-catalyzed molecular damage: protection by melatonin	2014
9408	J. Gomez-Pastora	Recent progress and future challenges on the use of high performance magnetic nano-adsorbents in environmental applications	2014
9409	R. Mikutta	Properties and reactivity of Fe-organic matter associations formed by coprecipitation versus adsorption: Clues from arsenate batch adsorption	2014
9410	A. Samanta	Exploring beta-FeOOH Nanorods as an Efficient Adsorbent for Arsenic and Organic Dyes	2018
9411	M. A. Amrani	Phoenix dactylifera mediated green synthesis of Cu2O particles for arsenite uptake from water	2016
9412	H. Wang	Low-Temperature As-Doped In2O3 Nanowires for Room Temperature NO2 Gas Sensing	2022

9413	I. Ali	Water Treatment by Adsorption Columns: Evaluation at Ground Level	2014
9414	N. Li	Removal of Cr(VI) ions from wastewater using nanosized ferric oxyhydroxide loaded anion exchanger on a fixedbed column	2014
9415	P. Cao	Differences of cyanide leaching between calcine and dust from refractory gold concentrates	2020
9416	S. Sultan	Calix 4 arene Derivative-Modified Glassy Carbon Electrode: A New Sensing Platform for Rapid, Simultaneous, and Picomolar Detection of Zn(II), Pb(II), As(III), and Hg(II)	2019
9417	J. Mares	Environmental Protection of Port Logistics Using Phase Analysis	2019
9418	H. E. Reynel-avila	Engineered Magnetic Carbon-Based Adsorbents for the Removal of Water Priority Pollutants: An Overview	2021
9419	M. H. Zahir	Efficient Capture of Heavy Metal Ions and Arsenic with a CaY-Carbonate Layered Double-Hydroxide Nanosheet	2021
9420	S. Wiegand	Malignant Vascular Tumors of the Head and Neck-Which Type of Therapy Works Best?	2021
9421	C. Y. Liu	The effect of iron plaque on lead translocation in soil-Carex cinerascens kukenth. system	2016
9422	F. Liu	Simultaneous photocatalytic redox removal of chromium(vi) and arsenic(iii) by hydrothermal carbon-sphere@nano-Fe3O4	2019
9423	K. Nohara	Augmenting effects of gestational arsenite exposure of C3H mice on the hepatic tumors of the F2 male offspring via the F1 male offspring	2016
9424	M. D'Orazio	Thallium-rich pyrite ores from the Apuan Alps, Tuscany, Italy:constraints for their origin and environmental concerns	2017
9425	M. Naddaf	Comparative study of structural and visible luminescence properties of AZO thin film deposited on GaAs and porous GaAs substrates	2015
9426	F. Fufa	Sorptive removal of arsenate using termite mound	2014
9427	M. Peng	PCN-224/rGO nanocomposite based photoelectrochemical sensor with intrinsic recognition ability for efficient p-arsanilic acid detection	2019
9428	J. A. Bhat	Defense interplay of the zinc-oxide nanoparticles and melatonin in alleviating the arsenic stress in soybean (Glycine max L.)	2022
9429	E. Deschamps	Characterization of indoor dust from Brazil and evaluation of the cytotoxicity in A549 lung cells	2014
9430	K. Cho	Effect of Soil Washing Solutions on Simultaneous Removal of Heavy Metals and Arsenic from Contaminated Soil	2020

9431	S. Thangavel	Determination of traces of As, B, Bi, Ga, Ge, P, Pb, Sb, Se, Si and Te in high-purity nickel using inductively coupled plasma-optical emission spectrometry (ICP-OES)	2015
9432	C. Zanotti	Linking local natural background levels in groundwater to their generating hydrogeochemical processes in Quaternary alluvial aquifers	2022
9433	F. M. Romero	The role of cassiterite controlling arsenic mobility in an abandoned stanniferous tailings impoundment at Llallagua, Bolivia	2014
9434	D. P. Gautam	EVALUATION OF CALCIUM ALGINATE ENTRAPPED NANO ZINC OXIDE TO REDUCE GASEOUS EMISSIONS FROM LIQUID DAIRY MANURE	2016
9435	F. Liang	Study on the Arsenate Removal from Raw As(V)-Rich Wastewater Using Zero-Valent Iron	2022
9436	R. P. Parajuli	Impact of caste on the neurodevelopment of young children from birth to 36 months of age: a birth cohort study in Chitwan Valley, Nepal	2014
9437	A. M. Ahmed	Environmental Concentrations of Copper, Alone or in Mixture With Arsenic, Can Impact River Sediment Microbial Community Structure and Functions	2018
9438	S. M. Owings	Development of a rate law for arsenite oxidation by manganese oxides	2019
9439	L. Zheng	Settleability and characteristics of ferrate(VI)-induced particles in advanced wastewater treatment	2016
9440	M. Bhatia	Application of nanoadsorbents for removal of lead from water	2017
9441	M. Verma	Adsorptive removal of Pb (II) ions from aqueous solution using CuO nanoparticles synthesized by sputtering method	2017
9442	S. S. Wagh	Phloretin-induced suppression of oxidative and nitrosative stress attenuates doxorubicin-induced cardiotoxicity in rats	2022
9443	G. M. Whyte	Experimental and theoretical studies of the solid-state performance of electrodeposited Yb2O3/As2Se3 nanocomposite films	2021
9444	M. H. Siddiqui	Melatonin and calcium function synergistically to promote the resilience through ROS metabolism under arsenic-induced stress	2020
9445	S. Mancillas-Salas	Impact of secondary salts, temperature, and pH on the colloidal stability of graphene oxide in water	2022
9446	J. Scheplitz	Optimization and assessment of a sequential extraction procedure for calcium carbonate rocks	2021
9447	Y. Wang	CeO2/ZrO2-based Diffusive Gradients in Thin Films Technique for Measurement of As(III) and As(V) in Water and Sediment	2018
9448	M. B. Galkaduwa	Iron Oxides Minimize Arsenic Mobility in Soil Material Saturated with Saline Wastewater	2018

9449	Z. Souri	Improved physiological defense responses by application of sodium nitroprusside in Isatis cappadocica Desv. under cadmium stress	2021
9450	L. L. Hao	Removal of As(III) and As(V) from water using iron doped amino functionalized sawdust: Characterization, adsorptive performance and UF membrane separation	2016
9451	I. M. Varentsov	Rare Elements-Markers of the Formation Setting of Manganese and Iron Ores in the Kalahari and Postmasburg Manganese Fields (South Africa): Communication 2. Postmasburg Iron and Manganese Field	2019
9452	P. Khamdahsag	Continuous fixed-bed column studies of arsenite removal via oxidation and adsorption coprocesses	2021
9453	Z. Y. Shen	Anchoring Al- and/or Mg-oxides to magnetic biochars for Co-uptake of arsenate and fluoride from water	2021
9454	A. K. Shukla	Removal of heavy metal ions using a carboxylated graphene oxide-incorporated polyphenylsulfone nanofiltration membrane	2018
9455	M. Aazami	The effect of solution parameters on the thiosulfate leaching of Zarshouran refractory gold ore	2014
9456	T. Chen	Highly efficient As(III) removal in water using millimeter-sized porous granular MgO-biochar with high adsorption capacity	2021
9457	C. Wu	Defective magnesium ferrite nano-platelets for the adsorption of As(V): The role of surface hydroxyl groups	2018
9458	M. Simmler	Reductive solubilization of arsenic in a mining-impacted river floodplain: Influence of soil properties and temperature	2017
9459	G. Q. Zhang	Fate of adsorbed arsenic during early stage sulfidization of nano-ferrihydrite	2019
9460	N. Khare	Efficient graphene-coated iron oxide (GCIO) nanoadsorbent for removal of lead and arsenic ions	2021
9461	L. P. Liu	Enhanced Arsenate Removal from Water by Novel Magnetic Fe-Mn-Y Tri-Metal Composite	2020
9462	H. W. Wang	Differences in Sb(V) and As(V) adsorption onto a poorly crystalline phyllomanganate (delta-MnO2): Adsorption kinetics, isotherms, and mechanisms	2018
9463	Y. Kim	Study of detailed geochemistry of hazardous elements in weathered coal ashes	2017
9464	S. B. Gautam	Adsorptive Removal of As(III) from Aqueous Solution by Raw Coconut Husk and Iron Impregnated Coconut Husk: Kinetics and Equilibrium Analyses	2017
9465	X. M. Zhao	The fate of co-existent cadmium and arsenic during Fe(II)-induced transformation of As(V)/Cd(II)-bearing ferrihydrite	2022
9466	C. H. Gu	X-ray Absorption Spectroscopic Quantification and Speciation Modeling of Sulfate Adsorption on Ferrihydrite Surfaces	2016

9467	T. T. Yue	Feasibility of cleaning As- and U-containing soil of a gold mine by using biologically nano-sized FeS: Implications for soil remediation technology	2021
9468	P. Ni	Aqueous geochemistry and its influence on the partitioning of arsenic between aquifer sediments and groundwater: a case study in the northwest of the Hetao Basin	2016
9469	R. F. Melo	Behavior of Eucalyptus urophylla and Eucalyptus citriodora Seedlings Grown in Soil Contaminated by Arsenate	2016
9470	Y. H. Wang	TiO2 pillared montmorillonite in-situ growth of CeOx/MnOy nanoparticles for effective arsenic (III) adsorption in wastewater	2020
9471	G. Kalyvas	Topsoil pollution as ecological footprint of historical mining activities in Greece	2018
9472	H. Sharifan	Investigation on the Modification of Physicochemical Properties of Cerium Oxide Nanoparticles through Adsorption of Cd and As(III)/As(V)	2018
9473	I. Q. Lima	Hydrogeochemical contrasts in the shallow aquifer systems of the Lower Katari Basin and Southern Poopo Basin, Bolivian Altiplano	2021
9474	F. Coppola	Remediation of arsenic from contaminated seawater using manganese spinel ferrite nanoparticles: Ecotoxicological evaluation in Mytilus galloprovincialis	2019
9475	H. Neidhardt	Biogeochemical phosphorus cycling in groundwater ecosystems - Insights from South and Southeast Asian floodplain and delta aquifers	2018
9476	X. J. Guo	Simple combination of oxidants with zero-valent-iron (ZVI) achieved very rapid and highly efficient removal of heavy metals from water	2016
9477	B. Li	Influence of straw-derived humic acid-like substance on the availability of Cd/As in paddy soil and their accumulation in rice grain	2022
9478	Y. Y. An	Hollow structured copper-loaded self-floating catalyst in sulfite-induced oxidation of arsenic(III) at neutral pH: Kinetics and mechanisms investigation	2021
9479	H. M. Guo	Contrasting distributions of groundwater arsenic and uranium in the western Hetao basin, Inner Mongolia: Implication for origins and fate controls	2016
9480	T. Igarashi	The two-step neutralization ferrite-formation process for sustainable acid mine drainage treatment: Removal of copper, zinc and arsenic, and the influence of coexisting ions on ferritization	2020
9481	S. M. Prabhu	Synthesis of modulator- driven highly stable zirconium- fumarate frameworks and mechanistic investigations of their arsenite and arsenate adsorption from aqueous solutions plus Phi	2019
9482	X. J. Xie	In situ arsenic immobilization by chemical oxidation: Field trial and reactive transport modelling	2020
9483	B. Courchesn e	MACRO- TO NANOSCALE MINERAL RELATIONSHIPS IN SURFICIAL COBALT-ARSENIC-BEARING MINE TAILINGS OF THE COBALT MINING CAMP, NORTHEASTERN ONTARIO, CANADA	2022
9484	E. Hiller	Contaminated soils of different natural pH and industrial origin: The role of (nano) iron- and manganese-based amendments in As, Sb, Pb, and Zn leachability	2021

9485	A. A. Kadir	Leachate Characteristic of Mosaic Sludge Brick	2016
9486	T. Y. Xu	Response of interfacial water to arsenate adsorption on corundum (001) surfaces: Effects of pH and adsorbate surface coverage	2018
9487	J. Markovski	An experimental approach for determining thermodynamic surface complexation descriptors of weak-acid oxyanions onto metal (hydr) oxides: Case study of arsenic and titanium dioxide	2019
9488	G. F. Wei	Study on the Slags from the Sites of Mining and Smelting in Tongling and Nanling Region	2019
9489	N. P. Dubey	Chronic study of arsenic trioxide-induced hepatotoxicity in relation to arsenic liver accumulation in rats	2014
9490	Y. H. Xi	Kinetics of Arsenic Removal in Waste Acid by the Combination of CuSO4 and Zero-Valent Iron	2019
9491	Y. Agrawal	F-box protein FBXO41 plays vital role in arsenic trioxide-mediated autophagic death of cancer cells	2022
9492	A. A. Kusnierz	Thermal analysis of the glass batch with a higher content of raw materials substitution based on selected fining agents	2017
9493	S. K. Ahmmad	Modification of the optical spectra of ZnF2-As2O3-TeO2 glass by nitrogen ion implantation	2015
9494	J. Ablain	Retinoic acid signaling in cancer: The parable of acute promyelocytic leukemia	2014
9495	Z. Vankova	Soil moisture influences performance of selected stabilizing amendments in soil remediation	2021
9496	S. M. Chernonozhkin	Evaluation of rammelsbergite (NiAs2) as a novel mineral for Re-187-Os-187 dating and implications for unconformity-related U deposits	2020
9497	S. Ye	A Highly Selective and Sensitive Chemiluminescent Probe for Real-Time Monitoring of Hydrogen Peroxide in Cells and Animals	2020
9498	S. Zdraljevic	Natural variation in C-elegans arsenic toxicity is explained by differences in branched chain amino acid metabolism	2019
9499	M. X. Su	Acute toxicity of intratracheal arsenic trioxide instillation in rat lungs	2019
9500	J. I. Garza-Arevalo	Fe doped TiO2 photocatalyst for the removal of As(III) under visible radiation and its potential application on the treatment of As-contaminated groundwater	2016
9501	M. Baghayeri	Magnetic MWCNTs-dendrimer: A potential modifier for electrochemical evaluation of As (III) ions in real water samples	2021
9502	Y. Feng	The dual role of oxygen in redox-mediated removal of aqueous arsenic(III/V) by Fe-modified biochar	2021

9503	Q. Wang	Arsenate phytotoxicity regulation by humic acid and related metabolic mechanisms	2021
9504	H. Wang	A Comparison Study on the Arsenate Adsorption Behavior of Calcium-Bearing Materials	2019
9505	S. Dubey	Application of common nano-materials for removal of selected metallic species from water and wastewaters: A critical review	2017
9506	Z. B. A. Zhai	Reduction Smelting Experiment of Copper Dross with Low Copper Content	2021
9507	F. Coppola	Can water remediated by manganese spinel ferrite nanoparticles be safe for marine bivalves?	2020
9508	L. Ye	Sulfate-Reducing Bacteria Mobilize Adsorbed Antimonate by Thioantimonate Formation	2019
9509	D. Cortes-Arriagada	Improving As(III) adsorption on graphene based surfaces: impact of chemical doping	2015
9510	J. M. Luo	Critical Review of Advances in Engineering Nanomaterial Adsorbents for Metal Removal and Recovery from Water: Mechanism Identification and Engineering Design	2021
9511	F. Medina-Estevez	Association between Heavy Metals and Rare Earth Elements with Acute Ischemic Stroke: A Case-Control Study Conducted in the Canary Islands (Spain)	2020
9512	Y. Wang	Removal of Trace Arsenite through Simultaneous Photocatalytic Oxidation and Adsorption by Magnetic Fe3O4@PpPDA@TiO2 Core-Shell Nanoparticles	2020
9513	H. W. Wang	Adsorption capacities of poorly crystalline Fe minerals for antimonate and arsenate removal from water: adsorption properties and effects of environmental and chemical conditions	2018
9514	A. Fakhri	Assessment of Ethidium bromide and Ethidium monoazide bromide removal from aqueous matrices by adsorption on cupric oxide nanoparticles	2014
9515	T. T. Nguyen	Removal of Heavy Metals from Tailing in Citrate Solution with Ferric Chloride	2018
9516	R. J. Chen	Autophagy-inducing effect of pterostilbene: A prospective therapeutic/preventive option for skin diseases	2017
9517	J. P. Zou	Hydrogen Evolution from Water Coupled with the Oxidation of As(III) in a Photocatalytic System	2015
9518	Q. F. Tian	Furfural-Mediated Synthesis of Mesoporous Ti0.5Sn0.5O2 Solid-Solution Microspheres for Effective Photocatalytic Removal of As(III)	2018
9519	B. Bansod	A review on various electrochemical techniques for heavy metal ions detection with different sensing platforms	2017
9520	X. S. Tian	Simultaneous adsorption of As(III) and Cd(II) by ferrihydrite-modified biochar in aqueous solution and their mutual effects	2022

9521	D. Vries	Impact of ionic composition of groundwater on oxidative iron precipitation	2022
9522	J. Cervini-Silva	One-nanometre-resolution evidence of As(III) anoxic and oxic transformations on the surfaces of expandable clay minerals	
9523	M. Vithanage	Interaction of arsenic with biochar in soil and water: A critical review	2017
9524	S. Y. Cao	Insights into the facet-dependent adsorption of phenylarsonic acid on hematite nanocrystals	2019
9525	Z. Markova	Iron(II,III)-Polyphenol Complex Nanoparticles Derived from Green Tea with Remarkable Ecotoxicological Impact	2014
9526	F. D. M. Camara	Yerba mate (Ilex paraguariensis St. Hil.) extract inhibits hand-rolled cornhusk cigarette smoke-induced oxidative pulmonary damage	2020
9527	H. W. Yan	Electrochemical Sensing of Heavy Metal Ions based on Monodisperse Single-crystal Fe3O4 Microspheres	2018
9528	W. Y. Li	A Novel Hollow Carbon@MnO2 Electrospun Nanofiber Adsorbent for Efficient Removal of Pb2+ in Wastewater	2021
9529	S. S. Li	Surface Fe(II)/Fe(III) Cycle Promoted Ultra-Highly Sensitive Electrochemical Sensing of Arsenic(III) with Dumbbell-Like Au/Fe3O4 Nanoparticles	2018
9530	M. Duda	Non-invasive surface analysis of ancient bronze arrowheads with scanning electron microscopy and X-ray powder diffractometry	2022
9531	H. Chen	Arsenic(III) Removal by Nanostructured Dialdehyde Cellulose-Cysteine Microscale and Nanoscale Fibers	2019
9532	U. K. Sahu	Synthesis and characterization of an eco-friendly composite of jute fiber and Fe2O3 nanoparticles and its application as an adsorbent for removal of As(V) from water	2017
9533	T. T. Pham	Removal of As (V) from the aqueous solution by a modified granular ferric hydroxide adsorbent	2020
9534	J. H. Liu	Removal of arsenic(v) from aqueous solutions using sulfur-doped Fe3O4 nanoparticles	2018
9535	P. A. Alekseev	Control of Conductivity of In(x)G(a1-x)As Nanowires by Applied Tension and Surface States	2019
9536	K. Bedu-Addo	The AKOBEN programme as a tool towards responsible gold mining in Ghana, business as usual or a commitment towards sustainable development	2019
9537	N. A. Vafeas	Mineralogical characterization of the thrusted manganese ore above the Blackridge Thrust Fault, Kalahari Manganese Field: The footprint of the Mukulu Enrichment	2019
9538	H. Singh	Auxin metabolic network regulates the plant response to metalloids stress	2021

9539	T. Y. Liu	Highly efficient and rapid removal of arsenic(iii) from aqueous solutions by nanoscale zero-valent iron supported on a zirconium 1,4-dicarboxybenzene metal-organic framework (UiO-66 MOF)	2019
9540	S. Sharma	Lossy Mode Resonance-Based Fiber Optic Sensor for the Detection of As (III) Using alpha-Fe2O3/SnO2 Core-Shell Nanostructures	2018
9541	M. Bhaumik	Composite nanofibers prepared from metallic iron nanoparticles and polyaniline: High performance for water treatment applications	2014
9542	R. Watanabe	Ataxia telangiectasia and rad3 related (ATR)-promyelocytic leukemia protein (PML) pahway of the DNA damage response in the brain of rats administered arsenic trioxide	2017
9543	R. Xin	Active-Learning-Based Generative Design for the Discovery of Wide-Band-Gap Materials	2021
9544	J. X. Wang	Immobilization of mercury and arsenic in a mine tailing from a typical Carlin-type gold mining site in southwestern part of China	2019
9545	M. Glodowska	Role of in Situ Natural Organic Matter in Mobilizing As during Microbial Reduction of Fe-III-Mineral-Bearing Aquifer Sediments from Hanoi (Vietnam)	2020
9546	C. Biagioni	Mercury-arsenic sulfosalts from the Apuan Alps (Tuscany, Italy). III. Aktashite, Cu6Hg3As4S12, and laffittite, AgHgAsS3, from the Monte Arsiccio mine: occurrence and crystal structure	2014
9547	M. Martin	Arsenic fixation and mobilization in the soils of the Ganges and Meghna floodplains. Impact of pedoenvironmental properties	2014
9548	S. Das	Water management impacts on arsenic behavior and rhizosphere bacterial communities and activities in a rice agro-ecosystem	2016
9549	Z. J. Andersen	Newborns health in the Danube Region: Environment, biomonitoring, interventions and economic benefits in a large prospective birth cohort study	2016
9550	J. P. H. Perez	Arsenic species delay structural ordering during green rust sulfate crystallization from ferrihydrite	2021
9551	R. B. Finkelman	Quantification of the modes of occurrence of 42 elements in coal	2018
9552	X. M. Li	Roles of different molecular weights of dissolved organic matter in arsenic enrichment in groundwater: Evidences from ultrafiltration and EEM-PARAFAC	2019
9553	S. Neu	Trace elements bioavailability to Triticum aestivum and Dendrobaena veneta in a multielement-contaminated agricultural soil amended with drinking water treatment residues	2018
9554	M. T. Wright	Assessing the solubility controls on vanadium in groundwater, northeastern San Joaquin Valley, CA	2014
9555	Y. J. Wang	Arsenite removal in a goethite/oxalate system under UVA irradiation: Roles of different reactive species in acidic and neutral conditions	2021
9556	L. N. Pincus	Toward Realizing Multifunctionality: Photoactive and Selective Adsorbents for the Removal of Inorganics in Water Treatment	2019

9557	E. Verhoeven	Nitrification and coupled nitrification-denitrification at shallow depths are responsible for early season N2O emissions under alternate wetting and drying management in an Italian rice paddy system	2018
9558	C. Biagioni	Mercury-arsenic sulfosalts from Apuan Alps (Tuscany, Italy). I. Routhierite, (Cu0.8Ag0.2)Hg2Tl(As1.4Sb0.6)(Sigma=2)S-6, from Monte Arsiccio mine: occurrence and crystal structure	2014
9559	B. Dousova	STABILITY OF IRON IN CLAYS UNDER DIFFERENT LEACHING CONDITIONS	2014
9560	Z. L. Yang	Stability of Fe-As composites formed with As(V) and aged ferrihydrite	2021
9561	H. Li	Sorption of arsenate(V) to naturally occurring secondary iron minerals formed at different conditions: The relationship between sorption behavior and surface structure	2021
9562	M. Fujioka	Dimethylarsinic acid (DMA) enhanced lung carcinogenesis via histone H3K9 modification in a transplacental mouse model	2020
9563	W. Jerzak	Trace elements retention in bottom ashes during coal combustion with hydrated lime additions	2021
9564	A. Ashok	Exposure to As-, Cd-, and Pb-Mixture Induces A beta, Amyloidogenic APP Processing and Cognitive Impairments via Oxidative Stress-Dependent Neuroinflammation in Young Rats	2015
9565	M. F. Dong	Inoculation of Fe/Mn-oxidizing bacteria enhances Fe/Mn plaque formation and reduces Cd and As accumulation in Rice Plant tissues	2016
9566	Y. L. Lin	Improved Arsine Removal Efficiency Over MnOx Supported Molecular Sieves Catalysts via Micro-Oxygen Oxidation	2017
9567	W. C. Ding	Realgar, a traditional Chinese medicine, induces apoptosis of HPV16-positive cervical cells through a HPV16 E7-related pathway	2018
9568	J. Mehlhorn	Carbon dioxide triggered metal(loid) mobilisation in a mofette	2014
9569	L. Jiang	Impacts of ammonium ion on triclinic birnessites towards the transformation of As(III)	2022
9570	A. Voegelin	Solid-phase characterisation of an effective household sand filter for As, Fe and Mn removal from groundwater in Vietnam	2014
9571	R. M. Dhoble	Arsenite removal from drinking water by bark-based magnetic iron oxide particle (BMIOP): a column study	
9572	T. Magrone	Impact of Heavy Metals on Host Cells: Special Focus on Nickel-Mediated Pathologies and Novel Interventional Approaches	2020
9573	S. B. Kim	Influences of surface treatment on In0.53Ga0.47As epitaxial layer grown on silicon substrate using trimethylaluminum	2018
9574	A. Ghosh	Facile synthesis of Sn(II)-MOF using waste PET bottles as an organic precursor and its derivative SnO2 NPs: Role of surface charge reversal in adsorption of toxic ions	2021

9575	G. Boix	MOF-Beads Containing Inorganic Nanoparticles for the Simultaneous Removal of Multiple Heavy Metals from Water	2020
9576	H. M. Guo	A review of high arsenic groundwater in Mainland and Taiwan, China: Distribution, characteristics and geochemical processes	2014
9577	L. Kong	Efficient removal of Pb(II) from water using magnetic Fe3S4/reduced graphene oxide composites	2017
9578	J. W. Lee	Agglomeration-Free Fe3O4 anchored via nitrogen mediation of carbon nanotubes for high-performance arsenic adsorption	2021
9579	N. Chanthapon	Lead removal by a reusable gel cation exchange resin containing nano-scale zero valent iron	2018
9580	N. Khan	Root Iron Plaque on Wetland Plants as a Dynamic Pool of Nutrients and Contaminants	2016
9581	A. I. Nde-Tchoupe	Technologies for Decentralized Fluoride Removal: Testing Metallic Iron-Based Filters	2015
9582	W. Yao	Synthesis of magnetic silica with quaternary ammonium salt and its application for chromium(VI) removal	2015
9583	S. D. Tao	Long-term effect of all-trans retinoic acid and arsenic trioxide sequential maintenance in patients with acute promyelocytic leukemia	2019
9584	R. Eisavi	Fe3O4@SiO2-PMA-Cu magnetic nanoparticles as a novel catalyst for green synthesis of beta-thiol-1,4-disubstituted-1,2,3-triazoles	2022
9585	R. Rashid	A state-of-the-art review on wastewater treatment techniques: the effectiveness of adsorption method	2021
9586	S. Rha	Reactive ceramic pellets incorporated iron for removing As(III), As(V), and Cr(VI) from aqueous solutions	2019
9587	M. Muppidathi	Immobilization of ssDNA on a metal-organic framework derived magnetic porous carbon (MPC) composite as a fluorescent sensing platform for the detection of arsenate ions	2019
9588	D. K. Gaikwad	Gamma ray shielding properties of TeO2-ZnF2-As2O3-Sm2O3 glasses	2018
9589	R. Shabbir	Calcium homeostasis and potential roles to combat environmental stresses in plants	2022
9590	H. Sugita	Effects of Silicic Acid on Leaching Behavior of Arsenic from Spent Calcium-Based Adsorbents with Arsenite	2021
9591	S. Mohanty	Adsorption/photodegradation of crystal violet (basic dye) from aqueous solution by hydrothermally synthesized titanate nanotube (TNT)	2020
9592	D. Kolodynska	Evaluation of iron-based hybrid materials for heavy metal ions removal	2014

9593	M. K. Shahid	Characterization and application of magnetite Particles, synthesized by reverse coprecipitation method in open air from mill scale	2020
9594	W. Xiu	Change of arsenite adsorption mechanism during aging of 2-line ferrihydrite in the absence of oxygen	2018
9595	X. S. Liu	Theoretical investigations on the defect structures and g factors for W5+ in lead bismuth arsenate glasses	2020
9596	J. Mahoney	Development of a Geochemical Model to Predict Surface Water Discharge Compositions in Northern Saskatchewan: Applications of PHREEQC and PhreePlot to Identify Active Geochemical Processes	2021
9597	A. Boonsrang	Factors controlling the release of metals and a metalloid from the tailings of a gold mine in Thailand	2018
9598	A. R. Lopez	Bioaccumulation Dynamics of Arsenate at the Base of Aquatic Food Webs	2016
9599	T. Ahmed	Recent progress on the heavy metals ameliorating potential of engineered nanomaterials in rice paddy: a comprehensive outlook on global food safety with nanotoxicitiy issues	
9600	S. Naz	Nanomaterials as nanocarriers: a critical assessment why these are multi-chore vanquisher in breast cancer treatment	2018
9601	P. X. Pinto	Assessing metal mobilization from industrially lead-contaminated soils located at an urban site	2017
9602	E. P. Kokhdan	A Narrative Review on Therapeutic Potentials of Watercress in Human Disorders	2021
9603	N. Kumar	GABA accretion reduces Lsi-1 and Lsi-2 gene expressions and modulates physiological responses in Oryza sativa to provide tolerance towards arsenic	2017
9604	S. Kalidindi	Aluminum-cerium double-metal impregnated activated carbon: a novel composite for fluoride removal from aqueous solution	2017
9605	R. B. Viana	Exploring the reaction channels between arsine and the hydroxyl radical	2017
9606	P. Jonsta	The Effect of Rare Earth Metals Alloying on the Internal Quality of Industrially Produced Heavy Steel Forgings	2021
9607	M. A. Rahman	New Citrate-Bicarbonate-Ethylenediaminetetraacetate (CBE) Method for Chemical Extraction of Hydrous Iron Oxides from Plant Root Surfaces	2014
9608	X. F. Shi	Zwitterionic glycine modified Fe/Mg-layered double hydroxides for highly selective and efficient removal of oxyanions from polluted water	2020
9609	K. Vaca-Escobar	Modeling the additive effects of Pb(II) and Cu(II) on the competitive attenuation of As(V) through solid precipitation versus adsorption to goethite	2015
9610	S. X. Zhao	Leaching mechanisms of constituents from fly ash under the influence of humic acid	2017

9611	J. Lohwacharin	Effects of Ferrihydrite-Impregnated Powdered Activated Carbon on Phosphate Removal and Biofouling of Ultrafiltration Membrane	2021
9612	Y. S. Ye	Functionalization of chitosan by grafting sulfhydryl groups to intensify the adsorption of arsenite from water	2021
9613	M. Mansor	Application of Single-Particle ICP-MS to Determine the Mass Distribution and Number Concentrations of Environmental Nanoparticles and Colloids	2021
9614	V. Comakli	Purification and characterization of glucose 6-phosphate dehydrogenase enzyme from rainbow trout (Oncorhynchus mykiss) liver and investigation of the effects of some metal ions on enzyme activity	2015
9615	J. Gorny	Development and application of a HPIC-ICP-MS method for the redox arsenic speciation in river sediment pore waters	2015
9616	B. R. Poudel	Effective remediation of arsenate from contaminated water by zirconium modified pomegranate peel as an anion exchanger	2021
9617	M. O. Akharame	Regeneration and reuse of polymeric nanocomposites in wastewater remediation: the future of economic water management	2019
9618	U. P. Azad	Tris(4,4 '-dimethyl-2,2 '-bipyridine)iron(II)-Exchanged Nafion for Arsenite Determination in Water Samples	2014
9619	B. Liu	Highly efficient removal of arsenic(III) from aqueous solution by zeolitic imidazolate frameworks with different morphology	2015
9620	A. C. S. Pimenta	Photodegradation of Si-doped GaAs nanowire	2019
9621	B. D. Bratkeu-K	Determining the optimum Fe-o ratio for sustainable granular Fe-o/sand water filters	2014
9622	S. S. Que	Modeling the Efficiency of the Iron Coprecipitation-Filtration Process for the Removal of Arsenate at Low Initial Concentrations	2016
9623	C. Noel	Gold nanoparticles induce apoptosis, endoplasmic reticulum stress events and cleavage of cytoskeletal proteins in human neutrophils	2016
9624	D. Singh	Transparent conducting Ga-doped ZnO thin films grown by reactive co-sputtering of Zn and GaAs	2014
9625	Y. X. Si	Energy-Efficient Oxidation and Removal of Arsenite from Groundwater Using Air-Cathode Iron Electrocoagulation	2017
9626	X. C. Li	Adsorptive removal of As(III) from aqueous solution by waste litchi pericarps	2016
9627	A. Zubiarrain-Laserna	Review-Graphene-Based Water Quality Sensors	2020
9628	E. Zielony	Deep levels in the MBE ZnO: As/n-GaN diodes - Photoluminescence, electrical properties and deep level transient spectroscopy	2018

9629	H. Lee	As(III) and As(V) removal from the aqueous phase via adsorption onto acid mine drainage sludge (AMDS) alginate beads and goethite alginate beads	2015
9630	A. Q. Dao	Determination of arsenic(III) in water using gold nanorods-modified electrode	2021
9631	J. Ouyang	Cryogenic Exfoliation of 2D Stanene Nanosheets for Cancer Theranostics	2021
9632	L. Yan	Insights into Antimony Adsorption on {001} TiO2: XAFS and DFT Study	2017
9633	Y. Qin	Defects on activated carbon determine the dispersion of active components and thus the simultaneous removal efficiency of SO2, NOx and Hg-0	2021
9634	L. K. Ouyang	Heterostructured MWCNTs@PANI@TiO2 Nanocomposites for Enhanced Adsorption of As(III) from Aqueous Solution: Adsorption and Photocatalytic Oxidation Behaviors	2020
9635	M. J. Santos	Leachability of major and minor elements from soils and sediments of an abandoned coal mining area in Southern Brazil	2015
9636	L. Rani	A critical review on recent developments in MOF adsorbents for the elimination of toxic heavy metals from aqueous solutions	2020
9637	M. Alimohammady	Preparation and characterization of functionalized MWCNTs-COOH with 3-amino-5-phenylpyrazole as an adsorbent and optimization study using central composite design	2019
9638	A. A. B. Christus	Novel, sensitive and selective colorimetric detection of arsenate in aqueous solution by a Fenton-like reaction of Fe3O4 nanoparticles	2018
9639	S. Liu	Cytotoxicity and Efflux Pump Inhibition Induced by Molybdenum Disulfide and Boron Nitride Nanomaterials with Sheetlike Structure	2017
9640	S. L. Iconaru	Magnetite (Fe3O4) nanoparticles as adsorbents for As and Cu removal	2016
9641	S. Aras	Effects of sodium arsenite on the some laboratory signs and therapeutic role of thymoquinone in the rats	2015
9642	F. Pacchierotti	Environmental Impact on DNA Methylation in the Germline: State of the Art and Gaps of Knowledge	2015
9643	Q. Lan	Extended structural materials composed of transition-metal-substituted arsenicniobates and their photocatalytic activity	2015
9644	C. J. Li	Progresses and emerging trends of arsenic research in the past 120 years	2021
9645	H. H. Xu	Influences of Realgar-Indigo naturalis, A Traditional Chinese Medicine Formula, on the Main CYP450 Activities in Rats Using a Cocktail Method	2017
9646	S. R. Walker	The effect of ore roasting on arsenic oxidation state and solid phase speciation in gold mine tailings	2015

9647	Y. L. He	phi-pH diagram of As-N-Na-H2O system for arsenic removal during alkaline pressure oxidation leaching of lead anode slime	2017
9648	N. J. Satheesh	The Role of Intracellular Calcium for the Development and Treatment of Neuroblastoma	2015
9649	K. S. Rao	Influence of Y2O3, Sc2O3 and HfO2 dopants on green emission of Er3+ ions in PbO-Sb2O3 glasses	2017
9650	W. Jiang	Systematic dissection of dysregulated transcription factor-miRNA feed-forward loops across tumor types	2016
9651	I. Shiue	Higher urinary heavy metal, phthalate and arsenic concentrations accounted for 3-19% of the population attributable risk for high blood pressure: US NHANES, 2009-2012	2014
9652	E. A. Antoniou	Aquifer pre-oxidation using permanganate to mitigate water quality deterioration during aquifer storage and recovery	2014
9653	C. Koyama	Densities of liquid lanthanoid sesquioxides measured with the electrostatic levitation furnace in the ISS	2021
9654	C. P. Cheng	Atomic Nature of the Growth Mechanism of Atomic Layer Deposited High-kappa Y2O3 on GaAs(001)-4 x 6 Based on in Situ Synchrotron Radiation Photoelectron Spectroscopy	2018
9655	Y. B. Song	Design, Synthesis and Anticancer Activity of N-3,N-11-Bis(2-hydroxyethyl)-14-aryl-14H-dibenzo a,j xanthenes-3,11-dica rboxamide	2013
9656	H. Hongu	XAFS study of Sb and As in Cretaceous-Tertiary boundary sediments: an index of soiling of the global environment with dust and ashes from impact ejecta falls	2019
9657	M. Rotiroti	Arsenic release and attenuation in a multilayer aquifer in the Po Plain (northern Italy): Reactive transport modeling	2015
9658	S. Grangeon	Cryptomelane formation from nanocrystalline vernadite precursor: a high energy X-ray scattering and transmission electron microscopy perspective on reaction mechanisms	2015
9659	I. Montes-Avila	SPATIAL DISTRIBUTION OF ARSENIC AND LEAD IN STREAM SEDIMENTS IN A MICRO-BASIN WITH ANCIENT MINING ACTIVITIES	2018
9660	M. Kost'al	Measurement of As-75(n,2n) cross section in well-defined spectrum of LR-0 special core	2017
9661	A. L. Mackie	Investigation into the use of cement kiln dust in high density sludge (HDS) treatment of acid mine water	2015
9662	E. Varghese	Anti-Cancer Agents in Proliferation and Cell Death: The Calcium Connection	2019
9663	C. Bureta	Synergistic effect of arsenic trioxide, vismodegib and temozolomide on glioblastoma	2019
9664	N. Orfali	Retinoid receptor signaling and autophagy in acute promyelocytic leukemia	2014

9665	W. H. Peng	Biostabilization of cadmium contaminated sediments using indigenous sulfate reducing bacteria: Efficiency and process	2018
9666	M. A. Inam	Removal of Sb(III) and Sb(V) by Ferric Chloride Coagulation: Implications of Fe Solubility	2018
9667	N. Jordan	Selenium(IV) Uptake by Maghemite (gamma-Fe2O3)	2014
9668	M. C. S. Faria	Arsenic removal from contaminated water by ultrafine delta-FeOOH adsorbents	2014
9669	R. Markovic	Electrorefining Process of the Non-Commercial Copper Anodes	2021
9670	V. Shukla	Metal-induced oxidative stress level in patients with fibromyalgia syndrome and its contribution to the severity of the disease: A correlational study	2021
9671	A. P. M. Udayan	Ultrathin quasi-hexagonal gold nanostructures for sensing arsenic in tap water	2020
9672	S. Liang	One-Step Treatment of Phosphite-Laden Wastewater: A Single Electrochemical Reactor Integrating Superoxide Radical-Induced Oxidation and Electrocoagulation	2019
9673	I. A. Kumar	A facile synthesis of magnetic particles sprayed gelatin embedded hydrotalcite composite for effective phosphate sorption	2018
9674	J. Wu	Fabrication of Core-Shell CMNP@PmPD Nanocomposite for Efficient As(V) Adsorption and Reduction	2017
9675	N. Inchaurrondo	Evaluation of low-cost geo-adsorbents for As(V) removal	2021
9676	R. S. Seisembayev	Extraction of Gold from Refractory Gold-Bearing Ores by Means of Reducing Pyrometallurgical Selection	2020
9677	V. Villacorta	Influences of As(v), Sb(iii), and Hg(ii) ions on the nucleation and growth of akaganeite	2019
9678	M. A. Chen	Development of a novel microfluidic device to study metal geochemistry in situ using X-ray fluorescence microprobe spectroscopy	2021
9679	B. Gankhurel	Comparison of Chemical Speciation of Lead, Arsenic, and Cadmium in Contaminated Soils from a Historical Mining Site: Implications for Different Mobilities of Heavy Metals	2020
9680	S. M. Beladi-Mousavi	2D-Pnictogens: alloy-based anode battery materials with ultrahigh cycling stability	2018
9681	Q. Zeng	Efficient removal of arsenite by a composite of amino modified silica supported MnO2/Fe-Al hydroxide (SNMFA) prepared from biotite	2021
9682	V. Puccia	On the mechanism controlling fluoride concentration in groundwaters of the south of the Province of Buenos Aires, Argentina: adsorption or solubility?	2018

9683	T. Y. Sun	Efficient removal of arsenite through photocatalytic oxidation and adsorption by ZrO2-Fe3O4 magnetic nanoparticles	2017
9684	S. Paikaray	Environmental Stability of Schwertmannite: A Review	2021
9685	U. Yenial	Recovery of nanoferrites from metal bearing wastes: Synthesis, characterization and adsorption study	2020
9686	N. Nitta	High-Capacity Anode Materials for Lithium- Ion Batteries: Choice of Elements and Structures for Active Particles	2014
9687	K. Zhao	Behavior and mechanism of arsenate adsorption on activated natural siderite: evidences from FTIR and XANES analysis	2014
9688	M. F. Keim	Weathering of Bi-bearing tennantite	2018
9689	M. M. Iqbal	Nanocomposites of sedimentary material with ZnO and magnetite for the effective sequestration of arsenic from aqueous systems: Reusability, modeling and kinetics	2021
9690	A. K. Prajapati	Hazardous As(III) removal using nanoporous activated carbon of waste garlic stem as adsorbent: Kinetic and mass transfer mechanisms	2019
9691	W. F. Wang	Germanium diffusion with vapor-phase GeAs and oxygen co-incorporation in GaAs	2018
9692	S. H. Lee	Enhanced Arsenate Removal Performance in Aqueous Solution by Yttrium-Based Adsorbents	2015
9693	C. Y. Han	Characterizations and mechanisms for synthesis of chitosan-coated Na-X zeolite from fly ash and As(V) adsorption study	2019
9694	M. Naddaf	Formation of superhydrophobic porous GaAs layer: effect of substrate doping type	2022
9695	D. W. Cho	Zirconia-Assisted Pyrolysis of Coffee Waste in CO2 Environment for the Simultaneous Production of Fuel Gas and Composite Adsorbent	2020
9696	T. Raychoudhury	Performance evaluation of metal impregnated activated carbon composite for removal of fluoride under varying solution chemistry	2017
9697	F. Yang	Corn straw-derived biochar impregnated with alpha-FeOOH nanorods for highly effective copper removal	2018
9698	T. Yue	Magnetic Separation and Recycling of Goethite and Calcium Sulfate in Zinc Hydrometallurgy in the Presence of Maghemite Fine Particles	2018
9699	M. El Adnani	Tailings Weathering and Arsenic Mobility at the Abandoned Zgounder Silver Mine, Morocco	2016
9700	B. D. Btatkeu-K	Designing metallic iron based water filters: Light from methylene blue discoloration	2016

9701	D. C. Ong	Manganese and iron recovery from groundwater treatment sludge by reductive acid leaching and hydroxide precipitation	2018
9702	S. Kansara	Theoretical Investigation of Metallic Nanolayers For Charge-Storage Applications	2018
9703	P. Bahmani	Application of modified electrospun nanofiber membranes with alpha-Fe2O3 nanoparticles in arsenate removal from aqueous media	2019
9704	J. Jamieson	Identifying and Quantifying the Intermediate Processes during Nitrate-Dependent Iron(II) Oxidation	2018
9705	A. Pedescoll	Design parameters affecting metals removal in horizontal constructed wetlands for domestic wastewater treatment	2015
9706	J. Singhal	The physio-chemical properties and applications of 2D nanomaterials in agricultural and environmental sustainability	2022
9707	V. K. Sharma	Ferrates: Greener Oxidants with Multimodal Action in Water Treatment Technologies	2015
9708	M. A. Amrani	Low-Cost Goethite Nanorods for As (III) and Se (VI) Removal from Water	2020
9709	D. Cao	Mn assisted electrochemical generation of two-dimensional Fe-Mn layered double hydroxides for efficient Sb(V) removal	2017
9710	X. H. Huang	Surface passivation of GaAs (001) by Hg2Cl2 nanoplates combined with hexadecanethiol	2019
9711	J. J. Huang	Induction treatments for acute promyelocytic leukemia: a network meta-analysis	2016
9712	M. A. Rahman	Antimonate sequestration from aqueous solution using zirconium, iron and zirconium-iron modified biochars	2021
9713	L. F. Ma	Novel Molecular Targets for Diagnosis and Treatment of Hepatocellular Carcinoma	2015
9714	D. Ramutshatsha	Simultaneous removal of Na, Ca, K and Mg from synthetic brine and seawater using Fe2O3-SiO2 mixed oxide nanostructures: kinetics and isotherms studies	2018
9715	J. H. Yang	Mechanism of metal sulfides accelerating the REDOX cycle of Fe(II)/Fe(III) and promoting the degradation of As(III) by oxalate	2022
9716	R. Eisavi	Preparation, characterization and application of MgFe2O4/Cu nanocomposite as a new magnetic catalyst for one-pot regioselective synthesis of beta-thiol-1,4-disubstituted-1,2,3-triazoles	2021
9717	S. H. Zhu	Simultaneous removal of Cd(II) and As(III) from co-contaminated aqueous solution by alpha-FeOOH modified biochar	2020
9718	M. S. Podder	Predictive approach for simultaneous biosorption and bioaccumulation of arsenic by Corynebacterium glutamicum MTCC 2745 biofilm supported on NL/MnFe2O4 composite	2016

9719	N. Sahiner	The use of superporous p(3-acrylamidopropyl)trimethyl ammonium chloride cryogels for removal of toxic arsenate anions	2015
9720	H. A. L. de Oliveira	Elaboration of a core@shell bimagnetic nanoadsorbent (CoFe2O4@gamma-Fe2O3) for the removal of As(V) from water	2020
9721	P. F. Qi	Competitive adsorption of As(III), As(V), Sb(III) and Sb(V) onto ferrihydrite in multi-component systems: Implications for mobility and distribution	2017
9722	S. P. Indraratne	A laboratory assay of in situ stabilization of toxic metals in contaminated boreal forest soil using organic and inorganic amendments	2020
9723	D. K. Lee	Improvement of Resistive Switching Characteristics of Titanium Oxide Based Nanowedge RRAM Through Nickel Silicidation	2021
9724	Y. R. Song	A novel halloysite-CeOx nanohybrid for efficient arsenic removal	2020
9725	P. K. Samantaray	Interlocked Dithi-Magnetospheres-Decorated MoS2 Nanosheets as Molecular Sieves and Traps for Heavy Metal Ions	2019
9726	T. Assawincharoenkij	Mineralogical and geochemical characterization of waste rocks from a gold mine in northeastern Thailand: application for environmental impact protection	2018
9727	Z. F. Wang	Influence of moderate pre-oxidation treatment on the physical, chemical and phosphate adsorption properties of iron-containing activated carbon	2014
9728	E. P. Munzhelele	Synthesis of Fe Doped Poly p-Phenylenediamine Composite: Co-Adsorption Application on Toxic Metal Ions (F- and As3+) and Microbial Disinfection in Aqueous Solution	2021
9729	S. K. Jalmi	Traversing the Links between Heavy Metal Stress and Plant Signaling	2018
9730	E. Shokri	Preparation and characterization of polysulfone/organoclay adsorptive nanocomposite membrane for arsenic removal from contaminated water	2016
9731	P. S. Ghosal	Bi-Objective Optimization through Pareto Frontier Analysis and Artificial Neural Network for Adsorptive Removal of Fluoride by a Novel Al/Olivine Composite	2018
9732	M. P. Karwowski	Toxicants in folk remedies: implications of elevated blood lead in an American-born infant due to imported diaper powder	2017
9733	E. E. M. Tripodi	Characterization and geostatistical modelling of contaminants and added value metals from an abandoned Cu-Au tailing dam in Taltal (Chile)	2019
9734	G. F. Wu	Taurine attenuates AFB1-induced liver injury by alleviating oxidative stress and regulating mitochondria-mediated apoptosis	2022
9735	E. Moreno-Jimenez	Efficiency of organic and mineral based amendments to reduce metal loid mobility and uptake (Lolium perenne) from a pyrite-waste contaminated soil	2017
9736	Z. Huang	y Preparation of a Novel Zn(II)-Imidazole Framework as an Efficient and Regenerative Adsorbent for Pb, Hg, and As Ion Removal From Water	2020

9737	H. Dong	Hybrid Ion Exchange Desalination (HIX-Desal) of Impaired Brackish Water Using Pressurized Carbon Dioxide (CO2) as the Source of Energy and Regenerant	2018
9738	S. Wachowski	Tailoring structural properties of lanthanum orthoniobates through an isovalent substitution on the Nb-site	2018
9739	K. Telfeyan	Cycling of oxyanion-forming trace elements in groundwaters from a freshwater deltaic marsh	2018
9740	G. Iervolino	Removal of arsenic from drinking water by photo-catalytic oxidation on MoOx/TiO2 and adsorption on gamma-Al2O3	2016
9741	Y. X. Li	Promoting dynamic adsorption of Pb2+ in a single pass flow using fibrous nano-TiO2/cellulose membranes	2016
9742	Y. X. Ma	SIMULTANEOUS REMOVAL OF PB, CD, HG AND AS FROM SMELTING FLUE GAS BY SODIUM SULFIDE SOLUTION	2016
9743	R. Gupta	Highly Sensitive Detection of Arsenite Based on Its Affinity toward Ruthenium Nanoparticles Decorated on Glassy Carbon Electrode	2016
9744	D. B. Sims	Trace elements migrating from tailings to rock varnish laminated sediments in an old mining region from Nelson, Nevada, USA	2022
9745	S. Shalvi	Trace level electrochemical analysis of arsenite in human serum utilising rGO/AuNPs based sensor platform	
9746	S. Mamuya	Dust Exposure, Fractional Exhaled Nitric Oxide and Respiratory Symptoms among Volcanic Rock Miners in Kilimanjaro, Tanzania	2018
9747	A. Sharma	Multi-element effects on arsenate accumulation in a geochemical matrix determined using mu-XRF, mu-XANES and spatial statistics	2019
9748	P. F. Qi	Closer Look at As(III) and As(V) Adsorption onto Ferrihydrite under Competitive Conditions	2014
9749	G. Alvarez	Hydrogen embrittlement testing procedure for the analysis of structural steels with Small Punch Tests using notched specimens	2021
9750	A. Pranudta	Selective removal of Pb from lead-acid battery wastewater using hybrid gel cation exchanger loaded with hydrated iron oxide nanoparticles: Fabrication, characterization, and pilot-scale validation	2021
9751	X. Z. Xiao	Phosphorus removal and recovery from secondary effluent in sewage treatment plant by magnetite mineral microparticles	2017
9752	M. Peli	Profiles and species of Mn, Fe and trace metals in soils near a ferromanganese plant in Bagnolo Mella (Brescia, IT)	2021
9753	M. Delavar	Fabrication and characterization of polyvinyl chloride mixed matrix membranes containing high aspect ratio anatase titania and hydrous manganese oxide nanoparticle for efficient removal of heavy metal ions: Competitive removal study	2020
9754	X. Chen	Effects of Surface Iron Hydroxyl Group Site Densities on Arsenate Adsorption by Iron Oxide Nanocomposites	2016

9755	G. Alvarez	Hydrogen embrittlement analysis in a CrMoV steel by means of sent specimens	2020
9756	N. Pujari	Effect of Li2O content on structural and optical properties of Li2O-TeO2-As2O3-B2O3 glasses	2021
9757	Y. C. Wu	Arsenic Trioxide Induces Apoptosis in Human Platelets via C-Jun NH2-Terminal Kinase Activation	2014
9758	E. Hernandez-Mendiola	Solid phases controlling the mobility of potentially toxic elements and the generation of acid drainage in abandoned mine gold wastes from San Antonio-El Triunfo mining district, Baja California Sur, Mexico	2016
9759	Q. L. Zhao	Effect of renal impairment on arsenic accumulation, methylation capacity, and safety in acute promyelocytic leukemia (APL) patients treated with arsenic trioxide	2021
9760	T. S. Shi	Deep purification of As(V) in drinking water by silica gel loaded with FeOOH and MnO2	2021
9761	E. Even	Geochemical distribution and fate of arsenic in water and sediments of rivers from the Hokusetsu area, Japan	2017
9762	T. L. Wu	Unraveling the effects of gallic acid on Sb(III) adsorption and oxidation on goethite	2019
9763	X. Y. Lin	Iron-doped chitosan microsphere for As(III) adsorption in aqueous solution: Kinetic, isotherm and thermodynamic studies	2019
9764	H. Mehdi	Investigation of N-2 plasma GaAs surface passivation efficiency against air exposure: Towards an enhanced diode	2022
9765	X. X. Long	Removal of Cd(II) from Micro-Polluted Water by Magnetic Core-Shell Fe3O4@Prussian Blue	2021
9766	H. Shin	Spectral Interference of Heavy Metal Contamination on Spectral Signals of Moisture Content for Heavy Metal Contaminated Soils	2020
9767	C. Chen	Mechanism of Arsenate Adsorption by Basic Yttrium Carbonate in a Fixed-Bed Column	2017
9768	M. N. Filimon	Environmental impact of mining activity in Bor area as indicated by the distribution of heavy metals and bacterial population dynamics in sediment	2016
9769	A. A. Kusnierz	Influence of the increased content of Calumite blast-furnace slag on the melting of sodium-calcium-silicate glass	2019
9770	G. K. R. Angaru	Portable SA/CMC entrapped bimetallic magnetic fly ash zeolite spheres for heavy metals contaminated industrial effluents treatment via batch and column studies	2022
9771	F. C. Hawthorne	Long-range and short-range cation order in the crystal structures of carlfrancisite and mcgovernite	2018
9772	S. Diquattro	Insights into the fate of antimony (Sb) in contaminated soils: Ageing influence on Sb mobility, bioavailability, bioaccessibility and speciation	2021

9773	K. Shehzad	Facile synthesis of novel calcined magnetic orange peel composites for efficient removal of arsenite through simultaneous oxidation and adsorption	2018
9774	C. Tokoro	Mechanism of arsenate coprecipitation at the solid/liquid interface of ferrihydrite: A perspective review	2020
9775	H. Y. Wang	Simultaneous adsorption and immobilization of As and Cd by birnessite-loaded biochar in water and soil	2019
9776	M. O. Schwartz	Modelling leakage and groundwater pollution in a hypothetical CO2 sequestration project	2014
9777	Z. H. Khan	Efficient As(III) Removal by Novel MoS2-Impregnated Fe-Oxide-Biochar Composites: Characterization and Mechanisms	2020
9778	J. van den Berg	Characterization of arsenic plasma doping and postimplant processing of silicon using medium energy ion scattering	2019
9779	P. Singh	Sustainable Low-Concentration Arsenite As(III) Removal in Single and Multicomponent Systems Using Hybrid Iron Oxide-Biochar Nanocomposite Adsorbents-A Mechanistic Study	2020
9780	X. Xia	Sensitive detection of As(III) on Fe3O4/MoS2 through interfacial engineering to accelerate the Fe2+/Fe3+ cycle: Identifying the dominant role of electron transfer induced by valence change in synergistic electroanalysis	2022
9781	Y. F. Wei	Enhanced arsenite removal from water by radially porous Fe-chitosan beads: Adsorption and H2O2 catalytic oxidation	2019
9782	R. Tao	The Suitability of Hybrid Fe-0/Aggregate Filtration Systems for Water Treatment	2022
9783	A. L. Seyfferth	Arsenic Concentrations in Paddy Soil and Rice and Health Implications for Major Rice-Growing Regions of Cambodia	2014
9784	S. L. Diaz	Control factors of the spatial distribution of arsenic and other associated elements in loess soils and waters of the southern Pampa (Argentina)	2016
9785	Y. Zhang	Impacts of pH on leaching potential of elements from MSW incineration fly ash	2016
9786	F. Zhang	Efficient arsenate removal by magnetite-modified water hyacinth biochar	2016
9787	T. M. Grygar	Segregation and retention of As, potentially toxic metals, and organic pollutants in a reservoir from the Ohre River (the Czech Republic)	2020
9788	T. T. Huynh	Retinoic acid and arsenic trioxide induce lasting differentiation and demethylation of target genes in APL cells	2019
9789	N. Sahu	Removal of arsenic from aqueous solution by novel iron and iron-zirconium modified activated carbon derived from chemical carbonization of Tectona grandis sawdust: Isotherm, kinetic, thermodynamic and breakthrough curve modelling	2021
9790	M. L. Sun	Heavy metal adsorption onto graphene oxide, amino group on magnetic nanoadsorbents and application for detection of Pb(II) by strip sensor	2018

9791	M. Ateia	Green and facile approach for enhancing the inherent magnetic properties of carbon nanotubes for water treatment applications	2017
9792	I. Shiue	Higher Urinary Heavy Metal, Phthalate, and Arsenic but Not Parabens Concentrations in People with High Blood Pressure, US NHANES, 2011-2012	2014
9793	G. Bia	Changes in the As solid speciation during weathering of volcanic ashes: A XAS study on Patagonian ashes and Chacopampean loess	2017
9794	T. Fresno	Assessing the combination of iron sulfate and organic materials as amendment for an arsenic and copper contaminated soil. A chemical and ecotoxicological approach	2016
9795	X. He	Effect of aluminum modification of rice straw-based biochar on arsenate adsorption	2020
9796	R. S. Bangari	Fe3O4-Functionalized Boron Nitride Nanosheets as Novel Adsorbents for Removal of Arsenic(III) from Contaminated Water	2020
9797	Z. Y. Ma	Efficient adsorption of Selenium(IV) from water by hematite modified magnetic nanoparticles	2018
9798	J. Han	Chemical extractability of As and Pb from soils across long-term abandoned metallic mine sites in Korea and their phytoavailability assessed by Brassica juncea	2015
9799	J. M. Wei	Experimental and theoretical investigations on Se(iv) and Se(vi) adsorption to UiO-66-based metal-organic frameworks	2018
9800	J. Sevink	Rare Moss-Built Microterraces in a High-Altitude, Acid Mine Drainage-Polluted Stream (Cordillera Negra, Peru)	2015
9801	Z. G. Li	Associations between metal exposure and global DNA methylation in potentially affected people in E-Waste recycling sites in Taizhou City, China	2020
9802	J. B. Huo	Magnetic responsive Fe3O4-ZIF-8 core-shell composites for efficient removal of As(III) from water	2018
9803	Y. N. Yuan	Immobilization of Cd and Pb in soils by polymeric hydroxyl ferric phosphate	2017
9804	J. J. Yee	Treatment of Contaminated Groundwater via Arsenate Removal Using Chitosan-Coated Bentonite	2019
9805	H. N. Huang	CuO and ZnO nanoparticles drive the propagation of antibiotic resistance genes during sludge anaerobic digestion: possible role of stimulated signal transduction	2019
9806	A. Pholosi	Enhanced Arsenic (III) adsorption from aqueous solution by magnetic pine cone biomass	2019
9807	J. Q. Liu	Synergistic deep removal of As(III) and Cd(II) by a calcined multifunctional MgZnFe-CO3 layered double hydroxide: Photooxidation, precipitation and adsorption	2019
9808	S. L. Hou	Green synthesis and evaluation of an iron-based metal-organic framework MIL-88B for efficient decontamination of arsenate from water	2018

9809	X. Lu	Thermodynamic Considerations of Contamination by Alloying Elements of Remelted End-of-Life Nickel- and Cobalt-Based Superalloys	2016
9810	W. Ding	Sulfite activation by a low-leaching silica-supported copper catalyst for oxidation of As(III) in water at circumneutral pH	2019
9811	A. Baranik	Ceria nanoparticles deposited on graphene nanosheets for adsorption of copper(II) and lead(II) ions and of anionic species of arsenic and selenium	2018
9812	Y. J. Lee	Low-Temperature Microwave Annealing Processes for Future IC Fabrication-A Review	2014
9813	N. Zhang	ZVI impregnation altered arsenic sorption by ordered mesoporous carbon in presence of Cr(VI): A mechanistic investigation	2021
9814	K. Cai	Pollution, Source, and Relationship of Trace Metal(loid)s in Soil-Wheat System in Hebei Plain, Northern China	2019
9815	L. Y. Jiang	Preparation of magnetically recoverable bentonite-Fe3O4-MnO2 composite particles for Cd(II) removal from aqueous solutions	2018
9816	S. Mourdikoudis	Colloidal chemical bottom-up synthesis routes of pnictogen (As, Sb, Bi) nanostructures with tailored properties and applications: a summary of the state of the art and main insights	2021
9817	T. H. Gu	Transformation of nanoscale zero-valent iron with antimony: Effects of the Sb spatial configuration	2021
9818	D. Xia	Mesoporous Activated Biochar for As(III) Adsorption: A New Utilization Approach for Biogas Residue	2019
9819	J. H. Li	Modification and use of biochar from wheat straw (Triticum aestivum L.) for nitrate and phosphate removal from water	2016
9820	H. T. Ren	Visible light-induced oxidation of aqueous arsenite using facile Ag2O/TiO2 composites: Performance and mechanism	2019
9821	H. Y. Chen	Microwave-assisted rapid synthesis of Fe2O3/ACF hybrid for high efficient As(V) removal	2016
9822	C. Shan	Efficient removal of trace antimony(III) through adsorption by hematite modified magnetic nanoparticles	2014
9823	P. K. Wang	A Glance of the Electrochemical Co-Deposition of Indium and Arsenic in a Choline Chloride/Ethylene Glycol Deep Eutectic Solvent	2019
9824	M. Zeinvand-Lorestani	Autophagy upregulation as a possible mechanism of arsenic induced diabetes	2018
9825	S. P. Wang	Tetraarsenictetrasulfide and Arsenic Trioxide Exert Synergistic Effects on Induction of Apoptosis and Differentiation in Acute Promyelocytic Leukemia Cells	2015
9826	S. H. Hu	Resource Recycling of Gallium Arsenide Scrap Using Leaching-Selective Precipitation	2015

9827	X. Y. Li	The antitumor effects of arsenic trioxide in mantle cell lymphoma via targeting Wnt/beta-catenin pathway and DNA methyltransferase-1	2017
9828	K. Panova	Modeling of Bohemian and Moravian glass recipes from Gothic to Baroque periods	2020
9829	L. Bindi	Ferrofettelite, Ag6As2S7 Ag10FeAs2S8 , a new sulfosalt from the Glasberg quarry, Odenwald, Germany	2022
9830	M. Wada	Reactions of tris(2,6-dimethoxyphenyl) arsine, tris(2,6-dimethoxyphenyl) stibine and tris(2,6-dimethoxyphenyl) bismuthine and their derivatives	1996
9831	A. R. Kampf	Chongite, Ca3Mg2(AsO4)2(AsO3OH)(2)center dot 4H(2)O, a new arsenate member of the hureaulite group from the Torrecillas mine, Iquique Province, Chile	2016
9832	S. Zhang	Effects of Mn average oxidation state on the oxidation behaviors of As(III) and Cr(III) by vernadite	2018
9833	Y. Cruz-Hernandez	Fractionation and mobility of thallium in areas impacted by mining-metallurgical activities: Identification of a water-soluble Tl(I) fraction	2018
9834	A. C. Senn	Arsenate co-precipitation with Fe(II) oxidation products and retention or release during precipitate aging	2018
9835	A. K. Cuevas	Metal rich particulate matter impairs acetylcholine-mediated vasorelaxation of microvessels in mice	2015
9836	J. Fan	Self-Assembled Nanocomplex for Co-Delivery of Arsenic-Retinoic Acid Prodrug into Acute Promyelocytic Leukemia Cells	2018
9837	S. M. Prabhu	Designed synthesis of sulfide-rich bimetallic-assembled graphene oxide sheets as flexible materials and self-tuning adsorption cum oxidation mechanisms of arsenic from water	2019
9838	C. M. Schwarz	Processing and properties of novel ZnO-Bi2O3-B2O3 glass-ceramic nanocomposites	2020
9839	M. Humbert	Distinct TP73-DAPK2-ATG5 pathway involvement in ATO-mediated cell death versus ATRA-mediated autophagy responses in APL	2017
9840	B. Q. Xia	Kinetics of antimony biogeochemical processes under pre-definite anaerobic and aerobic conditions in a paddy soil	2022
9841	M. A. Rahman	Removal of arsenate from contaminated waters by novel zirconium and zirconium-iron modified biochar	2021
9842	K. Simeonidis	Implementing nanoparticles for competitive drinking water purification	2019
9843	T. Sukitprapanon	Partitioning and potential mobilization of aluminum, arsenic, iron, and heavy metals in tropical active and post-active acid sulfate soils: Influence of long-term paddy rice cultivation	2018
9844	C. Z. Yan	Changes in arsenate bioaccumulation, subcellular distribution, depuration, and toxicity in Artemia salina nauplii in the presence of titanium dioxide nanoparticles	2017

9845	P. K. Raul	CuO nanorods: a potential and efficient adsorbent in water purification	2014
9846	A. Mirzaei	Combined anticancer effects of simvastatin and arsenic trioxide on prostate cancer cell lines via downregulation of the VEGF and OPN isoforms genes	2022
9847	K. H. Shah	Potential of natural ferruginous manganese (NFM) ore as a natural adsorbent for As(III) removal at low concentration	
9848	A. Borovik	Platform selection of engineered nanomaterials for water decontamination applications	2020
9849	H. X. Tang	Dual GSH-exhausting sorafenib loaded manganese-silica nanodrugs for inducing the ferroptosis of hepatocellular carcinoma cells	2019
9850	M. A. Rahman	Adsorption-Desorption Behavior of Arsenate Using Single and Binary Iron-Modified Biochars: Thermodynamics and Redox Transformation	2022
9851	D. M. Yan	Cleanup of arsenic, cadmium, and lead in the soil from a smelting site using N,N-bis(carboxymethyl)-L-glutamic acid combined with ascorbic acid: A lab-scale experiment	2021
9852	Z. Z. Tian	Phosphoric acid-induced activation of sepiolite for enhanced As(III) adsorption: role of in situ deposition of nano-hydroxyapatite	
9853	F. H. Narouei	Rapid characterization of arsenic adsorption on single magnetite nanoparticles by collisions at microelectrodes	2020
9854	P. M. Zandi	Do sulfur addition and rhizoplane iron plaque affect chromium uptake by rice (Oryza sativa L.) seedlings in solution culture?	2020
9855	J. A. Park	Influence of As(V) on bacteriophage MS2 removal by hematite in aqueous solutions	2015
9856	S. N. Aso	Mitigation of external costs of inorganic fertilizers with liquid fraction digestate	
9857	Y. L. Xie	Substitution-mediated enhanced adsorption of low concentration As(v) from water by mesoporous MnxFe3-xO4 microspheres	2019
9858	X. Han	Lepidocrocite-catalyzed Mn(II) oxygenation by air and its effect on the oxidation and mobilization of As(III)	2016
9859	D. Kenessary	Air Pollution in Kazakhstan and Its Health Risk Assessment	2019
9860	Y. Sun	Taurine suppresses ROS-dependent autophagy via activating Akt/mTOR signaling pathway in calcium oxalate crystals-induced renal tubular epithelial cell injury	2020
9861	T. Liu	Tetrandrine, a Chinese plant-derived alkaloid, is a potential candidate for cancer chemotherapy	2016
9862	C. J. Jiang	Fe3+ coordinated to amino-functionalized spherical mesoporous silica: Preparation, characterization, and application as a rapid and efficient adsorbent for As(V) removal	2021

9863	Y. M. Song	Insight into the inhibitory mechanism of soluble ionic liquids on the transport of TiO2 nanoparticles in saturated porous media: Roles of alkyl chain lengths and counteranion types	2021
9864	M. Karanac	The removal of Zn2+, Pb2+, and As(V) ions by lime activated fly ash and valorization of the exhausted adsorbent	2018
9865	M. T. Olguin	Ce-Fe-modified zeolite-rich tuff to remove Ba2+-like Ra-226(2+) in presence of As(V) and F- from aqueous media as pollutants of drinking water	2016
9866	T. T. Ma	The relationship between selenium retention and fine particles removal during coal combustion	2020
9867	Y. Georgiou	Mesoporous spinel CoFe2O4 as an efficient adsorbent for arsenite removal from water: high efficiency via control of the particle assemblage configuration	2019
9868	S. Jeong	Enhanced uptake and translocation of arsenic in Cretan brake fern (Pteris cretica L.) through siderophorearsenic complex formation with an aid of rhizospheric bacterial activity	2014
9869	R. Y. Li	Template-free synthesis of an eco-friendly flower-like Mg/Al/Fe-CLDH for efficient arsenate removal from aqueous solutions	2022
9870	S. M. Beladi-Mousavi	Recoverable Bismuth-Based Microrobots: Capture, Transport, and On-Demand Release of Heavy Metals and an Anticancer Drug in Confined Spaces	2019
9871	Z. D. Yuan	The stability of Fe(III)-As(V) co-precipitate in the presence of ascorbic acid: Effect of pH and Fe/As molar ratio	2019
9872	A. K. Meher	Simultaneous removal of selenite and selenate from drinking water using mesoporous activated alumina	2019
9873	S. H. Yao	Preparation, characterization of Fe, Ce Co-doped flower-like TiO2 photocatalysts and their properties in photocatalytic oxidation of arsenite	2017
9874	F. Y. Chen	Development of a hybrid paclitaxel-loaded arsenite nanoparticle (HPAN) delivery system for synergistic combined therapy of paclitaxel-resistant cancer	2017
9875	M. A. Salam	Synthesis and Characterization of Green ZnO@polynaniline/Bentonite Tripartite Structure (G.Zn@PN/BE) as Adsorbent for As (V) Ions: Integration, Steric, and Energetic Properties	2022
9876	J. S. Roh	The pretreatment of granular activated carbon using sodium persulfate and hydrogen peroxide under basic conditions: Properties, metal impregnation, and As(V) adsorption	2018
9877	J. Z. Li	Aluminum-Cycle Ion Exchange Process for Hardness Removal: A New Approach for Sustainable Softening	2016
9878	J. Kim	Influence of an iron-rich amendment on chemical lability and plant (Raphanus sativus L.) availability of two metallic elements (As and Pb) on mine-impacted agricultural soils	2016
9879	N. Daver	Clinical characteristics and outcomes in patients with acute promyelocytic leukaemia and hyperleucocytosis	2015
9880	J. S. Yang	The transport behavior of As, Cu, Pb, and Zn during electrokinetic remediation of a contaminated soil using electrolyte conditioning	2014

9881	T. N. Yu	Efficient removal of bismuth with supersoluble amorphous antimony acids: An insight into synthesis mechanism and Sb(V)-Bi(III) interaction behaviors	2021
9882	E. Priyadarshini	Metal-Fungus interaction: Review on cellular processes underlying heavy metal detoxification and synthesis of metal nanoparticles	2021
9883	X. C. Xu	Three-dimensional flower-like multifunctional adsorbents with excellent sorptive removal and colorimetric detection of arsenate	2020
9884	M. S. C. Barreto	Hausmannite as potential As(V) filter. Macroscopic and spectroscopic study of As(V) adsorption and desorption by citric acid	2020
9885	A. K. Shakya	A practical approach on reuse of drinking water treatment plant residuals for fluoride removal	2020
9886	Y. Kitamura	Effect of reaction temperature on the size and morphology of scorodite synthesized using ultrasound irradiation	2017
9887	M. Imtiaz	Interactive effects of vanadium and phosphorus on their uptake, growth and heat shock proteins in chickpea genotypes under hydroponic conditions	2017
9888	A. M. Eid	Surface chemistry of bovine serum albumin with hematite nanoparticles and its effect on arsenate adsorption	2021
9889	Y. H. Li	Effects of Dissolved Organic Matter on the Bioavailability of Heavy Metals During Microbial Dissimilatory Iron Reduction: A Review	2021
9890	D. Barh	Multi-omits-based identification of SARS-CoV-2 infection biology and candidate drugs against COVID-19	2020
9891	A. Singh	Batch leaching testing of stabilized dredged bottom-sea sediments	2020
9892	D. F. Fang	Tandem type PRBs-like technology implanted with targeted functional materials for efficient resourceful treatment of heavy metal ions from mining wastewater	2021
9893	H. T. Wang	Recycling spent iron-based disposable-chemical-warmer as adsorbent for as (v) removal from aqueous solution	2021
9894	M. Mulligan	The FLSmidth (R) Rapid Oxidative Leach (ROL) process: a mechano-chemical approach and industrial applications for rapid metal sulphide dissolution	2017
9895	D. K. Ingawale	Models of hepatotoxicity and the underlying cellular, biochemical and immunological mechanism(s): A critical discussion	2014
9896	J. Y. Liu	Varying responses of PML-RARA with different genetic mutations to arsenic trioxide	2016
9897	N. H. Ghori	Heavy metal stress and responses in plants	2019
9898	C. Garza-Lombo	Neurotoxicity Linked to Dysfunctional Metal Ion Homeostasis and Xenobiotic Metal Exposure: Redox Signaling and Oxidative Stress	2018

9899	Q. Du	Iron-mediated oxidation of arsenic(III) by oxygen and hydrogen peroxide: Dispersed versus resin-supported zero-valent iron	2014
9900	R. Xiao	Enhanced sorption of hexavalent chromium Cr(VI) from aqueous solutions by diluted sulfuric acid-assisted MgO-coated biochar composite	2018
9901	A. Mitovski	Thermodynamic and kinetic analysis of the polymetallic copper concentrate oxidation process	2014
9902	M. Paul	Phyto-remedial detoxification of arsenic by Pistia stratiotes and assessment of its anti-oxidative enzymatic changes	2019
9903	J. L. Liu	Syntheses, structural characterization and photoluminescence properties of {AsO2(OH)}-bridging arsenotungstates incorporating lanthanide ions	2017
9904	K. Yoon	Adsorption of As(V) and Ni(II) by Fe-Biochar composite fabricated by co-pyrolysis of orange peel and red mud	2020
9905	A. Rouwane	Role of microbial reducing activity in antimony and arsenic release from an unpolluted wetland soil: a lab scale study using sodium azide as a microbial inhibiting agent	2016
9906	D. K. Singh	Kinetic, isotherm and thermodynamic studies of adsorption behaviour of CNT/CuO nanocomposite for the removal of As(III) and As(V) from water	2016
9907	R. Warchulski	Composition and element mobilization in pyrometallurgical slags from the Orzel Bialy smelting plant in the Bytom-Piekary Slaskie area, Poland	2015
9908	K. Semhi	Impact of sewage and mining activities on distribution of heavy metals in the water-soil-vegetation system	2014
9909	V. N. Thekkudan	Review on nanoadsorbents: a solution for heavy metal removal from wastewater	2017
9910	S. Carrero	The potential role of aluminium hydroxysulphates in the removal of contaminants in acid mine drainage	2015
9911	Y. M. Fu	Highly Selective Photoelectrochemical Assay of Arsenate Based on Magnetic Co3O4-Fe3O4 Cubes and the Negative-Background Signal Strategy	2022
9912	M. Lei	Safety assessment and application of iron and manganese ore tailings for the remediation of As-contaminated soil	2019
9913	B. Xu	Influence of iron plaque on the uptake and accumulation of chromium by rice (oryza sativa L.) seedlings: Insights from hydroponic and soil cultivation	2018
9914	G. B. Jegadeesan	Adsorption of Se (IV) and Se (VI) Using Copper-Impregnated Activated Carbon and Fly Ash-Extracted Char Carbon	2015
9915	W. A. El-Said	A reusable Gemini surfactant-based electrochemical sensor for As(III) detection	
9916	T. Fresno	Aided phytostabilisation of As- and Cu-contaminated soils using white lupin and combined iron and organic amendments	2018

9917	H. Y. Yu	Iron Redox Cycling Coupled to Transformation and Immobilization of Heavy Metals: Implications for Paddy Rice Safety in the Red Soil of South China	2016
9918	F. Q. Yang	A review of geological characteristics and time-space distribution of cobalt deposits in the Central Asian Orogenic Belt, Xinjiang, Northwest China	
9919	Z. Y. Ma	Facile synthesis of ZrO2 coated BiOCl0.5I0.5 for photocatalytic oxidation-adsorption of As(III) under visible light irradiation	2018
9920	D. Virk	Ameliorative role of rolipram, PDE-4 inhibitor, against sodium arsenite-induced vascular dementia in rats	2021
9921	T. Q. Hong	A constant composition technique for quantifying the effect of As(V) on struvite crystallization under various operational conditions	2020
9922	H. P. Zeng	As(V) Removal from Water Using a Novel Magnetic Particle Adsorbent Prepared with Iron-Containing Water Treatment Residuals	2018
9923	Y. J. Zhao	Magnetic mesoporous TiO2 microspheres for sustainable arsenate removal from acidic environments	2018
9924	X. M. Dou	A property-performance correlation and mass transfer study of As(V) adsorption on three mesoporous aluminas	2016
9925	C. Mikutta	Bioaccessibility of Arsenic in Mining-Impacted Circumneutral River Floodplain Soils	2014
9926	Y. Luo	Taurine attenuates liver autophagy and injury of offspring in gestational diabetic mellitus rats	2020
9927	Y. Maimaitiyiming	Irreversibility of arsenic trioxide induced PML/RAR alpha fusion protein solubility changes	2019
9928	X. T. Vuong	Speciation and environmental risk assessment of heavy metals in soil from a lead/zinc mining site in Vietnam	
9929	J. C. Bullen	A Revised Pseudo-Second-Order Kinetic Model for Adsorption, Sensitive to Changes in Adsorbate and Adsorbent Concentrations	2021
9930	M. Zafar	Ethanol mediated As(III) adsorption onto Zn-loaded pinecone biochar: Experimental investigation, modeling, and optimization using hybrid artificial neural network-genetic algorithm approach	2017
9931	C. Shan	Dual-functional millisphere of anion-exchanger-supported nanoceria for synergistic As(III) removal with stoichiometric H2O2: Catalytic oxidation and sorption	2019
9932	T. H. Bui	Development of nanoscale zirconium molybdate, embedded anion exchange resin for selective removal of phosphate	2018
9933	G. Tepanosyan	Human health risk assessment and riskiest heavy metal origin identification in urban soils of Yerevan, Armenia	2017
9934	E. Arhin	Trace elements distributions at Datoko-Shega artisanal mining site, northern Ghana	2016

9935	L. B. Grossi	Purifying surface water contaminated with industrial failure using direct contact membrane distillation	2020
9936	Y. L. Tu	Field demonstration of on-site immobilization of arsenic and lead in soil using a ternary amending agent	2022
9937	P. Lopez-Arce	Historical roasting of thallium- and arsenic-bearing pyrite: Current Tl pollution in the Riotinto mine area	2019
9938	S. H. Wang	Kinetic modeling of As release from contaminated soils: Consideration of particle size and co-contamination of Cu	2022
9939	X. J. Song	Ligand effects on arsenite removal by zero-valent iron/O-2: Dissolution, corrosion, oxidation and coprecipitation	2019
9940	Y. R. Li	Ingredients from Litsea garrettii as Potential Preventive Agents against Oxidative Insult and Inflammatory Response	2018
9941	D. Baragano	Zero valent iron and goethite nanoparticles as new promising remediation techniques for As-polluted soils	2020
9942	K. K. Kadyrzhanov	Study of Corrosion Mechanisms in Corrosive Media and Their Influence on the Absorption Capacity of Fe2O3/NdFeO3 Nanocomposites	2022
9943	M. Popovic	Removal of the As(V) and Cr(VI) from the Water Using Magnetite/3D-Printed Wollastonite Hybrid Adsorbent	2022
9944	N. V. Hop	Metal speciation in sediment and bioaccumulation in Meretrix lyrata in the Tien Estuary in Vietnam	2017
9945	C. M. Yu	A new disposable electrode for electrochemical study of leukemia K562 cells and anticancer drug sensitivity test	2014
9946	T. Nitto	Molecular Mechanisms of the Antileukemia Activities of Retinoid and Arsenic	2014
9947	K. Kupczak	Chemical and Phase Reactions on the Contact between Refractory Materials and Slags, a Case from the 19th Century Zn-Pb Smelter in Ruda slaska, Poland	2020
9948	P. Voudouris	Arsenian Pyrite and Cinnabar from Active Submarine Nearshore Vents, Paleochori Bay, Milos Island, Greece	2021
9949	S. Khademinia	Solar Light Photocatalytic Degradation of Malachite Green by Hydrothermally Synthesized Strontium Arsenate Nanomaterial through Response Surface Methodology	2018
9950	Q. M. Wang	Reaction Mechanism and Distribution Behavior of Arsenic in the Bottom Blown Copper Smelting Process	2017
9951	A. Takeshita	Tamibarotene for the treatment of acute promyelocytic leukemia	2014
9952	C. Biagioni	Mercury-arsenic sulfosalts from the Apuan Alps (Tuscany, Italy). II. Arsiccioite, AgHg2TlAs2S6, a new mineral from the Monte Arsiccio mine: occurrence, crystal structure and crystal chemistry of the routhierite isotypic series	2014

9953	J. C. Rushton	RED-BED BLEACHING IN A CO2 STORAGE ANALOGUE: INSIGHTS FROM ENTRADA SANDSTONE FRACTURE-HOSTED MINERALIZATION	2020
9954	A. Ashok	Optical and Structural Studies of B2O3-ZnO-Na2O-Li2O Glasses Containing Ag Nano Particles	2020
9955	G. N. Mannis	Delayed hematopoietic recovery after auto-SCT in patients receiving arsenic trioxide-based therapy for acute promyelocytic leukemia: a multi-center analysis	2015
9956	H. Li	Carnosic Acid-combined Arsenic Trioxide Antileukaemia Cells in the Establishment of NB4/SCID Mouse Model	2016
9957	X. Q. Wang	High-Performance Arsine Removal Using CuOx/TiO2 Sorbents under Low-Temperature Conditions	2018
9958	A. Ahmad	Cytotoxic and Genotoxic Effects of Arsenic on Primary Human Breast Cancer Cell Lines	2015
9959	M. S. Safarzadeh	Effects of sulfuric acid dosage on the baking of an enargite concentrate	2014
9960	L. E. Nava-Rivera	Transgenerational effects in DNA methylation, genotoxicity and reproductive phenotype by chronic arsenic exposure	2021
9961	M. D. Adams	Cyanide-free recovery of metals from polymetallic and refractory gold concentrates by the KellGold process	2020
9962	J. Chen	A Novel Resveratrol-Arsenic Trioxide Combination Treatment Synergistically Induces Apoptosis of Adriamycin-Selected Drug-Resistant Leukemia K562 Cells	2019
9963	P. Drahota	Effect of relative humidity on oxidation products of arsenopyrite and l?llingite	2022
9964	Y. H. Xi	Mechanism of zero-valent lead reduction for removing high concentration of arsenic from waste acid of lead smelting system	2021
9965	M. A. Moosavi	Autophagy: New Insights into Mechanisms of Action and Resistance of Treatment in Acute Promyelocytic leukemia	2019
9966	Y. A. Jiang	The whole transcriptome analysis and the circRNA-lncRNA network construction in arsenic trioxide-treated mice myocardium	2022
9967	Y. J. Zhang	Simultaneous Removal of Elemental Mercury and Arsine from a Reducing Atmosphere Using Chloride and Cerium Modified Activated Carbon	2019
9968	J. J. Hoyos	Influence of hydrogen on the microstructure and fracture toughness of friction stir welded plates of API 5L X80 pipeline steel	2019
9969	L. Wei	Forms and mobility of heavy metals/metalloids in sewage-irrigated soils in the North China Plain	2021
9970	X. F. Zhang	The effects of Langchuangding on the Th17 cell-related transcription factors BATF and RORyt in MRL_1pr mice	2020

9971	Q. Yang	Arsenic Trioxide Impacts Viral Latency and Delays Viral Rebound after Termination of ART in Chronically SIV-Infected Macaques	2019
9972	J. M. Hong	Life cycle environmental and economic assessment of lead refining in China	2017
9973	J. Mehlhorn	Time and temperature dependency of carbon dioxide triggered metal(loid) mobilization in soil	2016
9974	M. H. Sk	Effects of microstructures on hydrogen induced cracking of electrochemically hydrogenated double notched tensile sample of 4340 steel	2016
9975	S. Sen	Mn and As doping of beta-FeSi2 via a chemical method	2016
9976	J. Majzlan	Prediction and observation of formation of Ca-Mg arsenates in acidic and alkaline fluids: Thermodynamic properties and mineral assemblages at Jachymov, Czech Republic and Rotgulden, Austria	2021
9977	J. Hu	A combined miR-34a and arsenic trioxide nanodrug delivery system for synergistic inhibition of HCC progression after microwave ablation	2021
9978	M. Moradzadeh	Study of the mechanisms of crocetin-induced differentiation and apoptosis in human acute promyelocytic leukemia cells	2019
9979	Y. Lu	2-Bromopalmitate targets retinoic acid receptor alpha and overcomes all-trans retinoic acid resistance of acute promyelocytic leukemia	2019
9980	J. Q. Mi	Synergistic targeted therapy for acute promyelocytic leukaemia: a model of translational research in human cancer	2015
9981	T. Pichler	Distribution and mobility of geogenic molybdenum and arsenic in a limestone aquifer matrix	2015
9982	N. Chubar	Layered double hydroxides as the next generation inorganic anion exchangers: Synthetic methods versus applicability	2017
9983	S. Hussaini	Testing of 17-different leaching agents for the recovery of zinc from a carbonate-type Pb-Zn ore flotation tailing	2021
9984	J. Song	Application of a high-surface-area schwertmannite in the removal of arsenate and arsenite	2015
9985	J. Patwa	Heavy Metal-Induced Cerebral Small Vessel Disease: Insights into Molecular Mechanisms and Possible Reversal Strategies	2020
9986	Y. Karamalakova	Pulmonary Protein Oxidation and Oxidative Stress Modulation by Lemna minor L. in Progressive Bleomycin-Induced Idiopathic Pulmonary Fibrosis	2022
9987	S. Chatterjee	Toxic Metals and Autophagy	2014
9988	I. Antcibor	Trace metal distribution in pristine permafrost-affected soils of the Lena River delta and its hinterland, northern Siberia, Russia	2014

9989	M. W. Gitari	Geochemical fractionation of metals and metalloids in tailings and appraisal of environmental pollution in the abandoned Musina Copper Mine, South Africa	2018
9990	J. Y. Kim	The Green Tea Component (-)-Epigallocatechin-3-Gallate Sensitizes Primary Endothelial Cells to Arsenite-Induced Apoptosis by Decreasing c-Jun N-Terminal Kinase-Mediated Catalase Activity	2015
9991	P. F. Qi	Sequential and simultaneous adsorption of Sb(III) and Sb(V) on ferrihydrite: Implications for oxidation and competition	2016
9992	K. Cai	Cycling and total risks of multiple As fractions in the Beijing-Tianjin-Hebei area on the agricultural plain, China	2020
9993	M. Rajendran	Effect of sulfur and sulfur-iron modified biochar on cadmium availability and transfer in the soil-rice system	2019
9994	B. Huang	Distribution characteristics of heavy metal(loid)s in aggregates of different size fractions along contaminated paddy soil profile	2017
9995	S. Gupta	Latest trends in heavy metal removal from wastewater by biochar based sorbents	2020
9996	H. M. Guo	High Radionuclides in Groundwater of an Inland Basin from Northwest China: Origin and Fate	2018
9997	M. Grafe	Speciation of metal(loid)s in environmental samples by X-ray absorption spectroscopy: A critical review	2014
9998	A. Agrawal	Comparative performance assessment of solvents for the extraction of H2SO4 from spent electrolytic bleed stream of copper industry	2016
9999	B. D. Btatkeu-K	Characterizing the impact of MnO2 on the efficiency of Fe-0-based filtration systems	2014
10000	Z. W. Qiu	Enhanced water permeability and rejection of As(III) in groundwater by nanochannels and active center formed in nanofibrillated celluloses UF membranes with ZIF-8	2022
10001	T. Y. Liu	Pivotal effects of external Fe2+ on remediation of arsenite by zero-valent iron/persulfate: Efficiencies and mechanism	2020
10002	X. L. Huangfu	Effective removal of trace thallium from surface water by nanosized manganese dioxide enhanced quartz sand filtration	2017
10003	J. S. Yamani	Towards a selective adsorbent for arsenate and selenite in the presence of phosphate: Assessment of adsorption efficiency, mechanism, and binary separation factors of the chitosan-copper complex	2016
10004	H. Milnerowicz	Pro-inflammatory effects of metals in persons and animals exposed to tobacco smoke	2015
10005	S. Musil	Speciation Analysis of Arsenic by Selective Hydride Generation-Cryotrapping-Atomic Fluorescence Spectrometry with Flame-in-Gas-Shield Atomizer: Achieving Extremely Low Detection Limits with Inexpensive Instrumentation	2014
10006	Y. J. Wang	Arsenite photo-oxidation and removal by ferrihydrite in the presence of oxalate: a pH dependence and surface-mediated process	2022

10007	J. Wang	Synergy between indigenous bacteria and extracellular electron shuttles enhances transformation and mobilization of Fe(III)/As(V)	2021
10008	I. Martinez-Abad	Geology and mineralogy of the Au-As (Ag-Pb-Zn-Cu-Sb) polymetallic deposit of Valina-Azumara (Lugo, NW Spain)	2015
10009	C. H. Yang	Covalent organic framework EB-COF:Br as adsorbent for phosphorus (V) or arsenic (V) removal from nearly neutral waters	2020
10010	C. M. van Genuchten	Antimonate and arsenate speciation on reactive soil minerals studied by differential pair distribution function analysis	2016
10011	F. N. Lyonga	As(III) adsorption onto Fe-impregnated food waste biochar: experimental investigation, modeling, and optimization using response surface methodology	2021
10012	T. Kataoka	Influence of the Chemical Form of Antimony on Soil Microbial Community Structure and Arsenite Oxidation Activity	2018
10013	S. M. Baba	p15(Ink4b) Loss of Expression by Promoter Hypermethylation Adds to Leukemogenesis and Confers a Poor Prognosis in Acute Promyelocytic Leukemia Patients	2017
10014	M. M. R. Emara	Removal of fluoride from aqueous solution using Amberlite-IRA-aluminum sorbent nanoexchanger	2017
10015	J. M. Blake	Sediment cores in a municipal drinking-water reservoir as a record of geochemical transport within a watershed, Farmington Lake, New Mexico, USA	2022
10016	K. Kalaitzidou	Adsorption of Se(IV) and Se(VI) species by iron oxy-hydroxides: Effect of positive surface charge density	2019
10017	S. Raj	Novel GO/Fe-Mn hybrid for the adsorptive removal of Pb(II) ions from aqueous solution and the spent adsorbent disposability in cement mix: compressive properties and leachability study for circular economy benefits	
10018	G. Kalyvas	Single and combined effect of chelating, reductive agents, and agro-industrial by-product treatments on As, Pb, and Zn mobility in a mine-affected soil over time	2020
10019	R. Roychowdhury	Surface and interface properties of ZrO2/GaAs, SiO2/GaAs and GaP/GaAs hetero structures investigated by surface photovoltage spectroscopy	2019
10020	B. Liu	Arsenic trioxide transarterial chemoembolization with and without additional intravenous administration of arsenic trioxide in unresectable hepatocellular carcinoma with lung metastasis: a single-blind, randomized trial	2015
10021	B. Sarkar	The role of soils in the disposition, sequestration and decontamination of environmental contaminants	2021
10022	G. Kerr	Jarosite Solid Solution Associated with Arsenic-Rich Mine Waters, Macraes Mine, New Zealand	2015
10023	P. M. Zandi	Iron plaque formation, characteristics, and its role as a barrier and/or facilitator to heavy metal uptake in hydrophyte rice (Oryza sativa L.)	
10024	A. Qureshi	Potential of fly ash for neutralisation of acid mine drainage	2016

10025	S. Azizi	Two-step chromium photo-precipitation in the sequential UV/Sulfite/Manganese dioxide processes: Efficiency, kinetic, energy-economic evaluation, and sludge survey	2022
10026	H. P. Zeng	As(V) adsorption by a novel core-shell magnetic nanoparticles prepared with Iron-containing water treatment residuals	2021
10027	Q. Z. Tian	Distributions and Leaching Behaviors of Toxic Elements in Fly Ash	2018
10028	L. K. ThomasArrigo	Sulfidization of Organic Freshwater Flocs from a Minerotrophic Peatland: Speciation Changes of Iron, Sulfur, and Arsenic	2016
10029	E. S. Costa	Geochemical records in sediments of a tropical estuary (Southeastern coast of Brazil)	2016
10030	R. J. Liu	Performances of Methyl Blue and Arsenic(V) Adsorption from Aqueous Solution onto Magnetic 0.8Ni(0.5)Zn(0.5)Fe(2)O(4)/0.2SiO(2) Nanocomposites	2014
10031	N. Y. Yin	Effect of gut microbiota on in vitro bioaccessibility of heavy metals and human health risk assessment from ingestion of contaminated soils*	2021
10032	G. F. Liu	Effects of carbide slag, lodestone and biochar on the immobilization, plant uptake and translocation of As and Cd in a contaminated paddy soil	2020
10033	M. Bizouerne	Low damage patterning of In0.53Ga0.47As film for its integration as n-channel in a fin metal oxide semiconductor field effect transistor architecture	2018
10034	W. H. Zhang	Sludge-Derived Biochar for Arsenic(III) Immobilization: Effects of Solution Chemistry on Sorption Behavior	2015
10035	D. Mohan	Lead sorptive removal using magnetic and nonmagnetic fast pyrolysis energy cane biochars	2015
10036	M. Arshadi	Nanoscale Zero-Valent Iron (NZVI) supported on sineguelas waste for Pb(II) removal from aqueous solution: Kinetics, thermodynamic and mechanism	2014
10037	H. S. Jahin	Facile auto-combustion synthesis of calcium aluminate nanoparticles for efficient removal of Ni(II) and As(III) ions from wastewater	
10038	M. Giels	Understanding the leaching behavior of inorganic polymers made of iron rich slags	2019
10039	H. H. Chen	EDTA Assisted Highly Selective Detection of As3+ on Au Nanoparticle Modified Glassy Carbon Electrodes: Facile in Situ Electrochemical Characterization of Au Nanoparticles	2014
10040	W. H. Park	Upregulation of thioredoxin and its reductase attenuates arsenic trioxide-induced growth suppression in human pulmonary artery smooth muscle cells by reducing oxidative stress	2020
10041	H. Y. Zhu	The toxicity and safety of Chinese medicine from the bench to the bedside	2021
10042	P. Kumar	Characterization, activity and process optimization with a biomass-based thermal power plant's fly ash as a potential catalyst for biodiesel production	2015

10043	H. M. Li	Chemical fractionation of arsenic and heavy metals in fine particle matter and its implications for risk assessment: A case study in Nanjing, China	2015
10044	J. P. Gustafsson	Competitive Arsenate and Phosphate Adsorption on Ferrihydrite as Described by the CD-MUSIC Model	2022
10045	L. N. Pincus	Exploring the Mechanisms of Selectivity for Environmentally Significant Oxo-Anion Removal during Water Treatment: A Review of Common Competing Oxo-Anions and Tools for Quantifying Selective Adsorption	2020
10046	P. Kumari	Usage of nanoparticles as adsorbents for waste water treatment: An emerging trend	2019
10047	N. Arif	Understanding Heavy Metal Stress in a Rice Crop: Toxicity, Tolerance Mechanisms, and Amelioration Strategies	2019
10048	E. J. Zhang	Separation of As and Bi and enrichment of As, Cu, and Zn from copper dust using an oxidation-leaching approach	2021
10049	W. H. Park	Arsenic trioxide induces growth inhibition and death in human pulmonary artery smooth muscle cells accompanied by mitochondrial O-2(center dot-) increase and GSH depletion	2018
10050	K. Y. Chen	Oxidative removal of thallium(I) using Al beverage can waste with amendments of Fe: Tl speciation and removal mechanisms	2022
10051	L. N. Pincus	Aerobic oxidation of arsenite to arsenate by Cu(ii)-chitosan/O-2 in Fenton-like reaction, a XANES investigation	2020
10052	Y. C. Liu	The protective effect of Zn2+ on As3+ toxicity in common carp: Resistance to oxidative stress, inhibition of endoplasmic reticulum stress, apoptosis and autophagy	2022
10053	A. R. Kampf	Cuyaite, Ca2Mn3+As143+O24Cl, a new mineral with an arsenite framework from near Cuya, Camarones Valley, Chile	2020
10054	Y. W. Song	Influence of Monovalent Cations on the Efficiency of Ferrous Ion Oxidation, Total Iron Precipitation, and Adsorptive Removal of Cr(VI) and As(III) in Simulated Acid Mine Drainage with Inoculation of Acidithiobacillus ferrooxidans	2018
10055	M. Ogawa	Arsenite suppresses the transcriptional activity of EVI1 through the binding to CCHC-type Zn finger domain	2020
10056	B. Kopp	Genotoxicity of 11 heavy metals detected as food contaminants in two human cell lines	2018
10057	A. M. Florea	Calcium-regulatory proteins as modulators of chemotherapy in human neuroblastoma	2017
10058	P. B. Hu	CO2 adsorption enhancement over alkaline metal-promoted MgO with SO2, O-2, and H2O present: A theoretical study	2022
10059	H. M. Chang	SUMO: FROM BENCH TO BEDSIDE	2020
10060	Y. D. Kim	Induction of telomere shortening and cellular apoptosis by sodium meta-arsenite in human cancer cell lines	2017

10061	Z. Liu	Microwave-assisted arsenic removal and the magnetic effects of typical arsenopyrite-bearing mine tailings	2015
10062	L. Zhang	Selective removal of arsenic from high arsenic dust in the NaOH-S system and leaching behavior of lead, antimony, zinc and tin	2021
10063	F. C. Goerigk	The Triclinic Lanthanoid(III) Halide Oxidoarsenates(III) Sm3Cl2 As2O5 AsO3 and Tm3Br2 As2O5 AsO3	2020
10064	M. H. Lin	Arsenic in Drinking Water and Incidences of Leukemia and Lymphoma: Implication for Its Dural Effects in Carcinogenicity	2022
10065	T. Y. Xu	Comparative response of interfacial water structure to pH variations and arsenate adsorption on corundum (012) and (001) surfaces	2019
10066	H. M. Prichard	Geochemistry and mineralogy of Pd in the magnetitite layer within the upper gabbro of the Mesoarchean Nuasahi Massif (Orissa, India)	2018
10067	I. A. Neamtiu	Impact of exposure to tobacco smoke, arsenic, and phthalates on locally advanced cervical cancer treatment-preliminary results	2016
10068	X. Y. Wang	Therapeutic Potential of Delivering Arsenic Trioxide into HPV-Infected Cervical Cancer Cells Using Liposomal Nanotechnology	2016
10069	M. D. Alvarez	Geochemical occurrence of arsenic, vanadium and fluoride in groundwater of Patagonia, Argentina: Sources and mobilization processes	2019
10070	A. M. Plumhoff	Thermodynamic properties, crystal structure and phase relations of pushcharovskite Cu(AsO3OH) (H2O) center dot 0.5H(2)O , geminite Cu(AsO3OH)(H2O) and liroconite Cu2Al(AsO4)(OH)(4) center dot 4H(2)O	2020
10071	D. Tardani	Copper-arsenic decoupling in an active geothermal system: A link between pyrite and fluid composition	2017
10072	H. Long	Recovery of alkali, selenium and arsenic from antimony smelting arsenic-alkali residue	2020
10073	E. Chendamarai	Comparison of Newly Diagnosed and Relapsed Patients with Acute Promyelocytic Leukemia Treated with Arsenic Trioxide: Insight into Mechanisms of Resistance	2015
10074	H. C. Lin	Charcoals Evaluation Regarding the Effect of Preservative-Treated Woods Leached Metal Loss on Water-Based Environment	2016
10075	R. H. Yu	The growth-inhibitory and apoptosis-inducing effect of deferoxamine combined with arsenic trioxide on HL-60 xenografts in nude mice	2014
10076	V. N. Yakovenchuk	Epifanovite, NaCaCu5(PO4)(4) AsO2(OH)(2) center dot 7H(2)O: a New Mineral from the Kester Deposit, Sakha (Yakutia) Republic, Russia	2018
10077	L. J. Huang	Effects of sulfur application on cadmium accumulation in brown rice under wheat-rice rotation	2021
10078	T. Abbas	Iron turning waste media for treating Endosulfan and Heptachlor contaminated water	2019

10079	J. N. Caplette	Antimony release and volatilization from rice paddy soils: Field and microcosm study	2022
10080	A. A. I. Khalil	Synthesis and characterisation of a low-cost sensor based on polymer nanocomposite for monitoring the level of pollution in different zones and its cross-validation using UV-LIPS technique	2022
10081	D. Sapsford	Conversion of coal mine drainage ochre to water treatment reagent: Production, characterisation and application for P and Zn removal	2015
10082	C. H. Miao	Combined effects of ferrihydrite coating and ionic type on the transport of compost-derived dissolved organic matter in saturated porous media	2022
10083	A. M. Almalki	Use of Spilopelia senegalensis as a Biomonitor of Heavy Metal Contamination from Mining Activities in Riyadh (Saudi Arabia)	2019
10084	E. A. A. Wahab	Basicity, Electronegativity, Optical Parameters and Radiation Attenuation Characteristics of P2O5-As2O3-PbO Glasses Doped Vanadium Ions	
10085	Q. L. Chen	Sb2O3 functionalized Plasmon, photoluminescence and Faraday rotation in glass	2020
10086	W. Zhang	Inducing cell cycle arrest and apoptosis by dimercaptosuccinic acid modified Fe3O4 magnetic nanoparticles combined with nontoxic concentration of bortezomib and gambogic acid in RPMI-8226 cells	2015
10087	S. G. Li	The Relationship between Liver Dysfunction and Arsenic Methylation in Mice	2015
10088	F. Zhou	Selenium bioaccessibility in native seleniferous soil and associated plants: Comparison between in vitro assays and chemical extraction methods	2021
10089	W. Wu	Excited states of a phosphorus pair in silicon: Combining valley-orbital interaction and electron-electron interactions	2021
10090	E. Disli	Hydrogeochemical investigation of an epithermal mineralization bearing basin using multivariate statistical techniques and isotopic evidence of groundwater: Kestanelik Sub-Basin, Lapseki, Turkey	2020
10091	T. P. Nguyen	Harmful and nutrient elements in paddy soils and their transfer into rice grains (Oryza sativa) along two river systems in northern and central Vietnam	2020
10092	S. de Mey	Antidiabetic Biguanides Radiosensitize Hypoxic Colorectal Cancer Cells Through a Decrease in Oxygen Consumption	2018
10093	D. K. Tripathi	Acquisition and Homeostasis of Iron in Higher Plants and Their Probable Role in Abiotic Stress Tolerance	2018
10094	G. Liu	Hotspots for selected metal elements and microbes accumulation and the corresponding water quality deterioration potential in an unchlorinated drinking water distribution system	2017
10095	D. T. Shi	Iron amended gravity-driven membrane (IGDM) system for heavy-metal-containing groundwater treatment	2022
10096	C. M. van Genuchten	Structure of Fe(III) precipitates generated by the electrolytic dissolution of Fe(0) in the presence of groundwater ions	2014

10097	A. Navas-Acien	Early Cardiovascular Risk in E-cigarette Users: the Potential Role of Metals	2020
10098	S. H. Wen	CdSe/ZnS quantum dots coated with carboxy-PEG and modified with the terbium(III) complex of guanosine 5-monophosphate as a fluorescent nanoprobe for ratiometric determination of arsenate via its inhibition of acid phosphatase activity	2019
10099	A. Wirth	Polysulfonylamine .75. Complexation of a dimeric diorganotin(IV)-amide hydroxide with neutral ligands: Synthesis of Me(2)Sn(mu-OH){N(SO(2)Me)(2)}(L) (2) (L=HMPA, Ph(3)PO, Ph(3)AsO) and crystal structures of complexes with L=HMPA and Ph(3)PO	1996
10100	M. Kim	Factors influencing As(V) stabilization in the mine soils amended with iron-rich materials	2018
10101	A. D. Jew	Mercury Interaction with the Fine Fraction of Coal-Combustion Fly Ash in a Simulated Coal Power Plant Flue Gas Stream	2015
10102	H. H. Du	Ferrihydrite-organo composites are a suitable analog for predicting Cd(II)- As(V) coexistence behaviors at the soil solid-liquid interfaces*	2021
10103	X. W. Ou	Surface defects enhance the adsorption affinity and selectivity of Mg(OH)(2) towards As(v) and Cr(vi) oxyanions: a combined theoretical and experimental study	2018
10104	D. Gnanasangeetha	Modelling of As3+ adsorption from aqueous solution using Azadirachta indica by artificial neural network	2015
10105	M. O. Ojemaye	Uptake of Zn2+ and As3+ from Wastewater by Adsorption onto Imine Functionalized Magnetic Nanoparticles	2018
10106	A. M. R. Neiva	Contaminated water, stream sediments and soils close to the abandoned Pinhal do Souto uranium mine, central Portugal	2014
10107	P. B. Hamilton	Late-Holocene diatom community response to climate driven chemical changes in a small, subarctic lake, Northwest Territories, Canada	2021
10108	J. Majzlan	SYNTHESIS AND STABILITY OF SOME MEMBERS OF THE PHARMACOSIDERITE GROUP, AFe(4)(OH)(4)(AsO4)(3)center dot nH(2)O (A = K, Na, 0.5Ba, 0.5Sr)	2019
10109	A. Wawra	Investigations of microbial degradation of polycyclic aromatic hydrocarbons based on C-13-labeled phenanthrene in a soil co-contaminated with trace elements using a plant assisted approach	2018
10110	A. M. Almalki	Effects of Mining Activities on Gerbillus nanus in Saudi Arabia: A Biochemical and Histological Study	2019
10111	G. Ratie	Iron speciation at the riverbank surface in wetland and potential impact on the mobility of trace metals	2019
10112	Y. Zhou	Cell Cycle-Dependent Uptake and Cytotoxicity of Arsenic-Based Drugs in Single Leukemia Cells	2018
10113	J. J. Liu	Alterations of antioxidant indexes and inflammatory cytokine expression aggravated hepatocellular apoptosis through mitochondria, and death receptor-dependent pathways in Gallus gallus exposed to arsenic and copper	2018
10114	X. Wu	Health risk assessment of arsenic in Realgar and NiuHuangJieDu Tablets based on pharmacokinetic study	2018

10115	D. C. Paz-Gomez	Copper recovery from sludges generated in the electrolyte treatment plant of a pyrometallurgical complex	2021
10116	S. Ribeiro	Genomic Abnormalities as Biomarkers and Therapeutic Targets in Acute Myeloid Leukemia	2021
10117	L. Zhang	Arsenic sulfide, the main component of realgar, a traditional Chinese medicine, induces apoptosis of gastric cancer cells in vitro and in vivo	2015
10118	M. Pastorek	Realgar (As4S4) nanopartides and arsenic trioxide (As2O3) induced autophagy and apoptosis in human melanoma cells in vitro	2014
10119	G. Q. Wu	Coal fly ash activated by NaOH roasting: Rare earth elements recovery and harmful trace elements migration	2022
10120	Y. X. Zhao	Antitumor effects of arsenic disulfide on the viability, migratory ability, apoptosis and autophagy of breast cancer cells	2019
10121	A. Djeridi	SYNTHESIS, CHARACTERIZATION AND THERMAL BEHAVIOR ASSESSMENT OF AN IRON PHOSPHATE GLASS DEDICATED FOR NUCLEAR WASTE CONFINEMENT	2017
10122	F. Y. Zhang	Determination of Palladium in Spent Automobile Exhaust Catalysts by Bismuth Fire Assay Preconcentration	2020
10123	L. P. Wu	Inorganic arsenic promotes apoptosis of human immortal keratinocytes through the TGF-beta 1/ERK signaling pathway	2022
10124	N. Al-Taweel	Cisplatin (CDDP) triggers cell death of MCF-7 cells following disruption of intracellular calcium (Ca2+ (i)) homeostasis	2014
10125	Z. D. Yuan	Characterization of Fe-5(AsO3)(3)Cl-2(OH)(4)center dot 5H(2)O, a new ferric arsenite hydroxychloride precipitated from FeCl3-As2O3-HCl solutions relevant to arsenic immobilization	2020
10126	X. Y. Zhang	ANXA1 silencing increases the sensitivity of cancer cells to low-concentration arsenic trioxide treatment by inhibiting ERK MAPK activation	2015
10127	D. D. Gregory	Trace Element Content of Sedimentary Pyrite in Black Shales	2015
10128	R. J. Bakker	Formation conditions of leucogranite dykes and aplite-pegmatite dykes in the eastern Mt. Capanne plutonic complex (Elba, Italy): fluid inclusion studies in quartz, tourmaline, andalusite and plagioclase	2016
10129	J. N. Caplette	BLACK ROCK-COATINGS IN TRAIL, BRITISH COLUMBIA, CANADA: RECORDS OF PAST EMISSIONS OF LEAD, ZINC, ANTIMONY, ARSENIC, TELLURIUM, TIN, SELENIUM, SILVER, BISMUTH, AND INDIUM-BEARING ATMOSPHERIC	2018
10130	R. Cela-Dablanca	Cu and As(V) Adsorption and Desorption on/from Different Soils and Bio-Adsorbents	2022
10131	D. Dwivedi	From legacy contamination to watershed systems science: a review of scientific insights and technologies developed through DOE-supported research in water and energy security	2022
10132	A. C. Senn	Composition and structure of Fe(III)-precipitates formed by Fe(II) oxidation in water at near-neutral pH: Interdependent effects of phosphate, silicate and Ca	2015

10133	T. Hua	Competitive adsorption and desorption of arsenate, vanadate, and molybdate onto the low-cost adsorbent materials alum water treatment sludge and bauxite	2018
10134	L. M. Santangelo	Occurrence and sources of lead in private wells, Sturbridge, Massachusetts	2022
10135	R. A. French	The structure and transformation of the nanomineral schwertmannite: a synthetic analog representative of field samples	2014
10136	B. Kribek	Contamination of soil and grass in the Tsumeb smelter area, Namibia: Modeling of contaminants dispersion and ground geochemical verification	2016
10137	D. Zhang	Effects of Phosphate, Red Mud, and Biochar on As, Cd, and Cu Immobilization and Enzymatic Activity in a Co-Contaminated Soil	2022
10138	S. J. Cobbina	A multivariate assessment of innate immune-related gene expressions due to exposure to low concentration individual and mixtures of four kinds of heavy metals on zebrafish (Danio rerio) embryos	2015
10139	D. Evangelou	Robust Al3+ MOF with Selective As(V) Sorption and Efficient Luminescence Sensing Properties toward Cr(VI)	2022
10140	A. J. Desbarats	Arsenic mobility in mildly alkaline drainage from an orogenic lode gold deposit, Bralorne mine, British Columbia	2015
10141	L. J. Bu	Enhanced Cathodic Preconcentration of As(0) at Au and Pt Electrodes for Anodic Stripping Voltammetry Analysis of As(III) and As(V)	2015
10142	P. T. Tho	Enhanced simultaneous adsorption of As(iii), Cd(ii), Pb(ii) and Cr(vi) ions from aqueous solution using cassava root husk-derived biochar loaded with ZnO nanoparticles	2021
10143	Q. Wu	Origin of the Qiyugou gold deposit in the southern margin of the North China Craton: Insights from trace elements of pyrite and mineralogy of Bi-minerals	2021
10144	E. Kruisdijk	Reactive transport modelling of push-pull tests: A versatile approach to quantify aquifer reactivity	2021
10145	Z. Z. Dong	Chemical oxidation of arsenopyrite using a novel oxidant-Chlorine dioxide	2019
10146	D. Ingerle	JGIXA - A software package for the calculation and fitting of grazing incidence X-ray fluorescence and X-ray reflectivity data for the characterization of nanometer-layers and ultra-shallow-implants	2016
10147	K. Tirez	Speciation of inorganic arsenic in particulate matter by combining HPLC/ICP-MS and XANES analyses	2015
10148	S. M. Prabhu	A Mechanistic Approach for the Synthesis of Carboxylate-Rich Carbonaceous Biomass-Doped Lanthanum-Oxalate Nanocomplex for Arsenate Adsorption	2018
10149	L. Zhang	Arsenic sulfide inhibits cell migration and invasion of gastric cancer in vitro and in vivo	2015
10150	G. C. Song	Study on the Transformation of Arsenic and Lead in Pyrite During Thermal Conversion	2019

10151	Z. H. Cai	Use of a Mouse Model and Human Umbilical Vein Endothelial Cells to Investigate the Effect of Arsenic Exposure on Vascular Endothelial Function and the Associated Role of Calpains	2019
10152	F. Ledderboge	Dimorphic cerium(III) oxoarsenate(III) Ce AsO3	2014
10153	Q. Zhao	Synergistic effects of arsenic trioxide combined with Salmonella typhimurium in treating the advanced hepatocellular carcinoma in rat models	2021
10154	I. Campostrini	Russoite, NH4ClAs23+O3(H2O)(0.5), a new phylloarsenite mineral from Solfatara Di Pozzuoli, Napoli, Italy	2019
10155	H. J. Zou	Upregulation of CD54 and downregulation of HLA-ABC contribute to the novel enhancement of the susceptibility of HL-60 cells to NK cell-mediated cytolysis induced by ATRA plus VPA	2017
10156	O. N. Filimonova	The state of Au and As in pyrite studied by X-ray absorption spectroscopy of natural minerals and synthetic phases	2020
10157	K. Kawamoto	The role of nanoscale aggregation of ferrihydrite and amorphous silica in the natural attenuation of contaminant metals at mill tailings sites	2021
10158	E. Marcoux	age et genese de la coupole granitique a metaux rares (Sn, Li, Nb-Ta, W) de Montebras (Creuse, Massif central francais)	2021
10159	M. Khorasanipour	Environmental mineralogy of Cu-porphyry mine tailings, a case study of semi-arid climate conditions, Sarcheshmeh mine, SE Iran	2015
10160	Y. X. Liu	A Critical Review on Removal of Gaseous Pollutants Using Sulfate Radical-based Advanced Oxidation Technologies	2021
10161	H. Thouin	Microcosm-scale biogeochemical stabilization of Pb, As, Ba and Zn in mine tailings amended with manure and ochre	2019
10162	E. S. Santos	Potential of Tamarix africana and other halophyte species for phytostabilisation of contaminated salt marsh soils	2017
10163	J. Sejkora	Honzaite, (Ni,Co)(2)(AsO3OH)(2)(H2O)(5), a new Ni-dominant analogue of burgessite, from Jachymov, Czech Republic	2018
10164	A. E. Williams-Jones	Constraints on the Genesis of Cobalt Deposits: Part I. Theoretical Considerations	2022
10165	J. Plasil	Hlousekite, (Ni,Co)Cu-4(AsO4)(2)(AsO3OH)(2)(H2O)(9), a new member of the lindackerite supergroup from Jachymov, Czech Republic	2014
10166	D. Holtstam	Brattforsite, Mn-19(AsO3)(12)Cl-2, a new arsenite mineral related to magnussonite, from Brattforsgruvan, Nordmark, Varmland, Sweden	2021
10167	Z. N. Li	Efficacy and Safety of the Arsenic Trioxide/Lipiodol Emulsion in the Transcatheter Arterial Chemoembolization Combined with Apatinib in the Treatment of Advanced Hepatocellular Carcinoma	2021
10168	L. Wang	Restoration of CCAAT enhancer binding protein a P42 induces myeloid differentiation and overcomes all-trans retinoic acid resistance in human acute promyelocytic leukemia NB4-R1 cells	2015

10169	C. Biagioni	Bianchiniite, Ba-2(Ti4+V3+)(As2O5)(2)OF, a new diarsenite mineral from the Monte Arsiccio mine, Apuan Alps, Tuscany, Italy	2021
10170	M. S. Safarzadeh	Reaction of enargite (Cu3AsS4) in hot concentrated sulfuric acid under an inert atmosphere. Part II: High-quality enargite	2014
10171	Y. J. Kuo	Synergistic inhibition of leukemia WEHI-3 cell growth by arsenic trioxide and Hedyotis diffusa Willd extract in vitro and in vivo	2017
10172	A. R. Kampf	Torrecillasite, Na(As, Sb)(4)3+O6Cl, a new mineral from the Torrecillas mine, Iquique Province, Chile: description and crystal structure	2014
10173	J. Chen	Mineralogy and geochemistry of Late Permian coals from the Donglin Coal Mine in the Nantong coalfield in Chongqing, southwestern China	2015
10174	A. Carrillo-Chavez	Geochemistry and mineralogy of mine-waste material from a "skarn-type" deposit in central Mexico: Modeling geochemical controls of metals in the surface environment	2014
10175	Y. Y. Li	Could the rhizoplane biofilm of wetland plants lead to rhizospheric heavy metal precipitation and iron-sulfur cycle termination?	2019
10176	D. B. Wu	Preservation of co-occurring As, Sb and Se species in water samples with EDTA and acidification	2016
10177	K. H. K. Lee	Iron(III)-Oxo Cluster Chemistry with Dimethylarsinate Ligands: Structures, Magnetic Properties, and Computational Studies	2020
10178	S. L. Brown	MRI-Tracked Tumor Vascular Changes in the Hours after Single-Fraction Irradiation	2015
10179	C. Biagioni	Lead-antimony sulfosalts from Tuscany (Italy). XXII. Marcobaldiite, similar to Pb-12(Sb3As2Bi)(Sigma 6)S-21, a new member of the jordanite homologous series from the Pollone mine, Valdicastello Carducci	2018
10180	I. Lykova	CHIYOKOITE, Ca3Si(CO3) B(OH)(4) O(OH)(5)center dot 12H(2)O, A NEW ETTRINGITE-GROUP MINERAL FROM THE FUKA MINE, OKAYAMA PREFECTURE, JAPAN	2020
10181	E. M. Schilling	The Human Cytomegalovirus IE1 Protein Antagonizes PML Nuclear Body-Mediated Intrinsic Immunity via the Inhibition of PML De Novo SUMOylation	2017
10182	J. M. Gonzalez-Jimenez	Precious metals in magmatic Fe-Ni-Cu sulfides from the Potosi chromitite deposit, eastern Cuba	2020
10183	J. F. Mei	Effects of human enclosure and farming activities on heavy metals in sediments/soils of the coastal reclamation areas in the Yangtze Estuary	
10184	H. Kaasalainen	The geochemistry of trace elements in geothermal fluids, Iceland	2015
10185	M. A. Cooper	Wiklundite, ideally Pb-2(4)(Mn2+,Zn)(3)(Fe3+,Mn2+)(2)(Mn2+,Mg)(19)(As3+O3)(2)(Si,As5+)O-4 (6)(OH)(18)Cl-6, a new mineral from Langban, Filipstad, Varmland, Sweden: Description and crystal structure	2017
10186	V. N. Yakovenchuk	Batagayite, CaZn2(Zn,Cu)(6)(PO4)(4)(PO3OH)(3)center dot 12H(2)O, a new phosphate mineral from KA << ster tin deposit (Yakutia, Russia): occurrence and crystal structure	2018

10187	F. Camara	Arsenic-bearing new mineral species from Valletta mine, Maira Valley, Piedmont, Italy: I. Grandaite, Sr2Al(AsO4)(2)(OH), description and crystal structure	2014
10188	J. A. Steadman	Paragenesis and composition of ore minerals in the Randalls BIF-hosted gold deposits, Yilgarn Craton, Western Australia: Implications for the timing of deposit formation and constraints on gold sources	2014
10189	L. J. Wang	Dissolution and Precipitation Dynamics at Environmental Mineral Interfaces Imaged by In Situ Atomic Force Microscopy	2020
10190	Z. D. Zhang	Cross-sectional study: Relationship between serum trace elements and hypertension	2022
10191	Y. Cen	Spectral Analysis of Main Mineral Pigments in Thangka	2019
10192	O. M. R. Campbell	Getting the basic rights - the role of water, sanitation and hygiene in maternal and reproductive health: a conceptual framework	2015
10193	Meili Hu	Experimental Study on the Inhibition of Endometrial Cancer Growth by Arsenic Trioxide In Vivo and In Vitro	2009
10194	Na Li	The Effect of Arsenic Trioxide on Medroxyprogesterone Acetate-Resistant Endometrial Cancer Cells	2017
10195	Wenjian Sun	Effects of Drinking Water Arsenic Exposure During Pregnancy and Lactation on Neurotransmitter Metabolism Enzymes in Offspring Rat Brain Tissues	2007
10196	Feng Zhang	Study on the Reproductive Toxicity of Arsenic Trioxide (As_2O_3) in KM Mice	2008
10197	Peng Liu	Effects of Chronic Arsenic Exposure on DNA Damage in Fetal Rat Brain Tissue and Memory Function in Offspring	2007
10198	Yan Xia	Study on the Synergistic Inhibition of HepG2 Hepatocellular Carcinoma Cells by Medroxyprogesterone Acetate Combined with Arsenic Trioxide In Vivo and In Vitro	2018
10199	Yuan Kong	Experimental Study on the Protective Effect of Taurine Against Autophagic Injury Induced by Arsenic Trioxide in Offspring Rat Pancreas	2017
10200	Dongying Qu	Effects of Prenatal and Postnatal Arsenic Exposure on Brain Development in Rats	2015
10201	Xuerong Han	Effects of Prenatal and Postnatal Arsenic Exposure on Sema3a Expression in Offspring Rat Brain Tissue	2012
10202	Yina Sun	Effects of Different Degrees of Iodine Deficiency During Pregnancy on Fetal Iodine Metabolism and Thyroid Function in Rats	2009
10203	Jie Bai	Experimental Study on the Protective Effect of Taurine Against Arsenic Trioxide-Induced Autophagic Injury in Offspring Rat Liver via the PPARγ Pathway	2016
10204	Guang Hao	Study on the Gonadotoxicity and Mechanism of Arsenic Trioxide in Female Mice	2009

10205	Yajuan Xia	Study on the Estrogenic Effects and Reproductive Developmental Toxicity of Arsenic Trioxide	2008
10206	Xiao Qiao	Study on Thyroid Function and Autoantibody Changes in Pregnant Rats	2015
10207	Chengmin Deng	Correlation Analysis of Maternal Urinary Arsenic Levels and Gestational Diabetes Mellitus	2020
10208	Shang Zhou	Effects of Prenatal and Postnatal Arsenic Exposure on the Development of Offspring Heart, Liver, Spleen, and Kidney in Rats	2015
10209	Jiaming Tang	Study on the Distribution and Excretion Pathways of Arsenic in Organs of Pregnant Rats After Oral Administration of Realgar	2012
10210	Xiaolin Tian	Effects of Fluoride and Arsenic Alone and in Combination on Nephrotoxicity and Autophagy in Offspring Rats	2022
10211	Xiumei Zhao	Intervention Study on the Effects of Folic Acid on Arsenic-Induced Congenital Heart Defects in Fetuses During Periconception	2017
10212	Jingting Liu	Prospective Cohort Study on the Correlation Between Maternal Heavy Metal and Trace Element Levels and Congenital Heart Disease in Offspring	2022
10213	Fang Chen	Survey on Iodine Nutrition Levels of Pregnant Women in Wuhan from 2016 to 2020	2022
10214	Yaping Zhang	Correlation Study Between Maternal Mid-Pregnancy Nickel and Arsenic Levels and Cardiovascular Disease Biomarkers in Umbilical Cord Blood	2021
10215	Changchun Hou	Survey on Iodine Intake and Iodine Nutrition Status of Pregnant Women in Different Water Iodine Regions in Tianjin	2022
10216	Ruiqiang Dong	Effects of Different Iodine Nutrition Levels on Maternal Iodine Metabolism During Pregnancy in Rats	2012
10217	Zhiquan He	Effects of Preconception Arsenic Exposure on Testicular Development and vasa Gene Expression in F1 Generation Newborn Rats	2016
10218	Yuan Lin	Study on Congenital Heart Abnormalities Induced by Preconception and Prenatal Arsenic Exposure in Fetuses	2016
10219	Qi Lu	Study on Lead, Cadmium, Arsenic, Mercury, Manganese, and Selenium Levels in Maternal and Neonatal Cord Blood and Their Placental Permeability	2020
10220	Xiuli Ding	Correlation Study on Maternal Blood Nickel and Arsenic Levels in Mid-Pregnancy and Fetal Growth and Development	2021
10221	Hong Hu	Study on the Transfer of Arsenic From Mothers to Newborns and Its Relationship With Pregnancy Complications	2020
10222	Huanhuan Guo	Mechanistic Study on the Effects of Preconception and Prenatal Drinking Water Arsenic Exposure on Fetal Heart Development	2016

10223	Lingzi Zhuang	Study on the Effects and Mechanisms of Preconception and Prenatal Arsenic Exposure on Fetal Heart Development	2016
10224	Lanlan Feng	Relationship Between Maternal and Cord Blood Arsenic Levels and Hypertensive Disorders During Pregnancy	2019
10225	Chuanling Zhang	Preimplantation Embryo Developmental Arrest Induced by Preconception Arsenic Exposure in Immature Female Mice	2010
10226	Jiaqi Wang	Correlation Analysis of Maternal Blood Lead and Arsenic Levels and Occupational Characteristics in Yongkang City	2021
10227	Mengchao Sun	Study on Thyroid Function and Autoantibody Changes During Pregnancy and Lactation in Rats	2016
10228	Feifei Qiao	Analysis of Iodine Deficiency Monitoring in 8-10 Year-Old Children and Pregnant Women in Yantai City, Shandong Province in 2019	2021
10229	Lihui Jia	Analysis of Iodine Nutrition Levels in Children and Pregnant Women in Non-High Iodine Areas of Hebei Province	2021
10230	Suqin Liu	Bioinformatics Study on the Effects of Environmental Chemical Exposure on Gestational Diabetes Mellitus	2021
10231	Cuicui Tian	Analysis of Pregnant Women's Knowledge and Behavior Regarding Iodine Deficiency Disorders Prevention in Iodine-Deficient Areas of Anhui Province	2021
10232	Huixuan Su	Monitoring and Analysis of Urinary Iodine Levels in Pregnant Women at Different Gestational Stages	2010
10233	Huixuan Su	Changes in Thyroid Hormones in Pregnant Women With Different Iodine Nutrition Levels and at Different Gestational Stages	2010
10234	Not Found	Interaction Between Genetic and Environmental Factors During Pregnancy and Maternal Arsenic Methylation Efficiency	2019
10235	Jingjing Shi	Analysis of Iodine Nutrition Levels in Pregnant Women in Yangzhou City from 2018 to 2020	2021
10236	Yanni Zheng	Study on Iodine Nutrition Levels of Pregnant Women in Xigu District, Lanzhou City, in 2018	2021
10237	Jinhuan Wang	Analysis of Urinary Iodine Levels in Pregnant Women in Coastal Areas	2019
10238	Liyan Huang	Association Between Periconceptional Air Pollution Exposure and Congenital Hypothyroidism in Offspring: A Study Based on 30 Chinese Provinces	2020
10239	Li Zhang	Survey on Iodine Nutrition Levels of Pregnant Women in Lanzhou City in 2019	2022
10240	Huixuan Su	Study on Iodine Nutrition Status of Pregnant Women at Different Gestational Stages	2010

10241	Xueling Lu	Correlation Between Thyroid Function and Iodine Nutrition Status in Han Pregnant Women in Urumqi City	2018
10242	Changchun Hou	Study on the Relationship Between Maternal Thyroid Function, Iodine Nutrition, and Autoantibodies in Pregnant Women	2021
10243	Yongtao Zhou	Analysis of the Significance of Urinary Iodine Testing in Pregnancy Health Guidance	2021
10244	Zhiquan He	Effects of Preconception Arsenic Exposure on the Developmental Potential of Fetal Spermatogonia in Rats	2016
10245	Jun Xiao	Effects of Chronic Arsenic Exposure on Learning and Memory Function in Rats	2006
10246	Not Found	Study on the Effects of Chronic Arsenic Exposure on DNA in the Hippocampus of Offspring Rats Using the Single-Cell Gel Electrophoresis Method	2005
10247	Ruihua Li	Effects of Embryonic Arsenic Exposure on Neuroinflammatory Responses in the Hippocampus of Adult Mice	2019
10248	Yaping Zhang	Correlation Between Prenatal Nickel and Arsenic Exposure and Oxidative Stress Markers in Umbilical Cord Blood of Newborns	2020
10249	Xue Liang	Effects of Maternal Iodine Excess During Pregnancy and Lactation on Offspring Rats and the Role of Adjusting to Adequate Iodine or Supplementing 1,25(OH)2D3	2018
10250	Yongfang Li	Effects of Early-Life Drinking Water Arsenic Exposure on Mammary Gland Development in Female Mice	2020
10251	Jingping Hu	Monitoring Results of Iodine Nutrition Levels in Pregnant Women in Xigu District, Lanzhou City	2020
10252	Pei Wang	Analysis of Lead, Arsenic, Cadmium, Manganese, and Zinc Levels in Pregnant Women and Fetuses and Their Influencing Factors	2008
10253	Wei Wei	Study on the Mechanism of Mild Iodine Deficiency During Pregnancy and Lactation Inducing Hypothyroxinemia and Causing Hippocampal Neurodevelopmental Damage and Long-Term Synaptic Plasticity Impairment in Offspring Rats	2013
10254	Zhijuan Yin	Analysis of Monitoring Results in High-Water-Iodine Areas of Hebei Province in 2018	2022
10255	Peng Liu	Discussion on Changes in Thyroid Function and Normal Urinary Iodine Reference Values in Pregnant Women	2015
10256	Yanjun Zhou	Case-Control Study on Urinary Arsenic Levels in Pregnant Women and Their Association With Gestational Diabetes	2017
10257	Yang Liu	Analysis of Pregnant Women's Iodine Nutrition Status in Henan Province After Implementation of the New Salt Iodization Standard in 2018	2020
10258	Zhaowen Chen	Establishing the Medical Reference Range for Early Pregnancy Thyroid Hormones After Adjusting Salt Iodine Content	2020

10259	Cuiping Zhang	Thyroid Function Analysis of Pregnant Women With Negative Thyroid Antibodies in High-Water-Iodine Areas	2010
10260	Xuhua Xi	Effects of Drinking Water Arsenic Exposure on Arsenic Metabolism in Offspring Rats	2010
10261	Ronghua Zhou	Relationship Between Iodine Nutrition in Different Pregnancy Stages and Neonatal Thyroid Function	2002
10262	Zhuxia Zhu	Effects of Arsenic on Energy Metabolism and Ultrastructural Damage in Rat Brain Cells	2009
10263	Yanhong Li	Distribution of Urinary Selenium and Arsenic in Pregnant Women Exposed to Drinking Water Arsenic	2006
10264	Yaqian Liu	Relationship Between Prenatal Arsenic Exposure, Atherosclerosis in Male Offspring, and ABCA1 Methylation	2019
10265	Mengyao Geng	Effects of Drinking Water Arsenic Exposure From Pregnancy to Maturity on SYP and PSD95 mRNA Expression in the Hippocampus of Offspring Mice	2020
10266	Wenbin Wu	Toxicity of Realgar (Traditional Chinese Medicine) on Pregnant Rats, Maternal Health, and Embryonic-Fetal Development	2009
10267	Xiaowen Wu	Analysis of Urinary Iodine Levels and Thyroid Function in Pregnant Women in Zhaoqing City	2019
10268	Chen Zhang	Effects of Fluoride-Arsenic Exposure on Male Rat Reproductive Function	2000
10269	Chen Zhang	Study on the Reproductive Developmental Toxicity of Fluoride-Arsenic Exposure in Rats	2000
10270	Ye Zhao	Analysis of Iodine Nutrition and Thyroid Function in Pregnant Women in Zhongshan City	2014
10271	Wenhui Ling	Analysis of Urinary Iodine Monitoring Results in Pregnant Women in Zhuhai City in 2013	2015
10272	Jianfang Sun	Survey on Urinary Iodine Levels in Pregnant Women in Zhangjiagang City	2015
10273	Zhe Mo	Investigation of Reference Ranges for Thyroid Hormones in Pregnant and Lactating Women in Zhejiang Province	2017
10274	Lin Feng	Study on Pregnancy Outcomes and Reproductive System Disease Biomarkers in Women From Arsenic-Contaminated Areas	2018
10275	Hushun Duan	Analysis of Pregnant Women's Iodine Nutrition Status Before and After Implementation of the New Iodized Salt Standard in Shanxi Province	2018
10276	Jun Wan	Effects of Low-Arsenic Drinking Water Exposure on Growth and Development of Offspring Rats	2019

10277	Hong Zhu	Analysis of Heavy Metal Exposure During Pregnancy and Its Effects on Maternal and Fetal Health	2010
10278	Hongyu Guo	Effects of Chronic Arsenic Exposure on Serum Estradiol and Progesterone Levels in Female Mice	2011
10279	Yulian Wang	Analysis of Urinary Iodine Monitoring Results in Pregnant Women at Different Gestational Stages	2012
10280	Lingxin Long	Analysis of Urinary Iodine Monitoring Results in 8-10-Year-Old School Children and Pregnant Women in Jinping County, Guizhou Province	2017
10281	Jianan Chen	Investigation of Pregnant Women's Iodine Nutrition Status in Zhangping City, Fujian Province, After Implementing the New Iodized Salt Standard	2015
10282	Zhuo Jia	Analysis of Iodine Nutrition Monitoring Results in Pregnant Women in Jinan City in 2018	2019
10283	YuanYuan Li	Case-Control Study on the Association Between Arsenic and Unexplained Recurrent Spontaneous Abortion	2019
10284	Xiaoling Ma	Analysis of Children's and Pregnant Women's Iodine Nutrition Status Before and After Salt Iodine Adjustment in Xinjiang Production and Construction Corps	2019
10285	Yinghong Yu	Study on Iodine Nutrition of Pregnant Women in Different Water Iodine Areas in Tianjin	2021
10286	Huijun Gang	Analysis of Urinary Iodine Monitoring Results in School-Age Children and Pregnant Women in Bincheng District, Binzhou City, in 2020	2021
10287	Shujun Liu	Analysis of Urinary Iodine Levels in Pregnant Women in Zhuhai City	2013
10288	Zhenxing Liu	Analysis of Urinary Iodine Levels in Pregnant Women in Coastal Areas	2003
10289	Yanting Ren	Thyroid Function Investigation of Pregnant and Lactating Women in Different Water Iodine Areas in Shanxi Province	2018
10290	YuanYuan Fan	Relationship Between Iodine Nutrition and Thyroid Function and Autoantibodies in Pregnant Women in Baotou City	2020
10291	Tao Peng	Role of miR-145 and PTEN/AKT/mTOR Pathway in Arsenic-Induced Miscarriage and Trophoblast Cell Damage in Rats	2022
10292	Jiaxin Li	Experimental Study on the Toxic Effects of Prenatal and Lactational Arsenic Poisoning on the Central Nervous System of Offspring	2018
10293	Xiangxin Tan	Analysis of Urinary Iodine Monitoring Results in Students and Pregnant Women in Duyun City in 2017	2018
10294	Lihui Jia	Analysis of Iodine Nutrition Status in Pregnant Women in Shijiazhuang City in 2002	2004

10295	Zushan Zhang	Effects of High Iodine Exposure From Pregnancy and Lactation to Adulthood on Learning, Memory, and Monoamine Neurotransmitter Concentration in Male Offspring Rats	2018
10296	Shufen Han	Study on Iodine Levels in Pregnant and Lactating Women After Iodine Supplementation in Iodine-Deficient Areas	2002
10297	Rui Qiao	Internal Quality Control of Laboratory Urinary Iodine Monitoring in Pregnant Women	2020
10298	Hongbo Li	Analysis of Iodine Nutrition and Thyroid Function in Pregnant and Lactating Women in Urban Areas of Wuwei City, Gansu Province	2013
10299	Yong Zeng	Analysis of Urinary Iodine Monitoring Results in Pregnant Women in Lu'an City From 2017 to 2019	2021
10300	Ruiwen Tao	Birth Cohort Study on the Effects of Prenatal Arsenic Exposure on the Developmental Behavior of 6-Month-Old Infants	2017
10301	Juan Li	Health Risk Assessment of Arsenic and Mercury in Edible Fungi in Jiangxi Province	2022
10302	Lifen Yang	Investigation of Iodine Nutrition and Thyroid Function in Pregnant and Lactating Women in Rural Areas of Jilin Province in 2009	2011
10303	Xiao Wang	Analysis of Iodine Nutrition in Pregnant Women in Jing'an District, Shanghai, From 2016 to 2018	2021
10304	Houming Wei	Iodine Nutrition and Metabolism in Pregnant, Lactating Women, and Infants	2002
10305	Hongbing Li	Analysis of Iodine Nutrition in Pregnant Women in Baoji City, Shaanxi Province, From 2017 to 2019	2021
10306	Aynur Tursun	Analysis of Urinary Iodine Monitoring Results in Children and Pregnant Women in Hoxot County and Related Prevention Strategies	2017
10307	Xiaofei Pan	Analysis of Iodine Nutrition Monitoring Results in 900 Pregnant and Lactating Women in Tonghua City	2007
10308	Gulina Tuerdi	Investigation of Iodine Nutrition in Pregnant and Lactating Women in Baicheng and Wushi Counties in Southern Xinjiang	2007
10309	Chengri Jin	Evaluation of Iodine Nutrition Status in Pregnant and Lactating Women in Jilin City	2007
10310	Wenbin Zhu	Study on the Relationship Between Urinary Iodine Levels in Pregnant Women and Neonatal TSH	2007
10311	Huanhuan Yang	Analysis of Iodine Nutrition Monitoring in Pregnant Women in Weinan City in 2013	2015
10312	Zengzhen Chi	Analysis of Iodine Nutrition Status of Pregnant Women in Huangdao District, Qingdao City	2015

10313	Chenchen Wang	Investigation on Iodine Nutrition of Pregnant Women in Early, Middle, and Late Pregnancy in Xinjiang	2021
10314	Mingyuan Fang	Analysis of Urinary Iodine Monitoring Results of Pregnant Women in Linhai, Zhejiang Province, From 2018 to 2020	2021
10315	Junqing Pei	Review of China's Salt Iodization Policy Based on Urinary Iodine Levels in Early Pregnancy	2003
10316	Xiaohong Liang	Investigation on Selenium and Iodine Nutrition of Pregnant Women and Their Newborns in Guangzhou City	2000
10317	Nanping Wu	Effects of Iodine Load in Pregnant Women on Neonatal Thyroid Function and Behavioral Neurodevelopment	1999
10318	Chong Zheng	Effects of Arsenic on Estrogen Receptor Genes and Their Downstream Cytokine Expression in Rats	2016
10319	Bian Hu	Analysis of Iodine Nutrition Levels in Pregnant Women and Infants in Hotan, Xinjiang	2005
10320	Yingying Qi	Effects of Arsenic Exposure During Pregnancy and Lactation on DNA Methyltransferase mRNA Expression in the Hippocampus of Offspring Mice	2019
10321	Rui Yan	Effects of Arsenic Exposure on Offspring Rats' Learning and Memory at Different Time Points	2009
10322	Xingjun Fan	Effects of Low-Dose Subchronic Arsenic Exposure on Male Rat Reproductive Function and Offspring	2012
10323	Chunrong Yang	Clinical Significance of Changes in Hemoglobin and Serum Ferritin in Pregnant Women With Mild Thalassemia	2016
10324	Yanan Wang	Analysis of Urinary Iodine Monitoring in Pregnant Women in Zhuhai	2018
10325	Shuqin Wang	Investigation on Iodine Deficiency Disease Prevention and Control in Zhoucun District in 2018	2021
10326	Li Jin	Study on the Association Between Urinary Arsenic Levels in Early Pregnancy and Gestational Diabetes Mellitus	2019
10327	Wei Wang	Investigation on Iodine Nutrition of Pregnant Women and Children in Xigu District, Lanzhou City, in 2017	2021
10328	Chao Yu	Analysis of Salt Iodine and Urinary Iodine Monitoring in Pregnant Women in Huai'an City From 2009 to 2015	2015
10329	Rongqin Wang	Effects of High-Arsenic Drinking Water on T Lymphocyte Subsets, Immunoglobulins, and Interleukin-2 Levels in Pregnant Women	2016
10330	Yilong Wei	Analysis of Urinary Iodine Monitoring Results of 8-10-Year-Old Children and Pregnant Women in Jinchengjiang District, Hechi City, From 2017 to 2019	2021

10331	Dongmei Li	Analysis of Iodine Nutrition Monitoring of Pregnant Women in Dongying City, Shandong Province, From 2018 to 2020	2021
10332	Guizhu Gao	Analysis of Iodine Nutrition Status of Pregnant Women After Nationwide Reduction of Salt Iodization Levels	2004
10333	Liangliang Huo	Analysis of Iodine Nutrition Levels of Pregnant Women Before and After Salt Iodine Adjustment in Hangzhou City	2016
10334	Haihong Zhang	Investigation on Iodine Nutrition of Pregnant Women in Rural Areas of Shijiazhuang City 17 Years After Salt Iodization	2016
10335	Juwei Wang	Investigation on Iodine Nutrition of Pregnant Women in Different Ecological Regions of Gansu Province	2016
10336	Hongyu Guo	Study on the Effects of Chronic Arsenic Exposure on Estrogen Effects and Gonadotoxicity in Female KM Mice	2010
10337	Mingxi Fang	Analysis of Iodine Nutrition and Neonatal TSH Levels in Pregnant Women in Pudong New Area, Shanghai	2012
10338	Huai Guan	Effects of Arsenic Exposure During Pregnancy and Lactation on Offspring Rats’ Central Nervous System Development	2012
10339	Liying Li	Relationship Between Arsenic Levels in Pregnant Women and Congenital Heart Disease in Fetuses	2017
10340	Aixia Zou	Study on the Relationship Between Arsenic Levels in Pregnant Women and Neonatal Congenital Heart Disease	2015
10341	Hong Zhou	Monitoring of Iodine Nutrition Status of Pregnant and Lactating Women in Baiyin City in 2011	2013
10342	Haoqing Lu	Monitoring of Iodine Nutrition Status of Pregnant and Lactating Women in a City in 2011	2013
10343	Tianqin Chu	Monitoring of Urinary Iodine in Pregnant Women and School-Age Children in Anshan City From 2011 to 2012	2013
10344	Shuhua Xi	Effects of Arsenic Exposure on Oxidative Damage and Ultrastructural Pathology in the Hippocampus of Offspring Rats	2008
10345	Xiaoen Wang	Study on the Significance of Urinary Iodine Monitoring for Pregnant Women's Health Management	2018
10346	Shuhua Xi	Effects of Drinking Water Arsenic Exposure on GAD and GABA-T mRNA Expression in the Hippocampus of Offspring Rats	2007
10347	Shuhua Xi	Effects of Arsenic Exposure on the Neurobehavioral and Learning Memory Function of Offspring Rats	2006
10348	Minghong Gu	Investigation on Iodine Nutrition of Pregnant Women in Qiannan Prefecture, Guizhou Province	2006

10349	Dongfeng Guo	Analysis of Urinary Iodine Monitoring in Pregnant Women in Qinyuan County in 2010	2011
10350	Banyin Ruan	Investigation on Iodine Nutrition of Pregnant Women in Lanzhou City in 2010	2014
10351	Mingzhu Yuan	Investigation on Iodine Nutrition of Pregnant and Lactating Women Before and After Iodine Reduction in Salt in Bengbu City	2017
10352	Jianzhuo Jia	Investigation on Iodine Nutrition of Pregnant Women and Their Newborns in Baoding City	2008
10353	Huan Wang	Effects of Drinking Water Arsenic Exposure From Pregnancy to Maturity on SYP and PSD95 mRNA Expression in the Hippocampus of Offspring Mice	2018
10354	Qianqian Gao	Preliminary Study on Iodine Nutrition and Thyroid Dysfunction in Early Pregnancy	2013
10355	Jie Tu	Investigation on Urinary Iodine Levels in Pregnant Women in Urumqi City	2004
10356	Wei Wei	Study on the Relationship Between Iodine Nutrition in Pregnant Women and Neonatal Thyroid Function in High-Iodine Areas	2012
10357	Caifeng Zhang	Analysis of Iodine Nutrition Monitoring in Pregnant Women in Lanzhou City in 2016	2018
10358	Qi Wang	Study on the Relationship Between Urinary Heavy Metals and Gestational Hypertension in Pregnant Women	2019
10359	Lanlan Feng	Prospective Cohort Study on the Effects of Prenatal Arsenic and Manganese Exposure on Autism-Like Behavior in Infants	2019
10360	Weiling Liu	Relationship Between Urinary Iodine, SOD, and Thyroid Hormones in Pregnant Women With Thyroid Disease and Its Impact on Pregnancy Outcomes	2016
10361	Haibing Ji	Study on Normal Reference Values of Trace Elements in Whole Blood of Pregnant Women in Hangzhou	2016
10362	Zhenming Lu	Investigation on Congenital Malformations in Arsenic-Poisoned Areas of Jilin Province	2008
10363	Weiqi Liu	Relationship Between Urinary Iodine and Superoxide Dismutase in Pregnant Women With Thyroid Disease and Pregnancy Outcomes	2016
10364	Guoying Yin	Study on the Reproductive Toxicity of Arsenic	2017
10365	Collaborative Group on Arsenic	Investigation on Reducing the Hazards of Arsenic Poisoning - Technical Report of the China-UNICEF Cooperation Project (2003-2004)	2006
10366	Guizhu Gao	Thyroid Function and Iodine Nutrition Status in Pregnant Women During Early Pregnancy	2005

10367	Qingxiong Wang	Investigation on Selenium and Iodine Nutrition in Pregnant Women in Guangzhou City	2005
10368	Xiaohong Liang	Study on Iodine Nutrition in Pregnant Women in Guangzhou City	2003
10369	Xiaoli Wu	Investigation on Iodine Nutrition in Rural Pregnant Women in Shangluo City, Shaanxi Province	2003
10370	Ronghua Zhou	Investigation on Thyroid Function in Pregnant Women With Sufficient Iodine Intake and Their Newborns	2002
10371	Zhifeng Zhang	Analysis of Iodine Nutrition in Pregnant and Lactating Women in Qinba Mountain Area	2001
10372	Huixuan Su	Effects of Different Iodine Intake Levels on Thyroid Function in Pregnant Women	2010
10373	Xiaohong Liang	Investigation on Iodine Nutrition of Pregnant Women and Their Newborns	2000
10374	Jingjing Zhang	Analysis of Urinary Iodine Monitoring in School-Age Children and Pregnant Women in Huaiyin District, Jinan City, in 2019	2020
10375	Hanwu Zhu	Analysis of Urinary Iodine Monitoring Results of Pregnant Women in Chenzhou, Hunan Province, From 2018 to 2019	2020
10376	Nanrong Xie	Investigation on Urinary Iodine Levels in Pregnant Women and Children in Yuxi Region in 2014	2016
10377	Liying Liu	Investigation on Maternal Iodine Nutrition in Pregnant Women in Huairou District, Beijing	2007
10378	Shaogui Liu	The Five Brothers of Traditional Chinese Medicine for Clearing Heat and Detoxifying	2021
10379	Deyu Zhu	Effects of Arsenic Trioxide on ER mRNA Expression in Prematurely Matured Female SD Rats and Protein Levels of VEGF, CyclinD1, and PR	2017
10380	Meifang Zhu	Investigation on Iodine Nutrition of Pregnant Women at Different Pregnancy Stages in Zhuhai City	2017
10381	Jing Zheng	Investigation on Iodine Nutrition in the Population of Wuwei City, Gansu Province, Before and After the Implementation of the New Salt Iodization Standard	2018
10382	Xia Zhao	Study on the Relationship Between Gestational Iodine Nutrition Status and Thyroid Function	2017
10383	Yulan Liu	Investigation on the Harm of Iodine Deficiency in Early Pregnancy	2009
10384	Huiping Wang	Analysis of Urinary Iodine Monitoring Results of 8-10-Year-Old Children and Pregnant Women in Kunqu District, Baotou City, in 2015	2015

10385	Qing Yang	Investigation on Iodine Nutrition in Pregnant Women in Linhai, Zhejiang Province, From 2016 to 2018	2020
10386	Jingjing Zhang	Study on the Exposure Levels and Influencing Factors of Nickel, Cobalt, Copper, Arsenic, and Selenium in Pregnant Women and Their Fetuses	2012
10387	Xiuping Zheng	Clinical Significance of Thyroid Autoantibody Detection During Pregnancy	2005
10388	Not Found	15 Harmful Factors That Pregnant Women Should Avoid	2004
10389	Huixuan Su	Detection and Significance of Urinary Iodine Levels in Pregnant Women in Mid and Late Pregnancy	2010
10390	Pinjiang Ma	Study on Iodine Nutrition of Pregnant Women in Iodine Deficiency Areas of Turpan Basin and Its Relationship with Fetal Growth and Development	2009
10391	Xiaosong Li	Study on Urinary Iodine Levels in Pregnant Women of Ethnic Minorities in Guizhou Province	2007
10392	Xiaoxue Wang	Relationship Between Iodine Nutrition, Thyroid Autoantibodies, and Thyroid Function During Pregnancy	2014
10393	Yan Zhou	Rapid Detection and Clinical Application of Urinary Iodine in Women	2014
10394	Qiaorou Zhang	Association Between Heavy Metal Exposure and Physical Development Indicators in School-age Children	2022
10395	Sihai Gao	Survey on Iodine Nutrition in Pregnant Women in Wenzhou City	2019
10396	Yanling Wang	Survey on Iodine Deficiency Disorders Prevention in Gansu Province (2014)	2017
10397	Hanwu Zhu	Survey and Analysis of Iodine Nutrition Levels in Key Populations in Chenzhou City, Hunan (2015)	2017
10398	Qiuying Zheng	Survey on Iodine Nutrition in Yanping District, Nanping City, Fujian Province (2014)	2017
10399	Jinjun Guo	Survey on Dietary Iodine Intake in Pregnant Women of Ethnic Minorities in Turpan City, Xinjiang	2008
10400	Haitao Zhang	Survey on Iodine Nutrition in Different Populations in Dali City, Yunnan After Salt Iodine Reduction	2019
10401	Qingzhen Jia	Survey on Iodine Nutrition and Thyroid Function in Different Populations After 20 Years of Universal Salt Iodization in Iodine Deficiency Areas of Shanxi Province	2019
10402	Xiaoqing Wu	Survey on Iodine Nutrition Levels in Six Different Populations in Urban and Rural Areas of Xiamen After Three Years of New Iodized Salt Standards	2019

10403	Huihui Miao	Comparative Analysis of Iodine Nutrition Before and After Adjusting Salt Iodine Content in Fujian Province	2019
10404	Yuehong Deng	Study on the Relationship Between Arsenic and Magnesium Levels in Pregnant Women and Congenital Heart Disease in Fetuses	2018
10405	Fengjuan Du	Survey on Iodine Nutrition Levels in Newly Married, Pregnant, and Lactating Women in Suzhou	2006
10406	Yanni Zheng	Survey on Iodine Nutrition in Pregnant Women and Children in Lanzhou City (2018)	2020
10407	Yan Song	Survey on Iodine Nutrition in Pregnant Women in Turpan, Xinjiang (2004-2005)	2006
10408	Zhiqun Chen	Correlation Between the Use of Cosmetics by Pregnant Women and Arsenic Levels in Their Newborns	2003
10409	Zhongchun Li	Analysis of Iodine Nutrition and Thyroid Function in Pregnant Women in Urban and Rural Areas of Mudanjiang	2010
10410	Yunzhi Luo	Relationship Between Iodine Nutrition Status and Thyroid Hormones in Pregnant Women in a Grade 3 Hospital in Xinjiang	2018
10411	Na Liang	Survey Results of Iodine Nutrition Levels in Key Populations in High Water Iodine Areas of Shandong Province (2018)	2020
10412	Hongying Wu	Survey on Iodine Nutrition in Key Populations in Hainan Province and Its Correlation with Water Iodine and Salt Iodine	2020
10413	Ying Yang	Survey on Iodine Nutrition in Pregnant Women in Hohhot City	2012
10414	Xiangming Xu	Monitoring and Analysis of Iodine Deficiency Disorders in Weinan During the "13th Five-Year Plan" Period	2021
10415	Yanling Wang	Evaluation of the Effect of Iodine Nutrition Intervention During the Key Period of Brain Development on the Intelligence Quotient of Infants	2013
10416	Xiaochun Zhao	Survey on High-risk Iodine Deficiency Disorders Monitoring in Wenzhou City, Zhejiang Province (2014)	2016
10417	Yue Li	Evaluation and Clinical Application of ICP-MS for Measuring 19 Trace Elements in Whole Blood	2020
10418	Xiaoqin Chen	Reproductive Toxicity of Sodium Arsenite in Male Mice	2009
10419	Minhong Pang	Survey on Iodine Nutrition in Pregnant Women in Zhangjiakou	2009
10420	Diqun Chen	Dynamic Analysis of Urinary Iodine Nutrition and the Rationality of Urine-Specific Gravity Correction in Key Populations in Fujian Province	2019

10421	Liying Li	Correlation Between Congenital Heart Disease in Fetuses and Chemical Element Levels in Pregnant Women's Hair	2017
10422	Huixuan Su	Discussion on Changes in Thyroid Hormones in Pregnant Women at Different Stages of Pregnancy	2010
10423	Liping Yu	Supplementation of Trace Elements in Pregnant Women	2001
10424	Wei Liu	Analysis of Iodine Nutrition in Pregnant Women in Shantou City	2016
10425	Yanling Zhang	Survey on Iodine Nutrition in Key Populations in Baofeng County, Henan (2005)	2007
10426	Shufen Han	Analysis of Iodine Nutrition Levels in Special Populations After Iodine Supplementation in Iodine Deficiency Areas	2003
10427	Yunhong Zhang	Discussion on the Catalytic Spectrophotometric Determination of Urinary Iodine by Ammonium Persulfate Digestion Method	2016
10428	Yingchun Yu	Survey on Urinary Iodine and Thyroid Function in Women at Different Pregnancy Stages in Zibo City	2015
10429	Jiani Wu	Monitoring and Analysis of Iodine Deficiency Disorders in Fujian Province (2011)	2013
10430	Shengchao Qin	Final Evaluation of Iodine Deficiency Disorders Control in Aba Prefecture, Sichuan Province During the "12th Five-Year Plan" (2014)	2018
10431	Minglong Li	Survey on Iodine Nutrition in Residents of Yunfu City, Guangdong Province (2009-2016)	2018
10432	Gaiqing Ma	Survey and Analysis of Iodine Nutrition in Rural Residents of Anyang City, Henan Province (2015)	2018
10433	Ping Chen	Comparative Analysis of Iodine Deficiency Disorders Monitoring Results in Xianyang, Shaanxi Province and Yinchuan, Ningxia After Salt Iodine Adjustment	2018
10434	Hang Sun	Research on the Selection and Establishment of Heavy Metals (Lead, Cadmium, Chromium, Mercury) in Plastics	2018
10435	Jianan Chen	Survey on Iodine Nutrition and Thyroid Function in Residents of Longyan City, Fujian Province	2012
10436	Yongqin Cao	Survey on Iodine Nutrition in Key Populations in Linxia Hui Autonomous Prefecture, Gansu Province (2006)	2009
10437	Xingyi Jin	Survey on Iodine Nutrition in Urban and Rural Pregnant Women in Hangzhou	2008
10438	Guang Hao	Effects of Chronic Arsenic Exposure on ER mRNA and PR mRNA Expression in Mammary Glands of Female Mice	2010

10439	Han Li	Urinary Metabolomics Study on Early Pregnancy Arsenic and Cadmium Exposure in Urban Populations	2016
10440	Xingyi Jin	Survey and Analysis of Urinary Iodine and Urinary Creatinine in Women at Different Pregnancy Stages	2008
10441	Xiaolan Jiang	Study on the Most Suitable Salt Iodine Content for Residents in Zhenjiang City, Jiangsu Province	2007
10442	Xiuli Ding	Canonical Correlation Analysis Between Cord Blood Heavy Metal Levels and Fetal Growth and Development	2020
10443	Yiqin Chen	Association Between Arsenic Exposure and Decreased Ovarian Reserve Function (DOR) and Its Effect on Steroid Hormone Synthesis in Ovarian Granulosa Cells	2021
10444	Wei Zhang	Effects of Mercury and Arsenic on the Occurrence of Polydactyly	2005
10445	Wei Huang	Iodine Nutrition and Pituitary-Thyroid Function in Early Pregnancy Women	2002
10446	Huixuan Su	Clinical Observation of Thyroid Dysfunction During Pregnancy	2010
10447	Aihua Guo	Analysis of Household Iodized Salt and Urinary Iodine Monitoring in Three Key Populations in Xicheng District, Beijing (2014)	2015
10448	Fengjiao Ding	Monitoring and Analysis of Urinary Iodine in Pregnant Women in Shihezi, Xinjiang	2015
10449	Yanrong Shi	Comparison of TNF-伪 and VEGF in Plasma Between Pregnant Women in Industrial and Non-Industrial Areas	2015
10450	Jianbin Liu	Monitoring and Analysis of Iodine Deficiency Disorders in Zhaoyang District, Yunnan (2009-2015)	2020
10451	Guiping Chen	Analysis of Iodine Deficiency and Influencing Factors in 810 Pregnant Women	2020
10452	Xiaohui Wang	Monitoring of Iodine Nutrition Levels in Pregnant Women in Pingliang (2018)	2019
10453	Yang Wang	Survey Results of Iodine Deficiency Disorders in Jiangsu Province (2014)	2017
10454	Yujun Shen	Survey of Iodine Nutrition in Key Populations in Xuyi County	2016
10455	Nanrong Xie	Evaluation of the Effectiveness of Universal Salt Iodization for Preventing Iodine Deficiency Disorders in Yongchuan District, Chongqing (2015)	2016
10456	Yuanyuan Yang	Survey on Iodine Nutrition in Key Populations in Yingkou, Liaoning (2014)	2016

10457	Shihong Guo	Monitoring and Analysis of Iodine Deficiency Disorders in Weinan (2015)	2016
10458	Laixiang Lin	Effects of High Iodine Intake on NIS mRNA Expression in the Mammary Gland and Placenta of Rats	2007
10459	Fang Yang	Methodological Evaluation of Automated Iodine Analyzers for Urinary Iodine Determination	2019
10460	Yuchuan Wang	Survey on Iodine Nutrition Status in Pregnant Working Women With Different Dietary Habits	2009
10461	Yuanyuan Zuo	Survey on Iodine Nutrition in Pregnant Women After Salt Iodine Adjustment in Hohhot	2014
10462	Fuyu Qiao	Analysis of Iodine Nutrition in Pregnant and Non-Pregnant Women in Xicheng District, Beijing	2013
10463	Weina Lai	Survey of Heavy Metals in Early Pregnancy Women's Urine	2020
10464	Li Chen	Association Between Arsenic Levels in Hair of Pregnant Women and Congenital Heart Disease in Fetuses in Fujian Province	2013
10465	Guoying Yin	Transgenerational Effects of Intrauterine Arsenic Exposure on Organ Weight and Organ Coefficients in Mice	2017
10466	Chengshan Wang	Survey on Iodine Nutrition in Key Populations in Liaoyang	2015
10467	Shihong Guo	Monitoring and Analysis of Iodine Deficiency Disorders in Weinan (2013-2015)	2015
10468	Linsen Xiong	Study on the Differential Expression of Estrogen Receptor 伪 in Type II Alveolar Epithelial Cells of Fetal Rats Exposed to Sodium Arsenite	2017
10469	Wenyan Ma	Effects of Arsenic and Fluoride Exposure on Spatial Learning, Memory, and Long-Term Potentiation in the CA1 Region of the Hippocampus in Offspring Rats	2018
10470	Jing Fang	Urinary Iodine Monitoring in Children and Pregnant Women in Tongxin County	2021
10471	Xiaoli Xu	Urinary Iodine Monitoring in Mothers and Infants in Jiaozuo City, Henan (2004)	2006
10472	Mengping Yang	Effects of arsenic exposure and estrogen receptor antagonist on erβ expression in aecII of male and female fetal rats	2018
10473	Gentao Yu	Urinary Iodine Monitoring in Key Populations in Pingdingshan (2017-2019)	2021
10474	Sheng Cui	Urinary Iodine Monitoring in Three Key Populations in Baodi District, Tianjin (2019)	2021

10475	Changchun Hou	Survey on Iodine Intake and Nutrition Status in Adults in Tianjin	2016
10476	Yongping Wang	Survey and Analysis of Iodine Deficiency Disorders in Shanxi (2014)	2016
10477	Huan Wan	Analysis of Urinary Iodine Levels in Pregnant Women After Salt Iodine Adjustment in Chengde	2016
10478	Demei Zhou	Urinary Iodine Monitoring in Women at Different Pregnancy Stages	2010
10479	Qin Qu	Urinary Iodine Monitoring in Key Populations in Bazhou District, Bazhong City	2014
10480	Guanjun Shangguan	Survey on Iodine Deficiency Disorders in Jiangxi (6th National Survey)	2014
10481	Lesheng Zhang	Survey on Iodine Nutrition in Residents of Yanggu County	2014
10482	Lihong Ruan	Determination of Urinary Iodine Levels in 426 Pregnant Women in Early Pregnancy in Jinan	2006
10483	Yang Zeng	Effects of fluoride and arsenic exposure on glutamate and γ-aminobutyric acid levels in the hippocampus and cerebral cortex of offspring rats	2014
10484	Haihong Zhang	Comparative Analysis of Iodine Nutrition in Pregnant and Non-Pregnant Women in Urban and Rural Areas of Shijiazhuang	2015
10485	Zhihui Chen	Report on the 7th Iodine Deficiency Disorders Surveillance in Fujian Province (2014)	2015
10486	Min Guo	Monitoring and Analysis of Iodine Deficiency Disorders in Tibet (2011)	2015
10487	Yaping Zhang	Experimental Study on the Rationality of Urinary Iodine Concentration Adjustment by Urine Specific Gravity	2015
10488	Yingdong Gao	Effects of Arsenic Trioxide Exposure on Testicular Development and Vasa Gene Expression in F1 Male Rats	2015
10489	Hong Lin	Study on Pregnancy Stress and Its Influencing Factors in Early Pregnancy Women	2019
10490	Tao Ye	Survey on Iodine Nutrition in Children Aged 0-6 in Tianjin	2003
10491	Lin Zhu	Monitoring and Analysis of Urinary Iodine in Pregnant Women in Iodine Deficiency Areas of Henan (2017)	2018
10492	Pengpeng Lan	Heavy Metal Pollution and Health Risk Assessment of Smelting Factory Residual Soil	2019

10493	Xiaoqing Wu	Survey on Salt Iodine and Iodine Nutrition in Key Populations in Xiamen, Fujian (2011)	2013
10494	Tian Yang	Interview with Chinese Academy of Engineering Academician Daopei Lu - The Guardians of Blood Connection	2004
10495	Jinliang Zhang	Metals and Women's Health	2004
10496	Xiaoling Ma	Survey on Iodine Deficiency Disorders in Xinjiang Production and Construction Corps (2015)	2018
10497	Chen Yu	Monitoring and Analysis of Iodine Deficiency Disorders in Anhui (2014)	2018
10498	Jianbin Cao	Survey on Iodine Nutrition in Different Populations in Harbin (2011)	2012
10499	Weimin Xu	Effects of Iodized Salt on Iodine Nutrition Levels in Urban and Rural Pregnant Women in Hangzhou	2013
10500	Jinbiao Wang	Survey on Iodine Nutrition in Key Populations in Iodine-Deficient Areas of Shandong	2011
10501	Zhongna Sang	Comparison of Thyroid Function Between Pregnant Women and Women of Childbearing Age in High Iodine Areas	2011
10502	Guihua Zhang	Analysis of Urinary Iodine Monitoring in Pregnant Women in Zhuzhou (2016)	2017
10503	Dan Du	Analysis of Iodine Nutrition Levels in Different Populations in Beijing (2016-2019)	2021
10504	Hongmei Wang	Survey Results of Key Populations in High-Risk Iodine Deficiency Areas of Hainan Province	2010
10505	Qingyi Liu	Teratogenic Effects of Arsenic Trioxide on Mice and Its Impact on Embryonic PAX3 and CX43 Gene Expression	2005
10506	Huanhuan Yang	Monitoring Analysis of Iodine Nutrition in Pregnant Women in Weinan (2013)	2015
10507	Peizhen Yang	Urinary Iodine Survey of Women of Childbearing Age in High-Risk Iodine Deficiency Areas in Qinghai (2007)	2009
10508	Fengrui Wang	Effects of Seafood on Urinary Iodine Levels in Women	2008
10509	Suzhen Zhu	Correlation Study Between Urinary Iodine Levels and Thyroid Function in Women at Different Pregnancy Stages	2010
10510	Xiujun Wang	Survey on Salt Iodine and Urinary Iodine in Qian'an City (2016)	2017

10511	Liyin Xuan	Analysis of Urinary Iodine Levels in Pregnant Women Three Years After Implementing the New Salt Iodization Standard in Chengde	2016
10512	Chong Zheng	Study on Arsenic-Induced Damage to the Female Rat Reproductive System	2016
10513	Yupeng Zhu	Effects of Fluoride-Arsenic Co-Exposure on Spatial Learning and Memory in Offspring Mice and the Expression of Phosphatidylinositol Kinase and Threonine Kinase Proteins in the Hippocampus and Cerebral Cortex	2016
10514	Deyu Zhu	Effects of Arsenic Exposure Before Sexual Maturity on Female SD Rat Reproductive Function	2016
10515	Weifang Miao	Effects of Fluoride and Arsenic Exposure on the Expression of Group I Metabotropic Glutamate Receptors in the Hippocampus of Male Offspring Mice	2016
10516	Xiaohui Su	Survey on Iodine Deficiency Disorders in Linxia Hui Autonomous Prefecture, Gansu	2005
10517	Yu Zhou	Survey on Iodine Nutrition Status in Pregnant and Lactating Women in Kashgar After Emergency Iodine Supplementation	2017
10518	Siyuan Peng	Risk Study of Gestational Diabetes Mellitus and Typical Environmental Pollutants	2015
10519	Jiansen Chen	Survey on the Prevention and Control of Iodine Deficiency Disorders in Longyan (2018)	2019
10520	Guihua Zeng	Longitudinal Monitoring Results of Iodine Deficiency Disorders in Loudi	2003
10521	Peng Zhang	Monitoring Results of Urinary Iodine in Children and Pregnant Women in Ninghe District (2018)	2020
10522	Guifang Lu	Cattle Gallbladder Detoxification Preparations Should Not Be Taken Casually	2019
10523	Chun Cui	Effects of Iodized Salt Concentration Adjustment on Iodine Nutrition in Residents of Wuhu	2015
10524	Chunling Jin	Clinical Application of Thalidomide in Hematology	2014
10525	Duan Shu	Survey on Iodine Nutrition in Children and Pregnant Women in Hanchuan (2015)	2016
10526	Mengmeng Shi	Preliminary study on sex differences in er伪 expression in lung tissue of offspring mice exposed to arsenic	2015
10527	Linbo Cai	Monitoring Results of Iodine Deficiency Disorders in a District of Tianjin (2019)	2021
10528	Jinxiang Kai	Analysis of Iodine Nutrition Status After Adjusting Iodized Salt Levels in Qianxinan	2016

10529	Shaoqing Li	Analysis of Iodine Nutrition Status in Pregnant Women With Hypothyroidism	2016
10530	Fangru Guo	Survey on Iodine Nutrition in Special Populations in Shijingshan District, Beijing (2011)	2012
10531	Dan Zhao	Monitoring of Urinary Iodine in 1000 Pregnant Women in Dalian (2015)	2016
10532	Haibing Ji	Investigation of Heavy Metal Concentrations in Whole Blood of Non-Occupationally Exposed Pregnant Women in Hangzhou and Its Influencing Factors	2016
10533	Yang Peng	Study on the Association Between Urinary Heavy Metals and Bisphenols in Pregnant Women and Fetal Growth	2018
10534	Haihong Zhang	Comparative Analysis of Iodine Nutrition in Pregnant and Non-Pregnant Women in Shijiazhuang	2012
10535	Mei Li	Study on the Optimal Salt Iodine Content for Residents in Pingluo County, Ningxia	2011
10536	Mikde	Survey on Iodine Nutrition in Special Populations in Three Counties of Mountainous Ningxia Before and After Oral Iodized Oil Supplementation	2011
10537	Zhicheng Zhang	Effects of Arsenic Exposure in Male Mice on Intrauterine Growth and Postnatal Neurobehavioral Development in Offspring	2020
10538	Wei Wei	Iodine Nutrition Status and Thyroid Function in Pregnant Women in Iodine-Sufficient Areas During Late Pregnancy	2011
10539	Zhiwei Guo	Effects of Chronic Arsenic Exposure on Endocrine Function in Female Rats	2011
10540	Jun Zhang	Analysis of Arsenic Speciation in Liver and Brain Tissues of Offspring Mice Exposed to Maternal Arsenic	2011
10541	Fengfeng Xu	Current Situation of Iodine Nutrition and Thyroid Nodules in Different Populations in Jiangbei District, Ningbo, Zhejiang	2016
10542	Weimin Xu	Survey on Iodine Nutrition in Women of Childbearing Age, Pregnant Women, and Lactating Mothers	2003
10543	Wei Wei	Survey on Iodine Nutrition in Early Pregnancy Women in Urumqi	2016
10544	Lingqin Zhao	Study on the Relationship Between Heavy Metals (Lead, Mercury, etc.) and Nervous System Malformations	2008
10545	Zhiyao Wang	Analysis of Iodine Nutrition in Key Populations in Severe Iodine Deficiency Areas of Xinjiang	2008
10546	Sufan Wang	Analysis of Iodine Nutrition in Key Populations in Benxi	2008

10547	Not Found	Medicine, Health	2001
10548	Pei Feng	Analysis of Urinary Iodine Levels in Pregnant Women in Shijiazhuang	2016
10549	Wangxuan Chen	Analysis of Iodine Nutrition Status in Pregnant Women in Futian District, Shenzhen	2016
10550	Zhihui Ma	Survey on Iodine Nutrition in 8-10-Year-Old Students and Pregnant Women in Shijiazhuang (2015)	2015
10551	Denghua Li	Analysis of Monitoring Results After Achieving the Goal of Eliminating Iodine Deficiency Disorders in Chenzhou	2003
10552	Changfang Dai	Survey on Iodine Nutrition Levels in Key Populations in Lechang City After Adjusting Iodized Salt Concentration	2002
10553	Siyu Wu	Study on the Effects of PM2.5 Fine Particulate Matter on Female Rat Fertility	2016
10554	Suzhen Zhu	Analysis of Urinary Iodine and Thyroid Function in Pregnant Women in Hangzhou	2008
10555	Zhe Mo	Analysis of Iodine Nutrition Levels and Thyroid Disease Prevalence in Populations After Iodized Salt Adjustment in Zhejiang Province	2015
10556	Yan Fu	Evaluation of Iodine Deficiency Disorder Monitoring Results in Hainan Province (2013)	2015
10557	Xuefei Zhao	Survey on Iodine Nutrition Levels and Thyroid Nodule Prevalence in Residents of Non-Iodized Salt Consumption Areas in Ningbo, Zhejiang	2015
10558	Chen Yu	Analysis of Iodine Deficiency Disorder Monitoring Results in Anhui Province (2011)	2015
10559	Huizhen Yu	Analysis of Iodine Deficiency Disorder Monitoring Results in High-Risk Areas in Qinghai Province (2013)	2015
10560	Hongwei Zhu	Screening Results of Congenital Neonatal Hypothyroidism in Baoji, Shaanxi (2008-2013)	2015
10561	Yanmin Mao	Survey Report on Iodine Deficiency Disorders in Lianyungang, Jiangsu (2013)	2015
10562	Yanling Wang	Comparative Study on Iodine Nutrition and Thyroid Function in Urban and Rural Populations in Wuwei, Gansu	2015
10563	Xiaohui Meng	Monitoring Analysis of Iodine Nutrition Levels in Residents of Liubei District, Liuzhou (2017)	2018
10564	Wei Zhang	Case-Control Study on the Relationship Between Trace Elements and Neural Tube Defects	2005

10565	Haihong Zhang	Analysis of Urinary Iodine Monitoring Results in Pregnant Women in Different Trimesters in Shijiazhuang	2015
10566	Xiong Liu	Toxicity Considerations When Using Red Ochre in Clinical Practice	2010
10567	Xiuyan Qin	Monitoring and Water Iodine Survey Results of Iodine Deficiency Disorders in Rongcheng (2017)	2019
10568	Yanghua Li	Analysis of Iodine Deficiency Disorders in Beijing (2011)	2014
10569	Ting Zhang	Analysis of Iodine Nutrition Levels and Influencing Factors in Special Needs Populations in Chongqing	2009
10570	Zhongyuan Kan	Monitoring Results of Iodine Deficiency Disorders in Liaoning Province (2011)	2014
10571	Zhihong Li	Monitoring Results of Iodine Deficiency Disorders in Jiangxi Province (2011)	2014
10572	Chunxian Li	Study on Early Pregnancy Thyroid Dysfunction and Autoantibody Screening	2013
10573	Changchun Hou	Survey on Iodine Nutrition Levels and Appropriate Salt Iodine Content in Tianjin Residents (2002-2011)	2013
10574	Hemin Zheng	Current Situation Survey of Iodine Deficiency Disorders in Henan Province (2011)	2013
10575	Wen Zhong	Analysis of Iodine Deficiency Disorder Monitoring Results in Guangdong Province (2011)	2013
10576	Feng Ye	Monitoring Results of Iodine Deficiency Disorders in Yunnan Province (2011)	2013
10577	Mei Liu	Monitoring Results of Iodine Deficiency Disorders in Baoji, Shaanxi (2009-2011)	2013
10578	Jie Ou	Study on the Effects and Mechanisms of Heavy Metal Exposure in Pregnant Women on Early Pregnancy Outcomes	2020
10579	Zhongna Sang	Effects of Excess Iodine Intake on Thyroid Function and Pregnancy Outcomes in Pregnant Women	2011
10580	Wenjuan Ding	Survey on Iodine Nutrition Status in Pregnant and Lactating Women in Bachu and Wushi Counties After Emergency Iodine Supplementation	2011
10581	Ruiran He	Effects of Heavy Metal Exposure on Fetal Development at Different Pregnancy Stages	2019
10582	Lianyun Yu	Mongolian Medicinal Resources	2016

10583	Ye Ruan	Survey on Iodine Nutrition in Agricultural and Pastoral Populations in Gannan Tibetan Autonomous Prefecture, Gansu (2011)	2012
10584	Qiaozhen Wei	Study on the Association Between Environmental Metal Exposure During Pregnancy and Neonatal Cardiovascular Damage Biomarkers	2018
10585	Xiaodan Yu	Levels and Risk Factors of Fetal Heavy Metal Exposure in Infants in Shanghai	2011
10586	Sha Zhu	Relationship Between Urinary Metal Levels and Childhood Acute Leukemia	2011
10587	Xiaolin Tian	Effects of Subchronic Arsenic-Fluoride Exposure on Rat Cardiotoxicity and Expression Changes of Autophagy-Related Genes	2019
10588	Zhihong Li	Monitoring Results of Iodine Deficiency Disorders in Jiangxi Province (2005-2008)	2009
10589	Xingshu Li	Survey and Analysis of Iodine Nutrition Levels in High-Risk Populations in Iodine-Deficient Areas in Chongqing	2009
10590	Min Liao	Analysis of Iodine Nutrition Levels in Women of Childbearing Age in High Non-Iodized Salt Areas in Guangxi (2008)	2009
10591	Not Found	How Harmful is Secondhand Smoke to Women	2017
10592	Wei Zheng	Discussion on the Testing and Detection Framework of Heavy Metals in Food	2017
10593	Ling Xiao	Discussion on Iodine Nutrition Status and Thyroid Function Indicators in Pregnant Women in Xiangyang	2016
10594	Yong Li	Investigation on Iodine Nutrition Status in Key Populations Before and After Iodized Oil Administration in the Qinghai Lake Region	2006
10595	Shengmei Zhu	Analysis of Factors Related to Congenital Hypothyroidism in Binzhou City	2005
10596	Li Huang	Research Progress on Environmental Factors Affecting Early Embryonic Development	2004
10597	Yushu Bao	Occupational Health of Women in China in the 21st Century	2001
10598	Weiqi Liu	Preliminary Study on Iodine Nutrition and Thyroid Diseases in Pregnant Women in Foshan	2015
10599	Ailing Ma	Analysis of Iodine Nutrition Monitoring Results in Key Populations in Dongying City, Shandong Province	2007
10600	Yalu Wang	Study on the Relationship Between Iodine Nutrition Levels and Thyroid Function in Pregnant Women	2018

10601	Suhong Gao	Survey on Urinary Iodine Levels in School-Age Children and Pregnant Women in Shantou City (2015)	2016
10602	Guijie Cai	Analysis of Urinary Iodine Levels in 1617 Pregnant Women in Puyang City	2016
10603	Yizhu Lu	Validation of the Electrothermal Constant Temperature Drying Box Digestion Method for Urinary Iodine Determination	2021
10604	Hui Liu	Analysis of Iodine Deficiency Disorder Monitoring Results in Yubei District, Chongqing (2011-2015)	2016
10605	Shaogui Liu	Five Brothers of Heat-Clearing and Detoxifying Drugs	2016
10606	Fengjiao Ding	Discussion on the Relationship Between Urinary Iodine and Thyroid Function in Pregnant Women	2015
10607	Yuanling Wang	Analysis of Urinary Iodine Monitoring Results in 8-10-Year-Old Children and Pregnant Women in Bishan District, Chongqing (2016)	2018
10608	Changqing Li	Analysis of Iodine Nutrition Levels in Pregnant Women in Shunyi District Before and After the Implementation of the New Iodized Salt Standard	2015
10609	Guanghui Jin	Analysis of Urinary Iodine Monitoring Results in Pregnant Women in Yixian County (2014)	2015
10610	Pei Pei	Study on the Activation of the IRE1伪/TNF-伪 Pathway by Inorganic Arsenic and the Protective Effects of Taurine on Pancreatic Beta Cell Pyroptosis	2019
10611	Huixuan Su	Analysis of Iodine Nutrition and Thyroid Hormone Levels in Pregnant Women in Beihai City	2010
10612	Rui Yan	Effects of Different Durations of Drinking Water Arsenic Exposure on Learning and Memory in Offspring Mice and Changes in NMDA Receptor Expression	2009
10613	Hong Wei	Monitoring Results of Iodine Deficiency Disorders in Tumushuke City, Third Division of Xinjiang (2010-2012)	2014
10614	Xiaohui Yu	Epidemiological Study on Thyroid Diseases in Pregnancy	2008
10615	Wei Zhang	Correlation Analysis of Trace Elements in Maternal Hair, Venous Blood, and Fetal Cord Blood	2005
10616	Bian Hu	Effects of Iodine Supplementation on Infant Growth, Development, and Iodine Nutrition Levels	2005
10617	Pinjiang Ma	Review and Prospects of Local Disease Prevention and Control in Xinjiang	2020
10618	Zhaozhong Zhu	Analysis of Salt Iodine and Urinary Iodine Monitoring Results in Key Populations in Lianshui County (2019)	2020

10619	Keying Cai	Investigation on the Incidence of Hypothyroidism in Early Pregnancy in High-Iodine and Iodine-Sufficient Areas in Xuzhou	2013
10620	Runming He	Rational Response to Diarrhea	2013
10621	Yan Qi	Study on the Content of Six Trace Elements in Pregnant Women's Blood and Fetal Cord Blood and Their Effects on Fetal Development	2010
10622	Linan Wu	Relationship Between Thyroid Diseases and Pregnancy Outcomes and the Effects of Iodine, Selenium, and Oxidative Stress on Thyroid Function in Pregnant Women	2009
10623	Ping Wu	Comparison of Urinary Iodine Concentration Measurements by ICP-MS and Arsenic-Cerium Catalytic Spectrophotometry	2017
10624	Lamao Cairang	Analysis of Iodine Nutrition Levels in Tibetan Populations in Hezuo City, Gansu Province (2010)	2012
10625	Chunmei Geng	Analysis of Salt Iodine and Urinary Iodine Monitoring Results in Key Populations in Bayan Nur City (2015-2016)	2017
10626	Yumei Chen	Nursing Observation of Vaginal Trial Labor in Cases of Umbilical Cord Around the Neck	2010
10627	Ze Cong	Role of Medical Geography in Environmental Issues in Nigeria	2010
10628	Yupeng Zhu	Effects of Fluoride and Arsenic Co-Exposure on PI3K-mTOR Signaling Molecule Expression in the Hippocampus and Cerebral Cortex of Offspring Mice	2016
10629	Jifang Liu	Drugs and Teratogenicity	2008
10630	Lei Liang	Analysis of Iodine Deficiency Disorder Monitoring Results in Ankang City (2017-2019)	2020
10631	Liao Xie	Study on the Effects of Trace Elements (Copper, Zinc, Nickel, Cadmium, Manganese) in Serum on Missed Abortion	2020
10632	Shengcheng Zhao	Monitoring Report on Iodine Deficiency Disorders in Tibet (2017)	2020
10633	Zhiqu Zhang	Effects of Sodium Arsenite on Nerve Growth Factor and Neurogrowth-Related Protein Expression in the Hippocampus of F1 Generation Rats	2011
10634	Na Li	Monitoring Results of Iodine Deficiency Disorders in Nyingchi City (2019)	2020
10635	Jinxia Yu	Relationship Between Cord Blood Manganese Concentration and Infant Weight at One Year: A Birth Cohort Study in Laizhou Bay, Shandong Province	2020
10636	Huijuan Zhang	Review on Heavy Metal Accumulation, Distribution, and Risk Analysis in Rice	2020

10637	Daowen Zhang	Study on Thyroid Function in Pregnant Women in Nanjing and the Effects of Age, Iodine Nutrition, and Thyroid Peroxidase Antibodies	2013
10638	Qin Bian	Effects of Heavy Metal Exposure During Pregnancy on Fetal and Neonatal Growth and Development and the Role of the Placental Barrier	2018
10639	Guoying Yin	Mechanistic Study on the Role of IGF2/H19 Imprinted Genes in Transgenerational Male Reproductive Damage Induced by Sodium Arsenite	2017
10640	Xiaoli Xu	Analysis of Iodine Deficiency Disorder Elimination Status in Jiaozuo City (2015)	2016
10641	Liao Xie	Case-Control Study on Factors Influencing Missed Abortion	2020
10642	Weilan Xu	Analysis of Iodine Nutrition Levels in the Population of Deqing County Before and After Iodized Salt Concentration Adjustment	2018
10643	Zuqun Jin	Analysis of Arsenic Levels and Correlation in Maternal Blood, Cord Blood, and Breast Milk in Normal Pregnant Women	2010
10644	Xing Li	Comparative Study on the Effects of Iodized Oil Intervention During Pregnancy and Lactation on Offspring Brain Development	2009
10645	Mingyan Li	Effects and Mechanisms of Fluoride and Arsenic Exposure on Growth, Development, and Learning and Memory Abilities in Offspring Mice	2014
10646	Weiqi Liu	Study on the Correlation Between Iodine Nutrition, SOD, and Thyroid Hormone Levels in Pregnant Women	2015
10647	Fengxian Zhao	Monitoring Results of Urinary Iodine in Children and Pregnant Women in Beichen District, Tianjin (2015)	2016
10648	Rong Qi	Analysis of Urinary Iodine Monitoring Results in Key Populations in Shuangliu County (2013-2015)	2017
10649	Not Found	Index of Subject Terms in the Journal of Harbin Medical University, 2000, Vol. 34 (Issues 1-6)	2000
10650	Xuanxuan Zhuang	Effects of Folic Acid on Congenital Heart Disease in Fetal Mice Induced by Arsenic Exposure	2017
10651	Jing Guo	Study on the Relationship Between Whole Blood Multi-Metal Exposure and Thyroid Hormones in Pregnant Women	2018
10652	Bing Wu	Genotoxicity and Carcinogenic Risk of Drinking Water from the Yangtze River Source in Nanjing	2010
10653	Jingfang Xie	Consistency Test of Urinary Iodine Levels Between Morning and Random Urine Samples	2010
10654	Nianzhong Yang	Survey on Iodine Nutrition in Special Needs Populations in Jiaojiang District	2010

10655	Bin Lian	Role and Mechanism of 5-Hydroxytryptamine Receptors in Autism-like Rats	2020
10656	Zhipeng Zhou	Analysis of Iodine Deficiency Disorder Prevention and Control in Longyan City Five Years After Adjusting Iodized Salt Concentrations	2019
10657	Zhuxia Zhu	Effects of Continuous Maternal-Fetal Arsenic Exposure on Mitochondrial Respiratory Function and Energy Metabolism in Offspring Rat Brain Cells	2010
10658	Xiaohong Chen	Relationship Between Chemical Element Levels in Pregnant Women's Hair in Fujian Province and Congenital Heart Disease in Fetuses	2014
10659	Chenyang Li	Effects of Iodine Nutrition on Maternal Thyroid Function and Offspring Intellectual Development	2014
10660	Jie Shi	Survey on Iodine Nutrition Levels in Key Populations in Tongchuan, Shaanxi Province (2017)	2018
10661	Danhua Li	Analysis of Iodine Nutrition Status in Residents of Qingpu District, Shanghai	2012
10662	Fenghong Zhao	Effects and Mechanisms of Arsenic Exposure on Synaptic Damage in the Hippocampus of Offspring Mice at Different Developmental Stages	2013
10663	Xiulan Chang	Analysis of Urinary Iodine Monitoring Results in 802 Key Populations in Pinggu District, Beijing	2011
10664	Zhizhong Du	Monitoring Report on Iodine Nutrition in Key Populations in Pinggu District, Beijing (2009)	2010
10665	Ning Zhang	Application of Urinary Iodine Microanalysis in Prenatal Screening	2009
10666	Yan Chen	Analysis of Urinary Iodine Monitoring Results in Zhenyuan County (2021)	2021
10667	Xiaohua Yuan	Effects and Molecular Mechanism of Arsenic on Progesterone Secretion in Ovarian Granulosa Cells	2011
10668	Chuanling Zhang	Effects and Mechanisms of Arsenic Exposure on In Vitro Development of Preimplantation Mouse Embryos	2010
10669	Xingyi Jin	Analysis of Urinary Iodine and Thyroid Hormone Levels in Urban and Rural Pregnant Women in Hangzhou	2009
10670	Ruirui Yang	Experimental Study on the Effects of CCK on Primary Sensory Input in Rats With Chronic Arsenic Poisoning	2008
10671	Huiping Pang	Analysis of Iodine Deficiency Disorder Monitoring in Jian'an District, Xuchang (2017)	2018
10672	Yanni Che	Polyethylene Tube Water Bath Digestion Method for Urinary Iodine Determination	2015

10673	Juan Wang	Monitoring Report on Iodine Deficiency Disorders in Guizhou Province (2011)	2012
10674	Chunhua Yu	Analysis of Iodine Nutrition Status in Pregnant Women in Qingdao and Its Effects on Maternal Thyroid Function and Neonatal Iodine Nutrition	2014
10675	Suzhen Zhu	Study on Thyroid Function in Pregnant Women in Urban Areas of Hangzhou	2009
10676	Zhonghui Liu	Analysis of Iodine Nutrition Status in Key Populations in Different Water Iodine Regions of Tianjin (2016)	2017
10677	Rencheng Zhao	Comparison of Subclinical Thyroid Disease Prevalence in Women From Iodine-Deficient, Iodine-Supplemented, and High-Iodine Areas	2014
10678	Yan Zhang	Research Progress on the Effects of Environmental Heavy Metals on Neural Tube Defects	2014
10679	Bingshuang Xue	Survey on Iodine Nutrition in the Population of Wujin District, Changzhou	2017
10680	Xiaoqing Wu	Survey and Analysis of Salt Iodine and Iodine Nutrition Status in Xiamen, Fujian Province	2015
10681	Qiang Lin	Survey on Iodine Nutrition in Key Populations in Yanqing County, Beijing (2013)	2015
10682	Jinian Lin	Monitoring Results of Iodine Nutrition in Key Populations in Danyang City (2016-2018)	2019
10683	Yanyan Lu	Monitoring Results of Iodine Nutrition in Residents of Liubei District, Liuzhou (2016-2018)	2019
10684	Qianqian Gao	Preliminary Study on Maternal Urinary Iodine Nutrition and Thyroid Dysfunction in Early Pregnancy	2013
10685	Siyu Wu	PM2.5-Induced Embryo Implantation Failure in Rats via MMPs/TIMPs Expression Changes in the Uterus	2016
10686	Not Found	GB2762-2017 "National Food Safety Standard - Maximum Levels of Contaminants in Food"	2018
10687	Baoli Zhang	Birth Cohort Study on the Association Between Low Cord Blood Manganese Levels and Neonatal Birth Weight	2018
10688	Nianzhong Yang	Study on Iodine Nutrition in the Population of Jiaojiang District, Taizhou, Zhejiang Province	2012
10689	Yuhong Niu	Distribution of Arsenic Species in the Liver and Brain of Offspring Rats at Different Developmental Stages Following Maternal Arsenic Exposure	2008
10690	Xibiao Liu	Survey on Iodine Nutrition in Residents of Jingning County	2016

10691	Houweimin Xia	Analysis of Iodine Deficiency Disorder Monitoring Results Before and After the Implementation of the New Iodized Salt Standard in Lechang City	2016
10692	Yan Tong	Effects of Iodine and Iron Nutrition on Thyroid Function in Pregnant Women in Western Fujian	2018
10693	Jing Kang	Analysis of Iodine Deficiency Disorder Monitoring Results in Heilongjiang Province in 2011 and 2014	2017
10694	Xiaojun Yin	Determination and Analysis of Serum TSH, FT4, TT4, and Urinary Iodine in Pregnant and Reproductive-Age Women in Changsha	2013
10695	Xiuhong Yang	Survey and Analysis of Urinary Iodine Levels in Key Populations in Renshou County (2015-2017)	2019
10696	Xiaowei Li	Determination and Analysis of 21 Trace Elements in Maternal and Cord Blood in Dalian	2009
10697	Xiuzhi Gui	Monitoring Report on HIV Mother-to-Child Transmission Prevention and Adverse Reactions to Infant Vaccination	2009
10698	Hongqiang Gong	2014 Monitoring Report on Iodine Deficiency Disorders in Tibet	2017
10699	Yalei Wang	Expression and Regulation of SR-B1 Gene in the Ovaries and Uterus of Rats	2015
10700	Qingping Chen	Analysis of Iodine Deficiency Disorder Monitoring Results in Longyan City (2015)	2016
10701	Jing Su	Survey and Analysis of Iodine Nutrition Levels in Key Populations in Suzhou (2017)	2018
10702	Yan Dong	Ecological Study on the Association Between Environmental Chemical Elements and Birth Defects and Their Pathogenic Mechanisms	2015
10703	Ya Zhai	Analysis of Iodine Deficiency Disorder Monitoring in Qianxinan Prefecture (2011)	2015
10704	Huiqiang Wu	Survey on Urinary Iodine Levels and Iodine Nutrition in Pregnant Women in Gaoming District, Foshan	2015
10705	Ruixia Xu	Survey and Analysis of Iodine Nutrition Status of Residents in Shanxi Province (2015)	2016
10706	Wenjuan Zeng	Effects of Prenatal Low-Dose Heavy Metal Exposure and Their Interactions on Full-Term Neonates with Low Birth Weight	2020
10707	Not Found	1999 Liuzhou City Science and Technology Achievements Statistics Summary	2000
10708	Ge Yang	Analysis of Urinary Iodine Monitoring Results in Key Populations in Shuangliu County (2006-2012)	2014

10709	Lingxiao Yue	Ptpn11E76K Activating Mutation Cooperates with miR-100 Inactivation to Promote Malignant Transformation of Mouse Embryonic Fibroblasts	2020
10710	Xiao Liu	Effects of Combined Exposure to PM2.5 and High-Fat Diet on TNF-α-Mediated Neuroinflammatory Response in the Cerebral Cortex of Rat Pups	2020
10711	Jinhua Zhou	Heavy Metal Levels in Newborns in Shaoguan, Guangdong, and Their Relationship with Low Birth Weight	2015
10712	Shitao Tang	Analysis of Iodine Nutrition in the Population of Tongshan District, Xuzhou	2015
10713	Wei Li	Survey on the Current Situation of Iodine Deficiency Disorders in Hulunbuir City (2011)	2012
10714	Kamixi Nursadek	2006 Survey Report on Iodine Deficiency Disorders in Five Counties (Cities) in Tacheng, Xinjiang	2010
10715	Yalin Chen	Analysis of Longitudinal Monitoring Results of Iodine Deficiency Disorders in Chongqing in 2012	2014
10716	Qunjian Lei	Analysis of Iodine Deficiency Disorders Monitoring in Changshou District, Chongqing (2006-2016)	2017
10717	Xuehong Li	Analysis of Iodine Nutrition Status in Cheng'an County, Hebei Province (2016)	2017
10718	Weixuan Chen	Analysis of Iodine Deficiency Disorders Monitoring in Various Counties of Wuxi (2015)	2017
10719	Yuxin Ning	A 1:1 Matched Case-Control Study on Neonatal Malformations in a Certain Area of Hubei Province	2019
10720	Danni Huang	Study on the Relationship Between Environmental Exposure, Body Metal Levels, and Autism in Children	2019
10721	Huan Wan	Study on Iodine Nutrition Status and Its Influencing Factors in Pregnant Women in Chengde	2017
10722	Qinglan Lin	Preliminary Study on Urinary Iodine Levels and Thyroid Dysfunction in Pregnant Women	2014
10723	Wulai Xia	Study on Pollutants in the Middle and Eastern Water Bodies of China—A Case of Phosphorus and Trace Elements Contamination in Fish	2020
10724	Dan Zhang	Survey on Heavy Metal and Trace Elements in Breast Milk in Eight Cities of China	2009
10725	Aiping Wei	Survey on Iodine Deficiency Disorders in Zhumadian City (2015)	2016
10726	Yan Yin	Monitoring Results of Urinary Iodine in Key Populations in Jurong City (2015)	2016

10727	Jiajie Zang	Comprehensive Evaluation of Iodine Nutrition and Dietary Iodine Intake in Shanghai Residents	2017
10728	Hong Wang	Monitoring and Analysis of Iodine Nutrition in Baotou City (2015)	2017
10729	Aiping Wei	Analysis of Iodine Nutrition Levels in Residents of Zhumadian City (2014)	2017
10730	Caitian Zhang	Study on the Inhibitory Effects of Tripterygium Glycosides on Sperm Production in Rats and Its Mechanism	2008
10731	Xiaojun Ji	Survey on Iodine Deficiency Disorders in Parts of Pingdingshan City (2014)	2015
10732	Jisheng Ma	Analysis of Iodine Deficiency Disorders Monitoring in Aba Prefecture (2011)	2015
10733	Caiping Yang	Iodine Deficiency Disorders Monitoring Report in Yichang (2014)	2015
10734	Jingyu Ma	Discussion on Normal Reference Ranges of Thyroid Function in Early Pregnancy in Changsha	2014
10735	Shixun Song	Analysis of Iodine Nutrition in Key Populations Before and After the Implementation of New Iodized Salt Standards in Shunyi District	2016
10736	Dongfen Wang	Analysis of Urinary Iodine Monitoring Results in Key Populations of Jianxi District, Luoyang (2015)	2016
10737	Liqiong Su	Survey on Iodine Nutrition in Residents of Haicang District, Xiamen (2015)	2016
10738	Adalaiti Tuoluhan	Analysis of Iodine Deficiency Disorders Monitoring in Tacheng, Xinjiang (2015)	2016
10739	Zhenyu Zhang	Clinical Observation of Iodine Nutrition and Thyroid Dysfunction in Early Pregnancy	2014
10740	Yang Xie	Hygienic Characteristics of Urinary Iodine Levels in Special Populations in Xuyi (2015-2017)	2018
10741	Huili Li	Analysis of Iodized Salt Consumption and Urinary Iodine Monitoring in Key Populations of Weidong District (2012-2014)	2015
10742	Huicheng Fu	Analysis of Iodine Nutrition Monitoring in Five Key Populations in Xinxiang City (2013)	2015
10743	Xiaojun Ji	Survey on Iodine Nutrition in Five Populations in Pingdingshan City (2014)	2015
10744	Zushan Zhang	Role of Apoptosis and Autophagy Disorders in High Iodine-Induced Neurotoxicity in SD Rats	2019

10745	Xingyi Jin	Study on Subclinical Thyroid Dysfunction in Pregnant Women in Iodine-Sufficient Urban Areas	2010
10746	Yanyan Chen	Screening of Hypothyroidism in Pregnant Women and Experimental Study on the Effects of Iron Deficiency on Thyroid Function	2009
10747	Gang Lei	Survey on Growth, Development, and Iodine Nutrition of Children in Iodine-Deficient Areas of Xinjiang	2011
10748	Meng Zhang	Iodine Nutrition of Lactating Mothers and Infants Within 12 Weeks Postpartum	2015
10749	Wenbin Wu	Study on the Reproductive and Genetic Toxicity of Realgar in Traditional Chinese Medicine	2009
10750	Tingting Wang	Effects of Iodine Overload on TRα1, TRβ1, NIS, and Dio1 in Lactating Rats	2017
10751	Yuehua Ding	Investigation of Corn ZEA Contamination in Heilongjiang and Its Effects on Chicken Lymphocyte Apoptosis and Selenium Antagonism	2017
10752	Junyan Nie	Effects of High Iodine on Learning, Memory, and Autophagy in Hippocampal Neurons of Rats	2018
10753	Guomin An	Evaluation of Iodine Nutrition in Key Populations in Zhangjiakou	2008
10754	Huihui Miao	Study on Iodine Nutrition Levels and Thyroid Disease Prevalence in Fujian Province	2015
10755	Xiaoxue Wang	Investigation of Iodine Nutrition and Thyroid Autoantibody Levels in Pregnant Women with Subclinical Hypothyroidism	2014
10756	Dandan Chen	Preliminary Study on the Correlation Between Nonsyndromic Cleft Lip and Palate and Trace Elements	2014
10757	Haibo Xue	Experimental Study on the Effects of Selenium Intervention in the Treatment of Iodine-Induced NOD.H-2h4 Thyroiditis on CD4+CD25+ Regulatory T Cells in Mice	2010
10758	Yan Gao	Study on the Reproductive Toxicity of Sodium Nitrite Exposure in Male Mice	2017
10759	Shanhui Chen	Epidemiological Study on Subclinical Hypothyroidism in Different Populations in China	2012
10760	Peilei Hou	Study on Polysaccharide Iron and Capsule Impurities	2018
10761	Zhongna Sang	Effects of Sulfur Overload on Thyroid Function in Different Populations and Safe Intake Levels for Adults	2011
10762	Lingqin Zhao	Epidemiological Study on the Effects of Lead, Mercury, and Other Metal and Trace Elements on Fetal Congenital Malformations and Early Miscarriages	2008

10763	Wei Zhang	Study on the Relationship Between Trace Elements and Birth Defects in Some Regions of China	2004
10764	Mingxue Qian	Safety Evaluation of Huoshan Dendrobium and Seasonal Changes in Its Polysaccharide Biochemical Properties	2016
10765	Juwei Wang	Study on the Relationship Between Environmental Iodine Levels and Iodine Nutrition in Women of Childbearing Age in Six Ecological Zones of Gansu Province	2016
10766	Lu Zhang	Experimental Study on the Effects of Different Sulfur Nutrition Levels on Maternal-Infant Iodine Metabolism, Thyroid Function, Offspring Development, and Mammary Gland Regulation During Lactation	2009
10767	Ying Ding	Evaluation of Iodine Nutrition Status in 8-10-Year-Old Children and Women of Childbearing Age in Aksu and Hotan Regions	2017
10768	Siqi Liu	Survey and Analysis of the Nutrition and Developmental Status of Lactating Women and Their Offspring	2017
10769	Yong Feng	Investigation on the Iodine Nutrition Status of Residents in a District of Qingdao and the Reference Range of Thyroid Hormones in Pregnant Women in the Second Trimester	2015
10770	Jingyuan Ma	Study on the Quality Standards of Ganqing Granules	2013
10771	Lin Lin	Study on the Changes in Mammary Gland Iodine Uptake Capacity and Related Hormonal Effects in Lactating Rats Under Different Iodine Nutrition Levels	2010
10772	Jitian Zhang	Study on the Distribution Characteristics of Iodine-Deficient Areas in the Taiyi Mountain Region of Shandong Province and the Iodine Nutrition Status of Key Populations	2013
10773	A. Li	Effects of Fluoride and Arsenic Exposure During Pregnancy and Lactation on the Growth and Neurobehavioral Development of Offspring Rats	2014
10774	A. Li	Effects of Prenatal and Postnatal Arsenic Exposure on Sema 3A Expression in the Brain Tissue of Offspring Rats	2013
10775	Hong Li	Five Key Actions During Pregnancy for Giving Birth to a Healthy Baby	2016
10776	Not Found	Low-Dose Arsenic Intake in Pregnant Women May Affect Fetal Growth	2016
10777	Feixiang Xu	Mitochondrial Dysfunction-Induced TET Enzyme Activity Reduction Mediates the Inhibition of Mesenchymal-Like Behavior in Trophoblast Cells Caused by Prenatal Arsenic Exposure	2022
10778	Xu Chen	Acute Arsenic Exposure During Pregnancy Reduces DNA Hydroxymethylation Levels in the Fetal Brain, Leading to Neural Tube Defects	2022
10779	Jingzhuo Han	Protective Effects of Resveratrol Against Learning, Memory, and Oxidative Stress Damage in Offspring Mice Exposed to Sodium Arsenite From Gestation to Sexual Maturity	2022
10780	Rui Jiang	Effects of Folic Acid Supplementation on Learning and Memory Abilities in Maternal Mice Exposed to Arsenic During Pregnancy and Lactation	2022

10781	Yaping Song	Depletion of S-Adenosylmethionine Mediates the Downregulation of CYR61 m~6A, Leading to Impaired Trophoblast Invasion and Fetal Growth Restriction Induced by Prenatal Arsenic Exposure	2022
10782	Hua Zhu	Discussion on the Quality Standards of Traditional Chinese Medicine Preparations Containing Realgar in the 2020 Edition of the Chinese Pharmacopoeia	2021
10783	Xin Chen	Effects of Developmental Arsenic Exposure on DNA Methylation and microRNA Expression in Mouse Liver	2021
10784	Ziling Li	Relationship Between Thyroid Status and Iodine Levels in the Population of Inner Mongolia	
10785	Wenqi Hu	Association Study Between Pregnant Women's Intake of Seafood and Animal Organs and Urinary Levels of Arsenic, Cadmium, Lead, and Molybdenum	2020
10786	Yi Wu	Assessment and Characteristics of Internal Arsenic Exposure in Pregnant Women	2020
10787	Wuye Pan	Correlation Between Persistent Toxic Substance Exposure and the Risk of Female Ovarian Function Decline	2020
10788	Yu Zhang	Mechanistic Study on DNA Methylation-Induced Insulin Resistance in Male Offspring Due to Prenatal Arsenic Exposure	2019
10789	Dongyan Ren	Role of Zinc-α2-Glycoprotein (ZAG) in the Association Between Arsenic and Diabetes	2018
10790	Xun Xia	Birth Cohort Study on the Association Between Arsenic, Chromium, Placental Inflammatory Factors, and Gestational Diabetes Mellitus	2018
10791	Hongrui Yang	Effects of Fluoride and Arsenic Poisoning on the ERK-MAPK Signaling Pathway in the Brain Tissue of Rat Pups	2017
10792	Yuanyuan Sun	Summary Analysis of Traditional Chinese Medicine Formulations Containing Realgar and Cinnabar in the 2015 Edition (Part One) of the Chinese Pharmacopoeia	2017
10793	Kai Zhong	How to Deal with Inorganic Arsenic in Rice?	2016
10794	Kai Zhong	The Arsenic Content in Rice—A Global Concern, What is the Situation in China?	2016
10795	Mingfang Diao	A Case of Congenital Inner Ear Malformation Caused by Realgar Intake During Pregnancy	2015
10796	Lihua Huang	Study on the Relationship Between Intrauterine Exposure to Arsenic, Cadmium, and Lead and Neonatal Growth and Development	2014
10797	Li Chen	Relationship Between Arsenic Levels in Pregnant Women in Fujian Province and Congenital Heart Disease in Fetuses	2013
10798	Yongwen Zhang	Quality Control and Safety Evaluation of Traditional Chinese Medicine Preparations Containing Cinnabar and Realgar	2010

10799	Xiaofang Huang	A Pregnant Woman's Misunderstanding About Kelp	2009
10800	Deng Pan	Successful Treatment of Pregnancy-Associated Acute Promyelocytic Leukemia with Combined Retinoic Acid and Arsenic Trioxide: A Case Report	2007
10801	Hongfang Cui	Adverse Reactions of Traditional Chinese Medicine and Their Contributing Factors	2004
10802	Not Found	Nutrition	2003
10803	Chen Zhang	Research Progress on the Effects of Arsenic on Offspring	2002
10804	Jing'ai Jin	A Case of Acute Arsenic Poisoning in a Pregnant Woman Receiving High-Dose Treatment	1999
10805	Not Found	Pregnant Women Should Avoid Eating Kelp	1998
10806	Yanxue Zhao	Developmental Toxicity and Carcinogenicity of Metals in Mammals—Review of the 6th National Conference on Veterinary Pharmacology and Toxicology	1997
10807	Changlai Du	Attention to the Toxic and Side Effects of Cinnabar and Realgar on Pregnant Women and Children	1997
10808	Long Ma	Preliminary Study on the Effects of Arsenic on the Immune Function of Mouse Offspring	1994
10809	Long Ma	Effects of Arsenic on the Growth and Development of Mouse Offspring	1994
10810	Long Ma	Effects of Arsenic on the Neurobehavioral Development of Mouse Offspring	1993
10811	Long Ma	Preliminary Study on the Developmental Toxicity of Arsenic	1993
10812	Long Ma	Effects of Inorganic Arsenic on the Neurobehavioral Development of Mouse Offspring	1992
10813	Long Ma	Study on the Effects of Arsenic on the Immune Function of Mouse Offspring	1992
10814	Chuanfang Xiao	Teratogenic Effects of Inorganic Arsenic Compounds in Experimental Studies	1985
10815	Guohe Zhang	Effects of Arsenic on Embryonic Development	1984
10816	R. M. Gardner	Pregnancy and the methyltransferase genotype independently influence the arsenic methylation phenotype	2012

10817	S. Shirai	Maternal exposure to low-level heavy metals during pregnancy and birth size	2010
10818	L. Huang	The health effects of exposure to arsenic-contaminated drinking water: a review by global geographical distribution	2015
10819	Y. T. Zhou	Arsenic in agricultural soils across China: Distribution pattern, accumulation trend, influencing factors, and risk assessment	2018
10820	Yinlong Jin	Investigation on the Distribution of Endemic Arsenic Poisoning in China (General Report)	2003
10821	Renhua Zhang	Characteristics of Reproductive System Damage and Benchmark Dose Analysis of Biological Markers in Female Rats with Arsenic Poisoning	2016
10822	Dong An	Current Situation and Countermeasures for the Prevention and Control of Endemic Arsenic Poisoning in Guizhou Province	2005
10823	K. YJ	Arsenic Toxicity in Male Reproduction and Development	2015
10824	H. S	Biotransformation of arsenic and toxicological implication of arsenic metabolites	2020
10825	L. FF	Biomarkers for the evaluation of population health status 16 years after the intervention of arsenic-contaminated groundwater in Xinjiang, China	2013
10826	L. K	Arsenic exposure perturbs the gut microbiome and its metabolic profile in mice: an integrated metagenomics and metabolomics analysis	2014
10827	A. JA	Vasculogenesis and angiogenesis in the IUGR placenta	2008
10828	N. J	E-cadherin polymorphisms and susceptibility to arsenic-related skin lesions in West Bengal, India	2012
10829	E. B	Serum dioxin concentrations and endometriosis: a cohort study in Seveso, Italy	2002
10830	N.-A. A	Arsenic exposure and prevalence of type 2 diabetes in US adults	2008
10831	M. KA	Association between exposure to low to moderate arsenic levels and incident cardiovascular disease. A prospective cohort study	2013
10832	N. MF	The broad scope of health effects from chronic arsenic exposure: update on a worldwide public health problem	2013
10833	R. T	Arsenic in drinking water and congenital heart anomalies in Hungary	2014
