# Supplementary material for: Association between arsenic exposure and intrauterine growth restriction: A systematic review and meta-analysis
Source: PLoS One. 2025 Jun 2;20(6):e0320603. doi: 10.1371/journal.pone.0320603 (PMC12129153; doi:10.1371/journal.pone.0320603)
Supplement: S1 Fig — (PDF) [file pone.0320603.s010.pdf]

S1 Fig. Meta-regression analysis results

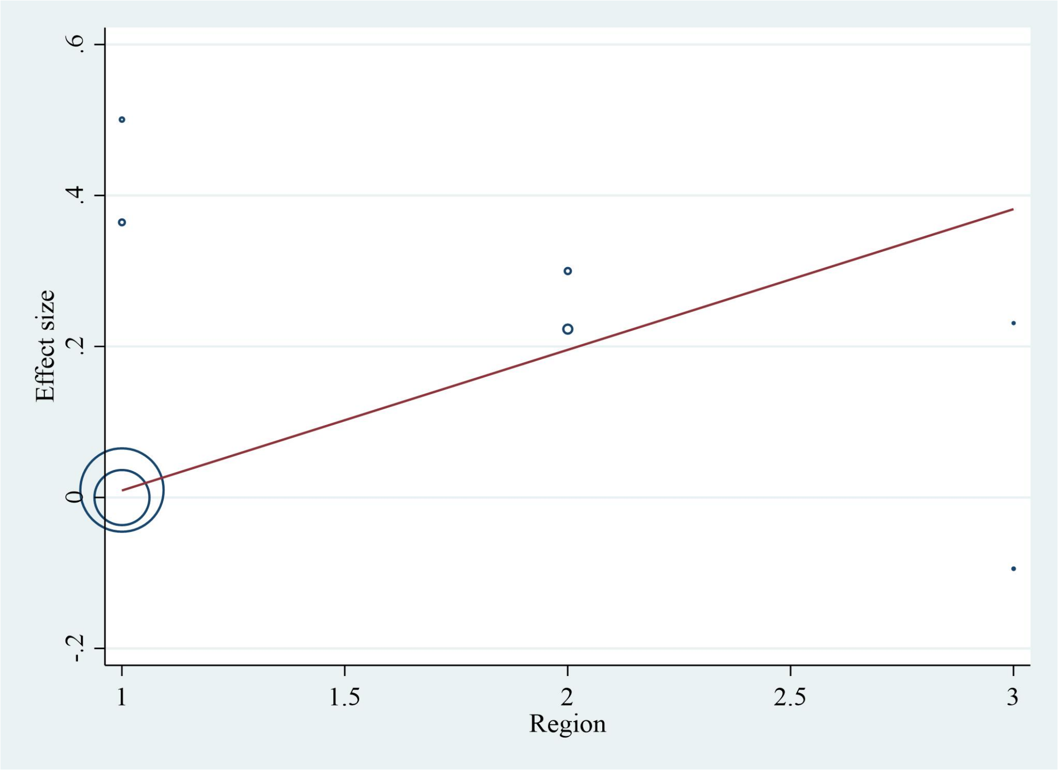

Fig. 1. As and SGA (Region)

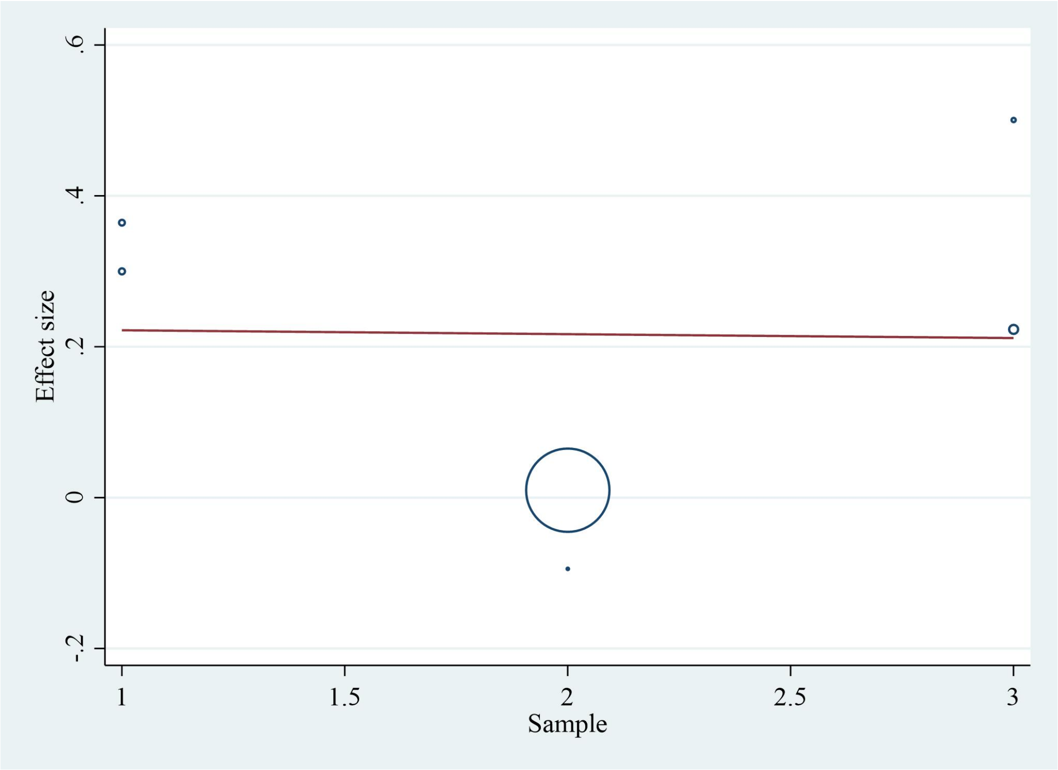

Fig. 2. As and SGA (Sample)

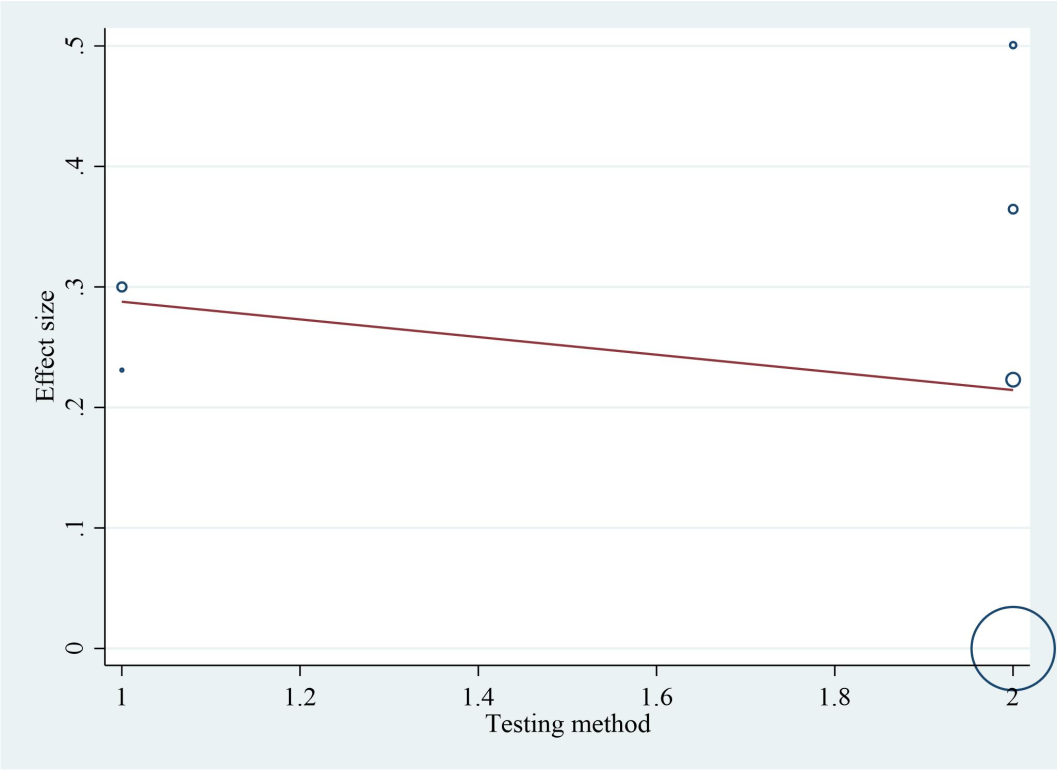

Fig. 3. As and SGA (Testing method)

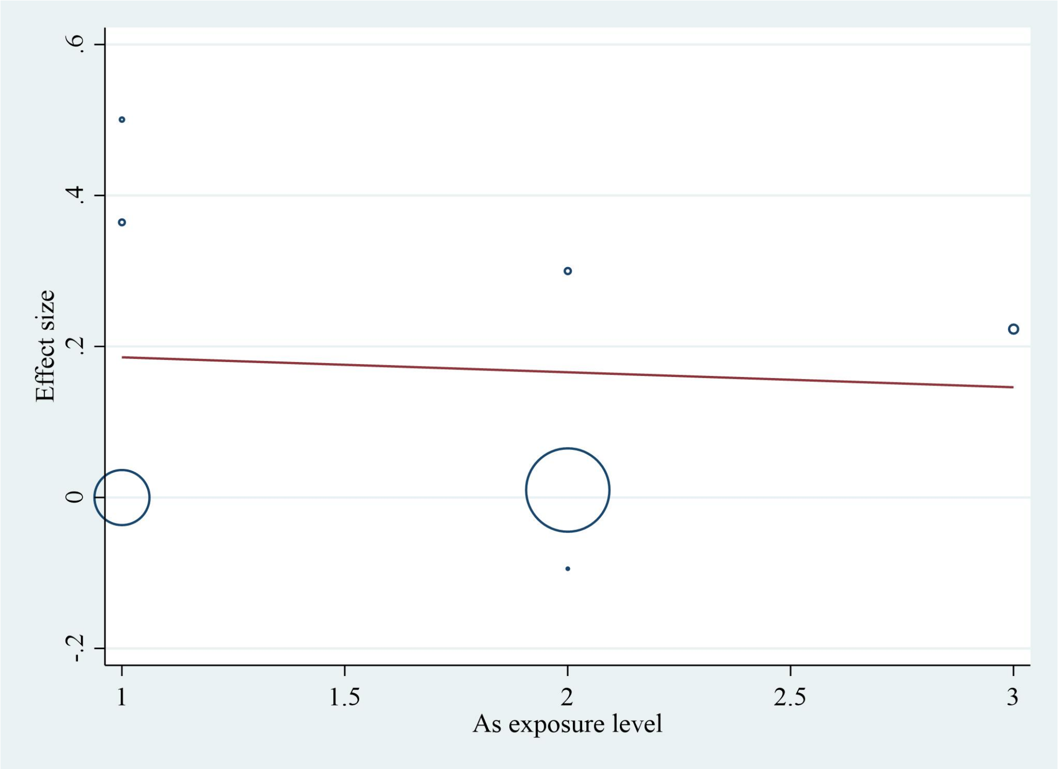

Fig. 4. As and SGA (As Exposure level)

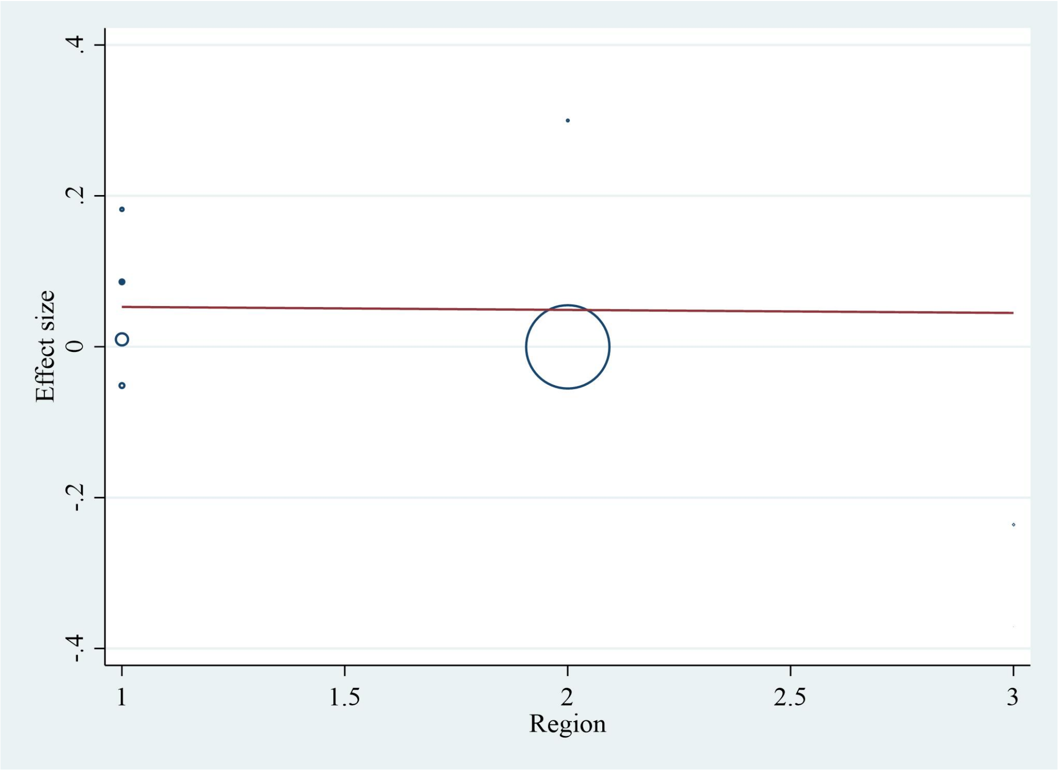

Fig. 5. As and PTB (Region)

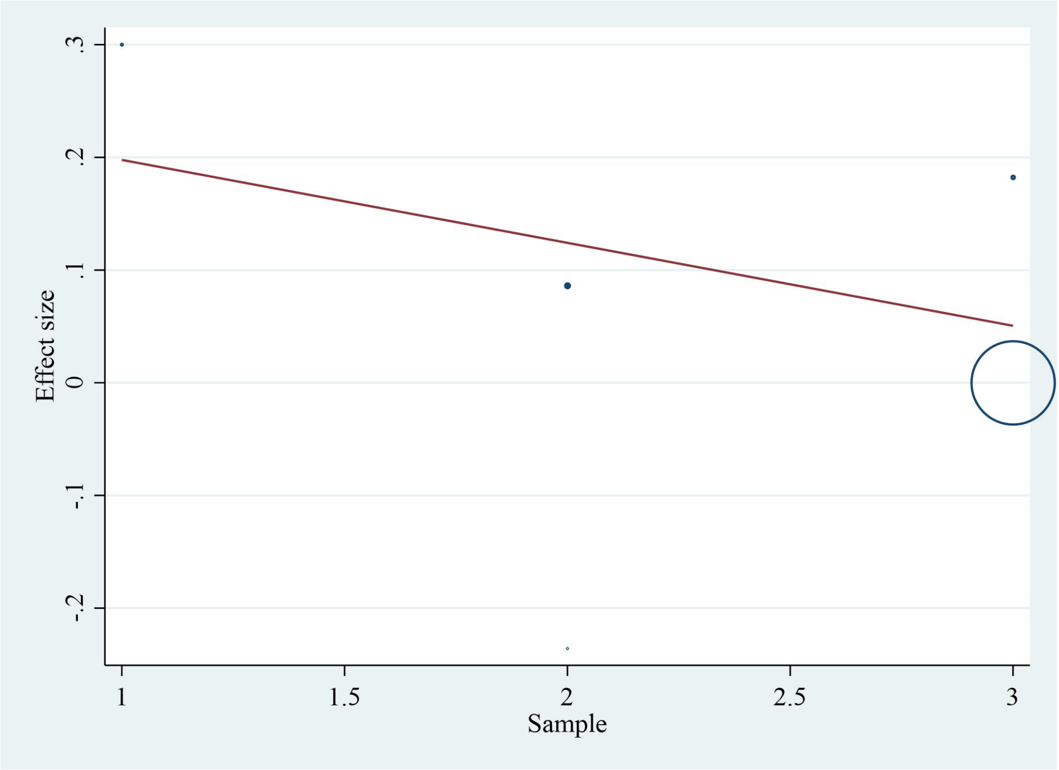

Fig. 6. As and PTB (Sample)

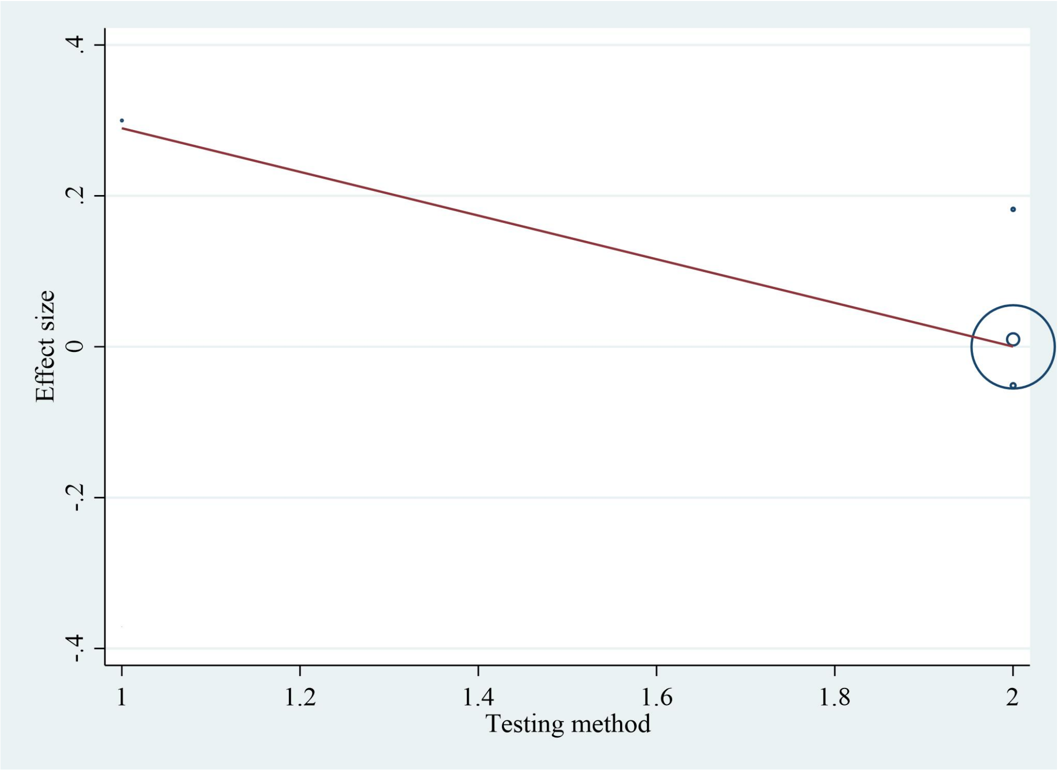

Fig. 7. As and PTB (Testing method)

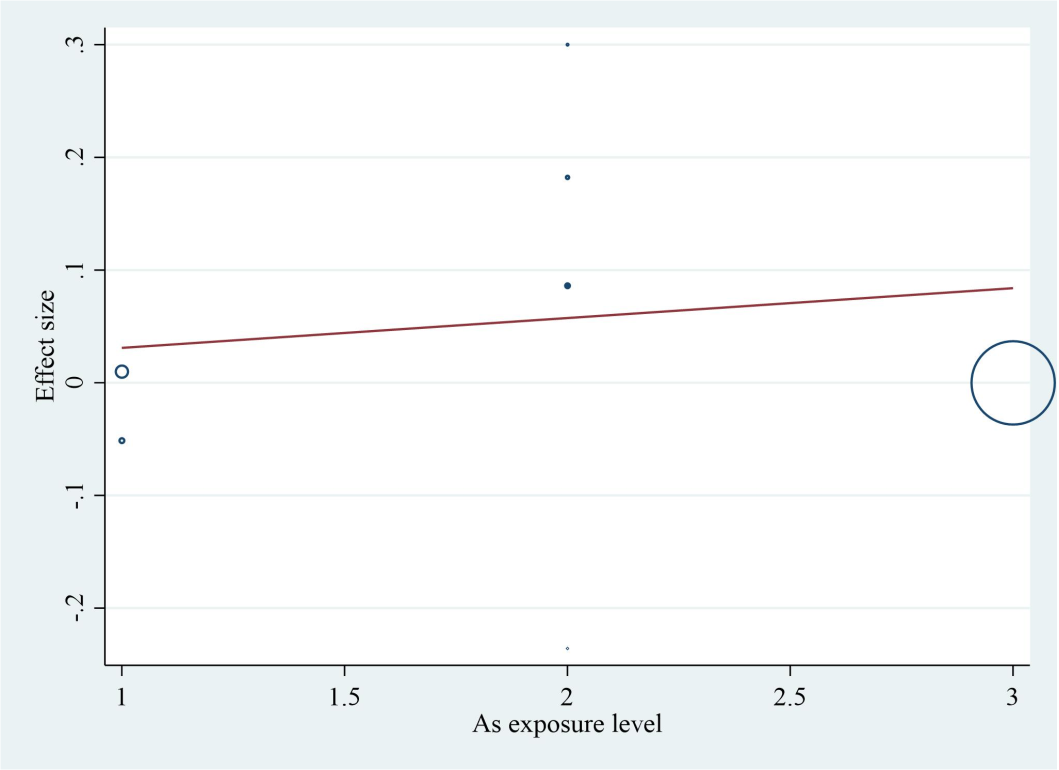

Fig. 8. As and PTB (As Exposure level)

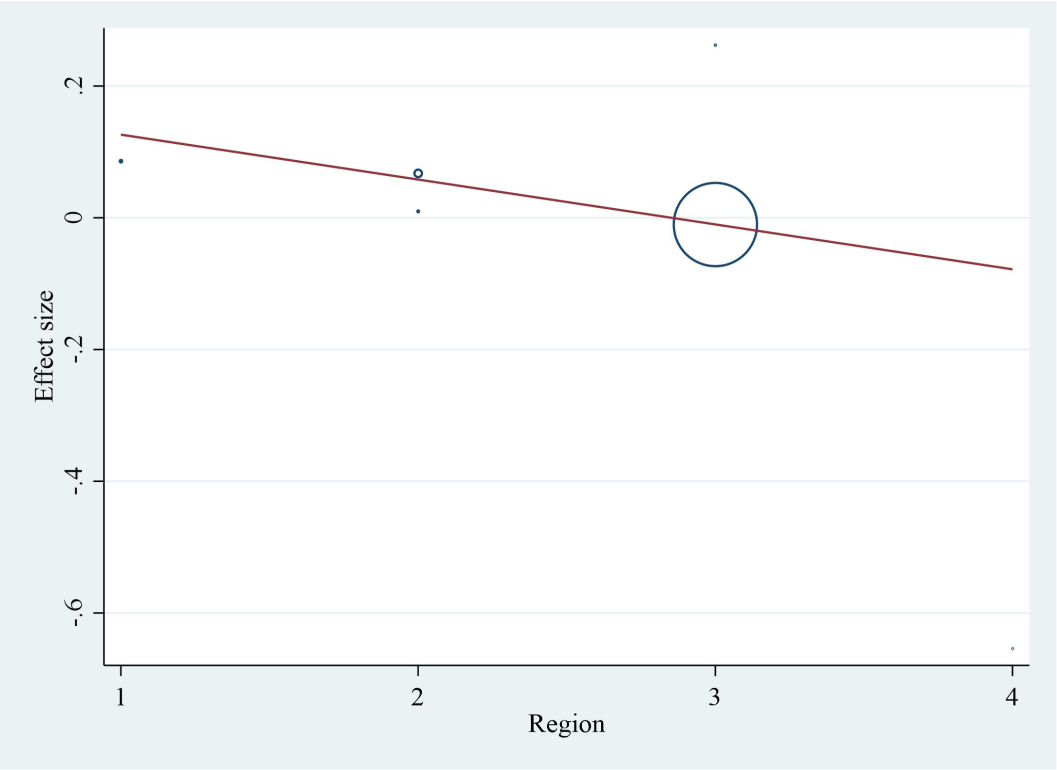

Fig. 9. As and LBW (Region)

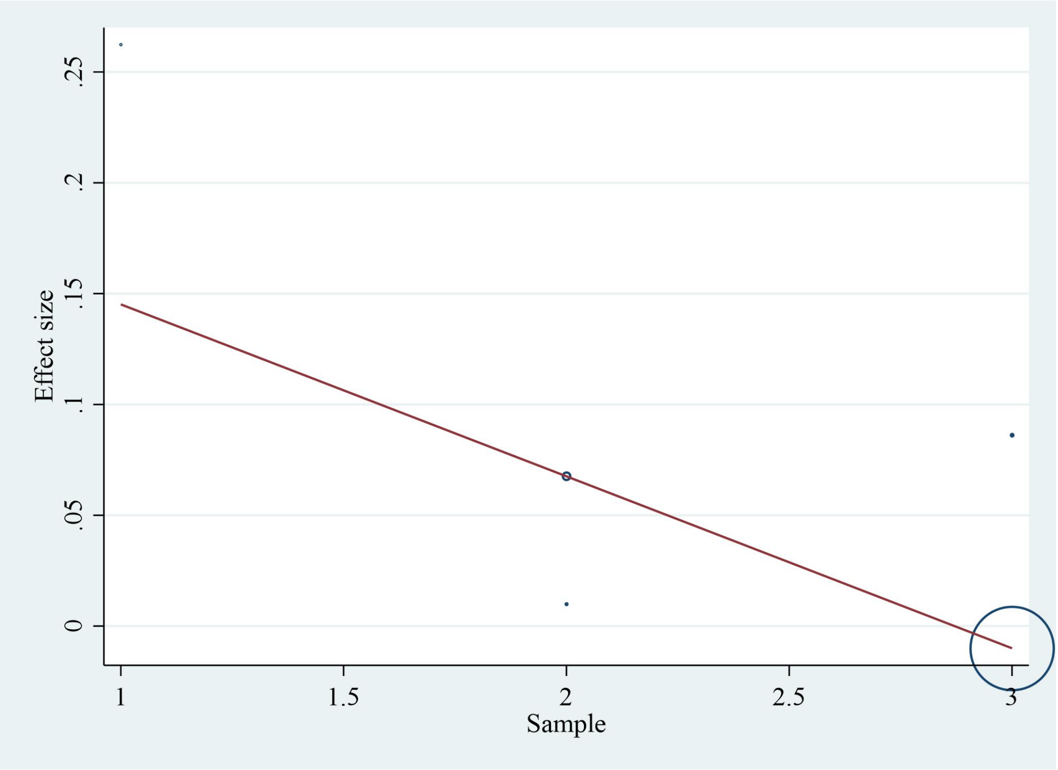

Fig. 10. As and LBW (Sample)

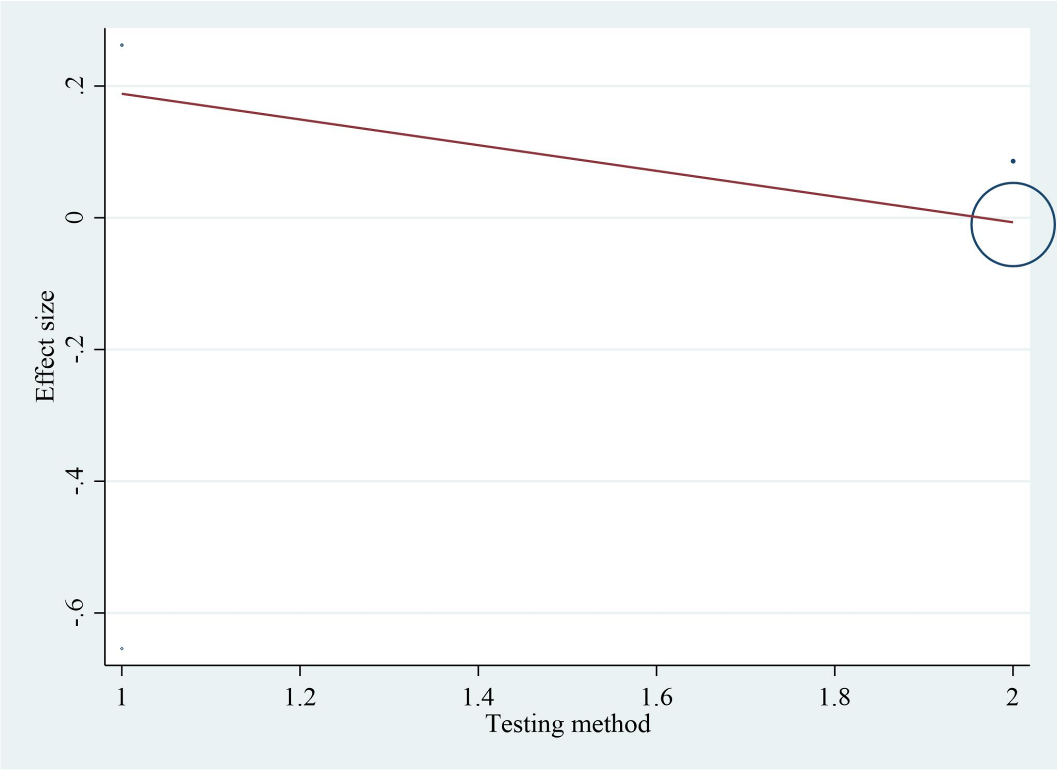

Fig. 11. As and LBW (Testing method)

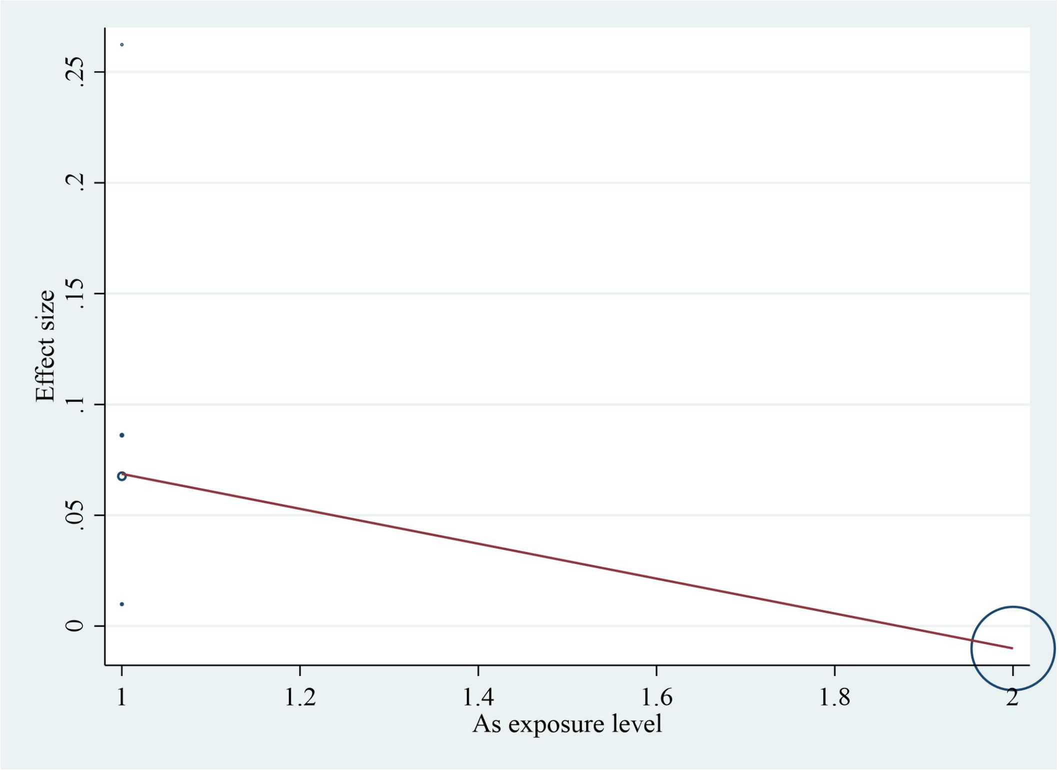

Fig. 12. As and LBW (As Exposure level)
