# Supplementary material for: Association between arsenic exposure and intrauterine growth restriction: A systematic review and meta-analysis
Source: PLoS One. 2025 Jun 2;20(6):e0320603. doi: 10.1371/journal.pone.0320603 (PMC12129153; doi:10.1371/journal.pone.0320603)
Supplement: S2 Fig — (PDF) [file pone.0320603.s011.pdf]

S2 Fig. Results of sensitivity analyses

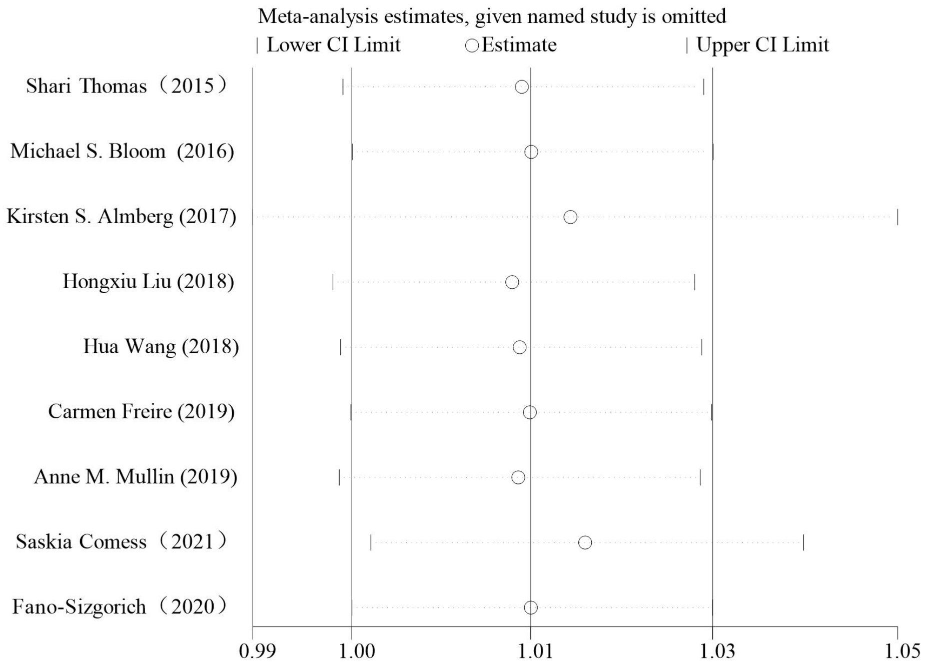

Fig. 1. Results of sensitivity analysis of association between arsenic exposure and risk in infants small for gestational age.

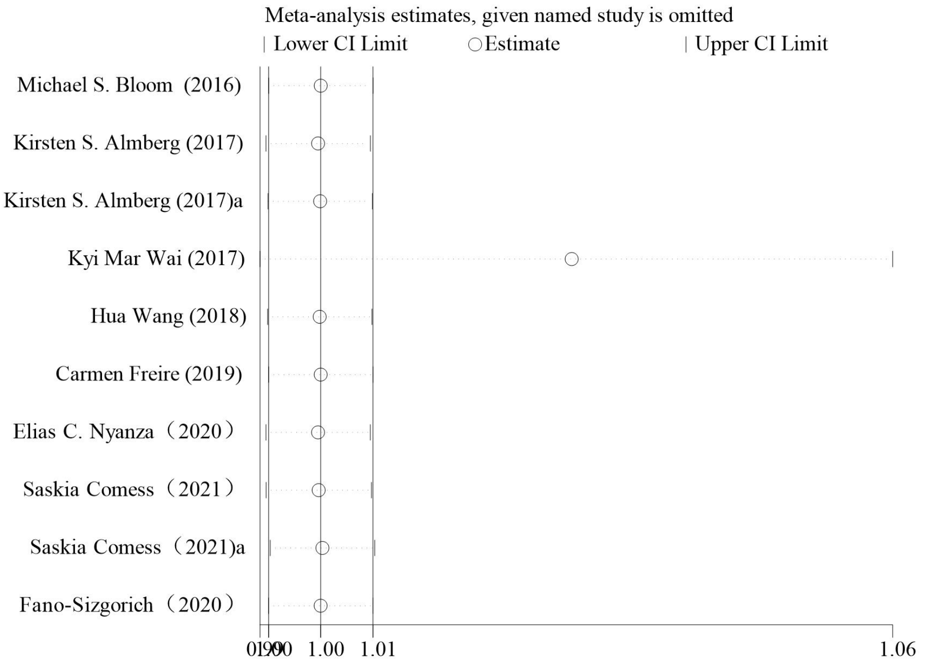

Fig. 2. Results of sensitivity analysis of association between arsenic exposure and preterm birth risk.

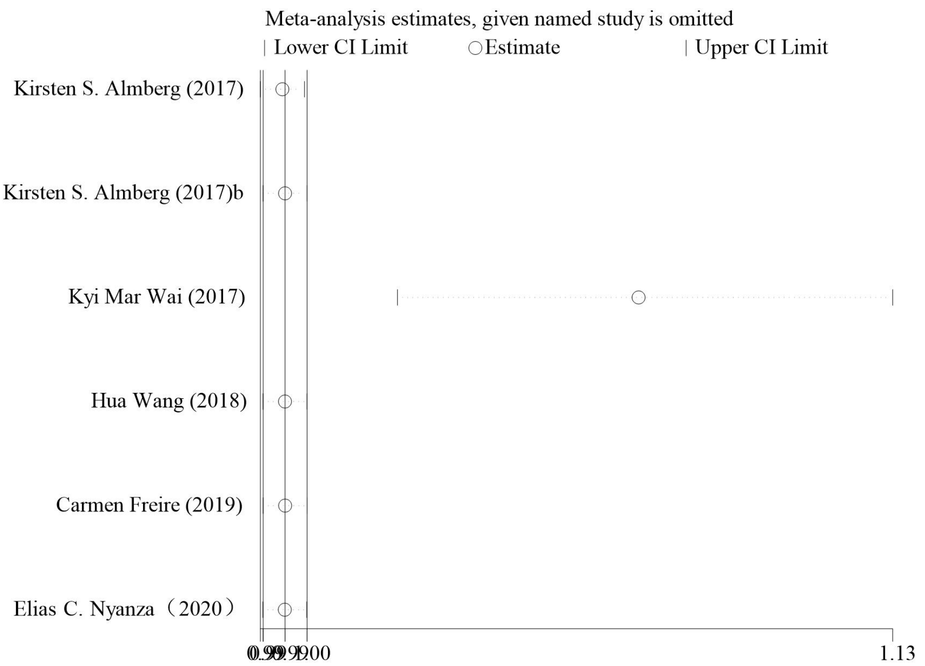

Fig. 3. Results of sensitivity analysis of association between arsenic exposure and risk of low birth weight.
