# Supplementary material for: Association between arsenic exposure and intrauterine growth restriction: A systematic review and meta-analysis
Source: PLoS One. 2025 Jun 2;20(6):e0320603. doi: 10.1371/journal.pone.0320603 (PMC12129153; doi:10.1371/journal.pone.0320603)
Supplement: S3 Fig — (PDF) [file pone.0320603.s012.pdf]

S3 Fig. The results of the bias assessment were published

Table 1

Publication bias of included studies.

Publication bias of included studies

Outcome	Numbers of studies involved	Exposure	Egger's test (*P value*)	Begg's test (*P value*)
SGA	9	As	**0.032**	0.754
PTB	10	As	0.112	0.592
LBW	6	As	0.239	1.000

Note: Data in bold indicate significant results; As, arsenic; SGA, small for gestational age; LBW, low birth weight; PTB, preterm birth.

3.1 SGA

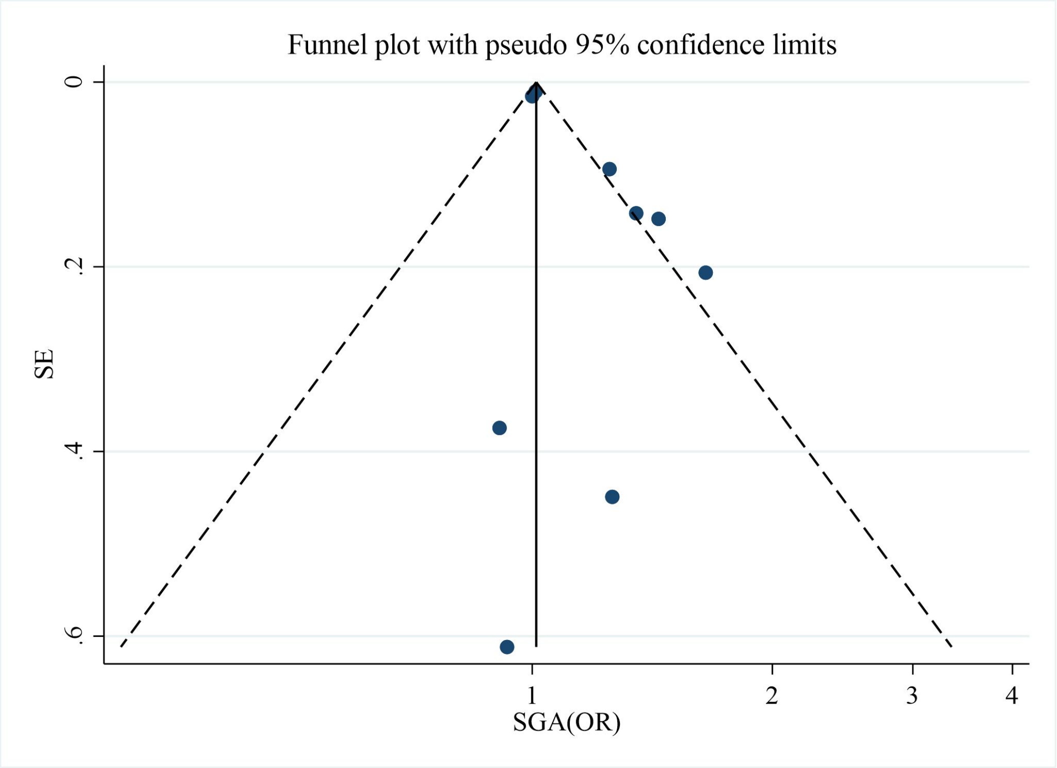

Fig. 1. Results of a publication bias assessment of arsenic in infants small for gestational age (Funnel Plot).

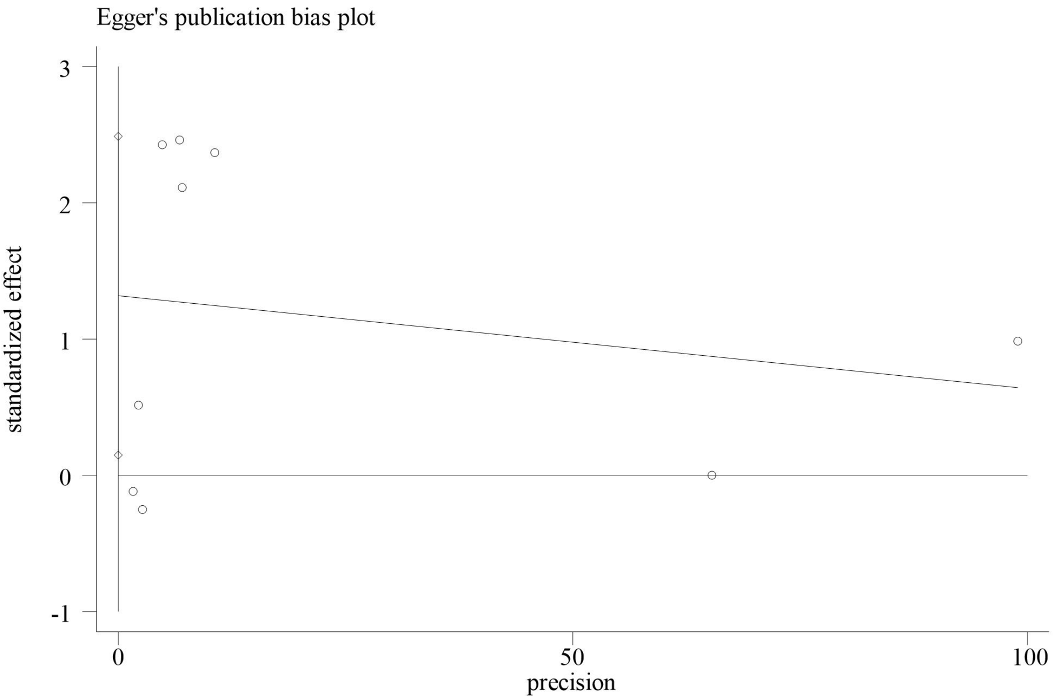

Fig. 2. Results of a publication bias assessment of arsenic in infants small for gestational age (Egger's).

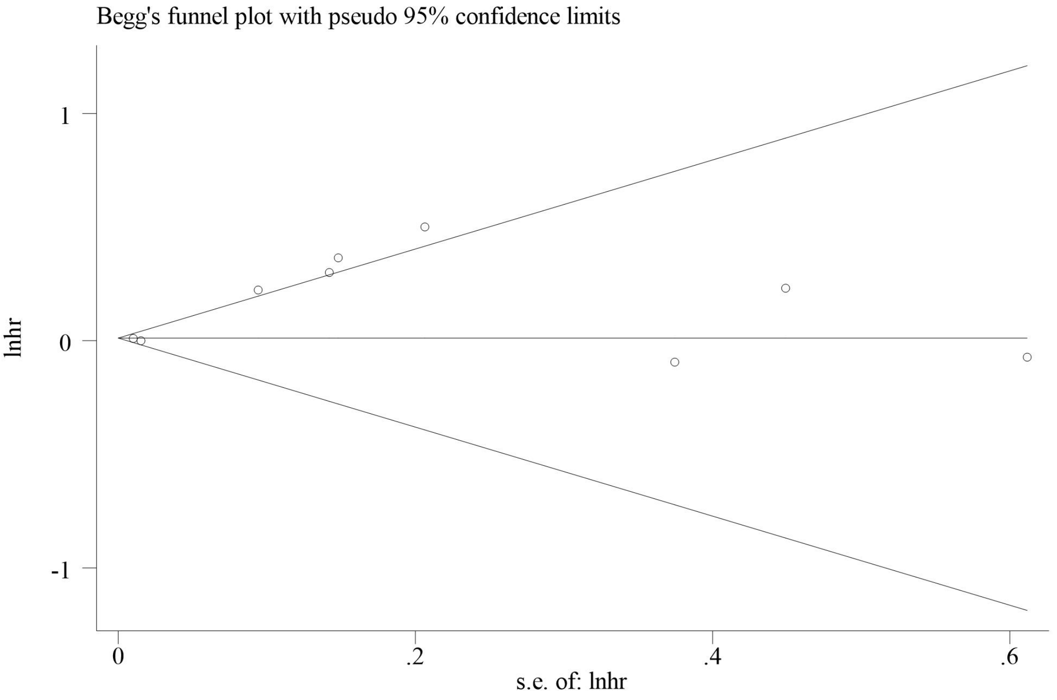

Fig. 3. Results of a publication bias assessment of arsenic in infants small for gestational age (Begg's).

3.2 PTB

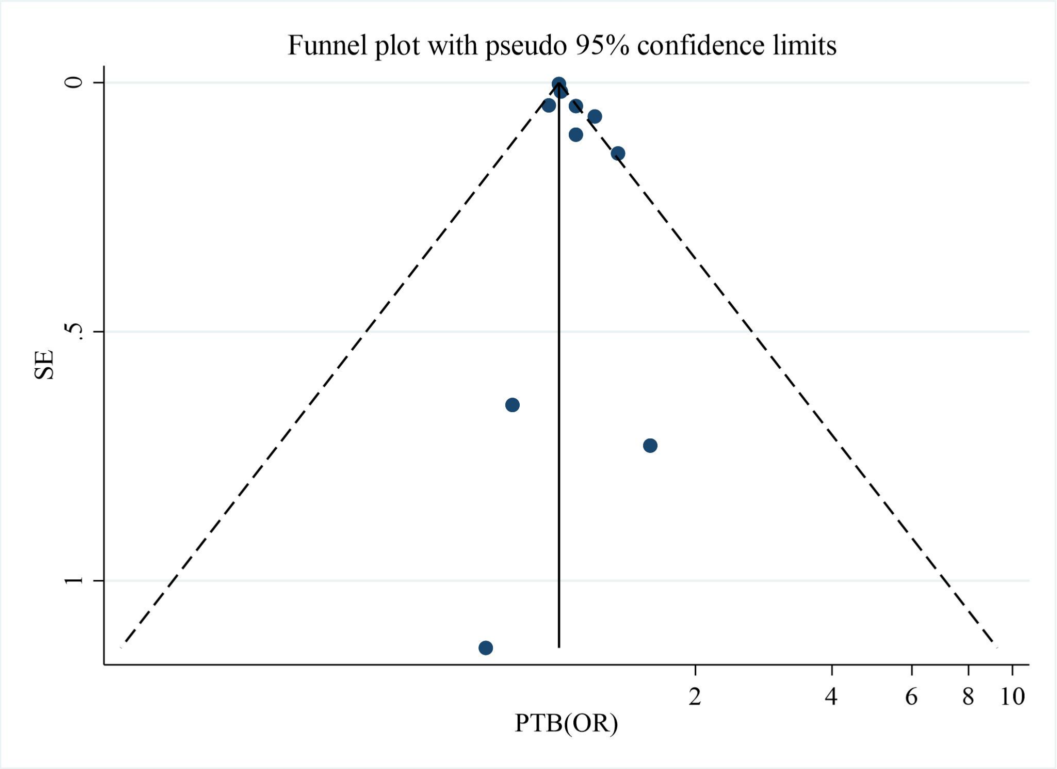

Fig. 4. Results of a publication bias assessment of arsenic on preterm birth (Funnel Plot).

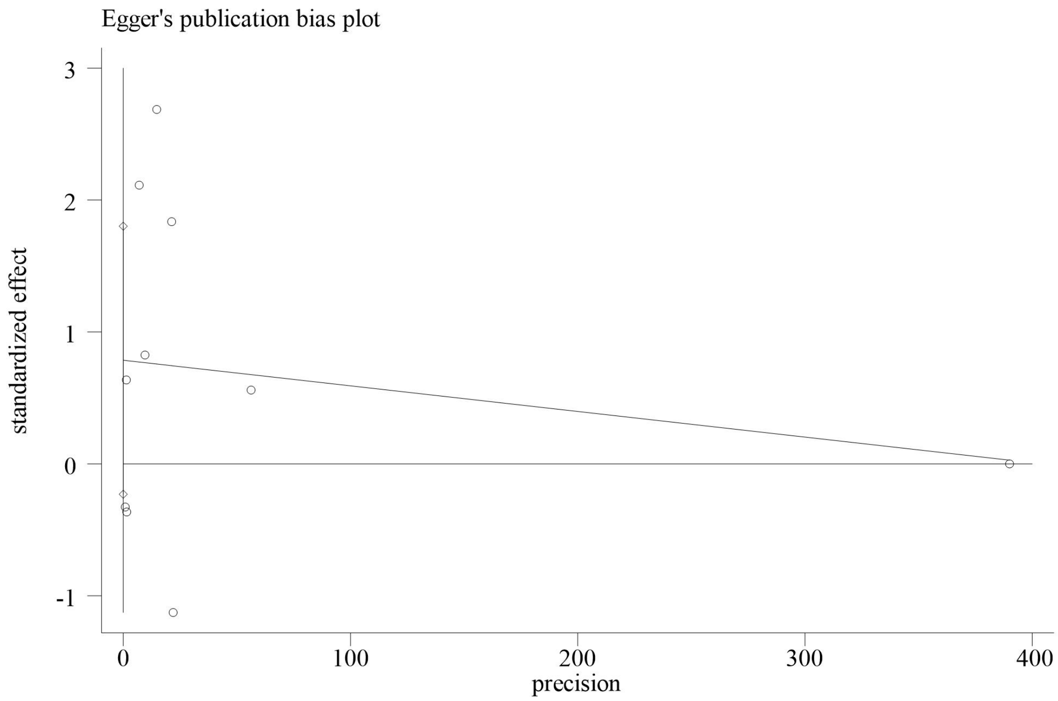

Fig. 5. Results of a publication bias assessment of arsenic on preterm birth (Egger's).

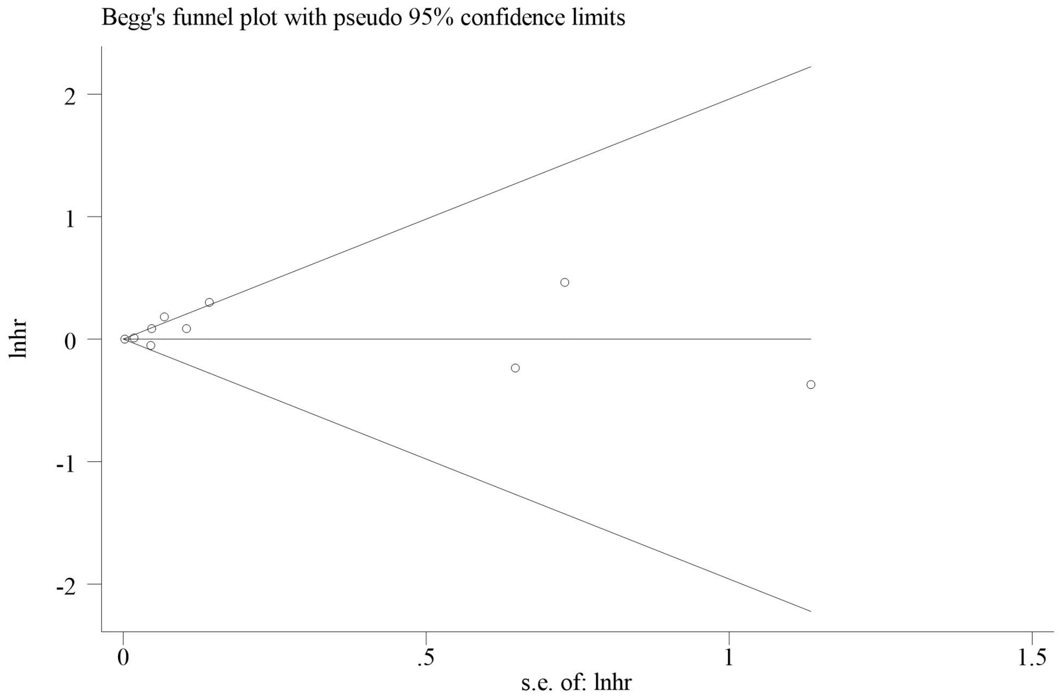

Fig. 6. Results of a publication bias assessment of arsenic on preterm birth (Begg's).

3.3 LBW

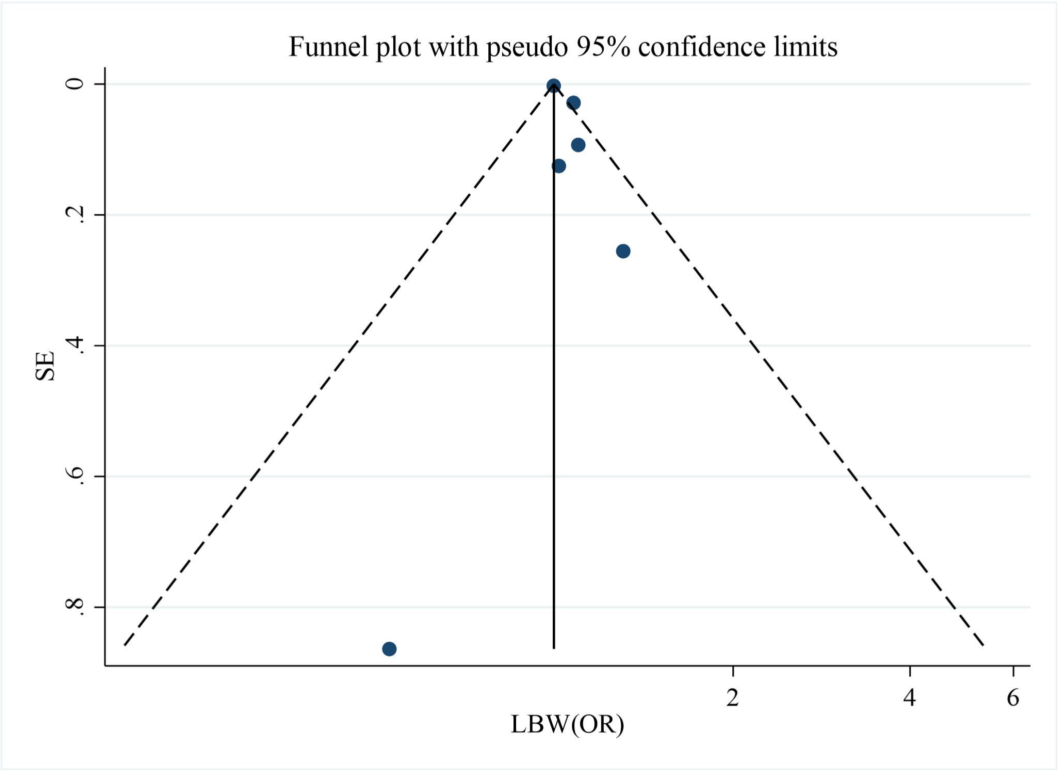

Fig. 7. Results of a publication bias assessment of arsenic for low birth weight (Funnel Plot).

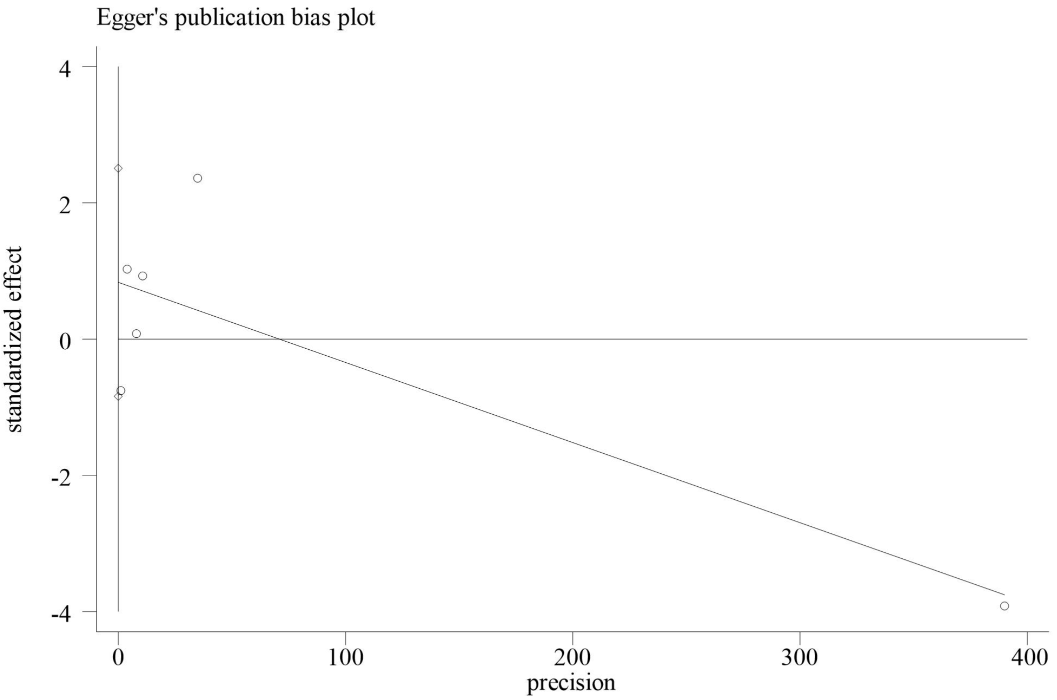

Fig. 8. Results of a publication bias assessment of arsenic for low birth weight (Egger's).

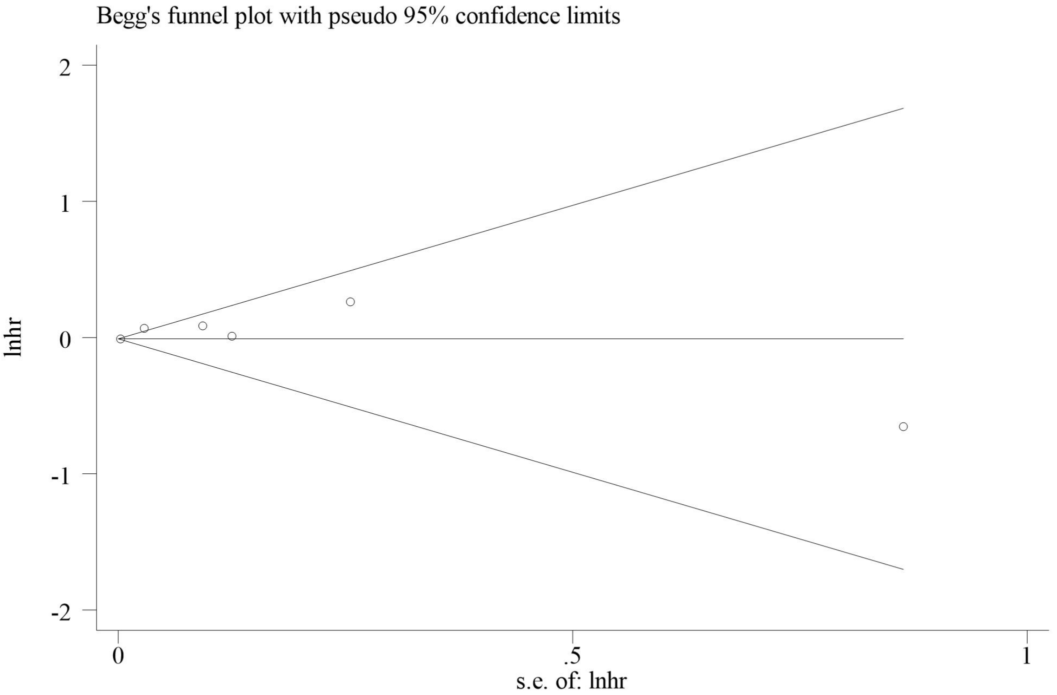

Fig. 9. Results of a publication bias assessment of arsenic for low birth weight (Begg's).
