# Supplementary material for: Association between arsenic exposure and intrauterine growth restriction: A systematic review and meta-analysis
Source: PLoS One. 2025 Jun 2;20(6):e0320603. doi: 10.1371/journal.pone.0320603 (PMC12129153; doi:10.1371/journal.pone.0320603)
Supplement: Attachment — Submitted filename: Response to Reviewers.docx [file pone.0320603.s014.docx]

**Responses to the comments**

Date: January 16, 2025

*PLOS ONE*

Dear Tamara Sljivancanin Jakovljevic editor,

We sincerely thank the reviewers and editors for their thorough review of our manuscript (Manuscript ID: PONE-D-24-51061) entitled “Association between arsenic exposure and intrauterine growth restriction: A systematic review and meta-analysis.” We have carefully considered all comments and made the corresponding revisions to the manuscript. Below, we provide detailed responses to each comments. All revisions have been addressed in the responses and are highlighted in the manuscript with a yellow background. We hope the revised manuscript will be deemed acceptable. Once again, thank you for your time and valuable feedback!

**Responses to the Editor's Comments**

**Comment 1:** When submitting your revision, we need you to address these additional requirements. Please ensure that your manuscript meets PLOS ONE's style requirements, including those for file naming.

**Response:** Thank you for your thorough review and feedback. We have carefully reviewed the manuscript and referred to the PLOS ONE formatting template to comprehensively optimize the title, section hierarchy, figure and table captions, file naming, and overall layout to ensure the manuscript meets the journal's requirements. Please feel free to let us know if further adjustments are needed.

**Comment 2:** We note that the grant information you provided in the ‘Funding Information’ and ‘Financial Disclosure’ sections do not match. When you resubmit, please ensure that you provide the correct grant numbers for the awards you received for your study in the ‘Funding Information’ section.

**Response:** We apologize for our oversight and have carefully reviewed the "Funding Information" and "Financial Disclosure" sections to ensure consistency between the two. We have also provided the correct grant numbers for all research funding. Thank you for your correction.

**Comment 3:** We note that there is identifying data in the Supporting Information file <Supplementary Data.docx>. Due to the inclusion of these potentially identifying data, we have removed this file from your file inventory. Prior to sharing human research participant data, authors should consult with an ethics committee to ensure data are shared in accordance with participant consent and all applicable local laws.

Data sharing should never compromise participant privacy. It is therefore not appropriate to publicly share personally identifiable data on human research participants. The following are examples of data that should not be shared:

-Name, initials, physical address

-Ages more specific than whole numbers

-Internet protocol (IP) address

-Specific dates (birth dates, death dates, examination dates, etc.)

-Contact information such as phone number or email address

-Location data

-ID numbers that seem specific (long numbers, include initials, titled “Hospital ID”) rather than random (small numbers in numerical order)

Data that are not directly identifying may also be inappropriate to share, as in combination they can become identifying. For example, data collected from a small group of participants, vulnerable populations, or private groups should not be shared if they involve indirect identifiers (such as sex, ethnicity, location, etc.) that may risk the identification of study participants.

Additional guidance on preparing raw data for publication can be found in our Data Policy (https://journals.plos.org/plosone/s/data-availability#loc-human-research-participant-data-and-other-sensitive-data) and in the following article: http://www.bmj.com/content/340/bmj.c181.long.

Please remove or anonymize all personal information (Name, Date), ensure that the data shared are in accordance with participant consent, and re-upload a fully anonymized data set. Please note that spreadsheet columns with personal information must be removed and not hidden as all hidden columns will appear in the published file.

**Response:** Thank you for your valuable comments on the data sharing section. In response to your feedback, we have carefully reviewed the submitted supporting information files (S6 Table, S5 Table, and S7 Table). We confirm that the "authors" and "years" listed are author information and publication years from publicly available literature, and do not involve any personal data of research participants. These data are sourced from publicly available literature, and the tables do not contain any personal information that could identify individual participants. Therefore, we believe that the tables do not compromise participant privacy. We understand and prioritize data privacy protection, and if the reviewer has any further concerns, we are happy to review and ensure that the data comply with privacy requirements. Thank you again for your suggestions, and we look forward to your further guidance.

**Comment 4:** Please include captions for your Supporting Information files at the end of your manuscript, and update any in-text citations to match accordingly. Please see our Supporting Information guidelines for more information: <http://journals.plos.org/plosone/s/supporting-information.>

**Response:** Thank you for your feedback and guidance! Based on your suggestions, we have added clear titles for all supporting information files at the end of the manuscript and ensured that the in-text citations align with the newly added titles. Additionally, we have thoroughly reviewed the format and content of the supporting information files to ensure compliance with the journal's requirements, enhancing the transparency and rigor of the study. If you have any further suggestions or adjustments, please feel free to let us know, and we will fully cooperate. Thank you for your support!

**Comment 5:** As required by our policy on Data Availability, please ensure your manuscript or supplementary information includes the following:

A numbered table of all studies identified in the literature search, including those that were excluded from the analyses.

For every excluded study, the table should list the reason(s) for exclusion.

If any of the included studies are unpublished, include a link (URL) to the primary source or detailed information about how the content can be accessed.

A table of all data extracted from the primary research sources for the systematic review and/or meta-analysis. The table must include the following information for each study:

Name of data extractors and date of data extraction

Confirmation that the study was eligible to be included in the review.

All data extracted from each study for the reported systematic review and/or meta-analysis that would be needed to replicate your analyses.

If data or supporting information were obtained from another source (e.g. correspondence with the author of the original research article), please provide the source of data and dates on which the data/information were obtained by your research group.

If applicable for your analysis, a table showing the completed risk of bias and quality/certainty assessments for each study or outcome. Please ensure this is provided for each domain or parameter assessed. For example, if you used the Cochrane risk-of-bias tool for randomized trials, provide answers to each of the signalling questions for each study. If you used GRADE to assess certainty of evidence, provide judgements about each of the quality of evidence factor. This should be provided for each outcome.

An explanation of how missing data were handled.

This information can be included in the main text, supplementary information, or relevant data repository. Please note that providing these underlying data is a requirement for publication in this journal, and if these data are not provided your manuscript might be rejected.

**Response:** Thank you for reviewing and providing feedback on our manuscript! In response to your requirements, we have carefully addressed each point and supplemented the necessary information:

1. We have submitted S5 Table: Study Screening and Exclusion Details, which provides a detailed list of all identified studies, including the screening stages, titles, inclusion/exclusion status, and reasons for exclusion, ensuring transparency in the screening process.
2. The data for this study were entirely derived from publicly available literature, and no unpublished studies were included. Therefore, the related explanation is not applicable. Specific search strategies (S2 Table) and details of included studies (S6 Table) have been provided in the supporting materials to facilitate reproducibility.
3. We have submitted S7 Table: Data Extraction Table, which includes the names of data extractors, extraction dates, inclusion eligibility confirmation, and detailed data required for the analysis, ensuring transparency and reproducibility.
4. This study used the NTP/OHAT tool to assess the risk of bias in the included studies and applied NOS and AHRQ standards to evaluate study quality. Specific details are as follows:

S3 Table: Provides detailed information about the NTP/OHAT risk of bias assessment tool, including evaluation criteria and scoring standards.

Fig 2: Visualizes the results of the risk of bias assessment.

S4 Table: Contains the study quality scores based on NOS and AHRQ standards.

5. During data extraction, no significant missing data were identified, so no additional processing was necessary. All relevant data are comprehensively presented in S7 Table.
6. All supporting materials, such as the PRISMA flowchart, search strategies, and evaluation tools, have been submitted along with the manuscript. The manuscript strictly adheres to the journal's data availability policy, ensuring transparency and reproducibility of the study.

If there are further requirements or additional information needed, please feel free to let us know. We will fully cooperate. Thank you for your patience and support!

**Comment 6:** Please review your reference list to ensure that it is complete and correct. If you have cited papers that have been retracted, please include the rationale for doing so in the manuscript text, or remove these references and replace them with relevant current references. Any changes to the reference list should be mentioned in the rebuttal letter that accompanies your revised manuscript. If you need to cite a retracted article, indicate the article’s retracted status in the References list and also include a citation and full reference for the retraction notice.

**Response:** We have carefully reviewed the reference list and verified the status of each cited article. Upon thorough examination, no retracted articles were identified in the reference list. Additionally, we have ensured that all cited references are complete, accurate, and up to date. If there are any further questions or adjustments required, we will fully cooperate.

**Responses to the Reviewers' Comments**

**Comment 1:** Is the manuscript technically sound, and do the data support the conclusions?

The manuscript must describe a technically sound piece of scientific research with data that supports the conclusions. Experiments must have been conducted rigorously, with appropriate controls, replication, and sample sizes. The conclusions must be drawn appropriately based on the data presented.

Reviewer #1: Yes

Reviewer #2: Yes

**Response:** Thank you to both reviewers for your positive feedback on our manuscript! We are delighted that the technical soundness, experimental design, and data support of our study have been acknowledged. Should you have any further suggestions, we are happy to make additional improvements. Once again, we sincerely appreciate your valuable comments!

**Comment 2:** Has the statistical analysis been performed appropriately and rigorously?

Reviewer #1: Yes

Reviewer #2: Yes

**Response:** Thank you to the reviewers for your positive feedback on our statistical analysis! Your comments have strengthened our confidence in maintaining a rigorous research approach. If you have any further suggestions, we are happy to make improvements at any time. Once again, we sincerely appreciate your valuable input!

**Comment 3:** Have the authors made all data underlying the findings in their manuscript fully available?

The PLOS Data policy requires authors to make all data underlying the findings described in their manuscript fully available without restriction, with rare exception (please refer to the Data Availability Statement in the manuscript PDF file). The data should be provided as part of the manuscript or its supporting information, or deposited to a public repository. For example, in addition to summary statistics, the data points behind means, medians and variance measures should be available. If there are restrictions on publicly sharing data—e.g. participant privacy or use of data from a third party—those must be specified.

Reviewer #1: Yes

Reviewer #2: Yes

**Response:** Thank you to the reviewers for your positive feedback on our data provision! We have provided the data as comprehensively as possible to ensure its completeness and availability. Should further supplementation or improvement be needed, we will fully cooperate. Once again, we sincerely appreciate your support!

**Comment 4:** Is the manuscript presented in an intelligible fashion and written in standard English?

PLOS ONE does not copyedit accepted manuscripts, so the language in submitted articles must be clear, correct, and unambiguous. Any typographical or grammatical errors should be corrected at revision, so please note any specific errors here.

Reviewer #1: Yes

Reviewer #2: Yes

**Response:** We thank the reviewers for their positive feedback on the language and expression of our manuscript. We place great emphasis on linguistic accuracy and have conducted multiple rounds of proofreading and revisions. Should there be further suggestions, we would be happy to make additional improvements. Thank you for your support!

**Comment 5:** Please use the space provided to explain your answers to the questions above. You may also include additional comments for the author, including concerns about dual publication, research ethics, or publication ethics. (Please upload your review as an attachment if it exceeds 20,000 characters)

Reviewer #1: This study aims to systematically evaluate the association between As exposure and IUGR ,SGA，PTB, LBW through a meta analysis.  The study has resolved the inconsistencies in previous studies and provides comprehensive evidence.The manuscript technically sound and the data support the conclusions.

Reviewer #2: Few grammatical and typographical errors can be reduced.

Reason for the presence of necessity is not justified adequately.

in addition of database search, use of EMBASE or SCOPUS would have been better

the Risk of bias was adequately assessed.

references were adequate

**Response:**

Response to Reviewer 1:

Thank you for your thoughtful review, particularly for your positive evaluation of the research objectives, technical soundness, and data support. This study aims to systematically assess the association between arsenic exposure and intrauterine growth restriction, low birth weight, and preterm birth, addressing inconsistencies in prior studies and providing more comprehensive evidence. Your feedback is a great encouragement to us, and we will continue to refine the research. Should you have any additional suggestions, we are more than willing to make further improvements!

Response to Reviewer 2:

Thank you for your valuable comments on this paper. In response to the two issues raised, we provide the following replies:

1. We have carefully reviewed and revised the manuscript to address grammar and typographical errors as much as possible.
2. Regarding the "insufficient explanation of the study’s necessity," we have thoroughly examined the introduction and believe that it adequately elaborates on the research background, inconsistencies in existing evidence, and the scientific value, clearly demonstrating the necessity of conducting this study. We greatly appreciate your feedback and will continue to ensure that the manuscript is clear and rigorous.
3. Concerning database selection, we sincerely appreciate your suggestion to include EMBASE and SCOPUS. However, due to limitations of time and database accessibility, we utilized CNKI, Wanfang, VIP, PubMed, Web of Science, and ScienceDirect, which are comprehensive and adequately support our research needs. Thank you again for acknowledging the bias risk assessment and the adequacy of our references. Should you have further suggestions, we are fully committed to improving the manuscript!

**Comment 6:** While revising your submission, please upload your figure files to the Preflight Analysis and Conversion Engine (PACE) digital diagnostic tool, https://pacev2.apexcovantage.com/. PACE helps ensure that figures meet PLOS requirements. To use PACE, you must first register as a user. Registration is free. Then, login and navigate to the UPLOAD tab, where you will find detailed instructions on how to use the tool. If you encounter any issues or have any questions when using PACE, please email PLOS at figures@plos.org. Please note that Supporting Information files do not need this step.

**Response:** Thank you for your guidance! We have registered and used the PACE tool as requested to review and revise the figures, ensuring they comply with PLOS formatting requirements. Please feel free to let us know if there are any further issues or additional information needed.
